# Meta-analysis of shotgun sequencing of gut microbiota in Parkinson’s disease

**DOI:** 10.1038/s41531-024-00724-z

**Published:** 2024-05-21

**Authors:** Hiroshi Nishiwaki, Jun Ueyama, Mikako Ito, Tomonari Hamaguchi, Keiichi Takimoto, Tetsuya Maeda, Kenichi Kashihara, Yoshio Tsuboi, Hiroshi Mori, Ken Kurokawa, Masahisa Katsuno, Masaaki Hirayama, Kinji Ohno

**Affiliations:** 1 Division of Neurogenetics, Center for Neurological Diseases and Cancer, Nagoya University Graduate School of Medicine, Nagoya, Japan; 2 Department of Pathophysiological Laboratory Sciences, Nagoya University Graduate School of Medicine, Nagoya, Japan; 3 Division of Neurology and Gerontology, Department of Internal Medicine, School of Medicine, Iwate Medical University, Iwate, Japan; 4 Okayama Neurology Clinic, Okayama, Japan; 5 Department of Neurology, Fukuoka University, Fukuoka, Japan; 6 Advanced Genomics Center, National Institute of Genetics, Mishima, Japan; 7 Department of Neurology, Nagoya University Graduate School of Medicine, Nagoya, Japan; 8 Department of Occupational Therapy, Chubu University College of Life and Health Sciences, Kasugai, Japan; 9 Graduate School of Nutritional Sciences, Nagoya University of Arts and Sciences, Nagoya, Japan

**Keywords:** Parkinson's disease, Genetic databases

## Abstract

We aimed to identify gut microbial features in Parkinson’s disease (PD) across countries by meta-analyzing our fecal shotgun sequencing dataset of 94 PD patients and 73 controls in Japan with five previously reported datasets from USA, Germany, China1, China2, and Taiwan. GC-MS and LC-MS/MS assays were established to quantify fecal short-chain fatty acids (SCFAs) and fecal polyamines, respectively. α-Diversity was increased in PD across six datasets. Taxonomic analysis showed that species *Akkermansia muciniphila* was increased in PD, while species *Roseburia intestinalis* and *Faecalibacterium prausnitzii* were decreased in PD. Pathway analysis showed that genes in the biosyntheses of riboflavin and biotin were markedly decreased in PD after adjusting for confounding factors. Five out of six categories in carbohydrate-active enzymes (CAZymes) were decreased in PD. Metabolomic analysis of our fecal samples revealed that fecal SCFAs and polyamines were significantly decreased in PD. Genes in the riboflavin and biotin biosyntheses were positively correlated with the fecal concentrations of SCFAs and polyamines. Bacteria that accounted for the decreased riboflavin biosynthesis in Japan, the USA, and Germany were different from those in China1, China2, and Taiwan. Similarly, different bacteria accounted for decreased biotin biosynthesis in the two country groups. We postulate that decreased SCFAs and polyamines reduce the intestinal mucus layer, which subsequently facilitates the formation of abnormal α-synuclein fibrils in the intestinal neural plexus in PD, and also cause neuroinflammation in PD.

## Introduction

Parkinson’s disease (PD) is a long-term neurogenerative disorder that presents not only motor symptoms but also non-motor symptoms. Motor symptoms in PD patients include rigidity (muscle stiffness), postural instability, gait disturbance, bradykinesia, and resting tremor, while non-motor symptoms in PD patients include dementia, depression, anosmia, bladder problems, constipation, and orthostatic hypotension. PD is characterized by abnormal accumulation of α-synuclein fibrils (Lewy bodies) in the dopaminergic neurons in the substantia nigra. Lewy bodies also appear in the autonomic nervous system, lower brainstem, cerebral cortex, and non-neuronal tissues, such as olfactory bulbs, skin, salivary glands, and intestinal mucosa^1^. In 2003, Braak hypothesized that abnormal α-synuclein fibrils start from the nucleus tractus solitarius of the vagal nerve and go through the locus coeruleus, finally ascending to the substantia nigra^2^. PD patients sometimes present with constipation, idiopathic rapid eye movement sleep behavior disorder (iRBD), and depression about 20, 10, and 5 years before developing motor symptoms^3^, which is in accordance with the ascendance of α-synuclein fibrils in Braak’s hypothesis. Total vagotomy reduces the risk of PD by about 50%^4^, implying that ~50% of PD patients may follow this hypothesis, while the others may not.

Gut microbiota in PD has been analyzed by three different methods: qPCR, 16S rRNA metagenomic sequencing, and shotgun metagenomic sequencing (shotgun-seq). qPCR was employed in three studies^5–7^. In contrast, 16S rRNA gene sequencing was employed in more than 20 studies^8–10^. We showed by meta-analysis of 16S rRNA gene sequencing datasets that mucin-degrading genus *Akkermansia* was increased and short-chain fatty acids (SCFAs)-producing genera *Roseburia* and *Faecalibacterium* were decreased in PD across countries^8^. We also showed by a prospective study that two decreased SCFA-producing genera, *Fusicatenibacter* and *Faecalibacterium*, were able to predict rapid progression of PD in the early stage in two years^9^. In contrast, Aho and colleagues showed that decreased *Prevotella* was associated with rapid progression of PD in two years^11^. Eight studies used shotgun-seq in PD in five countries^12–19^ (Supplementary Table 1). Gut microbiota in healthy subjects are markedly different from country to country^20^. Marked difference in gut microbiota across countries makes it difficult to identify causally associated intestinal bacteria and bacterial genes in PD patients. We here performed shotgun-seq of fecal samples of 94 PD patients and 73 controls, and meta-analyzed our dataset with five previously reported datasets to identify bacterial taxa, genes, and pathways that were specifically changed in PD across countries. Decreased genes in fecal riboflavin and biotin biosyntheses were positively correlated with the decreased fecal SCFAs and polyamines. In addition, different bacterial species were responsible for the decreased genes in fecal riboflavin and biotin biosyntheses in different countries.

## Results

### Shotgun-seq of our dataset in Japan and five other datasets from the USA, Germany, China1, China2, and Taiwan

**Fig. 1:**
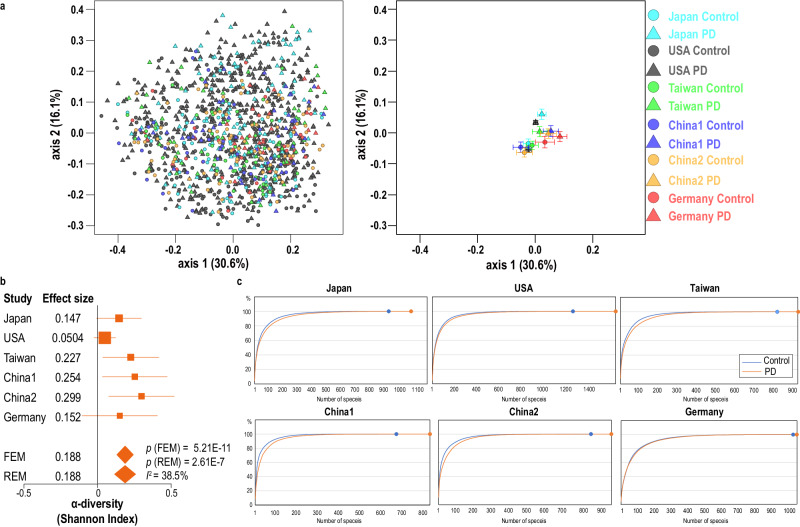
Global bacterial features of six datasets. **a** Left panel: PCoA plot of bacterial species in PD and controls in six countries with Bray–Curtis dissimilarity. Circles and triangles indicate controls and PD, respectively. Countries are color-coded. Right panel: PCoA plot showing the center of gravity and the standard error of the overall compositions of bacterial species in six datasets in PD and controls with Bray–Curtis dissimilarity. Both panels have the same x- and y-scales. **b** Forest plot of α-diversities of bacterial species in six datasets. FEM and REM represent fixed and random effect models, respectively. The square sizes represent the number of samples. Bars represent 95% confidence interval. Diamonds represent the mean and 95% confidence interval. *P*-values of FEM and REM and *I*^2^ are indicated. **c** The cumulative frequency plots of the relative abundance at the species level in six datasets. The number of identified bacterial species in controls and PD in each dataset is indicated by a small circle at the end of the curve.

We compared seven features (age, sex, BMI, constipation, proton pump inhibitors, H_2_ blockers, and anticholesterol drugs) between PD and controls in our dataset (Table 1). BMI was lower in PD than in controls, and constipation was more frequent in PD than in controls. Next, we meta-analyzed our dataset with five previously reported datasets from four countries (the USA, Germany, China, and Taiwan). Principal coordinate analysis (PCoA) of bacterial species showed that PD patients and controls made distinct centers of gravity in each country (Fig. 1a, Supplementary Table 2) and the overall bacterial compositions by PERMANOVA were different from country to country (Supplementary Table 2). We also meta-analyzed α-diversity at the species level with Shannon index, and found that α-diversity was higher in PD than in controls (Fig. 1b). Cumulative frequency plots at the species level in six datasets showed that the increased α-diversities were not accounted for by decrease or increase of specific sets of bacteria (Fig. 1c). Instead, the numbers of bacterial species were higher in PD and the relative bacterial abundances were more evenly distributed in PD compared to controls.

**Table 1:** Demographic and clinical features of PD patients and controls in our dataset

	Patients (*n* = 94)	Controls (*n* = 73)	*p*-value
Age (years)	69.1 ± 7.7^a^	67.8 ± 9.1^a^	0.34^b^
# Females/Males	58/36	35/38	0.086^c^
Body mass index (BMI)	21.4 ± 3.1^a^	22.7 ± 3.2^a^	0.012^b*^
# Constipation (twice a week or less)	36 out of 94	3 out of 73	7.3E−08^c*^
Disease duration (years)	8.0 ± 5.9^a^	–	–
Total UPDRS	53.9 ± 23.0^a^	–	–
	(range 16 to 115)		
UPDRS III	27.7 ± 13.5^a^	–	–
	(range 4 to 61)		
Hoehn-Yahr stage	2.78 ± 0.94^a^	–	–
# Proton pump inhibitors	11 out of 94	7 out of 73	0.80^c^
# H_2_ blockers	4 out of 94	4 out of 73	0.73^c^
# Anticholesterol drugs	17 out of 94	14 out of 73	1.00^c^

*p-value ≤ 0.05.

^a^Mean and SD.

^b^Student’s *t* test.

^c^Fisher’s exact test.

We next analyzed Enzyme Commission (EC) numbers with HUMAnN 3.6. Meta-analysis of the six datasets showed that, out of the 1678 EC numbers, 42 and 31 were significantly decreased and increased, respectively, in PD (Supplementary Table 3). Two types of pathway analyses were applied: the hypergeometric test and the gene set enrichment analysis (GSEA). First, the hypergeometric test was applied to each Kyoto Encyclopedia of Genes and Genomes (KEGG) pathway in increased and decreased directions (Supplementary Table 4). “Riboflavin metabolism” and “biotin metabolism” were significantly decreased in PD, while no increased pathway was identified. Second, GSEA was applied to each KEGG pathway in increased and decreased directions (Supplementary Table 5). Downregulations of “biotin metabolism” and “riboflavin metabolism” were again ranked first and second in GSEA analysis. Confounding factor analysis with MaAsLin2^21^ of our dataset and the USA dataset^17^, for which metadata on age, BMI, constipation, disease state, sex, and anticholesterol drugs were available, showed genes assigned to the EC numbers in “riboflavin metabolism” and “biotin metabolism” were significantly decreased in PD after adjusting for confounding factors (Supplementary Table 6).

We next analyzed bacterial species and genera. Meta-analysis of the six datasets showed that 31 species were significantly increased in PD, while seven species were significantly decreased (Supplementary Table 7). Additionally, 24 genera were significantly increased, and seven genera were significantly decreased (Supplementary Table 7). Notably, at the species level, *Akkermansia muciniphila* was significantly increased, while *Roseburia intestinalis* and *Faecalibacterium prausnitzii* were significantly decreased in PD (Supplementary Fig. 1). These bacteria were previously identified in our meta-analysis at the genus level^8^. However, unlike the previous meta-analysis, there was considerable heterogeneity (*I*^*2*^ > 40%) across datasets, possibly due to the inclusion of China1 (Shanghai, China; PRJNA433459^13^), China2 (Xiangyang, China ;PRJNA588035^14^), and Taiwan, which were not included in the previous analysis^8^. We also adjusted for confounding factors in our and USA datasets. At the species level, *Akkermansia muciniphila* was significantly increased, while *Roseburia intestinalis* and *Faecalibacterium prausnitzii* were significantly decreased in PD after adjusting for confounding factors (Supplementary Table 8). Similarly, at the genus level, *Akkermansia* was significantly increased, whereas *Roseburia* and *Faecalibacterium* were significantly decreased in PD after adjusting for confounding factors (Supplementary Table 8).

**Fig. 2:**
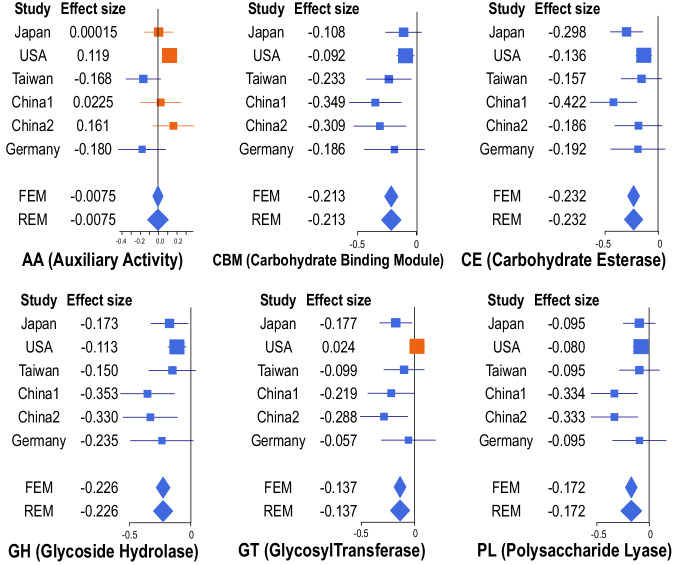
Forest plots of six categorical CAZymes in six datasets. Orange and blue symbols represent increased and decreased CAZymes in PD, respectively. The square sizes represent the number of samples. Bars represent 95% confidence interval. Diamonds represent the mean and 95% confidence interval.

We next analyzed carbohydrate-active enzymes (CAZyme). Meta-analysis of the six datasets showed that, out of 219 CAZymes, 72 and 17 were significantly decreased and increased, respectively (Supplementary Table 9). Categorical analysis showed that 5 of 6 CAZyme categories were significantly decreased in PD across six datasets (Fig. 2, Supplementary Table 10), suggesting extensive reductions in bacterial carbohydrate metabolisms in PD.

### Quantification of fecal SCFAs and polyamines

**Fig. 3:**
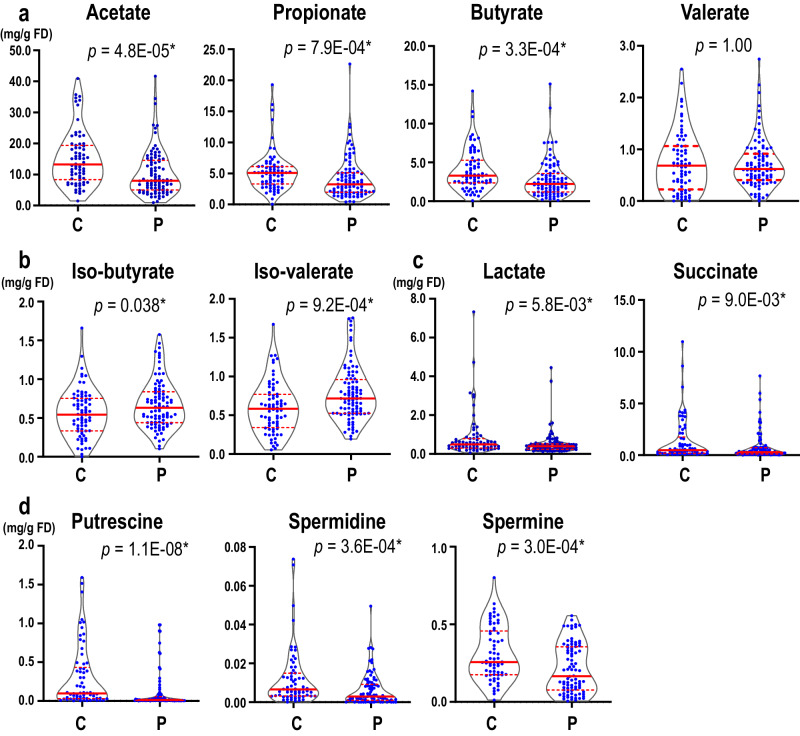
Fecal metabolites in controls (C) and PD (P). Violin plots of (**a**) short-chain fatty acids, (**b**) branched chain fatty acids, (**c**) lactate and succinate, and (**d**) polyamines in feces in controls and PD. Concentrations are indicated by mg per g of freeze-dried fecal sample (mg/g FD). Median and interquartile range are indicated. The sample sizes were 92 (PD) and 72 (controls) for acetate, propionate, butyrate, valerate, iso-butyrate, and iso-valerate, 91 (PD) and 70 (controls) for lactate and succinate, and 86 (PD) and 66 (controls) for polyamines. *P*-values were calculated by the Wilcoxon rank sum test. ^*^P < 0.05.

SCFA-producing bacteria were significantly decreased in our previous meta-analysis of five datasets of 16S rRNA gene sequencing^8^. In our current meta-analysis of six datasets of shotgun-seq, similar species and genera (Supplementary Table 7) were decreased in PD. We thus quantified fecal SCFAs, as well as fecal lactate and pyruvate, using our proprietary gas chromatography with mass spectrometry (GC-MS)-based assay systems^22,23^. We found that the fecal concentrations of three SCFAs (acetate, propionate, and butyrate) were significantly decreased in PD, while valerate was not (Fig. 3a). On the other hand, branched chain fatty acids (iso-butyrate and iso-valerate) were significantly increased in PD (Fig. 3b), although the underlying mechanisms remain elusive. Lactate and succinate, which are transformed into SCFAs by gut bacteria, were significantly decreased in PD (Fig. 3c).

In addition to the decreased “riboflavin metabolism” and “biotin metabolism” in PD as stated above, GSEA also showed significant decrease of “arginine and proline metabolism” in PD (Supplementary Table 5). As “arginine and proline metabolism” included 103 genes generating arginine, proline, glutamate, and polyamines, we extracted five arginine-related subpathways, two glutamate- and proline-related subpathways, and three polyamine-related subpathways from the MetaCyc database^24^. Meta-analysis of the ten MetaCyc pathways with GSEA showed that the three polyamine-related subpathways were all significantly decreased in PD (Supplementary Table 11). Indeed, a liquid chromatography with tandem mass spectrometry (LC-MS/MS)-based assay showed that fecal concentrations of three polyamines (putrescine, spermidine, and spermine) were significantly decreased in PD (Fig. 3d).

### Correlation between metagenomic genes in “riboflavin metabolism” or “biotin metabolism”, and fecal SCFAs or polyamines

**Fig. 4:**
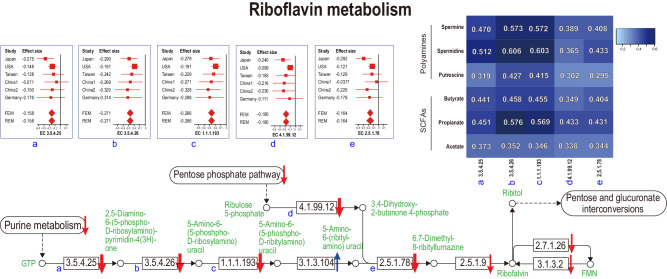
A schematic of the riboflavin metabolism pathway. Enzymes are indicated by EC numbers in squares. Metabolites are indicated in green letters and open circles. KEGG pathways are indicated by round squares. Arrows indicate decreased or increased in PD. Significant and non-significant changes are indicated by thick and thin arrows, respectively. Forest plots of six datasets are indicated for the five significantly changed enzymes. A right upper heatmap indicates the Spearman’s rank correlation coefficients between the five significantly changed enzymes and fecal SCFAs/polyamines. The square sizes represent the number of samples. Bars represent 95% confidence interval. Diamonds represent the mean and 95% confidence interval. The same EC numbers are labeled by a–e.

**Fig. 5:**
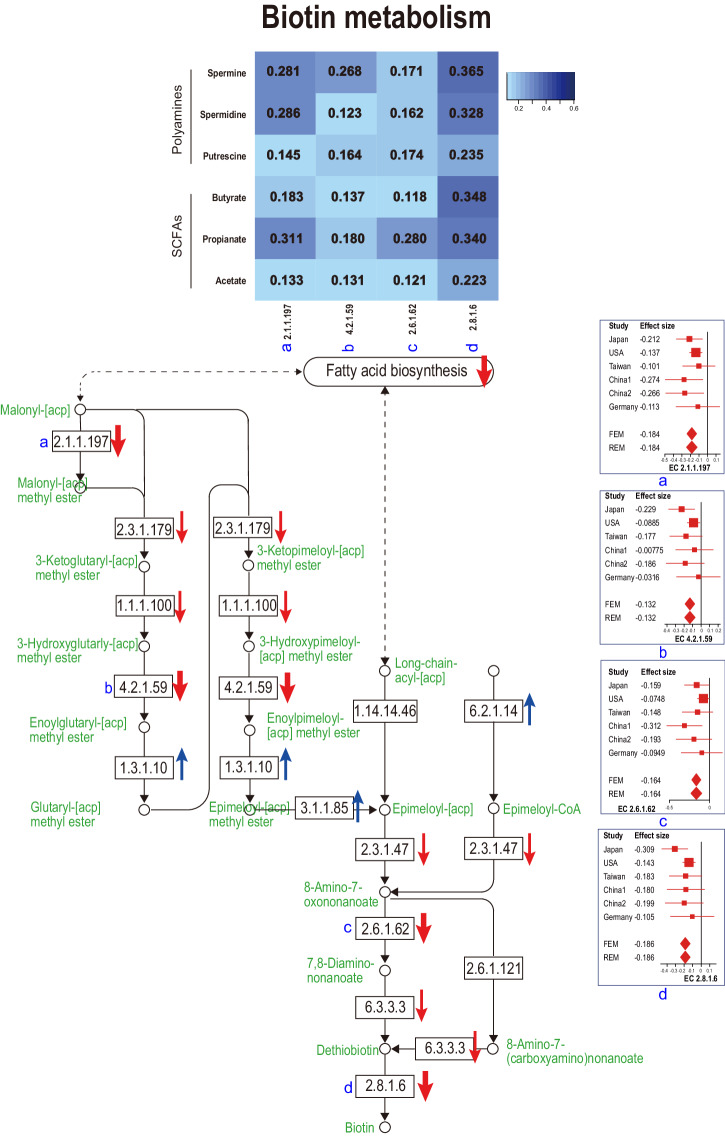
A schematic of the biotin metabolism pathway. Enzymes are indicated by EC numbers in squares. Metabolites are indicated in green letters and open circles. KEGG pathways are indicated by round squares. Arrows indicate decreased or increased in PD. Significant and non-significant changes are indicated by thick and thin arrows, respectively. Forest plots of six datasets are indicated for the four significantly changed enzymes. A right upper heatmap indicates the Spearman’s rank correlation coefficients between the four significantly changed enzymes and fecal SCFAs/polyamines. The square sizes represent the number of samples. Bars represent 95% confidence interval. Diamonds represent the mean and 95% confidence interval. The same EC numbers are labeled by a–d.

We calculated the Spearman’s rank correlation coefficient between CPMs (copies per millions) of metagenomic genes in “riboflavin metabolism” (Fig. 4) or “biotin metabolism” (Fig. 5), and fecal concentrations of SCFAs or polyamines. In both “riboflavin metabolism” and “biotin metabolism”, metagenomic genes that were significantly decreased in PD in meta-analysis were positively correlated with fecal concentrations of SCFAs and polyamines (Figs. 4 and 5).

### Intestinal bacterial species that accounted for decreased “riboflavin metabolism” and “biotin metabolism”

**Fig. 6:**
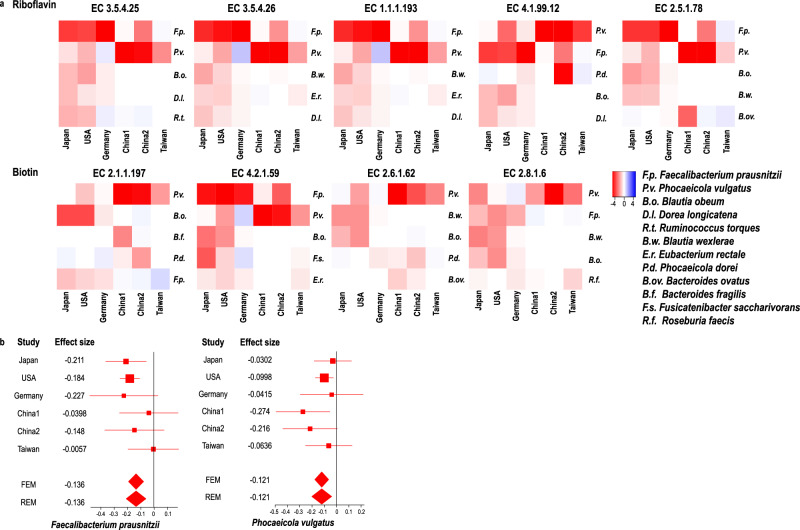
Most influential bacteria in riboflavin and biotin metabolisms. **a** Heatmap showing the difference of the fractional CPMs between PD and controls of five most influential bacteria in each dataset. **b** Forest plots of *Faecalibacterium prausnitzii* and *Phocaeicola vulgatus* in six datasets.

We examined the bacterial origins of genes in “riboflavin metabolism” and “biotin metabolism”. For each of the nine significantly decreased EC numbers in these metabolisms (Figs. 4 and 5), we calculated fractional CPM contributed by each bacterium (Supplementary Table 12). For each EC number, we selected five most influential bacteria in the six datasets. The difference of the fractional CPMs between PD and controls in each dataset is shown in the heatmap (Fig. 6a). Intestinal bacteria that accounted for the decreased riboflavin and biotin metabolisms were different from country to country. The most influential bacteria for decreased riboflavin metabolism were *Faecalibacterium prausnitzii* in Japan, the USA, and Germany, and *Phocaeicola vulgatus* (previously called *Bacteroides vulgatus*) in China1, China2, and Taiwan. Similarly, decreased genes in biotin metabolism were mostly mediated by decreased *Blautia obeum* in Japan, the USA, and Germany, and by decreased *Phocaeicola vulgatus* in China1, China2, and Taiwan. Indeed, *Faecalibacterium prausnitzii* was markedly decreased in Japan, the USA, and Germany, while *Phocaeicola vulgatus* was markedly decreased in China1 and China2 (Fig. 6b).

## Discussion

Eight shotgun-seq studies of gut microbiota have been reported in PD, but shared features cannot be readily recognized. We performed shotgun-seq analysis of PD patients and healthy cohabitants in Japan, and meta-analyze our dataset with five previously reported shotgun-seq datasets from Germany^12^, China^13,14^, Taiwan^15^, and the USA^17^ after adjusting for batch effects. PCoA (Fig. 1a) and PERMANOVA (Supplementary Table 2) of bacterial species showed that the overall bacterial compositions were significantly different across countries, and between PD and controls in each country. Meta-analysis revealed that α-diversity at the species level was significantly increased in PD (Fig. 1b), which was accounted for by more numbers of bacteria and more even distribution of bacterial abundances in PD compared to controls (Fig. 1c). A previous meta-analysis of α-diversity at the genus level using 16S rRNA gene sequencing datasets showed that α-diversity tended to be increased in PD in five out of seven datasets, but no significance was observed in the overall score^25^. Meta-analysis of 4347 shotgun-seq data of gut microbiota in twelve diseases showed that α-diversity remained unchanged in nine diseases and decreased in three diseases (Crohn’s disease, obesity, and type 2 diabetes)^26^. As far as we know, PD is the first disease that increases α-diversity of gut microbiota, which is in contrast to the notion that decreased α-diversity is a fecal marker for gastrointestinal, immunological, and metabolic disorders^26^.

Meta-analysis of the six datasets disclosed 73 significantly changed EC numbers out of 1678 (Supplementary Table 3). Pathway analysis with GSEA showed that significant decreases of “riboflavin metabolism” and “biotin metabolism” in PD were ranked first and second, respectively (Supplementary Table 5). Similarly, the significant decrease of “arginine and proline metabolism” was ranked sixth (Supplementary Table 5). These three pathways were also decreased by pathway analysis with the hypergeometric test, although significance was observed only in “riboflavin metabolism” and “biotin metabolism” (Supplementary Table 4). Analysis of confounding factors showed that significantly decreased EC numbers in the three pathways were indeed accounted for by PD (Supplementary Table 6).

Riboflavin (vitamin B2) in humans originates from food and gut microbiota. Therapeutically, riboflavin improves oxidative stress, mitochondrial dysfunction, neuroinflammation, and glutamate excitotoxicity, which are related to PD pathogenesis^27^. In a clinical study, high doses of riboflavin ameliorated motor deficits in PD patients^28^. In another disease, supplementation of riboflavin in patients with Crohn’s disease decreased systemic oxidative stress, inflammatory effects, and disease activity^29^. Supplementation of riboflavin to compensate for decreased riboflavin production by gut microbiota may be beneficial in PD patients.

Biotin (vitamin B7) produces anti-inflammatory substances and decreases inflammation, which leads to the relief of allergy, immunological symptoms, and inflammatory bowel disease^30^. The effects of biotin on PD have not been reported to the best of our knowledge. In contrast, in multiple sclerosis, open^31^ and double-blind^32^ studies showed that biotin ameliorated motor and optical defects.

As “arginine and proline metabolism” included as many as 103 genes and a large number of metabolites, we analyzed ten subpathways and found that “polyamine biosynthesis” subpathways were significantly decreased (Supplementary Table 11). Putrescine, spermidine, and spermine are enzymatically produced in this order. Quantification of fecal polyamines showed that the three polyamines were significantly decreased in PD (Fig. 3d). Metabolomic analysis of plasma in PD previously showed that plasma spermidine was increased while plasma spermine was decreased, yielding a marked decrease in the spermine/spermidine ratio^33^. As polyamines are either taken up from foods, generated by metabolisms of the host cells, or generated by gut microbiota^34^, concentrations of fecal polyamines may not be simply represented in plasma polyamines. However, it is interesting to note that decreased spermine is a shared feature between plasma and feces in PD.

SCFAs are made from fermentation of dietary fiber in the gut. SCFAs promote the gene expression of *FOXP3*, and differentiates naive T cells into regulatory T (Treg) cells by inhibiting histone deacetylases^35^. SCFAs suppress microglia-mediated neuroinflammation by inducing GLP-1^36^. SCFAs are involved in the formation of the mucus layer in the intestinal epithelium. SCFA-producing bacteria are also decreased in obesity, impaired glucose tolerance, and type 2 diabetes^37^. Similar to the metabolic diseases, SCFA-producing bacteria in PD were markedly decreased in meta-analyses of 16S rRNA gene sequencing^8^ and shotgun-seq (Supplementary Tables 7 and 8). Fecal concentrations of SCFAs were indeed decreased in PD in previous studies^7,15^ and in our current study (Fig. 3a). We also observed that lactate and succinate, which are substrates of SCFAs, were also significantly decreased (Fig. 3c).

Correlation analysis between pathways and metabolites disclosed that “riboflavin biosynthesis” and “biotin biosynthesis” were positively correlated with fecal concentrations of SCFAs and polyamines (Figs. 4 and 5). First, riboflavin constitutes the electron transfer flavoprotein complex of butyryl-CoA dehydrogenase, an enzyme to product butyrate^38^. In addition, the growth of some SCFA-producing bacteria was decreased in the lack of riboflavin^39^. Similarly, in mice depleted of riboflavin for three weeks, repletion of riboflavin markedly restored cecal SCFA contents^40^. In healthy subjects, oral supplementation of 50 or 100 mg/day riboflavin for 2 weeks increased fecal biosynthesis of SCFAs^41^. Second, the effect of riboflavin on polyamine biosynthesis is mediated by vitamin B6. Flavin mononucleotide (FMN), which is generated from riboflavin, acts as a cofactor for pyridoxine 5′-phosphate (PNP) oxidase, which is required for the conversion of PNP and pyridoxamine 5′-phosphate (PMP) to active vitamin B6, pyridoxal 5’-phosphate (PLP)^42^. An observational study suggested that riboflavin is the limiting nutrient for maintaining adequate vitamin B6 in humans^43^. l-Ornithine decarboxylase (ODC) is a PLP-dependent amino acid decarboxylase and catalyzes the first step in the polyamine biosynthetic pathway, in which putrescine, spermidine, and spermine are generated^44^. Therefore, decreased riboflavin may lead to decreased polyamines through decreased vitamin B6. Third, biotin may affect the SCFA and polyamine biosyntheses by serving as an essential cofactor. However, biotin-dependent enzymes have not been extensively analyzed to date, and we do not know which enzyme(s) become defective in the absence of biotin.

Bacteria that were responsible for decreased riboflavin and biotin biosynthesis were different from country to country (Supplementary Table 12). Especially, the most influential bacteria in Japan, the USA, and Germany were different from those in China1, China2, and Taiwan (Fig. 6a). A previous analysis of healthy subjects showed that Cluster of Orthologous Genes (COG) pathways were similar across subjects, although bacterial abundances were markedly different from subject to subject, which indicated that highly variable bacteria exerted similar biological functions^45^. Similarly, our analysis showed that the decreases of distinct sets of bacteria in PD in different countries led to a decrease of an identical pathway, which poses another burden in the meta-analysis and the systematic review of gut microbiota at the genus and species levels.

A meta-analysis of shotgun-seq of gut microbiota in controls and patients with PD, Alzheimer’s disease, and multiple sclerosis in 4490 subjects was recently deposited in bioRxiv (10.1101/2023.12.05.569565). They showed that a machine-learning model differentiated gut microbiota in controls, PD, Alzheimer’s disease, and multiple sclerosis. In PD patients, microbial pathways for the biotransformation of xenobiotics were enriched, which aligned with epidemiological evidence that links environmental exposure to increased PD risk^46^. However, in our meta-analysis, these pathways were either up- or down-regulated without any statistical significance.

**Fig. 7:**
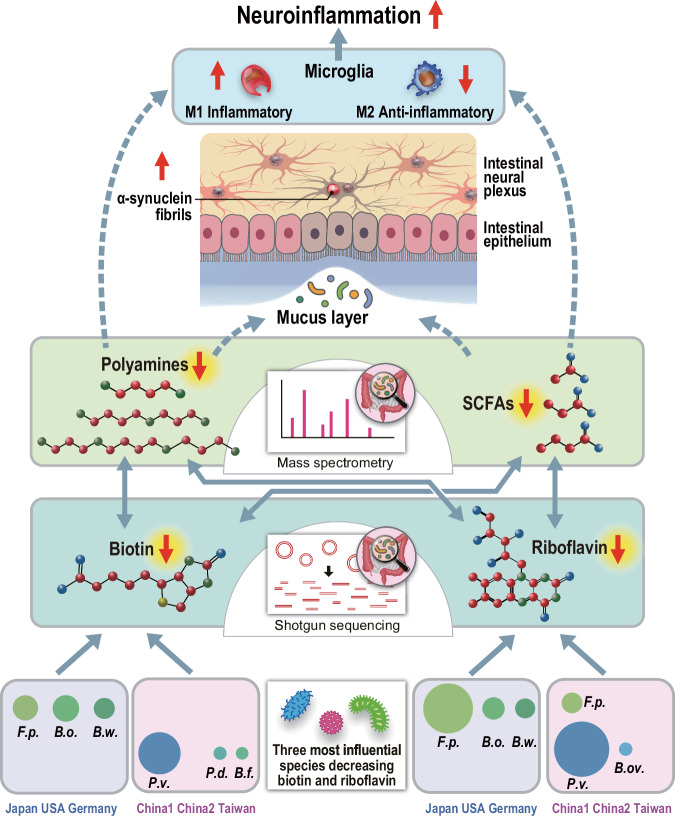
Summary and speculative perspectives of the current study. Solid arrows indicate the associations observed in the current study. Broken arrows indicate speculations based on previous reports. The sizes of the bacterial symbols represent the effects on biotin and riboflavin biosynthesis. *F.p., Faecalibacterium prausnitzii; B.o., Blautia obeum; B.w., Blautia wexlerae; P.v., Phocaeicola vulgatus; B.ov., Bacteroides ovatus; P.d., Phocaeicola dorei; and B.f., Bacteroides fragilis*.

Summary and speculative perspectives of the current study are indicated in Fig. 7. In this study, we showed that fecal biosyntheses of riboflavin and biotin were decreased in PD, which were accounted for by different bacteria in two groups of countries. We also showed that decreased fecal biosyntheses of riboflavin and biotin were correlated with decreased fecal polyamines and SCFAs. The following speculations emerge from our findings and previous studies. Decreased SCFAs and polyamines lead to thinning of the mucus layers^47^, which increases the intestinal permeability. Indeed, the intestinal permeability was increased in PD^5,48^. Increased intestinal permeability may expose the intestinal nerve plexus to pesticides, herbicides, and other toxins^49^, and lead to abnormal aggregation of α-synuclein fibrils. In addition, SCFAs and polyamines facilitate M2 macrophage polarization and relatively decreases M1 macrophage^50^, and their deficiency induces neuroinflammation^51^. We thus hypothesize that gut dysbiosis in PD causes decreased fecal productions of SCFAs and polyamines, which enhances intestinal α-synuclein fibril formation and neuroinflammation.

Decreased fecal biosyntheses of riboflavin and biotin are likely to lead to decreased fecal concentrations of riboflavin and biotin. A previously reported favorable effect of high-dose riboflavin in PD patients^28^ supports the notion that the supplementation of riboflavin and/or biotin is likely to be beneficial in a subset of PD patients, in which gut dysbiosis plays pivotal roles in PD.

## Methods

### Patients in our dataset

All studies were approved by the Ethical Review Committees (ERC) of the Nagoya University Graduate School of Medicine (approval #2016-0151), Iwate Medical College Hospital (H28-123), Okayama Kyokuto Hospital (approval #kyoIR-2016002), and Fukuoka Medical College (approval #2016M027). We obtained written informed consent from all PD patients and healthy controls (cohabitants) who lived with PD patients. Fecal samples, patient information, and the ERC approval numbers were the same as our previous study on 16S rRNA gene sequencing^8^.

We recruited 94 patients with idiopathic PD and their 73 healthy spouses who lived together from September 2015 to February 2018. Each PD patient was diagnosed according to the Movement Disorder Society’s (MDS) PD criteria. Subjects who had chronic illnesses, including diabetes mellitus, heart failure, liver cirrhosis, malignancy, hematological diseases, and autoimmune diseases were excluded. We also excluded subjects who claimed to have taken antibiotics in the last four weeks.

### Sample collection, DNA isolation and shotgun-seq of our dataset

A fecal sample was collected at the participant’s home and transported to Nagoya University while maintained at 0 °C with ice packs in a thermal insulation jar as previously described^8^. The fecal samples were freeze-dried using a freeze-dryer (FDU-2110, EYELA, Shanghai, China) and DNA was extracted from 20 mg of freeze-dried (FD) feces using the QIAamp PowerFecal DNA Kit (QIAGEN, Hilden, Germany) following the instructions of the manufacturer^22^. The protocol was partially modified to use Lysing Matrix E Beads (MP Biomedicals, Irvine, CA, USA) with FastPrep-24 5G (MP Biomedicals) for three cycles at 6.0 m/s for 60 s instead of vortex mixing. Sequencing libraries were generated using NEBNext Ultra DNA Library Prep Kit for Illumina (NEB, Ipswich, MA, USA) according to manufacturer’s protocols, and index codes were added to attribute sequencing fragments to each sample. The library quality was assessed using TapeStation (Agilent, Santa Clara, CA, USA). Metagenomic sequencing was conducted using the HiSeq 2500 sequencing platform, HiSeq Rapid SBS Kit v2 and HiSeq PE Rapid Cluster Kit v2 (Illumina, San Diego, CA, USA) to obtain 3 Gbp per sample by 2 × 150 bp paired end-reads.

### Six previously reported shotgun-seq datasets for meta-analysis

We downloaded six previously reported shotgun-seq datasets that were available in December 2022 from Bonn, Germany (accession number, ERP019674)^12^; Shanghai, China (herein “China1”) (PRJNA433459)^13^; Xiangyang, China (herein “China2”) (PRJNA588035)^14^; Taipei, Taiwan (SUB10308106)^15^; Seoul, South Korea (PRJNA743718)^16^; and Birmingham, Alabama, USA (PRJNA834801)^17^ (Supplementary Tables 1 and 13). The total numbers of subjects including our own dataset were 813 PD patients and 558 controls. The average read counts per sample ranged from about nine to sixty million reads.

### Analysis of shotgun-seq

First, we conducted quality control with fastp (v0.23.4) and removed human genome (GRCh38) with bowtie2 (Version 2.5.0) using default parameters. We calculated CPM of each EC number with HUMAnN 3.6. We also calculated the relative abundance of each bacterium at the genus and species levels with MetaPhlAn 4. As the taxonomic databases in HUMAnN 3.6 and MetaPhlAn 4 were different, the two tools yielded different taxonomic names. We next calculated CPM of each gene in carbohydrate metabolisms in CAZymes. To this end, we generated contigs from short-read fragments with MEGAHIT (v1.2.9) using default parameters. The contigs were assigned to the CAZymes database with three sequence similarity search tools (HMMER, eCAMI, and DIAMOND) in dbCAN3 (https://github.com/linnabrown/run_dbcan). Contigs that were assigned to the CAZymes database by two or more tools were used in the downstream analysis. Short reads were then mapped to the CAZymes-assigned contigs with bowtie 2 to calculate the number of mapped reads per contig. Duplicated mapped reads were removed by MarkDuplicates in the Picard (3.1.0) tool kit (https://broadinstitute.github.io/picard/) using default parameters. The number of mapped reads per contig was normalized to CPM.

### Meta-analysis of six datasets

The experimental methods and demographic features are indicated in Supplementary Table 1. The dataset from Korea^16^ was excluded from our meta-analysis, because a single EC number 2.1.1.43 constituted 25% or more of total CPM in 96 out of 156 samples (61.5%).

We performed PCoA of bacterial species to show the difference between controls and PD in six datasets. To remove batch effects in six datasets, we applied MMUPHin^52^ to bacterial species in six datasets, which were generated by MetaPhlAn 4^53^. In MMUPHin analysis, the disease state (PD or control) was set to a covariate and the differences in bacterial abundances across datasets were set to batch effects. PCoA was plotted with the function of vegdist in vegan (version 2.6-4) on R 4.2.3 using Bray–Curtis dissimilarity as a distance measurement and with the function of cmdscale in stats (version 3.6.2) on R 4.2.3. PERMANOVA was performed with the function of adonis2 in vegan (version 2.6-4) on R 4.2.3.

EC numbers in HUMAnN 3.6, taxa at the genus and species levels in MetaPhlAn 4, and CAZymes were filtered under the following conditions. For each EC number, each taxon, and each CAZyme in each dataset, we counted the number of samples in which the CPM of the EC number, the relative taxonomic abundance, and the CPM of the CAZyme was not 0, respectively. We next filtered EC numbers, taxa, and CAZymes, in which the number of such samples constituted more than 20% in each dataset. We thereby chose 1678 EC numbers, 129 species, 84 genera, and 219 CAZymes that exceeded the threshold of 20% in all the datasets. We previously reported a method to meta-analyze non-parametric 16S rRNA gene sequencing datasets for gut microbiota in PD in five countries^8^. The same meta-analysis method was employed for the six shotgun-seq datasets. For each EC number, species, genus, and CAZyme, the numeric data was transformed into the effect size between controls and PD in order to remove the batch effects arising from the differences in countries, storage methods, stool DNA stabilizer, and so on. A weight was applied to each dataset so that each dataset equally contributed to the meta-analysis. We also meta-analyzed α-diversity in six datasets with Shannon index using all unfiltered species with R (4.2.3) package, phyloseq (version 1.42.0). In the meta-analysis of EC numbers, an EC number was considered to be significant when *p*-values were less than 0.05 after Bonferroni correction for both fixed effect model (FEM) and random effect model (REM), and *I*^*2*^ representing heterogeneity was less than 40%^54^. In the meta-analyses of taxa and CAZymes, a taxon or a CAZyme was considered to be significant when false-discovery rate (*q*-value) by Benjamini–Hochberg method for both FEM and REM was less than 0.05.

### Confounding factor analysis

The metadata were available for our dataset, the USA dataset^17^, and the German dataset^12^. Six features (age, BMI, constipation, disease state, sex, and anticholesterol drugs), which were available for both the Japan and USA datasets but not for the German dataset, were set to possible confounding factors. Confounding factors in the two datasets were analyzed with CPLM (Compound Poisson Linear Model) in MaAsLin2^21^ on R 4.2.3 without any normalization or transformation for each of 73 EC numbers, 31 genera, and 38 species, that were significantly different between controls and PD in meta-analysis. The difference between the USA and Japan was set to a random effect in the MaAsLin2 analysis. The total number of samples analyzed with MaAsLin2 was 851 due to lack of the metadata. *P*-values between controls and PD were normalized by the Benjamini–Hochberg method for each analysis of 73 EC numbers, 31 genera, and 38 species. Statistical significance was set to *q*-value < 0.05.

### Pathway analysis

Two types of pathway analyses were performed with KEGG pathway database (2020-1-14)^55^. One was the pathway enrichment analysis with hypergeometric test (the “dhyper” functionality on R 4.2.3), and the other was GSEA (v4.3.2 for Windows). In hypergeometric test, the number of significantly changed EC numbers in meta-analysis was evaluated for each pathway, while background genes were appropriately adjusted for^56^. In GSEA analysis, we followed a previously reported method^57^. Briefly, *p*-value of each pathway in each dataset was first calculated by GSEA. An integrated *p*-value was then calculated for each pathway. In the analysis of a KEGG pathway that was decreased in PD, actual *p*-value was used when the pathway was decreased in PD in a dataset, but *p*-value was set to 1.00 when the pathway was increased in PD in a dataset. In the analysis of a KEGG pathway that was increased in PD, a similar rule was applied in an opposite way. A significance threshold of *q*-value by Benjamini–Hochberg method was set to 0.05.

### Quantification of fecal SCFAs and polyamines

We previously established a GC-MS-based method to quantify fecal SCFA levels^22^. Briefly, 20 mg of FD feces was mixed with 1000 µL of 5 mmol/L NaOH and 300 µL of H_2_O. The mixture was shaken vigorously with zirconia beads for 10 min and centrifuged at 13,200 *×* *g* for 20 min at 4°C. The supernatant (333 µL) was mixed with 200 µL of H_2_O, 50 µL hexanoic-6,6,6-d3 acid solution (internal standard), 200 µL of 2-methyl-1-propanol, 133 µL of pyridine, and 67 µL of isobutyl chloroformate. The resulting solution was then mixed for 1 min. Subsequently, the mixture was added to 0.3 mL of hexane, shaken vigorously for 10 min, and centrifuged at 1900 *×* *g* for 5 min at room temperature. Quantitative analysis was performed using an Agilent 7890A GC equipped with an Agilent 5975 inert mass spectrometer (Agilent Technologies). The precisions for all SCFA quantifications were less than 8.4% relative standard deviation (RSD).

We also reported a GC-MS-based method to quantify fecal succinate and lactate levels^23^. Briefly, 20 mg of FD feces was mixed with 400 μL of H_2_O, 10 μL of sulfosalicylic acid solution (1 mg/μL), and an internal standard solution (hexanoic-6,6,6-d3 acid). The mixture was shaken vigorously with zirconia beads for 10 min and centrifuged at 13,200 *×* *g* for 20 min at 4 °C. The supernatant was then mixed with 20 μL of 6 mol/L HCl and 2 ml μL of diethyl ether. The sample was centrifuged again, and the upper layer was transferred into a new glass test tube. The sample was dried by nitrogen gas at 40 °C. The residue was dissolved in 300 μL diethyl ether. After adding 20 μL N-tert-butyldimethylsilyl imidazole, the derivatization reaction was performed at 60 °C for 30 min. Finally, the solution was analyzed using GC-MS as described above. The precisions for quantifications of lactate and succinate were less than 16% RSD and 2% RSD, respectively.

Fecal polyamines (putrescine, spermidine, and spermine) were quantified by a LC-MS/MS according to a protocol proposed by Xiong et al.^58^. Briefly, after derivatization using *N*-(9-fluorenylmeth-oxycarbonyloxy) succinimide, polyamines were analyzed with LC-MS/MS, which was composed of an Agilent 1200 infinity LC coupled with an Agilent 6430 Triple Quadrupole LC/MS System (Agilent Technologies). 1,6-diaminohexane was used as an internal standard. The precisions for all polyamine quantifications were less than 4.9% RSD.

### Fractional CPM contributed by each bacterium for each EC number

In order to examine the bacterial origins of nine significantly decreased EC numbers in “riboflavin metabolism” and “biotin metabolism”, we calculated a fractional CPM contributed by each bacterium for each EC number. For each bacterium for each EC number, the median of fractional CPMs in controls was subtracted from the median of fractional CPMs in PD to calculate ΔfCPM. We assumed that a bacterium with the most negative ΔfCPM mostly accounted for the decreased EC number in a specific dataset. For each bacterium in each EC number, ΔfCPMs were averaged in the six datasets. For each EC number, the averaged ΔfCPMs were sorted in ascending order and the top five bacteria were selected.

### Statistical analysis

Relative abundances of intestinal bacteria were analyzed by the Wilcoxon rank sum test for unmatched pairs with the mannwhitneyu functionality of scipy.stat on Python 3.11.2. In multiple comparison analyses, we set three different thresholds. In the meta-analysis of EC numbers, the significance threshold for *p*-value after Bonferroni correction was set to less than 0.05 for both FEM and REM and the significance threshold of *I*^*2*^ was set to less than 40%. In the meta-analyses of taxa and CAZymes, the significance threshold for *q*-value by Benjamini–Hochberg method was set to less than 0.05 for both FEM and REM. In confounding factor analysis, the significance threshold for *q*-values by Benjamini–Hochberg method was set to less than 0.05.

### Supplementary information

Supplemental Material

reporting-summary

## Data Availability

Sequence data of our dataset are available at the DNA Data Bank of Japan (DDBJ) under the accession number of DRA016410.
