# Supplementary material for: Meta-analysis of shotgun sequencing of gut microbiota in Parkinson’s disease
Source: NPJ Parkinsons Dis. 2024 May 21;10:106. doi: 10.1038/s41531-024-00724-z (PMC11109112; doi:10.1038/s41531-024-00724-z)
Supplement: Supplementary file 1 — Supplemental Material [file 41531_2024_724_MOESM1_ESM.pdf]

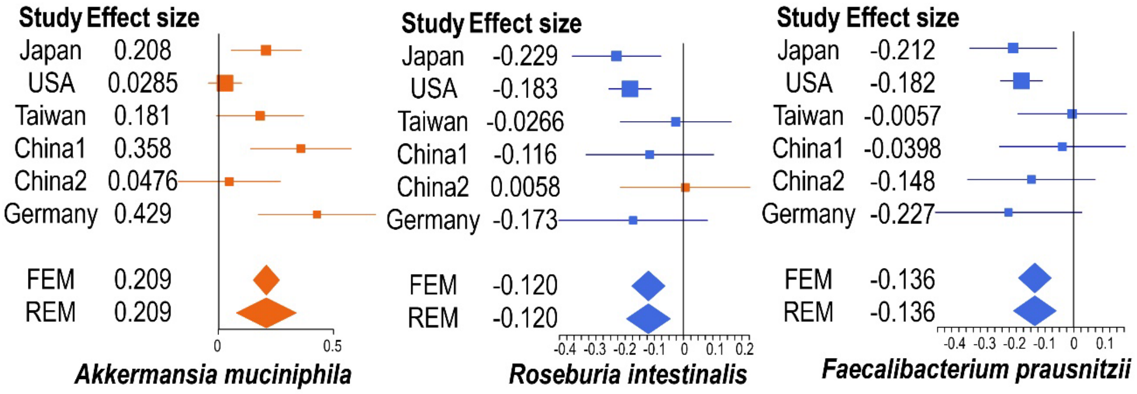

Supplementary Figure 1. Forest plot of species *Akkermansia muciniphila*, *Roseburia intestinalis*, and *Faecalibacterium prausnitzii*, all of which were significantly changed in our previous meta-analysis of five countries at the genus level, in six datasets. Orange and blue symbols represent increased and decreased species in PD. The square sizes represent the number of samples. Bars represent 95% confidence interval. Diamonds represent the mean and 95% confidence interval.

Supplementary Table 1. Previous reports and our current study of shotgun metagenome sequencing of gut microbiota in Parkinson's disease

	First author	City, Country	doi	Accession number	Date	#PD	#Controls	Transportation temperature	Storage method	Stool DNA stabilizer	Disease duration (mean ± SD, years)	Hoehn and Yahr scale (mean ± SD)
1	Bedarf[1]	Bonn, Germany	10.1186/s13073-017-0428-y	ERP019674	Apr, 2017	31	28	Ambient temperature	n.a.	no	n.a.	n.a.
2	Qian[2]	Shanghai, China	10.1093/brain/awaa201	PRJNA433459	Aug, 2020	40	40	On ice	-80°C	no	6.7 ± 4.6	2.3 ± 0.8
3	Mao[3]	Xiangyang, China	10.3389/fmicb.2021.728479	PRJNA588035	Sep, 2021	39	39	Ambient temperature	-20°C	no	n.a.	1.9 ± 0.9
4	Chen[4]	Taipei, Taiwan	10.1212/WNL.0000000000013225	SUB10308106	Feb, 2022	58	49	Ambient temperature	-80°C	yes	6.1 ± 5.1	2.3 ± 1.2
[a]5	Jo[5]	Seoul, South Korea	10.1038/s41531-022-00351-6	PRJNA743718	Jul, 2022	82	74	Ambient temperature	n.a	no	[b]2.0 (0.0-6.0)	[b]2.0 (2.0-3.0)
6	Wallen[6]	Alabama, USA	10.1038/s41467-022-34667-x	PRJNA834801	Nov, 2022	490	234	Ambient temperature	-20°C	yes	n.a.	n.a.
[c]7	Boktor[7]	Illinois, USA	10.1002/mds.29300	ERP138197, ERP138199	Jan, 2023	95	149	Ambient temperature	-80°C	yes	[b]7.0 (5.0-11.0)	[b]2.0 (2.0-2.0)
[c]8	Palacios[8]	Massachusetts, USA	10.1002/ana.26719	-	June, 2023	75	131	Ambient temperature	-80°C	yes	n.a.	n.a.
9	Nishiwaki	Nagoya, Japan	Current study	DRA016410	Current Study	94	73	On ice	Freeze-dried	no	8.0 ± 5.9	2.78 ± 0.94

n.a., not available.

[a]Not included in our meta-analysis, because a single EC number 2.1.1.43 constituted 25% or more of total CPM in 96 out of 156 subjects (61.5%).

[b]median (interquartile range)

[c]These studies were not included in our meta-analysis because the datasets were not able to obtain by the end of December 2022.

References

1. Bedarf JR, Hildebrand F, Coelho LP, et al. Functional implications of microbial and viral gut metagenome changes in early stage L-DOPA-naive Parkinson's disease patients. Genome Med. 2017;9:39.
2. Qian Y, Yang X, Xu S, et al. Gut metagenomics-derived genes as potential biomarkers of Parkinson's disease. Brain. 2020;143:2474-2489.
3. Mao L, Zhang Y, Tian J, et al. Cross-Sectional Study on the Gut Microbiome of Parkinson's Disease Patients in Central China. Front Microbiol. 2021;12:728479.
4. Chen SJ, Chen CC, Liao HY, et al. Association of Fecal and Plasma Levels of Short-Chain Fatty Acids With Gut Microbiota and Clinical Severity in Patients With Parkinson Disease. Neurology. 2022;98:e848-e858.
5. Jo S, Kang W, Hwang YS, et al. Oral and gut dysbiosis leads to functional alterations in Parkinson's disease. NPJ Parkinsons Dis. 2022;8:87.
6. Wallen ZD, Demirkan A, Twa G, et al. Metagenomics of Parkinson's disease implicates the gut microbiome in multiple disease mechanisms. Nat Commun. 2022;13:6958.
7. Boktor JC, Sharon G, Verhagen Metman LA, et al. Integrated Multi-Cohort Analysis of the Parkinson's Disease Gut Metagenome. Mov Disord. 2023;38:399-409.
8. Palacios N, Wilkinson J, Bjornevik K, et al. Metagenomics of the Gut Microbiome in Parkinson's Disease: Prodromal Changes. Ann Neurol. 2023;94:486-501.

Supplementary Table 2. PERMANOVA to examine the effect of disease status and the difference of countries on the overall bacterial composition in six datasets

Between PD and control	*p* -value
Japan	*2.00E-6
USA	*1.00E-7
Taiwan	*0.040
China1	*2.98E-3
China2	*0.0116
Gemany	*0.046
**Between each country**	***p* -value**
Japan vs USA	*1.00E-7
Japan vs Taiwan	*2.00E-5
Japan vs China1	*1.00E-7
Japan vs China2	*1.00E-7
Japan vs Germany	*1.00E-7
USA vs Taiwan	*1.00E-7
USA vs China1	*1.00E-7
USA vs China2	*1.00E-7
USA vs Germany	*1.00E-7
Taiwan vs China1	*6.00E-6
Taiwan vs China2	*7.00E-5
Taiwan vs Gemany	*1.00E-4
China1 vs China2	0.626
China1 vs Germany	*1.00E-7
China2 vs Germany	*2.00E-7

P -values are calculated by Bray-Curtis.

**P* -value < 0.05

Supplementary Table 3. Meta-analysis of EC numbers in six datasets

[a]EC number	[a]Enzyme	Increased or decreased in PD	[b]KEGG Pathway	[c]*p*-value (FEM)	[c]*p*-value (REM)	[c]I^2 (%)
**1.1.1.193**	**5-amino-6-(5-phosphoribosylamino)uracil reductase**	-	**Riboflavin metabolism**; Metabolic pathways; Biosynthesis of secondary metabolites	*< 1.0E-16	*< 1.0E-16	*0.0
**2.6.1.83**	**LL-diaminopimelate aminotransferase**	-	Lysine biosynthesis; Metabolic pathways; Biosynthesis of secondary metabolites; Biosynthesis of antibiotics	*< 1.0E-16	*< 1.0E-16	*0.0
**3.5.4.26**	**diaminohydroxyphosphoribosylaminopyrimidine deaminase**	-	**Riboflavin metabolism**; Metabolic pathways; Biosynthesis of secondary metabolites	*< 1.0E-16	*< 1.0E-16	*0.0
**3.5.4.31**	**S-methyl-5'-thioadenosine deaminase**	+	Cysteine and methionine metabolism; Metabolic pathways	*< 1.0E-16	*< 1.0E-16	*0.0
**4.1.1.19**	**arginine decarboxylase**	-	**Arginine and proline metabolism**; Metabolic pathways	*2.22E-16	*2.22E-16	*0.0
**3.1.3.71**	**2-phosphosulfolactate phosphatase**	+	Methane metabolism; Microbial metabolism in diverse environments	*2.22E-16	*8.88E-16	*6.1
**3.5.4.28**	**S-adenosylhomocysteine deaminase**	+	Cysteine and methionine metabolism, Metabolic pathways	*< 1.0E-16	*1.55E-15	*9.7
**4.1.1.11**	**aspartate 1-decarboxylase**	-	beta-Alanine metabolism; Pantothenate and CoA biosynthesis; Metabolic pathways; Biosynthesis of secondary metabolites	*3.33E-15	*3.33E-15	*0.0
**4.1.1.65**	**phosphatidylserine decarboxylase**	-	Glycerophospholipid metabolism; Metabolic pathways; Biosynthesis of secondary metabolites	*5.84E-14	*5.84E-14	*0.0
**3.2.1.89**	**arabinogalactan endo-beta-1,4-galactanase**	-		*7.44E-14	*7.44E-14	*0.0
**5.3.1.24**	**phosphoribosylanthranilate isomerase**	-	Phenylalanine, tyrosine and tryptophan biosynthesis; Metabolic pathways; Biosynthesis of secondary metabolites; Biosynthesis of antibiotics	*1.70E-12	*1.70E-12	*0.0
3.3.1.1	adenosylhomocysteinase	+	Cysteine and methionine metabolism; Metabolic pathways	*< 1.0E-16	*1.92E-12	44.9
**1.2.7.3**	**2-oxoglutarate synthase**	+	Citrate cycle (TCA cycle); Carbon fixation pathways in prokaryotes; Metabolic pathways; Microbial metabolism in diverse environments	*6.66E-16	*1.97E-12	*24.4
**5.3.3.3**	**vinylacetyl-CoA Delta-isomerase**	+	Butanoate metabolism	*1.24E-14	*5.03E-12	*19.8
**3.5.4.43**	**hydroxydechloroatrazine ethylaminohydrolase**	+	Atrazine degradation; Metabolic pathways; Microbial metabolism in diverse environments	*6.17E-12	*6.17E-12	*0.0
**1.8.4.12**	**peptide-methionine (R)-S-oxide reductase**	+		*8.10E-12	*8.10E-12	*0.0
**3.1.26.11**	**tRNase Z**	-		*1.21E-11	*1.21E-11	*0.0
4.1.1.32	phosphoenolpyruvate carboxykinase (GTP)	+	Glycolysis / Gluconeogenesis; Citrate cycle (TCA cycle); Pyruvate metabolism; Metabolic pathways; Biosynthesis of secondary metabolites; Microbial metabolism in diverse environments; Biosynthesis of antibiotics	*< 1.0E-16	*1.97E-11	52.8
**3.5.99.6**	**glucosamine-6-phosphate deaminase**	-	Amino sugar and nucleotide sugar metabolism; Metabolic pathways	*8.62E-14	*2.34E-11	*19.8
**4.3.1.14**	**3-aminobutyryl-CoA ammonia-lyase**	+	Lysine degradation	*4.87E-11	*4.88E-11	*0.0
**1.12.1.2**	**hydrogen dehydrogenase**	+		*3.11E-15	*6.63E-11	*31.5
**2.8.1.6**	**biotin synthase**	-	**Biotin metabolism**; Metabolic pathways	*8.19E-11	*8.19E-11	*0.0
1.8.98.1	dihydromethanophenazine:CoB-CoM heterodisulfide reductase	+	Methane metabolism; Metabolic pathways; Microbial metabolism in diverse environments	*< 1.0E-16	*9.48E-11	40.2
2.2.1.10	2-amino-3,7-dideoxy-D-threo-hept-6-ulosonate synthase	+	Phenylalanine, tyrosine and tryptophan biosynthesis; Biosynthesis of secondary metabolites; Biosynthesis of antibiotics	*< 1.0E-16	*1.71E-10	52.8
7.1.3.1	H+-exporting diphosphatase	+		*< 1.0E-16	*2.58E-10	63.1
**5.4.3.3**	**lysine 5,6-aminomutase**	+	Lysine degradation	*2.22E-15	*3.00E-10	*36.9
**4.1.99.12**	**3,4-dihydroxy-2-butanone-4-phosphate synthase**	-	**Riboflavin metabolism**; Metabolic pathways; Biosynthesis of secondary metabolites	*3.54E-10	*3.54E-10	*0.0
**3.5.3.12**	**agmatine deiminase**	-	**Arginine and proline metabolism**; Metabolic pathways	*3.69E-10	*3.69E-10	*0.0
**2.5.1.10**	**(2E,6E)-farnesyl diphosphate synthase**	-	Terpenoid backbone biosynthesis; Metabolic pathways; Biosynthesis of secondary metabolites; Biosynthesis of antibiotics	*6.70E-10	*6.70E-10	*0.0
**2.4.2.7**	**adenine phosphoribosyltransferase**	-	Purine metabolism; Metabolic pathways	< 1.0E-16	*1.76E-09	52.6
**4.1.99.1**	**tryptophanase**	+	Tryptophan metabolism	*1.75E-09	*2.04E-09	*0.8
**4.2.1.33**	**3-isopropylmalate dehydratase**	-	Valine, leucine and isoleucine biosynthesis; Metabolic pathways; Biosynthesis of secondary metabolites	*8.95E-10	*3.17E-09	*6.6
**1.7.1.13**	**preQ1 synthase**	-	**Folate biosynthesis**; Metabolic pathways	*9.81E-14	*3.60E-09	*37.1
**2.1.1.223**	**tRNA1Val (adenine37-N6)-methyltransferase**	-		*5.24E-12	*3.78E-09	*27.0
**3.6.1.17**	**bis(5'-nucleosyl)-tetraphosphatase (asymmetrical)**	+	Purine metabolism; Pyrimidine metabolism	*4.25E-09	*4.25E-09	*0.0
**1.1.1.77**	**lactaldehyde reductase**	-	Glyoxylate and dicarboxylate metabolism; Propanoate metabolism; Microbial metabolism in diverse environments	*5.05E-09	*5.06E-09	*0.0
**2.1.1.197**	**malonyl-[acyl-carrier protein] O-methyltransferase**	-	**Biotin metabolism**; Metabolic pathways	*1.46E-10	*6.10E-09	*17.7
2.7.4.9	dTMP kinase	+	Pyrimidine metabolism; Metabolic pathways	< 1.0E-16	*6.12E-09	65.3
**6.3.4.20**	**7-cyano-7-deazaguanine synthase**	-	**Folate biosynthesis**; Metabolic pathways	*2.22E-12	*6.33E-09	*31.6
**4.2.1.8**	**mannonate dehydratase**	-	Pentose and glucuronate interconversions; Metabolic pathways	*1.58E-12	*7.15E-09	*32.9
3.2.2.1	purine nucleosidase	+	Purine metabolism; Nicotinate and nicotinamide metabolism; Metabolic pathways	*1.11E-15	*9.28E-09	48.6
6.4.1.6	acetone carboxylase	+		*9.55E-15	*1.56E-08	46.7
**4.1.99.2**	**tyrosine phenol-lyase**	+	Tyrosine metabolism	*6.75E-10	*1.83E-08	*16.9
**2.4.1.293**	**GalNAc5-diNAcBac-PP-undecaprenol beta-1,3-glucosyltransferase**	+		*3.71E-10	*3.20E-08	*22.1
**4.2.1.120**	**4-hydroxybutanoyl-CoA dehydratase**	+	Butanoate metabolism; Carbon fixation pathways in prokaryotes; Metabolic pathways; Microbial metabolism in diverse environments	*3.31E-08	*3.31E-08	*0.0
**4.2.1.151**	**chorismate dehydratase**	+	Ubiquinone and other terpenoid-quinone biosynthesis; Biosynthesis of secondary metabolites	*8.50E-09	*3.47E-08	*8.2
**3.5.4.25**	**GTP cyclohydrolase II**	-	**Riboflavin metabolism**; **Folate biosynthesis**; Metabolic pathways; Biosynthesis of secondary metabolites	*3.61E-08	*3.61E-08	*0.0
**1.1.1.272**	**D-2-hydroxyacid dehydrogenase (NADP+)**	+		*1.38E-08	*5.00E-08	*7.7
**5.4.99.1**	**methylaspartate mutase**	+	Glyoxylate and dicarboxylate metabolism; C5-Branched dibasic acid metabolism; Metabolic pathways	*3.14E-10	*5.03E-08	*25.0
3.1.3.11	fructose-bisphosphatase	-	Glycolysis / Gluconeogenesis; Pentose phosphate pathway; Fructose and mannose metabolism; Methane metabolism; Carbon fixation in photosynthetic organisms; Metabolic pathways; Biosynthesis of secondary metabolites; Microbial metabolism in diverse environments; Biosynthesis of antibiotics	< 1.0E-16	*5.24E-08	62.6
**5.4.3.6**	**tyrosine 2,3-aminomutase**	+	Tyrosine metabolism; Biosynthesis of enediyne antibiotics; Biosynthesis of antibiotics	*7.31E-08	*7.31E-08	*0.0
**4.2.1.135**	**UDP-N-acetylglucosamine 4,6-dehydratase (configuration-retaining)**	+	Amino sugar and nucleotide sugar metabolism	*1.83E-08	*7.80E-08	*8.9
4.1.99.17	phosphomethylpyrimidine synthase	-	Thiamine metabolism; Metabolic pathways	*9.81E-13	*8.98E-08	43.8
**6.1.1.2**	**tryptophan---tRNA ligase**	+	Aminoacyl-tRNA biosynthesis	*9.11E-08	*9.11E-08	*0.0
**1.1.1.57**	**fructuronate reductase**	-	Pentose and glucuronate interconversions; Metabolic pathways	*4.24E-08	*2.36E-07	*11.1
**1.7.7.1**	**ferredoxin---nitrite reductase**	-	Nitrogen metabolism; Microbial metabolism in diverse environments	*2.58E-07	*2.58E-07	*0.0
**3.1.3.41**	**4-nitrophenylphosphatase**	+	Aminobenzoate degradation; Microbial metabolism in diverse environments	*2.69E-10	*2.77E-07	*33.8
2.1.3.9	N-acetylornithine carbamoyltransferase	-	Arginine biosynthesis; Metabolic pathways	*9.29E-13	*2.90E-07	48.4
7.3.2.2	ABC-type phosphonate transporter	+		*3.07E-13	*3.00E-07	50.6
**2.4.1.281**	**4-O-beta-D-mannosyl-D-glucose phosphorylase**	-		*1.27E-09	*3.87E-07	*30.1
**2.7.1.5**	**rhamnulokinase**	-	Pentose and glucuronate interconversions; Fructose and mannose metabolism; Microbial metabolism in diverse environments	*5.88E-10	*4.39E-07	*33.5
2.3.1.179	beta-ketoacyl-[acyl-carrier-protein] synthase II	-	Fatty acid biosynthesis; **Biotin metabolism**; Metabolic pathways	< 1.0E-16	*4.86E-07	65.6
**1.21.98.1**	**cyclic dehypoxanthinyl futalosine synthase**	+	Ubiquinone and other terpenoid-quinone biosynthesis; Biosynthesis of secondary metabolites	*6.49E-07	*6.50E-07	*0.0
**1.18.1.6**	**adrenodoxin-NADP+ reductase**	+		*7.03E-07	*7.03E-07	*0.0
**6.3.2.1**	**pantoate---beta-alanine ligase (AMP-forming)**	-	beta-Alanine metabolism; Pantothenate and CoA biosynthesis; Metabolic pathways; Biosynthesis of secondary metabolites	*4.30E-07	*9.72E-07	*6.1
1.2.99.5	formylmethanofuran dehydrogenase	+	Methane metabolism, Metabolic pathways, Microbial metabolism in diverse environments	*1.69E-12	*1.05E-06	52.1
**3.2.1.23**	**beta-galactosidase**	-	Galactose metabolism; Other glycan degradation; Glycosaminoglycan degradation; Sphingolipid metabolism; Glycosphingolipid biosynthesis - ganglio series; Metabolic pathways	*1.70E-08	*1.26E-06	*26.2
**3.6.3.28**	**phosphonate-transporting ATPase**	+		*5.26E-07	*1.41E-06	*7.5
**2.6.1.50**	**glutamine---scyllo-inositol transaminase**	+	Streptomycin biosynthesis; Biosynthesis of antibiotics	*3.99E-08	*1.42E-06	*22.9
3.5.1.25	N-acetylglucosamine-6-phosphate deacetylase	-	Galactose metabolism; Amino sugar and nucleotide sugar metabolism; Biosynthesis of antibiotics	*4.26E-10	*1.43E-06	40.4
3.1.3.48	protein-tyrosine-phosphatase	-		*2.49E-14	*1.44E-06	60.0
3.4.21.26	prolyl oligopeptidase	+		*2.22E-16	*1.51E-06	66.1
1.4.1.16	diaminopimelate dehydrogenase	-	Lysine biosynthesis; Metabolic pathways; Biosynthesis of secondary metabolites	*6.64E-12	*1.62E-06	51.2
**2.7.6.2**	**thiamine diphosphokinase**	-	Thiamine metabolism; Metabolic pathways	*1.19E-07	*1.72E-06	*18.4
**2.6.1.62**	**adenosylmethionine---8-amino-7-oxononanoate transaminase**	-	**Biotin metabolism**; Metabolic pathways	*1.18E-08	*2.18E-06	*31.0
**2.1.2.11**	**3-methyl-2-oxobutanoate hydroxymethyltransferase**	-	Pantothenate and CoA biosynthesis; Metabolic pathways; Biosynthesis of secondary metabolites	*5.31E-07	*2.24E-06	*11.0
2.7.10.2	non-specific protein-tyrosine kinase	-		*1.13E-09	*2.62E-06	40.5
3.5.1.11	penicillin amidase	+	Penicillin and cephalosporin biosynthesis; Biosynthesis of antibiotics	*2.22E-15	*3.11E-06	65.4
**1.12.7.2**	**ferredoxin hydrogenase**	-		*9.86E-07	*4.79E-06	*12.7
2.7.1.95	kanamycin kinase	+		*1.23E-10	*4.80E-06	49.5
**3.4.13.19**	**membrane dipeptidase**	-		*6.81E-07	*4.86E-06	*15.3
**2.3.2.6**	**lysine/arginine leucyltransferase**	-		*5.19E-07	*5.21E-06	*17.6
**2.7.1.22**	**ribosylnicotinamide kinase**	-	Nicotinate and nicotinamide metabolism; Metabolic pathways	*5.43E-06	*5.43E-06	*0.0
3.1.3.2	acid phosphatase	-	**Riboflavin metabolism**; Metabolic pathways	*9.00E-11	*5.50E-06	50.9
2.4.1.157	1,2-diacylglycerol 3-glucosyltransferase	+	Glycerolipid metabolism, Metabolic pathways	*9.69E-10	*5.60E-06	44.8
3.5.1.77	N-carbamoyl-D-amino-acid hydrolase	-		*2.29E-13	*5.84E-06	61.8
**2.5.1.78**	**6,7-dimethyl-8-ribityllumazine synthase**	-	**Riboflavin metabolism**; Metabolic pathways; Biosynthesis of secondary metabolites	*1.07E-08	*6.17E-06	*37.5
5.3.1.14	L-rhamnose isomerase	-	Fructose and mannose metabolism; Microbial metabolism in diverse environments	*4.24E-09	*6.41E-06	41.0
**3.2.1.8**	**endo-1,4-beta-xylanase**	-		*6.70E-06	*6.70E-06	*0.0
3.5.3.6	arginine deiminase	+	Arginine biosynthesis; Metabolic pathways; Biosynthesis of secondary metabolites; Biosynthesis of antibiotics	*6.61E-12	*7.06E-06	57.2
2.7.7.53	ATP adenylyltransferase	+	Purine metabolism	*2.98E-09	*7.44E-06	42.9
**5.4.99.9**	**UDP-galactopyranose mutase**	-	Galactose metabolism; Amino sugar and nucleotide sugar metabolism	*2.81E-07	*7.53E-06	*24
**3.2.1.20**	**alpha-glucosidase**	+	Galactose metabolism; Starch and sucrose metabolism; Metabolic pathways	*1.90E-08	*8.27E-06	*37.1
**2.4.1.305**	**UDP-Glc:alpha-D-GlcNAc-glucosaminyl-diphosphoundecaprenol beta-1,3-glucosyltransferase**	+		*8.35E-06	*8.35E-06	*0.0
6.3.5.4	asparagine synthase (glutamine-hydrolysing)	-	Alanine, aspartate and glutamate metabolism; Metabolic pathways; Biosynthesis of secondary metabolites	*5.14E-13	*1.01E-05	62.6
**3.8.1.5**	**haloalkane dehalogenase**	+	Chlorocyclohexane and chlorobenzene degradation; Chloroalkane and chloroalkene degradation; Metabolic pathways; Microbial metabolism in diverse environments	*1.02E-05	*1.02E-05	*0.0
**2.1.1.156**	**glycine/sarcosine N-methyltransferase**	-	Glycine, serine and threonine metabolism	*4.00E-07	*1.03E-05	*24.3
2.7.7.4	sulfate adenylyltransferase	-	Purine metabolism; Monobactam biosynthesis; Selenocompound metabolism; Sulfur metabolism; Metabolic pathways; Microbial metabolism in diverse environments; Biosynthesis of antibiotics	*2.50E-10	*1.08E-05	51.6
5.4.99.2	methylmalonyl-CoA mutase	+	Valine, leucine and isoleucine degradation; Glyoxylate and dicarboxylate metabolism; Propanoate metabolism; Carbon fixation pathways in prokaryotes; Metabolic pathways; Microbial metabolism in diverse environments	*3.74E-09	*1.11E-05	44.5
3.5.4.30	dCTP deaminase (dUMP-forming)	+	Pyrimidine metabolism	*8.11E-11	*1.20E-05	54.6
2.1.1.74	methylenetetrahydrofolate---tRNA-(uracil54-C5)-methyltransferase [NAD(P)H-oxidizing]	+		*8.06E-13	*1.22E-05	62.7
**1.1.1.163**	**cyclopentanol dehydrogenase**	+	Caprolactam degradation	*1.28E-05	*1.28E-05	*0.0
3.6.3.34	iron-chelate-transporting ATPase	-		*7.69E-09	*1.32E-05	43.1
**3.4.17.4**	**Gly-Xaa carboxypeptidase**	-		*1.33E-05	*1.33E-05	*0.0
**4.1.2.43**	**3-hexulose-6-phosphate synthase**	+	Pentose phosphate pathway; Methane metabolism; Metabolic pathways; Microbial metabolism in diverse environments	*2.40E-08	*1.40E-05	*39.4
2.7.7.56	tRNA nucleotidyltransferase	+		*8.44E-12	*1.77E-05	60.5
1.2.1.38	N-acetyl-gamma-glutamyl-phosphate reductase	-	Arginine biosynthesis; Metabolic pathways; Biosynthesis of secondary metabolites; Biosynthesis of antibiotics	*1.24E-11	*2.04E-05	60.5
2.1.1.192	23S rRNA (adenine2503-C2)-methyltransferase	+		*1.35E-08	*2.22E-05	44.2
**4.2.1.59**	**3-hydroxyacyl-[acyl-carrier-protein] dehydratase**	-	Fatty acid biosynthesis; **Biotin metabolism**; Metabolic pathways	*4.46E-06	*2.51E-05	*15.7
4.4.1.21	S-ribosylhomocysteine lyase	-	Cysteine and methionine metabolism; Metabolic pathways	*5.22E-14	*2.52E-05	68.7
**4.1.3.3**	**N-acetylneuraminate lyase**	-	Amino sugar and nucleotide sugar metabolism	*2.39E-06	*2.59E-05	*20.5
5.3.1.23	S-methyl-5-thioribose-1-phosphate isomerase	+	Cysteine and methionine metabolism; Metabolic pathways	*7.99E-15	*2.67E-05	70.7
2.7.1.105	6-phosphofructo-2-kinase	+	Fructose and mannose metabolism	*8.76E-09	*2.75E-05	46.9

1.3.99.31	phytoene desaturase (lycopene-forming)	+	Carotenoid biosynthesis; Metabolic pathways; Biosynthesis of secondary metabolites	*1.51E-06	3.10E-05	*25
6.3.1.20	lipoate---protein ligase	+	Lipoic acid metabolism; Metabolic pathways	*1.08E-09	3.26E-05	53.6
2.6.1.90	dTDP-3-amino-3,6-dideoxy-alpha-D-galactopyranose transaminase	+	Polyketide sugar unit biosynthesis; Biosynthesis of antibiotics	*2.52E-05	3.43E-05	*3.3
3.6.1.1	inorganic diphosphatase	+	Oxidative phosphorylation	*8.16E-13	3.48E-05	66.6
3.2.1.55	non-reducing end alpha-L-arabinofuranosidase	-	Amino sugar and nucleotide sugar metabolism	3.54E-05	3.54E-05	*0.0
2.1.1.132	precorrin-6B C5,15-methyltransferase (decarboxylating)	-	Porphyrin and chlorophyll metabolism; Metabolic pathways	*4.51E-07	3.61E-05	*33
4.6.1.13	phosphatidylinositol diacylglycerol-lyase	+	Inositol phosphate metabolism	3.76E-05	3.76E-05	*0.0
4.2.1.47	GDP-mannose 4,6-dehydratase	-	Fructose and mannose metabolism; Amino sugar and nucleotide sugar metabolism; Metabolic pathways	*6.88E-06	3.85E-05	*16.2
2.7.1.25	adenylyl-sulfate kinase	-	Purine metabolism; Sulfur metabolism; Metabolic pathways; Microbial metabolism in diverse environments	*2.19E-10	3.91E-05	58.0
3.2.1.135	neopullulanase	-		*1.55E-07	3.92E-05	*38.6
4.1.1.36	phosphopantothenoylcysteine decarboxylase	-	Pantothenate and CoA biosynthesis; Metabolic pathways	3.92E-05	3.93E-05	*0.0
6.3.1.1	aspartate---ammonia ligase	-	Alanine, aspartate and glutamate metabolism; Cyanoamino acid metabolism; Metabolic pathways; Biosynthesis of secondary metabolites	*4.63E-13	4.01E-05	67.8
3.2.1.22	alpha-galactosidase	-	Galactose metabolism; Glycerolipid metabolism; Sphingolipid metabolism; Glycosphingolipid biosynthesis - globo and isoglobo series	*2.15E-05	4.42E-05	*7.6
2.7.1.49	hydroxymethylpyrimidine kinase	-	Thiamine metabolism; Metabolic pathways	*1.09E-10	4.45E-05	60.0
2.7.1.11	6-phosphofructokinase	-	Glycolysis / Gluconeogenesis; Pentose phosphate pathway; Fructose and mannose metabolism; Galactose metabolism; Methane metabolism; Metabolic pathways; Biosynthesis of secondary metabolites; Microbial metabolism in diverse environments; Biosynthesis of antibiotics	< 1.0E-16	4.68E-05	77.3
3.2.1.45	glucosylceramidase	+	Other glycan degradation; Sphingolipid metabolism; Metabolic pathways	*7.78E-12	5.06E-05	64.9
3.1.3.37	sedoheptulose-bisphosphatase	+	Carbon fixation in photosynthetic organisms; Metabolic pathways; Microbial metabolism in diverse environments	*2.15E-05	5.21E-05	*9.3
2.1.1.173	23S rRNA (guanine2445-N2)-methyltransferase	-		*3.25E-06	5.34E-05	*24.6
6.3.2.31	coenzyme F420-0:L-glutamate ligase	+	Methane metabolism; Microbial metabolism in diverse environments	*1.46E-06	5.49E-05	*29.9
3.4.11.4	tripeptide aminopeptidase	-		*4.63E-08	5.51E-05	45.5
2.1.1.210	demethylspheroidene O-methyltransferase	+	Carotenoid biosynthesis; Metabolic pathways	5.63E-05	5.63E-05	*0.0
3.1.4.58	RNA 2',3'-cyclic 3'-phosphodiesterase	+		*4.00E-09	5.88E-05	53.4
2.5.1.76	cysteate synthase	+		*4.10E-13	5.92E-05	69.3
1.3.3.1	dihydroorotate oxidase	+		5.95E-05	5.95E-05	*0.0
2.8.3.16	formyl-CoA transferase	+		7.65E-05	7.65E-05	*0.0
2.4.1.247	beta-D-galactosyl-(1->4)-L-rhamnose phosphorylase	+		8.63E-05	8.63E-05	*0.0
3.2.2.27	uracil-DNA glycosylase	-		9.20E-05	9.20E-05	*0.0
3.6.3.8	Ca2+-transporting ATPase	-		*6.72E-10	9.49E-05	60.0
3.5.1.32	hippurate hydrolase	+	Phenylalanine metabolism	*1.60E-09	1.21E-04	59.4
1.20.4.1	arsenate reductase (glutathione/glutaredoxin)	-		1.22E-04	1.22E-04	*0.0
3.1.21.5	type III site-specific deoxyribonuclease	+		*8.01E-08	1.30E-04	49.2
4.2.1.42	galactarate dehydratase	-	Ascorbate and aldarate metabolism	*7.06E-08	1.34E-04	49.8
4.1.1.49	phosphoenolpyruvate carboxykinase (ATP)	-	Glycolysis / Gluconeogenesis; Citrate cycle (TCA cycle); Pyruvate metabolism; Carbon fixation in photosynthetic organisms; Metabolic pathways; Biosynthesis of secondary metabolites; Microbial metabolism in diverse environments; Biosynthesis of antibiotics	*1.74E-05	1.34E-04	*20.9
1.1.1.169	2-dehydropantoate 2-reductase	-	Pantothenate and CoA biosynthesis; Metabolic pathways; Biosynthesis of secondary metabolites	*2.32E-08	1.38E-04	53.4
3.2.1.41	pullulanase	-		*1.93E-05	1.42E-04	*20.8
3.1.3.15	histidinol-phosphatase	-	Histidine metabolism; Metabolic pathways; Biosynthesis of secondary metabolites	1.42E-04	1.42E-04	*0.0
1.1.1.58	tagaturonate reductase	-	Pentose and glucuronate interconversions; Metabolic pathways	*1.21E-08	1.50E-04	55.7
2.8.3.9	butyrate---acetoacetate CoA-transferase	+	Lysine degradation	*2.95E-06	1.56E-04	*34.6
2.1.1.13	methionine synthase	-	Cysteine and methionine metabolism; Selenocompound metabolism; One carbon pool by folate; Metabolic pathways; Biosynthesis of secondary metabolites	*3.05E-13	1.58E-04	73.1
2.5.1.29	geranylgeranyl diphosphate synthase	-	Terpenoid backbone biosynthesis; Metabolic pathways; Biosynthesis of secondary metabolites; Biosynthesis of antibiotics	*2.48E-09	1.58E-04	59.8
5.3.1.17	5-dehydro-4-deoxy-D-glucuronate isomerase	-	Pentose and glucuronate interconversions	*3.35E-09	1.61E-04	59.3
4.1.2.42	D-threonine aldolase	+		4.14E-05	1.67E-04	*15.7
1.1.1.301	D-arabitol-phosphate dehydrogenase	+		*4.83E-12	1.71E-04	70.4
1.5.1.20	methylenetetrahydrofolate reductase [NAD(P)H]	-	One carbon pool by folate; Carbon fixation pathways in prokaryotes; Metabolic pathways; Microbial metabolism in diverse environments	*9.91E-11	1.72E-04	66.3
2.2.1.9	2-succinyl-5-enolpyruvyl-6-hydroxy-3-cyclohexene-1-carboxylic-acid synthase	-	Ubiquinone and other terpenoid-quinone biosynthesis; Metabolic pathways; Biosynthesis of secondary metabolites	*1.51E-12	1.80E-04	72.0
5.1.3.4	L-ribulose-5-phosphate 4-epimerase	-	Pentose and glucuronate interconversions; Ascorbate and aldarate metabolism; Metabolic pathways; Microbial metabolism in diverse environments	*1.70E-11	1.85E-04	69.1
2.6.1.1	aspartate transaminase	-	Arginine biosynthesis; Alanine, aspartate and glutamate metabolism; Cysteine and methionine metabolism; **Arginine and proline metabolism**; Tyrosine metabolism; Phenylalanine metabolism; Phenylalanine, tyrosine and tryptophan biosynthesis; Novobiocin biosynthesis; Carbon fixation in photosynthetic organisms; Isoquinoline alkaloid biosynthesis; Tropane, piperidine and pyridine alkaloid biosynthesis; Metabolic pathways; Biosynthesis of secondary metabolites; Microbial metabolism in diverse environments; Biosynthesis of antibiotics	*4.94E-13	1.92E-04	73.4
2.1.1.133	precorrin-4 C11-methyltransferase	-	Porphyrin and chlorophyll metabolism; Metabolic pathways	*2.54E-05	2.02E-04	*22.1
1.1.1.290	4-phosphoerythronate dehydrogenase	-	Vitamin B6 metabolism; Metabolic pathways	*5.12E-10	2.09E-04	64.4
4.2.1.10	3-dehydroquinate dehydratase	-	Phenylalanine, tyrosine and tryptophan biosynthesis; Metabolic pathways; Biosynthesis of secondary metabolites; Biosynthesis of antibiotics	*7.98E-07	2.16E-04	43.8
1.16.3.1	ferroxidase	+	Porphyrin and chlorophyll metabolism	*3.36E-11	2.17E-04	68.9
2.7.6.3	2-amino-4-hydroxy-6-hydroxymethyldihydropteridine diphosphokinase	+	**Folate biosynthesis**; Metabolic pathways	*1.36E-08	2.18E-04	57.6
3.5.1.53	N-carbamoylputrescine amidase	-	**Arginine and proline metabolism**; Metabolic pathways	*2.36E-05	2.27E-04	*23.9
2.1.1.34	tRNA (guanosine18-2'-O)-methyltransferase	+		*6.11E-10	2.29E-04	64.5
2.7.2.3	phosphoglycerate kinase	+	Glycolysis / Gluconeogenesis; Carbon fixation in photosynthetic organisms; Metabolic pathways; Biosynthesis of secondary metabolites; Microbial metabolism in diverse environments; Biosynthesis of antibiotics	*2.45E-11	2.32E-04	69.6
1.1.1.93	tartrate dehydrogenase	+	Glyoxylate and dicarboxylate metabolism	*1.37E-11	2.42E-04	70.5
2.4.1.301	2'-deamino-2'-hydroxyneamine 1-alpha-D-kanosaminyltransferase	+	Neomycin, kanamycin and gentamicin biosynthesis; Biosynthesis of antibiotics	*2.69E-10	2.45E-04	66.3
2.8.1.10	thiazole synthase	-	Thiamine metabolism; Metabolic pathways	*2.08E-09	2.47E-04	62.6
3.4.25.2	HslU---HslV peptidase	+		*1.11E-09	2.50E-04	63.9
2.3.1.38	[acyl-carrier-protein] S-acetyltransferase	+		1.57E-04	2.82E-04	*7.7
2.3.1.129	acyl-[acyl-carrier-protein]---UDP-N-acetylglucosamine O-acyltransferase	-	Lipopolysaccharide biosynthesis; Metabolic pathways	*4.27E-07	2.83E-04	48.5
3.2.1.18	exo-alpha-sialidase	+	Other glycan degradation; Sphingolipid metabolism	2.50E-04	2.83E-04	*1.8
6.3.3.3	dethiobiotin synthase	-	**Biotin metabolism**; Metabolic pathways	*1.48E-08	3.01E-04	59.3
7.2.1.1	NADH:ubiquinone reductase (Na+-transporting)	-		*1.22E-05	3.07E-04	*31.9
1.3.1.74	2-alkenal reductase [NAD(P)+]	-		*3.04E-06	3.49E-04	41.3
3.1.2.26	bile-acid-CoA hydrolase	+		5.30E-05	3.49E-04	*21.7
2.3.1.157	glucosamine-1-phosphate N-acetyltransferase	+	Amino sugar and nucleotide sugar metabolism; Metabolic pathways; Biosynthesis of antibiotics	*1.30E-11	3.59E-04	72.2
2.1.2.3	phosphoribosylaminoimidazolecarboxamide formyltransferase	-	Purine metabolism; One carbon pool by folate; Metabolic pathways; Biosynthesis of secondary metabolites; Biosynthesis of antibiotics	4.30E-04	4.30E-04	*0.0
6.2.1.5	succinate---CoA ligase (ADP-forming)	+	Citrate cycle (TCA cycle); Propanoate metabolism; C5-Branched dibasic acid metabolism; Carbon fixation pathways in prokaryotes; Metabolic pathways; Biosynthesis of secondary metabolites; Microbial metabolism in diverse environments; Biosynthesis of antibiotics	*1.07E-05	4.87E-04	*37.3
1.8.2.3	sulfide-cytochrome-c reductase (flavocytochrome c)	+	Sulfur metabolism; Microbial metabolism in diverse environments	1.16E-04	5.20E-04	*19.0
2.7.1.4	fructokinase	-	Fructose and mannose metabolism; Starch and sucrose metabolism; Amino sugar and nucleotide sugar metabolism; Metabolic pathways	8.23E-05	5.33E-04	*22.6
1.2.1.27	methylmalonate-semialdehyde dehydrogenase (CoA-acylating)	+	Valine, leucine and isoleucine degradation; Propanoate metabolism; Metabolic pathways	1.96E-04	5.35E-04	*13.5
3.1.21.2	deoxyribonuclease IV	+		5.44E-04	5.44E-04	*0.0
6.4.1.2	acetyl-CoA carboxylase	-	Fatty acid biosynthesis; Aflatoxin biosynthesis; Pyruvate metabolism; Propanoate metabolism; Carbon fixation pathways in prokaryotes; Metabolic pathways; Biosynthesis of secondary metabolites; Microbial metabolism in diverse environments; Biosynthesis of antibiotics	*2.96E-06	5.58E-04	45.5
2.1.1.220	tRNA (adenine58-N1)-methyltransferase	+		*2.44E-10	6.03E-04	70.6
2.7.1.130	tetraacyldisaccharide 4'-kinase	-	Lipopolysaccharide biosynthesis; Metabolic pathways	*2.58E-05	6.06E-04	*33.6
6.4.1.7	2-oxoglutarate carboxylase	+		6.21E-04	6.21E-04	*0.0
1.3.1.20	trans-1,2-dihydrobenzene-1,2-diol dehydrogenase	+	Metabolism of xenobiotics by cytochrome P450	4.59E-05	6.50E-04	*30.0
2.8.3.12	glutaconate CoA-transferase	+	Styrene degradation; Butanoate metabolism; Microbial metabolism in diverse environments	6.60E-04	6.60E-04	*0.0
4.2.99.18	DNA-(apurinic or apyrimidinic site) lyase	+		*8.85E-10	6.91E-04	69.3
2.1.1.166	23S rRNA (uridine2552-2'-O)-methyltransferase	+		7.39E-04	7.39E-04	*0.0
2.3.1.51	1-acylglycerol-3-phosphate O-acyltransferase	-	Glycerolipid metabolism; Glycerophospholipid metabolism; Metabolic pathways; Biosynthesis of secondary metabolites	*2.27E-08	7.52E-04	63.7
2.7.7.13	mannose-1-phosphate guanylyltransferase	-	Fructose and mannose metabolism; Amino sugar and nucleotide sugar metabolism; Metabolic pathways; Biosynthesis of secondary metabolites	*1.93E-05	7.58E-04	*37.9
3.4.21.92	endopeptidase Clp	+		*1.69E-07	7.83E-04	58.8
3.2.1.32	endo-1,3-beta-xylanase	+		*2.82E-07	8.24E-04	57.6
3.1.3.46	fructose-2,6-bisphosphate 2-phosphatase	+	Fructose and mannose metabolism	*3.55E-06	8.62E-04	48.3
3.4.13.18	cytosol nonspecific dipeptidase	-	**Arginine and proline metabolism**; Histidine metabolism; beta-Alanine metabolism; Metabolic pathways	8.73E-04	8.73E-04	*0.0
2.3.1.274	phosphate acyltransferase	+		*1.98E-07	9.44E-04	59.6
6.3.1.5	NAD+ synthase	+	Nicotinate and nicotinamide metabolism; Metabolic pathways	*3.52E-08	9.54E-04	64.1
2.4.1.292	GalNAc-alpha-(1->4)-GalNAc-alpha-(1->3)-diNAcBac-PP-undecaprenol alpha-1,4-N-acetyl-D-galactosaminyltransferase	+		9.86E-04	9.86E-04	*0.0
6.1.1.21	histidine---tRNA ligase	+	Aminoacyl-tRNA biosynthesis	*3.23E-07	9.87E-04	58.4
3.1.3.5	5'-nucleotidase	-	Purine metabolism; Pyrimidine metabolism; Nicotinate and nicotinamide metabolism; Metabolic pathways; Biosynthesis of secondary metabolites	*3.84E-09	1.06E-03	69.1
2.5.1.72	quinolinate synthase	-		*7.14E-06	1.09E-03	47.0
1.1.1.26	glyoxylate reductase	-	Glyoxylate and dicarboxylate metabolism; Metabolic pathways; Biosynthesis of secondary metabolites; Microbial metabolism in diverse environments	*2.14E-06	1.14E-03	52.9
6.2.1.3	long-chain-fatty-acid---CoA ligase	-	Fatty acid biosynthesis; Fatty acid degradation; Metabolic pathways	1.19E-04	1.19E-03	*29.1
5.3.1.4	L-arabinose isomerase	-	Pentose and glucuronate interconversions; Metabolic pathways	*1.36E-09	1.22E-03	71.5
3.6.3.21	polar-amino-acid-transporting ATPase	-		1.26E-03	1.26E-03	*0.0
1.2.1.58	phenylglyoxylate dehydrogenase (acylating)	+	Phenylalanine metabolism	*9.76E-10	1.29E-03	72.3
6.3.4.21	nicotinate phosphoribosyltransferase	+	Nicotinate and nicotinamide metabolism; Metabolic pathways	3.02E-05	1.35E-03	40.9
2.4.2.18	anthranilate phosphoribosyltransferase	-	Phenylalanine, tyrosine and tryptophan biosynthesis; Metabolic pathways; Biosynthesis of secondary metabolites; Biosynthesis of antibiotics	*5.48E-10	1.36E-03	73.3
1.97.1.4	[formate-C-acetyltransferase]-activating enzyme	-		5.14E-05	1.36E-03	*37.5
1.12.1.3	hydrogen dehydrogenase (NADP+)	+		*5.41E-07	1.40E-03	59.3
5.3.1.12	glucuronate isomerase	-	Pentose and glucuronate interconversions; Metabolic pathways	*1.41E-12	1.51E-03	79.9
3.2.1.74	glucan 1,4-beta-glucosidase	-	Starch and sucrose metabolism; Metabolic pathways	*2.34E-05	1.51E-03	43.7
1.5.1.34	6,7-dihydropteridine reductase	-	**Folate biosynthesis**; Metabolic pathways	*9.42E-08	1.52E-03	64.7

1.3.8.1	short-chain acyl-CoA dehydrogenase	-	Fatty acid degradation; Valine, leucine and isoleucine degradation; Butanoate metabolism; Metabolic pathways; Biosynthesis of secondary metabolites; Microbial metabolism in diverse environments	1.59E-03	1.59E-03	*0.0
2.3.1.47	8-amino-7-oxononanoate synthase	-	**Biotin metabolism**; Metabolic pathways; Microbial metabolism in diverse environments	1.61E-03	1.61E-03	*0.0
1.5.1.7	saccharopine dehydrogenase (NAD+, L-lysine-forming)	-	Lysine biosynthesis; Lysine degradation; Metabolic pathways; Biosynthesis of secondary metabolites; Biosynthesis of antibiotics	*5.16E-07	1.66E-03	60.7
2.4.1.182	lipid-A-disaccharide synthase	-	Lipopolysaccharide biosynthesis; Metabolic pathways	*2.07E-07	1.73E-03	63.6
2.4.1.8	maltose phosphorylase	+	Starch and sucrose metabolism; Metabolic pathways	1.76E-03	1.76E-03	*0.0
5.4.99.18	5-(carboxyamino)imidazole ribonucleotide mutase	-	Purine metabolism; Metabolic pathways; Biosynthesis of secondary metabolites; Biosynthesis of antibiotics	*8.67E-07	1.83E-03	59.9
2.5.1.30	heptaprenyl diphosphate synthase	+	Terpenoid backbone biosynthesis; Biosynthesis of secondary metabolites	*6.74E-06	1.86E-03	52.2
2.7.1.162	N-acetylhexosamine 1-kinase	+		*1.10E-08	1.93E-03	70.6
4.2.1.70	pseudouridylate synthase	+	Pyrimidine metabolism	3.97E-05	1.95E-03	43.2
1.2.4.4	3-methyl-2-oxobutanoate dehydrogenase (2-methylpropanoyl-transferring)	+	Valine, leucine and isoleucine degradation; Propanoate metabolism; Metabolic pathways; Biosynthesis of secondary metabolites; Biosynthesis of antibiotics	*8.91E-06	2.16E-03	52.3
1.1.1.47	glucose 1-dehydrogenase [NAD(P)+]	+	Pentose phosphate pathway; Microbial metabolism in diverse environments	*5.71E-12	2.18E-03	80.2
5.1.99.1	methylmalonyl-CoA epimerase	+	Valine, leucine and isoleucine degradation; Glyoxylate and dicarboxylate metabolism; Propanoate metabolism; Carbon fixation pathways in prokaryotes; Metabolic pathways; Microbial metabolism in diverse environments	*5.74E-07	2.21E-03	62.5
2.1.1.184	23S rRNA (adenine2085-N6)-dimethyltransferase	+		*2.03E-07	2.25E-03	65.4
2.7.1.170	anhydro-N-acetylmuramic acid kinase	+		*2.45E-05	2.32E-03	47.9
3.1.1.11	pectinesterase	-	Pentose and glucuronate interconversions; Metabolic pathways	*5.56E-07	2.34E-03	63.1
2.1.1.51	rRNA (guanine-N1-)-methyltransferase	-		1.04E-03	2.37E-03	*14.0
2.4.2.22	xanthine phosphoribosyltransferase	-	Purine metabolism; Metabolic pathways; Biosynthesis of secondary metabolites	*1.45E-06	2.40E-03	60.3
2.4.2.52	triphosphoribosyl-dephospho-CoA synthase	+		5.19E-04	2.43E-03	*23.7
4.1.3.36	1,4-dihydroxy-2-naphthoyl-CoA synthase	-	Ubiquinone and other terpenoid-quinone biosynthesis; Metabolic pathways; Biosynthesis of secondary metabolites	*1.90E-09	2.44E-03	74.5
3.5.1.26	N4-(beta-N-acetylglucosaminyl)-L-asparaginase	+	Other glycan degradation	2.50E-03	2.50E-03	*0.0
1.4.1.13	glutamate synthase (NADPH)	-	Alanine, aspartate and glutamate metabolism; Nitrogen metabolism; Metabolic pathways; Biosynthesis of secondary metabolites; Microbial metabolism in diverse environments; Biosynthesis of antibiotics	*7.20E-12	2.53E-03	80.6
2.3.1.19	phosphate butyryltransferase	+	Butanoate metabolism; Metabolic pathways	2.41E-04	2.53E-03	*32.4
1.1.1.85	3-isopropylmalate dehydrogenase	-	Valine, leucine and isoleucine biosynthesis; C5-Branched dibasic acid metabolism; Metabolic pathways; Biosynthesis of secondary metabolites	*2.93E-05	2.54E-03	47.8
2.4.99.12	lipid IVA 3-deoxy-D-manno-octulosonic acid transferase	-	Lipopolysaccharide biosynthesis; Metabolic pathways	*3.22E-07	2.76E-03	65.7
2.1.1.227	16S rRNA (cytidine1409-2'-O)-methyltransferase	+		2.80E-03	2.80E-03	*0.0
3.2.1.83	kappa-carrageenase	+		*9.08E-06	2.81E-03	54.7
2.7.14.1	protein arginine kinase	+		*5.18E-07	2.85E-03	64.7
3.6.5.3	protein-synthesizing GTPase	+		*9.39E-11	3.06E-03	79.1
1.1.1.27	L-lactate dehydrogenase	+	Glycolysis / Gluconeogenesis; Cysteine and methionine metabolism; Pyruvate metabolism; Propanoate metabolism; Metabolic pathways; Biosynthesis of secondary metabolites; Microbial metabolism in diverse environments; Biosynthesis of antibiotics	3.07E-03	3.07E-03	*0.0
5.4.4.2	isochorismate synthase	-	Ubiquinone and other terpenoid-quinone biosynthesis; Biosynthesis of siderophore group nonribosomal peptides; Metabolic pathways; Biosynthesis of secondary metabolites; Biosynthesis of antibiotics	*1.04E-06	3.17E-03	63.5
2.7.1.92	5-dehydro-2-deoxygluconokinase	-	Inositol phosphate metabolism; Metabolic pathways; Microbial metabolism in diverse environments	*1.57E-09	3.19E-03	76.1
1.3.1.2	dihydropyrimidine dehydrogenase (NADP+)	+	Pyrimidine metabolism; beta-Alanine metabolism; Pantothenate and CoA biosynthesis; Drug metabolism - other enzymes; Metabolic pathways	4.20E-04	3.22E-03	*30.2
1.14.13.39	nitric-oxide synthase (NADPH)	+	Arginine biosynthesis; **Arginine and proline metabolism**; Cyanoamino acid metabolism; Metabolic pathways; Biosynthesis of secondary metabolites; Biosynthesis of antibiotics	7.19E-05	3.29E-03	45.2
3.6.1.3	adenosinetriphosphatase	+	Purine metabolism	*5.41E-12	3.31E-03	81.8
3.5.99.3	hydroxydechloroatrazine ethylaminohydrolase	+		*6.40E-06	3.32E-03	57.7
3.1.11.2	exodeoxyribonuclease III	-		*1.60E-12	3.37E-03	82.8
7.1.1.1	proton-translocating NAD(P)+ transhydrogenase	+		2.77E-03	3.40E-03	*4.2
3.2.2.23	DNA-formamidopyrimidine glycosylase	+		*2.19E-05	3.44E-03	52.5
6.2.1.16	acetoacetate---CoA ligase	-	Valine, leucine and isoleucine degradation; Butanoate metabolism	*3.34E-10	3.51E-03	78.4
3.1.26.4	ribonuclease H	+		*3.01E-08	3.58E-03	72.4
4.7.1.1	alpha-D-ribose 1-methylphosphonate 5-phosphate C-P-lyase	+	Phosphonate and phosphinate metabolism	*1.83E-06	3.59E-03	62.8
3.2.1.145	galactan 1,3-beta-galactosidase	+		3.72E-04	3.71E-03	*33.5
3.2.1.187	(Ara-f)3-Hyp beta-L-arabinobiosidase	+		3.72E-03	3.72E-03	*0.0
3.5.1.100	(R)-amidase	-		3.84E-03	3.84E-03	*0.0
2.7.4.1	ATP-polyphosphate phosphotransferase	-	Oxidative phosphorylation	*1.05E-12	3.94E-03	83.6
1.4.1.14	glutamate synthase (NADH)	-	Alanine, aspartate and glutamate metabolism; Nitrogen metabolism; Metabolic pathways; Biosynthesis of secondary metabolites; Microbial metabolism in diverse environments; Biosynthesis of antibiotics	*2.88E-06	3.94E-03	62.0
3.2.1.180	unsaturated chondroitin disaccharide hydrolase	+		*2.22E-07	3.99E-03	69.1
1.1.1.51	3(or 17)beta-hydroxysteroid dehydrogenase	+	Steroid hormone biosynthesis; Steroid degradation; Metabolic pathways; Microbial metabolism in diverse environments	4.69E-05	3.99E-03	50.0
1.2.1.22	lactaldehyde dehydrogenase	-	Pyruvate metabolism; Microbial metabolism in diverse environments	*1.38E-05	4.00E-03	56.2
1.1.1.69	gluconate 5-dehydrogenase	-		*2.94E-05	4.05E-03	52.7
3.5.1.44	protein-glutamine glutaminase	-		1.16E-04	4.06E-03	44.4
6.3.2.5	phosphopantothenate---cysteine ligase (CTP)	-	Pantothenate and CoA biosynthesis; Metabolic pathways	3.75E-03	4.06E-03	*1.7
2.3.1.189	mycothiol synthase	+		*1.63E-06	4.08E-03	64.1
3.1.1.83	monoterpene epsilon-lactone hydrolase	-	Limonene and pinene degradation; Caprolactam degradation	4.09E-03	4.09E-03	*0.0
5.1.3.20	ADP-glyceromanno-heptose 6-epimerase	+	Lipopolysaccharide biosynthesis; Metabolic pathways	1.42E-03	4.25E-03	*19.7
3.4.11.9	Xaa-Pro aminopeptidase	-		*5.09E-06	4.40E-03	61.0
2.1.1.64	3-demethylubiquinol 3-O-methyltransferase	-	Ubiquinone and other terpenoid-quinone biosynthesis; Metabolic pathways; Biosynthesis of secondary metabolites	*4.35E-06	4.42E-03	61.6
1.2.1.50	long-chain acyl-protein thioester reductase	+	Cutin, suberine and wax biosynthesis; Biosynthesis of secondary metabolites	1.61E-03	4.57E-03	*19.1
3.6.5.nl		+		*3.87E-07	4.64E-03	68.9
1.2.99.2	carbon-monoxide dehydrogenase (acceptor)	-		*1.39E-06	4.72E-03	65.7
3.4.21.116	SpoIVB peptidase	-		*4.71E-06	4.83E-03	62.1
2.7.7.47	streptomycin 3''-adenylyltransferase	+		*2.12E-06	4.84E-03	64.7
3.2.2.nl		-		*1.16E-10	4.90E-03	80.9
4.4.1.8	cystathionine beta-lyase	-	Cysteine and methionine metabolism; Selenocompound metabolism; Metabolic pathways; Biosynthesis of secondary metabolites	*1.17E-06	4.93E-03	66.5
3.5.1.88	peptide deformylase	+		*2.75E-08	5.01E-03	74.5
1.8.1.8	protein-disulfide reductase	+		6.29E-04	5.03E-03	*32.7
2.7.7.22	mannose-1-phosphate guanylyltransferase (GDP)	-	Fructose and mannose metabolism; Amino sugar and nucleotide sugar metabolism; Metabolic pathways	5.15E-03	5.15E-03	*0.0
4.1.2.13	fructose-bisphosphate aldolase	-	Glycolysis / Gluconeogenesis; Pentose phosphate pathway; Fructose and mannose metabolism; Methane metabolism; Carbon fixation in photosynthetic organisms; Metabolic pathways; Biosynthesis of secondary metabolites; Microbial metabolism in diverse environments; Biosynthesis of antibiotics	*4.60E-07	5.19E-03	69.3
4.1.1.48	indole-3-glycerol-phosphate synthase	-	Phenylalanine, tyrosine and tryptophan biosynthesis; Metabolic pathways; Biosynthesis of secondary metabolites; Biosynthesis of antibiotics	*1.73E-07	5.33E-03	71.6
2.5.1.9	riboflavin synthase	-	**Riboflavin metabolism**; Metabolic pathways; Biosynthesis of secondary metabolites	*2.84E-05	5.42E-03	55.9
3.2.2.9	adenosylhomocysteine nucleosidase	-	Cysteine and methionine metabolism; Metabolic pathways	5.87E-05	5.69E-03	52.6
5.3.1.1	triose-phosphate isomerase	+	Glycolysis / Gluconeogenesis; Fructose and mannose metabolism; Inositol phosphate metabolism; Carbon fixation in photosynthetic organisms; Metabolic pathways; Biosynthesis of secondary metabolites; Microbial metabolism in diverse environments; Biosynthesis of antibiotics	*1.07E-05	5.82E-03	60.8
2.2.1.3	formaldehyde transketolase	+	Methane metabolism; Microbial metabolism in diverse environments	7.39E-04	5.83E-03	*33.2
1.18.1.2	ferredoxin---NADP+ reductase	+	Photosynthesis; Metabolic pathways	*1.42E-06	5.94E-03	67.5
4.1.2.50	6-carboxytetrahydropterin synthase	-	**Folate biosynthesis**; Metabolic pathways	*1.09E-06	5.98E-03	68.2
1.3.99.1	succinate dehydrogenase	+		*1.21E-05	6.12E-03	60.8
2.5.1.1	dimethylallyltranstransferase	-	Terpenoid backbone biosynthesis; Metabolic pathways; Biosynthesis of secondary metabolites; Biosynthesis of antibiotics	*2.52E-07	6.28E-03	71.9
2.1.1.104	caffeoyl-CoA O-methyltransferase	+	Phenylalanine metabolism; Phenylpropanoid biosynthesis; Flavonoid biosynthesis; Stilbenoid, diarylheptanoid and gingerol biosynthesis; Metabolic pathways; Biosynthesis of secondary metabolites	9.56E-04	6.34E-03	*31.7
2.3.1.86	fatty-acyl-CoA synthase system	-	Fatty acid biosynthesis; Metabolic pathways	*2.99E-09	6.37E-03	78.8
2.7.1.90	diphosphate---fructose-6-phosphate 1-phosphotransferase	-	Glycolysis / Gluconeogenesis; Pentose phosphate pathway; Fructose and mannose metabolism; Metabolic pathways; Biosynthesis of secondary metabolites; Microbial metabolism in diverse environments; Biosynthesis of antibiotics	*9.16E-07	6.45E-03	69.2
5.1.3.3	aldose 1-epimerase	-	Glycolysis / Gluconeogenesis; Galactose metabolism; Metabolic pathways; Biosynthesis of secondary metabolites; Microbial metabolism in diverse environments; Biosynthesis of antibiotics	*1.60E-05	6.49E-03	60.2
1.1.1.179	D-xylose 1-dehydrogenase (NADP+, D-xylono-1,5-lactone-forming)	+	Pentose and glucuronate interconversions	6.53E-03	6.53E-03	*0.0
6.3.1.19	prokaryotic ubiquitin-like protein ligase	+		1.68E-03	6.64E-03	*25.4
4.1.2.25	dihydroneopterin aldolase	-	**Folate biosynthesis**; Metabolic pathways	*5.58E-08	6.73E-03	75.1
3.2.1.136	glucuronoarabinoxylan endo-1,4-beta-xylanase	+		1.20E-03	6.73E-03	*30.0
3.2.1.68	isoamylase	+	Starch and sucrose metabolism; Metabolic pathways; Biosynthesis of secondary metabolites	9.84E-05	6.96E-03	52.0
3.4.22.71	sortase B	-		8.08E-04	7.06E-03	*35.3
3.4.14.5	dipeptidyl-peptidase IV	+		7.09E-03	7.09E-03	*0.0
4.4.1.1	cystathionine gamma-lyase	-	Glycine, serine and threonine metabolism; Cysteine and methionine metabolism; Selenocompound metabolism; Metabolic pathways; Biosynthesis of antibiotics	2.46E-03	7.17E-03	*21.1
2.7.1.21	thymidine kinase	-	Pyrimidine metabolism; Drug metabolism - other enzymes; Metabolic pathways	7.26E-03	7.26E-03	*0.0
1.2.1.12	glyceraldehyde-3-phosphate dehydrogenase (phosphorylating)	+	Glycolysis / Gluconeogenesis; Carbon fixation in photosynthetic organisms; Metabolic pathways; Biosynthesis of secondary metabolites; Microbial metabolism in diverse environments; Biosynthesis of antibiotics	*6.02E-06	7.32E-03	64.9
1.1.1.244	methanol dehydrogenase	+	Methane metabolism; Metabolic pathways; Microbial metabolism in diverse environments	2.12E-03	7.36E-03	*23.9
5.1.99.6	NAD(P)H-hydrate epimerase	-		*6.49E-06	7.57E-03	64.9
6.2.1.27	4-hydroxybenzoate---CoA ligase	-	Benzoate degradation; Aminobenzoate degradation; Metabolic pathways; Microbial metabolism in diverse environments	6.16E-03	7.69E-03	*5.3
2.8.3.10	citrate CoA-transferase	+		7.92E-03	7.92E-03	*0.0
4.1.1.44	4-carboxymuconolactone decarboxylase	+	Benzoate degradation; Metabolic pathways; Microbial metabolism in diverse environments	2.16E-04	8.00E-03	48.6
2.6.1.51	serine---pyruvate transaminase	+	Glycine, serine and threonine metabolism; Metabolic pathways	8.17E-03	8.17E-03	*0.0
2.5.1.90	all-trans-octaprenyl-diphosphate synthase	-	Terpenoid backbone biosynthesis; Biosynthesis of secondary metabolites	8.22E-03	8.22E-03	*0.0
2.1.1.226	23S rRNA (cytidine1920-2'-O)-methyltransferase	-		2.12E-03	8.35E-03	*26.3
3.4.14.12	Xaa-Xaa-Pro tripeptidyl-peptidase	-		8.40E-03	8.40E-03	*0.0
1.4.1.3	glutamate dehydrogenase [NAD(P)+]	-	Arginine biosynthesis; Alanine, aspartate and glutamate metabolism; D-Glutamine and D-glutamate metabolism; Nitrogen metabolism; Metabolic pathways	*3.17E-07	8.41E-03	73.4
2.6.1.13	ornithine aminotransferase	-	**Arginine and proline metabolism**; Metabolic pathways; Biosynthesis of secondary metabolites; Biosynthesis of antibiotics	*9.36E-07	8.66E-03	71.4
4.1.3.27	anthranilate synthase	-	Phenylalanine, tyrosine and tryptophan biosynthesis; Phenazine biosynthesis; Metabolic pathways; Biosynthesis of secondary metabolites; Biosynthesis of antibiotics	5.01E-05	8.73E-03	58.2
1.1.1.133	dTDP-4-dehydrorhamnose reductase	-	Streptomycin biosynthesis; Polyketide sugar unit biosynthesis; Biosynthesis of antibiotics	8.95E-03	8.95E-03	*0.0
1.5.1.3	dihydrofolate reductase	-	One carbon pool by folate; **Folate biosynthesis**; Metabolic pathways	4.51E-04	9.01E-03	44.6
3.1.22.4	crossover junction endodeoxyribonuclease	+		*4.31E-08	9.22E-03	77.4

2.6.1.37	2-aminoethylphosphonate---pyruvate transaminase	-	Phosphonate and phosphinate metabolism; Metabolic pathways; Microbial metabolism in diverse environments	5.00E-05	9.33E-03	58.9
2.8.1.4	tRNA uracil 4-sulfurtransferase	+	Thiamine metabolism; Metabolic pathways	*8.54E-08	9.36E-03	76.5
6.3.5.3	phosphoribosylformylglycinamidine synthase	-	Purine metabolism; Metabolic pathways; Biosynthesis of secondary metabolites; Biosynthesis of antibiotics	*1.73E-07	9.51E-03	75.4
2.1.2.1	glycine hydroxymethyltransferase	+	Glycine, serine and threonine metabolism; Cyanoamino acid metabolism; Glyoxylate and dicarboxylate metabolism; One carbon pool by folate; Methane metabolism; Metabolic pathways; Biosynthesis of secondary metabolites; Microbial metabolism in diverse environments; Biosynthesis of antibiotics	*4.08E-06	9.65E-03	68.4
2.4.1.226	N-acetylgalactosaminyl-proteoglycan 3-beta-glucuronosyltransferase	+	Glycosaminoglycan biosynthesis - chondroitin sulfate / dermatan sulfate; Metabolic pathways	3.43E-03	0.010	*23.3
3.5.1.28	N-acetylmuramoyl-L-alanine amidase	-		*2.25E-11	0.011	85.4
1.4.1.2	glutamate dehydrogenase	-	Arginine biosynthesis; Alanine, aspartate and glutamate metabolism; Taurine and hypotaurine metabolism; Nitrogen metabolism; Metabolic pathways	*2.04E-06	0.011	71.3
2.1.1.208	23S rRNA (uridine2479-2'-O)-methyltransferase	+		*8.33E-06	0.011	67.7
4.3.1.7	ethanolamine ammonia-lyase	+	Glycerophospholipid metabolism; Metabolic pathways	*1.04E-05	0.011	66.7
3.4.13.3	Xaa-His dipeptidase	+		*1.30E-05	0.011	65.8
2.3.3.13	2-isopropylmalate synthase	-	Valine, leucine and isoleucine biosynthesis; Pyruvate metabolism; Metabolic pathways; Biosynthesis of secondary metabolites	2.34E-04	0.011	52.5
1.5.1.28	opine dehydrogenase	+		0.011	0.011	*0.0
2.7.1.15	ribokinase	+	Pentose phosphate pathway	0.011	0.011	*0.0
2.1.2.5	glutamate formimidoyltransferase	-	Histidine metabolism; One carbon pool by folate; Metabolic pathways	0.011	0.011	*0.0
3.1.6.6	choline-sulfatase	-		0.011	0.011	*0.0
2.7.7.23	UDP-N-acetylglucosamine diphosphorylase	+	Amino sugar and nucleotide sugar metabolism; Metabolic pathways; Biosynthesis of antibiotics	*1.72E-08	0.012	80.0
3.4.13.21	dipeptidase E	+		*1.01E-05	0.012	67.7
2.4.1.303	UDP-Gal:alpha-D-GlcNAc-diphosphoundecaprenol beta-1,3-galactosyltransferase	+		*1.50E-05	0.012	66.3
4.3.1.1	aspartate ammonia-lyase	-	Alanine, aspartate and glutamate metabolism; Metabolic pathways	*2.46E-05	0.012	64.4
3.11.1.1	phosphonoacetaldehyde hydrolase	-	Phosphonate and phosphinate metabolism; Metabolic pathways; Microbial metabolism in diverse environments	3.05E-05	0.012	63.9
2.7.1.63	polyphosphate---glucose phosphotransferase	+	Glycolysis / Gluconeogenesis; Amino sugar and nucleotide sugar metabolism; Metabolic pathways; Biosynthesis of secondary metabolites; Microbial metabolism in diverse environments; Biosynthesis of antibiotics	7.05E-04	0.012	45.5
2.4.1.175	glucuronosyl-N-acetylgalactosaminyl-proteoglycan 4-beta-N-acetylgalactosaminyltransferase	+	Glycosaminoglycan biosynthesis - chondroitin sulfate / dermatan sulfate; Metabolic pathways	4.25E-03	0.012	*23.6
3.4.24.84	Ste24 endopeptidase	+	Terpenoid backbone biosynthesis; Biosynthesis of antibiotics	9.40E-03	0.012	*7.2
1.6.5.3	NADH:ubiquinone reductase (H+-translocating)	+	Oxidative phosphorylation; Metabolic pathways	0.012	0.012	*0.0
1.1.1.103	L-threonine 3-dehydrogenase	+	Glycine, serine and threonine metabolism	0.012	0.012	*0.0
2.4.2.9	uracil phosphoribosyltransferase	+	Pyrimidine metabolism; Metabolic pathways	*2.27E-10	0.013	84.8
2.8.3.11	citramalate CoA-transferase	+	C5-Branched dibasic acid metabolism; Metabolic pathways	*8.28E-09	0.013	81.5
6.3.4.18	5-(carboxyamino)imidazole ribonucleotide synthase	+	Purine metabolism; Metabolic pathways; Biosynthesis of secondary metabolites; Biosynthesis of antibiotics	*3.32E-08	0.013	79.6
2.3.1.222	phosphate propanoyltransferase	+	Propanoate metabolism	*4.60E-07	0.013	75.5
2.6.1.76	diaminobutyrate---2-oxoglutarate transaminase	+	Glycine, serine and threonine metabolism; Metabolic pathways; Microbial metabolism in diverse environments	*2.11E-05	0.013	66.1
2.5.1.97	pseudaminic acid synthase	+	Amino sugar and nucleotide sugar metabolism	4.61E-04	0.013	49.6
3.2.1.172	unsaturated rhamnogalacturonyl hydrolase	-		0.013	0.013	*0.0
6.1.1.20	phenylalanine---tRNA ligase	+	Aminoacyl-tRNA biosynthesis	*3.95E-08	0.014	79.8
5.2.1.8	peptidylprolyl isomerase	-		2.51E-04	0.014	55.0
3.4.16.4	serine-type D-Ala-D-Ala carboxypeptidase	-	Peptidoglycan biosynthesis	*6.91E-10	0.015	84.5
3.2.1.40	alpha-L-rhamnosidase	-		*4.65E-06	0.015	71.6
3.4.19.3	pyroglutamyl-peptidase I	+		1.16E-04	0.015	60.4
3.2.1.96	mannosyl-glycoprotein endo-beta-N-acetylglucosaminidase	+	Other glycan degradation	3.16E-03	0.015	*32.1
2.7.8.36	undecaprenyl phosphate N,N'-diacetylbacillosamine 1-phosphate transferase	+		3.91E-03	0.015	*28.6
6.1.1.15	proline---tRNA ligase	+	Aminoacyl-tRNA biosynthesis	*2.86E-07	0.016	78.1
3.4.11.1	leucyl aminopeptidase	+	Glutathione metabolism; Metabolic pathways	*3.96E-07	0.016	77.4
3.5.99.2	aminopyrimidine aminohydrolase	+	Thiamine metabolism; Metabolic pathways	*3.33E-06	0.016	72.9
2.7.8.26	adenosylcobinamide-GDP ribazoletransferase	-	Porphyrin and chlorophyll metabolism; Metabolic pathways	*1.16E-05	0.016	69.9
2.7.4.6	nucleoside-diphosphate kinase	-	Purine metabolism; Pyrimidine metabolism; Drug metabolism - other enzymes; Metabolic pathways; Biosynthesis of secondary metabolites; Biosynthesis of antibiotics	2.02E-04	0.016	58.2
2.4.1.187	N-acetylglucosaminyldiphosphoundecaprenol N-acetyl-beta-D-mannosaminyltransferase	-		5.36E-03	0.016	*24.5
2.4.2.29	tRNA-guanosine34 preQ1 transglycosylase	+		*3.70E-07	0.017	78.1
1.4.3.1	D-aspartate oxidase	+	Alanine, aspartate and glutamate metabolism	*2.57E-06	0.017	74.0
2.7.7.9	UTP---glucose-1-phosphate uridylyltransferase	+	Pentose and glucuronate interconversions; Galactose metabolism; Starch and sucrose metabolism; Amino sugar and nucleotide sugar metabolism; Metabolic pathways; Biosynthesis of antibiotics	*1.62E-05	0.017	69.3
2.3.1.39	[acyl-carrier-protein] S-malonyltransferase	-	Fatty acid biosynthesis; Metabolic pathways	4.34E-05	0.017	66.0
3.2.1.82	exo-poly-alpha-digalacturonosidase	-		3.16E-04	0.017	56.0
2.4.99.16	starch synthase (maltosyl-transferring)	+	Starch and sucrose metabolism; Metabolic pathways	5.65E-04	0.017	52.0
2.7.1.172	protein-ribulosamine 3-kinase	+		1.02E-03	0.017	47.1
1.1.1.64	testosterone 17beta-dehydrogenase (NADP+)	+	Steroid hormone biosynthesis; Metabolic pathways	1.20E-03	0.017	45.7
1.1.1.271	GDP-L-fucose synthase	-	Fructose and mannose metabolism; Amino sugar and nucleotide sugar metabolism; Metabolic pathways	5.86E-03	0.017	*24.7
1.16.1.1	mercury(II) reductase	+		7.80E-03	0.017	*19.0
3.4.22.8	clostripain	+		0.017	0.017	*0.0
2.1.1.228	tRNA (guanine37-N1)-methyltransferase	+		*7.87E-08	0.018	80.5
5.1.3.32	L-rhamnose mutarotase	-		*8.07E-08	0.018	80.6
1.21.4.3	sarcosine reductase	+		*1.39E-07	0.018	79.7
1.1.1.23	histidinol dehydrogenase	-	Histidine metabolism; Metabolic pathways; Biosynthesis of secondary metabolites	3.80E-04	0.018	55.4
2.3.1.79	maltose O-acetyltransferase	+		0.018	0.018	*0.0
5.5.1.4	inositol-3-phosphate synthase	-	Streptomycin biosynthesis; Inositol phosphate metabolism; Metabolic pathways; Biosynthesis of antibiotics	*1.13E-08	0.019	83.3
5.3.1.28	D-sedoheptulose-7-phosphate isomerase	+	Lipopolysaccharide biosynthesis; Metabolic pathways	*9.63E-06	0.019	71.8
2.1.1.185	23S rRNA (guanosine2251-2'-O)-methyltransferase	-		1.06E-04	0.019	63.2
4.1.99.14	spore photoproduct lyase	-		1.13E-04	0.019	63.1
5.1.1.11	phenylalanine racemase (ATP-hydrolysing)	+	Phenylalanine metabolism	4.86E-04	0.019	54.4
5.4.1.2	precorrin-8X methylmutase	-		5.02E-03	0.019	*29.7
2.7.7.2	FAD synthase	-	**Riboflavin metabolism**; Metabolic pathways; Biosynthesis of secondary metabolites	8.58E-03	0.019	*20.2
3.2.2.4	AMP nucleosidase	-	Purine metabolism	*5.04E-07	0.020	78.5
6.1.1.14	glycine---tRNA ligase	+	Aminoacyl-tRNA biosynthesis	*2.35E-06	0.020	75.7
2.7.4.22	UMP kinase	+	Pyrimidine metabolism; Metabolic pathways	*7.83E-06	0.020	72.8
2.6.1.85	aminodeoxychorismate synthase	-	**Folate biosynthesis**	5.80E-04	0.020	54.2
6.1.1.17	glutamate---tRNA ligase	+	Porphyrin and chlorophyll metabolism; Aminoacyl-tRNA biosynthesis; Metabolic pathways; Biosynthesis of secondary metabolites; Microbial metabolism in diverse environments	*3.66E-07	0.021	79.4
6.3.3.1	phosphoribosylformylglycinamidine cyclo-ligase	+	Purine metabolism; Metabolic pathways; Biosynthesis of secondary metabolites; Biosynthesis of antibiotics	*3.93E-07	0.022	79.7
2.1.1.177	23S rRNA (pseudouridine1915-N3)-methyltransferase	+		*6.42E-06	0.022	74.2
1.1.1.405	ribitol-5-phosphate 2-dehydrogenase (NADP+)	+	Pentose and glucuronate interconversions; Metabolic pathways	3.55E-05	0.022	69.5
2.3.1.50	serine C-palmitoyltransferase	+	Sphingolipid metabolism; Metabolic pathways	0.022	0.022	*0.0
5.4.3.2	lysine 2,3-aminomutase	-	Lysine degradation	0.022	0.022	*0.0
2.7.7.6	DNA-directed RNA polymerase	+	Purine metabolism; Pyrimidine metabolism; Metabolic pathways	*1.19E-06	0.023	78.2
6.3.5.7	glutaminyl-tRNA synthase (glutamine-hydrolysing)	+	Aminoacyl-tRNA biosynthesis; Metabolic pathways	*1.63E-06	0.023	77.7
1.4.3.16	L-aspartate oxidase	-	Alanine, aspartate and glutamate metabolism; Nicotinate and nicotinamide metabolism; Metabolic pathways	4.11E-04	0.023	58.8
2.7.2.8	acetylglutamate kinase	-	Arginine biosynthesis; Metabolic pathways; Biosynthesis of secondary metabolites; Biosynthesis of antibiotics	*1.36E-06	0.024	78.1
2.7.11.17	Ca2+/calmodulin-dependent protein kinase	+		1.23E-04	0.025	65.9
2.5.1.3	thiamine phosphate synthase	-	Thiamine metabolism; Metabolic pathways	1.45E-03	0.025	50.7
1.1.1.283	methylglyoxal reductase (NADPH)	+	Pyruvate metabolism; Propanoate metabolism	0.013	0.025	*18.1
3.5.3.8	formimidoylglutamase	+	Histidine metabolism; Metabolic pathways	*7.21E-06	0.026	75.3
3.2.1.15	endo-polygalacturonase	-	Pentose and glucuronate interconversions; Metabolic pathways	1.78E-04	0.026	64.8
2.3.1.117	2,3,4,5-tetrahydropyridine-2,6-dicarboxylate N-succinyltransferase	+	Lysine biosynthesis; Metabolic pathways; Microbial metabolism in diverse environments	2.92E-04	0.026	62.1
2.7.1.26	riboflavin kinase	-	**Riboflavin metabolism**; Metabolic pathways; Biosynthesis of secondary metabolites	3.97E-04	0.026	60.6
2.4.2.21	nicotinate-nucleotide---dimethylbenzimidazole phosphoribosyltransferase	-	Porphyrin and chlorophyll metabolism; Metabolic pathways	*1.17E-06	0.027	79.2
3.8.1.7	4-chlorobenzoyl-CoA dehalogenase	+	Fluorobenzoate degradation; Metabolic pathways; Microbial metabolism in diverse environments	3.94E-04	0.027	61.3
2.1.1.164	demethylrebeccamycin-D-glucose O-methyltransferase	+	Staurosporine biosynthesis; Biosynthesis of antibiotics	4.06E-03	0.027	40.5
3.4.21.108	HtrA2 peptidase	+		0.027	0.027	*0.0
3.7.1.8	2,6-dioxo-6-phenylhexa-3-enoate hydrolase	+	Dioxin degradation; Metabolic pathways; Microbial metabolism in diverse environments	*1.88E-06	0.028	78.7
6.3.2.17	tetrahydrofolate synthase	-	**Folate biosynthesis**; Metabolic pathways	7.20E-05	0.029	69.7
1.11.1.6	catalase	+	Tryptophan metabolism; Glyoxylate and dicarboxylate metabolism; Biosynthesis of secondary metabolites; Biosynthesis of antibiotics	6.41E-03	0.029	*36.0
3.5.3.18	dimethylargininase	-		3.79E-05	0.030	72.3
3.5.4.12	dCMP deaminase	-	Pyrimidine metabolism; Metabolic pathways	8.30E-05	0.030	69.6
1.17.1.5	nicotinate dehydrogenase	-	Nicotinate and nicotinamide metabolism; Microbial metabolism in diverse environments	*9.94E-06	0.031	76.1
5.5.1.1	muconate cycloisomerase	-	Chlorocyclohexane and chlorobenzene degradation; Benzoate degradation; Fluorobenzoate degradation; Toluene degradation; Metabolic pathways; Microbial metabolism in diverse environments	1.09E-03	0.031	56.4
2.5.1.120	aminodeoxyfutalosine synthase	+	Ubiquinone and other terpenoid-quinone biosynthesis; Biosynthesis of secondary metabolites	2.05E-03	0.031	51.2
2.1.1.52	rRNA (guanine-N2-)-methyltransferase	+		3.79E-03	0.031	44.8
1.3.1.26	dihydrodipicolinate reductase	-		*3.44E-06	0.032	78.6
2.3.1.85	fatty-acid synthase system	+	Fatty acid biosynthesis; Metabolic pathways	0.012	0.032	*27.3
6.5.1.2	DNA ligase (NAD+)	+		*3.61E-08	0.033	85.0
6.3.4.13	phosphoribosylamine---glycine ligase	-	Purine metabolism; Metabolic pathways; Biosynthesis of secondary metabolites; Biosynthesis of antibiotics	3.97E-04	0.033	63.8
4.1.1.15	glutamate decarboxylase	+	Alanine, aspartate and glutamate metabolism; beta-Alanine metabolism; Taurine and hypotaurine metabolism; Butanoate metabolism; Metabolic pathways; Biosynthesis of secondary metabolites; Microbial metabolism in diverse environments	0.033	0.033	*0.0
4.1.1.2	oxalate decarboxylase	-	Glyoxylate and dicarboxylate metabolism; Metabolic pathways	*2.24E-05	0.034	75.0
1.3.1.9	enoyl-[acyl-carrier-protein] reductase (NADH)	-	Fatty acid biosynthesis; Metabolic pathways	*5.58E-06	0.035	78.5
3.8.1.2	(S)-2-haloacid dehalogenase	-	Chlorocyclohexane and chlorobenzene degradation; Chloroalkane and chloroalkene degradation; Metabolic pathways; Microbial metabolism in diverse environments	1.19E-03	0.035	57.8
1.1.1.131	mannuronate reductase	-		6.31E-05	0.036	72.4
4.2.3.1	threonine synthase	-	Glycine, serine and threonine metabolism; Vitamin B6 metabolism; Metabolic pathways; Biosynthesis of secondary metabolites; Microbial metabolism in diverse environments	5.13E-03	0.036	44.1
4.1.99.18	cyclic pyranopterin phosphate synthase	+		0.036	0.036	*0.0
3.1.1.72	acetylxylan esterase	+		0.036	0.036	*0.0
4.2.1.20	tryptophan synthase	-	Glycine, serine and threonine metabolism; Phenylalanine, tyrosine and tryptophan biosynthesis; Metabolic pathways; Biosynthesis of secondary metabolites; Biosynthesis of antibiotics	*8.54E-06	0.037	78.0
4.1.2.22	fructose-6-phosphate phosphoketolase	+	Carbon fixation in photosynthetic organisms; Microbial metabolism in diverse environments	1.43E-04	0.037	69.8

1.17.5.2	caffeine dehydrogenase	+	Caffeine metabolism; Microbial metabolism in diverse environments	2.47E-03	0.037	52.3
4.1.3.39	4-hydroxy-2-oxovalerate aldolase	-	Phenylalanine metabolism; Benzoate degradation; Dioxin degradation; Xylene degradation; Metabolic pathways; Microbial metabolism in diverse environments	3.51E-03	0.037	49.0
2.1.1.207	tRNA (cytidine34-2'-O)-methyltransferase	+		*1.46E-07	0.038	84.5
6.3.1.2	glutamine synthetase	-	Arginine biosynthesis; Alanine, aspartate and glutamate metabolism; Glyoxylate and dicarboxylate metabolism; Nitrogen metabolism; Metabolic pathways; Microbial metabolism in diverse environments	0.021	0.038	*19.1
4.3.99.3	7-carboxy-7-deazaguanine synthase	-	**Folate biosynthesis**; Metabolic pathways	0.030	0.038	*8.8
3.4.24.64	mitochondrial processing peptidase	+		1.55E-03	0.039	57.4
2.1.1.72	site-specific DNA-methyltransferase (adenine-specific)	+		3.92E-03	0.039	48.6
1.1.1.30	3-hydroxybutyrate dehydrogenase	+	Synthesis and degradation of ketone bodies; Butanoate metabolism; Metabolic pathways	6.72E-03	0.039	41.9
2.7.9.1	pyruvate, phosphate dikinase	+	Pyruvate metabolism; Carbon fixation in photosynthetic organisms; Carbon fixation pathways in prokaryotes; Metabolic pathways; Microbial metabolism in diverse environments	9.53E-05	0.040	72.3
2.1.1.271	cobalt-precorrin-4 methyltransferase	+	Porphyrin and chlorophyll metabolism; Metabolic pathways	0.011	0.040	*34.4
2.6.1.77	taurine---pyruvate aminotransferase	+	Taurine and hypotaurine metabolism; Metabolic pathways	1.30E-04	0.041	71.4
3.4.24.55	pitrilysin	+		0.041	0.041	*0.0
1.4.3.5	pyridoxal 5'-phosphate synthase	+	Vitamin B6 metabolism; Metabolic pathways; Microbial metabolism in diverse environments	0.041	0.041	*0.0
6.1.1.12	aspartate---tRNA ligase	+	Aminoacyl-tRNA biosynthesis	*1.11E-05	0.042	78.6
2.7.1.36	mevalonate kinase	+	Terpenoid backbone biosynthesis; Metabolic pathways; Biosynthesis of secondary metabolites; Biosynthesis of antibiotics	7.52E-05	0.042	73.6
6.3.4.6	urea carboxylase	+	Arginine biosynthesis; Atrazine degradation; Metabolic pathways	0.043	0.043	*0.0
6.3.4.5	argininosuccinate synthase	+	Arginine biosynthesis; Alanine, aspartate and glutamate metabolism; Metabolic pathways; Biosynthesis of secondary metabolites; Biosynthesis of antibiotics	*3.16E-06	0.044	81.2
1.1.1.28	D-lactate dehydrogenase	-	Pyruvate metabolism; Microbial metabolism in diverse environments	1.72E-03	0.044	58.7
5.1.3.23	UDP-2,3-diacetamido-2,3-dideoxyglucuronic acid 2-epimerase	+	Amino sugar and nucleotide sugar metabolism	8.25E-03	0.045	42.2
1.1.1.289	sorbose reductase	+	Fructose and mannose metabolism	0.045	0.045	*0.0
2.4.2.31	NAD+---protein-arginine ADP-ribosyltransferase	+		0.047	0.047	*0.0
6.3.3.2	5-formyltetrahydrofolate cyclo-ligase	-	One carbon pool by folate; Metabolic pathways	6.46E-04	0.048	66.4
2.5.1.52	L-mimosine synthase	+		2.18E-03	0.048	58.6
4.3.1.2	methylaspartate ammonia-lyase	+	Glyoxylate and dicarboxylate metabolism; C5-Branched dibasic acid metabolism; Metabolic pathways	0.013	0.048	*37.2
5.1.3.24	N-acetylneuraminate epimerase	-		1.11E-04	0.049	74.2
2.4.1.54	undecaprenyl-phosphate mannosyltransferase	-		3.36E-03	0.049	54.8
2.6.1.19	4-aminobutyrate---2-oxoglutarate transaminase	+	Alanine, aspartate and glutamate metabolism; beta-Alanine metabolism; Propanoate metabolism; Butanoate metabolism; Metabolic pathways; Microbial metabolism in diverse environments	3.37E-03	0.049	54.9
5.4.2.6	beta-phosphoglucomutase	-	Starch and sucrose metabolism	0.011	0.049	40.5
2.7.8.7	holo-[acyl-carrier-protein] synthase	+	Pantothenate and CoA biosynthesis	1.45E-04	0.050	73.3
2.3.1.54	formate C-acetyltransferase	-	Pyruvate metabolism; Propanoate metabolism; Butanoate metabolism; Metabolic pathways; Microbial metabolism in diverse environments	*4.95E-06	0.051	81.7
2.7.1.167	D-glycero-beta-D-manno-heptose-7-phosphate kinase	+	Lipopolysaccharide biosynthesis; Metabolic pathways	1.16E-04	0.051	74.5
5.4.2.1	phosphoglycerate mutase	-		0.016	0.051	*34.2
1.1.1.317	perakine reductase	+		7.99E-04	0.052	66.6
2.4.1.129	peptidoglycan glycosyltransferase	-	Peptidoglycan biosynthesis	4.75E-04	0.053	69.4
6.1.1.9	valine---tRNA ligase	+	Aminoacyl-tRNA biosynthesis	1.25E-03	0.053	64.0
2.3.1.89	tetrahydrodipicolinate N-acetyltransferase	+	Lysine biosynthesis; Metabolic pathways; Biosynthesis of secondary metabolites	0.011	0.053	42.0
4.2.1.45	CDP-glucose 4,6-dehydratase	-	Amino sugar and nucleotide sugar metabolism	0.045	0.053	*7.0
6.3.4.14	biotin carboxylase	-		8.46E-04	0.054	66.7
3.6.3.43	peptide-transporting ATPase	+		4.58E-03	0.054	53.7
2.3.1.234	N6-L-threonylcarbamoyladenine synthase	+		*2.05E-06	0.055	83.7
1.17.4.2	ribonucleoside-triphosphate reductase (thioredoxin)	-	Purine metabolism; Pyrimidine metabolism; Metabolic pathways	0.013	0.055	*39.8
2.7.1.48	uridine/cytidine kinase	-	Pyrimidine metabolism; Drug metabolism - other enzymes; Metabolic pathways	1.28E-04	0.056	75.1
2.5.1.55	3-deoxy-8-phosphooctulonate synthase	-	Lipopolysaccharide biosynthesis; Metabolic pathways	0.022	0.056	*30.2
3.4.21.105	rhomboid protease	-		0.056	0.056	*0.0
4.1.2.14	2-dehydro-3-deoxy-phosphogluconate aldolase	-	Pentose phosphate pathway; Metabolic pathways; Microbial metabolism in diverse environments	*4.84E-06	0.057	82.6
4.1.1.5	acetolactate decarboxylase	+	Butanoate metabolism; C5-Branched dibasic acid metabolism	*1.60E-05	0.057	80.6
2.7.1.16	ribulokinase	+	Pentose and glucuronate interconversions; Metabolic pathways	5.01E-04	0.057	70.1
1.2.7.8	indolepyruvate ferredoxin oxidoreductase	+		0.012	0.057	42.2
4.2.1.136	ADP-dependent NAD(P)H-hydrate dehydratase	-		0.013	0.057	41.6
1.3.99.22	coproporphyrinogen dehydrogenase	-		*7.69E-07	0.059	85.4
1.3.1.34	2,4-dienoyl-CoA reductase [(2E)-enoyl-CoA-producing]	-		2.26E-03	0.059	61.8
1.1.1.261	sn-glycerol-1-phosphate dehydrogenase	-	Glycerophospholipid metabolism	0.017	0.059	*38.0
2.3.1.n3		+		*7.00E-09	0.060	89.5
5.4.99.23	23S rRNA pseudouridine1911/1915/1917 synthase	-		2.41E-04	0.060	73.8
2.5.1.49	O-acetylhomoserine aminocarboxypropyltransferase	+	Cysteine and methionine metabolism; Metabolic pathways	1.41E-03	0.060	65.4
2.7.7.81	pseudaminic acid cytidylyltransferase	+	Amino sugar and nucleotide sugar metabolism	0.012	0.060	44.4
2.7.1.55	allose kinase	+	Fructose and mannose metabolism; Microbial metabolism in diverse environments	0.014	0.060	41.7
2.7.7.70	D-glycero-beta-D-manno-heptose 1-phosphate adenylyltransferase	+	Lipopolysaccharide biosynthesis; Metabolic pathways	2.89E-03	0.061	60.3
3.4.22.10	streptopain	+		3.01E-03	0.061	60.0
4.1.2.29	5-dehydro-2-deoxyphosphogluconate aldolase	+	Inositol phosphate metabolism; Metabolic pathways; Microbial metabolism in diverse environments	5.06E-03	0.062	55.8
2.5.1.75	tRNA dimethylallyltransferase	-	Zeatin biosynthesis; Metabolic pathways; Biosynthesis of secondary metabolites	0.017	0.062	*39.1
4.1.1.41	(S)-methylmalonyl-CoA decarboxylase	+	Propanoate metabolism	5.35E-05	0.063	78.8
1.3.5.2	dihydroorotate dehydrogenase (quinone)	+	Pyrimidine metabolism; Metabolic pathways	5.69E-03	0.063	54.9
3.2.1.97	endo-alpha-N-acetylgalactosaminidase	+		8.23E-03	0.063	50.4
2.8.3.17	3-(aryl)acryloyl-CoA:(R)-3-(aryl)lactate CoA-transferase	+	Tropane, piperidine and pyridine alkaloid biosynthesis	8.78E-03	0.063	49.8
2.7.1.39	homoserine kinase	+	Glycine, serine and threonine metabolism; Metabolic pathways; Biosynthesis of secondary metabolites; Microbial metabolism in diverse environments	6.83E-05	0.064	78.3
3.4.24.75	lysostaphin	+		2.83E-04	0.065	74.2
1.1.1.38	malate dehydrogenase (oxaloacetate-decarboxylating)	+	Pyruvate metabolism	3.29E-04	0.065	73.6
4.2.3.3	methylglyoxal synthase	-	Propanoate metabolism; Microbial metabolism in diverse environments	9.84E-04	0.065	68.7
2.1.1.45	thymidylate synthase	+	Pyrimidine metabolism; One carbon pool by folate; Metabolic pathways	0.012	0.065	46.4
2.6.1.92	UDP-4-amino-4,6-dideoxy-N-acetyl-beta-L-altrosamine transaminase	+	Amino sugar and nucleotide sugar metabolism	0.015	0.065	42.3
3.4.17.11	glutamate carboxypeptidase	-		0.027	0.066	*31.2
1.21.4.4	betaine reductase	+		8.67E-05	0.067	78.2
4.2.1.32	L(+)-tartrate dehydratase	-	Glyoxylate and dicarboxylate metabolism	*6.89E-06	0.069	83.7
1.10.9.1	plastoquinol---plastocyanin reductase	+	Photosynthesis	0.019	0.069	*39.4
3.1.6.1	arylsulfatase (type I)	-	Steroid hormone biosynthesis; Sphingolipid metabolism	0.054	0.069	*10.8
2.8.4.3	tRNA-2-methylthio-N6-dimethylallyladenosine synthase	+		*1.97E-06	0.070	85.5
5.1.3.8	N-acylglucosamine 2-epimerase	+	Amino sugar and nucleotide sugar metabolism	3.16E-04	0.071	74.9
3.2.1.80	fructan beta-fructosidase	-	Fructose and mannose metabolism	0.016	0.072	44.7
1.14.13.48	(S)-limonene 6-monooxygenase	+		0.023	0.072	*37.8
6.3.4.2	CTP synthase (glutamine hydrolysing)	+	Pyrimidine metabolism; Metabolic pathways	*1.80E-07	0.073	88.2
2.1.3.6	putrescine carbamoyltransferase	+		3.59E-04	0.073	74.8
2.1.1.201	2-methoxy-6-polyprenyl-1,4-benzoquinol methylase	+	Ubiquinone and other terpenoid-quinone biosynthesis; Metabolic pathways; Biosynthesis of secondary metabolites	2.61E-03	0.073	64.6
2.7.7.33	glucose-1-phosphate cytidylyltransferase	-	Starch and sucrose metabolism; Amino sugar and nucleotide sugar metabolism; Metabolic pathways	5.73E-03	0.073	58.0
6.3.5.11	cobyrinate a,c-diamide synthase	-	Porphyrin and chlorophyll metabolism	*2.22E-05	0.074	82.2
1.6.5.11	NADH dehydrogenase (quinone)	-	Oxidative phosphorylation	7.91E-04	0.074	71.7
2.3.1.30	serine O-acetyltransferase	-	Cysteine and methionine metabolism; Sulfur metabolism; Metabolic pathways; Biosynthesis of secondary metabolites; Microbial metabolism in diverse environments; Biosynthesis of antibiotics	1.14E-03	0.074	69.9
2.7.2.4	aspartate kinase	+	Glycine, serine and threonine metabolism; Monobactam biosynthesis; Cysteine and methionine metabolism; Lysine biosynthesis; Metabolic pathways; Biosynthesis of secondary metabolites; Microbial metabolism in diverse environments; Biosynthesis of antibiotics	0.011	0.075	50.9
2.7.8.33	UDP-N-acetylglucosamine---undecaprenyl-phosphate N-acetylglucosaminephosphotransferase	+		0.028	0.075	*34.5
2.7.6.1	ribose-phosphate diphosphokinase	+	Pentose phosphate pathway; Purine metabolism; Metabolic pathways; Biosynthesis of secondary metabolites; Microbial metabolism in diverse environments; Biosynthesis of antibiotics	0.075	0.075	*0.0
3.4.21.88	repressor LexA	+		5.23E-05	0.076	80.8
3.4.19.11	gamma-D-glutamyl-meso-diaminopimelate peptidase	+		0.012	0.076	50.4
3.5.1.10	formyltetrahydrofolate deformylase	-	Glyoxylate and dicarboxylate metabolism; One carbon pool by folate	0.077	0.077	*0.0
3.1.3.1	alkaline phosphatase	+	**Folate biosynthesis**; Metabolic pathways	5.77E-03	0.079	59.6
6.1.1.1	tyrosine---tRNA ligase	+	Aminoacyl-tRNA biosynthesis	6.98E-03	0.079	57.6
3.1.3.7	3'(2'),5'-bisphosphate nucleotidase	-	Sulfur metabolism; Metabolic pathways; Microbial metabolism in diverse environments	6.50E-03	0.080	58.7
1.7.99.1	hydroxylamine reductase	-	Nitrogen metabolism	*8.70E-06	0.081	84.6
3.4.11.24	aminopeptidase S	-		0.081	0.081	*0.0
2.7.1.31	glycerate 3-kinase	-	Glycine, serine and threonine metabolism; Glycerolipid metabolism; Glyoxylate and dicarboxylate metabolism; Metabolic pathways; Biosynthesis of secondary metabolites; Biosynthesis of antibiotics	9.84E-05	0.082	80.1
2.7.7.41	phosphatidate cytidylyltransferase	-	Glycerophospholipid metabolism; Metabolic pathways; Biosynthesis of secondary metabolites	7.59E-03	0.082	57.5
3.1.31.1	micrococcal nuclease	+		0.023	0.082	41.3
3.5.1.1	asparaginase	-	Alanine, aspartate and glutamate metabolism; Cyanoamino acid metabolism; Metabolic pathways; Biosynthesis of secondary metabolites	*2.46E-05	0.083	83.1
3.6.3.42	beta-glucan-transporting ATPase	+		0.029	0.083	*37.1
2.4.2.53	undecaprenyl-phosphate 4-deoxy-4-formamido-L-arabinose transferase	-	Amino sugar and nucleotide sugar metabolism	2.75E-03	0.086	67.1
1.1.1.122	D-threo-aldose 1-dehydrogenase	-	Fructose and mannose metabolism; Ascorbate and aldarate metabolism; Metabolic pathways; Biosynthesis of secondary metabolites; Microbial metabolism in diverse environments	5.01E-04	0.087	75.8
2.7.7.18	nicotinate-nucleotide adenylyltransferase	-	Nicotinate and nicotinamide metabolism; Metabolic pathways	0.020	0.087	45.6
3.1.13.1	exoribonuclease II	-		*1.72E-05	0.088	84.2
2.1.3.3	ornithine carbamoyltransferase	+	Arginine biosynthesis; Metabolic pathways; Biosynthesis of secondary metabolites; Biosynthesis of antibiotics	1.28E-04	0.088	80.1
4.2.1.2	fumarate hydratase	-	Citrate cycle (TCA cycle); Pyruvate metabolism; Carbon fixation pathways in prokaryotes; Metabolic pathways; Biosynthesis of secondary metabolites; Microbial metabolism in diverse environments; Biosynthesis of antibiotics	7.65E-04	0.088	74.3
6.1.1.16	cysteine---tRNA ligase	+	Aminoacyl-tRNA biosynthesis	8.19E-04	0.088	73.9
6.3.2.7	UDP-N-acetylmuramoyl-L-alanyl-D-glutamate---L-lysine ligase	+	Peptidoglycan biosynthesis; Metabolic pathways	6.03E-03	0.088	61.4
3.4.17.13	muramoyltetrapeptide carboxypeptidase	+		0.088	0.088	*0.0
6.2.1.33	4-chlorobenzoate---CoA ligase	+	Fluorobenzoate degradation; Metabolic pathways; Microbial metabolism in diverse environments	5.74E-03	0.091	62.6
2.3.1.94	6-deoxyerythronolide-B synthase	+	Biosynthesis of 12-, 14- and 16-membered macrolides; Biosynthesis of antibiotics	0.011	0.091	55.8
3.6.3.36	taurine-transporting ATPase	+	Sulfur metabolism	1.38E-04	0.092	80.5
1.1.1.304	diacetyl reductase [(S)-acetoin forming]	+	Butanoate metabolism	2.72E-03	0.092	68.4
2.7.11.32	[pyruvate, phosphate dikinase] kinase	+		2.85E-03	0.092	68.1
2.7.4.27	[pyruvate, phosphate dikinase]-phosphate phosphotransferase	+		2.85E-03	0.092	68.1

3.1.4.17	3',5'-cyclic-nucleotide phosphodiesterase	+	Purine metabolism	0.038	0.092	*34.3
4.1.3.4	hydroxymethylglutaryl-CoA lyase	+	Synthesis and degradation of ketone bodies; Valine, leucine and isoleucine degradation; Geraniol degradation; Butanoate metabolism; Metabolic pathways	6.57E-03	0.093	61.9
3.4.15.5	peptidyl-dipeptidase Dcp	-		0.093	0.093	*0.0
4.3.2.1	argininosuccinate lyase	-	Arginine biosynthesis; Alanine, aspartate and glutamate metabolism; Metabolic pathways; Biosynthesis of secondary metabolites; Biosynthesis of antibiotics	1.29E-03	0.095	73.1
2.1.1.195	cobalt-precorrin-5B (C1)-methyltransferase	-	Porphyrin and chlorophyll metabolism; Metabolic pathways	1.74E-04	0.096	80.3
5.1.3.6	UDP-glucuronate 4-epimerase	+	Amino sugar and nucleotide sugar metabolism; Metabolic pathways	8.99E-03	0.096	59.4
1.17.1.4	xanthine dehydrogenase	-	Purine metabolism; Metabolic pathways; Microbial metabolism in diverse environments	0.018	0.096	50.5
2.3.1.n2		+		*3.02E-07	0.097	89.5
5.1.1.1	alanine racemase	+	D-Alanine metabolism; Metabolic pathways	6.10E-05	0.097	82.9
3.1.4.52	cyclic-guanylate-specific phosphodiesterase	-		1.03E-03	0.097	74.5
2.5.1.31	ditrans,polycis-undecaprenyl-diphosphate synthase [(2E,6E)-farnesyl-diphosphate specific]	-	Terpenoid backbone biosynthesis; Biosynthesis of secondary metabolites	2.25E-03	0.097	70.5
3.5.4.19	phosphoribosyl-AMP cyclohydrolase	-	Histidine metabolism; Metabolic pathways; Biosynthesis of secondary metabolites	0.015	0.097	53.6
3.2.1.131	xylan alpha-1,2-glucuronosidase	-		1.72E-03	0.098	72.2
1.17.1.8	4-hydroxy-tetrahydrodipicolinate reductase	+	Monobactam biosynthesis; Lysine biosynthesis; Metabolic pathways; Biosynthesis of secondary metabolites; Microbial metabolism in diverse environments; Biosynthesis of antibiotics	*2.87E-05	0.099	84.5
6.1.1.5	isoleucine---tRNA ligase	+	Aminoacyl-tRNA biosynthesis	*4.08E-06	0.100	87.3
6.3.5.5	carbamoyl-phosphate synthase (glutamine-hydrolysing)	-	Pyrimidine metabolism; Alanine, aspartate and glutamate metabolism; Metabolic pathways	3.68E-04	0.100	78.7
4.2.2.8	heparin-sulfate lyase	+		0.100	0.100	*0.0
4.3.1.12	ornithine cyclodeaminase	+	**Arginine and proline metabolism**; Biosynthesis of secondary metabolites; Biosynthesis of antibiotics	1.45E-04	0.101	81.4
3.5.4.16	GTP cyclohydrolase I	-	**Folate biosynthesis**; Metabolic pathways	1.42E-03	0.101	73.6
2.1.4.1	glycine amidinotransferase	+	Glycine, serine and threonine metabolism; **Arginine and proline metabolism**; Metabolic pathways	7.63E-05	0.102	82.9
2.3.1.180	beta-ketoacyl-[acyl-carrier-protein] synthase III	-	Fatty acid biosynthesis; Metabolic pathways	2.04E-04	0.102	80.6
2.1.1.80	protein-glutamate O-methyltransferase	-		0.103	0.103	*0.0
6.1.1.4	leucine---tRNA ligase	+	Aminoacyl-tRNA biosynthesis	3.21E-03	0.104	69.5
3.2.1.177	alpha-D-xyloside xylohydrolase	-		0.026	0.104	46.7
2.2.1.6	acetolactate synthase	-	Valine, leucine and isoleucine biosynthesis; Butanoate metabolism; C5-Branched dibasic acid metabolism; Pantothenate and CoA biosynthesis; Metabolic pathways; Biosynthesis of secondary metabolites; Biosynthesis of antibiotics	*2.73E-05	0.105	85.1
4.3.1.18	D-serine ammonia-lyase	+	Glycine, serine and threonine metabolism	3.58E-04	0.105	79.4
1.2.7.7	3-methyl-2-oxobutanoate dehydrogenase (ferredoxin)	-	Valine, leucine and isoleucine degradation; Metabolic pathways	6.72E-04	0.106	77.4
2.3.1.128	ribosomal-protein-alanine N-acetyltransferase	+		0.027	0.106	46.6
2.3.1.183	phosphinothricin acetyltransferase	-	Phosphonate and phosphinate metabolism; Biosynthesis of antibiotics	0.106	0.106	*0.0
3.5.99.5	2-aminomuconate deaminase	+	Tryptophan metabolism	0.061	0.108	*26.3
2.7.2.7	butyrate kinase	+	Butanoate metabolism; Metabolic pathways	0.109	0.109	*0.0
3.2.1.21	beta-glucosidase	-	Cyanoamino acid metabolism; Starch and sucrose metabolism; Phenylpropanoid biosynthesis; Metabolic pathways; Biosynthesis of secondary metabolites	5.49E-04	0.110	78.6
2.7.8.40	UDP-N-acetylgalactosamine-undecaprenyl-phosphate N-acetylgalactosaminephosphotransferase	+		3.73E-03	0.110	69.6
4.2.1.55	3-hydroxybutyryl-CoA dehydratase	-	Glyoxylate and dicarboxylate metabolism; Butanoate metabolism; Microbial metabolism in diverse environments	1.47E-03	0.111	74.9
2.7.7.60	2-C-methyl-D-erythritol 4-phosphate cytidylyltransferase	-	Terpenoid backbone biosynthesis; Metabolic pathways; Biosynthesis of secondary metabolites; Biosynthesis of antibiotics	3.44E-04	0.112	80.3
3.2.1.50	alpha-N-acetylglucosaminidase	-	Glycosaminoglycan degradation; Metabolic pathways	4.61E-04	0.113	79.6
4.1.2.40	tagatose-bisphosphate aldolase	-	Galactose metabolism; Metabolic pathways	8.13E-04	0.113	77.6
2.8.3.5	3-oxoacid CoA-transferase	+	Synthesis and degradation of ketone bodies; Valine, leucine and isoleucine degradation; Butanoate metabolism	1.65E-03	0.113	74.6
3.2.2.20	DNA-3-methyladenine glycosylase I	+		0.077	0.113	*19.5
2.4.1.248	cycloisomaltooligosaccharide glucanotransferase	+		0.025	0.114	50.3
5.1.1.3	glutamate racemase	-	D-Glutamine and D-glutamate metabolism; Metabolic pathways	3.55E-03	0.115	70.9
1.1.1.369	D-chiro-inositol 1-dehydrogenase	-	Inositol phosphate metabolism; Microbial metabolism in diverse environments	3.78E-03	0.115	70.3
2.7.1.191	protein-Npi-phosphohistidine---D-mannose phosphotransferase	-	Fructose and mannose metabolism; Amino sugar and nucleotide sugar metabolism; Metabolic pathways	0.025	0.115	50.3
3.5.2.17	hydroxyisourate hydrolase	-	Purine metabolism; Metabolic pathways; Microbial metabolism in diverse environments	0.039	0.115	41.7
3.1.1.47	1-alkyl-2-acetylglycerophosphocholine esterase	+	Ether lipid metabolism; Metabolic pathways	0.022	0.116	52.9
2.1.1.63	methylated-DNA---[protein]-cysteine S-methyltransferase	+		0.116	0.116	*0.0
4.1.3.6	citrate (pro-3S)-lyase	+		0.015	0.118	58.4
2.7.1.24	dephospho-CoA kinase	+	Pantothenate and CoA biosynthesis; Metabolic pathways	3.19E-04	0.119	81.2
1.1.1.159	7alpha-hydroxysteroid dehydrogenase	+	Secondary bile acid biosynthesis	0.029	0.119	49.3
4.2.1.113	o-succinylbenzoate synthase	-	Ubiquinone and other terpenoid-quinone biosynthesis; Metabolic pathways; Biosynthesis of secondary metabolites	9.20E-03	0.121	64.6
2.4.1.230	kojibiose phosphorylase	+		0.082	0.123	*21.3
3.1.1.3	triacylglycerol lipase	+	Glycerolipid metabolism; Metabolic pathways	3.65E-03	0.124	72.1
2.7.2.1	acetate kinase	+	Taurine and hypotaurine metabolism; Pyruvate metabolism; Propanoate metabolism; Methane metabolism; Carbon fixation pathways in prokaryotes; Metabolic pathways; Microbial metabolism in diverse environments	8.51E-03	0.125	66.0
3.2.1.35	hyaluronoglucosaminidase	+	Glycosaminoglycan degradation; Metabolic pathways	0.019	0.125	57.2
6.2.1.1	acetate---CoA ligase	+	Glycolysis / Gluconeogenesis; Pyruvate metabolism; Propanoate metabolism; Methane metabolism; Carbon fixation pathways in prokaryotes; Metabolic pathways; Biosynthesis of secondary metabolites; Microbial metabolism in diverse environments; Biosynthesis of antibiotics	0.013	0.126	61.7
1.1.1.267	1-deoxy-D-xylulose-5-phosphate reductoisomerase	-	Terpenoid backbone biosynthesis; Metabolic pathways; Biosynthesis of secondary metabolites; Biosynthesis of antibiotics	0.015	0.126	60.7
1.2.4.2	oxoglutarate dehydrogenase (succinyl-transferring)	+	Citrate cycle (TCA cycle); Lysine degradation; Tryptophan metabolism; Metabolic pathways; Biosynthesis of secondary metabolites; Microbial metabolism in diverse environments; Biosynthesis of antibiotics	0.020	0.127	57.1
2.4.1.289	N-acetylglucosaminyl-diphospho-decaprenol L-rhamnosyltransferase	+		0.087	0.127	*20.5
3.6.4.6	vesicle-fusing ATPase	+		8.68E-04	0.129	79.3
2.4.2.4	thymidine phosphorylase	+	Pyrimidine metabolism; Drug metabolism - other enzymes; Metabolic pathways	0.078	0.129	*25.5
3.5.4.2	adenine deaminase	+	Purine metabolism; Metabolic pathways	9.94E-04	0.130	78.8
2.4.1.290	N,N'-diacetylbacillosaminyl-diphospho-undecaprenol alpha-1,3-N-acetylgalactosaminyltransferase	+		0.133	0.133	*0.0
3.1.3.73	adenosylcobalamin/alpha-ribazole phosphatase	-	Porphyrin and chlorophyll metabolism; Metabolic pathways	8.35E-03	0.135	67.9
1.2.1.41	glutamate-5-semialdehyde dehydrogenase	-	**Arginine and proline metabolism**; Carbapenem biosynthesis; Metabolic pathways; Biosynthesis of secondary metabolites; Biosynthesis of antibiotics	0.029	0.135	53.5
2.3.1.28	chloramphenicol O-acetyltransferase	+		0.082	0.136	*26.8
2.7.7.63	lipoate---protein ligase	+		8.56E-03	0.137	68.1
3.4.21.102	C-terminal processing peptidase	+		0.137	0.137	*0.0
6.3.4.19	tRNAIle-lysidine synthase	+		1.82E-03	0.138	77.3
3.2.1.37	xylan 1,4-beta-xylosidase	-	Amino sugar and nucleotide sugar metabolism; Metabolic pathways	0.139	0.139	*0.0
5.3.1.16	1-(5-phosphoribosyl)-5-[(5-phosphoribosylamino)methylideneamino]imidazole-4-carboxamide isomerase	-	Histidine metabolism; Metabolic pathways; Biosynthesis of secondary metabolites	7.93E-03	0.140	69.1
1.1.1.90	aryl-alcohol dehydrogenase	+	Tyrosine metabolism; Phenylalanine metabolism; Xylene degradation; Toluene degradation; Metabolic pathways; Microbial metabolism in diverse environments	5.03E-03	0.141	72.4
4.2.2.5	chondroitin AC lyase	+		0.094	0.141	*22.8
4.2.1.51	prephenate dehydratase	-	Phenylalanine, tyrosine and tryptophan biosynthesis; Metabolic pathways; Biosynthesis of secondary metabolites; Biosynthesis of antibiotics	8.45E-03	0.142	68.9
1.1.1.274	2,5-didehydrogluconate reductase (2-dehydro-D-gluconate-forming)	+		0.019	0.144	61.2
3.1.3.4	phosphatidate phosphatase	+	Glycerolipid metabolism; Glycerophospholipid metabolism; Ether lipid metabolism; Sphingolipid metabolism; Metabolic pathways; Biosynthesis of secondary metabolites	0.022	0.144	59.2
4.2.1.46	dTDP-glucose 4,6-dehydratase	-	Streptomycin biosynthesis; Polyketide sugar unit biosynthesis; Acarbose and validamycin biosynthesis; Biosynthesis of vancomycin group antibiotics; Biosynthesis of antibiotics	0.038	0.144	50.6
1.11.1.5	cytochrome-c peroxidase	+		0.088	0.144	*26.5
1.1.1.125	2-deoxy-D-gluconate 3-dehydrogenase	-		3.91E-03	0.145	74.4
2.3.1.174	3-oxoadipyl-CoA thiolase	+	Phenylalanine metabolism; Benzoate degradation; Microbial metabolism in diverse environments	0.013	0.145	65.6
3.1.3.103	3-deoxy-D-glycero-D-galacto-nonulopyranosonate 9-phosphatase	+		0.145	0.145	*0.0
1.3.1.54	precorrin-6A reductase	-	Porphyrin and chlorophyll metabolism; Metabolic pathways	3.30E-03	0.146	75.5
1.1.1.140	sorbitol-6-phosphate 2-dehydrogenase	+	Fructose and mannose metabolism	0.083	0.146	*29.8
1.3.1.1	dihydropyrimidine dehydrogenase (NAD+)	-	Pyrimidine metabolism; beta-Alanine metabolism; Pantothenate and CoA biosynthesis; Metabolic pathways	0.093	0.146	*24.9
3.1.3.45	3-deoxy-manno-octulosonate-8-phosphatase	+	Lipopolysaccharide biosynthesis; Metabolic pathways	0.140	0.146	*3.1
1.6.99.1	NADPH dehydrogenase	-		9.91E-03	0.147	68.4
3.1.2.6	hydroxyacylglutathione hydrolase	+	Pyruvate metabolism	0.039	0.148	50.8
2.6.99.2	pyridoxine 5'-phosphate synthase	-	Vitamin B6 metabolism; Metabolic pathways	0.031	0.149	55.3
2.7.7.24	glucose-1-phosphate thymidylyltransferase	-	Streptomycin biosynthesis; Polyketide sugar unit biosynthesis; Acarbose and validamycin biosynthesis; Biosynthesis of antibiotics	0.149	0.149	*0.0
1.3.7.9	4-hydroxybenzoyl-CoA reductase	-	Benzoate degradation; Aminobenzoate degradation; Metabolic pathways; Microbial metabolism in diverse environments	6.64E-04	0.150	82.1
5.3.1.9	glucose-6-phosphate isomerase	+	Glycolysis / Gluconeogenesis; Pentose phosphate pathway; Starch and sucrose metabolism; Amino sugar and nucleotide sugar metabolism; Metabolic pathways; Biosynthesis of secondary metabolites; Microbial metabolism in diverse environments; Biosynthesis of antibiotics	2.73E-04	0.154	84.7
3.1.1.23	acylglycerol lipase	+	Glycerolipid metabolism; Metabolic pathways	1.32E-03	0.155	80.4
5.99.1.3	DNA topoisomerase (ATP-hydrolysing)	+		4.73E-03	0.155	74.7
3.2.1.n2		-		*2.04E-05	0.156	88.9
6.3.4.3	formate---tetrahydrofolate ligase	+	One carbon pool by folate; Carbon fixation pathways in prokaryotes; Metabolic pathways; Microbial metabolism in diverse environments	3.78E-03	0.156	76.1
2.7.7.8	polyribonucleotide nucleotidyltransferase	+	Purine metabolism; Pyrimidine metabolism	6.51E-03	0.156	72.8
3.4.17.19	carboxypeptidase Taq	+		2.88E-04	0.157	84.7
2.4.1.211	1,3-beta-galactosyl-N-acetylhexosamine phosphorylase	-		0.044	0.157	50.7
3.5.4.32	8-oxoguanine deaminase	-		0.157	0.157	*0.0
3.2.2.8	ribosylpyrimidine nucleosidase	+	Purine metabolism; Pyrimidine metabolism; Metabolic pathways	0.021	0.158	62.9
2.2.1.7	1-deoxy-D-xylulose-5-phosphate synthase	-	Thiamine metabolism; Terpenoid backbone biosynthesis; Metabolic pathways; Biosynthesis of secondary metabolites; Biosynthesis of antibiotics	1.56E-04	0.159	86.1
2.7.4.7	phosphooxymethylpyrimidine kinase	-	Thiamine metabolism; Metabolic pathways	9.76E-04	0.159	81.8
5.1.3.7	UDP-N-acetylglucosamine 4-epimerase	+	Amino sugar and nucleotide sugar metabolism; Metabolic pathways	8.33E-03	0.160	71.6
4.1.1.61	4-hydroxybenzoate decarboxylase	+	Aminobenzoate degradation; Microbial metabolism in diverse environments	0.026	0.160	60.0
2.7.7.10	UTP---hexose-1-phosphate uridylyltransferase	+	Galactose metabolism; Amino sugar and nucleotide sugar metabolism; Metabolic pathways	2.73E-03	0.161	78.1
2.5.1.16	spermidine synthase	-	Cysteine and methionine metabolism; **Arginine and proline metabolism**; beta-Alanine metabolism; Glutathione metabolism; Metabolic pathways	1.76E-04	0.162	86.1
3.1.2.28	1,4-dihydroxy-2-naphthoyl-CoA hydrolase	+	Ubiquinone and other terpenoid-quinone biosynthesis; Metabolic pathways; Biosynthesis of secondary metabolites	0.010	0.162	70.2

4.2.1.115	UDP-N-acetylglucosamine 4,6-dehydratase (configuration-inverting)	+	Amino sugar and nucleotide sugar metabolism	4.88E-03	0.163	75.4
2.8.1.13	tRNA-uridine 2-sulfurtransferase	-		9.32E-04	0.164	82.3
3.6.1.31	phosphoribosyl-ATP diphosphatase	-	Histidine metabolism; Metabolic pathways; Biosynthesis of secondary metabolites	6.82E-03	0.166	73.8
4.2.1.91	arogenate dehydratase	+	Phenylalanine, tyrosine and tryptophan biosynthesis; Metabolic pathways; Biosynthesis of secondary metabolites; Biosynthesis of antibiotics	2.45E-03	0.167	79.2
5.3.2.3	TDP-4-oxo-6-deoxy-alpha-D-glucose-3,4-oxoisomerase (dTDP-3-dehydro-6-deoxy-alpha-D-galactopyranose-forming)	+	Polyketide sugar unit biosynthesis; Biosynthesis of antibiotics	0.024	0.167	62.7
6.4.1.3	propionyl-CoA carboxylase	+	Valine, leucine and isoleucine degradation; Glyoxylate and dicarboxylate metabolism; Propanoate metabolism; Carbon fixation pathways in prokaryotes; Metabolic pathways; Microbial metabolism in diverse environments; Biosynthesis of antibiotics	0.042	0.167	54.0
3.6.3.53	Ag+-exporting ATPase	+		0.167	0.167	*0.0
1.1.1.50	3alpha-hydroxysteroid 3-dehydrogenase (Si-specific)	+	Primary bile acid biosynthesis; Steroid hormone biosynthesis; Metabolic pathways	7.26E-03	0.168	73.7
2.1.1.170	16S rRNA (guanine527-N7)-methyltransferase	+		0.053	0.168	49.2
3.1.21.3	type I site-specific deoxyribonuclease	+		0.087	0.171	*35.9
1.3.1.10	enoyl-[acyl-carrier-protein] reductase (NADPH, Si-specific)	+	Fatty acid biosynthesis; **Biotin metabolism**; Metabolic pathways	0.042	0.172	54.8
4.1.1.12	aspartate 4-decarboxylase	+	Alanine, aspartate and glutamate metabolism; Cysteine and methionine metabolism; Metabolic pathways	0.173	0.173	*0.0
1.1.1.4	(R,R)-butanediol dehydrogenase	+	Butanoate metabolism	0.021	0.174	65.1
3.1.3.68	2-deoxyglucose-6-phosphatase	+		0.135	0.174	*17
4.1.3.16	4-hydroxy-2-oxoglutarate aldolase	-	**Arginine and proline metabolism**; Glyoxylate and dicarboxylate metabolism; Metabolic pathways	6.39E-04	0.175	84.2
2.7.7.75	molybdopterin adenylyltransferase	-	**Folate biosynthesis**; Metabolic pathways	1.08E-03	0.175	82.8
3.4.11.18	methionyl aminopeptidase	-		1.58E-03	0.175	81.5
2.7.1.58	2-dehydro-3-deoxygalactonokinase	-	Galactose metabolism; Metabolic pathways	8.99E-03	0.175	73.1
2.5.1.74	1,4-dihydroxy-2-naphthoate polyprenyltransferase	-	Ubiquinone and other terpenoid-quinone biosynthesis; Metabolic pathways; Biosynthesis of secondary metabolites	0.026	0.176	63.1
2.6.1.33	dTDP-4-amino-4,6-dideoxy-D-glucose transaminase	+	Polyketide sugar unit biosynthesis; Acarbose and validamycin biosynthesis; Biosynthesis of antibiotics	0.080	0.176	40.2
2.6.1.52	phosphoserine transaminase	+	Glycine, serine and threonine metabolism; Methane metabolism; Vitamin B6 metabolism; Metabolic pathways; Microbial metabolism in diverse environments; Biosynthesis of antibiotics	3.77E-03	0.177	78.3
2.7.7.40	D-ribitol-5-phosphate cytidylyltransferase	+	Pentose and glucuronate interconversions; Mannose type O-glycan biosynthesis; Metabolic pathways	3.08E-03	0.178	79.3
3.2.1.99	arabinan endo-1,5-alpha-L-arabinanase	-		6.33E-03	0.178	75.6
1.4.4.2	glycine dehydrogenase (aminomethyl-transferring)	+	Glycine, serine and threonine metabolism; Metabolic pathways	0.017	0.178	68.1
1.2.1.62	4-formylbenzenesulfonate dehydrogenase	+	Toluene degradation; Microbial metabolism in diverse environments	0.048	0.178	53.5
5.4.2.10	phosphoglucosamine mutase	+	Amino sugar and nucleotide sugar metabolism; Metabolic pathways; Biosynthesis of antibiotics	4.74E-04	0.179	85.2
3.2.1.73	licheninase	+		0.141	0.179	*16.7
2.6.1.59	dTDP-4-amino-4,6-dideoxygalactose transaminase	+		1.86E-04	0.180	87.1
3.4.23.36	signal peptidase II	+		6.69E-04	0.180	84.5
1.3.1.98	UDP-N-acetylmuramate dehydrogenase	+	Amino sugar and nucleotide sugar metabolism; Peptidoglycan biosynthesis; Metabolic pathways	6.64E-04	0.181	84.6
4.1.1.20	diaminopimelate decarboxylase	-	Lysine biosynthesis; Metabolic pathways; Biosynthesis of secondary metabolites; Microbial metabolism in diverse environments; Biosynthesis of antibiotics	0.050	0.182	53.4
4.2.1.9	dihydroxy-acid dehydratase	-	Valine, leucine and isoleucine biosynthesis; Pantothenate and CoA biosynthesis; Metabolic pathways; Biosynthesis of secondary metabolites; Biosynthesis of antibiotics	0.011	0.183	72.8
4.1.1.82	phosphonopyruvate decarboxylase	+	Phosphonate and phosphinate metabolism; Metabolic pathways; Microbial metabolism in diverse environments; Biosynthesis of antibiotics	0.183	0.183	*0.0
3.4.21.53	endopeptidase La	+		4.17E-05	0.184	89.5
3.6.3.32	quaternary-amine-transporting ATPase	+		4.45E-04	0.184	85.7
2.1.2.10	aminomethyltransferase	+	Glycine, serine and threonine metabolism; One carbon pool by folate; Metabolic pathways	0.038	0.184	59.0
2.7.4.3	adenylate kinase	+	Purine metabolism; Thiamine metabolism; Metabolic pathways; Biosynthesis of secondary metabolites; Biosynthesis of antibiotics	2.18E-03	0.186	81.3
2.7.1.23	NAD+ kinase	-	Nicotinate and nicotinamide metabolism; Metabolic pathways	0.081	0.186	42.5
1.1.1.262	4-hydroxythreonine-4-phosphate dehydrogenase	-	Vitamin B6 metabolism; Metabolic pathways	0.186	0.186	*0.0
2.3.2.3	lysyltransferase	+		0.012	0.188	72.7
2.7.2.11	glutamate 5-kinase	-	**Arginine and proline metabolism**; Carbapenem biosynthesis; Metabolic pathways; Biosynthesis of antibiotics	3.72E-03	0.189	79.5
3.6.3.40	teichoic-acid-transporting ATPase	-		2.51E-03	0.190	81.2
3.5.1.2	glutaminase	-	Arginine biosynthesis; Alanine, aspartate and glutamate metabolism; D-Glutamine and D-glutamate metabolism; Metabolic pathways	0.044	0.190	57.6
3.11.1.3	phosphonopyruvate hydrolase	+	Phosphonate and phosphinate metabolism	0.054	0.190	53.8
4.2.1.68	L-fuconate dehydratase	-	Fructose and mannose metabolism; Microbial metabolism in diverse environments	0.141	0.191	*21.0
2.1.1.148	thymidylate synthase (FAD)	+	Pyrimidine metabolism; One carbon pool by folate; Metabolic pathways	4.81E-03	0.192	78.6
3.5.1.14	N-acyl-aliphatic-L-amino acid amidohydrolase	+	Arginine biosynthesis; Metabolic pathways; Biosynthesis of secondary metabolites; Biosynthesis of antibiotics	6.28E-03	0.192	77.2
3.5.4.5	cytidine deaminase	-	Pyrimidine metabolism; Drug metabolism - other enzymes; Metabolic pathways	0.010	0.194	74.2
6.3.2.29	cyanophycin synthase (L-aspartate-adding)	+		0.057	0.194	53.4
1.3.1.91	tRNA-dihydrouridine20 synthase [NAD(P)+]	-		0.078	0.194	45.6
1.2.1.39	phenylacetaldehyde dehydrogenase	+	Phenylalanine metabolism; Styrene degradation; Metabolic pathways; Microbial metabolism in diverse environments	0.029	0.195	64.9
4.1.1.8	oxalyl-CoA decarboxylase	+	Glyoxylate and dicarboxylate metabolism; Metabolic pathways	0.045	0.195	58.3
2.1.1.43	histone-lysine N-methyltransferase	+	Lysine degradation	*7.13E-11	0.196	96.1
2.1.1.189	23S rRNA (uracil747-C5)-methyltransferase	-		1.44E-03	0.196	83.5
4.2.1.107	3alpha,7alpha,12alpha-trihydroxy-5beta-cholest-24-enoyl-CoA hydratase	+	Primary bile acid biosynthesis; Metabolic pathways	4.32E-03	0.196	79.5
2.1.1.10	homocysteine S-methyltransferase	+	Cysteine and methionine metabolism; Metabolic pathways; Biosynthesis of secondary metabolites	3.92E-03	0.198	80.1
3.1.21.4	type II site-specific deoxyribonuclease	+		0.143	0.199	*23.3
2.4.1.83	dolichyl-phosphate beta-D-mannosyltransferase	-	N-Glycan biosynthesis; Metabolic pathways	0.200	0.200	*0.0
1.3.1.14	dihydroorotate dehydrogenase (NAD+)	-	Pyrimidine metabolism; Metabolic pathways	0.021	0.202	69.2
2.3.1.15	glycerol-3-phosphate 1-O-acyltransferase	-	Glycerolipid metabolism; Glycerophospholipid metabolism; Metabolic pathways; Biosynthesis of secondary metabolites	0.032	0.203	64.6
7.3.2.1	ABC-type phosphate transporter	+		0.042	0.203	60.8
6.3.5.2	GMP synthase (glutamine-hydrolysing)	+	Purine metabolism; Drug metabolism - other enzymes; Metabolic pathways	6.47E-03	0.204	78.3
2.7.1.6	galactokinase	-	Galactose metabolism; Amino sugar and nucleotide sugar metabolism; Metabolic pathways	3.08E-03	0.205	81.7
1.1.1.88	hydroxymethylglutaryl-CoA reductase	+	Terpenoid backbone biosynthesis; Biosynthesis of secondary metabolites; Biosynthesis of antibiotics	0.038	0.207	63.0
6.3.2.2	glutamate---cysteine ligase	+	Cysteine and methionine metabolism; Glutathione metabolism; Metabolic pathways	0.045	0.208	60.4
1.1.1.14	L-iditol 2-dehydrogenase	-	Pentose and glucuronate interconversions; Fructose and mannose metabolism; Metabolic pathways	0.014	0.209	73.8
2.7.8.6	undecaprenyl-phosphate galactose phosphotransferase	+		0.077	0.209	49.5
5.3.1.26	galactose-6-phosphate isomerase	+	Galactose metabolism; Metabolic pathways	3.62E-03	0.210	81.4
2.7.7.43	N-acylneuraminate cytidylyltransferase	-	Amino sugar and nucleotide sugar metabolism; Metabolic pathways	0.057	0.211	56.6
5.99.1.2	DNA topoisomerase	-		0.077	0.211	49.9
5.1.1.13	aspartate racemase	+	Alanine, aspartate and glutamate metabolism	4.84E-03	0.212	80.4
3.4.11.5	prolyl aminopeptidase	+	**Arginine and proline metabolism**	0.015	0.212	73.6
1.3.1.43	arogenate dehydrogenase	-	Phenylalanine, tyrosine and tryptophan biosynthesis; Novobiocin biosynthesis; Metabolic pathways; Biosynthesis of secondary metabolites	0.021	0.212	70.9
3.1.13.5	ribonuclease D	+		0.030	0.213	67.0
1.1.1.29	glycerate dehydrogenase	+	Glycine, serine and threonine metabolism; Glyoxylate and dicarboxylate metabolism; Methane metabolism; Metabolic pathways; Biosynthesis of secondary metabolites; Microbial metabolism in diverse environments; Biosynthesis of antibiotics	0.032	0.214	66.4
2.4.2.19	nicotinate-nucleotide diphosphorylase (carboxylating)	-	Nicotinate and nicotinamide metabolism; Metabolic pathways	0.054	0.214	58.5
3.4.24.57	O-sialoglycoprotein endopeptidase	+		9.46E-03	0.215	77.2
3.5.1.47	N-acetyldiaminopimelate deacetylase	+	Lysine biosynthesis; Metabolic pathways; Biosynthesis of secondary metabolites	2.24E-03	0.217	83.7
1.18.6.1	nitrogenase	-	Chloroalkane and chloroalkene degradation; Nitrogen metabolism; Metabolic pathways; Microbial metabolism in diverse environments	8.84E-03	0.217	77.8
3.2.1.165	exo-1,4-beta-D-glucosaminidase	-	Amino sugar and nucleotide sugar metabolism; Metabolic pathways	0.198	0.217	*8.0
3.1.26.3	ribonuclease III	+		1.07E-04	0.218	89.9
5.1.3.15	glucose-6-phosphate 1-epimerase	+	Glycolysis / Gluconeogenesis; Metabolic pathways; Biosynthesis of secondary metabolites; Microbial metabolism in diverse environments; Biosynthesis of antibiotics	0.014	0.218	74.6
3.1.11.1	exodeoxyribonuclease I	+		0.072	0.218	53.2
3.5.2.5	allantoinase	-	Purine metabolism; Metabolic pathways; Microbial metabolism in diverse environments	8.13E-03	0.219	78.5
3.6.1.22	NAD+ diphosphatase	-	Nicotinate and nicotinamide metabolism; Metabolic pathways	0.076	0.219	52.0
1.8.99.3	hydrogensulfite reductase	-		2.22E-03	0.220	83.9
2.4.1.280	N,N'-diacetylchitobiose phosphorylase	+	Amino sugar and nucleotide sugar metabolism	0.172	0.220	*19.2
1.1.1.37	malate dehydrogenase	+	Citrate cycle (TCA cycle); Cysteine and methionine metabolism; Pyruvate metabolism; Glyoxylate and dicarboxylate metabolism; Methane metabolism; Carbon fixation in photosynthetic organisms; Carbon fixation pathways in prokaryotes; Metabolic pathways; Biosynthesis of secondary metabolites; Microbial metabolism in diverse environments; Biosynthesis of antibiotics	0.220	0.220	*0.0
4.3.1.4	formimidoyltetrahydrofolate cyclodeaminase	-	One carbon pool by folate; Metabolic pathways	0.220	0.220	*0.0
2.3.3.16	citrate synthase (unknown stereospecificity)	+		0.073	0.221	53.4
4.3.3.7	4-hydroxy-tetrahydrodipicolinate synthase	+	Monobactam biosynthesis; Lysine biosynthesis; Metabolic pathways; Biosynthesis of secondary metabolites; Microbial metabolism in diverse environments; Biosynthesis of antibiotics	0.023	0.222	70.9
5.4.99.5	chorismate mutase	+	Phenylalanine, tyrosine and tryptophan biosynthesis; Metabolic pathways; Biosynthesis of secondary metabolites; Biosynthesis of antibiotics	0.061	0.222	57.5
6.1.1.23	aspartate---tRNAAsn ligase	+	Aminoacyl-tRNA biosynthesis	0.013	0.224	75.9
4.6.1.1	adenylate cyclase	+	Purine metabolism	4.40E-03	0.225	81.9
2.3.1.266	[ribosomal protein S18]-alanine N-acetyltransferase	+		5.37E-03	0.226	81.1
3.1.1.81	quorum-quenching N-acyl-homoserine lactonase	+		0.018	0.227	73.8
3.4.14.4	dipeptidyl-peptidase III	+		5.66E-03	0.229	81.1
2.7.7.7	DNA-directed DNA polymerase	+	Purine metabolism; Pyrimidine metabolism; Metabolic pathways	2.41E-04	0.230	89.3
5.5.1.2	3-carboxy-cis,cis-muconate cycloisomerase	+	Benzoate degradation; Metabolic pathways; Microbial metabolism in diverse environments	0.164	0.230	*25.4
1.6.1.1	NAD(P)+ transhydrogenase (Si-specific)	-	Nicotinate and nicotinamide metabolism; Metabolic pathways	0.032	0.231	69.0
2.7.4.23	ribose 1,5-bisphosphate phosphokinase	+	Pentose phosphate pathway	0.033	0.231	68.6
4.3.99.2	carboxybiotin decarboxylase	+		0.142	0.231	*33.5
4.1.99.22	GTP 3',8-cyclase	-	**Folate biosynthesis**; Metabolic pathways	1.05E-03	0.233	86.8
3.5.4.3	guanine deaminase	-	Purine metabolism; Metabolic pathways	0.029	0.234	70.2
1.4.1.4	glutamate dehydrogenase (NADP+)	+	Arginine biosynthesis; Alanine, aspartate and glutamate metabolism; Nitrogen metabolism; Metabolic pathways	0.051	0.234	62.7
2.3.3.14	homocitrate synthase	+	Lysine biosynthesis; Pyruvate metabolism; Metabolic pathways; Microbial metabolism in diverse environments; Biosynthesis of antibiotics	0.234	0.234	*0.0
2.1.1.77	protein-L-isoaspartate(D-aspartate) O-methyltransferase	+		0.013	0.235	77.2
1.1.1.86	ketol-acid reductoisomerase (NADP+)	-	Valine, leucine and isoleucine biosynthesis; Pantothenate and CoA biosynthesis; Metabolic pathways; Biosynthesis of secondary metabolites; Biosynthesis of antibiotics	0.021	0.235	73.7
1.5.5.2	proline dehydrogenase	+	**Arginine and proline metabolism**; Metabolic pathways; Biosynthesis of secondary metabolites; Biosynthesis of antibiotics	0.087	0.235	51.6
3.5.3.3	creatinase	+	Glycine, serine and threonine metabolism; **Arginine and proline metabolism**; Metabolic pathways	0.116	0.235	43.0

1.2.1.9	glyceraldehyde-3-phosphate dehydrogenase (NADP+)	+	Glycolysis / Gluconeogenesis; Pentose phosphate pathway; Metabolic pathways; Microbial metabolism in diverse environments	3.05E-03	0.238	84.2
3.4.23.43	prepilin peptidase	+		0.022	0.240	73.8
3.1.3.83	D-glycero-alpha-D-manno-heptose 1,7-bisphosphate 7-phosphatase	+	Lipopolysaccharide biosynthesis	0.030	0.240	70.5
3.5.5.7	aliphatic nitrilase	+	Styrene degradation; Microbial metabolism in diverse environments	0.053	0.243	63.6
3.4.21.89	signal peptidase I	-		0.082	0.243	54.8
1.1.1.42	isocitrate dehydrogenase (NADP+)	-	Citrate cycle (TCA cycle); Glutathione metabolism; Carbon fixation pathways in prokaryotes; Metabolic pathways; Biosynthesis of secondary metabolites; Microbial metabolism in diverse environments; Biosynthesis of antibiotics	0.186	0.243	*22.1
1.2.7.4	anaerobic carbon monoxide dehydrogenase	+	Nitrotoluene degradation; Methane metabolism; Carbon fixation pathways in prokaryotes; Microbial metabolism in diverse environments	6.89E-04	0.244	88.2
1.2.1.68	coniferyl-aldehyde dehydrogenase	+	Phenylpropanoid biosynthesis	0.028	0.245	72.0
2.7.1.29	glycerone kinase	+	Glycerolipid metabolism; Methane metabolism; Metabolic pathways; Microbial metabolism in diverse environments	0.053	0.245	63.9
3.1.2.12	S-formylglutathione hydrolase	-	Methane metabolism; Microbial metabolism in diverse environments	0.040	0.246	68.1
3.2.1.169	protein O-GlcNAcase	+		0.164	0.246	*30.3
6.1.1.3	threonine---tRNA ligase	+	Aminoacyl-tRNA biosynthesis	0.017	0.247	76.6
1.6.99.3	NADH dehydrogenase	+	Oxidative phosphorylation; Metabolic pathways	0.019	0.247	75.6
2.1.1.61	tRNA 5-(aminomethyl)-2-thiouridylate-methyltransferase	-		0.011	0.248	79.3
3.6.4.12	DNA helicase	+		1.59E-03	0.249	86.6
3.6.1.57	UDP-2,4-diacetamido-2,4,6-trideoxy-beta-L-altropyranose hydrolase	+	Amino sugar and nucleotide sugar metabolism; Biosynthesis of antibiotics	0.037	0.250	69.6
6.1.1.6	lysine---tRNA ligase	+	Aminoacyl-tRNA biosynthesis	0.021	0.251	75.2
4.2.1.53	oleate hydratase	-		0.076	0.251	58.0
3.6.1.23	dUTP diphosphatase	-	Pyrimidine metabolism; Drug metabolism - other enzymes; Metabolic pathways	0.080	0.251	56.9
3.4.24.71	endothelin-converting enzyme 1	-		0.251	0.251	*0.0
2.1.1.157	sarcosine/dimethylglycine N-methyltransferase	+	Glycine, serine and threonine metabolism	0.011	0.252	79.5
1.5.1.5	methylenetetrahydrofolate dehydrogenase (NADP+)	+	One carbon pool by folate; Carbon fixation pathways in prokaryotes; Metabolic pathways; Microbial metabolism in diverse environments	0.048	0.253	66.6
3.2.2.21	DNA-3-methyladenine glycosylase II	-		0.029	0.254	72.8
1.1.1.132	GDP-mannose 6-dehydrogenase	+	Fructose and mannose metabolism; Amino sugar and nucleotide sugar metabolism	0.094	0.254	53.5
3.5.2.2	dihydropyrimidinase	+	Pyrimidine metabolism; beta-Alanine metabolism; Pantothenate and CoA biosynthesis; Drug metabolism - other enzymes; Metabolic pathways	0.013	0.255	79.1
1.15.1.2	superoxide reductase	+		0.256	0.256	*0.0
2.7.4.16	thiamine-phosphate kinase	-	Thiamine metabolism; Metabolic pathways	0.256	0.256	*0.0
2.5.1.6	methionine adenosyltransferase	+	Cysteine and methionine metabolism; Metabolic pathways; Biosynthesis of secondary metabolites	2.34E-04	0.257	90.5
2.8.4.4	[ribosomal protein S12] (aspartate89-C3)-methylthiotransferase	+		1.49E-03	0.257	87.3
2.4.1.7	sucrose phosphorylase	+	Starch and sucrose metabolism	0.042	0.257	68.9
1.6.1.2	NAD(P)+ transhydrogenase (Re/Si-specific)	+	Nicotinate and nicotinamide metabolism; Metabolic pathways	0.065	0.264	63.4
2.4.2.10	orotate phosphoribosyltransferase	-	Pyrimidine metabolism; Drug metabolism - other enzymes; Metabolic pathways	0.140	0.265	43.0
4.6.1.17	cyclic pyranopterin monophosphate synthase	-	**Folate biosynthesis**; Metabolic pathways	1.25E-03	0.266	88.1
2.6.1.42	branched-chain-amino-acid transaminase	+	Cysteine and methionine metabolism; Valine, leucine and isoleucine degradation; Valine, leucine and isoleucine biosynthesis; Pantothenate and CoA biosynthesis; Glucosinolate biosynthesis; Metabolic pathways; Biosynthesis of secondary metabolites; Biosynthesis of antibiotics	1.41E-03	0.266	87.9
3.1.27.6	Enterobacter ribonuclease	-		0.012	0.266	80.3
2.5.1.129	flavin prenyltransferase	+		0.055	0.266	66.5
2.7.1.168	D-glycero-alpha-D-manno-heptose-7-phosphate kinase	+	Lipopolysaccharide biosynthesis	0.147	0.266	41.1
3.5.1.91	N-substituted formamide deformylase	+		0.014	0.267	79.5
1.2.1.3	aldehyde dehydrogenase (NAD+)	+	Glycolysis / Gluconeogenesis; Ascorbate and aldarate metabolism; Fatty acid degradation; Valine, leucine and isoleucine degradation; Lysine degradation; **Arginine and proline metabolism**; Histidine metabolism; Tryptophan metabolism; beta-Alanine metabolism; Glycerolipid metabolism; Pyruvate metabolism; Chloroalkane and chloroalkene degradation; Limonene and pinene degradation; Insect hormone biosynthesis; Metabolic pathways; Biosynthesis of secondary metabolites; Microbial metabolism in diverse environments; Biosynthesis of antibiotics	0.073	0.267	61.6
1.9.3.1	cytochrome-c oxidase	+	Oxidative phosphorylation; Metabolic pathways	0.012	0.268	80.6
1.11.1.15	peroxiredoxin	-	Glutathione metabolism; Metabolic pathways	0.018	0.268	78.2
1.14.12.19	3-phenylpropanoate dioxygenase	+	Phenylalanine metabolism; Microbial metabolism in diverse environments	0.231	0.268	*14.6
5.1.3.13	dTDP-4-dehydrorhamnose 3,5-epimerase	-	Streptomycin biosynthesis; Polyketide sugar unit biosynthesis; Biosynthesis of antibiotics	0.175	0.269	*33.8
3.2.1.179	gellan tetrasaccharide unsaturated glucuronosyl hydrolase	+		0.235	0.270	*13.7
1.5.1.36	flavin reductase (NADH)	+	**Riboflavin metabolism**; Metabolic pathways	0.069	0.271	63.3
1.3.99.2	butyryl-CoA dehydrogenase	-		0.183	0.271	*31.6
2.1.1.193	16S rRNA (uracil1498-N3)-methyltransferase	+		0.018	0.272	78.6
1.2.7.5	aldehyde ferredoxin oxidoreductase	+	Pentose phosphate pathway; Metabolic pathways; Microbial metabolism in diverse environments	0.018	0.274	78.5
3.8.1.3	haloacetate dehalogenase	-	Chlorocyclohexane and chlorobenzene degradation; Chloroalkane and chloroalkene degradation; Metabolic pathways; Microbial metabolism in diverse environments	0.050	0.275	68.8
4.2.1.36	homoaconitate hydratase	+	Lysine biosynthesis; Metabolic pathways; Microbial metabolism in diverse environments; Biosynthesis of antibiotics	0.016	0.276	79.6
3.1.4.16	2',3'-cyclic-nucleotide 2'-phosphodiesterase	-	Purine metabolism; Pyrimidine metabolism	0.101	0.278	56.3
1.1.1.292	1,5-anhydro-D-fructose reductase (1,5-anhydro-D-mannitol-forming)	+		0.056	0.280	68.0
3.5.2.9	5-oxoprolinase (ATP-hydrolysing)	+	Glutathione metabolism	2.24E-03	0.281	87.6
3.2.1.26	beta-fructofuranosidase	-	Galactose metabolism; Starch and sucrose metabolism; Metabolic pathways	0.011	0.281	82.0
2.3.1.202	UDP-4-amino-4,6-dideoxy-N-acetyl-beta-L-altrosamine N-acetyltransferase	+	Amino sugar and nucleotide sugar metabolism	0.027	0.283	76.5
6.1.1.24	glutamate---tRNAGln ligase	+	Aminoacyl-tRNA biosynthesis; Metabolic pathways	0.283	0.283	*0.0
2.7.1.51	L-fuculokinase	+	Fructose and mannose metabolism; Microbial metabolism in diverse environments	0.011	0.285	82.5
4.2.2.9	pectate disaccharide-lyase	+	Pentose and glucuronate interconversions	0.285	0.285	*0.0
2.1.1.191	23S rRNA (cytosine1962-C5)-methyltransferase	-		0.011	0.289	82.5
3.5.2.6	beta-lactamase	-	Penicillin and cephalosporin biosynthesis; Biosynthesis of antibiotics	0.125	0.289	52.3
3.6.3.5	Zn2+-exporting ATPase	+		0.058	0.290	68.8
5.3.1.22	hydroxypyruvate isomerase	+	Glyoxylate and dicarboxylate metabolism; Metabolic pathways	0.103	0.290	57.9
4.1.1.21	phosphoribosylaminoimidazole carboxylase	+	Purine metabolism; Metabolic pathways; Biosynthesis of secondary metabolites	0.099	0.291	59.0
3.2.1.nl		-		1.57E-03	0.293	88.9
3.4.11.7	glutamyl aminopeptidase	+		0.024	0.293	78.4
3.5.1.81	N-acyl-D-amino-acid deacylase	+		0.039	0.294	74.1
1.4.1.11	L-erythro-3,5-diaminohexanoate dehydrogenase	+	Lysine degradation	0.070	0.294	66.5
2.7.10.1	receptor protein-tyrosine kinase	-		0.287	0.294	*2.8
3.6.3.41	heme-transporting ATPase	-		0.294	0.294	*0.0
2.7.4.8	guanylate kinase	-	Purine metabolism; Metabolic pathways	0.085	0.296	63.0
5.4.2.11	phosphoglycerate mutase (2,3-diphosphoglycerate-dependent)	+	Glycolysis / Gluconeogenesis; Glycine, serine and threonine metabolism; Methane metabolism; Metabolic pathways; Biosynthesis of secondary metabolites; Microbial metabolism in diverse environments; Biosynthesis of antibiotics	0.093	0.296	61.3
3.5.3.22	proclavaminate amidinohydrolase	+	Clavulanic acid biosynthesis; Biosynthesis of antibiotics	0.024	0.297	78.8
1.1.1.136	UDP-N-acetylglucosamine 6-dehydrogenase	+	Amino sugar and nucleotide sugar metabolism	0.298	0.298	*0.0
3.5.1.16	acetylornithine deacetylase	+	Arginine biosynthesis; Metabolic pathways; Biosynthesis of secondary metabolites; Biosynthesis of antibiotics	0.042	0.299	74.0
5.1.3.11	cellobiose epimerase	-		0.045	0.299	73.3
3.1.1.27	4-pyridoxolactonase	+	Vitamin B6 metabolism; Microbial metabolism in diverse environments	0.152	0.299	47.4
3.1.1.17	gluconolactonase	+	Pentose phosphate pathway; Ascorbate and aldarate metabolism; Caprolactam degradation; Metabolic pathways; Biosynthesis of secondary metabolites; Microbial metabolism in diverse environments; Biosynthesis of antibiotics	0.027	0.300	78.1
2.6.1.17	succinyldiaminopimelate transaminase	+	Lysine biosynthesis; Metabolic pathways; Microbial metabolism in diverse environments	0.075	0.300	66.1
4.3.3.6	pyridoxal 5'-phosphate synthase (glutamine hydrolysing)	-	Vitamin B6 metabolism	0.258	0.300	*15.9
4.3.1.17	L-serine ammonia-lyase	-	Glycine, serine and threonine metabolism; Cysteine and methionine metabolism; Metabolic pathways; Biosynthesis of secondary metabolites; Biosynthesis of antibiotics	7.71E-03	0.302	85.0
2.1.1.14	5-methyltetrahydropteroyltriglutamate---homocysteine S-methyltransferase	+	Cysteine and methionine metabolism; Selenocompound metabolism; Metabolic pathways; Biosynthesis of secondary metabolites	6.54E-03	0.303	85.7
3.1.3.82	D-glycero-beta-D-manno-heptose 1,7-bisphosphate 7-phosphatase	-	Lipopolysaccharide biosynthesis; Metabolic pathways	0.082	0.303	64.9
3.6.3.20	glycerol-3-phosphate-transporting ATPase	-		0.041	0.304	74.7
4.2.1.19	imidazoleglycerol-phosphate dehydratase	-	Histidine metabolism; Metabolic pathways; Biosynthesis of secondary metabolites	0.052	0.305	72.0
3.5.1.46	6-aminohexanoate-oligomer exohydrolase	+	Caprolactam degradation; Microbial metabolism in diverse environments	0.200	0.305	*36.0
2.4.1.25	4-alpha-glucanotransferase	+	Starch and sucrose metabolism; Metabolic pathways	3.65E-03	0.308	87.7
2.7.8.13	phospho-N-acetylmuramoyl-pentapeptide-transferase	+	Peptidoglycan biosynthesis; Metabolic pathways	0.042	0.309	75.1
1.2.3.3	pyruvate oxidase	+	Pyruvate metabolism; Metabolic pathways	0.026	0.310	79.2
2.3.1.8	phosphate acetyltransferase	-	Taurine and hypotaurine metabolism; Pyruvate metabolism; Propanoate metabolism; Methane metabolism; Carbon fixation pathways in prokaryotes; Metabolic pathways; Microbial metabolism in diverse environments	0.068	0.311	69.3
3.2.1.91	cellulose 1,4-beta-cellobiosidase (non-reducing end)	-	Starch and sucrose metabolism; Metabolic pathways	0.202	0.313	*37.5
6.3.2.4	D-alanine---D-alanine ligase	+	D-Alanine metabolism; Peptidoglycan biosynthesis; Metabolic pathways	9.36E-03	0.315	85.1
1.3.1.12	prephenate dehydrogenase	+	Phenylalanine, tyrosine and tryptophan biosynthesis; Novobiocin biosynthesis; Metabolic pathways; Biosynthesis of secondary metabolites; Biosynthesis of antibiotics	9.01E-03	0.316	85.3
4.1.1.96	carboxynorspermidine decarboxylase	-	**Arginine and proline metabolism**; Metabolic pathways	0.188	0.316	41.9
3.5.1.108	UDP-3-O-acyl-N-acetylglucosamine deacetylase	-	Lipopolysaccharide biosynthesis; Metabolic pathways	0.215	0.317	*34.8
6.3.5.9	hydrogenobyrinic acid a,c-diamide synthase (glutamine-hydrolysing)	+	Porphyrin and chlorophyll metabolism; Metabolic pathways	0.234	0.317	*29.5
3.5.99.10	2-iminobutanoate/2-iminopropanoate deaminase	-		0.033	0.318	78.0
5.3.1.25	L-fucose isomerase	-	Fructose and mannose metabolism; Microbial metabolism in diverse environments	0.189	0.318	42.1
2.3.3.1	citrate (Si)-synthase	+	Citrate cycle (TCA cycle); Glyoxylate and dicarboxylate metabolism; Metabolic pathways; Biosynthesis of secondary metabolites; Microbial metabolism in diverse environments; Biosynthesis of antibiotics	0.018	0.320	82.4
3.1.1.31	6-phosphogluconolactonase	-	Pentose phosphate pathway; Metabolic pathways; Biosynthesis of secondary metabolites; Microbial metabolism in diverse environments; Biosynthesis of antibiotics	0.320	0.320	*0.0
1.3.1.76	precorrin-2 dehydrogenase	-	Porphyrin and chlorophyll metabolism; Metabolic pathways; Biosynthesis of secondary metabolites; Microbial metabolism in diverse environments	5.51E-03	0.323	87.3
2.4.2.17	ATP phosphoribosyltransferase	-	Histidine metabolism; Metabolic pathways; Biosynthesis of secondary metabolites	0.149	0.323	53.1
3.2.2.31	adenine glycosylase	+		0.205	0.323	*39.2
2.3.1.18	galactoside O-acetyltransferase	-		0.050	0.324	74.8
3.6.3.27	phosphate-transporting ATPase	+		0.085	0.324	67.2
1.1.1.17	mannitol-1-phosphate 5-dehydrogenase	+	Fructose and mannose metabolism	0.084	0.326	67.7
3.5.1.78	glutathionylspermidine amidase	-	Glutathione metabolism; Metabolic pathways	0.096	0.326	65.3
1.1.1.53	3alpha(or 20beta)-hydroxysteroid dehydrogenase	+	Steroid hormone biosynthesis; Metabolic pathways	0.100	0.326	64.4
2.5.1.101	N,N'-diacetyllegionaminate synthase	+	Amino sugar and nucleotide sugar metabolism	0.156	0.328	52.5
5.4.2.2	phosphoglucomutase (alpha-D-glucose-1,6-bisphosphate-dependent)	-	Glycolysis / Gluconeogenesis; Pentose phosphate pathway; Galactose metabolism; Purine metabolism; Starch and sucrose metabolism; Amino sugar and nucleotide sugar metabolism; Streptomycin biosynthesis; Metabolic pathways; Biosynthesis of secondary metabolites; Microbial metabolism in diverse environments; Biosynthesis of antibiotics	9.48E-03	0.330	85.9
1.3.99.5	3-oxo-5alpha-steroid 4-dehydrogenase (acceptor)	+	Steroid degradation; Microbial metabolism in diverse environments	0.013	0.330	84.7

2.1.1.163	demethylmenaquinone methyltransferase	-	Ubiquinone and other terpenoid-quinone biosynthesis; Metabolic pathways; Biosynthesis of secondary metabolites	0.049	0.330	75.5
1.1.1.399	2-oxoglutarate reductase	-		0.025	0.331	81.1
2.1.3.1	methylmalonyl-CoA carboxytransferase	+	Propanoate metabolism	0.052	0.331	74.9
6.1.1.7	alanine---tRNA ligase	+	Aminoacyl-tRNA biosynthesis	2.14E-03	0.332	90.0
3.4.14.11	Xaa-Pro dipeptidyl-peptidase	+		0.019	0.332	83.0
3.2.1.24	alpha-mannosidase	+	Other glycan degradation	0.114	0.334	62.7
3.2.1.183	UDP-N-acetylglucosamine 2-epimerase (hydrolysing)	-	Amino sugar and nucleotide sugar metabolism	0.176	0.334	49.0
4.2.3.5	chorismate synthase	-	Phenylalanine, tyrosine and tryptophan biosynthesis; Metabolic pathways; Biosynthesis of secondary metabolites; Biosynthesis of antibiotics	0.043	0.335	77.4
3.2.1.94	glucan 1,6-alpha-isomaltosidase	+		0.022	0.336	82.3
3.5.1.49	formamidase	+	Cyanoamino acid metabolism; Glyoxylate and dicarboxylate metabolism; Nitrogen metabolism	0.074	0.336	71.0
1.14.13.81	magnesium-protoporphyrin IX monomethyl ester (oxidative) cyclase	+	Porphyrin and chlorophyll metabolism; Metabolic pathways; Biosynthesis of secondary metabolites	0.061	0.338	73.7
4.3.1.19	threonine ammonia-lyase	+	Glycine, serine and threonine metabolism; Valine, leucine and isoleucine biosynthesis; Metabolic pathways; Biosynthesis of secondary metabolites; Biosynthesis of antibiotics	0.014	0.339	84.8
1.1.1.94	glycerol-3-phosphate dehydrogenase [NAD(P)+]	+	Glycerophospholipid metabolism; Biosynthesis of secondary metabolites	0.036	0.339	79.1
2.3.1.46	homoserine O-succinyltransferase	-	Cysteine and methionine metabolism; Sulfur metabolism; Metabolic pathways; Biosynthesis of secondary metabolites	0.046	0.339	77.1
6.3.5.1	NAD+ synthase (glutamine-hydrolysing)	+	Nicotinate and nicotinamide metabolism; Metabolic pathways	0.030	0.341	80.9
2.5.1.7	UDP-N-acetylglucosamine 1-carboxyvinyltransferase	+	Amino sugar and nucleotide sugar metabolism; Peptidoglycan biosynthesis; Metabolic pathways	0.015	0.342	84.8
1.11.1.10	chloride peroxidase	-		0.028	0.342	81.4
4.2.2.nl		-		0.052	0.346	76.5
5.3.1.5	xylose isomerase	-	Pentose and glucuronate interconversions; Fructose and mannose metabolism; Metabolic pathways	0.137	0.348	60.0
1.1.1.336	UDP-N-acetyl-D-mannosamine dehydrogenase	+	Amino sugar and nucleotide sugar metabolism	0.246	0.348	*34.7
2.7.7.87	L-threonylcarbamoyladenylate synthase	+		0.012	0.350	86.1
4.1.1.18	lysine decarboxylase	-	Lysine degradation; Tropane, piperidine and pyridine alkaloid biosynthesis; Metabolic pathways; Biosynthesis of secondary metabolites	0.112	0.351	65.5
4.2.2.2	pectate lyase	-	Pentose and glucuronate interconversions	0.034	0.353	80.8
1.10.3.10	ubiquinol oxidase (H+-transporting)	+		0.356	0.356	*0.0
3.5.1.23	ceramidase	-	Sphingolipid metabolism; Metabolic pathways	0.080	0.357	72.4
2.7.7.1	nicotinamide-nucleotide adenylyltransferase	+	Nicotinate and nicotinamide metabolism; Metabolic pathways	0.225	0.358	42.6
3.1.2.14	oleoyl-[acyl-carrier-protein] hydrolase	-	Fatty acid biosynthesis; Metabolic pathways	0.030	0.359	82.1
4.6.1.12	2-C-methyl-D-erythritol 2,4-cyclodiphosphate synthase	-	Terpenoid backbone biosynthesis; Metabolic pathways; Biosynthesis of secondary metabolites; Biosynthesis of antibiotics	0.227	0.359	42.3
1.1.1.25	shikimate dehydrogenase (NADP+)	-	Phenylalanine, tyrosine and tryptophan biosynthesis; Metabolic pathways; Biosynthesis of secondary metabolites; Biosynthesis of antibiotics	0.060	0.362	76.6
4.2.2.17	inulin fructotransferase (DFA-I-forming)	+		0.106	0.362	68.2
2.3.3.10	hydroxymethylglutaryl-CoA synthase	+	Synthesis and degradation of ketone bodies; Valine, leucine and isoleucine degradation; Butanoate metabolism; Terpenoid backbone biosynthesis; Metabolic pathways; Biosynthesis of secondary metabolites; Biosynthesis of antibiotics	0.110	0.362	67.5
3.8.1.8	atrazine chlorohydrolase	+	Atrazine degradation; Metabolic pathways; Microbial metabolism in diverse environments	4.17E-03	0.364	90.0
2.1.1.217	tRNA (adenine22-N1)-methyltransferase	-		0.067	0.365	75.5
2.7.1.53	L-xylulokinase	+	Pentose and glucuronate interconversions; Ascorbate and aldarate metabolism	0.185	0.365	53.3
6.3.2.49	L-alanine---L-anticapsin ligase	+	Biosynthesis of antibiotics	0.160	0.367	58.7
6.2.1.20	long-chain-fatty-acid---[acyl-carrier-protein] ligase	-	Fatty acid degradation	0.044	0.368	79.9
4.99.1.12	pyridinium-3,5-bisthiocarboxylic acid mononucleotide nickel chelatase	+		0.062	0.370	77.0
2.4.1.52	poly(glycerol-phosphate) alpha-glucosyltransferase	-		0.137	0.370	63.7
2.9.1.1	L-seryl-tRNASec selenium transferase	+	Selenocompound metabolism; Aminoacyl-tRNA biosynthesis	0.009	0.372	88.4
4.2.2.23	rhamnogalacturonan endolyase	+		0.040	0.374	81.4
2.4.99.17	S-adenosylmethionine:tRNA ribosyltransferase-isomerase	-		0.188	0.374	54.4
1.9.6.1	nitrate reductase (cytochrome)	+	Nitrogen metabolism	0.262	0.375	*37.5
5.4.2.12	phosphoglycerate mutase (2,3-diphosphoglycerate-independent)	+	Glycolysis / Gluconeogenesis; Glycine, serine and threonine metabolism; Methane metabolism; Metabolic pathways; Biosynthesis of secondary metabolites; Microbial metabolism in diverse environments; Biosynthesis of antibiotics	0.011	0.377	88.0
1.1.1.35	3-hydroxyacyl-CoA dehydrogenase	-	Fatty acid elongation; Fatty acid degradation; Primary bile acid biosynthesis; Valine, leucine and isoleucine degradation; Geraniol degradation; Lysine degradation; Benzoate degradation; Tryptophan metabolism; Toluene degradation; Butanoate metabolism; Carbon fixation pathways in prokaryotes; Caprolactam degradation; Metabolic pathways; Biosynthesis of secondary metabolites; Microbial metabolism in diverse environments; Biosynthesis of antibiotics	0.059	0.377	78.1
4.2.1.109	methylthioribulose 1-phosphate dehydratase	+	Cysteine and methionine metabolism; Metabolic pathways	0.041	0.378	81.4
2.7.1.100	S-methyl-5-thioribose kinase	+	Cysteine and methionine metabolism; Metabolic pathways	0.019	0.379	85.9
3.1.5.1	dGTPase	+	Purine metabolism	0.032	0.380	83.1
4.2.1.163	2-oxo-hept-4-ene-1,7-dioate hydratase	+		0.096	0.380	72.1
3.2.1.10	oligo-1,6-glucosidase	-	Galactose metabolism; Starch and sucrose metabolism; Metabolic pathways	0.119	0.380	68.2
3.2.1.14	chitinase	+	Amino sugar and nucleotide sugar metabolism; Metabolic pathways	0.362	0.380	*7.1
2.3.1.241	Kdo2-lipid IVA acyltransferase	-	Lipopolysaccharide biosynthesis; Metabolic pathways	0.074	0.381	76.0
1.13.11.24	quercetin 2,3-dioxygenase	-		0.382	0.382	*0.0
2.1.1.130	precorrin-2 C20-methyltransferase	-	Porphyrin and chlorophyll metabolism; Metabolic pathways	0.382	0.382	*0.0
7.1.2.2	H+-transporting two-sector ATPase	-		0.017	0.383	86.7
4.4.1.11	methionine gamma-lyase	-	Cysteine and methionine metabolism; Selenocompound metabolism	0.019	0.384	86.3
2.7.8.5	CDP-diacylglycerol---glycerol-3-phosphate 1-phosphatidyltransferase	-	Glycerophospholipid metabolism; Metabolic pathways	0.091	0.384	73.5
2.1.1.200	tRNA (cytidine32/uridine32-2'-O)-methyltransferase	-		0.103	0.386	71.7
2.4.1.291	N-acetylgalactosamine-N,N'-diacetylbacillosaminyl-diphospho-undecaprenol 4-alpha-N-acetylgalactosaminyltransferase	+		0.137	0.386	66.1
1.8.1.4	dihydrolipoyl dehydrogenase	+	Glycolysis / Gluconeogenesis; Citrate cycle (TCA cycle); Glycine, serine and threonine metabolism; Valine, leucine and isoleucine degradation; Pyruvate metabolism; Propanoate metabolism; Metabolic pathways; Biosynthesis of secondary metabolites; Microbial metabolism in diverse environments; Biosynthesis of antibiotics	0.177	0.386	58.7
2.5.1.84	all-trans-nonaprenyl diphosphate synthase [geranyl-diphosphate specific]	+	Terpenoid backbone biosynthesis; Biosynthesis of secondary metabolites	0.388	0.388	*0.0
2.7.9.2	pyruvate, water dikinase	+	Pyruvate metabolism; Methane metabolism; Carbon fixation pathways in prokaryotes; Metabolic pathways; Microbial metabolism in diverse environments	0.042	0.389	82.1
4.2.2.20	chondroitin-sulfate-ABC endolyase	+		0.029	0.390	84.6
6.1.1.13	D-alanine---poly(phosphoribitol) ligase	-	D-Alanine metabolism	0.195	0.390	55.9
2.4.1.329	sucrose 6F-phosphate phosphorylase	-		0.176	0.391	59.8
6.3.5.6	asparaginyl-tRNA synthase (glutamine-hydrolysing)	+	Aminoacyl-tRNA biosynthesis	0.213	0.391	52.5
4.1.1.37	uroporphyrinogen decarboxylase	-	Porphyrin and chlorophyll metabolism; Metabolic pathways; Biosynthesis of secondary metabolites	0.073	0.392	77.3
3.6.3.30	Fe3+-transporting ATPase	-		0.027	0.395	85.1
4.1.2.21	2-dehydro-3-deoxy-6-phosphogalactonate aldolase	+	Galactose metabolism; Metabolic pathways	0.067	0.395	78.4
1.13.12.16	nitronate monooxygenase	-	Nitrogen metabolism	0.395	0.395	*0.0
2.5.1.18	glutathione transferase	-	Glutathione metabolism; Metabolism of xenobiotics by cytochrome P450; Drug metabolism - cytochrome P450; Drug metabolism - other enzymes	0.093	0.396	74.5
6.3.2.45	UDP-N-acetylmuramate---L-alanyl-gamma-D-glutamyl-meso-2,6-diaminoheptanedioate ligase	-		0.216	0.397	53.2
1.7.2.2	nitrite reductase (cytochrome; ammonia-forming)	-	Nitrogen metabolism; Microbial metabolism in diverse environments	0.262	0.398	43.2
1.17.99.6	epoxyqueuosine reductase	-		0.046	0.400	82.1
1.1.1.81	hydroxypyruvate reductase	-	Glycine, serine and threonine metabolism; Glyoxylate and dicarboxylate metabolism; Metabolic pathways; Biosynthesis of secondary metabolites	0.177	0.401	61.3
4.1.1.50	adenosylmethionine decarboxylase	+	Cysteine and methionine metabolism; **Arginine and proline metabolism**; Metabolic pathways	0.179	0.402	61.1
2.7.7.21	tRNA cytidylyltransferase	-		0.081	0.403	77.1
2.7.7.25	tRNA adenylyltransferase	-		0.083	0.406	77.1
2.1.1.176	16S rRNA (cytosine967-C5)-methyltransferase	+		0.012	0.408	89.1
1.1.1.40	malate dehydrogenase (oxaloacetate-decarboxylating) (NADP+)	-	Pyruvate metabolism; Carbon fixation in photosynthetic organisms; Metabolic pathways; Microbial metabolism in diverse environments	2.79E-03	0.409	92.4
1.1.1.1	alcohol dehydrogenase	+	Glycolysis / Gluconeogenesis; Fatty acid degradation; Glycine, serine and threonine metabolism; Tyrosine metabolism; alpha-Linolenic acid metabolism; Chloroalkane and chloroalkene degradation; Naphthalene degradation; Retinol metabolism; Metabolism of xenobiotics by cytochrome P450; Drug metabolism - cytochrome P450; Metabolic pathways; Biosynthesis of secondary metabolites; Microbial metabolism in diverse environments; Biosynthesis of antibiotics	0.051	0.409	82.1
1.7.1.6	azobenzene reductase	+		0.052	0.410	82.0
4.2.1.1	carbonic anhydrase	-	Nitrogen metabolism	0.214	0.412	56.4
1.5.3.1	sarcosine oxidase (formaldehyde-forming)	+	Glycine, serine and threonine metabolism; Metabolic pathways	0.310	0.412	*34.9
2.3.1.57	diamine N-acetyltransferase	+	**Arginine and proline metabolism**; Metabolic pathways	0.073	0.413	79.2
6.3.1.11	glutamate---putrescine ligase	+	**Arginine and proline metabolism**; Metabolic pathways	0.344	0.414	*25.3
2.7.1.156	adenosylcobinamide kinase	-	Porphyrin and chlorophyll metabolism; Metabolic pathways	6.37E-03	0.418	91.2
6.2.1.30	phenylacetate---CoA ligase	-	Phenylalanine metabolism; Microbial metabolism in diverse environments	0.133	0.418	71.0
4.1.2.2	ketotetrose-phosphate aldolase	-		0.051	0.419	82.9
5.3.3.10	5-carboxymethyl-2-hydroxymuconate Delta-isomerase	+	Tyrosine metabolism; Microbial metabolism in diverse environments	0.083	0.421	78.4
3.5.1.59	N-carbamoylsarcosine amidase	+	**Arginine and proline metabolism**; Metabolic pathways	0.061	0.422	81.6
2.7.1.71	shikimate kinase	-	Phenylalanine, tyrosine and tryptophan biosynthesis; Metabolic pathways; Biosynthesis of secondary metabolites; Biosynthesis of antibiotics	0.037	0.424	85.4
2.1.1.242	16S rRNA (guanine1516-N2)-methyltransferase	-		0.042	0.425	84.6
2.4.1.345	phosphatidyl-myo-inositol alpha-mannosyltransferase	-		0.066	0.427	81.4
1.14.14.9	4-hydroxyphenylacetate 3-monooxygenase	+	Tyrosine metabolism; Microbial metabolism in diverse environments	0.161	0.427	67.9
1.4.3.21	primary-amine oxidase	+	Glycine, serine and threonine metabolism; Tyrosine metabolism; Phenylalanine metabolism; beta-Alanine metabolism; Isoquinoline alkaloid biosynthesis; Tropane, piperidine and pyridine alkaloid biosynthesis; Metabolic pathways; Biosynthesis of secondary metabolites	0.214	0.430	59.7
4.1.2.52	4-hydroxy-2-oxoheptanedioate aldolase	+	Tyrosine metabolism; Microbial metabolism in diverse environments	0.176	0.431	66.1
3.5.1.18	succinyl-diaminopimelate desuccinylase	-	Lysine biosynthesis; Metabolic pathways; Microbial metabolism in diverse environments	0.147	0.432	70.7
3.6.3.23	oligopeptide-transporting ATPase	-		0.206	0.435	61.8
6.1.2.1	D-alanine---(R)-lactate ligase	+		0.435	0.435	*0.0
3.1.3.10	glucose-1-phosphatase	-	Glycolysis / Gluconeogenesis; Microbial metabolism in diverse environments	0.084	0.436	79.6
2.1.1.297	peptide chain release factor N5-glutamine methyltransferase	+		0.166	0.438	68.8
2.3.1.40	acyl-[acyl-carrier-protein]---phospholipid O-acyltransferase	-	Glycerophospholipid metabolism	0.094	0.439	78.6
3.5.1.82	N-acyl-D-glutamate deacylase	+		0.110	0.439	76.5
3.2.1.64	2,6-beta-fructan 6-levanbiohydrolase	-	Starch and sucrose metabolism	0.355	0.439	*30.2
4.1.1.31	phosphoenolpyruvate carboxylase	+	Pyruvate metabolism; Methane metabolism; Carbon fixation in photosynthetic organisms; Carbon fixation pathways in prokaryotes; Metabolic pathways; Microbial metabolism in diverse environments	0.171	0.440	68.3
2.1.1.298	ribosomal protein L3 N5-glutamine methyltransferase	-		0.179	0.440	67.1

3.4.21.83	oligopeptidase B	+		0.230	0.440	58.7
1.3.1.25	1,6-dihydroxycyclohexa-2,4-diene-1-carboxylate dehydrogenase	+	Benzoate degradation; Fluorobenzoate degradation; Metabolic pathways; Microbial metabolism in diverse environments	0.103	0.441	77.7
3.1.3.3	phosphoserine phosphatase	+	Glycine, serine and threonine metabolism; Methane metabolism; Metabolic pathways; Microbial metabolism in diverse environments; Biosynthesis of antibiotics	0.030	0.442	87.4
3.6.3.25	sulfate-transporting ATPase	+	Sulfur metabolism	0.062	0.443	83.1
3.2.2.10	pyrimidine-5'-nucleotide nucleosidase	-	Pyrimidine metabolism	0.186	0.443	66.4
3.1.3.74	pyridoxal phosphatase	+	Vitamin B6 metabolism; Metabolic pathways	0.320	0.443	40.5
3.6.1.55	8-oxo-dGTP diphosphatase	-		0.063	0.444	83.0
1.2.5.1	pyruvate dehydrogenase (quinone)	+	Pyruvate metabolism	0.124	0.444	75.3
2.7.1.12	gluconokinase	+	Pentose phosphate pathway; Metabolic pathways; Biosynthesis of secondary metabolites; Microbial metabolism in diverse environments; Biosynthesis of antibiotics	0.149	0.445	72.0
2.7.4.25	(d)CMP kinase	+	Pyrimidine metabolism; Metabolic pathways	0.042	0.446	85.9
3.5.4.1	cytosine deaminase	+	Pyrimidine metabolism; **Arginine and proline metabolism**; Metabolic pathways	0.101	0.446	78.5
2.3.1.243	acyl-Kdo2-lipid IVA acyltransferase	-	Lipopolysaccharide biosynthesis; Metabolic pathways	0.127	0.447	75.1
2.7.7.62	adenosylcobinamide-phosphate guanylyltransferase	-	Porphyrin and chlorophyll metabolism; Metabolic pathways	0.049	0.449	85.2
3.2.1.1	alpha-amylase	-	Starch and sucrose metabolism; Metabolic pathways	0.063	0.452	83.6
2.1.1.266	23S rRNA (adenine2030-N6)-methyltransferase	-		0.180	0.452	68.5
3.5.1.54	allophanate hydrolase	+	Arginine biosynthesis; Atrazine degradation; Metabolic pathways; Microbial metabolism in diverse environments	0.194	0.452	66.5
2.6.1.2	alanine transaminase	+	Arginine biosynthesis; Alanine, aspartate and glutamate metabolism; Carbon fixation in photosynthetic organisms; Metabolic pathways; Microbial metabolism in diverse environments	0.141	0.456	74.4
3.4.11.2	membrane alanyl aminopeptidase	-	Glutathione metabolism; Metabolic pathways	0.141	0.458	74.6
1.1.1.266	dTDP-4-dehydro-6-deoxyglucose reductase	+	Polyketide sugar unit biosynthesis; Biosynthesis of antibiotics	0.458	0.458	*0.0
2.3.1.16	acetyl-CoA C-acyltransferase	+	Fatty acid elongation; Fatty acid degradation; Valine, leucine and isoleucine degradation; Geraniol degradation; Benzoate degradation; alpha-Linolenic acid metabolism; Ethylbenzene degradation; Biosynthesis of unsaturated fatty acids; Metabolic pathways; Biosynthesis of secondary metabolites; Microbial metabolism in diverse environments; Biosynthesis of antibiotics	0.194	0.460	67.6
1.1.1.44	phosphogluconate dehydrogenase (NADP+-dependent, decarboxylating)	+	Pentose phosphate pathway; Glutathione metabolism; Metabolic pathways; Biosynthesis of secondary metabolites; Biosynthesis of antibiotics	0.281	0.460	53.0
1.17.98.1	bile-acid 7alpha-dehydroxylase	-		0.176	0.461	70.4
2.1.1.113	site-specific DNA-methyltransferase (cytosine-N4-specific)	+		0.066	0.463	84.0
3.5.2.3	dihydroorotase	-	Pyrimidine metabolism; Metabolic pathways	0.167	0.466	72.2
3.5.1.94	gamma-glutamyl-gamma-aminobutyrate hydrolase	+	**Arginine and proline metabolism**; Metabolic pathways	0.067	0.467	84.2
1.1.1.67	mannitol 2-dehydrogenase	-	Fructose and mannose metabolism	0.176	0.467	71.2
2.1.1.131	precorrin-3B C17-methyltransferase	-	Porphyrin and chlorophyll metabolism; Metabolic pathways	0.082	0.471	82.7
1.14.13.239	carnitine monooxygenase	+		0.250	0.473	61.0
4.2.2.6	oligogalacturonide lyase	+	Pentose and glucuronate interconversions	0.093	0.476	82.0
3.2.1.49	alpha-N-acetylgalactosaminidase	+	Glycosphingolipid biosynthesis - globo and isoglobo series	0.217	0.476	66.6
6.1.1.11	serine---tRNA ligase	-	Aminoacyl-tRNA biosynthesis	0.316	0.476	49.4
4.3.99.4	choline trimethylamine-lyase	+		0.144	0.478	76.4
1.21.4.2	glycine reductase	+		0.079	0.479	83.8
2.1.3.15	acetyl-CoA carboxytransferase	+		0.172	0.479	73.1
3.6.3.15	Na+-transporting two-sector ATPase	-		0.304	0.479	52.5
4.1.1.81	threonine-phosphate decarboxylase	-	Porphyrin and chlorophyll metabolism	0.075	0.481	84.3
1.1.1.3	homoserine dehydrogenase	+	Glycine, serine and threonine metabolism; Cysteine and methionine metabolism; Lysine biosynthesis; Metabolic pathways; Biosynthesis of secondary metabolites; Microbial metabolism in diverse environments; Biosynthesis of antibiotics	0.089	0.481	82.9
2.7.8.12	teichoic acid poly(glycerol phosphate) polymerase	-		0.104	0.481	81.2
2.1.1.37	DNA (cytosine-5-)-methyltransferase	+	Cysteine and methionine metabolism; Metabolic pathways	0.413	0.481	*26.1
2.5.1.19	3-phosphoshikimate 1-carboxyvinyltransferase	-	Phenylalanine, tyrosine and tryptophan biosynthesis; Metabolic pathways; Biosynthesis of secondary metabolites; Biosynthesis of antibiotics	0.484	0.484	*0.0
5.4.2.9	phosphoenolpyruvate mutase	+	Phosphonate and phosphinate metabolism; Metabolic pathways; Microbial metabolism in diverse environments; Biosynthesis of antibiotics	0.286	0.485	57.1
2.1.1.144	trans-aconitate 2-methyltransferase	+		0.113	0.486	80.7
1.1.1.355	2'-dehydrokanamycin reductase	+	Neomycin, kanamycin and gentamicin biosynthesis; Biosynthesis of antibiotics	0.246	0.486	63.9
4.2.1.44	myo-inosose-2 dehydratase	+	Inositol phosphate metabolism; Metabolic pathways; Microbial metabolism in diverse environments	0.144	0.487	77.4
4.3.2.10	imidazole glycerol-phosphate synthase	-		0.251	0.488	63.6
5.1.2.2	mandelate racemase	+	Aminobenzoate degradation; Microbial metabolism in diverse environments	0.092	0.490	83.2
6.3.2.9	UDP-N-acetylmuramoyl-L-alanine---D-glutamate ligase	-	D-Glutamine and D-glutamate metabolism; Peptidoglycan biosynthesis; Metabolic pathways	0.125	0.490	79.8
1.5.1.2	pyrroline-5-carboxylate reductase	-	**Arginine and proline metabolism**; Metabolic pathways; Biosynthesis of secondary metabolites; Biosynthesis of antibiotics	0.206	0.492	70.5
3.4.22.37	gingipain R	+		0.493	0.493	*0.0
1.1.1.41	isocitrate dehydrogenase (NAD+)	-	Citrate cycle (TCA cycle); Metabolic pathways; Biosynthesis of secondary metabolites; Microbial metabolism in diverse environments; Biosynthesis of antibiotics	0.416	0.494	*29.1
1.2.1.79	succinate-semialdehyde dehydrogenase (NADP+)	+	Alanine, aspartate and glutamate metabolism; Butanoate metabolism; Nicotinate and nicotinamide metabolism; Metabolic pathways	0.157	0.497	77.0
2.7.4.29	Kdo2-lipid A phosphotransferase	-		0.201	0.497	71.8
1.1.99.3	gluconate 2-dehydrogenase (acceptor)	+	Pentose phosphate pathway; Metabolic pathways; Microbial metabolism in diverse environments	0.321	0.497	53.0
1.1.1.281	GDP-4-dehydro-6-deoxy-D-mannose reductase	+	Fructose and mannose metabolism; Amino sugar and nucleotide sugar metabolism	0.225	0.498	68.8
4.2.1.149	crotonobetainyl-CoA hydratase	-		0.361	0.499	45.3
2.5.1.145	phosphatidylglycerol---prolipoprotein diacylglyceryl transferase	+		0.170	0.503	76.2
2.4.1.4	amylosucrase	-	Starch and sucrose metabolism	0.078	0.504	85.6
3.1.1.10	tropinesterase	+	Tropane, piperidine and pyridine alkaloid biosynthesis; Biosynthesis of secondary metabolites	0.093	0.504	84.2
1.3.8.7	medium-chain acyl-CoA dehydrogenase	-	Fatty acid degradation; Valine, leucine and isoleucine degradation; beta-Alanine metabolism; Propanoate metabolism; Metabolic pathways; Biosynthesis of secondary metabolites; Biosynthesis of antibiotics	0.229	0.504	69.2
4.1.2.4	deoxyribose-phosphate aldolase	-	Pentose phosphate pathway	0.383	0.504	41.2
3.3.2.1	isochorismatase	+	Biosynthesis of siderophore group nonribosomal peptides; Biosynthesis of secondary metabolites; Biosynthesis of antibiotics	0.313	0.505	56.4
1.3.1.44	trans-2-enoyl-CoA reductase (NAD+)	-	Butanoate metabolism; Metabolic pathways; Microbial metabolism in diverse environments	0.118	0.506	81.9
1.2.1.88	L-glutamate gamma-semialdehyde dehydrogenase	+	Alanine, aspartate and glutamate metabolism; **Arginine and proline metabolism**; Metabolic pathways	0.214	0.506	71.4
3.5.1.87	N-carbamoyl-L-amino-acid hydrolase	+		0.160	0.507	77.6
2.1.1.186	23S rRNA (cytidine2498-2'-O)-methyltransferase	-		0.194	0.507	73.9
1.12.99.6	hydrogenase (acceptor)	+	Nitrotoluene degradation; Microbial metabolism in diverse environments	0.297	0.507	59.5
4.2.1.80	2-oxopent-4-enoate hydratase	+	Phenylalanine metabolism; Benzoate degradation; Dioxin degradation; Xylene degradation; Metabolic pathways; Microbial metabolism in diverse environments	0.204	0.508	72.9
2.1.1.79	cyclopropane-fatty-acyl-phospholipid synthase	+		0.086	0.509	85.2
1.3.8.8	long-chain acyl-CoA dehydrogenase	-	Fatty acid degradation; Metabolic pathways	0.233	0.509	69.4
2.3.1.169	CO-methylating acetyl-CoA synthase	-	Carbon fixation pathways in prokaryotes; Microbial metabolism in diverse environments	0.125	0.510	81.6
1.3.3.4	protoporphyrinogen oxidase	+	Porphyrin and chlorophyll metabolism; Metabolic pathways; Biosynthesis of secondary metabolites	0.180	0.510	75.8
1.4.1.21	aspartate dehydrogenase	+	Nicotinate and nicotinamide metabolism; Metabolic pathways	0.281	0.510	62.6
3.2.1.52	beta-N-acetylhexosaminidase	-	Other glycan degradation; Various types of N-glycan biosynthesis; Amino sugar and nucleotide sugar metabolism; Glycosaminoglycan degradation; Glycosphingolipid biosynthesis - globo and isoglobo series; Glycosphingolipid biosynthesis - ganglio series; Metabolic pathways	0.510	0.510	*0.0
3.6.1.13	ADP-ribose diphosphatase	-	Purine metabolism	0.092	0.511	84.7
2.5.1.54	3-deoxy-7-phosphoheptulonate synthase	+	Phenylalanine, tyrosine and tryptophan biosynthesis; Metabolic pathways; Biosynthesis of secondary metabolites; Biosynthesis of antibiotics	0.023	0.513	91.7
4.2.1.85	dimethylmaleate hydratase	-	Nicotinate and nicotinamide metabolism; Microbial metabolism in diverse environments	0.169	0.514	77.5
2.4.2.2	pyrimidine-nucleoside phosphorylase	-	Pyrimidine metabolism; Metabolic pathways	0.088	0.516	85.5
3.2.2.24	ADP-ribosyl-[dinitrogen reductase] hydrolase	+		0.122	0.516	82.3
3.1.12.1	5' to 3' exodeoxyribonuclease (nucleoside 3'-phosphate-forming)	+		0.355	0.516	50.8
3.6.1.19	nucleoside-triphosphate diphosphatase	+		0.389	0.517	43.4
3.2.2.15	DNA-deoxyinosine glycosylase	-		0.287	0.518	63.2
2.7.4.14	UMP/CMP kinase	-	Pyrimidine metabolism; Drug metabolism - other enzymes; Metabolic pathways	0.287	0.518	63.1
4.3.2.2	adenylosuccinate lyase	+	Purine metabolism; Alanine, aspartate and glutamate metabolism; Metabolic pathways; Biosynthesis of secondary metabolites; Biosynthesis of antibiotics	0.229	0.520	71.4
2.6.1.16	glutamine---fructose-6-phosphate transaminase (isomerizing)	+	Alanine, aspartate and glutamate metabolism; Amino sugar and nucleotide sugar metabolism; Metabolic pathways; Biosynthesis of antibiotics	0.043	0.521	90.0
3.4.24.13	IgA-specific metalloendopeptidase	+		0.365	0.522	50.1
3.1.3.18	phosphoglycolate phosphatase	+	Glyoxylate and dicarboxylate metabolism; Metabolic pathways; Biosynthesis of secondary metabolites; Biosynthesis of antibiotics	0.070	0.523	87.6
1.5.1.38	FMN reductase (NADPH)	+	**Riboflavin metabolism**; Metabolic pathways	0.197	0.523	75.4
4.99.1.3	sirohydrochlorin cobaltochelatase	-	Porphyrin and chlorophyll metabolism; Metabolic pathways	0.222	0.523	72.7
2.4.1.109	dolichyl-phosphate-mannose---protein mannosyltransferase	-	Other types of O-glycan biosynthesis; Mannose type O-glycan biosynthesis; Metabolic pathways	0.229	0.523	71.8
1.1.98.6	ribonucleoside-triphosphate reductase (formate)	-	Purine metabolism; Pyrimidine metabolism; Metabolic pathways	0.237	0.523	70.8
3.7.1.14	2-hydroxy-6-oxonona-2,4-dienedioate hydrolase	+	Phenylalanine metabolism; Microbial metabolism in diverse environments	0.222	0.524	72.8
5.4.99.25	tRNA pseudouridine55 synthase	+		0.183	0.525	77.2
5.3.3.14	trans-2-decenoyl-[acyl-carrier protein] isomerase	-	Fatty acid biosynthesis	0.191	0.525	76.3
3.4.11.23	PepB aminopeptidase	-	Glutathione metabolism; Metabolic pathways	0.230	0.526	72.1
5.1.3.14	UDP-N-acetylglucosamine 2-epimerase (non-hydrolysing)	-	Amino sugar and nucleotide sugar metabolism; Metabolic pathways	0.457	0.526	*27.2
3.4.24.78	gpr endopeptidase	+		0.093	0.527	85.8
2.7.7.73	sulfur carrier protein ThiS adenylyltransferase	-	Thiamine metabolism; Metabolic pathways	0.177	0.527	78.0
3.4.11.10	bacterial leucyl aminopeptidase	-		0.280	0.529	66.1
2.3.3.5	2-methylcitrate synthase	+	Propanoate metabolism	0.289	0.529	64.8
3.1.3.104	5-amino-6-(5-phospho-D-ribitylamino)uracil phosphatase	+	**Riboflavin metabolism**; Metabolic pathways; Biosynthesis of secondary metabolites	0.211	0.533	75.2
3.2.1.185	non-reducing end beta-L-arabinofuranosidase	-		0.351	0.533	55.3
3.2.1.70	glucan 1,6-alpha-glucosidase	+		0.110	0.534	84.9
4.3.1.3	histidine ammonia-lyase	+	Histidine metabolism; Metabolic pathways	0.535	0.535	*0.0
1.2.99.7	aldehyde dehydrogenase (FAD-independent)	-		0.266	0.537	69.3
2.7.1.40	pyruvate kinase	+	Glycolysis / Gluconeogenesis; Purine metabolism; Pyruvate metabolism; Metabolic pathways; Biosynthesis of secondary metabolites; Microbial metabolism in diverse environments; Biosynthesis of antibiotics	0.139	0.538	82.6
2.7.7.nl		-		0.382	0.538	50.3
1.5.5.1	electron-transferring-flavoprotein dehydrogenase	+		0.325	0.539	61.1
3.2.1.25	beta-mannosidase	+	Other glycan degradation	0.541	0.541	*0.0
5.4.99.12	tRNA pseudouridine38-40 synthase	+		0.118	0.542	84.8
2.7.1.56	1-phosphofructokinase	-	Fructose and mannose metabolism	0.219	0.544	75.6

2.7.1.83	pseudouridine kinase	+	Pyrimidine metabolism	0.300	0.545	65.9
1.17.7.4	4-hydroxy-3-methylbut-2-en-1-yl diphosphate reductase	+	Terpenoid backbone biosynthesis; Metabolic pathways; Biosynthesis of secondary metabolites; Biosynthesis of antibiotics	0.103	0.547	86.3
1.1.1.264	L-idonate 5-dehydrogenase	-		0.504	0.549	*19.5
4.99.1.1	protoporphyrin ferrochelatase	+	Porphyrin and chlorophyll metabolism; Metabolic pathways; Biosynthesis of secondary metabolites	0.313	0.550	65.0
3.1.7.2	guanosine-3',5'-bis(diphosphate) 3'-diphosphatase	-	Purine metabolism	0.183	0.551	79.9
2.6.1.9	histidinol-phosphate transaminase	-	Histidine metabolism; Tyrosine metabolism; Phenylalanine metabolism; Phenylalanine, tyrosine and tryptophan biosynthesis; Novobiocin biosynthesis; Tropane, piperidine and pyridine alkaloid biosynthesis; Metabolic pathways; Biosynthesis of secondary metabolites; Biosynthesis of antibiotics	0.147	0.552	83.2
6.3.2.12	dihydrofolate synthase	-	**Folate biosynthesis**; Metabolic pathways	0.201	0.552	78.3
1.1.1.6	glycerol dehydrogenase	-	Glycerolipid metabolism; Propanoate metabolism; Metabolic pathways	0.204	0.552	78.1
1.3.1.6	fumarate reductase (NADH)	+	Carbon fixation pathways in prokaryotes; Microbial metabolism in diverse environments	0.364	0.552	57.1
3.6.3.24	nickel-transporting ATPase	+		0.276	0.553	70.3
2.5.1.56	N-acetylneuraminate synthase	-	Amino sugar and nucleotide sugar metabolism; Metabolic pathways	0.317	0.553	64.9
3.2.1.78	mannan endo-1,4-beta-mannosidase	+	Fructose and mannose metabolism	0.553	0.553	*0.0
1.1.1.31	3-hydroxyisobutyrate dehydrogenase	-	Valine, leucine and isoleucine degradation; Metabolic pathways	0.139	0.554	84.0
2.4.1.10	levansucrase	+	Starch and sucrose metabolism; Metabolic pathways	0.178	0.555	80.8
4.2.2.25	gellan lyase	+		0.355	0.556	59.4
3.2.1.156	oligosaccharide reducing-end xylanase	+		0.402	0.556	50.7
2.7.3.9	phosphoenolpyruvate---protein phosphotransferase	+		0.209	0.559	78.3
2.5.1.15	dihydropteroate synthase	+	**Folate biosynthesis**; Metabolic pathways	0.227	0.559	76.6
1.2.1.76	succinate-semialdehyde dehydrogenase (acylating)	+	Butanoate metabolism; Carbon fixation pathways in prokaryotes; Metabolic pathways; Microbial metabolism in diverse environments	0.259	0.559	73.2
5.4.99.16	maltose alpha-D-glucosyltransferase	+	Starch and sucrose metabolism; Metabolic pathways	0.232	0.560	76.3
2.4.2.3	uridine phosphorylase	+	Pyrimidine metabolism; Drug metabolism - other enzymes; Metabolic pathways	0.109	0.561	86.8
2.3.1.81	aminoglycoside 3-N-acetyltransferase	+		0.440	0.561	43.2
5.4.3.8	glutamate-1-semialdehyde 2,1-aminomutase	+	Porphyrin and chlorophyll metabolism; Metabolic pathways; Biosynthesis of secondary metabolites; Microbial metabolism in diverse environments	0.117	0.562	86.3
6.6.1.1	magnesium chelatase	+	Porphyrin and chlorophyll metabolism; Metabolic pathways; Biosynthesis of secondary metabolites	0.279	0.562	71.3
3.2.1.6	endo-1,3(4)-beta-glucanase	+		0.462	0.563	*38.1
1.1.1.346	2,5-didehydrogluconate reductase (2-dehydro-L-gulonate-forming)	-		0.206	0.564	79.2
6.2.1.26	o-succinylbenzoate---CoA ligase	-	Ubiquinone and other terpenoid-quinone biosynthesis; Metabolic pathways; Biosynthesis of secondary metabolites	0.396	0.564	53.8
1.1.1.154	ureidoglycolate dehydrogenase	-	Purine metabolism	0.268	0.565	73.0
5.1.3.1	ribulose-phosphate 3-epimerase	-	Pentose phosphate pathway; Pentose and glucuronate interconversions; Carbon fixation in photosynthetic organisms; Metabolic pathways; Biosynthesis of secondary metabolites; Microbial metabolism in diverse environments; Biosynthesis of antibiotics	0.184	0.566	81.3
7.3.2.3	ABC-type sulfate transporter	-		0.210	0.568	79.3
1.4.7.1	glutamate synthase (ferredoxin)	-	Glyoxylate and dicarboxylate metabolism; Nitrogen metabolism; Microbial metabolism in diverse environments	0.144	0.569	84.8
3.1.3.27	phosphatidylglycerophosphatase	-	Glycerophospholipid metabolism; Metabolic pathways	0.259	0.569	74.6
1.1.1.282	quinate/shikimate dehydrogenase [NAD(P)+]	+	Phenylalanine, tyrosine and tryptophan biosynthesis; Metabolic pathways; Biosynthesis of secondary metabolites; Biosynthesis of antibiotics	0.303	0.569	69.5
6.3.1.8	glutathionylspermidine synthase	-	Glutathione metabolism; Metabolic pathways	0.267	0.571	73.9
1.2.1.10	acetaldehyde dehydrogenase (acetylating)	+	Phenylalanine metabolism; Benzoate degradation; Pyruvate metabolism; Dioxin degradation; Xylene degradation; Butanoate metabolism; Metabolic pathways; Microbial metabolism in diverse environments	0.243	0.572	76.6
3.6.3.19	maltose-transporting ATPase	-		0.091	0.573	88.9
2.3.1.n4		-		0.474	0.573	*37.8
2.1.1.35	tRNA (uracil54-C5)-methyltransferase	-		0.186	0.574	82.0
2.7.1.19	phosphoribulokinase	-	Carbon fixation in photosynthetic organisms; Metabolic pathways; Microbial metabolism in diverse environments	0.260	0.576	75.4
2.6.1.88	methionine transaminase	-	Glucosinolate biosynthesis	0.276	0.576	73.6
5.1.2.3	3-hydroxybutyryl-CoA epimerase	-	Fatty acid degradation; Butanoate metabolism	0.288	0.576	72.2
3.4.13.22	D-Ala-D-Ala dipeptidase	+		0.471	0.576	*39.8
2.1.1.187	23S rRNA (guanine745-N1)-methyltransferase	+		0.155	0.577	84.6
3.5.1.104	peptidoglycan-N-acetylglucosamine deacetylase	+		0.538	0.577	*18.1
2.7.7.65	diguanylate cyclase	-		0.275	0.579	74.2
3.1.1.45	carboxymethylenebutenolidase	-	Chlorocyclohexane and chlorobenzene degradation; Fluorobenzoate degradation; Toluene degradation; Metabolic pathways; Microbial metabolism in diverse environments	0.321	0.579	68.7
3.6.3.54	Cu+-exporting ATPase	+		0.331	0.579	67.3
3.6.3.16	arsenite-transporting ATPase	+		0.580	0.580	*0.0
3.6.1.8	ATP diphosphatase	+	Purine metabolism; Pyrimidine metabolism	0.263	0.582	75.9
4.1.3.38	aminodeoxychorismate lyase	-	**Folate biosynthesis**	0.434	0.583	50.9
2.7.7.71	D-glycero-alpha-D-manno-heptose 1-phosphate guanylyltransferase	+	Lipopolysaccharide biosynthesis	0.556	0.583	*13.1
1.6.5.2	NAD(P)H dehydrogenase (quinone)	+	Ubiquinone and other terpenoid-quinone biosynthesis; Biosynthesis of secondary metabolites	0.259	0.584	76.4
5.4.99.26	tRNA pseudouridine65 synthase	+		0.262	0.585	76.3
2.2.1.2	transaldolase	-	Pentose phosphate pathway; Metabolic pathways; Biosynthesis of secondary metabolites; Microbial metabolism in diverse environments; Biosynthesis of antibiotics	0.457	0.585	46.3
5.3.1.27	6-phospho-3-hexuloisomerase	-	Pentose phosphate pathway; Methane metabolism; Metabolic pathways; Microbial metabolism in diverse environments	0.491	0.587	*37.8
4.2.1.28	propanediol dehydratase	+	Propanoate metabolism	0.224	0.588	80.1
1.7.1.15	nitrite reductase (NADH)	-	Nitrogen metabolism; Microbial metabolism in diverse environments	0.324	0.588	69.7
1.1.1.130	3-dehydro-L-gulonate 2-dehydrogenase	+	Pentose and glucuronate interconversions; Ascorbate and aldarate metabolism	0.231	0.590	79.7
3.5.1.110	ureidoacrylate amidohydrolase	-	Pyrimidine metabolism; Metabolic pathways	0.308	0.590	72.1
6.3.2.10	UDP-N-acetylmuramoyl-tripeptide---D-alanyl-D-alanine ligase	-	Lysine biosynthesis; Peptidoglycan biosynthesis; Metabolic pathways	0.218	0.592	81.0
5.4.99.62	D-ribose pyranase	+		0.183	0.593	83.9
2.1.1.188	23S rRNA (guanine748-N1)-methyltransferase	+		0.241	0.593	79.2
3.1.26.5	ribonuclease P	-		0.379	0.593	63.1
4.2.3.12	6-pyruvoyltetrahydropterin synthase	+	**Folate biosynthesis**; Metabolic pathways	0.214	0.594	81.6
3.2.1.39	glucan endo-1,3-beta-D-glucosidase	-	Starch and sucrose metabolism	0.543	0.595	*23.6
1.1.3.15	(S)-2-hydroxy-acid oxidase	-	Glyoxylate and dicarboxylate metabolism; Metabolic pathways; Biosynthesis of secondary metabolites; Microbial metabolism in diverse environments; Biosynthesis of antibiotics	0.169	0.596	85.1
2.5.1.61	hydroxymethylbilane synthase	-	Porphyrin and chlorophyll metabolism; Metabolic pathways; Biosynthesis of secondary metabolites; Microbial metabolism in diverse environments	0.141	0.597	87.1
3.4.22.40	bleomycin hydrolase	+		0.168	0.597	85.3
2.7.9.3	selenide, water dikinase	-	Selenocompound metabolism; Metabolic pathways	0.110	0.599	89.2
2.1.1.198	16S rRNA (cytidine1402-2'-O)-methyltransferase	-		0.209	0.599	82.5
1.8.1.9	thioredoxin-disulfide reductase	+	Selenocompound metabolism	0.240	0.599	79.9
1.1.1.49	glucose-6-phosphate dehydrogenase (NADP+)	-	Pentose phosphate pathway; Glutathione metabolism; Metabolic pathways; Biosynthesis of secondary metabolites; Biosynthesis of antibiotics	0.268	0.600	77.5
1.14.12.10	benzoate 1,2-dioxygenase	-	Benzoate degradation; Fluorobenzoate degradation; Metabolic pathways; Microbial metabolism in diverse environments	0.319	0.601	72.5
3.1.3.23	sugar-phosphatase	+		0.301	0.603	74.7
3.2.1.170	mannosylglycerate hydrolase	+		0.333	0.603	71.1
5.1.3.29	L-fucose mutarotase	+		0.348	0.603	69.3
1.1.1.218	morphine 6-dehydrogenase	+	Isoquinoline alkaloid biosynthesis; Biosynthesis of secondary metabolites	0.371	0.603	66.2
4.1.1.23	orotidine-5'-phosphate decarboxylase	+	Pyrimidine metabolism; Metabolic pathways	0.500	0.603	40.7
1.17.1.2	4-hydroxy-3-methylbut-2-enyl diphosphate reductase	+		0.224	0.604	81.7
6.5.1.1	DNA ligase (ATP)	+		0.047	0.605	93.3
2.7.1.69	protein-Npi-phosphohistidine---sugar phosphotransferase	-		0.206	0.605	83.3
2.3.1.35	glutamate N-acetyltransferase	+	Arginine biosynthesis; Metabolic pathways; Biosynthesis of antibiotics	0.268	0.606	78.3
4.2.99.20	2-succinyl-6-hydroxy-2,4-cyclohexadiene-1-carboxylate synthase	-	Ubiquinone and other terpenoid-quinone biosynthesis; Metabolic pathways; Biosynthesis of secondary metabolites	0.394	0.606	63.4
3.5.1.19	nicotinamidase	+	Nicotinate and nicotinamide metabolism; Metabolic pathways	0.381	0.607	65.6
2.7.1.121	phosphoenolpyruvate---glycerone phosphotransferase	-	Glycerolipid metabolism	0.595	0.607	*6.3
2.4.1.18	1,4-alpha-glucan branching enzyme	+	Starch and sucrose metabolism; Metabolic pathways; Biosynthesis of secondary metabolites	0.222	0.608	82.4
4.1.1.68	5-oxopent-3-ene-1,2,5-tricarboxylate decarboxylase	+	Tyrosine metabolism; Microbial metabolism in diverse environments	0.389	0.608	64.6
3.6.1.7	acylphosphatase	+	Pyruvate metabolism; Aminobenzoate degradation; Microbial metabolism in diverse environments	0.445	0.608	54.8
3.1.4.53	3',5'-cyclic-AMP phosphodiesterase	+	Purine metabolism	0.311	0.609	74.5
6.3.2.14	enterobactin synthase	-	Biosynthesis of siderophore group nonribosomal peptides; Biosynthesis of secondary metabolites; Biosynthesis of antibiotics	0.372	0.609	67.2
1.17.7.3	(E)-4-hydroxy-3-methylbut-2-enyl-diphosphate synthase (flavodoxin)	+	Terpenoid backbone biosynthesis; Metabolic pathways	0.364	0.611	68.6
5.1.3.2	UDP-glucose 4-epimerase	-	Galactose metabolism; Amino sugar and nucleotide sugar metabolism; Metabolic pathways	0.450	0.611	54.6
3.5.2.10	creatininase	-	**Arginine and proline metabolism**	0.113	0.612	89.7
3.1.1.1	carboxylesterase	+	Drug metabolism - other enzymes	0.357	0.613	70.0
2.7.1.30	glycerol kinase	+	Glycerolipid metabolism; Metabolic pathways	0.199	0.616	84.7
3.5.4.9	methenyltetrahydrofolate cyclohydrolase	+	One carbon pool by folate; Carbon fixation pathways in prokaryotes; Metabolic pathways; Microbial metabolism in diverse environments	0.257	0.616	80.4
7.6.2.2	ABC-type xenobiotic transporter	+		0.567	0.616	*23.4
2.1.1.222	2-polyprenyl-6-hydroxyphenol methylase	+	Ubiquinone and other terpenoid-quinone biosynthesis; Metabolic pathways; Biosynthesis of secondary metabolites	0.270	0.617	79.4
2.1.1.172	16S rRNA (guanine1207-N2)-methyltransferase	-		0.317	0.617	75.0
6.2.1.14	6-carboxyhexanoate---CoA ligase	+	**Biotin metabolism**; Metabolic pathways	0.475	0.617	50.8
4.2.1.7	altronate dehydratase	+	Pentose and glucuronate interconversions; Metabolic pathways	0.270	0.618	79.5
2.8.3.8	acetate CoA-transferase	+	Aminobenzoate degradation; Propanoate metabolism; Butanoate metabolism; Metabolic pathways; Microbial metabolism in diverse environments	0.396	0.619	65.7
6.4.1.1	pyruvate carboxylase	+	Citrate cycle (TCA cycle); Pyruvate metabolism; Carbon fixation pathways in prokaryotes; Metabolic pathways; Microbial metabolism in diverse environments	0.313	0.621	76.0
2.7.1.180	FAD:protein FMN transferase	-		0.362	0.621	70.5
2.7.7.77	molybdenum cofactor guanylyltransferase	-	**Folate biosynthesis**; Metabolic pathways	0.182	0.627	86.7
7.5.2.6	ABC-type lipid A-core oligosaccharide transporter	-		0.322	0.629	76.2
1.3.5.3	protoporphyrinogen IX dehydrogenase (quinone)	-	Porphyrin and chlorophyll metabolism; Metabolic pathways; Biosynthesis of secondary metabolites	0.377	0.630	70.3
4.2.1.119	enoyl-CoA hydratase 2	+		0.630	0.630	*0.0
2.1.1.151	cobalt-factor II C20-methyltransferase	-	Porphyrin and chlorophyll metabolism; Metabolic pathways	0.173	0.631	87.6
1.11.1.9	glutathione peroxidase	-	Glutathione metabolism; Arachidonic acid metabolism	0.405	0.631	66.8
2.6.1.82	putrescine---2-oxoglutarate transaminase	+	**Arginine and proline metabolism**; Metabolic pathways	0.441	0.631	61.1
3.1.1.5	lysophospholipase	+	Glycerophospholipid metabolism	0.631	0.631	*0.0
1.3.98.3	coproporphyrinogen dehydrogenase	-	Porphyrin and chlorophyll metabolism; Metabolic pathways; Biosynthesis of secondary metabolites	0.219	0.632	84.8
3.2.1.93	alpha,alpha-phosphotrehalase	-	Starch and sucrose metabolism	0.225	0.633	84.6

1.1.1.36	acetoacetyl-CoA reductase	+	Glyoxylate and dicarboxylate metabolism; Butanoate metabolism; Metabolic pathways; Microbial metabolism in diverse environments	0.345	0.633	74.4
4.2.1.130	D-lactate dehydratase	-	Pyruvate metabolism; Microbial metabolism in diverse environments	0.633	0.633	*0.0
2.7.6.5	GTP diphosphokinase	+	Purine metabolism	0.219	0.634	85.0
1.16.3.2	bacterial non-heme ferritin	-		0.374	0.634	71.3
2.5.1.47	cysteine synthase	-	Cysteine and methionine metabolism; Sulfur metabolism; Metabolic pathways; Biosynthesis of secondary metabolites; Biosynthesis of antibiotics	0.321	0.635	77.1
1.13.11.27	4-hydroxyphenylpyruvate dioxygenase	-	Ubiquinone and other terpenoid-quinone biosynthesis; Tyrosine metabolism; Phenylalanine metabolism; Metabolic pathways	0.258	0.636	82.4
4.3.2.3	ureidoglycolate lyase	+	Purine metabolism; Metabolic pathways	0.596	0.636	*20.1
1.3.98.1	dihydroorotate dehydrogenase (fumarate)	-	Pyrimidine metabolism; Metabolic pathways	0.462	0.637	58.8
1.8.1.14	CoA-disulfide reductase	+		0.603	0.637	*17.6
2.7.1.199	protein-Npi-phosphohistidine---D-glucose phosphotransferase	-	Glycolysis / Gluconeogenesis; Amino sugar and nucleotide sugar metabolism	0.344	0.638	75.3
1.1.1.18	inositol 2-dehydrogenase	+	Streptomycin biosynthesis; Inositol phosphate metabolism; Metabolic pathways; Microbial metabolism in diverse environments; Biosynthesis of antibiotics	0.638	0.638	*0.0
6.1.1.18	glutamine---tRNA ligase	+	Aminoacyl-tRNA biosynthesis; Metabolic pathways	0.333	0.639	76.5
4.2.1.52	dihydrodipicolinate synthase	-		0.354	0.641	74.8
5.1.3.10	CDP-paratose 2-epimerase	-	Amino sugar and nucleotide sugar metabolism	0.396	0.641	69.8
3.6.1.45	UDP-sugar diphosphatase	-		0.345	0.642	75.7
4.1.1.85	3-dehydro-L-gulonate-6-phosphate decarboxylase	+	Pentose and glucuronate interconversions; Ascorbate and aldarate metabolism; Metabolic pathways; Microbial metabolism in diverse environments	0.389	0.642	70.8
2.8.1.8	lipoyl synthase	-	Lipoic acid metabolism; Metabolic pathways	0.421	0.642	66.7
2.7.8.20	phosphatidylglycerol---membrane-oligosaccharide glycerophosphotransferase	-	Glycerolipid metabolism; Metabolic pathways	0.424	0.642	66.2
1.3.3.3	coproporphyrinogen oxidase	-	Porphyrin and chlorophyll metabolism; Metabolic pathways; Biosynthesis of secondary metabolites	0.411	0.643	68.4
2.7.7.3	pantetheine-phosphate adenylyltransferase	-	Pantothenate and CoA biosynthesis; Metabolic pathways	0.602	0.645	*21.6
1.17.99.7	formate dehydrogenase (acceptor)	-		0.337	0.646	77.1
3.2.1.151	xyloglucan-specific endo-beta-1,4-glucanase	-		0.437	0.646	65.0
3.6.3.3	Cd2+-exporting ATPase	-		0.205	0.648	87.0
2.3.1.1	amino-acid N-acetyltransferase	+	Arginine biosynthesis; Metabolic pathways; Biosynthesis of secondary metabolites; Biosynthesis of antibiotics	0.241	0.648	84.9
3.6.1.66	XTP/dITP diphosphatase	-	Purine metabolism	0.406	0.648	69.8
2.5.1.94	adenosyl-chloride synthase	+		0.330	0.650	78.3
3.2.1.122	maltose-6'-phosphate glucosidase	-	Starch and sucrose metabolism	0.375	0.652	74.2
3.5.3.26	(S)-ureidoglycine aminohydrolase	-	Purine metabolism; Microbial metabolism in diverse environments	0.524	0.652	49.9
1.8.4.14	L-methionine (R)-S-oxide reductase	+	Cysteine and methionine metabolism	0.268	0.653	83.6
2.8.1.12	molybdopterin synthase	-	**Folate biosynthesis**; Metabolic pathways	0.341	0.655	77.9
2.3.1.193	tRNAMet cytidine acetyltransferase	-		0.403	0.655	71.5
2.7.1.35	pyridoxal kinase	-	Vitamin B6 metabolism; Metabolic pathways	0.415	0.657	70.3
4.4.1.5	lactoylglutathione lyase	-	Pyruvate metabolism	0.244	0.658	85.6
2.7.7.39	glycerol-3-phosphate cytidylyltransferase	+	Glycerophospholipid metabolism	0.308	0.658	81.2
3.4.21.62	subtilisin	+		0.618	0.658	*21.0
3.5.1.24	choloylglycine hydrolase	+	Primary bile acid biosynthesis; Secondary bile acid biosynthesis; Metabolic pathways	0.365	0.660	76.4
1.1.1.298	3-hydroxypropionate dehydrogenase (NADP+)	-	Carbon fixation pathways in prokaryotes; Microbial metabolism in diverse environments	0.374	0.661	75.7
2.7.1.60	N-acylmannosamine kinase	-	Amino sugar and nucleotide sugar metabolism; Metabolic pathways	0.469	0.661	63.3
2.3.1.61	dihydrolipoyllysine-residue succinyltransferase	+	Citrate cycle (TCA cycle); Lysine degradation; Metabolic pathways; Biosynthesis of secondary metabolites; Microbial metabolism in diverse environments; Biosynthesis of antibiotics	0.519	0.661	53.7
5.3.1.8	mannose-6-phosphate isomerase	-	Fructose and mannose metabolism; Amino sugar and nucleotide sugar metabolism; Metabolic pathways; Biosynthesis of secondary metabolites; Biosynthesis of antibiotics	0.523	0.661	52.8
5.4.99.19	16S rRNA pseudouridine516 synthase	-		0.393	0.664	74.1
6.6.1.2	cobaltochelatase	-	Porphyrin and chlorophyll metabolism; Metabolic pathways	0.397	0.664	73.7
3.1.3.6	3'-nucleotidase	-	Purine metabolism; Pyrimidine metabolism	0.437	0.665	69.0
3.4.19.5	beta-aspartyl-peptidase	-		0.246	0.666	86.2
2.1.1.33	tRNA (guanine46-N7)-methyltransferase	-		0.312	0.667	81.9
3.2.1.31	beta-glucuronidase	+	Pentose and glucuronate interconversions; Glycosaminoglycan degradation; Porphyrin and chlorophyll metabolism; Flavone and flavonol biosynthesis; Drug metabolism - other enzymes; Metabolic pathways; Biosynthesis of secondary metabolites	0.476	0.667	63.5
2.3.1.210	dTDP-4-amino-4,6-dideoxy-D-galactose acyltransferase	-		0.471	0.668	64.6
3.1.8.1	aryldialkylphosphatase	-	Aminobenzoate degradation; Microbial metabolism in diverse environments	0.300	0.669	83.0
4.1.1.112	oxaloacetate decarboxylase	-		0.446	0.670	68.8
2.1.1.107	uroporphyrinogen-III C-methyltransferase	-	Porphyrin and chlorophyll metabolism; Metabolic pathways; Biosynthesis of secondary metabolites; Microbial metabolism in diverse environments	0.236	0.671	87.1
2.10.1.1	molybdopterin molybdotransferase	-	**Folate biosynthesis**; Metabolic pathways	0.310	0.671	82.5
2.1.1.190	23S rRNA (uracil1939-C5)-methyltransferase	-		0.372	0.671	77.3
1.6.99.5	NADH dehydrogenase (quinone)	+		0.403	0.671	74.2
1.11.1.1	NADH peroxidase	-		0.410	0.672	73.5
1.15.1.1	superoxide dismutase	-		0.494	0.672	61.6
4.1.1.98	4-hydroxy-3-polyprenylbenzoate decarboxylase	-	Ubiquinone and other terpenoid-quinone biosynthesis; Metabolic pathways	0.479	0.673	64.3
2.1.1.171	16S rRNA (guanine966-N2)-methyltransferase	-		0.404	0.674	74.5
5.3.3.2	isopentenyl-diphosphate Delta-isomerase	-	Terpenoid backbone biosynthesis; Metabolic pathways; Biosynthesis of secondary metabolites; Biosynthesis of antibiotics	0.583	0.674	41.3
4.2.1.90	L-rhamnonate dehydratase	+	Fructose and mannose metabolism; Microbial metabolism in diverse environments	0.406	0.675	74.5
4.1.2.17	L-fuculose-phosphate aldolase	-	Fructose and mannose metabolism; Microbial metabolism in diverse environments	0.222	0.676	88.3
1.16.1.3	aquacobalamin reductase	-	Porphyrin and chlorophyll metabolism	0.455	0.677	69.0
2.3.1.31	homoserine O-acetyltransferase	-	Cysteine and methionine metabolism; Metabolic pathways; Biosynthesis of antibiotics	0.498	0.677	62.2
3.1.3.26	4-phytase	-	Inositol phosphate metabolism	0.482	0.678	65.2
2.7.8.23	carboxyvinyl-carboxyphosphonate phosphorylmutase	-	Phosphonate and phosphinate metabolism; Biosynthesis of antibiotics	0.539	0.678	54.2
1.1.1.276	serine 3-dehydrogenase (NADP+)	-		0.319	0.679	82.8
3.5.1.42	nicotinamide-nucleotide amidase	+	Nicotinate and nicotinamide metabolism	0.454	0.679	69.5
1.16.1.9	ferric-chelate reductase (NADPH)	-		0.497	0.679	62.8
2.7.8.31	undecaprenyl-phosphate glucose phosphotransferase	-		0.593	0.680	40.5
1.1.1.268	2-(R)-hydroxypropyl-CoM dehydrogenase	+		0.372	0.681	78.8
2.8.3.1	propionate CoA-transferase	-	Pyruvate metabolism; Propanoate metabolism; Styrene degradation; Metabolic pathways; Microbial metabolism in diverse environments	0.454	0.681	69.9
3.1.1.53	sialate O-acetylesterase	+		0.631	0.682	*27.4
2.7.7.38	3-deoxy-manno-octulosonate cytidylyltransferase	-	Lipopolysaccharide biosynthesis; Metabolic pathways	0.683	0.683	*0.0
2.4.2.1	purine-nucleoside phosphorylase	+	Purine metabolism; Pyrimidine metabolism; Nicotinate and nicotinamide metabolism; Metabolic pathways; Biosynthesis of secondary metabolites	0.262	0.685	86.9
3.1.3.70	mannosyl-3-phosphoglycerate phosphatase	+	Fructose and mannose metabolism	0.549	0.685	54.1
4.2.1.3	aconitate hydratase	-	Citrate cycle (TCA cycle); Glyoxylate and dicarboxylate metabolism; Carbon fixation pathways in prokaryotes; Metabolic pathways; Biosynthesis of secondary metabolites; Microbial metabolism in diverse environments; Biosynthesis of antibiotics	0.382	0.686	78.7
3.1.3.16	protein-serine/threonine phosphatase	-		0.392	0.686	77.6
4.2.1.22	cystathionine beta-synthase	+	Glycine, serine and threonine metabolism; Cysteine and methionine metabolism; Metabolic pathways; Biosynthesis of antibiotics	0.421	0.686	74.7
2.1.2.9	methionyl-tRNA formyltransferase	+	One carbon pool by folate; Aminoacyl-tRNA biosynthesis	0.256	0.687	87.4
1.2.1.19	aminobutyraldehyde dehydrogenase	-	Arginine and proline metabolism; beta-Alanine metabolism; Metabolic pathways	0.451	0.687	71.5
2.1.1.264	23S rRNA (guanine2069-N7)-methyltransferase	+		0.405	0.688	76.6
2.3.1.n5		-		0.523	0.688	60.4
3.6.1.26	CDP-diacylglycerol diphosphatase	-	Glycerophospholipid metabolism; Biosynthesis of secondary metabolites	0.463	0.690	70.6
2.1.1.182	16S rRNA (adenine1518-N6/adenine1519-N6)-dimethyltransferase	+		0.356	0.691	81.4
7.2.4.2	oxaloacetate decarboxylase (Na+ extruding)	-		0.388	0.691	78.7
2.4.1.246	mannosylfructose-phosphate synthase	+		0.427	0.691	75.0
4.1.3.40	chorismate lyase	-	Ubiquinone and other terpenoid-quinone biosynthesis; Metabolic pathways; Biosynthesis of secondary metabolites	0.477	0.693	69.2
2.7.4.2	phosphomevalonate kinase	+	Terpenoid backbone biosynthesis; Metabolic pathways; Biosynthesis of secondary metabolites; Biosynthesis of antibiotics	0.240	0.697	89.0
5.3.3.8	Delta3-Delta2-enoyl-CoA isomerase	-	Fatty acid degradation	0.451	0.698	73.4
6.3.2.6	phosphoribosylaminoimidazolesuccinocarboxamide synthase	+	Purine metabolism; Metabolic pathways; Biosynthesis of secondary metabolites; Biosynthesis of antibiotics	0.476	0.698	70.5
4.1.2.5	L-threonine aldolase	-	Glycine, serine and threonine metabolism	0.488	0.698	68.6
5.4.99.27	tRNA pseudouridine13 synthase	+		0.567	0.698	54.1
1.5.1.42	FMN reductase (NADH)	-	**Riboflavin metabolism**; Metabolic pathways	0.405	0.700	78.5
4.1.2.48	low-specificity L-threonine aldolase	-	Glycine, serine and threonine metabolism; Metabolic pathways; Biosynthesis of secondary metabolites; Microbial metabolism in diverse environments; Biosynthesis of antibiotics	0.444	0.700	74.7
3.6.3.44	xenobiotic-transporting ATPase	+		0.465	0.700	72.1
1.1.1.8	glycerol-3-phosphate dehydrogenase (NAD+)	-	Glycerophospholipid metabolism; Biosynthesis of secondary metabolites	0.553	0.700	57.7
2.1.1.181	23S rRNA (adenine1618-N6)-methyltransferase	-		0.603	0.700	45.3
2.5.1.17	corrinoid adenosyltransferase	-	Porphyrin and chlorophyll metabolism; Metabolic pathways	0.457	0.702	73.6
4.2.1.103	cyclohexyl-isocyanide hydratase	+	Caprolactam degradation; Microbial metabolism in diverse environments	0.683	0.702	*12.1
1.4.1.9	leucine dehydrogenase	-	Valine, leucine and isoleucine degradation; Valine, leucine and isoleucine biosynthesis; Metabolic pathways; Biosynthesis of secondary metabolites; Biosynthesis of antibiotics	0.433	0.704	76.6
2.7.2.15	propionate kinase	-	Propanoate metabolism; Metabolic pathways	0.460	0.705	73.7
2.8.2.22	aryl-sulfate sulfotransferase	-		0.570	0.705	55.7
6.3.4.4	adenylosuccinate synthase	+	Purine metabolism; Alanine, aspartate and glutamate metabolism; Metabolic pathways	0.516	0.706	66.2
2.7.7.19	polynucleotide adenylyltransferase	+		0.640	0.706	*34.6
3.5.3.7	guanidinobutyrase	-	**Arginine and proline metabolism**; Metabolic pathways	0.708	0.708	*0.0
1.1.1.205	IMP dehydrogenase	+	Purine metabolism; Drug metabolism - other enzymes; Metabolic pathways; Biosynthesis of secondary metabolites	0.665	0.709	*25.5
1.2.1.8	betaine-aldehyde dehydrogenase	+	Glycine, serine and threonine metabolism; Metabolic pathways	0.497	0.710	70.0
2.7.1.50	hydroxyethylthiazole kinase	-	Thiamine metabolism; Metabolic pathways	0.391	0.711	81.4
3.2.1.54	cyclomaltodextrinase	+	Starch and sucrose metabolism	0.593	0.711	52.0
2.7.1.107	diacylglycerol kinase (ATP)	-	Glycerolipid metabolism; Glycerophospholipid metabolism; Metabolic pathways; Biosynthesis of secondary metabolites	0.711	0.711	*0.0
3.3.2.10	soluble epoxide hydrolase	-	Arachidonic acid metabolism; Chloroalkane and chloroalkene degradation; Metabolic pathways; Microbial metabolism in diverse environments	0.572	0.712	57.3
3.2.1.196	limit dextrin alpha-1,6-maltotetraose-hydrolase	-		0.506	0.713	69.5
1.13.11.16	3-carboxyethylcatechol 2,3-dioxygenase	-	Phenylalanine metabolism; Microbial metabolism in diverse environments	0.540	0.713	63.9
3.5.1.105	chitin disaccharide deacetylase	-		0.543	0.715	64.1

3.6.1.11	exopolyphosphatase	+	Purine metabolism	0.551	0.715	62.5
5.4.99.20	23S rRNA pseudouridine2457 synthase	-		0.614	0.716	47.9
2.7.8.11	CDP-diacylglycerol---inositol 3-phosphatidyltransferase	-	Inositol phosphate metabolism; Glycerophospholipid metabolism; Metabolic pathways	0.623	0.716	45.2
1.1.1.305	UDP-glucuronic acid dehydrogenase (UDP-4-keto-hexauronic acid decarboxylating)	-	Amino sugar and nucleotide sugar metabolism	0.535	0.717	65.7
2.1.2.13	UDP-4-amino-4-deoxy-L-arabinose formyltransferase	-	Amino sugar and nucleotide sugar metabolism	0.535	0.717	65.7
3.1.26.8	ribonuclease M5	-		0.320	0.718	86.9
6.4.1.8	acetophenone carboxylase	-	Ethylbenzene degradation; Microbial metabolism in diverse environments	0.719	0.719	*0.0
2.6.1.21	D-amino-acid transaminase	-	Lysine degradation; **Arginine and proline metabolism**; Phenylalanine metabolism; D-Arginine and D-ornithine metabolism; D-Alanine metabolism; Metabolic pathways	0.385	0.724	83.4
2.4.1.21	starch synthase (glycosyl-transferring)	-	Starch and sucrose metabolism; Metabolic pathways; Biosynthesis of secondary metabolites	0.424	0.724	80.5
1.13.11.15	3,4-dihydroxyphenylacetate 2,3-dioxygenase	-	Tyrosine metabolism; Microbial metabolism in diverse environments	0.438	0.725	79.4
2.7.1.184	sulfofructose kinase	-		0.591	0.725	57.0
1.2.1.5	aldehyde dehydrogenase [NAD(P)+]	+	Glycolysis / Gluconeogenesis; Histidine metabolism; Tyrosine metabolism; Phenylalanine metabolism; beta-Alanine metabolism; Metabolism of xenobiotics by cytochrome P450; Drug metabolism - cytochrome P450; Metabolic pathways; Biosynthesis of secondary metabolites; Microbial metabolism in diverse environments	0.610	0.726	53.0
3.5.2.7	imidazolonepropionase	+	Histidine metabolism; Metabolic pathways	0.726	0.726	*0.0
1.3.5.4	fumarate reductase (quinol)	-	Citrate cycle (TCA cycle); Pyruvate metabolism; Butanoate metabolism; Carbon fixation pathways in prokaryotes; Metabolic pathways; Biosynthesis of secondary metabolites; Microbial metabolism in diverse environments; Biosynthesis of antibiotics	0.498	0.727	73.4
1.2.1.46	formaldehyde dehydrogenase	+	Chloroalkane and chloroalkene degradation; Methane metabolism; Metabolic pathways; Microbial metabolism in diverse environments	0.403	0.728	82.7
2.6.1.11	acetylornithine transaminase	-	Arginine biosynthesis; Metabolic pathways; Biosynthesis of secondary metabolites; Biosynthesis of antibiotics	0.258	0.730	90.7
4.2.1.30	glycerol dehydratase	+	Glycerolipid metabolism	0.514	0.731	72.4
2.7.7.80	molybdopterin-synthase adenylyltransferase	+		0.303	0.732	88.9
5.1.3.22	L-ribulose-5-phosphate 3-epimerase	-	Pentose and glucuronate interconversions; Ascorbate and aldarate metabolism; Metabolic pathways; Microbial metabolism in diverse environments	0.426	0.732	81.5
2.7.1.193	protein-Npi-phosphohistidine---N-acetyl-D-glucosamine phosphotransferase	-	Amino sugar and nucleotide sugar metabolism	0.470	0.734	77.9
6.2.1.48	carnitine---CoA ligase	-		0.623	0.735	52.6
1.4.1.1	alanine dehydrogenase	-	Alanine, aspartate and glutamate metabolism; Taurine and hypotaurine metabolism; Metabolic pathways	0.712	0.736	*16.8
5.3.1.31	sulfoquinovose isomerase	+		0.736	0.736	*0.0
3.5.1.9	arylformamidase	+	Tryptophan metabolism; Glyoxylate and dicarboxylate metabolism; Metabolic pathways	0.485	0.737	76.9
1.3.5.1	succinate dehydrogenase	+	Citrate cycle (TCA cycle); Oxidative phosphorylation; Butanoate metabolism; Carbon fixation pathways in prokaryotes; Metabolic pathways; Biosynthesis of secondary metabolites; Microbial metabolism in diverse environments; Biosynthesis of antibiotics	0.527	0.738	72.0
2.7.7.89	[glutamine synthetase]-adenylyl-L-tyrosine phosphorylase	+		0.517	0.739	73.7
2.3.1.29	glycine C-acetyltransferase	-	Glycine, serine and threonine metabolism	0.538	0.739	70.7
3.2.1.28	alpha,alpha-trehalase	+	Starch and sucrose metabolism; Metabolic pathways	0.457	0.741	80.3
2.1.2.2	phosphoribosylglycinamide formyltransferase 1	-	Purine metabolism; One carbon pool by folate; Metabolic pathways; Biosynthesis of secondary metabolites; Biosynthesis of antibiotics	0.596	0.742	61.3
4.99.1.4	sirohydrochlorin ferrochelatase	-	Porphyrin and chlorophyll metabolism; Metabolic pathways; Biosynthesis of secondary metabolites	0.488	0.743	77.6
6.3.4.15	biotin---[biotin carboxyl-carrier protein] ligase	+	**Biotin metabolism**; Metabolic pathways	0.198	0.745	93.6
1.7.1.7	GMP reductase	+	Purine metabolism	0.426	0.745	83.3
3.4.21.96	lactocepin	+		0.635	0.746	53.4
2.7.1.66	undecaprenol kinase	+	Peptidoglycan biosynthesis	0.656	0.746	47.1
4.2.2.12	xanthan lyase	-		0.746	0.746	*0.0
3.6.1.54	UDP-2,3-diacylglucosamine diphosphatase	+	Lipopolysaccharide biosynthesis; Metabolic pathways	0.527	0.747	74.0
2.7.7.76	molybdenum cofactor cytidylyltransferase	-	**Folate biosynthesis**	0.553	0.747	70.5
7.6.2.7	ABC-type taurine transporter	-		0.629	0.747	55.3
1.1.1.22	UDP-glucose 6-dehydrogenase	-	Pentose and glucuronate interconversions; Ascorbate and aldarate metabolism; Amino sugar and nucleotide sugar metabolism; Metabolic pathways	0.541	0.748	72.4
2.4.2.43	lipid IVA 4-amino-4-deoxy-L-arabinosyltransferase	+		0.576	0.749	67.2
1.1.1.83	D-malate dehydrogenase (decarboxylating)	+	Butanoate metabolism	0.582	0.749	66.3
2.6.1.66	valine---pyruvate transaminase	-	Valine, leucine and isoleucine biosynthesis; Metabolic pathways; Biosynthesis of secondary metabolites; Biosynthesis of antibiotics	0.713	0.750	*25.4
3.1.3.25	inositol-phosphate phosphatase	-	Streptomycin biosynthesis; Inositol phosphate metabolism; Metabolic pathways; Biosynthesis of antibiotics	0.701	0.752	*32.4
4.1.99.3	deoxyribodipyrimidine photo-lyase	-		0.536	0.753	74.2
1.5.99.8	proline dehydrogenase	+		0.504	0.754	78.0
1.8.4.11	peptide-methionine (S)-S-oxide reductase	-		0.545	0.755	73.4
3.1.26.12	ribonuclease E	+		0.493	0.758	79.9
2.4.2.8	hypoxanthine phosphoribosyltransferase	+	Purine metabolism; Drug metabolism - other enzymes; Metabolic pathways; Biosynthesis of secondary metabolites	0.652	0.758	53.4
3.4.17.14	zinc D-Ala-D-Ala carboxypeptidase	+	Peptidoglycan biosynthesis	0.703	0.758	*34.7
3.6.1.9	nucleotide diphosphatase	-	Purine metabolism; Pyrimidine metabolism; Starch and sucrose metabolism; **Riboflavin metabolism**; Nicotinate and nicotinamide metabolism; Pantothenate and CoA biosynthesis; Metabolic pathways	0.536	0.759	75.5
1.2.1.21	glycolaldehyde dehydrogenase	-	Glyoxylate and dicarboxylate metabolism; Microbial metabolism in diverse environments	0.593	0.760	67.3
4.2.1.12	phosphogluconate dehydratase	-	Pentose phosphate pathway; Metabolic pathways; Microbial metabolism in diverse environments	0.573	0.761	70.8
4.2.2.7	heparin lyase	-		0.702	0.761	*36.5
4.2.3.4	3-dehydroquinate synthase	-	Phenylalanine, tyrosine and tryptophan biosynthesis; Metabolic pathways; Biosynthesis of secondary metabolites; Biosynthesis of antibiotics	0.459	0.762	83.3
2.4.1.325	TDP-N-acetylfucosamine:lipid II N-acetylfucosaminyltransferase	-		0.569	0.762	71.8
1.2.4.1	pyruvate dehydrogenase (acetyl-transferring)	+	Glycolysis / Gluconeogenesis; Citrate cycle (TCA cycle); Pyruvate metabolism; Metabolic pathways; Biosynthesis of secondary metabolites; Microbial metabolism in diverse environments; Biosynthesis of antibiotics	0.615	0.763	64.0
4.4.1.13	cysteine-S-conjugate beta-lyase	-	Selenocompound metabolism	0.614	0.765	65.0
3.5.1.n3		-		0.624	0.768	63.8
5.4.2.7	phosphopentomutase	+	Pentose phosphate pathway; Purine metabolism	0.416	0.770	87.1
3.6.3.12	K+-transporting ATPase	-		0.647	0.772	60.0
3.4.13.9	Xaa-Pro dipeptidase	-		0.526	0.773	79.3
6.1.1.22	asparagine---tRNA ligase	-	Aminoacyl-tRNA biosynthesis	0.599	0.774	70.3
3.4.21.107	peptidase Do	-		0.631	0.774	64.5
1.2.1.60	5-carboxymethyl-2-hydroxymuconic-semialdehyde dehydrogenase	+	Tyrosine metabolism; Microbial metabolism in diverse environments	0.774	0.774	*0.0
3.5.4.42	N-isopropylammelide isopropylaminohydrolase	+	Atrazine degradation; Metabolic pathways; Microbial metabolism in diverse environments	0.685	0.776	50.8
2.3.1.275	acyl phosphate:glycerol-3-phosphate acyltransferase	+		0.488	0.777	83.3
2.4.1.212	hyaluronan synthase	+		0.515	0.778	81.3
2.7.1.89	thiamine kinase	-	Thiamine metabolism; Metabolic pathways	0.547	0.778	78.0
3.1.1.2	arylesterase	+	Bisphenol degradation; Metabolic pathways; Microbial metabolism in diverse environments	0.612	0.778	69.0
2.7.7.12	UDP-glucose---hexose-1-phosphate uridylyltransferase	+	Galactose metabolism; Amino sugar and nucleotide sugar metabolism; Metabolic pathways	0.535	0.779	79.6
7.2.2.6	P-type K+ transporter	-		0.547	0.780	78.4
2.3.1.267	[ribosomal protein S5]-alanine N-acetyltransferase	+		0.639	0.780	64.4
2.3.1.118	N-hydroxyarylamine O-acetyltransferase	-		0.641	0.782	64.8
1.97.1.9	selenate reductase	-	Selenocompound metabolism	0.571	0.784	76.6
3.5.1.124	protein deglycase	-		0.677	0.784	56.9
5.4.2.8	phosphomannomutase	-	Fructose and mannose metabolism; Amino sugar and nucleotide sugar metabolism; Metabolic pathways; Biosynthesis of secondary metabolites; Biosynthesis of antibiotics	0.648	0.786	64.4
3.4.19.13	glutathione gamma-glutamate hydrolase	+	Glutathione metabolism; Metabolic pathways	0.591	0.788	75.0
1.14.14.5	alkanesulfonate monooxygenase	-	Sulfur metabolism	0.570	0.789	77.8
2.7.7.59	[protein-PII] uridylyltransferase	+		0.603	0.789	73.7
6.2.1.17	propionate---CoA ligase	+	Propanoate metabolism; Metabolic pathways	0.697	0.790	53.2
1.2.1.71	succinylglutamate-semialdehyde dehydrogenase	-	**Arginine and proline metabolism**; Metabolic pathways	0.605	0.791	73.6
2.3.1.190	acetoin dehydrogenase system	+		0.601	0.792	74.5
2.3.3.9	malate synthase	-	Pyruvate metabolism; Glyoxylate and dicarboxylate metabolism; Metabolic pathways; Biosynthesis of secondary metabolites; Microbial metabolism in diverse environments	0.595	0.793	75.5
3.1.21.7	deoxyribonuclease V	-		0.640	0.793	68.5
1.7.1.4	nitrite reductase [NAD(P)H]	-	Nitrogen metabolism	0.660	0.794	64.9
1.8.7.1	assimilatory sulfite reductase (ferredoxin)	-	Sulfur metabolism; Metabolic pathways; Microbial metabolism in diverse environments	0.720	0.794	46.9
3.6.3.2	Mg2+-importing ATPase	-		0.621	0.796	72.7
2.4.1.250	D-inositol-3-phosphate glycosyltransferase	-		0.796	0.796	*0.0
5.1.1.7	diaminopimelate epimerase	+	Lysine biosynthesis; Metabolic pathways; Biosynthesis of secondary metabolites; Microbial metabolism in diverse environments; Biosynthesis of antibiotics	0.473	0.797	87.1
3.6.1.12	dCTP diphosphatase	-	Pyrimidine metabolism; Metabolic pathways	0.587	0.797	77.5
5.4.99.22	23S rRNA pseudouridine2605 synthase	-		0.629	0.799	72.3
3.6.3.31	polyamine-transporting ATPase	+		0.556	0.800	81.5
2.6.1.81	succinylornithine transaminase	-	**Arginine and proline metabolism**; Metabolic pathways	0.595	0.801	77.5
1.2.1.70	glutamyl-tRNA reductase	+	Porphyrin and chlorophyll metabolism; Metabolic pathways; Biosynthesis of secondary metabolites; Microbial metabolism in diverse environments	0.546	0.803	82.9
1.2.1.72	erythrose-4-phosphate dehydrogenase	-	Vitamin B6 metabolism; Metabolic pathways	0.649	0.803	70.0
1.17.1.9	formate dehydrogenase	-	Glyoxylate and dicarboxylate metabolism; Methane metabolism; Metabolic pathways; Microbial metabolism in diverse environments	0.608	0.804	76.6
2.3.1.9	acetyl-CoA C-acetyltransferase	-	Fatty acid degradation; Synthesis and degradation of ketone bodies; Valine, leucine and isoleucine degradation; Lysine degradation; Benzoate degradation; Tryptophan metabolism; Pyruvate metabolism; Glyoxylate and dicarboxylate metabolism; Propanoate metabolism; Butanoate metabolism; Carbon fixation pathways in prokaryotes; Terpenoid backbone biosynthesis; Metabolic pathways; Biosynthesis of secondary metabolites; Microbial metabolism in diverse environments; Biosynthesis of antibiotics	0.459	0.806	89.0
2.3.2.2	gamma-glutamyltransferase	+	Taurine and hypotaurine metabolism; Cyanoamino acid metabolism; Glutathione metabolism	0.639	0.806	72.5
3.2.1.98	glucan 1,4-alpha-maltohexaosidase	+		0.685	0.806	63.5
2.4.2.15	guanosine phosphorylase	+	Purine metabolism; Metabolic pathways	0.705	0.806	57.7
3.1.3.12	trehalose-phosphatase	+	Starch and sucrose metabolism; Metabolic pathways	0.571	0.807	81.5
7.4.2.1	ABC-type polar-amino-acid transporter	-		0.713	0.807	55.7
2.6.1.39	2-aminoadipate transaminase	-	Lysine biosynthesis; Lysine degradation; Metabolic pathways; Biosynthesis of antibiotics	0.778	0.808	*26.0
2.3.1.242	Kdo2-lipid IVA palmitoleoyltransferase	-		0.646	0.809	72.1
2.7.8.37	alpha-D-ribose 1-methylphosphonate 5-triphosphate synthase	-	Phosphonate and phosphinate metabolism	0.723	0.811	54.5
2.3.1.191	UDP-3-O-(3-hydroxyacyl)glucosamine N-acyltransferase	-	Lipopolysaccharide biosynthesis; Metabolic pathways	0.759	0.811	*39.1
1.1.1.91	aryl-alcohol dehydrogenase (NADP+)	-		0.719	0.812	56.5
4.3.1.15	diaminopropionate ammonia-lyase	-		0.507	0.813	87.2
2.8.1.2	3-mercaptopyruvate sulfurtransferase	-	Cysteine and methionine metabolism; Metabolic pathways	0.664	0.813	70.3

2.7.11.33	[pyruvate, water dikinase] kinase	-		0.484	0.814	88.7
2.7.4.28	[pyruvate, water dikinase]-phosphate phosphotransferase	-		0.484	0.814	88.7
5.3.1.6	ribose-5-phosphate isomerase	-	Pentose phosphate pathway; Fructose and mannose metabolism; Carbon fixation in photosynthetic organisms; Metabolic pathways; Biosynthesis of secondary metabolites; Microbial metabolism in diverse environments; Biosynthesis of antibiotics	0.536	0.815	85.6
2.7.1.85	beta-glucoside kinase	+		0.567	0.817	83.6
3.6.1.40	guanosine-5'-triphosphate,3'-diphosphate phosphatase	-	Purine metabolism	0.701	0.817	63.8
1.2.1.77	3,4-dehydroadipyl-CoA semialdehyde dehydrogenase (NADP+)	+	Benzoate degradation	0.687	0.818	67.3
6.2.1.22	[citrate (pro-3S)-lyase] ligase	-		0.637	0.819	76.5
2.7.1.202	protein-Npi-phosphohistidine---D-fructose phosphotransferase	+	Fructose and mannose metabolism; Metabolic pathways; Microbial metabolism in diverse environments	0.707	0.820	63.4
2.3.1.245	3-hydroxy-5-phosphooxypentane-2,4-dione thiolase	+		0.708	0.820	63.0
1.8.1.7	glutathione-disulfide reductase	-	Glutathione metabolism	0.651	0.821	75.0
2.4.1.180	lipopolysaccharide N-acetylmannosaminouronosyltransferase	-		0.675	0.821	70.8
3.2.1.51	alpha-L-fucosidase	+	Other glycan degradation	0.819	0.821	*2.1
6.3.2.3	glutathione synthase	+	Cysteine and methionine metabolism; Glutathione metabolism; Metabolic pathways	0.724	0.822	59.7
2.4.2.14	amidophosphoribosyltransferase	+	Purine metabolism; Alanine, aspartate and glutamate metabolism; Metabolic pathways; Biosynthesis of secondary metabolites; Biosynthesis of antibiotics	0.538	0.823	86.8
1.1.3.21	glycerol-3-phosphate oxidase	-	Glycerophospholipid metabolism; Biosynthesis of secondary metabolites	0.642	0.823	77.0
5.3.3.6	methylitaconate Delta-isomerase	-	Nicotinate and nicotinamide metabolism; Microbial metabolism in diverse environments	0.670	0.824	72.6
6.3.5.8	aminodeoxychorismate synthase	+		0.691	0.825	69.0
4.1.3.17	4-hydroxy-4-methyl-2-oxoglutarate aldolase	-	Benzoate degradation; C5-Branched dibasic acid metabolism; Microbial metabolism in diverse environments	0.638	0.827	78.5
4.3.2.7	glutathione-specific gamma-glutamylcyclotransferase	-	Glutathione metabolism	0.659	0.827	75.3
2.3.1.181	lipoyl(octanoyl) transferase	-	Lipoic acid metabolism; Metabolic pathways	0.656	0.829	76.5
3.5.3.1	arginase	+	Arginine biosynthesis; **Arginine and proline metabolism**; Metabolic pathways; Biosynthesis of secondary metabolites; Biosynthesis of antibiotics	0.812	0.829	*17.2
1.14.12.17	nitric oxide dioxygenase	-		0.688	0.830	71.4
3.1.4.46	glycerophosphodiester phosphodiesterase	-	Glycerophospholipid metabolism	0.589	0.831	84.4
2.4.1.227	undecaprenyldiphospho-muramoylpentapeptide beta-N-acetylglucosaminyltransferase	-	Peptidoglycan biosynthesis; Metabolic pathways	0.603	0.831	83.1
2.7.2.2	carbamate kinase	-	Arginine biosynthesis; Purine metabolism; Nitrogen metabolism; Metabolic pathways; Microbial metabolism in diverse environments	0.709	0.831	67.4
4.2.1.17	enoyl-CoA hydratase	-	Fatty acid elongation; Fatty acid degradation; Valine, leucine and isoleucine degradation; Geraniol degradation; Lysine degradation; Phenylalanine metabolism; Benzoate degradation; Tryptophan metabolism; beta-Alanine metabolism; alpha-Linolenic acid metabolism; Aminobenzoate degradation; Propanoate metabolism; Butanoate metabolism; Carbon fixation pathways in prokaryotes; Limonene and pinene degradation; Caprolactam degradation; Biosynthesis of unsaturated fatty acids; Metabolic pathways; Biosynthesis of secondary metabolites; Microbial metabolism in diverse environments; Biosynthesis of antibiotics	0.831	0.831	*0.0
2.7.1.148	4-(cytidine 5'-diphospho)-2-C-methyl-D-erythritol kinase	+	Terpenoid backbone biosynthesis; Metabolic pathways; Biosynthesis of secondary metabolites; Biosynthesis of antibiotics	0.623	0.834	81.9
2.7.1.208	protein-Npi-phosphohistidine---maltose phosphotransferase	-	Starch and sucrose metabolism	0.701	0.834	70.2
3.5.4.10	IMP cyclohydrolase	+	Purine metabolism; Metabolic pathways; Biosynthesis of secondary metabolites; Biosynthesis of antibiotics	0.834	0.834	*0.0
1.1.2.3	L-lactate dehydrogenase (cytochrome)	-	Pyruvate metabolism; Metabolic pathways	0.614	0.835	82.9
7.1.1.3	ubiquinol oxidase (H+-transporting)	-		0.694	0.835	72.0
4.2.2.n2		-		0.709	0.835	68.8
3.1.1.41	cephalosporin-C deacetylase	+	Penicillin and cephalosporin biosynthesis; Biosynthesis of antibiotics	0.835	0.835	*0.0
1.4.3.19	glycine oxidase	+	Thiamine metabolism; Metabolic pathways	0.668	0.837	76.9
4.2.1.75	uroporphyrinogen-III synthase	-	Porphyrin and chlorophyll metabolism; Metabolic pathways; Biosynthesis of secondary metabolites; Microbial metabolism in diverse environments	0.702	0.837	71.3
3.2.2.28	double-stranded uracil-DNA glycosylase	+		0.720	0.837	67.2
3.4.23.51	HycI peptidase	+		0.723	0.840	67.7
1.1.1.127	2-dehydro-3-deoxy-D-gluconate 5-dehydrogenase	+	Pentose and glucuronate interconversions	0.662	0.841	78.8
4.2.1.40	glucarate dehydratase	-	Ascorbate and aldarate metabolism; Metabolic pathways	0.612	0.842	84.6
5.3.1.13	arabinose-5-phosphate isomerase	+	Lipopolysaccharide biosynthesis; Metabolic pathways	0.678	0.844	77.6
2.6.1.87	UDP-4-amino-4-deoxy-L-arabinose aminotransferase	-	Amino sugar and nucleotide sugar metabolism	0.662	0.846	80.3
5.4.99.28	tRNA pseudouridine32 synthase	+		0.711	0.847	72.9
2.7.1.189	autoinducer-2 kinase	-		0.686	0.848	77.6
2.7.1.59	N-acetylglucosamine kinase	-	Amino sugar and nucleotide sugar metabolism; Metabolic pathways	0.648	0.849	82.5
2.7.1.17	xylulokinase	+	Pentose and glucuronate interconversions; Metabolic pathways	0.673	0.849	79.6
1.5.1.41	riboflavin reductase [NAD(P)H]	+	**Riboflavin metabolism**; Metabolic pathways	0.752	0.849	63.5
1.1.1.2	alcohol dehydrogenase (NADP+)	-	Glycolysis / Gluconeogenesis; Pentose and glucuronate interconversions; Glycerolipid metabolism; Caprolactam degradation; Metabolic pathways; Biosynthesis of secondary metabolites; Microbial metabolism in diverse environments; Biosynthesis of antibiotics	0.635	0.850	84.1
2.7.7.72	CCA tRNA nucleotidyltransferase	-		0.697	0.850	76.3
3.2.1.11	dextranase	+	Starch and sucrose metabolism	0.654	0.851	82.5
1.6.5.5	NADPH:quinone reductase	+		0.711	0.851	74.4
2.4.2.28	S-methyl-5'-thioadenosine phosphorylase	+	Cysteine and methionine metabolism; Metabolic pathways	0.677	0.852	79.9
5.1.3.9	N-acylglucosamine-6-phosphate 2-epimerase	-	Amino sugar and nucleotide sugar metabolism	0.599	0.853	87.6
5.1.1.4	proline racemase	+	**Arginine and proline metabolism**; Metabolic pathways	0.770	0.854	60.3
3.1.1.84	cocaine esterase	-	Tropane, piperidine and pyridine alkaloid biosynthesis; Metabolic pathways; Biosynthesis of secondary metabolites	0.687	0.855	79.5
2.7.1.201	protein-Npi-phosphohistidine---trehalose phosphotransferase	-	Starch and sucrose metabolism	0.658	0.857	83.4
2.3.1.12	dihydrolipoyllysine-residue acetyltransferase	+	Glycolysis / Gluconeogenesis; Citrate cycle (TCA cycle); Pyruvate metabolism; Metabolic pathways; Biosynthesis of secondary metabolites; Microbial metabolism in diverse environments; Biosynthesis of antibiotics	0.689	0.858	79.9
3.5.1.5	urease	-	Arginine biosynthesis; Purine metabolism; Atrazine degradation; Metabolic pathways; Microbial metabolism in diverse environments	0.621	0.859	87.0
1.2.1.11	aspartate-semialdehyde dehydrogenase	+	Glycine, serine and threonine metabolism; Monobactam biosynthesis; Cysteine and methionine metabolism; Lysine biosynthesis; Metabolic pathways; Biosynthesis of secondary metabolites; Microbial metabolism in diverse environments; Biosynthesis of antibiotics	0.795	0.859	53.1
3.5.4.13	dCTP deaminase	+	Pyrimidine metabolism; Metabolic pathways	0.803	0.859	49.7
3.1.1.96	D-aminoacyl-tRNA deacylase	+		0.742	0.860	71.5
1.2.7.1	pyruvate synthase	+	Glycolysis / Gluconeogenesis; Citrate cycle (TCA cycle); Pyruvate metabolism; Nitrotoluene degradation; Propanoate metabolism; Butanoate metabolism; Methane metabolism; Carbon fixation pathways in prokaryotes; Metabolic pathways; Microbial metabolism in diverse environments; Biosynthesis of antibiotics	0.578	0.861	90.1
1.18.1.1	rubredoxin---NAD+ reductase	+	Fatty acid degradation	0.716	0.861	76.7
4.1.99.19	2-iminoacetate synthase	-	Thiamine metabolism; Metabolic pathways	0.738	0.861	72.8
4.1.1.3	oxaloacetate decarboxylase	-	Pyruvate metabolism; Metabolic pathways	0.774	0.861	62.9
6.3.2.8	UDP-N-acetylmuramate---L-alanine ligase	-	D-Glutamine and D-glutamate metabolism; Peptidoglycan biosynthesis; Metabolic pathways	0.734	0.862	73.8
2.7.7.61	citrate lyase holo-[acyl-carrier protein] synthase	-		0.758	0.862	68.2
4.4.1.16	selenocysteine lyase	-	Selenocompound metabolism; Metabolic pathways	0.694	0.863	80.7
2.7.1.196	protein-Npi-phosphohistidine---N,N'-diacetylchitobiose phosphotransferase	+		0.741	0.863	72.8
1.14.13.107	limonene 1,2-monooxygenase	-	Limonene and pinene degradation	0.791	0.863	57.8
1.1.1.157	3-hydroxybutyryl-CoA dehydrogenase	+	Phenylalanine metabolism; Benzoate degradation; Butanoate metabolism; Metabolic pathways; Microbial metabolism in diverse environments	0.592	0.866	90.1
1.3.7.8	benzoyl-CoA reductase	-	Benzoate degradation; Metabolic pathways; Microbial metabolism in diverse environments	0.710	0.866	79.4
3.6.1.41	bis(5'-nucleosyl)-tetraphosphatase (symmetrical)	-	Purine metabolism	0.726	0.866	76.9
2.3.1.109	arginine N-succinyltransferase	-	**Arginine and proline metabolism**; Metabolic pathways	0.786	0.866	61.3
4.2.1.11	phosphopyruvate hydratase	-	Glycolysis / Gluconeogenesis; Methane metabolism; Metabolic pathways; Biosynthesis of secondary metabolites; Microbial metabolism in diverse environments; Biosynthesis of antibiotics	0.694	0.867	82.0
3.6.4.13	RNA helicase	+		0.642	0.868	87.3
4.1.2.20	2-dehydro-3-deoxyglucarate aldolase	+	Ascorbate and aldarate metabolism; Metabolic pathways	0.767	0.869	69.1
2.1.1.199	16S rRNA (cytosine1402-N4)-methyltransferase	-		0.689	0.870	83.2
1.2.1.16	succinate-semialdehyde dehydrogenase [NAD(P)+]	+	Alanine, aspartate and glutamate metabolism; Tyrosine metabolism; Butanoate metabolism; Nicotinate and nicotinamide metabolism; Metabolic pathways; Microbial metabolism in diverse environments	0.727	0.870	78.1
1.11.1.18	bromide peroxidase	-		0.756	0.870	72.3
3.2.1.86	6-phospho-beta-glucosidase	-	Glycolysis / Gluconeogenesis; Starch and sucrose metabolism	0.602	0.872	90.5
1.7.99.4	nitrate reductase	+		0.745	0.873	76.0
4.1.3.30	methylisocitrate lyase	-	Propanoate metabolism	0.790	0.876	66.0
1.7.5.1	nitrate reductase (quinone)	-	Nitrogen metabolism; Microbial metabolism in diverse environments	0.801	0.877	62.1
1.8.99.2	adenylyl-sulfate reductase	+	Sulfur metabolism; Metabolic pathways; Microbial metabolism in diverse environments	0.774	0.878	71.5
2.1.1.174	23S rRNA (guanine1835-N2)-methyltransferase	+		0.813	0.878	57.5
2.4.1.57	phosphatidylinositol alpha-mannosyltransferase	-		0.791	0.879	66.9
2.6.1.57	aromatic-amino-acid transaminase	-	Cysteine and methionine metabolism; Lysine biosynthesis; Tyrosine metabolism; Phenylalanine metabolism; Phenylalanine, tyrosine and tryptophan biosynthesis; Novobiocin biosynthesis; Isoquinoline alkaloid biosynthesis; Tropane, piperidine and pyridine alkaloid biosynthesis; Metabolic pathways; Biosynthesis of secondary metabolites; Biosynthesis of antibiotics	0.742	0.881	79.2
2.7.11.1	non-specific serine/threonine protein kinase	+		0.727	0.883	82.3
1.7.2.3	trimethylamine-N-oxide reductase	-		0.794	0.884	68.8
6.1.1.10	methionine---tRNA ligase	-	Selenocompound metabolism; Aminoacyl-tRNA biosynthesis	0.764	0.886	77.2
5.3.1.32	(4S)-4-hydroxy-5-phosphooxypentane-2,3-dione isomerase	-		0.779	0.886	73.9
1.14.14.51	(S)-limonene 6-monooxygenase	-	Monoterpenoid biosynthesis; Limonene and pinene degradation; Biosynthesis of secondary metabolites	0.798	0.886	68.5
1.1.5.2	glucose 1-dehydrogenase (PQQ, quinone)	-	Pentose phosphate pathway	0.789	0.887	71.8
3.2.1.17	lysozyme	+		0.767	0.888	77.4
4.2.1.49	urocanate hydratase	+	Histidine metabolism; Metabolic pathways	0.856	0.889	41.1
1.1.1.291	2-hydroxymethylglutarate dehydrogenase	+	Nicotinate and nicotinamide metabolism; Microbial metabolism in diverse environments	0.746	0.890	81.9
2.5.1.64	2-succinyl-6-hydroxy-2,4-cyclohexadiene-1-carboxylate synthase	-		0.837	0.891	55.8
4.2.1.99	2-methylisocitrate dehydratase	-	Propanoate metabolism	0.821	0.893	64.7
3.1.1.32	phospholipase A1	-	Glycerophospholipid metabolism; alpha-Linolenic acid metabolism; Metabolic pathways; Biosynthesis of secondary metabolites	0.797	0.894	73.0
3.5.1.96	succinylglutamate desuccinylase	-	**Arginine and proline metabolism**; Metabolic pathways	0.799	0.894	72.6
6.1.1.19	arginine---tRNA ligase	-	Aminoacyl-tRNA biosynthesis	0.870	0.896	*36.5
1.1.1.60	2-hydroxy-3-oxopropionate reductase	+	Glyoxylate and dicarboxylate metabolism; Metabolic pathways	0.714	0.897	87.6
1.2.1.2	formate dehydrogenase	-		0.789	0.897	76.5
1.17.5.3	formate dehydrogenase-N	+		0.812	0.897	70.2
1.5.1.39	FMN reductase [NAD(P)H]	-	**Riboflavin metabolism**; Metabolic pathways	0.898	0.898	*0.0
3.2.1.141	4-alpha-D-{(1->4)-alpha-D-glucano}trehalose trehalohydrolase	+	Starch and sucrose metabolism; Metabolic pathways; Biosynthesis of secondary metabolites	0.852	0.900	55.0

1.4.99.6	D-arginine dehydrogenase	+	D-Arginine and D-ornithine metabolism; Metabolic pathways	0.817	0.901	71.3
2.2.1.1	transketolase	-	Pentose phosphate pathway; Carbon fixation in photosynthetic organisms; Biosynthesis of ansamycins; Metabolic pathways; Biosynthesis of secondary metabolites; Microbial metabolism in diverse environments; Biosynthesis of antibiotics	0.721	0.903	88.3
5.1.99.8	7,8-dihydroneopterin epimerase	-	**Folate biosynthesis**	0.818	0.905	72.9
2.4.1.5	dextransucrase	-	Starch and sucrose metabolism	0.825	0.905	70.8
3.4.13.20	beta-Ala-His dipeptidase	-	**Arginine and proline metabolism**; Histidine metabolism; beta-Alanine metabolism; Metabolic pathways	0.835	0.906	67.7
7.5.2.1	ABC-type maltose transporter	-		0.835	0.906	67.8
3.1.2.2	palmitoyl-CoA hydrolase	-	Fatty acid elongation; Biosynthesis of unsaturated fatty acids; Biosynthesis of secondary metabolites	0.848	0.906	61.7
4.2.1.24	porphobilinogen synthase	+	Porphyrin and chlorophyll metabolism; Metabolic pathways; Biosynthesis of secondary metabolites; Microbial metabolism in diverse environments	0.777	0.908	83.3
5.4.99.21	23S rRNA pseudouridine2604 synthase	-		0.832	0.908	70.4
4.1.1.70	glutaconyl-CoA decarboxylase	+	Benzoate degradation; Butanoate metabolism; Microbial metabolism in diverse environments	0.816	0.910	76.2
2.7.1.165	glycerate 2-kinase	+	Pentose phosphate pathway; Glycine, serine and threonine metabolism; Glycerolipid metabolism; Glyoxylate and dicarboxylate metabolism; Methane metabolism; Metabolic pathways; Microbial metabolism in diverse environments; Biosynthesis of antibiotics	0.906	0.910	*8.6
2.4.1.150	N-acetyllactosaminide beta-1,6-N-acetylglucosaminyltransferase	+	Glycosphingolipid biosynthesis - lacto and neolacto series; Metabolic pathways	0.799	0.911	80.6
1.8.5.3	respiratory dimethylsulfoxide reductase	-	Sulfur metabolism	0.804	0.912	80.3
2.4.1.58	lipopolysaccharide glucosyltransferase I	-	Lipopolysaccharide biosynthesis; Metabolic pathways	0.850	0.912	65.6
4.2.2.21	chondroitin-sulfate-ABC exolyase	-		0.883	0.912	43.3
2.7.7.27	glucose-1-phosphate adenylyltransferase	-	Starch and sucrose metabolism; Amino sugar and nucleotide sugar metabolism; Metabolic pathways; Biosynthesis of secondary metabolites	0.763	0.913	86.9
1.3.1.28	2,3-dihydro-2,3-dihydroxybenzoate dehydrogenase	-	Biosynthesis of siderophore group nonribosomal peptides; Biosynthesis of secondary metabolites; Biosynthesis of antibiotics	0.838	0.913	71.6
1.1.99.1	choline dehydrogenase	+	Glycine, serine and threonine metabolism; Metabolic pathways	0.843	0.913	69.9
4.1.1.83	4-hydroxyphenylacetate decarboxylase	+		0.762	0.914	87.2
2.4.1.1	glycogen phosphorylase	+	Starch and sucrose metabolism; Metabolic pathways; Biosynthesis of secondary metabolites	0.782	0.914	84.8
4.2.1.6	galactonate dehydratase	-	Galactose metabolism; Metabolic pathways; Microbial metabolism in diverse environments	0.823	0.914	76.4
4.2.1.81	D(-)-tartrate dehydratase	-		0.866	0.914	59.2
4.1.2.19	rhamnulose-1-phosphate aldolase	-	Pentose and glucuronate interconversions; Fructose and mannose metabolism; Microbial metabolism in diverse environments	0.819	0.915	78.0
1.1.1.284	S-(hydroxymethyl)glutathione dehydrogenase	+	Methane metabolism; Microbial metabolism in diverse environments	0.830	0.915	75.4
3.4.11.6	aminopeptidase B	+		0.760	0.917	88.3
2.7.7.85	diadenylate cyclase	+		0.829	0.917	76.6
3.6.3.29	molybdate-transporting ATPase	-		0.861	0.917	64.4
3.2.1.3	glucan 1,4-alpha-glucosidase	+	Starch and sucrose metabolism; Metabolic pathways	0.864	0.917	62.5
2.3.1.251	lipid IVA palmitoyltransferase	-		0.916	0.918	*4.9
2.5.1.141	heme o synthase	+	Porphyrin and chlorophyll metabolism; Metabolic pathways; Biosynthesis of secondary metabolites	0.841	0.920	74.6
1.18.1.3	ferredoxin---NAD+ reductase	+	Fatty acid degradation	0.853	0.922	72.4
5.1.1.20	L-Ala-D/L-Glu epimerase	-		0.871	0.922	63.4
2.7.13.3	histidine kinase	-		0.802	0.923	85.3
1.17.4.1	ribonucleoside-diphosphate reductase	+	Purine metabolism; Pyrimidine metabolism; Glutathione metabolism; Drug metabolism - other enzymes; Metabolic pathways	0.837	0.923	78.3
2.7.8.8	CDP-diacylglycerol---serine O-phosphatidyltransferase	+	Glycine, serine and threonine metabolism; Glycerophospholipid metabolism; Metabolic pathways; Biosynthesis of secondary metabolites	0.830	0.924	80.5
6.2.1.13	acetate---CoA ligase (ADP-forming)	-	Glycolysis / Gluconeogenesis; Pyruvate metabolism; Propanoate metabolism; Metabolic pathways; Microbial metabolism in diverse environments	0.863	0.925	70.5
4.1.1.47	tartronate-semialdehyde synthase	+	Glyoxylate and dicarboxylate metabolism; Metabolic pathways	0.871	0.925	66.0
2.7.7.42	[glutamine synthetase] adenylyltransferase	+		0.849	0.926	76.6
5.4.99.61	precorrin-8X methylmutase	-	Porphyrin and chlorophyll metabolism; Metabolic pathways	0.848	0.927	77.1
2.4.1.320	1,4-beta-mannosyl-N-acetylglucosamine phosphorylase	-		0.855	0.927	74.7
1.1.1.202	1,3-propanediol dehydrogenase	-	Glycerolipid metabolism; Propanoate metabolism	0.864	0.927	71.2
2.7.1.144	tagatose-6-phosphate kinase	+	Galactose metabolism; Metabolic pathways	0.829	0.928	82.4
3.2.1.85	6-phospho-beta-galactosidase	+	Galactose metabolism; Metabolic pathways	0.866	0.928	71.7
3.1.3.97	3',5'-nucleoside bisphosphate phosphatase	-		0.882	0.928	62.8
3.6.3.17	monosaccharide-transporting ATPase	+		0.828	0.929	83.0
1.11.1.21	catalase-peroxidase	-	Phenylalanine metabolism; Tryptophan metabolism; Phenylpropanoid biosynthesis; Drug metabolism - other enzymes; Biosynthesis of secondary metabolites	0.867	0.930	72.2
3.5.1.4	amidase	+	**Arginine and proline metabolism**; Phenylalanine metabolism; Tryptophan metabolism; Aminobenzoate degradation; Styrene degradation; Microbial metabolism in diverse environments	0.854	0.932	78.2
1.1.1.79	glyoxylate reductase (NADP+)	+	Pyruvate metabolism; Glyoxylate and dicarboxylate metabolism; Metabolic pathways; Biosynthesis of secondary metabolites; Microbial metabolism in diverse environments	0.850	0.933	80.5
3.5.3.23	N-succinylarginine dihydrolase	-	**Arginine and proline metabolism**; Metabolic pathways	0.866	0.933	75.2
2.7.7.58	(2,3-dihydroxybenzoyl)adenylate synthase	-	Biosynthesis of siderophore group nonribosomal peptides	0.876	0.933	70.8
1.1.1.306	S-(hydroxymethyl)mycothiol dehydrogenase	+		0.916	0.935	40.1
3.6.1.27	undecaprenyl-diphosphate phosphatase	+	Peptidoglycan biosynthesis	0.821	0.937	87.7
1.13.11.29	stizolobate synthase	-	Tyrosine metabolism	0.889	0.937	67.6
4.2.1.104	cyanase	+	Nitrogen metabolism	0.916	0.940	50.0
2.6.1.22	(S)-3-amino-2-methylpropionate transaminase	-	Valine, leucine and isoleucine degradation	0.887	0.942	74.1
4.1.3.34	citryl-CoA lyase	+	Carbon fixation pathways in prokaryotes; Metabolic pathways; Microbial metabolism in diverse environments	0.894	0.943	71.5
2.7.1.194	protein-Npi-phosphohistidine---L-ascorbate phosphotransferase	-	Ascorbate and aldarate metabolism; Metabolic pathways; Microbial metabolism in diverse environments	0.900	0.944	68.5
1.1.99.28	glucose-fructose oxidoreductase	-		0.855	0.945	86.1
3.1.1.4	phospholipase A2	+	Glycerophospholipid metabolism; Ether lipid metabolism; Arachidonic acid metabolism; Linoleic acid metabolism; alpha-Linolenic acid metabolism; Metabolic pathways; Biosynthesis of secondary metabolites	0.917	0.945	55.6
3.1.1.29	aminoacyl-tRNA hydrolase	+		0.847	0.946	87.9
1.3.99.4	3-oxosteroid 1-dehydrogenase	-	Steroid degradation; Metabolic pathways; Microbial metabolism in diverse environments	0.895	0.947	74.9
5.4.99.29	23S rRNA pseudouridine746 synthase	+		0.899	0.947	72.5
3.4.23.49	omptin	+		0.914	0.947	61.5
3.5.4.4	adenosine deaminase	-	Purine metabolism; Metabolic pathways	0.861	0.948	86.2
1.1.5.3	glycerol-3-phosphate dehydrogenase	-	Glycerophospholipid metabolism; Biosynthesis of secondary metabolites	0.888	0.948	78.9
1.17.7.1	(E)-4-hydroxy-3-methylbut-2-enyl-diphosphate synthase (ferredoxin)	+	Terpenoid backbone biosynthesis; Metabolic pathways; Biosynthesis of secondary metabolites; Biosynthesis of antibiotics	0.896	0.951	77.8
3.1.1.85	pimelyl-[acyl-carrier protein] methyl ester esterase	+	**Biotin metabolism**; Metabolic pathways	0.918	0.951	64.4
2.7.11.5	[isocitrate dehydrogenase (NADP+)] kinase	-		0.920	0.952	63.8
6.5.1.4	RNA 3'-terminal-phosphate cyclase (ATP)	+		0.927	0.952	55.8
1.1.1.251	galactitol-1-phosphate 5-dehydrogenase	-	Galactose metabolism; Metabolic pathways	0.955	0.955	*0.0
1.1.5.4	malate dehydrogenase (quinone)	-	Citrate cycle (TCA cycle); Pyruvate metabolism; Metabolic pathways; Biosynthesis of secondary metabolites; Microbial metabolism in diverse environments; Biosynthesis of antibiotics	0.912	0.956	75.0
2.4.1.11	glycogen(starch) synthase	+	Starch and sucrose metabolism; Metabolic pathways	0.945	0.956	*35.7
4.2.2.22	pectate trisaccharide-lyase	-		0.956	0.956	*0.0
7.2.2.8	P-type Cu+ transporter	+		0.908	0.957	77.7
2.1.3.2	aspartate carbamoyltransferase	+	Pyrimidine metabolism; Alanine, aspartate and glutamate metabolism; Metabolic pathways	0.925	0.957	67.9
1.1.1.373	sulfolactaldehyde 3-reductase	-		0.932	0.958	61.2
3.1.11.5	exodeoxyribonuclease V	+		0.929	0.959	67.1
3.1.1.24	3-oxoadipate enol-lactonase	+	Benzoate degradation; Metabolic pathways; Microbial metabolism in diverse environments	0.927	0.960	70.2
3.6.3.4	Cu2+-exporting ATPase	-		0.929	0.960	67.9
1.3.1.31	2-enoate reductase	+	Phenylalanine metabolism; Microbial metabolism in diverse environments	0.939	0.960	57.4
1.1.1.61	4-hydroxybutyrate dehydrogenase	+	Butanoate metabolism; Metabolic pathways	0.960	0.960	*0.0
4.1.1.17	ornithine decarboxylase	-	**Arginine and proline metabolism**; Glutathione metabolism; Metabolic pathways; Biosynthesis of secondary metabolites; Biosynthesis of antibiotics	0.898	0.962	86.2
2.7.7.48	RNA-directed RNA polymerase	+		0.941	0.962	58.9
2.4.99.13	(Kdo)-lipid IVA 3-deoxy-D-manno-octulosonic acid transferase	-	Lipopolysaccharide biosynthesis; Metabolic pathways	0.949	0.962	43.7
1.1.5.12	D-lactate dehydrogenase (quinone)	-	Pyruvate metabolism	0.938	0.964	66.9
2.4.1.12	cellulose synthase (UDP-forming)	+	Starch and sucrose metabolism; Metabolic pathways	0.946	0.964	55.1
3.6.1.15	nucleoside-triphosphate phosphatase	-	Purine metabolism; Thiamine metabolism; Metabolic pathways	0.964	0.965	*7.0
4.4.1.15	D-cysteine desulfhydrase	+	Cysteine and methionine metabolism	0.920	0.966	81.8
2.4.1.15	alpha,alpha-trehalose-phosphate synthase (UDP-forming)	-	Starch and sucrose metabolism; Metabolic pathways	0.936	0.966	72.8
3.5.3.9	allantoate deiminase	-	Purine metabolism; Microbial metabolism in diverse environments	0.938	0.967	71.6
1.1.1.21	aldose reductase	+	Pentose and glucuronate interconversions; Fructose and mannose metabolism; Galactose metabolism; Glycerolipid metabolism; **Folate biosynthesis**; Metabolic pathways	0.941	0.967	68.8
2.7.1.45	2-dehydro-3-deoxygluconokinase	+	Pentose phosphate pathway; Metabolic pathways; Microbial metabolism in diverse environments	0.961	0.969	*38.1
7.2.4.1	carboxybiotin decarboxylase	-		0.947	0.970	68.5
5.4.99.24	23S rRNA pseudouridine955/2504/2580 synthase	-		0.949	0.970	65.5
2.1.1.178	16S rRNA (cytosine1407-C5)-methyltransferase	+		0.950	0.970	64.6
2.7.7.49	RNA-directed DNA polymerase	-		0.950	0.970	64.8
3.2.1.4	cellulase	+	Starch and sucrose metabolism; Metabolic pathways	0.929	0.971	83.5
1.21.4.1	D-proline reductase	-	**Arginine and proline metabolism**	0.954	0.971	60.1
4.1.1.33	diphosphomevalonate decarboxylase	+	Terpenoid backbone biosynthesis; Metabolic pathways; Biosynthesis of secondary metabolites; Biosynthesis of antibiotics	0.940	0.972	79.1
2.5.1.48	cystathionine gamma-synthase	-	Cysteine and methionine metabolism; Selenocompound metabolism; Sulfur metabolism; Metabolic pathways; Biosynthesis of secondary metabolites; Biosynthesis of antibiotics	0.948	0.972	70.2
4.1.2.57	sulfofructosephosphate aldolase	-		0.962	0.972	45.5
3.6.3.14	H+-transporting two-sector ATPase	-	Oxidative phosphorylation; Photosynthesis; Metabolic pathways	0.917	0.973	89.5
2.7.1.197	protein-Npi-phosphohistidine---D-mannitol phosphotransferase	-	Fructose and mannose metabolism	0.946	0.974	77.7
2.7.1.33	pantothenate kinase	+	Pantothenate and CoA biosynthesis; Metabolic pathways	0.948	0.974	73.7
7.1.1.7	quinol oxidase (electrogenic, proton-motive force generating)	+		0.953	0.974	69.9
3.5.4.33	tRNA(adenine34) deaminase	-		0.940	0.976	84.3
1.1.1.100	3-oxoacyl-[acyl-carrier-protein] reductase	-	Fatty acid biosynthesis; **Biotin metabolism**; Biosynthesis of unsaturated fatty acids; Metabolic pathways	0.950	0.976	77.3
4.1.3.1	isocitrate lyase	-	Glyoxylate and dicarboxylate metabolism; Metabolic pathways; Biosynthesis of secondary metabolites; Microbial metabolism in diverse environments	0.958	0.976	67.3
3.1.3.89	5'-deoxynucleotidase	+	Pyrimidine metabolism; Metabolic pathways	0.957	0.977	72.3
2.4.1.20	cellobiose phosphorylase	+	Starch and sucrose metabolism; Metabolic pathways	0.961	0.978	66.7
3.6.3.33	vitamin B12-transporting ATPase	-		0.953	0.979	79.4
2.7.1.73	inosine kinase	+	Purine metabolism	0.963	0.981	72.4
1.14.99.46	pyrimidine oxygenase	-	Pyrimidine metabolism; Metabolic pathways	0.966	0.981	69.6

3.1.11.6	exodeoxyribonuclease VII	+		0.979	0.981	*17.8
3.5.3.11	agmatinase	-	**Arginine and proline metabolism**; Metabolic pathways	0.956	0.983	85.7
4.1.2.53	2-keto-3-deoxy-L-rhamnonate aldolase	+	Fructose and mannose metabolism; Microbial metabolism in diverse environments	0.965	0.983	76.5
1.14.13.127	3-(3-hydroxyphenyl)propanoate hydroxylase	-	Phenylalanine metabolism; Microbial metabolism in diverse environments	0.970	0.983	67.3
1.6.6.9	trimethylamine-N-oxide reductase	-	Methane metabolism; Microbial metabolism in diverse environments	0.977	0.983	45.6
1.13.11.54	acireductone dioxygenase [iron(II)-requiring]	-	Cysteine and methionine metabolism; Metabolic pathways	0.978	0.986	63.4
3.1.1.61	protein-glutamate methylesterase	+		0.986	0.986	*0.10
3.1.4.14	[acyl-carrier-protein] phosphodiesterase	-	Pantothenate and CoA biosynthesis	0.978	0.987	63.6
4.2.1.79	2-methylcitrate dehydratase	-	Propanoate metabolism	0.980	0.988	61.1
1.8.1.2	assimilatory sulfite reductase (NADPH)	+	Sulfur metabolism; Metabolic pathways; Microbial metabolism in diverse environments	0.982	0.990	68.9
1.1.1.95	phosphoglycerate dehydrogenase	+	Glycine, serine and threonine metabolism; Methane metabolism; Metabolic pathways; Microbial metabolism in diverse environments; Biosynthesis of antibiotics	0.991	0.991	*0.0
2.7.1.2	glucokinase	-	Glycolysis / Gluconeogenesis; Galactose metabolism; Starch and sucrose metabolism; Amino sugar and nucleotide sugar metabolism; Streptomycin biosynthesis; Neomycin, kanamycin and gentamicin biosynthesis; Metabolic pathways; Biosynthesis of secondary metabolites; Microbial metabolism in diverse environments; Biosynthesis of antibiotics	0.983	0.992	75.4
1.8.4.8	phosphoadenylyl-sulfate reductase (thioredoxin)	+	Sulfur metabolism; Metabolic pathways; Microbial metabolism in diverse environments	0.985	0.992	71.9
2.8.1.7	cysteine desulfurase	-	Thiamine metabolism; Metabolic pathways	0.978	0.993	89.0
4.2.1.126	N-acetylmuramic acid 6-phosphate etherase	-	Amino sugar and nucleotide sugar metabolism; Metabolic pathways	0.992	0.993	*25.9
6.3.2.13	UDP-N-acetylmuramoyl-L-alanyl-D-glutamate---2,6-diaminopimelate ligase	-	Lysine biosynthesis; Peptidoglycan biosynthesis	0.982	0.994	88.1
2.3.1.41	beta-ketoacyl-[acyl-carrier-protein] synthase I	-	Fatty acid biosynthesis; **Biotin metabolism**; Metabolic pathways	0.991	0.995	68.6
3.5.2.14	N-methylhydantoinase (ATP-hydrolysing)	-	**Arginine and proline metabolism**; Metabolic pathways	0.992	0.995	49.6
1.14.11.17	taurine dioxygenase	-	Taurine and hypotaurine metabolism; Sulfur metabolism	0.993	0.996	69.5
2.8.1.1	thiosulfate sulfurtransferase	-	Sulfur metabolism; Microbial metabolism in diverse environments	0.994	0.997	83.0
2.8.3.6	3-oxoadipate CoA-transferase	+	Benzoate degradation; Metabolic pathways; Microbial metabolism in diverse environments	0.998	0.999	67.5
3.4.24.70	oligopeptidase A	-		0.999	0.999	56.5
3.1.21.1	deoxyribonuclease I	+		0.999	1.000	68.1

[a]Significantly changed EC numbers and enzymes are shown in bold (* in FEM, REM, and I^2).

[b]"Riboflavin metabolism", "Biotin metabolism", "Folate metabolism", and "Arginine and proline metabolism" are shown in bold.

[c]**p*-value (FEM, REM) < 2.97E-05 (*p*-value < 0.05 before Bonferroni correction); *I^2 < 40%

Supplementary Table 4. KEGG pathways upregulated and downregulated in PD by hypergeometric test

Pathway	*p*-value	*q*-value
**Upregulated in PD**		
Biosynthesis of enediyne antibiotics	0.044	0.845
Lysine degradation	0.183	0.845
Aminobenzoate degradation	0.200	0.845
Chlorocyclohexane and chlorobenzene degradation	0.200	0.845
Ubiquinone and other terpenoid-quinone biosynthesis	0.215	0.845
Tyrosine metabolism	0.215	0.845
Atrazine degradation	0.235	0.845
Teichoic acid biosynthesis	0.300	0.845
C5-Branched dibasic acid metabolism	0.300	0.845
Chloroalkane and chloroalkene degradation	0.300	0.845
Caprolactam degradation	0.300	0.845
Streptomycin biosynthesis	0.360	0.911
Methane metabolism	0.425	0.911
Carbon fixation pathways in prokaryotes	0.440	0.911
Tryptophan metabolism	0.465	0.911
Butanoate metabolism	0.470	0.911
Cysteine and methionine metabolism	0.567	0.965
D-Amino acid metabolism	0.572	0.965
Citrate cycle (TCA cycle)	0.591	0.965
Aminoacyl-tRNA biosynthesis	0.674	0.995
Galactose metabolism	0.674	0.995
Pentose phosphate pathway	0.762	1.000
O-Antigen nucleotide sugar biosynthesis	0.835	1.000
Glyoxylate and dicarboxylate metabolism	0.842	1.000
Starch and sucrose metabolism	0.874	1.000
Pyrimidine metabolism	0.891	1.000
Microbial metabolism in diverse environments	0.947	1.000
Purine metabolism	0.952	1.000
Amino sugar and nucleotide sugar metabolism	0.958	1.000
Biosynthesis of secondary metabolites	0.999	1.000
Metabolic pathways	1.000	1.000
**Downregulated in PD**		
**Riboflavin metabolism**	5.58E-04	0.0170
**Biotin metabolism**	1.75E-03	0.0260
Pantothenate and CoA biosynthesis	0.0530	0.529
Glycosphingolipid biosynthesis - ganglio series	0.0850	0.549
Folate metabolism	0.0920	0.549
beta-Alanine metabolism	0.136	0.681
Pentose and glucuronate interconversions	0.193	0.749
Glycosaminoglycan degradation	0.200	0.749

Galactose metabolism	0.297	0.900
Other glycan degradation	0.300	0.900
Sphingolipid metabolism	0.331	0.900
Valine, leucine and isoleucine biosynthesis	0.360	0.900
Fatty acid biosynthesis	0.440	0.910
Arginine and proline metabolism	0.554	0.910
Nitrogen metabolism	0.591	0.910
Thiamine metabolism	0.591	0.910
Amino sugar and nucleotide sugar metabolism	0.597	0.910
Lysine biosynthesis	0.609	0.910
Phenylalanine, tyrosine and tryptophan biosynthesis	0.643	0.910
Terpenoid backbone biosynthesis	0.643	0.910
Glycerophospholipid metabolism	0.659	0.910
Nicotinate and nicotinamide metabolism	0.702	0.910
Biosynthesis of secondary metabolites	0.709	0.910
Propanoate metabolism	0.773	0.910
Glycine, serine and threonine metabolism	0.827	0.910
O-Antigen nucleotide sugar biosynthesis	0.835	0.910
Glyoxylate and dicarboxylate metabolism	0.842	0.910
Fructose and mannose metabolism	0.849	0.910
Metabolic pathways	0.998	1.000
Microbial metabolism in diverse environments	1.000	1.000

Thirty-one EC numbers that were significantly increased in PD were included in 31 KEGG pathways. Forty-two EC numbers that were significantly decreased in PD were included in 30 KEGG pathways. False discovery rates (q -values) were calculated for the 31 and 30 KEGG pathways, respectively, with the Benjamini-Hochberg method.

Bold letters represent significantly downregulated pathways ($q < 0.05$).

Supplementary Table 5. Significantly changed KEGG pathways by GSEA

Pathway	*p* -value	*q* -value
**Upregulated in PD**		
Methane metabolism	6.36E-11	3.50E-09
Aminoacyl-tRNA biosynthesis	1.96E-04	5.40E-03
**Downregulated in PD**		
Biotin metabolism	5.60E-05	2.67E-03
Riboflavin metabolism	8.47E-05	2.67E-03
Folate metabolism	2.57E-04	3.96E-03
Nitrogen metabolism	2.62E-04	3.96E-03
Fatty acid biosynthesis	3.14E-04	3.96E-03
Arginine and proline metabolism	2.02E-03	0.0212

KEGG pathways that were increased or decreased only in one or two out of six datasets were removed from GESA analysis. After the filtration, 55 and 63 KEGG pathways were increased and decreased, respectively, in PD. False discovery rates (q-values) were calculated for the 55 and 63 KEGG pathways, respectively, with the Benjamini-Hochberg method.

Supplementary Table 6. Confounding factor analysis of EC numbers that were significantly changed in meta-analysis of six datasets

EC number	Enzyme	Confounding factors	Beta coefficient ± SE	Increase or decrease	*p* -value	*q* -value
1.1.1.163	cyclopentanol dehydrogenase	age	-0.093 ± 0.067	-	0.165	
1.1.1.163	cyclopentanol dehydrogenase	BMI	-0.006 ± 0.062	-	0.924	
1.1.1.163	cyclopentanol dehydrogenase	constipation	0.070 ± 0.064	+	0.270	
1.1.1.163	cyclopentanol dehydrogenase	disease	0.559 ± 0.150	+	1.91E-04	*6.34E-04
1.1.1.163	cyclopentanol dehydrogenase	sex	-0.062 ± 0.064	-	0.337	
1.1.1.163	cyclopentanol dehydrogenase	anticholesterol drugs	0.182 ± 0.065	+	4.93E-03	
1.1.1.193	5-amino-6-(5-phosphoribosylamino)uracil reductase	age	-0.020 ± 0.009	-	0.0329	
1.1.1.193	5-amino-6-(5-phosphoribosylamino)uracil reductase	BMI	0.013 ± 0.010	+	0.185	
1.1.1.193	5-amino-6-(5-phosphoribosylamino)uracil reductase	constipation	-0.049 ± 0.010	-	5.03E-07	
**1.1.1.193**	**5-amino-6-(5-phosphoribosylamino)uracil reductase**	**disease**	**-0.089 ± 0.020**	**-**	**1.16E-05**	***8.12E-05**
1.1.1.193	5-amino-6-(5-phosphoribosylamino)uracil reductase	sex	-0.032 ± 0.009	-	5.91E-04	
1.1.1.193	5-amino-6-(5-phosphoribosylamino)uracil reductase	anticholesterol drugs	0.003 ± 0.010	+	0.786	
1.1.1.272	D-2-hydroxyacid dehydrogenase (NADP+)	age	-0.037 ± 0.039	-	0.345	
1.1.1.272	D-2-hydroxyacid dehydrogenase (NADP+)	BMI	-0.114 ± 0.041	-	5.24E-03	
1.1.1.272	D-2-hydroxyacid dehydrogenase (NADP+)	constipation	-0.056 ± 0.041	-	0.169	
1.1.1.272	D-2-hydroxyacid dehydrogenase (NADP+)	disease	0.095 ± 0.088	+	0.284	0.320
1.1.1.272	D-2-hydroxyacid dehydrogenase (NADP+)	sex	-0.084 ± 0.040	-	0.0363	
1.1.1.272	D-2-hydroxyacid dehydrogenase (NADP+)	anticholesterol drugs	0.016 ± 0.040	+	0.689	
1.1.1.57	fructuronate reductase	age	0.011 ± 0.029	+	0.713	
1.1.1.57	fructuronate reductase	BMI	0.042 ± 0.029	+	0.144	
1.1.1.57	fructuronate reductase	constipation	-0.096 ± 0.030	-	1.34E-03	
1.1.1.57	fructuronate reductase	disease	-0.201 ± 0.062	-	1.28E-03	*2.99E-03
1.1.1.57	fructuronate reductase	sex	-0.073 ± 0.029	-	0.0118	
1.1.1.57	fructuronate reductase	anticholesterol drugs	0.038 ± 0.029	+	0.191	
1.1.1.77	lactaldehyde reductase	age	NA	NA	NA	
1.1.1.77	lactaldehyde reductase	BMI	NA	NA	NA	
1.1.1.77	lactaldehyde reductase	constipation	NA	NA	NA	
1.1.1.77	lactaldehyde reductase	disease	NA	NA	NA	
1.1.1.77	lactaldehyde reductase	sex	NA	NA	NA	
1.1.1.77	lactaldehyde reductase	anticholesterol drugs	NA	NA	NA	
1.12.1.2	hydrogen dehydrogenase	age	0.055 ± 0.055	+	0.319	
1.12.1.2	hydrogen dehydrogenase	BMI	-0.048 ± 0.051	-	0.351	
1.12.1.2	hydrogen dehydrogenase	constipation	0.108 ± 0.050	+	0.0313	
1.12.1.2	hydrogen dehydrogenase	disease	0.284 ± 0.117	+	0.0149	*0.0254
1.12.1.2	hydrogen dehydrogenase	sex	0.066 ± 0.053	+	0.215	
1.12.1.2	hydrogen dehydrogenase	anticholesterol drugs	0.169 ± 0.051	+	9.44E-04	
1.12.7.2	ferredoxin hydrogenase	age	0.010 ± 0.013	+	0.472	
1.12.7.2	ferredoxin hydrogenase	BMI	0.003 ± 0.014	+	0.846	
1.12.7.2	ferredoxin hydrogenase	constipation	-0.032 ± 0.013	-	0.0175	
1.12.7.2	ferredoxin hydrogenase	disease	-0.070 ± 0.029	-	0.0152	*0.0254
1.12.7.2	ferredoxin hydrogenase	sex	0.045 ± 0.013	+	6.16E-04	
1.12.7.2	ferredoxin hydrogenase	anticholesterol drugs	0.019 ± 0.013	+	0.153	
1.18.1.6	adrenodoxin-NADP+ reductase	age	-0.141 ± 0.060	-	0.0197	
1.18.1.6	adrenodoxin-NADP+ reductase	BMI	-0.212 ± 0.064	-	8.65E-04	
1.18.1.6	adrenodoxin-NADP+ reductase	constipation	0.233 ± 0.057	+	4.51E-05	
1.18.1.6	adrenodoxin-NADP+ reductase	disease	0.467 ± 0.133	+	4.61E-04	*1.24E-03
1.18.1.6	adrenodoxin-NADP+ reductase	sex	0.043 ± 0.057	+	0.449	
1.18.1.6	adrenodoxin-NADP+ reductase	anticholesterol drugs	-0.057 ± 0.060	-	0.344	
1.2.7.3	2-oxoglutarate synthase	age	0.046 ± 0.047	+	0.327	
1.2.7.3	2-oxoglutarate synthase	BMI	-0.034 ± 0.053	-	0.519	
1.2.7.3	2-oxoglutarate synthase	constipation	0.052 ± 0.048	+	0.279	
1.2.7.3	2-oxoglutarate synthase	disease	-0.164 ± 0.106	-	0.124	0.150
1.2.7.3	2-oxoglutarate synthase	sex	-0.057 ± 0.050	-	0.251	
1.2.7.3	2-oxoglutarate synthase	anticholesterol drugs	-0.022 ± 0.049	-	0.651	
1.21.98.1	cyclic dehypoxanthinyl futalosine synthase	age	0.121 ± 0.049	+	0.0131	
1.21.98.1	cyclic dehypoxanthinyl futalosine synthase	BMI	-0.134 ± 0.052	-	0.0101	
1.21.98.1	cyclic dehypoxanthinyl futalosine synthase	constipation	0.166 ± 0.046	+	2.72E-04	
1.21.98.1	cyclic dehypoxanthinyl futalosine synthase	disease	0.092 ± 0.105	+	0.378	0.413
1.21.98.1	cyclic dehypoxanthinyl futalosine synthase	sex	0.023 ± 0.048	+	0.637	
1.21.98.1	cyclic dehypoxanthinyl futalosine synthase	anticholesterol drugs	0.026 ± 0.047	+	0.584	
1.7.1.13	preQ1 synthase	age	-0.014 ± 0.015	-	0.377	
1.7.1.13	preQ1 synthase	BMI	0.041 ± 0.017	+	0.0168	
1.7.1.13	preQ1 synthase	constipation	-0.035 ± 0.015	-	0.0189	
1.7.1.13	preQ1 synthase	disease	-0.097 ± 0.033	-	2.92E-03	*6.01E-03
1.7.1.13	preQ1 synthase	sex	-0.039 ± 0.015	-	7.66E-03	
1.7.1.13	preQ1 synthase	anticholesterol drugs	-0.027 ± 0.015	-	0.0779	
1.7.7.1	ferredoxin---nitrite reductase	age	-0.071 ± 0.062	-	0.252	
1.7.7.1	ferredoxin---nitrite reductase	BMI	0.031 ± 0.064	+	0.629	

1.7.7.1	ferredoxin---nitrite reductase	constipation	-0.433 ± 0.077	-	1.89E-08	
1.7.7.1	ferredoxin---nitrite reductase	disease	-0.286 ± 0.132	-	0.0303	*0.0451
1.7.7.1	ferredoxin---nitrite reductase	sex	-0.116 ± 0.064	-	0.0718	
1.7.7.1	ferredoxin---nitrite reductase	anticholesterol drugs	-0.169 ± 0.067	-	0.0116	
1.8.4.12	peptide-methionine (R)-S-oxide reductase	age	0.055 ± 0.019	+	3.52E-03	
1.8.4.12	peptide-methionine (R)-S-oxide reductase	BMI	0.000 ± 0.020	+	0.990	
1.8.4.12	peptide-methionine (R)-S-oxide reductase	constipation	0.089 ± 0.019	+	1.52E-06	
1.8.4.12	peptide-methionine (R)-S-oxide reductase	disease	0.223 ± 0.040	+	2.76E-08	*6.67E-07
1.8.4.12	peptide-methionine (R)-S-oxide reductase	sex	0.026 ± 0.018	+	0.163	
1.8.4.12	peptide-methionine (R)-S-oxide reductase	anticholesterol drugs	0.012 ± 0.019	+	0.507	
2.1.1.156	glycine/sarcosine N-methyltransferase	age	-0.024 ± 0.023	-	0.290	
2.1.1.156	glycine/sarcosine N-methyltransferase	BMI	0.014 ± 0.022	+	0.519	
2.1.1.156	glycine/sarcosine N-methyltransferase	constipation	-0.085 ± 0.023	-	2.71E-04	
2.1.1.156	glycine/sarcosine N-methyltransferase	disease	-0.184 ± 0.048	-	1.48E-04	*5.17E-04
2.1.1.156	glycine/sarcosine N-methyltransferase	sex	-0.014 ± 0.022	-	0.528	
2.1.1.156	glycine/sarcosine N-methyltransferase	anticholesterol drugs	0.039 ± 0.023	+	0.084	
2.1.1.197	malonyl-[acyl-carrier protein] O-methyltransferase	age	0.006 ± 0.014	+	0.677	
2.1.1.197	malonyl-[acyl-carrier protein] O-methyltransferase	BMI	0.015 ± 0.015	+	0.298	
2.1.1.197	malonyl-[acyl-carrier protein] O-methyltransferase	constipation	-0.048 ± 0.014	-	5.90E-04	
**2.1.1.197**	**malonyl-[acyl-carrier protein] O-methyltransferase**	**disease**	**-0.077 ± 0.030**	**-**	**9.36E-03**	***0.0164**
2.1.1.197	malonyl-[acyl-carrier protein] O-methyltransferase	sex	0.015 ± 0.014	+	0.264	
2.1.1.197	malonyl-[acyl-carrier protein] O-methyltransferase	anticholesterol drugs	0.022 ± 0.014	+	0.113	
2.1.1.223	tRNA1Val (adenine37-N6)-methyltransferase	age	-0.001 ± 0.011	-	0.899	
2.1.1.223	tRNA1Val (adenine37-N6)-methyltransferase	BMI	0.007 ± 0.011	+	0.537	
2.1.1.223	tRNA1Val (adenine37-N6)-methyltransferase	constipation	-0.013 ± 0.011	-	0.233	
2.1.1.223	tRNA1Val (adenine37-N6)-methyltransferase	disease	-0.095 ± 0.024	-	6.34E-05	*2.77E-04
2.1.1.223	tRNA1Val (adenine37-N6)-methyltransferase	sex	-0.003 ± 0.011	-	0.766	
2.1.1.223	tRNA1Val (adenine37-N6)-methyltransferase	anticholesterol drugs	0.013 ± 0.011	+	0.225	
2.1.2.11	3-methyl-2-oxobutanoate hydroxymethyltransferase	age	-0.002 ± 0.009	-	0.811	
2.1.2.11	3-methyl-2-oxobutanoate hydroxymethyltransferase	BMI	-0.003 ± 0.010	-	0.766	
2.1.2.11	3-methyl-2-oxobutanoate hydroxymethyltransferase	constipation	0.003 ± 0.009	+	0.736	
2.1.2.11	3-methyl-2-oxobutanoate hydroxymethyltransferase	disease	-0.055 ± 0.020	-	7.04E-03	*0.0130
2.1.2.11	3-methyl-2-oxobutanoate hydroxymethyltransferase	sex	-0.020 ± 0.009	-	0.0307	
2.1.2.11	3-methyl-2-oxobutanoate hydroxymethyltransferase	anticholesterol drugs	0.005 ± 0.009	+	0.614	
2.3.2.6	lysine/arginine leucyltransferase	age	0.028 ± 0.020	+	0.151	
2.3.2.6	lysine/arginine leucyltransferase	BMI	0.036 ± 0.021	+	0.0790	
2.3.2.6	lysine/arginine leucyltransferase	constipation	-0.011 ± 0.020	-	0.581	
2.3.2.6	lysine/arginine leucyltransferase	disease	-0.034 ± 0.043	-	0.421	0.446
2.3.2.6	lysine/arginine leucyltransferase	sex	-0.024 ± 0.019	-	0.216	
2.3.2.6	lysine/arginine leucyltransferase	anticholesterol drugs	-0.003 ± 0.020	-	0.877	
2.4.1.281	4-O-beta-D-mannosyl-D-glucose phosphorylase	age	-0.035 ± 0.016	-	0.0294	
2.4.1.281	4-O-beta-D-mannosyl-D-glucose phosphorylase	BMI	0.012 ± 0.017	+	0.488	
2.4.1.281	4-O-beta-D-mannosyl-D-glucose phosphorylase	constipation	-0.096 ± 0.017	-	7.33E-09	
2.4.1.281	4-O-beta-D-mannosyl-D-glucose phosphorylase	disease	-0.152 ± 0.034	-	8.46E-06	*6.58E-05
2.4.1.281	4-O-beta-D-mannosyl-D-glucose phosphorylase	sex	-0.011 ± 0.016	-	0.478	
2.4.1.281	4-O-beta-D-mannosyl-D-glucose phosphorylase	anticholesterol drugs	-0.012 ± 0.016	-	0.471	
2.4.1.293	GalNAc5-diNAcBac-PP-undecaprenol beta-1,3-glucosyltransferase	age	0.172 ± 0.067	+	0.0104	
2.4.1.293	GalNAc5-diNAcBac-PP-undecaprenol beta-1,3-glucosyltransferase	BMI	-0.056 ± 0.067	-	0.402	
2.4.1.293	GalNAc5-diNAcBac-PP-undecaprenol beta-1,3-glucosyltransferase	constipation	-0.063 ± 0.062	-	0.307	
2.4.1.293	GalNAc5-diNAcBac-PP-undecaprenol beta-1,3-glucosyltransferase	disease	0.192 ± 0.137	+	0.159	0.186
2.4.1.293	GalNAc5-diNAcBac-PP-undecaprenol beta-1,3-glucosyltransferase	sex	0.120 ± 0.063	+	0.0557	
2.4.1.293	GalNAc5-diNAcBac-PP-undecaprenol beta-1,3-glucosyltransferase	anticholesterol drugs	0.104 ± 0.061	+	0.0889	
2.4.1.305	ha-D-GlcNAc-glucosaminyl-diphosphoundecaprenol beta-1,3-gluco	age	0.211 ± 0.090	+	0.0194	
2.4.1.305	ha-D-GlcNAc-glucosaminyl-diphosphoundecaprenol beta-1,3-gluco	BMI	-0.148 ± 0.111	-	0.184	
2.4.1.305	ha-D-GlcNAc-glucosaminyl-diphosphoundecaprenol beta-1,3-gluco	constipation	0.085 ± 0.087	+	0.331	
2.4.1.305	ha-D-GlcNAc-glucosaminyl-diphosphoundecaprenol beta-1,3-gluco	disease	0.159 ± 0.189	+	0.399	0.430
2.4.1.305	ha-D-GlcNAc-glucosaminyl-diphosphoundecaprenol beta-1,3-gluco	sex	0.098 ± 0.089	+	0.267	
2.4.1.305	ha-D-GlcNAc-glucosaminyl-diphosphoundecaprenol beta-1,3-gluco	anticholesterol drugs	0.015 ± 0.090	+	0.864	
2.5.1.10	(2E,6E)-farnesyl diphosphate synthase	age	0.012 ± 0.014	+	0.404	
2.5.1.10	(2E,6E)-farnesyl diphosphate synthase	BMI	0.020 ± 0.014	+	0.146	
2.5.1.10	(2E,6E)-farnesyl diphosphate synthase	constipation	-0.019 ± 0.014	-	0.170	
2.5.1.10	(2E,6E)-farnesyl diphosphate synthase	disease	-0.086 ± 0.030	-	4.22E-03	*8.44E-03
2.5.1.10	(2E,6E)-farnesyl diphosphate synthase	sex	0.024 ± 0.014	+	0.0852	
2.5.1.10	(2E,6E)-farnesyl diphosphate synthase	anticholesterol drugs	0.018 ± 0.014	+	0.199	
2.5.1.78	6,7-dimethyl-8-ribityllumazine synthase	age	-0.002 ± 0.008	-	0.821	
2.5.1.78	6,7-dimethyl-8-ribityllumazine synthase	BMI	0.006 ± 0.008	+	0.469	
2.5.1.78	6,7-dimethyl-8-ribityllumazine synthase	constipation	-0.011 ± 0.009	-	0.214	
**2.5.1.78**	**6,7-dimethyl-8-ribityllumazine synthase**	**disease**	**-0.078 ± 0.018**	**-**	**1.90E-05**	***1.11E-04**
2.5.1.78	6,7-dimethyl-8-ribityllumazine synthase	sex	-0.033 ± 0.008	-	6.38E-05	
2.5.1.78	6,7-dimethyl-8-ribityllumazine synthase	anticholesterol drugs	0.005 ± 0.008	+	0.549	
2.6.1.50	glutamine---scyllo-inositol transaminase	age	-0.041 ± 0.065	-	0.532	

2.6.1.50	glutamine---scyllo-inositol transaminase	BMI	-0.151 ± 0.068	-	0.0278	
2.6.1.50	glutamine---scyllo-inositol transaminase	constipation	0.072 ± 0.060	+	0.233	
2.6.1.50	glutamine---scyllo-inositol transaminase	disease	0.367 ± 0.141	+	9.38E-03	*0.0164
2.6.1.50	glutamine---scyllo-inositol transaminase	sex	-0.001 ± 0.062	-	0.988	
2.6.1.50	glutamine---scyllo-inositol transaminase	anticholesterol drugs	0.150 ± 0.061	+	0.0134	
2.6.1.62	adenosylmethionine---8-amino-7-oxononanoate transaminase	age	0.007 ± 0.014	+	0.625	
2.6.1.62	adenosylmethionine---8-amino-7-oxononanoate transaminase	BMI	0.017 ± 0.015	+	0.258	
2.6.1.62	adenosylmethionine---8-amino-7-oxononanoate transaminase	constipation	-0.013 ± 0.014	-	0.371	
**2.6.1.62**	**adenosylmethionine---8-amino-7-oxononanoate transaminase**	**disease**	**-0.066 ± 0.031**	**-**	**0.0327**	***0.0477**
2.6.1.62	adenosylmethionine---8-amino-7-oxononanoate transaminase	sex	0.011 ± 0.014	+	0.454	
2.6.1.62	adenosylmethionine---8-amino-7-oxononanoate transaminase	anticholesterol drugs	0.017 ± 0.014	+	0.220	
2.6.1.83	LL-diaminopimelate aminotransferase	age	-0.025 ± 0.009	-	9.10E-03	
2.6.1.83	LL-diaminopimelate aminotransferase	BMI	0.010 ± 0.010	+	0.339	
2.6.1.83	LL-diaminopimelate aminotransferase	constipation	-0.046 ± 0.010	-	1.81E-06	
2.6.1.83	LL-diaminopimelate aminotransferase	disease	-0.131 ± 0.020	-	1.03E-10	*7.20E-09
2.6.1.83	LL-diaminopimelate aminotransferase	sex	-0.031 ± 0.009	-	9.92E-04	
2.6.1.83	LL-diaminopimelate aminotransferase	anticholesterol drugs	0.007 ± 0.010	+	0.482	
2.7.1.22	ribosylnicotinamide kinase	age	-0.009 ± 0.038	-	0.816	
2.7.1.22	ribosylnicotinamide kinase	BMI	0.101 ± 0.039	+	9.55E-03	
2.7.1.22	ribosylnicotinamide kinase	constipation	-0.041 ± 0.038	-	0.273	
2.7.1.22	ribosylnicotinamide kinase	disease	-0.133 ± 0.081	-	0.100	0.125
2.7.1.22	ribosylnicotinamide kinase	sex	-0.001 ± 0.037	-	0.973	
2.7.1.22	ribosylnicotinamide kinase	anticholesterol drugs	0.029 ± 0.037	+	0.432	
2.7.1.5	rhamnulokinase	age	0.027 ± 0.013	+	0.0377	
2.7.1.5	rhamnulokinase	BMI	-0.001 ± 0.014	-	0.963	
2.7.1.5	rhamnulokinase	constipation	-0.036 ± 0.013	-	6.71E-03	
2.7.1.5	rhamnulokinase	disease	-0.085 ± 0.028	-	2.58E-03	*5.56E-03
2.7.1.5	rhamnulokinase	sex	0.042 ± 0.013	+	1.21E-03	
2.7.1.5	rhamnulokinase	anticholesterol drugs	0.007 ± 0.013	+	0.608	
2.7.6.2	thiamine diphosphokinase	age	-0.024 ± 0.013	-	0.0679	
2.7.6.2	thiamine diphosphokinase	BMI	0.004 ± 0.013	+	0.766	
2.7.6.2	thiamine diphosphokinase	constipation	-0.041 ± 0.013	-	1.89E-03	
2.7.6.2	thiamine diphosphokinase	disease	-0.017 ± 0.028	-	0.549	0.566
2.7.6.2	thiamine diphosphokinase	sex	0.034 ± 0.013	+	8.28E-03	
2.7.6.2	thiamine diphosphokinase	anticholesterol drugs	0.006 ± 0.013	+	0.663	
2.8.1.6	biotin synthase	age	-0.007 ± 0.010	-	0.534	
2.8.1.6	biotin synthase	BMI	0.006 ± 0.011	+	0.571	
2.8.1.6	biotin synthase	constipation	-0.036 ± 0.011	-	6.54E-04	
**2.8.1.6**	**biotin synthase**	**disease**	**-0.081 ± 0.022**	**-**	**2.96E-04**	**8.65E-04***
2.8.1.6	biotin synthase	sex	-0.003 ± 0.010	-	0.754	
2.8.1.6	biotin synthase	anticholesterol drugs	0.013 ± 0.010	+	0.211	
3.1.26.11	tRNase Z	age	-0.009 ± 0.010	-	0.385	
3.1.26.11	tRNase Z	BMI	0.015 ± 0.010	+	0.125	
3.1.26.11	tRNase Z	constipation	-0.049 ± 0.010	-	1.32E-06	
3.1.26.11	tRNase Z	disease	-0.091 ± 0.021	-	1.63E-05	*1.04E-04
3.1.26.11	tRNase Z	sex	0.003 ± 0.010	+	0.770	
3.1.26.11	tRNase Z	anticholesterol drugs	0.005 ± 0.010	+	0.588	
3.1.3.41	4-nitrophenylphosphatase	age	-0.196 ± 0.053	-	2.30E-04	
3.1.3.41	4-nitrophenylphosphatase	BMI	-0.144 ± 0.056	-	0.0102	
3.1.3.41	4-nitrophenylphosphatase	constipation	0.151 ± 0.052	+	3.63E-03	
3.1.3.41	4-nitrophenylphosphatase	disease	0.211 ± 0.117	+	0.0714	0.0961
3.1.3.41	4-nitrophenylphosphatase	sex	-0.108 ± 0.051	-	0.0354	
3.1.3.41	4-nitrophenylphosphatase	anticholesterol drugs	-0.124 ± 0.054	-	0.0223	
3.1.3.71	2-phosphosulfolactate phosphatase	age	0.050 ± 0.039	+	0.199	
3.1.3.71	2-phosphosulfolactate phosphatase	BMI	-0.008 ± 0.040	-	0.839	
3.1.3.71	2-phosphosulfolactate phosphatase	constipation	0.081 ± 0.037	+	0.0274	
3.1.3.71	2-phosphosulfolactate phosphatase	disease	0.337 ± 0.085	+	6.83E-05	*2.81E-04
3.1.3.71	2-phosphosulfolactate phosphatase	sex	-0.031 ± 0.038	-	0.416	
3.1.3.71	2-phosphosulfolactate phosphatase	anticholesterol drugs	0.056 ± 0.037	+	0.132	
3.2.1.20	alpha-glucosidase	age	0.062 ± 0.028	+	0.0248	
3.2.1.20	alpha-glucosidase	BMI	-0.069 ± 0.030	-	0.0202	
3.2.1.20	alpha-glucosidase	constipation	0.102 ± 0.028	+	2.69E-04	
3.2.1.20	alpha-glucosidase	disease	0.106 ± 0.061	+	0.0817	0.108
3.2.1.20	alpha-glucosidase	sex	0.055 ± 0.028	+	0.0497	
3.2.1.20	alpha-glucosidase	anticholesterol drugs	0.031 ± 0.029	+	0.282	
3.2.1.23	beta-galactosidase	age	-0.017 ± 0.009	-	0.0700	
3.2.1.23	beta-galactosidase	BMI	0.003 ± 0.010	+	0.782	
3.2.1.23	beta-galactosidase	constipation	-0.034 ± 0.009	-	2.44E-04	
3.2.1.23	beta-galactosidase	disease	-0.024 ± 0.020	-	0.224	0.257
3.2.1.23	beta-galactosidase	sex	0.020 ± 0.009	+	0.0242	
3.2.1.23	beta-galactosidase	anticholesterol drugs	-0.002 ± 0.009	-	0.827	

3.2.1.8	endo-1,4-beta-xylanase	age	-0.018 ± 0.014	-	0.212	
3.2.1.8	endo-1,4-beta-xylanase	BMI	0.016 ± 0.016	+	0.300	
3.2.1.8	endo-1,4-beta-xylanase	constipation	-0.055 ± 0.015	-	2.02E-04	
3.2.1.8	endo-1,4-beta-xylanase	disease	-0.111 ± 0.031	-	3.48E-04	*9.75E-04
3.2.1.8	endo-1,4-beta-xylanase	sex	0.016 ± 0.014	+	0.269	
3.2.1.8	endo-1,4-beta-xylanase	anticholesterol drugs	-0.025 ± 0.015	-	0.0849	
3.2.1.89	arabinogalactan endo-beta-1,4-galactanase	age	NA	NA	NA	
3.2.1.89	arabinogalactan endo-beta-1,4-galactanase	BMI	NA	NA	NA	
3.2.1.89	arabinogalactan endo-beta-1,4-galactanase	constipation	NA	NA	NA	
3.2.1.89	arabinogalactan endo-beta-1,4-galactanase	disease	NA	NA	NA	NA
3.2.1.89	arabinogalactan endo-beta-1,4-galactanase	sex	NA	NA	NA	
3.2.1.89	arabinogalactan endo-beta-1,4-galactanase	anticholesterol drugs	NA	NA	NA	
3.4.13.19	membrane dipeptidase	age	0.003 ± 0.035	+	0.923	
3.4.13.19	membrane dipeptidase	BMI	-0.024 ± 0.042	-	0.576	
3.4.13.19	membrane dipeptidase	constipation	-0.156 ± 0.038	-	3.46E-05	
3.4.13.19	membrane dipeptidase	disease	-0.172 ± 0.078	-	0.0271	*0.0421
3.4.13.19	membrane dipeptidase	sex	0.091 ± 0.035	+	9.93E-03	
3.4.13.19	membrane dipeptidase	anticholesterol drugs	0.039 ± 0.037	+	0.287	
3.4.17.4	Gly-Xaa carboxypeptidase	age	-0.113 ± 0.066	-	0.0884	
3.4.17.4	Gly-Xaa carboxypeptidase	BMI	0.014 ± 0.069	+	0.844	
3.4.17.4	Gly-Xaa carboxypeptidase	constipation	-0.281 ± 0.081	-	5.27E-04	
3.4.17.4	Gly-Xaa carboxypeptidase	disease	-0.484 ± 0.146	-	9.47E-04	*2.29E-03
3.4.17.4	Gly-Xaa carboxypeptidase	sex	-0.204 ± 0.070	-	3.64E-03	
3.4.17.4	Gly-Xaa carboxypeptidase	anticholesterol drugs	-0.148 ± 0.073	-	0.0410	
3.5.3.12	agmatine deiminase	age	-0.038 ± 0.018	-	0.0396	
3.5.3.12	agmatine deiminase	BMI	0.032 ± 0.018	+	0.0790	
3.5.3.12	agmatine deiminase	constipation	-0.028 ± 0.019	-	0.134	
**3.5.3.12**	**agmatine deiminase**	**disease**	**-0.153 ± 0.040**	**-**	**1.28E-04**	***4.97E-04**
3.5.3.12	agmatine deiminase	sex	-0.017 ± 0.018	-	0.346	
3.5.3.12	agmatine deiminase	anticholesterol drugs	0.008 ± 0.019	+	0.653	
3.5.4.25	GTP cyclohydrolase II	age	-0.010 ± 0.008	-	0.228	
3.5.4.25	GTP cyclohydrolase II	BMI	0.004 ± 0.009	+	0.666	
3.5.4.25	GTP cyclohydrolase II	constipation	-0.021 ± 0.008	-	0.0106	
**3.5.4.25**	**GTP cyclohydrolase II**	**disease**	**-0.071 ± 0.017**	**-**	**4.77E-05**	***2.29E-04**
3.5.4.25	GTP cyclohydrolase II	sex	-0.029 ± 0.008	-	2.33E-04	
3.5.4.25	GTP cyclohydrolase II	anticholesterol drugs	0.009 ± 0.008	+	0.287	
3.5.4.26	diaminohydroxyphosphoribosylaminopyrimidine deaminase	age	-0.020 ± 0.009	-	0.0311	
3.5.4.26	diaminohydroxyphosphoribosylaminopyrimidine deaminase	BMI	0.013 ± 0.010	+	0.184	
3.5.4.26	diaminohydroxyphosphoribosylaminopyrimidine deaminase	constipation	-0.048 ± 0.010	-	6.26E-07	
**3.5.4.26**	**diaminohydroxyphosphoribosylaminopyrimidine deaminase**	**disease**	**-0.091 ± 0.020**	**-**	**7.33E-06**	***6.41E-05**
3.5.4.26	diaminohydroxyphosphoribosylaminopyrimidine deaminase	sex	-0.032 ± 0.009	-	6.45E-04	
3.5.4.26	diaminohydroxyphosphoribosylaminopyrimidine deaminase	anticholesterol drugs	0.002 ± 0.009	+	0.802	
3.5.4.28	S-adenosylhomocysteine deaminase	age	-0.013 ± 0.019	-	0.490	
3.5.4.28	S-adenosylhomocysteine deaminase	BMI	-0.056 ± 0.020	-	5.01E-03	
3.5.4.28	S-adenosylhomocysteine deaminase	constipation	0.073 ± 0.018	+	5.48E-05	
3.5.4.28	S-adenosylhomocysteine deaminase	disease	0.186 ± 0.040	+	2.64E-06	*3.08E-05
3.5.4.28	S-adenosylhomocysteine deaminase	sex	0.035 ± 0.018	+	0.0490	
3.5.4.28	S-adenosylhomocysteine deaminase	anticholesterol drugs	0.019 ± 0.018	+	0.305	
3.5.4.31	S-methyl-5'-thioadenosine deaminase	age	-0.031 ± 0.024	-	0.188	
3.5.4.31	S-methyl-5'-thioadenosine deaminase	BMI	-0.074 ± 0.026	-	3.90E-03	
3.5.4.31	S-methyl-5'-thioadenosine deaminase	constipation	0.085 ± 0.023	+	2.14E-04	
3.5.4.31	S-methyl-5'-thioadenosine deaminase	disease	0.254 ± 0.050	+	4.15E-07	*5.80E-06
3.5.4.31	S-methyl-5'-thioadenosine deaminase	sex	0.005 ± 0.023	+	0.842	
3.5.4.31	S-methyl-5'-thioadenosine deaminase	anticholesterol drugs	0.019 ± 0.023	+	0.408	
3.5.4.43	hydroxydechloroatrazine ethylaminohydrolase	age	0.144 ± 0.056	+	9.33E-03	
3.5.4.43	hydroxydechloroatrazine ethylaminohydrolase	BMI	0.068 ± 0.056	+	0.229	
3.5.4.43	hydroxydechloroatrazine ethylaminohydrolase	constipation	0.183 ± 0.050	+	2.20E-04	
3.5.4.43	hydroxydechloroatrazine ethylaminohydrolase	disease	0.404 ± 0.118	+	6.23E-04	*1.62E-03
3.5.4.43	hydroxydechloroatrazine ethylaminohydrolase	sex	-0.077 ± 0.052	-	0.137	
3.5.4.43	hydroxydechloroatrazine ethylaminohydrolase	anticholesterol drugs	-0.056 ± 0.052	-	0.283	
3.5.99.6	glucosamine-6-phosphate deaminase	age	NA	NA	NA	
3.5.99.6	glucosamine-6-phosphate deaminase	BMI	NA	NA	NA	
3.5.99.6	glucosamine-6-phosphate deaminase	constipation	NA	NA	NA	
3.5.99.6	glucosamine-6-phosphate deaminase	disease	NA	NA	NA	NA
3.5.99.6	glucosamine-6-phosphate deaminase	sex	NA	NA	NA	
3.5.99.6	glucosamine-6-phosphate deaminase	anticholesterol drugs	NA	NA	NA	
3.6.1.17	bis(5'-nucleosyl)-tetraphosphatase (asymmetrical)	age	0.268 ± 0.083	+	1.14E-03	
3.6.1.17	bis(5'-nucleosyl)-tetraphosphatase (asymmetrical)	BMI	-0.041 ± 0.077	-	0.594	
3.6.1.17	bis(5'-nucleosyl)-tetraphosphatase (asymmetrical)	constipation	0.149 ± 0.073	+	0.0409	
3.6.1.17	bis(5'-nucleosyl)-tetraphosphatase (asymmetrical)	disease	0.617 ± 0.170	+	2.88E-04	*8.65E-04
3.6.1.17	bis(5'-nucleosyl)-tetraphosphatase (asymmetrical)	sex	0.283 ± 0.075	+	1.59E-04	

3.6.1.17	bis(5'-nucleosyl)-tetraphosphatase (asymmetrical)	anticholesterol drugs	-0.079 ± 0.082	-	0.332	
3.6.3.28	phosphonate-transporting ATPase	age	-0.163 ± 0.044	-	2.14E-04	
3.6.3.28	phosphonate-transporting ATPase	BMI	-0.129 ± 0.044	-	3.44E-03	
3.6.3.28	phosphonate-transporting ATPase	constipation	0.217 ± 0.042	+	2.80E-07	
3.6.3.28	phosphonate-transporting ATPase	disease	0.351 ± 0.094	+	1.99E-04	*6.34E-04
3.6.3.28	phosphonate-transporting ATPase	sex	-0.076 ± 0.041	-	0.0632	
3.6.3.28	phosphonate-transporting ATPase	anticholesterol drugs	-0.064 ± 0.042	-	0.134	
3.8.1.5	haloalkane dehalogenase	age	0.108 ± 0.063	+	0.0855	
3.8.1.5	haloalkane dehalogenase	BMI	0.041 ± 0.067	+	0.543	
3.8.1.5	haloalkane dehalogenase	constipation	0.001 ± 0.066	+	0.984	
3.8.1.5	haloalkane dehalogenase	disease	-0.229 ± 0.142	-	0.106	0.130
3.8.1.5	haloalkane dehalogenase	sex	-0.009 ± 0.066	-	0.892	
3.8.1.5	haloalkane dehalogenase	anticholesterol drugs	-0.019 ± 0.065	-	0.775	
4.1.1.11	aspartate 1-decarboxylase	age	-0.009 ± 0.009	-	0.302	
4.1.1.11	aspartate 1-decarboxylase	BMI	0.005 ± 0.010	+	0.576	
4.1.1.11	aspartate 1-decarboxylase	constipation	-0.027 ± 0.009	-	3.20E-03	
4.1.1.11	aspartate 1-decarboxylase	disease	-0.107 ± 0.019	-	3.19E-08	*6.67E-07
4.1.1.11	aspartate 1-decarboxylase	sex	-0.018 ± 0.009	-	0.0409	
4.1.1.11	aspartate 1-decarboxylase	anticholesterol drugs	0.001 ± 0.009	+	0.870	
4.1.1.19	arginine decarboxylase	age	-0.009 ± 0.012	-	0.434	
4.1.1.19	arginine decarboxylase	BMI	0.020 ± 0.013	+	0.121	
4.1.1.19	arginine decarboxylase	constipation	-0.034 ± 0.012	-	5.01E-03	
**4.1.1.19**	**arginine decarboxylase**	**disease**	**-0.142 ± 0.026**	**-**	**3.81E-08**	***6.67E-07**
4.1.1.19	arginine decarboxylase	sex	-0.032 ± 0.012	-	6.40E-03	
4.1.1.19	arginine decarboxylase	anticholesterol drugs	0.008 ± 0.012	+	0.510	
4.1.1.65	phosphatidylserine decarboxylase	age	-0.010 ± 0.011	-	0.383	
4.1.1.65	phosphatidylserine decarboxylase	BMI	0.023 ± 0.011	+	0.0352	
4.1.1.65	phosphatidylserine decarboxylase	constipation	-0.038 ± 0.012	-	1.22E-03	
4.1.1.65	phosphatidylserine decarboxylase	disease	-0.099 ± 0.024	-	4.91E-05	*2.29E-04
4.1.1.65	phosphatidylserine decarboxylase	sex	-0.012 ± 0.011	-	0.292	
4.1.1.65	phosphatidylserine decarboxylase	anticholesterol drugs	-0.003 ± 0.011	-	0.791	
4.1.2.43	3-hexulose-6-phosphate synthase	age	0.096 ± 0.047	+	0.0407	
4.1.2.43	3-hexulose-6-phosphate synthase	BMI	-0.114 ± 0.047	-	0.0145	
4.1.2.43	3-hexulose-6-phosphate synthase	constipation	0.269 ± 0.042	+	2.20E-10	
4.1.2.43	3-hexulose-6-phosphate synthase	disease	0.139 ± 0.098	+	0.157	0.186
4.1.2.43	3-hexulose-6-phosphate synthase	sex	0.095 ± 0.044	+	0.0300	
4.1.2.43	3-hexulose-6-phosphate synthase	anticholesterol drugs	0.055 ± 0.044	+	0.209	
4.1.3.3	N-acetylneuraminate lyase	age	-0.012 ± 0.016	-	0.459	
4.1.3.3	N-acetylneuraminate lyase	BMI	-0.003 ± 0.018	-	0.858	
4.1.3.3	N-acetylneuraminate lyase	constipation	-0.051 ± 0.016	-	1.68E-03	
4.1.3.3	N-acetylneuraminate lyase	disease	-0.009 ± 0.035	-	0.804	0.804
4.1.3.3	N-acetylneuraminate lyase	sex	0.036 ± 0.016	+	0.0252	
4.1.3.3	N-acetylneuraminate lyase	anticholesterol drugs	0.021 ± 0.016	+	0.186	
4.1.99.1	tryptophanase	age	0.156 ± 0.043	+	2.82E-04	
4.1.99.1	tryptophanase	BMI	0.078 ± 0.045	+	0.0874	
4.1.99.1	tryptophanase	constipation	0.206 ± 0.041	+	4.98E-07	
4.1.99.1	tryptophanase	disease	-0.073 ± 0.092	-	0.430	0.449
4.1.99.1	tryptophanase	sex	-0.120 ± 0.042	-	4.72E-03	
4.1.99.1	tryptophanase	anticholesterol drugs	0.072 ± 0.041	+	0.0793	
4.1.99.12	3,4-dihydroxy-2-butanone-4-phosphate synthase	age	-0.007 ± 0.008	-	0.376	
4.1.99.12	3,4-dihydroxy-2-butanone-4-phosphate synthase	BMI	0.007 ± 0.009	+	0.411	
4.1.99.12	3,4-dihydroxy-2-butanone-4-phosphate synthase	constipation	-0.019 ± 0.008	-	0.0235	
**4.1.99.12**	**3,4-dihydroxy-2-butanone-4-phosphate synthase**	**disease**	**-0.053 ± 0.018**	**-**	**2.62E-03**	***5.56E-03**
4.1.99.12	3,4-dihydroxy-2-butanone-4-phosphate synthase	sex	-0.024 ± 0.008	-	2.73E-03	
4.1.99.12	3,4-dihydroxy-2-butanone-4-phosphate synthase	anticholesterol drugs	0.013 ± 0.008	+	0.105	
4.1.99.2	tyrosine phenol-lyase	age	0.074 ± 0.036	+	0.0387	
4.1.99.2	tyrosine phenol-lyase	BMI	-0.114 ± 0.038	-	3.03E-03	
4.1.99.2	tyrosine phenol-lyase	constipation	0.138 ± 0.034	+	5.01E-05	
4.1.99.2	tyrosine phenol-lyase	disease	0.131 ± 0.077	+	0.0879	0.112
4.1.99.2	tyrosine phenol-lyase	sex	0.032 ± 0.035	+	0.369	
4.1.99.2	tyrosine phenol-lyase	anticholesterol drugs	0.036 ± 0.035	+	0.307	
4.2.1.120	4-hydroxybutanoyl-CoA dehydratase	age	0.145 ± 0.032	+	5.66E-06	
4.2.1.120	4-hydroxybutanoyl-CoA dehydratase	BMI	-0.033 ± 0.033	-	0.313	
4.2.1.120	4-hydroxybutanoyl-CoA dehydratase	constipation	0.086 ± 0.030	+	4.33E-03	
4.2.1.120	4-hydroxybutanoyl-CoA dehydratase	disease	0.263 ± 0.069	+	1.48E-04	*5.17E-04
4.2.1.120	4-hydroxybutanoyl-CoA dehydratase	sex	0.163 ± 0.031	+	1.22E-07	
4.2.1.120	4-hydroxybutanoyl-CoA dehydratase	anticholesterol drugs	0.038 ± 0.031	+	0.222	
4.2.1.135	UDP-N-acetylglucosamine 4,6-dehydratase (configuration-retaining)	age	0.190 ± 0.049	+	9.96E-05	
4.2.1.135	UDP-N-acetylglucosamine 4,6-dehydratase (configuration-retaining)	BMI	-0.070 ± 0.049	-	0.156	
4.2.1.135	UDP-N-acetylglucosamine 4,6-dehydratase (configuration-retaining)	constipation	0.127 ± 0.045	+	4.77E-03	
4.2.1.135	UDP-N-acetylglucosamine 4,6-dehydratase (configuration-retaining)	disease	0.283 ± 0.104	+	6.25E-03	*0.0118

4.2.1.135	UDP-N-acetylglucosamine 4,6-dehydratase (configuration-retaining)	sex	0.279 ± 0.046	+	1.97E-09	
4.2.1.135	UDP-N-acetylglucosamine 4,6-dehydratase (configuration-retaining)	anticholesterol drugs	0.025 ± 0.046	+	0.587	
4.2.1.151	chorismate dehydratase	age	0.060 ± 0.037	+	0.104	
4.2.1.151	chorismate dehydratase	BMI	-0.090 ± 0.040	-	0.0232	
4.2.1.151	chorismate dehydratase	constipation	0.090 ± 0.035	+	0.0108	
4.2.1.151	chorismate dehydratase	disease	0.040 ± 0.079	+	0.618	0.627
4.2.1.151	chorismate dehydratase	sex	-0.001 ± 0.036	-	0.977	
4.2.1.151	chorismate dehydratase	anticholesterol drugs	0.014 ± 0.036	+	0.704	
4.2.1.33	3-isopropylmalate dehydratase	age	-0.005 ± 0.007	-	0.500	
4.2.1.33	3-isopropylmalate dehydratase	BMI	-0.003 ± 0.008	-	0.677	
4.2.1.33	3-isopropylmalate dehydratase	constipation	0.002 ± 0.007	+	0.814	
4.2.1.33	3-isopropylmalate dehydratase	disease	-0.052 ± 0.016	-	8.75E-04	*2.19E-03
4.2.1.33	3-isopropylmalate dehydratase	sex	-0.009 ± 0.007	-	0.228	
4.2.1.33	3-isopropylmalate dehydratase	anticholesterol drugs	0.005 ± 0.007	+	0.476	
4.2.1.59	3-hydroxyacyl-[acyl-carrier-protein] dehydratase	age	0.017 ± 0.008	+	0.0346	
4.2.1.59	3-hydroxyacyl-[acyl-carrier-protein] dehydratase	BMI	0.000 ± 0.009	+	0.996	
4.2.1.59	3-hydroxyacyl-[acyl-carrier-protein] dehydratase	constipation	-0.017 ± 0.008	-	0.0380	
**4.2.1.59**	**3-hydroxyacyl-[acyl-carrier-protein] dehydratase**	**disease**	**-0.031 ± 0.014**	**-**	**0.0300**	***0.0451**
4.2.1.59	3-hydroxyacyl-[acyl-carrier-protein] dehydratase	sex	0.012 ± 0.008	+	0.143	
4.2.1.59	3-hydroxyacyl-[acyl-carrier-protein] dehydratase	anticholesterol drugs	0.011 ± 0.008	+	0.176	
4.2.1.8	mannonate dehydratase	age	0.003 ± 0.012	+	0.827	
4.2.1.8	mannonate dehydratase	BMI	0.019 ± 0.013	+	0.145	
4.2.1.8	mannonate dehydratase	constipation	-0.031 ± 0.012	-	0.0131	
4.2.1.8	mannonate dehydratase	disease	-0.046 ± 0.027	-	0.0872	0.112
4.2.1.8	mannonate dehydratase	sex	0.011 ± 0.012	+	0.381	
4.2.1.8	mannonate dehydratase	anticholesterol drugs	0.000 ± 0.012	+	0.992	
4.3.1.14	3-aminobutyryl-CoA ammonia-lyase	age	0.113 ± 0.086	+	0.189	
4.3.1.14	3-aminobutyryl-CoA ammonia-lyase	BMI	-0.134 ± 0.082	-	0.101	
4.3.1.14	3-aminobutyryl-CoA ammonia-lyase	constipation	0.272 ± 0.079	+	5.73E-04	
4.3.1.14	3-aminobutyryl-CoA ammonia-lyase	disease	0.458 ± 0.203	+	0.0239	*0.0380
4.3.1.14	3-aminobutyryl-CoA ammonia-lyase	sex	0.045 ± 0.085	+	0.595	
4.3.1.14	3-aminobutyryl-CoA ammonia-lyase	anticholesterol drugs	0.079 ± 0.082	+	0.333	
5.3.1.24	phosphoribosylanthranilate isomerase	age	0.000 ± 0.008	-	0.965	
5.3.1.24	phosphoribosylanthranilate isomerase	BMI	0.012 ± 0.009	+	0.183	
5.3.1.24	phosphoribosylanthranilate isomerase	constipation	-0.015 ± 0.008	-	0.0836	
5.3.1.24	phosphoribosylanthranilate isomerase	disease	-0.056 ± 0.018	-	1.75E-03	*3.94E-03
5.3.1.24	phosphoribosylanthranilate isomerase	sex	0.015 ± 0.008	+	0.0629	
5.3.1.24	phosphoribosylanthranilate isomerase	anticholesterol drugs	0.002 ± 0.008	+	0.780	
5.3.3.3	vinylacetyl-CoA Delta-isomerase	age	0.182 ± 0.039	+	2.37E-06	
5.3.3.3	vinylacetyl-CoA Delta-isomerase	BMI	-0.083 ± 0.041	-	0.0424	
5.3.3.3	vinylacetyl-CoA Delta-isomerase	constipation	0.032 ± 0.036	+	0.366	
5.3.3.3	vinylacetyl-CoA Delta-isomerase	disease	0.354 ± 0.085	+	2.82E-05	*1.52E-04
5.3.3.3	vinylacetyl-CoA Delta-isomerase	sex	0.026 ± 0.037	+	0.483	
5.3.3.3	vinylacetyl-CoA Delta-isomerase	anticholesterol drugs	0.031 ± 0.036	+	0.385	
5.4.3.3	lysine 5,6-aminomutase	age	0.118 ± 0.070	+	0.093	
5.4.3.3	lysine 5,6-aminomutase	BMI	-0.114 ± 0.072	-	0.111	
5.4.3.3	lysine 5,6-aminomutase	constipation	0.214 ± 0.063	+	0.001	
5.4.3.3	lysine 5,6-aminomutase	disease	0.328 ± 0.157	+	0.037	0.0522
5.4.3.3	lysine 5,6-aminomutase	sex	0.052 ± 0.068	+	0.444	
5.4.3.3	lysine 5,6-aminomutase	anticholesterol drugs	0.116 ± 0.065	+	0.0759	
5.4.3.6	tyrosine 2,3-aminomutase	age	0.205 ± 0.057	+	3.60E-04	
5.4.3.6	tyrosine 2,3-aminomutase	BMI	-0.136 ± 0.061	-	0.0250	
5.4.3.6	tyrosine 2,3-aminomutase	constipation	0.174 ± 0.052	+	9.02E-04	
5.4.3.6	tyrosine 2,3-aminomutase	disease	0.252 ± 0.123	+	0.0404	0.0566
5.4.3.6	tyrosine 2,3-aminomutase	sex	0.127 ± 0.056	+	0.0234	
5.4.3.6	tyrosine 2,3-aminomutase	anticholesterol drugs	-0.002 ± 0.055	-	0.964	
5.4.99.1	methylaspartate mutase	age	0.130 ± 0.043	+	2.23E-03	
5.4.99.1	methylaspartate mutase	BMI	-0.021 ± 0.040	-	0.597	
5.4.99.1	methylaspartate mutase	constipation	0.162 ± 0.039	+	3.05E-05	
5.4.99.1	methylaspartate mutase	disease	0.086 ± 0.088	+	0.326	0.362
5.4.99.1	methylaspartate mutase	sex	-0.019 ± 0.040	-	0.635	
5.4.99.1	methylaspartate mutase	anticholesterol drugs	0.125 ± 0.039	+	1.17E-03	
5.4.99.9	UDP-galactopyranose mutase	age	-0.038 ± 0.015	-	0.0116	
5.4.99.9	UDP-galactopyranose mutase	BMI	-0.004 ± 0.017	-	0.804	
5.4.99.9	UDP-galactopyranose mutase	constipation	0.007 ± 0.015	+	0.664	
5.4.99.9	UDP-galactopyranose mutase	disease	-0.076 ± 0.033	-	0.0193	*0.0315
5.4.99.9	UDP-galactopyranose mutase	sex	-0.024 ± 0.015	-	0.103	
5.4.99.9	UDP-galactopyranose mutase	anticholesterol drugs	-0.001 ± 0.015	-	0.965	
6.1.1.2	tryptophan---tRNA ligase	age	0.010 ± 0.012	+	0.421	
6.1.1.2	tryptophan---tRNA ligase	BMI	-0.021 ± 0.013	-	0.109	
6.1.1.2	tryptophan---tRNA ligase	constipation	0.042 ± 0.012	+	4.68E-04	

6.1.1.2	tryptophan---tRNA ligase	disease	0.074 ± 0.026	+	4.89E-03	*9.51E-03
6.1.1.2	tryptophan---tRNA ligase	sex	-0.016 ± 0.012	-	0.178	
6.1.1.2	tryptophan---tRNA ligase	anticholesterol drugs	0.012 ± 0.012	+	0.306	
6.3.2.1	pantoate---beta-alanine ligase (AMP-forming)	age	0.001 ± 0.010	+	0.934	
6.3.2.1	pantoate---beta-alanine ligase (AMP-forming)	BMI	0.013 ± 0.012	+	0.257	
6.3.2.1	pantoate---beta-alanine ligase (AMP-forming)	constipation	-0.012 ± 0.010	-	0.244	
6.3.2.1	pantoate---beta-alanine ligase (AMP-forming)	disease	-0.102 ± 0.022	-	4.15E-06	*4.15E-05
6.3.2.1	pantoate---beta-alanine ligase (AMP-forming)	sex	-0.028 ± 0.010	-	5.08E-03	
6.3.2.1	pantoate---beta-alanine ligase (AMP-forming)	anticholesterol drugs	0.004 ± 0.010	+	0.708	
6.3.4.20	7-cyano-7-deazaguanine synthase	age	-0.018 ± 0.012	-	0.119	
6.3.4.20	7-cyano-7-deazaguanine synthase	BMI	0.038 ± 0.011	+	9.97E-04	
6.3.4.20	7-cyano-7-deazaguanine synthase	constipation	-0.015 ± 0.012	-	0.212	
6.3.4.20	7-cyano-7-deazaguanine synthase	disease	-0.049 ± 0.025	-	0.0548	0.0753
6.3.4.20	7-cyano-7-deazaguanine synthase	sex	-0.035 ± 0.012	-	2.74E-03	
6.3.4.20	7-cyano-7-deazaguanine synthase	anticholesterol drugs	-0.015 ± 0.012	-	0.196	

False discovery rates (q-values) for the disease state were calculated for the 70 EC numbers using the Banjamini-Hochberg method.

Note that "disease" has significant *q* -values for all the EC numbers in riboflavin, biotin, and arginine and proline pathways (indicated in bold).

**q* -value < 0.05

Supplementary Table 7. Meta-analysis of species and genera in six datasets

Species	Increased or decreased in PD	*p* -value (FEM)	*q* -value (FEM)	*p* -value (REM)	*q* -value (REM)	I^2 (%)	Relative abundance (%)
k__Bacteria|p__Firmicutes|c__Clostridia|o__Clostridia_unclassified|f__Clostridia_unclassified|g__Clostridia_unclassified|s__Clostridia_bacterium	+	0	*< 1.0E-16	< 1.0E-16	*< 1.0E-16	4.47	2.47
k__Bacteria|p__Firmicutes|c__Clostridia|o__Clostridiales|f__Ruminococcaceae|g__Anaerotruncus|s__Anaerotruncus_rubiinfantis	+	0	*< 1.0E-16	< 1.0E-16	*< 1.0E-16	0.00	4.3E-03
k__Bacteria|p__Firmicutes|c__Clostridia|o__Clostridiales|f__Ruminococcaceae|g__Ruminococcaceae_unclassified|s__Ruminococcaceae_bacterium	+	0	*< 1.0E-16	2.22E-16	*9.55E-15	12.14	0.84
k__Bacteria|p__Firmicutes|c__Clostridia|o__Clostridiales|f__Lachnospiraceae|g__Faecalicatena|s__Faecalicatena_contorta	+	4.21E-10	*5.43E-09	4.21E-10	*1.36E-08	0.00	8.7E-03
k__Bacteria|p__Firmicutes|c__Clostridia|o__Clostridiales|f__Christensenellaceae|g__Christensenellaceae_unclassified|s__Christensenellaceae_bacterium	+	2.22E-16	*5.73E-15	5.53E-10	*1.43E-08	42.90	0.027
k__Bacteria|p__Firmicutes|c__Clostridia|o__Clostridiales|f__Ruminococcaceae|g__Ruthenibacterium|s__Ruthenibacterium_lactatiformans	+	0	*< 1.0E-16	5.43E-08	*1.17E-06	62.97	0.25
k__Bacteria|p__Firmicutes|c__Clostridia|o__Clostridiales|f__Lachnospiraceae|g__Lacrimispora|s__Lacrimispora_amygdalina	-	9.54E-07	*6.36E-06	9.54E-07	*1.76E-05	0.00	0.16
k__Bacteria|p__Bacteroidetes|c__Bacteroidia|o__Bacteroidales|f__Rikenellaceae|g__Alistipes|s__Alistipes_onderdonkii	+	6.19E-06	*2.96E-05	6.19E-06	*9.98E-05	0.00	1.20
k__Bacteria|p__Firmicutes|c__Clostridia|o__Clostridiales|f__Ruminococcaceae|g__GGB9699|s__GGB9699_SGB15216	+	2.65E-11	*4.89E-10	7.06E-06	*1.01E-04	54.57	2.04
k__Bacteria|p__Actinobacteria|c__Actinobacteria|o__Bifidobacteriales|f__Bifidobacteriaceae|g__Bifidobacterium|s__Bifidobacterium_longum	+	6.12E-05	*2.55E-04	6.12E-05	*7.89E-04	0.00	0.95
k__Bacteria|p__Bacteroidetes|c__Bacteroidia|o__Bacteroidales|f__Tannerellaceae|g__Parabacteroides|s__Parabacteroides_merdae	+	7.82E-05	*3.06E-04	7.82E-05	*9.18E-04	0.00	0.62
k__Bacteria|p__Firmicutes|c__Clostridia|o__Clostridiales|f__Lachnospiraceae|g__Roseburia|s__Roseburia_faecis	-	1.84E-06	*1.03E-05	2.29E-04	*2.27E-03	39.92	0.70
k__Bacteria|p__Bacteroidetes|c__Bacteroidia|o__Bacteroidales|f__Rikenellaceae|g__Alistipes|s__Alistipes_shahii	+	4.66E-11	*7.51E-10	2.18E-04	*2.27E-03	68.65	0.53
k__Bacteria|p__Proteobacteria|c__Gammaproteobacteria|o__Pasteurellales|f__Pasteurellaceae|g__Haemophilus|s__Haemophilus_parainfluenzae	-	2.70E-04	*9.68E-04	2.70E-04	*2.49E-03	0.00	0.070
k__Bacteria|p__Firmicutes|c__Clostridia|o__Clostridiales|f__Ruminococcaceae|g__Faecalibacterium|s__Faecalibacterium_prausnitzii	-	2.23E-06	*1.20E-05	3.12E-04	*2.68E-03	41.92	3.30
k__Bacteria|p__Firmicutes|c__Clostridia|o__Clostridiales|f__Clostridiales_unclassified|g__Intestinimonas|s__Intestinimonas_butyriciproducens	+	1.01E-10	*1.44E-09	6.57E-04	*5.04E-03	72.24	0.040
k__Bacteria|p__Firmicutes|c__Clostridia|o__Clostridiales|f__Ruminococcaceae|g__Agathobaculum|s__Agathobaculum_butyriciproducens	-	9.86E-07	*6.36E-06	6.64E-04	*5.04E-03	51.63	0.12
k__Bacteria|p__Firmicutes|c__Clostridia|o__Clostridiales|f__Lachnospiraceae|g__Enterocloster|s__Enterocloster_asparagiformis	+	1.11E-08	*1.10E-07	1.13E-03	*8.06E-03	67.50	0.010
k__Bacteria|p__Firmicutes|c__Clostridia|o__Clostridiales|f__Oscillospiraceae|g__Dysosmobacter|s__Dysosmobacter_sp_NSJ_60	+	3.00E-09	*3.52E-08	1.38E-03	*8.07E-03	70.48	0.040
k__Bacteria|p__Firmicutes|c__Erysipelotrichia|o__Erysipelotrichales|f__Erysipelotrichaceae|g__Erysipelatoclostridium|s__Clostridium_innocuum	+	1.29E-03	*3.97E-03	1.27E-03	*8.07E-03	0.00	0.040
k__Bacteria|p__Firmicutes|c__Clostridia|o__Clostridiales|f__Ruminococcaceae|g__Anaeromassilibacillus|s__Anaeromassilibacillus_sp_An250	+	3.00E-08	*2.42E-07	1.36E-03	*8.07E-03	66.60	0.010
k__Bacteria|p__Bacteroidetes|c__Bacteroidia|o__Bacteroidales|f__Barnesiellaceae|g__Barnesiella|s__Barnesiella_intestinihominis	+	2.49E-04	*9.17E-04	1.29E-03	*8.07E-03	23.72	0.41
k__Bacteria|p__Verrucomicrobia|c__Verrucomicrobiae|o__Verrucomicrobiales|f__Akkermansiaceae|g__Akkermansia|s__Akkermansia_muciniphila	+	3.38E-13	*7.27E-12	1.54E-03	*8.28E-03	81.02	0.92
k__Bacteria|p__Firmicutes|c__Clostridia|o__Clostridiales|f__Lachnospiraceae|g__Roseburia|s__Roseburia_intestinalis	-	2.74E-05	*1.18E-04	1.52E-03	*8.28E-03	42.97	0.31
k__Bacteria|p__Bacteroidetes|c__Bacteroidia|o__Bacteroidales|f__Rikenellaceae|g__Alistipes|s__Alistipes_ihumii	+	9.11E-09	*9.79E-08	2.75E-03	*0.014	72.84	0.080
k__Bacteria|p__Bacteroidetes|c__Bacteroidia|o__Bacteroidales|f__Bacteroidaceae|g__Bacteroides|s__Bacteroides_cellulosilyticus	+	6.78E-06	*3.12E-05	2.99E-03	*0.015	56.49	0.44
k__Bacteria|p__Bacteroidetes|c__Bacteroidia|o__Bacteroidales|f__Bacteroidales_unclassified|g__Phocaeicola|s__Phocaeicola_vulgatus	-	2.51E-05	*1.12E-04	3.30E-03	*0.016	51.39	6.31
k__Bacteria|p__Firmicutes|c__Clostridia|o__Clostridiales|f__Lachnospiraceae|g__Roseburia|s__Roseburia_sp_AF02_12	+	3.19E-06	*1.65E-05	4.41E-03	*0.02	62.64	0.16
k__Bacteria|p__Bacteroidetes|c__Bacteroidia|o__Bacteroidales|f__Rikenellaceae|g__Alistipes|s__Alistipes_indistinctus	+	1.52E-08	*1.35E-07	5.28E-03	*0.023	75.70	0.10
k__Bacteria|p__Bacteroidetes|c__Bacteroidia|o__Bacteroidales|f__Rikenellaceae|g__Alistipes|s__Alistipes_putredinis	+	1.77E-06	*1.03E-05	5.54E-03	*0.024	66.30	1.97
k__Bacteria|p__Proteobacteria|c__Deltaproteobacteria|o__Desulfovibrionales|f__Desulfovibrionaceae|g__Bilophila|s__Bilophila_wadsworthia	+	1.56E-08	*1.35E-07	6.21E-03	*0.026	76.57	0.20
k__Bacteria|p__Firmicutes|c__CFGB1217|o__OFGB1217|f__FGB1217|g__GGB2982|s__GGB2982_SGB3964	+	5.90E-04	*1.95E-03	8.02E-03	*0.032	40.46	0.010
k__Bacteria|p__Firmicutes|c__Clostridia|o__Clostridiales|f__Clostridiaceae|g__Hungatella|s__Hungatella_hathewayi	+	6.51E-05	*2.62E-04	8.39E-03	*0.033	56.43	0.050
k__Bacteria|p__Bacteroidetes|c__Bacteroidia|o__Bacteroidales|f__Rikenellaceae|g__Alistipes|s__Alistipes_communis	+	7.58E-07	*5.43E-06	9.68E-03	*0.037	72.64	0.18
k__Bacteria|p__Firmicutes|c__Clostridia|o__Clostridiales|f__Ruminococcaceae|g__Anaerotruncus|s__Anaerotruncus_colihominis	+	2.06E-03	*5.60E-03	0.011	*0.04	31.57	0.020
k__Bacteria|p__Firmicutes|c__Clostridia|o__Clostridiales|f__Lachnospiraceae|g__Lachnoclostridium|s__Clostridium_symbiosum	+	7.71E-03	*0.017	0.011	*0.041	9.73	0.040
k__Bacteria|p__Firmicutes|c__Clostridia|o__Clostridiales|f__Oscillospiraceae|g__Dysosmobacter|s__Dysosmobacter_welbionis	+	3.58E-04	*1.25E-03	0.012	*0.042	50.41	0.33
k__Bacteria|p__Firmicutes|c__Clostridia|o__Clostridiales|f__Lachnospiraceae|g__Blautia|s__Blautia_wexlerae	-	1.60E-06	*9.83E-06	0.013	*0.043	73.04	1.20
k__Bacteria|p__Bacteroidetes|c__Bacteroidia|o__Bacteroidales|f__Tannerellaceae|g__Parabacteroides|s__Parabacteroides_goldsteinii	+	9.11E-04	*2.87E-03	0.015	0.050	46.43	0.070
k__Bacteria|p__Firmicutes|c__Clostridia|o__Clostridiales|f__Lachnospiraceae|g__Mediterraneibacter|s__Ruminococcus_gnavus	-	4.21E-04	*1.43E-03	0.016	0.053	53.68	0.44
k__Bacteria|p__Firmicutes|c__Clostridia|o__Clostridiales|f__Lachnospiraceae|g__Dorea|s__Dorea_longicatena	-	0.023	*0.046	0.023	0.074	0.00	0.28
k__Bacteria|p__Firmicutes|c__Clostridia|o__Clostridiales|f__Clostridiaceae|g__Clostridium|s__Clostridium_sp_AM22_11AC	-	1.54E-03	*4.63E-03	0.026	0.079	50.31	0.23
k__Bacteria|p__Firmicutes|c__Clostridia|o__Clostridiales|f__Ruminococcaceae|g__Ruminococcaceae_unclassified|s__Eubacterium_siraeum	+	1.90E-03	*5.32E-03	0.029	0.086	50.32	0.42
k__Bacteria|p__Firmicutes|c__Clostridia|o__Clostridiales|f__Lachnospiraceae|g__Dorea|s__Dorea_sp_AF36_15AT	+	2.13E-03	*5.60E-03	0.031	0.091	50.71	0.030
k__Bacteria|p__Bacteroidetes|c__Bacteroidia|o__Bacteroidales|f__Bacteroidaceae|g__Bacteroides|s__Bacteroides_nordii	+	0.036	0.064	0.036	0.103	0.00	0.10
k__Bacteria|p__Firmicutes|c__Clostridia|o__Clostridiales|f__Eubacteriaceae|g__Eubacteriaceae_unclassified|s__Eubacteriaceae_bacterium	+	2.93E-03	*7.29E-03	0.042	0.117	53.23	0.070
k__Bacteria|p__Firmicutes|c__Clostridia|o__Clostridiales|f__Lachnospiraceae|g__Lachnospiraceae_unclassified|s__Eubacterium_rectale	-	5.13E-03	*0.012	0.047	0.124	49.43	1.27
k__Bacteria|p__Firmicutes|c__Clostridia|o__Clostridiales|f__Ruminococcaceae|g__Ruminococcaceae_unclassified|s__Ruminococcaceae_unclassified_SGB15265	+	6.41E-07	*4.86E-06	0.047	0.124	84.06	0.21
k__Bacteria|p__Firmicutes|c__Clostridia|o__Clostridiales|f__Ruminococcaceae|g__GGB9615|s__GGB9615_SGB15053	+	2.09E-03	*5.60E-03	0.047	0.124	58.38	0.30
k__Bacteria|p__Firmicutes|c__Clostridia|o__Clostridiales|f__Ruminococcaceae|g__GGB9632|s__GGB9632_SGB15089	+	8.81E-05	*3.34E-04	0.049	0.128	74.89	0.27
k__Bacteria|p__Firmicutes|c__Clostridia|o__Clostridiales|f__Ruminococcaceae|g__GGB9707|s__GGB9707_SGB15229	+	2.47E-03	*6.37E-03	0.066	0.167	63.14	0.030
k__Bacteria|p__Firmicutes|c__Clostridia|o__Clostridiales|f__Lachnospiraceae|g__Enterocloster|s__Enterocloster_aldensis	+	0.011	*0.024	0.069	0.171	48.44	0.030
k__Bacteria|p__Firmicutes|c__CFGB3053|o__OFGB3053|f__FGB3053|g__GGB9581|s__GGB9581_SGB14999	+	1.80E-03	*5.16E-03	0.073	0.178	67.04	0.020
k__Bacteria|p__Firmicutes|c__Clostridia|o__Clostridiales|f__Lachnospiraceae|g__Dorea|s__Dorea_formicigenerans	-	0.081	0.131	0.081	0.195	0.00	0.10
k__Bacteria|p__Bacteroidetes|c__Bacteroidia|o__Bacteroidales|f__Tannerellaceae|g__Parabacteroides|s__Parabacteroides_distasonis	+	7.68E-03	*0.017	0.085	0.200	58.27	1.14
k__Bacteria|p__Firmicutes|c__Clostridia|o__Clostridiales|f__Lachnospiraceae|g__Enterocloster|s__Enterocloster_citroniae	+	7.89E-04	*2.55E-03	0.110	0.253	77.28	0.010
k__Bacteria|p__Firmicutes|c__Clostridia|o__Clostridiales|f__Ruminococcaceae|g__Ruminococcaceae_unclassified|s__Clostridium_leptum	+	5.11E-06	*2.53E-05	0.114	0.258	87.98	0.12
k__Bacteria|p__Firmicutes|c__Clostridia|o__Clostridiales|f__Lachnospiraceae|g__Roseburia|s__Roseburia_inulinivorans	-	0.024	*0.047	0.128	0.276	54.34	0.33
k__Bacteria|p__Firmicutes|c__Clostridia|o__Clostridiales|f__Eubacteriaceae|g__Eubacterium|s__Eubacterium_ventriosum	+	5.12E-03	*0.012	0.128	0.276	70.47	0.16
k__Bacteria|p__Firmicutes|c__Clostridia|o__Clostridia_unclassified|f__Clostridia_unclassified|g__Candidatus_Avimicrobium|s__Candidatus_Avimicrobium_caecorum	+	0.048	0.082	0.128	0.276	40.84	0.030

k__Bacteria|p__Firmicutes|c__Clostridia|o__Clostridiales|f__Lachnospiraceae|g__GGB3571|s__GGB3571_SGB4778	+	7.92E-03	*0.017	0.131	0.277	67.67	0.010
k__Bacteria|p__Bacteroidetes|c__Bacteroidia|o__Bacteroidales|f__Rikenellaceae|g__Alistipes|s__Alistipes_finegoldii	+	0.036	0.064	0.137	0.286	49.95	0.29
k__Bacteria|p__Firmicutes|c__Clostridia|o__Clostridiales|f__Ruminococcaceae|g__Candidatus_Cibiobacter|s__Candidatus_Cibiobacter_qucibialis	+	0.006	*0.013	0.144	0.290	71.68	0.58
k__Bacteria|p__Bacteroidetes|c__Bacteroidia|o__Bacteroidales|f__Bacteroidaceae|g__Bacteroides|s__Bacteroides_caccae	+	3.12E-02	0.057	0.142	0.290	54.03	0.75
k__Bacteria|p__Firmicutes|c__Clostridia|o__Clostridiales|f__Ruminococcaceae|g__Ruminococcus|s__Ruminococcus_bicirculans	-	0.018	*0.038	0.148	0.294	62.40	0.54
k__Bacteria|p__Firmicutes|c__Clostridia|o__Clostridia_unclassified|f__Clostridia_unclassified|g__Clostridia_unclassified|s__Clostridia_bacterium_UC5_1_1D1	+	2.94E-03	*7.29E-03	0.151	0.295	76.69	0.010
k__Bacteria|p__Firmicutes|c__Clostridia|o__Clostridiales|f__Lachnospiraceae|g__Lachnospiraceae_unclassified|s__Lachnospiraceae_bacterium_WCA3_601_WT_6H	+	0.164	0.232	0.170	0.308	0.00	0.32
k__Bacteria|p__Firmicutes|c__Clostridia|o__Clostridiales|f__Lachnospiraceae|g__Blautia|s__Blautia_SGB4815	+	0.036	0.064	0.168	0.308	56.77	0.030
k__Bacteria|p__Firmicutes|c__Clostridia|o__Clostridiales|f__Lachnospiraceae|g__Anaerobutyricum|s__Anaerobutyricum_hallii	+	0.069	0.114	0.168	0.308	42.52	0.33
k__Bacteria|p__Firmicutes|c__Clostridia|o__Clostridiales|f__Clostridiaceae|g__Clostridium|s__Clostridium_fessum	-	0.031	0.057	0.170	0.308	59.54	0.19
k__Bacteria|p__Firmicutes|c__Clostridia|o__Clostridiales|f__Lachnospiraceae|g__Lachnospira|s__Lachnospira_pectinoschiza	-	0.084	0.133	0.164	0.308	36.81	0.30
k__Bacteria|p__Bacteroidetes|c__Bacteroidia|o__Bacteroidales|f__Odoribacteraceae|g__Odoribacter|s__Odoribacter_splanchnicus	+	0.018	*0.037	0.176	0.315	67.32	0.28
k__Bacteria|p__Firmicutes|c__Clostridia|o__Clostridiales|f__Clostridiales_unclassified|g__Clostridiales_unclassified|s__Clostridiales_bacterium	+	1.78E-03	*0.005	0.191	0.338	82.51	0.10
k__Bacteria|p__Firmicutes|c__Clostridia|o__Clostridiales|f__Clostridiaceae|g__Clostridium|s__Clostridium_phoceensis	+	3.55E-03	*8.64E-03	0.201	0.351	80.79	0.090
k__Bacteria|p__Firmicutes|c__Clostridia|o__Clostridiales|f__Lachnospiraceae|g__Blautia|s__Blautia_obeum	+	0.043	0.074	0.205	0.352	60.83	0.24
k__Bacteria|p__Firmicutes|c__Clostridia|o__Clostridiales|f__Lachnospiraceae|g__Lachnospira|s__Lachnospira_eligens	+	0.085	0.134	0.208	0.352	46.50	0.52
k__Bacteria|p__Firmicutes|c__Erysipelotrichia|o__Erysipelotrichales|f__Erysipelotrichaceae|g__Faecalibacillus|s__Faecalibacillus_intestinalis	+	0.102	0.156	0.224	0.375	44.83	0.13
k__Bacteria|p__Firmicutes|c__Clostridia|o__Clostridiales|f__Clostridiales_unclassified|g__Evtepia|s__Evtepia_gabavorous	+	0.021	*0.042	0.234	0.387	73.53	0.080
k__Bacteria|p__Proteobacteria|c__Betaproteobacteria|o__Burkholderiales|f__Sutterellaceae|g__Parasutterella|s__Parasutterella_excrementihominis	+	0.237	0.329	0.237	0.387	0.00	0.13
k__Bacteria|p__Firmicutes|c__Clostridia|o__Clostridiales|f__Lachnospiraceae|g__Blautia|s__Blautia_massiliensis	-	0.025	*0.048	0.254	0.409	73.89	0.33
k__Bacteria|p__Firmicutes|c__Negativicutes|o__Acidaminococcales|f__Acidaminococcaceae|g__Phascolarctobacterium|s__Phascolarctobacterium_faecium	+	0.110	0.164	0.302	0.472	58.05	0.70
k__Bacteria|p__Firmicutes|c__Clostridia|o__Clostridiales|f__Lachnospiraceae|g__GGB3746|s__GGB3746_SGB5089	-	0.015	*0.032	0.304	0.472	81.96	0.21
k__Bacteria|p__Firmicutes|c__Clostridia|o__Clostridiales|f__Oscillospiraceae|g__Oscillibacter|s__Oscillibacter_sp_ER4	+	0.065	0.109	0.300	0.472	68.90	0.46
k__Bacteria|p__Firmicutes|c__Clostridia|o__Clostridiales|f__Lachnospiraceae|g__Enterocloster|s__Enterocloster_bolteae	+	0.276	0.371	0.318	0.488	15.78	0.12
k__Bacteria|p__Firmicutes|c__Clostridia|o__Clostridiales|f__Ruminococcaceae|g__Flavonifractor|s__Flavonifractor_plautii	-	0.322	0.424	0.322	0.489	0.00	0.30
k__Bacteria|p__Actinobacteria|c__Coriobacteriia|o__Eggerthellales|f__Eggerthellaceae|g__Adlercreutzia|s__Adlercreutzia_equolifaciens	+	0.022	*0.044	0.344	0.515	82.86	0.060
k__Bacteria|p__Firmicutes|c__Clostridia|o__Clostridiales|f__Lachnospiraceae|g__Fusicatenibacter|s__Fusicatenibacter_saccharivorans	-	0.040	0.069	0.353	0.524	79.65	0.68
k__Bacteria|p__Actinobacteria|c__Coriobacteriia|o__Coriobacteriales|f__Coriobacteriaceae|g__Collinsella|s__Collinsella_aerofaciens	+	0.080	0.131	0.375	0.545	74.23	0.55
k__Bacteria|p__Firmicutes|c__Clostridia|o__Clostridiales|f__Clostridiales_unclassified|g__Clostridiales_unclassified|s__Clostridiales_bacterium_KLE1615	-	0.105	0.159	0.376	0.545	70.25	0.14
k__Bacteria|p__Firmicutes|c__Clostridia|o__Clostridiales|f__Lachnospiraceae|g__Blautia|s__Blautia_faecis	+	0.131	0.188	0.387	0.548	66.85	0.29
k__Bacteria|p__Actinobacteria|c__Actinobacteria|o__Bifidobacteriales|f__Bifidobacteriaceae|g__Bifidobacterium|s__Bifidobacterium_adolescentis	+	0.264	0.362	0.385	0.548	40.00	1.20
k__Bacteria|p__Firmicutes|c__Bacilli|o__Lactobacillales|f__Streptococcaceae|g__Streptococcus|s__Streptococcus_salivarius	+	0.119	0.174	0.410	0.569	72.13	0.47
k__Bacteria|p__Firmicutes|c__Clostridia|o__Clostridiales|f__Clostridiaceae|g__Clostridiaceae_unclassified|s__Clostridiaceae_bacterium_OM08_6BH	-	0.284	0.378	0.410	0.569	40.98	0.020
k__Bacteria|p__Firmicutes|c__Clostridia|o__Clostridiales|f__Clostridiaceae|g__Clostridiaceae_unclassified|s__Clostridiaceae_bacterium	-	0.095	0.148	0.424	0.581	76.98	0.27
k__Bacteria|p__Bacteroidetes|c__Bacteroidia|o__Bacteroidales|f__Bacteroidaceae|g__Bacteroides|s__Bacteroides_uniformis	+	0.447	0.539	0.447	0.607	0.00	4.81
k__Bacteria|p__Actinobacteria|c__Coriobacteriia|o__Eggerthellales|f__Eggerthellaceae|g__Eggerthella|s__Eggerthella_lenta	+	0.433	0.527	0.465	0.619	13.08	0.12
k__Bacteria|p__Bacteroidetes|c__Bacteroidia|o__Bacteroidales|f__Bacteroidaceae|g__Bacteroides|s__Bacteroides_finegoldii	+	0.123	0.178	0.466	0.619	77.64	0.35
k__Bacteria|p__Firmicutes|c__Erysipelotrichia|o__Erysipelotrichales|f__Erysipelotrichaceae|g__Holdemania|s__Holdemania_filiformis	+	0.110	0.164	0.475	0.619	79.59	0.010
k__Bacteria|p__Firmicutes|c__Clostridia|o__Clostridiales|f__Lachnospiraceae|g__Roseburia|s__Roseburia_hominis	+	0.270	0.366	0.471	0.619	58.16	0.17
k__Bacteria|p__Firmicutes|c__Clostridia|o__Clostridiales|f__Peptostreptococcaceae|g__Romboutsia|s__Romboutsia_timonensis	+	0.237	0.329	0.535	0.690	72.40	0.060
k__Bacteria|p__Bacteroidetes|c__Bacteroidia|o__Bacteroidales|f__Bacteroidaceae|g__Bacteroides|s__Bacteroides_fragilis	-	0.541	0.623	0.547	0.699	3.13	0.77
k__Bacteria|p__Firmicutes|c__Bacilli|o__Lactobacillales|f__Streptococcaceae|g__Streptococcus|s__Streptococcus_parasanguinis	+	0.329	0.428	0.574	0.726	66.84	0.12
k__Bacteria|p__Bacteroidetes|c__Bacteroidia|o__Bacteroidales|f__Bacteroidaceae|g__Bacteroides|s__Bacteroides_xylanisolvens	-	0.427	0.525	0.583	0.731	52.29	0.69
k__Bacteria|p__Firmicutes|c__Clostridia|o__Clostridiales|f__Lachnospiraceae|g__Coprococcus|s__Coprococcus_catus	+	0.376	0.476	0.602	0.747	65.27	0.050
k__Bacteria|p__Bacteroidetes|c__Bacteroidia|o__Bacteroidales|f__Bacteroidales_unclassified|g__Phocaeicola|s__Phocaeicola_massiliensis	-	0.532	0.618	0.612	0.752	34.31	0.82
k__Bacteria|p__Firmicutes|c__Clostridia|o__Clostridiales|f__Clostridiaceae|g__Clostridium|s__Clostridium_sp_AF34_10BH	-	0.587	0.658	0.623	0.756	18.01	0.41
k__Bacteria|p__Firmicutes|c__Clostridia|o__Clostridiales|f__Ruminococcaceae|g__Phocea|s__Phocea_massiliensis	+	0.387	0.485	0.627	0.756	68.41	0.010
k__Bacteria|p__Bacteroidetes|c__Bacteroidia|o__Bacteroidales|f__Bacteroidaceae|g__Bacteroides|s__Bacteroides_ovatus	-	0.356	0.454	0.641	0.766	74.53	1.73
k__Bacteria|p__Firmicutes|c__Clostridia|o__Clostridiales|f__Lachnospiraceae|g__Lacrimispora|s__Lacrimispora_celerecrescens	+	0.398	0.494	0.669	0.785	73.92	0.12
k__Bacteria|p__Firmicutes|c__Clostridia|o__Clostridiales|f__Lachnospiraceae|g__Faecalicatena|s__Faecalicatena_fissicatena	+	0.484	0.577	0.666	0.785	62.82	0.080
k__Bacteria|p__Firmicutes|c__Clostridia|o__Clostridiales|f__Ruminococcaceae|g__Ruminococcus|s__Ruminococcus_bromii	+	0.611	0.679	0.682	0.792	35.13	1.16
k__Bacteria|p__Firmicutes|c__Clostridia|o__Clostridiales|f__Ruminococcaceae|g__GGB9619|s__GGB9619_SGB15067	+	0.340	0.439	0.697	0.803	83.37	0.010
k__Bacteria|p__Firmicutes|c__Clostridia|o__Clostridiales|f__Lachnospiraceae|g__Coprococcus|s__Coprococcus_comes	-	0.488	0.577	0.708	0.808	70.77	0.17
k__Bacteria|p__Firmicutes|c__Firmicutes_unclassified|o__Firmicutes_unclassified|f__Firmicutes_unclassified|g__Firmicutes_unclassified|s__Firmicutes_bacterium_AF16_15	-	0.525	0.616	0.734	0.824	70.88	0.13
k__Bacteria|p__Firmicutes|c__Erysipelotrichia|o__Erysipelotrichales|f__Erysipelotrichaceae|g__Erysipelatoclostridium|s__Erysipelatoclostridium_ramosum	-	0.581	0.657	0.732	0.824	62.27	0.070
k__Bacteria|p__Firmicutes|c__Clostridia|o__Clostridiales|f__Ruminococcaceae|g__Faecalibacterium|s__Faecalibacterium_SGB15346	-	0.752	0.808	0.765	0.849	10.52	0.37
k__Bacteria|p__Proteobacteria|c__Gammaproteobacteria|o__Enterobacterales|f__Enterobacteriaceae|g__Escherichia|s__Escherichia_coli	-	0.632	0.696	0.770	0.849	62.84	0.97
k__Bacteria|p__Firmicutes|c__Clostridia|o__Clostridiales|f__Eubacteriaceae|g__Eubacterium|s__Eubacterium_ramulus	+	0.704	0.770	0.791	0.865	51.19	0.080
k__Bacteria|p__Firmicutes|c__Clostridia|o__Clostridiales|f__Lachnospiraceae|g__Mediterraneibacter|s__Ruminococcus_torques	-	0.579	0.657	0.824	0.893	83.88	0.71
k__Bacteria|p__Firmicutes|c__Clostridia|o__Clostridiales|f__Lachnospiraceae|g__Anaerostipes|s__Anaerostipes_hadrus	-	0.739	0.801	0.832	0.894	59.31	0.43
k__Bacteria|p__Firmicutes|c__Clostridia|o__Clostridiales|f__Lachnospiraceae|g__Lachnospiraceae_unclassified|s__Lachnospiraceae_bacterium	-	0.817	0.864	0.855	0.911	37.50	0.73
k__Bacteria|p__Bacteroidetes|c__Bacteroidia|o__Bacteroidales|f__Bacteroidaceae|g__Bacteroides|s__Bacteroides_thetaiotaomicron	-	0.773	0.824	0.871	0.921	68.54	1.00
k__Bacteria|p__Bacteroidetes|c__Bacteroidia|o__Bacteroidales|f__Bacteroidales_unclassified|g__Phocaeicola|s__Phocaeicola_dorei	+	0.887	0.915	0.905	0.940	29.73	2.10
k__Bacteria|p__Firmicutes|c__Negativicutes|o__Veillonellales|f__Veillonellaceae|g__Veillonella|s__Veillonella_parvula	+	0.909	0.930	0.911	0.940	0.00	0.080

k__Bacteria|p__Firmicutes|c__Clostridia|o__Clostridia_unclassified|f__Clostridia_unclassified|g__Clostridia_unclassified|s__Clostridia_unclassified_SGB4121	-	0.885	0.915	0.909	0.940	40.16	0.050
k__Bacteria|p__Firmicutes|c__Clostridia|o__Clostridiales|f__Lachnospiraceae|g__Anaerotignum|s__Anaerotignum_faecicola	-	0.881	0.915	0.930	0.952	65.82	0.10
k__Bacteria|p__Firmicutes|c__Clostridia|o__Clostridiales|f__Lachnospiraceae|g__Mediterraneibacter|s__Mediterraneibacter_butyricigenes	+	0.939	0.954	0.939	0.954	0.00	0.010
k__Bacteria|p__Firmicutes|c__Clostridia|o__Clostridiales|f__Ruminococcaceae|g__Gemmiger|s__Gemmiger_formicilis	-	0.989	0.994	0.994	0.994	60.30	0.62
k__Bacteria|p__Bacteroidetes|c__Bacteroidia|o__Bacteroidales|f__Bacteroidaceae|g__Bacteroides|s__Bacteroides_stercoris	+	0.994	0.994	0.993	0.994	0.00	2.72

**Genus**	**Increased or decreased in PD**	***p*-value (FEM)**	***q*-value (FEM)**	***p*-value (REM)**	***q*-value (REM)**	**I^2 (%)**	**Relative abundance (%)**
k__Bacteria|p__Firmicutes|c__Clostridia|o__Clostridia_unclassified|f__Clostridia_unclassified|g__Clostridia_unclassified	+	3.77E-15	*4.53E-14	3.77E-15	*3.17E-13	0.00	3.35
k__Bacteria|p__Firmicutes|c__Clostridia|o__Clostridiales|f__Ruminococcaceae|g__Anaerotruncus	+	< 1.0E-16	*< 1.0E-16	1.09E-13	*4.57E-12	34.17	0.046
k__Bacteria|p__Firmicutes|c__Clostridia|o__Clostridiales|f__Christensenellaceae|g__Christensenellaceae_unclassified	+	< 1.0E-16	*< 1.0E-16	2.24E-11	*6.28E-10	42.26	0.029
k__Bacteria|p__Firmicutes|c__Clostridia|o__Clostridiales|f__Ruminococcaceae|g__Ruminococcaceae_unclassified	+	< 1.0E-16	*< 1.0E-16	4.81E-09	*1.01E-07	64.55	2.38
k__Bacteria|p__Firmicutes|c__Clostridia|o__Clostridiales|f__Ruminococcaceae|g__Ruthenibacterium	+	< 1.0E-16	*< 1.0E-16	5.43E-08	*9.13E-07	62.97	0.25
k__Bacteria|p__Firmicutes|c__Bacilli|o__Bacilli_unclassified|f__Bacilli_unclassified|g__Bacilli_unclassified	+	1.51E-11	*1.15E-10	2.51E-06	*3.51E-05	51.32	0.76
k__Bacteria|p__Firmicutes|c__Clostridia|o__Clostridiales|f__Ruminococcaceae|g__GGB9699	+	2.65E-11	*1.86E-10	7.06E-06	*8.47E-05	54.57	0.20
k__Bacteria|p__Bacteroidetes|c__Bacteroidia|o__Bacteroidales|f__Rikenellaceae|g__Alistipes	+	3.97E-13	*3.33E-12	2.67E-05	*2.81E-04	66.51	4.58
k__Bacteria|p__Firmicutes|c__Clostridia|o__Clostridiales|f__Clostridiales_unclassified|g__Intestinimonas	+	< 1.0E-16	*< 1.0E-16	3.06E-05	*2.86E-04	78.96	0.064
k__Bacteria|p__Proteobacteria|c__Gammaproteobacteria|o__Pasteurellales|f__Pasteurellaceae|g__Haemophilus	-	3.14E-04	*9.09E-04	3.14E-04	*2.45E-03	0.00	0.079
k__Bacteria|p__Firmicutes|c__Clostridia|o__Clostridiales|f__Ruminococcaceae|g__Anaeromassilibacillus	+	6.35E-09	*4.10E-08	3.21E-04	*2.45E-03	61.63	0.014
k__Bacteria|p__Firmicutes|c__Clostridia|o__Clostridiales|f__Lachnospiraceae|g__Lachnoclostridium	+	1.47E-04	*4.58E-04	4.00E-04	*2.80E-03	12.99	0.11
k__Bacteria|p__Firmicutes|c__Clostridia|o__Clostridiales|f__Lachnospiraceae|g__Eisenbergiella	+	2.94E-13	*2.75E-12	4.86E-04	*3.03E-03	77.14	0.10
k__Bacteria|p__Firmicutes|c__Clostridia|o__Clostridiales|f__Lachnospiraceae|g__Lacrimispora	-	1.69E-08	*9.71E-08	5.05E-04	*3.03E-03	61.98	0.31
k__Bacteria|p__Firmicutes|c__Clostridia|o__Clostridiales|f__Ruminococcaceae|g__Faecalibacterium	-	1.13E-06	*4.98E-06	5.70E-04	*3.19E-03	49.90	3.67
k__Bacteria|p__Verrucomicrobia|c__Verrucomicrobiae|o__Verrucomicrobiales|f__Akkermansiaceae|g__Akkermansia	+	1.33E-14	*1.40E-13	6.09E-04	*3.20E-03	80.20	1.02
k__Bacteria|p__Firmicutes|c__Clostridia|o__Clostridiales|f__Lachnospiraceae|g__Roseburia	-	3.29E-08	*1.73E-07	6.67E-04	*3.21E-03	62.08	1.73
k__Bacteria|p__Firmicutes|c__Clostridia|o__Clostridiales|f__Ruminococcaceae|g__Agathobaculum	-	9.53E-07	*4.45E-06	6.88E-04	*3.21E-03	52.03	0.12
k__Bacteria|p__Bacteroidetes|c__Bacteroidia|o__Bacteroidales|f__Bacteroidales_unclassified|g__Phocaeicola	-	4.44E-16	*6.22E-15	8.05E-04	*3.56E-03	82.97	12.95
k__Bacteria|p__Actinobacteria|c__Actinobacteria|o__Bifidobacteriales|f__Bifidobacteriaceae|g__Bifidobacterium	+	6.82E-04	*1.69E-03	1.42E-03	*5.97E-03	11.79	3.42
k__Bacteria|p__Bacteroidetes|c__Bacteroidia|o__Bacteroidales|f__Barnesiellaceae|g__Barnesiella	+	4.68E-04	*1.27E-03	1.62E-03	*6.49E-03	18.83	0.41
k__Bacteria|p__Firmicutes|c__Clostridia|o__Clostridiales|f__Eubacteriaceae|g__Eubacteriaceae_unclassified	+	1.80E-03	*4.33E-03	3.48E-03	*0.013	12.36	0.087
k__Bacteria|p__Firmicutes|c__Clostridia|o__Clostridiales|f__Lachnospiraceae|g__Dorea	-	4.79E-03	*0.010	4.79E-03	*0.018	0.00	0.43
k__Bacteria|p__Actinobacteria|c__Coriobacteriia|o__Eggerthellales|f__Eggerthellaceae|g__Gordonibacter	+	1.73E-08	*9.71E-08	5.75E-03	*0.020	75.99	0.025
k__Bacteria|p__Proteobacteria|c__Deltaproteobacteria|o__Desulfovibrionales|f__Desulfovibrionaceae|g__Desulfovibrio	+	1.85E-06	*7.38E-06	5.85E-03	*0.020	66.61	0.070
k__Bacteria|p__Firmicutes|c__CFGB1217|o__OFGB1217|f__FGB1217|g__GGB2982	+	5.90E-04	*1.55E-03	8.02E-03	*0.026	40.46	0.010
k__Bacteria|p__Firmicutes|c__Clostridia|o__Clostridiales|f__Clostridiaceae|g__Hungatella	+	6.51E-05	*2.28E-04	8.39E-03	*0.026	56.43	0.051
k__Bacteria|p__Firmicutes|c__Clostridia|o__Clostridiales|f__Oscillospiraceae|g__Dysosmobacter	+	1.07E-04	*3.44E-04	8.96E-03	*0.027	54.51	0.37
k__Bacteria|p__Bacteroidetes|c__Bacteroidia|o__Bacteroidales|f__Tannerellaceae|g__Parabacteroides	+	1.82E-05	*6.91E-05	9.63E-03	*0.028	63.52	1.95
k__Bacteria|p__Proteobacteria|c__Deltaproteobacteria|o__Desulfovibrionales|f__Desulfovibrionaceae|g__Bilophila	+	4.34E-08	*2.15E-07	0.011	*0.030	78.28	0.20
k__Bacteria|p__Firmicutes|c__Erysipelotrichia|o__Erysipelotrichales|f__Erysipelotrichaceae|g__Holdemania	+	1.89E-05	*6.91E-05	0.011	*0.031	64.98	0.020
k__Bacteria|p__Firmicutes|c__Clostridia|o__Clostridiales|f__Ruminococcaceae|g__Massilimaliae	+	1.70E-06	*7.13E-06	0.032	0.085	80.01	4.2E-03
k__Bacteria|p__Proteobacteria|c__Betaproteobacteria|o__Burkholderiales|f__Sutterellaceae|g__Parasutterella	+	0.020	*0.036	0.037	0.095	19.17	0.24
k__Bacteria|p__Firmicutes|c__Clostridia|o__Clostridiales|f__Clostridiaceae|g__Clostridium	-	1.98E-03	*4.63E-03	0.041	0.101	56.44	1.80
k__Bacteria|p__Firmicutes|c__CFGB3053|o__OFGB3053|f__FGB3053|g__GGB9581	+	2.23E-04	*6.68E-04	0.042	0.101	69.66	0.027
k__Bacteria|p__Firmicutes|c__Clostridia|o__Clostridiales|f__Ruminococcaceae|g__GGB9632	+	8.81E-05	*2.96E-04	0.049	0.115	74.89	0.27
k__Bacteria|p__Firmicutes|c__Clostridia|o__Clostridiales|f__Ruminococcaceae|g__GGB9633	+	0.012	*0.022	0.056	0.128	42.59	0.46
k__Bacteria|p__Firmicutes|c__Clostridia|o__Clostridiales|f__Lachnospiraceae|g__Faecalicatena	+	6.80E-03	*0.014	0.060	0.132	51.56	0.095
k__Bacteria|p__Firmicutes|c__Clostridia|o__Clostridiales|f__Ruminococcaceae|g__GGB9707	+	2.47E-03	*5.60E-03	0.066	0.142	63.14	0.032
k__Bacteria|p__Firmicutes|c__Clostridia|o__Clostridiales|f__Lachnospiraceae|g__Enterocloster	+	0.023	*0.038	0.073	0.153	37.63	0.24
k__Bacteria|p__Firmicutes|c__Clostridia|o__Clostridiales|f__Lachnospiraceae|g__Mediterraneibacter	-	4.66E-04	*1.27E-03	0.080	0.165	75.04	1.30
k__Bacteria|p__Firmicutes|c__Clostridia|o__Clostridiales|f__Lachnospiraceae|g__Lachnospira	-	0.087	0.122	0.108	0.215	11.67	1.07
k__Bacteria|p__Firmicutes|c__Clostridia|o__Clostridiales|f__Lachnospiraceae|g__Blautia	-	3.18E-03	*7.02E-03	0.114	0.222	71.25	2.58
k__Bacteria|p__Firmicutes|c__Clostridia|o__Clostridiales|f__Ruminococcaceae|g__GGB9615	+	9.93E-03	*0.019	0.124	0.236	64.33	0.32
k__Bacteria|p__Bacteroidetes|c__Bacteroidia|o__Bacteroidales|f__Odoribacteraceae|g__Odoribacter	+	4.71E-03	*0.010	0.126	0.236	70.76	0.32
k__Bacteria|p__Firmicutes|c__Clostridia|o__Clostridiales|f__Lachnospiraceae|g__GGB3571	+	7.92E-03	*0.015	0.131	0.239	67.67	6.1E-03
k__Bacteria|p__Actinobacteria|c__Coriobacteriia|o__Eggerthellales|f__Eggerthellaceae|g__Eggerthellaceae_unclassified	+	0.028	*0.046	0.141	0.248	54.85	0.055
k__Bacteria|p__Firmicutes|c__Clostridia|o__Clostridiales|f__Ruminococcaceae|g__Candidatus_Cibiobacter	+	5.79E-03	*0.012	0.142	0.248	71.68	0.58
k__Bacteria|p__Firmicutes|c__Erysipelotrichia|o__Erysipelotrichales|f__Erysipelotrichaceae|g__Erysipelatoclostridium	+	0.036	0.057	0.160	0.275	55.19	0.12
k__Bacteria|p__Firmicutes|c__Clostridia|o__Clostridiales|f__Oscillospiraceae|g__Oscillibacter	+	0.016	*0.029	0.168	0.277	67.05	0.46
k__Bacteria|p__Firmicutes|c__Clostridia|o__Clostridiales|f__Lachnospiraceae|g__Anaerobutyricum	+	0.069	0.101	0.168	0.277	42.52	0.33
k__Bacteria|p__Bacteroidetes|c__Bacteroidia|o__Bacteroidales|f__Odoribacteraceae|g__Butyricimonas	+	6.64E-04	*1.69E-03	0.174	0.279	84.08	0.19
k__Bacteria|p__Firmicutes|c__Clostridia|o__Clostridia_unclassified|f__Clostridia_unclassified|g__Candidatus_Avimicrobium	+	0.064	0.096	0.176	0.279	46.59	0.030
k__Bacteria|p__Firmicutes|c__Clostridia|o__Clostridiales|f__Clostridiales_unclassified|g__Evtepia	+	0.021	*0.036	0.234	0.365	73.53	0.080
k__Bacteria|p__Firmicutes|c__Clostridia|o__Clostridiales|f__Lachnospiraceae|g__Lachnospiraceae_unclassified	-	0.251	0.325	0.251	0.384	0.00	2.47
k__Bacteria|p__Firmicutes|c__Negativicutes|o__Veillonellales|f__Veillonellaceae|g__Veillonella	-	0.073	0.103	0.292	0.437	65.48	0.21
k__Bacteria|p__Firmicutes|c__Clostridia|o__Clostridiales|f__Lachnospiraceae|g__GGB3746	-	0.015	*0.027	0.302	0.445	81.96	0.21
k__Bacteria|p__Bacteroidetes|c__Bacteroidia|o__Bacteroidales|f__Prevotellaceae|g__Prevotella	-	0.070	0.101	0.313	0.453	69.04	7.93
k__Bacteria|p__Firmicutes|c__Clostridia|o__Clostridiales|f__Clostridiaceae|g__Clostridiaceae_unclassified	-	0.052	0.079	0.338	0.479	75.77	0.30
k__Bacteria|p__Actinobacteria|c__Coriobacteriia|o__Eggerthellales|f__Eggerthellaceae|g__Adlercreutzia	+	0.022	*0.037	0.342	0.479	82.84	0.06
k__Bacteria|p__Firmicutes|c__Clostridia|o__Clostridiales|f__Lachnospiraceae|g__Fusicatenibacter	-	0.040	0.062	0.353	0.487	79.65	0.68
k__Bacteria|p__Bacteroidetes|c__Bacteroidia|o__Bacteroidales|f__Barnesiellaceae|g__Coprobacter	+	0.362	0.454	0.362	0.491	0.00	0.086
k__Bacteria|p__Firmicutes|c__Erysipelotrichia|o__Erysipelotrichales|f__Erysipelotrichaceae|g__Faecalibacillus	+	0.175	0.241	0.383	0.511	58.71	0.14
k__Bacteria|p__Firmicutes|c__Clostridia|o__Clostridiales|f__Lachnospiraceae|g__Anaerostipes	-	0.197	0.263	0.427	0.56	62.08	0.45
k__Bacteria|p__Firmicutes|c__Clostridia|o__Clostridiales|f__Peptostreptococcaceae|g__Romboutsia	+	0.235	0.309	0.532	0.685	72.26	0.059
k__Bacteria|p__Actinobacteria|c__Coriobacteriia|o__Eggerthellales|f__Eggerthellaceae|g__Eggerthella	+	0.525	0.596	0.538	0.685	6.20	0.12
k__Bacteria|p__Firmicutes|c__Negativicutes|o__Veillonellales|f__Veillonellaceae|g__Dialister	+	0.447	0.521	0.574	0.719	45.26	0.59
k__Bacteria|p__Firmicutes|c__Clostridia|o__Clostridiales|f__Ruminococcaceae|g__Ruminococcus	-	0.588	0.650	0.605	0.745	9.04	2.50
k__Bacteria|p__Firmicutes|c__Clostridia|o__Clostridiales|f__Clostridiales_unclassified|g__Clostridiales_unclassified	-	0.397	0.476	0.612	0.745	64.13	0.32
k__Bacteria|p__Firmicutes|c__Clostridia|o__Clostridiales|f__Ruminococcaceae|g__Phocea	+	0.387	0.472	0.627	0.751	68.41	0.013

k__Bacteria|p__Proteobacteria|c__Betaproteobacteria|o__Burkholderiales|f__Sutterellaceae|g__Sutterella	-	0.549	0.615	0.639	0.751	38.71	0.61
k__Bacteria|p__Firmicutes|c__Clostridia|o__Clostridiales|f__Eubacteriaceae|g__Eubacterium	+	0.195	0.263	0.644	0.751	87.28	0.46
k__Bacteria|p__Actinobacteria|c__Coriobacteriia|o__Coriobacteriales|f__Coriobacteriaceae|g__Collinsella	+	0.430	0.509	0.659	0.759	68.77	0.67
k__Bacteria|p__Firmicutes|c__Firmicutes_unclassified|o__Firmicutes_unclassified|f__Firmicutes_unclassified|g__Firmicutes_uncl	+	0.457	0.526	0.686	0.779	70.49	0.15
k__Bacteria|p__Firmicutes|c__Clostridia|o__Clostridiales|f__Ruminococcaceae|g__GGB9619	+	0.340	0.433	0.697	0.781	83.37	0.013
k__Bacteria|p__Bacteroidetes|c__Bacteroidia|o__Bacteroidales|f__Bacteroidaceae|g__Bacteroides	-	0.736	0.783	0.736	0.814	0.00	14.95
k__Bacteria|p__Proteobacteria|c__Gammaproteobacteria|o__Enterobacterales|f__Enterobacteriaceae|g__Escherichia	-	0.624	0.681	0.766	0.828	63.08	0.97
k__Bacteria|p__Firmicutes|c__Bacilli|o__Lactobacillales|f__Streptococcaceae|g__Streptococcus	+	0.384	0.472	0.769	0.828	88.65	0.84
k__Bacteria|p__Firmicutes|c__Negativicutes|o__Acidaminococcales|f__Acidaminococcaceae|g__Phascolarctobacterium	+	0.829	0.859	0.842	0.895	15.40	0.82
k__Bacteria|p__Bacteroidetes|c__Bacteroidia|o__Bacteroidales|f__Prevotellaceae|g__Paraprevotella	+	0.657	0.707	0.864	0.907	85.09	0.25
k__Bacteria|p__Firmicutes|c__Clostridia|o__Clostridiales|f__Ruminococcaceae|g__Flavonifractor	-	0.870	0.881	0.896	0.918	35.65	0.31
k__Bacteria|p__Firmicutes|c__Clostridia|o__Clostridiales|f__Ruminococcaceae|g__Gemmiger	+	0.850	0.870	0.896	0.918	52.36	0.73
k__Bacteria|p__Firmicutes|c__Clostridia|o__Clostridiales|f__Lachnospiraceae|g__Anaerotignum	-	0.772	0.811	0.907	0.918	83.83	0.13
k__Bacteria|p__Firmicutes|c__Clostridia|o__Clostridiales|f__Lachnospiraceae|g__Coprococcus	+	0.932	0.932	0.956	0.956	57.29	0.51

False discovery rates (q-values) were calculated for the 129 species and the 84 genera using the Banjamini-Hochberg method.

**q* -value (FEM, REM) < 0.05

Supplementary Table 8. Confounding factor analysis of the species and the genera that were significantly changed in meta-analysis of six datasets

Species	Confounding factors	Beta coefficient ± SE	Increase or decrease	*p*- value	*q*- value
k__Bacteria.p__Actinobacteria.c__Actinobacteria.o__Bifidobacteriales.f__Bifidobacteriaceae.g__Bifidobacterium.s__Bifidobacterium_longum	age	-0.136 ± 0.065	-	0.0369	
k__Bacteria.p__Actinobacteria.c__Actinobacteria.o__Bifidobacteriales.f__Bifidobacteriaceae.g__Bifidobacterium.s__Bifidobacterium_longum	BMI	-0.228 ± 0.072	-	1.50E-03	
k__Bacteria.p__Actinobacteria.c__Actinobacteria.o__Bifidobacteriales.f__Bifidobacteriaceae.g__Bifidobacterium.s__Bifidobacterium_longum	constipation	0.140 ± 0.064	+	0.0291	
k__Bacteria.p__Actinobacteria.c__Actinobacteria.o__Bifidobacteriales.f__Bifidobacteriaceae.g__Bifidobacterium.s__Bifidobacterium_longum	disease	0.665 ± 0.145	+	4.22E-06	*4.23E-05
k__Bacteria.p__Actinobacteria.c__Actinobacteria.o__Bifidobacteriales.f__Bifidobacteriaceae.g__Bifidobacterium.s__Bifidobacterium_longum	sex	0.245 ± 0.062	+	6.94E-05	
k__Bacteria.p__Actinobacteria.c__Actinobacteria.o__Bifidobacteriales.f__Bifidobacteriaceae.g__Bifidobacterium.s__Bifidobacterium_longum	anticholesterol drugs	0.000 ± 0.065	+	0.996	
k__Bacteria.p__Bacteroidetes.c__Bacteroidia.o__Bacteroidales.f__Bacteroidaceae.g__Bacteroides.s__Bacteroides_cellulosilyticus	age	0.205 ± 0.090	+	0.0236	
k__Bacteria.p__Bacteroidetes.c__Bacteroidia.o__Bacteroidales.f__Bacteroidaceae.g__Bacteroides.s__Bacteroides_cellulosilyticus	BMI	-0.136 ± 0.091	-	0.137	
k__Bacteria.p__Bacteroidetes.c__Bacteroidia.o__Bacteroidales.f__Bacteroidaceae.g__Bacteroides.s__Bacteroides_cellulosilyticus	constipation	-0.142 ± 0.086	-	0.101	
k__Bacteria.p__Bacteroidetes.c__Bacteroidia.o__Bacteroidales.f__Bacteroidaceae.g__Bacteroides.s__Bacteroides_cellulosilyticus	disease	0.249 ± 0.185	+	0.178	0.282
k__Bacteria.p__Bacteroidetes.c__Bacteroidia.o__Bacteroidales.f__Bacteroidaceae.g__Bacteroides.s__Bacteroides_cellulosilyticus	sex	0.285 ± 0.086	+	8.86E-04	
k__Bacteria.p__Bacteroidetes.c__Bacteroidia.o__Bacteroidales.f__Bacteroidaceae.g__Bacteroides.s__Bacteroides_cellulosilyticus	anticholesterol drugs	-0.093 ± 0.085	-	0.272	
k__Bacteria.p__Bacteroidetes.c__Bacteroidia.o__Bacteroidales.f__Bacteroidales_unclassified.g__Phocaeicola.s__Phocaeicola_vulgatus	age	-0.065 ± 0.047	-	0.167	
k__Bacteria.p__Bacteroidetes.c__Bacteroidia.o__Bacteroidales.f__Bacteroidales_unclassified.g__Phocaeicola.s__Phocaeicola_vulgatus	BMI	0.047 ± 0.049	+	0.340	
k__Bacteria.p__Bacteroidetes.c__Bacteroidia.o__Bacteroidales.f__Bacteroidales_unclassified.g__Phocaeicola.s__Phocaeicola_vulgatus	constipation	-0.147 ± 0.048	-	2.20E-03	
k__Bacteria.p__Bacteroidetes.c__Bacteroidia.o__Bacteroidales.f__Bacteroidales_unclassified.g__Phocaeicola.s__Phocaeicola_vulgatus	disease	-0.097 ± 0.103	-	0.346	0.453
k__Bacteria.p__Bacteroidetes.c__Bacteroidia.o__Bacteroidales.f__Bacteroidales_unclassified.g__Phocaeicola.s__Phocaeicola_vulgatus	sex	0.017 ± 0.047	+	0.710	
k__Bacteria.p__Bacteroidetes.c__Bacteroidia.o__Bacteroidales.f__Bacteroidales_unclassified.g__Phocaeicola.s__Phocaeicola_vulgatus	anticholesterol drugs	-0.011 ± 0.047	-	0.812	
k__Bacteria.p__Bacteroidetes.c__Bacteroidia.o__Bacteroidales.f__Barnesiellaceae.g__Barnesiella.s__Barnesiella_intestinihominis	age	0.121 ± 0.095	+	0.201	
k__Bacteria.p__Bacteroidetes.c__Bacteroidia.o__Bacteroidales.f__Barnesiellaceae.g__Barnesiella.s__Barnesiella_intestinihominis	BMI	-0.020 ± 0.095	-	0.832	
k__Bacteria.p__Bacteroidetes.c__Bacteroidia.o__Bacteroidales.f__Barnesiellaceae.g__Barnesiella.s__Barnesiella_intestinihominis	constipation	-0.001 ± 0.093	-	0.989	
k__Bacteria.p__Bacteroidetes.c__Bacteroidia.o__Bacteroidales.f__Barnesiellaceae.g__Barnesiella.s__Barnesiella_intestinihominis	disease	0.172 ± 0.202	+	0.396	0.494
k__Bacteria.p__Bacteroidetes.c__Bacteroidia.o__Bacteroidales.f__Barnesiellaceae.g__Barnesiella.s__Barnesiella_intestinihominis	sex	-0.006 ± 0.090	-	0.951	
k__Bacteria.p__Bacteroidetes.c__Bacteroidia.o__Bacteroidales.f__Barnesiellaceae.g__Barnesiella.s__Barnesiella_intestinihominis	anticholesterol drugs	0.106 ± 0.089	+	0.237	
k__Bacteria.p__Bacteroidetes.c__Bacteroidia.o__Bacteroidales.f__Rikenellaceae.g__Alistipes.s__Alistipes_communis	age	0.118 ± 0.073	+	0.107	
k__Bacteria.p__Bacteroidetes.c__Bacteroidia.o__Bacteroidales.f__Rikenellaceae.g__Alistipes.s__Alistipes_communis	BMI	-0.097 ± 0.078	-	0.217	
k__Bacteria.p__Bacteroidetes.c__Bacteroidia.o__Bacteroidales.f__Rikenellaceae.g__Alistipes.s__Alistipes_communis	constipation	-0.013 ± 0.074	-	0.855	
k__Bacteria.p__Bacteroidetes.c__Bacteroidia.o__Bacteroidales.f__Rikenellaceae.g__Alistipes.s__Alistipes_communis	disease	0.299 ± 0.166	+	0.0707	0.122
k__Bacteria.p__Bacteroidetes.c__Bacteroidia.o__Bacteroidales.f__Rikenellaceae.g__Alistipes.s__Alistipes_communis	sex	-0.262 ± 0.073	-	3.59E-04	
k__Bacteria.p__Bacteroidetes.c__Bacteroidia.o__Bacteroidales.f__Rikenellaceae.g__Alistipes.s__Alistipes_communis	anticholesterol drugs	0.151 ± 0.073	+	0.0389	
k__Bacteria.p__Bacteroidetes.c__Bacteroidia.o__Bacteroidales.f__Rikenellaceae.g__Alistipes.s__Alistipes_ihumii	age	-0.112 ± 0.089	-	0.212	
k__Bacteria.p__Bacteroidetes.c__Bacteroidia.o__Bacteroidales.f__Rikenellaceae.g__Alistipes.s__Alistipes_ihumii	BMI	-0.160 ± 0.092	-	0.0799	
k__Bacteria.p__Bacteroidetes.c__Bacteroidia.o__Bacteroidales.f__Rikenellaceae.g__Alistipes.s__Alistipes_ihumii	constipation	0.369 ± 0.086	+	1.63E-05	
k__Bacteria.p__Bacteroidetes.c__Bacteroidia.o__Bacteroidales.f__Rikenellaceae.g__Alistipes.s__Alistipes_ihumii	disease	0.174 ± 0.208	+	0.403	0.494
k__Bacteria.p__Bacteroidetes.c__Bacteroidia.o__Bacteroidales.f__Rikenellaceae.g__Alistipes.s__Alistipes_ihumii	sex	-0.237 ± 0.093	-	0.0107	
k__Bacteria.p__Bacteroidetes.c__Bacteroidia.o__Bacteroidales.f__Rikenellaceae.g__Alistipes.s__Alistipes_ihumii	anticholesterol drugs	0.003 ± 0.091	+	0.971	
k__Bacteria.p__Bacteroidetes.c__Bacteroidia.o__Bacteroidales.f__Rikenellaceae.g__Alistipes.s__Alistipes_indistinctus	age	0.058 ± 0.084	+	0.495	
k__Bacteria.p__Bacteroidetes.c__Bacteroidia.o__Bacteroidales.f__Rikenellaceae.g__Alistipes.s__Alistipes_indistinctus	BMI	-0.141 ± 0.092	-	0.128	
k__Bacteria.p__Bacteroidetes.c__Bacteroidia.o__Bacteroidales.f__Rikenellaceae.g__Alistipes.s__Alistipes_indistinctus	constipation	0.189 ± 0.077	+	0.0135	
k__Bacteria.p__Bacteroidetes.c__Bacteroidia.o__Bacteroidales.f__Rikenellaceae.g__Alistipes.s__Alistipes_indistinctus	disease	0.575 ± 0.184	+	1.79E-03	*6.19E-03
k__Bacteria.p__Bacteroidetes.c__Bacteroidia.o__Bacteroidales.f__Rikenellaceae.g__Alistipes.s__Alistipes_indistinctus	sex	-0.233 ± 0.082	-	4.31E-03	
k__Bacteria.p__Bacteroidetes.c__Bacteroidia.o__Bacteroidales.f__Rikenellaceae.g__Alistipes.s__Alistipes_indistinctus	anticholesterol drugs	0.069 ± 0.080	+	0.390	
k__Bacteria.p__Bacteroidetes.c__Bacteroidia.o__Bacteroidales.f__Rikenellaceae.g__Alistipes.s__Alistipes_onderdonkii	age	0.212 ± 0.065	+	1.21E-03	
k__Bacteria.p__Bacteroidetes.c__Bacteroidia.o__Bacteroidales.f__Rikenellaceae.g__Alistipes.s__Alistipes_onderdonkii	BMI	-0.161 ± 0.069	-	0.0189	
k__Bacteria.p__Bacteroidetes.c__Bacteroidia.o__Bacteroidales.f__Rikenellaceae.g__Alistipes.s__Alistipes_onderdonkii	constipation	0.178 ± 0.062	+	3.82E-03	
k__Bacteria.p__Bacteroidetes.c__Bacteroidia.o__Bacteroidales.f__Rikenellaceae.g__Alistipes.s__Alistipes_onderdonkii	disease	0.281 ± 0.140	+	0.0445	0.0805
k__Bacteria.p__Bacteroidetes.c__Bacteroidia.o__Bacteroidales.f__Rikenellaceae.g__Alistipes.s__Alistipes_onderdonkii	sex	0.156 ± 0.065	+	0.0173	
k__Bacteria.p__Bacteroidetes.c__Bacteroidia.o__Bacteroidales.f__Rikenellaceae.g__Alistipes.s__Alistipes_onderdonkii	anticholesterol drugs	0.027 ± 0.064	+	0.673	
k__Bacteria.p__Bacteroidetes.c__Bacteroidia.o__Bacteroidales.f__Rikenellaceae.g__Alistipes.s__Alistipes_putredinis	age	-0.054 ± 0.052	-	0.302	
k__Bacteria.p__Bacteroidetes.c__Bacteroidia.o__Bacteroidales.f__Rikenellaceae.g__Alistipes.s__Alistipes_putredinis	BMI	0.000 ± 0.058	+	0.997	
k__Bacteria.p__Bacteroidetes.c__Bacteroidia.o__Bacteroidales.f__Rikenellaceae.g__Alistipes.s__Alistipes_putredinis	constipation	-0.038 ± 0.051	-	0.452	
k__Bacteria.p__Bacteroidetes.c__Bacteroidia.o__Bacteroidales.f__Rikenellaceae.g__Alistipes.s__Alistipes_putredinis	disease	0.030 ± 0.111	+	0.786	0.837
k__Bacteria.p__Bacteroidetes.c__Bacteroidia.o__Bacteroidales.f__Rikenellaceae.g__Alistipes.s__Alistipes_putredinis	sex	0.001 ± 0.051	+	0.978	
k__Bacteria.p__Bacteroidetes.c__Bacteroidia.o__Bacteroidales.f__Rikenellaceae.g__Alistipes.s__Alistipes_putredinis	anticholesterol drugs	0.008 ± 0.052	+	0.871	
k__Bacteria.p__Bacteroidetes.c__Bacteroidia.o__Bacteroidales.f__Rikenellaceae.g__Alistipes.s__Alistipes_shahii	age	0.002 ± 0.055	+	0.977	
k__Bacteria.p__Bacteroidetes.c__Bacteroidia.o__Bacteroidales.f__Rikenellaceae.g__Alistipes.s__Alistipes_shahii	BMI	0.048 ± 0.054	+	0.380	
k__Bacteria.p__Bacteroidetes.c__Bacteroidia.o__Bacteroidales.f__Rikenellaceae.g__Alistipes.s__Alistipes_shahii	constipation	0.038 ± 0.055	+	0.488	
k__Bacteria.p__Bacteroidetes.c__Bacteroidia.o__Bacteroidales.f__Rikenellaceae.g__Alistipes.s__Alistipes_shahii	disease	0.077 ± 0.122	+	0.529	0.609
k__Bacteria.p__Bacteroidetes.c__Bacteroidia.o__Bacteroidales.f__Rikenellaceae.g__Alistipes.s__Alistipes_shahii	sex	-0.065 ± 0.056	-	0.251	
k__Bacteria.p__Bacteroidetes.c__Bacteroidia.o__Bacteroidales.f__Rikenellaceae.g__Alistipes.s__Alistipes_shahii	anticholesterol drugs	0.034 ± 0.055	+	0.542	
k__Bacteria.p__Bacteroidetes.c__Bacteroidia.o__Bacteroidales.f__Tannerellaceae.g__Parabacteroides.s__Parabacteroides_merdae	age	-0.027 ± 0.044	-	0.548	
k__Bacteria.p__Bacteroidetes.c__Bacteroidia.o__Bacteroidales.f__Tannerellaceae.g__Parabacteroides.s__Parabacteroides_merdae	BMI	0.017 ± 0.045	+	0.704	

k__Bacteria.p__Bacteroidetes.c__Bacteroidia.o__Bacteroidales.f__Tannerellaceae.g__Parabacteroides.s__Parabacteroides_merdae	constipation	0.014 ± 0.047	+	0.769	
k__Bacteria.p__Bacteroidetes.c__Bacteroidia.o__Bacteroidales.f__Tannerellaceae.g__Parabacteroides.s__Parabacteroides_merdae	disease	0.007 ± 0.100	+	0.945	0.967
k__Bacteria.p__Bacteroidetes.c__Bacteroidia.o__Bacteroidales.f__Tannerellaceae.g__Parabacteroides.s__Parabacteroides_merdae	sex	-0.016 ± 0.045	-	0.718	
k__Bacteria.p__Bacteroidetes.c__Bacteroidia.o__Bacteroidales.f__Tannerellaceae.g__Parabacteroides.s__Parabacteroides_merdae	anticholesterol drugs	-0.089 ± 0.047	-	0.0574	
k__Bacteria.p__Firmicutes.c__CFGB1217.o__OFGB1217.f__FGB1217.g__GGB2982.s__GGB2982_SGB3964	age	-0.075 ± 0.072	-	0.295	
k__Bacteria.p__Firmicutes.c__CFGB1217.o__OFGB1217.f__FGB1217.g__GGB2982.s__GGB2982_SGB3964	BMI	-0.009 ± 0.078	-	0.905	
k__Bacteria.p__Firmicutes.c__CFGB1217.o__OFGB1217.f__FGB1217.g__GGB2982.s__GGB2982_SGB3964	constipation	-0.003 ± 0.077	-	0.973	
k__Bacteria.p__Firmicutes.c__CFGB1217.o__OFGB1217.f__FGB1217.g__GGB2982.s__GGB2982_SGB3964	disease	-0.174 ± 0.164	-	0.290	0.408
k__Bacteria.p__Firmicutes.c__CFGB1217.o__OFGB1217.f__FGB1217.g__GGB2982.s__GGB2982_SGB3964	sex	-0.160 ± 0.076	-	0.0348	
k__Bacteria.p__Firmicutes.c__CFGB1217.o__OFGB1217.f__FGB1217.g__GGB2982.s__GGB2982_SGB3964	anticholesterol drugs	0.006 ± 0.075	+	0.935	
k__Bacteria.p__Firmicutes.c__Clostridia.o__Clostridia_unclassified.f__Clostridia_unclassified.g__Clostridia_unclassified.s__Clostridia_bacterium	age	0.166 ± 0.040	+	3.64E-05	
k__Bacteria.p__Firmicutes.c__Clostridia.o__Clostridia_unclassified.f__Clostridia_unclassified.g__Clostridia_unclassified.s__Clostridia_bacterium	BMI	0.003 ± 0.041	+	0.944	
k__Bacteria.p__Firmicutes.c__Clostridia.o__Clostridia_unclassified.f__Clostridia_unclassified.g__Clostridia_unclassified.s__Clostridia_bacterium	constipation	0.181 ± 0.037	+	1.17E-06	
k__Bacteria.p__Firmicutes.c__Clostridia.o__Clostridia_unclassified.f__Clostridia_unclassified.g__Clostridia_unclassified.s__Clostridia_bacterium	disease	0.285 ± 0.088	+	1.15E-03	*4.88E-03
k__Bacteria.p__Firmicutes.c__Clostridia.o__Clostridia_unclassified.f__Clostridia_unclassified.g__Clostridia_unclassified.s__Clostridia_bacterium	sex	0.046 ± 0.039	+	0.240	
k__Bacteria.p__Firmicutes.c__Clostridia.o__Clostridia_unclassified.f__Clostridia_unclassified.g__Clostridia_unclassified.s__Clostridia_bacterium	anticholesterol drugs	-0.020 ± 0.038	-	0.608	
k__Bacteria.p__Firmicutes.c__Clostridia.o__Clostridiales.f__Christensenellaceae.g__Christensenellaceae_unclassified.s__Christensenellaceae_bacterium	age	0.150 ± 0.068	+	0.0272	
k__Bacteria.p__Firmicutes.c__Clostridia.o__Clostridiales.f__Christensenellaceae.g__Christensenellaceae_unclassified.s__Christensenellaceae_bacterium	BMI	-0.253 ± 0.065	-	1.08E-04	
k__Bacteria.p__Firmicutes.c__Clostridia.o__Clostridiales.f__Christensenellaceae.g__Christensenellaceae_unclassified.s__Christensenellaceae_bacterium	constipation	0.219 ± 0.062	+	4.12E-04	
k__Bacteria.p__Firmicutes.c__Clostridia.o__Clostridiales.f__Christensenellaceae.g__Christensenellaceae_unclassified.s__Christensenellaceae_bacterium	disease	0.499 ± 0.145	+	5.67E-04	*2.91E-03
k__Bacteria.p__Firmicutes.c__Clostridia.o__Clostridiales.f__Christensenellaceae.g__Christensenellaceae_unclassified.s__Christensenellaceae_bacterium	sex	0.096 ± 0.064	+	0.136	
k__Bacteria.p__Firmicutes.c__Clostridia.o__Clostridiales.f__Christensenellaceae.g__Christensenellaceae_unclassified.s__Christensenellaceae_bacterium	anticholesterol drugs	0.242 ± 0.062	+	1.03E-04	
k__Bacteria.p__Firmicutes.c__Clostridia.o__Clostridiales.f__Clostridiaceae.g__Hungatella.s__Hungatella_hathewayi	age	0.079 ± 0.072	+	0.269	
k__Bacteria.p__Firmicutes.c__Clostridia.o__Clostridiales.f__Clostridiaceae.g__Hungatella.s__Hungatella_hathewayi	BMI	-0.101 ± 0.079	-	0.201	
k__Bacteria.p__Firmicutes.c__Clostridia.o__Clostridiales.f__Clostridiaceae.g__Hungatella.s__Hungatella_hathewayi	constipation	0.451 ± 0.069	+	8.02E-11	
k__Bacteria.p__Firmicutes.c__Clostridia.o__Clostridiales.f__Clostridiaceae.g__Hungatella.s__Hungatella_hathewayi	disease	0.399 ± 0.163	+	0.0144	*0.0316
k__Bacteria.p__Firmicutes.c__Clostridia.o__Clostridiales.f__Clostridiaceae.g__Hungatella.s__Hungatella_hathewayi	sex	0.171 ± 0.070	+	0.0145	
k__Bacteria.p__Firmicutes.c__Clostridia.o__Clostridiales.f__Clostridiaceae.g__Hungatella.s__Hungatella_hathewayi	anticholesterol drugs	-0.037 ± 0.071	-	0.599	
k__Bacteria.p__Firmicutes.c__Clostridia.o__Clostridiales.f__Clostridiales_unclassified.g__Intestinimonas.s__Intestinimonas_butyriciproducens	age	0.137 ± 0.072	+	0.0573	
k__Bacteria.p__Firmicutes.c__Clostridia.o__Clostridiales.f__Clostridiales_unclassified.g__Intestinimonas.s__Intestinimonas_butyriciproducens	BMI	-0.144 ± 0.076	-	0.0601	
k__Bacteria.p__Firmicutes.c__Clostridia.o__Clostridiales.f__Clostridiales_unclassified.g__Intestinimonas.s__Intestinimonas_butyriciproducens	constipation	0.104 ± 0.075	+	0.165	
k__Bacteria.p__Firmicutes.c__Clostridia.o__Clostridiales.f__Clostridiales_unclassified.g__Intestinimonas.s__Intestinimonas_butyriciproducens	disease	0.176 ± 0.173	+	0.307	0.417
k__Bacteria.p__Firmicutes.c__Clostridia.o__Clostridiales.f__Clostridiales_unclassified.g__Intestinimonas.s__Intestinimonas_butyriciproducens	sex	0.230 ± 0.078	+	3.38E-03	
k__Bacteria.p__Firmicutes.c__Clostridia.o__Clostridiales.f__Clostridiales_unclassified.g__Intestinimonas.s__Intestinimonas_butyriciproducens	anticholesterol drugs	0.191 ± 0.077	+	0.0131	
k__Bacteria.p__Firmicutes.c__Clostridia.o__Clostridiales.f__Lachnospiraceae.g__Blautia.s__Blautia_wexlerae	age	-0.034 ± 0.041	-	0.400	
k__Bacteria.p__Firmicutes.c__Clostridia.o__Clostridiales.f__Lachnospiraceae.g__Blautia.s__Blautia_wexlerae	BMI	0.011 ± 0.047	+	0.818	
k__Bacteria.p__Firmicutes.c__Clostridia.o__Clostridiales.f__Lachnospiraceae.g__Blautia.s__Blautia_wexlerae	constipation	-0.114 ± 0.043	-	8.20E-03	
k__Bacteria.p__Firmicutes.c__Clostridia.o__Clostridiales.f__Lachnospiraceae.g__Blautia.s__Blautia_wexlerae	disease	-0.281 ± 0.092	-	2.30E-03	*6.73E-03
k__Bacteria.p__Firmicutes.c__Clostridia.o__Clostridiales.f__Lachnospiraceae.g__Blautia.s__Blautia_wexlerae	sex	0.086 ± 0.041	+	0.0381	
k__Bacteria.p__Firmicutes.c__Clostridia.o__Clostridiales.f__Lachnospiraceae.g__Blautia.s__Blautia_wexlerae	anticholesterol drugs	0.066 ± 0.043	+	0.124	
k__Bacteria.p__Firmicutes.c__Clostridia.o__Clostridiales.f__Lachnospiraceae.g__Enterocloster.s__Enterocloster_asparagiformis	age	0.080 ± 0.088	+	0.364	
k__Bacteria.p__Firmicutes.c__Clostridia.o__Clostridiales.f__Lachnospiraceae.g__Enterocloster.s__Enterocloster_asparagiformis	BMI	0.133 ± 0.094	+	0.156	
k__Bacteria.p__Firmicutes.c__Clostridia.o__Clostridiales.f__Lachnospiraceae.g__Enterocloster.s__Enterocloster_asparagiformis	constipation	-0.082 ± 0.083	-	0.322	
k__Bacteria.p__Firmicutes.c__Clostridia.o__Clostridiales.f__Lachnospiraceae.g__Enterocloster.s__Enterocloster_asparagiformis	disease	0.381 ± 0.186	+	0.0404	0.0767
k__Bacteria.p__Firmicutes.c__Clostridia.o__Clostridiales.f__Lachnospiraceae.g__Enterocloster.s__Enterocloster_asparagiformis	sex	0.265 ± 0.086	+	1.99E-03	
k__Bacteria.p__Firmicutes.c__Clostridia.o__Clostridiales.f__Lachnospiraceae.g__Enterocloster.s__Enterocloster_asparagiformis	anticholesterol drugs	0.040 ± 0.085	+	0.637	
k__Bacteria.p__Firmicutes.c__Clostridia.o__Clostridiales.f__Lachnospiraceae.g__Faecalicatena.s__Faecalicatena_contorta	age	0.143 ± 0.052	+	5.93E-03	
k__Bacteria.p__Firmicutes.c__Clostridia.o__Clostridiales.f__Lachnospiraceae.g__Faecalicatena.s__Faecalicatena_contorta	BMI	0.003 ± 0.048	+	0.944	
k__Bacteria.p__Firmicutes.c__Clostridia.o__Clostridiales.f__Lachnospiraceae.g__Faecalicatena.s__Faecalicatena_contorta	constipation	0.270 ± 0.050	+	6.02E-08	
k__Bacteria.p__Firmicutes.c__Clostridia.o__Clostridiales.f__Lachnospiraceae.g__Faecalicatena.s__Faecalicatena_contorta	disease	0.148 ± 0.116	+	0.204	0.309
k__Bacteria.p__Firmicutes.c__Clostridia.o__Clostridiales.f__Lachnospiraceae.g__Faecalicatena.s__Faecalicatena_contorta	sex	0.160 ± 0.053	+	2.38E-03	
k__Bacteria.p__Firmicutes.c__Clostridia.o__Clostridiales.f__Lachnospiraceae.g__Faecalicatena.s__Faecalicatena_contorta	anticholesterol drugs	0.002 ± 0.051	+	0.969	
k__Bacteria.p__Firmicutes.c__Clostridia.o__Clostridiales.f__Lachnospiraceae.g__Lachnoclostridium.s__Clostridium_symbiosum	age	0.177 ± 0.067	+	8.07E-03	
k__Bacteria.p__Firmicutes.c__Clostridia.o__Clostridiales.f__Lachnospiraceae.g__Lachnoclostridium.s__Clostridium_symbiosum	BMI	-0.057 ± 0.079	-	0.469	
k__Bacteria.p__Firmicutes.c__Clostridia.o__Clostridiales.f__Lachnospiraceae.g__Lachnoclostridium.s__Clostridium_symbiosum	constipation	0.259 ± 0.073	+	3.67E-04	
k__Bacteria.p__Firmicutes.c__Clostridia.o__Clostridiales.f__Lachnospiraceae.g__Lachnoclostridium.s__Clostridium_symbiosum	disease	-0.052 ± 0.159	-	0.746	0.834
k__Bacteria.p__Firmicutes.c__Clostridia.o__Clostridiales.f__Lachnospiraceae.g__Lachnoclostridium.s__Clostridium_symbiosum	sex	0.356 ± 0.069	+	2.73E-07	
k__Bacteria.p__Firmicutes.c__Clostridia.o__Clostridiales.f__Lachnospiraceae.g__Lachnoclostridium.s__Clostridium_symbiosum	anticholesterol drugs	0.067 ± 0.071	+	0.345	
k__Bacteria.p__Firmicutes.c__Clostridia.o__Clostridiales.f__Lachnospiraceae.g__Lacrimispora.s__Lacrimispora_amygdalina	age	-0.081 ± 0.067	-	0.226	
k__Bacteria.p__Firmicutes.c__Clostridia.o__Clostridiales.f__Lachnospiraceae.g__Lacrimispora.s__Lacrimispora_amygdalina	BMI	0.041 ± 0.075	+	0.580	

k__Bacteria.p__Firmicutes.c__Clostridia.o__Clostridiales.f__Lachnospiraceae.g__Lacrimispora.s__Lacrimispora_amygdalina	constipation	-0.377 ± 0.083	-	4.89E-06	
k__Bacteria.p__Firmicutes.c__Clostridia.o__Clostridiales.f__Lachnospiraceae.g__Lacrimispora.s__Lacrimispora_amygdalina	disease	-0.542 ± 0.154	-	4.39E-04	*2.78E-03
k__Bacteria.p__Firmicutes.c__Clostridia.o__Clostridiales.f__Lachnospiraceae.g__Lacrimispora.s__Lacrimispora_amygdalina	sex	-0.147 ± 0.072	-	0.0421	
k__Bacteria.p__Firmicutes.c__Clostridia.o__Clostridiales.f__Lachnospiraceae.g__Lacrimispora.s__Lacrimispora_amygdalina	anticholesterol drugs	-0.037 ± 0.075	-	0.625	
k__Bacteria.p__Firmicutes.c__Clostridia.o__Clostridiales.f__Lachnospiraceae.g__Roseburia.s__Roseburia_faecis	age	0.000 ± 0.078	+	0.997	
k__Bacteria.p__Firmicutes.c__Clostridia.o__Clostridiales.f__Lachnospiraceae.g__Roseburia.s__Roseburia_faecis	BMI	0.135 ± 0.079	+	0.0870	
k__Bacteria.p__Firmicutes.c__Clostridia.o__Clostridiales.f__Lachnospiraceae.g__Roseburia.s__Roseburia_faecis	constipation	-0.201 ± 0.079	-	0.0113	
k__Bacteria.p__Firmicutes.c__Clostridia.o__Clostridiales.f__Lachnospiraceae.g__Roseburia.s__Roseburia_faecis	disease	-0.515 ± 0.167	-	2.02E-03	*6.40E-03
k__Bacteria.p__Firmicutes.c__Clostridia.o__Clostridiales.f__Lachnospiraceae.g__Roseburia.s__Roseburia_faecis	sex	-0.161 ± 0.075	-	0.0329	
k__Bacteria.p__Firmicutes.c__Clostridia.o__Clostridiales.f__Lachnospiraceae.g__Roseburia.s__Roseburia_faecis	anticholesterol drugs	0.014 ± 0.077	+	0.854	
k__Bacteria.p__Firmicutes.c__Clostridia.o__Clostridiales.f__Lachnospiraceae.g__Roseburia.s__Roseburia_intestinalis	age	-0.147 ± 0.067	-	0.0272	
k__Bacteria.p__Firmicutes.c__Clostridia.o__Clostridiales.f__Lachnospiraceae.g__Roseburia.s__Roseburia_intestinalis	BMI	0.125 ± 0.067	+	0.0635	
k__Bacteria.p__Firmicutes.c__Clostridia.o__Clostridiales.f__Lachnospiraceae.g__Roseburia.s__Roseburia_intestinalis	constipation	-0.455 ± 0.078	-	5.47E-09	
k__Bacteria.p__Firmicutes.c__Clostridia.o__Clostridiales.f__Lachnospiraceae.g__Roseburia.s__Roseburia_intestinalis	disease	-0.689 ± 0.150	-	4.45E-06	*4.23E-05
k__Bacteria.p__Firmicutes.c__Clostridia.o__Clostridiales.f__Lachnospiraceae.g__Roseburia.s__Roseburia_intestinalis	sex	-0.086 ± 0.071	-	0.228	
k__Bacteria.p__Firmicutes.c__Clostridia.o__Clostridiales.f__Lachnospiraceae.g__Roseburia.s__Roseburia_intestinalis	anticholesterol drugs	-0.038 ± 0.072	-	0.594	
k__Bacteria.p__Firmicutes.c__Clostridia.o__Clostridiales.f__Lachnospiraceae.g__Roseburia.s__Roseburia_sp_AF02_12	age	-0.023 ± 0.130	-	0.863	
k__Bacteria.p__Firmicutes.c__Clostridia.o__Clostridiales.f__Lachnospiraceae.g__Roseburia.s__Roseburia_sp_AF02_12	BMI	0.000 ± 0.118	+	0.999	
k__Bacteria.p__Firmicutes.c__Clostridia.o__Clostridiales.f__Lachnospiraceae.g__Roseburia.s__Roseburia_sp_AF02_12	constipation	-0.045 ± 0.113	-	0.691	
k__Bacteria.p__Firmicutes.c__Clostridia.o__Clostridiales.f__Lachnospiraceae.g__Roseburia.s__Roseburia_sp_AF02_12	disease	-0.011 ± 0.257	-	0.967	0.967
k__Bacteria.p__Firmicutes.c__Clostridia.o__Clostridiales.f__Lachnospiraceae.g__Roseburia.s__Roseburia_sp_AF02_12	sex	-0.205 ± 0.121	-	0.0913	
k__Bacteria.p__Firmicutes.c__Clostridia.o__Clostridiales.f__Lachnospiraceae.g__Roseburia.s__Roseburia_sp_AF02_12	anticholesterol drugs	0.129 ± 0.113	+	0.253	
k__Bacteria.p__Firmicutes.c__Clostridia.o__Clostridiales.f__Oscillospiraceae.g__Dysosmobacter.s__Dysosmobacter_sp_NSJ_60	age	0.034 ± 0.048	+	0.472	
k__Bacteria.p__Firmicutes.c__Clostridia.o__Clostridiales.f__Oscillospiraceae.g__Dysosmobacter.s__Dysosmobacter_sp_NSJ_60	BMI	0.056 ± 0.046	+	0.229	
k__Bacteria.p__Firmicutes.c__Clostridia.o__Clostridiales.f__Oscillospiraceae.g__Dysosmobacter.s__Dysosmobacter_sp_NSJ_60	constipation	-0.013 ± 0.047	-	0.781	
k__Bacteria.p__Firmicutes.c__Clostridia.o__Clostridiales.f__Oscillospiraceae.g__Dysosmobacter.s__Dysosmobacter_sp_NSJ_60	disease	0.327 ± 0.104	+	1.68E-03	*6.19E-03
k__Bacteria.p__Firmicutes.c__Clostridia.o__Clostridiales.f__Oscillospiraceae.g__Dysosmobacter.s__Dysosmobacter_sp_NSJ_60	sex	0.167 ± 0.047	+	3.48E-04	
k__Bacteria.p__Firmicutes.c__Clostridia.o__Clostridiales.f__Oscillospiraceae.g__Dysosmobacter.s__Dysosmobacter_sp_NSJ_60	anticholesterol drugs	-0.029 ± 0.046	-	0.536	
k__Bacteria.p__Firmicutes.c__Clostridia.o__Clostridiales.f__Oscillospiraceae.g__Dysosmobacter.s__Dysosmobacter_welbionis	age	0.004 ± 0.037	+	0.918	
k__Bacteria.p__Firmicutes.c__Clostridia.o__Clostridiales.f__Oscillospiraceae.g__Dysosmobacter.s__Dysosmobacter_welbionis	BMI	0.073 ± 0.040	+	0.0644	
k__Bacteria.p__Firmicutes.c__Clostridia.o__Clostridiales.f__Oscillospiraceae.g__Dysosmobacter.s__Dysosmobacter_welbionis	constipation	0.029 ± 0.036	+	0.422	
k__Bacteria.p__Firmicutes.c__Clostridia.o__Clostridiales.f__Oscillospiraceae.g__Dysosmobacter.s__Dysosmobacter_welbionis	disease	0.198 ± 0.081	+	0.0150	*0.0316
k__Bacteria.p__Firmicutes.c__Clostridia.o__Clostridiales.f__Oscillospiraceae.g__Dysosmobacter.s__Dysosmobacter_welbionis	sex	0.183 ± 0.037	+	7.89E-07	
k__Bacteria.p__Firmicutes.c__Clostridia.o__Clostridiales.f__Oscillospiraceae.g__Dysosmobacter.s__Dysosmobacter_welbionis	anticholesterol drugs	0.036 ± 0.037	+	0.333	
k__Bacteria.p__Firmicutes.c__Clostridia.o__Clostridiales.f__Ruminococcaceae.g__Agathobaculum.s__Agathobaculum_butyriciproducens	age	-0.114 ± 0.050	-	0.0241	
k__Bacteria.p__Firmicutes.c__Clostridia.o__Clostridiales.f__Ruminococcaceae.g__Agathobaculum.s__Agathobaculum_butyriciproducens	BMI	-0.042 ± 0.052	-	0.421	
k__Bacteria.p__Firmicutes.c__Clostridia.o__Clostridiales.f__Ruminococcaceae.g__Agathobaculum.s__Agathobaculum_butyriciproducens	constipation	-0.252 ± 0.056	-	7.56E-06	
k__Bacteria.p__Firmicutes.c__Clostridia.o__Clostridiales.f__Ruminococcaceae.g__Agathobaculum.s__Agathobaculum_butyriciproducens	disease	-0.330 ± 0.110	-	2.70E-03	*7.34E-03
k__Bacteria.p__Firmicutes.c__Clostridia.o__Clostridiales.f__Ruminococcaceae.g__Agathobaculum.s__Agathobaculum_butyriciproducens	sex	-0.103 ± 0.052	-	0.0465	
k__Bacteria.p__Firmicutes.c__Clostridia.o__Clostridiales.f__Ruminococcaceae.g__Agathobaculum.s__Agathobaculum_butyriciproducens	anticholesterol drugs	-0.033 ± 0.052	-	0.524	
k__Bacteria.p__Firmicutes.c__Clostridia.o__Clostridiales.f__Ruminococcaceae.g__Anaeromassilibacillus.s__Anaeromassilibacillus_sp_An250	age	0.277 ± 0.080	+	5.20E-04	
k__Bacteria.p__Firmicutes.c__Clostridia.o__Clostridiales.f__Ruminococcaceae.g__Anaeromassilibacillus.s__Anaeromassilibacillus_sp_An250	BMI	0.063 ± 0.083	+	0.447	
k__Bacteria.p__Firmicutes.c__Clostridia.o__Clostridiales.f__Ruminococcaceae.g__Anaeromassilibacillus.s__Anaeromassilibacillus_sp_An250	constipation	0.100 ± 0.077	+	0.195	
k__Bacteria.p__Firmicutes.c__Clostridia.o__Clostridiales.f__Ruminococcaceae.g__Anaeromassilibacillus.s__Anaeromassilibacillus_sp_An250	disease	-0.122 ± 0.176	-	0.489	0.580
k__Bacteria.p__Firmicutes.c__Clostridia.o__Clostridiales.f__Ruminococcaceae.g__Anaeromassilibacillus.s__Anaeromassilibacillus_sp_An250	sex	0.201 ± 0.079	+	0.0112	
k__Bacteria.p__Firmicutes.c__Clostridia.o__Clostridiales.f__Ruminococcaceae.g__Anaeromassilibacillus.s__Anaeromassilibacillus_sp_An250	anticholesterol drugs	-0.012 ± 0.080	-	0.878	
k__Bacteria.p__Firmicutes.c__Clostridia.o__Clostridiales.f__Ruminococcaceae.g__Anaerotruncus.s__Anaerotruncus_colihominis	age	-0.075 ± 0.069	-	0.274	
k__Bacteria.p__Firmicutes.c__Clostridia.o__Clostridiales.f__Ruminococcaceae.g__Anaerotruncus.s__Anaerotruncus_colihominis	BMI	-0.140 ± 0.075	-	0.0614	
k__Bacteria.p__Firmicutes.c__Clostridia.o__Clostridiales.f__Ruminococcaceae.g__Anaerotruncus.s__Anaerotruncus_colihominis	constipation	0.260 ± 0.065	+	6.27E-05	
k__Bacteria.p__Firmicutes.c__Clostridia.o__Clostridiales.f__Ruminococcaceae.g__Anaerotruncus.s__Anaerotruncus_colihominis	disease	0.531 ± 0.155	+	6.14E-04	*2.91E-03
k__Bacteria.p__Firmicutes.c__Clostridia.o__Clostridiales.f__Ruminococcaceae.g__Anaerotruncus.s__Anaerotruncus_colihominis	sex	0.373 ± 0.068	+	3.47E-08	
k__Bacteria.p__Firmicutes.c__Clostridia.o__Clostridiales.f__Ruminococcaceae.g__Anaerotruncus.s__Anaerotruncus_colihominis	anticholesterol drugs	0.306 ± 0.067	+	4.50E-06	
k__Bacteria.p__Firmicutes.c__Clostridia.o__Clostridiales.f__Ruminococcaceae.g__Anaerotruncus.s__Anaerotruncus_rubiinfantis	age	0.315 ± 0.073	+	1.51E-05	
k__Bacteria.p__Firmicutes.c__Clostridia.o__Clostridiales.f__Ruminococcaceae.g__Anaerotruncus.s__Anaerotruncus_rubiinfantis	BMI	0.017 ± 0.078	+	0.826	
k__Bacteria.p__Firmicutes.c__Clostridia.o__Clostridiales.f__Ruminococcaceae.g__Anaerotruncus.s__Anaerotruncus_rubiinfantis	constipation	0.198 ± 0.062	+	1.47E-03	
k__Bacteria.p__Firmicutes.c__Clostridia.o__Clostridiales.f__Ruminococcaceae.g__Anaerotruncus.s__Anaerotruncus_rubiinfantis	disease	0.751 ± 0.159	+	2.43E-06	*4.23E-05
k__Bacteria.p__Firmicutes.c__Clostridia.o__Clostridiales.f__Ruminococcaceae.g__Anaerotruncus.s__Anaerotruncus_rubiinfantis	sex	-0.113 ± 0.068	-	0.0934	
k__Bacteria.p__Firmicutes.c__Clostridia.o__Clostridiales.f__Ruminococcaceae.g__Anaerotruncus.s__Anaerotruncus_rubiinfantis	anticholesterol drugs	-0.042 ± 0.065	-	0.523	
k__Bacteria.p__Firmicutes.c__Clostridia.o__Clostridiales.f__Ruminococcaceae.g__Faecalibacterium.s__Faecalibacterium_prausnitzii	age	-0.113 ± 0.048	-	0.0183	

k__Bacteria.p__Firmicutes.c__Clostridia.o__Clostridiales.f__Ruminococcaceae.g__Faecalibacterium.s__Faecalibacterium_prausnitzii	BMI	-0.026 ± 0.048	-	0.592	
k__Bacteria.p__Firmicutes.c__Clostridia.o__Clostridiales.f__Ruminococcaceae.g__Faecalibacterium.s__Faecalibacterium_prausnitzii	constipation	-0.268 ± 0.048	-	3.30E-08	
k__Bacteria.p__Firmicutes.c__Clostridia.o__Clostridiales.f__Ruminococcaceae.g__Faecalibacterium.s__Faecalibacterium_prausnitzii	disease	-0.249 ± 0.099	-	0.0121	*0.0287
k__Bacteria.p__Firmicutes.c__Clostridia.o__Clostridiales.f__Ruminococcaceae.g__Faecalibacterium.s__Faecalibacterium_prausnitzii	sex	-0.051 ± 0.047	-	0.271	
k__Bacteria.p__Firmicutes.c__Clostridia.o__Clostridiales.f__Ruminococcaceae.g__Faecalibacterium.s__Faecalibacterium_prausnitzii	anticholesterol drugs	0.093 ± 0.047	+	0.0469	
k__Bacteria.p__Firmicutes.c__Clostridia.o__Clostridiales.f__Ruminococcaceae.g__GGB9699.s__GGB9699_SGB15216	age	0.160 ± 0.077	+	0.0377	
k__Bacteria.p__Firmicutes.c__Clostridia.o__Clostridiales.f__Ruminococcaceae.g__GGB9699.s__GGB9699_SGB15216	BMI	-0.102 ± 0.082	-	0.212	
k__Bacteria.p__Firmicutes.c__Clostridia.o__Clostridiales.f__Ruminococcaceae.g__GGB9699.s__GGB9699_SGB15216	constipation	0.246 ± 0.073	+	8.01E-04	
k__Bacteria.p__Firmicutes.c__Clostridia.o__Clostridiales.f__Ruminococcaceae.g__GGB9699.s__GGB9699_SGB15216	disease	0.409 ± 0.171	+	0.0167	*0.0334
k__Bacteria.p__Firmicutes.c__Clostridia.o__Clostridiales.f__Ruminococcaceae.g__GGB9699.s__GGB9699_SGB15216	sex	-0.003 ± 0.075	-	0.969	
k__Bacteria.p__Firmicutes.c__Clostridia.o__Clostridiales.f__Ruminococcaceae.g__GGB9699.s__GGB9699_SGB15216	anticholesterol drugs	-0.006 ± 0.073	-	0.931	
k__Bacteria.p__Firmicutes.c__Clostridia.o__Clostridiales.f__Ruminococcaceae.g__Ruminococcaceae_unclassified.s__Ruminococcaceae_bacterium	age	0.169 ± 0.048	+	4.18E-04	
k__Bacteria.p__Firmicutes.c__Clostridia.o__Clostridiales.f__Ruminococcaceae.g__Ruminococcaceae_unclassified.s__Ruminococcaceae_bacterium	BMI	-0.023 ± 0.048	-	0.623	
k__Bacteria.p__Firmicutes.c__Clostridia.o__Clostridiales.f__Ruminococcaceae.g__Ruminococcaceae_unclassified.s__Ruminococcaceae_bacterium	constipation	0.119 ± 0.049	+	0.0151	
k__Bacteria.p__Firmicutes.c__Clostridia.o__Clostridiales.f__Ruminococcaceae.g__Ruminococcaceae_unclassified.s__Ruminococcaceae_bacterium	disease	0.406 ± 0.110	+	2.16E-04	*1.64E-03
k__Bacteria.p__Firmicutes.c__Clostridia.o__Clostridiales.f__Ruminococcaceae.g__Ruminococcaceae_unclassified.s__Ruminococcaceae_bacterium	sex	-0.023 ± 0.052	-	0.656	
k__Bacteria.p__Firmicutes.c__Clostridia.o__Clostridiales.f__Ruminococcaceae.g__Ruminococcaceae_unclassified.s__Ruminococcaceae_bacterium	anticholesterol drugs	-0.029 ± 0.050	-	0.555	
k__Bacteria.p__Firmicutes.c__Clostridia.o__Clostridiales.f__Ruminococcaceae.g__Ruthenibacterium.s__Ruthenibacterium_lactatiformans	age	0.130 ± 0.043	+	2.65E-03	
k__Bacteria.p__Firmicutes.c__Clostridia.o__Clostridiales.f__Ruminococcaceae.g__Ruthenibacterium.s__Ruthenibacterium_lactatiformans	BMI	0.077 ± 0.047	+	0.0991	
k__Bacteria.p__Firmicutes.c__Clostridia.o__Clostridiales.f__Ruminococcaceae.g__Ruthenibacterium.s__Ruthenibacterium_lactatiformans	constipation	0.307 ± 0.043	+	1.10E-12	
k__Bacteria.p__Firmicutes.c__Clostridia.o__Clostridiales.f__Ruminococcaceae.g__Ruthenibacterium.s__Ruthenibacterium_lactatiformans	disease	0.800 ± 0.101	+	2.62E-15	*9.95E-14
k__Bacteria.p__Firmicutes.c__Clostridia.o__Clostridiales.f__Ruminococcaceae.g__Ruthenibacterium.s__Ruthenibacterium_lactatiformans	sex	0.337 ± 0.044	+	3.46E-14	
k__Bacteria.p__Firmicutes.c__Clostridia.o__Clostridiales.f__Ruminococcaceae.g__Ruthenibacterium.s__Ruthenibacterium_lactatiformans	anticholesterol drugs	-0.016 ± 0.045	-	0.718	
k__Bacteria.p__Firmicutes.c__Erysipelotrichia.o__Erysipelotrichales.f__Erysipelotrichaceae.g__Erysipelatoclostridium.s__Clostridium_innocuum	age	0.285 ± 0.067	+	2.13E-05	
k__Bacteria.p__Firmicutes.c__Erysipelotrichia.o__Erysipelotrichales.f__Erysipelotrichaceae.g__Erysipelatoclostridium.s__Clostridium_innocuum	BMI	0.109 ± 0.083	+	0.192	
k__Bacteria.p__Firmicutes.c__Erysipelotrichia.o__Erysipelotrichales.f__Erysipelotrichaceae.g__Erysipelatoclostridium.s__Clostridium_innocuum	constipation	0.296 ± 0.069	+	1.87E-05	
k__Bacteria.p__Firmicutes.c__Erysipelotrichia.o__Erysipelotrichales.f__Erysipelotrichaceae.g__Erysipelatoclostridium.s__Clostridium_innocuum	disease	0.042 ± 0.159	+	0.793	0.837
k__Bacteria.p__Firmicutes.c__Erysipelotrichia.o__Erysipelotrichales.f__Erysipelotrichaceae.g__Erysipelatoclostridium.s__Clostridium_innocuum	sex	0.386 ± 0.071	+	4.69E-08	
k__Bacteria.p__Firmicutes.c__Erysipelotrichia.o__Erysipelotrichales.f__Erysipelotrichaceae.g__Erysipelatoclostridium.s__Clostridium_innocuum	anticholesterol drugs	0.035 ± 0.076	+	0.644	
k__Bacteria.p__Proteobacteria.c__Deltaproteobacteria.o__Desulfovibrionales.f__Desulfovibrionaceae.g__Bilophila.s__Bilophila_wadsworthia	age	-0.021 ± 0.045	-	0.648	
k__Bacteria.p__Proteobacteria.c__Deltaproteobacteria.o__Desulfovibrionales.f__Desulfovibrionaceae.g__Bilophila.s__Bilophila_wadsworthia	BMI	0.019 ± 0.048	+	0.696	
k__Bacteria.p__Proteobacteria.c__Deltaproteobacteria.o__Desulfovibrionales.f__Desulfovibrionaceae.g__Bilophila.s__Bilophila_wadsworthia	constipation	0.055 ± 0.044	+	0.217	
k__Bacteria.p__Proteobacteria.c__Deltaproteobacteria.o__Desulfovibrionales.f__Desulfovibrionaceae.g__Bilophila.s__Bilophila_wadsworthia	disease	0.175 ± 0.101	+	0.0838	0.138
k__Bacteria.p__Proteobacteria.c__Deltaproteobacteria.o__Desulfovibrionales.f__Desulfovibrionaceae.g__Bilophila.s__Bilophila_wadsworthia	sex	-0.025 ± 0.045	-	0.588	
k__Bacteria.p__Proteobacteria.c__Deltaproteobacteria.o__Desulfovibrionales.f__Desulfovibrionaceae.g__Bilophila.s__Bilophila_wadsworthia	anticholesterol drugs	-0.039 ± 0.045	-	0.387	
k__Bacteria.p__Proteobacteria.c__Gammaproteobacteria.o__Pasteurellales.f__Pasteurellaceae.g__Haemophilus.s__Haemophilus_parainfluenzae	age	0.178 ± 0.147	+	0.226	
k__Bacteria.p__Proteobacteria.c__Gammaproteobacteria.o__Pasteurellales.f__Pasteurellaceae.g__Haemophilus.s__Haemophilus_parainfluenzae	BMI	-0.069 ± 0.168	-	0.680	
k__Bacteria.p__Proteobacteria.c__Gammaproteobacteria.o__Pasteurellales.f__Pasteurellaceae.g__Haemophilus.s__Haemophilus_parainfluenzae	constipation	-0.457 ± 0.152	-	2.66E-03	
k__Bacteria.p__Proteobacteria.c__Gammaproteobacteria.o__Pasteurellales.f__Pasteurellaceae.g__Haemophilus.s__Haemophilus_parainfluenzae	disease	-0.354 ± 0.294	-	0.228	0.334
k__Bacteria.p__Proteobacteria.c__Gammaproteobacteria.o__Pasteurellales.f__Pasteurellaceae.g__Haemophilus.s__Haemophilus_parainfluenzae	sex	-0.135 ± 0.143	-	0.345	
k__Bacteria.p__Proteobacteria.c__Gammaproteobacteria.o__Pasteurellales.f__Pasteurellaceae.g__Haemophilus.s__Haemophilus_parainfluenzae	anticholesterol drugs	-0.187 ± 0.142	-	0.186	
k__Bacteria.p__Verrucomicrobia.c__Verrucomicrobiae.o__Verrucomicrobiales.f__Akkermansiaceae.g__Akkermansia.s__Akkermansia_muciniphila	age	0.117 ± 0.083	+	0.161	
k__Bacteria.p__Verrucomicrobia.c__Verrucomicrobiae.o__Verrucomicrobiales.f__Akkermansiaceae.g__Akkermansia.s__Akkermansia_muciniphila	BMI	-0.049 ± 0.089	-	0.578	
k__Bacteria.p__Verrucomicrobia.c__Verrucomicrobiae.o__Verrucomicrobiales.f__Akkermansiaceae.g__Akkermansia.s__Akkermansia_muciniphila	constipation	0.227 ± 0.077	+	3.11E-03	
k__Bacteria.p__Verrucomicrobia.c__Verrucomicrobiae.o__Verrucomicrobiales.f__Akkermansiaceae.g__Akkermansia.s__Akkermansia_muciniphila	disease	0.505 ± 0.177	+	4.32E-03	*0.0109
k__Bacteria.p__Verrucomicrobia.c__Verrucomicrobiae.o__Verrucomicrobiales.f__Akkermansiaceae.g__Akkermansia.s__Akkermansia_muciniphila	sex	0.065 ± 0.080	+	0.422	
k__Bacteria.p__Verrucomicrobia.c__Verrucomicrobiae.o__Verrucomicrobiales.f__Akkermansiaceae.g__Akkermansia.s__Akkermansia_muciniphila	anticholesterol drugs	0.062 ± 0.077	+	0.416	

**Genus**	**Confounding factors**	**Beta coefficient ± SE**	**Increase or decrease**	***p*-value**	***q*-value**
k__Bacteria.p__Actinobacteria.c__Actinobacteria.o__Bifidobacteriales.f__Bifidobacteriaceae.g__Bifidobacterium	age	-0.180 ± 0.058	-	1.90E-03	
k__Bacteria.p__Actinobacteria.c__Actinobacteria.o__Bifidobacteriales.f__Bifidobacteriaceae.g__Bifidobacterium	BMI	-0.172 ± 0.059	-	3.56E-03	
k__Bacteria.p__Actinobacteria.c__Actinobacteria.o__Bifidobacteriales.f__Bifidobacteriaceae.g__Bifidobacterium	constipation	0.177 ± 0.056	+	1.56E-03	
k__Bacteria.p__Actinobacteria.c__Actinobacteria.o__Bifidobacteriales.f__Bifidobacteriaceae.g__Bifidobacterium	disease	0.525 ± 0.126	+	3.07E-05	*3.02E-04
k__Bacteria.p__Actinobacteria.c__Actinobacteria.o__Bifidobacteriales.f__Bifidobacteriaceae.g__Bifidobacterium	sex	0.048 ± 0.054	+	0.375	
k__Bacteria.p__Actinobacteria.c__Actinobacteria.o__Bifidobacteriales.f__Bifidobacteriaceae.g__Bifidobacterium	anticholesterol drugs	-0.054 ± 0.056	-	0.337	
k__Bacteria.p__Actinobacteria.c__Coriobacteriia.o__Eggerthellales.f__Eggerthellaceae.g__Gordonibacter	age	0.167 ± 0.059	+	4.35E-03	
k__Bacteria.p__Actinobacteria.c__Coriobacteriia.o__Eggerthellales.f__Eggerthellaceae.g__Gordonibacter	BMI	-0.060 ± 0.056	-	0.284	

k__Bacteria.p__Actinobacteria.c__Coriobacteriia.o__Eggerthellales.f__Eggerthellaceae.g__Gordonibacter	constipation	0.108 ± 0.054	+	0.0472	
k__Bacteria.p__Actinobacteria.c__Coriobacteriia.o__Eggerthellales.f__Eggerthellaceae.g__Gordonibacter	disease	0.398 ± 0.126	+	1.60E-03	*6.20E-03
k__Bacteria.p__Actinobacteria.c__Coriobacteriia.o__Eggerthellales.f__Eggerthellaceae.g__Gordonibacter	sex	0.205 ± 0.057	+	3.34E-04	
k__Bacteria.p__Actinobacteria.c__Coriobacteriia.o__Eggerthellales.f__Eggerthellaceae.g__Gordonibacter	anticholesterol drugs	-0.038 ± 0.058	-	0.516	
k__Bacteria.p__Bacteroidetes.c__Bacteroidia.o__Bacteroidales.f__Bacteroidales_unclassified.g__Phocaeicola	age	-0.064 ± 0.033	-	0.0532	
k__Bacteria.p__Bacteroidetes.c__Bacteroidia.o__Bacteroidales.f__Bacteroidales_unclassified.g__Phocaeicola	BMI	0.072 ± 0.033	+	0.0286	
k__Bacteria.p__Bacteroidetes.c__Bacteroidia.o__Bacteroidales.f__Bacteroidales_unclassified.g__Phocaeicola	constipation	-0.129 ± 0.034	-	1.44E-04	
k__Bacteria.p__Bacteroidetes.c__Bacteroidia.o__Bacteroidales.f__Bacteroidales_unclassified.g__Phocaeicola	disease	-0.104 ± 0.071	-	0.146	0.188
k__Bacteria.p__Bacteroidetes.c__Bacteroidia.o__Bacteroidales.f__Bacteroidales_unclassified.g__Phocaeicola	sex	0.025 ± 0.033	+	0.453	
k__Bacteria.p__Bacteroidetes.c__Bacteroidia.o__Bacteroidales.f__Bacteroidales_unclassified.g__Phocaeicola	anticholesterol drugs	0.002 ± 0.033	+	0.943	
k__Bacteria.p__Bacteroidetes.c__Bacteroidia.o__Bacteroidales.f__Barnesiellaceae.g__Barnesiella	age	0.122 ± 0.095	+	0.199	
k__Bacteria.p__Bacteroidetes.c__Bacteroidia.o__Bacteroidales.f__Barnesiellaceae.g__Barnesiella	BMI	-0.023 ± 0.095	-	0.811	
k__Bacteria.p__Bacteroidetes.c__Bacteroidia.o__Bacteroidales.f__Barnesiellaceae.g__Barnesiella	constipation	-0.002 ± 0.093	-	0.987	
k__Bacteria.p__Bacteroidetes.c__Bacteroidia.o__Bacteroidales.f__Barnesiellaceae.g__Barnesiella	disease	0.168 ± 0.202	+	0.405	0.433
k__Bacteria.p__Bacteroidetes.c__Bacteroidia.o__Bacteroidales.f__Barnesiellaceae.g__Barnesiella	sex	-0.005 ± 0.090	-	0.955	
k__Bacteria.p__Bacteroidetes.c__Bacteroidia.o__Bacteroidales.f__Barnesiellaceae.g__Barnesiella	anticholesterol drugs	0.107 ± 0.089	+	0.228	
k__Bacteria.p__Bacteroidetes.c__Bacteroidia.o__Bacteroidales.f__Rikenellaceae.g__Alistipes	age	0.055 ± 0.035	+	0.122	
k__Bacteria.p__Bacteroidetes.c__Bacteroidia.o__Bacteroidales.f__Rikenellaceae.g__Alistipes	BMI	-0.072 ± 0.038	-	0.0554	
k__Bacteria.p__Bacteroidetes.c__Bacteroidia.o__Bacteroidales.f__Rikenellaceae.g__Alistipes	constipation	0.068 ± 0.034	+	0.045	
k__Bacteria.p__Bacteroidetes.c__Bacteroidia.o__Bacteroidales.f__Rikenellaceae.g__Alistipes	disease	0.101 ± 0.076	+	0.184	0.228
k__Bacteria.p__Bacteroidetes.c__Bacteroidia.o__Bacteroidales.f__Rikenellaceae.g__Alistipes	sex	0.028 ± 0.035	+	0.421	
k__Bacteria.p__Bacteroidetes.c__Bacteroidia.o__Bacteroidales.f__Rikenellaceae.g__Alistipes	anticholesterol drugs	0.007 ± 0.035	+	0.839	
k__Bacteria.p__Bacteroidetes.c__Bacteroidia.o__Bacteroidales.f__Tannerellaceae.g__Parabacteroides	age	-0.010 ± 0.035	-	0.766	
k__Bacteria.p__Bacteroidetes.c__Bacteroidia.o__Bacteroidales.f__Tannerellaceae.g__Parabacteroides	BMI	-0.037 ± 0.038	-	0.337	
k__Bacteria.p__Bacteroidetes.c__Bacteroidia.o__Bacteroidales.f__Tannerellaceae.g__Parabacteroides	constipation	-0.089 ± 0.036	-	0.0127	
k__Bacteria.p__Bacteroidetes.c__Bacteroidia.o__Bacteroidales.f__Tannerellaceae.g__Parabacteroides	disease	0.117 ± 0.077	+	0.128	0.173
k__Bacteria.p__Bacteroidetes.c__Bacteroidia.o__Bacteroidales.f__Tannerellaceae.g__Parabacteroides	sex	0.061 ± 0.035	+	0.0805	
k__Bacteria.p__Bacteroidetes.c__Bacteroidia.o__Bacteroidales.f__Tannerellaceae.g__Parabacteroides	anticholesterol drugs	0.027 ± 0.036	+	0.456	
k__Bacteria.p__Firmicutes.c__Bacilli.o__Bacilli_unclassified.f__Bacilli_unclassified.g__Bacilli_unclassified	age	0.137 ± 0.138	+	0.321	
k__Bacteria.p__Firmicutes.c__Bacilli.o__Bacilli_unclassified.f__Bacilli_unclassified.g__Bacilli_unclassified	BMI	-0.525 ± 0.179	-	3.43E-03	
k__Bacteria.p__Firmicutes.c__Bacilli.o__Bacilli_unclassified.f__Bacilli_unclassified.g__Bacilli_unclassified	constipation	0.038 ± 0.136	+	0.783	
k__Bacteria.p__Firmicutes.c__Bacilli.o__Bacilli_unclassified.f__Bacilli_unclassified.g__Bacilli_unclassified	disease	0.829 ± 0.325	+	0.0107	*0.022
k__Bacteria.p__Firmicutes.c__Bacilli.o__Bacilli_unclassified.f__Bacilli_unclassified.g__Bacilli_unclassified	sex	-0.523 ± 0.135	-	1.05E-04	
k__Bacteria.p__Firmicutes.c__Bacilli.o__Bacilli_unclassified.f__Bacilli_unclassified.g__Bacilli_unclassified	anticholesterol drugs	0.111 ± 0.132	+	0.399	
k__Bacteria.p__Firmicutes.c__CFGB1217.o__OFGB1217.f__FGB1217.g__GGB2982	age	-0.075 ± 0.072	-	0.295	
k__Bacteria.p__Firmicutes.c__CFGB1217.o__OFGB1217.f__FGB1217.g__GGB2982	BMI	-0.009 ± 0.078	-	0.905	
k__Bacteria.p__Firmicutes.c__CFGB1217.o__OFGB1217.f__FGB1217.g__GGB2982	constipation	-0.003 ± 0.077	-	0.973	
k__Bacteria.p__Firmicutes.c__CFGB1217.o__OFGB1217.f__FGB1217.g__GGB2982	disease	-0.174 ± 0.164	-	0.290	0.332
k__Bacteria.p__Firmicutes.c__CFGB1217.o__OFGB1217.f__FGB1217.g__GGB2982	sex	-0.160 ± 0.076	-	0.0348	
k__Bacteria.p__Firmicutes.c__CFGB1217.o__OFGB1217.f__FGB1217.g__GGB2982	anticholesterol drugs	0.006 ± 0.075	+	0.935	
k__Bacteria.p__Firmicutes.c__Clostridia.o__Clostridia_unclassified.f__Clostridia_unclassified.g__Clostridia_unclassified	age	0.154 ± 0.039	+	7.50E-05	
k__Bacteria.p__Firmicutes.c__Clostridia.o__Clostridia_unclassified.f__Clostridia_unclassified.g__Clostridia_unclassified	BMI	0.006 ± 0.040	+	0.875	
k__Bacteria.p__Firmicutes.c__Clostridia.o__Clostridia_unclassified.f__Clostridia_unclassified.g__Clostridia_unclassified	constipation	0.172 ± 0.036	+	2.29E-06	
k__Bacteria.p__Firmicutes.c__Clostridia.o__Clostridia_unclassified.f__Clostridia_unclassified.g__Clostridia_unclassified	disease	0.278 ± 0.085	+	1.03E-03	*4.56E-03
k__Bacteria.p__Firmicutes.c__Clostridia.o__Clostridia_unclassified.f__Clostridia_unclassified.g__Clostridia_unclassified	sex	0.018 ± 0.038	+	0.634	
k__Bacteria.p__Firmicutes.c__Clostridia.o__Clostridia_unclassified.f__Clostridia_unclassified.g__Clostridia_unclassified	anticholesterol drugs	-0.011 ± 0.037	-	0.759	
k__Bacteria.p__Firmicutes.c__Clostridia.o__Clostridiales.f__Christensenellaceae.g__Christensenellaceae_unclassified	age	0.144 ± 0.066	+	0.0285	
k__Bacteria.p__Firmicutes.c__Clostridia.o__Clostridiales.f__Christensenellaceae.g__Christensenellaceae_unclassified	BMI	-0.252 ± 0.077	-	1.02E-03	
k__Bacteria.p__Firmicutes.c__Clostridia.o__Clostridiales.f__Christensenellaceae.g__Christensenellaceae_unclassified	constipation	0.230 ± 0.060	+	1.11E-04	
k__Bacteria.p__Firmicutes.c__Clostridia.o__Clostridiales.f__Christensenellaceae.g__Christensenellaceae_unclassified	disease	0.525 ± 0.140	+	1.84E-04	*1.14E-03
k__Bacteria.p__Firmicutes.c__Clostridia.o__Clostridiales.f__Christensenellaceae.g__Christensenellaceae_unclassified	sex	0.083 ± 0.062	+	0.183	
k__Bacteria.p__Firmicutes.c__Clostridia.o__Clostridiales.f__Christensenellaceae.g__Christensenellaceae_unclassified	anticholesterol drugs	0.237 ± 0.064	+	2.09E-04	
k__Bacteria.p__Firmicutes.c__Clostridia.o__Clostridiales.f__Clostridiaceae.g__Hungatella	age	0.079 ± 0.072	+	0.269	
k__Bacteria.p__Firmicutes.c__Clostridia.o__Clostridiales.f__Clostridiaceae.g__Hungatella	BMI	-0.101 ± 0.079	-	0.201	
k__Bacteria.p__Firmicutes.c__Clostridia.o__Clostridiales.f__Clostridiaceae.g__Hungatella	constipation	0.451 ± 0.069	+	8.02E-11	
k__Bacteria.p__Firmicutes.c__Clostridia.o__Clostridiales.f__Clostridiaceae.g__Hungatella	disease	0.399 ± 0.163	+	0.0144	*0.0279
k__Bacteria.p__Firmicutes.c__Clostridia.o__Clostridiales.f__Clostridiaceae.g__Hungatella	sex	0.171 ± 0.070	+	0.0145	
k__Bacteria.p__Firmicutes.c__Clostridia.o__Clostridiales.f__Clostridiaceae.g__Hungatella	anticholesterol drugs	-0.037 ± 0.071	-	0.599	
k__Bacteria.p__Firmicutes.c__Clostridia.o__Clostridiales.f__Clostridiales_unclassified.g__Intestinimonas	age	0.165 ± 0.059	+	5.32E-03	
k__Bacteria.p__Firmicutes.c__Clostridia.o__Clostridiales.f__Clostridiales_unclassified.g__Intestinimonas	BMI	-0.079 ± 0.059	-	0.182	
k__Bacteria.p__Firmicutes.c__Clostridia.o__Clostridiales.f__Clostridiales_unclassified.g__Intestinimonas	constipation	0.160 ± 0.057	+	4.88E-03	
k__Bacteria.p__Firmicutes.c__Clostridia.o__Clostridiales.f__Clostridiales_unclassified.g__Intestinimonas	disease	0.278 ± 0.133	+	0.037	0.0604
k__Bacteria.p__Firmicutes.c__Clostridia.o__Clostridiales.f__Clostridiales_unclassified.g__Intestinimonas	sex	0.205 ± 0.061	+	6.87E-04	
k__Bacteria.p__Firmicutes.c__Clostridia.o__Clostridiales.f__Clostridiales_unclassified.g__Intestinimonas	anticholesterol drugs	0.175 ± 0.058	+	2.49E-03	
k__Bacteria.p__Firmicutes.c__Clostridia.o__Clostridiales.f__Eubacteriaceae.g__Eubacteriaceae_unclassified	age	0.276 ± 0.094	+	3.34E-03	
k__Bacteria.p__Firmicutes.c__Clostridia.o__Clostridiales.f__Eubacteriaceae.g__Eubacteriaceae_unclassified	BMI	-0.415 ± 0.106	-	9.15E-05	
k__Bacteria.p__Firmicutes.c__Clostridia.o__Clostridiales.f__Eubacteriaceae.g__Eubacteriaceae_unclassified	constipation	0.159 ± 0.089	+	0.0737	
k__Bacteria.p__Firmicutes.c__Clostridia.o__Clostridiales.f__Eubacteriaceae.g__Eubacteriaceae_unclassified	disease	0.100 ± 0.191	+	0.601	0.601
k__Bacteria.p__Firmicutes.c__Clostridia.o__Clostridiales.f__Eubacteriaceae.g__Eubacteriaceae_unclassified	sex	-0.003 ± 0.085	-	0.973	
k__Bacteria.p__Firmicutes.c__Clostridia.o__Clostridiales.f__Eubacteriaceae.g__Eubacteriaceae_unclassified	anticholesterol drugs	-0.196 ± 0.089	-	0.028	
k__Bacteria.p__Firmicutes.c__Clostridia.o__Clostridiales.f__Lachnospiraceae.g__Dorea	age	-0.184 ± 0.045	-	3.90E-05	
k__Bacteria.p__Firmicutes.c__Clostridia.o__Clostridiales.f__Lachnospiraceae.g__Dorea	BMI	-0.018 ± 0.050	-	0.720	
k__Bacteria.p__Firmicutes.c__Clostridia.o__Clostridiales.f__Lachnospiraceae.g__Dorea	constipation	-0.105 ± 0.046	-	0.023	
k__Bacteria.p__Firmicutes.c__Clostridia.o__Clostridiales.f__Lachnospiraceae.g__Dorea	disease	-0.152 ± 0.096	-	0.113	0.167
k__Bacteria.p__Firmicutes.c__Clostridia.o__Clostridiales.f__Lachnospiraceae.g__Dorea	sex	-0.197 ± 0.044	-	8.41E-06	
k__Bacteria.p__Firmicutes.c__Clostridia.o__Clostridiales.f__Lachnospiraceae.g__Dorea	anticholesterol drugs	-0.019 ± 0.046	-	0.684	
k__Bacteria.p__Firmicutes.c__Clostridia.o__Clostridiales.f__Lachnospiraceae.g__Eisenbergiella	age	0.226 ± 0.059	+	1.37E-04	
k__Bacteria.p__Firmicutes.c__Clostridia.o__Clostridiales.f__Lachnospiraceae.g__Eisenbergiella	BMI	-0.113 ± 0.078	-	0.152	
k__Bacteria.p__Firmicutes.c__Clostridia.o__Clostridiales.f__Lachnospiraceae.g__Eisenbergiella	constipation	0.220 ± 0.062	+	4.32E-04	

k__Bacteria.p__Firmicutes.c__Clostridia.o__Clostridiales.f__Lachnospiraceae.g__Eisenbergiella	disease	0.602 ± 0.146	+	3.90E-05	*3.02E-04
k__Bacteria.p__Firmicutes.c__Clostridia.o__Clostridiales.f__Lachnospiraceae.g__Eisenbergiella	sex	0.351 ± 0.068	+	2.27E-07	
k__Bacteria.p__Firmicutes.c__Clostridia.o__Clostridiales.f__Lachnospiraceae.g__Eisenbergiella	anticholesterol drugs	-0.021 ± 0.069	-	0.765	
k__Bacteria.p__Firmicutes.c__Clostridia.o__Clostridiales.f__Lachnospiraceae.g__Lachnoclostridium	age	0.182 ± 0.054	+	7.63E-04	
k__Bacteria.p__Firmicutes.c__Clostridia.o__Clostridiales.f__Lachnospiraceae.g__Lachnoclostridium	BMI	-0.003 ± 0.059	-	0.954	
k__Bacteria.p__Firmicutes.c__Clostridia.o__Clostridiales.f__Lachnospiraceae.g__Lachnoclostridium	constipation	0.179 ± 0.056	+	1.35E-03	
k__Bacteria.p__Firmicutes.c__Clostridia.o__Clostridiales.f__Lachnospiraceae.g__Lachnoclostridium	disease	0.081 ± 0.126	+	0.520	0.538
k__Bacteria.p__Firmicutes.c__Clostridia.o__Clostridiales.f__Lachnospiraceae.g__Lachnoclostridium	sex	0.361 ± 0.056	+	1.45E-10	
k__Bacteria.p__Firmicutes.c__Clostridia.o__Clostridiales.f__Lachnospiraceae.g__Lachnoclostridium	anticholesterol drugs	0.047 ± 0.056	+	0.401	
k__Bacteria.p__Firmicutes.c__Clostridia.o__Clostridiales.f__Lachnospiraceae.g__Lacrimispora	age	-0.197 ± 0.056	-	4.19E-04	
k__Bacteria.p__Firmicutes.c__Clostridia.o__Clostridiales.f__Lachnospiraceae.g__Lacrimispora	BMI	0.120 ± 0.057	+	0.0365	
k__Bacteria.p__Firmicutes.c__Clostridia.o__Clostridiales.f__Lachnospiraceae.g__Lacrimispora	constipation	-0.125 ± 0.059	-	0.034	
k__Bacteria.p__Firmicutes.c__Clostridia.o__Clostridiales.f__Lachnospiraceae.g__Lacrimispora	disease	-0.115 ± 0.120	-	0.339	0.375
k__Bacteria.p__Firmicutes.c__Clostridia.o__Clostridiales.f__Lachnospiraceae.g__Lacrimispora	sex	-0.103 ± 0.055	-	0.0614	
k__Bacteria.p__Firmicutes.c__Clostridia.o__Clostridiales.f__Lachnospiraceae.g__Lacrimispora	anticholesterol drugs	-0.028 ± 0.056	-	0.617	
k__Bacteria.p__Firmicutes.c__Clostridia.o__Clostridiales.f__Lachnospiraceae.g__Roseburia	age	-0.041 ± 0.046	-	0.366	
k__Bacteria.p__Firmicutes.c__Clostridia.o__Clostridiales.f__Lachnospiraceae.g__Roseburia	BMI	0.047 ± 0.045	+	0.296	
k__Bacteria.p__Firmicutes.c__Clostridia.o__Clostridiales.f__Lachnospiraceae.g__Roseburia	constipation	-0.189 ± 0.047	-	5.54E-05	
k__Bacteria.p__Firmicutes.c__Clostridia.o__Clostridiales.f__Lachnospiraceae.g__Roseburia	disease	-0.416 ± 0.098	-	2.03E-05	*3.02E-04
k__Bacteria.p__Firmicutes.c__Clostridia.o__Clostridiales.f__Lachnospiraceae.g__Roseburia	sex	-0.125 ± 0.045	-	5.54E-03	
k__Bacteria.p__Firmicutes.c__Clostridia.o__Clostridiales.f__Lachnospiraceae.g__Roseburia	anticholesterol drugs	0.029 ± 0.045	+	0.524	
k__Bacteria.p__Firmicutes.c__Clostridia.o__Clostridiales.f__Oscillospiraceae.g__Dysosmobacter	age	0.008 ± 0.036	+	0.817	
k__Bacteria.p__Firmicutes.c__Clostridia.o__Clostridiales.f__Oscillospiraceae.g__Dysosmobacter	BMI	0.073 ± 0.039	+	0.0603	
k__Bacteria.p__Firmicutes.c__Clostridia.o__Clostridiales.f__Oscillospiraceae.g__Dysosmobacter	constipation	0.022 ± 0.036	+	0.536	
k__Bacteria.p__Firmicutes.c__Clostridia.o__Clostridiales.f__Oscillospiraceae.g__Dysosmobacter	disease	0.218 ± 0.080	+	6.18E-03	*0.0174
k__Bacteria.p__Firmicutes.c__Clostridia.o__Clostridiales.f__Oscillospiraceae.g__Dysosmobacter	sex	0.184 ± 0.036	+	3.86E-07	
k__Bacteria.p__Firmicutes.c__Clostridia.o__Clostridiales.f__Oscillospiraceae.g__Dysosmobacter	anticholesterol drugs	0.033 ± 0.036	+	0.362	
k__Bacteria.p__Firmicutes.c__Clostridia.o__Clostridiales.f__Ruminococcaceae.g__Agathobaculum	age	-0.116 ± 0.049	-	0.0187	
k__Bacteria.p__Firmicutes.c__Clostridia.o__Clostridiales.f__Ruminococcaceae.g__Agathobaculum	BMI	-0.042 ± 0.050	-	0.401	
k__Bacteria.p__Firmicutes.c__Clostridia.o__Clostridiales.f__Ruminococcaceae.g__Agathobaculum	constipation	-0.253 ± 0.055	-	3.80E-06	
k__Bacteria.p__Firmicutes.c__Clostridia.o__Clostridiales.f__Ruminococcaceae.g__Agathobaculum	disease	-0.321 ± 0.108	-	2.85E-03	*8.84E-03
k__Bacteria.p__Firmicutes.c__Clostridia.o__Clostridiales.f__Ruminococcaceae.g__Agathobaculum	sex	-0.106 ± 0.051	-	0.0377	
k__Bacteria.p__Firmicutes.c__Clostridia.o__Clostridiales.f__Ruminococcaceae.g__Agathobaculum	anticholesterol drugs	-0.040 ± 0.051	-	0.435	
k__Bacteria.p__Firmicutes.c__Clostridia.o__Clostridiales.f__Ruminococcaceae.g__Anaeromassilibacillus	age	0.296 ± 0.085	+	5.44E-04	
k__Bacteria.p__Firmicutes.c__Clostridia.o__Clostridiales.f__Ruminococcaceae.g__Anaeromassilibacillus	BMI	0.031 ± 0.088	+	0.729	
k__Bacteria.p__Firmicutes.c__Clostridia.o__Clostridiales.f__Ruminococcaceae.g__Anaeromassilibacillus	constipation	0.145 ± 0.081	+	0.0748	
k__Bacteria.p__Firmicutes.c__Clostridia.o__Clostridiales.f__Ruminococcaceae.g__Anaeromassilibacillus	disease	-0.419 ± 0.180	-	0.0197	*0.0339
k__Bacteria.p__Firmicutes.c__Clostridia.o__Clostridiales.f__Ruminococcaceae.g__Anaeromassilibacillus	sex	0.068 ± 0.080	+	0.395	
k__Bacteria.p__Firmicutes.c__Clostridia.o__Clostridiales.f__Ruminococcaceae.g__Anaeromassilibacillus	anticholesterol drugs	0.048 ± 0.085	+	0.574	
k__Bacteria.p__Firmicutes.c__Clostridia.o__Clostridiales.f__Ruminococcaceae.g__Anaerotruncus	age	0.138 ± 0.058	+	0.018	
k__Bacteria.p__Firmicutes.c__Clostridia.o__Clostridiales.f__Ruminococcaceae.g__Anaerotruncus	BMI	-0.068 ± 0.066	-	0.303	
k__Bacteria.p__Firmicutes.c__Clostridia.o__Clostridiales.f__Ruminococcaceae.g__Anaerotruncus	constipation	0.349 ± 0.055	+	1.95E-10	
k__Bacteria.p__Firmicutes.c__Clostridia.o__Clostridiales.f__Ruminococcaceae.g__Anaerotruncus	disease	0.472 ± 0.132	+	3.69E-04	*1.91E-03
k__Bacteria.p__Firmicutes.c__Clostridia.o__Clostridiales.f__Ruminococcaceae.g__Anaerotruncus	sex	0.055 ± 0.060	+	0.358	
k__Bacteria.p__Firmicutes.c__Clostridia.o__Clostridiales.f__Ruminococcaceae.g__Anaerotruncus	anticholesterol drugs	0.104 ± 0.057	+	0.0654	
k__Bacteria.p__Firmicutes.c__Clostridia.o__Clostridiales.f__Ruminococcaceae.g__Faecalibacterium	age	-0.111 ± 0.048	-	0.0204	
k__Bacteria.p__Firmicutes.c__Clostridia.o__Clostridiales.f__Ruminococcaceae.g__Faecalibacterium	BMI	-0.036 ± 0.048	-	0.446	
k__Bacteria.p__Firmicutes.c__Clostridia.o__Clostridiales.f__Ruminococcaceae.g__Faecalibacterium	constipation	-0.279 ± 0.048	-	6.71E-09	
k__Bacteria.p__Firmicutes.c__Clostridia.o__Clostridiales.f__Ruminococcaceae.g__Faecalibacterium	disease	-0.253 ± 0.099	-	0.0103	*0.022
k__Bacteria.p__Firmicutes.c__Clostridia.o__Clostridiales.f__Ruminococcaceae.g__Faecalibacterium	sex	-0.063 ± 0.046	-	0.172	
k__Bacteria.p__Firmicutes.c__Clostridia.o__Clostridiales.f__Ruminococcaceae.g__Faecalibacterium	anticholesterol drugs	0.080 ± 0.047	+	0.0859	
k__Bacteria.p__Firmicutes.c__Clostridia.o__Clostridiales.f__Ruminococcaceae.g__GGB9699	age	0.160 ± 0.077	+	0.0377	
k__Bacteria.p__Firmicutes.c__Clostridia.o__Clostridiales.f__Ruminococcaceae.g__GGB9699	BMI	-0.102 ± 0.082	-	0.212	
k__Bacteria.p__Firmicutes.c__Clostridia.o__Clostridiales.f__Ruminococcaceae.g__GGB9699	constipation	0.246 ± 0.073	+	8.01E-04	
k__Bacteria.p__Firmicutes.c__Clostridia.o__Clostridiales.f__Ruminococcaceae.g__GGB9699	disease	0.409 ± 0.171	+	0.0167	*0.0304
k__Bacteria.p__Firmicutes.c__Clostridia.o__Clostridiales.f__Ruminococcaceae.g__GGB9699	sex	-0.003 ± 0.075	-	0.969	
k__Bacteria.p__Firmicutes.c__Clostridia.o__Clostridiales.f__Ruminococcaceae.g__GGB9699	anticholesterol drugs	-0.006 ± 0.073	-	0.931	
k__Bacteria.p__Firmicutes.c__Clostridia.o__Clostridiales.f__Ruminococcaceae.g__Ruminococcaceae_unclassified	age	0.101 ± 0.040	+	0.0113	
k__Bacteria.p__Firmicutes.c__Clostridia.o__Clostridiales.f__Ruminococcaceae.g__Ruminococcaceae_unclassified	BMI	-0.024 ± 0.043	-	0.588	
k__Bacteria.p__Firmicutes.c__Clostridia.o__Clostridiales.f__Ruminococcaceae.g__Ruminococcaceae_unclassified	constipation	0.032 ± 0.040	+	0.423	
k__Bacteria.p__Firmicutes.c__Clostridia.o__Clostridiales.f__Ruminococcaceae.g__Ruminococcaceae_unclassified	disease	0.136 ± 0.089	+	0.127	0.173
k__Bacteria.p__Firmicutes.c__Clostridia.o__Clostridiales.f__Ruminococcaceae.g__Ruminococcaceae_unclassified	sex	-0.066 ± 0.041	-	0.111	
k__Bacteria.p__Firmicutes.c__Clostridia.o__Clostridiales.f__Ruminococcaceae.g__Ruminococcaceae_unclassified	anticholesterol drugs	-0.015 ± 0.041	-	0.717	
k__Bacteria.p__Firmicutes.c__Clostridia.o__Clostridiales.f__Ruminococcaceae.g__Ruthenibacterium	age	0.130 ± 0.043	+	2.65E-03	
k__Bacteria.p__Firmicutes.c__Clostridia.o__Clostridiales.f__Ruminococcaceae.g__Ruthenibacterium	BMI	0.077 ± 0.047	+	0.0991	
k__Bacteria.p__Firmicutes.c__Clostridia.o__Clostridiales.f__Ruminococcaceae.g__Ruthenibacterium	constipation	0.307 ± 0.043	+	1.10E-12	
k__Bacteria.p__Firmicutes.c__Clostridia.o__Clostridiales.f__Ruminococcaceae.g__Ruthenibacterium	disease	0.800 ± 0.101	+	2.62E-15	*8.11E-14
k__Bacteria.p__Firmicutes.c__Clostridia.o__Clostridiales.f__Ruminococcaceae.g__Ruthenibacterium	sex	0.337 ± 0.044	+	3.46E-14	
k__Bacteria.p__Firmicutes.c__Clostridia.o__Clostridiales.f__Ruminococcaceae.g__Ruthenibacterium	anticholesterol drugs	-0.016 ± 0.045	-	0.718	
k__Bacteria.p__Firmicutes.c__Erysipelotrichia.o__Erysipelotrichales.f__Erysipelotrichaceae.g__Holdemania	age	0.043 ± 0.047	+	0.359	
k__Bacteria.p__Firmicutes.c__Erysipelotrichia.o__Erysipelotrichales.f__Erysipelotrichaceae.g__Holdemania	BMI	-0.039 ± 0.052	-	0.452	
k__Bacteria.p__Firmicutes.c__Erysipelotrichia.o__Erysipelotrichales.f__Erysipelotrichaceae.g__Holdemania	constipation	-0.026 ± 0.045	-	0.572	
k__Bacteria.p__Firmicutes.c__Erysipelotrichia.o__Erysipelotrichales.f__Erysipelotrichaceae.g__Holdemania	disease	0.266 ± 0.101	+	8.19E-03	*0.0198
k__Bacteria.p__Firmicutes.c__Erysipelotrichia.o__Erysipelotrichales.f__Erysipelotrichaceae.g__Holdemania	sex	0.004 ± 0.046	+	0.924	
k__Bacteria.p__Firmicutes.c__Erysipelotrichia.o__Erysipelotrichales.f__Erysipelotrichaceae.g__Holdemania	anticholesterol drugs	0.086 ± 0.046	+	0.0634	
k__Bacteria.p__Proteobacteria.c__Deltaproteobacteria.o__Desulfovibrionales.f__Desulfovibrionaceae.g__Bilophila	age	-0.018 ± 0.045	-	0.694	
k__Bacteria.p__Proteobacteria.c__Deltaproteobacteria.o__Desulfovibrionales.f__Desulfovibrionaceae.g__Bilophila	BMI	0.019 ± 0.048	+	0.688	
k__Bacteria.p__Proteobacteria.c__Deltaproteobacteria.o__Desulfovibrionales.f__Desulfovibrionaceae.g__Bilophila	constipation	0.054 ± 0.044	+	0.216	
k__Bacteria.p__Proteobacteria.c__Deltaproteobacteria.o__Desulfovibrionales.f__Desulfovibrionaceae.g__Bilophila	disease	0.160 ± 0.100	+	0.111	0.167

k__Bacteria.p__Proteobacteria.c__Deltaproteobacteria.o__Desulfovibrionales.f__Desulfovibrionaceae.g__Bilophila	sex	-0.022 ± 0.045	-	0.622	
k__Bacteria.p__Proteobacteria.c__Deltaproteobacteria.o__Desulfovibrionales.f__Desulfovibrionaceae.g__Bilophila	anticholesterol drugs	-0.033 ± 0.044	-	0.454	
k__Bacteria.p__Proteobacteria.c__Deltaproteobacteria.o__Desulfovibrionales.f__Desulfovibrionaceae.g__Desulfovibrio	age	0.174 ± 0.078	+	0.0252	
k__Bacteria.p__Proteobacteria.c__Deltaproteobacteria.o__Desulfovibrionales.f__Desulfovibrionaceae.g__Desulfovibrio	BMI	-0.065 ± 0.079	-	0.410	
k__Bacteria.p__Proteobacteria.c__Deltaproteobacteria.o__Desulfovibrionales.f__Desulfovibrionaceae.g__Desulfovibrio	constipation	0.219 ± 0.068	+	1.26E-03	
k__Bacteria.p__Proteobacteria.c__Deltaproteobacteria.o__Desulfovibrionales.f__Desulfovibrionaceae.g__Desulfovibrio	disease	0.446 ± 0.169	+	8.30E-03	*0.0198
k__Bacteria.p__Proteobacteria.c__Deltaproteobacteria.o__Desulfovibrionales.f__Desulfovibrionaceae.g__Desulfovibrio	sex	-0.176 ± 0.074	-	0.0169	
k__Bacteria.p__Proteobacteria.c__Deltaproteobacteria.o__Desulfovibrionales.f__Desulfovibrionaceae.g__Desulfovibrio	anticholesterol drugs	-0.065 ± 0.071	-	0.359	
k__Bacteria.p__Proteobacteria.c__Gammaproteobacteria.o__Pasteurellales.f__Pasteurellaceae.g__Haemophilus	age	0.177 ± 0.147	+	0.230	
k__Bacteria.p__Proteobacteria.c__Gammaproteobacteria.o__Pasteurellales.f__Pasteurellaceae.g__Haemophilus	BMI	-0.070 ± 0.169	-	0.676	
k__Bacteria.p__Proteobacteria.c__Gammaproteobacteria.o__Pasteurellales.f__Pasteurellaceae.g__Haemophilus	constipation	-0.457 ± 0.152	-	2.70E-03	
k__Bacteria.p__Proteobacteria.c__Gammaproteobacteria.o__Pasteurellales.f__Pasteurellaceae.g__Haemophilus	disease	-0.365 ± 0.295	-	0.215	0.256
k__Bacteria.p__Proteobacteria.c__Gammaproteobacteria.o__Pasteurellales.f__Pasteurellaceae.g__Haemophilus	sex	-0.136 ± 0.143	-	0.341	
k__Bacteria.p__Proteobacteria.c__Gammaproteobacteria.o__Pasteurellales.f__Pasteurellaceae.g__Haemophilus	anticholesterol drugs	-0.190 ± 0.142	-	0.181	
k__Bacteria.p__Verrucomicrobia.c__Verrucomicrobiae.o__Verrucomicrobiales.f__Akkermansiaceae.g__Akkermansia	age	0.146 ± 0.069	+	0.0329	
k__Bacteria.p__Verrucomicrobia.c__Verrucomicrobiae.o__Verrucomicrobiales.f__Akkermansiaceae.g__Akkermansia	BMI	-0.110 ± 0.071	-	0.121	
k__Bacteria.p__Verrucomicrobia.c__Verrucomicrobiae.o__Verrucomicrobiales.f__Akkermansiaceae.g__Akkermansia	constipation	0.248 ± 0.062	+	6.86E-05	
k__Bacteria.p__Verrucomicrobia.c__Verrucomicrobiae.o__Verrucomicrobiales.f__Akkermansiaceae.g__Akkermansia	disease	0.439 ± 0.143	+	2.21E-03	*7.63E-03
k__Bacteria.p__Verrucomicrobia.c__Verrucomicrobiae.o__Verrucomicrobiales.f__Akkermansiaceae.g__Akkermansia	sex	0.074 ± 0.066	+	0.258	
k__Bacteria.p__Verrucomicrobia.c__Verrucomicrobiae.o__Verrucomicrobiales.f__Akkermansiaceae.g__Akkermansia	anticholesterol drugs	0.058 ± 0.063	+	0.357	

False discovery rates (q -values) for the disease state were calculated for the 38 species and the 31 genera using the Benjamini-Hochberg method.

*q -value < 0.05

Supplementary Table 9 Meta-analysis of CAZymes in six datasets

Cazymes	Cazymes	Increased or decreased in PD	*p* -value (FEM)	*q* -value (FEM)	*p* -value (REM)	*q* -value (REM)	I^2 (%)
GH127	Glycoside Hydrolase 127	-	< 1.0E-16	*< 1.0E-16	< 1.0E-16	*< 1.0E-16	*0.00
GH53	Glycoside Hydrolase 53	-	7.20E-13	*9.67E-12	7.20E-13	*7.89E-11	*0.00
GH51	Glycoside Hydrolase 51	-	2.59E-12	*2.99E-11	2.59E-12	*1.89E-10	*0.00
GT80	Glycosyltransferase 80	+	2.84E-14	*6.22E-13	5.21E-11	*2.85E-09	*25.50
GH31	Glycoside Hydrolase 31	-	8.81E-11	*8.04E-10	8.81E-11	*3.86E-09	*0.00
GH36	Glycoside Hydrolase 36	-	8.01E-11	*7.62E-10	8.94E-10	*3.26E-08	*11.15
GH133	Glycoside Hydrolase 133	-	1.31E-09	*9.55E-09	5.04E-09	*1.58E-07	*7.14
CBM9	Carbohydrate Binding Module 9	-	< 1.0E-16	*< 1.0E-16	1.03E-07	*2.81E-06	68.57
CBM6	Carbohydrate Binding Module 6	-	1.85E-07	9.64E-07	1.85E-07	*4.50E-06	*0.00
CE12	Carbohydrate Esterase 12	-	< 1.0E-16	*< 1.0E-16	2.59E-07	*5.67E-06	65.71
GH2	Glycoside Hydrolase 2	-	1.57E-08	*1.04E-07	3.19E-07	*6.36E-06	*18.25
GH164	Glycoside Hydrolase 164	+	2.49E-08	*1.56E-07	4.53E-07	*8.26E-06	*18.06
GH77	Glycoside Hydrolase 77	-	6.29E-07	*2.65E-06	6.29E-07	*1.06E-05	*0.00
CE8	Carbohydrate Esterase 8	-	1.21E-13	*2.20E-12	9.09E-07	*1.42E-05	56.16
GT10	Glycosyltransferase 10	+	1.55E-15	*4.86E-14	1.07E-06	*1.56E-05	62.55
GT84	Glycosyltransferase 84	+	< 1.0E-16	*< 1.0E-16	2.23E-06	*2.87E-05	76.75
GH67	Glycoside Hydrolase 67	-	2.57E-08	*1.56E-07	2.23E-06	*2.87E-05	*27.80
GH10	Glycoside Hydrolase 10	-	2.72E-06	*9.75E-06	2.72E-06	*3.30E-05	*0.00
GH115	Glycoside Hydrolase 115	-	5.77E-14	*1.15E-12	3.01E-06	*3.47E-05	61.36
GT39	Glycosyltransferase 39	+	2.82E-11	*2.81E-10	3.29E-06	*3.61E-05	51.15
GH130	Glycoside Hydrolase 130	-	2.71E-14	*6.22E-13	3.79E-06	*3.95E-05	63.11
GH32	Glycoside Hydrolase 32	-	3.29E-12	*3.60E-11	7.04E-06	*7.00E-05	58.39
GH3	Glycoside Hydrolase 3	-	7.37E-06	*2.31E-05	7.37E-06	*7.02E-05	*0.00
GH28	Glycoside Hydrolase 28	-	< 1.0E-16	*< 1.0E-16	7.76E-06	*7.08E-05	72.64
GT66	Glycosyltransferase 66	+	< 1.0E-16	*< 1.0E-16	9.73E-06	*8.35E-05	72.96
CE6	Carbohydrate Esterase 6	-	9.91E-06	*3.01E-05	9.91E-06	*8.35E-05	*0.00
GT30	Glycosyltransferase 30	-	1.24E-05	*3.68E-05	1.24E-05	*1.01E-04	*0.00
GT83	Glycosyltransferase 83	-	7.75E-07	*3.20E-06	1.36E-05	*1.06E-04	*22.51
GH23	Glycoside Hydrolase 23	-	7.51E-13	*9.67E-12	1.51E-05	*1.14E-04	63.57
GH95	Glycoside Hydrolase 95	-	1.73E-13	*2.91E-12	2.45E-05	*1.79E-04	67.20
PL9	Polysaccharide Lyase 9	-	2.43E-06	*8.87E-06	2.59E-05	*1.83E-04	*20.34
GH43	Glycoside Hydrolase 43	-	1.55E-10	*1.31E-09	2.83E-05	*1.92E-04	57.21
GH97	Glycoside Hydrolase 97	-	1.25E-09	*9.47E-09	2.89E-05	*1.92E-04	52.58
GH146	Glycoside Hydrolase 146	-	1.01E-07	*5.83E-07	3.75E-05	*2.41E-04	40.05
GH38	Glycoside Hydrolase 38	+	6.39E-05	*1.69E-04	6.39E-05	*4.00E-04	*0.00
CBM20	Carbohydrate Binding Module 20	-	2.18E-13	*3.41E-12	1.57E-04	*9.56E-04	73.47
GT2	Glycosyltransferase 2	-	2.10E-06	*7.94E-06	1.64E-04	*9.69E-04	*36.84
CE11	Carbohydrate Esterase 11	-	1.76E-04	*4.25E-04	1.76E-04	*1.02E-03	*0.00
GH30	Glycoside Hydrolase 30	-	2.33E-09	*1.64E-08	1.82E-04	*1.02E-03	60.73
GT19	Glycosyltransferase 19	-	4.79E-07	*2.19E-06	1.97E-04	*1.08E-03	45.33
CE1	Carbohydrate Esterase 1	-	5.43E-06	*1.78E-05	2.38E-04	*1.27E-03	*34.7
GH136	Glycoside Hydrolase 136	-	2.16E-06	*8.02E-06	3.32E-04	*1.65E-03	42.61
CBM51	Carbohydrate Binding Module 51	+	8.76E-05	*2.16E-04	3.21E-04	*1.65E-03	*15.88
GH63	Glycoside Hydrolase 63	-	3.31E-04	*7.56E-04	3.31E-04	*1.65E-03	*0.00
GH29	Glycoside Hydrolase 29	-	6.89E-13	*9.67E-12	3.86E-04	*1.88E-03	75.57
CBM13	Carbohydrate Binding Module 13	-	2.60E-04	*6.11E-04	4.35E-04	*2.07E-03	*7.25
GH25	Glycoside Hydrolase 25	-	3.99E-05	*1.09E-04	5.23E-04	*2.44E-03	*28.7
PL1	Polysaccharide Lyase 1	-	7.73E-09	*5.29E-08	5.35E-04	*2.44E-03	64.04
CBM83	Carbohydrate Binding Module 83	-	1.25E-05	*3.68E-05	6.36E-04	*2.84E-03	*38.9
PL11	Polysaccharide Lyase 11	-	1.08E-12	*1.31E-11	8.20E-04	*3.59E-03	77.92
GH105	Glycoside Hydrolase 105	-	2.84E-14	*6.22E-13	1.03E-03	*4.41E-03	81.37
CBM82	Carbohydrate Binding Module 82	-	4.06E-04	*9.07E-04	1.12E-03	*4.72E-03	*15.09
PL10	Polysaccharide Lyase 10	-	1.27E-10	*1.11E-09	1.21E-03	*4.96E-03	74.66
GH142	Glycoside Hydrolase 142	-	7.68E-06	*2.37E-05	1.22E-03	*4.96E-03	47.76
GH42	Glycoside Hydrolase 42	-	7.17E-05	*1.84E-04	1.25E-03	*4.97E-03	*33.93
GT26	Glycosyltransferase 26	-	6.09E-07	*2.62E-06	1.41E-03	*5.50E-03	59.02
GH143	Glycoside Hydrolase 143	-	5.33E-07	*2.38E-06	1.47E-03	*5.65E-03	59.76
GT23	Glycosyltransferase 23	-	5.92E-07	*2.59E-06	1.77E-03	*6.68E-03	60.80
PL26	Polysaccharide Lyase 26	-	2.46E-11	*2.56E-10	1.99E-03	*7.39E-03	78.55
GT113	Glycosyltransferase 113	-	2.78E-03	*5.54E-03	2.78E-03	*0.010	*0.00
CBM50	Carbohydrate Binding Module 50	-	2.95E-07	*1.44E-06	3.25E-03	*0.011	67.04
GT9	Glycosyltransferase 9	-	3.85E-05	*1.07E-04	3.21E-03	*0.011	48.74
PL38	Polysaccharide Lyase 38	+	7.67E-05	*1.93E-04	3.21E-03	*0.011	44.46
GT3	Glycosyltransferase 3	-	7.22E-05	*1.84E-04	3.37E-03	*0.012	45.41
PL42	Polysaccharide Lyase 42	-	1.30E-07	*7.30E-07	4.06E-03	*0.014	70.37
GT25	Glycosyltransferase 25	+	5.51E-03	*0.010	5.51E-03	*0.018	*0.00
CE9	Carbohydrate Esterase 9	-	1.66E-10	**1.35E-09	5.64E-03	*0.018	81.23
GH113	Glycoside Hydrolase 113	-	7.92E-07	**3.21E-06	5.83E-03	*0.019	68.81
GH79	Glycoside Hydrolase 79	+	6.10E-03	*0.011	6.10E-03	*0.019	*0.00
GH116	Glycoside Hydrolase 116	+	6.52E-05	*1.70E-04	6.81E-03	*0.021	54.09
CBM66	Carbohydrate Binding Module 66	+	7.09E-03	*0.013	7.09E-03	*0.022	*0.00
GH140	Glycoside Hydrolase 140	-	5.31E-10	*4.16E-09	8.45E-03	*0.025	82.01
CE7	Carbohydrate Esterase 7	-	8.14E-07	*3.24E-06	8.37E-03	*0.025	71.42
GH65	Glycoside Hydrolase 65	+	1.19E-03	*2.49E-03	8.35E-03	*0.025	*33.75
GH161	Glycoside Hydrolase 161	+	9.46E-03	*0.017	9.46E-03	*0.028	*0.00
GH73	Glycoside Hydrolase 73	-	3.24E-07	*1.54E-06	9.60E-03	*0.028	74.30

GH121	Glycoside Hydrolase 121	+	2.97E-07	*1.44E-06	0.010	*0.029	74.90
PL27	Polysaccharide Lyase 27	-	4.58E-05	*1.24E-04	0.011	*0.03	60.94
CBM38	Carbohydrate Binding Module 38	+	9.88E-04	*2.08E-03	0.011	*0.031	40.43
CE19	Carbohydrate Esterase 19	-	5.10E-06	*1.72E-05	0.012	*0.032	69.51
GH154	Glycoside Hydrolase 154	-	2.07E-07	*1.05E-06	0.012	*0.033	76.77
CBM77	Carbohydrate Binding Module 77	-	0.013	*0.022	0.013	*0.034	*0.00
CBM27	Carbohydrate Binding Module 27	-	3.55E-06	*1.23E-05	0.013	*0.034	71.32
GT5	Glycosyltransferase 5	-	9.06E-05	*2.20E-04	0.016	*0.042	62.27
GH84	Glycoside Hydrolase 84	-	3.45E-07	*1.61E-06	0.017	*0.045	78.18
CBM23	Carbohydrate Binding Module 23	-	2.09E-03	*4.24E-03	0.018	*0.045	40.60
GH35	Glycoside Hydrolase 35	-	1.32E-05	*3.86E-05	0.019	*0.047	70.87
CE17	Carbohydrate Esterase 17	-	2.38E-08	*1.54E-07	0.020	*0.050	82.61
GH171	Glycoside Hydrolase 171	-	4.79E-03	*9.20E-03	0.020	*0.050	*32.3
PL37	Polysaccharide Lyase 37	+	0.021	*0.035	0.021	0.051	*0.00
CBM32	Carbohydrate Binding Module 32	-	1.42E-07	*7.75E-07	0.022	0.053	81.15
GH78	Glycoside Hydrolase 78	-	2.62E-05	*7.46E-05	0.022	0.053	70.51
GH24	Glycoside Hydrolase 24	-	5.07E-05	*1.35E-04	0.022	0.053	68.27
GH88	Glycoside Hydrolase 88	-	1.44E-03	*2.94E-03	0.023	0.053	48.79
GH92	Glycoside Hydrolase 92	-	1.11E-06	*4.35E-06	0.023	0.053	78.17
CBM35	Carbohydrate Binding Module 35	-	3.05E-06	*1.08E-05	0.024	0.054	76.50
GH101	Glycoside Hydrolase 101	+	3.69E-03	*7.28E-03	0.027	0.061	41.87
GH137	Glycoside Hydrolase 137	-	7.11E-06	*2.26E-05	0.028	0.062	76.04
GH57	Glycoside Hydrolase 57	-	5.86E-03	*0.011	0.029	0.064	*37.28
GH109	Glycoside Hydrolase 109	-	8.17E-08	*4.84E-07	0.030	0.066	83.67
GH138	Glycoside Hydrolase 138	-	6.00E-06	*1.93E-05	0.032	0.070	77.57
GH139	Glycoside Hydrolase 139	-	1.50E-05	*4.32E-05	0.033	0.071	75.71
CBM5	Carbohydrate Binding Module 5	+	1.47E-07	*7.88E-07	0.034	0.071	83.75
GH106	Glycoside Hydrolase 106	-	3.23E-05	*9.07E-05	0.034	0.071	74.06
PL4	Polysaccharide Lyase 4	-	0.034	0.055	0.034	0.071	*0.00
CE14	Carbohydrate Esterase 14	+	8.40E-04	*1.79E-03	0.035	0.072	60.15
PL40	Polysaccharide Lyase 40	-	6.64E-04	*1.44E-03	0.039	0.079	63.23
CBM65	Carbohydrate Binding Module 65	-	0.039	0.061	0.039	0.079	*0.00
GH8	Glycoside Hydrolase 8	-	5.93E-03	*0.011	0.043	0.086	45.67
CBM4	Carbohydrate Binding Module 4	-	5.19E-03	*9.88E-03	0.044	0.088	48.06
GH141	Glycoside Hydrolase 141	-	1.64E-06	*6.29E-06	0.046	0.092	82.74
GH165	Glycoside Hydrolase 165	-	0.028	*0.046	0.048	0.094	*19.02
PL29	Polysaccharide Lyase 29	-	8.75E-05	*2.16E-04	0.050	0.096	74.99
CBM41	Carbohydrate Binding Module 41	+	0.012	*0.021	0.050	0.096	*38.84
GH147	Glycoside Hydrolase 147	+	5.35E-03	*0.010	0.052	0.098	51.11
GH156	Glycoside Hydrolase 156	+	5.24E-06	*1.74E-05	0.053	0.099	81.91
PL6	Polysaccharide Lyase 6	+	0.010	*0.018	0.055	0.103	43.90
PL8	Polysaccharide Lyase 8	-	0.056	0.083	0.056	0.104	*0.00
CBM40	Carbohydrate Binding Module 40	+	9.68E-03	*0.017	0.060	0.110	46.98
CBM26	Carbohydrate Binding Module 26	+	4.00E-04	*9.04E-04	0.062	0.113	72.17
GT20	Glycosyltransferase 20	-	0.023	*0.038	0.063	0.113	*32.72
GH98	Glycoside Hydrolase 98	-	0.039	0.061	0.068	0.121	*21.57
GH87	Glycoside Hydrolase 87	+	7.04E-03	*0.013	0.069	0.123	54.52
PL33	Polysaccharide Lyase 33	-	4.37E-03	*8.46E-03	0.070	0.123	59.57
GH27	Glycoside Hydrolase 27	-	0.059	0.087	0.070	0.123	*7.89
GH20	Glycoside Hydrolase 20	-	4.69E-06	*1.60E-05	0.076	0.130	84.94
GH166	Glycoside Hydrolase 166	-	4.64E-04	*1.03E-03	0.075	0.130	74.18
GH163	Glycoside Hydrolase 163	-	1.32E-03	*2.73E-03	0.078	0.133	69.92
GH102	Glycoside Hydrolase 102	+	0.023	*0.038	0.078	0.133	*39.64
GH37	Glycoside Hydrolase 37	+	4.23E-03	*8.27E-03	0.082	0.138	63.00
CBM67	Carbohydrate Binding Module 67	-	0.058	0.085	0.100	0.168	*24.78
PL30	Polysaccharide Lyase 30	-	0.060	0.087	0.104	0.172	*24.97
CBM57	Carbohydrate Binding Module 57	-	0.017	*0.030	0.105	0.173	53.44
GT1	Glycosyltransferase 1	-	6.34E-04	*1.39E-03	0.110	0.179	78.18
CBM74	Carbohydrate Binding Module 74	+	0.110	0.149	0.110	0.179	*0.00
CBM61	Carbohydrate Binding Module 61	-	0.029	*0.047	0.117	0.188	48.30
CBM86	Carbohydrate Binding Module 86	-	0.017	*0.029	0.120	0.192	57.64
CBM71	Carbohydrate Binding Module 71	+	2.18E-03	*4.38E-03	0.139	0.220	76.66
GH129	Glycoside Hydrolase 129	+	0.066	0.095	0.147	0.231	*37.81
GT112	Glycosyltransferase 112	+	0.158	0.209	0.158	0.248	*0.00
GH59	Glycoside Hydrolase 59	+	0.094	0.132	0.162	0.251	*30.28
GH89	Glycoside Hydrolase 89	-	6.90E-04	*1.48E-03	0.164	0.253	83.19
GH33	Glycoside Hydrolase 33	-	1.83E-04	*4.36E-04	0.179	0.274	87.09
GH123	Glycoside Hydrolase 123	-	3.31E-04	*7.56E-04	0.184	0.280	86.31
GH55	Glycoside Hydrolase 55	+	0.153	0.203	0.185	0.280	*13.91
PL21	Polysaccharide Lyase 21	+	0.196	0.252	0.196	0.294	*0.00
GH16	Glycoside Hydrolase 16	-	3.19E-04	*7.43E-04	0.203	0.302	87.48
PL35	Polysaccharide Lyase 35	+	0.204	0.262	0.213	0.315	*3.73
GT4	Glycosyltransferase 4	-	0.023	*0.038	0.221	0.325	71.02
GH81	Glycoside Hydrolase 81	+	0.036	0.057	0.249	0.363	69.81
GT14	Glycosyltransferase 14	-	0.068	0.097	0.260	0.377	61.87
GT100	Glycosyltransferase 100	+	0.183	0.240	0.268	0.386	*30.61
GH151	Glycoside Hydrolase 151	+	0.040	0.062	0.300	0.429	74.55
GH120	Glycoside Hydrolase 120	+	0.044	0.067	0.308	0.429	74.41
GH50	Glycoside Hydrolase 50	+	0.070	0.099	0.306	0.429	67.97

GT111	Glycosyltransferase 111	-	0.102	0.142	0.304	0.429	60.43
GT0	Glycosyltransferase 0	-	0.133	0.178	0.303	0.429	53.09
GT104	Glycosyltransferase 104	-	0.310	0.384	0.310	0.430	*0.00
GT35	Glycosyltransferase 35	-	0.104	0.143	0.319	0.440	62.52
PL12	Polysaccharide Lyase 12	-	0.321	0.393	0.321	0.440	*0.00
GT51	Glycosyltransferase 51	-	0.048	0.073	0.327	0.445	75.35
CBM62	Carbohydrate Binding Module 62	-	0.338	0.412	0.338	0.457	*0.00
GH91	Glycoside Hydrolase 91	+	0.253	0.318	0.343	0.460	*31.18
GH19	Glycoside Hydrolase 19	+	0.108	0.148	0.354	0.473	66.76
CE4	Carbohydrate Esterase 4	-	0.049	0.073	0.358	0.475	78.19
GT6	Glycosyltransferase 6	+	0.039	0.061	0.364	0.480	80.60
CBM3	Carbohydrate Binding Module 3	+	0.103	0.142	0.384	0.504	71.54
GH110	Glycoside Hydrolase 110	-	0.035	0.057	0.393	0.506	83.49
GT28	Glycosyltransferase 28	+	0.053	0.080	0.392	0.506	80.34
GH144	Glycoside Hydrolase 144	-	0.388	0.460	0.388	0.506	*0.00
CBM2	Carbohydrate Binding Module 2	+	0.227	0.289	0.400	0.512	51.53
CE3	Carbohydrate Esterase 3	+	0.138	0.184	0.403	0.513	68.17
GT11	Glycosyltransferase 11	-	0.043	0.066	0.437	0.553	85.22
PL15	Polysaccharide Lyase 15	-	0.371	0.444	0.453	0.571	*29.83
GT101	Glycosyltransferase 101	-	0.184	0.240	0.470	0.588	70.45
GH103	Glycoside Hydrolase 103	+	0.239	0.302	0.482	0.594	64.39
GH15	Glycoside Hydrolase 15	-	0.310	0.384	0.483	0.594	52.34
GH9	Glycoside Hydrolase 9	-	0.479	0.549	0.479	0.594	*0.00
GH0	Glycoside Hydrolase 0	-	0.113	0.153	0.522	0.639	83.64
CBM48	Carbohydrate Binding Module 48	+	0.449	0.523	0.531	0.645	*31.47
PL22	Polysaccharide Lyase 22	-	0.070	0.099	0.556	0.669	89.44
GH158	Glycoside Hydrolase 158	+	0.547	0.621	0.555	0.669	*3.81
GH99	Glycoside Hydrolase 99	-	0.571	0.641	0.571	0.684	*0.00
GH125	Glycoside Hydrolase 125	-	0.472	0.544	0.578	0.688	40.37
GH13	Glycoside Hydrolase 13	-	0.347	0.420	0.592	0.701	67.59
GH48	Glycoside Hydrolase 48	+	0.420	0.492	0.620	0.729	62.01
GH44	Glycoside Hydrolase 44	+	0.191	0.248	0.635	0.732	86.80
GH170	Glycoside Hydrolase 170	+	0.367	0.442	0.634	0.732	72.04
CE15	Carbohydrate Esterase 15	-	0.401	0.472	0.628	0.732	66.81
CBM58	Carbohydrate Binding Module 58	-	0.624	0.673	0.634	0.732	*5.64
GH112	Glycoside Hydrolase 112	-	0.278	0.348	0.647	0.738	82.15
CBM34	Carbohydrate Binding Module 34	-	0.469	0.543	0.644	0.738	59.44
GH128	Glycoside Hydrolase 128	-	0.640	0.687	0.652	0.740	*7.11
CE2	Carbohydrate Esterase 2	+	0.594	0.657	0.707	0.798	50.23
GH153	Glycoside Hydrolase 153	-	0.389	0.460	0.722	0.807	82.91
GH117	Glycoside Hydrolase 117	-	0.722	0.757	0.722	0.807	*0.00
GH76	Glycoside Hydrolase 76	-	0.597	0.657	0.737	0.819	59.70
GT32	Glycosyltransferase 32	-	0.561	0.633	0.746	0.825	69.09
GH1	Glycoside Hydrolase 1	-	0.314	0.387	0.790	0.848	92.98
GH108	Glycoside Hydrolase 108	-	0.578	0.645	0.784	0.848	75.87
AA6	Auxiliary Activity 6	-	0.596	0.657	0.785	0.848	73.40
CBM79	Carbohydrate Binding Module 79	+	0.600	0.657	0.778	0.848	70.97
GH26	Glycoside Hydrolase 26	+	0.688	0.731	0.787	0.848	54.90
PL17	Polysaccharide Lyase 17	-	0.705	0.745	0.775	0.848	42.92
GH5	Glycoside Hydrolase 5	+	0.750	0.782	0.797	0.852	*34.92
PL13	Polysaccharide Lyase 13	-	0.809	0.839	0.809	0.860	*0.00
GH159	Glycoside Hydrolase 159	+	0.609	0.663	0.813	0.860	78.56
GH85	Glycoside Hydrolase 85	+	0.707	0.745	0.831	0.875	67.88
GT56	Glycosyltransferase 56	-	0.686	0.731	0.839	0.879	74.70
GH94	Glycoside Hydrolase 94	+	0.530	0.605	0.845	0.882	90.34
GH4	Glycoside Hydrolase 4	+	0.622	0.673	0.872	0.901	89.35
CBM22	Carbohydrate Binding Module 22	+	0.815	0.841	0.869	0.901	50.44
GH66	Glycoside Hydrolase 66	-	0.893	0.909	0.893	0.918	*0.00
GH18	Glycoside Hydrolase 18	-	0.895	0.909	0.910	0.931	*26.02
GT8	Glycosyltransferase 8	-	0.823	0.847	0.917	0.934	77.97
GH74	Glycoside Hydrolase 74	+	0.898	0.909	0.936	0.949	60.54
GH39	Glycoside Hydrolase 39	+	0.901	0.909	0.947	0.955	70.89
AA3	Auxiliary Activity 3	-	0.973	0.977	0.986	0.986	73.37
GT92	Glycosyltransferase 92	-	0.981	0.981	0.982	0.986	*12.37

False discovery rates (*q* -values) were calculated for the 219 CAZymes using the Benjamini-Hochberg method.

**q* -value (FEM, REM) < 0.05; *I^2 < 40%

Supplementary Table 10. Meta-analysis of summarized CAZymes in six datasets

Cazymes	Cazymes	Increased or decreased in PD	*p* -value (FEM)	*q* -value (FEM)	*p* -value (REM)	*q* -value (REM)	I^2 (%)
AA	Auxiliary Activity	-	0.793	0.793	0.897	0.897	75.55
CBM	Carbohydrate Binding Module	-	1.17E-13	*2.34E-13	6.01E-07	*1.20E-06	54.76
CE	Carbohydrate Esterase	-	6.66E-16	*4.00E-15	1.63E-07	*4.89E-07	58.07
GH	Glycoside Hydrolase	-	3.55E-15	*1.07E-14	1.93E-08	*1.16E-07	49.00
GT	Glycosyltransferase	-	2.09E-06	*2.51E-06	3.44E-03	*4.13E-03	61.98
PL	Polysaccharide Lyase	-	1.98E-09	*2.97E-09	7.55E-04	*1.13E-03	68.47

False discovery rates (*q* -values) were calculated for the 6 categories of CAZymes using the Benjamini-Hochberg method.

**q* -value < 0.05

Supplementary Table 11. Meta-analysis of GSEA of MetaCyc pathways related to arginine, glutamate, proline, and polyamine metabolisms

MetaCyc pathways	Metabolites included in pathway	*p* -value	*q* -value
**Upregulated in PD**			
Urea cycle	Arginine	0.431	1.000
Superpathway of L-citrulline metabolism	Arginine, Glutamate, Proline	0.749	1.000
L-arginine degradation II (AST pathway)	Arginine	0.825	1.000
Superpathway of arginine and polyamine biosynthesis	Polyamine	0.932	1.000
L-Nδ-acetylornithine biosynthesis	Arginine, Glutamate, Proline	1.000	1.000
Superpathway of hyoscyamine (atropine) and scopolamine biosynthesis	Arginine	1.000	1.000
Superpathway of polyamine biosynthesis I	Polyamine	1.000	1.000
**Downregulated in PD**			
Superpathway of polyamine biosynthesis II	Polyamine	7.31E-06	*5.85E-05
Superpathway of polyamine biosynthesis I	Polyamine	2.92E-04	*1.17E-03
Superpathway of arginine and polyamine biosynthesis	Polyamine	6.08E-04	*1.62E-03
L-Nδ-acetylornithine biosynthesis	Arginine, Glutamate, Proline	0.047	0.094
L-arginine degradation II (AST pathway)	Arginine	0.072	0.115
Superpathway of hyoscyamine (atropine) and scopolamine biosynthesis	Arginine	0.764	0.951
Superpathway of L-citrulline metabolism	Arginine, Glutamate, Proline	0.832	0.951
Urea cycle	Arginine	0.997	0.997

False discovery rates (*q* -values) were calculated for the 7 upregulated MetaCyc pathways and the 8 downregulated MetaCyc pathways using the Benjamini-Hochberg method.
**q* -value < 0.05

Supplementary Table 12. Bacteria that contribute to each EC number in six countries.

Country	EC number|Bacteria	Increased or decreased in PD	*p* -value	Fractional CPM (median) Control	PD	PD - Control	Relative CPM (average) Control	PD
Japan	3.5.4.25	-	3.80E-04	105.628	93.515	-12.113	1.000	1.000
	3.5.4.25|g__Faecalibacterium.s__Faecalibacterium_prausnitzii	-	1.46E-02	8.766	4.885	-3.882	0.090	0.070
	3.5.4.25|g__Blautia.s__Ruminococcus_torques	-	9.20E-02	3.673	2.162	-1.510	0.048	0.039
	3.5.4.25|g__Dorea.s__Dorea_longicatena	-	4.31E-02	1.562	0.169	-1.394	0.015	0.011
	3.5.4.25|g__Blautia.s__Blautia_obeum	-	5.06E-04	4.349	3.042	-1.307	0.051	0.034
	3.5.4.25|g__Blautia.s__Blautia_wexlerae	-	1.55E-04	1.639	0.670	-0.968	0.020	0.013
	3.5.4.25|g__Anaerostipes.s__Anaerostipes_hadrus	-	2.76E-01	2.298	1.371	-0.927	0.033	0.029
	3.5.4.25|g__Bacteroides.s__Bacteroides_vulgatus	-	4.93E-01	1.639	1.011	-0.628	0.046	0.033
	3.5.4.25|g__Lachnospiraceae_unclassified.s__Eubacterium_rectale	-	6.63E-02	0.532	0.000	-0.532	0.020	0.013
	3.5.4.25|g__Agathobaculum.s__Agathobaculum_butyriciproducens	-	1.33E-03	0.521	0.000	-0.521	0.005	0.003
	3.5.4.25|g__Bacteroides.s__Bacteroides_uniformis	+	5.69E-01	4.299	3.895	-0.403	0.051	0.064
	3.5.4.25|g__Roseburia.s__Roseburia_hominis	-	5.59E-01	0.935	0.834	-0.101	0.014	0.013
	3.5.4.25|g__Acidaminococcus.s__Acidaminococcus_fermentans	+	3.85E-01	0.000	0.000	0.000	0.000	0.000
	3.5.4.25|g__Acidaminococcus.s__Acidaminococcus_intestini	-	9.64E-01	0.000	0.000	0.000	0.009	0.003
	3.5.4.25|g__Actinomyces.s__Actinomyces_naeslundii	+	3.85E-01	0.000	0.000	0.000	0.000	0.000
	3.5.4.25|g__Actinomyces.s__Actinomyces_oris	+	3.85E-01	0.000	0.000	0.000	0.000	0.000
	3.5.4.25|g__Actinomyces.s__Actinomyces_viscosus	+	3.85E-01	0.000	0.000	0.000	0.000	0.000
	3.5.4.25|g__Adlercreutzia.s__Adlercreutzia_equolifaciens	+	1.48E-01	0.000	0.000	0.000	0.000	0.000
	3.5.4.25|g__Akkermansia.s__Akkermansia_muciniphila	+	2.41E-03	0.000	0.000	0.000	0.005	0.033
	3.5.4.25|g__Alistipes.s__Alistipes_indistinctus	+	4.83E-02	0.000	0.000	0.000	0.000	0.001
	3.5.4.25|g__Alistipes.s__Alistipes_inops	-	8.96E-01	0.000	0.000	0.000	0.001	0.002
	3.5.4.25|g__Alistipes.s__Alistipes_onderdonkii	+	4.02E-03	0.000	0.000	0.000	0.003	0.011
	3.5.4.25|g__Alistipes.s__Alistipes_sp_An31A	-	8.57E-01	0.000	0.000	0.000	0.001	0.001
	3.5.4.25|g__Alistipes.s__Alistipes_sp_An66	+	3.85E-01	0.000	0.000	0.000	0.000	0.000
	3.5.4.25|g__Alistipes.s__Alistipes_timonensis	+	7.21E-01	0.000	0.000	0.000	0.000	0.000
	3.5.4.25|g__Allisonella.s__Allisonella_histaminiformans	+	1.00E+00	0.000	0.000	0.000	0.000	0.000
	3.5.4.25|g__Anaeroglobus.s__Anaeroglobus_geminatus	+	1.00E+00	0.000	0.000	0.000	0.000	0.000
	3.5.4.25|g__Anaeromassilibacillus.s__Anaeromassilibacillus_sp_An250	+	7.31E-01	0.000	0.000	0.000	0.000	0.000
	3.5.4.25|g__Anaerostipes.s__Anaerostipes_caccae	+	5.44E-01	0.000	0.000	0.000	0.001	0.001
	3.5.4.25|g__Anaerotignum.s__Anaerotignum_lactatifermentans	+	7.78E-04	0.000	0.000	0.000	0.000	0.002
	3.5.4.25|g__Asaccharobacter.s__Asaccharobacter_celatus	+	1.98E-01	0.000	0.000	0.000	0.001	0.001
	3.5.4.25|g__Bacillus.s__Bacillus_gibsonii	+	9.85E-03	0.000	0.000	0.000	0.000	0.000
	3.5.4.25|g__Bacillus.s__Bacillus_murimartini	+	3.27E-02	0.000	0.000	0.000	0.000	0.001
	3.5.4.25|g__Bacteroides.s__Bacteroides_caccae	+	2.44E-01	0.000	0.000	0.000	0.004	0.007
	3.5.4.25|g__Bacteroides.s__Bacteroides_clarus	+	1.56E-01	0.000	0.000	0.000	0.001	0.004
	3.5.4.25|g__Bacteroides.s__Bacteroides_coprocola	+	2.02E-01	0.000	0.000	0.000	0.005	0.006
	3.5.4.25|g__Bacteroides.s__Bacteroides_coprophilus	+	4.41E-01	0.000	0.000	0.000	0.001	0.001
	3.5.4.25|g__Bacteroides.s__Bacteroides_eggerthii	+	2.71E-01	0.000	0.000	0.000	0.003	0.010
	3.5.4.25|g__Bacteroides.s__Bacteroides_faecis	-	2.69E-01	0.000	0.000	0.000	0.000	0.000
	3.5.4.25|g__Bacteroides.s__Bacteroides_finegoldii	+	2.44E-01	0.000	0.000	0.000	0.003	0.003
	3.5.4.25|g__Bacteroides.s__Bacteroides_fluxus	-	2.62E-01	0.000	0.000	0.000	0.000	0.000
	3.5.4.25|g__Bacteroides.s__Bacteroides_fragilis	-	7.81E-01	0.000	0.000	0.000	0.005	0.006
	3.5.4.25|g__Bacteroides.s__Bacteroides_intestinalis	-	9.40E-01	0.000	0.000	0.000	0.002	0.002
	3.5.4.25|g__Bacteroides.s__Bacteroides_massiliensis	+	1.88E-01	0.000	0.000	0.000	0.002	0.004
	3.5.4.25|g__Bacteroides.s__Bacteroides_nordii	+	1.10E-02	0.000	0.000	0.000	0.000	0.001
	3.5.4.25|g__Bacteroides.s__Bacteroides_oleiciplenus	+	3.85E-01	0.000	0.000	0.000	0.000	0.000
	3.5.4.25|g__Bacteroides.s__Bacteroides_plebeius	+	2.00E-01	0.000	0.000	0.000	0.020	0.018
	3.5.4.25|g__Bacteroides.s__Bacteroides_salyersiae	-	7.83E-01	0.000	0.000	0.000	0.000	0.000
	3.5.4.25|g__Bacteroides.s__Bacteroides_stercoris	-	6.67E-01	0.000	0.000	0.000	0.030	0.023
	3.5.4.25|g__Barnesiella.s__Barnesiella_intestinihominis	+	3.64E-02	0.000	0.000	0.000	0.003	0.008
	3.5.4.25|g__Barnesiella.s__Barnesiella_sp_An22	-	2.62E-01	0.000	0.000	0.000	0.000	0.000
	3.5.4.25|g__Bifidobacterium.s__Bifidobacterium_longum	-	8.71E-01	0.000	0.000	0.000	0.000	0.000
	3.5.4.25|g__Bilophila.s__Bilophila_wadsworthia	+	2.36E-03	0.000	0.000	0.000	0.001	0.003
	3.5.4.25|g__Blautia.s__Blautia_hansenii	-	2.78E-01	0.000	0.000	0.000	0.000	0.000
	3.5.4.25|g__Blautia.s__Blautia_sp_AF19_10LB	+	7.23E-01	0.000	0.000	0.000	0.002	0.004
	3.5.4.25|g__Blautia.s__Blautia_sp_An249	+	2.14E-01	0.000	0.000	0.000	0.000	0.001
	3.5.4.25|g__Butyricicoccus.s__Butyricicoccus_pullicaecorum	-	1.99E-01	0.000	0.000	0.000	0.000	0.000
	3.5.4.25|g__Butyricimonas.s__Butyricimonas_virosa	+	1.58E-02	0.000	0.000	0.000	0.000	0.001
	3.5.4.25|g__Butyrivibrio.s__Butyrivibrio_crossotus	+	3.85E-01	0.000	0.000	0.000	0.000	0.001
	3.5.4.25|g__Campylobacter.s__Campylobacter_concisus	-	2.62E-01	0.000	0.000	0.000	0.000	0.000
	3.5.4.25|g__Campylobacter.s__Campylobacter_upsaliensis	+	1.00E+00	0.000	0.000	0.000	0.000	0.000
	3.5.4.25|g__Candidatus_Gastranaerophilales_unclassified.s__Candidatus_Ga	-	8.57E-01	0.000	0.000	0.000	0.000	0.000
	3.5.4.25|g__Catenibacterium.s__Catenibacterium_mitsuokai	-	2.02E-03	0.000	0.000	0.000	0.001	0.000
	3.5.4.25|g__Cellulosilyticum.s__Cellulosilyticum_lentocellum	+	3.85E-01	0.000	0.000	0.000	0.000	0.000
	3.5.4.25|g__Cetobacterium.s__Cetobacterium_somerae	-	2.62E-01	0.000	0.000	0.000	0.000	0.000
	3.5.4.25|g__Citrobacter.s__Citrobacter_amalonaticus	+	7.10E-01	0.000	0.000	0.000	0.000	0.001
	3.5.4.25|g__Citrobacter.s__Citrobacter_braakii	+	4.46E-01	0.000	0.000	0.000	0.000	0.000
	3.5.4.25|g__Citrobacter.s__Citrobacter_freundii	+	3.85E-01	0.000	0.000	0.000	0.000	0.001

3.5.4.25|g__Citrobacter.s__Citrobacter_koseri	+	3.85E-01	0.000	0.000	0.000	0.000	0.000
3.5.4.25|g__Citrobacter.s__Citrobacter_portucalensis	+	7.63E-02	0.000	0.000	0.000	0.000	0.001
3.5.4.25|g__Citrobacter.s__Citrobacter_werkmanii	+	3.85E-01	0.000	0.000	0.000	0.000	0.001
3.5.4.25|g__Citrobacter.s__Citrobacter_youngae	+	7.63E-02	0.000	0.000	0.000	0.000	0.000
3.5.4.25|g__Cloacibacillus.s__Cloacibacillus_porcorum	+	1.26E-01	0.000	0.000	0.000	0.000	0.001
3.5.4.25|g__Clostridioides.s__Clostridioides_difficile	+	1.55E-01	0.000	0.000	0.000	0.006	0.006
3.5.4.25|g__Clostridium.s__Butyribacterium_methylotrophicum	+	4.39E-01	0.000	0.000	0.000	0.000	0.000
3.5.4.25|g__Clostridium.s__Clostridium_disporicum	+	8.35E-01	0.000	0.000	0.000	0.001	0.002
3.5.4.25|g__Clostridium.s__Clostridium_perfringens	-	9.89E-02	0.000	0.000	0.000	0.001	0.000
3.5.4.25|g__Clostridium.s__Clostridium_sp_AF36_4	-	7.90E-01	0.000	0.000	0.000	0.001	0.002
3.5.4.25|g__Clostridium.s__Clostridium_sp_AM22_11AC	-	1.06E-01	0.000	0.000	0.000	0.004	0.002
3.5.4.25|g__Coprobacillus.s__Coprobacillus_cateniformis	+	4.14E-02	0.000	0.000	0.000	0.000	0.001
3.5.4.25|g__Coprobacter.s__Coprobacter_fastidiosus	+	1.11E-01	0.000	0.000	0.000	0.001	0.001
3.5.4.25|g__Coprobacter.s__Coprobacter_secundus	+	5.21E-02	0.000	0.000	0.000	0.000	0.001
3.5.4.25|g__Coprococcus.s__Coprococcus_catus	-	6.85E-01	0.000	0.000	0.000	0.002	0.002
3.5.4.25|g__Coprococcus.s__Coprococcus_comes	-	1.38E-02	0.000	0.000	0.000	0.005	0.003
3.5.4.25|g__Coprococcus.s__Coprococcus_eutactus	-	2.89E-01	0.000	0.000	0.000	0.003	0.002
3.5.4.25|g__Desulfovibrio.s__Desulfovibrio_desulfuricans	+	1.78E-02	0.000	0.000	0.000	0.000	0.001
3.5.4.25|g__Desulfovibrio.s__Desulfovibrio_fairfieldensis	+	1.26E-01	0.000	0.000	0.000	0.000	0.000
3.5.4.25|g__Desulfovibrio.s__Desulfovibrio_piger	+	4.74E-02	0.000	0.000	0.000	0.000	0.001
3.5.4.25|g__Desulfovibrio.s__Desulfovibrio_sp_AM18_2	+	3.85E-01	0.000	0.000	0.000	0.000	0.000
3.5.4.25|g__Dialister.s__Dialister_invisus	-	1.64E-01	0.000	0.000	0.000	0.006	0.002
3.5.4.25|g__Dialister.s__Dialister_succinatiphilus	+	7.10E-01	0.000	0.000	0.000	0.000	0.001
3.5.4.25|g__Dorea.s__Dorea_formicigenerans	-	7.64E-01	0.000	0.000	0.000	0.004	0.004
3.5.4.25|g__Dorea.s__Dorea_sp_OM02_2LB	+	7.21E-01	0.000	0.000	0.000	0.000	0.000
3.5.4.25|g__Eggerthella.s__Eggerthella_lenta	+	2.03E-01	0.000	0.000	0.000	0.004	0.004
3.5.4.25|g__Enterobacter.s__Enterobacter_bugandensis	-	8.57E-01	0.000	0.000	0.000	0.000	0.000
3.5.4.25|g__Enterobacter.s__Enterobacter_cloacae	+	4.54E-01	0.000	0.000	0.000	0.000	0.000
3.5.4.25|g__Enterobacter.s__Enterobacter_mori	+	2.14E-01	0.000	0.000	0.000	0.000	0.000
3.5.4.25|g__Enterococcus.s__Enterococcus_avium	+	4.14E-01	0.000	0.000	0.000	0.005	0.001
3.5.4.25|g__Enterococcus.s__Enterococcus_casseliflavus	+	3.85E-01	0.000	0.000	0.000	0.000	0.000
3.5.4.25|g__Enterococcus.s__Enterococcus_faecalis	-	2.62E-01	0.000	0.000	0.000	0.000	0.000
3.5.4.25|g__Enterococcus.s__Enterococcus_faecium	-	9.34E-01	0.000	0.000	0.000	0.005	0.001
3.5.4.25|g__Enterococcus.s__Enterococcus_gallinarum	+	2.14E-01	0.000	0.000	0.000	0.000	0.000
3.5.4.25|g__Enterococcus.s__Enterococcus_saccharolyticus	+	3.85E-01	0.000	0.000	0.000	0.000	0.000
3.5.4.25|g__Enterococcus.s__Enterococcus_thailandicus	+	3.85E-01	0.000	0.000	0.000	0.000	0.000
3.5.4.25|g__Erysipelatoclostridium.s__Clostridium_innocuum	+	7.99E-01	0.000	0.000	0.000	0.002	0.001
3.5.4.25|g__Erysipelatoclostridium.s__Clostridium_spiroforme	+	8.71E-01	0.000	0.000	0.000	0.003	0.001
3.5.4.25|g__Erysipelotrichaceae_unclassified.s__Erysipelotrichaceae_bacteri	+	1.09E-01	0.000	0.000	0.000	0.000	0.001
3.5.4.25|g__Escherichia.s__Escherichia_coli	-	3.57E-01	0.000	0.000	0.000	0.028	0.013
3.5.4.25|g__Eubacterium.s__Eubacterium_callanderi	+	4.39E-01	0.000	0.000	0.000	0.000	0.000
3.5.4.25|g__Eubacterium.s__Eubacterium_limosum	+	1.26E-01	0.000	0.000	0.000	0.000	0.000
3.5.4.25|g__Eubacterium.s__Eubacterium_maltosivorans	+	2.14E-01	0.000	0.000	0.000	0.000	0.000
3.5.4.25|g__Eubacterium.s__Eubacterium_ramulus	-	9.67E-01	0.000	0.000	0.000	0.002	0.003
3.5.4.25|g__Eubacterium.s__Eubacterium_sp_AF17_7	+	2.56E-01	0.000	0.000	0.000	0.002	0.003
3.5.4.25|g__Eubacterium.s__Eubacterium_sp_AM18_10LB_B	-	8.60E-01	0.000	0.000	0.000	0.001	0.001
3.5.4.25|g__Faecalitalea.s__Faecalitalea_cylindroides	+	6.52E-02	0.000	0.000	0.000	0.000	0.001
3.5.4.25|g__Firmicutes_unclassified.s__Firmicutes_bacterium_AM10_47	+	7.26E-01	0.000	0.000	0.000	0.002	0.002
3.5.4.25|g__Flavonifractor.s__Flavonifractor_plautii	+	7.46E-02	0.000	0.000	0.000	0.000	0.000
3.5.4.25|g__Flavonifractor.s__Flavonifractor_sp_An10	+	6.91E-02	0.000	0.000	0.000	0.000	0.002
3.5.4.25|g__Flavonifractor.s__Flavonifractor_sp_An82	+	3.85E-01	0.000	0.000	0.000	0.000	0.000
3.5.4.25|g__Fusobacterium.s__Fusobacterium_mortiferum	-	2.12E-01	0.000	0.000	0.000	0.000	0.000
3.5.4.25|g__Fusobacterium.s__Fusobacterium_ulcerans	+	4.35E-01	0.000	0.000	0.000	0.001	0.000
3.5.4.25|g__Haemophilus.s__Haemophilus_parainfluenzae	-	9.89E-02	0.000	0.000	0.000	0.001	0.000
3.5.4.25|g__Holdemanella.s__Holdemanella_biformis	-	4.86E-01	0.000	0.000	0.000	0.000	0.002
3.5.4.25|g__Hungatella.s__Hungatella_hathewayi	+	5.29E-01	0.000	0.000	0.000	0.001	0.001
3.5.4.25|g__Intestinibacter.s__Intestinibacter_bartlettii	+	9.14E-01	0.000	0.000	0.000	0.002	0.002
3.5.4.25|g__Klebsiella.s__Klebsiella_aerogenes	-	6.45E-01	0.000	0.000	0.000	0.002	0.001
3.5.4.25|g__Klebsiella.s__Klebsiella_grimontii	-	1.09E-01	0.000	0.000	0.000	0.000	0.000
3.5.4.25|g__Klebsiella.s__Klebsiella_michiganensis	-	8.12E-01	0.000	0.000	0.000	0.000	0.000
3.5.4.25|g__Klebsiella.s__Klebsiella_oxytoca	+	6.22E-01	0.000	0.000	0.000	0.003	0.005
3.5.4.25|g__Klebsiella.s__Klebsiella_pneumoniae	-	7.59E-01	0.000	0.000	0.000	0.001	0.004
3.5.4.25|g__Klebsiella.s__Klebsiella_variicola	-	1.33E-01	0.000	0.000	0.000	0.001	0.001
3.5.4.25|g__Lachnoclostridium.s__Clostridium_aldenense	+	5.79E-01	0.000	0.000	0.000	0.000	0.000
3.5.4.25|g__Lachnoclostridium.s__Clostridium_bolteae	+	6.33E-01	0.000	0.000	0.000	0.001	0.001
3.5.4.25|g__Lachnoclostridium.s__Clostridium_citroniae	+	7.21E-01	0.000	0.000	0.000	0.000	0.000
3.5.4.25|g__Lachnoclostridium.s__Clostridium_clostridioforme	-	3.59E-02	0.000	0.000	0.000	0.000	0.000
3.5.4.25|g__Lachnoclostridium.s__Clostridium_symbiosum	-	7.93E-01	0.000	0.000	0.000	0.000	0.000
3.5.4.25|g__Lachnoclostridium.s__Lachnoclostridium_sp_An138	-	8.57E-01	0.000	0.000	0.000	0.000	0.000
3.5.4.25|g__Lachnospira.s__Lachnospira_pectinoschiza	-	2.38E-01	0.000	0.000	0.000	0.003	0.002
3.5.4.25|g__Lachnospiraceae_unclassified.s__Lachnospiraceae_bacterium_(	+	7.31E-01	0.000	0.000	0.000	0.000	0.000
3.5.4.25|g__Lactobacillus.s__Lactobacillus_acidophilus	+	3.85E-01	0.000	0.000	0.000	0.000	0.000

3.5.4.25|g__Lactobacillus.s__Lactobacillus_amylovorus	-	8.57E-01	0.000	0.000	0.000	0.000	0.000
3.5.4.25|g__Lactobacillus.s__Lactobacillus_antri	+	1.26E-01	0.000	0.000	0.000	0.000	0.000
3.5.4.25|g__Lactobacillus.s__Lactobacillus_crispatus	+	6.84E-03	0.000	0.000	0.000	0.000	0.002
3.5.4.25|g__Lactobacillus.s__Lactobacillus_delbrueckii	-	2.62E-01	0.000	0.000	0.000	0.000	0.000
3.5.4.25|g__Lactobacillus.s__Lactobacillus_fermentum	+	9.22E-02	0.000	0.000	0.000	0.000	0.003
3.5.4.25|g__Lactobacillus.s__Lactobacillus_kimbladii	+	3.85E-01	0.000	0.000	0.000	0.000	0.000
3.5.4.25|g__Lactobacillus.s__Lactobacillus_kullabergensis	+	3.85E-01	0.000	0.000	0.000	0.000	0.000
3.5.4.25|g__Lactobacillus.s__Lactobacillus_melliventris	+	3.85E-01	0.000	0.000	0.000	0.000	0.000
3.5.4.25|g__Lactobacillus.s__Lactobacillus_oris	+	3.61E-01	0.000	0.000	0.000	0.001	0.002
3.5.4.25|g__Lactobacillus.s__Lactobacillus_reuteri	+	3.85E-01	0.000	0.000	0.000	0.000	0.000
3.5.4.25|g__Lactobacillus.s__Lactobacillus_rogosae	-	2.60E-01	0.000	0.000	0.000	0.003	0.002
3.5.4.25|g__Lactococcus.s__Lactococcus_lactis	-	4.46E-01	0.000	0.000	0.000	0.001	0.000
3.5.4.25|g__Leclercia.s__Leclercia_adecarboxylata	-	8.03E-01	0.000	0.000	0.000	0.000	0.000
3.5.4.25|g__Lelliottia.s__Lelliottia_nimipressuralis	-	1.99E-01	0.000	0.000	0.000	0.000	0.000
3.5.4.25|g__Leuconostoc.s__Leuconostoc_garlicum	+	3.85E-01	0.000	0.000	0.000	0.000	0.000
3.5.4.25|g__Leuconostoc.s__Leuconostoc_lactis	+	3.85E-01	0.000	0.000	0.000	0.000	0.000
3.5.4.25|g__Megamonas.s__Megamonas_funiformis	-	8.83E-01	0.000	0.000	0.000	0.010	0.006
3.5.4.25|g__Megamonas.s__Megamonas_rupellensis	+	9.30E-01	0.000	0.000	0.000	0.006	0.002
3.5.4.25|g__Megasphaera.s__Megasphaera_elsdenii	-	8.71E-01	0.000	0.000	0.000	0.000	0.000
3.5.4.25|g__Megasphaera.s__Megasphaera_hexanoica	+	3.85E-01	0.000	0.000	0.000	0.000	0.000
3.5.4.25|g__Megasphaera.s__Megasphaera_sp_DISK_18	-	1.29E-01	0.000	0.000	0.000	0.005	0.001
3.5.4.25|g__Megasphaera.s__Megasphaera_sp_MJR8396C	-	7.68E-01	0.000	0.000	0.000	0.001	0.001
3.5.4.25|g__Megasphaera.s__Megasphaera_stantonii	+	4.54E-01	0.000	0.000	0.000	0.000	0.000
3.5.4.25|g__Mitsuokella.s__Mitsuokella_jalaludinii	+	3.85E-01	0.000	0.000	0.000	0.000	0.000
3.5.4.25|g__Mitsuokella.s__Mitsuokella_multacida	+	3.85E-01	0.000	0.000	0.000	0.000	0.000
3.5.4.25|g__Mogibacterium.s__Mogibacterium_diversum	+	6.06E-01	0.000	0.000	0.000	0.000	0.000
3.5.4.25|g__Odoribacter.s__Odoribacter_laneus	+	2.46E-01	0.000	0.000	0.000	0.000	0.001
3.5.4.25|g__Pantoea.s__Pantoea_sesami	-	8.71E-01	0.000	0.000	0.000	0.000	0.000
3.5.4.25|g__Parabacteroides.s__Parabacteroides_goldsteinii	+	1.79E-01	0.000	0.000	0.000	0.000	0.001
3.5.4.25|g__Parabacteroides.s__Parabacteroides_gordonii	+	3.85E-01	0.000	0.000	0.000	0.000	0.000
3.5.4.25|g__Parabacteroides.s__Parabacteroides_johnsonii	+	4.29E-01	0.000	0.000	0.000	0.001	0.001
3.5.4.25|g__Paraprevotella.s__Paraprevotella_clara	+	3.15E-01	0.000	0.000	0.000	0.001	0.002
3.5.4.25|g__Paraprevotella.s__Paraprevotella_xylaniphila	+	1.72E-01	0.000	0.000	0.000	0.000	0.000
3.5.4.25|g__Pediococcus.s__Pediococcus_acidilactici	+	2.92E-01	0.000	0.000	0.000	0.000	0.001
3.5.4.25|g__Peptostreptococcaceae_unclassified.s__Clostridium_hiranonis	-	2.62E-01	0.000	0.000	0.000	0.000	0.000
3.5.4.25|g__Phascolarctobacterium.s__Phascolarctobacterium_faecium	+	1.37E-01	0.000	0.000	0.000	0.004	0.008
3.5.4.25|g__Phascolarctobacterium.s__Phascolarctobacterium_succinatutens	-	2.76E-01	0.000	0.000	0.000	0.005	0.003
3.5.4.25|g__Prevotella.s__Prevotella_buccae	-	2.62E-01	0.000	0.000	0.000	0.001	0.000
3.5.4.25|g__Prevotella.s__Prevotella_copri	-	2.85E-01	0.000	0.000	0.000	0.005	0.002
3.5.4.25|g__Prevotella.s__Prevotella_sp_109	-	2.03E-01	0.000	0.000	0.000	0.003	0.001
3.5.4.25|g__Prevotella.s__Prevotella_sp_885	-	4.59E-02	0.000	0.000	0.000	0.004	0.000
3.5.4.25|g__Prevotella.s__Prevotella_sp_AM42_24	-	1.99E-01	0.000	0.000	0.000	0.003	0.000
3.5.4.25|g__Prevotella.s__Prevotella_stercorea	-	7.68E-01	0.000	0.000	0.000	0.002	0.005
3.5.4.25|g__Pseudoflavonifractor.s__Pseudoflavonifractor_sp_An184	+	4.24E-03	0.000	0.000	0.000	0.000	0.001
3.5.4.25|g__Pseudoramibacter.s__Pseudoramibacter_alactolyticus	-	2.62E-01	0.000	0.000	0.000	0.000	0.000
3.5.4.25|g__Pyramidobacter.s__Pyramidobacter_piscolens	+	2.14E-01	0.000	0.000	0.000	0.000	0.000
3.5.4.25|g__Raoultella.s__Raoultella_ornithinolytica	-	4.41E-01	0.000	0.000	0.000	0.003	0.000
3.5.4.25|g__Rikenella.s__Rikenella_microfusus	+	1.26E-01	0.000	0.000	0.000	0.000	0.000
3.5.4.25|g__Roseburia.s__Roseburia_faecis	-	3.39E-03	0.000	0.000	0.000	0.015	0.005
3.5.4.25|g__Roseburia.s__Roseburia_intestinalis	-	1.39E-03	0.000	0.000	0.000	0.012	0.002
3.5.4.25|g__Roseburia.s__Roseburia_inulinivorans	-	3.46E-01	0.000	0.000	0.000	0.003	0.001
3.5.4.25|g__Rothia.s__Rothia_mucilaginosa	-	8.57E-01	0.000	0.000	0.000	0.000	0.000
3.5.4.25|g__Ruminococcaceae_unclassified.s__Ruminococcaceae_bacteriun	+	4.71E-02	0.000	0.000	0.000	0.000	0.001
3.5.4.25|g__Ruminococcaceae_unclassified.s__Ruminococcaceae_bacteriun	+	8.53E-01	0.000	0.000	0.000	0.000	0.000
3.5.4.25|g__Ruminococcus.s__Ruminococcus_callidus	-	6.12E-02	0.000	0.000	0.000	0.005	0.002
3.5.4.25|g__Ruminococcus.s__Ruminococcus_sp_AF31_8BH	+	9.57E-02	0.000	0.000	0.000	0.001	0.002
3.5.4.25|g__Salmonella.s__Salmonella_enterica	-	2.62E-01	0.000	0.000	0.000	0.000	0.000
3.5.4.25|g__Sanguibacteroides.s__Sanguibacteroides_justesenii	+	3.85E-01	0.000	0.000	0.000	0.000	0.000
3.5.4.25|g__Senegalimassilia.s__Senegalimassilia_anaerobia	+	2.16E-02	0.000	0.000	0.000	0.000	0.002
3.5.4.25|g__Slackia.s__Slackia_isoflavoniconvertens	+	7.23E-01	0.000	0.000	0.000	0.001	0.002
3.5.4.25|g__Staphylococcus.s__Staphylococcus_aureus	+	2.14E-01	0.000	0.000	0.000	0.000	0.000
3.5.4.25|g__Streptococcus.s__Streptococcus_equinus	-	1.09E-01	0.000	0.000	0.000	0.002	0.000
3.5.4.25|g__Streptococcus.s__Streptococcus_gallolyticus	+	4.46E-01	0.000	0.000	0.000	0.000	0.000
3.5.4.25|g__Streptococcus.s__Streptococcus_infantarius	-	1.09E-01	0.000	0.000	0.000	0.002	0.000
3.5.4.25|g__Streptococcus.s__Streptococcus_lutetiensis	-	1.09E-01	0.000	0.000	0.000	0.003	0.000
3.5.4.25|g__Streptococcus.s__Streptococcus_macedonicus	-	4.86E-01	0.000	0.000	0.000	0.001	0.001
3.5.4.25|g__Streptococcus.s__Streptococcus_pasteurianus	-	4.92E-01	0.000	0.000	0.000	0.001	0.001
3.5.4.25|g__Streptococcus.s__Streptococcus_pneumoniae	+	7.60E-03	0.000	0.000	0.000	0.000	0.000
3.5.4.25|g__Succinatimonas.s__Succinatimonas_hippei	-	8.71E-01	0.000	0.000	0.000	0.000	0.000
3.5.4.25|g__Sutterella.s__Sutterella_wadsworthensis	-	9.52E-01	0.000	0.000	0.000	0.002	0.002
3.5.4.25|g__Terrisporobacter.s__Terrisporobacter_othiniensis	+	2.14E-01	0.000	0.000	0.000	0.000	0.000
3.5.4.25|g__Veillonella.s__Veillonella_atypica	-	6.53E-04	0.000	0.000	0.000	0.002	0.000

	3.5.4.25|g__Veillonella.s__Veillonella_denticariosi	+	7.10E-01	0.000	0.000	0.000	0.000	0.000
	3.5.4.25|g__Veillonella.s__Veillonella_dispar	-	7.26E-04	0.000	0.000	0.000	0.003	0.000
	3.5.4.25|g__Veillonella.s__Veillonella_infantium	-	1.04E-02	0.000	0.000	0.000	0.000	0.000
	3.5.4.25|g__Veillonella.s__Veillonella_parvula	-	4.55E-03	0.000	0.000	0.000	0.000	0.000
	3.5.4.25|g__Veillonella.s__Veillonella_rogosae	-	1.06E-04	0.000	0.000	0.000	0.000	0.000
	3.5.4.25|g__Veillonella.s__Veillonella_tobetsuensis	-	1.09E-01	0.000	0.000	0.000	0.000	0.000
	3.5.4.25|g__Victivallales_unclassified.s__Victivallales_bacterium_CCUG_4	-	8.57E-01	0.000	0.000	0.000	0.000	0.000
	3.5.4.25|g__Victivallis.s__Victivallis_vadensis	-	2.62E-01	0.000	0.000	0.000	0.000	0.000
	3.5.4.25|g__Weissella.s__Weissella_cibaria	+	7.63E-02	0.000	0.000	0.000	0.000	0.000
	3.5.4.25|g__Weissella.s__Weissella_confusa	+	3.85E-01	0.000	0.000	0.000	0.000	0.000
	3.5.4.25|g__Bacteroides.s__Bacteroides_ovatus	+	2.59E-01	0.276	0.315	0.039	0.011	0.015
	3.5.4.25|g__Bacteroides.s__Bacteroides_thetaiotaomicron	+	2.60E-01	0.430	0.489	0.059	0.007	0.012
	3.5.4.25|g__Bacteroides.s__Bacteroides_xylanisolvens	+	7.09E-01	0.075	0.177	0.102	0.007	0.008
	3.5.4.25|g__Eubacterium.s__Eubacterium_eligens	+	1.53E-01	0.000	0.108	0.108	0.010	0.018
	3.5.4.25|g__Bacteroides.s__Bacteroides_cellulosilyticus	+	1.32E-02	0.000	0.117	0.117	0.005	0.009
	3.5.4.25|g__Alistipes.s__Alistipes_finegoldii	+	4.22E-03	0.000	0.178	0.178	0.005	0.015
	3.5.4.25|g__Odoribacter.s__Odoribacter_splanchnicus	+	6.10E-04	0.000	0.248	0.248	0.001	0.003
	3.5.4.25|g__Parabacteroides.s__Parabacteroides_merdae	+	2.96E-01	0.399	0.731	0.332	0.009	0.011
	3.5.4.25|g__Parabacteroides.s__Parabacteroides_distasonis	+	3.12E-02	0.576	0.949	0.373	0.012	0.016
	3.5.4.25|g__Bacteroides.s__Bacteroides_dorei	+	7.48E-01	0.394	0.871	0.477	0.032	0.030
	3.5.4.25|g__Alistipes.s__Alistipes_putredinis	+	1.68E-02	0.000	1.585	1.585	0.011	0.022
	3.5.4.25|unclassified	+	4.53E-01	13.552	15.774	2.222	0.171	0.198
USA	3.5.4.25	-	6.48E-05	112.288	104.056	-8.232	1.000	1.000
	3.5.4.25|g__Faecalibacterium.s__Faecalibacterium_prausnitzii	-	1.35E-05	5.807	2.185	-3.621	0.060	0.045
	3.5.4.25|g__Blautia.s__Blautia_obeum	-	2.72E-09	5.628	3.749	-1.879	0.062	0.047
	3.5.4.25|g__Bacteroides.s__Bacteroides_vulgatus	-	5.36E-02	5.686	4.210	-1.476	0.066	0.060
	3.5.4.25|g__Blautia.s__Ruminococcus_torques	-	1.80E-04	5.204	3.869	-1.335	0.060	0.049
	3.5.4.25|g__Bacteroides.s__Bacteroides_uniformis	-	6.75E-01	5.938	4.791	-1.147	0.068	0.071
	3.5.4.25|g__Lachnospiraceae_unclassified.s__Eubacterium_rectale	-	6.46E-04	0.944	0.202	-0.741	0.019	0.015
	3.5.4.25|g__Dorea.s__Dorea_longicatena	-	3.17E-02	0.566	0.054	-0.512	0.013	0.010
	3.5.4.25|g__Anaerostipes.s__Anaerostipes_hadrus	-	1.46E-04	0.891	0.383	-0.508	0.014	0.012
	3.5.4.25|g__Blautia.s__Blautia_wexlerae	-	3.02E-08	1.162	0.694	-0.468	0.014	0.011
	3.5.4.25|g__Alistipes.s__Alistipes_putredinis	-	6.86E-01	2.697	2.314	-0.382	0.028	0.028
	3.5.4.25|g__Roseburia.s__Roseburia_intestinalis	-	2.33E-06	0.242	0.000	-0.242	0.009	0.005
	3.5.4.25|g__Roseburia.s__Roseburia_faecis	-	1.37E-03	0.215	0.000	-0.215	0.018	0.012
	3.5.4.25|g__Agathobaculum.s__Agathobaculum_butyriciproducens	-	1.27E-03	0.180	0.000	-0.180	0.004	0.002
	3.5.4.25|g__Asaccharobacter.s__Asaccharobacter_celatus	-	9.52E-01	0.163	0.128	-0.035	0.004	0.004
	3.5.4.25|g__Lachnoclostridium.s__Clostridium_bolteae	-	9.58E-01	0.027	0.000	-0.027	0.002	0.003
	3.5.4.25|g__Bacteroides.s__Bacteroides_ovatus	+	5.80E-01	0.505	0.481	-0.024	0.017	0.016
	3.5.4.25|g__Hungatella.s__Hungatella_hathewayi	-	4.08E-01	0.017	0.000	-0.017	0.004	0.002
	3.5.4.25|g__Acidaminococcus.s__Acidaminococcus_fermentans	+	2.32E-01	0.000	0.000	0.000	0.000	0.000
	3.5.4.25|g__Acidaminococcus.s__Acidaminococcus_intestini	+	1.95E-03	0.000	0.000	0.000	0.006	0.010
	3.5.4.25|g__Acidipropionibacterium.s__Acidipropionibacterium_acidipropic	+	4.92E-01	0.000	0.000	0.000	0.000	0.000
	3.5.4.25|g__Actinomyces.s__Actinomyces_johnsonii	-	9.73E-01	0.000	0.000	0.000	0.000	0.000
	3.5.4.25|g__Actinomyces.s__Actinomyces_naeslundii	+	3.07E-02	0.000	0.000	0.000	0.000	0.000
	3.5.4.25|g__Actinomyces.s__Actinomyces_oris	+	1.48E-02	0.000	0.000	0.000	0.000	0.000
	3.5.4.25|g__Actinomyces.s__Actinomyces_sp_oral_taxon_448	+	7.60E-01	0.000	0.000	0.000	0.000	0.000
	3.5.4.25|g__Actinomyces.s__Actinomyces_viscosus	+	7.40E-03	0.000	0.000	0.000	0.000	0.000
	3.5.4.25|g__Adlercreutzia.s__Adlercreutzia_equolifaciens	-	9.35E-01	0.000	0.000	0.000	0.001	0.001
	3.5.4.25|g__Aeromonas.s__Aeromonas_caviae	+	4.92E-01	0.000	0.000	0.000	0.000	0.000
	3.5.4.25|g__Aeromonas.s__Aeromonas_diversa	-	1.48E-01	0.000	0.000	0.000	0.000	0.000
	3.5.4.25|g__Aeromonas.s__Aeromonas_veronii	-	1.48E-01	0.000	0.000	0.000	0.000	0.000
	3.5.4.25|g__Aggregatibacter.s__Aggregatibacter_segnis	+	4.92E-01	0.000	0.000	0.000	0.000	0.000
	3.5.4.25|g__Akkermansia.s__Akkermansia_muciniphila	+	3.05E-01	0.000	0.000	0.000	0.023	0.040
	3.5.4.25|g__Alistipes.s__Alistipes_indistinctus	+	7.40E-04	0.000	0.000	0.000	0.001	0.002
	3.5.4.25|g__Alistipes.s__Alistipes_inops	+	1.65E-01	0.000	0.000	0.000	0.001	0.001
	3.5.4.25|g__Alistipes.s__Alistipes_sp_An66	-	1.48E-01	0.000	0.000	0.000	0.000	0.000
	3.5.4.25|g__Alistipes.s__Alistipes_timonensis	+	3.03E-01	0.000	0.000	0.000	0.001	0.001
	3.5.4.25|g__Allisonella.s__Allisonella_histaminiformans	+	7.64E-01	0.000	0.000	0.000	0.000	0.000
	3.5.4.25|g__Anaerococcus.s__Anaerococcus_hydrogenalis	+	4.92E-01	0.000	0.000	0.000	0.000	0.000
	3.5.4.25|g__Anaerococcus.s__Anaerococcus_octavius	+	4.92E-01	0.000	0.000	0.000	0.000	0.000
	3.5.4.25|g__Anaerococcus.s__Anaerococcus_vaginalis	+	3.30E-01	0.000	0.000	0.000	0.000	0.000
	3.5.4.25|g__Anaeroglobus.s__Anaeroglobus_geminatus	-	9.53E-01	0.000	0.000	0.000	0.000	0.000
	3.5.4.25|g__Anaeromassilibacillus.s__Anaeromassilibacillus_sp_An250	-	9.39E-01	0.000	0.000	0.000	0.000	0.000
	3.5.4.25|g__Anaerostipes.s__Anaerostipes_caccae	+	2.41E-01	0.000	0.000	0.000	0.001	0.001
	3.5.4.25|g__Anaerostipes.s__Anaerostipes_sp_494a	-	1.48E-01	0.000	0.000	0.000	0.000	0.000
	3.5.4.25|g__Anaerotignum.s__Anaerotignum_lactatifermentans	-	2.00E-01	0.000	0.000	0.000	0.002	0.002
	3.5.4.25|g__Bacillus.s__Bacillus_horneckiae	-	1.48E-01	0.000	0.000	0.000	0.000	0.000
	3.5.4.25|g__Bacteroides.s__Bacteroides_clarus	+	7.72E-01	0.000	0.000	0.000	0.001	0.001
	3.5.4.25|g__Bacteroides.s__Bacteroides_coprocola	-	3.34E-02	0.000	0.000	0.000	0.002	0.001
	3.5.4.25|g__Bacteroides.s__Bacteroides_coprophilus	+	4.06E-01	0.000	0.000	0.000	0.000	0.000
	3.5.4.25|g__Bacteroides.s__Bacteroides_eggerthii	+	7.08E-01	0.000	0.000	0.000	0.012	0.010

3.5.4.25|g__Bacteroides.s__Bacteroides_faecichinchillae	+	1.22E-01	0.000	0.000	0.000	0.000	0.000
3.5.4.25|g__Bacteroides.s__Bacteroides_faecis	+	5.33E-02	0.000	0.000	0.000	0.002	0.002
3.5.4.25|g__Bacteroides.s__Bacteroides_finegoldii	+	6.70E-01	0.000	0.000	0.000	0.002	0.002
3.5.4.25|g__Bacteroides.s__Bacteroides_fluxus	+	2.32E-01	0.000	0.000	0.000	0.000	0.000
3.5.4.25|g__Bacteroides.s__Bacteroides_fragilis	-	4.97E-01	0.000	0.000	0.000	0.006	0.006
3.5.4.25|g__Bacteroides.s__Bacteroides_intestinalis	+	1.74E-01	0.000	0.000	0.000	0.003	0.006
3.5.4.25|g__Bacteroides.s__Bacteroides_massiliensis	+	4.13E-01	0.000	0.000	0.000	0.010	0.008
3.5.4.25|g__Bacteroides.s__Bacteroides_nordii	-	1.65E-01	0.000	0.000	0.000	0.001	0.001
3.5.4.25|g__Bacteroides.s__Bacteroides_oleiciplenus	+	4.92E-01	0.000	0.000	0.000	0.000	0.000
3.5.4.25|g__Bacteroides.s__Bacteroides_plebeius	-	3.80E-01	0.000	0.000	0.000	0.012	0.004
3.5.4.25|g__Bacteroides.s__Bacteroides_salyersiae	+	4.24E-02	0.000	0.000	0.000	0.002	0.002
3.5.4.25|g__Bacteroides.s__Bacteroides_sartorii	-	4.06E-02	0.000	0.000	0.000	0.000	0.000
3.5.4.25|g__Bacteroides.s__Bacteroides_sp_OM08_11	+	4.92E-01	0.000	0.000	0.000	0.000	0.000
3.5.4.25|g__Bacteroides.s__Bacteroides_stercorirosoris	-	9.57E-01	0.000	0.000	0.000	0.000	0.000
3.5.4.25|g__Barnesiella.s__Barnesiella_intestinihominis	+	1.29E-01	0.000	0.000	0.000	0.005	0.006
3.5.4.25|g__Bifidobacterium.s__Bifidobacterium_longum	+	2.32E-01	0.000	0.000	0.000	0.000	0.000
3.5.4.25|g__Blautia.s__Blautia_hansenii	-	1.57E-01	0.000	0.000	0.000	0.002	0.001
3.5.4.25|g__Blautia.s__Blautia_sp_AF19_10LB	+	5.37E-01	0.000	0.000	0.000	0.001	0.001
3.5.4.25|g__Blautia.s__Blautia_sp_An249	-	1.12E-01	0.000	0.000	0.000	0.000	0.000
3.5.4.25|g__Brevibacterium.s__Brevibacterium_ravenspurgense	+	4.92E-01	0.000	0.000	0.000	0.000	0.000
3.5.4.25|g__Brochothrix.s__Brochothrix_thermosphacta	+	4.92E-01	0.000	0.000	0.000	0.000	0.000
3.5.4.25|g__Butyricicoccus.s__Butyricicoccus_pullicaecorum	-	1.39E-01	0.000	0.000	0.000	0.000	0.000
3.5.4.25|g__Butyricimonas.s__Butyricimonas_synergistica	-	1.48E-01	0.000	0.000	0.000	0.000	0.000
3.5.4.25|g__Butyricimonas.s__Butyricimonas_virosa	+	2.29E-02	0.000	0.000	0.000	0.000	0.001
3.5.4.25|g__Butyrivibrio.s__Butyrivibrio_crossotus	-	4.75E-01	0.000	0.000	0.000	0.002	0.001
3.5.4.25|g__Campylobacter.s__Campylobacter_curvus	+	3.30E-01	0.000	0.000	0.000	0.000	0.000
3.5.4.25|g__Campylobacter.s__Campylobacter_gracilis	-	6.01E-01	0.000	0.000	0.000	0.000	0.000
3.5.4.25|g__Campylobacter.s__Campylobacter_ureolyticus	+	4.92E-01	0.000	0.000	0.000	0.000	0.000
3.5.4.25|g__Candidatus_Gastranaerophilales_unclassified.s__Candidatus_Ga	+	2.81E-02	0.000	0.000	0.000	0.000	0.000
3.5.4.25|g__Catenibacterium.s__Catenibacterium_mitsuokai	+	4.91E-01	0.000	0.000	0.000	0.001	0.002
3.5.4.25|g__Cellulosilyticum.s__Cellulosilyticum_lentocellum	+	4.92E-01	0.000	0.000	0.000	0.000	0.000
3.5.4.25|g__Cetobacterium.s__Cetobacterium_somerae	+	4.92E-01	0.000	0.000	0.000	0.000	0.000
3.5.4.25|g__Citrobacter.s__Citrobacter_amalonaticus	+	8.65E-01	0.000	0.000	0.000	0.000	0.000
3.5.4.25|g__Citrobacter.s__Citrobacter_braakii	+	8.41E-01	0.000	0.000	0.000	0.000	0.000
3.5.4.25|g__Citrobacter.s__Citrobacter_farmeri	+	3.30E-01	0.000	0.000	0.000	0.000	0.000
3.5.4.25|g__Citrobacter.s__Citrobacter_freundii	-	5.92E-01	0.000	0.000	0.000	0.000	0.000
3.5.4.25|g__Citrobacter.s__Citrobacter_koseri	+	4.92E-01	0.000	0.000	0.000	0.000	0.000
3.5.4.25|g__Citrobacter.s__Citrobacter_portucalensis	+	6.32E-01	0.000	0.000	0.000	0.001	0.000
3.5.4.25|g__Citrobacter.s__Citrobacter_werkmanii	+	3.30E-01	0.000	0.000	0.000	0.000	0.000
3.5.4.25|g__Citrobacter.s__Citrobacter_youngae	+	1.52E-01	0.000	0.000	0.000	0.000	0.000
3.5.4.25|g__Cloacibacillus.s__Cloacibacillus_porcorum	+	7.50E-02	0.000	0.000	0.000	0.000	0.001
3.5.4.25|g__Clostridiales_Family_XIII_Incertae_Sedis_unclassified.s__Clost	+	4.92E-01	0.000	0.000	0.000	0.000	0.000
3.5.4.25|g__Clostridiales_Family_XIII_Incertae_Sedis_unclassified.s__Euba	+	4.92E-01	0.000	0.000	0.000	0.000	0.000
3.5.4.25|g__Clostridiales_Family_XIII_Incertae_Sedis_unclassified.s__Euba	+	4.92E-01	0.000	0.000	0.000	0.000	0.000
3.5.4.25|g__Clostridiales_unclassified.s__Clostridiales_bacterium_1_7_47FA	+	7.71E-01	0.000	0.000	0.000	0.000	0.000
3.5.4.25|g__Clostridioides.s__Clostridioides_difficile	+	2.26E-03	0.000	0.000	0.000	0.003	0.004
3.5.4.25|g__Clostridium.s__Butyribacterium_methylotrophicum	+	8.86E-04	0.000	0.000	0.000	0.000	0.001
3.5.4.25|g__Clostridium.s__Clostridium_butyricum	-	1.48E-01	0.000	0.000	0.000	0.000	0.000
3.5.4.25|g__Clostridium.s__Clostridium_cadaveris	-	5.95E-01	0.000	0.000	0.000	0.000	0.000
3.5.4.25|g__Clostridium.s__Clostridium_celatum	-	1.48E-01	0.000	0.000	0.000	0.000	0.000
3.5.4.25|g__Clostridium.s__Clostridium_disporicum	+	5.05E-01	0.000	0.000	0.000	0.000	0.000
3.5.4.25|g__Clostridium.s__Clostridium_perfringens	-	5.48E-01	0.000	0.000	0.000	0.000	0.000
3.5.4.25|g__Clostridium.s__Clostridium_sp_AF36_4	-	2.50E-01	0.000	0.000	0.000	0.002	0.002
3.5.4.25|g__Clostridium.s__Clostridium_sp_AM22_11AC	-	2.31E-03	0.000	0.000	0.000	0.002	0.001
3.5.4.25|g__Clostridium.s__Clostridium_sp_MSTE9	+	4.92E-01	0.000	0.000	0.000	0.000	0.000
3.5.4.25|g__Clostridium.s__Clostridium_sp_chh4_2	-	5.92E-01	0.000	0.000	0.000	0.000	0.000
3.5.4.25|g__Clostridium.s__Clostridium_ventriculi	-	5.92E-01	0.000	0.000	0.000	0.000	0.000
3.5.4.25|g__Coprobacillus.s__Coprobacillus_cateniformis	+	2.65E-04	0.000	0.000	0.000	0.000	0.001
3.5.4.25|g__Coprobacter.s__Coprobacter_fastidiosus	-	1.98E-02	0.000	0.000	0.000	0.001	0.000
3.5.4.25|g__Coprobacter.s__Coprobacter_secundus	-	7.75E-01	0.000	0.000	0.000	0.000	0.000
3.5.4.25|g__Coprococcus.s__Coprococcus_catus	+	2.87E-01	0.000	0.000	0.000	0.001	0.001
3.5.4.25|g__Coprococcus.s__Coprococcus_comes	-	8.37E-01	0.000	0.000	0.000	0.005	0.005
3.5.4.25|g__Coprococcus.s__Coprococcus_eutactus	-	4.67E-01	0.000	0.000	0.000	0.003	0.002
3.5.4.25|g__Corynebacterium.s__Corynebacterium_amycolatum	+	4.92E-01	0.000	0.000	0.000	0.000	0.000
3.5.4.25|g__Corynebacterium.s__Corynebacterium_coyleae	+	4.92E-01	0.000	0.000	0.000	0.000	0.000
3.5.4.25|g__Corynebacterium.s__Corynebacterium_frankenforstense	-	1.48E-01	0.000	0.000	0.000	0.000	0.000
3.5.4.25|g__Corynebacterium.s__Corynebacterium_imitans	+	4.92E-01	0.000	0.000	0.000	0.000	0.000
3.5.4.25|g__Corynebacterium.s__Corynebacterium_jeikeium	+	4.92E-01	0.000	0.000	0.000	0.000	0.000
3.5.4.25|g__Corynebacterium.s__Corynebacterium_kroppenstedtii	+	4.92E-01	0.000	0.000	0.000	0.000	0.000
3.5.4.25|g__Corynebacterium.s__Corynebacterium_pseudogenitalium	+	4.92E-01	0.000	0.000	0.000	0.000	0.000
3.5.4.25|g__Corynebacterium.s__Corynebacterium_riegelii	+	4.92E-01	0.000	0.000	0.000	0.000	0.000
3.5.4.25|g__Corynebacterium.s__Corynebacterium_tuberculostearicum	+	4.92E-01	0.000	0.000	0.000	0.000	0.000

3.5.4.25|g__Corynebacterium.s__Corynebacterium_urealyticum	+	4.92E-01	0.000	0.000	0.000	0.000	0.000
3.5.4.25|g__Cronobacter.s__Cronobacter_sakazakii	+	4.92E-01	0.000	0.000	0.000	0.000	0.000
3.5.4.25|g__Desulfovibrio.s__Desulfovibrio_desulfuricans	+	1.67E-01	0.000	0.000	0.000	0.000	0.000
3.5.4.25|g__Desulfovibrio.s__Desulfovibrio_fairfieldensis	+	1.53E-01	0.000	0.000	0.000	0.001	0.002
3.5.4.25|g__Desulfovibrio.s__Desulfovibrio_legallii	+	4.92E-01	0.000	0.000	0.000	0.000	0.000
3.5.4.25|g__Desulfovibrio.s__Desulfovibrio_piger	+	1.27E-01	0.000	0.000	0.000	0.000	0.000
3.5.4.25|g__Desulfovibrio.s__Desulfovibrio_sp_AM18_2	+	2.30E-01	0.000	0.000	0.000	0.000	0.000
3.5.4.25|g__Desulfovibrionaceae_unclassified.s__Desulfovibrionaceae_bact	+	2.41E-01	0.000	0.000	0.000	0.000	0.000
3.5.4.25|g__Dialister.s__Dialister_invisus	-	8.16E-03	0.000	0.000	0.000	0.005	0.003
3.5.4.25|g__Dialister.s__Dialister_pneumosintes	+	4.92E-01	0.000	0.000	0.000	0.000	0.000
3.5.4.25|g__Dialister.s__Dialister_succinatiphilus	+	4.92E-01	0.000	0.000	0.000	0.000	0.000
3.5.4.25|g__Dorea.s__Dorea_formicigenerans	+	9.08E-01	0.000	0.000	0.000	0.002	0.002
3.5.4.25|g__Dorea.s__Dorea_sp_OM02_2LB	+	6.36E-01	0.000	0.000	0.000	0.000	0.000
3.5.4.25|g__Dysgonomonas.s__Dysgonomonas_mossii	+	4.92E-01	0.000	0.000	0.000	0.000	0.000
3.5.4.25|g__Enterobacter.s__Enterobacter_bugandensis	+	3.30E-01	0.000	0.000	0.000	0.000	0.000
3.5.4.25|g__Enterobacter.s__Enterobacter_cloacae	+	4.29E-02	0.000	0.000	0.000	0.000	0.000
3.5.4.25|g__Enterobacter.s__Enterobacter_mori	+	4.92E-01	0.000	0.000	0.000	0.000	0.000
3.5.4.25|g__Enterococcus.s__Enterococcus_avium	+	2.44E-05	0.000	0.000	0.000	0.000	0.001
3.5.4.25|g__Enterococcus.s__Enterococcus_casseliflavus	+	4.12E-01	0.000	0.000	0.000	0.000	0.000
3.5.4.25|g__Enterococcus.s__Enterococcus_faecalis	+	3.30E-01	0.000	0.000	0.000	0.000	0.000
3.5.4.25|g__Enterococcus.s__Enterococcus_faecium	+	2.11E-01	0.000	0.000	0.000	0.000	0.000
3.5.4.25|g__Enterococcus.s__Enterococcus_gallinarum	+	3.74E-02	0.000	0.000	0.000	0.000	0.000
3.5.4.25|g__Enterococcus.s__Enterococcus_hirae	+	4.92E-01	0.000	0.000	0.000	0.000	0.000
3.5.4.25|g__Enterococcus.s__Enterococcus_mundtii	+	4.92E-01	0.000	0.000	0.000	0.000	0.000
3.5.4.25|g__Enterococcus.s__Enterococcus_saccharolyticus	+	6.68E-02	0.000	0.000	0.000	0.000	0.000
3.5.4.25|g__Enterococcus.s__Enterococcus_thailandicus	+	4.92E-01	0.000	0.000	0.000	0.000	0.000
3.5.4.25|g__Erysipelatoclostridium.s__Clostridium_innocuum	+	3.29E-01	0.000	0.000	0.000	0.003	0.001
3.5.4.25|g__Erysipelatoclostridium.s__Clostridium_spiroforme	+	3.75E-01	0.000	0.000	0.000	0.001	0.000
3.5.4.25|g__Erysipelotrichaceae_unclassified.s__Erysipelotrichaceae_bacteri	+	5.29E-01	0.000	0.000	0.000	0.001	0.000
3.5.4.25|g__Escherichia.s__Escherichia_coli	+	1.91E-02	0.000	0.000	0.000	0.015	0.018
3.5.4.25|g__Escherichia.s__Escherichia_fergusonii	+	4.92E-01	0.000	0.000	0.000	0.000	0.000
3.5.4.25|g__Escherichia.s__Escherichia_marmotae	+	4.92E-01	0.000	0.000	0.000	0.000	0.000
3.5.4.25|g__Eubacterium.s__Eubacterium_callanderi	+	7.64E-04	0.000	0.000	0.000	0.000	0.001
3.5.4.25|g__Eubacterium.s__Eubacterium_eligens	-	4.07E-02	0.000	0.000	0.000	0.006	0.005
3.5.4.25|g__Eubacterium.s__Eubacterium_limosum	+	1.18E-01	0.000	0.000	0.000	0.000	0.000
3.5.4.25|g__Eubacterium.s__Eubacterium_maltosivorans	+	2.48E-01	0.000	0.000	0.000	0.000	0.000
3.5.4.25|g__Eubacterium.s__Eubacterium_ramulus	-	4.53E-02	0.000	0.000	0.000	0.003	0.002
3.5.4.25|g__Eubacterium.s__Eubacterium_sp_AF17_7	-	9.79E-01	0.000	0.000	0.000	0.000	0.000
3.5.4.25|g__Eubacterium.s__Eubacterium_sp_AM18_10LB_B	-	2.95E-01	0.000	0.000	0.000	0.000	0.000
3.5.4.25|g__Eubacterium.s__Eubacterium_sp_An11	+	6.61E-01	0.000	0.000	0.000	0.000	0.000
3.5.4.25|g__Faecalitalea.s__Faecalitalea_cylindroides	+	1.71E-01	0.000	0.000	0.000	0.000	0.000
3.5.4.25|g__Firmicutes_unclassified.s__Firmicutes_bacterium_AM10_47	+	9.91E-01	0.000	0.000	0.000	0.000	0.000
3.5.4.25|g__Flavonifractor.s__Flavonifractor_plautii	+	4.80E-02	0.000	0.000	0.000	0.001	0.001
3.5.4.25|g__Flavonifractor.s__Flavonifractor_sp_An10	+	1.08E-01	0.000	0.000	0.000	0.000	0.001
3.5.4.25|g__Fusobacterium.s__Fusobacterium_mortiferum	-	9.57E-01	0.000	0.000	0.000	0.000	0.000
3.5.4.25|g__Fusobacterium.s__Fusobacterium_nucleatum	-	5.92E-01	0.000	0.000	0.000	0.000	0.000
3.5.4.25|g__Fusobacterium.s__Fusobacterium_ulcerans	-	4.27E-01	0.000	0.000	0.000	0.000	0.000
3.5.4.25|g__Fusobacterium.s__Fusobacterium_varium	+	4.92E-01	0.000	0.000	0.000	0.000	0.000
3.5.4.25|g__Gemella.s__Gemella_haemolysans	+	4.92E-01	0.000	0.000	0.000	0.000	0.000
3.5.4.25|g__Haemophilus.s__Haemophilus_parainfluenzae	+	6.78E-01	0.000	0.000	0.000	0.000	0.000
3.5.4.25|g__Hafnia.s__Hafnia_paralvei	-	1.48E-01	0.000	0.000	0.000	0.000	0.000
3.5.4.25|g__Holdemanella.s__Holdemanella_biformis	-	4.47E-01	0.000	0.000	0.000	0.000	0.000
3.5.4.25|g__Intestinibacter.s__Intestinibacter_bartlettii	+	9.77E-01	0.000	0.000	0.000	0.001	0.001
3.5.4.25|g__Klebsiella.s__Klebsiella_aerogenes	+	5.71E-02	0.000	0.000	0.000	0.001	0.001
3.5.4.25|g__Klebsiella.s__Klebsiella_grimontii	+	3.30E-01	0.000	0.000	0.000	0.000	0.000
3.5.4.25|g__Klebsiella.s__Klebsiella_michiganensis	+	6.67E-01	0.000	0.000	0.000	0.000	0.000
3.5.4.25|g__Klebsiella.s__Klebsiella_oxytoca	+	5.25E-03	0.000	0.000	0.000	0.003	0.005
3.5.4.25|g__Klebsiella.s__Klebsiella_pneumoniae	+	5.26E-03	0.000	0.000	0.000	0.003	0.003
3.5.4.25|g__Klebsiella.s__Klebsiella_variicola	+	1.07E-02	0.000	0.000	0.000	0.000	0.002
3.5.4.25|g__Kluyvera.s__Kluyvera_ascorbata	+	3.30E-01	0.000	0.000	0.000	0.000	0.000
3.5.4.25|g__Kluyvera.s__Kluyvera_cryocrescens	+	3.30E-01	0.000	0.000	0.000	0.000	0.000
3.5.4.25|g__Kosakonia.s__Kosakonia_cowanii	+	4.92E-01	0.000	0.000	0.000	0.000	0.000
3.5.4.25|g__Lachnoclostridium.s__Clostridium_aldenense	-	5.40E-01	0.000	0.000	0.000	0.001	0.001
3.5.4.25|g__Lachnoclostridium.s__Clostridium_citroniae	+	1.11E-01	0.000	0.000	0.000	0.000	0.000
3.5.4.25|g__Lachnoclostridium.s__Clostridium_clostridioforme	+	7.05E-01	0.000	0.000	0.000	0.003	0.004
3.5.4.25|g__Lachnoclostridium.s__Clostridium_symbiosum	+	4.60E-01	0.000	0.000	0.000	0.001	0.001
3.5.4.25|g__Lachnoclostridium.s__Lachnoclostridium_sp_An138	+	2.25E-01	0.000	0.000	0.000	0.000	0.000
3.5.4.25|g__Lachnospira.s__Lachnospira_pectinoschiza	+	8.54E-01	0.000	0.000	0.000	0.001	0.001
3.5.4.25|g__Lachnospiraceae_unclassified.s__Lachnospiraceae_bacterium_(	+	4.11E-02	0.000	0.000	0.000	0.000	0.000
3.5.4.25|g__Lactobacillus.s__Lactobacillus_amylovorus	-	1.48E-01	0.000	0.000	0.000	0.000	0.000
3.5.4.25|g__Lactobacillus.s__Lactobacillus_brevis	+	4.92E-01	0.000	0.000	0.000	0.000	0.000
3.5.4.25|g__Lactobacillus.s__Lactobacillus_crispatus	+	2.27E-01	0.000	0.000	0.000	0.000	0.000

3.5.4.25|g__Lactobacillus.s__Lactobacillus_delbrueckii	-	9.70E-01	0.000	0.000	0.000	0.000	0.000
3.5.4.25|g__Lactobacillus.s__Lactobacillus_farciminis	+	3.30E-01	0.000	0.000	0.000	0.000	0.000
3.5.4.25|g__Lactobacillus.s__Lactobacillus_fermentum	+	1.05E-02	0.000	0.000	0.000	0.000	0.001
3.5.4.25|g__Lactobacillus.s__Lactobacillus_kimbladii	+	4.92E-01	0.000	0.000	0.000	0.000	0.000
3.5.4.25|g__Lactobacillus.s__Lactobacillus_kullabergensis	+	4.92E-01	0.000	0.000	0.000	0.000	0.000
3.5.4.25|g__Lactobacillus.s__Lactobacillus_oris	+	8.46E-03	0.000	0.000	0.000	0.000	0.000
3.5.4.25|g__Lactobacillus.s__Lactobacillus_pentosus	+	9.00E-02	0.000	0.000	0.000	0.000	0.000
3.5.4.25|g__Lactobacillus.s__Lactobacillus_reuteri	+	1.48E-02	0.000	0.000	0.000	0.000	0.002
3.5.4.25|g__Lactobacillus.s__Lactobacillus_rogosae	-	9.52E-01	0.000	0.000	0.000	0.001	0.001
3.5.4.25|g__Lactococcus.s__Lactococcus_lactis	+	8.05E-01	0.000	0.000	0.000	0.000	0.000
3.5.4.25|g__Lactococcus.s__Lactococcus_piscium	+	4.92E-01	0.000	0.000	0.000	0.000	0.000
3.5.4.25|g__Leclercia.s__Leclercia_adecarboxylata	+	1.67E-01	0.000	0.000	0.000	0.000	0.000
3.5.4.25|g__Lelliottia.s__Lelliottia_nimipressuralis	+	2.32E-01	0.000	0.000	0.000	0.000	0.000
3.5.4.25|g__Leuconostoc.s__Leuconostoc_citreum	+	4.92E-01	0.000	0.000	0.000	0.000	0.000
3.5.4.25|g__Leuconostoc.s__Leuconostoc_garlicum	+	4.92E-01	0.000	0.000	0.000	0.000	0.000
3.5.4.25|g__Leuconostoc.s__Leuconostoc_lactis	+	3.30E-01	0.000	0.000	0.000	0.000	0.000
3.5.4.25|g__Leuconostoc.s__Leuconostoc_mesenteroides	+	2.32E-01	0.000	0.000	0.000	0.000	0.000
3.5.4.25|g__Megamonas.s__Megamonas_funiformis	+	9.91E-01	0.000	0.000	0.000	0.001	0.001
3.5.4.25|g__Megamonas.s__Megamonas_hypermegale	+	4.92E-01	0.000	0.000	0.000	0.000	0.000
3.5.4.25|g__Megamonas.s__Megamonas_rupellensis	-	7.81E-01	0.000	0.000	0.000	0.000	0.000
3.5.4.25|g__Megasphaera.s__Megasphaera_elsdenii	-	9.60E-01	0.000	0.000	0.000	0.000	0.000
3.5.4.25|g__Megasphaera.s__Megasphaera_hexanoica	+	4.92E-01	0.000	0.000	0.000	0.000	0.000
3.5.4.25|g__Megasphaera.s__Megasphaera_micronuciformis	+	3.30E-01	0.000	0.000	0.000	0.000	0.000
3.5.4.25|g__Megasphaera.s__Megasphaera_sp_DISK_18	+	4.25E-04	0.000	0.000	0.000	0.000	0.003
3.5.4.25|g__Megasphaera.s__Megasphaera_sp_MJR8396C	+	8.66E-02	0.000	0.000	0.000	0.001	0.002
3.5.4.25|g__Megasphaera.s__Megasphaera_stantonii	-	5.92E-01	0.000	0.000	0.000	0.000	0.000
3.5.4.25|g__Mitsuokella.s__Mitsuokella_jalaludinii	-	5.95E-01	0.000	0.000	0.000	0.000	0.000
3.5.4.25|g__Mitsuokella.s__Mitsuokella_multacida	+	4.92E-01	0.000	0.000	0.000	0.000	0.000
3.5.4.25|g__Mogibacterium.s__Mogibacterium_diversum	+	1.22E-01	0.000	0.000	0.000	0.000	0.000
3.5.4.25|g__Mogibacterium.s__Mogibacterium_timidum	+	4.92E-01	0.000	0.000	0.000	0.000	0.000
3.5.4.25|g__Muribaculum.s__Muribaculum_intestinale	-	5.92E-01	0.000	0.000	0.000	0.000	0.000
3.5.4.25|g__Odoribacter.s__Odoribacter_laneus	+	1.90E-01	0.000	0.000	0.000	0.001	0.003
3.5.4.25|g__Pantoea.s__Pantoea_sesami	+	4.16E-01	0.000	0.000	0.000	0.000	0.000
3.5.4.25|g__Parabacteroides.s__Parabacteroides_goldsteinii	+	1.90E-02	0.000	0.000	0.000	0.001	0.002
3.5.4.25|g__Parabacteroides.s__Parabacteroides_gordonii	-	9.24E-01	0.000	0.000	0.000	0.000	0.000
3.5.4.25|g__Parabacteroides.s__Parabacteroides_johnsonii	-	3.66E-01	0.000	0.000	0.000	0.001	0.001
3.5.4.25|g__Paraprevotella.s__Paraprevotella_clara	-	1.18E-01	0.000	0.000	0.000	0.003	0.003
3.5.4.25|g__Paraprevotella.s__Paraprevotella_xylaniphila	+	4.16E-01	0.000	0.000	0.000	0.000	0.000
3.5.4.25|g__Pediococcus.s__Pediococcus_acidilactici	+	4.99E-02	0.000	0.000	0.000	0.000	0.001
3.5.4.25|g__Pedobacter.s__Pedobacter_himalayensis	+	2.32E-01	0.000	0.000	0.000	0.000	0.000
3.5.4.25|g__Peptococcus.s__Peptococcus_niger	+	2.31E-01	0.000	0.000	0.000	0.000	0.000
3.5.4.25|g__Peptoniphilus.s__Peptoniphilus_coxii	-	1.48E-01	0.000	0.000	0.000	0.000	0.000
3.5.4.25|g__Peptoniphilus.s__Peptoniphilus_duerdenii	+	4.92E-01	0.000	0.000	0.000	0.000	0.000
3.5.4.25|g__Peptoniphilus.s__Peptoniphilus_harei	+	1.67E-01	0.000	0.000	0.000	0.000	0.000
3.5.4.25|g__Peptoniphilus.s__Peptoniphilus_lacrimalis	+	4.92E-01	0.000	0.000	0.000	0.000	0.000
3.5.4.25|g__Peptoniphilus.s__Peptoniphilus_sp_HMSC062D09	+	2.32E-01	0.000	0.000	0.000	0.000	0.000
3.5.4.25|g__Peptoniphilus.s__Peptoniphilus_sp_oral_taxon_375	+	4.92E-01	0.000	0.000	0.000	0.000	0.000
3.5.4.25|g__Peptostreptococcaceae_unclassified.s__Clostridium_hiranonis	-	5.92E-01	0.000	0.000	0.000	0.000	0.000
3.5.4.25|g__Peptostreptococcus.s__Peptostreptococcus_anaerobius	+	2.32E-01	0.000	0.000	0.000	0.000	0.000
3.5.4.25|g__Phascolarctobacterium.s__Phascolarctobacterium_succinatutens	+	1.82E-01	0.000	0.000	0.000	0.001	0.001
3.5.4.25|g__Phytobacter.s__Phytobacter_ursingii	+	4.92E-01	0.000	0.000	0.000	0.000	0.000
3.5.4.25|g__Pluralibacter.s__Pluralibacter_gergoviae	-	1.48E-01	0.000	0.000	0.000	0.000	0.000
3.5.4.25|g__Porphyromonas.s__Porphyromonas_asaccharolytica	+	9.90E-02	0.000	0.000	0.000	0.000	0.000
3.5.4.25|g__Porphyromonas.s__Porphyromonas_sp_HMSC065F10	+	7.55E-01	0.000	0.000	0.000	0.000	0.000
3.5.4.25|g__Porphyromonas.s__Porphyromonas_sp_HMSC077F02	+	5.55E-01	0.000	0.000	0.000	0.000	0.000
3.5.4.25|g__Porphyromonas.s__Porphyromonas_uenonis	-	9.70E-01	0.000	0.000	0.000	0.000	0.000
3.5.4.25|g__Prevotella.s__Prevotella_bergensis	+	4.92E-01	0.000	0.000	0.000	0.000	0.000
3.5.4.25|g__Prevotella.s__Prevotella_bivia	+	2.29E-01	0.000	0.000	0.000	0.000	0.000
3.5.4.25|g__Prevotella.s__Prevotella_buccae	-	9.73E-01	0.000	0.000	0.000	0.000	0.000
3.5.4.25|g__Prevotella.s__Prevotella_buccalis	+	4.99E-02	0.000	0.000	0.000	0.000	0.000
3.5.4.25|g__Prevotella.s__Prevotella_colorans	+	3.30E-01	0.000	0.000	0.000	0.000	0.000
3.5.4.25|g__Prevotella.s__Prevotella_copri	-	9.35E-01	0.000	0.000	0.000	0.002	0.001
3.5.4.25|g__Prevotella.s__Prevotella_corporis	-	4.35E-01	0.000	0.000	0.000	0.000	0.000
3.5.4.25|g__Prevotella.s__Prevotella_disiens	+	8.32E-01	0.000	0.000	0.000	0.000	0.000
3.5.4.25|g__Prevotella.s__Prevotella_histicola	+	4.92E-01	0.000	0.000	0.000	0.000	0.000
3.5.4.25|g__Prevotella.s__Prevotella_sp_109	-	7.86E-01	0.000	0.000	0.000	0.003	0.002
3.5.4.25|g__Prevotella.s__Prevotella_sp_885	-	4.45E-01	0.000	0.000	0.000	0.000	0.000
3.5.4.25|g__Prevotella.s__Prevotella_sp_AM42_24	-	2.20E-02	0.000	0.000	0.000	0.001	0.000
3.5.4.25|g__Prevotella.s__Prevotella_stercorea	-	4.75E-01	0.000	0.000	0.000	0.003	0.001
3.5.4.25|g__Prevotella.s__Prevotella_timonensis	+	9.79E-02	0.000	0.000	0.000	0.000	0.000
3.5.4.25|g__Propionibacterium.s__Propionibacterium_acidifaciens	+	4.92E-01	0.000	0.000	0.000	0.000	0.000
3.5.4.25|g__Propionibacterium.s__Propionibacterium_freudenreichii	-	9.57E-01	0.000	0.000	0.000	0.000	0.000

3.5.4.25|g__Proteus.s__Proteus_mirabilis	+	3.30E-01	0.000	0.000	0.000	0.000	0.000
3.5.4.25|g__Proteus.s__Proteus_penneri	+	4.92E-01	0.000	0.000	0.000	0.000	0.000
3.5.4.25|g__Pseudocitrobacter.s__Pseudocitrobacter_faecalis	+	4.92E-01	0.000	0.000	0.000	0.000	0.000
3.5.4.25|g__Pseudoflavonifractor.s__Pseudoflavonifractor_sp_An184	+	1.09E-01	0.000	0.000	0.000	0.001	0.001
3.5.4.25|g__Pseudomonas.s__Pseudomonas_aeruginosa	+	1.32E-01	0.000	0.000	0.000	0.000	0.000
3.5.4.25|g__Pseudomonas.s__Pseudomonas_aeruginosa_group	+	7.55E-02	0.000	0.000	0.000	0.000	0.000
3.5.4.25|g__Pseudomonas.s__Pseudomonas_citronellolis	+	4.92E-01	0.000	0.000	0.000	0.000	0.000
3.5.4.25|g__Pseudomonas.s__Pseudomonas_delhiensis	+	4.92E-01	0.000	0.000	0.000	0.000	0.000
3.5.4.25|g__Pseudoramibacter.s__Pseudoramibacter_alactolyticus	+	4.92E-01	0.000	0.000	0.000	0.000	0.000
3.5.4.25|g__Pyramidobacter.s__Pyramidobacter_piscolens	+	1.29E-01	0.000	0.000	0.000	0.000	0.001
3.5.4.25|g__Pyramidobacter.s__Pyramidobacter_sp_C12_8	+	4.92E-01	0.000	0.000	0.000	0.000	0.000
3.5.4.25|g__Raoultella.s__Raoultella_ornithinolytica	-	1.48E-01	0.000	0.000	0.000	0.000	0.000
3.5.4.25|g__Raoultella.s__Raoultella_planticola	-	9.73E-01	0.000	0.000	0.000	0.000	0.000
3.5.4.25|g__Rikenella.s__Rikenella_microfusus	+	4.92E-01	0.000	0.000	0.000	0.000	0.000
3.5.4.25|g__Roseburia.s__Roseburia_inulinivorans	-	2.92E-01	0.000	0.000	0.000	0.001	0.001
3.5.4.25|g__Rothia.s__Rothia_dentocariosa	+	6.61E-01	0.000	0.000	0.000	0.000	0.000
3.5.4.25|g__Rothia.s__Rothia_mucilaginosa	-	3.27E-01	0.000	0.000	0.000	0.000	0.000
3.5.4.25|g__Ruminococcaceae_unclassified.s__Ruminococcaceae_bacteriun	+	6.81E-01	0.000	0.000	0.000	0.002	0.003
3.5.4.25|g__Ruminococcaceae_unclassified.s__Ruminococcaceae_bacteriun	+	1.08E-02	0.000	0.000	0.000	0.000	0.000
3.5.4.25|g__Ruminococcus.s__Ruminococcus_callidus	-	1.27E-03	0.000	0.000	0.000	0.003	0.001
3.5.4.25|g__Ruminococcus.s__Ruminococcus_sp_AF31_8BH	-	1.05E-02	0.000	0.000	0.000	0.001	0.001
3.5.4.25|g__Saccharomyces.s__Saccharomyces_cerevisiae	-	1.48E-01	0.000	0.000	0.000	0.000	0.000
3.5.4.25|g__Salmonella.s__Salmonella_enterica	+	4.92E-01	0.000	0.000	0.000	0.000	0.000
3.5.4.25|g__Sanguibacteroides.s__Sanguibacteroides_justesenii	+	9.00E-02	0.000	0.000	0.000	0.000	0.000
3.5.4.25|g__Senegalimassilia.s__Senegalimassilia_anaerobia	+	2.19E-01	0.000	0.000	0.000	0.000	0.000
3.5.4.25|g__Serratia.s__Serratia_marcescens	+	4.92E-01	0.000	0.000	0.000	0.000	0.000
3.5.4.25|g__Slackia.s__Slackia_isoflavoniconvertens	+	7.39E-03	0.000	0.000	0.000	0.000	0.000
3.5.4.25|g__Staphylococcus.s__Staphylococcus_aureus	+	2.32E-01	0.000	0.000	0.000	0.000	0.000
3.5.4.25|g__Staphylococcus.s__Staphylococcus_haemolyticus	+	4.92E-01	0.000	0.000	0.000	0.000	0.000
3.5.4.25|g__Streptococcus.s__Streptococcus_agalactiae	+	4.92E-01	0.000	0.000	0.000	0.000	0.000
3.5.4.25|g__Streptococcus.s__Streptococcus_equinus	+	1.30E-02	0.000	0.000	0.000	0.000	0.001
3.5.4.25|g__Streptococcus.s__Streptococcus_gallolyticus	+	2.32E-03	0.000	0.000	0.000	0.000	0.001
3.5.4.25|g__Streptococcus.s__Streptococcus_infantarius	+	1.26E-02	0.000	0.000	0.000	0.000	0.001
3.5.4.25|g__Streptococcus.s__Streptococcus_infantis	-	1.48E-01	0.000	0.000	0.000	0.000	0.000
3.5.4.25|g__Streptococcus.s__Streptococcus_lutetiensis	+	1.25E-02	0.000	0.000	0.000	0.000	0.001
3.5.4.25|g__Streptococcus.s__Streptococcus_macedonicus	+	5.86E-02	0.000	0.000	0.000	0.000	0.002
3.5.4.25|g__Streptococcus.s__Streptococcus_oralis	+	2.32E-01	0.000	0.000	0.000	0.000	0.000
3.5.4.25|g__Streptococcus.s__Streptococcus_pasteurianus	+	7.39E-02	0.000	0.000	0.000	0.000	0.002
3.5.4.25|g__Streptococcus.s__Streptococcus_pneumoniae	-	7.08E-02	0.000	0.000	0.000	0.000	0.000
3.5.4.25|g__Succinatimonas.s__Succinatimonas_hippei	+	7.53E-01	0.000	0.000	0.000	0.000	0.000
3.5.4.25|g__Sutterella.s__Sutterella_wadsworthensis	+	1.89E-01	0.000	0.000	0.000	0.001	0.001
3.5.4.25|g__Terrisporobacter.s__Terrisporobacter_othiniensis	+	1.67E-01	0.000	0.000	0.000	0.000	0.000
3.5.4.25|g__Trichococcus.s__Trichococcus_collinsii	+	4.92E-01	0.000	0.000	0.000	0.000	0.000
3.5.4.25|g__Trichococcus.s__Trichococcus_flocculiformis	+	4.92E-01	0.000	0.000	0.000	0.000	0.000
3.5.4.25|g__Trichococcus.s__Trichococcus_pasteurii	+	4.92E-01	0.000	0.000	0.000	0.000	0.000
3.5.4.25|g__Varibaculum.s__Varibaculum_cambriense	+	3.30E-01	0.000	0.000	0.000	0.000	0.000
3.5.4.25|g__Veillonella.s__Veillonella_atypica	-	7.91E-01	0.000	0.000	0.000	0.000	0.001
3.5.4.25|g__Veillonella.s__Veillonella_dispar	-	4.89E-01	0.000	0.000	0.000	0.000	0.000
3.5.4.25|g__Veillonella.s__Veillonella_infantium	+	4.99E-02	0.000	0.000	0.000	0.000	0.000
3.5.4.25|g__Veillonella.s__Veillonella_parvula	+	6.97E-01	0.000	0.000	0.000	0.000	0.000
3.5.4.25|g__Veillonella.s__Veillonella_rogosae	-	9.20E-01	0.000	0.000	0.000	0.000	0.000
3.5.4.25|g__Veillonella.s__Veillonella_seminalis	-	4.47E-01	0.000	0.000	0.000	0.000	0.000
3.5.4.25|g__Vibrio.s__Vibrio_parahaemolyticus	+	4.10E-01	0.000	0.000	0.000	0.000	0.000
3.5.4.25|g__Victivallales_unclassified.s__Victivallales_bacterium_CCUG_4	+	3.58E-03	0.000	0.000	0.000	0.000	0.000
3.5.4.25|g__Victivallis.s__Victivallis_vadensis	+	9.64E-02	0.000	0.000	0.000	0.000	0.000
3.5.4.25|g__Weissella.s__Weissella_cibaria	-	9.67E-01	0.000	0.000	0.000	0.000	0.000
3.5.4.25|g__Weissella.s__Weissella_confusa	-	4.48E-01	0.000	0.000	0.000	0.000	0.000
3.5.4.25|g__Bacteroides.s__Bacteroides_dorei	+	6.63E-01	0.117	0.124	0.007	0.013	0.016
3.5.4.25|g__Bacteroides.s__Bacteroides_xylanisolvens	+	3.25E-01	0.000	0.015	0.015	0.007	0.006
3.5.4.25|g__Odoribacter.s__Odoribacter_splanchnicus	+	3.98E-01	0.223	0.243	0.020	0.003	0.004
3.5.4.25|g__Eggerthella.s__Eggerthella_lenta	+	2.14E-01	0.000	0.020	0.020	0.002	0.002
3.5.4.25|g__Bacteroides.s__Bacteroides_thetaiotaomicron	+	4.82E-01	0.409	0.439	0.029	0.012	0.013
3.5.4.25|g__Roseburia.s__Roseburia_hominis	-	8.99E-01	1.485	1.546	0.061	0.018	0.020
3.5.4.25|g__Bacteroides.s__Bacteroides_cellulosilyticus	+	3.60E-01	0.121	0.208	0.087	0.015	0.014
3.5.4.25|unclassified	-	9.57E-01	12.610	12.698	0.087	0.149	0.150
3.5.4.25|g__Phascolarctobacterium.s__Phascolarctobacterium_faecium	+	7.34E-01	0.475	0.564	0.089	0.009	0.010
3.5.4.25|g__Bacteroides.s__Bacteroides_caccae	+	2.33E-01	0.229	0.346	0.117	0.008	0.011
3.5.4.25|g__Bilophila.s__Bilophila_wadsworthia	+	2.66E-02	0.132	0.266	0.134	0.003	0.004
3.5.4.25|g__Parabacteroides.s__Parabacteroides_merdae	+	7.65E-02	0.523	0.699	0.176	0.008	0.009
3.5.4.25|g__Alistipes.s__Alistipes_onderdonkii	+	3.01E-03	0.435	0.647	0.211	0.011	0.016
3.5.4.25|g__Parabacteroides.s__Parabacteroides_distasonis	+	1.55E-02	1.054	1.348	0.295	0.020	0.022
3.5.4.25|g__Alistipes.s__Alistipes_finegoldii	+	5.76E-03	0.722	1.122	0.400	0.019	0.025

	3.5.4.25|g__Bacteroides.s__Bacteroides_stercoris	+	4.32E-02	0.044	0.478	0.435	0.039	0.045
Germany	3.5.4.25	-	1.79E-01	121.504	110.669	-10.835	1.000	1.000
	3.5.4.25|g__Faecalibacterium.s__Faecalibacterium_prausnitzii	-	2.83E-02	11.000	5.631	-5.370	0.092	0.062
	3.5.4.25|g__Bacteroides.s__Bacteroides_uniformis	+	8.08E-01	2.984	2.266	-0.717	0.044	0.060
	3.5.4.25|g__Roseburia.s__Roseburia_faecis	-	2.44E-01	0.911	0.296	-0.615	0.017	0.011
	3.5.4.25|g__Parabacteroides.s__Parabacteroides_distasonis	-	8.38E-01	1.668	1.172	-0.496	0.015	0.014
	3.5.4.25|g__Roseburia.s__Roseburia_intestinalis	-	1.57E-01	0.365	0.000	-0.365	0.018	0.005
	3.5.4.25|g__Dorea.s__Dorea_longicatena	-	8.97E-01	0.975	0.637	-0.338	0.010	0.011
	3.5.4.25|g__Blautia.s__Blautia_obeum	-	2.84E-01	0.863	0.535	-0.327	0.009	0.009
	3.5.4.25|g__Coprococcus.s__Coprococcus_comes	-	5.85E-01	0.519	0.305	-0.213	0.005	0.006
	3.5.4.25|g__Dorea.s__Dorea_formicigenerans	-	2.46E-02	0.272	0.121	-0.151	0.003	0.001
	3.5.4.25|g__Eubacterium.s__Eubacterium_ramulus	-	4.92E-01	0.147	0.000	-0.147	0.002	0.002
	3.5.4.25|g__Coprococcus.s__Coprococcus_catus	-	3.09E-01	0.126	0.000	-0.126	0.002	0.002
	3.5.4.25|g__Bacteroides.s__Bacteroides_ovatus	-	5.14E-01	0.261	0.140	-0.122	0.005	0.008
	3.5.4.25|g__Lachnospiraceae_unclassified.s__Eubacterium_rectale	+	7.21E-01	0.750	0.696	-0.053	0.015	0.017
	3.5.4.25|g__Eubacterium.s__Eubacterium_eligens	+	9.70E-01	0.842	0.813	-0.029	0.018	0.014
	3.5.4.25|g__Blautia.s__Blautia_wexlerae	-	3.16E-01	0.192	0.164	-0.028	0.003	0.002
	3.5.4.25|g__Agathobaculum.s__Agathobaculum_butyriciproducens	+	3.77E-01	0.220	0.217	-0.003	0.002	0.004
	3.5.4.25|g__Odoribacter.s__Odoribacter_splanchnicus	+	9.64E-01	0.523	0.522	-0.001	0.005	0.005
	3.5.4.25|g__Acidaminococcus.s__Acidaminococcus_fermentans	-	9.42E-01	0.000	0.000	0.000	0.000	0.000
	3.5.4.25|g__Acidaminococcus.s__Acidaminococcus_intestini	-	6.15E-01	0.000	0.000	0.000	0.001	0.002
	3.5.4.25|g__Actinomyces.s__Actinomyces_naeslundii	-	3.09E-01	0.000	0.000	0.000	0.000	0.000
	3.5.4.25|g__Actinomyces.s__Actinomyces_oris	-	3.09E-01	0.000	0.000	0.000	0.000	0.000
	3.5.4.25|g__Actinomyces.s__Actinomyces_viscosus	-	3.09E-01	0.000	0.000	0.000	0.000	0.000
	3.5.4.25|g__Adlercreutzia.s__Adlercreutzia_equolifaciens	+	9.80E-02	0.000	0.000	0.000	0.000	0.000
	3.5.4.25|g__Alistipes.s__Alistipes_indistinctus	-	9.21E-01	0.000	0.000	0.000	0.001	0.001
	3.5.4.25|g__Alistipes.s__Alistipes_inops	+	2.30E-01	0.000	0.000	0.000	0.002	0.004
	3.5.4.25|g__Alistipes.s__Alistipes_sp_An66	+	1.83E-01	0.000	0.000	0.000	0.000	0.000
	3.5.4.25|g__Alistipes.s__Alistipes_timonensis	+	6.04E-01	0.000	0.000	0.000	0.000	0.001
	3.5.4.25|g__Anaeromassilibacillus.s__Anaeromassilibacillus_sp_An250	+	2.86E-01	0.000	0.000	0.000	0.000	0.000
	3.5.4.25|g__Anaerotignum.s__Anaerotignum_lactatifermentans	-	9.81E-01	0.000	0.000	0.000	0.000	0.000
	3.5.4.25|g__Asaccharobacter.s__Asaccharobacter_celatus	+	9.86E-02	0.000	0.000	0.000	0.001	0.002
	3.5.4.25|g__Bacteroides.s__Bacteroides_clarus	-	7.80E-01	0.000	0.000	0.000	0.003	0.002
	3.5.4.25|g__Bacteroides.s__Bacteroides_coprocola	-	5.25E-01	0.000	0.000	0.000	0.007	0.002
	3.5.4.25|g__Bacteroides.s__Bacteroides_coprophilus	-	8.75E-01	0.000	0.000	0.000	0.004	0.000
	3.5.4.25|g__Bacteroides.s__Bacteroides_eggerthii	+	9.42E-01	0.000	0.000	0.000	0.014	0.004
	3.5.4.25|g__Bacteroides.s__Bacteroides_faecichinchillae	+	9.80E-02	0.000	0.000	0.000	0.000	0.000
	3.5.4.25|g__Bacteroides.s__Bacteroides_faecis	+	7.54E-01	0.000	0.000	0.000	0.003	0.003
	3.5.4.25|g__Bacteroides.s__Bacteroides_finegoldii	+	3.97E-01	0.000	0.000	0.000	0.002	0.004
	3.5.4.25|g__Bacteroides.s__Bacteroides_fluxus	-	1.40E-01	0.000	0.000	0.000	0.000	0.000
	3.5.4.25|g__Bacteroides.s__Bacteroides_fragilis	+	9.66E-01	0.000	0.000	0.000	0.002	0.006
	3.5.4.25|g__Bacteroides.s__Bacteroides_intestinalis	+	8.59E-01	0.000	0.000	0.000	0.001	0.002
	3.5.4.25|g__Bacteroides.s__Bacteroides_massiliensis	-	8.85E-01	0.000	0.000	0.000	0.014	0.013
	3.5.4.25|g__Bacteroides.s__Bacteroides_nordii	+	9.35E-01	0.000	0.000	0.000	0.000	0.000
	3.5.4.25|g__Bacteroides.s__Bacteroides_plebeius	+	3.37E-01	0.000	0.000	0.000	0.002	0.006
	3.5.4.25|g__Bacteroides.s__Bacteroides_salyersiae	+	8.06E-01	0.000	0.000	0.000	0.001	0.004
	3.5.4.25|g__Bacteroides.s__Bacteroides_sp_OM08_11	+	3.59E-01	0.000	0.000	0.000	0.000	0.000
	3.5.4.25|g__Bacteroides.s__Bacteroides_stercorirosoris	+	3.59E-01	0.000	0.000	0.000	0.000	0.000
	3.5.4.25|g__Bacteroides.s__Bacteroides_stercoris	-	7.80E-01	0.000	0.000	0.000	0.016	0.011
	3.5.4.25|g__Blautia.s__Blautia_hansenii	+	3.59E-01	0.000	0.000	0.000	0.000	0.000
	3.5.4.25|g__Blautia.s__Blautia_sp_AF19_10LB	+	1.80E-01	0.000	0.000	0.000	0.001	0.001
	3.5.4.25|g__Butyricicoccus.s__Butyricicoccus_pullicaecorum	+	3.59E-01	0.000	0.000	0.000	0.000	0.000
	3.5.4.25|g__Butyricimonas.s__Butyricimonas_virosa	-	2.60E-01	0.000	0.000	0.000	0.001	0.001
	3.5.4.25|g__Butyrivibrio.s__Butyrivibrio_crossotus	+	8.14E-01	0.000	0.000	0.000	0.011	0.003
	3.5.4.25|g__Candidatus_Gastranaerophilales_unclassified.s__Candidatus_Ga	+	2.20E-01	0.000	0.000	0.000	0.000	0.001
	3.5.4.25|g__Catenibacterium.s__Catenibacterium_mitsuokai	-	5.62E-02	0.000	0.000	0.000	0.010	0.004
	3.5.4.25|g__Citrobacter.s__Citrobacter_sp_MGH106	+	3.59E-01	0.000	0.000	0.000	0.000	0.000
	3.5.4.25|g__Cloacibacillus.s__Cloacibacillus_porcorum	+	6.04E-01	0.000	0.000	0.000	0.000	0.000
	3.5.4.25|g__Clostridioides.s__Clostridioides_difficile	-	9.58E-01	0.000	0.000	0.000	0.000	0.000
	3.5.4.25|g__Clostridium.s__Clostridium_celatum	-	3.09E-01	0.000	0.000	0.000	0.000	0.000
	3.5.4.25|g__Clostridium.s__Clostridium_disporicum	-	1.40E-01	0.000	0.000	0.000	0.000	0.000
	3.5.4.25|g__Clostridium.s__Clostridium_perfringens	-	3.09E-01	0.000	0.000	0.000	0.000	0.000
	3.5.4.25|g__Coprobacillus.s__Coprobacillus_cateniformis	+	4.43E-01	0.000	0.000	0.000	0.000	0.001
	3.5.4.25|g__Coprobacter.s__Coprobacter_fastidiosus	-	8.96E-01	0.000	0.000	0.000	0.002	0.001
	3.5.4.25|g__Coprobacter.s__Coprobacter_secundus	-	9.29E-01	0.000	0.000	0.000	0.000	0.000
	3.5.4.25|g__Coprococcus.s__Coprococcus_eutactus	-	8.36E-01	0.000	0.000	0.000	0.013	0.008
	3.5.4.25|g__Desulfovibrio.s__Desulfovibrio_fairfieldensis	+	3.38E-01	0.000	0.000	0.000	0.000	0.001
	3.5.4.25|g__Desulfovibrio.s__Desulfovibrio_piger	-	5.54E-01	0.000	0.000	0.000	0.001	0.005
	3.5.4.25|g__Desulfovibrionaceae_unclassified.s__Desulfovibrionaceae_bact	-	3.09E-01	0.000	0.000	0.000	0.000	0.000
	3.5.4.25|g__Dialister.s__Dialister_invisus	-	7.94E-01	0.000	0.000	0.000	0.009	0.009
	3.5.4.25|g__Dialister.s__Dialister_succinatiphilus	-	9.42E-01	0.000	0.000	0.000	0.001	0.001
	3.5.4.25|g__Dorea.s__Dorea_sp_OM02_2LB	-	9.31E-01	0.000	0.000	0.000	0.000	0.000

3.5.4.25|g__Eggerthella.s__Eggerthella_lenta	-	6.66E-02	0.000	0.000	0.000	0.000	0.000
3.5.4.25|g__Enterococcus.s__Enterococcus_hirae	-	3.09E-01	0.000	0.000	0.000	0.000	0.000
3.5.4.25|g__Erysipelatoclostridium.s__Clostridium_innocuum	+	3.59E-01	0.000	0.000	0.000	0.000	0.000
3.5.4.25|g__Erysipelatoclostridium.s__Clostridium_spiroforme	-	8.75E-01	0.000	0.000	0.000	0.000	0.000
3.5.4.25|g__Erysipelotrichaceae_unclassified.s__Erysipelotrichaceae_bacteri	-	3.09E-01	0.000	0.000	0.000	0.000	0.000
3.5.4.25|g__Escherichia.s__Escherichia_coli	-	4.63E-01	0.000	0.000	0.000	0.029	0.012
3.5.4.25|g__Escherichia.s__Escherichia_marmotae	-	3.09E-01	0.000	0.000	0.000	0.000	0.000
3.5.4.25|g__Eubacterium.s__Eubacterium_sp_AF17_7	+	1.01E-01	0.000	0.000	0.000	0.000	0.001
3.5.4.25|g__Eubacterium.s__Eubacterium_sp_AM18_10LB_B	-	3.09E-01	0.000	0.000	0.000	0.000	0.000
3.5.4.25|g__Faecalitalea.s__Faecalitalea_cylindroides	-	8.75E-01	0.000	0.000	0.000	0.002	0.000
3.5.4.25|g__Firmicutes_unclassified.s__Firmicutes_bacterium_AM10_47	-	2.58E-01	0.000	0.000	0.000	0.000	0.000
3.5.4.25|g__Flavonifractor.s__Flavonifractor_plautii	+	1.86E-02	0.000	0.000	0.000	0.000	0.002
3.5.4.25|g__Flavonifractor.s__Flavonifractor_sp_An10	-	9.31E-01	0.000	0.000	0.000	0.000	0.000
3.5.4.25|g__Fusobacterium.s__Fusobacterium_mortiferum	-	3.09E-01	0.000	0.000	0.000	0.000	0.000
3.5.4.25|g__Haemophilus.s__Haemophilus_parainfluenzae	-	1.40E-01	0.000	0.000	0.000	0.001	0.000
3.5.4.25|g__Hafnia.s__Hafnia_alvei	+	3.59E-01	0.000	0.000	0.000	0.000	0.000
3.5.4.25|g__Hafnia.s__Hafnia_paralvei	+	3.59E-01	0.000	0.000	0.000	0.000	0.001
3.5.4.25|g__Holdemanella.s__Holdemanella_biformis	-	1.40E-01	0.000	0.000	0.000	0.000	0.000
3.5.4.25|g__Hungatella.s__Hungatella_hathewayi	-	9.10E-03	0.000	0.000	0.000	0.000	0.000
3.5.4.25|g__Intestinibacter.s__Intestinibacter_bartlettii	-	8.75E-01	0.000	0.000	0.000	0.000	0.000
3.5.4.25|g__Klebsiella.s__Klebsiella_aerogenes	-	3.22E-02	0.000	0.000	0.000	0.000	0.000
3.5.4.25|g__Klebsiella.s__Klebsiella_oxytoca	-	8.37E-01	0.000	0.000	0.000	0.004	0.002
3.5.4.25|g__Klebsiella.s__Klebsiella_pneumoniae	-	3.22E-02	0.000	0.000	0.000	0.002	0.000
3.5.4.25|g__Klebsiella.s__Klebsiella_variicola	+	6.61E-01	0.000	0.000	0.000	0.000	0.000
3.5.4.25|g__Lachnoclostridium.s__Clostridium_aldenense	-	3.09E-01	0.000	0.000	0.000	0.000	0.000
3.5.4.25|g__Lachnoclostridium.s__Clostridium_bolteae	-	2.10E-02	0.000	0.000	0.000	0.000	0.000
3.5.4.25|g__Lachnoclostridium.s__Clostridium_clostridioforme	-	6.66E-02	0.000	0.000	0.000	0.000	0.000
3.5.4.25|g__Lachnoclostridium.s__Clostridium_symbiosum	-	4.85E-01	0.000	0.000	0.000	0.000	0.000
3.5.4.25|g__Lachnospira.s__Lachnospira_pectinoschiza	+	6.22E-01	0.000	0.000	0.000	0.001	0.003
3.5.4.25|g__Lachnospiraceae_unclassified.s__Lachnospiraceae_bacterium_(	-	1.40E-01	0.000	0.000	0.000	0.000	0.000
3.5.4.25|g__Lactobacillus.s__Lactobacillus_antri	-	3.09E-01	0.000	0.000	0.000	0.000	0.000
3.5.4.25|g__Lactobacillus.s__Lactobacillus_crispatus	-	3.09E-01	0.000	0.000	0.000	0.000	0.000
3.5.4.25|g__Lactobacillus.s__Lactobacillus_gastricus	-	3.09E-01	0.000	0.000	0.000	0.000	0.000
3.5.4.25|g__Lactobacillus.s__Lactobacillus_oris	-	3.09E-01	0.000	0.000	0.000	0.000	0.000
3.5.4.25|g__Lactobacillus.s__Lactobacillus_rogosae	+	5.83E-01	0.000	0.000	0.000	0.001	0.003
3.5.4.25|g__Lactococcus.s__Lactococcus_lactis	-	8.75E-01	0.000	0.000	0.000	0.002	0.000
3.5.4.25|g__Megamonas.s__Megamonas_funiformis	+	3.59E-01	0.000	0.000	0.000	0.000	0.001
3.5.4.25|g__Megamonas.s__Megamonas_rupellensis	+	3.59E-01	0.000	0.000	0.000	0.000	0.001
3.5.4.25|g__Megasphaera.s__Megasphaera_elsdenii	-	4.85E-01	0.000	0.000	0.000	0.002	0.000
3.5.4.25|g__Megasphaera.s__Megasphaera_hexanoica	-	3.09E-01	0.000	0.000	0.000	0.000	0.000
3.5.4.25|g__Megasphaera.s__Megasphaera_sp_DISK_18	+	3.59E-01	0.000	0.000	0.000	0.000	0.001
3.5.4.25|g__Mitsuokella.s__Mitsuokella_jalaludinii	-	5.11E-01	0.000	0.000	0.000	0.001	0.000
3.5.4.25|g__Mitsuokella.s__Mitsuokella_multacida	-	3.09E-01	0.000	0.000	0.000	0.000	0.000
3.5.4.25|g__Obesumbacterium.s__Obesumbacterium_proteus	+	6.04E-01	0.000	0.000	0.000	0.000	0.001
3.5.4.25|g__Parabacteroides.s__Parabacteroides_goldsteinii	-	5.94E-01	0.000	0.000	0.000	0.001	0.000
3.5.4.25|g__Parabacteroides.s__Parabacteroides_gordonii	+	3.38E-01	0.000	0.000	0.000	0.000	0.000
3.5.4.25|g__Parabacteroides.s__Parabacteroides_johnsonii	-	3.04E-01	0.000	0.000	0.000	0.001	0.000
3.5.4.25|g__Paraprevotella.s__Paraprevotella_clara	-	4.48E-02	0.000	0.000	0.000	0.005	0.002
3.5.4.25|g__Phascolarctobacterium.s__Phascolarctobacterium_faecium	+	2.16E-01	0.000	0.000	0.000	0.004	0.009
3.5.4.25|g__Phascolarctobacterium.s__Phascolarctobacterium_succinatutens	-	2.21E-02	0.000	0.000	0.000	0.007	0.001
3.5.4.25|g__Prevotella.s__Prevotella_copri	-	6.66E-02	0.000	0.000	0.000	0.010	0.000
3.5.4.25|g__Prevotella.s__Prevotella_disiens	-	3.09E-01	0.000	0.000	0.000	0.000	0.000
3.5.4.25|g__Prevotella.s__Prevotella_sp_109	+	1.83E-01	0.000	0.000	0.000	0.000	0.009
3.5.4.25|g__Prevotella.s__Prevotella_sp_885	-	5.37E-01	0.000	0.000	0.000	0.001	0.002
3.5.4.25|g__Prevotella.s__Prevotella_sp_AM42_24	-	5.11E-01	0.000	0.000	0.000	0.011	0.007
3.5.4.25|g__Pseudoflavonifractor.s__Pseudoflavonifractor_sp_An184	+	8.71E-01	0.000	0.000	0.000	0.001	0.000
3.5.4.25|g__Pseudomonas.s__Pseudomonas_aeruginosa	-	1.40E-01	0.000	0.000	0.000	0.000	0.000
3.5.4.25|g__Pseudomonas.s__Pseudomonas_aeruginosa_group	-	1.40E-01	0.000	0.000	0.000	0.000	0.000
3.5.4.25|g__Pyramidobacter.s__Pyramidobacter_piscolens	-	3.09E-01	0.000	0.000	0.000	0.000	0.000
3.5.4.25|g__Roseburia.s__Roseburia_inulinivorans	+	1.63E-01	0.000	0.000	0.000	0.002	0.002
3.5.4.25|g__Ruminococcaceae_unclassified.s__Ruminococcaceae_bacteriun	-	5.50E-01	0.000	0.000	0.000	0.001	0.000
3.5.4.25|g__Ruminococcaceae_unclassified.s__Ruminococcaceae_bacteriun	+	1.83E-01	0.000	0.000	0.000	0.000	0.000
3.5.4.25|g__Ruminococcus.s__Ruminococcus_callidus	-	3.65E-01	0.000	0.000	0.000	0.002	0.001
3.5.4.25|g__Ruminococcus.s__Ruminococcus_sp_AF31_8BH	+	2.37E-01	0.000	0.000	0.000	0.001	0.001
3.5.4.25|g__Sanguibacteroides.s__Sanguibacteroides_justesenii	-	5.37E-01	0.000	0.000	0.000	0.000	0.000
3.5.4.25|g__Senegalimassilia.s__Senegalimassilia_anaerobia	+	7.52E-01	0.000	0.000	0.000	0.001	0.002
3.5.4.25|g__Slackia.s__Slackia_isoflavoniconvertens	-	3.23E-01	0.000	0.000	0.000	0.002	0.003
3.5.4.25|g__Streptococcus.s__Streptococcus_equinus	+	3.59E-01	0.000	0.000	0.000	0.000	0.000
3.5.4.25|g__Streptococcus.s__Streptococcus_infantarius	+	3.59E-01	0.000	0.000	0.000	0.000	0.000
3.5.4.25|g__Streptococcus.s__Streptococcus_lutetiensis	+	3.59E-01	0.000	0.000	0.000	0.000	0.000
3.5.4.25|g__Streptococcus.s__Streptococcus_macedonicus	-	3.09E-01	0.000	0.000	0.000	0.000	0.000
3.5.4.25|g__Streptococcus.s__Streptococcus_pasteurianus	-	3.09E-01	0.000	0.000	0.000	0.000	0.000

	3.5.4.25|g__Sutterella.s__Sutterella_wadsworthensis	-	8.93E-01	0.000	0.000	0.000	0.003	0.003
	3.5.4.25|g__Veillonella.s__Veillonella_atypica	-	1.40E-01	0.000	0.000	0.000	0.000	0.000
	3.5.4.25|g__Veillonella.s__Veillonella_dispar	-	5.72E-01	0.000	0.000	0.000	0.000	0.000
	3.5.4.25|g__Veillonella.s__Veillonella_infantium	+	1.83E-01	0.000	0.000	0.000	0.000	0.000
	3.5.4.25|g__Veillonella.s__Veillonella_parvula	+	1.83E-01	0.000	0.000	0.000	0.000	0.000
	3.5.4.25|g__Veillonella.s__Veillonella_rogosae	-	9.81E-01	0.000	0.000	0.000	0.000	0.000
	3.5.4.25|g__Victivallales_unclassified.s__Victivallales_bacterium_CCUG_4	+	3.16E-01	0.000	0.000	0.000	0.001	0.001
	3.5.4.25|g__Victivallis.s__Victivallis_vadensis	+	1.87E-01	0.000	0.000	0.000	0.000	0.001
	3.5.4.25|g__Bacteroides.s__Bacteroides_xylanisolvens	+	8.40E-01	0.204	0.207	0.003	0.006	0.011
	3.5.4.25|g__Bacteroides.s__Bacteroides_thetaiotaomicron	-	4.16E-01	0.360	0.365	0.005	0.010	0.005
	3.5.4.25|g__Bacteroides.s__Bacteroides_dorei	-	5.39E-01	0.974	1.003	0.029	0.023	0.019
	3.5.4.25|g__Bacteroides.s__Bacteroides_caccae	-	5.96E-01	0.695	0.790	0.095	0.013	0.010
	3.5.4.25|g__Clostridium.s__Clostridium_sp_AM22_11AC	+	5.20E-01	0.000	0.127	0.127	0.002	0.003
	3.5.4.25|g__Alistipes.s__Alistipes_finegoldii	+	2.71E-01	0.665	0.827	0.162	0.018	0.024
	3.5.4.25|g__Roseburia.s__Roseburia_hominis	+	1.20E-01	0.450	0.646	0.196	0.006	0.011
	3.5.4.25|g__Barnesiella.s__Barnesiella_intestinihominis	+	5.24E-01	0.584	0.789	0.205	0.011	0.013
	3.5.4.25|g__Blautia.s__Ruminococcus_torques	+	5.74E-01	1.689	2.003	0.314	0.026	0.035
	3.5.4.25|g__Bilophila.s__Bilophila_wadsworthia	+	1.49E-01	0.000	0.321	0.321	0.002	0.003
	3.5.4.25|g__Anaerostipes.s__Anaerostipes_hadrus	+	2.14E-01	0.191	0.512	0.322	0.005	0.009
	3.5.4.25|g__Bacteroides.s__Bacteroides_cellulosilyticus	+	6.19E-01	0.369	0.700	0.331	0.012	0.013
	3.5.4.25|g__Clostridium.s__Clostridium_sp_AF36_4	+	1.50E-02	0.000	0.398	0.398	0.002	0.006
	3.5.4.25|g__Parabacteroides.s__Parabacteroides_merdae	+	6.39E-01	0.387	0.790	0.403	0.008	0.011
	3.5.4.25|g__Bacteroides.s__Bacteroides_vulgatus	-	8.38E-01	2.875	3.368	0.493	0.062	0.055
	3.5.4.25|g__Alistipes.s__Alistipes_onderdonkii	+	1.00E-02	0.175	0.879	0.705	0.010	0.021
	3.5.4.25|g__Alistipes.s__Alistipes_putredinis	+	1.69E-01	2.066	2.892	0.826	0.029	0.038
	3.5.4.25|g__Akkermansia.s__Akkermansia_muciniphila	+	1.45E-03	0.000	3.139	3.139	0.013	0.072
	3.5.4.25|unclassified	+	9.82E-01	18.588	21.910	3.322	0.240	0.230
China1	3.5.4.25	-	5.29E-01	113.837	114.019	0.182	1.000	1.000
	3.5.4.25|g__Bacteroides.s__Bacteroides_vulgatus	-	1.19E-02	9.293	2.579	-6.714	0.136	0.061
	3.5.4.25|g__Bacteroides.s__Bacteroides_fragilis	-	1.11E-01	1.373	0.752	-0.621	0.025	0.018
	3.5.4.25|g__Bacteroides.s__Bacteroides_ovatus	-	9.13E-02	1.894	1.411	-0.483	0.052	0.024
	3.5.4.25|g__Bacteroides.s__Bacteroides_xylanisolvens	-	2.55E-01	1.431	0.966	-0.465	0.022	0.013
	3.5.4.25|g__Escherichia.s__Escherichia_coli	-	6.00E-01	0.376	0.172	-0.204	0.022	0.026
	3.5.4.25|g__Faecalibacterium.s__Faecalibacterium_prausnitzii	+	7.69E-01	1.023	0.878	-0.145	0.016	0.018
	3.5.4.25|g__Klebsiella.s__Klebsiella_oxytoca	-	3.78E-01	0.430	0.317	-0.113	0.006	0.007
	3.5.4.25|g__Roseburia.s__Roseburia_faecis	-	6.27E-01	0.183	0.117	-0.067	0.004	0.004
	3.5.4.25|g__Bacteroides.s__Bacteroides_nordii	-	7.93E-01	0.101	0.084	-0.017	0.003	0.005
	3.5.4.25|g__Dorea.s__Dorea_longicatena	-	9.49E-01	0.076	0.061	-0.015	0.001	0.001
	3.5.4.25|g__Acidaminococcus.s__Acidaminococcus_intestini	+	3.30E-01	0.000	0.000	0.000	0.000	0.000
	3.5.4.25|g__Adlercreutzia.s__Adlercreutzia_equolifaciens	+	3.30E-01	0.000	0.000	0.000	0.000	0.000
	3.5.4.25|g__Aeromonas.s__Aeromonas_allosaccharophila	-	3.30E-01	0.000	0.000	0.000	0.000	0.000
	3.5.4.25|g__Aeromonas.s__Aeromonas_dhakensis	-	3.30E-01	0.000	0.000	0.000	0.000	0.000
	3.5.4.25|g__Aeromonas.s__Aeromonas_diversa	-	3.30E-01	0.000	0.000	0.000	0.000	0.000
	3.5.4.25|g__Aeromonas.s__Aeromonas_hydrophila	-	3.30E-01	0.000	0.000	0.000	0.000	0.000
	3.5.4.25|g__Agathobaculum.s__Agathobaculum_butyriciproducens	-	7.03E-01	0.000	0.000	0.000	0.001	0.000
	3.5.4.25|g__Aggregatibacter.s__Aggregatibacter_segnis	-	1.00E+00	0.000	0.000	0.000	0.000	0.000
	3.5.4.25|g__Akkermansia.s__Akkermansia_muciniphila	+	9.33E-03	0.000	0.000	0.000	0.001	0.005
	3.5.4.25|g__Alistipes.s__Alistipes_inops	-	9.66E-01	0.000	0.000	0.000	0.002	0.001
	3.5.4.25|g__Alistipes.s__Alistipes_sp_An66	+	5.49E-01	0.000	0.000	0.000	0.000	0.000
	3.5.4.25|g__Alistipes.s__Alistipes_timonensis	+	6.55E-01	0.000	0.000	0.000	0.001	0.001
	3.5.4.25|g__Anaeroglobus.s__Anaeroglobus_geminatus	+	3.30E-01	0.000	0.000	0.000	0.000	0.000
	3.5.4.25|g__Anaeromassilibacillus.s__Anaeromassilibacillus_sp_An250	+	3.30E-01	0.000	0.000	0.000	0.000	0.000
	3.5.4.25|g__Anaerostipes.s__Anaerostipes_caccae	-	1.60E-01	0.000	0.000	0.000	0.000	0.000
	3.5.4.25|g__Anaerostipes.s__Anaerostipes_hadrus	-	7.04E-01	0.000	0.000	0.000	0.001	0.001
	3.5.4.25|g__Anaerotignum.s__Anaerotignum_lactatifermentans	-	3.30E-01	0.000	0.000	0.000	0.000	0.000
	3.5.4.25|g__Asaccharobacter.s__Asaccharobacter_celatus	+	3.30E-01	0.000	0.000	0.000	0.000	0.000
	3.5.4.25|g__Bacteroides.s__Bacteroides_clarus	+	2.16E-01	0.000	0.000	0.000	0.001	0.005
	3.5.4.25|g__Bacteroides.s__Bacteroides_coprocola	-	6.84E-02	0.000	0.000	0.000	0.020	0.003
	3.5.4.25|g__Bacteroides.s__Bacteroides_coprophilus	-	4.05E-01	0.000	0.000	0.000	0.005	0.001
	3.5.4.25|g__Bacteroides.s__Bacteroides_eggerthii	+	1.09E-01	0.000	0.000	0.000	0.017	0.030
	3.5.4.25|g__Bacteroides.s__Bacteroides_faecichinchillae	+	4.28E-02	0.000	0.000	0.000	0.000	0.000
	3.5.4.25|g__Bacteroides.s__Bacteroides_faecis	-	1.58E-01	0.000	0.000	0.000	0.001	0.000
	3.5.4.25|g__Bacteroides.s__Bacteroides_finegoldii	+	3.16E-01	0.000	0.000	0.000	0.008	0.012
	3.5.4.25|g__Bacteroides.s__Bacteroides_fluxus	+	3.30E-01	0.000	0.000	0.000	0.000	0.000
	3.5.4.25|g__Bacteroides.s__Bacteroides_massiliensis	+	7.11E-01	0.000	0.000	0.000	0.008	0.012
	3.5.4.25|g__Bacteroides.s__Bacteroides_oleiciplenus	+	9.90E-01	0.000	0.000	0.000	0.000	0.000
	3.5.4.25|g__Bacteroides.s__Bacteroides_plebeius	-	3.05E-01	0.000	0.000	0.000	0.054	0.018
	3.5.4.25|g__Bacteroides.s__Bacteroides_salyersiae	-	7.77E-01	0.000	0.000	0.000	0.001	0.001
	3.5.4.25|g__Bacteroides.s__Bacteroides_sp_OM08_11	+	1.60E-01	0.000	0.000	0.000	0.000	0.000
	3.5.4.25|g__Bacteroides.s__Bacteroides_stercorirosoris	+	9.70E-01	0.000	0.000	0.000	0.000	0.000
	3.5.4.25|g__Barnesiella.s__Barnesiella_intestinihominis	+	2.79E-01	0.000	0.000	0.000	0.006	0.005
	3.5.4.25|g__Barnesiella.s__Barnesiella_sp_An22	-	3.30E-01	0.000	0.000	0.000	0.000	0.000

3.5.4.25|g__Blautia.s__Blautia_hansenii	+	3.30E-01	0.000	0.000	0.000	0.000	0.000
3.5.4.25|g__Blautia.s__Blautia_sp_AF19_10LB	+	9.63E-01	0.000	0.000	0.000	0.000	0.001
3.5.4.25|g__Butyricimonas.s__Butyricimonas_synergistica	-	1.00E+00	0.000	0.000	0.000	0.000	0.000
3.5.4.25|g__Butyricimonas.s__Butyricimonas_virosa	+	9.69E-01	0.000	0.000	0.000	0.002	0.002
3.5.4.25|g__Butyrivibrio.s__Butyrivibrio_crossotus	+	5.69E-01	0.000	0.000	0.000	0.000	0.000
3.5.4.25|g__Campylobacter.s__Campylobacter_concisus	-	3.30E-01	0.000	0.000	0.000	0.000	0.000
3.5.4.25|g__Campylobacter.s__Campylobacter_gracilis	+	3.30E-01	0.000	0.000	0.000	0.000	0.000
3.5.4.25|g__Catenibacterium.s__Catenibacterium_mitsuokai	+	6.06E-01	0.000	0.000	0.000	0.001	0.003
3.5.4.25|g__Citrobacter.s__Citrobacter_amalonaticus	+	1.60E-01	0.000	0.000	0.000	0.000	0.000
3.5.4.25|g__Citrobacter.s__Citrobacter_braakii	+	3.30E-01	0.000	0.000	0.000	0.000	0.000
3.5.4.25|g__Citrobacter.s__Citrobacter_farmeri	+	1.00E+00	0.000	0.000	0.000	0.000	0.000
3.5.4.25|g__Citrobacter.s__Citrobacter_portucalensis	+	1.58E-01	0.000	0.000	0.000	0.000	0.000
3.5.4.25|g__Citrobacter.s__Citrobacter_youngae	+	6.88E-01	0.000	0.000	0.000	0.000	0.000
3.5.4.25|g__Cloacibacillus.s__Cloacibacillus_porcorum	+	1.60E-01	0.000	0.000	0.000	0.000	0.000
3.5.4.25|g__Clostridioides.s__Clostridioides_difficile	+	8.53E-01	0.000	0.000	0.000	0.001	0.000
3.5.4.25|g__Clostridium.s__Clostridium_neonatale	-	3.30E-01	0.000	0.000	0.000	0.000	0.000
3.5.4.25|g__Clostridium.s__Clostridium_perfringens	-	5.69E-01	0.000	0.000	0.000	0.000	0.000
3.5.4.25|g__Clostridium.s__Clostridium_sp_AF36_4	+	6.43E-02	0.000	0.000	0.000	0.001	0.004
3.5.4.25|g__Clostridium.s__Clostridium_sp_AM22_11AC	-	5.67E-02	0.000	0.000	0.000	0.001	0.000
3.5.4.25|g__Clostridium.s__Clostridium_sp_chh4_2	+	3.30E-01	0.000	0.000	0.000	0.000	0.000
3.5.4.25|g__Coprobacillus.s__Coprobacillus_cateniformis	+	2.81E-01	0.000	0.000	0.000	0.000	0.000
3.5.4.25|g__Coprobacter.s__Coprobacter_fastidiosus	+	7.10E-01	0.000	0.000	0.000	0.001	0.001
3.5.4.25|g__Coprobacter.s__Coprobacter_secundus	-	7.91E-01	0.000	0.000	0.000	0.000	0.000
3.5.4.25|g__Coprococcus.s__Coprococcus_catus	+	2.07E-01	0.000	0.000	0.000	0.000	0.000
3.5.4.25|g__Coprococcus.s__Coprococcus_comes	+	9.36E-02	0.000	0.000	0.000	0.001	0.001
3.5.4.25|g__Coprococcus.s__Coprococcus_eutactus	+	3.55E-01	0.000	0.000	0.000	0.001	0.002
3.5.4.25|g__Desulfovibrio.s__Desulfovibrio_piger	+	2.35E-01	0.000	0.000	0.000	0.000	0.000
3.5.4.25|g__Desulfovibrio.s__Desulfovibrio_sp_AM18_2	+	8.20E-02	0.000	0.000	0.000	0.000	0.001
3.5.4.25|g__Desulfovibrionaceae_unclassified.s__Desulfovibrionaceae_bact	+	1.60E-01	0.000	0.000	0.000	0.000	0.000
3.5.4.25|g__Dialister.s__Dialister_invisus	+	8.52E-01	0.000	0.000	0.000	0.003	0.008
3.5.4.25|g__Dialister.s__Dialister_succinatiphilus	+	1.00E+00	0.000	0.000	0.000	0.000	0.000
3.5.4.25|g__Dorea.s__Dorea_formicigenerans	-	2.75E-01	0.000	0.000	0.000	0.001	0.000
3.5.4.25|g__Eggerthella.s__Eggerthella_lenta	+	6.87E-01	0.000	0.000	0.000	0.000	0.000
3.5.4.25|g__Enterobacter.s__Enterobacter_cloacae	+	9.90E-01	0.000	0.000	0.000	0.000	0.000
3.5.4.25|g__Enterococcus.s__Enterococcus_faecium	+	3.30E-01	0.000	0.000	0.000	0.000	0.001
3.5.4.25|g__Enterococcus.s__Enterococcus_hirae	+	3.30E-01	0.000	0.000	0.000	0.000	0.000
3.5.4.25|g__Erysipelatoclostridium.s__Clostridium_innocuum	+	3.30E-01	0.000	0.000	0.000	0.000	0.000
3.5.4.25|g__Erysipelatoclostridium.s__Clostridium_spiroforme	+	5.49E-01	0.000	0.000	0.000	0.000	0.000
3.5.4.25|g__Erysipelotrichaceae_unclassified.s__Erysipelotrichaceae_bacteri	-	3.30E-01	0.000	0.000	0.000	0.000	0.000
3.5.4.25|g__Eubacterium.s__Eubacterium_ramulus	+	4.05E-01	0.000	0.000	0.000	0.000	0.000
3.5.4.25|g__Eubacterium.s__Eubacterium_sp_AF17_7	+	6.44E-02	0.000	0.000	0.000	0.000	0.001
3.5.4.25|g__Eubacterium.s__Eubacterium_sp_AM18_10LB_B	+	1.00E+00	0.000	0.000	0.000	0.000	0.001
3.5.4.25|g__Faecalitalea.s__Faecalitalea_cylindroides	+	3.30E-01	0.000	0.000	0.000	0.000	0.000
3.5.4.25|g__Firmicutes_unclassified.s__Firmicutes_bacterium_AM10_47	-	5.89E-01	0.000	0.000	0.000	0.000	0.000
3.5.4.25|g__Flavonifractor.s__Flavonifractor_plautii	+	4.28E-02	0.000	0.000	0.000	0.000	0.000
3.5.4.25|g__Fusobacterium.s__Fusobacterium_hwasookii	-	3.30E-01	0.000	0.000	0.000	0.000	0.000
3.5.4.25|g__Fusobacterium.s__Fusobacterium_mortiferum	+	6.55E-01	0.000	0.000	0.000	0.001	0.000
3.5.4.25|g__Fusobacterium.s__Fusobacterium_ulcerans	-	6.71E-01	0.000	0.000	0.000	0.001	0.001
3.5.4.25|g__Fusobacterium.s__Fusobacterium_varium	-	1.60E-01	0.000	0.000	0.000	0.001	0.000
3.5.4.25|g__Haemophilus.s__Haemophilus_parainfluenzae	+	9.73E-01	0.000	0.000	0.000	0.001	0.001
3.5.4.25|g__Haemophilus.s__Haemophilus_paraphrohaemolyticus	+	3.30E-01	0.000	0.000	0.000	0.000	0.000
3.5.4.25|g__Haemophilus.s__Haemophilus_sputorum	+	1.00E+00	0.000	0.000	0.000	0.000	0.000
3.5.4.25|g__Holdemanella.s__Holdemanella_biformis	+	1.60E-01	0.000	0.000	0.000	0.000	0.000
3.5.4.25|g__Hungatella.s__Hungatella_hathewayi	+	3.11E-01	0.000	0.000	0.000	0.001	0.000
3.5.4.25|g__Intestinibacter.s__Intestinibacter_bartlettii	-	9.83E-01	0.000	0.000	0.000	0.000	0.000
3.5.4.25|g__Klebsiella.s__Klebsiella_aerogenes	+	9.73E-01	0.000	0.000	0.000	0.000	0.000
3.5.4.25|g__Klebsiella.s__Klebsiella_pneumoniae	+	4.49E-01	0.000	0.000	0.000	0.000	0.002
3.5.4.25|g__Klebsiella.s__Klebsiella_variicola	+	8.20E-02	0.000	0.000	0.000	0.000	0.000
3.5.4.25|g__Lachnoclostridium.s__Clostridium_aldenense	-	4.74E-01	0.000	0.000	0.000	0.000	0.000
3.5.4.25|g__Lachnoclostridium.s__Clostridium_bolteae	+	5.41E-01	0.000	0.000	0.000	0.001	0.001
3.5.4.25|g__Lachnoclostridium.s__Clostridium_citroniae	+	3.33E-01	0.000	0.000	0.000	0.000	0.000
3.5.4.25|g__Lachnoclostridium.s__Clostridium_clostridioforme	+	8.63E-01	0.000	0.000	0.000	0.000	0.000
3.5.4.25|g__Lachnoclostridium.s__Clostridium_symbiosum	-	7.54E-01	0.000	0.000	0.000	0.000	0.000
3.5.4.25|g__Lachnospira.s__Lachnospira_pectinoschiza	-	3.88E-01	0.000	0.000	0.000	0.002	0.002
3.5.4.25|g__Lactobacillus.s__Lactobacillus_crispatus	-	1.60E-01	0.000	0.000	0.000	0.000	0.000
3.5.4.25|g__Lactobacillus.s__Lactobacillus_delbrueckii	+	3.30E-01	0.000	0.000	0.000	0.000	0.000
3.5.4.25|g__Lactobacillus.s__Lactobacillus_fermentum	+	1.00E+00	0.000	0.000	0.000	0.000	0.000
3.5.4.25|g__Lactobacillus.s__Lactobacillus_rogosae	-	4.07E-01	0.000	0.000	0.000	0.003	0.002
3.5.4.25|g__Megamonas.s__Megamonas_funiformis	+	7.36E-01	0.000	0.000	0.000	0.004	0.002
3.5.4.25|g__Megamonas.s__Megamonas_rupellensis	-	9.47E-01	0.000	0.000	0.000	0.001	0.000
3.5.4.25|g__Megasphaera.s__Megasphaera_micronuciformis	-	3.30E-01	0.000	0.000	0.000	0.000	0.000
3.5.4.25|g__Obesumbacterium.s__Obesumbacterium_proteus	-	3.30E-01	0.000	0.000	0.000	0.000	0.000

	3.5.4.25|g__Parabacteroides.s__Parabacteroides_goldsteinii	+	1.31E-01	0.000	0.000	0.000	0.001	0.003
	3.5.4.25|g__Parabacteroides.s__Parabacteroides_gordonii	+	3.23E-01	0.000	0.000	0.000	0.000	0.001
	3.5.4.25|g__Parabacteroides.s__Parabacteroides_johnsonii	+	6.61E-02	0.000	0.000	0.000	0.001	0.003
	3.5.4.25|g__Paraprevotella.s__Paraprevotella_clara	+	5.45E-03	0.000	0.000	0.000	0.001	0.005
	3.5.4.25|g__Paraprevotella.s__Paraprevotella_xylaniphila	+	9.90E-01	0.000	0.000	0.000	0.000	0.001
	3.5.4.25|g__Peptostreptococcaceae_unclassified.s__Clostridium_hiranonis	-	3.30E-01	0.000	0.000	0.000	0.000	0.000
	3.5.4.25|g__Phascolarctobacterium.s__Phascolarctobacterium_succinatutens	+	2.97E-01	0.000	0.000	0.000	0.001	0.002
	3.5.4.25|g__Prevotella.s__Prevotella_bivia	+	3.30E-01	0.000	0.000	0.000	0.000	0.000
	3.5.4.25|g__Prevotella.s__Prevotella_copri	+	6.80E-01	0.000	0.000	0.000	0.011	0.013
	3.5.4.25|g__Prevotella.s__Prevotella_multisaccharivorax	-	3.30E-01	0.000	0.000	0.000	0.000	0.000
	3.5.4.25|g__Prevotella.s__Prevotella_sp_109	-	8.20E-02	0.000	0.000	0.000	0.001	0.000
	3.5.4.25|g__Prevotella.s__Prevotella_sp_AM42_24	-	1.60E-01	0.000	0.000	0.000	0.002	0.000
	3.5.4.25|g__Prevotella.s__Prevotella_stercorea	-	7.31E-01	0.000	0.000	0.000	0.008	0.005
	3.5.4.25|g__Proteus.s__Proteus_mirabilis	+	3.30E-01	0.000	0.000	0.000	0.000	0.000
	3.5.4.25|g__Pseudocitrobacter.s__Pseudocitrobacter_faecalis	+	3.30E-01	0.000	0.000	0.000	0.000	0.000
	3.5.4.25|g__Pyramidobacter.s__Pyramidobacter_piscolens	+	5.28E-02	0.000	0.000	0.000	0.000	0.000
	3.5.4.25|g__Pyramidobacter.s__Pyramidobacter_sp_C12_8	+	1.60E-01	0.000	0.000	0.000	0.000	0.000
	3.5.4.25|g__Raoultella.s__Raoultella_ornithinolytica	-	3.30E-01	0.000	0.000	0.000	0.000	0.000
	3.5.4.25|g__Roseburia.s__Roseburia_intestinalis	-	4.99E-01	0.000	0.000	0.000	0.001	0.001
	3.5.4.25|g__Roseburia.s__Roseburia_inulinivorans	+	2.28E-01	0.000	0.000	0.000	0.001	0.001
	3.5.4.25|g__Ruminococcaceae_unclassified.s__Ruminococcaceae_bacteriun	-	1.60E-01	0.000	0.000	0.000	0.000	0.000
	3.5.4.25|g__Ruminococcaceae_unclassified.s__Ruminococcaceae_bacteriun	+	5.56E-02	0.000	0.000	0.000	0.000	0.001
	3.5.4.25|g__Ruminococcus.s__Ruminococcus_callidus	+	9.07E-01	0.000	0.000	0.000	0.000	0.001
	3.5.4.25|g__Ruminococcus.s__Ruminococcus_sp_AF31_8BH	-	7.99E-01	0.000	0.000	0.000	0.000	0.000
	3.5.4.25|g__Sanguibacteroides.s__Sanguibacteroides_justesenii	+	2.96E-01	0.000	0.000	0.000	0.000	0.000
	3.5.4.25|g__Senegalimassilia.s__Senegalimassilia_anaerobia	+	6.38E-01	0.000	0.000	0.000	0.000	0.000
	3.5.4.25|g__Slackia.s__Slackia_isoflavoniconvertens	+	5.69E-01	0.000	0.000	0.000	0.000	0.000
	3.5.4.25|g__Streptococcus.s__Streptococcus_mitis	+	3.30E-01	0.000	0.000	0.000	0.000	0.000
	3.5.4.25|g__Streptococcus.s__Streptococcus_oralis	+	3.30E-01	0.000	0.000	0.000	0.000	0.000
	3.5.4.25|g__Streptococcus.s__Streptococcus_pneumoniae	+	3.30E-01	0.000	0.000	0.000	0.000	0.000
	3.5.4.25|g__Streptococcus.s__Streptococcus_pseudopneumoniae	+	3.30E-01	0.000	0.000	0.000	0.000	0.000
	3.5.4.25|g__Succinatimonas.s__Succinatimonas_hippei	-	3.30E-01	0.000	0.000	0.000	0.000	0.000
	3.5.4.25|g__Sutterella.s__Sutterella_wadsworthensis	+	3.71E-01	0.000	0.000	0.000	0.000	0.001
	3.5.4.25|g__Synergistes.s__Synergistes_jonesii	+	3.30E-01	0.000	0.000	0.000	0.000	0.000
	3.5.4.25|g__Veillonella.s__Veillonella_atypica	+	4.41E-01	0.000	0.000	0.000	0.000	0.001
	3.5.4.25|g__Veillonella.s__Veillonella_dispar	+	6.87E-01	0.000	0.000	0.000	0.005	0.001
	3.5.4.25|g__Veillonella.s__Veillonella_infantium	+	6.48E-01	0.000	0.000	0.000	0.001	0.000
	3.5.4.25|g__Veillonella.s__Veillonella_parvula	+	1.38E-01	0.000	0.000	0.000	0.001	0.001
	3.5.4.25|g__Veillonella.s__Veillonella_rogosae	+	1.05E-01	0.000	0.000	0.000	0.001	0.000
	3.5.4.25|g__Veillonella.s__Veillonella_tobetsuensis	+	4.05E-01	0.000	0.000	0.000	0.000	0.000
	3.5.4.25|g__Victivallales_unclassified.s__Victivallales_bacterium_CCUG_4	+	2.96E-01	0.000	0.000	0.000	0.000	0.001
	3.5.4.25|g__Victivallis.s__Victivallis_vadensis	+	1.00E+00	0.000	0.000	0.000	0.000	0.000
	3.5.4.25|g__Blautia.s__Blautia_wexlerae	-	9.15E-01	0.008	0.012	0.004	0.000	0.000
	3.5.4.25|g__Roseburia.s__Roseburia_hominis	+	1.44E-01	0.190	0.199	0.009	0.003	0.005
	3.5.4.25|g__Blautia.s__Blautia_obeum	+	4.35E-01	0.056	0.082	0.026	0.001	0.001
	3.5.4.25|g__Lachnospiraceae_unclassified.s__Eubacterium_rectale	-	9.08E-01	0.106	0.134	0.028	0.004	0.003
	3.5.4.25|g__Bacteroides.s__Bacteroides_cellulosilyticus	+	7.47E-01	0.645	0.690	0.046	0.015	0.022
	3.5.4.25|g__Alistipes.s__Alistipes_indistinctus	+	5.11E-03	0.000	0.051	0.051	0.002	0.003
	3.5.4.25|g__Parabacteroides.s__Parabacteroides_distasonis	+	5.60E-01	1.069	1.135	0.066	0.023	0.018
	3.5.4.25|g__Bilophila.s__Bilophila_wadsworthia	+	1.76E-01	0.029	0.111	0.082	0.001	0.002
	3.5.4.25|g__Bacteroides.s__Bacteroides_dorei	+	8.81E-01	0.334	0.449	0.115	0.049	0.032
	3.5.4.25|g__Alistipes.s__Alistipes_onderdonkii	+	2.29E-01	0.029	0.189	0.160	0.006	0.009
	3.5.4.25|g__Blautia.s__Ruminococcus_torques	+	5.25E-01	0.284	0.468	0.183	0.005	0.007
	3.5.4.25|g__Phascolarctobacterium.s__Phascolarctobacterium_faecium	+	4.11E-01	0.496	0.684	0.188	0.014	0.015
	3.5.4.25|g__Odoribacter.s__Odoribacter_splanchnicus	-	8.67E-01	0.031	0.289	0.259	0.005	0.004
	3.5.4.25|g__Bacteroides.s__Bacteroides_thetaiotaomicron	+	8.06E-01	1.244	1.608	0.365	0.023	0.031
	3.5.4.25|g__Parabacteroides.s__Parabacteroides_merdae	+	2.54E-01	0.103	0.475	0.371	0.009	0.012
	3.5.4.25|g__Eubacterium.s__Eubacterium_eligens	+	9.16E-02	0.271	0.652	0.380	0.008	0.019
	3.5.4.25|g__Bacteroides.s__Bacteroides_caccae	+	5.04E-01	0.223	0.687	0.464	0.014	0.020
	3.5.4.25|g__Bacteroides.s__Bacteroides_stercoris	+	7.62E-01	0.330	0.835	0.504	0.058	0.036
	3.5.4.25|g__Alistipes.s__Alistipes_finegoldii	+	2.03E-02	0.123	0.760	0.637	0.006	0.034
	3.5.4.25|g__Bacteroides.s__Bacteroides_intestinalis	+	2.56E-02	0.045	0.782	0.737	0.005	0.017
	3.5.4.25|unclassified	+	6.34E-01	11.416	12.939	1.524	0.160	0.178
	3.5.4.25|g__Alistipes.s__Alistipes_putredinis	+	5.67E-02	0.910	4.164	3.254	0.032	0.055
	3.5.4.25|g__Bacteroides.s__Bacteroides_uniformis	+	1.76E-01	6.400	10.503	4.103	0.077	0.117
China2	3.5.4.25	-	1.87E-01	127.720	123.354	-4.366	1.000	1.000
	3.5.4.25|g__Bacteroides.s__Bacteroides_vulgatus	-	7.61E-02	9.970	5.243	-4.727	0.132	0.081
	3.5.4.25|g__Faecalibacterium.s__Faecalibacterium_prausnitzii	-	3.22E-01	7.177	4.545	-2.633	0.063	0.054
	3.5.4.25|g__Roseburia.s__Roseburia_faecis	-	1.18E-01	0.920	0.277	-0.643	0.021	0.010
	3.5.4.25|g__Phascolarctobacterium.s__Phascolarctobacterium_faecium	-	5.51E-01	0.732	0.210	-0.522	0.009	0.009
	3.5.4.25|g__Bacteroides.s__Bacteroides_thetaiotaomicron	-	1.72E-01	0.517	0.267	-0.250	0.010	0.007
	3.5.4.25|g__Bacteroides.s__Bacteroides_dorei	-	1.52E-01	0.620	0.416	-0.204	0.046	0.025

3.5.4.25|g__Clostridium.s__Clostridium_sp_AM22_11AC	-	2.89E-01	0.530	0.330	-0.201	0.007	0.006
3.5.4.25|g__Parabacteroides.s__Parabacteroides_distasonis	+	5.35E-01	1.172	0.983	-0.188	0.013	0.020
3.5.4.25|g__Bacteroides.s__Bacteroides_xylanisolvens	-	8.82E-02	0.305	0.176	-0.129	0.011	0.005
3.5.4.25|g__Roseburia.s__Roseburia_intestinalis	-	7.16E-01	0.077	0.000	-0.077	0.005	0.003
3.5.4.25|g__Agathobaculum.s__Agathobaculum_butyriciproducens	-	1.32E-01	0.098	0.038	-0.060	0.002	0.001
3.5.4.25|g__Roseburia.s__Roseburia_hominis	+	7.07E-01	0.303	0.258	-0.046	0.003	0.005
3.5.4.25|g__Bacteroides.s__Bacteroides_cellulosilyticus	-	7.02E-01	0.077	0.038	-0.039	0.007	0.010
3.5.4.25|g__Veillonella.s__Veillonella_parvula	-	7.08E-01	0.012	0.000	-0.012	0.001	0.001
3.5.4.25|g__Bacteroides.s__Bacteroides_ovatus	+	9.04E-01	0.366	0.353	-0.012	0.020	0.015
3.5.4.25|g__Klebsiella.s__Klebsiella_pneumoniae	+	3.33E-01	0.094	0.089	-0.005	0.004	0.020
3.5.4.25|g__Lachnospiraceae_unclassified.s__Eubacterium_rectale	+	9.67E-01	0.160	0.158	-0.003	0.004	0.003
3.5.4.25|g__Acidaminococcus.s__Acidaminococcus_intestini	-	5.69E-01	0.000	0.000	0.000	0.001	0.001
3.5.4.25|g__Adlercreutzia.s__Adlercreutzia_equolifaciens	+	6.38E-01	0.000	0.000	0.000	0.000	0.000
3.5.4.25|g__Aggregatibacter.s__Aggregatibacter_segnis	-	3.30E-01	0.000	0.000	0.000	0.000	0.000
3.5.4.25|g__Akkermansia.s__Akkermansia_muciniphila	+	4.61E-01	0.000	0.000	0.000	0.002	0.007
3.5.4.25|g__Alcaligenes.s__Alcaligenes_faecalis	+	3.30E-01	0.000	0.000	0.000	0.000	0.001
3.5.4.25|g__Alistipes.s__Alistipes_indistinctus	+	2.36E-03	0.000	0.000	0.000	0.000	0.001
3.5.4.25|g__Alistipes.s__Alistipes_inops	+	5.89E-01	0.000	0.000	0.000	0.000	0.000
3.5.4.25|g__Alistipes.s__Alistipes_timonensis	+	1.00E+00	0.000	0.000	0.000	0.000	0.000
3.5.4.25|g__Allisonella.s__Allisonella_histaminiformans	+	3.93E-01	0.000	0.000	0.000	0.000	0.000
3.5.4.25|g__Anaeromassilibacillus.s__Anaeromassilibacillus_sp_An250	+	4.27E-02	0.000	0.000	0.000	0.000	0.000
3.5.4.25|g__Anaerotignum.s__Anaerotignum_lactatifermentans	-	1.60E-01	0.000	0.000	0.000	0.000	0.000
3.5.4.25|g__Asaccharobacter.s__Asaccharobacter_celatus	+	6.38E-01	0.000	0.000	0.000	0.000	0.000
3.5.4.25|g__Atlantibacter.s__Atlantibacter_hermannii	-	3.30E-01	0.000	0.000	0.000	0.000	0.000
3.5.4.25|g__Bacteroides.s__Bacteroides_clarus	-	5.86E-01	0.000	0.000	0.000	0.001	0.004
3.5.4.25|g__Bacteroides.s__Bacteroides_coprocola	-	6.15E-02	0.000	0.000	0.000	0.012	0.002
3.5.4.25|g__Bacteroides.s__Bacteroides_coprophilus	+	5.07E-01	0.000	0.000	0.000	0.004	0.004
3.5.4.25|g__Bacteroides.s__Bacteroides_eggerthii	-	2.91E-01	0.000	0.000	0.000	0.008	0.002
3.5.4.25|g__Bacteroides.s__Bacteroides_faecichinchillae	+	2.42E-01	0.000	0.000	0.000	0.000	0.000
3.5.4.25|g__Bacteroides.s__Bacteroides_faecis	+	4.05E-01	0.000	0.000	0.000	0.000	0.001
3.5.4.25|g__Bacteroides.s__Bacteroides_finegoldii	-	1.32E-01	0.000	0.000	0.000	0.010	0.001
3.5.4.25|g__Bacteroides.s__Bacteroides_fluxus	+	3.30E-01	0.000	0.000	0.000	0.000	0.000
3.5.4.25|g__Bacteroides.s__Bacteroides_massiliensis	-	6.38E-01	0.000	0.000	0.000	0.017	0.004
3.5.4.25|g__Bacteroides.s__Bacteroides_nordii	+	7.55E-02	0.000	0.000	0.000	0.001	0.001
3.5.4.25|g__Bacteroides.s__Bacteroides_oleiciplenus	-	1.60E-01	0.000	0.000	0.000	0.000	0.000
3.5.4.25|g__Bacteroides.s__Bacteroides_plebeius	-	3.60E-01	0.000	0.000	0.000	0.076	0.030
3.5.4.25|g__Bacteroides.s__Bacteroides_salyersiae	-	4.69E-01	0.000	0.000	0.000	0.001	0.001
3.5.4.25|g__Bacteroides.s__Bacteroides_sartorii	-	3.30E-01	0.000	0.000	0.000	0.000	0.000
3.5.4.25|g__Bacteroides.s__Bacteroides_sp_OM08_11	-	3.30E-01	0.000	0.000	0.000	0.000	0.000
3.5.4.25|g__Bacteroides.s__Bacteroides_stercorirosoris	+	5.89E-01	0.000	0.000	0.000	0.000	0.000
3.5.4.25|g__Barnesiella.s__Barnesiella_intestinihominis	+	6.22E-01	0.000	0.000	0.000	0.004	0.005
3.5.4.25|g__Blautia.s__Blautia_hansenii	-	1.75E-01	0.000	0.000	0.000	0.001	0.002
3.5.4.25|g__Blautia.s__Blautia_sp_AF19_10LB	+	3.06E-01	0.000	0.000	0.000	0.002	0.004
3.5.4.25|g__Butyricimonas.s__Butyricimonas_synergistica	+	7.47E-01	0.000	0.000	0.000	0.000	0.000
3.5.4.25|g__Butyricimonas.s__Butyricimonas_virosa	+	9.66E-02	0.000	0.000	0.000	0.001	0.002
3.5.4.25|g__Butyrivibrio.s__Butyrivibrio_crossotus	+	3.30E-01	0.000	0.000	0.000	0.000	0.007
3.5.4.25|g__Campylobacter.s__Campylobacter_concisus	-	7.17E-01	0.000	0.000	0.000	0.000	0.000
3.5.4.25|g__Campylobacter.s__Campylobacter_hominis	+	3.30E-01	0.000	0.000	0.000	0.000	0.000
3.5.4.25|g__Catenibacterium.s__Catenibacterium_mitsuokai	+	5.49E-01	0.000	0.000	0.000	0.000	0.000
3.5.4.25|g__Cetobacterium.s__Cetobacterium_somerae	-	3.30E-01	0.000	0.000	0.000	0.000	0.000
3.5.4.25|g__Chryseobacterium.s__Chryseobacterium_sp_VAUSW3	+	3.30E-01	0.000	0.000	0.000	0.000	0.000
3.5.4.25|g__Chryseobacterium.s__Chryseobacterium_sp_YLOS41	+	3.30E-01	0.000	0.000	0.000	0.000	0.000
3.5.4.25|g__Citrobacter.s__Citrobacter_amalonaticus	+	1.72E-01	0.000	0.000	0.000	0.000	0.001
3.5.4.25|g__Citrobacter.s__Citrobacter_braakii	+	6.38E-01	0.000	0.000	0.000	0.000	0.001
3.5.4.25|g__Citrobacter.s__Citrobacter_freundii	+	1.00E+00	0.000	0.000	0.000	0.000	0.000
3.5.4.25|g__Citrobacter.s__Citrobacter_portucalensis	-	7.73E-01	0.000	0.000	0.000	0.000	0.006
3.5.4.25|g__Citrobacter.s__Citrobacter_werkmanii	+	1.00E+00	0.000	0.000	0.000	0.000	0.001
3.5.4.25|g__Citrobacter.s__Citrobacter_youngae	-	7.92E-01	0.000	0.000	0.000	0.000	0.000
3.5.4.25|g__Cloacibacillus.s__Cloacibacillus_porcorum	+	3.30E-01	0.000	0.000	0.000	0.000	0.000
3.5.4.25|g__Clostridioides.s__Clostridioides_difficile	+	7.46E-02	0.000	0.000	0.000	0.001	0.001
3.5.4.25|g__Clostridium.s__Clostridium_butyricum	+	3.30E-01	0.000	0.000	0.000	0.000	0.000
3.5.4.25|g__Clostridium.s__Clostridium_celatum	+	3.30E-01	0.000	0.000	0.000	0.000	0.000
3.5.4.25|g__Clostridium.s__Clostridium_disporicum	-	6.48E-02	0.000	0.000	0.000	0.001	0.000
3.5.4.25|g__Clostridium.s__Clostridium_perfringens	-	4.27E-02	0.000	0.000	0.000	0.000	0.000
3.5.4.25|g__Clostridium.s__Clostridium_sp_AF36_4	+	5.45E-02	0.000	0.000	0.000	0.000	0.001
3.5.4.25|g__Clostridium.s__Clostridium_sp_chh4_2	+	3.30E-01	0.000	0.000	0.000	0.000	0.000
3.5.4.25|g__Coprobacillus.s__Coprobacillus_cateniformis	+	1.10E-02	0.000	0.000	0.000	0.000	0.002
3.5.4.25|g__Coprobacter.s__Coprobacter_fastidiosus	+	6.00E-01	0.000	0.000	0.000	0.001	0.001
3.5.4.25|g__Coprobacter.s__Coprobacter_secundus	+	9.73E-01	0.000	0.000	0.000	0.000	0.000
3.5.4.25|g__Coprococcus.s__Coprococcus_catus	+	2.88E-01	0.000	0.000	0.000	0.000	0.001
3.5.4.25|g__Coprococcus.s__Coprococcus_eutactus	+	1.29E-01	0.000	0.000	0.000	0.003	0.004
3.5.4.25|g__Cronobacter.s__Cronobacter_dublinensis	-	3.30E-01	0.000	0.000	0.000	0.000	0.000

3.5.4.25|g__Desulfovibrio.s__Desulfovibrio_fairfieldensis	+	1.00E+00	0.000	0.000	0.000	0.000	0.000
3.5.4.25|g__Desulfovibrio.s__Desulfovibrio_piger	+	3.22E-01	0.000	0.000	0.000	0.000	0.001
3.5.4.25|g__Desulfovibrio.s__Desulfovibrio_sp_AM18_2	+	8.19E-02	0.000	0.000	0.000	0.000	0.000
3.5.4.25|g__Desulfovibrionaceae_unclassified.s__Desulfovibrionaceae_bact	+	1.60E-01	0.000	0.000	0.000	0.000	0.000
3.5.4.25|g__Dialister.s__Dialister_invisus	-	7.66E-01	0.000	0.000	0.000	0.002	0.007
3.5.4.25|g__Dialister.s__Dialister_pneumosintes	+	3.30E-01	0.000	0.000	0.000	0.000	0.000
3.5.4.25|g__Dialister.s__Dialister_succinatiphilus	+	9.90E-01	0.000	0.000	0.000	0.002	0.003
3.5.4.25|g__Dorea.s__Dorea_sp_OM02_2LB	+	6.89E-01	0.000	0.000	0.000	0.000	0.000
3.5.4.25|g__Eggerthella.s__Eggerthella_lenta	+	4.46E-01	0.000	0.000	0.000	0.001	0.002
3.5.4.25|g__Enterobacter.s__Enterobacter_bugandensis	-	3.22E-01	0.000	0.000	0.000	0.000	0.000
3.5.4.25|g__Enterobacter.s__Enterobacter_cloacae	+	6.39E-01	0.000	0.000	0.000	0.000	0.000
3.5.4.25|g__Enterobacter.s__Enterobacter_mori	-	1.60E-01	0.000	0.000	0.000	0.000	0.000
3.5.4.25|g__Enterococcus.s__Enterococcus_hirae	+	3.30E-01	0.000	0.000	0.000	0.000	0.000
3.5.4.25|g__Erysipelatoclostridium.s__Clostridium_innocuum	+	6.72E-01	0.000	0.000	0.000	0.000	0.000
3.5.4.25|g__Erysipelatoclostridium.s__Clostridium_spiroforme	+	3.20E-01	0.000	0.000	0.000	0.000	0.001
3.5.4.25|g__Erysipelotrichaceae_unclassified.s__Erysipelotrichaceae_bacteri	-	1.60E-01	0.000	0.000	0.000	0.000	0.000
3.5.4.25|g__Escherichia.s__Escherichia_fergusonii	+	3.30E-01	0.000	0.000	0.000	0.000	0.000
3.5.4.25|g__Eubacterium.s__Eubacterium_ramulus	+	3.47E-01	0.000	0.000	0.000	0.000	0.001
3.5.4.25|g__Eubacterium.s__Eubacterium_sp_AF17_7	+	4.48E-01	0.000	0.000	0.000	0.000	0.000
3.5.4.25|g__Eubacterium.s__Eubacterium_sp_AM18_10LB_B	-	1.87E-01	0.000	0.000	0.000	0.000	0.000
3.5.4.25|g__Firmicutes_unclassified.s__Firmicutes_bacterium_AM10_47	-	8.41E-01	0.000	0.000	0.000	0.001	0.000
3.5.4.25|g__Flavonifractor.s__Flavonifractor_plautii	+	8.38E-02	0.000	0.000	0.000	0.000	0.000
3.5.4.25|g__Flavonifractor.s__Flavonifractor_sp_An10	+	3.30E-01	0.000	0.000	0.000	0.000	0.000
3.5.4.25|g__Fusobacterium.s__Fusobacterium_hwasookii	-	3.30E-01	0.000	0.000	0.000	0.000	0.000
3.5.4.25|g__Fusobacterium.s__Fusobacterium_mortiferum	-	6.89E-01	0.000	0.000	0.000	0.001	0.003
3.5.4.25|g__Fusobacterium.s__Fusobacterium_nucleatum	-	5.89E-01	0.000	0.000	0.000	0.000	0.000
3.5.4.25|g__Fusobacterium.s__Fusobacterium_periodonticum	-	3.08E-01	0.000	0.000	0.000	0.000	0.000
3.5.4.25|g__Fusobacterium.s__Fusobacterium_ulcerans	+	9.90E-01	0.000	0.000	0.000	0.000	0.000
3.5.4.25|g__Fusobacterium.s__Fusobacterium_varium	-	1.60E-01	0.000	0.000	0.000	0.000	0.000
3.5.4.25|g__Haemophilus.s__Haemophilus_haemolyticus	+	3.30E-01	0.000	0.000	0.000	0.000	0.000
3.5.4.25|g__Haemophilus.s__Haemophilus_influenzae	-	5.89E-01	0.000	0.000	0.000	0.000	0.000
3.5.4.25|g__Haemophilus.s__Haemophilus_parainfluenzae	-	8.66E-01	0.000	0.000	0.000	0.001	0.002
3.5.4.25|g__Haemophilus.s__Haemophilus_paraphrohaemolyticus	-	3.30E-01	0.000	0.000	0.000	0.000	0.000
3.5.4.25|g__Holdemanella.s__Holdemanella_biformis	+	1.87E-01	0.000	0.000	0.000	0.000	0.000
3.5.4.25|g__Hungatella.s__Hungatella_hathewayi	+	4.77E-01	0.000	0.000	0.000	0.000	0.000
3.5.4.25|g__Intestinibacter.s__Intestinibacter_bartlettii	+	2.55E-01	0.000	0.000	0.000	0.000	0.000
3.5.4.25|g__Klebsiella.s__Klebsiella_michiganensis	-	1.00E+00	0.000	0.000	0.000	0.000	0.000
3.5.4.25|g__Klebsiella.s__Klebsiella_variicola	+	2.93E-01	0.000	0.000	0.000	0.003	0.002
3.5.4.25|g__Kluyvera.s__Kluyvera_ascorbata	+	3.30E-01	0.000	0.000	0.000	0.000	0.000
3.5.4.25|g__Kluyvera.s__Kluyvera_cryocrescens	+	3.30E-01	0.000	0.000	0.000	0.000	0.000
3.5.4.25|g__Kluyvera.s__Kluyvera_georgiana	+	1.00E+00	0.000	0.000	0.000	0.000	0.000
3.5.4.25|g__Lachnoclostridium.s__Clostridium_aldenense	+	2.19E-01	0.000	0.000	0.000	0.000	0.000
3.5.4.25|g__Lachnoclostridium.s__Clostridium_citroniae	+	1.30E-02	0.000	0.000	0.000	0.000	0.000
3.5.4.25|g__Lachnoclostridium.s__Clostridium_clostridioforme	+	6.82E-01	0.000	0.000	0.000	0.001	0.000
3.5.4.25|g__Lachnoclostridium.s__Clostridium_symbiosum	+	1.59E-01	0.000	0.000	0.000	0.000	0.000
3.5.4.25|g__Lachnospira.s__Lachnospira_pectinoschiza	-	7.08E-01	0.000	0.000	0.000	0.006	0.003
3.5.4.25|g__Lactobacillus.s__Lactobacillus_crispatus	+	3.30E-01	0.000	0.000	0.000	0.000	0.000
3.5.4.25|g__Lactobacillus.s__Lactobacillus_fermentum	-	5.69E-01	0.000	0.000	0.000	0.000	0.000
3.5.4.25|g__Lactobacillus.s__Lactobacillus_reuteri	-	3.30E-01	0.000	0.000	0.000	0.000	0.000
3.5.4.25|g__Lactobacillus.s__Lactobacillus_rogosae	-	6.51E-01	0.000	0.000	0.000	0.009	0.005
3.5.4.25|g__Lactobacillus.s__Lactobacillus_zymae	-	3.30E-01	0.000	0.000	0.000	0.000	0.000
3.5.4.25|g__Leclercia.s__Leclercia_adecarboxylata	-	3.73E-01	0.000	0.000	0.000	0.000	0.000
3.5.4.25|g__Lelliottia.s__Lelliottia_amnigena	-	3.30E-01	0.000	0.000	0.000	0.000	0.000
3.5.4.25|g__Lelliottia.s__Lelliottia_nimipressuralis	-	1.00E+00	0.000	0.000	0.000	0.000	0.001
3.5.4.25|g__Megamonas.s__Megamonas_funiformis	+	9.77E-01	0.000	0.000	0.000	0.022	0.013
3.5.4.25|g__Megamonas.s__Megamonas_rupellensis	+	9.41E-01	0.000	0.000	0.000	0.011	0.004
3.5.4.25|g__Megasphaera.s__Megasphaera_elsdenii	+	3.30E-01	0.000	0.000	0.000	0.000	0.000
3.5.4.25|g__Megasphaera.s__Megasphaera_micronuciformis	+	9.90E-01	0.000	0.000	0.000	0.000	0.000
3.5.4.25|g__Megasphaera.s__Megasphaera_stantonii	+	3.30E-01	0.000	0.000	0.000	0.000	0.000
3.5.4.25|g__Mitsuokella.s__Mitsuokella_jalaludinii	+	3.30E-01	0.000	0.000	0.000	0.000	0.000
3.5.4.25|g__Mitsuokella.s__Mitsuokella_multacida	+	8.19E-02	0.000	0.000	0.000	0.000	0.001
3.5.4.25|g__Morganella.s__Morganella_morganii	+	1.00E+00	0.000	0.000	0.000	0.000	0.000
3.5.4.25|g__Pantoea.s__Pantoea_agglomerans	-	1.60E-01	0.000	0.000	0.000	0.000	0.000
3.5.4.25|g__Pantoea.s__Pantoea_dispersa	+	3.30E-01	0.000	0.000	0.000	0.000	0.000
3.5.4.25|g__Pantoea.s__Pantoea_sesami	-	6.87E-01	0.000	0.000	0.000	0.000	0.000
3.5.4.25|g__Parabacteroides.s__Parabacteroides_chinchillae	+	3.30E-01	0.000	0.000	0.000	0.000	0.000
3.5.4.25|g__Parabacteroides.s__Parabacteroides_goldsteinii	+	2.49E-01	0.000	0.000	0.000	0.000	0.001
3.5.4.25|g__Parabacteroides.s__Parabacteroides_gordonii	+	1.60E-01	0.000	0.000	0.000	0.000	0.001
3.5.4.25|g__Parabacteroides.s__Parabacteroides_johnsonii	+	6.92E-01	0.000	0.000	0.000	0.001	0.002
3.5.4.25|g__Paraprevotella.s__Paraprevotella_clara	+	9.37E-01	0.000	0.000	0.000	0.003	0.002
3.5.4.25|g__Paraprevotella.s__Paraprevotella_xylaniphila	-	3.30E-01	0.000	0.000	0.000	0.000	0.000
3.5.4.25|g__Pedobacter.s__Pedobacter_himalayensis	-	4.97E-01	0.000	0.000	0.000	0.000	0.000

	3.5.4.25|g__Phascolarctobacterium.s__Phascolarctobacterium_succinatutens	+	9.62E-01	0.000	0.000	0.000	0.002	0.002
	3.5.4.25|g__Prevotella.s__Prevotella_copri	+	2.71E-01	0.000	0.000	0.000	0.017	0.027
	3.5.4.25|g__Prevotella.s__Prevotella_corporis	+	1.60E-01	0.000	0.000	0.000	0.000	0.000
	3.5.4.25|g__Prevotella.s__Prevotella_sp_109	+	5.89E-01	0.000	0.000	0.000	0.003	0.003
	3.5.4.25|g__Prevotella.s__Prevotella_sp_AM42_24	-	9.90E-01	0.000	0.000	0.000	0.005	0.002
	3.5.4.25|g__Prevotella.s__Prevotella_stercorea	-	9.90E-01	0.000	0.000	0.000	0.008	0.005
	3.5.4.25|g__Proteus.s__Proteus_mirabilis	-	3.20E-01	0.000	0.000	0.000	0.000	0.000
	3.5.4.25|g__Proteus.s__Proteus_penneri	-	5.89E-01	0.000	0.000	0.000	0.000	0.000
	3.5.4.25|g__Proteus.s__Proteus_vulgaris	+	1.00E+00	0.000	0.000	0.000	0.000	0.000
	3.5.4.25|g__Providencia.s__Providencia_alcalifaciens	+	3.30E-01	0.000	0.000	0.000	0.000	0.000
	3.5.4.25|g__Providencia.s__Providencia_rettgeri	+	3.30E-01	0.000	0.000	0.000	0.000	0.005
	3.5.4.25|g__Pseudoflavonifractor.s__Pseudoflavonifractor_sp_An184	+	1.60E-01	0.000	0.000	0.000	0.000	0.000
	3.5.4.25|g__Pyramidobacter.s__Pyramidobacter_piscolens	+	1.58E-01	0.000	0.000	0.000	0.000	0.000
	3.5.4.25|g__Pyramidobacter.s__Pyramidobacter_sp_C12_8	+	3.30E-01	0.000	0.000	0.000	0.000	0.000
	3.5.4.25|g__Raoultella.s__Raoultella_ornithinolytica	-	1.00E+00	0.000	0.000	0.000	0.000	0.000
	3.5.4.25|g__Raoultella.s__Raoultella_planticola	+	1.60E-01	0.000	0.000	0.000	0.000	0.000
	3.5.4.25|g__Roseburia.s__Roseburia_inulinivorans	-	6.78E-01	0.000	0.000	0.000	0.001	0.001
	3.5.4.25|g__Ruminococcaceae_unclassified.s__Ruminococcaceae_bacteriun	+	5.89E-01	0.000	0.000	0.000	0.000	0.000
	3.5.4.25|g__Ruminococcaceae_unclassified.s__Ruminococcaceae_bacteriun	+	4.27E-02	0.000	0.000	0.000	0.000	0.000
	3.5.4.25|g__Ruminococcus.s__Ruminococcus_callidus	+	8.05E-01	0.000	0.000	0.000	0.001	0.001
	3.5.4.25|g__Ruminococcus.s__Ruminococcus_sp_AF31_8BH	-	6.30E-01	0.000	0.000	0.000	0.001	0.001
	3.5.4.25|g__Salmonella.s__Salmonella_enterica	-	3.30E-01	0.000	0.000	0.000	0.000	0.000
	3.5.4.25|g__Sanguibacteroides.s__Sanguibacteroides_justesenii	+	1.60E-01	0.000	0.000	0.000	0.000	0.000
	3.5.4.25|g__Staphylococcus.s__Staphylococcus_pasteuri	-	3.30E-01	0.000	0.000	0.000	0.000	0.000
	3.5.4.25|g__Staphylococcus.s__Staphylococcus_warneri	-	3.30E-01	0.000	0.000	0.000	0.000	0.000
	3.5.4.25|g__Streptococcus.s__Streptococcus_equinus	-	3.30E-01	0.000	0.000	0.000	0.000	0.000
	3.5.4.25|g__Streptococcus.s__Streptococcus_gallolyticus	+	3.30E-01	0.000	0.000	0.000	0.000	0.000
	3.5.4.25|g__Streptococcus.s__Streptococcus_infantarius	-	3.30E-01	0.000	0.000	0.000	0.000	0.000
	3.5.4.25|g__Streptococcus.s__Streptococcus_lutetiensis	-	3.30E-01	0.000	0.000	0.000	0.000	0.000
	3.5.4.25|g__Streptococcus.s__Streptococcus_macedonicus	-	1.00E+00	0.000	0.000	0.000	0.000	0.000
	3.5.4.25|g__Streptococcus.s__Streptococcus_mitis	-	1.60E-01	0.000	0.000	0.000	0.000	0.000
	3.5.4.25|g__Streptococcus.s__Streptococcus_oralis	-	1.60E-01	0.000	0.000	0.000	0.000	0.000
	3.5.4.25|g__Streptococcus.s__Streptococcus_pasteurianus	-	1.00E+00	0.000	0.000	0.000	0.000	0.000
	3.5.4.25|g__Streptococcus.s__Streptococcus_pneumoniae	-	8.36E-02	0.000	0.000	0.000	0.000	0.000
	3.5.4.25|g__Streptococcus.s__Streptococcus_pseudopneumoniae	-	3.30E-01	0.000	0.000	0.000	0.000	0.000
	3.5.4.25|g__Sutterella.s__Sutterella_wadsworthensis	+	7.46E-02	0.000	0.000	0.000	0.001	0.002
	3.5.4.25|g__Veillonella.s__Veillonella_atypica	-	7.18E-01	0.000	0.000	0.000	0.001	0.001
	3.5.4.25|g__Veillonella.s__Veillonella_dispar	-	8.05E-01	0.000	0.000	0.000	0.001	0.001
	3.5.4.25|g__Veillonella.s__Veillonella_infantium	-	4.53E-01	0.000	0.000	0.000	0.000	0.000
	3.5.4.25|g__Veillonella.s__Veillonella_rogosae	-	5.63E-03	0.000	0.000	0.000	0.001	0.000
	3.5.4.25|g__Veillonella.s__Veillonella_tobetsuensis	-	1.29E-01	0.000	0.000	0.000	0.000	0.000
	3.5.4.25|g__Victivallales_unclassified.s__Victivallales_bacterium_CCUG_4	+	6.62E-02	0.000	0.000	0.000	0.000	0.001
	3.5.4.25|g__Victivallis.s__Victivallis_vadensis	+	2.68E-01	0.000	0.000	0.000	0.000	0.001
	3.5.4.25|g__Weissella.s__Weissella_confusa	-	1.00E+00	0.000	0.000	0.000	0.000	0.000
	3.5.4.25|g__Yokenella.s__Yokenella_regensburgei	-	8.19E-02	0.000	0.000	0.000	0.000	0.000
	3.5.4.25|g__Anaerostipes.s__Anaerostipes_hadrus	-	7.65E-01	0.137	0.137	0.000	0.004	0.004
	3.5.4.25|g__Klebsiella.s__Klebsiella_aerogenes	+	3.68E-01	0.011	0.016	0.005	0.001	0.004
	3.5.4.25|g__Lachnoclostridium.s__Clostridium_bolteae	+	1.51E-01	0.000	0.023	0.023	0.000	0.001
	3.5.4.25|g__Bacteroides.s__Bacteroides_fragilis	-	9.75E-01	0.113	0.138	0.025	0.008	0.010
	3.5.4.25|g__Bacteroides.s__Bacteroides_intestinalis	+	4.66E-01	0.000	0.031	0.031	0.002	0.003
	3.5.4.25|g__Coprococcus.s__Coprococcus_comes	+	8.98E-01	0.000	0.036	0.036	0.001	0.001
	3.5.4.25|g__Dorea.s__Dorea_formicigenerans	+	4.73E-01	0.036	0.072	0.036	0.000	0.001
	3.5.4.25|g__Blautia.s__Blautia_wexlerae	+	9.28E-01	0.185	0.237	0.052	0.006	0.007
	3.5.4.25|g__Blautia.s__Blautia_obeum	+	9.92E-01	0.312	0.367	0.055	0.006	0.008
	3.5.4.25|g__Dorea.s__Dorea_longicatena	+	4.12E-01	0.168	0.230	0.062	0.002	0.002
	3.5.4.25|g__Parabacteroides.s__Parabacteroides_merdae	+	2.57E-01	0.447	0.572	0.125	0.007	0.013
	3.5.4.25|g__Klebsiella.s__Klebsiella_oxytoca	+	5.89E-01	0.285	0.451	0.166	0.006	0.007
	3.5.4.25|g__Blautia.s__Ruminococcus_torques	+	6.97E-01	0.748	0.978	0.230	0.011	0.012
	3.5.4.25|g__Eubacterium.s__Eubacterium_eligens	+	7.03E-01	0.000	0.253	0.253	0.007	0.005
	3.5.4.25|g__Odoribacter.s__Odoribacter_splanchnicus	+	1.79E-01	0.101	0.375	0.274	0.002	0.004
	3.5.4.25|g__Bilophila.s__Bilophila_wadsworthia	+	1.85E-03	0.091	0.385	0.294	0.001	0.004
	3.5.4.25|g__Alistipes.s__Alistipes_onderdonkii	+	3.23E-02	0.000	0.365	0.365	0.003	0.007
	3.5.4.25|g__Bacteroides.s__Bacteroides_caccae	+	4.04E-01	0.154	0.596	0.442	0.007	0.010
	3.5.4.25|g__Bacteroides.s__Bacteroides_stercoris	+	6.68E-01	0.359	0.835	0.476	0.040	0.041
	3.5.4.25|g__Alistipes.s__Alistipes_finegoldii	+	5.50E-02	0.111	0.664	0.553	0.006	0.011
	3.5.4.25|g__Escherichia.s__Escherichia_coli	+	2.30E-01	0.433	1.134	0.701	0.033	0.050
	3.5.4.25|g__Bacteroides.s__Bacteroides_uniformis	+	4.84E-01	3.090	4.091	1.002	0.050	0.069
	3.5.4.25|unclassified	+	2.99E-01	13.959	17.083	3.124	0.159	0.198
	3.5.4.25|g__Alistipes.s__Alistipes_putredinis	+	2.81E-03	0.149	3.308	3.158	0.014	0.039
Taiwan	3.5.4.25	-	1.88E-01	125.118	119.553	-5.565	1.000	1.000
	3.5.4.25|unclassified	-	9.78E-01	22.218	19.257	-2.961	0.231	0.243
	3.5.4.25|g__Bacteroides.s__Bacteroides_vulgatus	-	6.19E-01	7.690	5.431	-2.259	0.081	0.072

3.5.4.25|g__Bacteroides.s__Bacteroides_plebeius	-	4.80E-01	0.710	0.299	-0.410	0.065	0.043
3.5.4.25|g__Lachnospiraceae_unclassified.s__Eubacterium_rectale	-	3.22E-02	0.313	0.089	-0.224	0.010	0.004
3.5.4.25|g__Escherichia.s__Escherichia_coli	-	2.02E-01	0.272	0.055	-0.217	0.023	0.007
3.5.4.25|g__Agathobaculum.s__Agathobaculum_butyriciproducens	-	1.08E-01	0.168	0.000	-0.168	0.002	0.002
3.5.4.25|g__Coprococcus.s__Coprococcus_comes	-	1.72E-01	0.110	0.000	-0.110	0.002	0.001
3.5.4.25|g__Roseburia.s__Roseburia_faecis	-	4.93E-01	0.224	0.117	-0.106	0.005	0.005
3.5.4.25|g__Bacteroides.s__Bacteroides_xylanisolvens	+	9.40E-01	0.593	0.500	-0.092	0.016	0.015
3.5.4.25|g__Bilophila.s__Bilophila_wadsworthia	-	4.77E-01	0.207	0.130	-0.077	0.004	0.002
3.5.4.25|g__Clostridium.s__Clostridium_sp_AM22_11AC	-	2.92E-01	0.178	0.128	-0.050	0.003	0.002
3.5.4.25|g__Dorea.s__Dorea_longicatena	-	9.55E-01	0.137	0.105	-0.032	0.002	0.003
3.5.4.25|g__Blautia.s__Blautia_obeum	-	7.04E-01	0.102	0.077	-0.025	0.001	0.001
3.5.4.25|g__Acidaminococcus.s__Acidaminococcus_fermentans	+	1.10E-01	0.000	0.000	0.000	0.000	0.000
3.5.4.25|g__Acidaminococcus.s__Acidaminococcus_intestini	-	7.27E-01	0.000	0.000	0.000	0.001	0.003
3.5.4.25|g__Adlercreutzia.s__Adlercreutzia_equolifaciens	-	3.41E-01	0.000	0.000	0.000	0.000	0.000
3.5.4.25|g__Aeromonas.s__Aeromonas_caviae	-	2.85E-01	0.000	0.000	0.000	0.000	0.000
3.5.4.25|g__Aeromonas.s__Aeromonas_hydrophila	-	2.85E-01	0.000	0.000	0.000	0.000	0.000
3.5.4.25|g__Akkermansia.s__Akkermansia_muciniphila	+	9.52E-02	0.000	0.000	0.000	0.010	0.012
3.5.4.25|g__Alistipes.s__Alistipes_indistinctus	-	7.19E-01	0.000	0.000	0.000	0.002	0.004
3.5.4.25|g__Alistipes.s__Alistipes_inops	+	9.13E-01	0.000	0.000	0.000	0.001	0.001
3.5.4.25|g__Alistipes.s__Alistipes_timonensis	-	2.85E-01	0.000	0.000	0.000	0.000	0.000
3.5.4.25|g__Allisonella.s__Allisonella_histaminiformans	-	1.16E-01	0.000	0.000	0.000	0.000	0.000
3.5.4.25|g__Anaeromassilibacillus.s__Anaeromassilibacillus_sp_An250	-	8.57E-01	0.000	0.000	0.000	0.000	0.000
3.5.4.25|g__Anaerostipes.s__Anaerostipes_hadrus	-	9.18E-01	0.000	0.000	0.000	0.001	0.001
3.5.4.25|g__Anaerotignum.s__Anaerotignum_lactatifermentans	-	8.42E-01	0.000	0.000	0.000	0.001	0.001
3.5.4.25|g__Asaccharobacter.s__Asaccharobacter_celatus	-	3.34E-01	0.000	0.000	0.000	0.000	0.000
3.5.4.25|g__Bacteroides.s__Bacteroides_clarus	-	9.17E-01	0.000	0.000	0.000	0.002	0.007
3.5.4.25|g__Bacteroides.s__Bacteroides_coprocola	+	4.16E-01	0.000	0.000	0.000	0.009	0.011
3.5.4.25|g__Bacteroides.s__Bacteroides_coprophilus	-	6.83E-01	0.000	0.000	0.000	0.006	0.005
3.5.4.25|g__Bacteroides.s__Bacteroides_eggerthii	+	2.39E-01	0.000	0.000	0.000	0.007	0.008
3.5.4.25|g__Bacteroides.s__Bacteroides_faecichinchillae	+	3.43E-01	0.000	0.000	0.000	0.000	0.000
3.5.4.25|g__Bacteroides.s__Bacteroides_fluxus	-	9.26E-01	0.000	0.000	0.000	0.000	0.002
3.5.4.25|g__Bacteroides.s__Bacteroides_fragilis	+	3.21E-01	0.000	0.000	0.000	0.006	0.009
3.5.4.25|g__Bacteroides.s__Bacteroides_nordii	+	4.84E-01	0.000	0.000	0.000	0.001	0.001
3.5.4.25|g__Bacteroides.s__Bacteroides_oleiciplenus	+	3.68E-01	0.000	0.000	0.000	0.000	0.000
3.5.4.25|g__Bacteroides.s__Bacteroides_salyersiae	-	9.78E-01	0.000	0.000	0.000	0.005	0.004
3.5.4.25|g__Bacteroides.s__Bacteroides_sartorii	+	8.11E-01	0.000	0.000	0.000	0.005	0.001
3.5.4.25|g__Bacteroides.s__Bacteroides_sp_OM08_11	+	5.46E-01	0.000	0.000	0.000	0.000	0.000
3.5.4.25|g__Barnesiella.s__Barnesiella_intestinihominis	+	6.82E-02	0.000	0.000	0.000	0.005	0.009
3.5.4.25|g__Blautia.s__Blautia_hansenii	+	8.37E-01	0.000	0.000	0.000	0.000	0.000
3.5.4.25|g__Blautia.s__Blautia_sp_AF19_10LB	-	7.86E-01	0.000	0.000	0.000	0.000	0.001
3.5.4.25|g__Butyricicoccus.s__Butyricicoccus_pullicaecorum	-	2.85E-01	0.000	0.000	0.000	0.000	0.000
3.5.4.25|g__Butyricimonas.s__Butyricimonas_synergistica	-	8.72E-01	0.000	0.000	0.000	0.000	0.000
3.5.4.25|g__Campylobacter.s__Campylobacter_concisus	-	2.85E-01	0.000	0.000	0.000	0.000	0.000
3.5.4.25|g__Candidatus_Gastranaerophilales_unclassified.s__Candidatus_Ga	-	9.26E-01	0.000	0.000	0.000	0.000	0.000
3.5.4.25|g__Catenibacterium.s__Catenibacterium_mitsuokai	-	5.65E-01	0.000	0.000	0.000	0.004	0.005
3.5.4.25|g__Cetobacterium.s__Cetobacterium_somerae	+	1.96E-01	0.000	0.000	0.000	0.000	0.000
3.5.4.25|g__Citrobacter.s__Citrobacter_amalonaticus	-	2.34E-01	0.000	0.000	0.000	0.000	0.000
3.5.4.25|g__Citrobacter.s__Citrobacter_braakii	-	4.57E-01	0.000	0.000	0.000	0.000	0.000
3.5.4.25|g__Citrobacter.s__Citrobacter_farmeri	-	2.85E-01	0.000	0.000	0.000	0.000	0.000
3.5.4.25|g__Citrobacter.s__Citrobacter_freundii	+	3.68E-01	0.000	0.000	0.000	0.000	0.000
3.5.4.25|g__Citrobacter.s__Citrobacter_koseri	+	6.54E-01	0.000	0.000	0.000	0.000	0.000
3.5.4.25|g__Citrobacter.s__Citrobacter_portucalensis	+	9.07E-01	0.000	0.000	0.000	0.000	0.000
3.5.4.25|g__Citrobacter.s__Citrobacter_werkmanii	+	3.68E-01	0.000	0.000	0.000	0.000	0.000
3.5.4.25|g__Citrobacter.s__Citrobacter_youngae	-	7.84E-01	0.000	0.000	0.000	0.000	0.000
3.5.4.25|g__Cloacibacillus.s__Cloacibacillus_porcorum	+	3.70E-02	0.000	0.000	0.000	0.000	0.000
3.5.4.25|g__Clostridiales_unclassified.s__Clostridiales_bacterium_1_7_47F	-	9.05E-01	0.000	0.000	0.000	0.000	0.000
3.5.4.25|g__Clostridioides.s__Clostridioides_difficile	+	3.18E-01	0.000	0.000	0.000	0.000	0.002
3.5.4.25|g__Clostridium.s__Butyribacterium_methylotrophicum	-	9.05E-01	0.000	0.000	0.000	0.000	0.000
3.5.4.25|g__Clostridium.s__Clostridium_disporicum	+	6.70E-01	0.000	0.000	0.000	0.000	0.000
3.5.4.25|g__Clostridium.s__Clostridium_neonatale	+	3.68E-01	0.000	0.000	0.000	0.000	0.000
3.5.4.25|g__Clostridium.s__Clostridium_perfringens	-	1.25E-01	0.000	0.000	0.000	0.000	0.000
3.5.4.25|g__Clostridium.s__Clostridium_sp_AF36_4	+	8.17E-01	0.000	0.000	0.000	0.001	0.000
3.5.4.25|g__Clostridium.s__Clostridium_sp_AM58_1XD	-	2.85E-01	0.000	0.000	0.000	0.000	0.000
3.5.4.25|g__Coprobacillus.s__Coprobacillus_cateniformis	+	4.08E-01	0.000	0.000	0.000	0.000	0.000
3.5.4.25|g__Coprobacter.s__Coprobacter_fastidiosus	+	1.19E-01	0.000	0.000	0.000	0.001	0.003
3.5.4.25|g__Coprobacter.s__Coprobacter_secundus	+	2.52E-01	0.000	0.000	0.000	0.000	0.000
3.5.4.25|g__Coprococcus.s__Coprococcus_catus	-	3.70E-01	0.000	0.000	0.000	0.000	0.000
3.5.4.25|g__Coprococcus.s__Coprococcus_eutactus	+	6.62E-01	0.000	0.000	0.000	0.001	0.002
3.5.4.25|g__Desulfovibrio.s__Desulfovibrio_desulfuricans	+	3.68E-01	0.000	0.000	0.000	0.000	0.000
3.5.4.25|g__Desulfovibrio.s__Desulfovibrio_fairfieldensis	+	3.70E-02	0.000	0.000	0.000	0.000	0.000
3.5.4.25|g__Desulfovibrio.s__Desulfovibrio_legallii	+	3.68E-01	0.000	0.000	0.000	0.000	0.000
3.5.4.25|g__Desulfovibrio.s__Desulfovibrio_piger	+	7.57E-01	0.000	0.000	0.000	0.001	0.001

3.5.4.25|g__Desulfovibrio.s__Desulfovibrio_sp_AM18_2	+	3.68E-01	0.000	0.000	0.000	0.000	0.000
3.5.4.25|g__Desulfovibrionaceae_unclassified.s__Desulfovibrionaceae_bact	-	3.12E-01	0.000	0.000	0.000	0.000	0.000
3.5.4.25|g__Dialister.s__Dialister_invisus	-	2.42E-01	0.000	0.000	0.000	0.001	0.000
3.5.4.25|g__Dialister.s__Dialister_succinatiphilus	-	3.56E-01	0.000	0.000	0.000	0.002	0.001
3.5.4.25|g__Dorea.s__Dorea_formicigenerans	+	4.04E-01	0.000	0.000	0.000	0.000	0.001
3.5.4.25|g__Dorea.s__Dorea_sp_OM02_2LB	+	3.68E-01	0.000	0.000	0.000	0.000	0.000
3.5.4.25|g__Eggerthella.s__Eggerthella_lenta	+	5.42E-01	0.000	0.000	0.000	0.001	0.001
3.5.4.25|g__Enterobacter.s__Enterobacter_bugandensis	-	4.57E-01	0.000	0.000	0.000	0.000	0.000
3.5.4.25|g__Enterobacter.s__Enterobacter_cloacae	-	2.90E-01	0.000	0.000	0.000	0.000	0.000
3.5.4.25|g__Enterococcus.s__Enterococcus_faecium	+	3.68E-01	0.000	0.000	0.000	0.000	0.000
3.5.4.25|g__Erysipelatoclostridium.s__Clostridium_innocuum	+	1.10E-01	0.000	0.000	0.000	0.000	0.000
3.5.4.25|g__Erysipelatoclostridium.s__Clostridium_spiroforme	+	2.48E-01	0.000	0.000	0.000	0.000	0.000
3.5.4.25|g__Erysipelotrichaceae_unclassified.s__Erysipelotrichaceae_bacteri	-	2.85E-01	0.000	0.000	0.000	0.000	0.000
3.5.4.25|g__Eubacterium.s__Eubacterium_callanderi	-	9.05E-01	0.000	0.000	0.000	0.000	0.000
3.5.4.25|g__Eubacterium.s__Eubacterium_limosum	+	3.68E-01	0.000	0.000	0.000	0.000	0.000
3.5.4.25|g__Eubacterium.s__Eubacterium_ramulus	-	5.70E-01	0.000	0.000	0.000	0.000	0.000
3.5.4.25|g__Eubacterium.s__Eubacterium_sp_AF17_7	+	6.97E-01	0.000	0.000	0.000	0.000	0.001
3.5.4.25|g__Eubacterium.s__Eubacterium_sp_AM18_10LB_B	-	9.26E-01	0.000	0.000	0.000	0.000	0.000
3.5.4.25|g__Faecalitalea.s__Faecalitalea_cylindroides	-	9.26E-01	0.000	0.000	0.000	0.000	0.000
3.5.4.25|g__Firmicutes_unclassified.s__Firmicutes_bacterium_AM10_47	+	6.36E-02	0.000	0.000	0.000	0.000	0.000
3.5.4.25|g__Flavonifractor.s__Flavonifractor_plautii	+	3.19E-01	0.000	0.000	0.000	0.000	0.000
3.5.4.25|g__Flavonifractor.s__Flavonifractor_sp_An10	+	3.68E-01	0.000	0.000	0.000	0.000	0.000
3.5.4.25|g__Fusobacterium.s__Fusobacterium_mortiferum	-	3.03E-01	0.000	0.000	0.000	0.004	0.001
3.5.4.25|g__Fusobacterium.s__Fusobacterium_nucleatum	+	3.68E-01	0.000	0.000	0.000	0.000	0.000
3.5.4.25|g__Fusobacterium.s__Fusobacterium_ulcerans	+	2.28E-01	0.000	0.000	0.000	0.000	0.002
3.5.4.25|g__Fusobacterium.s__Fusobacterium_varium	-	2.42E-01	0.000	0.000	0.000	0.001	0.000
3.5.4.25|g__Haemophilus.s__Haemophilus_parainfluenzae	-	2.23E-01	0.000	0.000	0.000	0.002	0.000
3.5.4.25|g__Hafnia.s__Hafnia_paralvei	-	2.85E-01	0.000	0.000	0.000	0.000	0.000
3.5.4.25|g__Holdemanella.s__Holdemanella_biformis	+	3.68E-01	0.000	0.000	0.000	0.000	0.000
3.5.4.25|g__Hungatella.s__Hungatella_hathewayi	+	7.82E-01	0.000	0.000	0.000	0.001	0.001
3.5.4.25|g__Intestinibacter.s__Intestinibacter_bartlettii	+	3.68E-01	0.000	0.000	0.000	0.000	0.000
3.5.4.25|g__Klebsiella.s__Klebsiella_aerogenes	-	4.61E-01	0.000	0.000	0.000	0.001	0.001
3.5.4.25|g__Klebsiella.s__Klebsiella_michiganensis	-	9.05E-01	0.000	0.000	0.000	0.000	0.000
3.5.4.25|g__Klebsiella.s__Klebsiella_pneumoniae	-	3.83E-01	0.000	0.000	0.000	0.004	0.002
3.5.4.25|g__Klebsiella.s__Klebsiella_variicola	+	8.89E-01	0.000	0.000	0.000	0.001	0.000
3.5.4.25|g__Kluyvera.s__Kluyvera_ascorbata	-	2.85E-01	0.000	0.000	0.000	0.000	0.000
3.5.4.25|g__Kluyvera.s__Kluyvera_georgiana	-	2.85E-01	0.000	0.000	0.000	0.000	0.000
3.5.4.25|g__Lachnoclostridium.s__Clostridium_aldenense	+	2.44E-01	0.000	0.000	0.000	0.000	0.000
3.5.4.25|g__Lachnoclostridium.s__Clostridium_bolteae	+	8.18E-01	0.000	0.000	0.000	0.001	0.001
3.5.4.25|g__Lachnoclostridium.s__Clostridium_citroniae	-	9.38E-01	0.000	0.000	0.000	0.000	0.000
3.5.4.25|g__Lachnoclostridium.s__Clostridium_clostridioforme	-	2.40E-01	0.000	0.000	0.000	0.000	0.000
3.5.4.25|g__Lachnoclostridium.s__Clostridium_symbiosum	+	4.66E-02	0.000	0.000	0.000	0.000	0.001
3.5.4.25|g__Lachnoclostridium.s__Lachnoclostridium_sp_An138	+	3.68E-01	0.000	0.000	0.000	0.000	0.000
3.5.4.25|g__Lachnospira.s__Lachnospira_pectinoschiza	-	2.57E-01	0.000	0.000	0.000	0.001	0.001
3.5.4.25|g__Lactobacillus.s__Lactobacillus_fermentum	-	2.85E-01	0.000	0.000	0.000	0.000	0.000
3.5.4.25|g__Lactobacillus.s__Lactobacillus_rogosae	-	1.71E-01	0.000	0.000	0.000	0.002	0.001
3.5.4.25|g__Leclercia.s__Leclercia_adecarboxylata	-	4.71E-01	0.000	0.000	0.000	0.000	0.000
3.5.4.25|g__Lelliottia.s__Lelliottia_nimipressuralis	-	1.20E-01	0.000	0.000	0.000	0.000	0.000
3.5.4.25|g__Leuconostoc.s__Leuconostoc_garlicum	-	2.85E-01	0.000	0.000	0.000	0.000	0.000
3.5.4.25|g__Megamonas.s__Megamonas_funiformis	-	5.18E-01	0.000	0.000	0.000	0.007	0.004
3.5.4.25|g__Megamonas.s__Megamonas_rupellensis	-	6.08E-01	0.000	0.000	0.000	0.001	0.001
3.5.4.25|g__Megasphaera.s__Megasphaera_elsdenii	-	1.25E-01	0.000	0.000	0.000	0.000	0.000
3.5.4.25|g__Megasphaera.s__Megasphaera_hexanoica	+	3.68E-01	0.000	0.000	0.000	0.000	0.000
3.5.4.25|g__Megasphaera.s__Megasphaera_micronuciformis	+	6.86E-01	0.000	0.000	0.000	0.000	0.000
3.5.4.25|g__Megasphaera.s__Megasphaera_stantonii	-	2.97E-01	0.000	0.000	0.000	0.000	0.000
3.5.4.25|g__Metakosakonia.s__Kluyvera_intestini	+	3.68E-01	0.000	0.000	0.000	0.000	0.000
3.5.4.25|g__Mitsuokella.s__Mitsuokella_jalaludinii	-	5.21E-01	0.000	0.000	0.000	0.000	0.000
3.5.4.25|g__Mitsuokella.s__Mitsuokella_multacida	-	7.90E-01	0.000	0.000	0.000	0.000	0.000
3.5.4.25|g__Neisseria.s__Neisseria_meningitidis	-	2.85E-01	0.000	0.000	0.000	0.000	0.000
3.5.4.25|g__Neisseria.s__Neisseria_subflava	-	2.85E-01	0.000	0.000	0.000	0.000	0.000
3.5.4.25|g__Odoribacter.s__Odoribacter_laneus	+	3.87E-01	0.000	0.000	0.000	0.000	0.002
3.5.4.25|g__Paeniclostridium.s__Paeniclostridium_sordellii	+	3.68E-01	0.000	0.000	0.000	0.000	0.000
3.5.4.25|g__Pantoea.s__Pantoea_sesami	-	8.42E-01	0.000	0.000	0.000	0.000	0.000
3.5.4.25|g__Parabacteroides.s__Parabacteroides_chinchillae	-	9.05E-01	0.000	0.000	0.000	0.000	0.000
3.5.4.25|g__Parabacteroides.s__Parabacteroides_goldsteinii	+	2.63E-02	0.000	0.000	0.000	0.001	0.003
3.5.4.25|g__Parabacteroides.s__Parabacteroides_gordonii	-	3.01E-01	0.000	0.000	0.000	0.000	0.000
3.5.4.25|g__Parabacteroides.s__Parabacteroides_johnsonii	+	8.00E-01	0.000	0.000	0.000	0.001	0.001
3.5.4.25|g__Paraprevotella.s__Paraprevotella_clara	-	5.33E-01	0.000	0.000	0.000	0.006	0.007
3.5.4.25|g__Paraprevotella.s__Paraprevotella_xylaniphila	+	8.39E-02	0.000	0.000	0.000	0.000	0.002
3.5.4.25|g__Pedobacter.s__Pedobacter_himalayensis	-	1.25E-01	0.000	0.000	0.000	0.000	0.000
3.5.4.25|g__Phascolarctobacterium.s__Phascolarctobacterium_succinatutens	+	3.93E-01	0.000	0.000	0.000	0.002	0.003
3.5.4.25|g__Phytobacter.s__Phytobacter_ursingii	+	3.68E-01	0.000	0.000	0.000	0.000	0.000

	3.5.4.25|g__Plesiomonas.s__Plesiomonas_shigelloides	-	2.85E-01	0.000	0.000	0.000	0.001	0.000
	3.5.4.25|g__Prevotella.s__Prevotella_bivia	-	2.85E-01	0.000	0.000	0.000	0.000	0.000
	3.5.4.25|g__Prevotella.s__Prevotella_buccae	-	2.85E-01	0.000	0.000	0.000	0.004	0.000
	3.5.4.25|g__Prevotella.s__Prevotella_copri	-	7.68E-01	0.000	0.000	0.000	0.024	0.006
	3.5.4.25|g__Prevotella.s__Prevotella_sp_109	+	6.55E-01	0.000	0.000	0.000	0.014	0.009
	3.5.4.25|g__Prevotella.s__Prevotella_sp_885	-	9.05E-01	0.000	0.000	0.000	0.005	0.001
	3.5.4.25|g__Prevotella.s__Prevotella_sp_AM42_24	-	2.85E-01	0.000	0.000	0.000	0.003	0.000
	3.5.4.25|g__Prevotella.s__Prevotella_stercorea	+	8.79E-01	0.000	0.000	0.000	0.030	0.013
	3.5.4.25|g__Prevotella.s__Prevotella_timonensis	+	3.68E-01	0.000	0.000	0.000	0.000	0.000
	3.5.4.25|g__Pseudocitrobacter.s__Pseudocitrobacter_faecalis	+	3.68E-01	0.000	0.000	0.000	0.000	0.000
	3.5.4.25|g__Pseudoflavonifractor.s__Pseudoflavonifractor_sp_An184	+	1.96E-01	0.000	0.000	0.000	0.000	0.000
	3.5.4.25|g__Pyramidobacter.s__Pyramidobacter_piscolens	-	8.57E-01	0.000	0.000	0.000	0.000	0.000
	3.5.4.25|g__Pyramidobacter.s__Pyramidobacter_sp_C12_8	+	3.68E-01	0.000	0.000	0.000	0.000	0.000
	3.5.4.25|g__Raoultella.s__Raoultella_ornithinolytica	-	8.42E-01	0.000	0.000	0.000	0.000	0.000
	3.5.4.25|g__Raoultella.s__Raoultella_planticola	-	9.26E-01	0.000	0.000	0.000	0.000	0.000
	3.5.4.25|g__Roseburia.s__Roseburia_intestinalis	+	8.84E-01	0.000	0.000	0.000	0.005	0.002
	3.5.4.25|g__Roseburia.s__Roseburia_inulinivorans	-	4.51E-01	0.000	0.000	0.000	0.001	0.001
	3.5.4.25|g__Rothia.s__Rothia_mucilaginosa	-	2.85E-01	0.000	0.000	0.000	0.000	0.000
	3.5.4.25|g__Ruminococcaceae_unclassified.s__Ruminococcaceae_bacteriun	-	3.76E-01	0.000	0.000	0.000	0.000	0.000
	3.5.4.25|g__Ruminococcaceae_unclassified.s__Ruminococcaceae_bacteriun	+	2.28E-01	0.000	0.000	0.000	0.000	0.000
	3.5.4.25|g__Ruminococcus.s__Ruminococcus_callidus	-	8.97E-01	0.000	0.000	0.000	0.001	0.001
	3.5.4.25|g__Ruminococcus.s__Ruminococcus_sp_AF31_8BH	+	7.88E-02	0.000	0.000	0.000	0.000	0.000
	3.5.4.25|g__Sanguibacteroides.s__Sanguibacteroides_justesenii	+	1.34E-01	0.000	0.000	0.000	0.000	0.000
	3.5.4.25|g__Senegalimassilia.s__Senegalimassilia_anaerobia	+	2.20E-01	0.000	0.000	0.000	0.000	0.000
	3.5.4.25|g__Slackia.s__Slackia_isoflavoniconvertens	+	3.68E-01	0.000	0.000	0.000	0.000	0.000
	3.5.4.25|g__Streptococcus.s__Streptococcus_gallolyticus	+	1.96E-01	0.000	0.000	0.000	0.000	0.000
	3.5.4.25|g__Streptococcus.s__Streptococcus_macedonicus	+	1.96E-01	0.000	0.000	0.000	0.000	0.000
	3.5.4.25|g__Streptococcus.s__Streptococcus_pasteurianus	+	1.96E-01	0.000	0.000	0.000	0.000	0.000
	3.5.4.25|g__Streptococcus.s__Streptococcus_pneumoniae	-	9.26E-01	0.000	0.000	0.000	0.000	0.000
	3.5.4.25|g__Succinatimonas.s__Succinatimonas_hippei	+	6.51E-01	0.000	0.000	0.000	0.000	0.000
	3.5.4.25|g__Sutterella.s__Sutterella_wadsworthensis	+	4.83E-01	0.000	0.000	0.000	0.004	0.005
	3.5.4.25|g__Veillonella.s__Veillonella_atypica	-	2.15E-01	0.000	0.000	0.000	0.000	0.000
	3.5.4.25|g__Veillonella.s__Veillonella_dispar	-	6.52E-01	0.000	0.000	0.000	0.001	0.000
	3.5.4.25|g__Veillonella.s__Veillonella_infantium	-	3.14E-01	0.000	0.000	0.000	0.000	0.000
	3.5.4.25|g__Veillonella.s__Veillonella_parvula	-	6.58E-01	0.000	0.000	0.000	0.000	0.000
	3.5.4.25|g__Veillonella.s__Veillonella_rogosae	-	7.81E-02	0.000	0.000	0.000	0.000	0.000
	3.5.4.25|g__Veillonella.s__Veillonella_tobetsuensis	-	8.87E-01	0.000	0.000	0.000	0.000	0.000
	3.5.4.25|g__Veillonellaceae_unclassified.s__Veillonellaceae_bacterium	+	3.68E-01	0.000	0.000	0.000	0.000	0.000
	3.5.4.25|g__Vibrio.s__Vibrio_parahaemolyticus	-	2.85E-01	0.000	0.000	0.000	0.000	0.000
	3.5.4.25|g__Victivallales_unclassified.s__Victivallales_bacterium_CCUG_4	+	1.56E-01	0.000	0.000	0.000	0.001	0.001
	3.5.4.25|g__Victivallis.s__Victivallis_vadensis	+	5.24E-02	0.000	0.000	0.000	0.000	0.001
	3.5.4.25|g__Weissella.s__Weissella_confusa	-	9.26E-01	0.000	0.000	0.000	0.000	0.000
	3.5.4.25|g__Blautia.s__Blautia_wexlerae	+	9.97E-01	0.032	0.043	0.011	0.001	0.001
	3.5.4.25|g__Bacteroides.s__Bacteroides_massiliensis	-	9.77E-01	0.382	0.397	0.015	0.022	0.022
	3.5.4.25|g__Bacteroides.s__Bacteroides_finegoldii	+	2.15E-01	0.000	0.027	0.027	0.004	0.006
	3.5.4.25|g__Klebsiella.s__Klebsiella_oxytoca	+	6.38E-01	0.418	0.472	0.053	0.005	0.009
	3.5.4.25|g__Roseburia.s__Roseburia_hominis	+	3.64E-01	0.147	0.212	0.065	0.003	0.004
	3.5.4.25|g__Blautia.s__Ruminococcus_torques	+	9.38E-01	0.502	0.583	0.081	0.013	0.012
	3.5.4.25|g__Bacteroides.s__Bacteroides_dorei	+	2.54E-01	0.272	0.357	0.085	0.015	0.028
	3.5.4.25|g__Alistipes.s__Alistipes_finegoldii	+	4.16E-01	0.253	0.342	0.089	0.009	0.016
	3.5.4.25|g__Bacteroides.s__Bacteroides_intestinalis	+	5.35E-02	0.000	0.090	0.090	0.003	0.010
	3.5.4.25|g__Butyricimonas.s__Butyricimonas_virosa	+	2.11E-02	0.000	0.124	0.124	0.002	0.004
	3.5.4.25|g__Eubacterium.s__Eubacterium_eligens	+	9.24E-01	0.252	0.413	0.160	0.008	0.007
	3.5.4.25|g__Alistipes.s__Alistipes_onderdonkii	+	3.77E-01	0.121	0.285	0.164	0.006	0.009
	3.5.4.25|g__Faecalibacterium.s__Faecalibacterium_prausnitzii	+	6.32E-01	2.847	3.014	0.167	0.037	0.039
	3.5.4.25|g__Bacteroides.s__Bacteroides_ovatus	+	1.92E-01	0.184	0.444	0.260	0.010	0.012
	3.5.4.25|g__Odoribacter.s__Odoribacter_splanchnicus	+	5.28E-02	0.210	0.663	0.452	0.006	0.008
	3.5.4.25|g__Phascolarctobacterium.s__Phascolarctobacterium_faecium	+	9.15E-01	0.253	0.706	0.453	0.010	0.010
	3.5.4.25|g__Bacteroides.s__Bacteroides_cellulosilyticus	+	8.03E-03	0.018	0.522	0.504	0.012	0.012
	3.5.4.25|g__Bacteroides.s__Bacteroides_stercoris	+	9.97E-01	1.372	1.945	0.573	0.043	0.039
	3.5.4.25|g__Bacteroides.s__Bacteroides_thetaiotaomicron	+	6.20E-02	0.753	1.381	0.628	0.014	0.021
	3.5.4.25|g__Parabacteroides.s__Parabacteroides_distasonis	+	1.72E-02	0.772	1.499	0.727	0.014	0.021
	3.5.4.25|g__Parabacteroides.s__Parabacteroides_merdae	+	1.62E-02	0.441	1.251	0.810	0.007	0.014
	3.5.4.25|g__Bacteroides.s__Bacteroides_caccae	+	2.37E-02	0.371	1.508	1.137	0.012	0.017
	3.5.4.25|g__Alistipes.s__Alistipes_putredinis	+	2.26E-01	1.122	2.536	1.415	0.023	0.032
	3.5.4.25|g__Bacteroides.s__Bacteroides_uniformis	+	5.05E-01	3.496	5.206	1.710	0.063	0.070

Country	EC number|Bacteria	Increased or decreased in PD	*p* -value	Fractional CPM (median)			Relative CPM (average)	
				Control	PD	PD - Control	Control	PD
Japan	3.5.4.26	-	1.76E-04	94.758	82.473	-12.285	1.000	1.000
	3.5.4.26|g__Faecalibacterium.s__Faecalibacterium_prausnitzii	-	1.61E-02	9.122	4.960	-4.162	0.095	0.080
	3.5.4.26|unclassified	-	1.58E-02	10.785	8.530	-2.255	0.129	0.125
	3.5.4.26|g__Blautia.s__Blautia_wexlerae	-	2.63E-04	3.337	1.600	-1.737	0.044	0.029

3.5.4.26|g__Dorea.s__Dorea_longicatena	-	5.36E-02	1.281	0.113	-1.168	0.017	0.012
3.5.4.26|g__Blautia.s__Blautia_obeum	-	2.76E-03	2.818	1.907	-0.911	0.035	0.028
3.5.4.26|g__Lachnospiraceae_unclassified.s__Eubacterium_rectale	-	9.98E-02	0.813	0.000	-0.813	0.035	0.028
3.5.4.26|g__Blautia.s__Ruminococcus_torques	-	4.47E-02	2.331	1.649	-0.682	0.035	0.026
3.5.4.26|g__Bacteroides.s__Bacteroides_uniformis	+	7.24E-01	4.368	3.934	-0.433	0.058	0.076
3.5.4.26|g__Agathobaculum.s__Agathobaculum_butyriciproducens	-	3.54E-02	0.433	0.000	-0.433	0.005	0.004
3.5.4.26|g__Roseburia.s__Roseburia_hominis	-	1.91E-01	1.402	1.050	-0.352	0.019	0.017
3.5.4.26|g__Bacteroides.s__Bacteroides_vulgatus	-	6.03E-01	1.678	1.366	-0.312	0.055	0.046
3.5.4.26|g__Anaerostipes.s__Anaerostipes_hadrus	-	2.06E-01	1.341	1.084	-0.257	0.029	0.025
3.5.4.26|g__Bacteroides.s__Bacteroides_xylanisolvens	-	3.89E-01	0.064	0.000	-0.064	0.007	0.007
3.5.4.26|g__Bacteroides.s__Bacteroides_ovatus	+	9.43E-01	0.379	0.358	-0.021	0.013	0.016
3.5.4.26|g__Acidaminococcus.s__Acidaminococcus_fermentans	+	3.85E-01	0.000	0.000	0.000	0.000	0.000
3.5.4.26|g__Acidaminococcus.s__Acidaminococcus_intestini	+	9.82E-01	0.000	0.000	0.000	0.009	0.003
3.5.4.26|g__Adlercreutzia.s__Adlercreutzia_equolifaciens	+	1.41E-01	0.000	0.000	0.000	0.000	0.001
3.5.4.26|g__Akkermansia.s__Akkermansia_muciniphila	+	8.28E-04	0.000	0.000	0.000	0.006	0.045
3.5.4.26|g__Allisonella.s__Allisonella_histaminiformans	-	2.62E-01	0.000	0.000	0.000	0.000	0.000
3.5.4.26|g__Anaeroglobus.s__Anaeroglobus_geminatus	+	3.85E-01	0.000	0.000	0.000	0.000	0.000
3.5.4.26|g__Anaeromassilibacillus.s__Anaeromassilibacillus_sp_An250	-	8.57E-01	0.000	0.000	0.000	0.000	0.000
3.5.4.26|g__Anaerostipes.s__Anaerostipes_caccae	-	1.76E-01	0.000	0.000	0.000	0.001	0.001
3.5.4.26|g__Anaerotignum.s__Anaerotignum_lactatifermentans	+	1.32E-02	0.000	0.000	0.000	0.000	0.002
3.5.4.26|g__Asaccharobacter.s__Asaccharobacter_celatus	+	4.20E-01	0.000	0.000	0.000	0.001	0.001
3.5.4.26|g__Bacillus.s__Bacillus_gibsonii	+	5.06E-02	0.000	0.000	0.000	0.000	0.001
3.5.4.26|g__Bacillus.s__Bacillus_murimartini	+	2.93E-01	0.000	0.000	0.000	0.000	0.000
3.5.4.26|g__Bacteroides.s__Bacteroides_caccae	+	1.76E-01	0.000	0.000	0.000	0.004	0.008
3.5.4.26|g__Bacteroides.s__Bacteroides_cellulosilyticus	+	9.01E-02	0.000	0.000	0.000	0.004	0.007
3.5.4.26|g__Bacteroides.s__Bacteroides_clarus	+	2.56E-01	0.000	0.000	0.000	0.000	0.005
3.5.4.26|g__Bacteroides.s__Bacteroides_coprocola	+	3.83E-01	0.000	0.000	0.000	0.012	0.014
3.5.4.26|g__Bacteroides.s__Bacteroides_coprophilus	+	4.41E-01	0.000	0.000	0.000	0.001	0.001
3.5.4.26|g__Bacteroides.s__Bacteroides_eggerthii	+	2.30E-01	0.000	0.000	0.000	0.004	0.012
3.5.4.26|g__Bacteroides.s__Bacteroides_faecis	-	8.03E-01	0.000	0.000	0.000	0.000	0.000
3.5.4.26|g__Bacteroides.s__Bacteroides_finegoldii	+	7.57E-01	0.000	0.000	0.000	0.003	0.003
3.5.4.26|g__Bacteroides.s__Bacteroides_fluxus	-	2.62E-01	0.000	0.000	0.000	0.000	0.000
3.5.4.26|g__Bacteroides.s__Bacteroides_fragilis	-	8.41E-01	0.000	0.000	0.000	0.005	0.008
3.5.4.26|g__Bacteroides.s__Bacteroides_intestinalis	+	6.96E-02	0.000	0.000	0.000	0.003	0.005
3.5.4.26|g__Bacteroides.s__Bacteroides_massiliensis	+	2.71E-01	0.000	0.000	0.000	0.003	0.004
3.5.4.26|g__Bacteroides.s__Bacteroides_nordii	+	2.88E-02	0.000	0.000	0.000	0.000	0.001
3.5.4.26|g__Bacteroides.s__Bacteroides_oleiciplenus	+	3.85E-01	0.000	0.000	0.000	0.000	0.000
3.5.4.26|g__Bacteroides.s__Bacteroides_plebeius	+	1.79E-01	0.000	0.000	0.000	0.027	0.023
3.5.4.26|g__Bacteroides.s__Bacteroides_salyersiae	+	1.05E-01	0.000	0.000	0.000	0.000	0.001
3.5.4.26|g__Bacteroides.s__Bacteroides_stercoris	-	8.09E-01	0.000	0.000	0.000	0.034	0.026
3.5.4.26|g__Barnesiella.s__Barnesiella_intestinihominis	+	1.11E-03	0.000	0.000	0.000	0.003	0.010
3.5.4.26|g__Barnesiella.s__Barnesiella_sp_An22	-	2.62E-01	0.000	0.000	0.000	0.000	0.000
3.5.4.26|g__Bifidobacterium.s__Bifidobacterium_longum	-	8.57E-01	0.000	0.000	0.000	0.000	0.000
3.5.4.26|g__Bilophila.s__Bilophila_wadsworthia	+	2.16E-02	0.000	0.000	0.000	0.002	0.004
3.5.4.26|g__Blautia.s__Blautia_hansenii	-	9.89E-02	0.000	0.000	0.000	0.001	0.000
3.5.4.26|g__Blautia.s__Blautia_sp_AF19_10LB	+	9.05E-01	0.000	0.000	0.000	0.004	0.005
3.5.4.26|g__Blautia.s__Blautia_sp_An249	+	1.26E-01	0.000	0.000	0.000	0.000	0.001
3.5.4.26|g__Butyricicoccus.s__Butyricicoccus_pullicaecorum	-	4.41E-01	0.000	0.000	0.000	0.001	0.000
3.5.4.26|g__Butyricimonas.s__Butyricimonas_virosa	+	2.83E-02	0.000	0.000	0.000	0.000	0.001
3.5.4.26|g__Butyrivibrio.s__Butyrivibrio_crossotus	+	3.85E-01	0.000	0.000	0.000	0.000	0.001
3.5.4.26|g__Catenibacterium.s__Catenibacterium_mitsuokai	-	8.17E-03	0.000	0.000	0.000	0.002	0.000
3.5.4.26|g__Cellulosilyticum.s__Cellulosilyticum_lentocellum	+	3.85E-01	0.000	0.000	0.000	0.000	0.000
3.5.4.26|g__Cetobacterium.s__Cetobacterium_somerae	-	2.62E-01	0.000	0.000	0.000	0.000	0.000
3.5.4.26|g__Citrobacter.s__Citrobacter_braakii	+	4.46E-01	0.000	0.000	0.000	0.000	0.001
3.5.4.26|g__Citrobacter.s__Citrobacter_freundii	+	1.72E-01	0.000	0.000	0.000	0.000	0.000
3.5.4.26|g__Citrobacter.s__Citrobacter_koseri	-	8.71E-01	0.000	0.000	0.000	0.000	0.000
3.5.4.26|g__Citrobacter.s__Citrobacter_portucalensis	+	7.63E-02	0.000	0.000	0.000	0.000	0.000
3.5.4.26|g__Citrobacter.s__Citrobacter_werkmanii	+	3.85E-01	0.000	0.000	0.000	0.000	0.003
3.5.4.26|g__Citrobacter.s__Citrobacter_youngae	+	2.72E-01	0.000	0.000	0.000	0.000	0.001
3.5.4.26|g__Cloacibacillus.s__Cloacibacillus_porcorum	+	2.14E-01	0.000	0.000	0.000	0.000	0.001
3.5.4.26|g__Clostridioides.s__Clostridioides_difficile	+	7.85E-01	0.000	0.000	0.000	0.004	0.004
3.5.4.26|g__Clostridium.s__Butyribacterium_methylotrophicum	+	5.79E-01	0.000	0.000	0.000	0.000	0.000
3.5.4.26|g__Clostridium.s__Clostridium_celatum	+	1.00E+00	0.000	0.000	0.000	0.000	0.000
3.5.4.26|g__Clostridium.s__Clostridium_disporicum	+	9.44E-01	0.000	0.000	0.000	0.002	0.001
3.5.4.26|g__Clostridium.s__Clostridium_perfringens	-	2.52E-01	0.000	0.000	0.000	0.001	0.000
3.5.4.26|g__Clostridium.s__Clostridium_sp_AF36_4	+	5.84E-01	0.000	0.000	0.000	0.001	0.002
3.5.4.26|g__Clostridium.s__Clostridium_sp_AM22_11AC	-	2.32E-01	0.000	0.000	0.000	0.003	0.002
3.5.4.26|g__Coprobacillus.s__Coprobacillus_cateniformis	+	3.84E-02	0.000	0.000	0.000	0.000	0.001
3.5.4.26|g__Coprobacter.s__Coprobacter_fastidiosus	+	8.23E-01	0.000	0.000	0.000	0.002	0.002
3.5.4.26|g__Coprobacter.s__Coprobacter_secundus	+	9.67E-01	0.000	0.000	0.000	0.000	0.001
3.5.4.26|g__Coprococcus.s__Coprococcus_catus	+	9.02E-01	0.000	0.000	0.000	0.001	0.002
3.5.4.26|g__Coprococcus.s__Coprococcus_comes	-	4.45E-02	0.000	0.000	0.000	0.006	0.003

3.5.4.26|g__Coprococcus.s__Coprococcus_eutactus	-	2.86E-01	0.000	0.000	0.000	0.003	0.002
3.5.4.26|g__Desulfovibrio.s__Desulfovibrio_desulfuricans	+	1.78E-02	0.000	0.000	0.000	0.000	0.001
3.5.4.26|g__Desulfovibrio.s__Desulfovibrio_fairfieldensis	+	1.26E-01	0.000	0.000	0.000	0.000	0.000
3.5.4.26|g__Desulfovibrio.s__Desulfovibrio_piger	+	6.20E-01	0.000	0.000	0.000	0.000	0.000
3.5.4.26|g__Desulfovibrio.s__Desulfovibrio_sp_AM18_2	+	3.85E-01	0.000	0.000	0.000	0.000	0.000
3.5.4.26|g__Desulfovibrionaceae_unclassified.s__Desulfovibrionaceae_bact	-	2.62E-01	0.000	0.000	0.000	0.000	0.000
3.5.4.26|g__Dialister.s__Dialister_succinatiphilus	+	7.10E-01	0.000	0.000	0.000	0.000	0.001
3.5.4.26|g__Dorea.s__Dorea_formicigenerans	-	7.17E-01	0.000	0.000	0.000	0.005	0.005
3.5.4.26|g__Dorea.s__Dorea_sp_OM02_2LB	+	2.14E-01	0.000	0.000	0.000	0.000	0.000
3.5.4.26|g__Eggerthella.s__Eggerthella_lenta	+	3.16E-01	0.000	0.000	0.000	0.005	0.004
3.5.4.26|g__Enterobacter.s__Enterobacter_bugandensis	-	2.62E-01	0.000	0.000	0.000	0.000	0.000
3.5.4.26|g__Enterobacter.s__Enterobacter_cloacae	+	1.15E-01	0.000	0.000	0.000	0.000	0.000
3.5.4.26|g__Enterobacter.s__Enterobacter_mori	+	1.26E-01	0.000	0.000	0.000	0.000	0.001
3.5.4.26|g__Enterococcus.s__Enterococcus_avium	+	4.14E-01	0.000	0.000	0.000	0.006	0.002
3.5.4.26|g__Enterococcus.s__Enterococcus_faecalis	-	2.62E-01	0.000	0.000	0.000	0.000	0.000
3.5.4.26|g__Enterococcus.s__Enterococcus_faecium	+	5.56E-01	0.000	0.000	0.000	0.005	0.002
3.5.4.26|g__Enterococcus.s__Enterococcus_gallinarum	+	3.85E-01	0.000	0.000	0.000	0.000	0.000
3.5.4.26|g__Enterococcus.s__Enterococcus_saccharolyticus	+	3.85E-01	0.000	0.000	0.000	0.000	0.000
3.5.4.26|g__Enterococcus.s__Enterococcus_thailandicus	+	3.85E-01	0.000	0.000	0.000	0.000	0.000
3.5.4.26|g__Erysipelatoclostridium.s__Clostridium_innocuum	+	6.98E-01	0.000	0.000	0.000	0.004	0.004
3.5.4.26|g__Erysipelatoclostridium.s__Clostridium_spiroforme	-	4.16E-01	0.000	0.000	0.000	0.003	0.001
3.5.4.26|g__Erysipelotrichaceae_unclassified.s__Erysipelotrichaceae_bacteri	+	4.30E-02	0.000	0.000	0.000	0.000	0.001
3.5.4.26|g__Escherichia.s__Escherichia_coli	+	4.78E-02	0.000	0.000	0.000	0.004	0.005
3.5.4.26|g__Eubacterium.s__Eubacterium_callanderi	+	4.03E-01	0.000	0.000	0.000	0.000	0.000
3.5.4.26|g__Eubacterium.s__Eubacterium_eligens	+	3.79E-01	0.000	0.000	0.000	0.011	0.018
3.5.4.26|g__Eubacterium.s__Eubacterium_limosum	+	2.72E-02	0.000	0.000	0.000	0.000	0.001
3.5.4.26|g__Eubacterium.s__Eubacterium_maltosivorans	+	3.85E-01	0.000	0.000	0.000	0.000	0.000
3.5.4.26|g__Eubacterium.s__Eubacterium_ramulus	+	8.89E-01	0.000	0.000	0.000	0.002	0.002
3.5.4.26|g__Eubacterium.s__Eubacterium_sp_AF17_7	+	4.04E-02	0.000	0.000	0.000	0.001	0.003
3.5.4.26|g__Eubacterium.s__Eubacterium_sp_AM18_10LB_B	-	6.46E-01	0.000	0.000	0.000	0.001	0.001
3.5.4.26|g__Faecalicatena.s__Faecalicatena_contorta	+	4.39E-01	0.000	0.000	0.000	0.000	0.000
3.5.4.26|g__Firmicutes_unclassified.s__Firmicutes_bacterium_AM10_47	+	6.38E-01	0.000	0.000	0.000	0.002	0.002
3.5.4.26|g__Flavonifractor.s__Flavonifractor_plautii	+	1.85E-01	0.000	0.000	0.000	0.000	0.000
3.5.4.26|g__Fusobacterium.s__Fusobacterium_mortiferum	-	2.12E-01	0.000	0.000	0.000	0.000	0.000
3.5.4.26|g__Fusobacterium.s__Fusobacterium_ulcerans	+	6.13E-01	0.000	0.000	0.000	0.000	0.000
3.5.4.26|g__Geobacillus.s__Bacillus_caldolyticus	+	3.85E-01	0.000	0.000	0.000	0.000	0.000
3.5.4.26|g__Geobacillus.s__Geobacillus_stearothermophilus	+	3.85E-01	0.000	0.000	0.000	0.000	0.000
3.5.4.26|g__Geobacillus.s__Geobacillus_thermocatenulatus	+	3.85E-01	0.000	0.000	0.000	0.000	0.000
3.5.4.26|g__Geobacillus.s__Geobacillus_thermoleovorans	+	3.85E-01	0.000	0.000	0.000	0.000	0.000
3.5.4.26|g__Haemophilus.s__Haemophilus_parainfluenzae	-	4.43E-02	0.000	0.000	0.000	0.002	0.001
3.5.4.26|g__Holdemanella.s__Holdemanella_biformis	-	2.22E-01	0.000	0.000	0.000	0.001	0.003
3.5.4.26|g__Hungatella.s__Hungatella_hathewayi	+	4.12E-01	0.000	0.000	0.000	0.001	0.002
3.5.4.26|g__Intestinibacter.s__Intestinibacter_bartlettii	-	1.29E-01	0.000	0.000	0.000	0.003	0.002
3.5.4.26|g__Klebsiella.s__Klebsiella_aerogenes	-	8.57E-01	0.000	0.000	0.000	0.000	0.000
3.5.4.26|g__Klebsiella.s__Klebsiella_grimontii	-	1.09E-01	0.000	0.000	0.000	0.000	0.000
3.5.4.26|g__Klebsiella.s__Klebsiella_michiganensis	-	8.71E-01	0.000	0.000	0.000	0.000	0.000
3.5.4.26|g__Klebsiella.s__Klebsiella_oxytoca	-	5.30E-01	0.000	0.000	0.000	0.004	0.002
3.5.4.26|g__Klebsiella.s__Klebsiella_pneumoniae	-	9.02E-01	0.000	0.000	0.000	0.007	0.006
3.5.4.26|g__Klebsiella.s__Klebsiella_variicola	-	6.52E-01	0.000	0.000	0.000	0.001	0.000
3.5.4.26|g__Lachnoclostridium.s__Clostridium_aldenense	+	2.86E-01	0.000	0.000	0.000	0.000	0.000
3.5.4.26|g__Lachnoclostridium.s__Clostridium_bolteae	+	3.68E-01	0.000	0.000	0.000	0.001	0.002
3.5.4.26|g__Lachnoclostridium.s__Clostridium_citroniae	+	3.85E-01	0.000	0.000	0.000	0.000	0.000
3.5.4.26|g__Lachnoclostridium.s__Clostridium_clostridioforme	-	7.77E-01	0.000	0.000	0.000	0.000	0.000
3.5.4.26|g__Lachnoclostridium.s__Clostridium_symbiosum	+	9.96E-01	0.000	0.000	0.000	0.000	0.000
3.5.4.26|g__Lachnospira.s__Lachnospira_pectinoschiza	-	2.27E-01	0.000	0.000	0.000	0.003	0.002
3.5.4.26|g__Lactobacillus.s__Lactobacillus_amylovorus	-	8.57E-01	0.000	0.000	0.000	0.000	0.000
3.5.4.26|g__Lactobacillus.s__Lactobacillus_antri	+	2.14E-01	0.000	0.000	0.000	0.000	0.000
3.5.4.26|g__Lactobacillus.s__Lactobacillus_crispatus	+	6.84E-03	0.000	0.000	0.000	0.000	0.002
3.5.4.26|g__Lactobacillus.s__Lactobacillus_fermentum	+	6.51E-02	0.000	0.000	0.000	0.000	0.004
3.5.4.26|g__Lactobacillus.s__Lactobacillus_kimbladii	+	3.85E-01	0.000	0.000	0.000	0.000	0.000
3.5.4.26|g__Lactobacillus.s__Lactobacillus_kullabergensis	+	3.85E-01	0.000	0.000	0.000	0.000	0.000
3.5.4.26|g__Lactobacillus.s__Lactobacillus_melliventris	-	2.62E-01	0.000	0.000	0.000	0.000	0.000
3.5.4.26|g__Lactobacillus.s__Lactobacillus_oris	+	3.98E-01	0.000	0.000	0.000	0.000	0.003
3.5.4.26|g__Lactobacillus.s__Lactobacillus_plantarum	+	3.85E-01	0.000	0.000	0.000	0.000	0.000
3.5.4.26|g__Lactobacillus.s__Lactobacillus_reuteri	+	3.85E-01	0.000	0.000	0.000	0.000	0.000
3.5.4.26|g__Lactobacillus.s__Lactobacillus_rogosae	-	2.87E-01	0.000	0.000	0.000	0.003	0.003
3.5.4.26|g__Lactococcus.s__Lactococcus_lactis	-	4.16E-01	0.000	0.000	0.000	0.000	0.000
3.5.4.26|g__Leclercia.s__Leclercia_adecarboxylata	-	1.09E-01	0.000	0.000	0.000	0.000	0.000
3.5.4.26|g__Lelliottia.s__Lelliottia_nimipressuralis	-	2.03E-01	0.000	0.000	0.000	0.000	0.000
3.5.4.26|g__Leuconostoc.s__Leuconostoc_garlicum	+	2.14E-01	0.000	0.000	0.000	0.000	0.000
3.5.4.26|g__Leuconostoc.s__Leuconostoc_lactis	+	2.14E-01	0.000	0.000	0.000	0.000	0.000
3.5.4.26|g__Listeria.s__Listeria_monocytogenes	-	2.62E-01	0.000	0.000	0.000	0.000	0.000

	3.5.4.26|g__Megamonas.s__Megamonas_funiformis	-	9.19E-01	0.000	0.000	0.000	0.005	0.004
	3.5.4.26|g__Megamonas.s__Megamonas_hypermegale	-	7.64E-01	0.000	0.000	0.000	0.006	0.002
	3.5.4.26|g__Megamonas.s__Megamonas_rupellensis	-	9.51E-01	0.000	0.000	0.000	0.006	0.003
	3.5.4.26|g__Megasphaera.s__Megasphaera_elsdenii	-	8.57E-01	0.000	0.000	0.000	0.000	0.000
	3.5.4.26|g__Megasphaera.s__Megasphaera_hexanoica	+	3.85E-01	0.000	0.000	0.000	0.000	0.000
	3.5.4.26|g__Megasphaera.s__Megasphaera_stantonii	-	8.12E-01	0.000	0.000	0.000	0.000	0.000
	3.5.4.26|g__Mesosutterella.s__Mesosutterella_multiformis	-	1.54E-01	0.000	0.000	0.000	0.002	0.000
	3.5.4.26|g__Metakosakonia.s__Kluyvera_intestini	+	3.85E-01	0.000	0.000	0.000	0.000	0.000
	3.5.4.26|g__Mitsuokella.s__Mitsuokella_jalaludinii	+	3.85E-01	0.000	0.000	0.000	0.000	0.000
	3.5.4.26|g__Mitsuokella.s__Mitsuokella_multacida	+	3.85E-01	0.000	0.000	0.000	0.000	0.000
	3.5.4.26|g__Odoribacter.s__Odoribacter_laneus	+	7.63E-01	0.000	0.000	0.000	0.000	0.001
	3.5.4.26|g__Odoribacter.s__Odoribacter_splanchnicus	+	5.23E-02	0.000	0.000	0.000	0.002	0.004
	3.5.4.26|g__Pantoea.s__Pantoea_sesami	-	8.03E-01	0.000	0.000	0.000	0.000	0.000
	3.5.4.26|g__Parabacteroides.s__Parabacteroides_goldsteinii	+	4.81E-01	0.000	0.000	0.000	0.000	0.001
	3.5.4.26|g__Parabacteroides.s__Parabacteroides_johnsonii	-	6.63E-01	0.000	0.000	0.000	0.001	0.001
	3.5.4.26|g__Paraprevotella.s__Paraprevotella_clara	+	9.26E-01	0.000	0.000	0.000	0.002	0.002
	3.5.4.26|g__Paraprevotella.s__Paraprevotella_xylaniphila	+	4.46E-01	0.000	0.000	0.000	0.000	0.000
	3.5.4.26|g__Parasutterella.s__Parasutterella_excrementihominis	-	6.69E-01	0.000	0.000	0.000	0.001	0.001
	3.5.4.26|g__Pediococcus.s__Pediococcus_acidilactici	+	4.61E-01	0.000	0.000	0.000	0.000	0.000
	3.5.4.26|g__Peptostreptococcaceae_unclassified.s__Clostridium_hiranonis	-	2.62E-01	0.000	0.000	0.000	0.000	0.000
	3.5.4.26|g__Phascolarctobacterium.s__Phascolarctobacterium_faecium	+	2.70E-01	0.000	0.000	0.000	0.005	0.010
	3.5.4.26|g__Phascolarctobacterium.s__Phascolarctobacterium_succinatutens	-	4.86E-01	0.000	0.000	0.000	0.005	0.004
	3.5.4.26|g__Prevotella.s__Prevotella_buccae	-	1.09E-01	0.000	0.000	0.000	0.002	0.000
	3.5.4.26|g__Prevotella.s__Prevotella_copri	+	9.14E-01	0.000	0.000	0.000	0.045	0.035
	3.5.4.26|g__Prevotella.s__Prevotella_sp_109	-	2.03E-01	0.000	0.000	0.000	0.003	0.001
	3.5.4.26|g__Prevotella.s__Prevotella_sp_AM42_24	-	1.99E-01	0.000	0.000	0.000	0.003	0.000
	3.5.4.26|g__Pseudoflavonifractor.s__Pseudoflavonifractor_sp_An184	+	1.26E-01	0.000	0.000	0.000	0.000	0.001
	3.5.4.26|g__Pyramidobacter.s__Pyramidobacter_piscolens	+	2.14E-01	0.000	0.000	0.000	0.000	0.000
	3.5.4.26|g__Raoultella.s__Raoultella_ornithinolytica	-	4.16E-01	0.000	0.000	0.000	0.004	0.000
	3.5.4.26|g__Raoultella.s__Raoultella_planticola	-	1.09E-01	0.000	0.000	0.000	0.000	0.000
	3.5.4.26|g__Rikenella.s__Rikenella_microfusus	+	1.26E-01	0.000	0.000	0.000	0.000	0.001
	3.5.4.26|g__Roseburia.s__Roseburia_faecis	+	5.00E-01	0.000	0.000	0.000	0.002	0.002
	3.5.4.26|g__Roseburia.s__Roseburia_intestinalis	-	4.89E-05	0.000	0.000	0.000	0.003	0.000
	3.5.4.26|g__Roseburia.s__Roseburia_inulinivorans	-	6.59E-01	0.000	0.000	0.000	0.003	0.001
	3.5.4.26|g__Ruminococcaceae_unclassified.s__Ruminococcaceae_bacteriun	+	1.82E-01	0.000	0.000	0.000	0.000	0.000
	3.5.4.26|g__Ruminococcaceae_unclassified.s__Ruminococcaceae_bacteriun	-	7.53E-01	0.000	0.000	0.000	0.000	0.000
	3.5.4.26|g__Ruminococcus.s__Ruminococcus_callidus	-	9.99E-02	0.000	0.000	0.000	0.005	0.002
	3.5.4.26|g__Ruminococcus.s__Ruminococcus_sp_AF31_8BH	+	1.10E-01	0.000	0.000	0.000	0.001	0.002
	3.5.4.26|g__Saccharomyces.s__Saccharomyces_cerevisiae	-	2.62E-01	0.000	0.000	0.000	0.000	0.000
	3.5.4.26|g__Salmonella.s__Salmonella_enterica	+	2.14E-01	0.000	0.000	0.000	0.000	0.000
	3.5.4.26|g__Slackia.s__Slackia_isoflavoniconvertens	-	9.98E-01	0.000	0.000	0.000	0.001	0.002
	3.5.4.26|g__Staphylococcus.s__Staphylococcus_aureus	+	2.14E-01	0.000	0.000	0.000	0.000	0.000
	3.5.4.26|g__Streptococcus.s__Streptococcus_equinus	-	2.89E-01	0.000	0.000	0.000	0.003	0.001
	3.5.4.26|g__Streptococcus.s__Streptococcus_gallolyticus	-	4.32E-01	0.000	0.000	0.000	0.000	0.000
	3.5.4.26|g__Streptococcus.s__Streptococcus_infantarius	-	1.09E-01	0.000	0.000	0.000	0.003	0.000
	3.5.4.26|g__Streptococcus.s__Streptococcus_lutetiensis	-	1.09E-01	0.000	0.000	0.000	0.001	0.000
	3.5.4.26|g__Streptococcus.s__Streptococcus_macedonicus	-	7.53E-01	0.000	0.000	0.000	0.000	0.000
	3.5.4.26|g__Streptococcus.s__Streptococcus_pasteurianus	-	7.30E-01	0.000	0.000	0.000	0.000	0.000
	3.5.4.26|g__Streptococcus.s__Streptococcus_pneumoniae	+	6.84E-02	0.000	0.000	0.000	0.000	0.001
	3.5.4.26|g__Succinatimonas.s__Succinatimonas_hippei	+	3.85E-01	0.000	0.000	0.000	0.000	0.000
	3.5.4.26|g__Sutterella.s__Sutterella_wadsworthensis	-	5.64E-01	0.000	0.000	0.000	0.002	0.002
	3.5.4.26|g__Terrisporobacter.s__Terrisporobacter_othiniensis	+	3.85E-01	0.000	0.000	0.000	0.000	0.000
	3.5.4.26|g__Veillonella.s__Veillonella_atypica	-	2.30E-04	0.000	0.000	0.000	0.001	0.000
	3.5.4.26|g__Veillonella.s__Veillonella_denticariosi	+	3.85E-01	0.000	0.000	0.000	0.000	0.000
	3.5.4.26|g__Veillonella.s__Veillonella_dispar	-	2.59E-02	0.000	0.000	0.000	0.001	0.000
	3.5.4.26|g__Veillonella.s__Veillonella_infantium	-	1.33E-01	0.000	0.000	0.000	0.000	0.000
	3.5.4.26|g__Veillonella.s__Veillonella_parvula	-	1.09E-01	0.000	0.000	0.000	0.001	0.001
	3.5.4.26|g__Veillonella.s__Veillonella_rogosae	-	2.27E-03	0.000	0.000	0.000	0.000	0.000
	3.5.4.26|g__Veillonella.s__Veillonella_tobetsuensis	-	2.62E-01	0.000	0.000	0.000	0.000	0.000
	3.5.4.26|g__Victivallales_unclassified.s__Victivallales_bacterium_CCUG_4	+	7.31E-01	0.000	0.000	0.000	0.000	0.000
	3.5.4.26|g__Victivallis.s__Victivallis_vadensis	+	3.85E-01	0.000	0.000	0.000	0.000	0.000
	3.5.4.26|g__Weissella.s__Weissella_cibaria	-	8.71E-01	0.000	0.000	0.000	0.000	0.000
	3.5.4.26|g__Weissella.s__Weissella_confusa	+	3.85E-01	0.000	0.000	0.000	0.000	0.000
	3.5.4.26|g__Yokenella.s__Yokenella_regensburgei	+	3.85E-01	0.000	0.000	0.000	0.000	0.000
	3.5.4.26|g__Bacteroides.s__Bacteroides_thetaiotaomicron	+	2.25E-01	0.387	0.615	0.228	0.008	0.014
	3.5.4.26|g__Parabacteroides.s__Parabacteroides_merdae	+	3.26E-01	0.345	0.676	0.331	0.010	0.012
	3.5.4.26|g__Parabacteroides.s__Parabacteroides_distasonis	+	1.75E-02	0.510	0.968	0.458	0.011	0.018
	3.5.4.26|g__Bacteroides.s__Bacteroides_dorei	+	6.85E-01	0.458	1.055	0.597	0.039	0.038
USA	3.5.4.26	-	2.63E-07	99.919	90.079	-9.840	1.000	1.000
	3.5.4.26|g__Faecalibacterium.s__Faecalibacterium_prausnitzii	-	1.19E-05	7.285	2.631	-4.654	0.081	0.063
	3.5.4.26|g__Bacteroides.s__Bacteroides_vulgatus	-	6.65E-02	6.085	4.356	-1.729	0.077	0.072
	3.5.4.26|g__Bacteroides.s__Bacteroides_uniformis	-	7.90E-01	5.696	4.692	-1.004	0.073	0.080

3.5.4.26|g__Lachnospiraceae_unclassified.s__Eubacterium_rectale	-	2.82E-05	1.302	0.349	-0.953	0.033	0.022
3.5.4.26|g__Blautia.s__Ruminococcus_torques	-	1.23E-05	2.754	1.839	-0.915	0.035	0.028
3.5.4.26|g__Blautia.s__Blautia_wexlerae	-	6.23E-08	2.294	1.419	-0.875	0.037	0.026
3.5.4.26|g__Blautia.s__Blautia_obeum	-	4.02E-04	3.245	2.662	-0.582	0.043	0.037
3.5.4.26|g__Dorea.s__Dorea_longicatena	-	3.55E-02	0.566	0.000	-0.566	0.013	0.011
3.5.4.26|unclassified	-	4.48E-01	9.064	8.759	-0.305	0.113	0.120
3.5.4.26|g__Anaerostipes.s__Anaerostipes_hadrus	-	8.05E-04	0.491	0.237	-0.254	0.009	0.008
3.5.4.26|g__Roseburia.s__Roseburia_hominis	-	2.82E-01	1.503	1.273	-0.231	0.018	0.019
3.5.4.26|g__Bacteroides.s__Bacteroides_ovatus	-	7.35E-01	0.616	0.571	-0.045	0.022	0.020
3.5.4.26|g__Lachnoclostridium.s__Clostridium_bolteae	-	4.79E-01	0.044	0.000	-0.044	0.003	0.004
3.5.4.26|g__Asaccharobacter.s__Asaccharobacter_celatus	-	5.76E-01	0.070	0.051	-0.019	0.004	0.004
3.5.4.26|g__Acidaminococcus.s__Acidaminococcus_fermentans	+	2.32E-01	0.000	0.000	0.000	0.000	0.000
3.5.4.26|g__Acidaminococcus.s__Acidaminococcus_intestini	+	1.74E-03	0.000	0.000	0.000	0.007	0.013
3.5.4.26|g__Acidipropionibacterium.s__Acidipropionibacterium_acidipropic	+	4.92E-01	0.000	0.000	0.000	0.000	0.000
3.5.4.26|g__Actinomyces.s__Actinomyces_naeslundii	+	6.68E-02	0.000	0.000	0.000	0.000	0.000
3.5.4.26|g__Actinomyces.s__Actinomyces_oris	+	2.38E-02	0.000	0.000	0.000	0.000	0.000
3.5.4.26|g__Actinomyces.s__Actinomyces_sp_oral_taxon_448	+	3.30E-01	0.000	0.000	0.000	0.000	0.000
3.5.4.26|g__Actinomyces.s__Actinomyces_viscosus	+	3.09E-01	0.000	0.000	0.000	0.000	0.000
3.5.4.26|g__Adlercreutzia.s__Adlercreutzia_equolifaciens	-	4.59E-01	0.000	0.000	0.000	0.001	0.001
3.5.4.26|g__Aeromonas.s__Aeromonas_allosaccharophila	-	1.48E-01	0.000	0.000	0.000	0.000	0.000
3.5.4.26|g__Aeromonas.s__Aeromonas_diversa	-	1.48E-01	0.000	0.000	0.000	0.000	0.000
3.5.4.26|g__Aeromonas.s__Aeromonas_sobria	-	1.48E-01	0.000	0.000	0.000	0.000	0.000
3.5.4.26|g__Aeromonas.s__Aeromonas_veronii	-	1.48E-01	0.000	0.000	0.000	0.000	0.000
3.5.4.26|g__Agathobaculum.s__Agathobaculum_butyriciproducens	-	4.82E-03	0.000	0.000	0.000	0.003	0.002
3.5.4.26|g__Akkermansia.s__Akkermansia_muciniphila	+	1.77E-01	0.000	0.000	0.000	0.023	0.042
3.5.4.26|g__Allisonella.s__Allisonella_histaminiformans	-	6.68E-01	0.000	0.000	0.000	0.000	0.000
3.5.4.26|g__Anaerococcus.s__Anaerococcus_hydrogenalis	+	4.92E-01	0.000	0.000	0.000	0.000	0.000
3.5.4.26|g__Anaerococcus.s__Anaerococcus_octavius	+	4.92E-01	0.000	0.000	0.000	0.000	0.000
3.5.4.26|g__Anaerococcus.s__Anaerococcus_vaginalis	+	2.32E-01	0.000	0.000	0.000	0.000	0.000
3.5.4.26|g__Anaeroglobus.s__Anaeroglobus_geminatus	-	5.50E-01	0.000	0.000	0.000	0.000	0.000
3.5.4.26|g__Anaeromassilibacillus.s__Anaeromassilibacillus_sp_An250	-	4.19E-01	0.000	0.000	0.000	0.000	0.000
3.5.4.26|g__Anaerostipes.s__Anaerostipes_caccae	+	4.59E-01	0.000	0.000	0.000	0.001	0.001
3.5.4.26|g__Anaerostipes.s__Anaerostipes_sp_494a	-	1.48E-01	0.000	0.000	0.000	0.000	0.000
3.5.4.26|g__Anaerotignum.s__Anaerotignum_lactatifermentans	-	1.78E-01	0.000	0.000	0.000	0.002	0.002
3.5.4.26|g__Bacillus.s__Bacillus_horneckiae	-	1.48E-01	0.000	0.000	0.000	0.000	0.000
3.5.4.26|g__Bacteroidales_unclassified.s__Bacteroidales_bacterium_KA003	+	4.92E-01	0.000	0.000	0.000	0.000	0.000
3.5.4.26|g__Bacteroides.s__Bacteroides_clarus	+	8.36E-01	0.000	0.000	0.000	0.001	0.001
3.5.4.26|g__Bacteroides.s__Bacteroides_coprocola	-	1.03E-01	0.000	0.000	0.000	0.004	0.002
3.5.4.26|g__Bacteroides.s__Bacteroides_coprophilus	+	4.07E-01	0.000	0.000	0.000	0.000	0.000
3.5.4.26|g__Bacteroides.s__Bacteroides_eggerthii	+	6.53E-01	0.000	0.000	0.000	0.014	0.013
3.5.4.26|g__Bacteroides.s__Bacteroides_faecichinchillae	+	4.92E-01	0.000	0.000	0.000	0.000	0.000
3.5.4.26|g__Bacteroides.s__Bacteroides_faecis	+	1.40E-01	0.000	0.000	0.000	0.003	0.004
3.5.4.26|g__Bacteroides.s__Bacteroides_finegoldii	+	3.98E-01	0.000	0.000	0.000	0.002	0.003
3.5.4.26|g__Bacteroides.s__Bacteroides_fluxus	+	2.32E-01	0.000	0.000	0.000	0.000	0.000
3.5.4.26|g__Bacteroides.s__Bacteroides_fragilis	-	6.40E-01	0.000	0.000	0.000	0.007	0.008
3.5.4.26|g__Bacteroides.s__Bacteroides_massiliensis	+	4.99E-01	0.000	0.000	0.000	0.011	0.009
3.5.4.26|g__Bacteroides.s__Bacteroides_nordii	-	6.57E-01	0.000	0.000	0.000	0.001	0.001
3.5.4.26|g__Bacteroides.s__Bacteroides_plebeius	-	2.70E-01	0.000	0.000	0.000	0.014	0.005
3.5.4.26|g__Bacteroides.s__Bacteroides_salyersiae	+	3.36E-02	0.000	0.000	0.000	0.001	0.002
3.5.4.26|g__Bacteroides.s__Bacteroides_sartorii	-	1.48E-01	0.000	0.000	0.000	0.000	0.000
3.5.4.26|g__Bacteroides.s__Bacteroides_sp_OM08_11	+	4.92E-01	0.000	0.000	0.000	0.000	0.000
3.5.4.26|g__Bacteroides.s__Bacteroides_stercorirosoris	+	7.57E-01	0.000	0.000	0.000	0.000	0.000
3.5.4.26|g__Bacteroides.s__Bacteroides_xylanisolvens	+	2.05E-01	0.000	0.000	0.000	0.010	0.009
3.5.4.26|g__Barnesiella.s__Barnesiella_intestinihominis	+	7.67E-02	0.000	0.000	0.000	0.005	0.007
3.5.4.26|g__Bifidobacterium.s__Bifidobacterium_longum	+	2.32E-01	0.000	0.000	0.000	0.000	0.000
3.5.4.26|g__Blautia.s__Blautia_hansenii	-	8.56E-02	0.000	0.000	0.000	0.002	0.001
3.5.4.26|g__Blautia.s__Blautia_sp_AF19_10LB	+	5.57E-01	0.000	0.000	0.000	0.001	0.001
3.5.4.26|g__Blautia.s__Blautia_sp_An249	-	4.29E-01	0.000	0.000	0.000	0.000	0.000
3.5.4.26|g__Brevibacterium.s__Brevibacterium_ravenspurgense	+	4.92E-01	0.000	0.000	0.000	0.000	0.000
3.5.4.26|g__Butyricicoccus.s__Butyricicoccus_pullicaecorum	-	3.11E-02	0.000	0.000	0.000	0.000	0.000
3.5.4.26|g__Butyricimonas.s__Butyricimonas_synergistica	-	1.48E-01	0.000	0.000	0.000	0.000	0.000
3.5.4.26|g__Butyricimonas.s__Butyricimonas_virosa	+	1.14E-01	0.000	0.000	0.000	0.000	0.001
3.5.4.26|g__Butyrivibrio.s__Butyrivibrio_crossotus	-	6.16E-01	0.000	0.000	0.000	0.002	0.001
3.5.4.26|g__Campylobacter.s__Campylobacter_curvus	+	4.92E-01	0.000	0.000	0.000	0.000	0.000
3.5.4.26|g__Campylobacter.s__Campylobacter_gracilis	+	1.67E-01	0.000	0.000	0.000	0.000	0.000
3.5.4.26|g__Campylobacter.s__Campylobacter_upsaliensis	+	4.92E-01	0.000	0.000	0.000	0.000	0.000
3.5.4.26|g__Campylobacter.s__Campylobacter_ureolyticus	+	4.92E-01	0.000	0.000	0.000	0.000	0.000
3.5.4.26|g__Candidatus_Gastranaerophilales_unclassified.s__Candidatus_Ga	+	4.92E-01	0.000	0.000	0.000	0.000	0.000
3.5.4.26|g__Candidatus_Methanomethylophilus.s__Candidatus_Methanome	+	3.30E-01	0.000	0.000	0.000	0.000	0.000
3.5.4.26|g__Catenibacterium.s__Catenibacterium_mitsuokai	+	5.33E-01	0.000	0.000	0.000	0.002	0.004
3.5.4.26|g__Cellulosilyticum.s__Cellulosilyticum_lentocellum	+	4.92E-01	0.000	0.000	0.000	0.000	0.000
3.5.4.26|g__Cetobacterium.s__Cetobacterium_somerae	+	4.92E-01	0.000	0.000	0.000	0.000	0.000

3.5.4.26|g__Citrobacter.s__Citrobacter_amalonaticus	+	1.67E-01	0.000	0.000	0.000	0.000	0.000
3.5.4.26|g__Citrobacter.s__Citrobacter_braakii	-	9.67E-01	0.000	0.000	0.000	0.000	0.000
3.5.4.26|g__Citrobacter.s__Citrobacter_farmeri	+	1.67E-01	0.000	0.000	0.000	0.000	0.000
3.5.4.26|g__Citrobacter.s__Citrobacter_freundii	+	5.83E-01	0.000	0.000	0.000	0.001	0.000
3.5.4.26|g__Citrobacter.s__Citrobacter_koseri	+	3.30E-01	0.000	0.000	0.000	0.000	0.000
3.5.4.26|g__Citrobacter.s__Citrobacter_portucalensis	+	2.32E-01	0.000	0.000	0.000	0.000	0.000
3.5.4.26|g__Citrobacter.s__Citrobacter_werkmanii	+	4.92E-01	0.000	0.000	0.000	0.000	0.000
3.5.4.26|g__Citrobacter.s__Citrobacter_youngae	+	5.73E-02	0.000	0.000	0.000	0.000	0.000
3.5.4.26|g__Cloacibacillus.s__Cloacibacillus_porcorum	+	5.73E-02	0.000	0.000	0.000	0.000	0.001
3.5.4.26|g__Clostridiales_Family_XIII_Incertae_Sedis_unclassified.s__Euba	+	4.92E-01	0.000	0.000	0.000	0.000	0.000
3.5.4.26|g__Clostridiales_unclassified.s__Clostridiales_bacterium_1_7_47F/	-	7.41E-01	0.000	0.000	0.000	0.000	0.000
3.5.4.26|g__Clostridioides.s__Clostridioides_difficile	+	5.40E-03	0.000	0.000	0.000	0.003	0.003
3.5.4.26|g__Clostridium.s__Butyribacterium_methylotrophicum	+	3.43E-05	0.000	0.000	0.000	0.000	0.001
3.5.4.26|g__Clostridium.s__Clostridium_butyricum	-	1.48E-01	0.000	0.000	0.000	0.000	0.000
3.5.4.26|g__Clostridium.s__Clostridium_cadaveris	-	5.95E-01	0.000	0.000	0.000	0.000	0.000
3.5.4.26|g__Clostridium.s__Clostridium_celatum	-	9.67E-01	0.000	0.000	0.000	0.000	0.000
3.5.4.26|g__Clostridium.s__Clostridium_disporicum	-	5.45E-01	0.000	0.000	0.000	0.000	0.000
3.5.4.26|g__Clostridium.s__Clostridium_perfringens	-	9.67E-01	0.000	0.000	0.000	0.000	0.000
3.5.4.26|g__Clostridium.s__Clostridium_sp_AF36_4	-	4.12E-01	0.000	0.000	0.000	0.003	0.003
3.5.4.26|g__Clostridium.s__Clostridium_sp_AM22_11AC	-	7.47E-03	0.000	0.000	0.000	0.003	0.002
3.5.4.26|g__Clostridium.s__Clostridium_sp_MSTE9	+	3.30E-01	0.000	0.000	0.000	0.000	0.000
3.5.4.26|g__Clostridium.s__Clostridium_sp_chh4_2	-	7.08E-01	0.000	0.000	0.000	0.000	0.000
3.5.4.26|g__Clostridium.s__Clostridium_ventriculi	+	7.60E-01	0.000	0.000	0.000	0.000	0.000
3.5.4.26|g__Comamonas.s__Comamonas_kerstersii	+	3.30E-01	0.000	0.000	0.000	0.000	0.000
3.5.4.26|g__Coprobacillus.s__Coprobacillus_cateniformis	+	1.92E-05	0.000	0.000	0.000	0.000	0.001
3.5.4.26|g__Coprobacter.s__Coprobacter_fastidiosus	-	9.56E-03	0.000	0.000	0.000	0.001	0.001
3.5.4.26|g__Coprobacter.s__Coprobacter_secundus	-	3.47E-01	0.000	0.000	0.000	0.000	0.000
3.5.4.26|g__Coprococcus.s__Coprococcus_catus	+	7.35E-01	0.000	0.000	0.000	0.002	0.002
3.5.4.26|g__Coprococcus.s__Coprococcus_comes	-	7.43E-01	0.000	0.000	0.000	0.005	0.006
3.5.4.26|g__Coprococcus.s__Coprococcus_eutactus	-	5.33E-01	0.000	0.000	0.000	0.003	0.002
3.5.4.26|g__Corynebacterium.s__Corynebacterium_amycolatum	+	4.92E-01	0.000	0.000	0.000	0.000	0.000
3.5.4.26|g__Corynebacterium.s__Corynebacterium_aurimucosum	+	4.92E-01	0.000	0.000	0.000	0.000	0.000
3.5.4.26|g__Corynebacterium.s__Corynebacterium_coyleae	+	4.92E-01	0.000	0.000	0.000	0.000	0.000
3.5.4.26|g__Corynebacterium.s__Corynebacterium_freneyi	+	4.92E-01	0.000	0.000	0.000	0.000	0.000
3.5.4.26|g__Corynebacterium.s__Corynebacterium_imitans	+	4.92E-01	0.000	0.000	0.000	0.000	0.000
3.5.4.26|g__Corynebacterium.s__Corynebacterium_jeikeium	+	3.30E-01	0.000	0.000	0.000	0.000	0.000
3.5.4.26|g__Corynebacterium.s__Corynebacterium_kroppenstedtii	+	4.92E-01	0.000	0.000	0.000	0.000	0.000
3.5.4.26|g__Corynebacterium.s__Corynebacterium_minutissimum	+	4.92E-01	0.000	0.000	0.000	0.000	0.000
3.5.4.26|g__Corynebacterium.s__Corynebacterium_pseudogenitalium	+	4.92E-01	0.000	0.000	0.000	0.000	0.000
3.5.4.26|g__Corynebacterium.s__Corynebacterium_riegelii	+	4.92E-01	0.000	0.000	0.000	0.000	0.000
3.5.4.26|g__Corynebacterium.s__Corynebacterium_sp_HMSC08A12	+	4.92E-01	0.000	0.000	0.000	0.000	0.000
3.5.4.26|g__Corynebacterium.s__Corynebacterium_tuberculostearicum	+	4.92E-01	0.000	0.000	0.000	0.000	0.000
3.5.4.26|g__Corynebacterium.s__Corynebacterium_urealyticum	+	4.92E-01	0.000	0.000	0.000	0.000	0.000
3.5.4.26|g__Cronobacter.s__Cronobacter_malonaticus	+	4.92E-01	0.000	0.000	0.000	0.000	0.000
3.5.4.26|g__Cronobacter.s__Cronobacter_sakazakii	+	4.92E-01	0.000	0.000	0.000	0.000	0.000
3.5.4.26|g__Cutibacterium.s__Cutibacterium_avidum	+	4.92E-01	0.000	0.000	0.000	0.000	0.000
3.5.4.26|g__Desulfovibrio.s__Desulfovibrio_desulfuricans	+	1.67E-01	0.000	0.000	0.000	0.000	0.000
3.5.4.26|g__Desulfovibrio.s__Desulfovibrio_fairfieldensis	+	4.42E-01	0.000	0.000	0.000	0.001	0.002
3.5.4.26|g__Desulfovibrio.s__Desulfovibrio_legallii	+	4.92E-01	0.000	0.000	0.000	0.000	0.000
3.5.4.26|g__Desulfovibrio.s__Desulfovibrio_piger	+	3.47E-01	0.000	0.000	0.000	0.000	0.001
3.5.4.26|g__Desulfovibrio.s__Desulfovibrio_sp_AM18_2	+	1.71E-01	0.000	0.000	0.000	0.000	0.000
3.5.4.26|g__Desulfovibrionaceae_unclassified.s__Desulfovibrionaceae_bact	+	7.41E-02	0.000	0.000	0.000	0.000	0.000
3.5.4.26|g__Dialister.s__Dialister_pneumosintes	+	4.92E-01	0.000	0.000	0.000	0.000	0.000
3.5.4.26|g__Dialister.s__Dialister_succinatiphilus	+	4.92E-01	0.000	0.000	0.000	0.000	0.000
3.5.4.26|g__Dorea.s__Dorea_formicigenerans	-	6.72E-01	0.000	0.000	0.000	0.002	0.003
3.5.4.26|g__Dorea.s__Dorea_sp_OM02_2LB	+	1.14E-01	0.000	0.000	0.000	0.000	0.000
3.5.4.26|g__Dysgonomonas.s__Dysgonomonas_mossii	-	9.73E-01	0.000	0.000	0.000	0.000	0.000
3.5.4.26|g__Eggerthella.s__Eggerthella_lenta	+	5.05E-01	0.000	0.000	0.000	0.003	0.002
3.5.4.26|g__Enterobacter.s__Enterobacter_bugandensis	+	5.60E-01	0.000	0.000	0.000	0.000	0.000
3.5.4.26|g__Enterobacter.s__Enterobacter_cloacae	+	8.00E-02	0.000	0.000	0.000	0.000	0.000
3.5.4.26|g__Enterobacter.s__Enterobacter_mori	+	4.92E-01	0.000	0.000	0.000	0.000	0.000
3.5.4.26|g__Enterococcus.s__Enterococcus_avium	+	8.01E-06	0.000	0.000	0.000	0.000	0.001
3.5.4.26|g__Enterococcus.s__Enterococcus_casseliflavus	+	1.67E-01	0.000	0.000	0.000	0.000	0.000
3.5.4.26|g__Enterococcus.s__Enterococcus_faecalis	+	4.92E-01	0.000	0.000	0.000	0.000	0.000
3.5.4.26|g__Enterococcus.s__Enterococcus_faecium	+	4.60E-02	0.000	0.000	0.000	0.000	0.000
3.5.4.26|g__Enterococcus.s__Enterococcus_gallinarum	+	4.01E-03	0.000	0.000	0.000	0.000	0.000
3.5.4.26|g__Enterococcus.s__Enterococcus_malodoratus	+	4.92E-01	0.000	0.000	0.000	0.000	0.000
3.5.4.26|g__Enterococcus.s__Enterococcus_mundtii	+	4.92E-01	0.000	0.000	0.000	0.000	0.000
3.5.4.26|g__Enterococcus.s__Enterococcus_saccharolyticus	+	5.28E-03	0.000	0.000	0.000	0.000	0.000
3.5.4.26|g__Enterococcus.s__Enterococcus_thailandicus	+	4.92E-01	0.000	0.000	0.000	0.000	0.000
3.5.4.26|g__Erysipelatoclostridium.s__Clostridium_innocuum	+	7.69E-03	0.000	0.000	0.000	0.003	0.003
3.5.4.26|g__Erysipelatoclostridium.s__Clostridium_spiroforme	+	4.48E-01	0.000	0.000	0.000	0.002	0.000

3.5.4.26|g__Erysipelotrichaceae_unclassified.s__Erysipelotrichaceae_bacteri	+	9.01E-01	0.000	0.000	0.000	0.001	0.000
3.5.4.26|g__Escherichia.s__Escherichia_coli	+	1.16E-02	0.000	0.000	0.000	0.009	0.009
3.5.4.26|g__Escherichia.s__Escherichia_fergusonii	+	5.15E-01	0.000	0.000	0.000	0.000	0.000
3.5.4.26|g__Escherichia.s__Escherichia_marmotae	+	4.92E-01	0.000	0.000	0.000	0.000	0.000
3.5.4.26|g__Eubacterium.s__Eubacterium_callanderi	+	6.36E-05	0.000	0.000	0.000	0.000	0.001
3.5.4.26|g__Eubacterium.s__Eubacterium_eligens	-	9.71E-02	0.000	0.000	0.000	0.006	0.005
3.5.4.26|g__Eubacterium.s__Eubacterium_limosum	+	1.12E-04	0.000	0.000	0.000	0.000	0.000
3.5.4.26|g__Eubacterium.s__Eubacterium_maltosivorans	+	1.50E-01	0.000	0.000	0.000	0.000	0.000
3.5.4.26|g__Eubacterium.s__Eubacterium_ramulus	-	5.79E-02	0.000	0.000	0.000	0.003	0.002
3.5.4.26|g__Eubacterium.s__Eubacterium_sp_AF17_7	-	4.85E-01	0.000	0.000	0.000	0.000	0.000
3.5.4.26|g__Eubacterium.s__Eubacterium_sp_AM18_10LB_B	-	2.67E-01	0.000	0.000	0.000	0.001	0.000
3.5.4.26|g__Eubacterium.s__Eubacterium_sp_An11	+	6.61E-01	0.000	0.000	0.000	0.000	0.001
3.5.4.26|g__Faecalicatena.s__Faecalicatena_contorta	+	1.25E-02	0.000	0.000	0.000	0.000	0.000
3.5.4.26|g__Firmicutes_unclassified.s__Firmicutes_bacterium_AM10_47	-	4.98E-01	0.000	0.000	0.000	0.000	0.000
3.5.4.26|g__Flavonifractor.s__Flavonifractor_plautii	+	8.63E-02	0.000	0.000	0.000	0.001	0.001
3.5.4.26|g__Fusobacterium.s__Fusobacterium_mortiferum	-	7.14E-01	0.000	0.000	0.000	0.000	0.000
3.5.4.26|g__Fusobacterium.s__Fusobacterium_naviforme	+	4.92E-01	0.000	0.000	0.000	0.000	0.000
3.5.4.26|g__Fusobacterium.s__Fusobacterium_nucleatum	-	9.70E-01	0.000	0.000	0.000	0.000	0.000
3.5.4.26|g__Fusobacterium.s__Fusobacterium_ulcerans	-	3.55E-01	0.000	0.000	0.000	0.000	0.000
3.5.4.26|g__Fusobacterium.s__Fusobacterium_varium	+	4.92E-01	0.000	0.000	0.000	0.000	0.000
3.5.4.26|g__Gemella.s__Gemella_haemolysans	+	4.92E-01	0.000	0.000	0.000	0.000	0.000
3.5.4.26|g__Haemophilus.s__Haemophilus_parainfluenzae	-	7.87E-01	0.000	0.000	0.000	0.000	0.000
3.5.4.26|g__Hafnia.s__Hafnia_paralvei	-	2.03E-01	0.000	0.000	0.000	0.000	0.000
3.5.4.26|g__Holdemanella.s__Holdemanella_biformis	-	2.12E-01	0.000	0.000	0.000	0.000	0.000
3.5.4.26|g__Intestinibacter.s__Intestinibacter_bartlettii	-	7.04E-01	0.000	0.000	0.000	0.000	0.000
3.5.4.26|g__Klebsiella.s__Klebsiella_aerogenes	+	1.22E-01	0.000	0.000	0.000	0.000	0.000
3.5.4.26|g__Klebsiella.s__Klebsiella_grimontii	+	3.30E-01	0.000	0.000	0.000	0.000	0.000
3.5.4.26|g__Klebsiella.s__Klebsiella_michiganensis	+	3.09E-01	0.000	0.000	0.000	0.000	0.000
3.5.4.26|g__Klebsiella.s__Klebsiella_oxytoca	+	5.53E-02	0.000	0.000	0.000	0.001	0.002
3.5.4.26|g__Klebsiella.s__Klebsiella_pneumoniae	+	1.16E-03	0.000	0.000	0.000	0.006	0.007
3.5.4.26|g__Klebsiella.s__Klebsiella_variicola	+	1.64E-03	0.000	0.000	0.000	0.000	0.002
3.5.4.26|g__Kluyvera.s__Kluyvera_ascorbata	-	9.73E-01	0.000	0.000	0.000	0.000	0.000
3.5.4.26|g__Kluyvera.s__Kluyvera_cryocrescens	+	3.30E-01	0.000	0.000	0.000	0.000	0.000
3.5.4.26|g__Kosakonia.s__Kosakonia_cowanii	+	4.92E-01	0.000	0.000	0.000	0.000	0.000
3.5.4.26|g__Lachnoclostridium.s__Clostridium_aldenense	+	6.27E-01	0.000	0.000	0.000	0.001	0.001
3.5.4.26|g__Lachnoclostridium.s__Clostridium_citroniae	+	2.73E-02	0.000	0.000	0.000	0.000	0.000
3.5.4.26|g__Lachnoclostridium.s__Clostridium_clostridioforme	-	3.84E-01	0.000	0.000	0.000	0.003	0.006
3.5.4.26|g__Lachnoclostridium.s__Clostridium_symbiosum	-	9.53E-01	0.000	0.000	0.000	0.002	0.002
3.5.4.26|g__Lachnospira.s__Lachnospira_pectinoschiza	-	4.91E-01	0.000	0.000	0.000	0.002	0.002
3.5.4.26|g__Lactobacillus.s__Lactobacillus_amylovorus	-	1.48E-01	0.000	0.000	0.000	0.000	0.000
3.5.4.26|g__Lactobacillus.s__Lactobacillus_brevis	+	4.92E-01	0.000	0.000	0.000	0.000	0.000
3.5.4.26|g__Lactobacillus.s__Lactobacillus_crispatus	+	1.22E-01	0.000	0.000	0.000	0.000	0.000
3.5.4.26|g__Lactobacillus.s__Lactobacillus_delbrueckii	-	4.06E-02	0.000	0.000	0.000	0.000	0.000
3.5.4.26|g__Lactobacillus.s__Lactobacillus_fermentum	+	4.99E-02	0.000	0.000	0.000	0.000	0.001
3.5.4.26|g__Lactobacillus.s__Lactobacillus_gastricus	+	4.92E-01	0.000	0.000	0.000	0.000	0.000
3.5.4.26|g__Lactobacillus.s__Lactobacillus_kimbladii	+	4.92E-01	0.000	0.000	0.000	0.000	0.000
3.5.4.26|g__Lactobacillus.s__Lactobacillus_kullabergensis	+	4.92E-01	0.000	0.000	0.000	0.000	0.000
3.5.4.26|g__Lactobacillus.s__Lactobacillus_oris	+	8.53E-03	0.000	0.000	0.000	0.001	0.001
3.5.4.26|g__Lactobacillus.s__Lactobacillus_pentosus	+	3.05E-01	0.000	0.000	0.000	0.000	0.000
3.5.4.26|g__Lactobacillus.s__Lactobacillus_plantarum	+	2.48E-01	0.000	0.000	0.000	0.000	0.000
3.5.4.26|g__Lactobacillus.s__Lactobacillus_reuteri	+	7.63E-02	0.000	0.000	0.000	0.000	0.002
3.5.4.26|g__Lactobacillus.s__Lactobacillus_rogosae	-	5.65E-01	0.000	0.000	0.000	0.002	0.002
3.5.4.26|g__Lactococcus.s__Lactococcus_lactis	-	7.40E-01	0.000	0.000	0.000	0.000	0.000
3.5.4.26|g__Lactonifactor.s__Lactonifactor_longoviformis	+	4.92E-01	0.000	0.000	0.000	0.000	0.000
3.5.4.26|g__Leclercia.s__Leclercia_adecarboxylata	+	4.15E-01	0.000	0.000	0.000	0.000	0.000
3.5.4.26|g__Lelliottia.s__Lelliottia_nimipressuralis	+	1.30E-01	0.000	0.000	0.000	0.000	0.000
3.5.4.26|g__Leuconostoc.s__Leuconostoc_citreum	+	4.92E-01	0.000	0.000	0.000	0.000	0.000
3.5.4.26|g__Leuconostoc.s__Leuconostoc_garlicum	+	4.92E-01	0.000	0.000	0.000	0.000	0.000
3.5.4.26|g__Leuconostoc.s__Leuconostoc_lactis	+	4.92E-01	0.000	0.000	0.000	0.000	0.000
3.5.4.26|g__Leuconostoc.s__Leuconostoc_mesenteroides	-	5.95E-01	0.000	0.000	0.000	0.000	0.000
3.5.4.26|g__Listeria.s__Listeria_monocytogenes	+	1.70E-01	0.000	0.000	0.000	0.000	0.001
3.5.4.26|g__Megamonas.s__Megamonas_funiformis	+	6.48E-01	0.000	0.000	0.000	0.000	0.000
3.5.4.26|g__Megamonas.s__Megamonas_hypermegale	+	6.76E-01	0.000	0.000	0.000	0.000	0.000
3.5.4.26|g__Megamonas.s__Megamonas_rupellensis	+	6.73E-01	0.000	0.000	0.000	0.000	0.000
3.5.4.26|g__Megasphaera.s__Megasphaera_elsdenii	-	9.62E-01	0.000	0.000	0.000	0.000	0.000
3.5.4.26|g__Megasphaera.s__Megasphaera_hexanoica	+	4.92E-01	0.000	0.000	0.000	0.000	0.000
3.5.4.26|g__Megasphaera.s__Megasphaera_micronuciformis	+	2.32E-01	0.000	0.000	0.000	0.000	0.000
3.5.4.26|g__Megasphaera.s__Megasphaera_stantonii	-	5.92E-01	0.000	0.000	0.000	0.000	0.000
3.5.4.26|g__Mesosutterella.s__Mesosutterella_multiformis	+	2.02E-02	0.000	0.000	0.000	0.001	0.001
3.5.4.26|g__Mitsuokella.s__Mitsuokella_jalaludinii	-	5.92E-01	0.000	0.000	0.000	0.000	0.000
3.5.4.26|g__Mitsuokella.s__Mitsuokella_multacida	+	4.92E-01	0.000	0.000	0.000	0.000	0.000
3.5.4.26|g__Morganella.s__Morganella_morganii	+	3.74E-02	0.000	0.000	0.000	0.000	0.000

3.5.4.26|g__Muribaculum.s__Muribaculum_intestinale	-	5.92E-01	0.000	0.000	0.000	0.000	0.000
3.5.4.26|g__Odoribacter.s__Odoribacter_laneus	+	4.49E-01	0.000	0.000	0.000	0.001	0.004
3.5.4.26|g__Oligella.s__Oligella_urethralis	+	4.92E-01	0.000	0.000	0.000	0.000	0.000
3.5.4.26|g__Oxalobacter.s__Oxalobacter_formigenes	+	3.59E-01	0.000	0.000	0.000	0.000	0.000
3.5.4.26|g__Pantoea.s__Pantoea_sesami	+	2.32E-01	0.000	0.000	0.000	0.000	0.000
3.5.4.26|g__Parabacteroides.s__Parabacteroides_goldsteinii	+	4.65E-02	0.000	0.000	0.000	0.001	0.002
3.5.4.26|g__Parabacteroides.s__Parabacteroides_gordonii	-	3.94E-01	0.000	0.000	0.000	0.000	0.000
3.5.4.26|g__Parabacteroides.s__Parabacteroides_johnsonii	-	4.15E-01	0.000	0.000	0.000	0.001	0.001
3.5.4.26|g__Paraprevotella.s__Paraprevotella_clara	-	1.42E-01	0.000	0.000	0.000	0.004	0.003
3.5.4.26|g__Paraprevotella.s__Paraprevotella_xylaniphila	+	4.16E-01	0.000	0.000	0.000	0.000	0.000
3.5.4.26|g__Parasutterella.s__Parasutterella_excrementihominis	-	3.71E-01	0.000	0.000	0.000	0.003	0.003
3.5.4.26|g__Pediococcus.s__Pediococcus_acidilactici	+	2.12E-02	0.000	0.000	0.000	0.000	0.001
3.5.4.26|g__Pedobacter.s__Pedobacter_himalayensis	+	5.53E-01	0.000	0.000	0.000	0.000	0.000
3.5.4.26|g__Peptococcus.s__Peptococcus_niger	+	3.06E-01	0.000	0.000	0.000	0.000	0.000
3.5.4.26|g__Peptoniphilus.s__Peptoniphilus_harei	+	4.92E-01	0.000	0.000	0.000	0.000	0.000
3.5.4.26|g__Peptoniphilus.s__Peptoniphilus_lacrimalis	+	4.92E-01	0.000	0.000	0.000	0.000	0.000
3.5.4.26|g__Peptoniphilus.s__Peptoniphilus_sp_HMSC062D09	+	3.30E-01	0.000	0.000	0.000	0.000	0.000
3.5.4.26|g__Peptoniphilus.s__Peptoniphilus_sp_oral_taxon_375	+	4.92E-01	0.000	0.000	0.000	0.000	0.000
3.5.4.26|g__Peptostreptococcaceae_unclassified.s__Clostridium_hiranonis	-	5.92E-01	0.000	0.000	0.000	0.000	0.000
3.5.4.26|g__Peptostreptococcus.s__Peptostreptococcus_stomatis	+	3.30E-01	0.000	0.000	0.000	0.000	0.000
3.5.4.26|g__Phascolarctobacterium.s__Phascolarctobacterium_succinatutens	+	1.81E-01	0.000	0.000	0.000	0.001	0.002
3.5.4.26|g__Pluralibacter.s__Pluralibacter_gergoviae	-	1.48E-01	0.000	0.000	0.000	0.000	0.000
3.5.4.26|g__Porphyromonas.s__Porphyromonas_asaccharolytica	+	1.31E-01	0.000	0.000	0.000	0.000	0.000
3.5.4.26|g__Porphyromonas.s__Porphyromonas_sp_HMSC065F10	+	5.53E-01	0.000	0.000	0.000	0.000	0.000
3.5.4.26|g__Porphyromonas.s__Porphyromonas_sp_HMSC077F02	+	5.55E-01	0.000	0.000	0.000	0.000	0.000
3.5.4.26|g__Porphyromonas.s__Porphyromonas_uenonis	-	5.95E-01	0.000	0.000	0.000	0.000	0.000
3.5.4.26|g__Prevotella.s__Prevotella_buccae	+	3.30E-01	0.000	0.000	0.000	0.000	0.000
3.5.4.26|g__Prevotella.s__Prevotella_buccalis	+	3.74E-02	0.000	0.000	0.000	0.000	0.000
3.5.4.26|g__Prevotella.s__Prevotella_colorans	+	3.30E-01	0.000	0.000	0.000	0.000	0.000
3.5.4.26|g__Prevotella.s__Prevotella_copri	-	1.47E-01	0.000	0.000	0.000	0.040	0.016
3.5.4.26|g__Prevotella.s__Prevotella_corporis	+	2.32E-01	0.000	0.000	0.000	0.000	0.000
3.5.4.26|g__Prevotella.s__Prevotella_disiens	+	8.36E-01	0.000	0.000	0.000	0.000	0.000
3.5.4.26|g__Prevotella.s__Prevotella_sp_109	-	7.83E-01	0.000	0.000	0.000	0.003	0.002
3.5.4.26|g__Prevotella.s__Prevotella_sp_AM42_24	-	2.20E-02	0.000	0.000	0.000	0.002	0.000
3.5.4.26|g__Propionibacterium.s__Propionibacterium_acidifaciens	+	4.92E-01	0.000	0.000	0.000	0.000	0.000
3.5.4.26|g__Proteus.s__Proteus_mirabilis	+	4.12E-01	0.000	0.000	0.000	0.000	0.000
3.5.4.26|g__Pseudocitrobacter.s__Pseudocitrobacter_faecalis	+	4.92E-01	0.000	0.000	0.000	0.000	0.000
3.5.4.26|g__Pseudoflavonifractor.s__Pseudoflavonifractor_sp_An184	+	9.97E-02	0.000	0.000	0.000	0.000	0.000
3.5.4.26|g__Pseudomonas.s__Pseudomonas_aeruginosa	+	2.33E-01	0.000	0.000	0.000	0.000	0.000
3.5.4.26|g__Pseudomonas.s__Pseudomonas_aeruginosa_group	+	2.33E-01	0.000	0.000	0.000	0.000	0.000
3.5.4.26|g__Pyramidobacter.s__Pyramidobacter_piscolens	+	2.43E-01	0.000	0.000	0.000	0.000	0.001
3.5.4.26|g__Pyramidobacter.s__Pyramidobacter_sp_C12_8	+	4.92E-01	0.000	0.000	0.000	0.000	0.000
3.5.4.26|g__Raoultella.s__Raoultella_ornithinolytica	-	2.03E-01	0.000	0.000	0.000	0.000	0.000
3.5.4.26|g__Raoultella.s__Raoultella_planticola	-	4.06E-02	0.000	0.000	0.000	0.000	0.000
3.5.4.26|g__Rikenella.s__Rikenella_microfusus	+	4.92E-01	0.000	0.000	0.000	0.000	0.000
3.5.4.26|g__Roseburia.s__Roseburia_faecis	-	7.78E-01	0.000	0.000	0.000	0.009	0.007
3.5.4.26|g__Roseburia.s__Roseburia_intestinalis	-	5.41E-05	0.000	0.000	0.000	0.003	0.001
3.5.4.26|g__Roseburia.s__Roseburia_inulinivorans	-	5.38E-02	0.000	0.000	0.000	0.002	0.002
3.5.4.26|g__Ruminococcaceae_unclassified.s__Ruminococcaceae_bacteriun	+	3.25E-01	0.000	0.000	0.000	0.001	0.002
3.5.4.26|g__Ruminococcaceae_unclassified.s__Ruminococcaceae_bacteriun	+	6.92E-03	0.000	0.000	0.000	0.000	0.001
3.5.4.26|g__Ruminococcus.s__Ruminococcus_callidus	-	4.60E-04	0.000	0.000	0.000	0.003	0.001
3.5.4.26|g__Ruminococcus.s__Ruminococcus_sp_AF31_8BH	-	9.45E-03	0.000	0.000	0.000	0.002	0.001
3.5.4.26|g__Salmonella.s__Salmonella_enterica	+	6.59E-01	0.000	0.000	0.000	0.000	0.000
3.5.4.26|g__Sanguibacteroides.s__Sanguibacteroides_justesenii	+	7.55E-01	0.000	0.000	0.000	0.000	0.000
3.5.4.26|g__Slackia.s__Slackia_isoflavoniconvertens	+	3.94E-02	0.000	0.000	0.000	0.000	0.000
3.5.4.26|g__Staphylococcus.s__Staphylococcus_aureus	+	2.32E-01	0.000	0.000	0.000	0.000	0.000
3.5.4.26|g__Staphylococcus.s__Staphylococcus_epidermidis	+	4.92E-01	0.000	0.000	0.000	0.000	0.000
3.5.4.26|g__Staphylococcus.s__Staphylococcus_haemolyticus	+	4.92E-01	0.000	0.000	0.000	0.000	0.000
3.5.4.26|g__Stomatobaculum.s__Stomatobaculum_longum	+	4.92E-01	0.000	0.000	0.000	0.000	0.000
3.5.4.26|g__Streptococcus.s__Streptococcus_agalactiae	+	4.92E-01	0.000	0.000	0.000	0.000	0.000
3.5.4.26|g__Streptococcus.s__Streptococcus_equinus	+	3.10E-03	0.000	0.000	0.000	0.000	0.002
3.5.4.26|g__Streptococcus.s__Streptococcus_gallolyticus	+	4.24E-02	0.000	0.000	0.000	0.000	0.000
3.5.4.26|g__Streptococcus.s__Streptococcus_infantarius	+	1.63E-02	0.000	0.000	0.000	0.001	0.001
3.5.4.26|g__Streptococcus.s__Streptococcus_lutetiensis	+	1.96E-02	0.000	0.000	0.000	0.000	0.001
3.5.4.26|g__Streptococcus.s__Streptococcus_macedonicus	+	9.30E-02	0.000	0.000	0.000	0.000	0.001
3.5.4.26|g__Streptococcus.s__Streptococcus_mitis	-	1.48E-01	0.000	0.000	0.000	0.000	0.000
3.5.4.26|g__Streptococcus.s__Streptococcus_oralis	-	9.67E-01	0.000	0.000	0.000	0.000	0.000
3.5.4.26|g__Streptococcus.s__Streptococcus_pasteurianus	+	1.19E-01	0.000	0.000	0.000	0.000	0.001
3.5.4.26|g__Streptococcus.s__Streptococcus_pneumoniae	-	2.81E-01	0.000	0.000	0.000	0.000	0.000
3.5.4.26|g__Streptococcus.s__Streptococcus_pseudopneumoniae	+	4.92E-01	0.000	0.000	0.000	0.000	0.000
3.5.4.26|g__Succinatimonas.s__Succinatimonas_hippei	-	1.48E-01	0.000	0.000	0.000	0.000	0.000
3.5.4.26|g__Sutterella.s__Sutterella_wadsworthensis	+	2.48E-01	0.000	0.000	0.000	0.001	0.002

	3.5.4.26|g__Terrisporobacter.s__Terrisporobacter_othiniensis	+	1.67E-01	0.000	0.000	0.000	0.000	0.000
	3.5.4.26|g__Trichococcus.s__Trichococcus_flocculiformis	+	4.92E-01	0.000	0.000	0.000	0.000	0.000
	3.5.4.26|g__Turicimonas.s__Turicimonas_muris	+	8.31E-01	0.000	0.000	0.000	0.000	0.000
	3.5.4.26|g__Varibaculum.s__Varibaculum_cambriense	+	3.30E-01	0.000	0.000	0.000	0.000	0.000
	3.5.4.26|g__Veillonella.s__Veillonella_atypica	-	9.20E-01	0.000	0.000	0.000	0.000	0.001
	3.5.4.26|g__Veillonella.s__Veillonella_dispar	-	4.20E-01	0.000	0.000	0.000	0.000	0.000
	3.5.4.26|g__Veillonella.s__Veillonella_infantium	+	4.06E-01	0.000	0.000	0.000	0.000	0.000
	3.5.4.26|g__Veillonella.s__Veillonella_parvula	+	4.93E-01	0.000	0.000	0.000	0.000	0.001
	3.5.4.26|g__Veillonella.s__Veillonella_rogosae	-	9.21E-01	0.000	0.000	0.000	0.000	0.000
	3.5.4.26|g__Vibrio.s__Vibrio_parahaemolyticus	+	8.29E-01	0.000	0.000	0.000	0.000	0.000
	3.5.4.26|g__Victivallales_unclassified.s__Victivallales_bacterium_CCUG_4	+	1.54E-03	0.000	0.000	0.000	0.000	0.000
	3.5.4.26|g__Victivallis.s__Victivallis_vadensis	+	1.47E-01	0.000	0.000	0.000	0.000	0.000
	3.5.4.26|g__Weeksella.s__Weeksella_virosa	+	4.92E-01	0.000	0.000	0.000	0.000	0.000
	3.5.4.26|g__Weissella.s__Weissella_cibaria	-	9.73E-01	0.000	0.000	0.000	0.000	0.000
	3.5.4.26|g__Weissella.s__Weissella_confusa	-	6.66E-02	0.000	0.000	0.000	0.000	0.000
	3.5.4.26|g__Yokenella.s__Yokenella_regensburgei	+	4.15E-01	0.000	0.000	0.000	0.000	0.000
	3.5.4.26|g__Hungatella.s__Hungatella_hathewayi	-	8.56E-01	0.070	0.072	0.002	0.005	0.004
	3.5.4.26|g__Bacteroides.s__Bacteroides_thetaiotaomicron	+	4.00E-01	0.450	0.455	0.005	0.014	0.016
	3.5.4.26|g__Bacteroides.s__Bacteroides_dorei	+	2.03E-01	0.030	0.044	0.014	0.015	0.020
	3.5.4.26|g__Odoribacter.s__Odoribacter_splanchnicus	+	3.16E-01	0.199	0.243	0.044	0.003	0.004
	3.5.4.26|g__Bilophila.s__Bilophila_wadsworthia	+	2.31E-02	0.260	0.364	0.104	0.004	0.006
	3.5.4.26|g__Bacteroides.s__Bacteroides_cellulosilyticus	+	2.58E-01	0.045	0.157	0.112	0.011	0.011
	3.5.4.26|g__Bacteroides.s__Bacteroides_intestinalis	+	2.07E-01	0.000	0.115	0.115	0.011	0.015
	3.5.4.26|g__Phascolarctobacterium.s__Phascolarctobacterium_faecium	+	6.72E-01	0.426	0.551	0.125	0.010	0.012
	3.5.4.26|g__Parabacteroides.s__Parabacteroides_merdae	+	1.34E-01	0.662	0.854	0.192	0.011	0.014
	3.5.4.26|g__Bacteroides.s__Bacteroides_caccae	+	1.66E-01	0.085	0.337	0.252	0.008	0.013
	3.5.4.26|g__Parabacteroides.s__Parabacteroides_distasonis	+	6.79E-03	0.989	1.266	0.277	0.019	0.023
	3.5.4.26|g__Bacteroides.s__Bacteroides_stercoris	+	1.34E-01	0.051	0.674	0.623	0.050	0.059
Germany	3.5.4.26	-	1.61E-02	108.889	93.929	-14.960	1.000	1.000
	3.5.4.26|g__Faecalibacterium.s__Faecalibacterium_prausnitzii	-	2.72E-02	11.343	5.667	-5.676	0.110	0.085
	3.5.4.26|g__Bacteroides.s__Bacteroides_dorei	-	8.38E-01	0.885	0.501	-0.384	0.028	0.020
	3.5.4.26|g__Bacteroides.s__Bacteroides_caccae	-	3.91E-01	0.707	0.345	-0.362	0.017	0.014
	3.5.4.26|g__Dorea.s__Dorea_longicatena	-	6.65E-01	0.979	0.752	-0.227	0.013	0.013
	3.5.4.26|g__Parabacteroides.s__Parabacteroides_distasonis	+	6.32E-01	1.394	1.169	-0.226	0.015	0.017
	3.5.4.26|g__Dorea.s__Dorea_formicigenerans	-	1.27E-01	0.324	0.110	-0.214	0.003	0.002
	3.5.4.26|g__Lachnospiraceae_unclassified.s__Eubacterium_rectale	+	6.87E-01	1.186	0.985	-0.200	0.027	0.030
	3.5.4.26|g__Bacteroides.s__Bacteroides_ovatus	-	4.92E-01	0.178	0.026	-0.151	0.006	0.016
	3.5.4.26|g__Odoribacter.s__Odoribacter_splanchnicus	-	4.84E-01	0.602	0.468	-0.134	0.006	0.006
	3.5.4.26|g__Bacteroides.s__Bacteroides_xylanisolvens	-	8.69E-01	0.312	0.182	-0.130	0.008	0.011
	3.5.4.26|g__Blautia.s__Blautia_wexlerae	-	4.03E-01	0.294	0.194	-0.100	0.006	0.004
	3.5.4.26|g__Bacteroides.s__Bacteroides_thetaiotaomicron	-	2.60E-01	0.372	0.277	-0.094	0.014	0.007
	3.5.4.26|g__Coprococcus.s__Coprococcus_comes	-	9.08E-01	0.364	0.311	-0.053	0.006	0.006
	3.5.4.26|g__Prevotella.s__Prevotella_copri	-	7.97E-02	0.052	0.000	-0.052	0.084	0.037
	3.5.4.26|g__Escherichia.s__Escherichia_coli	-	1.54E-01	0.040	0.000	-0.040	0.011	0.003
	3.5.4.26|g__Acidaminococcus.s__Acidaminococcus_fermentans	-	9.42E-01	0.000	0.000	0.000	0.001	0.000
	3.5.4.26|g__Acidaminococcus.s__Acidaminococcus_intestini	-	9.31E-01	0.000	0.000	0.000	0.002	0.002
	3.5.4.26|g__Adlercreutzia.s__Adlercreutzia_equolifaciens	+	5.32E-02	0.000	0.000	0.000	0.000	0.000
	3.5.4.26|g__Allisonella.s__Allisonella_histaminiformans	+	3.59E-01	0.000	0.000	0.000	0.000	0.000
	3.5.4.26|g__Anaeromassilibacillus.s__Anaeromassilibacillus_sp_An250	+	7.65E-01	0.000	0.000	0.000	0.000	0.001
	3.5.4.26|g__Anaerotignum.s__Anaerotignum_lactatifermentans	-	9.81E-01	0.000	0.000	0.000	0.000	0.000
	3.5.4.26|g__Asaccharobacter.s__Asaccharobacter_celatus	+	2.26E-01	0.000	0.000	0.000	0.001	0.002
	3.5.4.26|g__Bacteroides.s__Bacteroides_clarus	-	5.44E-01	0.000	0.000	0.000	0.004	0.002
	3.5.4.26|g__Bacteroides.s__Bacteroides_coprocola	-	3.39E-01	0.000	0.000	0.000	0.016	0.007
	3.5.4.26|g__Bacteroides.s__Bacteroides_coprophilus	-	8.75E-01	0.000	0.000	0.000	0.005	0.000
	3.5.4.26|g__Bacteroides.s__Bacteroides_eggerthii	+	9.25E-01	0.000	0.000	0.000	0.014	0.004
	3.5.4.26|g__Bacteroides.s__Bacteroides_faecis	+	9.29E-01	0.000	0.000	0.000	0.004	0.004
	3.5.4.26|g__Bacteroides.s__Bacteroides_finegoldii	+	2.91E-01	0.000	0.000	0.000	0.002	0.004
	3.5.4.26|g__Bacteroides.s__Bacteroides_fluxus	-	3.09E-01	0.000	0.000	0.000	0.000	0.000
	3.5.4.26|g__Bacteroides.s__Bacteroides_fragilis	-	9.79E-01	0.000	0.000	0.000	0.004	0.003
	3.5.4.26|g__Bacteroides.s__Bacteroides_massiliensis	-	8.09E-01	0.000	0.000	0.000	0.018	0.016
	3.5.4.26|g__Bacteroides.s__Bacteroides_nordii	-	8.06E-01	0.000	0.000	0.000	0.001	0.000
	3.5.4.26|g__Bacteroides.s__Bacteroides_plebeius	+	3.37E-01	0.000	0.000	0.000	0.003	0.008
	3.5.4.26|g__Bacteroides.s__Bacteroides_salyersiae	+	7.62E-01	0.000	0.000	0.000	0.001	0.004
	3.5.4.26|g__Bacteroides.s__Bacteroides_stercorirosoris	+	3.59E-01	0.000	0.000	0.000	0.000	0.000
	3.5.4.26|g__Bacteroides.s__Bacteroides_stercoris	-	7.80E-01	0.000	0.000	0.000	0.018	0.011
	3.5.4.26|g__Blautia.s__Blautia_hansenii	+	3.59E-01	0.000	0.000	0.000	0.000	0.000
	3.5.4.26|g__Blautia.s__Blautia_sp_AF19_10LB	+	1.73E-01	0.000	0.000	0.000	0.001	0.001
	3.5.4.26|g__Butyricicoccus.s__Butyricicoccus_pullicaecorum	+	3.59E-01	0.000	0.000	0.000	0.000	0.000
	3.5.4.26|g__Butyricimonas.s__Butyricimonas_virosa	-	3.00E-01	0.000	0.000	0.000	0.001	0.001
	3.5.4.26|g__Butyrivibrio.s__Butyrivibrio_crossotus	+	8.14E-01	0.000	0.000	0.000	0.012	0.004
	3.5.4.26|g__Candidatus_Gastranaerophilales_unclassified.s__Candidatus_Ga	-	9.81E-01	0.000	0.000	0.000	0.000	0.000
	3.5.4.26|g__Candidatus_Methanomethylophilus.s__Candidatus_Methanome	+	3.59E-01	0.000	0.000	0.000	0.000	0.001

3.5.4.26|g__Catenibacterium.s__Catenibacterium_mitsuokai	-	9.76E-02	0.000	0.000	0.000	0.015	0.006
3.5.4.26|g__Citrobacter.s__Citrobacter_sp_MGH106	+	3.59E-01	0.000	0.000	0.000	0.000	0.000
3.5.4.26|g__Cloacibacillus.s__Cloacibacillus_porcorum	+	1.83E-01	0.000	0.000	0.000	0.000	0.001
3.5.4.26|g__Clostridioides.s__Clostridioides_difficile	-	1.40E-01	0.000	0.000	0.000	0.000	0.000
3.5.4.26|g__Clostridium.s__Clostridium_disporicum	-	1.40E-01	0.000	0.000	0.000	0.000	0.000
3.5.4.26|g__Coprobacillus.s__Coprobacillus_cateniformis	+	2.74E-01	0.000	0.000	0.000	0.000	0.001
3.5.4.26|g__Coprobacter.s__Coprobacter_fastidiosus	-	6.01E-01	0.000	0.000	0.000	0.009	0.003
3.5.4.26|g__Coprobacter.s__Coprobacter_secundus	-	9.42E-01	0.000	0.000	0.000	0.000	0.000
3.5.4.26|g__Coprococcus.s__Coprococcus_catus	-	7.73E-01	0.000	0.000	0.000	0.002	0.002
3.5.4.26|g__Coprococcus.s__Coprococcus_eutactus	-	8.49E-01	0.000	0.000	0.000	0.016	0.013
3.5.4.26|g__Desulfovibrio.s__Desulfovibrio_fairfieldensis	+	3.38E-01	0.000	0.000	0.000	0.000	0.001
3.5.4.26|g__Desulfovibrio.s__Desulfovibrio_piger	-	7.71E-01	0.000	0.000	0.000	0.001	0.010
3.5.4.26|g__Desulfovibrionaceae_unclassified.s__Desulfovibrionaceae_bact	-	1.40E-01	0.000	0.000	0.000	0.000	0.000
3.5.4.26|g__Dialister.s__Dialister_succinatiphilus	-	9.42E-01	0.000	0.000	0.000	0.001	0.001
3.5.4.26|g__Dorea.s__Dorea_sp_OM02_2LB	-	9.81E-01	0.000	0.000	0.000	0.000	0.000
3.5.4.26|g__Eggerthella.s__Eggerthella_lenta	-	6.66E-02	0.000	0.000	0.000	0.000	0.000
3.5.4.26|g__Enterobacter.s__Enterobacter_cloacae	-	3.09E-01	0.000	0.000	0.000	0.000	0.000
3.5.4.26|g__Enterococcus.s__Enterococcus_faecium	-	3.09E-01	0.000	0.000	0.000	0.000	0.000
3.5.4.26|g__Enterococcus.s__Enterococcus_hirae	-	3.09E-01	0.000	0.000	0.000	0.001	0.000
3.5.4.26|g__Erysipelatoclostridium.s__Clostridium_innocuum	-	1.40E-01	0.000	0.000	0.000	0.000	0.000
3.5.4.26|g__Erysipelatoclostridium.s__Clostridium_spiroforme	+	7.41E-01	0.000	0.000	0.000	0.001	0.000
3.5.4.26|g__Erysipelotrichaceae_unclassified.s__Erysipelotrichaceae_bacteri	-	3.09E-01	0.000	0.000	0.000	0.000	0.000
3.5.4.26|g__Escherichia.s__Escherichia_fergusonii	+	3.59E-01	0.000	0.000	0.000	0.000	0.000
3.5.4.26|g__Escherichia.s__Escherichia_marmotae	-	3.09E-01	0.000	0.000	0.000	0.000	0.000
3.5.4.26|g__Eubacterium.s__Eubacterium_ramulus	-	5.66E-01	0.000	0.000	0.000	0.001	0.002
3.5.4.26|g__Eubacterium.s__Eubacterium_sp_AF17_7	+	1.98E-01	0.000	0.000	0.000	0.000	0.001
3.5.4.26|g__Eubacterium.s__Eubacterium_sp_AM18_10LB_B	-	3.09E-01	0.000	0.000	0.000	0.000	0.000
3.5.4.26|g__Firmicutes_unclassified.s__Firmicutes_bacterium_AM10_47	-	5.50E-01	0.000	0.000	0.000	0.000	0.000
3.5.4.26|g__Flavonifractor.s__Flavonifractor_plautii	+	5.85E-02	0.000	0.000	0.000	0.000	0.002
3.5.4.26|g__Fusobacterium.s__Fusobacterium_mortiferum	-	3.09E-01	0.000	0.000	0.000	0.000	0.000
3.5.4.26|g__Haemophilus.s__Haemophilus_parainfluenzae	-	7.63E-02	0.000	0.000	0.000	0.001	0.000
3.5.4.26|g__Hafnia.s__Hafnia_alvei	+	1.83E-01	0.000	0.000	0.000	0.000	0.001
3.5.4.26|g__Hafnia.s__Hafnia_paralvei	+	3.59E-01	0.000	0.000	0.000	0.000	0.001
3.5.4.26|g__Holdemanella.s__Holdemanella_biformis	-	2.76E-02	0.000	0.000	0.000	0.001	0.000
3.5.4.26|g__Hungatella.s__Hungatella_hathewayi	-	2.11E-01	0.000	0.000	0.000	0.001	0.000
3.5.4.26|g__Intestinibacter.s__Intestinibacter_bartlettii	-	9.42E-01	0.000	0.000	0.000	0.000	0.000
3.5.4.26|g__Klebsiella.s__Klebsiella_oxytoca	-	5.77E-01	0.000	0.000	0.000	0.003	0.001
3.5.4.26|g__Klebsiella.s__Klebsiella_pneumoniae	-	8.00E-02	0.000	0.000	0.000	0.003	0.002
3.5.4.26|g__Klebsiella.s__Klebsiella_variicola	-	5.37E-01	0.000	0.000	0.000	0.000	0.000
3.5.4.26|g__Lachnoclostridium.s__Clostridium_aldenense	-	3.09E-01	0.000	0.000	0.000	0.000	0.000
3.5.4.26|g__Lachnoclostridium.s__Clostridium_bolteae	-	5.94E-01	0.000	0.000	0.000	0.000	0.001
3.5.4.26|g__Lachnoclostridium.s__Clostridium_citroniae	-	3.09E-01	0.000	0.000	0.000	0.000	0.000
3.5.4.26|g__Lachnoclostridium.s__Clostridium_clostridioforme	-	3.22E-02	0.000	0.000	0.000	0.000	0.000
3.5.4.26|g__Lachnoclostridium.s__Clostridium_symbiosum	-	1.40E-01	0.000	0.000	0.000	0.000	0.000
3.5.4.26|g__Lachnospira.s__Lachnospira_pectinoschiza	+	5.52E-01	0.000	0.000	0.000	0.001	0.002
3.5.4.26|g__Lactobacillus.s__Lactobacillus_antri	-	3.09E-01	0.000	0.000	0.000	0.000	0.000
3.5.4.26|g__Lactobacillus.s__Lactobacillus_crispatus	-	3.09E-01	0.000	0.000	0.000	0.000	0.000
3.5.4.26|g__Lactobacillus.s__Lactobacillus_delbrueckii	-	3.09E-01	0.000	0.000	0.000	0.000	0.000
3.5.4.26|g__Lactobacillus.s__Lactobacillus_fermentum	-	1.40E-01	0.000	0.000	0.000	0.000	0.000
3.5.4.26|g__Lactobacillus.s__Lactobacillus_gastricus	-	3.09E-01	0.000	0.000	0.000	0.000	0.000
3.5.4.26|g__Lactobacillus.s__Lactobacillus_oris	-	3.09E-01	0.000	0.000	0.000	0.000	0.000
3.5.4.26|g__Lactobacillus.s__Lactobacillus_rogosae	+	5.04E-01	0.000	0.000	0.000	0.001	0.003
3.5.4.26|g__Lactococcus.s__Lactococcus_lactis	-	1.40E-01	0.000	0.000	0.000	0.001	0.000
3.5.4.26|g__Listeria.s__Listeria_monocytogenes	+	1.83E-01	0.000	0.000	0.000	0.000	0.000
3.5.4.26|g__Megamonas.s__Megamonas_funiformis	+	3.59E-01	0.000	0.000	0.000	0.000	0.000
3.5.4.26|g__Megamonas.s__Megamonas_hypermegale	+	3.59E-01	0.000	0.000	0.000	0.000	0.001
3.5.4.26|g__Megamonas.s__Megamonas_rupellensis	+	3.59E-01	0.000	0.000	0.000	0.000	0.001
3.5.4.26|g__Megasphaera.s__Megasphaera_elsdenii	-	4.85E-01	0.000	0.000	0.000	0.001	0.000
3.5.4.26|g__Megasphaera.s__Megasphaera_hexanoica	-	3.09E-01	0.000	0.000	0.000	0.000	0.000
3.5.4.26|g__Mesosutterella.s__Mesosutterella_multiformis	+	1.98E-01	0.000	0.000	0.000	0.000	0.001
3.5.4.26|g__Mitsuokella.s__Mitsuokella_jalaludinii	-	5.11E-01	0.000	0.000	0.000	0.001	0.000
3.5.4.26|g__Mitsuokella.s__Mitsuokella_multacida	-	3.09E-01	0.000	0.000	0.000	0.001	0.000
3.5.4.26|g__Obesumbacterium.s__Obesumbacterium_proteus	+	1.83E-01	0.000	0.000	0.000	0.000	0.000
3.5.4.26|g__Oxalobacter.s__Oxalobacter_formigenes	-	9.31E-01	0.000	0.000	0.000	0.000	0.000
3.5.4.26|g__Parabacteroides.s__Parabacteroides_goldsteinii	-	3.32E-01	0.000	0.000	0.000	0.001	0.000
3.5.4.26|g__Parabacteroides.s__Parabacteroides_gordonii	+	9.80E-02	0.000	0.000	0.000	0.000	0.000
3.5.4.26|g__Parabacteroides.s__Parabacteroides_johnsonii	-	2.91E-01	0.000	0.000	0.000	0.002	0.000
3.5.4.26|g__Paraprevotella.s__Paraprevotella_clara	-	7.45E-02	0.000	0.000	0.000	0.005	0.002
3.5.4.26|g__Parasutterella.s__Parasutterella_excrementihominis	+	8.74E-01	0.000	0.000	0.000	0.001	0.002
3.5.4.26|g__Phascolarctobacterium.s__Phascolarctobacterium_faecium	+	2.83E-01	0.000	0.000	0.000	0.004	0.011
3.5.4.26|g__Phascolarctobacterium.s__Phascolarctobacterium_succinatutens	-	2.92E-02	0.000	0.000	0.000	0.009	0.002
3.5.4.26|g__Prevotella.s__Prevotella_disiens	-	3.09E-01	0.000	0.000	0.000	0.000	0.000

	3.5.4.26|g__Prevotella.s__Prevotella_sp_109	+	1.83E-01	0.000	0.000	0.000	0.000	0.012
	3.5.4.26|g__Prevotella.s__Prevotella_sp_AM42_24	-	5.11E-01	0.000	0.000	0.000	0.013	0.008
	3.5.4.26|g__Pseudoflavonifractor.s__Pseudoflavonifractor_sp_An184	-	9.42E-01	0.000	0.000	0.000	0.001	0.000
	3.5.4.26|g__Pseudomonas.s__Pseudomonas_aeruginosa	-	3.09E-01	0.000	0.000	0.000	0.000	0.000
	3.5.4.26|g__Pseudomonas.s__Pseudomonas_aeruginosa_group	-	3.09E-01	0.000	0.000	0.000	0.000	0.000
	3.5.4.26|g__Pyramidobacter.s__Pyramidobacter_piscolens	-	3.09E-01	0.000	0.000	0.000	0.000	0.000
	3.5.4.26|g__Roseburia.s__Roseburia_faecis	-	8.78E-01	0.000	0.000	0.000	0.011	0.007
	3.5.4.26|g__Roseburia.s__Roseburia_intestinalis	-	4.59E-01	0.000	0.000	0.000	0.007	0.003
	3.5.4.26|g__Roseburia.s__Roseburia_inulinivorans	+	2.24E-01	0.000	0.000	0.000	0.002	0.003
	3.5.4.26|g__Ruminococcaceae_unclassified.s__Ruminococcaceae_bacteriun	-	2.73E-01	0.000	0.000	0.000	0.001	0.000
	3.5.4.26|g__Ruminococcaceae_unclassified.s__Ruminococcaceae_bacteriun	+	5.32E-02	0.000	0.000	0.000	0.000	0.001
	3.5.4.26|g__Ruminococcus.s__Ruminococcus_callidus	-	5.60E-01	0.000	0.000	0.000	0.002	0.002
	3.5.4.26|g__Ruminococcus.s__Ruminococcus_sp_AF31_8BH	+	2.54E-01	0.000	0.000	0.000	0.001	0.002
	3.5.4.26|g__Salmonella.s__Salmonella_enterica	+	3.59E-01	0.000	0.000	0.000	0.000	0.000
	3.5.4.26|g__Sanguibacteroides.s__Sanguibacteroides_justesenii	-	3.09E-01	0.000	0.000	0.000	0.000	0.000
	3.5.4.26|g__Slackia.s__Slackia_isoflavoniconvertens	-	2.02E-01	0.000	0.000	0.000	0.003	0.004
	3.5.4.26|g__Streptococcus.s__Streptococcus_equinus	-	9.81E-01	0.000	0.000	0.000	0.000	0.000
	3.5.4.26|g__Streptococcus.s__Streptococcus_infantarius	+	3.59E-01	0.000	0.000	0.000	0.000	0.000
	3.5.4.26|g__Streptococcus.s__Streptococcus_lutetiensis	+	3.59E-01	0.000	0.000	0.000	0.000	0.000
	3.5.4.26|g__Streptococcus.s__Streptococcus_macedonicus	-	3.09E-01	0.000	0.000	0.000	0.000	0.000
	3.5.4.26|g__Streptococcus.s__Streptococcus_pasteurianus	-	3.09E-01	0.000	0.000	0.000	0.000	0.000
	3.5.4.26|g__Sutterella.s__Sutterella_wadsworthensis	-	5.72E-01	0.000	0.000	0.000	0.001	0.001
	3.5.4.26|g__Turicimonas.s__Turicimonas_muris	-	1.40E-01	0.000	0.000	0.000	0.001	0.000
	3.5.4.26|g__Veillonella.s__Veillonella_atypica	-	3.09E-01	0.000	0.000	0.000	0.000	0.000
	3.5.4.26|g__Veillonella.s__Veillonella_dispar	-	5.11E-01	0.000	0.000	0.000	0.000	0.000
	3.5.4.26|g__Veillonella.s__Veillonella_infantium	-	3.09E-01	0.000	0.000	0.000	0.000	0.000
	3.5.4.26|g__Veillonella.s__Veillonella_parvula	+	1.83E-01	0.000	0.000	0.000	0.000	0.000
	3.5.4.26|g__Veillonella.s__Veillonella_rogosae	-	3.09E-01	0.000	0.000	0.000	0.000	0.000
	3.5.4.26|g__Victivallales_unclassified.s__Victivallales_bacterium_CCUG_4	+	4.28E-01	0.000	0.000	0.000	0.001	0.002
	3.5.4.26|g__Victivallis.s__Victivallis_vadensis	+	1.87E-01	0.000	0.000	0.000	0.000	0.001
	3.5.4.26|g__Blautia.s__Blautia_obeum	-	7.16E-01	0.741	0.789	0.048	0.010	0.012
	3.5.4.26|g__Agathobaculum.s__Agathobaculum_butyriciproducens	+	6.23E-01	0.196	0.253	0.057	0.003	0.006
	3.5.4.26|g__Eubacterium.s__Eubacterium_eligens	+	8.61E-01	0.782	0.978	0.196	0.021	0.017
	3.5.4.26|g__Clostridium.s__Clostridium_sp_AM22_11AC	+	2.15E-01	0.000	0.201	0.201	0.002	0.003
	3.5.4.26|g__Roseburia.s__Roseburia_hominis	+	1.55E-01	0.557	0.771	0.215	0.007	0.013
	3.5.4.26|g__Bilophila.s__Bilophila_wadsworthia	+	2.88E-01	0.000	0.217	0.217	0.003	0.004
	3.5.4.26|g__Anaerostipes.s__Anaerostipes_hadrus	+	1.12E-01	0.055	0.276	0.221	0.004	0.008
	3.5.4.26|g__Bacteroides.s__Bacteroides_cellulosilyticus	+	5.53E-01	0.255	0.507	0.252	0.010	0.010
	3.5.4.26|g__Blautia.s__Ruminococcus_torques	+	5.90E-01	1.226	1.485	0.259	0.016	0.026
	3.5.4.26|g__Clostridium.s__Clostridium_sp_AF36_4	+	1.30E-02	0.000	0.280	0.280	0.002	0.007
	3.5.4.26|g__Bacteroides.s__Bacteroides_intestinalis	+	2.76E-01	0.151	0.442	0.291	0.007	0.008
	3.5.4.26|g__Bacteroides.s__Bacteroides_uniformis	+	4.34E-01	1.901	2.226	0.325	0.040	0.061
	3.5.4.26|g__Barnesiella.s__Barnesiella_intestinihominis	+	4.06E-01	0.695	1.046	0.351	0.013	0.018
	3.5.4.26|g__Parabacteroides.s__Parabacteroides_merdae	+	3.37E-01	0.297	0.938	0.640	0.008	0.012
	3.5.4.26|g__Bacteroides.s__Bacteroides_vulgatus	-	8.67E-01	2.719	3.878	1.159	0.079	0.073
	3.5.4.26|unclassified	+	6.76E-01	12.024	14.671	2.647	0.159	0.192
	3.5.4.26|g__Akkermansia.s__Akkermansia_muciniphila	+	6.97E-04	0.000	3.030	3.030	0.020	0.058
China1	3.5.4.26	-	1.64E-02	109.557	99.634	-9.923	1.000	1.000
	3.5.4.26|g__Bacteroides.s__Bacteroides_vulgatus	-	1.36E-02	10.166	3.046	-7.119	0.155	0.077
	3.5.4.26|g__Bacteroides.s__Bacteroides_ovatus	-	3.08E-02	2.870	1.503	-1.367	0.061	0.028
	3.5.4.26|g__Bacteroides.s__Bacteroides_fragilis	-	2.36E-01	1.776	1.001	-0.775	0.028	0.032
	3.5.4.26|g__Bacteroides.s__Bacteroides_xylanisolvens	-	4.70E-01	0.784	0.592	-0.192	0.024	0.015
	3.5.4.26|g__Faecalibacterium.s__Faecalibacterium_prausnitzii	+	9.35E-01	1.002	0.889	-0.113	0.016	0.019
	3.5.4.26|g__Parabacteroides.s__Parabacteroides_distasonis	+	6.62E-01	1.189	1.097	-0.092	0.024	0.020
	3.5.4.26|g__Escherichia.s__Escherichia_coli	-	2.00E-01	0.045	0.001	-0.044	0.003	0.002
	3.5.4.26|g__Bacteroides.s__Bacteroides_cellulosilyticus	+	9.08E-01	0.405	0.372	-0.033	0.013	0.022
	3.5.4.26|g__Prevotella.s__Prevotella_copri	+	8.75E-01	0.067	0.038	-0.029	0.118	0.114
	3.5.4.26|g__Blautia.s__Blautia_wexlerae	-	4.85E-01	0.016	0.000	-0.016	0.001	0.001
	3.5.4.26|g__Acidaminococcus.s__Acidaminococcus_intestini	+	3.30E-01	0.000	0.000	0.000	0.000	0.000
	3.5.4.26|g__Adlercreutzia.s__Adlercreutzia_equolifaciens	-	6.71E-01	0.000	0.000	0.000	0.000	0.000
	3.5.4.26|g__Aeromonas.s__Aeromonas_dhakensis	-	3.30E-01	0.000	0.000	0.000	0.000	0.000
	3.5.4.26|g__Aeromonas.s__Aeromonas_enteropelogenes	-	3.30E-01	0.000	0.000	0.000	0.000	0.000
	3.5.4.26|g__Aeromonas.s__Aeromonas_hydrophila	-	3.30E-01	0.000	0.000	0.000	0.000	0.000
	3.5.4.26|g__Aeromonas.s__Aeromonas_veronii	-	3.30E-01	0.000	0.000	0.000	0.000	0.000
	3.5.4.26|g__Agathobaculum.s__Agathobaculum_butyriciproducens	-	9.90E-01	0.000	0.000	0.000	0.001	0.000
	3.5.4.26|g__Aggregatibacter.s__Aggregatibacter_segnis	-	1.00E+00	0.000	0.000	0.000	0.000	0.000
	3.5.4.26|g__Akkermansia.s__Akkermansia_muciniphila	+	6.21E-03	0.000	0.000	0.000	0.001	0.009
	3.5.4.26|g__Allisonella.s__Allisonella_histaminiformans	+	9.90E-01	0.000	0.000	0.000	0.000	0.000
	3.5.4.26|g__Anaeroglobus.s__Anaeroglobus_geminatus	+	3.30E-01	0.000	0.000	0.000	0.000	0.000
	3.5.4.26|g__Anaeromassilibacillus.s__Anaeromassilibacillus_sp_An250	+	3.30E-01	0.000	0.000	0.000	0.000	0.000
	3.5.4.26|g__Anaerostipes.s__Anaerostipes_caccae	-	3.30E-01	0.000	0.000	0.000	0.000	0.000
	3.5.4.26|g__Anaerostipes.s__Anaerostipes_hadrus	-	7.14E-01	0.000	0.000	0.000	0.001	0.000

3.5.4.26|g__Asaccharobacter.s__Asaccharobacter_celatus	-	6.55E-01	0.000	0.000	0.000	0.000	0.000
3.5.4.26|g__Bacteroides.s__Bacteroides_clarus	+	1.40E-01	0.000	0.000	0.000	0.001	0.005
3.5.4.26|g__Bacteroides.s__Bacteroides_coprocola	-	1.50E-01	0.000	0.000	0.000	0.049	0.012
3.5.4.26|g__Bacteroides.s__Bacteroides_coprophilus	-	3.93E-01	0.000	0.000	0.000	0.005	0.001
3.5.4.26|g__Bacteroides.s__Bacteroides_eggerthii	+	1.05E-01	0.000	0.000	0.000	0.018	0.037
3.5.4.26|g__Bacteroides.s__Bacteroides_faecis	-	1.58E-01	0.000	0.000	0.000	0.001	0.000
3.5.4.26|g__Bacteroides.s__Bacteroides_finegoldii	+	4.16E-01	0.000	0.000	0.000	0.009	0.014
3.5.4.26|g__Bacteroides.s__Bacteroides_fluxus	+	3.30E-01	0.000	0.000	0.000	0.000	0.000
3.5.4.26|g__Bacteroides.s__Bacteroides_massiliensis	+	6.35E-01	0.000	0.000	0.000	0.010	0.015
3.5.4.26|g__Bacteroides.s__Bacteroides_oleiciplenus	+	5.89E-01	0.000	0.000	0.000	0.000	0.000
3.5.4.26|g__Bacteroides.s__Bacteroides_plebeius	-	2.80E-01	0.000	0.000	0.000	0.058	0.020
3.5.4.26|g__Bacteroides.s__Bacteroides_salyersiae	-	4.07E-01	0.000	0.000	0.000	0.002	0.002
3.5.4.26|g__Bacteroides.s__Bacteroides_sp_OM08_11	+	8.20E-02	0.000	0.000	0.000	0.000	0.000
3.5.4.26|g__Bacteroides.s__Bacteroides_stercorirosoris	+	5.69E-01	0.000	0.000	0.000	0.000	0.000
3.5.4.26|g__Barnesiella.s__Barnesiella_intestinihominis	+	3.80E-01	0.000	0.000	0.000	0.007	0.007
3.5.4.26|g__Barnesiella.s__Barnesiella_sp_An22	-	3.30E-01	0.000	0.000	0.000	0.000	0.000
3.5.4.26|g__Blautia.s__Blautia_hansenii	+	3.30E-01	0.000	0.000	0.000	0.000	0.000
3.5.4.26|g__Blautia.s__Blautia_sp_AF19_10LB	+	9.63E-01	0.000	0.000	0.000	0.000	0.001
3.5.4.26|g__Butyricimonas.s__Butyricimonas_virosa	+	9.14E-01	0.000	0.000	0.000	0.002	0.003
3.5.4.26|g__Butyrivibrio.s__Butyrivibrio_crossotus	+	3.20E-01	0.000	0.000	0.000	0.000	0.001
3.5.4.26|g__Campylobacter.s__Campylobacter_gracilis	+	3.30E-01	0.000	0.000	0.000	0.000	0.000
3.5.4.26|g__Catenibacterium.s__Catenibacterium_mitsuokai	+	6.06E-01	0.000	0.000	0.000	0.001	0.007
3.5.4.26|g__Citrobacter.s__Citrobacter_braakii	+	1.60E-01	0.000	0.000	0.000	0.000	0.000
3.5.4.26|g__Citrobacter.s__Citrobacter_freundii	+	1.60E-01	0.000	0.000	0.000	0.000	0.000
3.5.4.26|g__Citrobacter.s__Citrobacter_portucalensis	+	3.20E-01	0.000	0.000	0.000	0.000	0.000
3.5.4.26|g__Citrobacter.s__Citrobacter_youngae	+	4.28E-02	0.000	0.000	0.000	0.000	0.000
3.5.4.26|g__Cloacibacillus.s__Cloacibacillus_porcorum	+	1.60E-01	0.000	0.000	0.000	0.000	0.000
3.5.4.26|g__Clostridioides.s__Clostridioides_difficile	-	7.06E-01	0.000	0.000	0.000	0.001	0.000
3.5.4.26|g__Clostridium.s__Clostridium_neonatale	-	1.60E-01	0.000	0.000	0.000	0.000	0.000
3.5.4.26|g__Clostridium.s__Clostridium_perfringens	+	3.30E-01	0.000	0.000	0.000	0.000	0.000
3.5.4.26|g__Clostridium.s__Clostridium_sp_AF36_4	+	8.96E-02	0.000	0.000	0.000	0.001	0.005
3.5.4.26|g__Clostridium.s__Clostridium_sp_AM22_11AC	-	2.35E-01	0.000	0.000	0.000	0.001	0.000
3.5.4.26|g__Clostridium.s__Clostridium_sp_chh4_2	+	3.30E-01	0.000	0.000	0.000	0.000	0.000
3.5.4.26|g__Comamonas.s__Comamonas_kerstersii	+	3.30E-01	0.000	0.000	0.000	0.000	0.000
3.5.4.26|g__Coprobacillus.s__Coprobacillus_cateniformis	+	9.05E-02	0.000	0.000	0.000	0.000	0.001
3.5.4.26|g__Coprobacter.s__Coprobacter_fastidiosus	-	4.77E-01	0.000	0.000	0.000	0.002	0.001
3.5.4.26|g__Coprobacter.s__Coprobacter_secundus	-	7.99E-01	0.000	0.000	0.000	0.000	0.000
3.5.4.26|g__Coprococcus.s__Coprococcus_catus	+	2.58E-01	0.000	0.000	0.000	0.000	0.000
3.5.4.26|g__Coprococcus.s__Coprococcus_comes	+	6.30E-02	0.000	0.000	0.000	0.001	0.002
3.5.4.26|g__Coprococcus.s__Coprococcus_eutactus	+	1.81E-01	0.000	0.000	0.000	0.001	0.003
3.5.4.26|g__Desulfovibrio.s__Desulfovibrio_piger	+	1.65E-01	0.000	0.000	0.000	0.000	0.000
3.5.4.26|g__Desulfovibrio.s__Desulfovibrio_sp_AM18_2	+	8.20E-02	0.000	0.000	0.000	0.000	0.002
3.5.4.26|g__Desulfovibrionaceae_unclassified.s__Desulfovibrionaceae_bact	+	3.30E-01	0.000	0.000	0.000	0.000	0.000
3.5.4.26|g__Dialister.s__Dialister_succinatiphilus	-	5.89E-01	0.000	0.000	0.000	0.000	0.000
3.5.4.26|g__Dorea.s__Dorea_formicigenerans	-	3.78E-01	0.000	0.000	0.000	0.001	0.000
3.5.4.26|g__Dorea.s__Dorea_sp_OM02_2LB	+	3.30E-01	0.000	0.000	0.000	0.000	0.000
3.5.4.26|g__Eggerthella.s__Eggerthella_lenta	+	4.49E-01	0.000	0.000	0.000	0.000	0.000
3.5.4.26|g__Enterobacter.s__Enterobacter_cloacae	+	9.90E-01	0.000	0.000	0.000	0.000	0.000
3.5.4.26|g__Enterococcus.s__Enterococcus_faecium	+	3.30E-01	0.000	0.000	0.000	0.000	0.001
3.5.4.26|g__Enterococcus.s__Enterococcus_hirae	+	3.30E-01	0.000	0.000	0.000	0.000	0.000
3.5.4.26|g__Erysipelatoclostridium.s__Clostridium_innocuum	-	6.82E-01	0.000	0.000	0.000	0.001	0.000
3.5.4.26|g__Erysipelatoclostridium.s__Clostridium_spiroforme	+	3.30E-01	0.000	0.000	0.000	0.000	0.000
3.5.4.26|g__Erysipelotrichaceae_unclassified.s__Erysipelotrichaceae_bacteri	-	3.30E-01	0.000	0.000	0.000	0.000	0.000
3.5.4.26|g__Escherichia.s__Escherichia_fergusonii	+	1.60E-01	0.000	0.000	0.000	0.000	0.000
3.5.4.26|g__Eubacterium.s__Eubacterium_ramulus	+	6.71E-01	0.000	0.000	0.000	0.000	0.000
3.5.4.26|g__Eubacterium.s__Eubacterium_sp_AF17_7	+	1.79E-01	0.000	0.000	0.000	0.000	0.001
3.5.4.26|g__Eubacterium.s__Eubacterium_sp_AM18_10LB_B	+	1.00E+00	0.000	0.000	0.000	0.000	0.001
3.5.4.26|g__Faecalicatena.s__Faecalicatena_contorta	+	3.30E-01	0.000	0.000	0.000	0.000	0.000
3.5.4.26|g__Firmicutes_unclassified.s__Firmicutes_bacterium_AM10_47	+	5.69E-01	0.000	0.000	0.000	0.000	0.000
3.5.4.26|g__Flavonifractor.s__Flavonifractor_plautii	+	8.20E-02	0.000	0.000	0.000	0.000	0.000
3.5.4.26|g__Fusobacterium.s__Fusobacterium_hwasookii	-	3.30E-01	0.000	0.000	0.000	0.000	0.000
3.5.4.26|g__Fusobacterium.s__Fusobacterium_mortiferum	+	3.33E-01	0.000	0.000	0.000	0.001	0.000
3.5.4.26|g__Fusobacterium.s__Fusobacterium_ulcerans	-	4.17E-01	0.000	0.000	0.000	0.001	0.001
3.5.4.26|g__Fusobacterium.s__Fusobacterium_varium	-	1.60E-01	0.000	0.000	0.000	0.001	0.000
3.5.4.26|g__Haemophilus.s__Haemophilus_parainfluenzae	-	7.26E-01	0.000	0.000	0.000	0.002	0.002
3.5.4.26|g__Haemophilus.s__Haemophilus_sputorum	+	1.00E+00	0.000	0.000	0.000	0.000	0.000
3.5.4.26|g__Hafnia.s__Hafnia_paralvei	-	3.30E-01	0.000	0.000	0.000	0.000	0.000
3.5.4.26|g__Holdemanella.s__Holdemanella_biformis	+	1.64E-03	0.000	0.000	0.000	0.000	0.000
3.5.4.26|g__Hungatella.s__Hungatella_hathewayi	-	9.91E-01	0.000	0.000	0.000	0.001	0.001
3.5.4.26|g__Intestinibacter.s__Intestinibacter_bartlettii	+	1.52E-01	0.000	0.000	0.000	0.000	0.000
3.5.4.26|g__Klebsiella.s__Klebsiella_aerogenes	+	3.30E-01	0.000	0.000	0.000	0.000	0.000
3.5.4.26|g__Klebsiella.s__Klebsiella_oxytoca	+	6.32E-01	0.000	0.000	0.000	0.003	0.004

3.5.4.26|g__Klebsiella.s__Klebsiella_pneumoniae	+	5.78E-01	0.000	0.000	0.000	0.002	0.003
3.5.4.26|g__Klebsiella.s__Klebsiella_variicola	+	4.28E-02	0.000	0.000	0.000	0.000	0.001
3.5.4.26|g__Kosakonia.s__Kosakonia_cowanii	-	3.30E-01	0.000	0.000	0.000	0.000	0.000
3.5.4.26|g__Lachnoclostridium.s__Clostridium_aldenense	-	7.14E-01	0.000	0.000	0.000	0.000	0.000
3.5.4.26|g__Lachnoclostridium.s__Clostridium_bolteae	+	8.93E-01	0.000	0.000	0.000	0.001	0.001
3.5.4.26|g__Lachnoclostridium.s__Clostridium_citroniae	+	8.20E-02	0.000	0.000	0.000	0.000	0.000
3.5.4.26|g__Lachnoclostridium.s__Clostridium_clostridioforme	+	4.40E-01	0.000	0.000	0.000	0.000	0.000
3.5.4.26|g__Lachnoclostridium.s__Clostridium_symbiosum	+	7.70E-01	0.000	0.000	0.000	0.000	0.000
3.5.4.26|g__Lachnospira.s__Lachnospira_pectinoschiza	-	4.31E-01	0.000	0.000	0.000	0.002	0.002
3.5.4.26|g__Lactobacillus.s__Lactobacillus_crispatus	-	1.60E-01	0.000	0.000	0.000	0.000	0.000
3.5.4.26|g__Lactobacillus.s__Lactobacillus_fermentum	+	3.30E-01	0.000	0.000	0.000	0.000	0.000
3.5.4.26|g__Lactobacillus.s__Lactobacillus_rogosae	-	4.24E-01	0.000	0.000	0.000	0.002	0.002
3.5.4.26|g__Listeria.s__Listeria_monocytogenes	+	3.30E-01	0.000	0.000	0.000	0.000	0.000
3.5.4.26|g__Megamonas.s__Megamonas_funiformis	+	1.00E+00	0.000	0.000	0.000	0.002	0.001
3.5.4.26|g__Megamonas.s__Megamonas_hypermegale	+	8.28E-01	0.000	0.000	0.000	0.001	0.001
3.5.4.26|g__Megamonas.s__Megamonas_rupellensis	+	7.86E-01	0.000	0.000	0.000	0.002	0.001
3.5.4.26|g__Megasphaera.s__Megasphaera_micronuciformis	-	1.00E+00	0.000	0.000	0.000	0.000	0.000
3.5.4.26|g__Mesosutterella.s__Mesosutterella_multiformis	-	7.37E-01	0.000	0.000	0.000	0.003	0.004
3.5.4.26|g__Oxalobacter.s__Oxalobacter_formigenes	+	6.73E-01	0.000	0.000	0.000	0.000	0.001
3.5.4.26|g__Parabacteroides.s__Parabacteroides_goldsteinii	+	1.22E-01	0.000	0.000	0.000	0.001	0.005
3.5.4.26|g__Parabacteroides.s__Parabacteroides_gordonii	+	9.83E-01	0.000	0.000	0.000	0.000	0.001
3.5.4.26|g__Parabacteroides.s__Parabacteroides_johnsonii	+	6.24E-02	0.000	0.000	0.000	0.001	0.002
3.5.4.26|g__Paraprevotella.s__Paraprevotella_clara	+	1.06E-02	0.000	0.000	0.000	0.001	0.005
3.5.4.26|g__Paraprevotella.s__Paraprevotella_xylaniphila	+	9.70E-01	0.000	0.000	0.000	0.000	0.001
3.5.4.26|g__Parasutterella.s__Parasutterella_excrementihominis	-	5.95E-01	0.000	0.000	0.000	0.002	0.002
3.5.4.26|g__Phascolarctobacterium.s__Phascolarctobacterium_succinatutens	+	2.97E-01	0.000	0.000	0.000	0.001	0.002
3.5.4.26|g__Prevotella.s__Prevotella_denticola	+	3.30E-01	0.000	0.000	0.000	0.000	0.000
3.5.4.26|g__Prevotella.s__Prevotella_disiens	+	3.30E-01	0.000	0.000	0.000	0.000	0.000
3.5.4.26|g__Prevotella.s__Prevotella_sp_109	-	1.60E-01	0.000	0.000	0.000	0.001	0.000
3.5.4.26|g__Prevotella.s__Prevotella_sp_AM42_24	-	1.60E-01	0.000	0.000	0.000	0.003	0.000
3.5.4.26|g__Propionibacterium.s__Propionibacterium_acidifaciens	+	3.30E-01	0.000	0.000	0.000	0.000	0.000
3.5.4.26|g__Proteus.s__Proteus_mirabilis	+	3.30E-01	0.000	0.000	0.000	0.000	0.000
3.5.4.26|g__Pseudocitrobacter.s__Pseudocitrobacter_faecalis	+	3.30E-01	0.000	0.000	0.000	0.000	0.000
3.5.4.26|g__Pyramidobacter.s__Pyramidobacter_piscolens	+	2.92E-02	0.000	0.000	0.000	0.000	0.000
3.5.4.26|g__Pyramidobacter.s__Pyramidobacter_sp_C12_8	+	1.60E-01	0.000	0.000	0.000	0.000	0.000
3.5.4.26|g__Roseburia.s__Roseburia_faecis	+	7.83E-01	0.000	0.000	0.000	0.001	0.002
3.5.4.26|g__Roseburia.s__Roseburia_intestinalis	+	4.28E-02	0.000	0.000	0.000	0.000	0.000
3.5.4.26|g__Roseburia.s__Roseburia_inulinivorans	+	7.14E-01	0.000	0.000	0.000	0.001	0.001
3.5.4.26|g__Ruminococcaceae_unclassified.s__Ruminococcaceae_bacteriun	-	3.30E-01	0.000	0.000	0.000	0.000	0.000
3.5.4.26|g__Ruminococcaceae_unclassified.s__Ruminococcaceae_bacteriun	+	8.79E-02	0.000	0.000	0.000	0.000	0.001
3.5.4.26|g__Ruminococcus.s__Ruminococcus_callidus	+	3.26E-01	0.000	0.000	0.000	0.000	0.001
3.5.4.26|g__Ruminococcus.s__Ruminococcus_sp_AF31_8BH	-	8.26E-01	0.000	0.000	0.000	0.000	0.000
3.5.4.26|g__Salmonella.s__Salmonella_enterica	+	5.89E-01	0.000	0.000	0.000	0.000	0.000
3.5.4.26|g__Sanguibacteroides.s__Sanguibacteroides_justesenii	+	9.70E-01	0.000	0.000	0.000	0.000	0.000
3.5.4.26|g__Slackia.s__Slackia_isoflavoniconvertens	+	1.60E-01	0.000	0.000	0.000	0.000	0.001
3.5.4.26|g__Succinatimonas.s__Succinatimonas_hippei	-	3.30E-01	0.000	0.000	0.000	0.000	0.000
3.5.4.26|g__Sutterella.s__Sutterella_wadsworthensis	+	2.59E-01	0.000	0.000	0.000	0.001	0.001
3.5.4.26|g__Synergistes.s__Synergistes_jonesii	+	3.30E-01	0.000	0.000	0.000	0.000	0.000
3.5.4.26|g__Turicimonas.s__Turicimonas_muris	+	5.49E-01	0.000	0.000	0.000	0.000	0.000
3.5.4.26|g__Veillonella.s__Veillonella_atypica	+	7.79E-02	0.000	0.000	0.000	0.000	0.000
3.5.4.26|g__Veillonella.s__Veillonella_dispar	+	5.18E-01	0.000	0.000	0.000	0.004	0.001
3.5.4.26|g__Veillonella.s__Veillonella_infantium	-	8.03E-01	0.000	0.000	0.000	0.000	0.000
3.5.4.26|g__Veillonella.s__Veillonella_parvula	+	4.08E-01	0.000	0.000	0.000	0.003	0.001
3.5.4.26|g__Veillonella.s__Veillonella_rogosae	+	1.05E-01	0.000	0.000	0.000	0.001	0.000
3.5.4.26|g__Veillonella.s__Veillonella_tobetsuensis	+	4.17E-01	0.000	0.000	0.000	0.000	0.000
3.5.4.26|g__Victivallales_unclassified.s__Victivallales_bacterium_CCUG_4	+	5.49E-01	0.000	0.000	0.000	0.000	0.001
3.5.4.26|g__Victivallis.s__Victivallis_vadensis	+	5.69E-01	0.000	0.000	0.000	0.000	0.000
3.5.4.26|g__Bacteroides.s__Bacteroides_nordii	-	7.12E-01	0.102	0.121	0.020	0.005	0.006
3.5.4.26|g__Blautia.s__Blautia_obeum	+	1.44E-01	0.111	0.131	0.020	0.002	0.003
3.5.4.26|g__Dorea.s__Dorea_longicatena	-	9.51E-01	0.000	0.031	0.031	0.001	0.001
3.5.4.26|g__Roseburia.s__Roseburia_hominis	+	1.36E-01	0.172	0.216	0.044	0.004	0.006
3.5.4.26|g__Blautia.s__Ruminococcus_torques	+	3.06E-01	0.190	0.235	0.046	0.003	0.005
3.5.4.26|g__Bilophila.s__Bilophila_wadsworthia	+	1.92E-01	0.033	0.098	0.065	0.001	0.002
3.5.4.26|g__Lachnospiraceae_unclassified.s__Eubacterium_rectale	+	9.64E-01	0.093	0.196	0.104	0.006	0.005
3.5.4.26|g__Bacteroides.s__Bacteroides_thetaiotaomicron	-	9.54E-01	1.439	1.581	0.143	0.025	0.039
3.5.4.26|g__Phascolarctobacterium.s__Phascolarctobacterium_faecium	+	5.68E-01	0.630	0.808	0.178	0.014	0.019
3.5.4.26|g__Odoribacter.s__Odoribacter_splanchnicus	-	8.65E-01	0.072	0.270	0.197	0.005	0.005
3.5.4.26|g__Eubacterium.s__Eubacterium_eligens	+	5.32E-02	0.276	0.551	0.274	0.007	0.021
3.5.4.26|g__Parabacteroides.s__Parabacteroides_merdae	+	2.13E-01	0.041	0.423	0.382	0.009	0.015
3.5.4.26|g__Bacteroides.s__Bacteroides_stercoris	+	9.01E-01	0.385	0.803	0.418	0.062	0.041
3.5.4.26|g__Bacteroides.s__Bacteroides_dorei	+	4.67E-01	0.141	0.618	0.478	0.053	0.040
3.5.4.26|g__Bacteroides.s__Bacteroides_caccae	+	3.04E-01	0.235	0.935	0.700	0.016	0.026

	3.5.4.26|g__Bacteroides.s__Bacteroides_intestinalis	+	3.47E-02	0.239	1.174	0.935	0.007	0.024
	3.5.4.26|unclassified	+	3.23E-02	3.802	5.228	1.426	0.042	0.074
	3.5.4.26|g__Bacteroides.s__Bacteroides_uniformis	+	2.24E-01	6.212	9.553	3.341	0.077	0.130
China2	3.5.4.26	-	4.83E-03	136.921	123.031	-13.890	1.000	1.000
	3.5.4.26|g__Bacteroides.s__Bacteroides_vulgatus	-	7.77E-02	12.107	6.396	-5.711	0.161	0.098
	3.5.4.26|g__Faecalibacterium.s__Faecalibacterium_prausnitzii	-	2.59E-01	6.772	4.660	-2.113	0.062	0.056
	3.5.4.26|g__Phascolarctobacterium.s__Phascolarctobacterium_faecium	-	3.99E-01	0.816	0.293	-0.523	0.009	0.010
	3.5.4.26|g__Bacteroides.s__Bacteroides_xylanisolvens	-	1.16E-01	0.641	0.157	-0.484	0.017	0.010
	3.5.4.26|g__Bacteroides.s__Bacteroides_dorei	-	1.71E-01	0.883	0.496	-0.387	0.052	0.032
	3.5.4.26|g__Clostridium.s__Clostridium_sp_AM22_11AC	-	2.34E-01	0.555	0.307	-0.248	0.007	0.006
	3.5.4.26|g__Parabacteroides.s__Parabacteroides_distasonis	+	5.22E-01	1.182	0.984	-0.197	0.012	0.021
	3.5.4.26|g__Blautia.s__Blautia_wexlerae	-	9.24E-01	0.409	0.268	-0.141	0.008	0.012
	3.5.4.26|g__Bacteroides.s__Bacteroides_thetaiotaomicron	-	2.07E-01	0.469	0.361	-0.108	0.011	0.009
	3.5.4.26|g__Lachnospiraceae_unclassified.s__Eubacterium_rectale	-	7.43E-01	0.358	0.302	-0.056	0.008	0.007
	3.5.4.26|g__Blautia.s__Blautia_obeum	-	6.75E-01	0.436	0.386	-0.050	0.005	0.008
	3.5.4.26|g__Haemophilus.s__Haemophilus_parainfluenzae	-	1.93E-01	0.038	0.000	-0.038	0.002	0.002
	3.5.4.26|g__Agathobaculum.s__Agathobaculum_butyriciproducens	-	1.74E-01	0.037	0.000	-0.037	0.001	0.001
	3.5.4.26|g__Veillonella.s__Veillonella_parvula	-	4.78E-01	0.030	0.000	-0.030	0.001	0.001
	3.5.4.26|g__Bacteroides.s__Bacteroides_cellulosilyticus	-	9.16E-01	0.038	0.012	-0.026	0.005	0.008
	3.5.4.26|g__Escherichia.s__Escherichia_coli	+	5.47E-01	0.124	0.120	-0.004	0.004	0.004
	3.5.4.26|g__Acidaminococcus.s__Acidaminococcus_intestini	-	5.69E-01	0.000	0.000	0.000	0.001	0.001
	3.5.4.26|g__Adlercreutzia.s__Adlercreutzia_equolifaciens	+	3.58E-01	0.000	0.000	0.000	0.000	0.000
	3.5.4.26|g__Aggregatibacter.s__Aggregatibacter_segnis	-	3.30E-01	0.000	0.000	0.000	0.000	0.000
	3.5.4.26|g__Akkermansia.s__Akkermansia_muciniphila	+	4.63E-01	0.000	0.000	0.000	0.002	0.010
	3.5.4.26|g__Alcaligenes.s__Alcaligenes_faecalis	+	3.30E-01	0.000	0.000	0.000	0.000	0.002
	3.5.4.26|g__Allisonella.s__Allisonella_histaminiformans	+	8.01E-01	0.000	0.000	0.000	0.000	0.000
	3.5.4.26|g__Anaeromassilibacillus.s__Anaeromassilibacillus_sp_An250	+	3.08E-01	0.000	0.000	0.000	0.000	0.000
	3.5.4.26|g__Anaerotignum.s__Anaerotignum_lactatifermentans	-	1.60E-01	0.000	0.000	0.000	0.000	0.000
	3.5.4.26|g__Asaccharobacter.s__Asaccharobacter_celatus	+	3.69E-01	0.000	0.000	0.000	0.000	0.000
	3.5.4.26|g__Atlantibacter.s__Atlantibacter_hermannii	-	3.30E-01	0.000	0.000	0.000	0.000	0.000
	3.5.4.26|g__Bacteroides.s__Bacteroides_clarus	-	4.15E-01	0.000	0.000	0.000	0.001	0.004
	3.5.4.26|g__Bacteroides.s__Bacteroides_coprocola	-	1.31E-01	0.000	0.000	0.000	0.027	0.009
	3.5.4.26|g__Bacteroides.s__Bacteroides_coprophilus	+	2.85E-01	0.000	0.000	0.000	0.004	0.005
	3.5.4.26|g__Bacteroides.s__Bacteroides_eggerthii	-	6.95E-01	0.000	0.000	0.000	0.009	0.003
	3.5.4.26|g__Bacteroides.s__Bacteroides_faecis	+	3.93E-01	0.000	0.000	0.000	0.000	0.002
	3.5.4.26|g__Bacteroides.s__Bacteroides_finegoldii	-	1.06E-01	0.000	0.000	0.000	0.009	0.002
	3.5.4.26|g__Bacteroides.s__Bacteroides_massiliensis	-	6.03E-01	0.000	0.000	0.000	0.019	0.005
	3.5.4.26|g__Bacteroides.s__Bacteroides_nordii	+	5.85E-02	0.000	0.000	0.000	0.001	0.002
	3.5.4.26|g__Bacteroides.s__Bacteroides_oleiciplenus	-	1.60E-01	0.000	0.000	0.000	0.000	0.000
	3.5.4.26|g__Bacteroides.s__Bacteroides_plebeius	-	3.42E-01	0.000	0.000	0.000	0.093	0.037
	3.5.4.26|g__Bacteroides.s__Bacteroides_salyersiae	-	5.38E-01	0.000	0.000	0.000	0.001	0.001
	3.5.4.26|g__Bacteroides.s__Bacteroides_sartorii	-	3.30E-01	0.000	0.000	0.000	0.000	0.000
	3.5.4.26|g__Bacteroides.s__Bacteroides_stercorirosoris	+	5.89E-01	0.000	0.000	0.000	0.000	0.000
	3.5.4.26|g__Barnesiella.s__Barnesiella_intestinihominis	+	6.65E-01	0.000	0.000	0.000	0.003	0.006
	3.5.4.26|g__Blautia.s__Blautia_hansenii	-	1.75E-01	0.000	0.000	0.000	0.001	0.001
	3.5.4.26|g__Blautia.s__Blautia_sp_AF19_10LB	+	3.12E-01	0.000	0.000	0.000	0.002	0.005
	3.5.4.26|g__Butyricimonas.s__Butyricimonas_synergistica	+	1.00E+00	0.000	0.000	0.000	0.000	0.000
	3.5.4.26|g__Butyricimonas.s__Butyricimonas_virosa	+	2.19E-01	0.000	0.000	0.000	0.001	0.002
	3.5.4.26|g__Butyrivibrio.s__Butyrivibrio_crossotus	+	3.30E-01	0.000	0.000	0.000	0.000	0.006
	3.5.4.26|g__Campylobacter.s__Campylobacter_hominis	+	1.60E-01	0.000	0.000	0.000	0.000	0.000
	3.5.4.26|g__Catenibacterium.s__Catenibacterium_mitsuokai	+	5.49E-01	0.000	0.000	0.000	0.000	0.000
	3.5.4.26|g__Cetobacterium.s__Cetobacterium_somerae	-	3.30E-01	0.000	0.000	0.000	0.000	0.000
	3.5.4.26|g__Chryseobacterium.s__Chryseobacterium_sp_VAUSW3	+	3.30E-01	0.000	0.000	0.000	0.000	0.000
	3.5.4.26|g__Chryseobacterium.s__Chryseobacterium_sp_YLOS41	+	3.30E-01	0.000	0.000	0.000	0.000	0.000
	3.5.4.26|g__Citrobacter.s__Citrobacter_amalonaticus	-	5.89E-01	0.000	0.000	0.000	0.000	0.000
	3.5.4.26|g__Citrobacter.s__Citrobacter_braakii	+	1.60E-01	0.000	0.000	0.000	0.000	0.000
	3.5.4.26|g__Citrobacter.s__Citrobacter_freundii	+	6.87E-01	0.000	0.000	0.000	0.000	0.001
	3.5.4.26|g__Citrobacter.s__Citrobacter_portucalensis	-	6.92E-01	0.000	0.000	0.000	0.000	0.004
	3.5.4.26|g__Citrobacter.s__Citrobacter_werkmanii	+	1.00E+00	0.000	0.000	0.000	0.000	0.000
	3.5.4.26|g__Citrobacter.s__Citrobacter_youngae	-	7.92E-01	0.000	0.000	0.000	0.000	0.003
	3.5.4.26|g__Cloacibacillus.s__Cloacibacillus_porcorum	+	3.30E-01	0.000	0.000	0.000	0.000	0.000
	3.5.4.26|g__Clostridioides.s__Clostridioides_difficile	+	7.46E-02	0.000	0.000	0.000	0.000	0.001
	3.5.4.26|g__Clostridium.s__Clostridium_butyricum	+	3.30E-01	0.000	0.000	0.000	0.000	0.000
	3.5.4.26|g__Clostridium.s__Clostridium_celatum	+	3.30E-01	0.000	0.000	0.000	0.000	0.000
	3.5.4.26|g__Clostridium.s__Clostridium_disporicum	-	5.61E-02	0.000	0.000	0.000	0.001	0.000
	3.5.4.26|g__Clostridium.s__Clostridium_perfringens	-	8.19E-02	0.000	0.000	0.000	0.000	0.000
	3.5.4.26|g__Clostridium.s__Clostridium_sp_AF36_4	+	8.93E-02	0.000	0.000	0.000	0.000	0.001
	3.5.4.26|g__Comamonas.s__Comamonas_kerstersii	-	1.00E+00	0.000	0.000	0.000	0.000	0.000
	3.5.4.26|g__Comamonas.s__Comamonas_testosteroni	+	3.30E-01	0.000	0.000	0.000	0.000	0.000
	3.5.4.26|g__Comamonas.s__Comamonas_thiooxydans	+	3.30E-01	0.000	0.000	0.000	0.000	0.000
	3.5.4.26|g__Coprobacillus.s__Coprobacillus_cateniformis	+	2.06E-02	0.000	0.000	0.000	0.000	0.002
	3.5.4.26|g__Coprobacter.s__Coprobacter_fastidiosus	+	9.14E-01	0.000	0.000	0.000	0.001	0.001

3.5.4.26|g__Coprobacter.s__Coprobacter_secundus	-	6.87E-01	0.000	0.000	0.000	0.000	0.000
3.5.4.26|g__Coprococcus.s__Coprococcus_catus	+	2.99E-01	0.000	0.000	0.000	0.000	0.001
3.5.4.26|g__Coprococcus.s__Coprococcus_comes	-	9.91E-01	0.000	0.000	0.000	0.001	0.001
3.5.4.26|g__Coprococcus.s__Coprococcus_eutactus	+	1.23E-01	0.000	0.000	0.000	0.003	0.005
3.5.4.26|g__Desulfovibrio.s__Desulfovibrio_fairfieldensis	-	5.89E-01	0.000	0.000	0.000	0.000	0.000
3.5.4.26|g__Desulfovibrio.s__Desulfovibrio_piger	+	1.85E-01	0.000	0.000	0.000	0.000	0.001
3.5.4.26|g__Desulfovibrio.s__Desulfovibrio_sp_AM18_2	+	8.19E-02	0.000	0.000	0.000	0.000	0.000
3.5.4.26|g__Desulfovibrionaceae_unclassified.s__Desulfovibrionaceae_bact	+	8.19E-02	0.000	0.000	0.000	0.000	0.000
3.5.4.26|g__Dialister.s__Dialister_pneumosintes	+	3.30E-01	0.000	0.000	0.000	0.000	0.000
3.5.4.26|g__Dialister.s__Dialister_succinatiphilus	+	9.90E-01	0.000	0.000	0.000	0.002	0.002
3.5.4.26|g__Dorea.s__Dorea_sp_OM02_2LB	+	4.30E-01	0.000	0.000	0.000	0.000	0.000
3.5.4.26|g__Eggerthella.s__Eggerthella_lenta	+	5.80E-01	0.000	0.000	0.000	0.001	0.002
3.5.4.26|g__Enterobacter.s__Enterobacter_bugandensis	-	4.94E-01	0.000	0.000	0.000	0.000	0.000
3.5.4.26|g__Enterobacter.s__Enterobacter_cloacae	+	9.21E-01	0.000	0.000	0.000	0.000	0.002
3.5.4.26|g__Enterobacter.s__Enterobacter_mori	-	1.60E-01	0.000	0.000	0.000	0.000	0.000
3.5.4.26|g__Enterococcus.s__Enterococcus_hirae	+	3.30E-01	0.000	0.000	0.000	0.000	0.000
3.5.4.26|g__Erysipelatoclostridium.s__Clostridium_innocuum	+	7.46E-02	0.000	0.000	0.000	0.000	0.001
3.5.4.26|g__Erysipelatoclostridium.s__Clostridium_spiroforme	+	3.20E-01	0.000	0.000	0.000	0.000	0.001
3.5.4.26|g__Erysipelotrichaceae_unclassified.s__Erysipelotrichaceae_bacteri	-	1.60E-01	0.000	0.000	0.000	0.000	0.000
3.5.4.26|g__Escherichia.s__Escherichia_fergusonii	+	7.00E-01	0.000	0.000	0.000	0.000	0.000
3.5.4.26|g__Eubacterium.s__Eubacterium_ramulus	+	2.48E-01	0.000	0.000	0.000	0.000	0.001
3.5.4.26|g__Eubacterium.s__Eubacterium_sp_AF17_7	+	9.90E-01	0.000	0.000	0.000	0.000	0.000
3.5.4.26|g__Eubacterium.s__Eubacterium_sp_AM18_10LB_B	-	4.05E-01	0.000	0.000	0.000	0.000	0.000
3.5.4.26|g__Faecalicatena.s__Faecalicatena_contorta	+	2.59E-01	0.000	0.000	0.000	0.000	0.000
3.5.4.26|g__Firmicutes_unclassified.s__Firmicutes_bacterium_AM10_47	-	6.29E-01	0.000	0.000	0.000	0.001	0.000
3.5.4.26|g__Flavonifractor.s__Flavonifractor_plautii	+	5.49E-01	0.000	0.000	0.000	0.000	0.000
3.5.4.26|g__Fusobacterium.s__Fusobacterium_mortiferum	-	6.89E-01	0.000	0.000	0.000	0.001	0.003
3.5.4.26|g__Fusobacterium.s__Fusobacterium_nucleatum	+	1.00E+00	0.000	0.000	0.000	0.000	0.000
3.5.4.26|g__Fusobacterium.s__Fusobacterium_periodonticum	-	1.58E-01	0.000	0.000	0.000	0.000	0.000
3.5.4.26|g__Fusobacterium.s__Fusobacterium_ulcerans	+	9.90E-01	0.000	0.000	0.000	0.000	0.000
3.5.4.26|g__Fusobacterium.s__Fusobacterium_varium	-	1.60E-01	0.000	0.000	0.000	0.000	0.000
3.5.4.26|g__Haemophilus.s__Haemophilus_haemolyticus	+	3.30E-01	0.000	0.000	0.000	0.000	0.000
3.5.4.26|g__Haemophilus.s__Haemophilus_influenzae	+	3.30E-01	0.000	0.000	0.000	0.000	0.000
3.5.4.26|g__Haemophilus.s__Haemophilus_paraphrohaemolyticus	-	3.30E-01	0.000	0.000	0.000	0.000	0.000
3.5.4.26|g__Holdemanella.s__Holdemanella_biformis	+	5.07E-01	0.000	0.000	0.000	0.000	0.000
3.5.4.26|g__Intestinibacter.s__Intestinibacter_bartlettii	+	7.82E-01	0.000	0.000	0.000	0.000	0.000
3.5.4.26|g__Klebsiella.s__Klebsiella_aerogenes	-	5.68E-01	0.000	0.000	0.000	0.000	0.001
3.5.4.26|g__Klebsiella.s__Klebsiella_grimontii	+	3.30E-01	0.000	0.000	0.000	0.000	0.000
3.5.4.26|g__Klebsiella.s__Klebsiella_michiganensis	-	7.06E-01	0.000	0.000	0.000	0.000	0.000
3.5.4.26|g__Klebsiella.s__Klebsiella_variicola	+	1.94E-01	0.000	0.000	0.000	0.002	0.002
3.5.4.26|g__Kluyvera.s__Kluyvera_ascorbata	+	3.30E-01	0.000	0.000	0.000	0.000	0.000
3.5.4.26|g__Kluyvera.s__Kluyvera_cryocrescens	+	3.30E-01	0.000	0.000	0.000	0.000	0.000
3.5.4.26|g__Kluyvera.s__Kluyvera_georgiana	-	3.30E-01	0.000	0.000	0.000	0.000	0.000
3.5.4.26|g__Lachnoclostridium.s__Clostridium_aldenense	+	9.13E-02	0.000	0.000	0.000	0.000	0.001
3.5.4.26|g__Lachnoclostridium.s__Clostridium_citroniae	+	7.81E-03	0.000	0.000	0.000	0.000	0.001
3.5.4.26|g__Lachnoclostridium.s__Clostridium_clostridioforme	-	8.22E-01	0.000	0.000	0.000	0.001	0.000
3.5.4.26|g__Lachnoclostridium.s__Clostridium_symbiosum	+	5.34E-01	0.000	0.000	0.000	0.000	0.000
3.5.4.26|g__Lachnospira.s__Lachnospira_pectinoschiza	-	9.52E-01	0.000	0.000	0.000	0.006	0.004
3.5.4.26|g__Lactobacillus.s__Lactobacillus_amylovorus	+	3.30E-01	0.000	0.000	0.000	0.000	0.000
3.5.4.26|g__Lactobacillus.s__Lactobacillus_fermentum	+	9.90E-01	0.000	0.000	0.000	0.000	0.000
3.5.4.26|g__Lactobacillus.s__Lactobacillus_paralimentarius	-	3.30E-01	0.000	0.000	0.000	0.000	0.000
3.5.4.26|g__Lactobacillus.s__Lactobacillus_rogosae	-	8.56E-01	0.000	0.000	0.000	0.006	0.004
3.5.4.26|g__Lactobacillus.s__Lactobacillus_zymae	-	3.30E-01	0.000	0.000	0.000	0.000	0.000
3.5.4.26|g__Leclercia.s__Leclercia_adecarboxylata	-	3.03E-01	0.000	0.000	0.000	0.000	0.000
3.5.4.26|g__Lelliottia.s__Lelliottia_amnigena	+	3.30E-01	0.000	0.000	0.000	0.000	0.000
3.5.4.26|g__Lelliottia.s__Lelliottia_nimipressuralis	-	3.24E-01	0.000	0.000	0.000	0.000	0.001
3.5.4.26|g__Megamonas.s__Megamonas_funiformis	+	9.41E-01	0.000	0.000	0.000	0.011	0.006
3.5.4.26|g__Megamonas.s__Megamonas_hypermegale	-	9.82E-01	0.000	0.000	0.000	0.009	0.004
3.5.4.26|g__Megamonas.s__Megamonas_rupellensis	+	9.41E-01	0.000	0.000	0.000	0.009	0.004
3.5.4.26|g__Megasphaera.s__Megasphaera_elsdenii	+	3.30E-01	0.000	0.000	0.000	0.000	0.000
3.5.4.26|g__Megasphaera.s__Megasphaera_micronuciformis	+	1.72E-01	0.000	0.000	0.000	0.000	0.000
3.5.4.26|g__Megasphaera.s__Megasphaera_stantonii	+	3.30E-01	0.000	0.000	0.000	0.000	0.000
3.5.4.26|g__Mesosutterella.s__Mesosutterella_multiformis	-	5.55E-01	0.000	0.000	0.000	0.002	0.001
3.5.4.26|g__Mitsuokella.s__Mitsuokella_jalaludinii	+	3.30E-01	0.000	0.000	0.000	0.000	0.000
3.5.4.26|g__Mitsuokella.s__Mitsuokella_multacida	+	1.60E-01	0.000	0.000	0.000	0.000	0.001
3.5.4.26|g__Morganella.s__Morganella_morganii	+	3.30E-01	0.000	0.000	0.000	0.000	0.004
3.5.4.26|g__Oxalobacter.s__Oxalobacter_formigenes	+	2.64E-02	0.000	0.000	0.000	0.000	0.000
3.5.4.26|g__Pantoea.s__Pantoea_dispersa	+	3.30E-01	0.000	0.000	0.000	0.000	0.000
3.5.4.26|g__Pantoea.s__Pantoea_sesami	-	7.94E-01	0.000	0.000	0.000	0.000	0.000
3.5.4.26|g__Parabacteroides.s__Parabacteroides_chinchillae	+	3.30E-01	0.000	0.000	0.000	0.000	0.000
3.5.4.26|g__Parabacteroides.s__Parabacteroides_goldsteinii	+	2.24E-01	0.000	0.000	0.000	0.000	0.002
3.5.4.26|g__Parabacteroides.s__Parabacteroides_gordonii	+	1.60E-01	0.000	0.000	0.000	0.000	0.001

	3.5.4.26|g__Parabacteroides.s__Parabacteroides_johnsonii	+	6.79E-01	0.000	0.000	0.000	0.001	0.002
	3.5.4.26|g__Paraprevotella.s__Paraprevotella_clara	-	8.92E-01	0.000	0.000	0.000	0.003	0.002
	3.5.4.26|g__Paraprevotella.s__Paraprevotella_xylaniphila	-	3.30E-01	0.000	0.000	0.000	0.000	0.000
	3.5.4.26|g__Parasutterella.s__Parasutterella_excrementihominis	+	6.17E-01	0.000	0.000	0.000	0.003	0.002
	3.5.4.26|g__Pedobacter.s__Pedobacter_himalayensis	-	8.04E-01	0.000	0.000	0.000	0.000	0.000
	3.5.4.26|g__Phascolarctobacterium.s__Phascolarctobacterium_succinatutens	+	9.77E-01	0.000	0.000	0.000	0.002	0.002
	3.5.4.26|g__Prevotella.s__Prevotella_copri	+	8.81E-01	0.000	0.000	0.000	0.110	0.166
	3.5.4.26|g__Prevotella.s__Prevotella_corporis	+	1.60E-01	0.000	0.000	0.000	0.000	0.000
	3.5.4.26|g__Prevotella.s__Prevotella_sp_109	-	1.00E+00	0.000	0.000	0.000	0.003	0.003
	3.5.4.26|g__Prevotella.s__Prevotella_sp_AM42_24	-	9.90E-01	0.000	0.000	0.000	0.005	0.002
	3.5.4.26|g__Proteus.s__Proteus_hauseri	-	1.00E+00	0.000	0.000	0.000	0.000	0.000
	3.5.4.26|g__Proteus.s__Proteus_mirabilis	-	5.69E-01	0.000	0.000	0.000	0.000	0.000
	3.5.4.26|g__Proteus.s__Proteus_penneri	-	1.00E+00	0.000	0.000	0.000	0.000	0.000
	3.5.4.26|g__Proteus.s__Proteus_vulgaris	+	1.00E+00	0.000	0.000	0.000	0.000	0.000
	3.5.4.26|g__Providencia.s__Providencia_rettgeri	+	3.30E-01	0.000	0.000	0.000	0.000	0.005
	3.5.4.26|g__Pyramidobacter.s__Pyramidobacter_piscolens	+	2.96E-01	0.000	0.000	0.000	0.000	0.000
	3.5.4.26|g__Pyramidobacter.s__Pyramidobacter_sp_C12_8	+	3.30E-01	0.000	0.000	0.000	0.000	0.000
	3.5.4.26|g__Raoultella.s__Raoultella_ornithinolytica	+	5.69E-01	0.000	0.000	0.000	0.000	0.000
	3.5.4.26|g__Raoultella.s__Raoultella_planticola	+	1.00E+00	0.000	0.000	0.000	0.000	0.000
	3.5.4.26|g__Roseburia.s__Roseburia_faecis	-	8.93E-02	0.000	0.000	0.000	0.006	0.002
	3.5.4.26|g__Roseburia.s__Roseburia_intestinalis	-	4.98E-01	0.000	0.000	0.000	0.001	0.001
	3.5.4.26|g__Roseburia.s__Roseburia_inulinivorans	-	7.12E-01	0.000	0.000	0.000	0.001	0.001
	3.5.4.26|g__Ruminococcaceae_unclassified.s__Ruminococcaceae_bacteriun	+	5.89E-01	0.000	0.000	0.000	0.000	0.000
	3.5.4.26|g__Ruminococcaceae_unclassified.s__Ruminococcaceae_bacteriun	+	2.24E-02	0.000	0.000	0.000	0.000	0.000
	3.5.4.26|g__Ruminococcus.s__Ruminococcus_callidus	+	6.10E-01	0.000	0.000	0.000	0.001	0.001
	3.5.4.26|g__Ruminococcus.s__Ruminococcus_sp_AF31_8BH	-	6.77E-01	0.000	0.000	0.000	0.001	0.001
	3.5.4.26|g__Saccharomyces.s__Saccharomyces_cerevisiae	-	3.30E-01	0.000	0.000	0.000	0.000	0.000
	3.5.4.26|g__Salmonella.s__Salmonella_enterica	-	7.19E-01	0.000	0.000	0.000	0.000	0.000
	3.5.4.26|g__Staphylococcus.s__Staphylococcus_epidermidis	-	3.30E-01	0.000	0.000	0.000	0.000	0.000
	3.5.4.26|g__Staphylococcus.s__Staphylococcus_pasteuri	-	3.30E-01	0.000	0.000	0.000	0.000	0.000
	3.5.4.26|g__Staphylococcus.s__Staphylococcus_warneri	-	3.30E-01	0.000	0.000	0.000	0.000	0.000
	3.5.4.26|g__Streptococcus.s__Streptococcus_equinus	-	5.69E-01	0.000	0.000	0.000	0.000	0.000
	3.5.4.26|g__Streptococcus.s__Streptococcus_gallolyticus	+	3.30E-01	0.000	0.000	0.000	0.000	0.000
	3.5.4.26|g__Streptococcus.s__Streptococcus_infantarius	-	3.30E-01	0.000	0.000	0.000	0.000	0.000
	3.5.4.26|g__Streptococcus.s__Streptococcus_macedonicus	-	3.30E-01	0.000	0.000	0.000	0.000	0.000
	3.5.4.26|g__Streptococcus.s__Streptococcus_mitis	-	1.60E-01	0.000	0.000	0.000	0.000	0.000
	3.5.4.26|g__Streptococcus.s__Streptococcus_oralis	-	1.60E-01	0.000	0.000	0.000	0.000	0.000
	3.5.4.26|g__Streptococcus.s__Streptococcus_pasteurianus	+	1.00E+00	0.000	0.000	0.000	0.000	0.000
	3.5.4.26|g__Streptococcus.s__Streptococcus_pneumoniae	-	3.93E-01	0.000	0.000	0.000	0.000	0.000
	3.5.4.26|g__Streptococcus.s__Streptococcus_pseudopneumoniae	-	1.60E-01	0.000	0.000	0.000	0.000	0.000
	3.5.4.26|g__Sutterella.s__Sutterella_wadsworthensis	+	7.06E-01	0.000	0.000	0.000	0.001	0.000
	3.5.4.26|g__Veillonella.s__Veillonella_atypica	+	9.40E-01	0.000	0.000	0.000	0.000	0.000
	3.5.4.26|g__Veillonella.s__Veillonella_dispar	-	8.00E-01	0.000	0.000	0.000	0.000	0.000
	3.5.4.26|g__Veillonella.s__Veillonella_infantium	-	7.91E-01	0.000	0.000	0.000	0.000	0.001
	3.5.4.26|g__Veillonella.s__Veillonella_rogosae	-	1.01E-02	0.000	0.000	0.000	0.001	0.000
	3.5.4.26|g__Veillonella.s__Veillonella_tobetsuensis	-	1.91E-01	0.000	0.000	0.000	0.001	0.000
	3.5.4.26|g__Victivallales_unclassified.s__Victivallales_bacterium_CCUG_4	+	6.10E-02	0.000	0.000	0.000	0.000	0.001
	3.5.4.26|g__Victivallis.s__Victivallis_vadensis	+	2.68E-01	0.000	0.000	0.000	0.000	0.001
	3.5.4.26|g__Weissella.s__Weissella_confusa	-	1.00E+00	0.000	0.000	0.000	0.000	0.000
	3.5.4.26|g__Yokenella.s__Yokenella_regensburgei	-	3.48E-01	0.000	0.000	0.000	0.000	0.000
	3.5.4.26|g__Dorea.s__Dorea_formicigenerans	-	6.06E-01	0.056	0.058	0.002	0.001	0.001
	3.5.4.26|g__Roseburia.s__Roseburia_hominis	+	5.99E-01	0.238	0.257	0.019	0.003	0.005
	3.5.4.26|g__Lachnoclostridium.s__Clostridium_bolteae	+	1.18E-01	0.000	0.028	0.028	0.001	0.001
	3.5.4.26|g__Hungatella.s__Hungatella_hathewayi	+	4.02E-01	0.000	0.029	0.029	0.001	0.001
	3.5.4.26|g__Anaerostipes.s__Anaerostipes_hadrus	-	9.34E-01	0.142	0.174	0.031	0.003	0.004
	3.5.4.26|g__Dorea.s__Dorea_longicatena	+	2.40E-01	0.133	0.167	0.034	0.001	0.002
	3.5.4.26|g__Klebsiella.s__Klebsiella_oxytoca	+	1.13E-01	0.015	0.068	0.053	0.002	0.003
	3.5.4.26|g__Bacteroides.s__Bacteroides_fragilis	-	9.08E-01	0.140	0.238	0.099	0.013	0.015
	3.5.4.26|g__Bacteroides.s__Bacteroides_intestinalis	+	1.76E-01	0.000	0.104	0.104	0.004	0.006
	3.5.4.26|g__Blautia.s__Ruminococcus_torques	+	4.84E-01	0.364	0.494	0.130	0.005	0.007
	3.5.4.26|g__Eubacterium.s__Eubacterium_eligens	+	6.48E-01	0.000	0.240	0.240	0.006	0.004
	3.5.4.26|g__Bilophila.s__Bilophila_wadsworthia	+	9.43E-04	0.094	0.340	0.246	0.001	0.004
	3.5.4.26|g__Odoribacter.s__Odoribacter_splanchnicus	+	1.19E-01	0.143	0.394	0.251	0.002	0.004
	3.5.4.26|g__Bacteroides.s__Bacteroides_ovatus	+	9.28E-01	0.382	0.698	0.316	0.023	0.019
	3.5.4.26|g__Parabacteroides.s__Parabacteroides_merdae	+	2.02E-01	0.416	0.732	0.317	0.006	0.013
	3.5.4.26|g__Bacteroides.s__Bacteroides_caccae	+	3.73E-01	0.179	0.522	0.343	0.008	0.011
	3.5.4.26|g__Klebsiella.s__Klebsiella_pneumoniae	+	1.89E-01	0.059	0.411	0.353	0.006	0.024
	3.5.4.26|unclassified	+	8.73E-01	8.456	9.019	0.564	0.079	0.099
	3.5.4.26|g__Bacteroides.s__Bacteroides_stercoris	+	8.21E-01	0.415	1.032	0.617	0.043	0.049
	3.5.4.26|g__Bacteroides.s__Bacteroides_uniformis	+	4.72E-01	3.293	4.318	1.024	0.049	0.077
Taiwan	3.5.4.26	-	1.23E-02	121.545	111.321	-10.224	1.000	1.000
	3.5.4.26|g__Bacteroides.s__Bacteroides_vulgatus	-	5.38E-01	7.917	6.050	-1.866	0.094	0.083

3.5.4.26|g__Bacteroides.s__Bacteroides_plebeius	-	3.80E-01	1.064	0.300	-0.764	0.081	0.051
3.5.4.26|g__Lachnospiraceae_unclassified.s__Eubacterium_rectale	-	2.96E-02	0.389	0.081	-0.309	0.015	0.008
3.5.4.26|unclassified	+	8.88E-01	7.656	7.387	-0.270	0.074	0.109
3.5.4.26|g__Coprococcus.s__Coprococcus_comes	-	1.58E-01	0.094	0.000	-0.094	0.002	0.002
3.5.4.26|g__Escherichia.s__Escherichia_coli	-	7.02E-02	0.052	0.000	-0.052	0.003	0.002
3.5.4.26|g__Bacteroides.s__Bacteroides_xylanisolvens	+	6.16E-01	0.486	0.443	-0.044	0.016	0.017
3.5.4.26|g__Blautia.s__Blautia_wexlerae	-	6.53E-01	0.067	0.047	-0.019	0.001	0.001
3.5.4.26|g__Clostridium.s__Clostridium_sp_AM22_11AC	-	1.63E-01	0.164	0.146	-0.018	0.003	0.002
3.5.4.26|g__Agathobaculum.s__Agathobaculum_butyriciproducens	-	3.21E-01	0.123	0.106	-0.017	0.003	0.002
3.5.4.26|g__Dorea.s__Dorea_longicatena	+	9.95E-01	0.095	0.091	-0.004	0.002	0.003
3.5.4.26|g__Acidaminococcus.s__Acidaminococcus_fermentans	+	3.87E-01	0.000	0.000	0.000	0.000	0.000
3.5.4.26|g__Acidaminococcus.s__Acidaminococcus_intestini	-	9.16E-01	0.000	0.000	0.000	0.001	0.003
3.5.4.26|g__Adlercreutzia.s__Adlercreutzia_equolifaciens	-	4.71E-01	0.000	0.000	0.000	0.000	0.000
3.5.4.26|g__Aeromonas.s__Aeromonas_caviae	-	2.85E-01	0.000	0.000	0.000	0.000	0.000
3.5.4.26|g__Akkermansia.s__Akkermansia_muciniphila	+	5.22E-02	0.000	0.000	0.000	0.009	0.019
3.5.4.26|g__Allisonella.s__Allisonella_histaminiformans	-	1.94E-01	0.000	0.000	0.000	0.000	0.000
3.5.4.26|g__Anaeromassilibacillus.s__Anaeromassilibacillus_sp_An250	+	7.98E-01	0.000	0.000	0.000	0.000	0.000
3.5.4.26|g__Anaerostipes.s__Anaerostipes_hadrus	+	2.26E-01	0.000	0.000	0.000	0.001	0.001
3.5.4.26|g__Anaerotignum.s__Anaerotignum_lactatifermentans	-	8.32E-01	0.000	0.000	0.000	0.001	0.001
3.5.4.26|g__Asaccharobacter.s__Asaccharobacter_celatus	-	1.20E-01	0.000	0.000	0.000	0.000	0.000
3.5.4.26|g__Bacteroides.s__Bacteroides_clarus	-	8.42E-01	0.000	0.000	0.000	0.002	0.009
3.5.4.26|g__Bacteroides.s__Bacteroides_coprocola	+	7.98E-01	0.000	0.000	0.000	0.029	0.030
3.5.4.26|g__Bacteroides.s__Bacteroides_coprophilus	-	6.76E-01	0.000	0.000	0.000	0.006	0.006
3.5.4.26|g__Bacteroides.s__Bacteroides_eggerthii	+	2.45E-01	0.000	0.000	0.000	0.009	0.008
3.5.4.26|g__Bacteroides.s__Bacteroides_finegoldii	+	5.06E-01	0.000	0.000	0.000	0.005	0.006
3.5.4.26|g__Bacteroides.s__Bacteroides_fluxus	-	9.26E-01	0.000	0.000	0.000	0.000	0.002
3.5.4.26|g__Bacteroides.s__Bacteroides_fragilis	+	4.01E-01	0.000	0.000	0.000	0.007	0.012
3.5.4.26|g__Bacteroides.s__Bacteroides_nordii	+	5.91E-01	0.000	0.000	0.000	0.000	0.001
3.5.4.26|g__Bacteroides.s__Bacteroides_oleiciplenus	+	3.68E-01	0.000	0.000	0.000	0.000	0.000
3.5.4.26|g__Bacteroides.s__Bacteroides_salyersiae	-	6.63E-01	0.000	0.000	0.000	0.005	0.003
3.5.4.26|g__Bacteroides.s__Bacteroides_sartorii	+	6.86E-01	0.000	0.000	0.000	0.005	0.001
3.5.4.26|g__Bacteroides.s__Bacteroides_sp_OM08_11	-	8.72E-01	0.000	0.000	0.000	0.000	0.000
3.5.4.26|g__Barnesiella.s__Barnesiella_intestinihominis	+	5.60E-02	0.000	0.000	0.000	0.006	0.011
3.5.4.26|g__Blautia.s__Blautia_hansenii	+	8.24E-01	0.000	0.000	0.000	0.000	0.000
3.5.4.26|g__Blautia.s__Blautia_sp_AF19_10LB	-	8.02E-01	0.000	0.000	0.000	0.000	0.001
3.5.4.26|g__Butyricicoccus.s__Butyricicoccus_pullicaecorum	-	9.05E-01	0.000	0.000	0.000	0.000	0.000
3.5.4.26|g__Butyricimonas.s__Butyricimonas_synergistica	+	6.70E-01	0.000	0.000	0.000	0.000	0.000
3.5.4.26|g__Catenibacterium.s__Catenibacterium_mitsuokai	-	6.06E-01	0.000	0.000	0.000	0.007	0.008
3.5.4.26|g__Cetobacterium.s__Cetobacterium_somerae	+	3.68E-01	0.000	0.000	0.000	0.000	0.000
3.5.4.26|g__Citrobacter.s__Citrobacter_amalonaticus	-	2.85E-01	0.000	0.000	0.000	0.000	0.000
3.5.4.26|g__Citrobacter.s__Citrobacter_braakii	-	1.25E-01	0.000	0.000	0.000	0.000	0.000
3.5.4.26|g__Citrobacter.s__Citrobacter_farmeri	-	2.85E-01	0.000	0.000	0.000	0.000	0.000
3.5.4.26|g__Citrobacter.s__Citrobacter_freundii	-	8.00E-01	0.000	0.000	0.000	0.000	0.000
3.5.4.26|g__Citrobacter.s__Citrobacter_koseri	+	6.86E-01	0.000	0.000	0.000	0.000	0.000
3.5.4.26|g__Citrobacter.s__Citrobacter_portucalensis	-	8.42E-01	0.000	0.000	0.000	0.000	0.000
3.5.4.26|g__Citrobacter.s__Citrobacter_werkmanii	+	3.68E-01	0.000	0.000	0.000	0.000	0.000
3.5.4.26|g__Citrobacter.s__Citrobacter_youngae	-	8.08E-01	0.000	0.000	0.000	0.000	0.000
3.5.4.26|g__Cloacibacillus.s__Cloacibacillus_porcorum	+	1.26E-02	0.000	0.000	0.000	0.000	0.000
3.5.4.26|g__Clostridiales_unclassified.s__Clostridiales_bacterium_1_7_47F/	-	4.84E-01	0.000	0.000	0.000	0.000	0.000
3.5.4.26|g__Clostridioides.s__Clostridioides_difficile	+	3.12E-01	0.000	0.000	0.000	0.000	0.001
3.5.4.26|g__Clostridium.s__Butyribacterium_methylotrophicum	-	9.26E-01	0.000	0.000	0.000	0.000	0.000
3.5.4.26|g__Clostridium.s__Clostridium_disporicum	-	9.26E-01	0.000	0.000	0.000	0.000	0.000
3.5.4.26|g__Clostridium.s__Clostridium_neonatale	+	3.68E-01	0.000	0.000	0.000	0.000	0.000
3.5.4.26|g__Clostridium.s__Clostridium_perfringens	-	1.25E-01	0.000	0.000	0.000	0.001	0.000
3.5.4.26|g__Clostridium.s__Clostridium_sp_AF36_4	-	6.83E-01	0.000	0.000	0.000	0.001	0.000
3.5.4.26|g__Comamonas.s__Comamonas_kerstersii	+	3.68E-01	0.000	0.000	0.000	0.000	0.000
3.5.4.26|g__Coprobacillus.s__Coprobacillus_cateniformis	+	2.48E-01	0.000	0.000	0.000	0.000	0.000
3.5.4.26|g__Coprobacter.s__Coprobacter_fastidiosus	+	7.13E-01	0.000	0.000	0.000	0.001	0.006
3.5.4.26|g__Coprobacter.s__Coprobacter_secundus	+	3.70E-01	0.000	0.000	0.000	0.000	0.001
3.5.4.26|g__Coprococcus.s__Coprococcus_catus	+	8.06E-01	0.000	0.000	0.000	0.000	0.000
3.5.4.26|g__Coprococcus.s__Coprococcus_eutactus	+	6.29E-01	0.000	0.000	0.000	0.001	0.002
3.5.4.26|g__Desulfovibrio.s__Desulfovibrio_desulfuricans	+	3.68E-01	0.000	0.000	0.000	0.000	0.000
3.5.4.26|g__Desulfovibrio.s__Desulfovibrio_fairfieldensis	+	3.70E-02	0.000	0.000	0.000	0.000	0.000
3.5.4.26|g__Desulfovibrio.s__Desulfovibrio_piger	+	9.45E-01	0.000	0.000	0.000	0.001	0.001
3.5.4.26|g__Desulfovibrio.s__Desulfovibrio_sp_AM18_2	+	3.68E-01	0.000	0.000	0.000	0.000	0.000
3.5.4.26|g__Desulfovibrionaceae_unclassified.s__Desulfovibrionaceae_bact	-	2.83E-01	0.000	0.000	0.000	0.000	0.000
3.5.4.26|g__Dialister.s__Dialister_succinatiphilus	-	3.56E-01	0.000	0.000	0.000	0.001	0.001
3.5.4.26|g__Dorea.s__Dorea_formicigenerans	+	4.66E-01	0.000	0.000	0.000	0.000	0.001
3.5.4.26|g__Dorea.s__Dorea_sp_OM02_2LB	+	3.68E-01	0.000	0.000	0.000	0.000	0.000
3.5.4.26|g__Eggerthella.s__Eggerthella_lenta	+	6.69E-01	0.000	0.000	0.000	0.001	0.001
3.5.4.26|g__Enterobacter.s__Enterobacter_bugandensis	-	5.86E-02	0.000	0.000	0.000	0.000	0.000
3.5.4.26|g__Enterobacter.s__Enterobacter_cloacae	-	5.93E-02	0.000	0.000	0.000	0.000	0.000

3.5.4.26|g__Enterococcus.s__Enterococcus_faecium	+	3.68E-01	0.000	0.000	0.000	0.000	0.000
3.5.4.26|g__Erysipelatoclostridium.s__Clostridium_innocuum	+	1.71E-01	0.000	0.000	0.000	0.000	0.001
3.5.4.26|g__Erysipelatoclostridium.s__Clostridium_spiroforme	+	2.48E-01	0.000	0.000	0.000	0.000	0.000
3.5.4.26|g__Erysipelotrichaceae_unclassified.s__Erysipelotrichaceae_bacteri	-	2.85E-01	0.000	0.000	0.000	0.000	0.000
3.5.4.26|g__Escherichia.s__Escherichia_fergusonii	+	3.68E-01	0.000	0.000	0.000	0.000	0.000
3.5.4.26|g__Eubacterium.s__Eubacterium_callanderi	-	9.05E-01	0.000	0.000	0.000	0.000	0.000
3.5.4.26|g__Eubacterium.s__Eubacterium_limosum	+	3.68E-01	0.000	0.000	0.000	0.000	0.000
3.5.4.26|g__Eubacterium.s__Eubacterium_maltosivorans	+	3.68E-01	0.000	0.000	0.000	0.000	0.000
3.5.4.26|g__Eubacterium.s__Eubacterium_ramulus	-	4.08E-01	0.000	0.000	0.000	0.000	0.000
3.5.4.26|g__Eubacterium.s__Eubacterium_sp_AF17_7	+	5.93E-02	0.000	0.000	0.000	0.000	0.000
3.5.4.26|g__Eubacterium.s__Eubacterium_sp_AM18_10LB_B	-	9.26E-01	0.000	0.000	0.000	0.000	0.000
3.5.4.26|g__Faecalicatena.s__Faecalicatena_contorta	+	7.98E-01	0.000	0.000	0.000	0.000	0.000
3.5.4.26|g__Firmicutes_unclassified.s__Firmicutes_bacterium_AM10_47	+	6.54E-01	0.000	0.000	0.000	0.000	0.000
3.5.4.26|g__Flavonifractor.s__Flavonifractor_plautii	+	2.90E-01	0.000	0.000	0.000	0.000	0.000
3.5.4.26|g__Fusobacterium.s__Fusobacterium_mortiferum	-	3.24E-01	0.000	0.000	0.000	0.004	0.001
3.5.4.26|g__Fusobacterium.s__Fusobacterium_nucleatum	+	3.68E-01	0.000	0.000	0.000	0.000	0.000
3.5.4.26|g__Fusobacterium.s__Fusobacterium_ulcerans	+	2.28E-01	0.000	0.000	0.000	0.000	0.002
3.5.4.26|g__Fusobacterium.s__Fusobacterium_varium	-	4.57E-01	0.000	0.000	0.000	0.001	0.000
3.5.4.26|g__Haemophilus.s__Haemophilus_parahaemolyticus	+	3.68E-01	0.000	0.000	0.000	0.000	0.000
3.5.4.26|g__Haemophilus.s__Haemophilus_parainfluenzae	-	1.22E-01	0.000	0.000	0.000	0.004	0.001
3.5.4.26|g__Holdemanella.s__Holdemanella_biformis	+	2.80E-02	0.000	0.000	0.000	0.000	0.000
3.5.4.26|g__Hungatella.s__Hungatella_hathewayi	-	6.87E-01	0.000	0.000	0.000	0.001	0.001
3.5.4.26|g__Intestinibacter.s__Intestinibacter_bartlettii	+	3.68E-01	0.000	0.000	0.000	0.000	0.000
3.5.4.26|g__Klebsiella.s__Klebsiella_aerogenes	-	1.24E-01	0.000	0.000	0.000	0.000	0.000
3.5.4.26|g__Klebsiella.s__Klebsiella_grimontii	-	2.85E-01	0.000	0.000	0.000	0.000	0.000
3.5.4.26|g__Klebsiella.s__Klebsiella_michiganensis	-	4.57E-01	0.000	0.000	0.000	0.000	0.000
3.5.4.26|g__Klebsiella.s__Klebsiella_oxytoca	-	3.64E-01	0.000	0.000	0.000	0.007	0.001
3.5.4.26|g__Klebsiella.s__Klebsiella_pneumoniae	-	2.34E-01	0.000	0.000	0.000	0.008	0.003
3.5.4.26|g__Klebsiella.s__Klebsiella_variicola	+	5.82E-01	0.000	0.000	0.000	0.001	0.000
3.5.4.26|g__Kluyvera.s__Kluyvera_ascorbata	-	2.85E-01	0.000	0.000	0.000	0.000	0.000
3.5.4.26|g__Kluyvera.s__Kluyvera_georgiana	-	2.85E-01	0.000	0.000	0.000	0.000	0.000
3.5.4.26|g__Lachnoclostridium.s__Clostridium_aldenense	+	4.29E-01	0.000	0.000	0.000	0.000	0.000
3.5.4.26|g__Lachnoclostridium.s__Clostridium_bolteae	-	8.79E-01	0.000	0.000	0.000	0.001	0.001
3.5.4.26|g__Lachnoclostridium.s__Clostridium_citroniae	+	5.93E-01	0.000	0.000	0.000	0.000	0.000
3.5.4.26|g__Lachnoclostridium.s__Clostridium_clostridioforme	-	9.30E-01	0.000	0.000	0.000	0.000	0.000
3.5.4.26|g__Lachnoclostridium.s__Clostridium_symbiosum	+	2.44E-02	0.000	0.000	0.000	0.000	0.001
3.5.4.26|g__Lachnospira.s__Lachnospira_pectinoschiza	-	1.29E-01	0.000	0.000	0.000	0.001	0.001
3.5.4.26|g__Lactobacillus.s__Lactobacillus_fermentum	-	2.85E-01	0.000	0.000	0.000	0.000	0.000
3.5.4.26|g__Lactobacillus.s__Lactobacillus_plantarum	+	3.68E-01	0.000	0.000	0.000	0.000	0.000
3.5.4.26|g__Lactobacillus.s__Lactobacillus_rogosae	-	1.36E-01	0.000	0.000	0.000	0.002	0.001
3.5.4.26|g__Leclercia.s__Leclercia_adecarboxylata	-	1.25E-01	0.000	0.000	0.000	0.000	0.000
3.5.4.26|g__Lelliottia.s__Lelliottia_nimipressuralis	-	3.34E-01	0.000	0.000	0.000	0.000	0.000
3.5.4.26|g__Listeria.s__Listeria_monocytogenes	+	3.68E-01	0.000	0.000	0.000	0.000	0.000
3.5.4.26|g__Megamonas.s__Megamonas_funiformis	-	7.60E-01	0.000	0.000	0.000	0.002	0.002
3.5.4.26|g__Megamonas.s__Megamonas_hypermegale	-	4.38E-01	0.000	0.000	0.000	0.003	0.001
3.5.4.26|g__Megamonas.s__Megamonas_rupellensis	-	3.93E-01	0.000	0.000	0.000	0.003	0.002
3.5.4.26|g__Megasphaera.s__Megasphaera_elsdenii	-	1.25E-01	0.000	0.000	0.000	0.000	0.000
3.5.4.26|g__Megasphaera.s__Megasphaera_hexanoica	-	9.26E-01	0.000	0.000	0.000	0.000	0.000
3.5.4.26|g__Megasphaera.s__Megasphaera_micronuciformis	+	1.96E-01	0.000	0.000	0.000	0.000	0.000
3.5.4.26|g__Megasphaera.s__Megasphaera_stantonii	-	8.87E-01	0.000	0.000	0.000	0.000	0.000
3.5.4.26|g__Mesosutterella.s__Mesosutterella_multiformis	-	4.11E-01	0.000	0.000	0.000	0.003	0.003
3.5.4.26|g__Metakosakonia.s__Kluyvera_intestini	+	3.68E-01	0.000	0.000	0.000	0.000	0.000
3.5.4.26|g__Mitsuokella.s__Mitsuokella_jalaludinii	-	2.50E-01	0.000	0.000	0.000	0.000	0.000
3.5.4.26|g__Mitsuokella.s__Mitsuokella_multacida	-	5.21E-01	0.000	0.000	0.000	0.000	0.000
3.5.4.26|g__Odoribacter.s__Odoribacter_laneus	+	3.97E-01	0.000	0.000	0.000	0.000	0.002
3.5.4.26|g__Oxalobacter.s__Oxalobacter_formigenes	+	2.90E-01	0.000	0.000	0.000	0.000	0.000
3.5.4.26|g__Pantoea.s__Pantoea_sesami	-	5.02E-01	0.000	0.000	0.000	0.000	0.000
3.5.4.26|g__Parabacteroides.s__Parabacteroides_chinchillae	-	1.25E-01	0.000	0.000	0.000	0.000	0.000
3.5.4.26|g__Parabacteroides.s__Parabacteroides_goldsteinii	+	2.48E-02	0.000	0.000	0.000	0.001	0.003
3.5.4.26|g__Parabacteroides.s__Parabacteroides_gordonii	-	8.42E-01	0.000	0.000	0.000	0.000	0.000
3.5.4.26|g__Parabacteroides.s__Parabacteroides_johnsonii	+	8.22E-01	0.000	0.000	0.000	0.001	0.001
3.5.4.26|g__Paraprevotella.s__Paraprevotella_clara	-	6.06E-01	0.000	0.000	0.000	0.007	0.008
3.5.4.26|g__Paraprevotella.s__Paraprevotella_xylaniphila	+	8.39E-02	0.000	0.000	0.000	0.000	0.002
3.5.4.26|g__Parasutterella.s__Parasutterella_excrementihominis	+	5.28E-01	0.000	0.000	0.000	0.004	0.003
3.5.4.26|g__Pedobacter.s__Pedobacter_himalayensis	-	1.25E-01	0.000	0.000	0.000	0.000	0.000
3.5.4.26|g__Phascolarctobacterium.s__Phascolarctobacterium_succinatutens	+	3.98E-01	0.000	0.000	0.000	0.002	0.003
3.5.4.26|g__Plesiomonas.s__Plesiomonas_shigelloides	-	2.85E-01	0.000	0.000	0.000	0.001	0.000
3.5.4.26|g__Prevotella.s__Prevotella_buccae	-	2.85E-01	0.000	0.000	0.000	0.004	0.000
3.5.4.26|g__Prevotella.s__Prevotella_copri	+	8.41E-01	0.000	0.000	0.000	0.179	0.130
3.5.4.26|g__Prevotella.s__Prevotella_sp_109	+	6.55E-01	0.000	0.000	0.000	0.015	0.009
3.5.4.26|g__Prevotella.s__Prevotella_sp_AM42_24	-	2.85E-01	0.000	0.000	0.000	0.004	0.000
3.5.4.26|g__Pseudomonas.s__Pseudomonas_helleri	-	2.85E-01	0.000	0.000	0.000	0.000	0.000

	3.5.4.26|g__Pyramidobacter.s__Pyramidobacter_piscolens	+	5.56E-01	0.000	0.000	0.000	0.000	0.000
	3.5.4.26|g__Pyramidobacter.s__Pyramidobacter_sp_C12_8	+	3.68E-01	0.000	0.000	0.000	0.000	0.000
	3.5.4.26|g__Raoultella.s__Raoultella_ornithinolytica	-	4.57E-01	0.000	0.000	0.000	0.001	0.000
	3.5.4.26|g__Raoultella.s__Raoultella_planticola	+	3.68E-01	0.000	0.000	0.000	0.000	0.000
	3.5.4.26|g__Roseburia.s__Roseburia_faecis	+	3.56E-01	0.000	0.000	0.000	0.001	0.002
	3.5.4.26|g__Roseburia.s__Roseburia_intestinalis	-	3.34E-01	0.000	0.000	0.000	0.000	0.000
	3.5.4.26|g__Roseburia.s__Roseburia_inulinivorans	-	3.36E-01	0.000	0.000	0.000	0.001	0.000
	3.5.4.26|g__Ruminococcaceae_unclassified.s__Ruminococcaceae_bacteriun	-	5.33E-01	0.000	0.000	0.000	0.000	0.000
	3.5.4.26|g__Ruminococcaceae_unclassified.s__Ruminococcaceae_bacteriun	+	2.28E-01	0.000	0.000	0.000	0.000	0.000
	3.5.4.26|g__Ruminococcus.s__Ruminococcus_callidus	-	3.51E-01	0.000	0.000	0.000	0.001	0.001
	3.5.4.26|g__Ruminococcus.s__Ruminococcus_sp_AF31_8BH	+	8.07E-02	0.000	0.000	0.000	0.000	0.001
	3.5.4.26|g__Salmonella.s__Salmonella_enterica	-	2.80E-02	0.000	0.000	0.000	0.000	0.000
	3.5.4.26|g__Sanguibacteroides.s__Sanguibacteroides_justesenii	+	6.86E-01	0.000	0.000	0.000	0.000	0.000
	3.5.4.26|g__Slackia.s__Slackia_isoflavoniconvertens	+	1.96E-01	0.000	0.000	0.000	0.000	0.000
	3.5.4.26|g__Streptococcus.s__Streptococcus_equinus	+	1.96E-01	0.000	0.000	0.000	0.000	0.000
	3.5.4.26|g__Streptococcus.s__Streptococcus_gallolyticus	+	3.68E-01	0.000	0.000	0.000	0.000	0.000
	3.5.4.26|g__Streptococcus.s__Streptococcus_macedonicus	+	1.96E-01	0.000	0.000	0.000	0.000	0.000
	3.5.4.26|g__Streptococcus.s__Streptococcus_pasteurianus	+	1.96E-01	0.000	0.000	0.000	0.000	0.000
	3.5.4.26|g__Streptococcus.s__Streptococcus_pneumoniae	+	3.68E-01	0.000	0.000	0.000	0.000	0.000
	3.5.4.26|g__Succinatimonas.s__Succinatimonas_hippei	+	4.32E-01	0.000	0.000	0.000	0.000	0.000
	3.5.4.26|g__Sutterella.s__Sutterella_wadsworthensis	+	6.29E-01	0.000	0.000	0.000	0.010	0.009
	3.5.4.26|g__Turicimonas.s__Turicimonas_muris	+	6.54E-01	0.000	0.000	0.000	0.000	0.000
	3.5.4.26|g__Veillonella.s__Veillonella_atypica	-	2.97E-01	0.000	0.000	0.000	0.000	0.000
	3.5.4.26|g__Veillonella.s__Veillonella_dispar	-	7.54E-01	0.000	0.000	0.000	0.000	0.000
	3.5.4.26|g__Veillonella.s__Veillonella_infantium	-	8.10E-01	0.000	0.000	0.000	0.000	0.000
	3.5.4.26|g__Veillonella.s__Veillonella_parvula	-	2.05E-01	0.000	0.000	0.000	0.001	0.000
	3.5.4.26|g__Veillonella.s__Veillonella_rogosae	-	2.88E-01	0.000	0.000	0.000	0.000	0.000
	3.5.4.26|g__Veillonella.s__Veillonella_tobetsuensis	-	8.72E-01	0.000	0.000	0.000	0.000	0.000
	3.5.4.26|g__Veillonellaceae_unclassified.s__Veillonellaceae_bacterium	+	3.68E-01	0.000	0.000	0.000	0.000	0.000
	3.5.4.26|g__Victivallales_unclassified.s__Victivallales_bacterium_CCUG_4	+	4.53E-01	0.000	0.000	0.000	0.001	0.002
	3.5.4.26|g__Victivallis.s__Victivallis_vadensis	+	1.96E-02	0.000	0.000	0.000	0.000	0.001
	3.5.4.26|g__Yokenella.s__Yokenella_regensburgei	-	2.42E-01	0.000	0.000	0.000	0.000	0.000
	3.5.4.26|g__Blautia.s__Blautia_obeum	-	6.89E-01	0.210	0.221	0.012	0.003	0.004
	3.5.4.26|g__Bilophila.s__Bilophila_wadsworthia	-	5.31E-01	0.156	0.187	0.032	0.004	0.003
	3.5.4.26|g__Blautia.s__Ruminococcus_torques	+	7.92E-01	0.266	0.318	0.052	0.008	0.007
	3.5.4.26|g__Bacteroides.s__Bacteroides_massiliensis	-	9.19E-01	0.291	0.358	0.067	0.023	0.023
	3.5.4.26|g__Bacteroides.s__Bacteroides_ovatus	+	3.18E-01	0.514	0.594	0.080	0.013	0.016
	3.5.4.26|g__Roseburia.s__Roseburia_hominis	+	3.66E-01	0.100	0.189	0.089	0.002	0.004
	3.5.4.26|g__Bacteroides.s__Bacteroides_dorei	+	3.87E-01	0.089	0.192	0.103	0.019	0.031
	3.5.4.26|g__Butyricimonas.s__Butyricimonas_virosa	+	3.01E-02	0.000	0.124	0.124	0.002	0.004
	3.5.4.26|g__Faecalibacterium.s__Faecalibacterium_prausnitzii	+	6.61E-01	3.165	3.337	0.171	0.043	0.043
	3.5.4.26|g__Eubacterium.s__Eubacterium_eligens	+	5.78E-01	0.227	0.421	0.194	0.008	0.007
	3.5.4.26|g__Bacteroides.s__Bacteroides_uniformis	+	7.76E-01	3.531	3.742	0.211	0.065	0.068
	3.5.4.26|g__Bacteroides.s__Bacteroides_stercoris	-	8.48E-01	1.718	1.939	0.222	0.055	0.046
	3.5.4.26|g__Bacteroides.s__Bacteroides_cellulosilyticus	+	9.88E-03	0.000	0.317	0.317	0.009	0.008
	3.5.4.26|g__Phascolarctobacterium.s__Phascolarctobacterium_faecium	+	9.41E-01	0.263	0.634	0.371	0.010	0.010
	3.5.4.26|g__Odoribacter.s__Odoribacter_splanchnicus	+	3.29E-02	0.324	0.696	0.372	0.006	0.008
	3.5.4.26|g__Bacteroides.s__Bacteroides_thetaiotaomicron	+	5.61E-02	0.721	1.239	0.518	0.013	0.023
	3.5.4.26|g__Bacteroides.s__Bacteroides_intestinalis	+	1.39E-02	0.000	0.599	0.599	0.006	0.015
	3.5.4.26|g__Parabacteroides.s__Parabacteroides_merdae	+	2.29E-02	0.398	1.057	0.659	0.008	0.017
	3.5.4.26|g__Parabacteroides.s__Parabacteroides_distasonis	+	3.32E-02	0.848	1.509	0.660	0.014	0.022
	3.5.4.26|g__Bacteroides.s__Bacteroides_caccae	+	3.59E-02	0.364	1.264	0.900	0.012	0.018

				Fractional CPM (median)			Relative CPM (average)	
Country	EC number|Bacteria	Increased or decreased in PD	*p* -value	Control	PD	PD - Control	Control	PD
Japan	1.1.1.193	-	3.12E-04	95.212	82.865	-12.347	1.000	1.000
	1.1.1.193|g__Faecalibacterium.s__Faecalibacterium_prausnitzii	-	1.61E-02	9.122	4.960	-4.162	0.095	0.080
	1.1.1.193|unclassified	-	1.23E-02	10.626	8.239	-2.387	0.127	0.119
	1.1.1.193|g__Blautia.s__Blautia_wexlerae	-	2.63E-04	3.337	1.600	-1.737	0.044	0.029
	1.1.1.193|g__Dorea.s__Dorea_longicatena	-	5.36E-02	1.281	0.113	-1.168	0.016	0.011
	1.1.1.193|g__Blautia.s__Blautia_obeum	-	2.76E-03	2.818	1.907	-0.911	0.035	0.028
	1.1.1.193|g__Lachnospiraceae_unclassified.s__Eubacterium_rectale	-	9.98E-02	0.813	0.000	-0.813	0.034	0.028
	1.1.1.193|g__Blautia.s__Ruminococcus_torques	-	4.47E-02	2.331	1.649	-0.682	0.035	0.026
	1.1.1.193|g__Bacteroides.s__Bacteroides_uniformis	+	7.24E-01	4.368	3.934	-0.433	0.058	0.076
	1.1.1.193|g__Agathobaculum.s__Agathobaculum_butyriciproducens	-	3.54E-02	0.433	0.000	-0.433	0.005	0.004
	1.1.1.193|g__Roseburia.s__Roseburia_hominis	-	1.91E-01	1.402	1.050	-0.352	0.019	0.017
	1.1.1.193|g__Bacteroides.s__Bacteroides_vulgatus	-	6.03E-01	1.678	1.366	-0.312	0.055	0.046
	1.1.1.193|g__Anaerostipes.s__Anaerostipes_hadrus	-	2.06E-01	1.341	1.084	-0.257	0.029	0.025
	1.1.1.193|g__Bacteroides.s__Bacteroides_xylanisolvens	-	3.89E-01	0.064	0.000	-0.064	0.007	0.007
	1.1.1.193|g__Bacteroides.s__Bacteroides_ovatus	+	9.43E-01	0.379	0.358	-0.021	0.013	0.016
	1.1.1.193|g__Acidaminococcus.s__Acidaminococcus_fermentans	+	3.85E-01	0.000	0.000	0.000	0.000	0.000
	1.1.1.193|g__Acidaminococcus.s__Acidaminococcus_intestini	+	9.82E-01	0.000	0.000	0.000	0.009	0.003
	1.1.1.193|g__Adlercreutzia.s__Adlercreutzia_equolifaciens	+	1.41E-01	0.000	0.000	0.000	0.000	0.001

1.1.1.193|g__Akkermansia.s__Akkermansia_muciniphila	+	8.28E-04	0.000	0.000	0.000	0.006	0.045
1.1.1.193|g__Alistipes.s__Alistipes_shahii	+	1.48E-02	0.000	0.000	0.000	0.004	0.012
1.1.1.193|g__Allisonella.s__Allisonella_histaminiformans	-	2.62E-01	0.000	0.000	0.000	0.000	0.000
1.1.1.193|g__Anaeroglobus.s__Anaeroglobus_geminatus	+	3.85E-01	0.000	0.000	0.000	0.000	0.000
1.1.1.193|g__Anaeromassilibacillus.s__Anaeromassilibacillus_sp_An250	-	8.57E-01	0.000	0.000	0.000	0.000	0.000
1.1.1.193|g__Anaerostipes.s__Anaerostipes_caccae	-	1.76E-01	0.000	0.000	0.000	0.001	0.001
1.1.1.193|g__Anaerotignum.s__Anaerotignum_lactatifermentans	+	1.32E-02	0.000	0.000	0.000	0.000	0.002
1.1.1.193|g__Asaccharobacter.s__Asaccharobacter_celatus	+	4.20E-01	0.000	0.000	0.000	0.001	0.001
1.1.1.193|g__Bacillus.s__Bacillus_gibsonii	+	5.06E-02	0.000	0.000	0.000	0.000	0.001
1.1.1.193|g__Bacillus.s__Bacillus_murimartini	+	2.93E-01	0.000	0.000	0.000	0.000	0.000
1.1.1.193|g__Bacteroides.s__Bacteroides_caccae	+	1.76E-01	0.000	0.000	0.000	0.004	0.008
1.1.1.193|g__Bacteroides.s__Bacteroides_cellulosilyticus	+	9.01E-02	0.000	0.000	0.000	0.004	0.007
1.1.1.193|g__Bacteroides.s__Bacteroides_clarus	+	2.56E-01	0.000	0.000	0.000	0.000	0.005
1.1.1.193|g__Bacteroides.s__Bacteroides_coprocola	+	3.83E-01	0.000	0.000	0.000	0.012	0.014
1.1.1.193|g__Bacteroides.s__Bacteroides_coprophilus	+	4.41E-01	0.000	0.000	0.000	0.001	0.001
1.1.1.193|g__Bacteroides.s__Bacteroides_eggerthii	+	2.30E-01	0.000	0.000	0.000	0.004	0.012
1.1.1.193|g__Bacteroides.s__Bacteroides_faecis	-	8.03E-01	0.000	0.000	0.000	0.000	0.000
1.1.1.193|g__Bacteroides.s__Bacteroides_finegoldii	+	7.57E-01	0.000	0.000	0.000	0.003	0.003
1.1.1.193|g__Bacteroides.s__Bacteroides_fluxus	-	2.62E-01	0.000	0.000	0.000	0.000	0.000
1.1.1.193|g__Bacteroides.s__Bacteroides_fragilis	-	8.41E-01	0.000	0.000	0.000	0.005	0.008
1.1.1.193|g__Bacteroides.s__Bacteroides_intestinalis	+	6.96E-02	0.000	0.000	0.000	0.003	0.005
1.1.1.193|g__Bacteroides.s__Bacteroides_massiliensis	+	2.71E-01	0.000	0.000	0.000	0.002	0.004
1.1.1.193|g__Bacteroides.s__Bacteroides_nordii	+	2.88E-02	0.000	0.000	0.000	0.000	0.001
1.1.1.193|g__Bacteroides.s__Bacteroides_oleiciplenus	+	3.85E-01	0.000	0.000	0.000	0.000	0.000
1.1.1.193|g__Bacteroides.s__Bacteroides_plebeius	+	1.79E-01	0.000	0.000	0.000	0.027	0.022
1.1.1.193|g__Bacteroides.s__Bacteroides_salyersiae	+	1.05E-01	0.000	0.000	0.000	0.000	0.001
1.1.1.193|g__Bacteroides.s__Bacteroides_stercoris	-	8.09E-01	0.000	0.000	0.000	0.034	0.026
1.1.1.193|g__Barnesiella.s__Barnesiella_intestinihominis	+	1.11E-03	0.000	0.000	0.000	0.003	0.010
1.1.1.193|g__Barnesiella.s__Barnesiella_sp_An22	-	2.62E-01	0.000	0.000	0.000	0.000	0.000
1.1.1.193|g__Bifidobacterium.s__Bifidobacterium_longum	-	8.57E-01	0.000	0.000	0.000	0.000	0.000
1.1.1.193|g__Bilophila.s__Bilophila_wadsworthia	+	2.16E-02	0.000	0.000	0.000	0.002	0.004
1.1.1.193|g__Blautia.s__Blautia_hansenii	-	9.89E-02	0.000	0.000	0.000	0.001	0.000
1.1.1.193|g__Blautia.s__Blautia_sp_AF19_10LB	+	9.05E-01	0.000	0.000	0.000	0.004	0.005
1.1.1.193|g__Blautia.s__Blautia_sp_An249	+	1.26E-01	0.000	0.000	0.000	0.000	0.001
1.1.1.193|g__Butyricicoccus.s__Butyricicoccus_pullicaecorum	-	4.41E-01	0.000	0.000	0.000	0.001	0.000
1.1.1.193|g__Butyricimonas.s__Butyricimonas_virosa	+	2.83E-02	0.000	0.000	0.000	0.000	0.001
1.1.1.193|g__Butyrivibrio.s__Butyrivibrio_crossotus	+	3.85E-01	0.000	0.000	0.000	0.000	0.001
1.1.1.193|g__Catenibacterium.s__Catenibacterium_mitsuokai	-	8.17E-03	0.000	0.000	0.000	0.002	0.000
1.1.1.193|g__Cellulosilyticum.s__Cellulosilyticum_lentocellum	+	3.85E-01	0.000	0.000	0.000	0.000	0.000
1.1.1.193|g__Cetobacterium.s__Cetobacterium_somerae	-	2.62E-01	0.000	0.000	0.000	0.000	0.000
1.1.1.193|g__Citrobacter.s__Citrobacter_braakii	+	4.46E-01	0.000	0.000	0.000	0.000	0.001
1.1.1.193|g__Citrobacter.s__Citrobacter_freundii	+	1.72E-01	0.000	0.000	0.000	0.000	0.000
1.1.1.193|g__Citrobacter.s__Citrobacter_koseri	-	8.71E-01	0.000	0.000	0.000	0.000	0.000
1.1.1.193|g__Citrobacter.s__Citrobacter_portucalensis	+	7.63E-02	0.000	0.000	0.000	0.000	0.000
1.1.1.193|g__Citrobacter.s__Citrobacter_werkmanii	+	3.85E-01	0.000	0.000	0.000	0.000	0.002
1.1.1.193|g__Citrobacter.s__Citrobacter_youngae	+	2.72E-01	0.000	0.000	0.000	0.000	0.001
1.1.1.193|g__Cloacibacillus.s__Cloacibacillus_porcorum	+	2.14E-01	0.000	0.000	0.000	0.000	0.001
1.1.1.193|g__Clostridioides.s__Clostridioides_difficile	+	7.85E-01	0.000	0.000	0.000	0.004	0.004
1.1.1.193|g__Clostridium.s__Butyribacterium_methylotrophicum	+	5.79E-01	0.000	0.000	0.000	0.000	0.000
1.1.1.193|g__Clostridium.s__Clostridium_celatum	+	1.00E+00	0.000	0.000	0.000	0.000	0.000
1.1.1.193|g__Clostridium.s__Clostridium_disporicum	+	9.44E-01	0.000	0.000	0.000	0.002	0.001
1.1.1.193|g__Clostridium.s__Clostridium_perfringens	-	2.52E-01	0.000	0.000	0.000	0.001	0.000
1.1.1.193|g__Clostridium.s__Clostridium_sp_AF36_4	+	5.84E-01	0.000	0.000	0.000	0.001	0.002
1.1.1.193|g__Clostridium.s__Clostridium_sp_AM22_11AC	-	2.32E-01	0.000	0.000	0.000	0.003	0.002
1.1.1.193|g__Coprobacillus.s__Coprobacillus_cateniformis	+	3.84E-02	0.000	0.000	0.000	0.000	0.001
1.1.1.193|g__Coprobacter.s__Coprobacter_fastidiosus	+	8.23E-01	0.000	0.000	0.000	0.002	0.002
1.1.1.193|g__Coprobacter.s__Coprobacter_secundus	+	9.67E-01	0.000	0.000	0.000	0.000	0.001
1.1.1.193|g__Coprococcus.s__Coprococcus_catus	+	9.02E-01	0.000	0.000	0.000	0.001	0.002
1.1.1.193|g__Coprococcus.s__Coprococcus_comes	-	4.45E-02	0.000	0.000	0.000	0.006	0.003
1.1.1.193|g__Coprococcus.s__Coprococcus_eutactus	-	2.86E-01	0.000	0.000	0.000	0.003	0.002
1.1.1.193|g__Desulfovibrio.s__Desulfovibrio_desulfuricans	+	1.78E-02	0.000	0.000	0.000	0.000	0.001
1.1.1.193|g__Desulfovibrio.s__Desulfovibrio_fairfieldensis	+	1.26E-01	0.000	0.000	0.000	0.000	0.000
1.1.1.193|g__Desulfovibrio.s__Desulfovibrio_piger	+	6.20E-01	0.000	0.000	0.000	0.000	0.000
1.1.1.193|g__Desulfovibrio.s__Desulfovibrio_sp_AM18_2	+	3.85E-01	0.000	0.000	0.000	0.000	0.000
1.1.1.193|g__Desulfovibrionaceae_unclassified.s__Desulfovibrionaceae_bac	-	2.62E-01	0.000	0.000	0.000	0.000	0.000
1.1.1.193|g__Dialister.s__Dialister_succinatiphilus	+	7.10E-01	0.000	0.000	0.000	0.000	0.001
1.1.1.193|g__Dorea.s__Dorea_formicigenerans	-	7.17E-01	0.000	0.000	0.000	0.005	0.005
1.1.1.193|g__Dorea.s__Dorea_sp_OM02_2LB	+	2.14E-01	0.000	0.000	0.000	0.000	0.000
1.1.1.193|g__Eggerthella.s__Eggerthella_lenta	+	3.16E-01	0.000	0.000	0.000	0.005	0.004
1.1.1.193|g__Enterobacter.s__Enterobacter_bugandensis	-	2.62E-01	0.000	0.000	0.000	0.000	0.000
1.1.1.193|g__Enterobacter.s__Enterobacter_cloacae	+	1.15E-01	0.000	0.000	0.000	0.000	0.000
1.1.1.193|g__Enterobacter.s__Enterobacter_mori	+	1.26E-01	0.000	0.000	0.000	0.000	0.001

1.1.1.193|g__Enterococcus.s__Enterococcus_avium	+	4.14E-01	0.000	0.000	0.000	0.006	0.002
1.1.1.193|g__Enterococcus.s__Enterococcus_faecalis	-	2.62E-01	0.000	0.000	0.000	0.000	0.000
1.1.1.193|g__Enterococcus.s__Enterococcus_faecium	+	5.56E-01	0.000	0.000	0.000	0.005	0.002
1.1.1.193|g__Enterococcus.s__Enterococcus_gallinarum	+	3.85E-01	0.000	0.000	0.000	0.000	0.000
1.1.1.193|g__Enterococcus.s__Enterococcus_saccharolyticus	+	3.85E-01	0.000	0.000	0.000	0.000	0.000
1.1.1.193|g__Enterococcus.s__Enterococcus_thailandicus	+	3.85E-01	0.000	0.000	0.000	0.000	0.000
1.1.1.193|g__Erysipelatoclostridium.s__Clostridium_innocuum	+	6.98E-01	0.000	0.000	0.000	0.004	0.004
1.1.1.193|g__Erysipelatoclostridium.s__Clostridium_spiroforme	-	4.16E-01	0.000	0.000	0.000	0.003	0.001
1.1.1.193|g__Erysipelotrichaceae_unclassified.s__Erysipelotrichaceae_bacte	+	4.30E-02	0.000	0.000	0.000	0.000	0.001
1.1.1.193|g__Escherichia.s__Escherichia_coli	+	4.78E-02	0.000	0.000	0.000	0.004	0.005
1.1.1.193|g__Eubacterium.s__Eubacterium_callanderi	+	4.03E-01	0.000	0.000	0.000	0.000	0.000
1.1.1.193|g__Eubacterium.s__Eubacterium_eligens	+	3.79E-01	0.000	0.000	0.000	0.011	0.018
1.1.1.193|g__Eubacterium.s__Eubacterium_limosum	+	2.72E-02	0.000	0.000	0.000	0.000	0.001
1.1.1.193|g__Eubacterium.s__Eubacterium_maltosivorans	+	3.85E-01	0.000	0.000	0.000	0.000	0.000
1.1.1.193|g__Eubacterium.s__Eubacterium_ramulus	+	8.89E-01	0.000	0.000	0.000	0.002	0.002
1.1.1.193|g__Eubacterium.s__Eubacterium_sp_AF17_7	+	4.04E-02	0.000	0.000	0.000	0.001	0.003
1.1.1.193|g__Eubacterium.s__Eubacterium_sp_AM18_10LB_B	-	6.46E-01	0.000	0.000	0.000	0.001	0.001
1.1.1.193|g__Faecalicatena.s__Faecalicatena_contorta	+	4.39E-01	0.000	0.000	0.000	0.000	0.000
1.1.1.193|g__Firmicutes_unclassified.s__Firmicutes_bacterium_AM10_47	+	6.38E-01	0.000	0.000	0.000	0.002	0.002
1.1.1.193|g__Flavonifractor.s__Flavonifractor_plautii	+	1.85E-01	0.000	0.000	0.000	0.000	0.000
1.1.1.193|g__Fusobacterium.s__Fusobacterium_mortiferum	-	2.12E-01	0.000	0.000	0.000	0.000	0.000
1.1.1.193|g__Fusobacterium.s__Fusobacterium_ulcerans	+	6.13E-01	0.000	0.000	0.000	0.000	0.000
1.1.1.193|g__Geobacillus.s__Bacillus_caldolyticus	+	3.85E-01	0.000	0.000	0.000	0.000	0.000
1.1.1.193|g__Geobacillus.s__Geobacillus_stearothermophilus	+	3.85E-01	0.000	0.000	0.000	0.000	0.000
1.1.1.193|g__Geobacillus.s__Geobacillus_thermocatenulatus	+	3.85E-01	0.000	0.000	0.000	0.000	0.000
1.1.1.193|g__Geobacillus.s__Geobacillus_thermoleovorans	+	3.85E-01	0.000	0.000	0.000	0.000	0.000
1.1.1.193|g__Haemophilus.s__Haemophilus_parainfluenzae	-	4.43E-02	0.000	0.000	0.000	0.002	0.001
1.1.1.193|g__Holdemanella.s__Holdemanella_biformis	-	2.22E-01	0.000	0.000	0.000	0.001	0.003
1.1.1.193|g__Hungatella.s__Hungatella_hathewayi	+	4.12E-01	0.000	0.000	0.000	0.001	0.002
1.1.1.193|g__Intestinibacter.s__Intestinibacter_bartlettii	-	1.29E-01	0.000	0.000	0.000	0.003	0.002
1.1.1.193|g__Klebsiella.s__Klebsiella_aerogenes	-	8.57E-01	0.000	0.000	0.000	0.000	0.000
1.1.1.193|g__Klebsiella.s__Klebsiella_grimontii	-	1.09E-01	0.000	0.000	0.000	0.000	0.000
1.1.1.193|g__Klebsiella.s__Klebsiella_michiganensis	-	8.71E-01	0.000	0.000	0.000	0.000	0.000
1.1.1.193|g__Klebsiella.s__Klebsiella_oxytoca	-	5.30E-01	0.000	0.000	0.000	0.004	0.002
1.1.1.193|g__Klebsiella.s__Klebsiella_pneumoniae	-	9.02E-01	0.000	0.000	0.000	0.007	0.006
1.1.1.193|g__Klebsiella.s__Klebsiella_variicola	-	6.52E-01	0.000	0.000	0.000	0.001	0.000
1.1.1.193|g__Lachnoclostridium.s__Clostridium_aldenense	+	2.86E-01	0.000	0.000	0.000	0.000	0.000
1.1.1.193|g__Lachnoclostridium.s__Clostridium_bolteae	+	3.68E-01	0.000	0.000	0.000	0.001	0.002
1.1.1.193|g__Lachnoclostridium.s__Clostridium_citroniae	+	3.85E-01	0.000	0.000	0.000	0.000	0.000
1.1.1.193|g__Lachnoclostridium.s__Clostridium_clostridioforme	-	7.77E-01	0.000	0.000	0.000	0.000	0.000
1.1.1.193|g__Lachnoclostridium.s__Clostridium_symbiosum	+	9.96E-01	0.000	0.000	0.000	0.000	0.000
1.1.1.193|g__Lachnospira.s__Lachnospira_pectinoschiza	-	2.27E-01	0.000	0.000	0.000	0.003	0.002
1.1.1.193|g__Lactobacillus.s__Lactobacillus_amylovorus	-	8.57E-01	0.000	0.000	0.000	0.000	0.000
1.1.1.193|g__Lactobacillus.s__Lactobacillus_antri	+	2.14E-01	0.000	0.000	0.000	0.000	0.000
1.1.1.193|g__Lactobacillus.s__Lactobacillus_crispatus	+	6.84E-03	0.000	0.000	0.000	0.000	0.002
1.1.1.193|g__Lactobacillus.s__Lactobacillus_fermentum	+	6.51E-02	0.000	0.000	0.000	0.000	0.004
1.1.1.193|g__Lactobacillus.s__Lactobacillus_kimbladii	+	3.85E-01	0.000	0.000	0.000	0.000	0.000
1.1.1.193|g__Lactobacillus.s__Lactobacillus_kullabergensis	+	3.85E-01	0.000	0.000	0.000	0.000	0.000
1.1.1.193|g__Lactobacillus.s__Lactobacillus_melliventris	-	2.62E-01	0.000	0.000	0.000	0.000	0.000
1.1.1.193|g__Lactobacillus.s__Lactobacillus_oris	+	3.98E-01	0.000	0.000	0.000	0.000	0.003
1.1.1.193|g__Lactobacillus.s__Lactobacillus_plantarum	+	3.85E-01	0.000	0.000	0.000	0.000	0.000
1.1.1.193|g__Lactobacillus.s__Lactobacillus_reuteri	+	3.85E-01	0.000	0.000	0.000	0.000	0.000
1.1.1.193|g__Lactobacillus.s__Lactobacillus_rogosae	-	2.87E-01	0.000	0.000	0.000	0.003	0.003
1.1.1.193|g__Lactococcus.s__Lactococcus_lactis	-	4.16E-01	0.000	0.000	0.000	0.000	0.000
1.1.1.193|g__Leclercia.s__Leclercia_adecarboxylata	-	1.09E-01	0.000	0.000	0.000	0.000	0.000
1.1.1.193|g__Lelliottia.s__Lelliottia_nimipressuralis	-	2.03E-01	0.000	0.000	0.000	0.000	0.000
1.1.1.193|g__Leuconostoc.s__Leuconostoc_garlicum	+	2.14E-01	0.000	0.000	0.000	0.000	0.000
1.1.1.193|g__Leuconostoc.s__Leuconostoc_lactis	+	2.14E-01	0.000	0.000	0.000	0.000	0.000
1.1.1.193|g__Listeria.s__Listeria_monocytogenes	-	2.62E-01	0.000	0.000	0.000	0.000	0.000
1.1.1.193|g__Megamonas.s__Megamonas_funiformis	-	9.19E-01	0.000	0.000	0.000	0.005	0.004
1.1.1.193|g__Megamonas.s__Megamonas_hypermegale	-	7.64E-01	0.000	0.000	0.000	0.006	0.002
1.1.1.193|g__Megamonas.s__Megamonas_rupellensis	-	9.51E-01	0.000	0.000	0.000	0.006	0.003
1.1.1.193|g__Megasphaera.s__Megasphaera_elsdenii	-	8.57E-01	0.000	0.000	0.000	0.000	0.000
1.1.1.193|g__Megasphaera.s__Megasphaera_hexanoica	+	3.85E-01	0.000	0.000	0.000	0.000	0.000
1.1.1.193|g__Megasphaera.s__Megasphaera_stantonii	-	8.12E-01	0.000	0.000	0.000	0.000	0.000
1.1.1.193|g__Mesosutterella.s__Mesosutterella_multiformis	-	1.54E-01	0.000	0.000	0.000	0.002	0.000
1.1.1.193|g__Metakosakonia.s__Kluyvera_intestini	+	3.85E-01	0.000	0.000	0.000	0.000	0.000
1.1.1.193|g__Mitsuokella.s__Mitsuokella_jalaludinii	+	3.85E-01	0.000	0.000	0.000	0.000	0.000
1.1.1.193|g__Mitsuokella.s__Mitsuokella_multacida	+	3.85E-01	0.000	0.000	0.000	0.000	0.000
1.1.1.193|g__Odoribacter.s__Odoribacter_laneus	+	7.63E-01	0.000	0.000	0.000	0.000	0.001
1.1.1.193|g__Odoribacter.s__Odoribacter_splanchnicus	+	5.23E-02	0.000	0.000	0.000	0.002	0.003
1.1.1.193|g__Pantoea.s__Pantoea_sesami	-	8.03E-01	0.000	0.000	0.000	0.000	0.000

	1.1.1.193|g__Parabacteroides.s__Parabacteroides_goldsteinii	+	4.81E-01	0.000	0.000	0.000	0.000	0.001
	1.1.1.193|g__Parabacteroides.s__Parabacteroides_johnsonii	-	6.63E-01	0.000	0.000	0.000	0.001	0.001
	1.1.1.193|g__Paraprevotella.s__Paraprevotella_clara	+	9.26E-01	0.000	0.000	0.000	0.002	0.002
	1.1.1.193|g__Paraprevotella.s__Paraprevotella_xylaniphila	+	4.46E-01	0.000	0.000	0.000	0.000	0.000
	1.1.1.193|g__Parasutterella.s__Parasutterella_excrementihominis	-	6.69E-01	0.000	0.000	0.000	0.001	0.001
	1.1.1.193|g__Pediococcus.s__Pediococcus_acidilactici	+	4.61E-01	0.000	0.000	0.000	0.000	0.000
	1.1.1.193|g__Peptostreptococcaceae_unclassified.s__Clostridium_hiranonis	-	2.62E-01	0.000	0.000	0.000	0.000	0.000
	1.1.1.193|g__Phascolarctobacterium.s__Phascolarctobacterium_faecium	+	2.70E-01	0.000	0.000	0.000	0.005	0.010
	1.1.1.193|g__Phascolarctobacterium.s__Phascolarctobacterium_succinatuter	-	4.86E-01	0.000	0.000	0.000	0.005	0.004
	1.1.1.193|g__Prevotella.s__Prevotella_buccae	-	1.09E-01	0.000	0.000	0.000	0.002	0.000
	1.1.1.193|g__Prevotella.s__Prevotella_copri	+	9.14E-01	0.000	0.000	0.000	0.044	0.035
	1.1.1.193|g__Prevotella.s__Prevotella_sp_109	-	2.03E-01	0.000	0.000	0.000	0.003	0.001
	1.1.1.193|g__Prevotella.s__Prevotella_sp_AM42_24	-	1.99E-01	0.000	0.000	0.000	0.003	0.000
	1.1.1.193|g__Pseudoflavonifractor.s__Pseudoflavonifractor_sp_An184	+	1.26E-01	0.000	0.000	0.000	0.000	0.001
	1.1.1.193|g__Pyramidobacter.s__Pyramidobacter_piscolens	+	2.14E-01	0.000	0.000	0.000	0.000	0.000
	1.1.1.193|g__Raoultella.s__Raoultella_ornithinolytica	-	4.16E-01	0.000	0.000	0.000	0.004	0.000
	1.1.1.193|g__Raoultella.s__Raoultella_planticola	-	1.09E-01	0.000	0.000	0.000	0.000	0.000
	1.1.1.193|g__Rikenella.s__Rikenella_microfusus	+	1.26E-01	0.000	0.000	0.000	0.000	0.001
	1.1.1.193|g__Roseburia.s__Roseburia_faecis	+	5.00E-01	0.000	0.000	0.000	0.002	0.002
	1.1.1.193|g__Roseburia.s__Roseburia_intestinalis	-	4.89E-05	0.000	0.000	0.000	0.003	0.000
	1.1.1.193|g__Roseburia.s__Roseburia_inulinivorans	-	6.59E-01	0.000	0.000	0.000	0.003	0.001
	1.1.1.193|g__Ruminococcaceae_unclassified.s__Ruminococcaceae_bacteriu	+	1.82E-01	0.000	0.000	0.000	0.000	0.000
	1.1.1.193|g__Ruminococcaceae_unclassified.s__Ruminococcaceae_bacteriu	-	7.53E-01	0.000	0.000	0.000	0.000	0.000
	1.1.1.193|g__Ruminococcus.s__Ruminococcus_callidus	-	9.99E-02	0.000	0.000	0.000	0.005	0.002
	1.1.1.193|g__Ruminococcus.s__Ruminococcus_sp_AF31_8BH	+	1.10E-01	0.000	0.000	0.000	0.001	0.002
	1.1.1.193|g__Salmonella.s__Salmonella_enterica	+	2.14E-01	0.000	0.000	0.000	0.000	0.000
	1.1.1.193|g__Slackia.s__Slackia_isoflavoniconvertens	-	9.98E-01	0.000	0.000	0.000	0.001	0.002
	1.1.1.193|g__Streptococcus.s__Streptococcus_equinus	-	2.89E-01	0.000	0.000	0.000	0.003	0.001
	1.1.1.193|g__Streptococcus.s__Streptococcus_gallolyticus	-	4.32E-01	0.000	0.000	0.000	0.000	0.000
	1.1.1.193|g__Streptococcus.s__Streptococcus_infantarius	-	1.09E-01	0.000	0.000	0.000	0.003	0.000
	1.1.1.193|g__Streptococcus.s__Streptococcus_lutetiensis	-	1.09E-01	0.000	0.000	0.000	0.001	0.000
	1.1.1.193|g__Streptococcus.s__Streptococcus_macedonicus	-	7.53E-01	0.000	0.000	0.000	0.000	0.000
	1.1.1.193|g__Streptococcus.s__Streptococcus_pasteurianus	-	7.30E-01	0.000	0.000	0.000	0.000	0.000
	1.1.1.193|g__Streptococcus.s__Streptococcus_pneumoniae	+	6.84E-02	0.000	0.000	0.000	0.000	0.001
	1.1.1.193|g__Succinatimonas.s__Succinatimonas_hippei	+	3.85E-01	0.000	0.000	0.000	0.000	0.000
	1.1.1.193|g__Sutterella.s__Sutterella_wadsworthensis	-	5.64E-01	0.000	0.000	0.000	0.002	0.002
	1.1.1.193|g__Terrisporobacter.s__Terrisporobacter_othiniensis	+	3.85E-01	0.000	0.000	0.000	0.000	0.000
	1.1.1.193|g__Veillonella.s__Veillonella_atypica	-	2.30E-04	0.000	0.000	0.000	0.001	0.000
	1.1.1.193|g__Veillonella.s__Veillonella_denticariosi	+	3.85E-01	0.000	0.000	0.000	0.000	0.000
	1.1.1.193|g__Veillonella.s__Veillonella_dispar	-	2.59E-02	0.000	0.000	0.000	0.001	0.000
	1.1.1.193|g__Veillonella.s__Veillonella_infantium	-	1.33E-01	0.000	0.000	0.000	0.000	0.000
	1.1.1.193|g__Veillonella.s__Veillonella_parvula	-	1.09E-01	0.000	0.000	0.000	0.001	0.001
	1.1.1.193|g__Veillonella.s__Veillonella_rogosae	-	2.27E-03	0.000	0.000	0.000	0.000	0.000
	1.1.1.193|g__Veillonella.s__Veillonella_tobetsuensis	-	2.62E-01	0.000	0.000	0.000	0.000	0.000
	1.1.1.193|g__Victivallales_unclassified.s__Victivallales_bacterium_CCUG_	+	7.31E-01	0.000	0.000	0.000	0.000	0.000
	1.1.1.193|g__Victivallis.s__Victivallis_vadensis	+	3.85E-01	0.000	0.000	0.000	0.000	0.000
	1.1.1.193|g__Weissella.s__Weissella_cibaria	-	8.71E-01	0.000	0.000	0.000	0.000	0.000
	1.1.1.193|g__Weissella.s__Weissella_confusa	+	3.85E-01	0.000	0.000	0.000	0.000	0.000
	1.1.1.193|g__Yokenella.s__Yokenella_regensburgei	+	3.85E-01	0.000	0.000	0.000	0.000	0.000
	1.1.1.193|g__Bacteroides.s__Bacteroides_thetaiotaomicron	+	2.25E-01	0.387	0.615	0.228	0.008	0.014
	1.1.1.193|g__Parabacteroides.s__Parabacteroides_merdae	+	3.26E-01	0.345	0.676	0.331	0.010	0.012
	1.1.1.193|g__Parabacteroides.s__Parabacteroides_distasonis	+	1.75E-02	0.510	0.968	0.458	0.011	0.018
	1.1.1.193|g__Bacteroides.s__Bacteroides_dorei	+	6.85E-01	0.458	1.055	0.597	0.039	0.037
USA	1.1.1.193	-	2.74E-07	99.638	90.131	-9.507	1.000	1.000
	1.1.1.193|g__Faecalibacterium.s__Faecalibacterium_prausnitzii	-	1.19E-05	7.285	2.631	-4.654	0.081	0.063
	1.1.1.193|g__Bacteroides.s__Bacteroides_vulgatus	-	6.65E-02	6.085	4.356	-1.729	0.077	0.072
	1.1.1.193|g__Bacteroides.s__Bacteroides_uniformis	-	7.90E-01	5.696	4.692	-1.004	0.073	0.080
	1.1.1.193|g__Lachnospiraceae_unclassified.s__Eubacterium_rectale	-	2.82E-05	1.302	0.349	-0.953	0.032	0.022
	1.1.1.193|g__Blautia.s__Ruminococcus_torques	-	1.23E-05	2.754	1.839	-0.915	0.035	0.028
	1.1.1.193|g__Blautia.s__Blautia_wexlerae	-	6.23E-08	2.294	1.419	-0.875	0.037	0.026
	1.1.1.193|unclassified	-	3.98E-01	8.560	7.956	-0.605	0.105	0.112
	1.1.1.193|g__Blautia.s__Blautia_obeum	-	4.02E-04	3.245	2.662	-0.582	0.043	0.037
	1.1.1.193|g__Dorea.s__Dorea_longicatena	-	3.55E-02	0.566	0.000	-0.566	0.013	0.011
	1.1.1.193|g__Anaerostipes.s__Anaerostipes_hadrus	-	8.05E-04	0.491	0.237	-0.254	0.009	0.008
	1.1.1.193|g__Roseburia.s__Roseburia_hominis	-	2.82E-01	1.503	1.273	-0.231	0.018	0.018
	1.1.1.193|g__Bacteroides.s__Bacteroides_ovatus	-	7.35E-01	0.616	0.571	-0.045	0.022	0.020
	1.1.1.193|g__Lachnoclostridium.s__Clostridium_bolteae	-	4.79E-01	0.044	0.000	-0.044	0.003	0.004
	1.1.1.193|g__Asaccharobacter.s__Asaccharobacter_celatus	-	5.76E-01	0.070	0.051	-0.019	0.004	0.004
	1.1.1.193|g__Acidaminococcus.s__Acidaminococcus_fermentans	+	2.32E-01	0.000	0.000	0.000	0.000	0.000
	1.1.1.193|g__Acidaminococcus.s__Acidaminococcus_intestini	+	1.74E-03	0.000	0.000	0.000	0.007	0.013
	1.1.1.193|g__Acidipropionibacterium.s__Acidipropionibacterium_acidiprop	+	4.92E-01	0.000	0.000	0.000	0.000	0.000
	1.1.1.193|g__Actinomyces.s__Actinomyces_naeslundii	+	6.68E-02	0.000	0.000	0.000	0.000	0.000

1.1.1.193|g__Actinomyces.s__Actinomyces_oris	+	2.38E-02	0.000	0.000	0.000	0.000	0.000
1.1.1.193|g__Actinomyces.s__Actinomyces_sp_oral_taxon_448	+	4.92E-01	0.000	0.000	0.000	0.000	0.000
1.1.1.193|g__Actinomyces.s__Actinomyces_viscosus	+	3.09E-01	0.000	0.000	0.000	0.000	0.000
1.1.1.193|g__Adlercreutzia.s__Adlercreutzia_equolifaciens	-	4.59E-01	0.000	0.000	0.000	0.001	0.001
1.1.1.193|g__Aeromonas.s__Aeromonas_allosaccharophila	-	1.48E-01	0.000	0.000	0.000	0.000	0.000
1.1.1.193|g__Aeromonas.s__Aeromonas_diversa	-	1.48E-01	0.000	0.000	0.000	0.000	0.000
1.1.1.193|g__Aeromonas.s__Aeromonas_sobria	-	1.48E-01	0.000	0.000	0.000	0.000	0.000
1.1.1.193|g__Aeromonas.s__Aeromonas_veronii	-	1.48E-01	0.000	0.000	0.000	0.000	0.000
1.1.1.193|g__Agathobaculum.s__Agathobaculum_butyriciproducens	-	4.82E-03	0.000	0.000	0.000	0.003	0.002
1.1.1.193|g__Akkermansia.s__Akkermansia_muciniphila	+	1.77E-01	0.000	0.000	0.000	0.023	0.042
1.1.1.193|g__Allisonella.s__Allisonella_histaminiformans	-	6.68E-01	0.000	0.000	0.000	0.000	0.000
1.1.1.193|g__Anaerococcus.s__Anaerococcus_hydrogenalis	+	4.92E-01	0.000	0.000	0.000	0.000	0.000
1.1.1.193|g__Anaerococcus.s__Anaerococcus_octavius	+	4.92E-01	0.000	0.000	0.000	0.000	0.000
1.1.1.193|g__Anaerococcus.s__Anaerococcus_vaginalis	+	2.32E-01	0.000	0.000	0.000	0.000	0.000
1.1.1.193|g__Anaeroglobus.s__Anaeroglobus_geminatus	-	5.50E-01	0.000	0.000	0.000	0.000	0.000
1.1.1.193|g__Anaeromassilibacillus.s__Anaeromassilibacillus_sp_An250	-	4.19E-01	0.000	0.000	0.000	0.000	0.000
1.1.1.193|g__Anaerostipes.s__Anaerostipes_caccae	+	4.59E-01	0.000	0.000	0.000	0.001	0.001
1.1.1.193|g__Anaerostipes.s__Anaerostipes_sp_494a	-	1.48E-01	0.000	0.000	0.000	0.000	0.000
1.1.1.193|g__Anaerotignum.s__Anaerotignum_lactatifermentans	-	1.78E-01	0.000	0.000	0.000	0.002	0.002
1.1.1.193|g__Bacillus.s__Bacillus_horneckiae	-	1.48E-01	0.000	0.000	0.000	0.000	0.000
1.1.1.193|g__Bacteroidales_unclassified.s__Bacteroidales_bacterium_KA00	+	4.92E-01	0.000	0.000	0.000	0.000	0.000
1.1.1.193|g__Bacteroides.s__Bacteroides_clarus	+	8.36E-01	0.000	0.000	0.000	0.001	0.001
1.1.1.193|g__Bacteroides.s__Bacteroides_coprocola	-	1.03E-01	0.000	0.000	0.000	0.004	0.002
1.1.1.193|g__Bacteroides.s__Bacteroides_coprophilus	+	4.07E-01	0.000	0.000	0.000	0.000	0.000
1.1.1.193|g__Bacteroides.s__Bacteroides_eggerthii	+	6.53E-01	0.000	0.000	0.000	0.014	0.013
1.1.1.193|g__Bacteroides.s__Bacteroides_faecichinchillae	+	4.92E-01	0.000	0.000	0.000	0.000	0.000
1.1.1.193|g__Bacteroides.s__Bacteroides_faecis	+	1.40E-01	0.000	0.000	0.000	0.003	0.004
1.1.1.193|g__Bacteroides.s__Bacteroides_finegoldii	+	3.98E-01	0.000	0.000	0.000	0.002	0.003
1.1.1.193|g__Bacteroides.s__Bacteroides_fluxus	+	2.32E-01	0.000	0.000	0.000	0.000	0.000
1.1.1.193|g__Bacteroides.s__Bacteroides_fragilis	-	6.40E-01	0.000	0.000	0.000	0.007	0.008
1.1.1.193|g__Bacteroides.s__Bacteroides_massiliensis	+	4.99E-01	0.000	0.000	0.000	0.011	0.009
1.1.1.193|g__Bacteroides.s__Bacteroides_nordii	-	6.57E-01	0.000	0.000	0.000	0.001	0.001
1.1.1.193|g__Bacteroides.s__Bacteroides_plebeius	-	2.70E-01	0.000	0.000	0.000	0.014	0.005
1.1.1.193|g__Bacteroides.s__Bacteroides_salyersiae	+	3.36E-02	0.000	0.000	0.000	0.001	0.002
1.1.1.193|g__Bacteroides.s__Bacteroides_sartorii	-	1.48E-01	0.000	0.000	0.000	0.000	0.000
1.1.1.193|g__Bacteroides.s__Bacteroides_sp_OM08_11	+	4.92E-01	0.000	0.000	0.000	0.000	0.000
1.1.1.193|g__Bacteroides.s__Bacteroides_stercorirosoris	+	7.57E-01	0.000	0.000	0.000	0.000	0.000
1.1.1.193|g__Bacteroides.s__Bacteroides_xylanisolvens	+	2.05E-01	0.000	0.000	0.000	0.010	0.009
1.1.1.193|g__Barnesiella.s__Barnesiella_intestinihominis	+	7.67E-02	0.000	0.000	0.000	0.005	0.007
1.1.1.193|g__Bifidobacterium.s__Bifidobacterium_longum	+	2.32E-01	0.000	0.000	0.000	0.000	0.000
1.1.1.193|g__Blautia.s__Blautia_hansenii	-	8.56E-02	0.000	0.000	0.000	0.002	0.001
1.1.1.193|g__Blautia.s__Blautia_sp_AF19_10LB	+	5.57E-01	0.000	0.000	0.000	0.001	0.001
1.1.1.193|g__Blautia.s__Blautia_sp_An249	-	4.29E-01	0.000	0.000	0.000	0.000	0.000
1.1.1.193|g__Brevibacterium.s__Brevibacterium_ravenspurgense	+	4.92E-01	0.000	0.000	0.000	0.000	0.000
1.1.1.193|g__Butyricicoccus.s__Butyricicoccus_pullicaecorum	-	3.11E-02	0.000	0.000	0.000	0.000	0.000
1.1.1.193|g__Butyricimonas.s__Butyricimonas_synergistica	-	1.48E-01	0.000	0.000	0.000	0.000	0.000
1.1.1.193|g__Butyricimonas.s__Butyricimonas_virosa	+	1.14E-01	0.000	0.000	0.000	0.000	0.001
1.1.1.193|g__Butyrivibrio.s__Butyrivibrio_crossotus	-	6.16E-01	0.000	0.000	0.000	0.002	0.001
1.1.1.193|g__Campylobacter.s__Campylobacter_curvus	+	4.92E-01	0.000	0.000	0.000	0.000	0.000
1.1.1.193|g__Campylobacter.s__Campylobacter_gracilis	+	1.67E-01	0.000	0.000	0.000	0.000	0.000
1.1.1.193|g__Campylobacter.s__Campylobacter_ureolyticus	+	4.92E-01	0.000	0.000	0.000	0.000	0.000
1.1.1.193|g__Candidatus_Gastranaerophilales_unclassified.s__Candidatus_(	+	4.92E-01	0.000	0.000	0.000	0.000	0.000
1.1.1.193|g__Candidatus_Methanomethylophilus.s__Candidatus_Methanom	-	9.70E-01	0.000	0.000	0.000	0.000	0.000
1.1.1.193|g__Catenibacterium.s__Catenibacterium_mitsuokai	+	5.33E-01	0.000	0.000	0.000	0.002	0.004
1.1.1.193|g__Cellulosilyticum.s__Cellulosilyticum_lentocellum	+	4.92E-01	0.000	0.000	0.000	0.000	0.000
1.1.1.193|g__Cetobacterium.s__Cetobacterium_somerae	+	4.92E-01	0.000	0.000	0.000	0.000	0.000
1.1.1.193|g__Citrobacter.s__Citrobacter_amalonaticus	+	1.67E-01	0.000	0.000	0.000	0.000	0.000
1.1.1.193|g__Citrobacter.s__Citrobacter_braakii	-	9.67E-01	0.000	0.000	0.000	0.000	0.000
1.1.1.193|g__Citrobacter.s__Citrobacter_farmeri	+	1.67E-01	0.000	0.000	0.000	0.000	0.000
1.1.1.193|g__Citrobacter.s__Citrobacter_freundii	+	5.83E-01	0.000	0.000	0.000	0.001	0.000
1.1.1.193|g__Citrobacter.s__Citrobacter_koseri	+	3.30E-01	0.000	0.000	0.000	0.000	0.000
1.1.1.193|g__Citrobacter.s__Citrobacter_portucalensis	+	2.32E-01	0.000	0.000	0.000	0.000	0.000
1.1.1.193|g__Citrobacter.s__Citrobacter_werkmanii	+	4.92E-01	0.000	0.000	0.000	0.000	0.000
1.1.1.193|g__Citrobacter.s__Citrobacter_youngae	+	5.73E-02	0.000	0.000	0.000	0.000	0.000
1.1.1.193|g__Cloacibacillus.s__Cloacibacillus_porcorum	+	5.73E-02	0.000	0.000	0.000	0.000	0.001
1.1.1.193|g__Clostridiales_Family_XIII_Incertae_Sedis_unclassified.s__Eul	+	4.92E-01	0.000	0.000	0.000	0.000	0.000
1.1.1.193|g__Clostridiales_unclassified.s__Clostridiales_bacterium_1_7_47I	-	7.41E-01	0.000	0.000	0.000	0.000	0.000
1.1.1.193|g__Clostridioides.s__Clostridioides_difficile	+	5.40E-03	0.000	0.000	0.000	0.003	0.003
1.1.1.193|g__Clostridium.s__Butyribacterium_methylotrophicum	+	3.43E-05	0.000	0.000	0.000	0.000	0.001
1.1.1.193|g__Clostridium.s__Clostridium_butyricum	-	1.48E-01	0.000	0.000	0.000	0.000	0.000
1.1.1.193|g__Clostridium.s__Clostridium_cadaveris	-	5.95E-01	0.000	0.000	0.000	0.000	0.000
1.1.1.193|g__Clostridium.s__Clostridium_celatum	-	9.67E-01	0.000	0.000	0.000	0.000	0.000

1.1.1.193|g__Clostridium.s__Clostridium_disporicum	-	5.45E-01	0.000	0.000	0.000	0.000	0.000
1.1.1.193|g__Clostridium.s__Clostridium_perfringens	-	9.67E-01	0.000	0.000	0.000	0.000	0.000
1.1.1.193|g__Clostridium.s__Clostridium_sp_AF36_4	-	4.12E-01	0.000	0.000	0.000	0.003	0.003
1.1.1.193|g__Clostridium.s__Clostridium_sp_AM22_11AC	-	7.47E-03	0.000	0.000	0.000	0.003	0.002
1.1.1.193|g__Clostridium.s__Clostridium_sp_chh4_2	-	7.08E-01	0.000	0.000	0.000	0.000	0.000
1.1.1.193|g__Clostridium.s__Clostridium_sp_MSTE9	+	3.30E-01	0.000	0.000	0.000	0.000	0.000
1.1.1.193|g__Clostridium.s__Clostridium_ventriculi	+	7.60E-01	0.000	0.000	0.000	0.000	0.000
1.1.1.193|g__Comamonas.s__Comamonas_kerstersii	+	3.30E-01	0.000	0.000	0.000	0.000	0.000
1.1.1.193|g__Coprobacillus.s__Coprobacillus_cateniformis	+	1.92E-05	0.000	0.000	0.000	0.000	0.001
1.1.1.193|g__Coprobacter.s__Coprobacter_fastidiosus	-	9.56E-03	0.000	0.000	0.000	0.001	0.001
1.1.1.193|g__Coprobacter.s__Coprobacter_secundus	-	3.47E-01	0.000	0.000	0.000	0.000	0.000
1.1.1.193|g__Coprococcus.s__Coprococcus_catus	+	7.35E-01	0.000	0.000	0.000	0.002	0.002
1.1.1.193|g__Coprococcus.s__Coprococcus_comes	-	7.43E-01	0.000	0.000	0.000	0.005	0.006
1.1.1.193|g__Coprococcus.s__Coprococcus_eutactus	-	5.33E-01	0.000	0.000	0.000	0.003	0.002
1.1.1.193|g__Corynebacterium.s__Corynebacterium_amycolatum	+	4.92E-01	0.000	0.000	0.000	0.000	0.000
1.1.1.193|g__Corynebacterium.s__Corynebacterium_aurimucosum	+	4.92E-01	0.000	0.000	0.000	0.000	0.000
1.1.1.193|g__Corynebacterium.s__Corynebacterium_coyleae	+	4.92E-01	0.000	0.000	0.000	0.000	0.000
1.1.1.193|g__Corynebacterium.s__Corynebacterium_freneyi	+	4.92E-01	0.000	0.000	0.000	0.000	0.000
1.1.1.193|g__Corynebacterium.s__Corynebacterium_imitans	+	4.92E-01	0.000	0.000	0.000	0.000	0.000
1.1.1.193|g__Corynebacterium.s__Corynebacterium_jeikeium	+	3.30E-01	0.000	0.000	0.000	0.000	0.000
1.1.1.193|g__Corynebacterium.s__Corynebacterium_kroppenstedtii	+	4.92E-01	0.000	0.000	0.000	0.000	0.000
1.1.1.193|g__Corynebacterium.s__Corynebacterium_minutissimum	+	4.92E-01	0.000	0.000	0.000	0.000	0.000
1.1.1.193|g__Corynebacterium.s__Corynebacterium_pseudogenitalium	+	4.92E-01	0.000	0.000	0.000	0.000	0.000
1.1.1.193|g__Corynebacterium.s__Corynebacterium_riegelii	+	4.92E-01	0.000	0.000	0.000	0.000	0.000
1.1.1.193|g__Corynebacterium.s__Corynebacterium_sp_HMSC08A12	+	4.92E-01	0.000	0.000	0.000	0.000	0.000
1.1.1.193|g__Corynebacterium.s__Corynebacterium_tuberculostearicum	+	4.92E-01	0.000	0.000	0.000	0.000	0.000
1.1.1.193|g__Corynebacterium.s__Corynebacterium_urealyticum	+	4.92E-01	0.000	0.000	0.000	0.000	0.000
1.1.1.193|g__Cronobacter.s__Cronobacter_malonaticus	+	4.92E-01	0.000	0.000	0.000	0.000	0.000
1.1.1.193|g__Cronobacter.s__Cronobacter_sakazakii	+	4.92E-01	0.000	0.000	0.000	0.000	0.000
1.1.1.193|g__Cutibacterium.s__Cutibacterium_avidum	+	4.92E-01	0.000	0.000	0.000	0.000	0.000
1.1.1.193|g__Desulfovibrio.s__Desulfovibrio_desulfuricans	+	1.67E-01	0.000	0.000	0.000	0.000	0.000
1.1.1.193|g__Desulfovibrio.s__Desulfovibrio_fairfieldensis	+	4.42E-01	0.000	0.000	0.000	0.001	0.002
1.1.1.193|g__Desulfovibrio.s__Desulfovibrio_legallii	+	4.92E-01	0.000	0.000	0.000	0.000	0.000
1.1.1.193|g__Desulfovibrio.s__Desulfovibrio_piger	+	3.47E-01	0.000	0.000	0.000	0.000	0.001
1.1.1.193|g__Desulfovibrio.s__Desulfovibrio_sp_AM18_2	+	1.71E-01	0.000	0.000	0.000	0.000	0.000
1.1.1.193|g__Desulfovibrionaceae_unclassified.s__Desulfovibrionaceae_bac	+	7.41E-02	0.000	0.000	0.000	0.000	0.000
1.1.1.193|g__Dialister.s__Dialister_pneumosintes	+	4.92E-01	0.000	0.000	0.000	0.000	0.000
1.1.1.193|g__Dialister.s__Dialister_succinatiphilus	+	4.92E-01	0.000	0.000	0.000	0.000	0.000
1.1.1.193|g__Dorea.s__Dorea_formicigenerans	-	6.72E-01	0.000	0.000	0.000	0.002	0.003
1.1.1.193|g__Dorea.s__Dorea_sp_OM02_2LB	+	1.14E-01	0.000	0.000	0.000	0.000	0.000
1.1.1.193|g__Dysgonomonas.s__Dysgonomonas_mossii	-	9.73E-01	0.000	0.000	0.000	0.000	0.000
1.1.1.193|g__Eggerthella.s__Eggerthella_lenta	+	5.05E-01	0.000	0.000	0.000	0.003	0.002
1.1.1.193|g__Enterobacter.s__Enterobacter_bugandensis	+	5.60E-01	0.000	0.000	0.000	0.000	0.000
1.1.1.193|g__Enterobacter.s__Enterobacter_cloacae	+	7.98E-02	0.000	0.000	0.000	0.000	0.000
1.1.1.193|g__Enterobacter.s__Enterobacter_mori	+	4.92E-01	0.000	0.000	0.000	0.000	0.000
1.1.1.193|g__Enterococcus.s__Enterococcus_avium	+	8.01E-06	0.000	0.000	0.000	0.000	0.001
1.1.1.193|g__Enterococcus.s__Enterococcus_casseliflavus	+	1.67E-01	0.000	0.000	0.000	0.000	0.000
1.1.1.193|g__Enterococcus.s__Enterococcus_faecalis	+	4.92E-01	0.000	0.000	0.000	0.000	0.000
1.1.1.193|g__Enterococcus.s__Enterococcus_faecium	+	4.60E-02	0.000	0.000	0.000	0.000	0.000
1.1.1.193|g__Enterococcus.s__Enterococcus_gallinarum	+	4.01E-03	0.000	0.000	0.000	0.000	0.000
1.1.1.193|g__Enterococcus.s__Enterococcus_malodoratus	+	4.92E-01	0.000	0.000	0.000	0.000	0.000
1.1.1.193|g__Enterococcus.s__Enterococcus_mundtii	+	4.92E-01	0.000	0.000	0.000	0.000	0.000
1.1.1.193|g__Enterococcus.s__Enterococcus_saccharolyticus	+	5.28E-03	0.000	0.000	0.000	0.000	0.000
1.1.1.193|g__Enterococcus.s__Enterococcus_thailandicus	+	4.92E-01	0.000	0.000	0.000	0.000	0.000
1.1.1.193|g__Erysipelatoclostridium.s__Clostridium_innocuum	+	7.69E-03	0.000	0.000	0.000	0.003	0.003
1.1.1.193|g__Erysipelatoclostridium.s__Clostridium_spiroforme	+	4.48E-01	0.000	0.000	0.000	0.002	0.000
1.1.1.193|g__Erysipelotrichaceae_unclassified.s__Erysipelotrichaceae_bacte	+	9.01E-01	0.000	0.000	0.000	0.001	0.000
1.1.1.193|g__Escherichia.s__Escherichia_coli	+	1.16E-02	0.000	0.000	0.000	0.009	0.009
1.1.1.193|g__Escherichia.s__Escherichia_fergusonii	+	5.15E-01	0.000	0.000	0.000	0.000	0.000
1.1.1.193|g__Escherichia.s__Escherichia_marmotae	+	4.92E-01	0.000	0.000	0.000	0.000	0.000
1.1.1.193|g__Eubacterium.s__Eubacterium_callanderi	+	6.36E-05	0.000	0.000	0.000	0.000	0.001
1.1.1.193|g__Eubacterium.s__Eubacterium_eligens	-	9.71E-02	0.000	0.000	0.000	0.006	0.005
1.1.1.193|g__Eubacterium.s__Eubacterium_limosum	+	1.12E-04	0.000	0.000	0.000	0.000	0.000
1.1.1.193|g__Eubacterium.s__Eubacterium_maltosivorans	+	1.50E-01	0.000	0.000	0.000	0.000	0.000
1.1.1.193|g__Eubacterium.s__Eubacterium_ramulus	-	5.79E-02	0.000	0.000	0.000	0.003	0.002
1.1.1.193|g__Eubacterium.s__Eubacterium_sp_AF17_7	-	4.85E-01	0.000	0.000	0.000	0.000	0.000
1.1.1.193|g__Eubacterium.s__Eubacterium_sp_AM18_10LB_B	-	2.67E-01	0.000	0.000	0.000	0.001	0.000
1.1.1.193|g__Eubacterium.s__Eubacterium_sp_An11	+	6.61E-01	0.000	0.000	0.000	0.000	0.001
1.1.1.193|g__Faecalicatena.s__Faecalicatena_contorta	+	1.25E-02	0.000	0.000	0.000	0.000	0.000
1.1.1.193|g__Firmicutes_unclassified.s__Firmicutes_bacterium_AM10_47	-	4.98E-01	0.000	0.000	0.000	0.000	0.000
1.1.1.193|g__Flavonifractor.s__Flavonifractor_plautii	+	8.63E-02	0.000	0.000	0.000	0.001	0.001
1.1.1.193|g__Fusobacterium.s__Fusobacterium_mortiferum	-	7.14E-01	0.000	0.000	0.000	0.000	0.000

1.1.1.193|g__Fusobacterium.s__Fusobacterium_naviforme	+	4.92E-01	0.000	0.000	0.000	0.000	0.000
1.1.1.193|g__Fusobacterium.s__Fusobacterium_nucleatum	-	9.70E-01	0.000	0.000	0.000	0.000	0.000
1.1.1.193|g__Fusobacterium.s__Fusobacterium_ulcerans	-	3.55E-01	0.000	0.000	0.000	0.000	0.000
1.1.1.193|g__Fusobacterium.s__Fusobacterium_varium	+	4.92E-01	0.000	0.000	0.000	0.000	0.000
1.1.1.193|g__Gemella.s__Gemella_haemolysans	+	4.92E-01	0.000	0.000	0.000	0.000	0.000
1.1.1.193|g__Haemophilus.s__Haemophilus_parainfluenzae	-	7.87E-01	0.000	0.000	0.000	0.000	0.000
1.1.1.193|g__Hafnia.s__Hafnia_paralvei	-	2.03E-01	0.000	0.000	0.000	0.000	0.000
1.1.1.193|g__Holdemanella.s__Holdemanella_biformis	-	2.12E-01	0.000	0.000	0.000	0.000	0.000
1.1.1.193|g__Intestinibacter.s__Intestinibacter_bartlettii	-	7.04E-01	0.000	0.000	0.000	0.000	0.000
1.1.1.193|g__Klebsiella.s__Klebsiella_aerogenes	+	1.22E-01	0.000	0.000	0.000	0.000	0.000
1.1.1.193|g__Klebsiella.s__Klebsiella_grimontii	+	3.30E-01	0.000	0.000	0.000	0.000	0.000
1.1.1.193|g__Klebsiella.s__Klebsiella_michiganensis	+	3.09E-01	0.000	0.000	0.000	0.000	0.000
1.1.1.193|g__Klebsiella.s__Klebsiella_oxytoca	+	5.53E-02	0.000	0.000	0.000	0.001	0.002
1.1.1.193|g__Klebsiella.s__Klebsiella_pneumoniae	+	1.16E-03	0.000	0.000	0.000	0.006	0.007
1.1.1.193|g__Klebsiella.s__Klebsiella_variicola	+	1.64E-03	0.000	0.000	0.000	0.000	0.002
1.1.1.193|g__Kluyvera.s__Kluyvera_ascorbata	-	9.73E-01	0.000	0.000	0.000	0.000	0.000
1.1.1.193|g__Kluyvera.s__Kluyvera_cryocrescens	+	3.30E-01	0.000	0.000	0.000	0.000	0.000
1.1.1.193|g__Kosakonia.s__Kosakonia_cowanii	+	4.92E-01	0.000	0.000	0.000	0.000	0.000
1.1.1.193|g__Lachnoclostridium.s__Clostridium_aldenense	+	6.27E-01	0.000	0.000	0.000	0.001	0.001
1.1.1.193|g__Lachnoclostridium.s__Clostridium_citroniae	+	2.73E-02	0.000	0.000	0.000	0.000	0.000
1.1.1.193|g__Lachnoclostridium.s__Clostridium_clostridioforme	-	3.84E-01	0.000	0.000	0.000	0.003	0.006
1.1.1.193|g__Lachnoclostridium.s__Clostridium_symbiosum	-	9.53E-01	0.000	0.000	0.000	0.002	0.002
1.1.1.193|g__Lachnospira.s__Lachnospira_pectinoschiza	-	4.91E-01	0.000	0.000	0.000	0.002	0.002
1.1.1.193|g__Lactobacillus.s__Lactobacillus_amylovorus	-	1.48E-01	0.000	0.000	0.000	0.000	0.000
1.1.1.193|g__Lactobacillus.s__Lactobacillus_brevis	+	4.92E-01	0.000	0.000	0.000	0.000	0.000
1.1.1.193|g__Lactobacillus.s__Lactobacillus_crispatus	+	1.22E-01	0.000	0.000	0.000	0.000	0.000
1.1.1.193|g__Lactobacillus.s__Lactobacillus_delbrueckii	-	4.06E-02	0.000	0.000	0.000	0.000	0.000
1.1.1.193|g__Lactobacillus.s__Lactobacillus_fermentum	+	4.99E-02	0.000	0.000	0.000	0.000	0.001
1.1.1.193|g__Lactobacillus.s__Lactobacillus_gastricus	+	4.92E-01	0.000	0.000	0.000	0.000	0.000
1.1.1.193|g__Lactobacillus.s__Lactobacillus_kimbladii	+	4.92E-01	0.000	0.000	0.000	0.000	0.000
1.1.1.193|g__Lactobacillus.s__Lactobacillus_kullabergensis	+	4.92E-01	0.000	0.000	0.000	0.000	0.000
1.1.1.193|g__Lactobacillus.s__Lactobacillus_oris	+	8.53E-03	0.000	0.000	0.000	0.001	0.001
1.1.1.193|g__Lactobacillus.s__Lactobacillus_pentosus	+	3.05E-01	0.000	0.000	0.000	0.000	0.000
1.1.1.193|g__Lactobacillus.s__Lactobacillus_plantarum	+	2.48E-01	0.000	0.000	0.000	0.000	0.000
1.1.1.193|g__Lactobacillus.s__Lactobacillus_reuteri	+	7.63E-02	0.000	0.000	0.000	0.000	0.002
1.1.1.193|g__Lactobacillus.s__Lactobacillus_rogosae	-	5.65E-01	0.000	0.000	0.000	0.002	0.002
1.1.1.193|g__Lactococcus.s__Lactococcus_lactis	-	7.40E-01	0.000	0.000	0.000	0.000	0.000
1.1.1.193|g__Lactonifactor.s__Lactonifactor_longoviformis	+	4.92E-01	0.000	0.000	0.000	0.000	0.000
1.1.1.193|g__Leclercia.s__Leclercia_adecarboxylata	+	4.15E-01	0.000	0.000	0.000	0.000	0.000
1.1.1.193|g__Lelliottia.s__Lelliottia_nimipressuralis	+	1.30E-01	0.000	0.000	0.000	0.000	0.000
1.1.1.193|g__Leuconostoc.s__Leuconostoc_citreum	+	4.92E-01	0.000	0.000	0.000	0.000	0.000
1.1.1.193|g__Leuconostoc.s__Leuconostoc_garlicum	+	4.92E-01	0.000	0.000	0.000	0.000	0.000
1.1.1.193|g__Leuconostoc.s__Leuconostoc_lactis	+	4.92E-01	0.000	0.000	0.000	0.000	0.000
1.1.1.193|g__Leuconostoc.s__Leuconostoc_mesenteroides	-	5.95E-01	0.000	0.000	0.000	0.000	0.000
1.1.1.193|g__Listeria.s__Listeria_monocytogenes	+	1.70E-01	0.000	0.000	0.000	0.000	0.001
1.1.1.193|g__Megamonas.s__Megamonas_funiformis	+	6.48E-01	0.000	0.000	0.000	0.000	0.000
1.1.1.193|g__Megamonas.s__Megamonas_hypermegale	+	6.76E-01	0.000	0.000	0.000	0.000	0.000
1.1.1.193|g__Megamonas.s__Megamonas_rupellensis	+	6.73E-01	0.000	0.000	0.000	0.000	0.000
1.1.1.193|g__Megasphaera.s__Megasphaera_elsdenii	-	9.62E-01	0.000	0.000	0.000	0.000	0.000
1.1.1.193|g__Megasphaera.s__Megasphaera_hexanoica	+	4.92E-01	0.000	0.000	0.000	0.000	0.000
1.1.1.193|g__Megasphaera.s__Megasphaera_micronuciformis	+	2.32E-01	0.000	0.000	0.000	0.000	0.000
1.1.1.193|g__Megasphaera.s__Megasphaera_stantonii	-	5.92E-01	0.000	0.000	0.000	0.000	0.000
1.1.1.193|g__Mesosutterella.s__Mesosutterella_multiformis	+	2.02E-02	0.000	0.000	0.000	0.001	0.001
1.1.1.193|g__Mitsuokella.s__Mitsuokella_jalaludinii	-	5.92E-01	0.000	0.000	0.000	0.000	0.000
1.1.1.193|g__Mitsuokella.s__Mitsuokella_multacida	+	4.92E-01	0.000	0.000	0.000	0.000	0.000
1.1.1.193|g__Morganella.s__Morganella_morganii	+	3.74E-02	0.000	0.000	0.000	0.000	0.000
1.1.1.193|g__Muribaculum.s__Muribaculum_intestinale	-	5.92E-01	0.000	0.000	0.000	0.000	0.000
1.1.1.193|g__Odoribacter.s__Odoribacter_laneus	+	4.49E-01	0.000	0.000	0.000	0.001	0.004
1.1.1.193|g__Oligella.s__Oligella_urethralis	+	4.92E-01	0.000	0.000	0.000	0.000	0.000
1.1.1.193|g__Oxalobacter.s__Oxalobacter_formigenes	+	3.59E-01	0.000	0.000	0.000	0.000	0.000
1.1.1.193|g__Pantoea.s__Pantoea_sesami	+	2.32E-01	0.000	0.000	0.000	0.000	0.000
1.1.1.193|g__Parabacteroides.s__Parabacteroides_goldsteinii	+	4.65E-02	0.000	0.000	0.000	0.001	0.002
1.1.1.193|g__Parabacteroides.s__Parabacteroides_gordonii	-	3.94E-01	0.000	0.000	0.000	0.000	0.000
1.1.1.193|g__Parabacteroides.s__Parabacteroides_johnsonii	-	4.15E-01	0.000	0.000	0.000	0.001	0.001
1.1.1.193|g__Paraprevotella.s__Paraprevotella_clara	-	1.42E-01	0.000	0.000	0.000	0.004	0.003
1.1.1.193|g__Paraprevotella.s__Paraprevotella_xylaniphila	+	4.16E-01	0.000	0.000	0.000	0.000	0.000
1.1.1.193|g__Parasutterella.s__Parasutterella_excrementihominis	-	3.71E-01	0.000	0.000	0.000	0.003	0.003
1.1.1.193|g__Pediococcus.s__Pediococcus_acidilactici	+	2.12E-02	0.000	0.000	0.000	0.000	0.001
1.1.1.193|g__Pedobacter.s__Pedobacter_himalayensis	+	5.53E-01	0.000	0.000	0.000	0.000	0.000
1.1.1.193|g__Peptococcus.s__Peptococcus_niger	+	3.06E-01	0.000	0.000	0.000	0.000	0.000
1.1.1.193|g__Peptoniphilus.s__Peptoniphilus_harei	+	4.92E-01	0.000	0.000	0.000	0.000	0.000
1.1.1.193|g__Peptoniphilus.s__Peptoniphilus_lacrimalis	+	4.92E-01	0.000	0.000	0.000	0.000	0.000

1.1.1.193|g__Peptoniphilus.s__Peptoniphilus_sp_HMSC062D09	+	3.30E-01	0.000	0.000	0.000	0.000	0.000
1.1.1.193|g__Peptoniphilus.s__Peptoniphilus_sp_oral_taxon_375	+	4.92E-01	0.000	0.000	0.000	0.000	0.000
1.1.1.193|g__Peptostreptococcaceae_unclassified.s__Clostridium_hiranonis	-	5.92E-01	0.000	0.000	0.000	0.000	0.000
1.1.1.193|g__Peptostreptococcus.s__Peptostreptococcus_stomatis	+	3.30E-01	0.000	0.000	0.000	0.000	0.000
1.1.1.193|g__Phascolarctobacterium.s__Phascolarctobacterium_succinatuter	+	1.81E-01	0.000	0.000	0.000	0.001	0.002
1.1.1.193|g__Pluralibacter.s__Pluralibacter_gergoviae	-	1.48E-01	0.000	0.000	0.000	0.000	0.000
1.1.1.193|g__Porphyromonas.s__Porphyromonas_asaccharolytica	+	1.31E-01	0.000	0.000	0.000	0.000	0.000
1.1.1.193|g__Porphyromonas.s__Porphyromonas_sp_HMSC065F10	+	5.53E-01	0.000	0.000	0.000	0.000	0.000
1.1.1.193|g__Porphyromonas.s__Porphyromonas_sp_HMSC077F02	+	5.55E-01	0.000	0.000	0.000	0.000	0.000
1.1.1.193|g__Porphyromonas.s__Porphyromonas_uenonis	-	5.95E-01	0.000	0.000	0.000	0.000	0.000
1.1.1.193|g__Prevotella.s__Prevotella_buccae	+	3.30E-01	0.000	0.000	0.000	0.000	0.000
1.1.1.193|g__Prevotella.s__Prevotella_buccalis	+	3.74E-02	0.000	0.000	0.000	0.000	0.000
1.1.1.193|g__Prevotella.s__Prevotella_colorans	+	3.30E-01	0.000	0.000	0.000	0.000	0.000
1.1.1.193|g__Prevotella.s__Prevotella_copri	-	1.47E-01	0.000	0.000	0.000	0.040	0.016
1.1.1.193|g__Prevotella.s__Prevotella_corporis	+	2.32E-01	0.000	0.000	0.000	0.000	0.000
1.1.1.193|g__Prevotella.s__Prevotella_disiens	+	8.36E-01	0.000	0.000	0.000	0.000	0.000
1.1.1.193|g__Prevotella.s__Prevotella_sp_109	-	7.83E-01	0.000	0.000	0.000	0.003	0.002
1.1.1.193|g__Prevotella.s__Prevotella_sp_AM42_24	-	2.20E-02	0.000	0.000	0.000	0.002	0.000
1.1.1.193|g__Propionibacterium.s__Propionibacterium_acidifaciens	+	4.92E-01	0.000	0.000	0.000	0.000	0.000
1.1.1.193|g__Proteus.s__Proteus_mirabilis	+	4.12E-01	0.000	0.000	0.000	0.000	0.000
1.1.1.193|g__Pseudocitrobacter.s__Pseudocitrobacter_faecalis	+	4.92E-01	0.000	0.000	0.000	0.000	0.000
1.1.1.193|g__Pseudoflavonifractor.s__Pseudoflavonifractor_sp_An184	+	9.97E-02	0.000	0.000	0.000	0.000	0.000
1.1.1.193|g__Pseudomonas.s__Pseudomonas_aeruginosa	+	2.33E-01	0.000	0.000	0.000	0.000	0.000
1.1.1.193|g__Pseudomonas.s__Pseudomonas_aeruginosa_group	+	2.33E-01	0.000	0.000	0.000	0.000	0.000
1.1.1.193|g__Pyramidobacter.s__Pyramidobacter_piscolens	+	2.43E-01	0.000	0.000	0.000	0.000	0.001
1.1.1.193|g__Pyramidobacter.s__Pyramidobacter_sp_C12_8	+	4.92E-01	0.000	0.000	0.000	0.000	0.000
1.1.1.193|g__Raoultella.s__Raoultella_ornithinolytica	-	2.03E-01	0.000	0.000	0.000	0.000	0.000
1.1.1.193|g__Raoultella.s__Raoultella_planticola	-	4.06E-02	0.000	0.000	0.000	0.000	0.000
1.1.1.193|g__Rikenella.s__Rikenella_microfusus	+	4.92E-01	0.000	0.000	0.000	0.000	0.000
1.1.1.193|g__Roseburia.s__Roseburia_faecis	-	7.78E-01	0.000	0.000	0.000	0.009	0.007
1.1.1.193|g__Roseburia.s__Roseburia_intestinalis	-	5.41E-05	0.000	0.000	0.000	0.003	0.001
1.1.1.193|g__Roseburia.s__Roseburia_inulinivorans	-	5.38E-02	0.000	0.000	0.000	0.002	0.002
1.1.1.193|g__Ruminococcaceae_unclassified.s__Ruminococcaceae_bacteriu	+	3.25E-01	0.000	0.000	0.000	0.001	0.002
1.1.1.193|g__Ruminococcaceae_unclassified.s__Ruminococcaceae_bacteriu	+	6.92E-03	0.000	0.000	0.000	0.000	0.001
1.1.1.193|g__Ruminococcus.s__Ruminococcus_callidus	-	4.60E-04	0.000	0.000	0.000	0.003	0.001
1.1.1.193|g__Ruminococcus.s__Ruminococcus_sp_AF31_8BH	-	9.45E-03	0.000	0.000	0.000	0.002	0.001
1.1.1.193|g__Salmonella.s__Salmonella_enterica	+	6.59E-01	0.000	0.000	0.000	0.000	0.000
1.1.1.193|g__Sanguibacteroides.s__Sanguibacteroides_justesenii	+	7.55E-01	0.000	0.000	0.000	0.000	0.000
1.1.1.193|g__Slackia.s__Slackia_isoflavoniconvertens	+	3.94E-02	0.000	0.000	0.000	0.000	0.000
1.1.1.193|g__Staphylococcus.s__Staphylococcus_aureus	+	4.92E-01	0.000	0.000	0.000	0.000	0.000
1.1.1.193|g__Staphylococcus.s__Staphylococcus_epidermidis	+	4.92E-01	0.000	0.000	0.000	0.000	0.000
1.1.1.193|g__Staphylococcus.s__Staphylococcus_haemolyticus	+	4.92E-01	0.000	0.000	0.000	0.000	0.000
1.1.1.193|g__Stomatobaculum.s__Stomatobaculum_longum	+	4.92E-01	0.000	0.000	0.000	0.000	0.000
1.1.1.193|g__Streptococcus.s__Streptococcus_agalactiae	+	4.92E-01	0.000	0.000	0.000	0.000	0.000
1.1.1.193|g__Streptococcus.s__Streptococcus_equinus	+	3.10E-03	0.000	0.000	0.000	0.000	0.002
1.1.1.193|g__Streptococcus.s__Streptococcus_gallolyticus	+	4.24E-02	0.000	0.000	0.000	0.000	0.000
1.1.1.193|g__Streptococcus.s__Streptococcus_infantarius	+	1.63E-02	0.000	0.000	0.000	0.001	0.001
1.1.1.193|g__Streptococcus.s__Streptococcus_lutetiensis	+	1.96E-02	0.000	0.000	0.000	0.000	0.001
1.1.1.193|g__Streptococcus.s__Streptococcus_macedonicus	+	9.30E-02	0.000	0.000	0.000	0.000	0.001
1.1.1.193|g__Streptococcus.s__Streptococcus_mitis	-	1.48E-01	0.000	0.000	0.000	0.000	0.000
1.1.1.193|g__Streptococcus.s__Streptococcus_oralis	-	9.67E-01	0.000	0.000	0.000	0.000	0.000
1.1.1.193|g__Streptococcus.s__Streptococcus_pasteurianus	+	1.19E-01	0.000	0.000	0.000	0.000	0.001
1.1.1.193|g__Streptococcus.s__Streptococcus_pneumoniae	-	2.81E-01	0.000	0.000	0.000	0.000	0.000
1.1.1.193|g__Streptococcus.s__Streptococcus_pseudopneumoniae	+	4.92E-01	0.000	0.000	0.000	0.000	0.000
1.1.1.193|g__Succinatimonas.s__Succinatimonas_hippei	-	1.48E-01	0.000	0.000	0.000	0.000	0.000
1.1.1.193|g__Sutterella.s__Sutterella_wadsworthensis	+	2.48E-01	0.000	0.000	0.000	0.001	0.002
1.1.1.193|g__Terrisporobacter.s__Terrisporobacter_othiniensis	+	1.67E-01	0.000	0.000	0.000	0.000	0.000
1.1.1.193|g__Trichococcus.s__Trichococcus_flocculiformis	+	4.92E-01	0.000	0.000	0.000	0.000	0.000
1.1.1.193|g__Turicimonas.s__Turicimonas_muris	+	8.31E-01	0.000	0.000	0.000	0.000	0.000
1.1.1.193|g__Varibaculum.s__Varibaculum_cambriense	+	3.30E-01	0.000	0.000	0.000	0.000	0.000
1.1.1.193|g__Veillonella.s__Veillonella_atypica	-	9.20E-01	0.000	0.000	0.000	0.000	0.001
1.1.1.193|g__Veillonella.s__Veillonella_dispar	-	4.20E-01	0.000	0.000	0.000	0.000	0.000
1.1.1.193|g__Veillonella.s__Veillonella_infantium	+	4.06E-01	0.000	0.000	0.000	0.000	0.000
1.1.1.193|g__Veillonella.s__Veillonella_parvula	+	4.93E-01	0.000	0.000	0.000	0.000	0.001
1.1.1.193|g__Veillonella.s__Veillonella_rogosae	-	9.21E-01	0.000	0.000	0.000	0.000	0.000
1.1.1.193|g__Vibrio.s__Vibrio_parahaemolyticus	+	8.29E-01	0.000	0.000	0.000	0.000	0.000
1.1.1.193|g__Victivallales_unclassified.s__Victivallales_bacterium_CCUG_	+	1.54E-03	0.000	0.000	0.000	0.000	0.000
1.1.1.193|g__Victivallis.s__Victivallis_vadensis	+	1.47E-01	0.000	0.000	0.000	0.000	0.000
1.1.1.193|g__Weeksella.s__Weeksella_virosa	+	4.92E-01	0.000	0.000	0.000	0.000	0.000
1.1.1.193|g__Weissella.s__Weissella_cibaria	-	9.73E-01	0.000	0.000	0.000	0.000	0.000
1.1.1.193|g__Weissella.s__Weissella_confusa	-	6.66E-02	0.000	0.000	0.000	0.000	0.000
1.1.1.193|g__Yokenella.s__Yokenella_regensburgei	+	4.15E-01	0.000	0.000	0.000	0.000	0.000

	1.1.1.193|g__Hungatella.s__Hungatella_hathewayi	-	8.56E-01	0.070	0.072	0.002	0.005	0.004
	1.1.1.193|g__Bacteroides.s__Bacteroides_thetaiotaomicron	+	4.00E-01	0.450	0.455	0.005	0.014	0.016
	1.1.1.193|g__Bacteroides.s__Bacteroides_dorei	+	2.03E-01	0.030	0.044	0.014	0.015	0.020
	1.1.1.193|g__Odoribacter.s__Odoribacter_splanchnicus	+	3.16E-01	0.199	0.243	0.044	0.003	0.004
	1.1.1.193|g__Bilophila.s__Bilophila_wadsworthia	+	2.31E-02	0.260	0.364	0.104	0.004	0.006
	1.1.1.193|g__Bacteroides.s__Bacteroides_cellulosilyticus	+	2.58E-01	0.045	0.157	0.112	0.011	0.011
	1.1.1.193|g__Bacteroides.s__Bacteroides_intestinalis	+	2.07E-01	0.000	0.115	0.115	0.011	0.015
	1.1.1.193|g__Phascolarctobacterium.s__Phascolarctobacterium_faecium	+	6.72E-01	0.426	0.551	0.125	0.010	0.012
	1.1.1.193|g__Alistipes.s__Alistipes_shahii	+	3.97E-01	0.201	0.341	0.140	0.007	0.009
	1.1.1.193|g__Parabacteroides.s__Parabacteroides_merdae	+	1.34E-01	0.662	0.854	0.192	0.011	0.014
	1.1.1.193|g__Bacteroides.s__Bacteroides_caccae	+	1.66E-01	0.085	0.337	0.252	0.008	0.013
	1.1.1.193|g__Parabacteroides.s__Parabacteroides_distasonis	+	6.79E-03	0.989	1.266	0.277	0.019	0.023
	1.1.1.193|g__Bacteroides.s__Bacteroides_stercoris	+	1.34E-01	0.051	0.674	0.623	0.050	0.059
Germany	1.1.1.193	-	2.23E-02	108.518	94.847	-13.671	1.000	1.000
	1.1.1.193|g__Faecalibacterium.s__Faecalibacterium_prausnitzii	-	2.72E-02	11.343	5.667	-5.676	0.111	0.084
	1.1.1.193|g__Bacteroides.s__Bacteroides_dorei	-	8.38E-01	0.885	0.501	-0.384	0.028	0.020
	1.1.1.193|g__Bacteroides.s__Bacteroides_caccae	-	3.91E-01	0.707	0.345	-0.362	0.017	0.013
	1.1.1.193|g__Dorea.s__Dorea_longicatena	-	6.65E-01	0.979	0.752	-0.227	0.013	0.013
	1.1.1.193|g__Parabacteroides.s__Parabacteroides_distasonis	+	6.32E-01	1.394	1.169	-0.226	0.014	0.017
	1.1.1.193|g__Dorea.s__Dorea_formicigenerans	-	1.27E-01	0.324	0.110	-0.214	0.003	0.002
	1.1.1.193|g__Lachnospiraceae_unclassified.s__Eubacterium_rectale	+	6.87E-01	1.186	0.985	-0.200	0.027	0.030
	1.1.1.193|g__Bacteroides.s__Bacteroides_ovatus	-	4.92E-01	0.178	0.026	-0.151	0.006	0.016
	1.1.1.193|g__Odoribacter.s__Odoribacter_splanchnicus	-	4.84E-01	0.602	0.468	-0.134	0.006	0.006
	1.1.1.193|g__Bacteroides.s__Bacteroides_xylanisolvens	-	8.69E-01	0.312	0.182	-0.130	0.008	0.010
	1.1.1.193|g__Blautia.s__Blautia_wexlerae	-	4.03E-01	0.294	0.194	-0.100	0.006	0.004
	1.1.1.193|g__Bacteroides.s__Bacteroides_thetaiotaomicron	-	2.60E-01	0.372	0.277	-0.094	0.014	0.007
	1.1.1.193|g__Coprococcus.s__Coprococcus_comes	-	9.08E-01	0.364	0.311	-0.053	0.006	0.006
	1.1.1.193|g__Prevotella.s__Prevotella_copri	-	7.97E-02	0.052	0.000	-0.052	0.084	0.037
	1.1.1.193|g__Escherichia.s__Escherichia_coli	-	1.54E-01	0.040	0.000	-0.040	0.011	0.003
	1.1.1.193|g__Acidaminococcus.s__Acidaminococcus_fermentans	-	9.42E-01	0.000	0.000	0.000	0.001	0.000
	1.1.1.193|g__Acidaminococcus.s__Acidaminococcus_intestini	-	9.31E-01	0.000	0.000	0.000	0.002	0.002
	1.1.1.193|g__Adlercreutzia.s__Adlercreutzia_equolifaciens	+	5.32E-02	0.000	0.000	0.000	0.000	0.000
	1.1.1.193|g__Allisonella.s__Allisonella_histaminiformans	+	3.59E-01	0.000	0.000	0.000	0.000	0.000
	1.1.1.193|g__Anaeromassilibacillus.s__Anaeromassilibacillus_sp_An250	+	7.65E-01	0.000	0.000	0.000	0.000	0.000
	1.1.1.193|g__Anaerotignum.s__Anaerotignum_lactatifermentans	-	9.81E-01	0.000	0.000	0.000	0.000	0.000
	1.1.1.193|g__Asaccharobacter.s__Asaccharobacter_celatus	+	2.26E-01	0.000	0.000	0.000	0.001	0.002
	1.1.1.193|g__Bacteroides.s__Bacteroides_clarus	-	5.44E-01	0.000	0.000	0.000	0.004	0.002
	1.1.1.193|g__Bacteroides.s__Bacteroides_coprocola	-	3.39E-01	0.000	0.000	0.000	0.016	0.007
	1.1.1.193|g__Bacteroides.s__Bacteroides_coprophilus	-	8.75E-01	0.000	0.000	0.000	0.005	0.000
	1.1.1.193|g__Bacteroides.s__Bacteroides_eggerthii	+	9.25E-01	0.000	0.000	0.000	0.014	0.004
	1.1.1.193|g__Bacteroides.s__Bacteroides_faecis	+	9.29E-01	0.000	0.000	0.000	0.004	0.004
	1.1.1.193|g__Bacteroides.s__Bacteroides_finegoldii	+	2.91E-01	0.000	0.000	0.000	0.002	0.004
	1.1.1.193|g__Bacteroides.s__Bacteroides_fluxus	-	3.09E-01	0.000	0.000	0.000	0.000	0.000
	1.1.1.193|g__Bacteroides.s__Bacteroides_fragilis	-	9.79E-01	0.000	0.000	0.000	0.004	0.003
	1.1.1.193|g__Bacteroides.s__Bacteroides_massiliensis	-	8.09E-01	0.000	0.000	0.000	0.018	0.016
	1.1.1.193|g__Bacteroides.s__Bacteroides_nordii	-	8.06E-01	0.000	0.000	0.000	0.001	0.000
	1.1.1.193|g__Bacteroides.s__Bacteroides_plebeius	+	3.37E-01	0.000	0.000	0.000	0.003	0.008
	1.1.1.193|g__Bacteroides.s__Bacteroides_salyersiae	+	7.62E-01	0.000	0.000	0.000	0.001	0.004
	1.1.1.193|g__Bacteroides.s__Bacteroides_stercorirosoris	+	3.59E-01	0.000	0.000	0.000	0.000	0.000
	1.1.1.193|g__Bacteroides.s__Bacteroides_stercoris	-	7.80E-01	0.000	0.000	0.000	0.018	0.011
	1.1.1.193|g__Blautia.s__Blautia_hansenii	+	3.59E-01	0.000	0.000	0.000	0.000	0.000
	1.1.1.193|g__Blautia.s__Blautia_sp_AF19_10LB	+	1.73E-01	0.000	0.000	0.000	0.001	0.001
	1.1.1.193|g__Butyricicoccus.s__Butyricicoccus_pullicaecorum	+	3.59E-01	0.000	0.000	0.000	0.000	0.000
	1.1.1.193|g__Butyricimonas.s__Butyricimonas_virosa	-	3.00E-01	0.000	0.000	0.000	0.001	0.001
	1.1.1.193|g__Butyrivibrio.s__Butyrivibrio_crossotus	+	8.14E-01	0.000	0.000	0.000	0.012	0.004
	1.1.1.193|g__Candidatus_Gastranaerophilales_unclassified.s__Candidatus_(	-	9.81E-01	0.000	0.000	0.000	0.000	0.000
	1.1.1.193|g__Candidatus_Methanomethylophilus.s__Candidatus_Methanom	+	3.59E-01	0.000	0.000	0.000	0.000	0.001
	1.1.1.193|g__Catenibacterium.s__Catenibacterium_mitsuokai	-	9.76E-02	0.000	0.000	0.000	0.015	0.006
	1.1.1.193|g__Citrobacter.s__Citrobacter_sp_MGH106	+	3.59E-01	0.000	0.000	0.000	0.000	0.000
	1.1.1.193|g__Cloacibacillus.s__Cloacibacillus_porcorum	+	1.83E-01	0.000	0.000	0.000	0.000	0.001
	1.1.1.193|g__Clostridioides.s__Clostridioides_difficile	-	1.40E-01	0.000	0.000	0.000	0.000	0.000
	1.1.1.193|g__Clostridium.s__Clostridium_disporicum	-	1.40E-01	0.000	0.000	0.000	0.000	0.000
	1.1.1.193|g__Coprobacillus.s__Coprobacillus_cateniformis	+	2.74E-01	0.000	0.000	0.000	0.000	0.001
	1.1.1.193|g__Coprobacter.s__Coprobacter_fastidiosus	-	6.01E-01	0.000	0.000	0.000	0.009	0.003
	1.1.1.193|g__Coprobacter.s__Coprobacter_secundus	-	9.42E-01	0.000	0.000	0.000	0.000	0.000
	1.1.1.193|g__Coprococcus.s__Coprococcus_catus	-	7.73E-01	0.000	0.000	0.000	0.002	0.002
	1.1.1.193|g__Coprococcus.s__Coprococcus_eutactus	-	8.49E-01	0.000	0.000	0.000	0.017	0.012
	1.1.1.193|g__Desulfovibrio.s__Desulfovibrio_fairfieldensis	+	3.38E-01	0.000	0.000	0.000	0.000	0.001
	1.1.1.193|g__Desulfovibrio.s__Desulfovibrio_piger	-	7.71E-01	0.000	0.000	0.000	0.001	0.010
	1.1.1.193|g__Desulfovibrionaceae_unclassified.s__Desulfovibrionaceae_bac	-	1.40E-01	0.000	0.000	0.000	0.000	0.000
	1.1.1.193|g__Dialister.s__Dialister_succinatiphilus	-	9.42E-01	0.000	0.000	0.000	0.001	0.001
	1.1.1.193|g__Dorea.s__Dorea_sp_OM02_2LB	-	9.81E-01	0.000	0.000	0.000	0.000	0.000

1.1.1.193|g__Eggerthella.s__Eggerthella_lenta	-	6.66E-02	0.000	0.000	0.000	0.000	0.000
1.1.1.193|g__Enterobacter.s__Enterobacter_cloacae	-	3.09E-01	0.000	0.000	0.000	0.000	0.000
1.1.1.193|g__Enterococcus.s__Enterococcus_faecium	-	3.09E-01	0.000	0.000	0.000	0.000	0.000
1.1.1.193|g__Enterococcus.s__Enterococcus_hirae	-	3.09E-01	0.000	0.000	0.000	0.001	0.000
1.1.1.193|g__Erysipelatoclostridium.s__Clostridium_innocuum	-	1.40E-01	0.000	0.000	0.000	0.000	0.000
1.1.1.193|g__Erysipelatoclostridium.s__Clostridium_spiroforme	+	7.41E-01	0.000	0.000	0.000	0.001	0.000
1.1.1.193|g__Erysipelotrichaceae_unclassified.s__Erysipelotrichaceae_bacte	-	3.09E-01	0.000	0.000	0.000	0.000	0.000
1.1.1.193|g__Escherichia.s__Escherichia_fergusonii	+	3.59E-01	0.000	0.000	0.000	0.000	0.000
1.1.1.193|g__Escherichia.s__Escherichia_marmotae	-	3.09E-01	0.000	0.000	0.000	0.000	0.000
1.1.1.193|g__Eubacterium.s__Eubacterium_ramulus	-	5.66E-01	0.000	0.000	0.000	0.001	0.002
1.1.1.193|g__Eubacterium.s__Eubacterium_sp_AF17_7	+	1.98E-01	0.000	0.000	0.000	0.000	0.001
1.1.1.193|g__Eubacterium.s__Eubacterium_sp_AM18_10LB_B	-	3.09E-01	0.000	0.000	0.000	0.000	0.000
1.1.1.193|g__Firmicutes_unclassified.s__Firmicutes_bacterium_AM10_47	-	5.50E-01	0.000	0.000	0.000	0.000	0.000
1.1.1.193|g__Flavonifractor.s__Flavonifractor_plautii	+	5.85E-02	0.000	0.000	0.000	0.000	0.002
1.1.1.193|g__Fusobacterium.s__Fusobacterium_mortiferum	-	3.09E-01	0.000	0.000	0.000	0.000	0.000
1.1.1.193|g__Haemophilus.s__Haemophilus_parainfluenzae	-	7.63E-02	0.000	0.000	0.000	0.001	0.000
1.1.1.193|g__Hafnia.s__Hafnia_alvei	+	1.83E-01	0.000	0.000	0.000	0.000	0.001
1.1.1.193|g__Hafnia.s__Hafnia_paralvei	+	3.59E-01	0.000	0.000	0.000	0.000	0.001
1.1.1.193|g__Holdemanella.s__Holdemanella_biformis	-	2.76E-02	0.000	0.000	0.000	0.001	0.000
1.1.1.193|g__Hungatella.s__Hungatella_hathewayi	-	2.11E-01	0.000	0.000	0.000	0.001	0.000
1.1.1.193|g__Intestinibacter.s__Intestinibacter_bartlettii	-	9.42E-01	0.000	0.000	0.000	0.000	0.000
1.1.1.193|g__Klebsiella.s__Klebsiella_oxytoca	-	5.77E-01	0.000	0.000	0.000	0.003	0.001
1.1.1.193|g__Klebsiella.s__Klebsiella_pneumoniae	-	8.00E-02	0.000	0.000	0.000	0.003	0.002
1.1.1.193|g__Klebsiella.s__Klebsiella_variicola	-	5.37E-01	0.000	0.000	0.000	0.000	0.000
1.1.1.193|g__Lachnoclostridium.s__Clostridium_aldenense	-	3.09E-01	0.000	0.000	0.000	0.000	0.000
1.1.1.193|g__Lachnoclostridium.s__Clostridium_bolteae	-	5.94E-01	0.000	0.000	0.000	0.000	0.001
1.1.1.193|g__Lachnoclostridium.s__Clostridium_citroniae	-	3.09E-01	0.000	0.000	0.000	0.000	0.000
1.1.1.193|g__Lachnoclostridium.s__Clostridium_clostridioforme	-	3.22E-02	0.000	0.000	0.000	0.000	0.000
1.1.1.193|g__Lachnoclostridium.s__Clostridium_symbiosum	-	1.40E-01	0.000	0.000	0.000	0.000	0.000
1.1.1.193|g__Lachnospira.s__Lachnospira_pectinoschiza	+	5.52E-01	0.000	0.000	0.000	0.001	0.002
1.1.1.193|g__Lactobacillus.s__Lactobacillus_antri	-	3.09E-01	0.000	0.000	0.000	0.000	0.000
1.1.1.193|g__Lactobacillus.s__Lactobacillus_crispatus	-	3.09E-01	0.000	0.000	0.000	0.000	0.000
1.1.1.193|g__Lactobacillus.s__Lactobacillus_delbrueckii	-	3.09E-01	0.000	0.000	0.000	0.000	0.000
1.1.1.193|g__Lactobacillus.s__Lactobacillus_fermentum	-	1.40E-01	0.000	0.000	0.000	0.000	0.000
1.1.1.193|g__Lactobacillus.s__Lactobacillus_gastricus	-	3.09E-01	0.000	0.000	0.000	0.000	0.000
1.1.1.193|g__Lactobacillus.s__Lactobacillus_oris	-	3.09E-01	0.000	0.000	0.000	0.000	0.000
1.1.1.193|g__Lactobacillus.s__Lactobacillus_rogosae	+	5.04E-01	0.000	0.000	0.000	0.001	0.003
1.1.1.193|g__Lactococcus.s__Lactococcus_lactis	-	1.40E-01	0.000	0.000	0.000	0.001	0.000
1.1.1.193|g__Listeria.s__Listeria_monocytogenes	+	1.83E-01	0.000	0.000	0.000	0.000	0.000
1.1.1.193|g__Megamonas.s__Megamonas_funiformis	+	3.59E-01	0.000	0.000	0.000	0.000	0.000
1.1.1.193|g__Megamonas.s__Megamonas_hypermegale	+	3.59E-01	0.000	0.000	0.000	0.000	0.001
1.1.1.193|g__Megamonas.s__Megamonas_rupellensis	+	3.59E-01	0.000	0.000	0.000	0.000	0.001
1.1.1.193|g__Megasphaera.s__Megasphaera_elsdenii	-	4.85E-01	0.000	0.000	0.000	0.001	0.000
1.1.1.193|g__Megasphaera.s__Megasphaera_hexanoica	-	3.09E-01	0.000	0.000	0.000	0.000	0.000
1.1.1.193|g__Mesosutterella.s__Mesosutterella_multiformis	+	1.98E-01	0.000	0.000	0.000	0.000	0.001
1.1.1.193|g__Mitsuokella.s__Mitsuokella_jalaludinii	-	5.11E-01	0.000	0.000	0.000	0.001	0.000
1.1.1.193|g__Mitsuokella.s__Mitsuokella_multacida	-	3.09E-01	0.000	0.000	0.000	0.001	0.000
1.1.1.193|g__Obesumbacterium.s__Obesumbacterium_proteus	+	1.83E-01	0.000	0.000	0.000	0.000	0.000
1.1.1.193|g__Oxalobacter.s__Oxalobacter_formigenes	-	9.31E-01	0.000	0.000	0.000	0.000	0.000
1.1.1.193|g__Parabacteroides.s__Parabacteroides_goldsteinii	-	3.32E-01	0.000	0.000	0.000	0.001	0.000
1.1.1.193|g__Parabacteroides.s__Parabacteroides_gordonii	+	9.80E-02	0.000	0.000	0.000	0.000	0.000
1.1.1.193|g__Parabacteroides.s__Parabacteroides_johnsonii	-	2.91E-01	0.000	0.000	0.000	0.002	0.000
1.1.1.193|g__Paraprevotella.s__Paraprevotella_clara	-	7.45E-02	0.000	0.000	0.000	0.005	0.002
1.1.1.193|g__Parasutterella.s__Parasutterella_excrementihominis	+	8.74E-01	0.000	0.000	0.000	0.001	0.002
1.1.1.193|g__Phascolarctobacterium.s__Phascolarctobacterium_faecium	+	2.83E-01	0.000	0.000	0.000	0.004	0.011
1.1.1.193|g__Phascolarctobacterium.s__Phascolarctobacterium_succinatuter	-	2.92E-02	0.000	0.000	0.000	0.009	0.002
1.1.1.193|g__Prevotella.s__Prevotella_disiens	-	3.09E-01	0.000	0.000	0.000	0.000	0.000
1.1.1.193|g__Prevotella.s__Prevotella_sp_109	+	1.83E-01	0.000	0.000	0.000	0.000	0.011
1.1.1.193|g__Prevotella.s__Prevotella_sp_AM42_24	-	5.11E-01	0.000	0.000	0.000	0.013	0.008
1.1.1.193|g__Pseudoflavonifractor.s__Pseudoflavonifractor_sp_An184	-	9.42E-01	0.000	0.000	0.000	0.001	0.000
1.1.1.193|g__Pseudomonas.s__Pseudomonas_aeruginosa	-	3.09E-01	0.000	0.000	0.000	0.000	0.000
1.1.1.193|g__Pseudomonas.s__Pseudomonas_aeruginosa_group	-	3.09E-01	0.000	0.000	0.000	0.000	0.000
1.1.1.193|g__Pyramidobacter.s__Pyramidobacter_piscolens	-	3.09E-01	0.000	0.000	0.000	0.000	0.000
1.1.1.193|g__Roseburia.s__Roseburia_faecis	-	8.78E-01	0.000	0.000	0.000	0.011	0.007
1.1.1.193|g__Roseburia.s__Roseburia_intestinalis	-	4.59E-01	0.000	0.000	0.000	0.007	0.003
1.1.1.193|g__Roseburia.s__Roseburia_inulinivorans	+	2.24E-01	0.000	0.000	0.000	0.002	0.003
1.1.1.193|g__Ruminococcaceae_unclassified.s__Ruminococcaceae_bacteriu	-	2.73E-01	0.000	0.000	0.000	0.001	0.000
1.1.1.193|g__Ruminococcaceae_unclassified.s__Ruminococcaceae_bacteriu	+	5.32E-02	0.000	0.000	0.000	0.000	0.001
1.1.1.193|g__Ruminococcus.s__Ruminococcus_callidus	-	5.60E-01	0.000	0.000	0.000	0.002	0.001
1.1.1.193|g__Ruminococcus.s__Ruminococcus_sp_AF31_8BH	+	2.54E-01	0.000	0.000	0.000	0.001	0.002
1.1.1.193|g__Salmonella.s__Salmonella_enterica	+	3.59E-01	0.000	0.000	0.000	0.000	0.000
1.1.1.193|g__Sanguibacteroides.s__Sanguibacteroides_justesenii	-	3.09E-01	0.000	0.000	0.000	0.000	0.000

	1.1.1.193|g__Slackia.s__Slackia_isoflavoniconvertens	-	2.02E-01	0.000	0.000	0.000	0.003	0.004
	1.1.1.193|g__Streptococcus.s__Streptococcus_equinus	-	9.81E-01	0.000	0.000	0.000	0.000	0.000
	1.1.1.193|g__Streptococcus.s__Streptococcus_infantarius	+	3.59E-01	0.000	0.000	0.000	0.000	0.000
	1.1.1.193|g__Streptococcus.s__Streptococcus_lutetiensis	+	3.59E-01	0.000	0.000	0.000	0.000	0.000
	1.1.1.193|g__Streptococcus.s__Streptococcus_macedonicus	-	3.09E-01	0.000	0.000	0.000	0.000	0.000
	1.1.1.193|g__Streptococcus.s__Streptococcus_pasteurianus	-	3.09E-01	0.000	0.000	0.000	0.000	0.000
	1.1.1.193|g__Sutterella.s__Sutterella_wadsworthensis	-	5.72E-01	0.000	0.000	0.000	0.001	0.001
	1.1.1.193|g__Turicimonas.s__Turicimonas_muris	-	1.40E-01	0.000	0.000	0.000	0.001	0.000
	1.1.1.193|g__Veillonella.s__Veillonella_atypica	-	3.09E-01	0.000	0.000	0.000	0.000	0.000
	1.1.1.193|g__Veillonella.s__Veillonella_dispar	-	5.11E-01	0.000	0.000	0.000	0.000	0.000
	1.1.1.193|g__Veillonella.s__Veillonella_infantium	-	3.09E-01	0.000	0.000	0.000	0.000	0.000
	1.1.1.193|g__Veillonella.s__Veillonella_parvula	+	1.83E-01	0.000	0.000	0.000	0.000	0.000
	1.1.1.193|g__Veillonella.s__Veillonella_rogosae	-	3.09E-01	0.000	0.000	0.000	0.000	0.000
	1.1.1.193|g__Victivallales_unclassified.s__Victivallales_bacterium_CCUG_	+	4.28E-01	0.000	0.000	0.000	0.001	0.002
	1.1.1.193|g__Victivallis.s__Victivallis_vadensis	+	1.87E-01	0.000	0.000	0.000	0.000	0.001
	1.1.1.193|g__Blautia.s__Blautia_obeum	-	7.16E-01	0.741	0.789	0.048	0.010	0.012
	1.1.1.193|g__Agathobaculum.s__Agathobaculum_butyriciproducens	+	6.23E-01	0.196	0.253	0.057	0.003	0.006
	1.1.1.193|g__Eubacterium.s__Eubacterium_eligens	+	8.61E-01	0.782	0.978	0.196	0.021	0.017
	1.1.1.193|g__Clostridium.s__Clostridium_sp_AM22_11AC	+	2.15E-01	0.000	0.201	0.201	0.002	0.003
	1.1.1.193|g__Roseburia.s__Roseburia_hominis	+	1.55E-01	0.557	0.771	0.215	0.007	0.012
	1.1.1.193|g__Bilophila.s__Bilophila_wadsworthia	+	2.88E-01	0.000	0.217	0.217	0.003	0.004
	1.1.1.193|g__Anaerostipes.s__Anaerostipes_hadrus	+	1.12E-01	0.055	0.276	0.221	0.004	0.008
	1.1.1.193|g__Bacteroides.s__Bacteroides_cellulosilyticus	+	5.53E-01	0.255	0.507	0.252	0.010	0.010
	1.1.1.193|g__Blautia.s__Ruminococcus_torques	+	5.90E-01	1.226	1.485	0.259	0.016	0.026
	1.1.1.193|g__Clostridium.s__Clostridium_sp_AF36_4	+	1.30E-02	0.000	0.280	0.280	0.002	0.007
	1.1.1.193|g__Bacteroides.s__Bacteroides_intestinalis	+	2.76E-01	0.151	0.442	0.291	0.007	0.008
	1.1.1.193|g__Bacteroides.s__Bacteroides_uniformis	+	4.34E-01	1.901	2.226	0.325	0.040	0.060
	1.1.1.193|g__Barnesiella.s__Barnesiella_intestinihominis	+	4.06E-01	0.695	1.046	0.351	0.013	0.018
	1.1.1.193|g__Parabacteroides.s__Parabacteroides_merdae	+	3.37E-01	0.297	0.938	0.640	0.008	0.012
	1.1.1.193|g__Alistipes.s__Alistipes_shahii	+	3.58E-04	0.080	0.976	0.895	0.005	0.017
	1.1.1.193|g__Bacteroides.s__Bacteroides_vulgatus	-	8.67E-01	2.719	3.878	1.159	0.079	0.072
	1.1.1.193|unclassified	+	5.29E-01	11.438	14.100	2.662	0.153	0.185
	1.1.1.193|g__Akkermansia.s__Akkermansia_muciniphila	+	6.97E-04	0.000	3.030	3.030	0.020	0.056
China1	1.1.1.193	-	1.55E-02	110.244	101.073	-9.172	1.000	1.000
	1.1.1.193|g__Bacteroides.s__Bacteroides_vulgatus	-	1.36E-02	10.166	3.046	-7.119	0.154	0.077
	1.1.1.193|g__Bacteroides.s__Bacteroides_ovatus	-	3.08E-02	2.870	1.503	-1.367	0.060	0.028
	1.1.1.193|g__Bacteroides.s__Bacteroides_fragilis	-	2.36E-01	1.776	1.001	-0.775	0.028	0.031
	1.1.1.193|g__Bacteroides.s__Bacteroides_xylanisolvens	-	4.70E-01	0.784	0.592	-0.192	0.024	0.014
	1.1.1.193|g__Faecalibacterium.s__Faecalibacterium_prausnitzii	+	9.35E-01	1.002	0.889	-0.113	0.016	0.019
	1.1.1.193|g__Parabacteroides.s__Parabacteroides_distasonis	+	6.62E-01	1.189	1.097	-0.092	0.024	0.019
	1.1.1.193|g__Escherichia.s__Escherichia_coli	-	2.00E-01	0.045	0.001	-0.044	0.003	0.002
	1.1.1.193|g__Bacteroides.s__Bacteroides_cellulosilyticus	+	9.08E-01	0.405	0.372	-0.033	0.013	0.021
	1.1.1.193|g__Prevotella.s__Prevotella_copri	+	8.75E-01	0.067	0.038	-0.029	0.117	0.113
	1.1.1.193|g__Blautia.s__Blautia_wexlerae	-	4.85E-01	0.016	0.000	-0.016	0.001	0.001
	1.1.1.193|g__Acidaminococcus.s__Acidaminococcus_intestini	+	3.30E-01	0.000	0.000	0.000	0.000	0.000
	1.1.1.193|g__Adlercreutzia.s__Adlercreutzia_equolifaciens	-	6.71E-01	0.000	0.000	0.000	0.000	0.000
	1.1.1.193|g__Aeromonas.s__Aeromonas_dhakensis	-	3.30E-01	0.000	0.000	0.000	0.000	0.000
	1.1.1.193|g__Aeromonas.s__Aeromonas_enteropelogenes	-	3.30E-01	0.000	0.000	0.000	0.000	0.000
	1.1.1.193|g__Aeromonas.s__Aeromonas_hydrophila	-	3.30E-01	0.000	0.000	0.000	0.000	0.000
	1.1.1.193|g__Aeromonas.s__Aeromonas_veronii	-	3.30E-01	0.000	0.000	0.000	0.000	0.000
	1.1.1.193|g__Agathobaculum.s__Agathobaculum_butyriciproducens	-	9.90E-01	0.000	0.000	0.000	0.001	0.000
	1.1.1.193|g__Aggregatibacter.s__Aggregatibacter_segnis	-	1.00E+00	0.000	0.000	0.000	0.000	0.000
	1.1.1.193|g__Akkermansia.s__Akkermansia_muciniphila	+	6.21E-03	0.000	0.000	0.000	0.001	0.008
	1.1.1.193|g__Allisonella.s__Allisonella_histaminiformans	+	9.90E-01	0.000	0.000	0.000	0.000	0.000
	1.1.1.193|g__Anaeroglobus.s__Anaeroglobus_geminatus	+	3.30E-01	0.000	0.000	0.000	0.000	0.000
	1.1.1.193|g__Anaeromassilibacillus.s__Anaeromassilibacillus_sp_An250	+	3.30E-01	0.000	0.000	0.000	0.000	0.000
	1.1.1.193|g__Anaerostipes.s__Anaerostipes_caccae	-	3.30E-01	0.000	0.000	0.000	0.000	0.000
	1.1.1.193|g__Anaerostipes.s__Anaerostipes_hadrus	-	7.14E-01	0.000	0.000	0.000	0.001	0.000
	1.1.1.193|g__Asaccharobacter.s__Asaccharobacter_celatus	-	6.55E-01	0.000	0.000	0.000	0.000	0.000
	1.1.1.193|g__Bacteroides.s__Bacteroides_clarus	+	1.40E-01	0.000	0.000	0.000	0.001	0.005
	1.1.1.193|g__Bacteroides.s__Bacteroides_coprocola	-	1.50E-01	0.000	0.000	0.000	0.048	0.011
	1.1.1.193|g__Bacteroides.s__Bacteroides_coprophilus	-	3.93E-01	0.000	0.000	0.000	0.005	0.001
	1.1.1.193|g__Bacteroides.s__Bacteroides_eggerthii	+	1.05E-01	0.000	0.000	0.000	0.018	0.036
	1.1.1.193|g__Bacteroides.s__Bacteroides_faecis	-	1.58E-01	0.000	0.000	0.000	0.001	0.000
	1.1.1.193|g__Bacteroides.s__Bacteroides_finegoldii	+	4.16E-01	0.000	0.000	0.000	0.009	0.014
	1.1.1.193|g__Bacteroides.s__Bacteroides_fluxus	+	3.30E-01	0.000	0.000	0.000	0.000	0.000
	1.1.1.193|g__Bacteroides.s__Bacteroides_massiliensis	+	6.35E-01	0.000	0.000	0.000	0.009	0.015
	1.1.1.193|g__Bacteroides.s__Bacteroides_oleiciplenus	+	5.89E-01	0.000	0.000	0.000	0.000	0.000
	1.1.1.193|g__Bacteroides.s__Bacteroides_plebeius	-	2.80E-01	0.000	0.000	0.000	0.058	0.020
	1.1.1.193|g__Bacteroides.s__Bacteroides_salyersiae	-	4.07E-01	0.000	0.000	0.000	0.002	0.002
	1.1.1.193|g__Bacteroides.s__Bacteroides_sp_OM08_11	+	8.20E-02	0.000	0.000	0.000	0.000	0.000
	1.1.1.193|g__Bacteroides.s__Bacteroides_stercorirosoris	+	5.69E-01	0.000	0.000	0.000	0.000	0.000

1.1.1.193|g__Barnesiella.s__Barnesiella_intestinihominis	+	3.80E-01	0.000	0.000	0.000	0.007	0.007
1.1.1.193|g__Barnesiella.s__Barnesiella_sp_An22	-	3.30E-01	0.000	0.000	0.000	0.000	0.000
1.1.1.193|g__Blautia.s__Blautia_hansenii	+	3.30E-01	0.000	0.000	0.000	0.000	0.000
1.1.1.193|g__Blautia.s__Blautia_sp_AF19_10LB	+	9.63E-01	0.000	0.000	0.000	0.000	0.001
1.1.1.193|g__Butyricimonas.s__Butyricimonas_virosa	+	9.14E-01	0.000	0.000	0.000	0.002	0.003
1.1.1.193|g__Butyrivibrio.s__Butyrivibrio_crossotus	+	3.20E-01	0.000	0.000	0.000	0.000	0.001
1.1.1.193|g__Campylobacter.s__Campylobacter_gracilis	+	3.30E-01	0.000	0.000	0.000	0.000	0.000
1.1.1.193|g__Catenibacterium.s__Catenibacterium_mitsuokai	+	6.06E-01	0.000	0.000	0.000	0.001	0.007
1.1.1.193|g__Citrobacter.s__Citrobacter_braakii	+	1.60E-01	0.000	0.000	0.000	0.000	0.000
1.1.1.193|g__Citrobacter.s__Citrobacter_freundii	+	1.60E-01	0.000	0.000	0.000	0.000	0.000
1.1.1.193|g__Citrobacter.s__Citrobacter_portucalensis	+	3.20E-01	0.000	0.000	0.000	0.000	0.000
1.1.1.193|g__Citrobacter.s__Citrobacter_youngae	+	4.28E-02	0.000	0.000	0.000	0.000	0.000
1.1.1.193|g__Cloacibacillus.s__Cloacibacillus_porcorum	+	1.60E-01	0.000	0.000	0.000	0.000	0.000
1.1.1.193|g__Clostridioides.s__Clostridioides_difficile	-	7.06E-01	0.000	0.000	0.000	0.001	0.000
1.1.1.193|g__Clostridium.s__Clostridium_neonatale	-	1.60E-01	0.000	0.000	0.000	0.000	0.000
1.1.1.193|g__Clostridium.s__Clostridium_perfringens	+	3.30E-01	0.000	0.000	0.000	0.000	0.000
1.1.1.193|g__Clostridium.s__Clostridium_sp_AF36_4	+	8.96E-02	0.000	0.000	0.000	0.001	0.005
1.1.1.193|g__Clostridium.s__Clostridium_sp_AM22_11AC	-	2.35E-01	0.000	0.000	0.000	0.001	0.000
1.1.1.193|g__Clostridium.s__Clostridium_sp_chh4_2	+	3.30E-01	0.000	0.000	0.000	0.000	0.000
1.1.1.193|g__Comamonas.s__Comamonas_kerstersii	+	3.30E-01	0.000	0.000	0.000	0.000	0.000
1.1.1.193|g__Coprobacillus.s__Coprobacillus_cateniformis	+	9.05E-02	0.000	0.000	0.000	0.000	0.001
1.1.1.193|g__Coprobacter.s__Coprobacter_fastidiosus	-	4.77E-01	0.000	0.000	0.000	0.002	0.001
1.1.1.193|g__Coprobacter.s__Coprobacter_secundus	-	7.99E-01	0.000	0.000	0.000	0.000	0.000
1.1.1.193|g__Coprococcus.s__Coprococcus_catus	+	2.58E-01	0.000	0.000	0.000	0.000	0.000
1.1.1.193|g__Coprococcus.s__Coprococcus_comes	+	6.30E-02	0.000	0.000	0.000	0.000	0.001
1.1.1.193|g__Coprococcus.s__Coprococcus_eutactus	+	1.81E-01	0.000	0.000	0.000	0.001	0.003
1.1.1.193|g__Desulfovibrio.s__Desulfovibrio_piger	+	1.65E-01	0.000	0.000	0.000	0.000	0.000
1.1.1.193|g__Desulfovibrio.s__Desulfovibrio_sp_AM18_2	+	8.20E-02	0.000	0.000	0.000	0.000	0.002
1.1.1.193|g__Desulfovibrionaceae_unclassified.s__Desulfovibrionaceae_bac	+	3.30E-01	0.000	0.000	0.000	0.000	0.000
1.1.1.193|g__Dialister.s__Dialister_succinatiphilus	-	5.89E-01	0.000	0.000	0.000	0.000	0.000
1.1.1.193|g__Dorea.s__Dorea_formicigenerans	-	3.78E-01	0.000	0.000	0.000	0.001	0.000
1.1.1.193|g__Dorea.s__Dorea_sp_OM02_2LB	+	3.30E-01	0.000	0.000	0.000	0.000	0.000
1.1.1.193|g__Eggerthella.s__Eggerthella_lenta	+	4.49E-01	0.000	0.000	0.000	0.000	0.000
1.1.1.193|g__Enterobacter.s__Enterobacter_cloacae	+	9.90E-01	0.000	0.000	0.000	0.000	0.000
1.1.1.193|g__Enterococcus.s__Enterococcus_faecium	+	3.30E-01	0.000	0.000	0.000	0.000	0.000
1.1.1.193|g__Enterococcus.s__Enterococcus_hirae	+	3.30E-01	0.000	0.000	0.000	0.000	0.000
1.1.1.193|g__Erysipelatoclostridium.s__Clostridium_innocuum	-	6.82E-01	0.000	0.000	0.000	0.001	0.000
1.1.1.193|g__Erysipelatoclostridium.s__Clostridium_spiroforme	+	3.30E-01	0.000	0.000	0.000	0.000	0.000
1.1.1.193|g__Erysipelotrichaceae_unclassified.s__Erysipelotrichaceae_bacte	-	3.30E-01	0.000	0.000	0.000	0.000	0.000
1.1.1.193|g__Escherichia.s__Escherichia_fergusonii	+	1.60E-01	0.000	0.000	0.000	0.000	0.000
1.1.1.193|g__Eubacterium.s__Eubacterium_ramulus	+	6.71E-01	0.000	0.000	0.000	0.000	0.000
1.1.1.193|g__Eubacterium.s__Eubacterium_sp_AF17_7	+	1.79E-01	0.000	0.000	0.000	0.000	0.001
1.1.1.193|g__Eubacterium.s__Eubacterium_sp_AM18_10LB_B	+	1.00E+00	0.000	0.000	0.000	0.000	0.001
1.1.1.193|g__Faecalicatena.s__Faecalicatena_contorta	+	3.30E-01	0.000	0.000	0.000	0.000	0.000
1.1.1.193|g__Firmicutes_unclassified.s__Firmicutes_bacterium_AM10_47	+	5.69E-01	0.000	0.000	0.000	0.000	0.000
1.1.1.193|g__Flavonifractor.s__Flavonifractor_plautii	+	8.20E-02	0.000	0.000	0.000	0.000	0.000
1.1.1.193|g__Fusobacterium.s__Fusobacterium_hwasookii	-	3.30E-01	0.000	0.000	0.000	0.000	0.000
1.1.1.193|g__Fusobacterium.s__Fusobacterium_mortiferum	+	3.33E-01	0.000	0.000	0.000	0.001	0.000
1.1.1.193|g__Fusobacterium.s__Fusobacterium_ulcerans	-	4.17E-01	0.000	0.000	0.000	0.001	0.001
1.1.1.193|g__Fusobacterium.s__Fusobacterium_varium	-	1.60E-01	0.000	0.000	0.000	0.001	0.000
1.1.1.193|g__Haemophilus.s__Haemophilus_parainfluenzae	-	7.26E-01	0.000	0.000	0.000	0.002	0.002
1.1.1.193|g__Haemophilus.s__Haemophilus_sputorum	+	1.00E+00	0.000	0.000	0.000	0.000	0.000
1.1.1.193|g__Hafnia.s__Hafnia_paralvei	-	3.30E-01	0.000	0.000	0.000	0.000	0.000
1.1.1.193|g__Holdemanella.s__Holdemanella_biformis	+	1.64E-03	0.000	0.000	0.000	0.000	0.000
1.1.1.193|g__Hungatella.s__Hungatella_hathewayi	-	9.91E-01	0.000	0.000	0.000	0.001	0.001
1.1.1.193|g__Intestinibacter.s__Intestinibacter_bartlettii	+	1.52E-01	0.000	0.000	0.000	0.000	0.000
1.1.1.193|g__Klebsiella.s__Klebsiella_aerogenes	+	3.30E-01	0.000	0.000	0.000	0.000	0.000
1.1.1.193|g__Klebsiella.s__Klebsiella_oxytoca	+	6.32E-01	0.000	0.000	0.000	0.003	0.004
1.1.1.193|g__Klebsiella.s__Klebsiella_pneumoniae	+	5.78E-01	0.000	0.000	0.000	0.002	0.003
1.1.1.193|g__Klebsiella.s__Klebsiella_variicola	+	4.28E-02	0.000	0.000	0.000	0.000	0.001
1.1.1.193|g__Kosakonia.s__Kosakonia_cowanii	-	3.30E-01	0.000	0.000	0.000	0.000	0.000
1.1.1.193|g__Lachnoclostridium.s__Clostridium_aldenense	-	7.14E-01	0.000	0.000	0.000	0.000	0.000
1.1.1.193|g__Lachnoclostridium.s__Clostridium_bolteae	+	8.93E-01	0.000	0.000	0.000	0.001	0.001
1.1.1.193|g__Lachnoclostridium.s__Clostridium_citroniae	+	8.20E-02	0.000	0.000	0.000	0.000	0.000
1.1.1.193|g__Lachnoclostridium.s__Clostridium_clostridioforme	+	4.40E-01	0.000	0.000	0.000	0.000	0.000
1.1.1.193|g__Lachnoclostridium.s__Clostridium_symbiosum	+	7.70E-01	0.000	0.000	0.000	0.000	0.000
1.1.1.193|g__Lachnospira.s__Lachnospira_pectinoschiza	-	4.31E-01	0.000	0.000	0.000	0.002	0.002
1.1.1.193|g__Lactobacillus.s__Lactobacillus_crispatus	-	1.60E-01	0.000	0.000	0.000	0.000	0.000
1.1.1.193|g__Lactobacillus.s__Lactobacillus_fermentum	+	3.30E-01	0.000	0.000	0.000	0.000	0.000
1.1.1.193|g__Lactobacillus.s__Lactobacillus_rogosae	-	4.24E-01	0.000	0.000	0.000	0.002	0.002
1.1.1.193|g__Listeria.s__Listeria_monocytogenes	+	3.30E-01	0.000	0.000	0.000	0.000	0.000
1.1.1.193|g__Megamonas.s__Megamonas_funiformis	+	1.00E+00	0.000	0.000	0.000	0.002	0.001

	1.1.1.193|g__Megamonas.s__Megamonas_hypermegale	+	8.28E-01	0.000	0.000	0.000	0.001	0.001
	1.1.1.193|g__Megamonas.s__Megamonas_rupellensis	+	7.86E-01	0.000	0.000	0.000	0.002	0.001
	1.1.1.193|g__Megasphaera.s__Megasphaera_micronuciformis	-	1.00E+00	0.000	0.000	0.000	0.000	0.000
	1.1.1.193|g__Mesosutterella.s__Mesosutterella_multiformis	-	7.37E-01	0.000	0.000	0.000	0.003	0.004
	1.1.1.193|g__Oxalobacter.s__Oxalobacter_formigenes	+	6.73E-01	0.000	0.000	0.000	0.000	0.001
	1.1.1.193|g__Parabacteroides.s__Parabacteroides_goldsteinii	+	1.22E-01	0.000	0.000	0.000	0.001	0.005
	1.1.1.193|g__Parabacteroides.s__Parabacteroides_gordonii	+	9.83E-01	0.000	0.000	0.000	0.000	0.001
	1.1.1.193|g__Parabacteroides.s__Parabacteroides_johnsonii	+	6.24E-02	0.000	0.000	0.000	0.001	0.002
	1.1.1.193|g__Paraprevotella.s__Paraprevotella_clara	+	1.06E-02	0.000	0.000	0.000	0.001	0.005
	1.1.1.193|g__Paraprevotella.s__Paraprevotella_xylaniphila	+	9.70E-01	0.000	0.000	0.000	0.000	0.001
	1.1.1.193|g__Parasutterella.s__Parasutterella_excrementihominis	-	5.95E-01	0.000	0.000	0.000	0.002	0.002
	1.1.1.193|g__Phascolarctobacterium.s__Phascolarctobacterium_succinatuter	+	2.97E-01	0.000	0.000	0.000	0.001	0.002
	1.1.1.193|g__Prevotella.s__Prevotella_denticola	+	3.30E-01	0.000	0.000	0.000	0.000	0.000
	1.1.1.193|g__Prevotella.s__Prevotella_disiens	+	3.30E-01	0.000	0.000	0.000	0.000	0.000
	1.1.1.193|g__Prevotella.s__Prevotella_sp_109	-	1.60E-01	0.000	0.000	0.000	0.001	0.000
	1.1.1.193|g__Prevotella.s__Prevotella_sp_AM42_24	-	1.60E-01	0.000	0.000	0.000	0.003	0.000
	1.1.1.193|g__Propionibacterium.s__Propionibacterium_acidifaciens	+	3.30E-01	0.000	0.000	0.000	0.000	0.000
	1.1.1.193|g__Proteus.s__Proteus_mirabilis	+	3.30E-01	0.000	0.000	0.000	0.000	0.000
	1.1.1.193|g__Pseudocitrobacter.s__Pseudocitrobacter_faecalis	+	3.30E-01	0.000	0.000	0.000	0.000	0.000
	1.1.1.193|g__Pyramidobacter.s__Pyramidobacter_piscolens	+	2.92E-02	0.000	0.000	0.000	0.000	0.000
	1.1.1.193|g__Pyramidobacter.s__Pyramidobacter_sp_C12_8	+	1.60E-01	0.000	0.000	0.000	0.000	0.000
	1.1.1.193|g__Roseburia.s__Roseburia_faecis	+	7.83E-01	0.000	0.000	0.000	0.001	0.002
	1.1.1.193|g__Roseburia.s__Roseburia_intestinalis	+	4.28E-02	0.000	0.000	0.000	0.000	0.000
	1.1.1.193|g__Roseburia.s__Roseburia_inulinivorans	+	7.14E-01	0.000	0.000	0.000	0.001	0.001
	1.1.1.193|g__Ruminococcaceae_unclassified.s__Ruminococcaceae_bacteriu	-	3.30E-01	0.000	0.000	0.000	0.000	0.000
	1.1.1.193|g__Ruminococcaceae_unclassified.s__Ruminococcaceae_bacteriu	+	8.79E-02	0.000	0.000	0.000	0.000	0.001
	1.1.1.193|g__Ruminococcus.s__Ruminococcus_callidus	+	3.26E-01	0.000	0.000	0.000	0.000	0.001
	1.1.1.193|g__Ruminococcus.s__Ruminococcus_sp_AF31_8BH	-	8.26E-01	0.000	0.000	0.000	0.000	0.000
	1.1.1.193|g__Salmonella.s__Salmonella_enterica	+	5.89E-01	0.000	0.000	0.000	0.000	0.000
	1.1.1.193|g__Sanguibacteroides.s__Sanguibacteroides_justesenii	+	9.70E-01	0.000	0.000	0.000	0.000	0.000
	1.1.1.193|g__Slackia.s__Slackia_isoflavoniconvertens	+	1.60E-01	0.000	0.000	0.000	0.000	0.001
	1.1.1.193|g__Succinatimonas.s__Succinatimonas_hippei	-	3.30E-01	0.000	0.000	0.000	0.000	0.000
	1.1.1.193|g__Sutterella.s__Sutterella_wadsworthensis	+	2.59E-01	0.000	0.000	0.000	0.001	0.001
	1.1.1.193|g__Synergistes.s__Synergistes_jonesii	+	3.30E-01	0.000	0.000	0.000	0.000	0.000
	1.1.1.193|g__Turicimonas.s__Turicimonas_muris	+	5.49E-01	0.000	0.000	0.000	0.000	0.000
	1.1.1.193|g__Veillonella.s__Veillonella_atypica	+	7.79E-02	0.000	0.000	0.000	0.000	0.000
	1.1.1.193|g__Veillonella.s__Veillonella_dispar	+	5.18E-01	0.000	0.000	0.000	0.004	0.001
	1.1.1.193|g__Veillonella.s__Veillonella_infantium	-	8.03E-01	0.000	0.000	0.000	0.000	0.000
	1.1.1.193|g__Veillonella.s__Veillonella_parvula	+	4.08E-01	0.000	0.000	0.000	0.003	0.001
	1.1.1.193|g__Veillonella.s__Veillonella_rogosae	+	1.05E-01	0.000	0.000	0.000	0.001	0.000
	1.1.1.193|g__Veillonella.s__Veillonella_tobetsuensis	+	4.17E-01	0.000	0.000	0.000	0.000	0.000
	1.1.1.193|g__Victivallales_unclassified.s__Victivallales_bacterium_CCUG_	+	5.49E-01	0.000	0.000	0.000	0.000	0.001
	1.1.1.193|g__Victivallis.s__Victivallis_vadensis	+	5.69E-01	0.000	0.000	0.000	0.000	0.000
	1.1.1.193|g__Bacteroides.s__Bacteroides_nordii	-	7.12E-01	0.102	0.121	0.020	0.005	0.006
	1.1.1.193|g__Blautia.s__Blautia_obeum	+	1.44E-01	0.111	0.131	0.020	0.002	0.003
	1.1.1.193|g__Dorea.s__Dorea_longicatena	-	9.51E-01	0.000	0.031	0.031	0.001	0.001
	1.1.1.193|g__Roseburia.s__Roseburia_hominis	+	1.36E-01	0.172	0.216	0.044	0.004	0.006
	1.1.1.193|g__Blautia.s__Ruminococcus_torques	+	3.06E-01	0.190	0.235	0.046	0.003	0.005
	1.1.1.193|g__Bilophila.s__Bilophila_wadsworthia	+	1.92E-01	0.033	0.098	0.065	0.001	0.002
	1.1.1.193|g__Lachnospiraceae_unclassified.s__Eubacterium_rectale	+	9.64E-01	0.093	0.196	0.104	0.005	0.005
	1.1.1.193|g__Bacteroides.s__Bacteroides_thetaiotaomicron	-	9.54E-01	1.439	1.581	0.143	0.025	0.038
	1.1.1.193|g__Phascolarctobacterium.s__Phascolarctobacterium_faecium	+	5.68E-01	0.630	0.808	0.178	0.014	0.019
	1.1.1.193|g__Odoribacter.s__Odoribacter_splanchnicus	-	8.65E-01	0.072	0.270	0.197	0.005	0.005
	1.1.1.193|g__Eubacterium.s__Eubacterium_eligens	+	5.32E-02	0.276	0.551	0.274	0.006	0.021
	1.1.1.193|g__Parabacteroides.s__Parabacteroides_merdae	+	2.13E-01	0.041	0.423	0.382	0.009	0.015
	1.1.1.193|unclassified	+	9.69E-02	3.780	4.197	0.417	0.040	0.068
	1.1.1.193|g__Bacteroides.s__Bacteroides_stercoris	+	9.01E-01	0.385	0.803	0.418	0.061	0.039
	1.1.1.193|g__Bacteroides.s__Bacteroides_dorei	+	4.67E-01	0.141	0.618	0.478	0.053	0.039
	1.1.1.193|g__Bacteroides.s__Bacteroides_caccae	+	3.04E-01	0.235	0.935	0.700	0.016	0.025
	1.1.1.193|g__Alistipes.s__Alistipes_shahii	+	1.53E-02	0.090	1.002	0.913	0.009	0.023
	1.1.1.193|g__Bacteroides.s__Bacteroides_intestinalis	+	3.47E-02	0.239	1.174	0.935	0.007	0.023
	1.1.1.193|g__Bacteroides.s__Bacteroides_uniformis	+	2.24E-01	6.212	9.553	3.341	0.076	0.127
China2	1.1.1.193	-	3.88E-03	136.999	123.124	-13.875	1.000	1.000
	1.1.1.193|g__Bacteroides.s__Bacteroides_vulgatus	-	7.77E-02	12.107	6.396	-5.711	0.160	0.098
	1.1.1.193|g__Faecalibacterium.s__Faecalibacterium_prausnitzii	-	2.59E-01	6.772	4.660	-2.113	0.062	0.056
	1.1.1.193|g__Phascolarctobacterium.s__Phascolarctobacterium_faecium	-	3.99E-01	0.816	0.293	-0.523	0.009	0.010
	1.1.1.193|g__Bacteroides.s__Bacteroides_xylanisolvens	-	1.16E-01	0.641	0.157	-0.484	0.017	0.010
	1.1.1.193|g__Bacteroides.s__Bacteroides_dorei	-	1.71E-01	0.883	0.496	-0.387	0.052	0.032
	1.1.1.193|g__Clostridium.s__Clostridium_sp_AM22_11AC	-	2.34E-01	0.555	0.307	-0.248	0.007	0.006
	1.1.1.193|g__Parabacteroides.s__Parabacteroides_distasonis	+	5.22E-01	1.182	0.984	-0.197	0.011	0.022
	1.1.1.193|g__Blautia.s__Blautia_wexlerae	-	9.24E-01	0.409	0.268	-0.141	0.008	0.012
	1.1.1.193|g__Bacteroides.s__Bacteroides_thetaiotaomicron	-	2.07E-01	0.469	0.361	-0.108	0.011	0.009

1.1.1.193|g__Lachnospiraceae_unclassified.s__Eubacterium_rectale	-	7.43E-01	0.358	0.302	-0.056	0.008	0.007
1.1.1.193|g__Blautia.s__Blautia_obeum	-	6.75E-01	0.436	0.386	-0.050	0.005	0.008
1.1.1.193|g__Haemophilus.s__Haemophilus_parainfluenzae	-	1.93E-01	0.038	0.000	-0.038	0.002	0.002
1.1.1.193|g__Agathobaculum.s__Agathobaculum_butyriciproducens	-	1.74E-01	0.037	0.000	-0.037	0.001	0.001
1.1.1.193|g__Veillonella.s__Veillonella_parvula	-	4.78E-01	0.030	0.000	-0.030	0.001	0.001
1.1.1.193|g__Bacteroides.s__Bacteroides_cellulosilyticus	-	9.16E-01	0.038	0.012	-0.026	0.005	0.008
1.1.1.193|g__Escherichia.s__Escherichia_coli	+	5.47E-01	0.124	0.120	-0.004	0.004	0.004
1.1.1.193|g__Acidaminococcus.s__Acidaminococcus_intestini	-	5.69E-01	0.000	0.000	0.000	0.001	0.001
1.1.1.193|g__Adlercreutzia.s__Adlercreutzia_equolifaciens	+	3.58E-01	0.000	0.000	0.000	0.000	0.000
1.1.1.193|g__Aggregatibacter.s__Aggregatibacter_segnis	-	3.30E-01	0.000	0.000	0.000	0.000	0.000
1.1.1.193|g__Akkermansia.s__Akkermansia_muciniphila	+	4.63E-01	0.000	0.000	0.000	0.002	0.010
1.1.1.193|g__Alcaligenes.s__Alcaligenes_faecalis	+	3.30E-01	0.000	0.000	0.000	0.000	0.002
1.1.1.193|g__Allisonella.s__Allisonella_histaminiformans	+	8.01E-01	0.000	0.000	0.000	0.000	0.000
1.1.1.193|g__Anaeromassilibacillus.s__Anaeromassilibacillus_sp_An250	+	3.08E-01	0.000	0.000	0.000	0.000	0.000
1.1.1.193|g__Anaerotignum.s__Anaerotignum_lactatifermentans	-	1.60E-01	0.000	0.000	0.000	0.000	0.000
1.1.1.193|g__Asaccharobacter.s__Asaccharobacter_celatus	+	3.69E-01	0.000	0.000	0.000	0.000	0.000
1.1.1.193|g__Atlantibacter.s__Atlantibacter_hermannii	-	3.30E-01	0.000	0.000	0.000	0.000	0.000
1.1.1.193|g__Bacteroides.s__Bacteroides_clarus	-	4.15E-01	0.000	0.000	0.000	0.001	0.005
1.1.1.193|g__Bacteroides.s__Bacteroides_coprocola	-	1.31E-01	0.000	0.000	0.000	0.027	0.009
1.1.1.193|g__Bacteroides.s__Bacteroides_coprophilus	+	2.85E-01	0.000	0.000	0.000	0.004	0.005
1.1.1.193|g__Bacteroides.s__Bacteroides_eggerthii	-	6.95E-01	0.000	0.000	0.000	0.008	0.003
1.1.1.193|g__Bacteroides.s__Bacteroides_faecis	+	3.93E-01	0.000	0.000	0.000	0.000	0.002
1.1.1.193|g__Bacteroides.s__Bacteroides_finegoldii	-	1.06E-01	0.000	0.000	0.000	0.009	0.002
1.1.1.193|g__Bacteroides.s__Bacteroides_massiliensis	-	6.03E-01	0.000	0.000	0.000	0.019	0.005
1.1.1.193|g__Bacteroides.s__Bacteroides_nordii	+	5.85E-02	0.000	0.000	0.000	0.001	0.002
1.1.1.193|g__Bacteroides.s__Bacteroides_oleiciplenus	-	1.60E-01	0.000	0.000	0.000	0.000	0.000
1.1.1.193|g__Bacteroides.s__Bacteroides_plebeius	-	3.42E-01	0.000	0.000	0.000	0.092	0.036
1.1.1.193|g__Bacteroides.s__Bacteroides_salyersiae	-	5.38E-01	0.000	0.000	0.000	0.001	0.001
1.1.1.193|g__Bacteroides.s__Bacteroides_sartorii	-	3.30E-01	0.000	0.000	0.000	0.000	0.000
1.1.1.193|g__Bacteroides.s__Bacteroides_stercorirosoris	+	5.89E-01	0.000	0.000	0.000	0.000	0.000
1.1.1.193|g__Barnesiella.s__Barnesiella_intestinihominis	+	6.65E-01	0.000	0.000	0.000	0.003	0.006
1.1.1.193|g__Blautia.s__Blautia_hansenii	-	1.75E-01	0.000	0.000	0.000	0.001	0.001
1.1.1.193|g__Blautia.s__Blautia_sp_AF19_10LB	+	3.12E-01	0.000	0.000	0.000	0.002	0.004
1.1.1.193|g__Butyricimonas.s__Butyricimonas_synergistica	+	1.00E+00	0.000	0.000	0.000	0.000	0.000
1.1.1.193|g__Butyricimonas.s__Butyricimonas_virosa	+	2.19E-01	0.000	0.000	0.000	0.001	0.002
1.1.1.193|g__Butyrivibrio.s__Butyrivibrio_crossotus	+	3.30E-01	0.000	0.000	0.000	0.000	0.006
1.1.1.193|g__Campylobacter.s__Campylobacter_hominis	+	1.60E-01	0.000	0.000	0.000	0.000	0.000
1.1.1.193|g__Catenibacterium.s__Catenibacterium_mitsuokai	+	5.49E-01	0.000	0.000	0.000	0.000	0.000
1.1.1.193|g__Cetobacterium.s__Cetobacterium_somerae	-	3.30E-01	0.000	0.000	0.000	0.000	0.000
1.1.1.193|g__Chryseobacterium.s__Chryseobacterium_sp_VAUSW3	+	3.30E-01	0.000	0.000	0.000	0.000	0.000
1.1.1.193|g__Chryseobacterium.s__Chryseobacterium_sp_YLOS41	+	3.30E-01	0.000	0.000	0.000	0.000	0.000
1.1.1.193|g__Citrobacter.s__Citrobacter_amalonaticus	-	5.89E-01	0.000	0.000	0.000	0.000	0.000
1.1.1.193|g__Citrobacter.s__Citrobacter_braakii	+	1.60E-01	0.000	0.000	0.000	0.000	0.000
1.1.1.193|g__Citrobacter.s__Citrobacter_freundii	+	6.87E-01	0.000	0.000	0.000	0.000	0.001
1.1.1.193|g__Citrobacter.s__Citrobacter_portucalensis	-	6.92E-01	0.000	0.000	0.000	0.000	0.004
1.1.1.193|g__Citrobacter.s__Citrobacter_werkmanii	+	1.00E+00	0.000	0.000	0.000	0.000	0.000
1.1.1.193|g__Citrobacter.s__Citrobacter_youngae	-	7.92E-01	0.000	0.000	0.000	0.000	0.003
1.1.1.193|g__Cloacibacillus.s__Cloacibacillus_porcorum	+	3.30E-01	0.000	0.000	0.000	0.000	0.000
1.1.1.193|g__Clostridioides.s__Clostridioides_difficile	+	7.46E-02	0.000	0.000	0.000	0.000	0.001
1.1.1.193|g__Clostridium.s__Clostridium_butyricum	+	3.30E-01	0.000	0.000	0.000	0.000	0.000
1.1.1.193|g__Clostridium.s__Clostridium_celatum	+	3.30E-01	0.000	0.000	0.000	0.000	0.000
1.1.1.193|g__Clostridium.s__Clostridium_disporicum	-	5.61E-02	0.000	0.000	0.000	0.001	0.000
1.1.1.193|g__Clostridium.s__Clostridium_perfringens	-	8.19E-02	0.000	0.000	0.000	0.000	0.000
1.1.1.193|g__Clostridium.s__Clostridium_sp_AF36_4	+	8.93E-02	0.000	0.000	0.000	0.000	0.001
1.1.1.193|g__Comamonas.s__Comamonas_kerstersii	-	1.00E+00	0.000	0.000	0.000	0.000	0.000
1.1.1.193|g__Comamonas.s__Comamonas_testosteroni	+	3.30E-01	0.000	0.000	0.000	0.000	0.000
1.1.1.193|g__Comamonas.s__Comamonas_thiooxydans	+	3.30E-01	0.000	0.000	0.000	0.000	0.000
1.1.1.193|g__Coprobacillus.s__Coprobacillus_cateniformis	+	2.06E-02	0.000	0.000	0.000	0.000	0.002
1.1.1.193|g__Coprobacter.s__Coprobacter_fastidiosus	+	9.14E-01	0.000	0.000	0.000	0.001	0.001
1.1.1.193|g__Coprobacter.s__Coprobacter_secundus	-	6.87E-01	0.000	0.000	0.000	0.000	0.000
1.1.1.193|g__Coprococcus.s__Coprococcus_catus	+	2.99E-01	0.000	0.000	0.000	0.000	0.001
1.1.1.193|g__Coprococcus.s__Coprococcus_comes	-	9.91E-01	0.000	0.000	0.000	0.001	0.001
1.1.1.193|g__Coprococcus.s__Coprococcus_eutactus	+	1.23E-01	0.000	0.000	0.000	0.003	0.005
1.1.1.193|g__Desulfovibrio.s__Desulfovibrio_fairfieldensis	-	5.89E-01	0.000	0.000	0.000	0.000	0.000
1.1.1.193|g__Desulfovibrio.s__Desulfovibrio_piger	+	1.85E-01	0.000	0.000	0.000	0.000	0.001
1.1.1.193|g__Desulfovibrio.s__Desulfovibrio_sp_AM18_2	+	8.19E-02	0.000	0.000	0.000	0.000	0.000
1.1.1.193|g__Desulfovibrionaceae_unclassified.s__Desulfovibrionaceae_bac	+	8.19E-02	0.000	0.000	0.000	0.000	0.000
1.1.1.193|g__Dialister.s__Dialister_pneumosintes	+	3.30E-01	0.000	0.000	0.000	0.000	0.000
1.1.1.193|g__Dialister.s__Dialister_succinatiphilus	+	9.90E-01	0.000	0.000	0.000	0.002	0.002
1.1.1.193|g__Dorea.s__Dorea_sp_OM02_2LB	+	4.30E-01	0.000	0.000	0.000	0.000	0.000
1.1.1.193|g__Eggerthella.s__Eggerthella_lenta	+	5.80E-01	0.000	0.000	0.000	0.001	0.002
1.1.1.193|g__Enterobacter.s__Enterobacter_bugandensis	-	4.94E-01	0.000	0.000	0.000	0.000	0.000

1.1.1.193|g__Enterobacter.s__Enterobacter_cloacae	+	8.18E-01	0.000	0.000	0.000	0.000	0.002
1.1.1.193|g__Enterobacter.s__Enterobacter_mori	-	1.60E-01	0.000	0.000	0.000	0.000	0.000
1.1.1.193|g__Enterococcus.s__Enterococcus_hirae	+	3.30E-01	0.000	0.000	0.000	0.000	0.000
1.1.1.193|g__Erysipelatoclostridium.s__Clostridium_innocuum	+	7.46E-02	0.000	0.000	0.000	0.000	0.001
1.1.1.193|g__Erysipelatoclostridium.s__Clostridium_spiroforme	+	3.20E-01	0.000	0.000	0.000	0.000	0.001
1.1.1.193|g__Erysipelotrichaceae_unclassified.s__Erysipelotrichaceae_bacte	-	1.60E-01	0.000	0.000	0.000	0.000	0.000
1.1.1.193|g__Escherichia.s__Escherichia_fergusonii	+	7.00E-01	0.000	0.000	0.000	0.000	0.000
1.1.1.193|g__Eubacterium.s__Eubacterium_ramulus	+	2.48E-01	0.000	0.000	0.000	0.000	0.001
1.1.1.193|g__Eubacterium.s__Eubacterium_sp_AF17_7	+	9.90E-01	0.000	0.000	0.000	0.000	0.000
1.1.1.193|g__Eubacterium.s__Eubacterium_sp_AM18_10LB_B	-	4.05E-01	0.000	0.000	0.000	0.000	0.000
1.1.1.193|g__Faecalicatena.s__Faecalicatena_contorta	+	2.59E-01	0.000	0.000	0.000	0.000	0.000
1.1.1.193|g__Firmicutes_unclassified.s__Firmicutes_bacterium_AM10_47	-	6.29E-01	0.000	0.000	0.000	0.001	0.000
1.1.1.193|g__Flavonifractor.s__Flavonifractor_plautii	+	5.49E-01	0.000	0.000	0.000	0.000	0.000
1.1.1.193|g__Fusobacterium.s__Fusobacterium_mortiferum	-	6.89E-01	0.000	0.000	0.000	0.001	0.003
1.1.1.193|g__Fusobacterium.s__Fusobacterium_nucleatum	+	1.00E+00	0.000	0.000	0.000	0.000	0.000
1.1.1.193|g__Fusobacterium.s__Fusobacterium_periodonticum	-	1.58E-01	0.000	0.000	0.000	0.000	0.000
1.1.1.193|g__Fusobacterium.s__Fusobacterium_ulcerans	+	9.90E-01	0.000	0.000	0.000	0.000	0.000
1.1.1.193|g__Fusobacterium.s__Fusobacterium_varium	-	1.60E-01	0.000	0.000	0.000	0.000	0.000
1.1.1.193|g__Haemophilus.s__Haemophilus_haemolyticus	+	3.30E-01	0.000	0.000	0.000	0.000	0.000
1.1.1.193|g__Haemophilus.s__Haemophilus_influenzae	+	3.30E-01	0.000	0.000	0.000	0.000	0.000
1.1.1.193|g__Haemophilus.s__Haemophilus_paraphrohaemolyticus	-	3.30E-01	0.000	0.000	0.000	0.000	0.000
1.1.1.193|g__Holdemanella.s__Holdemanella_biformis	+	5.07E-01	0.000	0.000	0.000	0.000	0.000
1.1.1.193|g__Intestinibacter.s__Intestinibacter_bartlettii	+	7.82E-01	0.000	0.000	0.000	0.000	0.000
1.1.1.193|g__Klebsiella.s__Klebsiella_aerogenes	-	5.68E-01	0.000	0.000	0.000	0.000	0.001
1.1.1.193|g__Klebsiella.s__Klebsiella_grimontii	+	3.30E-01	0.000	0.000	0.000	0.000	0.000
1.1.1.193|g__Klebsiella.s__Klebsiella_michiganensis	-	7.06E-01	0.000	0.000	0.000	0.000	0.000
1.1.1.193|g__Klebsiella.s__Klebsiella_variicola	+	1.94E-01	0.000	0.000	0.000	0.002	0.002
1.1.1.193|g__Kluyvera.s__Kluyvera_ascorbata	+	3.30E-01	0.000	0.000	0.000	0.000	0.000
1.1.1.193|g__Kluyvera.s__Kluyvera_cryocrescens	+	3.30E-01	0.000	0.000	0.000	0.000	0.000
1.1.1.193|g__Kluyvera.s__Kluyvera_georgiana	-	3.30E-01	0.000	0.000	0.000	0.000	0.000
1.1.1.193|g__Lachnoclostridium.s__Clostridium_aldenense	+	9.13E-02	0.000	0.000	0.000	0.000	0.001
1.1.1.193|g__Lachnoclostridium.s__Clostridium_citroniae	+	7.81E-03	0.000	0.000	0.000	0.000	0.001
1.1.1.193|g__Lachnoclostridium.s__Clostridium_clostridioforme	-	8.22E-01	0.000	0.000	0.000	0.001	0.000
1.1.1.193|g__Lachnoclostridium.s__Clostridium_symbiosum	+	5.34E-01	0.000	0.000	0.000	0.000	0.000
1.1.1.193|g__Lachnospira.s__Lachnospira_pectinoschiza	-	9.52E-01	0.000	0.000	0.000	0.006	0.004
1.1.1.193|g__Lactobacillus.s__Lactobacillus_amylovorus	+	3.30E-01	0.000	0.000	0.000	0.000	0.000
1.1.1.193|g__Lactobacillus.s__Lactobacillus_fermentum	+	9.90E-01	0.000	0.000	0.000	0.000	0.000
1.1.1.193|g__Lactobacillus.s__Lactobacillus_paralimentarius	-	3.30E-01	0.000	0.000	0.000	0.000	0.000
1.1.1.193|g__Lactobacillus.s__Lactobacillus_rogosae	-	8.56E-01	0.000	0.000	0.000	0.006	0.004
1.1.1.193|g__Lactobacillus.s__Lactobacillus_zymae	-	3.30E-01	0.000	0.000	0.000	0.000	0.000
1.1.1.193|g__Leclercia.s__Leclercia_adecarboxylata	-	3.03E-01	0.000	0.000	0.000	0.000	0.000
1.1.1.193|g__Lelliottia.s__Lelliottia_amnigena	+	3.30E-01	0.000	0.000	0.000	0.000	0.000
1.1.1.193|g__Lelliottia.s__Lelliottia_nimipressuralis	-	3.24E-01	0.000	0.000	0.000	0.000	0.001
1.1.1.193|g__Megamonas.s__Megamonas_funiformis	+	9.41E-01	0.000	0.000	0.000	0.011	0.006
1.1.1.193|g__Megamonas.s__Megamonas_hypermegale	-	9.82E-01	0.000	0.000	0.000	0.009	0.004
1.1.1.193|g__Megamonas.s__Megamonas_rupellensis	+	9.41E-01	0.000	0.000	0.000	0.009	0.004
1.1.1.193|g__Megasphaera.s__Megasphaera_elsdenii	+	3.30E-01	0.000	0.000	0.000	0.000	0.000
1.1.1.193|g__Megasphaera.s__Megasphaera_micronuciformis	+	1.72E-01	0.000	0.000	0.000	0.000	0.000
1.1.1.193|g__Megasphaera.s__Megasphaera_stantonii	+	3.30E-01	0.000	0.000	0.000	0.000	0.000
1.1.1.193|g__Mesosutterella.s__Mesosutterella_multiformis	-	5.55E-01	0.000	0.000	0.000	0.002	0.001
1.1.1.193|g__Mitsuokella.s__Mitsuokella_jalaludinii	+	3.30E-01	0.000	0.000	0.000	0.000	0.000
1.1.1.193|g__Mitsuokella.s__Mitsuokella_multacida	+	1.60E-01	0.000	0.000	0.000	0.000	0.001
1.1.1.193|g__Morganella.s__Morganella_morganii	+	3.30E-01	0.000	0.000	0.000	0.000	0.004
1.1.1.193|g__Oxalobacter.s__Oxalobacter_formigenes	+	2.64E-02	0.000	0.000	0.000	0.000	0.000
1.1.1.193|g__Pantoea.s__Pantoea_dispersa	+	3.30E-01	0.000	0.000	0.000	0.000	0.000
1.1.1.193|g__Pantoea.s__Pantoea_sesami	-	7.94E-01	0.000	0.000	0.000	0.000	0.000
1.1.1.193|g__Parabacteroides.s__Parabacteroides_chinchillae	+	3.30E-01	0.000	0.000	0.000	0.000	0.000
1.1.1.193|g__Parabacteroides.s__Parabacteroides_goldsteinii	+	2.24E-01	0.000	0.000	0.000	0.000	0.002
1.1.1.193|g__Parabacteroides.s__Parabacteroides_gordonii	+	1.60E-01	0.000	0.000	0.000	0.000	0.001
1.1.1.193|g__Parabacteroides.s__Parabacteroides_johnsonii	+	6.79E-01	0.000	0.000	0.000	0.001	0.002
1.1.1.193|g__Paraprevotella.s__Paraprevotella_clara	-	8.92E-01	0.000	0.000	0.000	0.003	0.002
1.1.1.193|g__Paraprevotella.s__Paraprevotella_xylaniphila	-	3.30E-01	0.000	0.000	0.000	0.000	0.000
1.1.1.193|g__Parasutterella.s__Parasutterella_excrementihominis	+	6.17E-01	0.000	0.000	0.000	0.003	0.002
1.1.1.193|g__Pedobacter.s__Pedobacter_himalayensis	-	8.04E-01	0.000	0.000	0.000	0.000	0.000
1.1.1.193|g__Phascolarctobacterium.s__Phascolarctobacterium_succinatuter	+	9.77E-01	0.000	0.000	0.000	0.002	0.002
1.1.1.193|g__Prevotella.s__Prevotella_copri	+	8.81E-01	0.000	0.000	0.000	0.110	0.165
1.1.1.193|g__Prevotella.s__Prevotella_corporis	+	1.60E-01	0.000	0.000	0.000	0.000	0.000
1.1.1.193|g__Prevotella.s__Prevotella_sp_109	-	1.00E+00	0.000	0.000	0.000	0.003	0.003
1.1.1.193|g__Prevotella.s__Prevotella_sp_AM42_24	-	9.90E-01	0.000	0.000	0.000	0.005	0.002
1.1.1.193|g__Proteus.s__Proteus_hauseri	-	1.00E+00	0.000	0.000	0.000	0.000	0.000
1.1.1.193|g__Proteus.s__Proteus_mirabilis	-	5.69E-01	0.000	0.000	0.000	0.000	0.000
1.1.1.193|g__Proteus.s__Proteus_penneri	-	1.00E+00	0.000	0.000	0.000	0.000	0.000

	1.1.1.193|g__Proteus.s__Proteus_vulgaris	+	1.00E+00	0.000	0.000	0.000	0.000	0.000
	1.1.1.193|g__Providencia.s__Providencia_rettgeri	+	3.30E-01	0.000	0.000	0.000	0.000	0.005
	1.1.1.193|g__Pyramidobacter.s__Pyramidobacter_piscolens	+	2.96E-01	0.000	0.000	0.000	0.000	0.000
	1.1.1.193|g__Pyramidobacter.s__Pyramidobacter_sp_C12_8	+	3.30E-01	0.000	0.000	0.000	0.000	0.000
	1.1.1.193|g__Raoultella.s__Raoultella_ornithinolytica	+	5.69E-01	0.000	0.000	0.000	0.000	0.000
	1.1.1.193|g__Raoultella.s__Raoultella_planticola	+	1.00E+00	0.000	0.000	0.000	0.000	0.000
	1.1.1.193|g__Roseburia.s__Roseburia_faecis	-	8.93E-02	0.000	0.000	0.000	0.006	0.002
	1.1.1.193|g__Roseburia.s__Roseburia_intestinalis	-	4.98E-01	0.000	0.000	0.000	0.001	0.001
	1.1.1.193|g__Roseburia.s__Roseburia_inulinivorans	-	7.12E-01	0.000	0.000	0.000	0.001	0.001
	1.1.1.193|g__Ruminococcaceae_unclassified.s__Ruminococcaceae_bacteriu	+	5.89E-01	0.000	0.000	0.000	0.000	0.000
	1.1.1.193|g__Ruminococcaceae_unclassified.s__Ruminococcaceae_bacteriu	+	2.24E-02	0.000	0.000	0.000	0.000	0.000
	1.1.1.193|g__Ruminococcus.s__Ruminococcus_callidus	+	6.10E-01	0.000	0.000	0.000	0.001	0.001
	1.1.1.193|g__Ruminococcus.s__Ruminococcus_sp_AF31_8BH	-	6.77E-01	0.000	0.000	0.000	0.001	0.001
	1.1.1.193|g__Salmonella.s__Salmonella_enterica	-	7.19E-01	0.000	0.000	0.000	0.000	0.000
	1.1.1.193|g__Staphylococcus.s__Staphylococcus_epidermidis	-	3.30E-01	0.000	0.000	0.000	0.000	0.000
	1.1.1.193|g__Staphylococcus.s__Staphylococcus_pasteuri	-	3.30E-01	0.000	0.000	0.000	0.000	0.000
	1.1.1.193|g__Staphylococcus.s__Staphylococcus_warneri	-	3.30E-01	0.000	0.000	0.000	0.000	0.000
	1.1.1.193|g__Streptococcus.s__Streptococcus_equinus	-	5.69E-01	0.000	0.000	0.000	0.000	0.000
	1.1.1.193|g__Streptococcus.s__Streptococcus_gallolyticus	+	3.30E-01	0.000	0.000	0.000	0.000	0.000
	1.1.1.193|g__Streptococcus.s__Streptococcus_infantarius	-	3.30E-01	0.000	0.000	0.000	0.000	0.000
	1.1.1.193|g__Streptococcus.s__Streptococcus_macedonicus	-	3.30E-01	0.000	0.000	0.000	0.000	0.000
	1.1.1.193|g__Streptococcus.s__Streptococcus_mitis	-	1.60E-01	0.000	0.000	0.000	0.000	0.000
	1.1.1.193|g__Streptococcus.s__Streptococcus_oralis	-	1.60E-01	0.000	0.000	0.000	0.000	0.000
	1.1.1.193|g__Streptococcus.s__Streptococcus_pasteurianus	+	1.00E+00	0.000	0.000	0.000	0.000	0.000
	1.1.1.193|g__Streptococcus.s__Streptococcus_pneumoniae	-	3.93E-01	0.000	0.000	0.000	0.000	0.000
	1.1.1.193|g__Streptococcus.s__Streptococcus_pseudopneumoniae	-	1.60E-01	0.000	0.000	0.000	0.000	0.000
	1.1.1.193|g__Sutterella.s__Sutterella_wadsworthensis	+	7.06E-01	0.000	0.000	0.000	0.001	0.000
	1.1.1.193|g__Veillonella.s__Veillonella_atypica	+	9.40E-01	0.000	0.000	0.000	0.000	0.000
	1.1.1.193|g__Veillonella.s__Veillonella_dispar	-	8.00E-01	0.000	0.000	0.000	0.000	0.000
	1.1.1.193|g__Veillonella.s__Veillonella_infantium	-	7.91E-01	0.000	0.000	0.000	0.000	0.001
	1.1.1.193|g__Veillonella.s__Veillonella_rogosae	-	1.01E-02	0.000	0.000	0.000	0.001	0.000
	1.1.1.193|g__Veillonella.s__Veillonella_tobetsuensis	-	1.91E-01	0.000	0.000	0.000	0.001	0.000
	1.1.1.193|g__Victivallales_unclassified.s__Victivallales_bacterium_CCUG_	+	6.10E-02	0.000	0.000	0.000	0.000	0.001
	1.1.1.193|g__Victivallis.s__Victivallis_vadensis	+	2.68E-01	0.000	0.000	0.000	0.000	0.001
	1.1.1.193|g__Weissella.s__Weissella_confusa	-	1.00E+00	0.000	0.000	0.000	0.000	0.000
	1.1.1.193|g__Yokenella.s__Yokenella_regensburgei	-	3.48E-01	0.000	0.000	0.000	0.000	0.000
	1.1.1.193|g__Dorea.s__Dorea_formicigenerans	-	6.06E-01	0.056	0.058	0.002	0.001	0.001
	1.1.1.193|g__Roseburia.s__Roseburia_hominis	+	5.99E-01	0.238	0.257	0.019	0.003	0.005
	1.1.1.193|g__Lachnoclostridium.s__Clostridium_bolteae	+	1.18E-01	0.000	0.028	0.028	0.001	0.001
	1.1.1.193|g__Hungatella.s__Hungatella_hathewayi	+	4.02E-01	0.000	0.029	0.029	0.001	0.001
	1.1.1.193|g__Anaerostipes.s__Anaerostipes_hadrus	-	9.34E-01	0.142	0.174	0.031	0.003	0.004
	1.1.1.193|g__Dorea.s__Dorea_longicatena	+	2.40E-01	0.133	0.167	0.034	0.001	0.002
	1.1.1.193|g__Klebsiella.s__Klebsiella_oxytoca	+	1.13E-01	0.015	0.068	0.053	0.002	0.003
	1.1.1.193|g__Bacteroides.s__Bacteroides_fragilis	-	9.08E-01	0.140	0.238	0.099	0.013	0.016
	1.1.1.193|g__Bacteroides.s__Bacteroides_intestinalis	+	1.76E-01	0.000	0.104	0.104	0.004	0.006
	1.1.1.193|g__Blautia.s__Ruminococcus_torques	+	4.84E-01	0.364	0.494	0.130	0.005	0.007
	1.1.1.193|g__Eubacterium.s__Eubacterium_eligens	+	6.48E-01	0.000	0.240	0.240	0.006	0.004
	1.1.1.193|g__Bilophila.s__Bilophila_wadsworthia	+	9.43E-04	0.094	0.340	0.246	0.001	0.004
	1.1.1.193|g__Odoribacter.s__Odoribacter_splanchnicus	+	1.19E-01	0.143	0.394	0.251	0.002	0.004
	1.1.1.193|g__Alistipes.s__Alistipes_shahii	+	1.29E-01	0.067	0.364	0.297	0.004	0.009
	1.1.1.193|g__Bacteroides.s__Bacteroides_ovatus	+	9.28E-01	0.382	0.698	0.316	0.022	0.019
	1.1.1.193|g__Parabacteroides.s__Parabacteroides_merdae	+	2.02E-01	0.416	0.732	0.317	0.006	0.012
	1.1.1.193|g__Bacteroides.s__Bacteroides_caccae	+	3.73E-01	0.179	0.522	0.343	0.008	0.011
	1.1.1.193|g__Klebsiella.s__Klebsiella_pneumoniae	+	1.89E-01	0.059	0.411	0.353	0.006	0.024
	1.1.1.193|unclassified	+	9.68E-01	8.272	8.730	0.458	0.078	0.094
	1.1.1.193|g__Bacteroides.s__Bacteroides_stercoris	+	8.21E-01	0.415	1.032	0.617	0.043	0.048
	1.1.1.193|g__Bacteroides.s__Bacteroides_uniformis	+	4.72E-01	3.293	4.318	1.024	0.048	0.077
Taiwan	1.1.1.193	-	1.80E-02	122.076	113.372	-8.705	1.000	1.000
	1.1.1.193|g__Bacteroides.s__Bacteroides_vulgatus	-	5.38E-01	7.917	6.050	-1.866	0.093	0.082
	1.1.1.193|g__Bacteroides.s__Bacteroides_plebeius	-	3.80E-01	1.064	0.300	-0.764	0.081	0.050
	1.1.1.193|g__Lachnospiraceae_unclassified.s__Eubacterium_rectale	-	2.96E-02	0.389	0.081	-0.309	0.015	0.008
	1.1.1.193|g__Coprococcus.s__Coprococcus_comes	-	1.58E-01	0.094	0.000	-0.094	0.002	0.002
	1.1.1.193|g__Escherichia.s__Escherichia_coli	-	7.02E-02	0.052	0.000	-0.052	0.003	0.002
	1.1.1.193|g__Bacteroides.s__Bacteroides_xylanisolvens	+	6.16E-01	0.486	0.443	-0.044	0.016	0.017
	1.1.1.193|g__Blautia.s__Blautia_wexlerae	-	6.53E-01	0.067	0.047	-0.019	0.001	0.001
	1.1.1.193|g__Clostridium.s__Clostridium_sp_AM22_11AC	-	1.63E-01	0.164	0.146	-0.018	0.003	0.002
	1.1.1.193|g__Agathobaculum.s__Agathobaculum_butyriciproducens	-	3.21E-01	0.123	0.106	-0.017	0.003	0.002
	1.1.1.193|g__Dorea.s__Dorea_longicatena	+	9.95E-01	0.095	0.091	-0.004	0.002	0.003
	1.1.1.193|g__Acidaminococcus.s__Acidaminococcus_fermentans	+	3.87E-01	0.000	0.000	0.000	0.000	0.000
	1.1.1.193|g__Acidaminococcus.s__Acidaminococcus_intestini	-	9.16E-01	0.000	0.000	0.000	0.001	0.003
	1.1.1.193|g__Adlercreutzia.s__Adlercreutzia_equolifaciens	-	4.71E-01	0.000	0.000	0.000	0.000	0.000
	1.1.1.193|g__Aeromonas.s__Aeromonas_caviae	-	2.85E-01	0.000	0.000	0.000	0.000	0.000

1.1.1.193|g__Akkermansia.s__Akkermansia_muciniphila	+	5.22E-02	0.000	0.000	0.000	0.009	0.019
1.1.1.193|g__Allisonella.s__Allisonella_histaminiformans	-	1.94E-01	0.000	0.000	0.000	0.000	0.000
1.1.1.193|g__Anaeromassilibacillus.s__Anaeromassilibacillus_sp_An250	+	7.98E-01	0.000	0.000	0.000	0.000	0.000
1.1.1.193|g__Anaerostipes.s__Anaerostipes_hadrus	+	2.26E-01	0.000	0.000	0.000	0.001	0.001
1.1.1.193|g__Anaerotignum.s__Anaerotignum_lactatifermentans	-	8.32E-01	0.000	0.000	0.000	0.001	0.001
1.1.1.193|g__Asaccharobacter.s__Asaccharobacter_celatus	-	1.20E-01	0.000	0.000	0.000	0.000	0.000
1.1.1.193|g__Bacteroides.s__Bacteroides_clarus	-	8.42E-01	0.000	0.000	0.000	0.002	0.009
1.1.1.193|g__Bacteroides.s__Bacteroides_coprocola	+	7.98E-01	0.000	0.000	0.000	0.028	0.029
1.1.1.193|g__Bacteroides.s__Bacteroides_coprophilus	-	6.76E-01	0.000	0.000	0.000	0.006	0.006
1.1.1.193|g__Bacteroides.s__Bacteroides_eggerthii	+	2.45E-01	0.000	0.000	0.000	0.008	0.008
1.1.1.193|g__Bacteroides.s__Bacteroides_finegoldii	+	5.06E-01	0.000	0.000	0.000	0.005	0.006
1.1.1.193|g__Bacteroides.s__Bacteroides_fluxus	-	9.26E-01	0.000	0.000	0.000	0.000	0.002
1.1.1.193|g__Bacteroides.s__Bacteroides_fragilis	+	4.01E-01	0.000	0.000	0.000	0.007	0.012
1.1.1.193|g__Bacteroides.s__Bacteroides_nordii	+	5.91E-01	0.000	0.000	0.000	0.000	0.001
1.1.1.193|g__Bacteroides.s__Bacteroides_oleiciplenus	+	3.68E-01	0.000	0.000	0.000	0.000	0.000
1.1.1.193|g__Bacteroides.s__Bacteroides_salyersiae	-	6.63E-01	0.000	0.000	0.000	0.005	0.003
1.1.1.193|g__Bacteroides.s__Bacteroides_sartorii	+	6.86E-01	0.000	0.000	0.000	0.005	0.001
1.1.1.193|g__Bacteroides.s__Bacteroides_sp_OM08_11	-	8.72E-01	0.000	0.000	0.000	0.000	0.000
1.1.1.193|g__Barnesiella.s__Barnesiella_intestinihominis	+	5.60E-02	0.000	0.000	0.000	0.006	0.010
1.1.1.193|g__Blautia.s__Blautia_hansenii	+	8.24E-01	0.000	0.000	0.000	0.000	0.000
1.1.1.193|g__Blautia.s__Blautia_sp_AF19_10LB	-	8.02E-01	0.000	0.000	0.000	0.000	0.001
1.1.1.193|g__Butyricicoccus.s__Butyricicoccus_pullicaecorum	-	9.05E-01	0.000	0.000	0.000	0.000	0.000
1.1.1.193|g__Butyricimonas.s__Butyricimonas_synergistica	+	6.70E-01	0.000	0.000	0.000	0.000	0.000
1.1.1.193|g__Catenibacterium.s__Catenibacterium_mitsuokai	-	6.06E-01	0.000	0.000	0.000	0.007	0.008
1.1.1.193|g__Cetobacterium.s__Cetobacterium_somerae	+	3.68E-01	0.000	0.000	0.000	0.000	0.000
1.1.1.193|g__Citrobacter.s__Citrobacter_amalonaticus	-	2.85E-01	0.000	0.000	0.000	0.000	0.000
1.1.1.193|g__Citrobacter.s__Citrobacter_braakii	-	1.25E-01	0.000	0.000	0.000	0.000	0.000
1.1.1.193|g__Citrobacter.s__Citrobacter_farmeri	-	2.85E-01	0.000	0.000	0.000	0.000	0.000
1.1.1.193|g__Citrobacter.s__Citrobacter_freundii	-	8.00E-01	0.000	0.000	0.000	0.000	0.000
1.1.1.193|g__Citrobacter.s__Citrobacter_koseri	+	6.86E-01	0.000	0.000	0.000	0.000	0.000
1.1.1.193|g__Citrobacter.s__Citrobacter_portucalensis	-	8.42E-01	0.000	0.000	0.000	0.000	0.000
1.1.1.193|g__Citrobacter.s__Citrobacter_werkmanii	+	3.68E-01	0.000	0.000	0.000	0.000	0.000
1.1.1.193|g__Citrobacter.s__Citrobacter_youngae	-	8.08E-01	0.000	0.000	0.000	0.000	0.000
1.1.1.193|g__Cloacibacillus.s__Cloacibacillus_porcorum	+	1.26E-02	0.000	0.000	0.000	0.000	0.000
1.1.1.193|g__Clostridiales_unclassified.s__Clostridiales_bacterium_1_7_47I	-	4.84E-01	0.000	0.000	0.000	0.000	0.000
1.1.1.193|g__Clostridioides.s__Clostridioides_difficile	+	3.12E-01	0.000	0.000	0.000	0.000	0.001
1.1.1.193|g__Clostridium.s__Butyribacterium_methylotrophicum	-	9.26E-01	0.000	0.000	0.000	0.000	0.000
1.1.1.193|g__Clostridium.s__Clostridium_disporicum	-	9.26E-01	0.000	0.000	0.000	0.000	0.000
1.1.1.193|g__Clostridium.s__Clostridium_neonatale	+	3.68E-01	0.000	0.000	0.000	0.000	0.000
1.1.1.193|g__Clostridium.s__Clostridium_perfringens	-	1.25E-01	0.000	0.000	0.000	0.001	0.000
1.1.1.193|g__Clostridium.s__Clostridium_sp_AF36_4	-	6.83E-01	0.000	0.000	0.000	0.001	0.000
1.1.1.193|g__Comamonas.s__Comamonas_kerstersii	+	3.68E-01	0.000	0.000	0.000	0.000	0.000
1.1.1.193|g__Coprobacillus.s__Coprobacillus_cateniformis	+	2.48E-01	0.000	0.000	0.000	0.000	0.000
1.1.1.193|g__Coprobacter.s__Coprobacter_fastidiosus	+	7.13E-01	0.000	0.000	0.000	0.001	0.006
1.1.1.193|g__Coprobacter.s__Coprobacter_secundus	+	3.70E-01	0.000	0.000	0.000	0.000	0.001
1.1.1.193|g__Coprococcus.s__Coprococcus_catus	+	8.06E-01	0.000	0.000	0.000	0.000	0.000
1.1.1.193|g__Coprococcus.s__Coprococcus_eutactus	+	6.29E-01	0.000	0.000	0.000	0.001	0.002
1.1.1.193|g__Desulfovibrio.s__Desulfovibrio_desulfuricans	+	3.68E-01	0.000	0.000	0.000	0.000	0.000
1.1.1.193|g__Desulfovibrio.s__Desulfovibrio_fairfieldensis	+	3.70E-02	0.000	0.000	0.000	0.000	0.000
1.1.1.193|g__Desulfovibrio.s__Desulfovibrio_piger	+	9.45E-01	0.000	0.000	0.000	0.001	0.001
1.1.1.193|g__Desulfovibrio.s__Desulfovibrio_sp_AM18_2	+	3.68E-01	0.000	0.000	0.000	0.000	0.000
1.1.1.193|g__Desulfovibrionaceae_unclassified.s__Desulfovibrionaceae_bac	-	2.83E-01	0.000	0.000	0.000	0.000	0.000
1.1.1.193|g__Dialister.s__Dialister_succinatiphilus	-	3.56E-01	0.000	0.000	0.000	0.001	0.001
1.1.1.193|g__Dorea.s__Dorea_formicigenerans	+	4.66E-01	0.000	0.000	0.000	0.000	0.001
1.1.1.193|g__Dorea.s__Dorea_sp_OM02_2LB	+	3.68E-01	0.000	0.000	0.000	0.000	0.000
1.1.1.193|g__Eggerthella.s__Eggerthella_lenta	+	6.69E-01	0.000	0.000	0.000	0.001	0.001
1.1.1.193|g__Enterobacter.s__Enterobacter_bugandensis	-	5.86E-02	0.000	0.000	0.000	0.000	0.000
1.1.1.193|g__Enterobacter.s__Enterobacter_cloacae	-	5.61E-02	0.000	0.000	0.000	0.000	0.000
1.1.1.193|g__Enterococcus.s__Enterococcus_faecium	+	3.68E-01	0.000	0.000	0.000	0.000	0.000
1.1.1.193|g__Erysipelatoclostridium.s__Clostridium_innocuum	+	1.71E-01	0.000	0.000	0.000	0.000	0.001
1.1.1.193|g__Erysipelatoclostridium.s__Clostridium_spiroforme	+	2.48E-01	0.000	0.000	0.000	0.000	0.000
1.1.1.193|g__Erysipelotrichaceae_unclassified.s__Erysipelotrichaceae_bacte	-	2.85E-01	0.000	0.000	0.000	0.000	0.000
1.1.1.193|g__Escherichia.s__Escherichia_fergusonii	+	3.68E-01	0.000	0.000	0.000	0.000	0.000
1.1.1.193|g__Eubacterium.s__Eubacterium_callanderi	-	9.05E-01	0.000	0.000	0.000	0.000	0.000
1.1.1.193|g__Eubacterium.s__Eubacterium_limosum	+	3.68E-01	0.000	0.000	0.000	0.000	0.000
1.1.1.193|g__Eubacterium.s__Eubacterium_maltosivorans	+	3.68E-01	0.000	0.000	0.000	0.000	0.000
1.1.1.193|g__Eubacterium.s__Eubacterium_ramulus	-	4.08E-01	0.000	0.000	0.000	0.000	0.000
1.1.1.193|g__Eubacterium.s__Eubacterium_sp_AF17_7	+	5.93E-02	0.000	0.000	0.000	0.000	0.000
1.1.1.193|g__Eubacterium.s__Eubacterium_sp_AM18_10LB_B	-	9.26E-01	0.000	0.000	0.000	0.000	0.000
1.1.1.193|g__Faecalicatena.s__Faecalicatena_contorta	+	7.98E-01	0.000	0.000	0.000	0.000	0.000
1.1.1.193|g__Firmicutes_unclassified.s__Firmicutes_bacterium_AM10_47	+	6.54E-01	0.000	0.000	0.000	0.000	0.000
1.1.1.193|g__Flavonifractor.s__Flavonifractor_plautii	+	2.90E-01	0.000	0.000	0.000	0.000	0.000

1.1.1.193|g__Fusobacterium.s__Fusobacterium_mortiferum	-	3.24E-01	0.000	0.000	0.000	0.004	0.001
1.1.1.193|g__Fusobacterium.s__Fusobacterium_nucleatum	+	3.68E-01	0.000	0.000	0.000	0.000	0.000
1.1.1.193|g__Fusobacterium.s__Fusobacterium_ulcerans	+	2.28E-01	0.000	0.000	0.000	0.000	0.002
1.1.1.193|g__Fusobacterium.s__Fusobacterium_varium	-	4.57E-01	0.000	0.000	0.000	0.001	0.000
1.1.1.193|g__Haemophilus.s__Haemophilus_parahaemolyticus	+	3.68E-01	0.000	0.000	0.000	0.000	0.000
1.1.1.193|g__Haemophilus.s__Haemophilus_parainfluenzae	-	1.22E-01	0.000	0.000	0.000	0.004	0.001
1.1.1.193|g__Holdemanella.s__Holdemanella_biformis	+	2.80E-02	0.000	0.000	0.000	0.000	0.000
1.1.1.193|g__Hungatella.s__Hungatella_hathewayi	-	6.87E-01	0.000	0.000	0.000	0.001	0.001
1.1.1.193|g__Intestinibacter.s__Intestinibacter_bartlettii	+	3.68E-01	0.000	0.000	0.000	0.000	0.000
1.1.1.193|g__Klebsiella.s__Klebsiella_aerogenes	-	1.24E-01	0.000	0.000	0.000	0.000	0.000
1.1.1.193|g__Klebsiella.s__Klebsiella_grimontii	-	2.85E-01	0.000	0.000	0.000	0.000	0.000
1.1.1.193|g__Klebsiella.s__Klebsiella_michiganensis	-	4.57E-01	0.000	0.000	0.000	0.000	0.000
1.1.1.193|g__Klebsiella.s__Klebsiella_oxytoca	-	3.64E-01	0.000	0.000	0.000	0.007	0.001
1.1.1.193|g__Klebsiella.s__Klebsiella_pneumoniae	-	2.34E-01	0.000	0.000	0.000	0.008	0.003
1.1.1.193|g__Klebsiella.s__Klebsiella_variicola	+	5.82E-01	0.000	0.000	0.000	0.001	0.000
1.1.1.193|g__Kluyvera.s__Kluyvera_ascorbata	-	2.85E-01	0.000	0.000	0.000	0.000	0.000
1.1.1.193|g__Kluyvera.s__Kluyvera_georgiana	-	2.85E-01	0.000	0.000	0.000	0.000	0.000
1.1.1.193|g__Lachnoclostridium.s__Clostridium_aldenense	+	4.29E-01	0.000	0.000	0.000	0.000	0.000
1.1.1.193|g__Lachnoclostridium.s__Clostridium_bolteae	-	8.79E-01	0.000	0.000	0.000	0.001	0.001
1.1.1.193|g__Lachnoclostridium.s__Clostridium_citroniae	+	5.93E-01	0.000	0.000	0.000	0.000	0.000
1.1.1.193|g__Lachnoclostridium.s__Clostridium_clostridioforme	-	9.30E-01	0.000	0.000	0.000	0.000	0.000
1.1.1.193|g__Lachnoclostridium.s__Clostridium_symbiosum	+	2.44E-02	0.000	0.000	0.000	0.000	0.001
1.1.1.193|g__Lachnospira.s__Lachnospira_pectinoschiza	-	1.29E-01	0.000	0.000	0.000	0.001	0.001
1.1.1.193|g__Lactobacillus.s__Lactobacillus_fermentum	-	2.85E-01	0.000	0.000	0.000	0.000	0.000
1.1.1.193|g__Lactobacillus.s__Lactobacillus_plantarum	+	3.68E-01	0.000	0.000	0.000	0.000	0.000
1.1.1.193|g__Lactobacillus.s__Lactobacillus_rogosae	-	1.36E-01	0.000	0.000	0.000	0.002	0.001
1.1.1.193|g__Leclercia.s__Leclercia_adecarboxylata	-	1.25E-01	0.000	0.000	0.000	0.000	0.000
1.1.1.193|g__Lelliottia.s__Lelliottia_nimipressuralis	-	3.34E-01	0.000	0.000	0.000	0.000	0.000
1.1.1.193|g__Listeria.s__Listeria_monocytogenes	+	3.68E-01	0.000	0.000	0.000	0.000	0.000
1.1.1.193|g__Megamonas.s__Megamonas_funiformis	-	7.60E-01	0.000	0.000	0.000	0.002	0.001
1.1.1.193|g__Megamonas.s__Megamonas_hypermegale	-	4.38E-01	0.000	0.000	0.000	0.003	0.001
1.1.1.193|g__Megamonas.s__Megamonas_rupellensis	-	3.93E-01	0.000	0.000	0.000	0.003	0.002
1.1.1.193|g__Megasphaera.s__Megasphaera_elsdenii	-	1.25E-01	0.000	0.000	0.000	0.000	0.000
1.1.1.193|g__Megasphaera.s__Megasphaera_hexanoica	-	9.26E-01	0.000	0.000	0.000	0.000	0.000
1.1.1.193|g__Megasphaera.s__Megasphaera_micronuciformis	+	1.96E-01	0.000	0.000	0.000	0.000	0.000
1.1.1.193|g__Megasphaera.s__Megasphaera_stantonii	-	8.87E-01	0.000	0.000	0.000	0.000	0.000
1.1.1.193|g__Mesosutterella.s__Mesosutterella_multiformis	-	4.11E-01	0.000	0.000	0.000	0.003	0.003
1.1.1.193|g__Metakosakonia.s__Kluyvera_intestini	+	3.68E-01	0.000	0.000	0.000	0.000	0.000
1.1.1.193|g__Mitsuokella.s__Mitsuokella_jalaludinii	-	2.50E-01	0.000	0.000	0.000	0.000	0.000
1.1.1.193|g__Mitsuokella.s__Mitsuokella_multacida	-	5.21E-01	0.000	0.000	0.000	0.000	0.000
1.1.1.193|g__Odoribacter.s__Odoribacter_laneus	+	3.97E-01	0.000	0.000	0.000	0.000	0.002
1.1.1.193|g__Oxalobacter.s__Oxalobacter_formigenes	+	2.90E-01	0.000	0.000	0.000	0.000	0.000
1.1.1.193|g__Pantoea.s__Pantoea_sesami	-	5.02E-01	0.000	0.000	0.000	0.000	0.000
1.1.1.193|g__Parabacteroides.s__Parabacteroides_chinchillae	-	1.25E-01	0.000	0.000	0.000	0.000	0.000
1.1.1.193|g__Parabacteroides.s__Parabacteroides_goldsteinii	+	2.48E-02	0.000	0.000	0.000	0.001	0.003
1.1.1.193|g__Parabacteroides.s__Parabacteroides_gordonii	-	8.42E-01	0.000	0.000	0.000	0.000	0.000
1.1.1.193|g__Parabacteroides.s__Parabacteroides_johnsonii	+	8.22E-01	0.000	0.000	0.000	0.002	0.001
1.1.1.193|g__Paraprevotella.s__Paraprevotella_clara	-	6.06E-01	0.000	0.000	0.000	0.007	0.007
1.1.1.193|g__Paraprevotella.s__Paraprevotella_xylaniphila	+	8.39E-02	0.000	0.000	0.000	0.000	0.002
1.1.1.193|g__Parasutterella.s__Parasutterella_excrementihominis	+	5.28E-01	0.000	0.000	0.000	0.004	0.003
1.1.1.193|g__Pedobacter.s__Pedobacter_himalayensis	-	1.25E-01	0.000	0.000	0.000	0.000	0.000
1.1.1.193|g__Phascolarctobacterium.s__Phascolarctobacterium_succinatuter	+	3.98E-01	0.000	0.000	0.000	0.002	0.003
1.1.1.193|g__Plesiomonas.s__Plesiomonas_shigelloides	-	2.85E-01	0.000	0.000	0.000	0.001	0.000
1.1.1.193|g__Prevotella.s__Prevotella_buccae	-	2.85E-01	0.000	0.000	0.000	0.004	0.000
1.1.1.193|g__Prevotella.s__Prevotella_copri	+	8.41E-01	0.000	0.000	0.000	0.179	0.129
1.1.1.193|g__Prevotella.s__Prevotella_sp_109	+	6.55E-01	0.000	0.000	0.000	0.015	0.008
1.1.1.193|g__Prevotella.s__Prevotella_sp_AM42_24	-	2.85E-01	0.000	0.000	0.000	0.004	0.000
1.1.1.193|g__Pseudomonas.s__Pseudomonas_helleri	-	2.85E-01	0.000	0.000	0.000	0.000	0.000
1.1.1.193|g__Pyramidobacter.s__Pyramidobacter_piscolens	+	5.56E-01	0.000	0.000	0.000	0.000	0.000
1.1.1.193|g__Pyramidobacter.s__Pyramidobacter_sp_C12_8	+	3.68E-01	0.000	0.000	0.000	0.000	0.000
1.1.1.193|g__Raoultella.s__Raoultella_ornithinolytica	-	4.57E-01	0.000	0.000	0.000	0.001	0.000
1.1.1.193|g__Raoultella.s__Raoultella_planticola	+	3.68E-01	0.000	0.000	0.000	0.000	0.000
1.1.1.193|g__Roseburia.s__Roseburia_faecis	+	3.56E-01	0.000	0.000	0.000	0.001	0.002
1.1.1.193|g__Roseburia.s__Roseburia_intestinalis	-	3.34E-01	0.000	0.000	0.000	0.000	0.000
1.1.1.193|g__Roseburia.s__Roseburia_inulinivorans	-	3.36E-01	0.000	0.000	0.000	0.001	0.000
1.1.1.193|g__Ruminococcaceae_unclassified.s__Ruminococcaceae_bacteriu	-	5.33E-01	0.000	0.000	0.000	0.000	0.000
1.1.1.193|g__Ruminococcaceae_unclassified.s__Ruminococcaceae_bacteriu	+	2.28E-01	0.000	0.000	0.000	0.000	0.000
1.1.1.193|g__Ruminococcus.s__Ruminococcus_callidus	-	3.51E-01	0.000	0.000	0.000	0.001	0.001
1.1.1.193|g__Ruminococcus.s__Ruminococcus_sp_AF31_8BH	+	8.07E-02	0.000	0.000	0.000	0.000	0.001
1.1.1.193|g__Salmonella.s__Salmonella_enterica	-	2.80E-02	0.000	0.000	0.000	0.000	0.000
1.1.1.193|g__Sanguibacteroides.s__Sanguibacteroides_justesenii	+	6.86E-01	0.000	0.000	0.000	0.000	0.000
1.1.1.193|g__Slackia.s__Slackia_isoflavoniconvertens	+	1.96E-01	0.000	0.000	0.000	0.000	0.000

<table>
<tr><td></td><td>1.1.1.193|g__Streptococcus.s__Streptococcus_equinus</td><td>+</td><td>1.96E-01</td><td>0.000</td><td>0.000</td><td>0.000</td><td>0.000</td><td>0.000</td></tr>
<tr><td></td><td>1.1.1.193|g__Streptococcus.s__Streptococcus_gallolyticus</td><td>+</td><td>3.68E-01</td><td>0.000</td><td>0.000</td><td>0.000</td><td>0.000</td><td>0.000</td></tr>
<tr><td></td><td>1.1.1.193|g__Streptococcus.s__Streptococcus_macedonicus</td><td>+</td><td>1.96E-01</td><td>0.000</td><td>0.000</td><td>0.000</td><td>0.000</td><td>0.000</td></tr>
<tr><td></td><td>1.1.1.193|g__Streptococcus.s__Streptococcus_pasteurianus</td><td>+</td><td>1.96E-01</td><td>0.000</td><td>0.000</td><td>0.000</td><td>0.000</td><td>0.000</td></tr>
<tr><td></td><td>1.1.1.193|g__Streptococcus.s__Streptococcus_pneumoniae</td><td>+</td><td>3.68E-01</td><td>0.000</td><td>0.000</td><td>0.000</td><td>0.000</td><td>0.000</td></tr>
<tr><td></td><td>1.1.1.193|g__Succinatimonas.s__Succinatimonas_hippei</td><td>+</td><td>4.32E-01</td><td>0.000</td><td>0.000</td><td>0.000</td><td>0.000</td><td>0.000</td></tr>
<tr><td></td><td>1.1.1.193|g__Sutterella.s__Sutterella_wadsworthensis</td><td>+</td><td>6.29E-01</td><td>0.000</td><td>0.000</td><td>0.000</td><td>0.010</td><td>0.009</td></tr>
<tr><td></td><td>1.1.1.193|g__Turicimonas.s__Turicimonas_muris</td><td>+</td><td>6.54E-01</td><td>0.000</td><td>0.000</td><td>0.000</td><td>0.000</td><td>0.000</td></tr>
<tr><td></td><td>1.1.1.193|g__Veillonella.s__Veillonella_atypica</td><td>-</td><td>2.97E-01</td><td>0.000</td><td>0.000</td><td>0.000</td><td>0.000</td><td>0.000</td></tr>
<tr><td></td><td>1.1.1.193|g__Veillonella.s__Veillonella_dispar</td><td>-</td><td>7.54E-01</td><td>0.000</td><td>0.000</td><td>0.000</td><td>0.000</td><td>0.000</td></tr>
<tr><td></td><td>1.1.1.193|g__Veillonella.s__Veillonella_infantium</td><td>-</td><td>8.10E-01</td><td>0.000</td><td>0.000</td><td>0.000</td><td>0.000</td><td>0.000</td></tr>
<tr><td></td><td>1.1.1.193|g__Veillonella.s__Veillonella_parvula</td><td>-</td><td>2.05E-01</td><td>0.000</td><td>0.000</td><td>0.000</td><td>0.001</td><td>0.000</td></tr>
<tr><td></td><td>1.1.1.193|g__Veillonella.s__Veillonella_rogosae</td><td>-</td><td>2.88E-01</td><td>0.000</td><td>0.000</td><td>0.000</td><td>0.000</td><td>0.000</td></tr>
<tr><td></td><td>1.1.1.193|g__Veillonella.s__Veillonella_tobetsuensis</td><td>-</td><td>8.72E-01</td><td>0.000</td><td>0.000</td><td>0.000</td><td>0.000</td><td>0.000</td></tr>
<tr><td></td><td>1.1.1.193|g__Veillonellaceae_unclassified.s__Veillonellaceae_bacterium</td><td>+</td><td>3.68E-01</td><td>0.000</td><td>0.000</td><td>0.000</td><td>0.000</td><td>0.000</td></tr>
<tr><td></td><td>1.1.1.193|g__Victivallales_unclassified.s__Victivallales_bacterium_CCUG_</td><td>+</td><td>4.53E-01</td><td>0.000</td><td>0.000</td><td>0.000</td><td>0.001</td><td>0.002</td></tr>
<tr><td></td><td>1.1.1.193|g__Victivallis.s__Victivallis_vadensis</td><td>+</td><td>1.96E-02</td><td>0.000</td><td>0.000</td><td>0.000</td><td>0.000</td><td>0.001</td></tr>
<tr><td></td><td>1.1.1.193|g__Yokenella.s__Yokenella_regensburgei</td><td>-</td><td>2.42E-01</td><td>0.000</td><td>0.000</td><td>0.000</td><td>0.000</td><td>0.000</td></tr>
<tr><td></td><td>1.1.1.193|g__Blautia.s__Blautia_obeum</td><td>-</td><td>6.89E-01</td><td>0.210</td><td>0.221</td><td>0.012</td><td>0.003</td><td>0.004</td></tr>
<tr><td></td><td>1.1.1.193|g__Bilophila.s__Bilophila_wadsworthia</td><td>-</td><td>5.31E-01</td><td>0.156</td><td>0.187</td><td>0.032</td><td>0.004</td><td>0.003</td></tr>
<tr><td></td><td>1.1.1.193|g__Blautia.s__Ruminococcus_torques</td><td>+</td><td>7.92E-01</td><td>0.266</td><td>0.318</td><td>0.052</td><td>0.008</td><td>0.007</td></tr>
<tr><td></td><td>1.1.1.193|g__Bacteroides.s__Bacteroides_massiliensis</td><td>-</td><td>9.19E-01</td><td>0.291</td><td>0.358</td><td>0.067</td><td>0.023</td><td>0.023</td></tr>
<tr><td></td><td>1.1.1.193|g__Bacteroides.s__Bacteroides_ovatus</td><td>+</td><td>3.18E-01</td><td>0.514</td><td>0.594</td><td>0.080</td><td>0.013</td><td>0.016</td></tr>
<tr><td></td><td>1.1.1.193|g__Roseburia.s__Roseburia_hominis</td><td>+</td><td>3.66E-01</td><td>0.100</td><td>0.189</td><td>0.089</td><td>0.002</td><td>0.004</td></tr>
<tr><td></td><td>1.1.1.193|g__Bacteroides.s__Bacteroides_dorei</td><td>+</td><td>3.87E-01</td><td>0.089</td><td>0.192</td><td>0.103</td><td>0.019</td><td>0.031</td></tr>
<tr><td></td><td>1.1.1.193|g__Butyricimonas.s__Butyricimonas_virosa</td><td>+</td><td>3.01E-02</td><td>0.000</td><td>0.124</td><td>0.124</td><td>0.002</td><td>0.004</td></tr>
<tr><td></td><td>1.1.1.193|g__Faecalibacterium.s__Faecalibacterium_prausnitzii</td><td>+</td><td>6.61E-01</td><td>3.165</td><td>3.337</td><td>0.171</td><td>0.043</td><td>0.043</td></tr>
<tr><td></td><td>1.1.1.193|g__Eubacterium.s__Eubacterium_eligens</td><td>+</td><td>5.78E-01</td><td>0.227</td><td>0.421</td><td>0.194</td><td>0.008</td><td>0.007</td></tr>
<tr><td></td><td>1.1.1.193|g__Bacteroides.s__Bacteroides_uniformis</td><td>+</td><td>7.76E-01</td><td>3.531</td><td>3.742</td><td>0.211</td><td>0.065</td><td>0.067</td></tr>
<tr><td></td><td>1.1.1.193|g__Bacteroides.s__Bacteroides_stercoris</td><td>-</td><td>8.48E-01</td><td>1.718</td><td>1.939</td><td>0.222</td><td>0.055</td><td>0.045</td></tr>
<tr><td></td><td>1.1.1.193|g__Bacteroides.s__Bacteroides_cellulosilyticus</td><td>+</td><td>9.88E-03</td><td>0.000</td><td>0.317</td><td>0.317</td><td>0.009</td><td>0.008</td></tr>
<tr><td></td><td>1.1.1.193|g__Phascolarctobacterium.s__Phascolarctobacterium_faecium</td><td>+</td><td>9.41E-01</td><td>0.263</td><td>0.634</td><td>0.371</td><td>0.010</td><td>0.010</td></tr>
<tr><td></td><td>1.1.1.193|g__Odoribacter.s__Odoribacter_splanchnicus</td><td>+</td><td>3.29E-02</td><td>0.324</td><td>0.696</td><td>0.372</td><td>0.006</td><td>0.008</td></tr>
<tr><td></td><td>1.1.1.193|g__Alistipes.s__Alistipes_shahii</td><td>+</td><td>3.94E-02</td><td>0.206</td><td>0.719</td><td>0.512</td><td>0.007</td><td>0.014</td></tr>
<tr><td></td><td>1.1.1.193|g__Bacteroides.s__Bacteroides_thetaiotaomicron</td><td>+</td><td>5.61E-02</td><td>0.721</td><td>1.239</td><td>0.518</td><td>0.013</td><td>0.023</td></tr>
<tr><td></td><td>1.1.1.193|g__Bacteroides.s__Bacteroides_intestinalis</td><td>+</td><td>1.39E-02</td><td>0.000</td><td>0.599</td><td>0.599</td><td>0.006</td><td>0.014</td></tr>
<tr><td></td><td>1.1.1.193|g__Parabacteroides.s__Parabacteroides_merdae</td><td>+</td><td>2.29E-02</td><td>0.398</td><td>1.057</td><td>0.659</td><td>0.008</td><td>0.016</td></tr>
<tr><td></td><td>1.1.1.193|g__Parabacteroides.s__Parabacteroides_distasonis</td><td>+</td><td>3.32E-02</td><td>0.848</td><td>1.509</td><td>0.660</td><td>0.014</td><td>0.021</td></tr>
<tr><td></td><td>1.1.1.193|unclassified</td><td>+</td><td>6.64E-01</td><td>6.470</td><td>7.162</td><td>0.692</td><td>0.069</td><td>0.105</td></tr>
<tr><td></td><td>1.1.1.193|g__Bacteroides.s__Bacteroides_caccae</td><td>+</td><td>3.59E-02</td><td>0.364</td><td>1.264</td><td>0.900</td><td>0.012</td><td>0.018</td></tr>
</table>

<table>
<tr><th></th><th></th><th></th><th></th><th>Fractional CPM (median)</th><th></th><th></th><th>Relative CPM (average)</th><th></th></tr>
<tr><th>Country</th><th>EC number|Bacteria</th><th>Increased or decreased in PD</th><th>p -value</th><th>Control</th><th>PD</th><th>PD - Control</th><th>Control</th><th>PD</th></tr>
<tr><td>Japan</td><td>4.1.99.12</td><td>-</td><td>1.90E-03</td><td>121.137</td><td>108.317</td><td>-12.820</td><td>1.000</td><td>1.000</td></tr>
<tr><td></td><td>4.1.99.12|g__Faecalibacterium.s__Faecalibacterium_prausnitzii</td><td>-</td><td>1.46E-02</td><td>8.766</td><td>4.885</td><td>-3.882</td><td>0.080</td><td>0.062</td></tr>
<tr><td></td><td>4.1.99.12|g__Blautia.s__Ruminococcus_torques</td><td>-</td><td>9.20E-02</td><td>3.673</td><td>2.162</td><td>-1.510</td><td>0.042</td><td>0.033</td></tr>
<tr><td></td><td>4.1.99.12|g__Dorea.s__Dorea_longicatena</td><td>-</td><td>4.31E-02</td><td>1.562</td><td>0.169</td><td>-1.394</td><td>0.014</td><td>0.010</td></tr>
<tr><td></td><td>4.1.99.12|g__Blautia.s__Blautia_obeum</td><td>-</td><td>5.06E-04</td><td>4.349</td><td>3.042</td><td>-1.307</td><td>0.045</td><td>0.030</td></tr>
<tr><td></td><td>4.1.99.12|g__Blautia.s__Blautia_wexlerae</td><td>-</td><td>1.55E-04</td><td>1.639</td><td>0.670</td><td>-0.968</td><td>0.017</td><td>0.011</td></tr>
<tr><td></td><td>4.1.99.12|g__Lachnospiraceae_unclassified.s__Eubacterium_rectale</td><td>-</td><td>6.63E-02</td><td>0.532</td><td>0.000</td><td>-0.532</td><td>0.017</td><td>0.012</td></tr>
<tr><td></td><td>4.1.99.12|g__Agathobaculum.s__Agathobaculum_butyriciproducens</td><td>-</td><td>1.33E-03</td><td>0.521</td><td>0.000</td><td>-0.521</td><td>0.004</td><td>0.003</td></tr>
<tr><td></td><td>4.1.99.12|g__Bacteroides.s__Bacteroides_vulgatus</td><td>-</td><td>5.18E-01</td><td>3.263</td><td>3.133</td><td>-0.130</td><td>0.076</td><td>0.060</td></tr>
<tr><td></td><td>4.1.99.12|g__Roseburia.s__Roseburia_hominis</td><td>-</td><td>5.59E-01</td><td>0.935</td><td>0.834</td><td>-0.101</td><td>0.012</td><td>0.012</td></tr>
<tr><td></td><td>4.1.99.12|g__Acidaminococcus.s__Acidaminococcus_fermentans</td><td>+</td><td>3.85E-01</td><td>0.000</td><td>0.000</td><td>0.000</td><td>0.000</td><td>0.000</td></tr>
<tr><td></td><td>4.1.99.12|g__Acidaminococcus.s__Acidaminococcus_intestini</td><td>-</td><td>9.64E-01</td><td>0.000</td><td>0.000</td><td>0.000</td><td>0.009</td><td>0.003</td></tr>
<tr><td></td><td>4.1.99.12|g__Acinetobacter.s__Acinetobacter_baumannii</td><td>+</td><td>3.85E-01</td><td>0.000</td><td>0.000</td><td>0.000</td><td>0.000</td><td>0.000</td></tr>
<tr><td></td><td>4.1.99.12|g__Actinomyces.s__Actinomyces_naeslundii</td><td>+</td><td>3.85E-01</td><td>0.000</td><td>0.000</td><td>0.000</td><td>0.000</td><td>0.000</td></tr>
<tr><td></td><td>4.1.99.12|g__Actinomyces.s__Actinomyces_oris</td><td>+</td><td>3.85E-01</td><td>0.000</td><td>0.000</td><td>0.000</td><td>0.000</td><td>0.000</td></tr>
<tr><td></td><td>4.1.99.12|g__Actinomyces.s__Actinomyces_viscosus</td><td>+</td><td>3.85E-01</td><td>0.000</td><td>0.000</td><td>0.000</td><td>0.000</td><td>0.000</td></tr>
<tr><td></td><td>4.1.99.12|g__Adlercreutzia.s__Adlercreutzia_equolifaciens</td><td>+</td><td>1.48E-01</td><td>0.000</td><td>0.000</td><td>0.000</td><td>0.000</td><td>0.000</td></tr>
<tr><td></td><td>4.1.99.12|g__Akkermansia.s__Akkermansia_muciniphila</td><td>+</td><td>2.41E-03</td><td>0.000</td><td>0.000</td><td>0.000</td><td>0.004</td><td>0.028</td></tr>
<tr><td></td><td>4.1.99.12|g__Alistipes.s__Alistipes_indistinctus</td><td>+</td><td>4.83E-02</td><td>0.000</td><td>0.000</td><td>0.000</td><td>0.000</td><td>0.001</td></tr>
<tr><td></td><td>4.1.99.12|g__Alistipes.s__Alistipes_inops</td><td>-</td><td>8.96E-01</td><td>0.000</td><td>0.000</td><td>0.000</td><td>0.001</td><td>0.001</td></tr>
<tr><td></td><td>4.1.99.12|g__Alistipes.s__Alistipes_onderdonkii</td><td>+</td><td>4.02E-03</td><td>0.000</td><td>0.000</td><td>0.000</td><td>0.003</td><td>0.010</td></tr>
<tr><td></td><td>4.1.99.12|g__Alistipes.s__Alistipes_sp_An31A</td><td>-</td><td>8.57E-01</td><td>0.000</td><td>0.000</td><td>0.000</td><td>0.001</td><td>0.001</td></tr>
<tr><td></td><td>4.1.99.12|g__Alistipes.s__Alistipes_sp_An66</td><td>+</td><td>3.85E-01</td><td>0.000</td><td>0.000</td><td>0.000</td><td>0.000</td><td>0.000</td></tr>
<tr><td></td><td>4.1.99.12|g__Alistipes.s__Alistipes_timonensis</td><td>+</td><td>7.21E-01</td><td>0.000</td><td>0.000</td><td>0.000</td><td>0.000</td><td>0.000</td></tr>
<tr><td></td><td>4.1.99.12|g__Allisonella.s__Allisonella_histaminiformans</td><td>+</td><td>1.00E+00</td><td>0.000</td><td>0.000</td><td>0.000</td><td>0.000</td><td>0.000</td></tr>
<tr><td></td><td>4.1.99.12|g__Anaeroglobus.s__Anaeroglobus_geminatus</td><td>+</td><td>2.14E-01</td><td>0.000</td><td>0.000</td><td>0.000</td><td>0.000</td><td>0.000</td></tr>
<tr><td></td><td>4.1.99.12|g__Anaeromassilibacillus.s__Anaeromassilibacillus_sp_An250</td><td>+</td><td>7.31E-01</td><td>0.000</td><td>0.000</td><td>0.000</td><td>0.000</td><td>0.000</td></tr>
<tr><td></td><td>4.1.99.12|g__Anaerostipes.s__Anaerostipes_caccae</td><td>+</td><td>5.44E-01</td><td>0.000</td><td>0.000</td><td>0.000</td><td>0.001</td><td>0.001</td></tr>
<tr><td></td><td>4.1.99.12|g__Anaerostipes.s__Anaerostipes_hadrus</td><td>-</td><td>4.94E-01</td><td>0.000</td><td>0.000</td><td>0.000</td><td>0.016</td><td>0.012</td></tr>
<tr><td></td><td>4.1.99.12|g__Anaerotignum.s__Anaerotignum_lactatifermentans</td><td>+</td><td>7.78E-04</td><td>0.000</td><td>0.000</td><td>0.000</td><td>0.000</td><td>0.002</td></tr>
</table>

4.1.99.12|g__Asaccharobacter.s__Asaccharobacter_celatus	+	1.98E-01	0.000	0.000	0.000	0.000	0.001
4.1.99.12|g__Bacillus.s__Bacillus_gibsonii	+	9.85E-03	0.000	0.000	0.000	0.000	0.000
4.1.99.12|g__Bacillus.s__Bacillus_murimartini	+	3.27E-02	0.000	0.000	0.000	0.000	0.001
4.1.99.12|g__Bacteroides.s__Bacteroides_caccae	+	2.44E-01	0.000	0.000	0.000	0.004	0.006
4.1.99.12|g__Bacteroides.s__Bacteroides_clarus	+	1.56E-01	0.000	0.000	0.000	0.000	0.003
4.1.99.12|g__Bacteroides.s__Bacteroides_coprocola	+	2.03E-01	0.000	0.000	0.000	0.008	0.010
4.1.99.12|g__Bacteroides.s__Bacteroides_coprophilus	+	4.41E-01	0.000	0.000	0.000	0.001	0.001
4.1.99.12|g__Bacteroides.s__Bacteroides_eggerthii	+	2.71E-01	0.000	0.000	0.000	0.003	0.009
4.1.99.12|g__Bacteroides.s__Bacteroides_faecis	-	2.69E-01	0.000	0.000	0.000	0.000	0.000
4.1.99.12|g__Bacteroides.s__Bacteroides_finegoldii	+	2.44E-01	0.000	0.000	0.000	0.003	0.002
4.1.99.12|g__Bacteroides.s__Bacteroides_fluxus	-	2.62E-01	0.000	0.000	0.000	0.000	0.000
4.1.99.12|g__Bacteroides.s__Bacteroides_fragilis	-	6.95E-01	0.000	0.000	0.000	0.006	0.006
4.1.99.12|g__Bacteroides.s__Bacteroides_intestinalis	-	9.40E-01	0.000	0.000	0.000	0.002	0.002
4.1.99.12|g__Bacteroides.s__Bacteroides_massiliensis	+	1.88E-01	0.000	0.000	0.000	0.002	0.004
4.1.99.12|g__Bacteroides.s__Bacteroides_nordii	+	1.10E-02	0.000	0.000	0.000	0.000	0.001
4.1.99.12|g__Bacteroides.s__Bacteroides_oleiciplenus	+	3.85E-01	0.000	0.000	0.000	0.000	0.000
4.1.99.12|g__Bacteroides.s__Bacteroides_plebeius	+	2.00E-01	0.000	0.000	0.000	0.018	0.015
4.1.99.12|g__Bacteroides.s__Bacteroides_salyersiae	-	7.83E-01	0.000	0.000	0.000	0.000	0.000
4.1.99.12|g__Bacteroides.s__Bacteroides_stercoris	-	6.67E-01	0.000	0.000	0.000	0.027	0.020
4.1.99.12|g__Barnesiella.s__Barnesiella_intestinihominis	+	3.64E-02	0.000	0.000	0.000	0.003	0.007
4.1.99.12|g__Barnesiella.s__Barnesiella_sp_An22	-	2.62E-01	0.000	0.000	0.000	0.000	0.000
4.1.99.12|g__Bifidobacterium.s__Bifidobacterium_longum	-	8.71E-01	0.000	0.000	0.000	0.000	0.000
4.1.99.12|g__Blautia.s__Blautia_hansenii	-	2.78E-01	0.000	0.000	0.000	0.000	0.000
4.1.99.12|g__Blautia.s__Blautia_sp_AF19_10LB	+	7.23E-01	0.000	0.000	0.000	0.002	0.003
4.1.99.12|g__Blautia.s__Blautia_sp_An249	+	2.14E-01	0.000	0.000	0.000	0.000	0.000
4.1.99.12|g__Butyricicoccus.s__Butyricicoccus_pullicaecorum	-	1.99E-01	0.000	0.000	0.000	0.000	0.000
4.1.99.12|g__Butyricimonas.s__Butyricimonas_virosa	+	1.58E-02	0.000	0.000	0.000	0.000	0.001
4.1.99.12|g__Butyrivibrio.s__Butyrivibrio_crossotus	+	3.85E-01	0.000	0.000	0.000	0.000	0.001
4.1.99.12|g__Candidatus_Gastranaerophilales_unclassified.s__Candidatus_(	-	8.57E-01	0.000	0.000	0.000	0.000	0.000
4.1.99.12|g__Catenibacterium.s__Catenibacterium_mitsuokai	-	5.62E-03	0.000	0.000	0.000	0.002	0.000
4.1.99.12|g__Cellulosilyticum.s__Cellulosilyticum_lentocellum	+	3.85E-01	0.000	0.000	0.000	0.000	0.000
4.1.99.12|g__Cetobacterium.s__Cetobacterium_somerae	-	2.62E-01	0.000	0.000	0.000	0.000	0.000
4.1.99.12|g__Citrobacter.s__Citrobacter_braakii	+	1.78E-01	0.000	0.000	0.000	0.000	0.001
4.1.99.12|g__Citrobacter.s__Citrobacter_freundii	+	2.14E-01	0.000	0.000	0.000	0.000	0.000
4.1.99.12|g__Citrobacter.s__Citrobacter_koseri	+	3.85E-01	0.000	0.000	0.000	0.000	0.000
4.1.99.12|g__Citrobacter.s__Citrobacter_portucalensis	+	2.77E-01	0.000	0.000	0.000	0.000	0.001
4.1.99.12|g__Citrobacter.s__Citrobacter_werkmanii	+	3.85E-01	0.000	0.000	0.000	0.000	0.001
4.1.99.12|g__Citrobacter.s__Citrobacter_youngae	+	3.85E-01	0.000	0.000	0.000	0.000	0.000
4.1.99.12|g__Cloacibacillus.s__Cloacibacillus_porcorum	+	1.26E-01	0.000	0.000	0.000	0.000	0.001
4.1.99.12|g__Clostridioides.s__Clostridioides_difficile	+	1.55E-01	0.000	0.000	0.000	0.005	0.005
4.1.99.12|g__Clostridium.s__Butyribacterium_methylotrophicum	+	4.39E-01	0.000	0.000	0.000	0.000	0.000
4.1.99.12|g__Clostridium.s__Clostridium_disporicum	+	8.35E-01	0.000	0.000	0.000	0.001	0.001
4.1.99.12|g__Clostridium.s__Clostridium_perfringens	-	9.89E-02	0.000	0.000	0.000	0.000	0.000
4.1.99.12|g__Clostridium.s__Clostridium_sp_AF36_4	-	7.90E-01	0.000	0.000	0.000	0.001	0.001
4.1.99.12|g__Clostridium.s__Clostridium_sp_AM22_11AC	-	1.06E-01	0.000	0.000	0.000	0.003	0.001
4.1.99.12|g__Coprobacillus.s__Coprobacillus_cateniformis	+	4.14E-02	0.000	0.000	0.000	0.000	0.001
4.1.99.12|g__Coprobacter.s__Coprobacter_fastidiosus	+	1.11E-01	0.000	0.000	0.000	0.001	0.001
4.1.99.12|g__Coprobacter.s__Coprobacter_secundus	+	3.24E-02	0.000	0.000	0.000	0.000	0.001
4.1.99.12|g__Coprococcus.s__Coprococcus_catus	-	6.85E-01	0.000	0.000	0.000	0.002	0.002
4.1.99.12|g__Coprococcus.s__Coprococcus_comes	-	1.38E-02	0.000	0.000	0.000	0.005	0.003
4.1.99.12|g__Coprococcus.s__Coprococcus_eutactus	-	2.89E-01	0.000	0.000	0.000	0.002	0.002
4.1.99.12|g__Desulfovibrio.s__Desulfovibrio_desulfuricans	+	1.62E-03	0.000	0.000	0.000	0.000	0.002
4.1.99.12|g__Desulfovibrio.s__Desulfovibrio_fairfieldensis	+	1.26E-01	0.000	0.000	0.000	0.000	0.001
4.1.99.12|g__Desulfovibrio.s__Desulfovibrio_piger	+	1.33E-01	0.000	0.000	0.000	0.001	0.001
4.1.99.12|g__Desulfovibrio.s__Desulfovibrio_sp_AM18_2	+	3.85E-01	0.000	0.000	0.000	0.000	0.000
4.1.99.12|g__Desulfovibrionaceae_unclassified.s__Desulfovibrionaceae_bac	+	3.85E-01	0.000	0.000	0.000	0.000	0.000
4.1.99.12|g__Dialister.s__Dialister_invisus	-	1.64E-01	0.000	0.000	0.000	0.005	0.002
4.1.99.12|g__Dialister.s__Dialister_succinatiphilus	+	7.10E-01	0.000	0.000	0.000	0.000	0.000
4.1.99.12|g__Dorea.s__Dorea_formicigenerans	-	7.64E-01	0.000	0.000	0.000	0.004	0.004
4.1.99.12|g__Dorea.s__Dorea_sp_OM02_2LB	+	7.21E-01	0.000	0.000	0.000	0.000	0.000
4.1.99.12|g__Eggerthella.s__Eggerthella_lenta	+	2.03E-01	0.000	0.000	0.000	0.004	0.003
4.1.99.12|g__Enterobacter.s__Enterobacter_cloacae	+	8.87E-01	0.000	0.000	0.000	0.000	0.000
4.1.99.12|g__Enterobacter.s__Enterobacter_mori	+	1.26E-01	0.000	0.000	0.000	0.000	0.000
4.1.99.12|g__Enterococcus.s__Enterococcus_avium	+	4.14E-01	0.000	0.000	0.000	0.005	0.001
4.1.99.12|g__Enterococcus.s__Enterococcus_casseliflavus	+	3.85E-01	0.000	0.000	0.000	0.000	0.000
4.1.99.12|g__Enterococcus.s__Enterococcus_faecalis	-	2.62E-01	0.000	0.000	0.000	0.000	0.000
4.1.99.12|g__Enterococcus.s__Enterococcus_faecium	-	6.92E-01	0.000	0.000	0.000	0.005	0.001
4.1.99.12|g__Enterococcus.s__Enterococcus_gallinarum	+	2.14E-01	0.000	0.000	0.000	0.000	0.000
4.1.99.12|g__Enterococcus.s__Enterococcus_saccharolyticus	+	3.85E-01	0.000	0.000	0.000	0.000	0.000
4.1.99.12|g__Erysipelatoclostridium.s__Clostridium_innocuum	+	7.99E-01	0.000	0.000	0.000	0.001	0.001
4.1.99.12|g__Erysipelatoclostridium.s__Clostridium_spiroforme	+	8.71E-01	0.000	0.000	0.000	0.003	0.001
4.1.99.12|g__Erysipelotrichaceae_unclassified.s__Erysipelotrichaceae_bacte	+	1.09E-01	0.000	0.000	0.000	0.000	0.001

4.1.99.12|g__Escherichia.s__Escherichia_coli	-	5.91E-01	0.000	0.000	0.000	0.034	0.014
4.1.99.12|g__Escherichia.s__Escherichia_fergusonii	+	3.85E-01	0.000	0.000	0.000	0.000	0.000
4.1.99.12|g__Eubacterium.s__Eubacterium_callanderi	+	4.39E-01	0.000	0.000	0.000	0.000	0.000
4.1.99.12|g__Eubacterium.s__Eubacterium_limosum	+	1.26E-01	0.000	0.000	0.000	0.000	0.000
4.1.99.12|g__Eubacterium.s__Eubacterium_maltosivorans	+	2.14E-01	0.000	0.000	0.000	0.000	0.000
4.1.99.12|g__Eubacterium.s__Eubacterium_ramulus	-	9.67E-01	0.000	0.000	0.000	0.002	0.003
4.1.99.12|g__Eubacterium.s__Eubacterium_sp_AF17_7	+	2.56E-01	0.000	0.000	0.000	0.001	0.003
4.1.99.12|g__Eubacterium.s__Eubacterium_sp_AM18_10LB_B	-	8.60E-01	0.000	0.000	0.000	0.001	0.000
4.1.99.12|g__Faecalitalea.s__Faecalitalea_cylindroides	+	6.52E-02	0.000	0.000	0.000	0.000	0.000
4.1.99.12|g__Firmicutes_unclassified.s__Firmicutes_bacterium_AM10_47	+	7.26E-01	0.000	0.000	0.000	0.002	0.002
4.1.99.12|g__Flavonifractor.s__Flavonifractor_plautii	+	7.46E-02	0.000	0.000	0.000	0.000	0.000
4.1.99.12|g__Flavonifractor.s__Flavonifractor_sp_An10	+	6.91E-02	0.000	0.000	0.000	0.000	0.002
4.1.99.12|g__Flavonifractor.s__Flavonifractor_sp_An82	+	3.85E-01	0.000	0.000	0.000	0.000	0.000
4.1.99.12|g__Fusobacterium.s__Fusobacterium_mortiferum	-	2.12E-01	0.000	0.000	0.000	0.000	0.000
4.1.99.12|g__Fusobacterium.s__Fusobacterium_ulcerans	+	4.35E-01	0.000	0.000	0.000	0.000	0.000
4.1.99.12|g__Haemophilus.s__Haemophilus_parainfluenzae	-	2.21E-02	0.000	0.000	0.000	0.002	0.000
4.1.99.12|g__Holdemanella.s__Holdemanella_biformis	-	4.86E-01	0.000	0.000	0.000	0.000	0.002
4.1.99.12|g__Hungatella.s__Hungatella_hathewayi	+	5.29E-01	0.000	0.000	0.000	0.001	0.001
4.1.99.12|g__Intestinibacter.s__Intestinibacter_bartlettii	+	9.14E-01	0.000	0.000	0.000	0.002	0.002
4.1.99.12|g__Klebsiella.s__Klebsiella_aerogenes	-	7.93E-01	0.000	0.000	0.000	0.002	0.000
4.1.99.12|g__Klebsiella.s__Klebsiella_grimontii	-	8.57E-01	0.000	0.000	0.000	0.000	0.000
4.1.99.12|g__Klebsiella.s__Klebsiella_michiganensis	-	8.03E-01	0.000	0.000	0.000	0.000	0.000
4.1.99.12|g__Klebsiella.s__Klebsiella_oxytoca	+	4.47E-01	0.000	0.000	0.000	0.003	0.003
4.1.99.12|g__Klebsiella.s__Klebsiella_pneumoniae	-	5.63E-01	0.000	0.000	0.000	0.003	0.009
4.1.99.12|g__Klebsiella.s__Klebsiella_variicola	-	9.75E-01	0.000	0.000	0.000	0.001	0.001
4.1.99.12|g__Kluyvera.s__Kluyvera_cryocrescens	+	1.00E+00	0.000	0.000	0.000	0.000	0.000
4.1.99.12|g__Lachnoclostridium.s__Clostridium_aldenense	+	5.79E-01	0.000	0.000	0.000	0.000	0.000
4.1.99.12|g__Lachnoclostridium.s__Clostridium_bolteae	+	6.33E-01	0.000	0.000	0.000	0.001	0.001
4.1.99.12|g__Lachnoclostridium.s__Clostridium_citroniae	+	7.21E-01	0.000	0.000	0.000	0.000	0.000
4.1.99.12|g__Lachnoclostridium.s__Clostridium_clostridioforme	-	3.59E-02	0.000	0.000	0.000	0.000	0.000
4.1.99.12|g__Lachnoclostridium.s__Clostridium_symbiosum	-	7.93E-01	0.000	0.000	0.000	0.000	0.000
4.1.99.12|g__Lachnoclostridium.s__Lachnoclostridium_sp_An138	-	8.57E-01	0.000	0.000	0.000	0.000	0.000
4.1.99.12|g__Lachnospira.s__Lachnospira_pectinoschiza	-	2.38E-01	0.000	0.000	0.000	0.002	0.002
4.1.99.12|g__Lachnospiraceae_unclassified.s__Lachnospiraceae_bacterium_	+	7.31E-01	0.000	0.000	0.000	0.000	0.000
4.1.99.12|g__Lactobacillus.s__Lactobacillus_amylovorus	-	8.57E-01	0.000	0.000	0.000	0.000	0.000
4.1.99.12|g__Lactobacillus.s__Lactobacillus_antri	+	1.26E-01	0.000	0.000	0.000	0.000	0.001
4.1.99.12|g__Lactobacillus.s__Lactobacillus_delbrueckii	-	2.62E-01	0.000	0.000	0.000	0.000	0.000
4.1.99.12|g__Lactobacillus.s__Lactobacillus_fermentum	+	2.92E-02	0.000	0.000	0.000	0.000	0.003
4.1.99.12|g__Lactobacillus.s__Lactobacillus_mucosae	+	2.33E-01	0.000	0.000	0.000	0.001	0.005
4.1.99.12|g__Lactobacillus.s__Lactobacillus_oris	+	6.05E-01	0.000	0.000	0.000	0.002	0.004
4.1.99.12|g__Lactobacillus.s__Lactobacillus_reuteri	+	3.85E-01	0.000	0.000	0.000	0.000	0.000
4.1.99.12|g__Lactobacillus.s__Lactobacillus_rogosae	-	2.60E-01	0.000	0.000	0.000	0.003	0.002
4.1.99.12|g__Lactococcus.s__Lactococcus_lactis	-	4.80E-01	0.000	0.000	0.000	0.001	0.000
4.1.99.12|g__Leclercia.s__Leclercia_adecarboxylata	-	4.41E-01	0.000	0.000	0.000	0.000	0.000
4.1.99.12|g__Lelliottia.s__Lelliottia_amnigena	-	1.09E-01	0.000	0.000	0.000	0.000	0.000
4.1.99.12|g__Lelliottia.s__Lelliottia_nimipressuralis	-	2.62E-01	0.000	0.000	0.000	0.000	0.000
4.1.99.12|g__Megamonas.s__Megamonas_funiformis	-	8.83E-01	0.000	0.000	0.000	0.009	0.006
4.1.99.12|g__Megamonas.s__Megamonas_rupellensis	+	9.30E-01	0.000	0.000	0.000	0.005	0.002
4.1.99.12|g__Megasphaera.s__Megasphaera_elsdenii	-	8.71E-01	0.000	0.000	0.000	0.000	0.000
4.1.99.12|g__Megasphaera.s__Megasphaera_hexanoica	+	3.85E-01	0.000	0.000	0.000	0.000	0.000
4.1.99.12|g__Megasphaera.s__Megasphaera_sp_DISK_18	-	1.29E-01	0.000	0.000	0.000	0.004	0.001
4.1.99.12|g__Megasphaera.s__Megasphaera_sp_MJR8396C	-	7.68E-01	0.000	0.000	0.000	0.001	0.001
4.1.99.12|g__Megasphaera.s__Megasphaera_stantonii	+	4.54E-01	0.000	0.000	0.000	0.000	0.000
4.1.99.12|g__Methanobrevibacter.s__Methanobrevibacter_smithii	+	1.13E-01	0.000	0.000	0.000	0.003	0.008
4.1.99.12|g__Methanosphaera.s__Methanosphaera_stadtmanae	-	8.03E-01	0.000	0.000	0.000	0.000	0.001
4.1.99.12|g__Mitsuokella.s__Mitsuokella_jalaludinii	+	3.85E-01	0.000	0.000	0.000	0.000	0.000
4.1.99.12|g__Mitsuokella.s__Mitsuokella_multacida	+	3.85E-01	0.000	0.000	0.000	0.000	0.000
4.1.99.12|g__Mogibacterium.s__Mogibacterium_diversum	+	6.06E-01	0.000	0.000	0.000	0.000	0.000
4.1.99.12|g__Neisseria.s__Neisseria_flavescens	-	2.62E-01	0.000	0.000	0.000	0.000	0.000
4.1.99.12|g__Neisseria.s__Neisseria_mucosa	-	2.62E-01	0.000	0.000	0.000	0.000	0.000
4.1.99.12|g__Neisseria.s__Neisseria_perflava	-	2.62E-01	0.000	0.000	0.000	0.000	0.000
4.1.99.12|g__Odoribacter.s__Odoribacter_laneus	+	2.46E-01	0.000	0.000	0.000	0.000	0.001
4.1.99.12|g__Pantoea.s__Pantoea_sesami	-	4.74E-01	0.000	0.000	0.000	0.000	0.000
4.1.99.12|g__Parabacteroides.s__Parabacteroides_goldsteinii	+	1.96E-01	0.000	0.000	0.000	0.000	0.001
4.1.99.12|g__Parabacteroides.s__Parabacteroides_gordonii	-	8.71E-01	0.000	0.000	0.000	0.000	0.000
4.1.99.12|g__Parabacteroides.s__Parabacteroides_johnsonii	+	4.29E-01	0.000	0.000	0.000	0.001	0.001
4.1.99.12|g__Paraprevotella.s__Paraprevotella_clara	+	3.15E-01	0.000	0.000	0.000	0.001	0.002
4.1.99.12|g__Paraprevotella.s__Paraprevotella_xylaniphila	+	1.72E-01	0.000	0.000	0.000	0.000	0.000
4.1.99.12|g__Parasutterella.s__Parasutterella_excrementihominis	-	6.32E-01	0.000	0.000	0.000	0.001	0.001
4.1.99.12|g__Pedobacter.s__Pedobacter_himalayensis	-	4.16E-01	0.000	0.000	0.000	0.000	0.000
4.1.99.12|g__Peptostreptococcaceae_unclassified.s__Clostridium_hiranonis	-	2.62E-01	0.000	0.000	0.000	0.000	0.000
4.1.99.12|g__Phascolarctobacterium.s__Phascolarctobacterium_faecium	+	1.37E-01	0.000	0.000	0.000	0.003	0.007

	4.1.99.12|g__Phascolarctobacterium.s__Phascolarctobacterium_succinatuter	-	2.76E-01	0.000	0.000	0.000	0.004	0.003
	4.1.99.12|g__Prevotella.s__Prevotella_buccae	-	2.62E-01	0.000	0.000	0.000	0.001	0.000
	4.1.99.12|g__Prevotella.s__Prevotella_copri	-	2.85E-01	0.000	0.000	0.000	0.004	0.002
	4.1.99.12|g__Prevotella.s__Prevotella_sp_109	-	2.03E-01	0.000	0.000	0.000	0.003	0.001
	4.1.99.12|g__Prevotella.s__Prevotella_sp_885	-	4.59E-02	0.000	0.000	0.000	0.004	0.000
	4.1.99.12|g__Prevotella.s__Prevotella_sp_AM42_24	-	1.99E-01	0.000	0.000	0.000	0.003	0.000
	4.1.99.12|g__Prevotella.s__Prevotella_stercorea	-	7.68E-01	0.000	0.000	0.000	0.002	0.005
	4.1.99.12|g__Pseudoflavonifractor.s__Pseudoflavonifractor_sp_An184	+	4.24E-03	0.000	0.000	0.000	0.000	0.001
	4.1.99.12|g__Pseudoramibacter.s__Pseudoramibacter_alactolyticus	-	2.62E-01	0.000	0.000	0.000	0.000	0.000
	4.1.99.12|g__Pyramidobacter.s__Pyramidobacter_piscolens	+	2.14E-01	0.000	0.000	0.000	0.000	0.000
	4.1.99.12|g__Raoultella.s__Raoultella_ornithinolytica	-	7.22E-01	0.000	0.000	0.000	0.003	0.000
	4.1.99.12|g__Raoultella.s__Raoultella_planticola	-	4.24E-01	0.000	0.000	0.000	0.000	0.000
	4.1.99.12|g__Rikenella.s__Rikenella_microfusus	+	1.26E-01	0.000	0.000	0.000	0.000	0.001
	4.1.99.12|g__Roseburia.s__Roseburia_faecis	-	3.39E-03	0.000	0.000	0.000	0.014	0.004
	4.1.99.12|g__Roseburia.s__Roseburia_intestinalis	-	1.39E-03	0.000	0.000	0.000	0.010	0.001
	4.1.99.12|g__Roseburia.s__Roseburia_inulinivorans	-	3.46E-01	0.000	0.000	0.000	0.002	0.001
	4.1.99.12|g__Ruminococcaceae_unclassified.s__Ruminococcaceae_bacteriu	+	4.71E-02	0.000	0.000	0.000	0.000	0.001
	4.1.99.12|g__Ruminococcaceae_unclassified.s__Ruminococcaceae_bacteriu	+	8.53E-01	0.000	0.000	0.000	0.000	0.000
	4.1.99.12|g__Ruminococcus.s__Ruminococcus_callidus	-	6.12E-02	0.000	0.000	0.000	0.004	0.002
	4.1.99.12|g__Ruminococcus.s__Ruminococcus_sp_AF31_8BH	+	9.57E-02	0.000	0.000	0.000	0.001	0.002
	4.1.99.12|g__Salmonella.s__Salmonella_enterica	+	3.85E-01	0.000	0.000	0.000	0.000	0.000
	4.1.99.12|g__Sanguibacteroides.s__Sanguibacteroides_justesenii	+	3.85E-01	0.000	0.000	0.000	0.000	0.000
	4.1.99.12|g__Senegalimassilia.s__Senegalimassilia_anaerobia	+	2.16E-02	0.000	0.000	0.000	0.000	0.001
	4.1.99.12|g__Slackia.s__Slackia_isoflavoniconvertens	+	7.23E-01	0.000	0.000	0.000	0.001	0.001
	4.1.99.12|g__Staphylococcus.s__Staphylococcus_aureus	+	2.14E-01	0.000	0.000	0.000	0.000	0.000
	4.1.99.12|g__Streptococcus.s__Streptococcus_equinus	-	1.09E-01	0.000	0.000	0.000	0.002	0.000
	4.1.99.12|g__Streptococcus.s__Streptococcus_gallolyticus	+	4.46E-01	0.000	0.000	0.000	0.000	0.000
	4.1.99.12|g__Streptococcus.s__Streptococcus_infantarius	-	1.09E-01	0.000	0.000	0.000	0.001	0.000
	4.1.99.12|g__Streptococcus.s__Streptococcus_lutetiensis	-	1.09E-01	0.000	0.000	0.000	0.002	0.000
	4.1.99.12|g__Streptococcus.s__Streptococcus_macedonicus	-	4.86E-01	0.000	0.000	0.000	0.000	0.001
	4.1.99.12|g__Streptococcus.s__Streptococcus_pasteurianus	-	4.92E-01	0.000	0.000	0.000	0.000	0.001
	4.1.99.12|g__Streptococcus.s__Streptococcus_pneumoniae	+	1.23E-02	0.000	0.000	0.000	0.000	0.000
	4.1.99.12|g__Sutterella.s__Sutterella_wadsworthensis	-	5.92E-01	0.000	0.000	0.000	0.003	0.003
	4.1.99.12|g__Terrisporobacter.s__Terrisporobacter_othiniensis	+	2.14E-01	0.000	0.000	0.000	0.000	0.000
	4.1.99.12|g__Veillonella.s__Veillonella_atypica	-	6.53E-04	0.000	0.000	0.000	0.002	0.000
	4.1.99.12|g__Veillonella.s__Veillonella_denticariosi	+	7.10E-01	0.000	0.000	0.000	0.000	0.000
	4.1.99.12|g__Veillonella.s__Veillonella_dispar	-	1.30E-03	0.000	0.000	0.000	0.002	0.000
	4.1.99.12|g__Veillonella.s__Veillonella_infantium	-	1.04E-02	0.000	0.000	0.000	0.000	0.000
	4.1.99.12|g__Veillonella.s__Veillonella_parvula	-	4.55E-03	0.000	0.000	0.000	0.000	0.000
	4.1.99.12|g__Veillonella.s__Veillonella_rogosae	-	1.06E-04	0.000	0.000	0.000	0.000	0.000
	4.1.99.12|g__Veillonella.s__Veillonella_tobetsuensis	-	1.09E-01	0.000	0.000	0.000	0.000	0.000
	4.1.99.12|g__Victivallales_unclassified.s__Victivallales_bacterium_CCUG_	+	7.31E-01	0.000	0.000	0.000	0.000	0.000
	4.1.99.12|g__Victivallis.s__Victivallis_vadensis	-	2.62E-01	0.000	0.000	0.000	0.000	0.000
	4.1.99.12|g__Weissella.s__Weissella_cibaria	+	2.87E-01	0.000	0.000	0.000	0.000	0.000
	4.1.99.12|g__Bacteroides.s__Bacteroides_ovatus	+	2.59E-01	0.276	0.315	0.039	0.009	0.012
	4.1.99.12|g__Bacteroides.s__Bacteroides_thetaiotaomicron	+	2.60E-01	0.430	0.489	0.059	0.006	0.010
	4.1.99.12|g__Bacteroides.s__Bacteroides_xylanisolvens	+	7.09E-01	0.075	0.177	0.102	0.006	0.007
	4.1.99.12|g__Eubacterium.s__Eubacterium_eligens	+	1.53E-01	0.000	0.108	0.108	0.008	0.016
	4.1.99.12|g__Bacteroides.s__Bacteroides_cellulosilyticus	+	1.47E-02	0.000	0.120	0.120	0.006	0.008
	4.1.99.12|g__Alistipes.s__Alistipes_finegoldii	+	5.76E-03	0.000	0.155	0.155	0.003	0.007
	4.1.99.12|g__Bacteroides.s__Bacteroides_dorei	+	8.63E-01	1.957	2.182	0.226	0.058	0.055
	4.1.99.12|g__Odoribacter.s__Odoribacter_splanchnicus	+	6.10E-04	0.000	0.248	0.248	0.001	0.003
	4.1.99.12|g__Parabacteroides.s__Parabacteroides_merdae	+	2.96E-01	0.399	0.731	0.332	0.008	0.009
	4.1.99.12|g__Parabacteroides.s__Parabacteroides_distasonis	+	3.12E-02	0.576	0.949	0.373	0.010	0.014
	4.1.99.12|g__Bilophila.s__Bilophila_wadsworthia	+	1.96E-02	0.000	0.425	0.425	0.003	0.006
	4.1.99.12|g__Bacteroides.s__Bacteroides_uniformis	+	4.12E-01	4.475	5.353	0.878	0.053	0.072
	4.1.99.12|unclassified	+	3.86E-01	15.094	16.877	1.784	0.171	0.196
	4.1.99.12|g__Alistipes.s__Alistipes_putredinis	+	3.31E-02	0.000	2.267	2.267	0.018	0.033
USA	4.1.99.12	-	7.95E-03	130.060	123.561	-6.499	1.000	1.000
	4.1.99.12|g__Faecalibacterium.s__Faecalibacterium_prausnitzii	-	1.35E-05	5.807	2.185	-3.621	0.053	0.039
	4.1.99.12|g__Bacteroides.s__Bacteroides_vulgatus	-	7.69E-02	11.340	8.395	-2.945	0.110	0.099
	4.1.99.12|g__Blautia.s__Blautia_obeum	-	2.72E-09	5.628	3.749	-1.879	0.056	0.041
	4.1.99.12|g__Blautia.s__Ruminococcus_torques	-	1.80E-04	5.204	3.869	-1.335	0.055	0.043
	4.1.99.12|g__Lachnospiraceae_unclassified.s__Eubacterium_rectale	-	6.46E-04	0.944	0.202	-0.741	0.018	0.013
	4.1.99.12|g__Bacteroides.s__Bacteroides_uniformis	-	8.39E-01	7.455	6.879	-0.577	0.084	0.088
	4.1.99.12|g__Dorea.s__Dorea_longicatena	-	3.17E-02	0.566	0.054	-0.512	0.011	0.009
	4.1.99.12|g__Blautia.s__Blautia_wexlerae	-	3.02E-08	1.162	0.694	-0.468	0.013	0.010
	4.1.99.12|g__Alistipes.s__Alistipes_putredinis	-	5.74E-01	3.299	2.856	-0.444	0.032	0.031
	4.1.99.12|g__Roseburia.s__Roseburia_intestinalis	-	2.33E-06	0.242	0.000	-0.242	0.009	0.004
	4.1.99.12|g__Roseburia.s__Roseburia_faecis	-	1.37E-03	0.215	0.000	-0.215	0.016	0.010
	4.1.99.12|g__Agathobaculum.s__Agathobaculum_butyriciproducens	-	1.27E-03	0.180	0.000	-0.180	0.003	0.002
	4.1.99.12|g__Asaccharobacter.s__Asaccharobacter_celatus	-	9.52E-01	0.163	0.128	-0.035	0.003	0.003

4.1.99.12|g__Lachnoclostridium.s__Clostridium_bolteae	-	9.58E-01	0.027	0.000	-0.027	0.002	0.002
4.1.99.12|g__Bacteroides.s__Bacteroides_ovatus	+	5.80E-01	0.505	0.481	-0.024	0.015	0.014
4.1.99.12|g__Hungatella.s__Hungatella_hathewayi	-	4.08E-01	0.017	0.000	-0.017	0.003	0.002
4.1.99.12|g__Acidaminococcus.s__Acidaminococcus_fermentans	+	2.32E-01	0.000	0.000	0.000	0.000	0.000
4.1.99.12|g__Acidaminococcus.s__Acidaminococcus_intestini	+	1.95E-03	0.000	0.000	0.000	0.005	0.009
4.1.99.12|g__Actinomyces.s__Actinomyces_johnsonii	-	9.73E-01	0.000	0.000	0.000	0.000	0.000
4.1.99.12|g__Actinomyces.s__Actinomyces_naeslundii	+	3.07E-02	0.000	0.000	0.000	0.000	0.000
4.1.99.12|g__Actinomyces.s__Actinomyces_oris	+	1.48E-02	0.000	0.000	0.000	0.000	0.000
4.1.99.12|g__Actinomyces.s__Actinomyces_sp_oral_taxon_448	+	7.60E-01	0.000	0.000	0.000	0.000	0.000
4.1.99.12|g__Actinomyces.s__Actinomyces_viscosus	+	7.40E-03	0.000	0.000	0.000	0.000	0.000
4.1.99.12|g__Adlercreutzia.s__Adlercreutzia_equolifaciens	-	9.35E-01	0.000	0.000	0.000	0.001	0.001
4.1.99.12|g__Aeromonas.s__Aeromonas_allosaccharophila	-	1.48E-01	0.000	0.000	0.000	0.000	0.000
4.1.99.12|g__Aeromonas.s__Aeromonas_caviae	+	4.92E-01	0.000	0.000	0.000	0.000	0.000
4.1.99.12|g__Aeromonas.s__Aeromonas_diversa	-	1.48E-01	0.000	0.000	0.000	0.000	0.000
4.1.99.12|g__Aeromonas.s__Aeromonas_hydrophila	+	4.92E-01	0.000	0.000	0.000	0.000	0.000
4.1.99.12|g__Aggregatibacter.s__Aggregatibacter_segnis	+	4.92E-01	0.000	0.000	0.000	0.000	0.000
4.1.99.12|g__Akkermansia.s__Akkermansia_muciniphila	+	3.05E-01	0.000	0.000	0.000	0.020	0.035
4.1.99.12|g__Alistipes.s__Alistipes_indistinctus	+	7.40E-04	0.000	0.000	0.000	0.000	0.001
4.1.99.12|g__Alistipes.s__Alistipes_inops	+	1.65E-01	0.000	0.000	0.000	0.000	0.001
4.1.99.12|g__Alistipes.s__Alistipes_sp_An66	-	1.48E-01	0.000	0.000	0.000	0.000	0.000
4.1.99.12|g__Alistipes.s__Alistipes_timonensis	+	3.03E-01	0.000	0.000	0.000	0.001	0.001
4.1.99.12|g__Allisonella.s__Allisonella_histaminiformans	+	7.64E-01	0.000	0.000	0.000	0.000	0.000
4.1.99.12|g__Anaeroglobus.s__Anaeroglobus_geminatus	-	7.52E-01	0.000	0.000	0.000	0.000	0.000
4.1.99.12|g__Anaeromassilibacillus.s__Anaeromassilibacillus_sp_An250	-	9.39E-01	0.000	0.000	0.000	0.000	0.000
4.1.99.12|g__Anaerostipes.s__Anaerostipes_caccae	+	2.41E-01	0.000	0.000	0.000	0.001	0.001
4.1.99.12|g__Anaerostipes.s__Anaerostipes_hadrus	-	3.28E-02	0.000	0.000	0.000	0.004	0.003
4.1.99.12|g__Anaerostipes.s__Anaerostipes_sp_494a	-	1.48E-01	0.000	0.000	0.000	0.000	0.000
4.1.99.12|g__Anaerotignum.s__Anaerotignum_lactatifermentans	-	2.00E-01	0.000	0.000	0.000	0.001	0.001
4.1.99.12|g__Bacteroidales_unclassified.s__Bacteroidales_bacterium_KA00	+	4.92E-01	0.000	0.000	0.000	0.000	0.000
4.1.99.12|g__Bacteroides.s__Bacteroides_clarus	+	7.72E-01	0.000	0.000	0.000	0.001	0.001
4.1.99.12|g__Bacteroides.s__Bacteroides_coprocola	-	3.10E-02	0.000	0.000	0.000	0.003	0.001
4.1.99.12|g__Bacteroides.s__Bacteroides_coprophilus	+	4.06E-01	0.000	0.000	0.000	0.000	0.000
4.1.99.12|g__Bacteroides.s__Bacteroides_eggerthii	+	7.08E-01	0.000	0.000	0.000	0.010	0.008
4.1.99.12|g__Bacteroides.s__Bacteroides_faecichinchillae	+	1.22E-01	0.000	0.000	0.000	0.000	0.000
4.1.99.12|g__Bacteroides.s__Bacteroides_faecis	+	5.33E-02	0.000	0.000	0.000	0.002	0.002
4.1.99.12|g__Bacteroides.s__Bacteroides_finegoldii	+	6.70E-01	0.000	0.000	0.000	0.001	0.002
4.1.99.12|g__Bacteroides.s__Bacteroides_fluxus	+	2.32E-01	0.000	0.000	0.000	0.000	0.000
4.1.99.12|g__Bacteroides.s__Bacteroides_fragilis	-	5.26E-01	0.000	0.000	0.000	0.006	0.007
4.1.99.12|g__Bacteroides.s__Bacteroides_intestinalis	+	1.74E-01	0.000	0.000	0.000	0.002	0.005
4.1.99.12|g__Bacteroides.s__Bacteroides_massiliensis	+	4.13E-01	0.000	0.000	0.000	0.008	0.007
4.1.99.12|g__Bacteroides.s__Bacteroides_nordii	-	1.65E-01	0.000	0.000	0.000	0.001	0.000
4.1.99.12|g__Bacteroides.s__Bacteroides_oleiciplenus	+	4.92E-01	0.000	0.000	0.000	0.000	0.000
4.1.99.12|g__Bacteroides.s__Bacteroides_plebeius	-	3.80E-01	0.000	0.000	0.000	0.010	0.003
4.1.99.12|g__Bacteroides.s__Bacteroides_salyersiae	+	4.24E-02	0.000	0.000	0.000	0.001	0.002
4.1.99.12|g__Bacteroides.s__Bacteroides_sartorii	-	4.06E-02	0.000	0.000	0.000	0.000	0.000
4.1.99.12|g__Bacteroides.s__Bacteroides_sp_OM08_11	+	4.92E-01	0.000	0.000	0.000	0.000	0.000
4.1.99.12|g__Bacteroides.s__Bacteroides_stercorirosoris	-	9.57E-01	0.000	0.000	0.000	0.000	0.000
4.1.99.12|g__Barnesiella.s__Barnesiella_intestinihominis	+	1.29E-01	0.000	0.000	0.000	0.004	0.005
4.1.99.12|g__Bifidobacterium.s__Bifidobacterium_longum	+	2.32E-01	0.000	0.000	0.000	0.000	0.000
4.1.99.12|g__Blautia.s__Blautia_hansenii	-	1.57E-01	0.000	0.000	0.000	0.001	0.001
4.1.99.12|g__Blautia.s__Blautia_sp_AF19_10LB	+	5.37E-01	0.000	0.000	0.000	0.000	0.001
4.1.99.12|g__Blautia.s__Blautia_sp_An249	-	1.12E-01	0.000	0.000	0.000	0.000	0.000
4.1.99.12|g__Brevibacterium.s__Brevibacterium_ravenspurgense	+	4.92E-01	0.000	0.000	0.000	0.000	0.000
4.1.99.12|g__Brochothrix.s__Brochothrix_thermosphacta	+	4.92E-01	0.000	0.000	0.000	0.000	0.000
4.1.99.12|g__Butyricicoccus.s__Butyricicoccus_pullicaecorum	-	1.39E-01	0.000	0.000	0.000	0.000	0.000
4.1.99.12|g__Butyricimonas.s__Butyricimonas_synergistica	-	1.48E-01	0.000	0.000	0.000	0.000	0.000
4.1.99.12|g__Butyricimonas.s__Butyricimonas_virosa	+	2.29E-02	0.000	0.000	0.000	0.000	0.001
4.1.99.12|g__Butyrivibrio.s__Butyrivibrio_crossotus	-	4.75E-01	0.000	0.000	0.000	0.002	0.001
4.1.99.12|g__Campylobacter.s__Campylobacter_gracilis	+	5.86E-01	0.000	0.000	0.000	0.000	0.000
4.1.99.12|g__Campylobacter.s__Campylobacter_hominis	+	3.30E-01	0.000	0.000	0.000	0.000	0.000
4.1.99.12|g__Campylobacter.s__Campylobacter_ureolyticus	+	4.92E-01	0.000	0.000	0.000	0.000	0.000
4.1.99.12|g__Candida.s__Candida_albicans	-	9.67E-01	0.000	0.000	0.000	0.000	0.000
4.1.99.12|g__Candidatus_Gastranaerophilales_unclassified.s__Candidatus_(	+	2.81E-02	0.000	0.000	0.000	0.000	0.000
4.1.99.12|g__Candidatus_Methanomethylophilus.s__Candidatus_Methanom	+	3.30E-01	0.000	0.000	0.000	0.000	0.000
4.1.99.12|g__Catenibacterium.s__Catenibacterium_mitsuokai	+	5.16E-01	0.000	0.000	0.000	0.002	0.002
4.1.99.12|g__Cellulosilyticum.s__Cellulosilyticum_lentocellum	+	4.92E-01	0.000	0.000	0.000	0.000	0.000
4.1.99.12|g__Cetobacterium.s__Cetobacterium_somerae	+	4.92E-01	0.000	0.000	0.000	0.000	0.000
4.1.99.12|g__Citrobacter.s__Citrobacter_amalonaticus	+	1.32E-01	0.000	0.000	0.000	0.000	0.000
4.1.99.12|g__Citrobacter.s__Citrobacter_braakii	+	2.47E-01	0.000	0.000	0.000	0.000	0.000
4.1.99.12|g__Citrobacter.s__Citrobacter_farmeri	+	2.32E-01	0.000	0.000	0.000	0.000	0.000
4.1.99.12|g__Citrobacter.s__Citrobacter_freundii	+	9.39E-01	0.000	0.000	0.000	0.001	0.000
4.1.99.12|g__Citrobacter.s__Citrobacter_koseri	-	5.95E-01	0.000	0.000	0.000	0.000	0.000

4.1.99.12|g__Citrobacter.s__Citrobacter_portucalensis	+	3.31E-02	0.000	0.000	0.000	0.000	0.000
4.1.99.12|g__Citrobacter.s__Citrobacter_werkmanii	-	9.70E-01	0.000	0.000	0.000	0.000	0.000
4.1.99.12|g__Citrobacter.s__Citrobacter_youngae	+	1.75E-01	0.000	0.000	0.000	0.000	0.000
4.1.99.12|g__Cloacibacillus.s__Cloacibacillus_porcorum	+	7.50E-02	0.000	0.000	0.000	0.000	0.001
4.1.99.12|g__Clostridiales_Family_XIII_Incertae_Sedis_unclassified.s__Clo	+	4.92E-01	0.000	0.000	0.000	0.000	0.000
4.1.99.12|g__Clostridiales_Family_XIII_Incertae_Sedis_unclassified.s__Eub	+	4.92E-01	0.000	0.000	0.000	0.000	0.000
4.1.99.12|g__Clostridiales_Family_XIII_Incertae_Sedis_unclassified.s__Eub	+	4.92E-01	0.000	0.000	0.000	0.000	0.000
4.1.99.12|g__Clostridiales_unclassified.s__Clostridiales_bacterium_1_7_47F	+	7.71E-01	0.000	0.000	0.000	0.000	0.000
4.1.99.12|g__Clostridioides.s__Clostridioides_difficile	+	2.26E-03	0.000	0.000	0.000	0.003	0.003
4.1.99.12|g__Clostridium.s__Butyribacterium_methylotrophicum	+	8.86E-04	0.000	0.000	0.000	0.000	0.001
4.1.99.12|g__Clostridium.s__Clostridium_butyricum	-	1.48E-01	0.000	0.000	0.000	0.000	0.000
4.1.99.12|g__Clostridium.s__Clostridium_cadaveris	-	5.95E-01	0.000	0.000	0.000	0.000	0.000
4.1.99.12|g__Clostridium.s__Clostridium_celatum	-	1.48E-01	0.000	0.000	0.000	0.000	0.000
4.1.99.12|g__Clostridium.s__Clostridium_disporicum	+	5.05E-01	0.000	0.000	0.000	0.000	0.000
4.1.99.12|g__Clostridium.s__Clostridium_perfringens	-	5.48E-01	0.000	0.000	0.000	0.000	0.000
4.1.99.12|g__Clostridium.s__Clostridium_sp_AF36_4	-	2.50E-01	0.000	0.000	0.000	0.002	0.002
4.1.99.12|g__Clostridium.s__Clostridium_sp_AM22_11AC	-	2.31E-03	0.000	0.000	0.000	0.002	0.001
4.1.99.12|g__Clostridium.s__Clostridium_sp_MSTE9	+	4.92E-01	0.000	0.000	0.000	0.000	0.000
4.1.99.12|g__Clostridium.s__Clostridium_sp_chh4_2	-	5.92E-01	0.000	0.000	0.000	0.000	0.000
4.1.99.12|g__Clostridium.s__Clostridium_ventriculi	-	5.92E-01	0.000	0.000	0.000	0.000	0.000
4.1.99.12|g__Comamonas.s__Comamonas_kerstersii	+	3.30E-01	0.000	0.000	0.000	0.000	0.000
4.1.99.12|g__Coprobacillus.s__Coprobacillus_cateniformis	+	2.65E-04	0.000	0.000	0.000	0.000	0.001
4.1.99.12|g__Coprobacter.s__Coprobacter_fastidiosus	-	1.98E-02	0.000	0.000	0.000	0.000	0.000
4.1.99.12|g__Coprobacter.s__Coprobacter_secundus	-	2.81E-01	0.000	0.000	0.000	0.000	0.000
4.1.99.12|g__Coprococcus.s__Coprococcus_catus	+	2.87E-01	0.000	0.000	0.000	0.001	0.001
4.1.99.12|g__Coprococcus.s__Coprococcus_comes	-	8.37E-01	0.000	0.000	0.000	0.005	0.005
4.1.99.12|g__Coprococcus.s__Coprococcus_eutactus	-	4.67E-01	0.000	0.000	0.000	0.003	0.002
4.1.99.12|g__Corynebacterium.s__Corynebacterium_amycolatum	+	4.92E-01	0.000	0.000	0.000	0.000	0.000
4.1.99.12|g__Corynebacterium.s__Corynebacterium_coyleae	+	4.92E-01	0.000	0.000	0.000	0.000	0.000
4.1.99.12|g__Corynebacterium.s__Corynebacterium_frankenforstense	-	1.48E-01	0.000	0.000	0.000	0.000	0.000
4.1.99.12|g__Corynebacterium.s__Corynebacterium_imitans	+	4.92E-01	0.000	0.000	0.000	0.000	0.000
4.1.99.12|g__Corynebacterium.s__Corynebacterium_jeikeium	+	4.92E-01	0.000	0.000	0.000	0.000	0.000
4.1.99.12|g__Corynebacterium.s__Corynebacterium_kroppenstedtii	+	4.92E-01	0.000	0.000	0.000	0.000	0.000
4.1.99.12|g__Corynebacterium.s__Corynebacterium_pseudogenitalium	+	4.92E-01	0.000	0.000	0.000	0.000	0.000
4.1.99.12|g__Corynebacterium.s__Corynebacterium_riegelii	+	4.92E-01	0.000	0.000	0.000	0.000	0.000
4.1.99.12|g__Corynebacterium.s__Corynebacterium_tuberculostearicum	+	4.92E-01	0.000	0.000	0.000	0.000	0.000
4.1.99.12|g__Corynebacterium.s__Corynebacterium_urealyticum	+	4.92E-01	0.000	0.000	0.000	0.000	0.000
4.1.99.12|g__Cronobacter.s__Cronobacter_malonaticus	+	4.92E-01	0.000	0.000	0.000	0.000	0.000
4.1.99.12|g__Cronobacter.s__Cronobacter_sakazakii	+	4.92E-01	0.000	0.000	0.000	0.000	0.000
4.1.99.12|g__Delftia.s__Delftia_lacustris	+	4.92E-01	0.000	0.000	0.000	0.000	0.000
4.1.99.12|g__Delftia.s__Delftia_tsuruhatensis	+	4.92E-01	0.000	0.000	0.000	0.000	0.000
4.1.99.12|g__Desulfovibrio.s__Desulfovibrio_desulfuricans	+	5.55E-01	0.000	0.000	0.000	0.000	0.000
4.1.99.12|g__Desulfovibrio.s__Desulfovibrio_fairfieldensis	+	2.75E-01	0.000	0.000	0.000	0.002	0.004
4.1.99.12|g__Desulfovibrio.s__Desulfovibrio_legallii	+	4.92E-01	0.000	0.000	0.000	0.000	0.000
4.1.99.12|g__Desulfovibrio.s__Desulfovibrio_piger	+	1.19E-01	0.000	0.000	0.000	0.000	0.001
4.1.99.12|g__Desulfovibrio.s__Desulfovibrio_sp_AM18_2	+	1.72E-01	0.000	0.000	0.000	0.000	0.000
4.1.99.12|g__Desulfovibrionaceae_unclassified.s__Desulfovibrionaceae_bac	+	2.40E-01	0.000	0.000	0.000	0.000	0.000
4.1.99.12|g__Dialister.s__Dialister_invisus	-	8.16E-03	0.000	0.000	0.000	0.004	0.002
4.1.99.12|g__Dialister.s__Dialister_pneumosintes	+	4.92E-01	0.000	0.000	0.000	0.000	0.000
4.1.99.12|g__Dialister.s__Dialister_succinatiphilus	+	4.92E-01	0.000	0.000	0.000	0.000	0.000
4.1.99.12|g__Dorea.s__Dorea_formicigenerans	+	9.08E-01	0.000	0.000	0.000	0.002	0.002
4.1.99.12|g__Dorea.s__Dorea_sp_OM02_2LB	+	6.36E-01	0.000	0.000	0.000	0.000	0.000
4.1.99.12|g__Dysgonomonas.s__Dysgonomonas_mossii	+	4.92E-01	0.000	0.000	0.000	0.000	0.000
4.1.99.12|g__Enterobacter.s__Enterobacter_bugandensis	+	4.16E-01	0.000	0.000	0.000	0.000	0.000
4.1.99.12|g__Enterobacter.s__Enterobacter_cloacae	+	9.75E-02	0.000	0.000	0.000	0.000	0.000
4.1.99.12|g__Enterobacter.s__Enterobacter_mori	+	4.92E-01	0.000	0.000	0.000	0.000	0.000
4.1.99.12|g__Enterococcus.s__Enterococcus_avium	+	2.44E-05	0.000	0.000	0.000	0.000	0.001
4.1.99.12|g__Enterococcus.s__Enterococcus_casseliflavus	+	4.12E-01	0.000	0.000	0.000	0.000	0.000
4.1.99.12|g__Enterococcus.s__Enterococcus_faecalis	+	3.30E-01	0.000	0.000	0.000	0.000	0.000
4.1.99.12|g__Enterococcus.s__Enterococcus_faecium	+	3.38E-01	0.000	0.000	0.000	0.000	0.000
4.1.99.12|g__Enterococcus.s__Enterococcus_gallinarum	+	3.74E-02	0.000	0.000	0.000	0.000	0.000
4.1.99.12|g__Enterococcus.s__Enterococcus_saccharolyticus	+	6.68E-02	0.000	0.000	0.000	0.000	0.000
4.1.99.12|g__Erysipelatoclostridium.s__Clostridium_innocuum	+	3.29E-01	0.000	0.000	0.000	0.003	0.001
4.1.99.12|g__Erysipelatoclostridium.s__Clostridium_spiroforme	+	3.75E-01	0.000	0.000	0.000	0.001	0.000
4.1.99.12|g__Erysipelotrichaceae_unclassified.s__Erysipelotrichaceae_bacte	+	5.29E-01	0.000	0.000	0.000	0.001	0.000
4.1.99.12|g__Escherichia.s__Escherichia_coli	+	5.10E-03	0.000	0.000	0.000	0.017	0.025
4.1.99.12|g__Escherichia.s__Escherichia_fergusonii	+	3.01E-01	0.000	0.000	0.000	0.000	0.000
4.1.99.12|g__Escherichia.s__Escherichia_marmotae	+	4.92E-01	0.000	0.000	0.000	0.000	0.000
4.1.99.12|g__Eubacterium.s__Eubacterium_callanderi	+	7.64E-04	0.000	0.000	0.000	0.000	0.001
4.1.99.12|g__Eubacterium.s__Eubacterium_eligens	-	4.07E-02	0.000	0.000	0.000	0.005	0.004
4.1.99.12|g__Eubacterium.s__Eubacterium_limosum	+	1.18E-01	0.000	0.000	0.000	0.000	0.000
4.1.99.12|g__Eubacterium.s__Eubacterium_maltosivorans	+	2.48E-01	0.000	0.000	0.000	0.000	0.000

4.1.99.12|g__Eubacterium.s__Eubacterium_ramulus	-	4.53E-02	0.000	0.000	0.000	0.002	0.002
4.1.99.12|g__Eubacterium.s__Eubacterium_sp_AF17_7	-	9.79E-01	0.000	0.000	0.000	0.000	0.000
4.1.99.12|g__Eubacterium.s__Eubacterium_sp_AM18_10LB_B	-	2.95E-01	0.000	0.000	0.000	0.000	0.000
4.1.99.12|g__Eubacterium.s__Eubacterium_sp_An11	+	6.61E-01	0.000	0.000	0.000	0.000	0.000
4.1.99.12|g__Faecalitalea.s__Faecalitalea_cylindroides	+	1.71E-01	0.000	0.000	0.000	0.000	0.000
4.1.99.12|g__Firmicutes_unclassified.s__Firmicutes_bacterium_AM10_47	+	9.91E-01	0.000	0.000	0.000	0.000	0.000
4.1.99.12|g__Flavonifractor.s__Flavonifractor_plautii	+	4.80E-02	0.000	0.000	0.000	0.001	0.001
4.1.99.12|g__Flavonifractor.s__Flavonifractor_sp_An10	+	1.08E-01	0.000	0.000	0.000	0.000	0.001
4.1.99.12|g__Fusobacterium.s__Fusobacterium_mortiferum	-	9.57E-01	0.000	0.000	0.000	0.000	0.000
4.1.99.12|g__Fusobacterium.s__Fusobacterium_nucleatum	-	5.92E-01	0.000	0.000	0.000	0.000	0.000
4.1.99.12|g__Fusobacterium.s__Fusobacterium_ulcerans	-	4.27E-01	0.000	0.000	0.000	0.000	0.000
4.1.99.12|g__Fusobacterium.s__Fusobacterium_varium	+	4.92E-01	0.000	0.000	0.000	0.000	0.000
4.1.99.12|g__Gemella.s__Gemella_haemolysans	+	4.92E-01	0.000	0.000	0.000	0.000	0.000
4.1.99.12|g__Haemophilus.s__Haemophilus_parainfluenzae	-	1.82E-01	0.000	0.000	0.000	0.000	0.000
4.1.99.12|g__Hafnia.s__Hafnia_paralvei	-	5.95E-01	0.000	0.000	0.000	0.000	0.000
4.1.99.12|g__Holdemanella.s__Holdemanella_biformis	-	4.47E-01	0.000	0.000	0.000	0.000	0.000
4.1.99.12|g__Intestinibacter.s__Intestinibacter_bartlettii	+	9.77E-01	0.000	0.000	0.000	0.000	0.000
4.1.99.12|g__Klebsiella.s__Klebsiella_aerogenes	+	5.27E-01	0.000	0.000	0.000	0.000	0.000
4.1.99.12|g__Klebsiella.s__Klebsiella_grimontii	-	9.70E-01	0.000	0.000	0.000	0.000	0.000
4.1.99.12|g__Klebsiella.s__Klebsiella_michiganensis	+	8.38E-01	0.000	0.000	0.000	0.000	0.000
4.1.99.12|g__Klebsiella.s__Klebsiella_oxytoca	+	5.61E-02	0.000	0.000	0.000	0.002	0.003
4.1.99.12|g__Klebsiella.s__Klebsiella_pneumoniae	+	1.63E-02	0.000	0.000	0.000	0.005	0.005
4.1.99.12|g__Klebsiella.s__Klebsiella_variicola	+	3.57E-02	0.000	0.000	0.000	0.001	0.002
4.1.99.12|g__Kluyvera.s__Kluyvera_ascorbata	+	7.55E-01	0.000	0.000	0.000	0.000	0.000
4.1.99.12|g__Kluyvera.s__Kluyvera_cryocrescens	+	3.30E-01	0.000	0.000	0.000	0.000	0.000
4.1.99.12|g__Kluyvera.s__Kluyvera_intermedia	+	4.92E-01	0.000	0.000	0.000	0.000	0.000
4.1.99.12|g__Kosakonia.s__Kosakonia_cowanii	+	4.92E-01	0.000	0.000	0.000	0.000	0.000
4.1.99.12|g__Lachnoclostridium.s__Clostridium_aldenense	-	5.40E-01	0.000	0.000	0.000	0.000	0.001
4.1.99.12|g__Lachnoclostridium.s__Clostridium_citroniae	+	1.11E-01	0.000	0.000	0.000	0.000	0.000
4.1.99.12|g__Lachnoclostridium.s__Clostridium_clostridioforme	+	7.05E-01	0.000	0.000	0.000	0.002	0.003
4.1.99.12|g__Lachnoclostridium.s__Clostridium_symbiosum	+	4.60E-01	0.000	0.000	0.000	0.001	0.001
4.1.99.12|g__Lachnoclostridium.s__Lachnoclostridium_sp_An138	+	2.25E-01	0.000	0.000	0.000	0.000	0.000
4.1.99.12|g__Lachnospira.s__Lachnospira_pectinoschiza	+	8.54E-01	0.000	0.000	0.000	0.001	0.001
4.1.99.12|g__Lachnospiraceae_unclassified.s__Lachnospiraceae_bacterium_	+	4.11E-02	0.000	0.000	0.000	0.000	0.000
4.1.99.12|g__Lactobacillus.s__Lactobacillus_amylovorus	-	1.48E-01	0.000	0.000	0.000	0.000	0.000
4.1.99.12|g__Lactobacillus.s__Lactobacillus_delbrueckii	-	9.70E-01	0.000	0.000	0.000	0.000	0.000
4.1.99.12|g__Lactobacillus.s__Lactobacillus_fermentum	+	5.39E-02	0.000	0.000	0.000	0.000	0.001
4.1.99.12|g__Lactobacillus.s__Lactobacillus_mucosae	+	7.57E-01	0.000	0.000	0.000	0.001	0.000
4.1.99.12|g__Lactobacillus.s__Lactobacillus_oris	+	1.47E-02	0.000	0.000	0.000	0.000	0.001
4.1.99.12|g__Lactobacillus.s__Lactobacillus_reuteri	+	1.43E-02	0.000	0.000	0.000	0.000	0.002
4.1.99.12|g__Lactobacillus.s__Lactobacillus_rogosae	-	9.52E-01	0.000	0.000	0.000	0.001	0.001
4.1.99.12|g__Lactobacillus.s__Lactobacillus_vaccinostercus	+	1.67E-01	0.000	0.000	0.000	0.000	0.000
4.1.99.12|g__Lactococcus.s__Lactococcus_lactis	+	4.39E-01	0.000	0.000	0.000	0.000	0.000
4.1.99.12|g__Lactococcus.s__Lactococcus_piscium	+	4.92E-01	0.000	0.000	0.000	0.000	0.000
4.1.99.12|g__Leclercia.s__Leclercia_adecarboxylata	+	2.31E-01	0.000	0.000	0.000	0.000	0.000
4.1.99.12|g__Lelliottia.s__Lelliottia_amnigena	+	2.32E-01	0.000	0.000	0.000	0.000	0.000
4.1.99.12|g__Lelliottia.s__Lelliottia_nimipressuralis	+	3.10E-01	0.000	0.000	0.000	0.000	0.000
4.1.99.12|g__Megamonas.s__Megamonas_funiformis	+	9.91E-01	0.000	0.000	0.000	0.001	0.001
4.1.99.12|g__Megamonas.s__Megamonas_hypermegale	+	4.92E-01	0.000	0.000	0.000	0.000	0.000
4.1.99.12|g__Megamonas.s__Megamonas_rupellensis	-	7.81E-01	0.000	0.000	0.000	0.000	0.000
4.1.99.12|g__Megasphaera.s__Megasphaera_elsdenii	-	9.60E-01	0.000	0.000	0.000	0.000	0.000
4.1.99.12|g__Megasphaera.s__Megasphaera_hexanoica	+	4.92E-01	0.000	0.000	0.000	0.000	0.000
4.1.99.12|g__Megasphaera.s__Megasphaera_micronuciformis	+	3.30E-01	0.000	0.000	0.000	0.000	0.000
4.1.99.12|g__Megasphaera.s__Megasphaera_sp_DISK_18	+	4.25E-04	0.000	0.000	0.000	0.000	0.002
4.1.99.12|g__Megasphaera.s__Megasphaera_sp_MJR8396C	+	8.66E-02	0.000	0.000	0.000	0.001	0.002
4.1.99.12|g__Megasphaera.s__Megasphaera_stantonii	-	5.92E-01	0.000	0.000	0.000	0.000	0.000
4.1.99.12|g__Methanobrevibacter.s__Methanobrevibacter_arboriphilus	+	4.92E-01	0.000	0.000	0.000	0.000	0.000
4.1.99.12|g__Methanobrevibacter.s__Methanobrevibacter_smithii	+	5.01E-04	0.000	0.000	0.000	0.005	0.010
4.1.99.12|g__Methanomassiliicoccus.s__Candidatus_Methanomassiliicoccus	+	3.27E-03	0.000	0.000	0.000	0.000	0.000
4.1.99.12|g__Methanosphaera.s__Methanosphaera_stadtmanae	+	4.85E-01	0.000	0.000	0.000	0.000	0.000
4.1.99.12|g__Mitsuokella.s__Mitsuokella_jalaludinii	-	5.95E-01	0.000	0.000	0.000	0.000	0.000
4.1.99.12|g__Mitsuokella.s__Mitsuokella_multacida	+	4.92E-01	0.000	0.000	0.000	0.000	0.000
4.1.99.12|g__Mogibacterium.s__Mogibacterium_diversum	+	1.22E-01	0.000	0.000	0.000	0.000	0.000
4.1.99.12|g__Mogibacterium.s__Mogibacterium_timidum	+	4.92E-01	0.000	0.000	0.000	0.000	0.000
4.1.99.12|g__Morganella.s__Morganella_morganii	+	3.74E-02	0.000	0.000	0.000	0.000	0.000
4.1.99.12|g__Muribaculum.s__Muribaculum_intestinale	-	5.92E-01	0.000	0.000	0.000	0.000	0.000
4.1.99.12|g__Odoribacter.s__Odoribacter_laneus	+	1.90E-01	0.000	0.000	0.000	0.001	0.003
4.1.99.12|g__Oligella.s__Oligella_urethralis	+	4.92E-01	0.000	0.000	0.000	0.000	0.000
4.1.99.12|g__Oxalobacter.s__Oxalobacter_formigenes	+	3.10E-01	0.000	0.000	0.000	0.000	0.000
4.1.99.12|g__Pantoea.s__Pantoea_sesami	+	2.33E-01	0.000	0.000	0.000	0.000	0.000
4.1.99.12|g__Parabacteroides.s__Parabacteroides_goldsteinii	+	2.64E-02	0.000	0.000	0.000	0.002	0.003
4.1.99.12|g__Parabacteroides.s__Parabacteroides_gordonii	-	9.23E-01	0.000	0.000	0.000	0.000	0.000

4.1.99.12|g__Parabacteroides.s__Parabacteroides_johnsonii	-	3.66E-01	0.000	0.000	0.000	0.001	0.000
4.1.99.12|g__Paraprevotella.s__Paraprevotella_clara	-	1.18E-01	0.000	0.000	0.000	0.003	0.002
4.1.99.12|g__Paraprevotella.s__Paraprevotella_xylaniphila	+	4.16E-01	0.000	0.000	0.000	0.000	0.000
4.1.99.12|g__Parasutterella.s__Parasutterella_excrementihominis	-	4.93E-01	0.000	0.000	0.000	0.002	0.002
4.1.99.12|g__Pedobacter.s__Pedobacter_himalayensis	+	2.32E-01	0.000	0.000	0.000	0.000	0.000
4.1.99.12|g__Peptococcus.s__Peptococcus_niger	+	2.31E-01	0.000	0.000	0.000	0.000	0.000
4.1.99.12|g__Peptoniphilus.s__Peptoniphilus_coxii	-	1.48E-01	0.000	0.000	0.000	0.000	0.000
4.1.99.12|g__Peptoniphilus.s__Peptoniphilus_duerdenii	+	4.92E-01	0.000	0.000	0.000	0.000	0.000
4.1.99.12|g__Peptoniphilus.s__Peptoniphilus_harei	+	1.67E-01	0.000	0.000	0.000	0.000	0.000
4.1.99.12|g__Peptoniphilus.s__Peptoniphilus_lacrimalis	+	4.92E-01	0.000	0.000	0.000	0.000	0.000
4.1.99.12|g__Peptoniphilus.s__Peptoniphilus_sp_HMSC062D09	+	2.32E-01	0.000	0.000	0.000	0.000	0.000
4.1.99.12|g__Peptoniphilus.s__Peptoniphilus_sp_oral_taxon_375	+	4.92E-01	0.000	0.000	0.000	0.000	0.000
4.1.99.12|g__Peptostreptococcaceae_unclassified.s__Clostridium_hiranonis	-	5.92E-01	0.000	0.000	0.000	0.000	0.000
4.1.99.12|g__Peptostreptococcus.s__Peptostreptococcus_anaerobius	+	2.32E-01	0.000	0.000	0.000	0.000	0.000
4.1.99.12|g__Phascolarctobacterium.s__Phascolarctobacterium_succinatuter	+	1.82E-01	0.000	0.000	0.000	0.001	0.001
4.1.99.12|g__Phytobacter.s__Phytobacter_ursingii	+	4.92E-01	0.000	0.000	0.000	0.000	0.000
4.1.99.12|g__Pluralibacter.s__Pluralibacter_gergoviae	-	1.48E-01	0.000	0.000	0.000	0.000	0.000
4.1.99.12|g__Porphyromonas.s__Porphyromonas_asaccharolytica	+	5.65E-02	0.000	0.000	0.000	0.000	0.000
4.1.99.12|g__Porphyromonas.s__Porphyromonas_sp_HMSC065F10	+	7.55E-01	0.000	0.000	0.000	0.000	0.000
4.1.99.12|g__Porphyromonas.s__Porphyromonas_sp_HMSC077F02	-	9.64E-01	0.000	0.000	0.000	0.000	0.000
4.1.99.12|g__Porphyromonas.s__Porphyromonas_uenonis	-	9.70E-01	0.000	0.000	0.000	0.000	0.000
4.1.99.12|g__Prevotella.s__Prevotella_bergensis	+	4.92E-01	0.000	0.000	0.000	0.000	0.000
4.1.99.12|g__Prevotella.s__Prevotella_bivia	+	2.29E-01	0.000	0.000	0.000	0.000	0.000
4.1.99.12|g__Prevotella.s__Prevotella_buccae	-	9.73E-01	0.000	0.000	0.000	0.000	0.000
4.1.99.12|g__Prevotella.s__Prevotella_buccalis	+	4.99E-02	0.000	0.000	0.000	0.000	0.000
4.1.99.12|g__Prevotella.s__Prevotella_colorans	+	3.30E-01	0.000	0.000	0.000	0.000	0.000
4.1.99.12|g__Prevotella.s__Prevotella_copri	-	9.35E-01	0.000	0.000	0.000	0.001	0.001
4.1.99.12|g__Prevotella.s__Prevotella_corporis	-	4.35E-01	0.000	0.000	0.000	0.000	0.000
4.1.99.12|g__Prevotella.s__Prevotella_disiens	+	8.32E-01	0.000	0.000	0.000	0.000	0.000
4.1.99.12|g__Prevotella.s__Prevotella_histicola	+	4.92E-01	0.000	0.000	0.000	0.000	0.000
4.1.99.12|g__Prevotella.s__Prevotella_sp_109	-	7.86E-01	0.000	0.000	0.000	0.002	0.001
4.1.99.12|g__Prevotella.s__Prevotella_sp_885	-	4.45E-01	0.000	0.000	0.000	0.000	0.000
4.1.99.12|g__Prevotella.s__Prevotella_sp_AM42_24	-	2.20E-02	0.000	0.000	0.000	0.002	0.000
4.1.99.12|g__Prevotella.s__Prevotella_stercorea	-	4.75E-01	0.000	0.000	0.000	0.003	0.001
4.1.99.12|g__Prevotella.s__Prevotella_timonensis	+	9.79E-02	0.000	0.000	0.000	0.000	0.000
4.1.99.12|g__Propionibacterium.s__Propionibacterium_freudenreichii	+	4.92E-01	0.000	0.000	0.000	0.000	0.000
4.1.99.12|g__Proteus.s__Proteus_mirabilis	+	8.92E-01	0.000	0.000	0.000	0.000	0.000
4.1.99.12|g__Pseudocitrobacter.s__Pseudocitrobacter_faecalis	+	2.32E-01	0.000	0.000	0.000	0.000	0.000
4.1.99.12|g__Pseudoflavonifractor.s__Pseudoflavonifractor_sp_An184	+	1.09E-01	0.000	0.000	0.000	0.001	0.001
4.1.99.12|g__Pseudomonas.s__Pseudomonas_aeruginosa	+	1.32E-01	0.000	0.000	0.000	0.001	0.000
4.1.99.12|g__Pseudomonas.s__Pseudomonas_aeruginosa_group	+	7.55E-02	0.000	0.000	0.000	0.001	0.000
4.1.99.12|g__Pseudomonas.s__Pseudomonas_citronellolis	+	4.92E-01	0.000	0.000	0.000	0.000	0.000
4.1.99.12|g__Pseudomonas.s__Pseudomonas_delhiensis	+	4.92E-01	0.000	0.000	0.000	0.000	0.000
4.1.99.12|g__Pseudomonas.s__Pseudomonas_taiwanensis	+	4.92E-01	0.000	0.000	0.000	0.000	0.000
4.1.99.12|g__Pseudoramibacter.s__Pseudoramibacter_alactolyticus	+	4.92E-01	0.000	0.000	0.000	0.000	0.000
4.1.99.12|g__Pyramidobacter.s__Pyramidobacter_piscolens	+	1.29E-01	0.000	0.000	0.000	0.000	0.000
4.1.99.12|g__Pyramidobacter.s__Pyramidobacter_sp_C12_8	+	4.92E-01	0.000	0.000	0.000	0.000	0.000
4.1.99.12|g__Raoultella.s__Raoultella_ornithinolytica	-	9.57E-01	0.000	0.000	0.000	0.000	0.000
4.1.99.12|g__Raoultella.s__Raoultella_planticola	-	9.67E-01	0.000	0.000	0.000	0.000	0.000
4.1.99.12|g__Rikenella.s__Rikenella_microfusus	+	4.92E-01	0.000	0.000	0.000	0.000	0.000
4.1.99.12|g__Roseburia.s__Roseburia_inulinivorans	-	2.92E-01	0.000	0.000	0.000	0.001	0.001
4.1.99.12|g__Rothia.s__Rothia_dentocariosa	+	8.36E-01	0.000	0.000	0.000	0.000	0.000
4.1.99.12|g__Rothia.s__Rothia_mucilaginosa	-	9.54E-01	0.000	0.000	0.000	0.000	0.000
4.1.99.12|g__Ruminococcaceae_unclassified.s__Ruminococcaceae_bacteriu	+	6.81E-01	0.000	0.000	0.000	0.001	0.002
4.1.99.12|g__Ruminococcaceae_unclassified.s__Ruminococcaceae_bacteriu	+	1.08E-02	0.000	0.000	0.000	0.000	0.000
4.1.99.12|g__Ruminococcus.s__Ruminococcus_callidus	-	1.27E-03	0.000	0.000	0.000	0.003	0.001
4.1.99.12|g__Ruminococcus.s__Ruminococcus_sp_AF31_8BH	-	1.05E-02	0.000	0.000	0.000	0.001	0.001
4.1.99.12|g__Salmonella.s__Salmonella_enterica	+	3.04E-01	0.000	0.000	0.000	0.000	0.000
4.1.99.12|g__Sanguibacteroides.s__Sanguibacteroides_justesenii	+	9.00E-02	0.000	0.000	0.000	0.000	0.000
4.1.99.12|g__Senegalimassilia.s__Senegalimassilia_anaerobia	+	2.19E-01	0.000	0.000	0.000	0.000	0.000
4.1.99.12|g__Serratia.s__Serratia_nematodiphila	+	4.92E-01	0.000	0.000	0.000	0.000	0.000
4.1.99.12|g__Serratia.s__Serratia_ureilytica	+	4.92E-01	0.000	0.000	0.000	0.000	0.000
4.1.99.12|g__Slackia.s__Slackia_isoflavoniconvertens	+	7.39E-03	0.000	0.000	0.000	0.000	0.000
4.1.99.12|g__Staphylococcus.s__Staphylococcus_aureus	+	2.32E-01	0.000	0.000	0.000	0.000	0.000
4.1.99.12|g__Staphylococcus.s__Staphylococcus_haemolyticus	+	4.92E-01	0.000	0.000	0.000	0.000	0.000
4.1.99.12|g__Streptococcus.s__Streptococcus_agalactiae	+	4.92E-01	0.000	0.000	0.000	0.000	0.000
4.1.99.12|g__Streptococcus.s__Streptococcus_equinus	+	1.30E-02	0.000	0.000	0.000	0.000	0.001
4.1.99.12|g__Streptococcus.s__Streptococcus_gallolyticus	+	2.32E-03	0.000	0.000	0.000	0.000	0.000
4.1.99.12|g__Streptococcus.s__Streptococcus_infantarius	+	1.26E-02	0.000	0.000	0.000	0.000	0.001
4.1.99.12|g__Streptococcus.s__Streptococcus_infantis	-	1.48E-01	0.000	0.000	0.000	0.000	0.000
4.1.99.12|g__Streptococcus.s__Streptococcus_lutetiensis	+	1.25E-02	0.000	0.000	0.000	0.000	0.001
4.1.99.12|g__Streptococcus.s__Streptococcus_macedonicus	+	5.86E-02	0.000	0.000	0.000	0.000	0.002

	4.1.99.12|g__Streptococcus.s__Streptococcus_oralis	+	2.32E-01	0.000	0.000	0.000	0.000	0.000
	4.1.99.12|g__Streptococcus.s__Streptococcus_pasteurianus	+	7.39E-02	0.000	0.000	0.000	0.000	0.001
	4.1.99.12|g__Streptococcus.s__Streptococcus_pneumoniae	-	2.82E-01	0.000	0.000	0.000	0.000	0.000
	4.1.99.12|g__Sutterella.s__Sutterella_wadsworthensis	+	1.95E-01	0.000	0.000	0.000	0.002	0.002
	4.1.99.12|g__Terrisporobacter.s__Terrisporobacter_othiniensis	+	1.67E-01	0.000	0.000	0.000	0.000	0.000
	4.1.99.12|g__Trichococcus.s__Trichococcus_collinsii	+	4.92E-01	0.000	0.000	0.000	0.000	0.000
	4.1.99.12|g__Trichococcus.s__Trichococcus_flocculiformis	+	4.92E-01	0.000	0.000	0.000	0.000	0.000
	4.1.99.12|g__Trichococcus.s__Trichococcus_pasteurii	+	4.92E-01	0.000	0.000	0.000	0.000	0.000
	4.1.99.12|g__Turicimonas.s__Turicimonas_muris	-	9.64E-01	0.000	0.000	0.000	0.000	0.000
	4.1.99.12|g__Veillonella.s__Veillonella_atypica	-	7.91E-01	0.000	0.000	0.000	0.000	0.001
	4.1.99.12|g__Veillonella.s__Veillonella_dispar	-	4.28E-01	0.000	0.000	0.000	0.000	0.000
	4.1.99.12|g__Veillonella.s__Veillonella_infantium	+	4.99E-02	0.000	0.000	0.000	0.000	0.000
	4.1.99.12|g__Veillonella.s__Veillonella_parvula	+	6.97E-01	0.000	0.000	0.000	0.000	0.000
	4.1.99.12|g__Veillonella.s__Veillonella_rogosae	-	9.20E-01	0.000	0.000	0.000	0.000	0.000
	4.1.99.12|g__Veillonella.s__Veillonella_seminalis	-	4.47E-01	0.000	0.000	0.000	0.000	0.000
	4.1.99.12|g__Vibrio.s__Vibrio_parahaemolyticus	+	1.75E-01	0.000	0.000	0.000	0.000	0.000
	4.1.99.12|g__Victivallales_unclassified.s__Victivallales_bacterium_CCUG_	+	1.55E-03	0.000	0.000	0.000	0.000	0.000
	4.1.99.12|g__Victivallis.s__Victivallis_vadensis	+	4.16E-02	0.000	0.000	0.000	0.000	0.000
	4.1.99.12|g__Weeksella.s__Weeksella_virosa	+	4.92E-01	0.000	0.000	0.000	0.000	0.000
	4.1.99.12|g__Weissella.s__Weissella_cibaria	+	7.60E-01	0.000	0.000	0.000	0.000	0.000
	4.1.99.12|g__Weissella.s__Weissella_confusa	-	5.51E-01	0.000	0.000	0.000	0.000	0.000
	4.1.99.12|g__Bacteroides.s__Bacteroides_xylanisolvens	+	3.25E-01	0.000	0.015	0.015	0.007	0.005
	4.1.99.12|g__Odoribacter.s__Odoribacter_splanchnicus	+	3.98E-01	0.223	0.243	0.020	0.002	0.003
	4.1.99.12|g__Eggerthella.s__Eggerthella_lenta	+	2.14E-01	0.000	0.020	0.020	0.002	0.002
	4.1.99.12|g__Bacteroides.s__Bacteroides_thetaiotaomicron	+	4.82E-01	0.409	0.439	0.029	0.010	0.011
	4.1.99.12|g__Bacteroides.s__Bacteroides_dorei	-	9.79E-01	1.083	1.118	0.034	0.025	0.030
	4.1.99.12|g__Roseburia.s__Roseburia_hominis	-	8.99E-01	1.485	1.546	0.061	0.015	0.017
	4.1.99.12|g__Phascolarctobacterium.s__Phascolarctobacterium_faecium	+	7.34E-01	0.475	0.564	0.089	0.007	0.008
	4.1.99.12|g__Bacteroides.s__Bacteroides_cellulosilyticus	+	4.07E-01	0.131	0.245	0.115	0.013	0.013
	4.1.99.12|g__Bacteroides.s__Bacteroides_caccae	+	2.33E-01	0.229	0.346	0.117	0.006	0.009
	4.1.99.12|g__Parabacteroides.s__Parabacteroides_merdae	+	7.65E-02	0.523	0.699	0.176	0.007	0.008
	4.1.99.12|g__Alistipes.s__Alistipes_onderdonkii	+	3.01E-03	0.435	0.647	0.211	0.010	0.014
	4.1.99.12|g__Alistipes.s__Alistipes_finegoldii	+	2.20E-03	0.369	0.581	0.213	0.008	0.012
	4.1.99.12|g__Parabacteroides.s__Parabacteroides_distasonis	+	1.52E-02	1.054	1.348	0.295	0.017	0.019
	4.1.99.12|g__Bilophila.s__Bilophila_wadsworthia	+	3.70E-03	0.536	0.877	0.341	0.007	0.010
	4.1.99.12|g__Bacteroides.s__Bacteroides_stercoris	+	4.32E-02	0.044	0.478	0.435	0.033	0.039
	4.1.99.12|unclassified	+	9.74E-01	13.883	14.357	0.474	0.144	0.144
Germany	4.1.99.12	-	4.00E-01	132.393	130.996	-1.398	1.000	1.000
	4.1.99.12|g__Faecalibacterium.s__Faecalibacterium_prausnitzii	-	2.83E-02	11.000	5.631	-5.370	0.081	0.054
	4.1.99.12|g__Roseburia.s__Roseburia_faecis	-	2.44E-01	0.911	0.296	-0.615	0.015	0.010
	4.1.99.12|g__Bacteroides.s__Bacteroides_dorei	-	4.80E-01	4.109	3.562	-0.547	0.046	0.038
	4.1.99.12|g__Parabacteroides.s__Parabacteroides_distasonis	-	8.38E-01	1.668	1.172	-0.496	0.012	0.012
	4.1.99.12|g__Bacteroides.s__Bacteroides_vulgatus	-	7.10E-01	5.733	5.258	-0.475	0.094	0.085
	4.1.99.12|g__Roseburia.s__Roseburia_intestinalis	-	1.57E-01	0.365	0.000	-0.365	0.015	0.004
	4.1.99.12|g__Dorea.s__Dorea_longicatena	-	8.97E-01	0.975	0.637	-0.338	0.009	0.010
	4.1.99.12|g__Blautia.s__Blautia_obeum	-	2.84E-01	0.863	0.535	-0.327	0.008	0.008
	4.1.99.12|g__Coprococcus.s__Coprococcus_comes	-	5.85E-01	0.519	0.305	-0.213	0.004	0.005
	4.1.99.12|g__Dorea.s__Dorea_formicigenerans	-	2.46E-02	0.272	0.121	-0.151	0.003	0.001
	4.1.99.12|g__Eubacterium.s__Eubacterium_ramulus	-	4.92E-01	0.147	0.000	-0.147	0.001	0.002
	4.1.99.12|g__Coprococcus.s__Coprococcus_catus	-	3.09E-01	0.126	0.000	-0.126	0.001	0.001
	4.1.99.12|g__Bacteroides.s__Bacteroides_ovatus	-	5.14E-01	0.261	0.140	-0.122	0.004	0.006
	4.1.99.12|g__Sutterella.s__Sutterella_wadsworthensis	-	7.34E-01	0.117	0.000	-0.117	0.003	0.003
	4.1.99.12|g__Lachnospiraceae_unclassified.s__Eubacterium_rectale	+	7.21E-01	0.750	0.696	-0.053	0.013	0.015
	4.1.99.12|g__Eubacterium.s__Eubacterium_eligens	+	9.70E-01	0.842	0.813	-0.029	0.016	0.012
	4.1.99.12|g__Blautia.s__Blautia_wexlerae	-	3.16E-01	0.192	0.164	-0.028	0.002	0.002
	4.1.99.12|g__Agathobaculum.s__Agathobaculum_butyriciproducens	+	3.77E-01	0.220	0.217	-0.003	0.002	0.003
	4.1.99.12|g__Odoribacter.s__Odoribacter_splanchnicus	+	9.64E-01	0.523	0.522	-0.001	0.004	0.004
	4.1.99.12|g__Acidaminococcus.s__Acidaminococcus_fermentans	-	9.42E-01	0.000	0.000	0.000	0.000	0.000
	4.1.99.12|g__Acidaminococcus.s__Acidaminococcus_intestini	-	6.15E-01	0.000	0.000	0.000	0.001	0.002
	4.1.99.12|g__Actinomyces.s__Actinomyces_naeslundii	-	3.09E-01	0.000	0.000	0.000	0.000	0.000
	4.1.99.12|g__Actinomyces.s__Actinomyces_oris	-	3.09E-01	0.000	0.000	0.000	0.000	0.000
	4.1.99.12|g__Actinomyces.s__Actinomyces_viscosus	-	3.09E-01	0.000	0.000	0.000	0.000	0.000
	4.1.99.12|g__Adlercreutzia.s__Adlercreutzia_equolifaciens	+	9.80E-02	0.000	0.000	0.000	0.000	0.000
	4.1.99.12|g__Aggregatibacter.s__Aggregatibacter_aphrophilus	+	3.59E-01	0.000	0.000	0.000	0.000	0.000
	4.1.99.12|g__Alistipes.s__Alistipes_indistinctus	-	9.21E-01	0.000	0.000	0.000	0.001	0.001
	4.1.99.12|g__Alistipes.s__Alistipes_inops	+	2.30E-01	0.000	0.000	0.000	0.002	0.003
	4.1.99.12|g__Alistipes.s__Alistipes_sp_An66	+	1.83E-01	0.000	0.000	0.000	0.000	0.000
	4.1.99.12|g__Alistipes.s__Alistipes_timonensis	+	6.04E-01	0.000	0.000	0.000	0.000	0.001
	4.1.99.12|g__Anaeromassilibacillus.s__Anaeromassilibacillus_sp_An250	+	2.86E-01	0.000	0.000	0.000	0.000	0.000
	4.1.99.12|g__Anaerostipes.s__Anaerostipes_hadrus	+	4.90E-01	0.000	0.000	0.000	0.002	0.002
	4.1.99.12|g__Anaerotignum.s__Anaerotignum_lactatifermentans	-	9.81E-01	0.000	0.000	0.000	0.000	0.000
	4.1.99.12|g__Asaccharobacter.s__Asaccharobacter_celatus	+	9.86E-02	0.000	0.000	0.000	0.000	0.002

4.1.99.12|g__Bacteroides.s__Bacteroides_clarus	-	7.80E-01	0.000	0.000	0.000	0.003	0.001
4.1.99.12|g__Bacteroides.s__Bacteroides_coprocola	-	5.25E-01	0.000	0.000	0.000	0.011	0.004
4.1.99.12|g__Bacteroides.s__Bacteroides_coprophilus	-	8.75E-01	0.000	0.000	0.000	0.003	0.000
4.1.99.12|g__Bacteroides.s__Bacteroides_eggerthii	+	9.42E-01	0.000	0.000	0.000	0.011	0.003
4.1.99.12|g__Bacteroides.s__Bacteroides_faecichinchillae	+	9.80E-02	0.000	0.000	0.000	0.000	0.000
4.1.99.12|g__Bacteroides.s__Bacteroides_faecis	+	7.54E-01	0.000	0.000	0.000	0.002	0.003
4.1.99.12|g__Bacteroides.s__Bacteroides_finegoldii	+	3.97E-01	0.000	0.000	0.000	0.002	0.003
4.1.99.12|g__Bacteroides.s__Bacteroides_fluxus	-	1.40E-01	0.000	0.000	0.000	0.000	0.000
4.1.99.12|g__Bacteroides.s__Bacteroides_fragilis	+	8.98E-01	0.000	0.000	0.000	0.003	0.005
4.1.99.12|g__Bacteroides.s__Bacteroides_intestinalis	+	8.59E-01	0.000	0.000	0.000	0.001	0.002
4.1.99.12|g__Bacteroides.s__Bacteroides_massiliensis	-	8.85E-01	0.000	0.000	0.000	0.012	0.011
4.1.99.12|g__Bacteroides.s__Bacteroides_nordii	+	9.35E-01	0.000	0.000	0.000	0.000	0.000
4.1.99.12|g__Bacteroides.s__Bacteroides_plebeius	+	3.37E-01	0.000	0.000	0.000	0.002	0.005
4.1.99.12|g__Bacteroides.s__Bacteroides_salyersiae	+	8.06E-01	0.000	0.000	0.000	0.001	0.003
4.1.99.12|g__Bacteroides.s__Bacteroides_sp_OM08_11	+	3.59E-01	0.000	0.000	0.000	0.000	0.000
4.1.99.12|g__Bacteroides.s__Bacteroides_stercorirosoris	+	3.59E-01	0.000	0.000	0.000	0.000	0.000
4.1.99.12|g__Bacteroides.s__Bacteroides_stercoris	-	7.80E-01	0.000	0.000	0.000	0.015	0.009
4.1.99.12|g__Blautia.s__Blautia_hansenii	+	3.59E-01	0.000	0.000	0.000	0.000	0.000
4.1.99.12|g__Blautia.s__Blautia_sp_AF19_10LB	+	1.80E-01	0.000	0.000	0.000	0.001	0.001
4.1.99.12|g__Butyricicoccus.s__Butyricicoccus_pullicaecorum	+	3.59E-01	0.000	0.000	0.000	0.000	0.000
4.1.99.12|g__Butyricimonas.s__Butyricimonas_virosa	-	2.60E-01	0.000	0.000	0.000	0.001	0.001
4.1.99.12|g__Butyrivibrio.s__Butyrivibrio_crossotus	+	8.14E-01	0.000	0.000	0.000	0.010	0.003
4.1.99.12|g__Campylobacter.s__Campylobacter_concisus	-	3.09E-01	0.000	0.000	0.000	0.000	0.000
4.1.99.12|g__Candidatus_Gastranaerophilales_unclassified.s__Candidatus_(	+	2.20E-01	0.000	0.000	0.000	0.000	0.001
4.1.99.12|g__Candidatus_Methanomethylophilus.s__Candidatus_Methanom	+	3.59E-01	0.000	0.000	0.000	0.000	0.001
4.1.99.12|g__Catenibacterium.s__Catenibacterium_mitsuokai	-	5.87E-02	0.000	0.000	0.000	0.009	0.004
4.1.99.12|g__Cloacibacillus.s__Cloacibacillus_porcorum	+	6.04E-01	0.000	0.000	0.000	0.000	0.000
4.1.99.12|g__Clostridioides.s__Clostridioides_difficile	-	9.58E-01	0.000	0.000	0.000	0.000	0.000
4.1.99.12|g__Clostridium.s__Clostridium_celatum	-	3.09E-01	0.000	0.000	0.000	0.000	0.000
4.1.99.12|g__Clostridium.s__Clostridium_disporicum	-	1.40E-01	0.000	0.000	0.000	0.000	0.000
4.1.99.12|g__Clostridium.s__Clostridium_perfringens	-	3.09E-01	0.000	0.000	0.000	0.000	0.000
4.1.99.12|g__Coprobacillus.s__Coprobacillus_cateniformis	+	4.43E-01	0.000	0.000	0.000	0.000	0.001
4.1.99.12|g__Coprobacter.s__Coprobacter_fastidiosus	-	8.96E-01	0.000	0.000	0.000	0.002	0.001
4.1.99.12|g__Coprobacter.s__Coprobacter_secundus	+	8.18E-01	0.000	0.000	0.000	0.000	0.000
4.1.99.12|g__Coprococcus.s__Coprococcus_eutactus	-	8.36E-01	0.000	0.000	0.000	0.011	0.008
4.1.99.12|g__Desulfovibrio.s__Desulfovibrio_fairfieldensis	+	3.38E-01	0.000	0.000	0.000	0.000	0.002
4.1.99.12|g__Desulfovibrio.s__Desulfovibrio_piger	-	3.84E-01	0.000	0.000	0.000	0.002	0.013
4.1.99.12|g__Desulfovibrionaceae_unclassified.s__Desulfovibrionaceae_bac	-	3.09E-01	0.000	0.000	0.000	0.001	0.000
4.1.99.12|g__Dialister.s__Dialister_invisus	-	7.94E-01	0.000	0.000	0.000	0.007	0.008
4.1.99.12|g__Dialister.s__Dialister_succinatiphilus	-	9.42E-01	0.000	0.000	0.000	0.001	0.001
4.1.99.12|g__Dorea.s__Dorea_sp_OM02_2LB	-	9.31E-01	0.000	0.000	0.000	0.000	0.000
4.1.99.12|g__Eggerthella.s__Eggerthella_lenta	-	6.66E-02	0.000	0.000	0.000	0.000	0.000
4.1.99.12|g__Erysipelatoclostridium.s__Clostridium_innocuum	+	3.59E-01	0.000	0.000	0.000	0.000	0.000
4.1.99.12|g__Erysipelatoclostridium.s__Clostridium_spiroforme	-	8.75E-01	0.000	0.000	0.000	0.000	0.000
4.1.99.12|g__Erysipelotrichaceae_unclassified.s__Erysipelotrichaceae_bacte	-	3.09E-01	0.000	0.000	0.000	0.000	0.000
4.1.99.12|g__Escherichia.s__Escherichia_coli	-	5.76E-01	0.000	0.000	0.000	0.028	0.014
4.1.99.12|g__Escherichia.s__Escherichia_fergusonii	-	1.37E-01	0.000	0.000	0.000	0.000	0.000
4.1.99.12|g__Escherichia.s__Escherichia_marmotae	-	3.09E-01	0.000	0.000	0.000	0.000	0.000
4.1.99.12|g__Eubacterium.s__Eubacterium_sp_AF17_7	+	1.01E-01	0.000	0.000	0.000	0.000	0.001
4.1.99.12|g__Eubacterium.s__Eubacterium_sp_AM18_10LB_B	-	3.09E-01	0.000	0.000	0.000	0.000	0.000
4.1.99.12|g__Faecalitalea.s__Faecalitalea_cylindroides	-	8.75E-01	0.000	0.000	0.000	0.001	0.000
4.1.99.12|g__Firmicutes_unclassified.s__Firmicutes_bacterium_AM10_47	-	2.58E-01	0.000	0.000	0.000	0.000	0.000
4.1.99.12|g__Flavonifractor.s__Flavonifractor_plautii	+	1.86E-02	0.000	0.000	0.000	0.000	0.002
4.1.99.12|g__Flavonifractor.s__Flavonifractor_sp_An10	-	9.31E-01	0.000	0.000	0.000	0.000	0.000
4.1.99.12|g__Fusobacterium.s__Fusobacterium_mortiferum	-	3.09E-01	0.000	0.000	0.000	0.000	0.000
4.1.99.12|g__Haemophilus.s__Haemophilus_parainfluenzae	-	1.84E-02	0.000	0.000	0.000	0.002	0.000
4.1.99.12|g__Hafnia.s__Hafnia_alvei	+	3.59E-01	0.000	0.000	0.000	0.000	0.000
4.1.99.12|g__Hafnia.s__Hafnia_paralvei	+	3.59E-01	0.000	0.000	0.000	0.000	0.001
4.1.99.12|g__Holdemanella.s__Holdemanella_biformis	-	1.40E-01	0.000	0.000	0.000	0.000	0.000
4.1.99.12|g__Hungatella.s__Hungatella_hathewayi	-	9.10E-03	0.000	0.000	0.000	0.000	0.000
4.1.99.12|g__Intestinibacter.s__Intestinibacter_bartlettii	-	8.75E-01	0.000	0.000	0.000	0.000	0.000
4.1.99.12|g__Klebsiella.s__Klebsiella_oxytoca	+	7.34E-01	0.000	0.000	0.000	0.002	0.002
4.1.99.12|g__Klebsiella.s__Klebsiella_pneumoniae	-	1.20E-01	0.000	0.000	0.000	0.005	0.000
4.1.99.12|g__Klebsiella.s__Klebsiella_variicola	-	5.98E-01	0.000	0.000	0.000	0.001	0.000
4.1.99.12|g__Lachnoclostridium.s__Clostridium_aldenense	-	3.09E-01	0.000	0.000	0.000	0.000	0.000
4.1.99.12|g__Lachnoclostridium.s__Clostridium_bolteae	-	2.10E-02	0.000	0.000	0.000	0.000	0.000
4.1.99.12|g__Lachnoclostridium.s__Clostridium_clostridioforme	-	6.66E-02	0.000	0.000	0.000	0.000	0.000
4.1.99.12|g__Lachnoclostridium.s__Clostridium_symbiosum	-	4.85E-01	0.000	0.000	0.000	0.000	0.000
4.1.99.12|g__Lachnospira.s__Lachnospira_pectinoschiza	+	6.22E-01	0.000	0.000	0.000	0.001	0.002
4.1.99.12|g__Lachnospiraceae_unclassified.s__Lachnospiraceae_bacterium_	-	1.40E-01	0.000	0.000	0.000	0.000	0.000
4.1.99.12|g__Lactobacillus.s__Lactobacillus_antri	-	3.09E-01	0.000	0.000	0.000	0.000	0.000
4.1.99.12|g__Lactobacillus.s__Lactobacillus_gastricus	-	3.09E-01	0.000	0.000	0.000	0.000	0.000

4.1.99.12|g__Lactobacillus.s__Lactobacillus_mucosae	-	3.09E-01	0.000	0.000	0.000	0.000	0.000
4.1.99.12|g__Lactobacillus.s__Lactobacillus_oris	-	3.09E-01	0.000	0.000	0.000	0.000	0.000
4.1.99.12|g__Lactobacillus.s__Lactobacillus_rogosae	+	5.83E-01	0.000	0.000	0.000	0.001	0.003
4.1.99.12|g__Lactococcus.s__Lactococcus_lactis	-	8.75E-01	0.000	0.000	0.000	0.003	0.000
4.1.99.12|g__Megamonas.s__Megamonas_funiformis	+	3.59E-01	0.000	0.000	0.000	0.000	0.001
4.1.99.12|g__Megamonas.s__Megamonas_rupellensis	+	3.59E-01	0.000	0.000	0.000	0.000	0.000
4.1.99.12|g__Megasphaera.s__Megasphaera_elsdenii	-	4.85E-01	0.000	0.000	0.000	0.001	0.000
4.1.99.12|g__Megasphaera.s__Megasphaera_hexanoica	-	3.09E-01	0.000	0.000	0.000	0.000	0.000
4.1.99.12|g__Megasphaera.s__Megasphaera_sp_DISK_18	+	3.59E-01	0.000	0.000	0.000	0.000	0.001
4.1.99.12|g__Methanosphaera.s__Methanosphaera_stadtmanae	+	3.59E-01	0.000	0.000	0.000	0.000	0.000
4.1.99.12|g__Mitsuokella.s__Mitsuokella_jalaludinii	-	5.11E-01	0.000	0.000	0.000	0.001	0.000
4.1.99.12|g__Mitsuokella.s__Mitsuokella_multacida	-	3.09E-01	0.000	0.000	0.000	0.000	0.000
4.1.99.12|g__Obesumbacterium.s__Obesumbacterium_proteus	+	6.04E-01	0.000	0.000	0.000	0.000	0.001
4.1.99.12|g__Oxalobacter.s__Oxalobacter_formigenes	+	3.59E-01	0.000	0.000	0.000	0.000	0.000
4.1.99.12|g__Parabacteroides.s__Parabacteroides_goldsteinii	-	6.28E-01	0.000	0.000	0.000	0.001	0.000
4.1.99.12|g__Parabacteroides.s__Parabacteroides_gordonii	+	3.38E-01	0.000	0.000	0.000	0.000	0.000
4.1.99.12|g__Parabacteroides.s__Parabacteroides_johnsonii	-	3.04E-01	0.000	0.000	0.000	0.001	0.000
4.1.99.12|g__Paraprevotella.s__Paraprevotella_clara	-	4.48E-02	0.000	0.000	0.000	0.004	0.002
4.1.99.12|g__Parasutterella.s__Parasutterella_excrementihominis	+	6.27E-01	0.000	0.000	0.000	0.001	0.002
4.1.99.12|g__Phascolarctobacterium.s__Phascolarctobacterium_faecium	+	2.16E-01	0.000	0.000	0.000	0.003	0.007
4.1.99.12|g__Phascolarctobacterium.s__Phascolarctobacterium_succinatuter	-	2.21E-02	0.000	0.000	0.000	0.007	0.001
4.1.99.12|g__Prevotella.s__Prevotella_copri	-	6.66E-02	0.000	0.000	0.000	0.009	0.000
4.1.99.12|g__Prevotella.s__Prevotella_disiens	-	3.09E-01	0.000	0.000	0.000	0.000	0.000
4.1.99.12|g__Prevotella.s__Prevotella_sp_109	+	1.83E-01	0.000	0.000	0.000	0.000	0.008
4.1.99.12|g__Prevotella.s__Prevotella_sp_885	-	5.37E-01	0.000	0.000	0.000	0.001	0.001
4.1.99.12|g__Prevotella.s__Prevotella_sp_AM42_24	-	5.11E-01	0.000	0.000	0.000	0.009	0.007
4.1.99.12|g__Pseudoflavonifractor.s__Pseudoflavonifractor_sp_An184	+	8.71E-01	0.000	0.000	0.000	0.001	0.000
4.1.99.12|g__Pseudomonas.s__Pseudomonas_aeruginosa	-	3.09E-01	0.000	0.000	0.000	0.000	0.000
4.1.99.12|g__Pseudomonas.s__Pseudomonas_aeruginosa_group	-	3.09E-01	0.000	0.000	0.000	0.000	0.000
4.1.99.12|g__Psychrobacter.s__Psychrobacter_immobilis	-	3.09E-01	0.000	0.000	0.000	0.000	0.000
4.1.99.12|g__Pyramidobacter.s__Pyramidobacter_piscolens	-	3.09E-01	0.000	0.000	0.000	0.000	0.000
4.1.99.12|g__Raoultella.s__Raoultella_ornithinolytica	+	3.59E-01	0.000	0.000	0.000	0.000	0.000
4.1.99.12|g__Raoultella.s__Raoultella_planticola	+	3.59E-01	0.000	0.000	0.000	0.000	0.000
4.1.99.12|g__Roseburia.s__Roseburia_inulinivorans	+	1.63E-01	0.000	0.000	0.000	0.002	0.002
4.1.99.12|g__Ruminococcaceae_unclassified.s__Ruminococcaceae_bacteriu	-	5.50E-01	0.000	0.000	0.000	0.001	0.000
4.1.99.12|g__Ruminococcaceae_unclassified.s__Ruminococcaceae_bacteriu	+	1.83E-01	0.000	0.000	0.000	0.000	0.000
4.1.99.12|g__Ruminococcus.s__Ruminococcus_callidus	-	3.65E-01	0.000	0.000	0.000	0.002	0.001
4.1.99.12|g__Ruminococcus.s__Ruminococcus_sp_AF31_8BH	+	2.37E-01	0.000	0.000	0.000	0.001	0.001
4.1.99.12|g__Sanguibacteroides.s__Sanguibacteroides_justesenii	-	5.37E-01	0.000	0.000	0.000	0.000	0.000
4.1.99.12|g__Senegalimassilia.s__Senegalimassilia_anaerobia	+	7.52E-01	0.000	0.000	0.000	0.001	0.001
4.1.99.12|g__Slackia.s__Slackia_isoflavoniconvertens	-	3.23E-01	0.000	0.000	0.000	0.002	0.002
4.1.99.12|g__Streptococcus.s__Streptococcus_equinus	+	3.59E-01	0.000	0.000	0.000	0.000	0.000
4.1.99.12|g__Streptococcus.s__Streptococcus_infantarius	+	3.59E-01	0.000	0.000	0.000	0.000	0.000
4.1.99.12|g__Streptococcus.s__Streptococcus_lutetiensis	+	3.59E-01	0.000	0.000	0.000	0.000	0.000
4.1.99.12|g__Streptococcus.s__Streptococcus_macedonicus	-	3.09E-01	0.000	0.000	0.000	0.000	0.000
4.1.99.12|g__Streptococcus.s__Streptococcus_pasteurianus	-	3.09E-01	0.000	0.000	0.000	0.000	0.000
4.1.99.12|g__Turicimonas.s__Turicimonas_muris	-	3.09E-01	0.000	0.000	0.000	0.000	0.000
4.1.99.12|g__Veillonella.s__Veillonella_atypica	-	1.40E-01	0.000	0.000	0.000	0.000	0.000
4.1.99.12|g__Veillonella.s__Veillonella_dispar	-	5.50E-01	0.000	0.000	0.000	0.000	0.000
4.1.99.12|g__Veillonella.s__Veillonella_infantium	+	1.83E-01	0.000	0.000	0.000	0.000	0.000
4.1.99.12|g__Veillonella.s__Veillonella_parvula	+	1.83E-01	0.000	0.000	0.000	0.000	0.000
4.1.99.12|g__Veillonella.s__Veillonella_rogosae	-	9.81E-01	0.000	0.000	0.000	0.000	0.000
4.1.99.12|g__Victivallales_unclassified.s__Victivallales_bacterium_CCUG_	+	8.74E-01	0.000	0.000	0.000	0.001	0.002
4.1.99.12|g__Victivallis.s__Victivallis_vadensis	+	2.51E-01	0.000	0.000	0.000	0.000	0.001
4.1.99.12|g__Bacteroides.s__Bacteroides_xylanisolvens	+	8.40E-01	0.204	0.207	0.003	0.005	0.009
4.1.99.12|g__Bacteroides.s__Bacteroides_thetaiotaomicron	-	4.16E-01	0.360	0.365	0.005	0.008	0.004
4.1.99.12|g__Bacteroides.s__Bacteroides_caccae	-	5.96E-01	0.695	0.790	0.095	0.011	0.008
4.1.99.12|g__Clostridium.s__Clostridium_sp_AM22_11AC	+	5.20E-01	0.000	0.127	0.127	0.002	0.002
4.1.99.12|g__Alistipes.s__Alistipes_finegoldii	+	1.99E-01	0.318	0.470	0.152	0.009	0.012
4.1.99.12|g__Roseburia.s__Roseburia_hominis	+	1.20E-01	0.450	0.646	0.196	0.005	0.010
4.1.99.12|g__Methanobrevibacter.s__Methanobrevibacter_smithii	+	2.74E-01	0.000	0.197	0.197	0.007	0.009
4.1.99.12|g__Barnesiella.s__Barnesiella_intestinihominis	+	5.24E-01	0.584	0.789	0.205	0.010	0.011
4.1.99.12|g__Blautia.s__Ruminococcus_torques	+	5.74E-01	1.689	2.003	0.314	0.022	0.031
4.1.99.12|g__Bacteroides.s__Bacteroides_cellulosilyticus	+	6.52E-01	0.369	0.700	0.331	0.011	0.011
4.1.99.12|g__Clostridium.s__Clostridium_sp_AF36_4	+	1.50E-02	0.000	0.398	0.398	0.001	0.005
4.1.99.12|g__Parabacteroides.s__Parabacteroides_merdae	+	6.39E-01	0.387	0.790	0.403	0.007	0.009
4.1.99.12|g__Alistipes.s__Alistipes_onderdonkii	+	1.00E-02	0.175	0.879	0.705	0.010	0.019
4.1.99.12|g__Bilophila.s__Bilophila_wadsworthia	+	1.13E-01	0.000	0.709	0.709	0.004	0.006
4.1.99.12|g__Bacteroides.s__Bacteroides_uniformis	+	6.06E-01	3.104	4.431	1.327	0.046	0.073
4.1.99.12|g__Alistipes.s__Alistipes_putredinis	+	2.71E-01	3.074	4.662	1.588	0.038	0.048
4.1.99.12|unclassified	-	8.38E-01	23.014	25.975	2.961	0.239	0.219
4.1.99.12|g__Akkermansia.s__Akkermansia_muciniphila	+	1.45E-03	0.000	3.139	3.139	0.010	0.064

China1	4.1.99.12	-	5.37E-02	158.118	146.131	-11.987	1.000	1.000
	4.1.99.12|g__Bacteroides.s__Bacteroides_vulgatus	-	1.06E-02	19.754	5.808	-13.946	0.200	0.101
	4.1.99.12|g__Bacteroides.s__Bacteroides_fragilis	-	1.59E-01	1.624	0.976	-0.649	0.024	0.023
	4.1.99.12|g__Bacteroides.s__Bacteroides_ovatus	-	9.13E-02	1.894	1.411	-0.483	0.038	0.018
	4.1.99.12|g__Bacteroides.s__Bacteroides_xylanisolvens	-	2.55E-01	1.431	0.966	-0.465	0.016	0.010
	4.1.99.12|g__Escherichia.s__Escherichia_coli	-	5.97E-01	0.546	0.243	-0.303	0.030	0.032
	4.1.99.12|g__Klebsiella.s__Klebsiella_oxytoca	-	2.23E-01	0.430	0.246	-0.184	0.005	0.005
	4.1.99.12|g__Faecalibacterium.s__Faecalibacterium_prausnitzii	+	7.69E-01	1.023	0.878	-0.145	0.011	0.013
	4.1.99.12|unclassified	+	5.22E-01	14.572	14.429	-0.143	0.145	0.166
	4.1.99.12|g__Roseburia.s__Roseburia_faecis	-	6.27E-01	0.183	0.117	-0.067	0.003	0.003
	4.1.99.12|g__Bacteroides.s__Bacteroides_cellulosilyticus	+	8.21E-01	0.768	0.731	-0.037	0.014	0.022
	4.1.99.12|g__Bacteroides.s__Bacteroides_nordii	-	7.93E-01	0.101	0.084	-0.017	0.002	0.004
	4.1.99.12|g__Dorea.s__Dorea_longicatena	-	9.49E-01	0.076	0.061	-0.015	0.001	0.001
	4.1.99.12|g__Acidaminococcus.s__Acidaminococcus_intestini	+	3.30E-01	0.000	0.000	0.000	0.000	0.000
	4.1.99.12|g__Adlercreutzia.s__Adlercreutzia_equolifaciens	+	3.30E-01	0.000	0.000	0.000	0.000	0.000
	4.1.99.12|g__Aeromonas.s__Aeromonas_allosaccharophila	-	3.30E-01	0.000	0.000	0.000	0.000	0.000
	4.1.99.12|g__Aeromonas.s__Aeromonas_hydrophila	-	3.30E-01	0.000	0.000	0.000	0.000	0.000
	4.1.99.12|g__Aeromonas.s__Aeromonas_veronii	-	3.30E-01	0.000	0.000	0.000	0.000	0.000
	4.1.99.12|g__Agathobaculum.s__Agathobaculum_butyriciproducens	-	7.03E-01	0.000	0.000	0.000	0.000	0.000
	4.1.99.12|g__Aggregatibacter.s__Aggregatibacter_segnis	-	3.30E-01	0.000	0.000	0.000	0.000	0.000
	4.1.99.12|g__Akkermansia.s__Akkermansia_muciniphila	+	9.33E-03	0.000	0.000	0.000	0.001	0.004
	4.1.99.12|g__Alistipes.s__Alistipes_inops	-	9.66E-01	0.000	0.000	0.000	0.001	0.001
	4.1.99.12|g__Alistipes.s__Alistipes_sp_An66	+	5.49E-01	0.000	0.000	0.000	0.000	0.000
	4.1.99.12|g__Alistipes.s__Alistipes_timonensis	+	6.55E-01	0.000	0.000	0.000	0.001	0.001
	4.1.99.12|g__Anaeroglobus.s__Anaeroglobus_geminatus	+	1.00E+00	0.000	0.000	0.000	0.000	0.000
	4.1.99.12|g__Anaeromassilibacillus.s__Anaeromassilibacillus_sp_An250	+	3.30E-01	0.000	0.000	0.000	0.000	0.000
	4.1.99.12|g__Anaerostipes.s__Anaerostipes_caccae	-	1.60E-01	0.000	0.000	0.000	0.000	0.000
	4.1.99.12|g__Anaerostipes.s__Anaerostipes_hadrus	-	1.60E-01	0.000	0.000	0.000	0.000	0.000
	4.1.99.12|g__Anaerotignum.s__Anaerotignum_lactatifermentans	-	3.30E-01	0.000	0.000	0.000	0.000	0.000
	4.1.99.12|g__Asaccharobacter.s__Asaccharobacter_celatus	+	3.30E-01	0.000	0.000	0.000	0.000	0.000
	4.1.99.12|g__Bacteroides.s__Bacteroides_clarus	+	2.16E-01	0.000	0.000	0.000	0.001	0.003
	4.1.99.12|g__Bacteroides.s__Bacteroides_coprocola	-	6.84E-02	0.000	0.000	0.000	0.030	0.005
	4.1.99.12|g__Bacteroides.s__Bacteroides_coprophilus	-	4.05E-01	0.000	0.000	0.000	0.004	0.001
	4.1.99.12|g__Bacteroides.s__Bacteroides_eggerthii	+	1.09E-01	0.000	0.000	0.000	0.013	0.021
	4.1.99.12|g__Bacteroides.s__Bacteroides_faecichinchillae	+	4.28E-02	0.000	0.000	0.000	0.000	0.000
	4.1.99.12|g__Bacteroides.s__Bacteroides_faecis	-	1.58E-01	0.000	0.000	0.000	0.001	0.000
	4.1.99.12|g__Bacteroides.s__Bacteroides_finegoldii	+	3.16E-01	0.000	0.000	0.000	0.005	0.009
	4.1.99.12|g__Bacteroides.s__Bacteroides_fluxus	+	3.30E-01	0.000	0.000	0.000	0.000	0.000
	4.1.99.12|g__Bacteroides.s__Bacteroides_massiliensis	+	7.11E-01	0.000	0.000	0.000	0.006	0.009
	4.1.99.12|g__Bacteroides.s__Bacteroides_oleiciplenus	+	9.90E-01	0.000	0.000	0.000	0.000	0.000
	4.1.99.12|g__Bacteroides.s__Bacteroides_plebeius	-	3.05E-01	0.000	0.000	0.000	0.042	0.014
	4.1.99.12|g__Bacteroides.s__Bacteroides_salyersiae	-	7.77E-01	0.000	0.000	0.000	0.001	0.001
	4.1.99.12|g__Bacteroides.s__Bacteroides_sp_OM08_11	+	1.60E-01	0.000	0.000	0.000	0.000	0.000
	4.1.99.12|g__Bacteroides.s__Bacteroides_stercorirosoris	+	9.70E-01	0.000	0.000	0.000	0.000	0.000
	4.1.99.12|g__Barnesiella.s__Barnesiella_intestinihominis	+	2.79E-01	0.000	0.000	0.000	0.005	0.004
	4.1.99.12|g__Barnesiella.s__Barnesiella_sp_An22	-	3.30E-01	0.000	0.000	0.000	0.000	0.000
	4.1.99.12|g__Blautia.s__Blautia_hansenii	+	3.30E-01	0.000	0.000	0.000	0.000	0.000
	4.1.99.12|g__Blautia.s__Blautia_sp_AF19_10LB	+	9.63E-01	0.000	0.000	0.000	0.000	0.000
	4.1.99.12|g__Butyricimonas.s__Butyricimonas_synergistica	-	1.00E+00	0.000	0.000	0.000	0.000	0.000
	4.1.99.12|g__Butyricimonas.s__Butyricimonas_virosa	+	9.69E-01	0.000	0.000	0.000	0.001	0.002
	4.1.99.12|g__Butyrivibrio.s__Butyrivibrio_crossotus	+	5.69E-01	0.000	0.000	0.000	0.000	0.000
	4.1.99.12|g__Campylobacter.s__Campylobacter_concisus	-	3.30E-01	0.000	0.000	0.000	0.000	0.000
	4.1.99.12|g__Campylobacter.s__Campylobacter_gracilis	+	3.30E-01	0.000	0.000	0.000	0.000	0.000
	4.1.99.12|g__Catenibacterium.s__Catenibacterium_mitsuokai	+	6.22E-01	0.000	0.000	0.000	0.001	0.003
	4.1.99.12|g__Citrobacter.s__Citrobacter_braakii	+	4.28E-02	0.000	0.000	0.000	0.000	0.000
	4.1.99.12|g__Citrobacter.s__Citrobacter_farmeri	+	3.30E-01	0.000	0.000	0.000	0.000	0.000
	4.1.99.12|g__Citrobacter.s__Citrobacter_freundii	+	4.28E-02	0.000	0.000	0.000	0.000	0.000
	4.1.99.12|g__Citrobacter.s__Citrobacter_portucalensis	+	3.71E-01	0.000	0.000	0.000	0.000	0.000
	4.1.99.12|g__Citrobacter.s__Citrobacter_youngae	+	1.60E-01	0.000	0.000	0.000	0.000	0.000
	4.1.99.12|g__Cloacibacillus.s__Cloacibacillus_porcorum	+	1.60E-01	0.000	0.000	0.000	0.000	0.000
	4.1.99.12|g__Clostridioides.s__Clostridioides_difficile	+	8.53E-01	0.000	0.000	0.000	0.001	0.000
	4.1.99.12|g__Clostridium.s__Clostridium_neonatale	-	3.30E-01	0.000	0.000	0.000	0.000	0.000
	4.1.99.12|g__Clostridium.s__Clostridium_perfringens	-	5.69E-01	0.000	0.000	0.000	0.000	0.000
	4.1.99.12|g__Clostridium.s__Clostridium_sp_AF36_4	+	6.43E-02	0.000	0.000	0.000	0.001	0.003
	4.1.99.12|g__Clostridium.s__Clostridium_sp_AM22_11AC	-	5.67E-02	0.000	0.000	0.000	0.001	0.000
	4.1.99.12|g__Clostridium.s__Clostridium_sp_chh4_2	+	3.30E-01	0.000	0.000	0.000	0.000	0.000
	4.1.99.12|g__Coprobacillus.s__Coprobacillus_cateniformis	+	2.81E-01	0.000	0.000	0.000	0.000	0.000
	4.1.99.12|g__Coprobacter.s__Coprobacter_fastidiosus	+	7.10E-01	0.000	0.000	0.000	0.001	0.000
	4.1.99.12|g__Coprobacter.s__Coprobacter_secundus	-	7.66E-01	0.000	0.000	0.000	0.000	0.001
	4.1.99.12|g__Coprococcus.s__Coprococcus_catus	+	2.07E-01	0.000	0.000	0.000	0.000	0.000
	4.1.99.12|g__Coprococcus.s__Coprococcus_comes	+	9.36E-02	0.000	0.000	0.000	0.000	0.001
	4.1.99.12|g__Coprococcus.s__Coprococcus_eutactus	+	3.55E-01	0.000	0.000	0.000	0.000	0.002

4.1.99.12|g__Desulfovibrio.s__Desulfovibrio_piger	+	2.27E-01	0.000	0.000	0.000	0.000	0.001
4.1.99.12|g__Desulfovibrio.s__Desulfovibrio_sp_AM18_2	+	8.20E-02	0.000	0.000	0.000	0.000	0.001
4.1.99.12|g__Desulfovibrionaceae_unclassified.s__Desulfovibrionaceae_bac	+	1.60E-01	0.000	0.000	0.000	0.000	0.000
4.1.99.12|g__Dialister.s__Dialister_invisus	+	8.52E-01	0.000	0.000	0.000	0.002	0.007
4.1.99.12|g__Dialister.s__Dialister_succinatiphilus	+	1.00E+00	0.000	0.000	0.000	0.000	0.000
4.1.99.12|g__Dorea.s__Dorea_formicigenerans	-	2.75E-01	0.000	0.000	0.000	0.001	0.000
4.1.99.12|g__Eggerthella.s__Eggerthella_lenta	+	6.87E-01	0.000	0.000	0.000	0.000	0.000
4.1.99.12|g__Enterobacter.s__Enterobacter_cloacae	+	2.25E-02	0.000	0.000	0.000	0.000	0.000
4.1.99.12|g__Enterococcus.s__Enterococcus_faecium	+	3.30E-01	0.000	0.000	0.000	0.000	0.000
4.1.99.12|g__Erysipelatoclostridium.s__Clostridium_innocuum	+	3.30E-01	0.000	0.000	0.000	0.000	0.000
4.1.99.12|g__Erysipelatoclostridium.s__Clostridium_spiroforme	+	5.49E-01	0.000	0.000	0.000	0.000	0.000
4.1.99.12|g__Erysipelotrichaceae_unclassified.s__Erysipelotrichaceae_bacte	-	3.30E-01	0.000	0.000	0.000	0.000	0.000
4.1.99.12|g__Escherichia.s__Escherichia_fergusonii	-	5.58E-01	0.000	0.000	0.000	0.000	0.000
4.1.99.12|g__Eubacterium.s__Eubacterium_ramulus	+	4.05E-01	0.000	0.000	0.000	0.000	0.000
4.1.99.12|g__Eubacterium.s__Eubacterium_sp_AF17_7	+	6.44E-02	0.000	0.000	0.000	0.000	0.001
4.1.99.12|g__Eubacterium.s__Eubacterium_sp_AM18_10LB_B	+	1.00E+00	0.000	0.000	0.000	0.000	0.001
4.1.99.12|g__Faecalitalea.s__Faecalitalea_cylindroides	+	3.30E-01	0.000	0.000	0.000	0.000	0.000
4.1.99.12|g__Firmicutes_unclassified.s__Firmicutes_bacterium_AM10_47	-	5.89E-01	0.000	0.000	0.000	0.000	0.000
4.1.99.12|g__Flavonifractor.s__Flavonifractor_plautii	+	4.28E-02	0.000	0.000	0.000	0.000	0.000
4.1.99.12|g__Fusobacterium.s__Fusobacterium_hwasookii	-	3.30E-01	0.000	0.000	0.000	0.000	0.000
4.1.99.12|g__Fusobacterium.s__Fusobacterium_mortiferum	+	6.55E-01	0.000	0.000	0.000	0.001	0.000
4.1.99.12|g__Fusobacterium.s__Fusobacterium_ulcerans	-	6.71E-01	0.000	0.000	0.000	0.001	0.001
4.1.99.12|g__Fusobacterium.s__Fusobacterium_varium	-	1.60E-01	0.000	0.000	0.000	0.001	0.000
4.1.99.12|g__Haemophilus.s__Haemophilus_parainfluenzae	-	7.71E-01	0.000	0.000	0.000	0.002	0.002
4.1.99.12|g__Haemophilus.s__Haemophilus_paraphrohaemolyticus	+	3.30E-01	0.000	0.000	0.000	0.000	0.000
4.1.99.12|g__Haemophilus.s__Haemophilus_sputorum	+	1.00E+00	0.000	0.000	0.000	0.000	0.000
4.1.99.12|g__Holdemanella.s__Holdemanella_biformis	+	1.60E-01	0.000	0.000	0.000	0.000	0.000
4.1.99.12|g__Hungatella.s__Hungatella_hathewayi	+	3.11E-01	0.000	0.000	0.000	0.001	0.000
4.1.99.12|g__Intestinibacter.s__Intestinibacter_bartlettii	-	9.83E-01	0.000	0.000	0.000	0.000	0.000
4.1.99.12|g__Klebsiella.s__Klebsiella_pneumoniae	+	1.54E-01	0.000	0.000	0.000	0.001	0.003
4.1.99.12|g__Klebsiella.s__Klebsiella_variicola	+	7.79E-02	0.000	0.000	0.000	0.000	0.000
4.1.99.12|g__Lachnoclostridium.s__Clostridium_aldenense	-	4.74E-01	0.000	0.000	0.000	0.000	0.000
4.1.99.12|g__Lachnoclostridium.s__Clostridium_bolteae	+	5.41E-01	0.000	0.000	0.000	0.000	0.000
4.1.99.12|g__Lachnoclostridium.s__Clostridium_citroniae	+	3.33E-01	0.000	0.000	0.000	0.000	0.000
4.1.99.12|g__Lachnoclostridium.s__Clostridium_clostridioforme	+	8.63E-01	0.000	0.000	0.000	0.000	0.000
4.1.99.12|g__Lachnoclostridium.s__Clostridium_symbiosum	-	7.54E-01	0.000	0.000	0.000	0.000	0.000
4.1.99.12|g__Lachnospira.s__Lachnospira_pectinoschiza	-	3.88E-01	0.000	0.000	0.000	0.001	0.001
4.1.99.12|g__Lactobacillus.s__Lactobacillus_delbrueckii	+	3.30E-01	0.000	0.000	0.000	0.000	0.000
4.1.99.12|g__Lactobacillus.s__Lactobacillus_fermentum	+	3.30E-01	0.000	0.000	0.000	0.000	0.000
4.1.99.12|g__Lactobacillus.s__Lactobacillus_mucosae	+	3.33E-01	0.000	0.000	0.000	0.000	0.000
4.1.99.12|g__Lactobacillus.s__Lactobacillus_rogosae	-	4.07E-01	0.000	0.000	0.000	0.002	0.001
4.1.99.12|g__Megamonas.s__Megamonas_funiformis	+	7.36E-01	0.000	0.000	0.000	0.003	0.002
4.1.99.12|g__Megamonas.s__Megamonas_rupellensis	-	9.47E-01	0.000	0.000	0.000	0.001	0.000
4.1.99.12|g__Megasphaera.s__Megasphaera_micronuciformis	-	3.30E-01	0.000	0.000	0.000	0.000	0.000
4.1.99.12|g__Methanobrevibacter.s__Methanobrevibacter_smithii	+	1.58E-01	0.000	0.000	0.000	0.000	0.005
4.1.99.12|g__Obesumbacterium.s__Obesumbacterium_proteus	-	3.30E-01	0.000	0.000	0.000	0.000	0.000
4.1.99.12|g__Oxalobacter.s__Oxalobacter_formigenes	+	9.90E-01	0.000	0.000	0.000	0.000	0.001
4.1.99.12|g__Parabacteroides.s__Parabacteroides_goldsteinii	+	1.47E-01	0.000	0.000	0.000	0.001	0.006
4.1.99.12|g__Parabacteroides.s__Parabacteroides_gordonii	+	5.67E-01	0.000	0.000	0.000	0.001	0.002
4.1.99.12|g__Parabacteroides.s__Parabacteroides_johnsonii	+	6.61E-02	0.000	0.000	0.000	0.001	0.002
4.1.99.12|g__Paraprevotella.s__Paraprevotella_clara	+	5.45E-03	0.000	0.000	0.000	0.001	0.004
4.1.99.12|g__Paraprevotella.s__Paraprevotella_xylaniphila	+	9.90E-01	0.000	0.000	0.000	0.000	0.001
4.1.99.12|g__Parasutterella.s__Parasutterella_excrementihominis	-	7.47E-01	0.000	0.000	0.000	0.002	0.001
4.1.99.12|g__Peptostreptococcaceae_unclassified.s__Clostridium_hiranonis	-	3.30E-01	0.000	0.000	0.000	0.000	0.000
4.1.99.12|g__Phascolarctobacterium.s__Phascolarctobacterium_succinatuter	+	2.97E-01	0.000	0.000	0.000	0.001	0.002
4.1.99.12|g__Prevotella.s__Prevotella_bivia	+	3.30E-01	0.000	0.000	0.000	0.000	0.000
4.1.99.12|g__Prevotella.s__Prevotella_copri	+	6.80E-01	0.000	0.000	0.000	0.011	0.012
4.1.99.12|g__Prevotella.s__Prevotella_multisaccharivorax	-	3.30E-01	0.000	0.000	0.000	0.000	0.000
4.1.99.12|g__Prevotella.s__Prevotella_sp_109	-	8.20E-02	0.000	0.000	0.000	0.001	0.000
4.1.99.12|g__Prevotella.s__Prevotella_sp_AM42_24	-	1.60E-01	0.000	0.000	0.000	0.002	0.000
4.1.99.12|g__Prevotella.s__Prevotella_stercorea	-	7.31E-01	0.000	0.000	0.000	0.007	0.005
4.1.99.12|g__Proteus.s__Proteus_mirabilis	+	3.30E-01	0.000	0.000	0.000	0.000	0.000
4.1.99.12|g__Pseudocitrobacter.s__Pseudocitrobacter_faecalis	+	3.30E-01	0.000	0.000	0.000	0.000	0.000
4.1.99.12|g__Pyramidobacter.s__Pyramidobacter_piscolens	+	5.28E-02	0.000	0.000	0.000	0.000	0.000
4.1.99.12|g__Pyramidobacter.s__Pyramidobacter_sp_C12_8	+	1.60E-01	0.000	0.000	0.000	0.000	0.000
4.1.99.12|g__Raoultella.s__Raoultella_ornithinolytica	-	3.30E-01	0.000	0.000	0.000	0.000	0.000
4.1.99.12|g__Roseburia.s__Roseburia_intestinalis	-	4.99E-01	0.000	0.000	0.000	0.001	0.001
4.1.99.12|g__Roseburia.s__Roseburia_inulinivorans	+	2.28E-01	0.000	0.000	0.000	0.000	0.001
4.1.99.12|g__Ruminococcaceae_unclassified.s__Ruminococcaceae_bacteriu	-	1.60E-01	0.000	0.000	0.000	0.000	0.000
4.1.99.12|g__Ruminococcaceae_unclassified.s__Ruminococcaceae_bacteriu	+	5.56E-02	0.000	0.000	0.000	0.000	0.000
4.1.99.12|g__Ruminococcus.s__Ruminococcus_callidus	+	9.07E-01	0.000	0.000	0.000	0.000	0.001
4.1.99.12|g__Ruminococcus.s__Ruminococcus_sp_AF31_8BH	-	7.99E-01	0.000	0.000	0.000	0.000	0.000

	4.1.99.12|g__Salmonella.s__Salmonella_enterica	-	1.60E-01	0.000	0.000	0.000	0.000	0.000
	4.1.99.12|g__Sanguibacteroides.s__Sanguibacteroides_justesenii	+	2.96E-01	0.000	0.000	0.000	0.000	0.000
	4.1.99.12|g__Senegalimassilia.s__Senegalimassilia_anaerobia	+	6.38E-01	0.000	0.000	0.000	0.000	0.000
	4.1.99.12|g__Slackia.s__Slackia_isoflavoniconvertens	+	5.69E-01	0.000	0.000	0.000	0.000	0.000
	4.1.99.12|g__Streptococcus.s__Streptococcus_mitis	+	3.30E-01	0.000	0.000	0.000	0.000	0.000
	4.1.99.12|g__Streptococcus.s__Streptococcus_oralis	+	3.30E-01	0.000	0.000	0.000	0.000	0.000
	4.1.99.12|g__Streptococcus.s__Streptococcus_pneumoniae	+	3.30E-01	0.000	0.000	0.000	0.000	0.000
	4.1.99.12|g__Streptococcus.s__Streptococcus_pseudopneumoniae	+	3.30E-01	0.000	0.000	0.000	0.000	0.000
	4.1.99.12|g__Sutterella.s__Sutterella_wadsworthensis	+	3.15E-01	0.000	0.000	0.000	0.001	0.002
	4.1.99.12|g__Synergistes.s__Synergistes_jonesii	+	3.30E-01	0.000	0.000	0.000	0.000	0.000
	4.1.99.12|g__Turicimonas.s__Turicimonas_muris	+	1.00E+00	0.000	0.000	0.000	0.000	0.000
	4.1.99.12|g__Veillonella.s__Veillonella_atypica	+	4.41E-01	0.000	0.000	0.000	0.000	0.001
	4.1.99.12|g__Veillonella.s__Veillonella_dispar	+	6.77E-01	0.000	0.000	0.000	0.002	0.000
	4.1.99.12|g__Veillonella.s__Veillonella_infantium	+	6.48E-01	0.000	0.000	0.000	0.000	0.000
	4.1.99.12|g__Veillonella.s__Veillonella_parvula	+	1.38E-01	0.000	0.000	0.000	0.001	0.001
	4.1.99.12|g__Veillonella.s__Veillonella_rogosae	+	1.05E-01	0.000	0.000	0.000	0.000	0.000
	4.1.99.12|g__Veillonella.s__Veillonella_tobetsuensis	+	4.05E-01	0.000	0.000	0.000	0.000	0.000
	4.1.99.12|g__Victivallales_unclassified.s__Victivallales_bacterium_CCUG_	+	2.96E-01	0.000	0.000	0.000	0.000	0.002
	4.1.99.12|g__Victivallis.s__Victivallis_vadensis	+	1.00E+00	0.000	0.000	0.000	0.000	0.000
	4.1.99.12|g__Blautia.s__Blautia_wexlerae	-	9.15E-01	0.008	0.012	0.004	0.000	0.000
	4.1.99.12|g__Roseburia.s__Roseburia_hominis	+	1.44E-01	0.190	0.199	0.009	0.002	0.004
	4.1.99.12|g__Bacteroides.s__Bacteroides_dorei	-	4.50E-01	2.082	2.102	0.021	0.080	0.053
	4.1.99.12|g__Blautia.s__Blautia_obeum	+	4.35E-01	0.056	0.082	0.026	0.001	0.001
	4.1.99.12|g__Lachnospiraceae_unclassified.s__Eubacterium_rectale	-	9.08E-01	0.106	0.134	0.028	0.003	0.003
	4.1.99.12|g__Alistipes.s__Alistipes_indistinctus	+	5.11E-03	0.000	0.051	0.051	0.002	0.002
	4.1.99.12|g__Parabacteroides.s__Parabacteroides_distasonis	+	5.60E-01	1.069	1.135	0.066	0.019	0.014
	4.1.99.12|g__Alistipes.s__Alistipes_onderdonkii	+	2.29E-01	0.029	0.189	0.160	0.004	0.008
	4.1.99.12|g__Blautia.s__Ruminococcus_torques	+	5.25E-01	0.284	0.468	0.183	0.004	0.006
	4.1.99.12|g__Phascolarctobacterium.s__Phascolarctobacterium_faecium	+	4.11E-01	0.496	0.684	0.188	0.010	0.012
	4.1.99.12|g__Bilophila.s__Bilophila_wadsworthia	+	7.61E-02	0.114	0.317	0.203	0.002	0.003
	4.1.99.12|g__Odoribacter.s__Odoribacter_splanchnicus	-	8.67E-01	0.031	0.289	0.259	0.004	0.003
	4.1.99.12|g__Alistipes.s__Alistipes_finegoldii	+	2.03E-02	0.080	0.393	0.313	0.002	0.017
	4.1.99.12|g__Bacteroides.s__Bacteroides_thetaiotaomicron	+	8.06E-01	1.244	1.608	0.365	0.018	0.025
	4.1.99.12|g__Parabacteroides.s__Parabacteroides_merdae	+	2.54E-01	0.103	0.475	0.371	0.007	0.010
	4.1.99.12|g__Eubacterium.s__Eubacterium_eligens	+	9.16E-02	0.271	0.652	0.380	0.006	0.015
	4.1.99.12|g__Bacteroides.s__Bacteroides_caccae	+	5.04E-01	0.223	0.687	0.464	0.010	0.015
	4.1.99.12|g__Bacteroides.s__Bacteroides_stercoris	+	7.62E-01	0.330	0.835	0.504	0.042	0.031
	4.1.99.12|g__Bacteroides.s__Bacteroides_intestinalis	+	2.56E-02	0.045	0.782	0.737	0.004	0.014
	4.1.99.12|g__Bacteroides.s__Bacteroides_uniformis	+	1.83E-01	7.698	11.289	3.591	0.072	0.118
	4.1.99.12|g__Alistipes.s__Alistipes_putredinis	+	7.68E-02	1.426	7.079	5.653	0.047	0.084
China2	4.1.99.12	-	4.25E-02	173.020	159.422	-13.598	1.000	1.000
	4.1.99.12|g__Bacteroides.s__Bacteroides_vulgatus	-	4.20E-02	19.211	10.341	-8.870	0.191	0.116
	4.1.99.12|g__Bacteroides.s__Bacteroides_dorei	-	1.05E-01	7.110	2.234	-4.875	0.080	0.049
	4.1.99.12|g__Faecalibacterium.s__Faecalibacterium_prausnitzii	-	3.22E-01	7.177	4.545	-2.633	0.051	0.045
	4.1.99.12|g__Roseburia.s__Roseburia_faecis	-	1.18E-01	0.920	0.277	-0.643	0.016	0.008
	4.1.99.12|g__Phascolarctobacterium.s__Phascolarctobacterium_faecium	-	5.51E-01	0.732	0.210	-0.522	0.007	0.007
	4.1.99.12|g__Bacteroides.s__Bacteroides_thetaiotaomicron	-	1.72E-01	0.517	0.267	-0.250	0.008	0.005
	4.1.99.12|g__Clostridium.s__Clostridium_sp_AM22_11AC	-	2.89E-01	0.530	0.330	-0.201	0.006	0.005
	4.1.99.12|g__Parabacteroides.s__Parabacteroides_distasonis	+	5.35E-01	1.172	0.983	-0.188	0.010	0.016
	4.1.99.12|g__Bacteroides.s__Bacteroides_xylanisolvens	-	8.82E-02	0.305	0.176	-0.129	0.008	0.004
	4.1.99.12|g__Roseburia.s__Roseburia_intestinalis	-	7.16E-01	0.077	0.000	-0.077	0.004	0.002
	4.1.99.12|g__Agathobaculum.s__Agathobaculum_butyriciproducens	-	1.32E-01	0.098	0.038	-0.060	0.001	0.001
	4.1.99.12|g__Klebsiella.s__Klebsiella_oxytoca	-	4.45E-01	0.217	0.165	-0.052	0.004	0.003
	4.1.99.12|g__Roseburia.s__Roseburia_hominis	+	7.07E-01	0.303	0.258	-0.046	0.003	0.004
	4.1.99.12|g__Bacteroides.s__Bacteroides_cellulosilyticus	-	7.02E-01	0.077	0.038	-0.039	0.006	0.010
	4.1.99.12|g__Veillonella.s__Veillonella_parvula	-	7.08E-01	0.012	0.000	-0.012	0.001	0.001
	4.1.99.12|g__Bacteroides.s__Bacteroides_ovatus	+	9.04E-01	0.366	0.353	-0.012	0.015	0.012
	4.1.99.12|g__Bacteroides.s__Bacteroides_fragilis	+	9.83E-01	0.148	0.138	-0.010	0.007	0.010
	4.1.99.12|g__Lachnospiraceae_unclassified.s__Eubacterium_rectale	+	9.67E-01	0.160	0.158	-0.003	0.004	0.003
	4.1.99.12|g__Acidaminococcus.s__Acidaminococcus_intestini	-	5.69E-01	0.000	0.000	0.000	0.001	0.001
	4.1.99.12|g__Adlercreutzia.s__Adlercreutzia_equolifaciens	+	6.38E-01	0.000	0.000	0.000	0.000	0.000
	4.1.99.12|g__Aggregatibacter.s__Aggregatibacter_segnis	-	3.30E-01	0.000	0.000	0.000	0.000	0.000
	4.1.99.12|g__Akkermansia.s__Akkermansia_muciniphila	+	4.61E-01	0.000	0.000	0.000	0.001	0.006
	4.1.99.12|g__Alcaligenes.s__Alcaligenes_faecalis	+	3.30E-01	0.000	0.000	0.000	0.000	0.001
	4.1.99.12|g__Alistipes.s__Alistipes_indistinctus	+	2.36E-03	0.000	0.000	0.000	0.000	0.001
	4.1.99.12|g__Alistipes.s__Alistipes_inops	+	5.89E-01	0.000	0.000	0.000	0.000	0.000
	4.1.99.12|g__Alistipes.s__Alistipes_timonensis	+	1.00E+00	0.000	0.000	0.000	0.000	0.000
	4.1.99.12|g__Allisonella.s__Allisonella_histaminiformans	+	3.93E-01	0.000	0.000	0.000	0.000	0.000
	4.1.99.12|g__Anaeromassilibacillus.s__Anaeromassilibacillus_sp_An250	+	4.27E-02	0.000	0.000	0.000	0.000	0.000
	4.1.99.12|g__Anaerostipes.s__Anaerostipes_hadrus	-	8.92E-01	0.000	0.000	0.000	0.001	0.001
	4.1.99.12|g__Anaerotignum.s__Anaerotignum_lactatifermentans	-	1.60E-01	0.000	0.000	0.000	0.000	0.000
	4.1.99.12|g__Asaccharobacter.s__Asaccharobacter_celatus	+	6.38E-01	0.000	0.000	0.000	0.000	0.000

4.1.99.12|g__Atlantibacter.s__Atlantibacter_hermannii	-	3.30E-01	0.000	0.000	0.000	0.000	0.000
4.1.99.12|g__Bacteroides.s__Bacteroides_clarus	-	5.86E-01	0.000	0.000	0.000	0.001	0.003
4.1.99.12|g__Bacteroides.s__Bacteroides_coprocola	-	6.56E-02	0.000	0.000	0.000	0.019	0.003
4.1.99.12|g__Bacteroides.s__Bacteroides_coprophilus	+	5.07E-01	0.000	0.000	0.000	0.004	0.003
4.1.99.12|g__Bacteroides.s__Bacteroides_eggerthii	-	2.91E-01	0.000	0.000	0.000	0.006	0.002
4.1.99.12|g__Bacteroides.s__Bacteroides_faecichinchillae	+	2.42E-01	0.000	0.000	0.000	0.000	0.000
4.1.99.12|g__Bacteroides.s__Bacteroides_faecis	+	4.05E-01	0.000	0.000	0.000	0.000	0.001
4.1.99.12|g__Bacteroides.s__Bacteroides_finegoldii	-	1.32E-01	0.000	0.000	0.000	0.008	0.001
4.1.99.12|g__Bacteroides.s__Bacteroides_fluxus	+	3.30E-01	0.000	0.000	0.000	0.000	0.000
4.1.99.12|g__Bacteroides.s__Bacteroides_massiliensis	-	6.38E-01	0.000	0.000	0.000	0.016	0.004
4.1.99.12|g__Bacteroides.s__Bacteroides_nordii	+	7.55E-02	0.000	0.000	0.000	0.001	0.001
4.1.99.12|g__Bacteroides.s__Bacteroides_oleiciplenus	-	1.60E-01	0.000	0.000	0.000	0.000	0.000
4.1.99.12|g__Bacteroides.s__Bacteroides_plebeius	-	3.60E-01	0.000	0.000	0.000	0.066	0.025
4.1.99.12|g__Bacteroides.s__Bacteroides_salyersiae	-	4.69E-01	0.000	0.000	0.000	0.001	0.001
4.1.99.12|g__Bacteroides.s__Bacteroides_sartorii	-	3.30E-01	0.000	0.000	0.000	0.000	0.000
4.1.99.12|g__Bacteroides.s__Bacteroides_sp_OM08_11	-	3.30E-01	0.000	0.000	0.000	0.000	0.000
4.1.99.12|g__Bacteroides.s__Bacteroides_stercorirosoris	+	5.89E-01	0.000	0.000	0.000	0.000	0.000
4.1.99.12|g__Barnesiella.s__Barnesiella_intestinihominis	+	6.22E-01	0.000	0.000	0.000	0.003	0.004
4.1.99.12|g__Blautia.s__Blautia_hansenii	-	1.75E-01	0.000	0.000	0.000	0.001	0.001
4.1.99.12|g__Blautia.s__Blautia_sp_AF19_10LB	+	3.06E-01	0.000	0.000	0.000	0.002	0.004
4.1.99.12|g__Butyricimonas.s__Butyricimonas_synergistica	+	7.47E-01	0.000	0.000	0.000	0.000	0.000
4.1.99.12|g__Butyricimonas.s__Butyricimonas_virosa	+	9.66E-02	0.000	0.000	0.000	0.000	0.001
4.1.99.12|g__Butyrivibrio.s__Butyrivibrio_crossotus	+	3.30E-01	0.000	0.000	0.000	0.000	0.006
4.1.99.12|g__Campylobacter.s__Campylobacter_concisus	-	8.19E-02	0.000	0.000	0.000	0.000	0.000
4.1.99.12|g__Campylobacter.s__Campylobacter_hominis	+	3.30E-01	0.000	0.000	0.000	0.000	0.000
4.1.99.12|g__Catenibacterium.s__Catenibacterium_mitsuokai	+	5.49E-01	0.000	0.000	0.000	0.000	0.000
4.1.99.12|g__Cetobacterium.s__Cetobacterium_somerae	-	3.30E-01	0.000	0.000	0.000	0.000	0.000
4.1.99.12|g__Chryseobacterium.s__Chryseobacterium_sp_YLOS41	+	3.30E-01	0.000	0.000	0.000	0.000	0.000
4.1.99.12|g__Citrobacter.s__Citrobacter_braakii	+	9.31E-01	0.000	0.000	0.000	0.000	0.002
4.1.99.12|g__Citrobacter.s__Citrobacter_freundii	-	8.57E-01	0.000	0.000	0.000	0.000	0.001
4.1.99.12|g__Citrobacter.s__Citrobacter_portucalensis	+	9.77E-01	0.000	0.000	0.000	0.000	0.005
4.1.99.12|g__Citrobacter.s__Citrobacter_werkmanii	+	1.00E+00	0.000	0.000	0.000	0.000	0.000
4.1.99.12|g__Citrobacter.s__Citrobacter_youngae	+	1.00E+00	0.000	0.000	0.000	0.000	0.000
4.1.99.12|g__Cloacibacillus.s__Cloacibacillus_porcorum	+	3.30E-01	0.000	0.000	0.000	0.000	0.000
4.1.99.12|g__Clostridioides.s__Clostridioides_difficile	+	7.46E-02	0.000	0.000	0.000	0.000	0.001
4.1.99.12|g__Clostridium.s__Clostridium_butyricum	+	3.30E-01	0.000	0.000	0.000	0.000	0.000
4.1.99.12|g__Clostridium.s__Clostridium_celatum	+	3.30E-01	0.000	0.000	0.000	0.000	0.000
4.1.99.12|g__Clostridium.s__Clostridium_disporicum	-	6.48E-02	0.000	0.000	0.000	0.001	0.000
4.1.99.12|g__Clostridium.s__Clostridium_perfringens	-	4.27E-02	0.000	0.000	0.000	0.000	0.000
4.1.99.12|g__Clostridium.s__Clostridium_sp_AF36_4	+	5.45E-02	0.000	0.000	0.000	0.000	0.001
4.1.99.12|g__Clostridium.s__Clostridium_sp_chh4_2	+	3.30E-01	0.000	0.000	0.000	0.000	0.000
4.1.99.12|g__Comamonas.s__Comamonas_aquatica	+	3.30E-01	0.000	0.000	0.000	0.000	0.000
4.1.99.12|g__Comamonas.s__Comamonas_kerstersii	-	1.00E+00	0.000	0.000	0.000	0.000	0.000
4.1.99.12|g__Comamonas.s__Comamonas_testosteroni	+	3.30E-01	0.000	0.000	0.000	0.000	0.000
4.1.99.12|g__Comamonas.s__Comamonas_thiooxydans	+	3.30E-01	0.000	0.000	0.000	0.000	0.000
4.1.99.12|g__Coprobacillus.s__Coprobacillus_cateniformis	+	1.10E-02	0.000	0.000	0.000	0.000	0.002
4.1.99.12|g__Coprobacter.s__Coprobacter_fastidiosus	+	6.00E-01	0.000	0.000	0.000	0.000	0.001
4.1.99.12|g__Coprobacter.s__Coprobacter_secundus	-	9.73E-01	0.000	0.000	0.000	0.000	0.000
4.1.99.12|g__Coprococcus.s__Coprococcus_catus	+	2.88E-01	0.000	0.000	0.000	0.000	0.001
4.1.99.12|g__Coprococcus.s__Coprococcus_eutactus	+	1.29E-01	0.000	0.000	0.000	0.002	0.004
4.1.99.12|g__Cronobacter.s__Cronobacter_dublinensis	-	3.30E-01	0.000	0.000	0.000	0.000	0.000
4.1.99.12|g__Desulfovibrio.s__Desulfovibrio_fairfieldensis	+	1.00E+00	0.000	0.000	0.000	0.000	0.000
4.1.99.12|g__Desulfovibrio.s__Desulfovibrio_piger	+	3.22E-01	0.000	0.000	0.000	0.001	0.002
4.1.99.12|g__Desulfovibrio.s__Desulfovibrio_sp_AM18_2	+	8.19E-02	0.000	0.000	0.000	0.000	0.000
4.1.99.12|g__Desulfovibrionaceae_unclassified.s__Desulfovibrionaceae_bac	+	1.60E-01	0.000	0.000	0.000	0.000	0.000
4.1.99.12|g__Dialister.s__Dialister_invisus	-	7.66E-01	0.000	0.000	0.000	0.002	0.006
4.1.99.12|g__Dialister.s__Dialister_pneumosintes	+	3.30E-01	0.000	0.000	0.000	0.000	0.000
4.1.99.12|g__Dialister.s__Dialister_succinatiphilus	+	9.90E-01	0.000	0.000	0.000	0.002	0.003
4.1.99.12|g__Dorea.s__Dorea_sp_OM02_2LB	+	6.89E-01	0.000	0.000	0.000	0.000	0.000
4.1.99.12|g__Eggerthella.s__Eggerthella_lenta	+	4.46E-01	0.000	0.000	0.000	0.001	0.002
4.1.99.12|g__Enterobacter.s__Enterobacter_bugandensis	-	6.89E-01	0.000	0.000	0.000	0.000	0.000
4.1.99.12|g__Enterobacter.s__Enterobacter_cloacae	+	5.75E-01	0.000	0.000	0.000	0.000	0.000
4.1.99.12|g__Enterobacter.s__Enterobacter_mori	-	1.60E-01	0.000	0.000	0.000	0.000	0.000
4.1.99.12|g__Erysipelatoclostridium.s__Clostridium_innocuum	+	6.72E-01	0.000	0.000	0.000	0.000	0.000
4.1.99.12|g__Erysipelatoclostridium.s__Clostridium_spiroforme	+	3.20E-01	0.000	0.000	0.000	0.000	0.001
4.1.99.12|g__Erysipelotrichaceae_unclassified.s__Erysipelotrichaceae_bacte	-	1.60E-01	0.000	0.000	0.000	0.000	0.000
4.1.99.12|g__Eubacterium.s__Eubacterium_ramulus	+	3.47E-01	0.000	0.000	0.000	0.000	0.000
4.1.99.12|g__Eubacterium.s__Eubacterium_sp_AF17_7	+	4.48E-01	0.000	0.000	0.000	0.000	0.000
4.1.99.12|g__Eubacterium.s__Eubacterium_sp_AM18_10LB_B	-	1.87E-01	0.000	0.000	0.000	0.000	0.000
4.1.99.12|g__Firmicutes_unclassified.s__Firmicutes_bacterium_AM10_47	-	8.41E-01	0.000	0.000	0.000	0.001	0.000
4.1.99.12|g__Flavonifractor.s__Flavonifractor_plautii	+	8.38E-02	0.000	0.000	0.000	0.000	0.000
4.1.99.12|g__Flavonifractor.s__Flavonifractor_sp_An10	+	3.30E-01	0.000	0.000	0.000	0.000	0.000

4.1.99.12|g__Fusobacterium.s__Fusobacterium_hwasookii	-	3.30E-01	0.000	0.000	0.000	0.000	0.000
4.1.99.12|g__Fusobacterium.s__Fusobacterium_mortiferum	-	6.89E-01	0.000	0.000	0.000	0.001	0.003
4.1.99.12|g__Fusobacterium.s__Fusobacterium_nucleatum	-	5.89E-01	0.000	0.000	0.000	0.000	0.000
4.1.99.12|g__Fusobacterium.s__Fusobacterium_periodonticum	-	3.08E-01	0.000	0.000	0.000	0.000	0.000
4.1.99.12|g__Fusobacterium.s__Fusobacterium_ulcerans	+	9.90E-01	0.000	0.000	0.000	0.000	0.000
4.1.99.12|g__Fusobacterium.s__Fusobacterium_varium	-	1.60E-01	0.000	0.000	0.000	0.000	0.000
4.1.99.12|g__Haemophilus.s__Haemophilus_parainfluenzae	-	3.25E-01	0.000	0.000	0.000	0.002	0.002
4.1.99.12|g__Haemophilus.s__Haemophilus_paraphrohaemolyticus	-	3.30E-01	0.000	0.000	0.000	0.000	0.000
4.1.99.12|g__Holdemanella.s__Holdemanella_biformis	+	1.87E-01	0.000	0.000	0.000	0.000	0.000
4.1.99.12|g__Hungatella.s__Hungatella_hathewayi	+	4.77E-01	0.000	0.000	0.000	0.000	0.000
4.1.99.12|g__Intestinibacter.s__Intestinibacter_bartlettii	+	2.55E-01	0.000	0.000	0.000	0.000	0.000
4.1.99.12|g__Klebsiella.s__Klebsiella_aerogenes	-	2.18E-01	0.000	0.000	0.000	0.001	0.002
4.1.99.12|g__Klebsiella.s__Klebsiella_grimontii	+	1.60E-01	0.000	0.000	0.000	0.000	0.000
4.1.99.12|g__Klebsiella.s__Klebsiella_michiganensis	-	6.55E-01	0.000	0.000	0.000	0.000	0.000
4.1.99.12|g__Kluyvera.s__Kluyvera_ascorbata	+	3.30E-01	0.000	0.000	0.000	0.000	0.000
4.1.99.12|g__Kluyvera.s__Kluyvera_cryocrescens	+	3.30E-01	0.000	0.000	0.000	0.000	0.000
4.1.99.12|g__Kluyvera.s__Kluyvera_georgiana	+	5.69E-01	0.000	0.000	0.000	0.000	0.000
4.1.99.12|g__Lachnoclostridium.s__Clostridium_aldenense	+	2.19E-01	0.000	0.000	0.000	0.000	0.000
4.1.99.12|g__Lachnoclostridium.s__Clostridium_citroniae	+	1.30E-02	0.000	0.000	0.000	0.000	0.000
4.1.99.12|g__Lachnoclostridium.s__Clostridium_clostridioforme	+	6.82E-01	0.000	0.000	0.000	0.001	0.000
4.1.99.12|g__Lachnoclostridium.s__Clostridium_symbiosum	+	1.59E-01	0.000	0.000	0.000	0.000	0.000
4.1.99.12|g__Lachnospira.s__Lachnospira_pectinoschiza	-	7.08E-01	0.000	0.000	0.000	0.005	0.003
4.1.99.12|g__Lactobacillus.s__Lactobacillus_fermentum	-	6.72E-01	0.000	0.000	0.000	0.000	0.000
4.1.99.12|g__Lactobacillus.s__Lactobacillus_reuteri	-	3.30E-01	0.000	0.000	0.000	0.000	0.000
4.1.99.12|g__Lactobacillus.s__Lactobacillus_rogosae	-	6.51E-01	0.000	0.000	0.000	0.007	0.004
4.1.99.12|g__Lactobacillus.s__Lactobacillus_sanfranciscensis	-	5.49E-01	0.000	0.000	0.000	0.000	0.000
4.1.99.12|g__Leclercia.s__Leclercia_adecarboxylata	-	8.50E-01	0.000	0.000	0.000	0.000	0.000
4.1.99.12|g__Lelliottia.s__Lelliottia_amnigena	-	3.93E-01	0.000	0.000	0.000	0.000	0.000
4.1.99.12|g__Lelliottia.s__Lelliottia_nimipressuralis	-	6.59E-01	0.000	0.000	0.000	0.000	0.001
4.1.99.12|g__Megamonas.s__Megamonas_funiformis	+	9.77E-01	0.000	0.000	0.000	0.018	0.011
4.1.99.12|g__Megamonas.s__Megamonas_rupellensis	+	9.41E-01	0.000	0.000	0.000	0.009	0.003
4.1.99.12|g__Megasphaera.s__Megasphaera_elsdenii	+	3.30E-01	0.000	0.000	0.000	0.000	0.000
4.1.99.12|g__Megasphaera.s__Megasphaera_micronuciformis	+	9.90E-01	0.000	0.000	0.000	0.000	0.000
4.1.99.12|g__Megasphaera.s__Megasphaera_stantonii	+	3.30E-01	0.000	0.000	0.000	0.000	0.000
4.1.99.12|g__Methanobrevibacter.s__Methanobrevibacter_smithii	+	3.08E-01	0.000	0.000	0.000	0.000	0.000
4.1.99.12|g__Mitsuokella.s__Mitsuokella_jalaludinii	+	3.30E-01	0.000	0.000	0.000	0.000	0.000
4.1.99.12|g__Mitsuokella.s__Mitsuokella_multacida	+	8.19E-02	0.000	0.000	0.000	0.000	0.001
4.1.99.12|g__Morganella.s__Morganella_morganii	+	5.69E-01	0.000	0.000	0.000	0.000	0.003
4.1.99.12|g__Oxalobacter.s__Oxalobacter_formigenes	+	2.91E-02	0.000	0.000	0.000	0.000	0.000
4.1.99.12|g__Pantoea.s__Pantoea_dispersa	+	3.30E-01	0.000	0.000	0.000	0.000	0.000
4.1.99.12|g__Pantoea.s__Pantoea_sesami	-	6.27E-01	0.000	0.000	0.000	0.000	0.000
4.1.99.12|g__Parabacteroides.s__Parabacteroides_chinchillae	+	3.30E-01	0.000	0.000	0.000	0.000	0.000
4.1.99.12|g__Parabacteroides.s__Parabacteroides_goldsteinii	+	2.54E-01	0.000	0.000	0.000	0.000	0.002
4.1.99.12|g__Parabacteroides.s__Parabacteroides_gordonii	+	1.60E-01	0.000	0.000	0.000	0.000	0.001
4.1.99.12|g__Parabacteroides.s__Parabacteroides_johnsonii	+	6.92E-01	0.000	0.000	0.000	0.001	0.001
4.1.99.12|g__Paraprevotella.s__Paraprevotella_clara	+	9.37E-01	0.000	0.000	0.000	0.002	0.001
4.1.99.12|g__Paraprevotella.s__Paraprevotella_xylaniphila	-	3.30E-01	0.000	0.000	0.000	0.000	0.000
4.1.99.12|g__Parasutterella.s__Parasutterella_excrementihominis	+	7.73E-01	0.000	0.000	0.000	0.002	0.002
4.1.99.12|g__Pedobacter.s__Pedobacter_himalayensis	-	5.55E-01	0.000	0.000	0.000	0.000	0.000
4.1.99.12|g__Phascolarctobacterium.s__Phascolarctobacterium_succinatuter	+	9.62E-01	0.000	0.000	0.000	0.002	0.002
4.1.99.12|g__Prevotella.s__Prevotella_copri	+	2.71E-01	0.000	0.000	0.000	0.016	0.026
4.1.99.12|g__Prevotella.s__Prevotella_corporis	+	1.60E-01	0.000	0.000	0.000	0.000	0.000
4.1.99.12|g__Prevotella.s__Prevotella_sp_109	+	5.89E-01	0.000	0.000	0.000	0.002	0.002
4.1.99.12|g__Prevotella.s__Prevotella_sp_AM42_24	-	9.90E-01	0.000	0.000	0.000	0.005	0.002
4.1.99.12|g__Prevotella.s__Prevotella_stercorea	-	9.90E-01	0.000	0.000	0.000	0.007	0.004
4.1.99.12|g__Proteus.s__Proteus_mirabilis	-	3.30E-01	0.000	0.000	0.000	0.000	0.000
4.1.99.12|g__Proteus.s__Proteus_penneri	-	5.89E-01	0.000	0.000	0.000	0.000	0.000
4.1.99.12|g__Proteus.s__Proteus_vulgaris	-	5.89E-01	0.000	0.000	0.000	0.000	0.000
4.1.99.12|g__Providencia.s__Providencia_rettgeri	+	3.30E-01	0.000	0.000	0.000	0.000	0.007
4.1.99.12|g__Pseudoflavonifractor.s__Pseudoflavonifractor_sp_An184	+	1.60E-01	0.000	0.000	0.000	0.000	0.000
4.1.99.12|g__Pyramidobacter.s__Pyramidobacter_piscolens	+	1.58E-01	0.000	0.000	0.000	0.000	0.000
4.1.99.12|g__Pyramidobacter.s__Pyramidobacter_sp_C12_8	+	3.30E-01	0.000	0.000	0.000	0.000	0.000
4.1.99.12|g__Raoultella.s__Raoultella_ornithinolytica	+	3.33E-01	0.000	0.000	0.000	0.000	0.000
4.1.99.12|g__Raoultella.s__Raoultella_planticola	+	8.19E-02	0.000	0.000	0.000	0.000	0.000
4.1.99.12|g__Roseburia.s__Roseburia_inulinivorans	-	6.78E-01	0.000	0.000	0.000	0.001	0.001
4.1.99.12|g__Ruminococcaceae_unclassified.s__Ruminococcaceae_bacteriu	+	5.89E-01	0.000	0.000	0.000	0.000	0.000
4.1.99.12|g__Ruminococcaceae_unclassified.s__Ruminococcaceae_bacteriu	+	4.27E-02	0.000	0.000	0.000	0.000	0.000
4.1.99.12|g__Ruminococcus.s__Ruminococcus_callidus	+	8.05E-01	0.000	0.000	0.000	0.001	0.001
4.1.99.12|g__Ruminococcus.s__Ruminococcus_sp_AF31_8BH	-	6.30E-01	0.000	0.000	0.000	0.001	0.001
4.1.99.12|g__Saccharomyces.s__Saccharomyces_cerevisiae	-	3.30E-01	0.000	0.000	0.000	0.000	0.000
4.1.99.12|g__Salmonella.s__Salmonella_enterica	-	9.32E-01	0.000	0.000	0.000	0.000	0.000
4.1.99.12|g__Sanguibacteroides.s__Sanguibacteroides_justesenii	+	1.60E-01	0.000	0.000	0.000	0.000	0.000

	4.1.99.12|g__Staphylococcus.s__Staphylococcus_epidermidis	-	3.30E-01	0.000	0.000	0.000	0.000	0.000
	4.1.99.12|g__Staphylococcus.s__Staphylococcus_pasteuri	-	3.30E-01	0.000	0.000	0.000	0.000	0.000
	4.1.99.12|g__Staphylococcus.s__Staphylococcus_warneri	-	3.30E-01	0.000	0.000	0.000	0.000	0.000
	4.1.99.12|g__Streptococcus.s__Streptococcus_equinus	-	3.30E-01	0.000	0.000	0.000	0.000	0.000
	4.1.99.12|g__Streptococcus.s__Streptococcus_gallolyticus	+	3.30E-01	0.000	0.000	0.000	0.000	0.000
	4.1.99.12|g__Streptococcus.s__Streptococcus_infantarius	-	3.30E-01	0.000	0.000	0.000	0.000	0.000
	4.1.99.12|g__Streptococcus.s__Streptococcus_lutetiensis	-	3.30E-01	0.000	0.000	0.000	0.000	0.000
	4.1.99.12|g__Streptococcus.s__Streptococcus_macedonicus	-	1.00E+00	0.000	0.000	0.000	0.000	0.000
	4.1.99.12|g__Streptococcus.s__Streptococcus_mitis	-	1.60E-01	0.000	0.000	0.000	0.000	0.000
	4.1.99.12|g__Streptococcus.s__Streptococcus_oralis	-	1.60E-01	0.000	0.000	0.000	0.000	0.000
	4.1.99.12|g__Streptococcus.s__Streptococcus_pasteurianus	-	1.00E+00	0.000	0.000	0.000	0.000	0.000
	4.1.99.12|g__Streptococcus.s__Streptococcus_pneumoniae	-	8.19E-02	0.000	0.000	0.000	0.000	0.000
	4.1.99.12|g__Streptococcus.s__Streptococcus_pseudopneumoniae	-	3.30E-01	0.000	0.000	0.000	0.000	0.000
	4.1.99.12|g__Sutterella.s__Sutterella_wadsworthensis	+	2.50E-01	0.000	0.000	0.000	0.001	0.002
	4.1.99.12|g__Veillonella.s__Veillonella_atypica	-	7.18E-01	0.000	0.000	0.000	0.001	0.001
	4.1.99.12|g__Veillonella.s__Veillonella_dispar	-	4.80E-01	0.000	0.000	0.000	0.000	0.000
	4.1.99.12|g__Veillonella.s__Veillonella_infantium	-	4.53E-01	0.000	0.000	0.000	0.000	0.000
	4.1.99.12|g__Veillonella.s__Veillonella_rogosae	-	5.63E-03	0.000	0.000	0.000	0.001	0.000
	4.1.99.12|g__Veillonella.s__Veillonella_tobetsuensis	-	1.29E-01	0.000	0.000	0.000	0.000	0.000
	4.1.99.12|g__Victivallales_unclassified.s__Victivallales_bacterium_CCUG_	+	3.82E-02	0.000	0.000	0.000	0.000	0.001
	4.1.99.12|g__Victivallis.s__Victivallis_vadensis	+	4.71E-01	0.000	0.000	0.000	0.001	0.001
	4.1.99.12|g__Weissella.s__Weissella_confusa	-	1.60E-01	0.000	0.000	0.000	0.000	0.000
	4.1.99.12|g__Yokenella.s__Yokenella_regensburgei	-	1.60E-01	0.000	0.000	0.000	0.000	0.000
	4.1.99.12|g__Escherichia.s__Escherichia_fergusonii	+	5.31E-01	0.000	0.012	0.012	0.001	0.001
	4.1.99.12|g__Lachnoclostridium.s__Clostridium_bolteae	+	1.51E-01	0.000	0.023	0.023	0.000	0.001
	4.1.99.12|g__Bacteroides.s__Bacteroides_intestinalis	+	4.66E-01	0.000	0.031	0.031	0.002	0.003
	4.1.99.12|g__Coprococcus.s__Coprococcus_comes	+	8.98E-01	0.000	0.036	0.036	0.001	0.001
	4.1.99.12|g__Dorea.s__Dorea_formicigenerans	+	4.73E-01	0.036	0.072	0.036	0.000	0.000
	4.1.99.12|g__Klebsiella.s__Klebsiella_pneumoniae	+	4.05E-01	0.162	0.208	0.046	0.006	0.021
	4.1.99.12|g__Blautia.s__Blautia_wexlerae	+	9.28E-01	0.185	0.237	0.052	0.005	0.006
	4.1.99.12|g__Blautia.s__Blautia_obeum	+	9.92E-01	0.312	0.367	0.055	0.004	0.007
	4.1.99.12|g__Dorea.s__Dorea_longicatena	+	4.12E-01	0.168	0.230	0.062	0.002	0.002
	4.1.99.12|g__Klebsiella.s__Klebsiella_variicola	+	1.17E-01	0.018	0.125	0.107	0.004	0.004
	4.1.99.12|g__Parabacteroides.s__Parabacteroides_merdae	+	2.57E-01	0.447	0.572	0.125	0.006	0.011
	4.1.99.12|g__Blautia.s__Ruminococcus_torques	+	6.97E-01	0.748	0.978	0.230	0.009	0.010
	4.1.99.12|g__Alistipes.s__Alistipes_finegoldii	+	4.88E-02	0.083	0.325	0.242	0.003	0.005
	4.1.99.12|g__Eubacterium.s__Eubacterium_eligens	+	7.03E-01	0.000	0.253	0.253	0.006	0.004
	4.1.99.12|g__Odoribacter.s__Odoribacter_splanchnicus	+	1.79E-01	0.101	0.375	0.274	0.002	0.003
	4.1.99.12|g__Alistipes.s__Alistipes_onderdonkii	+	3.23E-02	0.000	0.365	0.365	0.002	0.006
	4.1.99.12|g__Bacteroides.s__Bacteroides_caccae	+	4.04E-01	0.154	0.596	0.442	0.006	0.008
	4.1.99.12|g__Bacteroides.s__Bacteroides_stercoris	+	6.68E-01	0.359	0.835	0.476	0.031	0.032
	4.1.99.12|g__Bilophila.s__Bilophila_wadsworthia	+	3.54E-03	0.173	0.750	0.577	0.002	0.006
	4.1.99.12|g__Escherichia.s__Escherichia_coli	+	2.30E-01	0.509	1.369	0.860	0.030	0.041
	4.1.99.12|unclassified	+	3.22E-01	17.737	20.748	3.011	0.151	0.190
	4.1.99.12|g__Bacteroides.s__Bacteroides_uniformis	+	4.36E-01	4.074	7.331	3.257	0.051	0.082
	4.1.99.12|g__Alistipes.s__Alistipes_putredinis	+	2.77E-03	0.383	6.798	6.415	0.022	0.060
Taiwan	4.1.99.12	-	5.86E-02	165.433	152.091	-13.342	1.000	1.000
	4.1.99.12|g__Bacteroides.s__Bacteroides_vulgatus	-	7.33E-01	15.982	12.103	-3.879	0.125	0.115
	4.1.99.12|unclassified	+	9.53E-01	26.211	24.969	-1.242	0.217	0.235
	4.1.99.12|g__Bacteroides.s__Bacteroides_plebeius	-	4.80E-01	0.710	0.299	-0.410	0.055	0.036
	4.1.99.12|g__Lachnospiraceae_unclassified.s__Eubacterium_rectale	-	3.22E-02	0.313	0.089	-0.224	0.008	0.004
	4.1.99.12|g__Escherichia.s__Escherichia_coli	-	4.69E-01	0.420	0.236	-0.184	0.028	0.009
	4.1.99.12|g__Agathobaculum.s__Agathobaculum_butyriciproducens	-	1.08E-01	0.168	0.000	-0.168	0.002	0.002
	4.1.99.12|g__Coprococcus.s__Coprococcus_comes	-	1.72E-01	0.110	0.000	-0.110	0.002	0.001
	4.1.99.12|g__Roseburia.s__Roseburia_faecis	-	4.93E-01	0.224	0.117	-0.106	0.004	0.004
	4.1.99.12|g__Bacteroides.s__Bacteroides_xylanisolvens	+	9.40E-01	0.593	0.500	-0.092	0.011	0.012
	4.1.99.12|g__Clostridium.s__Clostridium_sp_AM22_11AC	-	2.92E-01	0.178	0.128	-0.050	0.002	0.002
	4.1.99.12|g__Dorea.s__Dorea_longicatena	-	9.55E-01	0.137	0.105	-0.032	0.001	0.003
	4.1.99.12|g__Bilophila.s__Bilophila_wadsworthia	-	4.00E-01	0.369	0.340	-0.029	0.007	0.005
	4.1.99.12|g__Blautia.s__Blautia_obeum	-	7.04E-01	0.102	0.077	-0.025	0.001	0.001
	4.1.99.12|g__Acidaminococcus.s__Acidaminococcus_fermentans	+	1.10E-01	0.000	0.000	0.000	0.000	0.000
	4.1.99.12|g__Acidaminococcus.s__Acidaminococcus_intestini	-	7.27E-01	0.000	0.000	0.000	0.001	0.002
	4.1.99.12|g__Adlercreutzia.s__Adlercreutzia_equolifaciens	-	3.41E-01	0.000	0.000	0.000	0.000	0.000
	4.1.99.12|g__Aeromonas.s__Aeromonas_caviae	-	2.85E-01	0.000	0.000	0.000	0.000	0.000
	4.1.99.12|g__Aggregatibacter.s__Aggregatibacter_aphrophilus	-	2.85E-01	0.000	0.000	0.000	0.000	0.000
	4.1.99.12|g__Aggregatibacter.s__Aggregatibacter_segnis	-	2.85E-01	0.000	0.000	0.000	0.000	0.000
	4.1.99.12|g__Akkermansia.s__Akkermansia_muciniphila	+	9.52E-02	0.000	0.000	0.000	0.007	0.009
	4.1.99.12|g__Alistipes.s__Alistipes_indistinctus	-	7.19E-01	0.000	0.000	0.000	0.001	0.004
	4.1.99.12|g__Alistipes.s__Alistipes_inops	+	9.13E-01	0.000	0.000	0.000	0.001	0.001
	4.1.99.12|g__Alistipes.s__Alistipes_timonensis	-	2.85E-01	0.000	0.000	0.000	0.000	0.000
	4.1.99.12|g__Allisonella.s__Allisonella_histaminiformans	-	1.16E-01	0.000	0.000	0.000	0.000	0.000
	4.1.99.12|g__Anaeromassilibacillus.s__Anaeromassilibacillus_sp_An250	-	8.57E-01	0.000	0.000	0.000	0.000	0.000

4.1.99.12|g__Anaerostipes.s__Anaerostipes_hadrus	+	3.69E-02	0.000	0.000	0.000	0.001	0.000
4.1.99.12|g__Anaerotignum.s__Anaerotignum_lactatifermentans	-	8.42E-01	0.000	0.000	0.000	0.000	0.001
4.1.99.12|g__Asaccharobacter.s__Asaccharobacter_celatus	-	3.34E-01	0.000	0.000	0.000	0.000	0.000
4.1.99.12|g__Bacteroides.s__Bacteroides_clarus	-	9.17E-01	0.000	0.000	0.000	0.001	0.005
4.1.99.12|g__Bacteroides.s__Bacteroides_coprocola	+	4.33E-01	0.000	0.000	0.000	0.014	0.017
4.1.99.12|g__Bacteroides.s__Bacteroides_coprophilus	-	6.83E-01	0.000	0.000	0.000	0.005	0.004
4.1.99.12|g__Bacteroides.s__Bacteroides_eggerthii	+	2.39E-01	0.000	0.000	0.000	0.005	0.006
4.1.99.12|g__Bacteroides.s__Bacteroides_faecichinchillae	+	3.43E-01	0.000	0.000	0.000	0.000	0.000
4.1.99.12|g__Bacteroides.s__Bacteroides_fluxus	-	9.26E-01	0.000	0.000	0.000	0.000	0.001
4.1.99.12|g__Bacteroides.s__Bacteroides_fragilis	+	3.32E-01	0.000	0.000	0.000	0.006	0.009
4.1.99.12|g__Bacteroides.s__Bacteroides_nordii	+	4.84E-01	0.000	0.000	0.000	0.000	0.001
4.1.99.12|g__Bacteroides.s__Bacteroides_oleiciplenus	+	3.68E-01	0.000	0.000	0.000	0.000	0.000
4.1.99.12|g__Bacteroides.s__Bacteroides_salyersiae	-	9.78E-01	0.000	0.000	0.000	0.004	0.003
4.1.99.12|g__Bacteroides.s__Bacteroides_sartorii	+	8.11E-01	0.000	0.000	0.000	0.007	0.002
4.1.99.12|g__Bacteroides.s__Bacteroides_sp_OM08_11	+	5.46E-01	0.000	0.000	0.000	0.000	0.000
4.1.99.12|g__Barnesiella.s__Barnesiella_intestinihominis	+	6.82E-02	0.000	0.000	0.000	0.004	0.008
4.1.99.12|g__Blautia.s__Blautia_hansenii	+	8.37E-01	0.000	0.000	0.000	0.000	0.000
4.1.99.12|g__Blautia.s__Blautia_sp_AF19_10LB	-	7.86E-01	0.000	0.000	0.000	0.000	0.000
4.1.99.12|g__Butyricicoccus.s__Butyricicoccus_pullicaecorum	-	2.85E-01	0.000	0.000	0.000	0.000	0.000
4.1.99.12|g__Butyricimonas.s__Butyricimonas_synergistica	-	8.72E-01	0.000	0.000	0.000	0.000	0.000
4.1.99.12|g__Campylobacter.s__Campylobacter_concisus	-	2.85E-01	0.000	0.000	0.000	0.000	0.000
4.1.99.12|g__Candidatus_Gastranaerophilales_unclassified.s__Candidatus_(	-	9.26E-01	0.000	0.000	0.000	0.000	0.000
4.1.99.12|g__Catenibacterium.s__Catenibacterium_mitsuokai	-	5.99E-01	0.000	0.000	0.000	0.005	0.005
4.1.99.12|g__Cetobacterium.s__Cetobacterium_somerae	+	1.96E-01	0.000	0.000	0.000	0.000	0.000
4.1.99.12|g__Citrobacter.s__Citrobacter_amalonaticus	-	2.85E-01	0.000	0.000	0.000	0.000	0.000
4.1.99.12|g__Citrobacter.s__Citrobacter_braakii	-	8.32E-01	0.000	0.000	0.000	0.000	0.000
4.1.99.12|g__Citrobacter.s__Citrobacter_farmeri	-	2.85E-01	0.000	0.000	0.000	0.000	0.000
4.1.99.12|g__Citrobacter.s__Citrobacter_freundii	-	8.42E-01	0.000	0.000	0.000	0.000	0.000
4.1.99.12|g__Citrobacter.s__Citrobacter_koseri	+	6.86E-01	0.000	0.000	0.000	0.000	0.000
4.1.99.12|g__Citrobacter.s__Citrobacter_portucalensis	+	9.28E-01	0.000	0.000	0.000	0.000	0.000
4.1.99.12|g__Citrobacter.s__Citrobacter_werkmanii	-	9.26E-01	0.000	0.000	0.000	0.000	0.000
4.1.99.12|g__Citrobacter.s__Citrobacter_youngae	-	1.25E-01	0.000	0.000	0.000	0.000	0.000
4.1.99.12|g__Cloacibacillus.s__Cloacibacillus_porcorum	+	3.70E-02	0.000	0.000	0.000	0.000	0.000
4.1.99.12|g__Clostridiales_unclassified.s__Clostridiales_bacterium_1_7_47I	-	9.05E-01	0.000	0.000	0.000	0.000	0.000
4.1.99.12|g__Clostridioides.s__Clostridioides_difficile	+	3.18E-01	0.000	0.000	0.000	0.000	0.001
4.1.99.12|g__Clostridium.s__Butyribacterium_methylotrophicum	-	9.05E-01	0.000	0.000	0.000	0.000	0.000
4.1.99.12|g__Clostridium.s__Clostridium_disporicum	+	6.70E-01	0.000	0.000	0.000	0.000	0.000
4.1.99.12|g__Clostridium.s__Clostridium_neonatale	+	3.68E-01	0.000	0.000	0.000	0.000	0.000
4.1.99.12|g__Clostridium.s__Clostridium_perfringens	-	1.25E-01	0.000	0.000	0.000	0.000	0.000
4.1.99.12|g__Clostridium.s__Clostridium_sp_AF36_4	+	8.17E-01	0.000	0.000	0.000	0.001	0.000
4.1.99.12|g__Clostridium.s__Clostridium_sp_AM58_1XD	-	2.85E-01	0.000	0.000	0.000	0.000	0.000
4.1.99.12|g__Coprobacillus.s__Coprobacillus_cateniformis	+	4.08E-01	0.000	0.000	0.000	0.000	0.000
4.1.99.12|g__Coprobacter.s__Coprobacter_fastidiosus	+	1.19E-01	0.000	0.000	0.000	0.001	0.003
4.1.99.12|g__Coprobacter.s__Coprobacter_secundus	+	3.03E-01	0.000	0.000	0.000	0.000	0.001
4.1.99.12|g__Coprococcus.s__Coprococcus_catus	-	3.70E-01	0.000	0.000	0.000	0.000	0.000
4.1.99.12|g__Coprococcus.s__Coprococcus_eutactus	+	6.62E-01	0.000	0.000	0.000	0.001	0.001
4.1.99.12|g__Desulfovibrio.s__Desulfovibrio_desulfuricans	+	3.68E-01	0.000	0.000	0.000	0.000	0.000
4.1.99.12|g__Desulfovibrio.s__Desulfovibrio_fairfieldensis	+	1.95E-01	0.000	0.000	0.000	0.000	0.000
4.1.99.12|g__Desulfovibrio.s__Desulfovibrio_legallii	+	3.68E-01	0.000	0.000	0.000	0.000	0.000
4.1.99.12|g__Desulfovibrio.s__Desulfovibrio_piger	-	9.07E-01	0.000	0.000	0.000	0.002	0.002
4.1.99.12|g__Desulfovibrio.s__Desulfovibrio_sp_AM18_2	+	3.68E-01	0.000	0.000	0.000	0.000	0.000
4.1.99.12|g__Desulfovibrionaceae_unclassified.s__Desulfovibrionaceae_bac	-	1.73E-01	0.000	0.000	0.000	0.000	0.000
4.1.99.12|g__Dialister.s__Dialister_invisus	-	2.42E-01	0.000	0.000	0.000	0.001	0.000
4.1.99.12|g__Dialister.s__Dialister_succinatiphilus	-	3.56E-01	0.000	0.000	0.000	0.002	0.001
4.1.99.12|g__Dorea.s__Dorea_formicigenerans	+	4.04E-01	0.000	0.000	0.000	0.000	0.000
4.1.99.12|g__Dorea.s__Dorea_sp_OM02_2LB	+	3.68E-01	0.000	0.000	0.000	0.000	0.000
4.1.99.12|g__Eggerthella.s__Eggerthella_lenta	+	5.42E-01	0.000	0.000	0.000	0.000	0.001
4.1.99.12|g__Enterobacter.s__Enterobacter_bugandensis	-	2.27E-01	0.000	0.000	0.000	0.000	0.000
4.1.99.12|g__Enterobacter.s__Enterobacter_cloacae	-	5.48E-01	0.000	0.000	0.000	0.001	0.000
4.1.99.12|g__Enterococcus.s__Enterococcus_faecium	+	3.68E-01	0.000	0.000	0.000	0.000	0.000
4.1.99.12|g__Erysipelatoclostridium.s__Clostridium_innocuum	+	1.10E-01	0.000	0.000	0.000	0.000	0.000
4.1.99.12|g__Erysipelatoclostridium.s__Clostridium_spiroforme	+	2.48E-01	0.000	0.000	0.000	0.000	0.000
4.1.99.12|g__Erysipelotrichaceae_unclassified.s__Erysipelotrichaceae_bacte	-	2.85E-01	0.000	0.000	0.000	0.000	0.000
4.1.99.12|g__Escherichia.s__Escherichia_fergusonii	-	2.98E-01	0.000	0.000	0.000	0.001	0.000
4.1.99.12|g__Eubacterium.s__Eubacterium_callanderi	-	9.05E-01	0.000	0.000	0.000	0.000	0.000
4.1.99.12|g__Eubacterium.s__Eubacterium_limosum	+	3.68E-01	0.000	0.000	0.000	0.000	0.000
4.1.99.12|g__Eubacterium.s__Eubacterium_ramulus	-	5.70E-01	0.000	0.000	0.000	0.000	0.000
4.1.99.12|g__Eubacterium.s__Eubacterium_sp_AF17_7	+	6.97E-01	0.000	0.000	0.000	0.000	0.000
4.1.99.12|g__Eubacterium.s__Eubacterium_sp_AM18_10LB_B	-	9.26E-01	0.000	0.000	0.000	0.000	0.000
4.1.99.12|g__Faecalitalea.s__Faecalitalea_cylindroides	-	9.26E-01	0.000	0.000	0.000	0.000	0.000
4.1.99.12|g__Firmicutes_unclassified.s__Firmicutes_bacterium_AM10_47	+	6.36E-02	0.000	0.000	0.000	0.000	0.000
4.1.99.12|g__Flavonifractor.s__Flavonifractor_plautii	+	3.19E-01	0.000	0.000	0.000	0.000	0.000

4.1.99.12|g__Flavonifractor.s__Flavonifractor_sp_An10	+	3.68E-01	0.000	0.000	0.000	0.000	0.000
4.1.99.12|g__Fusobacterium.s__Fusobacterium_mortiferum	-	3.03E-01	0.000	0.000	0.000	0.003	0.001
4.1.99.12|g__Fusobacterium.s__Fusobacterium_nucleatum	+	3.68E-01	0.000	0.000	0.000	0.000	0.000
4.1.99.12|g__Fusobacterium.s__Fusobacterium_ulcerans	+	2.28E-01	0.000	0.000	0.000	0.000	0.001
4.1.99.12|g__Fusobacterium.s__Fusobacterium_varium	-	2.42E-01	0.000	0.000	0.000	0.001	0.000
4.1.99.12|g__Haemophilus.s__Haemophilus_parainfluenzae	-	1.08E-01	0.000	0.000	0.000	0.004	0.001
4.1.99.12|g__Holdemanella.s__Holdemanella_biformis	+	3.68E-01	0.000	0.000	0.000	0.000	0.000
4.1.99.12|g__Hungatella.s__Hungatella_hathewayi	+	7.82E-01	0.000	0.000	0.000	0.000	0.001
4.1.99.12|g__Intestinibacter.s__Intestinibacter_bartlettii	+	3.68E-01	0.000	0.000	0.000	0.000	0.000
4.1.99.12|g__Klebsiella.s__Klebsiella_aerogenes	+	2.56E-01	0.000	0.000	0.000	0.000	0.000
4.1.99.12|g__Klebsiella.s__Klebsiella_grimontii	-	9.05E-01	0.000	0.000	0.000	0.000	0.000
4.1.99.12|g__Klebsiella.s__Klebsiella_michiganensis	-	9.05E-01	0.000	0.000	0.000	0.000	0.000
4.1.99.12|g__Klebsiella.s__Klebsiella_pneumoniae	-	3.54E-01	0.000	0.000	0.000	0.009	0.003
4.1.99.12|g__Klebsiella.s__Klebsiella_variicola	+	8.08E-01	0.000	0.000	0.000	0.001	0.001
4.1.99.12|g__Kluyvera.s__Kluyvera_ascorbata	-	2.85E-01	0.000	0.000	0.000	0.000	0.000
4.1.99.12|g__Kluyvera.s__Kluyvera_georgiana	-	2.85E-01	0.000	0.000	0.000	0.000	0.000
4.1.99.12|g__Kluyvera.s__Kluyvera_intermedia	+	3.68E-01	0.000	0.000	0.000	0.000	0.000
4.1.99.12|g__Lachnoclostridium.s__Clostridium_aldenense	+	2.44E-01	0.000	0.000	0.000	0.000	0.000
4.1.99.12|g__Lachnoclostridium.s__Clostridium_bolteae	+	8.18E-01	0.000	0.000	0.000	0.001	0.001
4.1.99.12|g__Lachnoclostridium.s__Clostridium_citroniae	-	9.38E-01	0.000	0.000	0.000	0.000	0.000
4.1.99.12|g__Lachnoclostridium.s__Clostridium_clostridioforme	-	2.40E-01	0.000	0.000	0.000	0.000	0.000
4.1.99.12|g__Lachnoclostridium.s__Clostridium_symbiosum	+	4.66E-02	0.000	0.000	0.000	0.000	0.001
4.1.99.12|g__Lachnoclostridium.s__Lachnoclostridium_sp_An138	+	3.68E-01	0.000	0.000	0.000	0.000	0.000
4.1.99.12|g__Lachnospira.s__Lachnospira_pectinoschiza	-	2.57E-01	0.000	0.000	0.000	0.001	0.001
4.1.99.12|g__Lactobacillus.s__Lactobacillus_fermentum	-	9.05E-01	0.000	0.000	0.000	0.000	0.000
4.1.99.12|g__Lactobacillus.s__Lactobacillus_rogosae	-	1.71E-01	0.000	0.000	0.000	0.001	0.001
4.1.99.12|g__Lactococcus.s__Lactococcus_lactis	-	2.85E-01	0.000	0.000	0.000	0.000	0.000
4.1.99.12|g__Leclercia.s__Leclercia_adecarboxylata	-	1.55E-01	0.000	0.000	0.000	0.000	0.000
4.1.99.12|g__Lelliottia.s__Lelliottia_amnigena	-	1.25E-01	0.000	0.000	0.000	0.000	0.000
4.1.99.12|g__Lelliottia.s__Lelliottia_nimipressuralis	-	1.24E-01	0.000	0.000	0.000	0.000	0.000
4.1.99.12|g__Megamonas.s__Megamonas_funiformis	-	5.18E-01	0.000	0.000	0.000	0.006	0.003
4.1.99.12|g__Megamonas.s__Megamonas_rupellensis	-	6.08E-01	0.000	0.000	0.000	0.001	0.000
4.1.99.12|g__Megasphaera.s__Megasphaera_elsdenii	-	1.25E-01	0.000	0.000	0.000	0.000	0.000
4.1.99.12|g__Megasphaera.s__Megasphaera_hexanoica	+	3.68E-01	0.000	0.000	0.000	0.000	0.000
4.1.99.12|g__Megasphaera.s__Megasphaera_micronuciformis	+	6.86E-01	0.000	0.000	0.000	0.000	0.000
4.1.99.12|g__Megasphaera.s__Megasphaera_stantonii	-	2.97E-01	0.000	0.000	0.000	0.000	0.000
4.1.99.12|g__Metakosakonia.s__Kluyvera_intestini	+	3.68E-01	0.000	0.000	0.000	0.000	0.000
4.1.99.12|g__Methanobrevibacter.s__Methanobrevibacter_smithii	+	4.10E-02	0.000	0.000	0.000	0.000	0.009
4.1.99.12|g__Mitsuokella.s__Mitsuokella_jalaludinii	-	5.21E-01	0.000	0.000	0.000	0.000	0.000
4.1.99.12|g__Mitsuokella.s__Mitsuokella_multacida	-	7.90E-01	0.000	0.000	0.000	0.000	0.000
4.1.99.12|g__Odoribacter.s__Odoribacter_laneus	+	3.87E-01	0.000	0.000	0.000	0.000	0.002
4.1.99.12|g__Oxalobacter.s__Oxalobacter_formigenes	-	6.94E-01	0.000	0.000	0.000	0.000	0.000
4.1.99.12|g__Paeniclostridium.s__Paeniclostridium_sordellii	+	3.68E-01	0.000	0.000	0.000	0.000	0.000
4.1.99.12|g__Pantoea.s__Pantoea_sesami	-	2.83E-01	0.000	0.000	0.000	0.000	0.000
4.1.99.12|g__Parabacteroides.s__Parabacteroides_chinchillae	-	9.05E-01	0.000	0.000	0.000	0.000	0.000
4.1.99.12|g__Parabacteroides.s__Parabacteroides_goldsteinii	+	3.14E-02	0.000	0.000	0.000	0.001	0.004
4.1.99.12|g__Parabacteroides.s__Parabacteroides_gordonii	-	3.13E-01	0.000	0.000	0.000	0.000	0.000
4.1.99.12|g__Parabacteroides.s__Parabacteroides_johnsonii	+	8.00E-01	0.000	0.000	0.000	0.001	0.001
4.1.99.12|g__Paraprevotella.s__Paraprevotella_clara	-	5.33E-01	0.000	0.000	0.000	0.005	0.005
4.1.99.12|g__Paraprevotella.s__Paraprevotella_xylaniphila	+	8.39E-02	0.000	0.000	0.000	0.000	0.002
4.1.99.12|g__Parasutterella.s__Parasutterella_excrementihominis	+	7.85E-01	0.000	0.000	0.000	0.002	0.001
4.1.99.12|g__Pedobacter.s__Pedobacter_himalayensis	-	1.16E-01	0.000	0.000	0.000	0.000	0.000
4.1.99.12|g__Phascolarctobacterium.s__Phascolarctobacterium_succinatuter	+	3.93E-01	0.000	0.000	0.000	0.002	0.002
4.1.99.12|g__Phytobacter.s__Phytobacter_ursingii	+	3.68E-01	0.000	0.000	0.000	0.000	0.000
4.1.99.12|g__Plesiomonas.s__Plesiomonas_shigelloides	-	2.85E-01	0.000	0.000	0.000	0.001	0.000
4.1.99.12|g__Prevotella.s__Prevotella_bivia	-	2.85E-01	0.000	0.000	0.000	0.000	0.000
4.1.99.12|g__Prevotella.s__Prevotella_buccae	-	2.85E-01	0.000	0.000	0.000	0.004	0.000
4.1.99.12|g__Prevotella.s__Prevotella_copri	-	7.68E-01	0.000	0.000	0.000	0.022	0.006
4.1.99.12|g__Prevotella.s__Prevotella_sp_109	+	6.55E-01	0.000	0.000	0.000	0.011	0.007
4.1.99.12|g__Prevotella.s__Prevotella_sp_885	-	9.05E-01	0.000	0.000	0.000	0.005	0.001
4.1.99.12|g__Prevotella.s__Prevotella_sp_AM42_24	-	2.85E-01	0.000	0.000	0.000	0.003	0.000
4.1.99.12|g__Prevotella.s__Prevotella_stercorea	+	8.79E-01	0.000	0.000	0.000	0.027	0.011
4.1.99.12|g__Prevotella.s__Prevotella_timonensis	+	3.68E-01	0.000	0.000	0.000	0.000	0.000
4.1.99.12|g__Pseudocitrobacter.s__Pseudocitrobacter_faecalis	-	9.05E-01	0.000	0.000	0.000	0.000	0.000
4.1.99.12|g__Pseudoflavonifractor.s__Pseudoflavonifractor_sp_An184	+	1.96E-01	0.000	0.000	0.000	0.000	0.000
4.1.99.12|g__Pyramidobacter.s__Pyramidobacter_piscolens	-	8.57E-01	0.000	0.000	0.000	0.000	0.000
4.1.99.12|g__Pyramidobacter.s__Pyramidobacter_sp_C12_8	+	3.68E-01	0.000	0.000	0.000	0.000	0.000
4.1.99.12|g__Raoultella.s__Raoultella_ornithinolytica	-	5.10E-01	0.000	0.000	0.000	0.001	0.000
4.1.99.12|g__Raoultella.s__Raoultella_planticola	+	6.70E-01	0.000	0.000	0.000	0.000	0.000
4.1.99.12|g__Roseburia.s__Roseburia_intestinalis	+	8.84E-01	0.000	0.000	0.000	0.004	0.002
4.1.99.12|g__Roseburia.s__Roseburia_inulinivorans	-	4.51E-01	0.000	0.000	0.000	0.001	0.001
4.1.99.12|g__Ruminococcaceae_unclassified.s__Ruminococcaceae_bacteriu	-	3.76E-01	0.000	0.000	0.000	0.000	0.000

<table>
<tr><td></td><td>4.1.99.12|g__Ruminococcaceae_unclassified.s__Ruminococcaceae_bacteriu</td><td>+</td><td>2.28E-01</td><td>0.000</td><td>0.000</td><td>0.000</td><td>0.000</td><td>0.000</td></tr>
<tr><td></td><td>4.1.99.12|g__Ruminococcus.s__Ruminococcus_callidus</td><td>-</td><td>8.97E-01</td><td>0.000</td><td>0.000</td><td>0.000</td><td>0.001</td><td>0.000</td></tr>
<tr><td></td><td>4.1.99.12|g__Ruminococcus.s__Ruminococcus_sp_AF31_8BH</td><td>+</td><td>7.88E-02</td><td>0.000</td><td>0.000</td><td>0.000</td><td>0.000</td><td>0.000</td></tr>
<tr><td></td><td>4.1.99.12|g__Salmonella.s__Salmonella_enterica</td><td>+</td><td>6.70E-01</td><td>0.000</td><td>0.000</td><td>0.000</td><td>0.000</td><td>0.000</td></tr>
<tr><td></td><td>4.1.99.12|g__Sanguibacteroides.s__Sanguibacteroides_justesenii</td><td>+</td><td>1.34E-01</td><td>0.000</td><td>0.000</td><td>0.000</td><td>0.000</td><td>0.000</td></tr>
<tr><td></td><td>4.1.99.12|g__Senegalimassilia.s__Senegalimassilia_anaerobia</td><td>+</td><td>2.20E-01</td><td>0.000</td><td>0.000</td><td>0.000</td><td>0.000</td><td>0.000</td></tr>
<tr><td></td><td>4.1.99.12|g__Slackia.s__Slackia_isoflavoniconvertens</td><td>+</td><td>3.68E-01</td><td>0.000</td><td>0.000</td><td>0.000</td><td>0.000</td><td>0.000</td></tr>
<tr><td></td><td>4.1.99.12|g__Streptococcus.s__Streptococcus_gallolyticus</td><td>+</td><td>1.96E-01</td><td>0.000</td><td>0.000</td><td>0.000</td><td>0.000</td><td>0.000</td></tr>
<tr><td></td><td>4.1.99.12|g__Streptococcus.s__Streptococcus_macedonicus</td><td>+</td><td>1.96E-01</td><td>0.000</td><td>0.000</td><td>0.000</td><td>0.000</td><td>0.000</td></tr>
<tr><td></td><td>4.1.99.12|g__Streptococcus.s__Streptococcus_pasteurianus</td><td>+</td><td>1.96E-01</td><td>0.000</td><td>0.000</td><td>0.000</td><td>0.000</td><td>0.000</td></tr>
<tr><td></td><td>4.1.99.12|g__Streptococcus.s__Streptococcus_pneumoniae</td><td>+</td><td>3.68E-01</td><td>0.000</td><td>0.000</td><td>0.000</td><td>0.000</td><td>0.000</td></tr>
<tr><td></td><td>4.1.99.12|g__Turicimonas.s__Turicimonas_muris</td><td>+</td><td>3.68E-01</td><td>0.000</td><td>0.000</td><td>0.000</td><td>0.000</td><td>0.000</td></tr>
<tr><td></td><td>4.1.99.12|g__Veillonella.s__Veillonella_atypica</td><td>-</td><td>2.15E-01</td><td>0.000</td><td>0.000</td><td>0.000</td><td>0.000</td><td>0.000</td></tr>
<tr><td></td><td>4.1.99.12|g__Veillonella.s__Veillonella_dispar</td><td>-</td><td>7.28E-01</td><td>0.000</td><td>0.000</td><td>0.000</td><td>0.000</td><td>0.000</td></tr>
<tr><td></td><td>4.1.99.12|g__Veillonella.s__Veillonella_infantium</td><td>-</td><td>3.14E-01</td><td>0.000</td><td>0.000</td><td>0.000</td><td>0.000</td><td>0.000</td></tr>
<tr><td></td><td>4.1.99.12|g__Veillonella.s__Veillonella_parvula</td><td>-</td><td>6.58E-01</td><td>0.000</td><td>0.000</td><td>0.000</td><td>0.000</td><td>0.000</td></tr>
<tr><td></td><td>4.1.99.12|g__Veillonella.s__Veillonella_rogosae</td><td>-</td><td>7.81E-02</td><td>0.000</td><td>0.000</td><td>0.000</td><td>0.000</td><td>0.000</td></tr>
<tr><td></td><td>4.1.99.12|g__Veillonella.s__Veillonella_tobetsuensis</td><td>-</td><td>8.87E-01</td><td>0.000</td><td>0.000</td><td>0.000</td><td>0.000</td><td>0.000</td></tr>
<tr><td></td><td>4.1.99.12|g__Veillonellaceae_unclassified.s__Veillonellaceae_bacterium</td><td>+</td><td>3.68E-01</td><td>0.000</td><td>0.000</td><td>0.000</td><td>0.000</td><td>0.000</td></tr>
<tr><td></td><td>4.1.99.12|g__Vibrio.s__Vibrio_parahaemolyticus</td><td>-</td><td>2.85E-01</td><td>0.000</td><td>0.000</td><td>0.000</td><td>0.000</td><td>0.000</td></tr>
<tr><td></td><td>4.1.99.12|g__Victivallales_unclassified.s__Victivallales_bacterium_CCUG_</td><td>+</td><td>9.02E-02</td><td>0.000</td><td>0.000</td><td>0.000</td><td>0.001</td><td>0.002</td></tr>
<tr><td></td><td>4.1.99.12|g__Victivallis.s__Victivallis_vadensis</td><td>+</td><td>5.40E-02</td><td>0.000</td><td>0.000</td><td>0.000</td><td>0.000</td><td>0.001</td></tr>
<tr><td></td><td>4.1.99.12|g__Weissella.s__Weissella_confusa</td><td>+</td><td>3.68E-01</td><td>0.000</td><td>0.000</td><td>0.000</td><td>0.000</td><td>0.000</td></tr>
<tr><td></td><td>4.1.99.12|g__Blautia.s__Blautia_wexlerae</td><td>+</td><td>9.97E-01</td><td>0.032</td><td>0.043</td><td>0.011</td><td>0.000</td><td>0.001</td></tr>
<tr><td></td><td>4.1.99.12|g__Bacteroides.s__Bacteroides_massiliensis</td><td>-</td><td>9.77E-01</td><td>0.382</td><td>0.397</td><td>0.015</td><td>0.017</td><td>0.017</td></tr>
<tr><td></td><td>4.1.99.12|g__Bacteroides.s__Bacteroides_finegoldii</td><td>+</td><td>2.15E-01</td><td>0.000</td><td>0.027</td><td>0.027</td><td>0.003</td><td>0.004</td></tr>
<tr><td></td><td>4.1.99.12|g__Roseburia.s__Roseburia_hominis</td><td>+</td><td>3.64E-01</td><td>0.147</td><td>0.212</td><td>0.065</td><td>0.002</td><td>0.003</td></tr>
<tr><td></td><td>4.1.99.12|g__Alistipes.s__Alistipes_finegoldii</td><td>+</td><td>5.39E-01</td><td>0.147</td><td>0.226</td><td>0.079</td><td>0.004</td><td>0.008</td></tr>
<tr><td></td><td>4.1.99.12|g__Blautia.s__Ruminococcus_torques</td><td>+</td><td>9.38E-01</td><td>0.502</td><td>0.583</td><td>0.081</td><td>0.011</td><td>0.010</td></tr>
<tr><td></td><td>4.1.99.12|g__Bacteroides.s__Bacteroides_intestinalis</td><td>+</td><td>5.35E-02</td><td>0.000</td><td>0.090</td><td>0.090</td><td>0.002</td><td>0.008</td></tr>
<tr><td></td><td>4.1.99.12|g__Butyricimonas.s__Butyricimonas_virosa</td><td>+</td><td>2.11E-02</td><td>0.000</td><td>0.124</td><td>0.124</td><td>0.001</td><td>0.003</td></tr>
<tr><td></td><td>4.1.99.12|g__Klebsiella.s__Klebsiella_oxytoca</td><td>+</td><td>2.89E-01</td><td>0.241</td><td>0.385</td><td>0.144</td><td>0.003</td><td>0.006</td></tr>
<tr><td></td><td>4.1.99.12|g__Eubacterium.s__Eubacterium_eligens</td><td>+</td><td>9.24E-01</td><td>0.252</td><td>0.413</td><td>0.160</td><td>0.006</td><td>0.006</td></tr>
<tr><td></td><td>4.1.99.12|g__Alistipes.s__Alistipes_onderdonkii</td><td>+</td><td>3.77E-01</td><td>0.121</td><td>0.285</td><td>0.164</td><td>0.005</td><td>0.008</td></tr>
<tr><td></td><td>4.1.99.12|g__Faecalibacterium.s__Faecalibacterium_prausnitzii</td><td>+</td><td>6.32E-01</td><td>2.847</td><td>3.014</td><td>0.167</td><td>0.031</td><td>0.032</td></tr>
<tr><td></td><td>4.1.99.12|g__Sutterella.s__Sutterella_wadsworthensis</td><td>+</td><td>8.30E-01</td><td>0.342</td><td>0.513</td><td>0.171</td><td>0.009</td><td>0.010</td></tr>
<tr><td></td><td>4.1.99.12|g__Bacteroides.s__Bacteroides_ovatus</td><td>+</td><td>1.92E-01</td><td>0.184</td><td>0.444</td><td>0.260</td><td>0.007</td><td>0.010</td></tr>
<tr><td></td><td>4.1.99.12|g__Bacteroides.s__Bacteroides_dorei</td><td>+</td><td>4.90E-01</td><td>1.745</td><td>2.090</td><td>0.345</td><td>0.031</td><td>0.048</td></tr>
<tr><td></td><td>4.1.99.12|g__Odoribacter.s__Odoribacter_splanchnicus</td><td>+</td><td>5.28E-02</td><td>0.210</td><td>0.663</td><td>0.452</td><td>0.005</td><td>0.006</td></tr>
<tr><td></td><td>4.1.99.12|g__Phascolarctobacterium.s__Phascolarctobacterium_faecium</td><td>+</td><td>9.15E-01</td><td>0.253</td><td>0.706</td><td>0.453</td><td>0.008</td><td>0.008</td></tr>
<tr><td></td><td>4.1.99.12|g__Bacteroides.s__Bacteroides_cellulosilyticus</td><td>+</td><td>1.18E-02</td><td>0.018</td><td>0.522</td><td>0.504</td><td>0.011</td><td>0.012</td></tr>
<tr><td></td><td>4.1.99.12|g__Bacteroides.s__Bacteroides_stercoris</td><td>+</td><td>9.97E-01</td><td>1.372</td><td>1.945</td><td>0.573</td><td>0.035</td><td>0.032</td></tr>
<tr><td></td><td>4.1.99.12|g__Bacteroides.s__Bacteroides_thetaiotaomicron</td><td>+</td><td>6.20E-02</td><td>0.753</td><td>1.381</td><td>0.628</td><td>0.011</td><td>0.017</td></tr>
<tr><td></td><td>4.1.99.12|g__Parabacteroides.s__Parabacteroides_distasonis</td><td>+</td><td>1.72E-02</td><td>0.772</td><td>1.499</td><td>0.727</td><td>0.011</td><td>0.017</td></tr>
<tr><td></td><td>4.1.99.12|g__Parabacteroides.s__Parabacteroides_merdae</td><td>+</td><td>1.62E-02</td><td>0.441</td><td>1.251</td><td>0.810</td><td>0.006</td><td>0.012</td></tr>
<tr><td></td><td>4.1.99.12|g__Bacteroides.s__Bacteroides_caccae</td><td>+</td><td>2.37E-02</td><td>0.371</td><td>1.508</td><td>1.137</td><td>0.009</td><td>0.013</td></tr>
<tr><td></td><td>4.1.99.12|g__Bacteroides.s__Bacteroides_uniformis</td><td>+</td><td>5.46E-01</td><td>5.669</td><td>7.611</td><td>1.942</td><td>0.070</td><td>0.067</td></tr>
<tr><td></td><td>4.1.99.12|g__Alistipes.s__Alistipes_putredinis</td><td>+</td><td>3.92E-01</td><td>2.514</td><td>5.272</td><td>2.758</td><td>0.037</td><td>0.048</td></tr>
</table>

<table>
<tr><th></th><th></th><th></th><th></th><th colspan="3">Fractional CPM (median)</th><th colspan="2">Relative CPM (average)</th></tr>
<tr><th>Country</th><th>EC number|Bacteria</th><th>Increased or decreased in PD</th><th>p-value</th><th>Control</th><th>PD</th><th>PD - Control</th><th>Control</th><th>PD</th></tr>
<tr><td>Japan</td><td>2.5.1.78</td><td>-</td><td>1.59E-04</td><td>109.917</td><td>95.671</td><td>-14.246</td><td>1.000</td><td>1.000</td></tr>
<tr><td></td><td>2.5.1.78|g__Faecalibacterium.s__Faecalibacterium_prausnitzii</td><td>-</td><td>2.13E-02</td><td>9.652</td><td>5.667</td><td>-3.985</td><td>0.094</td><td>0.076</td></tr>
<tr><td></td><td>2.5.1.78|g__Blautia.s__Blautia_obeum</td><td>-</td><td>8.72E-07</td><td>3.531</td><td>1.604</td><td>-1.927</td><td>0.038</td><td>0.023</td></tr>
<tr><td></td><td>2.5.1.78|g__Dorea.s__Dorea_longicatena</td><td>-</td><td>3.08E-02</td><td>1.455</td><td>0.000</td><td>-1.455</td><td>0.018</td><td>0.012</td></tr>
<tr><td></td><td>2.5.1.78|g__Blautia.s__Blautia_wexlerae</td><td>-</td><td>2.27E-04</td><td>2.771</td><td>1.531</td><td>-1.240</td><td>0.039</td><td>0.023</td></tr>
<tr><td></td><td>2.5.1.78|g__Lachnospiraceae_unclassified.s__Eubacterium_rectale</td><td>-</td><td>1.19E-01</td><td>0.938</td><td>0.000</td><td>-0.938</td><td>0.026</td><td>0.019</td></tr>
<tr><td></td><td>2.5.1.78|g__Blautia.s__Ruminococcus_torques</td><td>-</td><td>3.45E-02</td><td>0.488</td><td>0.000</td><td>-0.488</td><td>0.026</td><td>0.017</td></tr>
<tr><td></td><td>2.5.1.78|g__Anaerostipes.s__Anaerostipes_hadrus</td><td>-</td><td>1.65E-01</td><td>1.885</td><td>1.412</td><td>-0.472</td><td>0.032</td><td>0.027</td></tr>
<tr><td></td><td>2.5.1.78|g__Bacteroides.s__Bacteroides_vulgatus</td><td>-</td><td>6.98E-01</td><td>1.491</td><td>1.076</td><td>-0.416</td><td>0.040</td><td>0.033</td></tr>
<tr><td></td><td>2.5.1.78|g__Agathobaculum.s__Agathobaculum_butyriciproducens</td><td>-</td><td>7.17E-02</td><td>0.367</td><td>0.000</td><td>-0.367</td><td>0.005</td><td>0.004</td></tr>
<tr><td></td><td>2.5.1.78|g__Dorea.s__Dorea_formicigenerans</td><td>-</td><td>7.44E-01</td><td>0.178</td><td>0.000</td><td>-0.178</td><td>0.005</td><td>0.005</td></tr>
<tr><td></td><td>2.5.1.78|g__Acidaminococcus.s__Acidaminococcus_fermentans</td><td>+</td><td>3.85E-01</td><td>0.000</td><td>0.000</td><td>0.000</td><td>0.000</td><td>0.000</td></tr>
<tr><td></td><td>2.5.1.78|g__Acidaminococcus.s__Acidaminococcus_intestini</td><td>-</td><td>8.63E-01</td><td>0.000</td><td>0.000</td><td>0.000</td><td>0.007</td><td>0.003</td></tr>
<tr><td></td><td>2.5.1.78|g__Actinomyces.s__Actinomyces_naeslundii</td><td>+</td><td>7.31E-01</td><td>0.000</td><td>0.000</td><td>0.000</td><td>0.000</td><td>0.000</td></tr>
<tr><td></td><td>2.5.1.78|g__Actinomyces.s__Actinomyces_viscosus</td><td>-</td><td>1.99E-01</td><td>0.000</td><td>0.000</td><td>0.000</td><td>0.000</td><td>0.000</td></tr>
<tr><td></td><td>2.5.1.78|g__Adlercreutzia.s__Adlercreutzia_equolifaciens</td><td>+</td><td>1.67E-01</td><td>0.000</td><td>0.000</td><td>0.000</td><td>0.000</td><td>0.001</td></tr>
<tr><td></td><td>2.5.1.78|g__Akkermansia.s__Akkermansia_muciniphila</td><td>+</td><td>8.96E-03</td><td>0.000</td><td>0.000</td><td>0.000</td><td>0.004</td><td>0.029</td></tr>
<tr><td></td><td>2.5.1.78|g__Alistipes.s__Alistipes_indistinctus</td><td>+</td><td>7.97E-02</td><td>0.000</td><td>0.000</td><td>0.000</td><td>0.000</td><td>0.002</td></tr>
<tr><td></td><td>2.5.1.78|g__Alistipes.s__Alistipes_inops</td><td>-</td><td>5.93E-01</td><td>0.000</td><td>0.000</td><td>0.000</td><td>0.002</td><td>0.001</td></tr>
<tr><td></td><td>2.5.1.78|g__Alistipes.s__Alistipes_onderdonkii</td><td>+</td><td>5.78E-02</td><td>0.000</td><td>0.000</td><td>0.000</td><td>0.002</td><td>0.008</td></tr>
<tr><td></td><td>2.5.1.78|g__Alistipes.s__Alistipes_sp_An31A</td><td>-</td><td>8.57E-01</td><td>0.000</td><td>0.000</td><td>0.000</td><td>0.001</td><td>0.001</td></tr>
<tr><td></td><td>2.5.1.78|g__Alistipes.s__Alistipes_sp_An66</td><td>+</td><td>3.85E-01</td><td>0.000</td><td>0.000</td><td>0.000</td><td>0.000</td><td>0.000</td></tr>
</table>

2.5.1.78|g__Alistipes.s__Alistipes_sp_CHKCI003	-	2.62E-01	0.000	0.000	0.000	0.000	0.000
2.5.1.78|g__Alistipes.s__Alistipes_timonensis	+	7.31E-01	0.000	0.000	0.000	0.000	0.000
2.5.1.78|g__Allisonella.s__Allisonella_histaminiformans	-	4.32E-01	0.000	0.000	0.000	0.000	0.000
2.5.1.78|g__Anaeroglobus.s__Anaeroglobus_geminatus	+	3.85E-01	0.000	0.000	0.000	0.000	0.000
2.5.1.78|g__Anaeromassilibacillus.s__Anaeromassilibacillus_sp_An250	-	2.62E-01	0.000	0.000	0.000	0.000	0.000
2.5.1.78|g__Anaerostipes.s__Anaerostipes_caccae	-	7.01E-01	0.000	0.000	0.000	0.001	0.001
2.5.1.78|g__Anaerotignum.s__Anaerotignum_lactatifermentans	+	3.51E-02	0.000	0.000	0.000	0.000	0.002
2.5.1.78|g__Asaccharobacter.s__Asaccharobacter_celatus	+	1.25E-01	0.000	0.000	0.000	0.000	0.000
2.5.1.78|g__Bacillus.s__Bacillus_murimartini	+	7.63E-02	0.000	0.000	0.000	0.000	0.000
2.5.1.78|g__Bacillus.s__Bacillus_subtilis_group	-	9.96E-01	0.000	0.000	0.000	0.000	0.000
2.5.1.78|g__Bacteroides.s__Bacteroides_caccae	+	1.30E-01	0.000	0.000	0.000	0.005	0.007
2.5.1.78|g__Bacteroides.s__Bacteroides_cellulosilyticus	+	5.16E-02	0.000	0.000	0.000	0.006	0.009
2.5.1.78|g__Bacteroides.s__Bacteroides_clarus	+	3.82E-01	0.000	0.000	0.000	0.000	0.004
2.5.1.78|g__Bacteroides.s__Bacteroides_coprocola	+	2.76E-01	0.000	0.000	0.000	0.009	0.014
2.5.1.78|g__Bacteroides.s__Bacteroides_coprophilus	+	3.02E-01	0.000	0.000	0.000	0.001	0.001
2.5.1.78|g__Bacteroides.s__Bacteroides_eggerthii	+	4.56E-01	0.000	0.000	0.000	0.004	0.009
2.5.1.78|g__Bacteroides.s__Bacteroides_faecis	-	8.22E-01	0.000	0.000	0.000	0.000	0.000
2.5.1.78|g__Bacteroides.s__Bacteroides_finegoldii	+	1.60E-01	0.000	0.000	0.000	0.002	0.003
2.5.1.78|g__Bacteroides.s__Bacteroides_fluxus	-	2.62E-01	0.000	0.000	0.000	0.000	0.000
2.5.1.78|g__Bacteroides.s__Bacteroides_fragilis	+	9.53E-01	0.000	0.000	0.000	0.002	0.002
2.5.1.78|g__Bacteroides.s__Bacteroides_intestinalis	+	2.44E-01	0.000	0.000	0.000	0.001	0.002
2.5.1.78|g__Bacteroides.s__Bacteroides_massiliensis	+	2.32E-01	0.000	0.000	0.000	0.002	0.004
2.5.1.78|g__Bacteroides.s__Bacteroides_nordii	+	4.51E-01	0.000	0.000	0.000	0.000	0.001
2.5.1.78|g__Bacteroides.s__Bacteroides_oleiciplenus	+	3.85E-01	0.000	0.000	0.000	0.000	0.000
2.5.1.78|g__Bacteroides.s__Bacteroides_plebeius	+	2.85E-01	0.000	0.000	0.000	0.021	0.017
2.5.1.78|g__Bacteroides.s__Bacteroides_salyersiae	+	1.16E-01	0.000	0.000	0.000	0.000	0.001
2.5.1.78|g__Bacteroides.s__Bacteroides_stercoris	-	7.46E-01	0.000	0.000	0.000	0.031	0.024
2.5.1.78|g__Bacteroides.s__Bacteroides_xylanisolvens	-	3.56E-01	0.000	0.000	0.000	0.007	0.007
2.5.1.78|g__Barnesiella.s__Barnesiella_intestinihominis	+	1.23E-02	0.000	0.000	0.000	0.004	0.009
2.5.1.78|g__Barnesiella.s__Barnesiella_sp_An22	-	2.62E-01	0.000	0.000	0.000	0.000	0.000
2.5.1.78|g__Bifidobacterium.s__Bifidobacterium_longum	+	7.31E-01	0.000	0.000	0.000	0.000	0.000
2.5.1.78|g__Bilophila.s__Bilophila_wadsworthia	+	1.23E-01	0.000	0.000	0.000	0.002	0.003
2.5.1.78|g__Blautia.s__Blautia_hansenii	-	9.01E-01	0.000	0.000	0.000	0.000	0.001
2.5.1.78|g__Blautia.s__Blautia_sp_An249	+	7.63E-02	0.000	0.000	0.000	0.000	0.001
2.5.1.78|g__Butyricicoccus.s__Butyricicoccus_pullicaecorum	-	2.03E-01	0.000	0.000	0.000	0.000	0.000
2.5.1.78|g__Butyricimonas.s__Butyricimonas_virosa	+	3.59E-01	0.000	0.000	0.000	0.000	0.001
2.5.1.78|g__Butyrivibrio.s__Butyrivibrio_crossotus	+	3.85E-01	0.000	0.000	0.000	0.000	0.001
2.5.1.78|g__Campylobacter.s__Campylobacter_concisus	-	2.62E-01	0.000	0.000	0.000	0.000	0.000
2.5.1.78|g__Candidatus_Gastranaerophilales_unclassified.s__Candidatus_Ga	-	8.71E-01	0.000	0.000	0.000	0.000	0.000
2.5.1.78|g__Catenibacterium.s__Catenibacterium_mitsuokai	-	1.62E-02	0.000	0.000	0.000	0.002	0.000
2.5.1.78|g__Cellulosilyticum.s__Cellulosilyticum_lentocellum	+	2.14E-01	0.000	0.000	0.000	0.000	0.000
2.5.1.78|g__Cetobacterium.s__Cetobacterium_somerae	-	2.62E-01	0.000	0.000	0.000	0.000	0.000
2.5.1.78|g__Citrobacter.s__Citrobacter_braakii	+	2.14E-01	0.000	0.000	0.000	0.000	0.000
2.5.1.78|g__Citrobacter.s__Citrobacter_freundii	+	2.14E-01	0.000	0.000	0.000	0.000	0.000
2.5.1.78|g__Citrobacter.s__Citrobacter_koseri	+	3.85E-01	0.000	0.000	0.000	0.000	0.000
2.5.1.78|g__Citrobacter.s__Citrobacter_portucalensis	+	4.03E-01	0.000	0.000	0.000	0.000	0.000
2.5.1.78|g__Citrobacter.s__Citrobacter_werkmanii	+	3.85E-01	0.000	0.000	0.000	0.000	0.001
2.5.1.78|g__Citrobacter.s__Citrobacter_youngae	+	4.08E-01	0.000	0.000	0.000	0.000	0.001
2.5.1.78|g__Cloacibacillus.s__Cloacibacillus_porcorum	+	7.63E-02	0.000	0.000	0.000	0.000	0.001
2.5.1.78|g__Clostridiales_unclassified.s__Clostridiales_bacterium_1_7_47FA	+	3.85E-01	0.000	0.000	0.000	0.000	0.000
2.5.1.78|g__Clostridioides.s__Clostridioides_difficile	+	3.42E-01	0.000	0.000	0.000	0.005	0.006
2.5.1.78|g__Clostridium.s__Butyribacterium_methylotrophicum	+	2.72E-01	0.000	0.000	0.000	0.000	0.000
2.5.1.78|g__Clostridium.s__Clostridium_celatum	+	1.00E+00	0.000	0.000	0.000	0.000	0.000
2.5.1.78|g__Clostridium.s__Clostridium_disporicum	-	6.76E-01	0.000	0.000	0.000	0.002	0.002
2.5.1.78|g__Clostridium.s__Clostridium_perfringens	-	4.48E-01	0.000	0.000	0.000	0.001	0.000
2.5.1.78|g__Clostridium.s__Clostridium_sp_AF36_4	+	6.95E-01	0.000	0.000	0.000	0.001	0.001
2.5.1.78|g__Clostridium.s__Clostridium_sp_AM22_11AC	-	1.82E-02	0.000	0.000	0.000	0.004	0.002
2.5.1.78|g__Comamonas.s__Comamonas_kerstersii	-	2.62E-01	0.000	0.000	0.000	0.000	0.000
2.5.1.78|g__Coprobacillus.s__Coprobacillus_cateniformis	+	4.79E-02	0.000	0.000	0.000	0.000	0.001
2.5.1.78|g__Coprobacter.s__Coprobacter_fastidiosus	+	6.02E-01	0.000	0.000	0.000	0.001	0.001
2.5.1.78|g__Coprobacter.s__Coprobacter_secundus	+	1.93E-01	0.000	0.000	0.000	0.000	0.001
2.5.1.78|g__Coprococcus.s__Coprococcus_catus	+	2.47E-01	0.000	0.000	0.000	0.001	0.002
2.5.1.78|g__Coprococcus.s__Coprococcus_comes	-	3.14E-02	0.000	0.000	0.000	0.005	0.002
2.5.1.78|g__Coprococcus.s__Coprococcus_eutactus	-	1.46E-01	0.000	0.000	0.000	0.002	0.002
2.5.1.78|g__Desulfovibrio.s__Desulfovibrio_desulfuricans	+	4.24E-03	0.000	0.000	0.000	0.000	0.001
2.5.1.78|g__Desulfovibrio.s__Desulfovibrio_fairfieldensis	+	1.26E-01	0.000	0.000	0.000	0.000	0.000
2.5.1.78|g__Desulfovibrio.s__Desulfovibrio_piger	+	2.86E-01	0.000	0.000	0.000	0.000	0.000
2.5.1.78|g__Desulfovibrio.s__Desulfovibrio_sp_AM18_2	+	3.85E-01	0.000	0.000	0.000	0.000	0.000
2.5.1.78|g__Dialister.s__Dialister_invisus	-	1.91E-01	0.000	0.000	0.000	0.005	0.002
2.5.1.78|g__Dialister.s__Dialister_succinatiphilus	+	7.10E-01	0.000	0.000	0.000	0.000	0.001
2.5.1.78|g__Dorea.s__Dorea_sp_OM02_2LB	-	9.87E-01	0.000	0.000	0.000	0.000	0.000
2.5.1.78|g__Eggerthella.s__Eggerthella_lenta	+	2.65E-01	0.000	0.000	0.000	0.006	0.005

2.5.1.78|g__Eisenbergiella.s__Eisenbergiella_tayi	-	8.57E-01	0.000	0.000	0.000	0.000	0.000
2.5.1.78|g__Enterobacter.s__Enterobacter_bugandensis	+	7.31E-01	0.000	0.000	0.000	0.000	0.000
2.5.1.78|g__Enterobacter.s__Enterobacter_cloacae	-	4.86E-01	0.000	0.000	0.000	0.000	0.000
2.5.1.78|g__Enterobacter.s__Enterobacter_mori	+	1.26E-01	0.000	0.000	0.000	0.000	0.000
2.5.1.78|g__Enterococcus.s__Enterococcus_avium	+	4.14E-01	0.000	0.000	0.000	0.005	0.001
2.5.1.78|g__Enterococcus.s__Enterococcus_faecalis	-	2.62E-01	0.000	0.000	0.000	0.000	0.000
2.5.1.78|g__Enterococcus.s__Enterococcus_faecium	-	4.52E-01	0.000	0.000	0.000	0.005	0.000
2.5.1.78|g__Enterococcus.s__Enterococcus_gallinarum	+	1.26E-01	0.000	0.000	0.000	0.000	0.000
2.5.1.78|g__Enterococcus.s__Enterococcus_saccharolyticus	+	2.14E-01	0.000	0.000	0.000	0.000	0.000
2.5.1.78|g__Enterococcus.s__Enterococcus_thailandicus	+	3.85E-01	0.000	0.000	0.000	0.000	0.000
2.5.1.78|g__Erysipelatoclostridium.s__Clostridium_innocuum	-	2.95E-01	0.000	0.000	0.000	0.002	0.001
2.5.1.78|g__Erysipelatoclostridium.s__Clostridium_spiroforme	-	8.07E-01	0.000	0.000	0.000	0.004	0.002
2.5.1.78|g__Erysipelotrichaceae_unclassified.s__Erysipelotrichaceae_bacteri	+	1.69E-01	0.000	0.000	0.000	0.000	0.001
2.5.1.78|g__Escherichia.s__Escherichia_coli	-	9.93E-01	0.000	0.000	0.000	0.036	0.017
2.5.1.78|g__Escherichia.s__Escherichia_fergusonii	-	2.62E-01	0.000	0.000	0.000	0.000	0.000
2.5.1.78|g__Eubacterium.s__Eubacterium_callanderi	+	2.72E-01	0.000	0.000	0.000	0.000	0.000
2.5.1.78|g__Eubacterium.s__Eubacterium_ramulus	-	5.81E-01	0.000	0.000	0.000	0.004	0.004
2.5.1.78|g__Eubacterium.s__Eubacterium_sp_AF17_7	+	3.12E-02	0.000	0.000	0.000	0.001	0.003
2.5.1.78|g__Eubacterium.s__Eubacterium_sp_AM18_10LB_B	-	6.58E-01	0.000	0.000	0.000	0.001	0.001
2.5.1.78|g__Faecalicatena.s__Faecalicatena_contorta	+	7.21E-01	0.000	0.000	0.000	0.000	0.000
2.5.1.78|g__Faecalitalea.s__Faecalitalea_cylindroides	+	2.88E-02	0.000	0.000	0.000	0.000	0.001
2.5.1.78|g__Firmicutes_unclassified.s__Firmicutes_bacterium_AM10_47	+	1.84E-01	0.000	0.000	0.000	0.002	0.002
2.5.1.78|g__Flavonifractor.s__Flavonifractor_plautii	+	1.13E-01	0.000	0.000	0.000	0.000	0.001
2.5.1.78|g__Flavonifractor.s__Flavonifractor_sp_An10	+	1.10E-01	0.000	0.000	0.000	0.000	0.002
2.5.1.78|g__Flavonifractor.s__Flavonifractor_sp_An82	+	3.85E-01	0.000	0.000	0.000	0.000	0.000
2.5.1.78|g__Fusobacterium.s__Fusobacterium_mortiferum	-	4.32E-01	0.000	0.000	0.000	0.000	0.000
2.5.1.78|g__Fusobacterium.s__Fusobacterium_ulcerans	+	6.27E-01	0.000	0.000	0.000	0.001	0.000
2.5.1.78|g__Fusobacterium.s__Fusobacterium_varium	-	2.62E-01	0.000	0.000	0.000	0.000	0.000
2.5.1.78|g__Geobacillus.s__Bacillus_caldolyticus	+	3.85E-01	0.000	0.000	0.000	0.000	0.000
2.5.1.78|g__Geobacillus.s__Geobacillus_kaustophilus	+	3.85E-01	0.000	0.000	0.000	0.000	0.000
2.5.1.78|g__Haemophilus.s__Haemophilus_influenzae	-	2.62E-01	0.000	0.000	0.000	0.000	0.000
2.5.1.78|g__Haemophilus.s__Haemophilus_parainfluenzae	-	5.10E-02	0.000	0.000	0.000	0.002	0.000
2.5.1.78|g__Hafnia.s__Hafnia_alvei	+	3.85E-01	0.000	0.000	0.000	0.000	0.000
2.5.1.78|g__Holdemanella.s__Holdemanella_biformis	-	4.32E-01	0.000	0.000	0.000	0.000	0.003
2.5.1.78|g__Hungatella.s__Hungatella_hathewayi	+	5.90E-01	0.000	0.000	0.000	0.000	0.001
2.5.1.78|g__Intestinibacter.s__Intestinibacter_bartlettii	-	3.47E-02	0.000	0.000	0.000	0.003	0.002
2.5.1.78|g__Klebsiella.s__Klebsiella_aerogenes	+	5.34E-01	0.000	0.000	0.000	0.001	0.000
2.5.1.78|g__Klebsiella.s__Klebsiella_grimontii	-	1.09E-01	0.000	0.000	0.000	0.001	0.000
2.5.1.78|g__Klebsiella.s__Klebsiella_michiganensis	-	2.60E-01	0.000	0.000	0.000	0.000	0.000
2.5.1.78|g__Klebsiella.s__Klebsiella_oxytoca	-	8.36E-02	0.000	0.000	0.000	0.002	0.000
2.5.1.78|g__Klebsiella.s__Klebsiella_pneumoniae	-	8.76E-01	0.000	0.000	0.000	0.004	0.010
2.5.1.78|g__Klebsiella.s__Klebsiella_variicola	-	2.26E-02	0.000	0.000	0.000	0.001	0.000
2.5.1.78|g__Lachnoclostridium.s__Clostridium_aldenense	-	4.58E-01	0.000	0.000	0.000	0.000	0.000
2.5.1.78|g__Lachnoclostridium.s__Clostridium_bolteae	-	2.81E-01	0.000	0.000	0.000	0.001	0.001
2.5.1.78|g__Lachnoclostridium.s__Clostridium_citroniae	-	4.32E-01	0.000	0.000	0.000	0.000	0.000
2.5.1.78|g__Lachnoclostridium.s__Clostridium_clostridioforme	+	4.59E-01	0.000	0.000	0.000	0.001	0.001
2.5.1.78|g__Lachnoclostridium.s__Clostridium_symbiosum	+	8.87E-01	0.000	0.000	0.000	0.000	0.000
2.5.1.78|g__Lachnoclostridium.s__Lachnoclostridium_sp_An138	+	7.31E-01	0.000	0.000	0.000	0.000	0.000
2.5.1.78|g__Lachnospira.s__Lachnospira_pectinoschiza	-	1.20E-01	0.000	0.000	0.000	0.003	0.002
2.5.1.78|g__Lachnospiraceae_unclassified.s__Lachnospiraceae_bacterium_(	+	4.67E-02	0.000	0.000	0.000	0.000	0.000
2.5.1.78|g__Lactobacillus.s__Lactobacillus_amylovorus	-	8.57E-01	0.000	0.000	0.000	0.000	0.000
2.5.1.78|g__Lactobacillus.s__Lactobacillus_antri	+	1.26E-01	0.000	0.000	0.000	0.000	0.001
2.5.1.78|g__Lactobacillus.s__Lactobacillus_crispatus	+	1.07E-01	0.000	0.000	0.000	0.000	0.001
2.5.1.78|g__Lactobacillus.s__Lactobacillus_delbrueckii	-	4.16E-01	0.000	0.000	0.000	0.000	0.000
2.5.1.78|g__Lactobacillus.s__Lactobacillus_fermentum	+	8.98E-02	0.000	0.000	0.000	0.000	0.003
2.5.1.78|g__Lactobacillus.s__Lactobacillus_gasseri	+	2.49E-01	0.000	0.000	0.000	0.000	0.006
2.5.1.78|g__Lactobacillus.s__Lactobacillus_kimbladii	+	3.85E-01	0.000	0.000	0.000	0.000	0.000
2.5.1.78|g__Lactobacillus.s__Lactobacillus_kullabergensis	+	3.85E-01	0.000	0.000	0.000	0.000	0.000
2.5.1.78|g__Lactobacillus.s__Lactobacillus_melliventris	+	3.85E-01	0.000	0.000	0.000	0.000	0.000
2.5.1.78|g__Lactobacillus.s__Lactobacillus_oris	+	4.14E-01	0.000	0.000	0.000	0.002	0.002
2.5.1.78|g__Lactobacillus.s__Lactobacillus_paragasseri	+	1.18E-01	0.000	0.000	0.000	0.000	0.003
2.5.1.78|g__Lactobacillus.s__Lactobacillus_plantarum	+	3.85E-01	0.000	0.000	0.000	0.000	0.000
2.5.1.78|g__Lactobacillus.s__Lactobacillus_rogosae	-	3.94E-01	0.000	0.000	0.000	0.003	0.003
2.5.1.78|g__Lactococcus.s__Lactococcus_lactis	-	9.45E-02	0.000	0.000	0.000	0.001	0.000
2.5.1.78|g__Lelliottia.s__Lelliottia_amnigena	-	2.62E-01	0.000	0.000	0.000	0.000	0.000
2.5.1.78|g__Lelliottia.s__Lelliottia_nimipressuralis	-	4.88E-02	0.000	0.000	0.000	0.000	0.000
2.5.1.78|g__Leuconostoc.s__Leuconostoc_garlicum	+	2.14E-01	0.000	0.000	0.000	0.000	0.000
2.5.1.78|g__Leuconostoc.s__Leuconostoc_lactis	+	2.14E-01	0.000	0.000	0.000	0.000	0.000
2.5.1.78|g__Listeria.s__Listeria_monocytogenes	+	3.85E-01	0.000	0.000	0.000	0.000	0.000
2.5.1.78|g__Megamonas.s__Megamonas_funiformis	+	6.73E-01	0.000	0.000	0.000	0.008	0.005
2.5.1.78|g__Megamonas.s__Megamonas_hypermegale	+	8.80E-01	0.000	0.000	0.000	0.004	0.002
2.5.1.78|g__Megamonas.s__Megamonas_rupellensis	-	5.76E-01	0.000	0.000	0.000	0.003	0.002

2.5.1.78|g__Megasphaera.s__Megasphaera_elsdenii	-	8.71E-01	0.000	0.000	0.000	0.000	0.000
2.5.1.78|g__Megasphaera.s__Megasphaera_hexanoica	+	3.85E-01	0.000	0.000	0.000	0.000	0.000
2.5.1.78|g__Megasphaera.s__Megasphaera_micronuciformis	-	1.09E-01	0.000	0.000	0.000	0.000	0.000
2.5.1.78|g__Megasphaera.s__Megasphaera_sp_DISK_18	-	1.26E-01	0.000	0.000	0.000	0.004	0.001
2.5.1.78|g__Megasphaera.s__Megasphaera_sp_MJR8396C	-	7.60E-01	0.000	0.000	0.000	0.001	0.001
2.5.1.78|g__Megasphaera.s__Megasphaera_stantonii	-	8.12E-01	0.000	0.000	0.000	0.000	0.000
2.5.1.78|g__Mesosutterella.s__Mesosutterella_multiformis	-	1.31E-01	0.000	0.000	0.000	0.002	0.000
2.5.1.78|g__Metakosakonia.s__Kluyvera_intestini	+	3.85E-01	0.000	0.000	0.000	0.000	0.000
2.5.1.78|g__Methanobrevibacter.s__Methanobrevibacter_smithii	+	3.85E-02	0.000	0.000	0.000	0.003	0.010
2.5.1.78|g__Methanosphaera.s__Methanosphaera_stadtmanae	-	7.76E-01	0.000	0.000	0.000	0.000	0.001
2.5.1.78|g__Mitsuokella.s__Mitsuokella_jalaludinii	+	3.85E-01	0.000	0.000	0.000	0.000	0.000
2.5.1.78|g__Mitsuokella.s__Mitsuokella_multacida	+	3.85E-01	0.000	0.000	0.000	0.000	0.000
2.5.1.78|g__Mogibacterium.s__Mogibacterium_diversum	+	1.84E-01	0.000	0.000	0.000	0.000	0.001
2.5.1.78|g__Mogibacterium.s__Mogibacterium_timidum	+	3.85E-01	0.000	0.000	0.000	0.000	0.000
2.5.1.78|g__Odoribacter.s__Odoribacter_laneus	+	1.05E-01	0.000	0.000	0.000	0.000	0.001
2.5.1.78|g__Odoribacter.s__Odoribacter_splanchnicus	+	4.83E-02	0.000	0.000	0.000	0.002	0.003
2.5.1.78|g__Pantoea.s__Pantoea_sesami	-	4.67E-01	0.000	0.000	0.000	0.001	0.000
2.5.1.78|g__Parabacteroides.s__Parabacteroides_goldsteinii	+	8.17E-02	0.000	0.000	0.000	0.000	0.001
2.5.1.78|g__Parabacteroides.s__Parabacteroides_gordonii	+	3.85E-01	0.000	0.000	0.000	0.000	0.000
2.5.1.78|g__Parabacteroides.s__Parabacteroides_johnsonii	-	8.26E-01	0.000	0.000	0.000	0.001	0.001
2.5.1.78|g__Paraprevotella.s__Paraprevotella_clara	+	1.49E-01	0.000	0.000	0.000	0.001	0.002
2.5.1.78|g__Paraprevotella.s__Paraprevotella_xylaniphila	+	4.39E-01	0.000	0.000	0.000	0.000	0.001
2.5.1.78|g__Parasutterella.s__Parasutterella_excrementihominis	-	2.94E-01	0.000	0.000	0.000	0.001	0.001
2.5.1.78|g__Pediococcus.s__Pediococcus_acidilactici	+	4.61E-01	0.000	0.000	0.000	0.000	0.000
2.5.1.78|g__Peptostreptococcaceae_unclassified.s__Clostridium_hiranonis	-	2.62E-01	0.000	0.000	0.000	0.000	0.000
2.5.1.78|g__Phascolarctobacterium.s__Phascolarctobacterium_faecium	+	3.50E-01	0.000	0.000	0.000	0.005	0.007
2.5.1.78|g__Phascolarctobacterium.s__Phascolarctobacterium_succinatutens	-	2.87E-01	0.000	0.000	0.000	0.006	0.004
2.5.1.78|g__Prevotella.s__Prevotella_buccae	-	2.62E-01	0.000	0.000	0.000	0.001	0.000
2.5.1.78|g__Prevotella.s__Prevotella_copri	-	1.59E-01	0.000	0.000	0.000	0.004	0.002
2.5.1.78|g__Prevotella.s__Prevotella_sp_109	-	1.01E-01	0.000	0.000	0.000	0.002	0.001
2.5.1.78|g__Prevotella.s__Prevotella_sp_885	-	4.48E-02	0.000	0.000	0.000	0.005	0.000
2.5.1.78|g__Prevotella.s__Prevotella_sp_AM42_24	-	1.99E-01	0.000	0.000	0.000	0.003	0.000
2.5.1.78|g__Prevotella.s__Prevotella_stercorea	-	7.68E-01	0.000	0.000	0.000	0.002	0.005
2.5.1.78|g__Proteus.s__Proteus_vulgaris	+	3.85E-01	0.000	0.000	0.000	0.000	0.000
2.5.1.78|g__Pseudoflavonifractor.s__Pseudoflavonifractor_sp_An184	+	1.75E-01	0.000	0.000	0.000	0.000	0.001
2.5.1.78|g__Pseudoramibacter.s__Pseudoramibacter_alactolyticus	-	2.62E-01	0.000	0.000	0.000	0.000	0.000
2.5.1.78|g__Pyramidobacter.s__Pyramidobacter_piscolens	+	3.85E-01	0.000	0.000	0.000	0.000	0.000
2.5.1.78|g__Raoultella.s__Raoultella_ornithinolytica	-	9.89E-02	0.000	0.000	0.000	0.005	0.000
2.5.1.78|g__Raoultella.s__Raoultella_planticola	-	4.48E-01	0.000	0.000	0.000	0.001	0.000
2.5.1.78|g__Rikenella.s__Rikenella_microfusus	+	1.26E-01	0.000	0.000	0.000	0.000	0.001
2.5.1.78|g__Roseburia.s__Roseburia_faecis	-	2.32E-03	0.000	0.000	0.000	0.016	0.004
2.5.1.78|g__Roseburia.s__Roseburia_intestinalis	-	6.75E-03	0.000	0.000	0.000	0.014	0.002
2.5.1.78|g__Roseburia.s__Roseburia_inulinivorans	-	3.76E-01	0.000	0.000	0.000	0.003	0.001
2.5.1.78|g__Rothia.s__Rothia_dentocariosa	+	3.85E-01	0.000	0.000	0.000	0.000	0.000
2.5.1.78|g__Rothia.s__Rothia_mucilaginosa	+	3.85E-01	0.000	0.000	0.000	0.000	0.000
2.5.1.78|g__Ruminococcaceae_unclassified.s__Ruminococcaceae_bacteriun	+	6.05E-01	0.000	0.000	0.000	0.000	0.001
2.5.1.78|g__Ruminococcaceae_unclassified.s__Ruminococcaceae_bacteriun	+	7.10E-01	0.000	0.000	0.000	0.000	0.000
2.5.1.78|g__Ruminococcus.s__Ruminococcus_callidus	-	6.70E-02	0.000	0.000	0.000	0.005	0.003
2.5.1.78|g__Ruminococcus.s__Ruminococcus_sp_AF31_8BH	+	1.56E-01	0.000	0.000	0.000	0.001	0.002
2.5.1.78|g__Sanguibacteroides.s__Sanguibacteroides_justesenii	+	3.85E-01	0.000	0.000	0.000	0.000	0.000
2.5.1.78|g__Senegalimassilia.s__Senegalimassilia_anaerobia	+	2.53E-01	0.000	0.000	0.000	0.000	0.001
2.5.1.78|g__Slackia.s__Slackia_isoflavoniconvertens	-	9.59E-01	0.000	0.000	0.000	0.001	0.001
2.5.1.78|g__Staphylococcus.s__Staphylococcus_aureus	+	7.63E-02	0.000	0.000	0.000	0.000	0.000
2.5.1.78|g__Streptococcus.s__Streptococcus_equinus	-	1.09E-01	0.000	0.000	0.000	0.000	0.000
2.5.1.78|g__Streptococcus.s__Streptococcus_gallolyticus	+	2.14E-01	0.000	0.000	0.000	0.000	0.000
2.5.1.78|g__Streptococcus.s__Streptococcus_infantarius	-	2.62E-01	0.000	0.000	0.000	0.000	0.000
2.5.1.78|g__Streptococcus.s__Streptococcus_lutetiensis	-	1.09E-01	0.000	0.000	0.000	0.006	0.000
2.5.1.78|g__Streptococcus.s__Streptococcus_macedonicus	-	7.68E-01	0.000	0.000	0.000	0.001	0.001
2.5.1.78|g__Streptococcus.s__Streptococcus_oralis	-	2.62E-01	0.000	0.000	0.000	0.000	0.000
2.5.1.78|g__Streptococcus.s__Streptococcus_pasteurianus	-	7.83E-01	0.000	0.000	0.000	0.001	0.002
2.5.1.78|g__Streptococcus.s__Streptococcus_pneumoniae	+	4.19E-01	0.000	0.000	0.000	0.000	0.000
2.5.1.78|g__Succinatimonas.s__Succinatimonas_hippei	+	2.14E-01	0.000	0.000	0.000	0.000	0.000
2.5.1.78|g__Sutterella.s__Sutterella_parvirubra	+	3.85E-01	0.000	0.000	0.000	0.000	0.000
2.5.1.78|g__Sutterella.s__Sutterella_wadsworthensis	-	7.39E-01	0.000	0.000	0.000	0.005	0.006
2.5.1.78|g__Terrisporobacter.s__Terrisporobacter_othiniensis	+	3.85E-01	0.000	0.000	0.000	0.000	0.000
2.5.1.78|g__Veillonella.s__Veillonella_atypica	-	3.50E-04	0.000	0.000	0.000	0.002	0.000
2.5.1.78|g__Veillonella.s__Veillonella_denticariosi	-	8.71E-01	0.000	0.000	0.000	0.000	0.000
2.5.1.78|g__Veillonella.s__Veillonella_dispar	-	1.04E-03	0.000	0.000	0.000	0.002	0.000
2.5.1.78|g__Veillonella.s__Veillonella_infantium	-	7.64E-03	0.000	0.000	0.000	0.002	0.000
2.5.1.78|g__Veillonella.s__Veillonella_parvula	-	4.93E-02	0.000	0.000	0.000	0.000	0.000
2.5.1.78|g__Veillonella.s__Veillonella_rogosae	-	4.85E-03	0.000	0.000	0.000	0.001	0.000
2.5.1.78|g__Victivallales_unclassified.s__Victivallales_bacterium_CCUG_4	+	4.61E-01	0.000	0.000	0.000	0.000	0.000

	2.5.1.78|g__Weissella.s__Weissella_cibaria	+	3.85E-01	0.000	0.000	0.000	0.000	0.000
	2.5.1.78|g__Weissella.s__Weissella_confusa	+	3.85E-01	0.000	0.000	0.000	0.000	0.000
	2.5.1.78|g__Yokenella.s__Yokenella_regensburgei	-	4.24E-01	0.000	0.000	0.000	0.000	0.000
	2.5.1.78|g__Bacteroides.s__Bacteroides_thetaiotaomicron	+	9.14E-02	0.382	0.504	0.122	0.007	0.012
	2.5.1.78|g__Bacteroides.s__Bacteroides_ovatus	+	2.44E-01	0.377	0.550	0.172	0.014	0.020
	2.5.1.78|g__Bacteroides.s__Bacteroides_dorei	+	5.31E-01	0.411	0.619	0.208	0.031	0.029
	2.5.1.78|g__Alistipes.s__Alistipes_finegoldii	+	6.60E-03	0.000	0.210	0.210	0.005	0.015
	2.5.1.78|g__Eubacterium.s__Eubacterium_eligens	+	1.14E-01	0.000	0.333	0.333	0.011	0.020
	2.5.1.78|g__Parabacteroides.s__Parabacteroides_merdae	+	4.77E-01	0.256	0.615	0.359	0.010	0.010
	2.5.1.78|g__Bacteroides.s__Bacteroides_uniformis	+	5.90E-01	4.161	4.624	0.463	0.050	0.063
	2.5.1.78|g__Parabacteroides.s__Parabacteroides_distasonis	+	2.94E-02	0.503	0.982	0.479	0.011	0.016
	2.5.1.78|g__Alistipes.s__Alistipes_shahii	+	2.75E-02	0.000	0.576	0.576	0.005	0.013
	2.5.1.78|unclassified	+	5.82E-01	11.616	12.773	1.156	0.147	0.172
	2.5.1.78|g__Alistipes.s__Alistipes_putredinis	+	9.84E-03	0.000	1.713	1.713	0.012	0.026
USA	2.5.1.78	-	1.12E-03	106.825	99.501	-7.324	1.000	1.000
	2.5.1.78|g__Faecalibacterium.s__Faecalibacterium_prausnitzii	-	2.29E-05	6.748	2.831	-3.917	0.071	0.053
	2.5.1.78|g__Blautia.s__Blautia_obeum	-	5.67E-08	4.032	2.483	-1.550	0.050	0.037
	2.5.1.78|g__Bacteroides.s__Bacteroides_vulgatus	-	1.33E-01	5.771	4.326	-1.446	0.071	0.066
	2.5.1.78|g__Blautia.s__Blautia_wexlerae	-	9.59E-10	2.423	1.247	-1.176	0.035	0.024
	2.5.1.78|g__Alistipes.s__Alistipes_putredinis	-	2.39E-01	3.124	2.427	-0.697	0.033	0.031
	2.5.1.78|g__Lachnospiraceae_unclassified.s__Eubacterium_rectale	-	1.06E-03	0.716	0.124	-0.592	0.022	0.016
	2.5.1.78|g__Dorea.s__Dorea_longicatena	-	2.48E-02	0.577	0.000	-0.577	0.016	0.013
	2.5.1.78|g__Anaerostipes.s__Anaerostipes_hadrus	-	2.80E-04	0.834	0.333	-0.502	0.015	0.012
	2.5.1.78|g__Blautia.s__Ruminococcus_torques	-	8.98E-04	0.523	0.047	-0.476	0.020	0.013
	2.5.1.78|g__Bacteroides.s__Bacteroides_uniformis	-	7.35E-01	4.509	4.045	-0.464	0.059	0.062
	2.5.1.78|g__Roseburia.s__Roseburia_intestinalis	-	3.71E-07	0.351	0.000	-0.351	0.014	0.007
	2.5.1.78|g__Roseburia.s__Roseburia_faecis	-	5.98E-04	0.229	0.000	-0.229	0.020	0.013
	2.5.1.78|g__Lachnoclostridium.s__Clostridium_clostridioforme	+	8.81E-01	0.063	0.052	-0.011	0.009	0.009
	2.5.1.78|g__Acidaminococcus.s__Acidaminococcus_fermentans	+	2.32E-01	0.000	0.000	0.000	0.000	0.000
	2.5.1.78|g__Acidaminococcus.s__Acidaminococcus_intestini	+	9.73E-04	0.000	0.000	0.000	0.004	0.009
	2.5.1.78|g__Acidipropionibacterium.s__Acidipropionibacterium_acidipropic	+	4.92E-01	0.000	0.000	0.000	0.000	0.000
	2.5.1.78|g__Acidovorax.s__Acidovorax_sp_SD340	+	4.92E-01	0.000	0.000	0.000	0.000	0.000
	2.5.1.78|g__Actinomyces.s__Actinomyces_naeslundii	+	1.43E-02	0.000	0.000	0.000	0.000	0.000
	2.5.1.78|g__Actinomyces.s__Actinomyces_oris	+	6.71E-03	0.000	0.000	0.000	0.000	0.000
	2.5.1.78|g__Actinomyces.s__Actinomyces_radicidentis	-	1.48E-01	0.000	0.000	0.000	0.000	0.000
	2.5.1.78|g__Actinomyces.s__Actinomyces_sp_oral_taxon_448	-	9.73E-01	0.000	0.000	0.000	0.000	0.000
	2.5.1.78|g__Actinomyces.s__Actinomyces_viscosus	+	1.24E-03	0.000	0.000	0.000	0.000	0.000
	2.5.1.78|g__Adlercreutzia.s__Adlercreutzia_equolifaciens	-	8.29E-01	0.000	0.000	0.000	0.001	0.001
	2.5.1.78|g__Aeromonas.s__Aeromonas_caviae	+	4.92E-01	0.000	0.000	0.000	0.000	0.000
	2.5.1.78|g__Aeromonas.s__Aeromonas_veronii	-	1.48E-01	0.000	0.000	0.000	0.000	0.000
	2.5.1.78|g__Agathobaculum.s__Agathobaculum_butyriciproducens	-	5.44E-03	0.000	0.000	0.000	0.004	0.003
	2.5.1.78|g__Akkermansia.s__Akkermansia_muciniphila	+	2.68E-01	0.000	0.000	0.000	0.024	0.040
	2.5.1.78|g__Alistipes.s__Alistipes_indistinctus	+	1.04E-04	0.000	0.000	0.000	0.001	0.002
	2.5.1.78|g__Alistipes.s__Alistipes_inops	+	6.03E-01	0.000	0.000	0.000	0.001	0.001
	2.5.1.78|g__Alistipes.s__Alistipes_sp_An66	-	1.48E-01	0.000	0.000	0.000	0.000	0.000
	2.5.1.78|g__Alistipes.s__Alistipes_timonensis	+	3.34E-01	0.000	0.000	0.000	0.000	0.000
	2.5.1.78|g__Allisonella.s__Allisonella_histaminiformans	-	1.58E-01	0.000	0.000	0.000	0.000	0.000
	2.5.1.78|g__Anaerococcus.s__Anaerococcus_hydrogenalis	+	4.92E-01	0.000	0.000	0.000	0.000	0.000
	2.5.1.78|g__Anaerococcus.s__Anaerococcus_nagyae	+	4.92E-01	0.000	0.000	0.000	0.000	0.000
	2.5.1.78|g__Anaerococcus.s__Anaerococcus_octavius	+	4.92E-01	0.000	0.000	0.000	0.000	0.000
	2.5.1.78|g__Anaerococcus.s__Anaerococcus_vaginalis	+	2.32E-01	0.000	0.000	0.000	0.000	0.000
	2.5.1.78|g__Anaeroglobus.s__Anaeroglobus_geminatus	+	8.80E-01	0.000	0.000	0.000	0.001	0.000
	2.5.1.78|g__Anaeromassilibacillus.s__Anaeromassilibacillus_sp_An250	-	6.42E-01	0.000	0.000	0.000	0.000	0.000
	2.5.1.78|g__Anaerostipes.s__Anaerostipes_caccae	+	7.51E-01	0.000	0.000	0.000	0.001	0.002
	2.5.1.78|g__Anaerostipes.s__Anaerostipes_sp_494a	-	5.92E-01	0.000	0.000	0.000	0.000	0.000
	2.5.1.78|g__Anaerotignum.s__Anaerotignum_lactatifermentans	-	2.59E-01	0.000	0.000	0.000	0.001	0.002
	2.5.1.78|g__Asaccharobacter.s__Asaccharobacter_celatus	-	6.97E-01	0.000	0.000	0.000	0.001	0.001
	2.5.1.78|g__Bacteroides.s__Bacteroides_clarus	-	8.06E-01	0.000	0.000	0.000	0.001	0.000
	2.5.1.78|g__Bacteroides.s__Bacteroides_coprocola	-	6.91E-02	0.000	0.000	0.000	0.004	0.002
	2.5.1.78|g__Bacteroides.s__Bacteroides_coprophilus	+	1.32E-01	0.000	0.000	0.000	0.000	0.000
	2.5.1.78|g__Bacteroides.s__Bacteroides_dorei	+	3.27E-01	0.000	0.000	0.000	0.013	0.016
	2.5.1.78|g__Bacteroides.s__Bacteroides_eggerthii	+	8.12E-01	0.000	0.000	0.000	0.008	0.008
	2.5.1.78|g__Bacteroides.s__Bacteroides_faecichinchillae	+	3.30E-01	0.000	0.000	0.000	0.000	0.000
	2.5.1.78|g__Bacteroides.s__Bacteroides_faecis	+	1.68E-01	0.000	0.000	0.000	0.002	0.002
	2.5.1.78|g__Bacteroides.s__Bacteroides_finegoldii	+	4.35E-01	0.000	0.000	0.000	0.001	0.002
	2.5.1.78|g__Bacteroides.s__Bacteroides_fluxus	+	3.30E-01	0.000	0.000	0.000	0.000	0.000
	2.5.1.78|g__Bacteroides.s__Bacteroides_fragilis	+	7.57E-01	0.000	0.000	0.000	0.003	0.002
	2.5.1.78|g__Bacteroides.s__Bacteroides_intestinalis	+	8.17E-02	0.000	0.000	0.000	0.001	0.002
	2.5.1.78|g__Bacteroides.s__Bacteroides_massiliensis	+	3.94E-01	0.000	0.000	0.000	0.011	0.010
	2.5.1.78|g__Bacteroides.s__Bacteroides_nordii	+	9.40E-01	0.000	0.000	0.000	0.000	0.001
	2.5.1.78|g__Bacteroides.s__Bacteroides_plebeius	-	3.20E-01	0.000	0.000	0.000	0.013	0.004
	2.5.1.78|g__Bacteroides.s__Bacteroides_salyersiae	+	5.74E-02	0.000	0.000	0.000	0.001	0.002

2.5.1.78|g__Bacteroides.s__Bacteroides_sartorii	-	4.06E-02	0.000	0.000	0.000	0.000	0.000
2.5.1.78|g__Bacteroides.s__Bacteroides_sp_OM08_11	+	4.92E-01	0.000	0.000	0.000	0.000	0.000
2.5.1.78|g__Bacteroides.s__Bacteroides_stercorirosoris	+	5.55E-01	0.000	0.000	0.000	0.000	0.000
2.5.1.78|g__Bacteroides.s__Bacteroides_xylanisolvens	+	5.15E-01	0.000	0.000	0.000	0.005	0.004
2.5.1.78|g__Barnesiella.s__Barnesiella_intestinihominis	+	1.22E-01	0.000	0.000	0.000	0.008	0.009
2.5.1.78|g__Bifidobacterium.s__Bifidobacterium_longum	+	1.67E-01	0.000	0.000	0.000	0.000	0.000
2.5.1.78|g__Blautia.s__Blautia_hansenii	-	4.87E-01	0.000	0.000	0.000	0.002	0.001
2.5.1.78|g__Blautia.s__Blautia_sp_An249	+	3.04E-01	0.000	0.000	0.000	0.000	0.000
2.5.1.78|g__Brevibacterium.s__Brevibacterium_ravenspurgense	+	4.92E-01	0.000	0.000	0.000	0.000	0.000
2.5.1.78|g__Butyricicoccus.s__Butyricicoccus_porcorum	+	4.92E-01	0.000	0.000	0.000	0.000	0.000
2.5.1.78|g__Butyricicoccus.s__Butyricicoccus_pullicaecorum	-	9.87E-03	0.000	0.000	0.000	0.000	0.000
2.5.1.78|g__Butyricimonas.s__Butyricimonas_virosa	+	1.45E-01	0.000	0.000	0.000	0.000	0.000
2.5.1.78|g__Butyrivibrio.s__Butyrivibrio_crossotus	-	4.70E-01	0.000	0.000	0.000	0.002	0.001
2.5.1.78|g__Campylobacter.s__Campylobacter_coli	+	4.92E-01	0.000	0.000	0.000	0.000	0.000
2.5.1.78|g__Campylobacter.s__Campylobacter_concisus	+	4.92E-01	0.000	0.000	0.000	0.000	0.000
2.5.1.78|g__Campylobacter.s__Campylobacter_gracilis	-	9.32E-01	0.000	0.000	0.000	0.000	0.000
2.5.1.78|g__Campylobacter.s__Campylobacter_hominis	+	4.92E-01	0.000	0.000	0.000	0.000	0.000
2.5.1.78|g__Campylobacter.s__Campylobacter_upsaliensis	+	4.92E-01	0.000	0.000	0.000	0.000	0.000
2.5.1.78|g__Campylobacter.s__Campylobacter_ureolyticus	+	4.92E-01	0.000	0.000	0.000	0.000	0.000
2.5.1.78|g__Candida.s__Candida_albicans	-	5.92E-01	0.000	0.000	0.000	0.000	0.000
2.5.1.78|g__Candidatus_Gastranaerophilales_unclassified.s__Candidatus_Ga	+	9.00E-02	0.000	0.000	0.000	0.000	0.000
2.5.1.78|g__Candidatus_Methanomethylophilus.s__Candidatus_Methanome	-	9.73E-01	0.000	0.000	0.000	0.000	0.000
2.5.1.78|g__Catenibacterium.s__Catenibacterium_mitsuokai	+	3.73E-01	0.000	0.000	0.000	0.001	0.002
2.5.1.78|g__Cetobacterium.s__Cetobacterium_somerae	+	3.30E-01	0.000	0.000	0.000	0.000	0.000
2.5.1.78|g__Citrobacter.s__Citrobacter_amalonaticus	+	3.30E-01	0.000	0.000	0.000	0.000	0.000
2.5.1.78|g__Citrobacter.s__Citrobacter_braakii	+	3.16E-01	0.000	0.000	0.000	0.000	0.000
2.5.1.78|g__Citrobacter.s__Citrobacter_farmeri	+	1.67E-01	0.000	0.000	0.000	0.000	0.000
2.5.1.78|g__Citrobacter.s__Citrobacter_freundii	+	7.30E-01	0.000	0.000	0.000	0.000	0.000
2.5.1.78|g__Citrobacter.s__Citrobacter_koseri	+	7.55E-01	0.000	0.000	0.000	0.000	0.000
2.5.1.78|g__Citrobacter.s__Citrobacter_portucalensis	+	4.97E-01	0.000	0.000	0.000	0.001	0.000
2.5.1.78|g__Citrobacter.s__Citrobacter_werkmanii	-	1.48E-01	0.000	0.000	0.000	0.000	0.000
2.5.1.78|g__Citrobacter.s__Citrobacter_youngae	+	7.65E-01	0.000	0.000	0.000	0.000	0.000
2.5.1.78|g__Cloacibacillus.s__Cloacibacillus_porcorum	+	6.86E-02	0.000	0.000	0.000	0.000	0.001
2.5.1.78|g__Clostridiales_Family_XIII_Incertae_Sedis_unclassified.s__Euba	+	3.30E-01	0.000	0.000	0.000	0.000	0.000
2.5.1.78|g__Clostridiales_Family_XIII_Incertae_Sedis_unclassified.s__Euba	+	4.92E-01	0.000	0.000	0.000	0.000	0.000
2.5.1.78|g__Clostridiales_unclassified.s__Clostridiales_bacterium_1_7_47F/	-	9.05E-01	0.000	0.000	0.000	0.000	0.000
2.5.1.78|g__Clostridioides.s__Clostridioides_difficile	+	3.39E-03	0.000	0.000	0.000	0.004	0.004
2.5.1.78|g__Clostridium.s__Butyribacterium_methylotrophicum	+	2.67E-04	0.000	0.000	0.000	0.000	0.001
2.5.1.78|g__Clostridium.s__Clostridium_butyricum	-	1.48E-01	0.000	0.000	0.000	0.000	0.000
2.5.1.78|g__Clostridium.s__Clostridium_cadaveris	-	5.95E-01	0.000	0.000	0.000	0.000	0.000
2.5.1.78|g__Clostridium.s__Clostridium_celatum	+	4.92E-01	0.000	0.000	0.000	0.000	0.000
2.5.1.78|g__Clostridium.s__Clostridium_disporicum	+	2.66E-02	0.000	0.000	0.000	0.000	0.001
2.5.1.78|g__Clostridium.s__Clostridium_neonatale	+	4.92E-01	0.000	0.000	0.000	0.000	0.000
2.5.1.78|g__Clostridium.s__Clostridium_perfringens	-	3.53E-01	0.000	0.000	0.000	0.000	0.000
2.5.1.78|g__Clostridium.s__Clostridium_sp_AF36_4	-	1.82E-01	0.000	0.000	0.000	0.004	0.003
2.5.1.78|g__Clostridium.s__Clostridium_sp_AM22_11AC	-	2.93E-02	0.000	0.000	0.000	0.002	0.002
2.5.1.78|g__Clostridium.s__Clostridium_sp_MSTE9	+	4.92E-01	0.000	0.000	0.000	0.000	0.000
2.5.1.78|g__Clostridium.s__Clostridium_sp_chh4_2	-	4.06E-02	0.000	0.000	0.000	0.000	0.000
2.5.1.78|g__Clostridium.s__Clostridium_ventriculi	-	9.67E-01	0.000	0.000	0.000	0.000	0.000
2.5.1.78|g__Comamonas.s__Comamonas_kerstersii	+	2.32E-01	0.000	0.000	0.000	0.000	0.000
2.5.1.78|g__Comamonas.s__Comamonas_thiooxydans	+	4.92E-01	0.000	0.000	0.000	0.000	0.000
2.5.1.78|g__Coprobacillus.s__Coprobacillus_cateniformis	+	1.12E-05	0.000	0.000	0.000	0.000	0.001
2.5.1.78|g__Coprobacter.s__Coprobacter_fastidiosus	-	1.13E-01	0.000	0.000	0.000	0.001	0.000
2.5.1.78|g__Coprobacter.s__Coprobacter_secundus	-	9.55E-01	0.000	0.000	0.000	0.000	0.000
2.5.1.78|g__Coprococcus.s__Coprococcus_catus	+	4.37E-01	0.000	0.000	0.000	0.001	0.002
2.5.1.78|g__Coprococcus.s__Coprococcus_comes	-	7.54E-01	0.000	0.000	0.000	0.006	0.006
2.5.1.78|g__Coprococcus.s__Coprococcus_eutactus	-	5.18E-01	0.000	0.000	0.000	0.003	0.002
2.5.1.78|g__Corynebacterium.s__Corynebacterium_amycolatum	+	3.30E-01	0.000	0.000	0.000	0.000	0.000
2.5.1.78|g__Corynebacterium.s__Corynebacterium_coyleae	+	4.92E-01	0.000	0.000	0.000	0.000	0.000
2.5.1.78|g__Corynebacterium.s__Corynebacterium_frankenforstense	-	1.48E-01	0.000	0.000	0.000	0.000	0.000
2.5.1.78|g__Corynebacterium.s__Corynebacterium_freneyi	+	4.92E-01	0.000	0.000	0.000	0.000	0.000
2.5.1.78|g__Corynebacterium.s__Corynebacterium_imitans	+	4.92E-01	0.000	0.000	0.000	0.000	0.000
2.5.1.78|g__Corynebacterium.s__Corynebacterium_jeikeium	+	3.30E-01	0.000	0.000	0.000	0.000	0.000
2.5.1.78|g__Corynebacterium.s__Corynebacterium_kroppenstedtii	+	4.92E-01	0.000	0.000	0.000	0.000	0.000
2.5.1.78|g__Corynebacterium.s__Corynebacterium_minutissimum	+	4.92E-01	0.000	0.000	0.000	0.000	0.000
2.5.1.78|g__Corynebacterium.s__Corynebacterium_pseudogenitalium	+	4.92E-01	0.000	0.000	0.000	0.000	0.000
2.5.1.78|g__Corynebacterium.s__Corynebacterium_riegelii	+	4.92E-01	0.000	0.000	0.000	0.000	0.000
2.5.1.78|g__Corynebacterium.s__Corynebacterium_sp_HMSC08A12	+	4.92E-01	0.000	0.000	0.000	0.000	0.000
2.5.1.78|g__Corynebacterium.s__Corynebacterium_tuberculostearicum	+	4.92E-01	0.000	0.000	0.000	0.000	0.000
2.5.1.78|g__Corynebacterium.s__Corynebacterium_urealyticum	+	4.92E-01	0.000	0.000	0.000	0.000	0.000
2.5.1.78|g__Cutibacterium.s__Cutibacterium_avidum	+	4.92E-01	0.000	0.000	0.000	0.000	0.000
2.5.1.78|g__Delftia.s__Delftia_acidovorans	+	4.92E-01	0.000	0.000	0.000	0.000	0.000

2.5.1.78|g__Delftia.s__Delftia_lacustris	+	4.92E-01	0.000	0.000	0.000	0.000	0.000
2.5.1.78|g__Delftia.s__Delftia_tsuruhatensis	+	4.92E-01	0.000	0.000	0.000	0.000	0.000
2.5.1.78|g__Desulfovibrio.s__Desulfovibrio_desulfuricans	+	2.32E-01	0.000	0.000	0.000	0.000	0.000
2.5.1.78|g__Desulfovibrio.s__Desulfovibrio_fairfieldensis	+	5.59E-02	0.000	0.000	0.000	0.001	0.003
2.5.1.78|g__Desulfovibrio.s__Desulfovibrio_legallii	+	4.92E-01	0.000	0.000	0.000	0.000	0.000
2.5.1.78|g__Desulfovibrio.s__Desulfovibrio_piger	+	1.55E-01	0.000	0.000	0.000	0.000	0.001
2.5.1.78|g__Desulfovibrio.s__Desulfovibrio_sp_AM18_2	+	2.27E-01	0.000	0.000	0.000	0.000	0.000
2.5.1.78|g__Desulfovibrionaceae_unclassified.s__Desulfovibrionaceae_bact	+	4.22E-02	0.000	0.000	0.000	0.000	0.000
2.5.1.78|g__Dialister.s__Dialister_invisus	-	6.02E-03	0.000	0.000	0.000	0.006	0.004
2.5.1.78|g__Dialister.s__Dialister_pneumosintes	+	4.92E-01	0.000	0.000	0.000	0.000	0.000
2.5.1.78|g__Dialister.s__Dialister_succinatiphilus	+	4.92E-01	0.000	0.000	0.000	0.000	0.000
2.5.1.78|g__Dorea.s__Dorea_formicigenerans	-	6.01E-01	0.000	0.000	0.000	0.002	0.002
2.5.1.78|g__Dorea.s__Dorea_sp_OM02_2LB	+	1.39E-01	0.000	0.000	0.000	0.000	0.000
2.5.1.78|g__Dysgonomonas.s__Dysgonomonas_mossii	+	4.92E-01	0.000	0.000	0.000	0.000	0.000
2.5.1.78|g__Eggerthella.s__Eggerthella_lenta	+	8.51E-01	0.000	0.000	0.000	0.004	0.004
2.5.1.78|g__Eisenbergiella.s__Eisenbergiella_tayi	+	3.05E-03	0.000	0.000	0.000	0.000	0.002
2.5.1.78|g__Enterobacter.s__Enterobacter_bugandensis	+	3.08E-01	0.000	0.000	0.000	0.000	0.000
2.5.1.78|g__Enterobacter.s__Enterobacter_cloacae	+	2.41E-02	0.000	0.000	0.000	0.000	0.000
2.5.1.78|g__Enterobacter.s__Enterobacter_mori	+	4.92E-01	0.000	0.000	0.000	0.000	0.000
2.5.1.78|g__Enterococcus.s__Enterococcus_avium	+	2.59E-05	0.000	0.000	0.000	0.000	0.002
2.5.1.78|g__Enterococcus.s__Enterococcus_casseliflavus	+	3.30E-01	0.000	0.000	0.000	0.000	0.000
2.5.1.78|g__Enterococcus.s__Enterococcus_faecium	+	5.41E-01	0.000	0.000	0.000	0.000	0.000
2.5.1.78|g__Enterococcus.s__Enterococcus_gallinarum	+	9.17E-03	0.000	0.000	0.000	0.000	0.000
2.5.1.78|g__Enterococcus.s__Enterococcus_mundtii	+	4.92E-01	0.000	0.000	0.000	0.000	0.000
2.5.1.78|g__Enterococcus.s__Enterococcus_saccharolyticus	+	3.74E-02	0.000	0.000	0.000	0.000	0.000
2.5.1.78|g__Enterococcus.s__Enterococcus_thailandicus	+	4.92E-01	0.000	0.000	0.000	0.000	0.000
2.5.1.78|g__Erysipelatoclostridium.s__Clostridium_innocuum	+	1.06E-01	0.000	0.000	0.000	0.002	0.001
2.5.1.78|g__Erysipelatoclostridium.s__Clostridium_spiroforme	-	6.62E-01	0.000	0.000	0.000	0.001	0.000
2.5.1.78|g__Erysipelotrichaceae_unclassified.s__Erysipelotrichaceae_bacteri	+	6.39E-01	0.000	0.000	0.000	0.001	0.000
2.5.1.78|g__Escherichia.s__Escherichia_coli	+	9.66E-04	0.000	0.000	0.000	0.023	0.029
2.5.1.78|g__Escherichia.s__Escherichia_fergusonii	-	7.57E-01	0.000	0.000	0.000	0.000	0.000
2.5.1.78|g__Escherichia.s__Escherichia_marmotae	+	4.92E-01	0.000	0.000	0.000	0.000	0.000
2.5.1.78|g__Eubacterium.s__Eubacterium_callanderi	+	6.18E-04	0.000	0.000	0.000	0.000	0.001
2.5.1.78|g__Eubacterium.s__Eubacterium_eligens	-	2.48E-02	0.000	0.000	0.000	0.006	0.005
2.5.1.78|g__Eubacterium.s__Eubacterium_limosum	+	5.21E-01	0.000	0.000	0.000	0.000	0.000
2.5.1.78|g__Eubacterium.s__Eubacterium_maltosivorans	+	3.15E-01	0.000	0.000	0.000	0.000	0.000
2.5.1.78|g__Eubacterium.s__Eubacterium_ramulus	-	1.27E-02	0.000	0.000	0.000	0.003	0.002
2.5.1.78|g__Eubacterium.s__Eubacterium_sp_AF17_7	-	5.13E-01	0.000	0.000	0.000	0.000	0.000
2.5.1.78|g__Eubacterium.s__Eubacterium_sp_AM18_10LB_B	-	1.88E-01	0.000	0.000	0.000	0.000	0.000
2.5.1.78|g__Eubacterium.s__Eubacterium_sp_An11	+	6.61E-01	0.000	0.000	0.000	0.000	0.001
2.5.1.78|g__Faecalicatena.s__Faecalicatena_contorta	+	7.27E-01	0.000	0.000	0.000	0.000	0.000
2.5.1.78|g__Faecalitalea.s__Faecalitalea_cylindroides	+	9.15E-02	0.000	0.000	0.000	0.000	0.000
2.5.1.78|g__Firmicutes_unclassified.s__Firmicutes_bacterium_AM10_47	-	2.80E-01	0.000	0.000	0.000	0.000	0.000
2.5.1.78|g__Flavonifractor.s__Flavonifractor_plautii	+	3.54E-01	0.000	0.000	0.000	0.001	0.002
2.5.1.78|g__Flavonifractor.s__Flavonifractor_sp_An10	+	4.54E-02	0.000	0.000	0.000	0.000	0.001
2.5.1.78|g__Fusobacterium.s__Fusobacterium_mortiferum	-	9.57E-01	0.000	0.000	0.000	0.000	0.000
2.5.1.78|g__Fusobacterium.s__Fusobacterium_nucleatum	-	5.92E-01	0.000	0.000	0.000	0.000	0.000
2.5.1.78|g__Fusobacterium.s__Fusobacterium_ulcerans	-	1.34E-01	0.000	0.000	0.000	0.000	0.000
2.5.1.78|g__Fusobacterium.s__Fusobacterium_varium	-	5.95E-01	0.000	0.000	0.000	0.000	0.000
2.5.1.78|g__Haemophilus.s__Haemophilus_parainfluenzae	-	7.91E-01	0.000	0.000	0.000	0.000	0.000
2.5.1.78|g__Hafnia.s__Hafnia_alvei	-	1.48E-01	0.000	0.000	0.000	0.000	0.000
2.5.1.78|g__Hafnia.s__Hafnia_paralvei	-	9.67E-01	0.000	0.000	0.000	0.000	0.000
2.5.1.78|g__Holdemanella.s__Holdemanella_biformis	-	2.20E-02	0.000	0.000	0.000	0.000	0.000
2.5.1.78|g__Hungatella.s__Hungatella_hathewayi	+	3.62E-01	0.000	0.000	0.000	0.002	0.001
2.5.1.78|g__Intestinibacter.s__Intestinibacter_bartlettii	+	8.20E-01	0.000	0.000	0.000	0.001	0.001
2.5.1.78|g__Klebsiella.s__Klebsiella_aerogenes	+	2.74E-01	0.000	0.000	0.000	0.000	0.001
2.5.1.78|g__Klebsiella.s__Klebsiella_grimontii	-	9.73E-01	0.000	0.000	0.000	0.000	0.000
2.5.1.78|g__Klebsiella.s__Klebsiella_michiganensis	-	9.55E-01	0.000	0.000	0.000	0.000	0.000
2.5.1.78|g__Klebsiella.s__Klebsiella_oxytoca	-	7.91E-01	0.000	0.000	0.000	0.000	0.000
2.5.1.78|g__Klebsiella.s__Klebsiella_pneumoniae	+	4.06E-03	0.000	0.000	0.000	0.006	0.008
2.5.1.78|g__Klebsiella.s__Klebsiella_variicola	+	1.71E-02	0.000	0.000	0.000	0.000	0.002
2.5.1.78|g__Kluyvera.s__Kluyvera_ascorbata	-	4.50E-01	0.000	0.000	0.000	0.000	0.000
2.5.1.78|g__Kluyvera.s__Kluyvera_cryocrescens	+	4.92E-01	0.000	0.000	0.000	0.000	0.000
2.5.1.78|g__Kosakonia.s__Kosakonia_cowanii	+	4.92E-01	0.000	0.000	0.000	0.000	0.000
2.5.1.78|g__Lachnoclostridium.s__Clostridium_aldenense	-	9.66E-01	0.000	0.000	0.000	0.001	0.001
2.5.1.78|g__Lachnoclostridium.s__Clostridium_bolteae	+	9.46E-01	0.000	0.000	0.000	0.002	0.003
2.5.1.78|g__Lachnoclostridium.s__Clostridium_citroniae	-	9.88E-01	0.000	0.000	0.000	0.000	0.000
2.5.1.78|g__Lachnoclostridium.s__Clostridium_symbiosum	+	6.39E-01	0.000	0.000	0.000	0.001	0.002
2.5.1.78|g__Lachnoclostridium.s__Lachnoclostridium_sp_An138	+	3.00E-01	0.000	0.000	0.000	0.000	0.000
2.5.1.78|g__Lachnospira.s__Lachnospira_pectinoschiza	-	4.76E-01	0.000	0.000	0.000	0.001	0.002
2.5.1.78|g__Lachnospiraceae_unclassified.s__Lachnospiraceae_bacterium_(	+	9.13E-01	0.000	0.000	0.000	0.000	0.000
2.5.1.78|g__Lactobacillus.s__Lactobacillus_amylovorus	-	1.48E-01	0.000	0.000	0.000	0.000	0.000

2.5.1.78|g__Lactobacillus.s__Lactobacillus_brevis	+	4.92E-01	0.000	0.000	0.000	0.000	0.000
2.5.1.78|g__Lactobacillus.s__Lactobacillus_crispatus	+	3.04E-01	0.000	0.000	0.000	0.000	0.000
2.5.1.78|g__Lactobacillus.s__Lactobacillus_delbrueckii	+	5.53E-01	0.000	0.000	0.000	0.000	0.000
2.5.1.78|g__Lactobacillus.s__Lactobacillus_farciminis	+	3.30E-01	0.000	0.000	0.000	0.000	0.000
2.5.1.78|g__Lactobacillus.s__Lactobacillus_fermentum	+	3.89E-02	0.000	0.000	0.000	0.000	0.001
2.5.1.78|g__Lactobacillus.s__Lactobacillus_gasseri	+	1.68E-02	0.000	0.000	0.000	0.000	0.001
2.5.1.78|g__Lactobacillus.s__Lactobacillus_gastricus	+	4.92E-01	0.000	0.000	0.000	0.000	0.000
2.5.1.78|g__Lactobacillus.s__Lactobacillus_kullabergensis	+	4.92E-01	0.000	0.000	0.000	0.000	0.000
2.5.1.78|g__Lactobacillus.s__Lactobacillus_oris	+	1.92E-02	0.000	0.000	0.000	0.000	0.000
2.5.1.78|g__Lactobacillus.s__Lactobacillus_paragasseri	+	7.58E-02	0.000	0.000	0.000	0.000	0.000
2.5.1.78|g__Lactobacillus.s__Lactobacillus_paralimentarius	+	4.92E-01	0.000	0.000	0.000	0.000	0.000
2.5.1.78|g__Lactobacillus.s__Lactobacillus_pentosus	+	1.67E-01	0.000	0.000	0.000	0.000	0.000
2.5.1.78|g__Lactobacillus.s__Lactobacillus_plantarum	+	2.39E-01	0.000	0.000	0.000	0.000	0.000
2.5.1.78|g__Lactobacillus.s__Lactobacillus_reuteri	+	2.89E-02	0.000	0.000	0.000	0.000	0.002
2.5.1.78|g__Lactobacillus.s__Lactobacillus_rogosae	-	2.18E-01	0.000	0.000	0.000	0.001	0.002
2.5.1.78|g__Lactococcus.s__Lactococcus_lactis	-	5.37E-01	0.000	0.000	0.000	0.000	0.000
2.5.1.78|g__Leclercia.s__Leclercia_adecarboxylata	+	3.30E-01	0.000	0.000	0.000	0.000	0.000
2.5.1.78|g__Lelliottia.s__Lelliottia_amnigena	+	4.92E-01	0.000	0.000	0.000	0.000	0.000
2.5.1.78|g__Lelliottia.s__Lelliottia_nimipressuralis	+	9.90E-02	0.000	0.000	0.000	0.000	0.000
2.5.1.78|g__Leuconostoc.s__Leuconostoc_citreum	+	4.92E-01	0.000	0.000	0.000	0.000	0.000
2.5.1.78|g__Leuconostoc.s__Leuconostoc_lactis	+	4.92E-01	0.000	0.000	0.000	0.000	0.000
2.5.1.78|g__Leuconostoc.s__Leuconostoc_mesenteroides	+	3.30E-01	0.000	0.000	0.000	0.000	0.000
2.5.1.78|g__Listeria.s__Listeria_monocytogenes	+	4.92E-01	0.000	0.000	0.000	0.000	0.000
2.5.1.78|g__Megamonas.s__Megamonas_funiformis	+	7.25E-01	0.000	0.000	0.000	0.000	0.000
2.5.1.78|g__Megamonas.s__Megamonas_hypermegale	+	6.55E-01	0.000	0.000	0.000	0.001	0.001
2.5.1.78|g__Megamonas.s__Megamonas_rupellensis	+	3.30E-01	0.000	0.000	0.000	0.000	0.000
2.5.1.78|g__Megasphaera.s__Megasphaera_cerevisiae	+	4.92E-01	0.000	0.000	0.000	0.000	0.000
2.5.1.78|g__Megasphaera.s__Megasphaera_elsdenii	-	9.60E-01	0.000	0.000	0.000	0.000	0.000
2.5.1.78|g__Megasphaera.s__Megasphaera_hexanoica	+	4.92E-01	0.000	0.000	0.000	0.000	0.000
2.5.1.78|g__Megasphaera.s__Megasphaera_micronuciformis	+	3.30E-01	0.000	0.000	0.000	0.000	0.000
2.5.1.78|g__Megasphaera.s__Megasphaera_sp_DISK_18	+	2.23E-03	0.000	0.000	0.000	0.000	0.003
2.5.1.78|g__Megasphaera.s__Megasphaera_sp_MJR8396C	+	1.36E-01	0.000	0.000	0.000	0.001	0.002
2.5.1.78|g__Megasphaera.s__Megasphaera_stantonii	-	5.92E-01	0.000	0.000	0.000	0.000	0.000
2.5.1.78|g__Mesosutterella.s__Mesosutterella_multiformis	+	3.03E-02	0.000	0.000	0.000	0.000	0.001
2.5.1.78|g__Methanobrevibacter.s__Methanobrevibacter_smithii	+	5.12E-04	0.000	0.000	0.000	0.008	0.013
2.5.1.78|g__Methanomassiliicoccus.s__Candidatus_Methanomassiliicoccus_	+	1.52E-02	0.000	0.000	0.000	0.000	0.000
2.5.1.78|g__Methanosphaera.s__Methanosphaera_stadtmanae	+	3.59E-01	0.000	0.000	0.000	0.000	0.000
2.5.1.78|g__Mitsuokella.s__Mitsuokella_jalaludinii	-	5.92E-01	0.000	0.000	0.000	0.000	0.000
2.5.1.78|g__Mogibacterium.s__Mogibacterium_diversum	+	1.22E-01	0.000	0.000	0.000	0.000	0.000
2.5.1.78|g__Mogibacterium.s__Mogibacterium_timidum	+	4.92E-01	0.000	0.000	0.000	0.000	0.000
2.5.1.78|g__Morganella.s__Morganella_morganii	+	6.68E-02	0.000	0.000	0.000	0.000	0.000
2.5.1.78|g__Muribaculum.s__Muribaculum_intestinale	-	1.48E-01	0.000	0.000	0.000	0.000	0.000
2.5.1.78|g__Odoribacter.s__Odoribacter_laneus	+	2.60E-01	0.000	0.000	0.000	0.001	0.004
2.5.1.78|g__Odoribacter.s__Odoribacter_splanchnicus	-	8.59E-01	0.000	0.000	0.000	0.002	0.002
2.5.1.78|g__Oligella.s__Oligella_urethralis	+	4.92E-01	0.000	0.000	0.000	0.000	0.000
2.5.1.78|g__Oxalobacter.s__Oxalobacter_formigenes	-	2.67E-01	0.000	0.000	0.000	0.000	0.000
2.5.1.78|g__Pantoea.s__Pantoea_sesami	+	4.15E-01	0.000	0.000	0.000	0.000	0.000
2.5.1.78|g__Parabacteroides.s__Parabacteroides_chinchillae	+	4.92E-01	0.000	0.000	0.000	0.000	0.000
2.5.1.78|g__Parabacteroides.s__Parabacteroides_goldsteinii	+	2.85E-03	0.000	0.000	0.000	0.001	0.002
2.5.1.78|g__Parabacteroides.s__Parabacteroides_gordonii	+	7.67E-01	0.000	0.000	0.000	0.000	0.000
2.5.1.78|g__Parabacteroides.s__Parabacteroides_johnsonii	-	3.52E-01	0.000	0.000	0.000	0.001	0.001
2.5.1.78|g__Paraprevotella.s__Paraprevotella_clara	-	1.70E-01	0.000	0.000	0.000	0.003	0.003
2.5.1.78|g__Paraprevotella.s__Paraprevotella_xylaniphila	+	5.59E-01	0.000	0.000	0.000	0.000	0.000
2.5.1.78|g__Parasutterella.s__Parasutterella_excrementihominis	-	5.18E-01	0.000	0.000	0.000	0.003	0.003
2.5.1.78|g__Pediococcus.s__Pediococcus_acidilactici	+	2.81E-02	0.000	0.000	0.000	0.000	0.001
2.5.1.78|g__Pedobacter.s__Pedobacter_himalayensis	+	7.53E-01	0.000	0.000	0.000	0.000	0.000
2.5.1.78|g__Peptococcus.s__Peptococcus_niger	+	4.15E-01	0.000	0.000	0.000	0.000	0.000
2.5.1.78|g__Peptoniphilus.s__Peptoniphilus_coxii	+	4.92E-01	0.000	0.000	0.000	0.000	0.000
2.5.1.78|g__Peptoniphilus.s__Peptoniphilus_duerdenii	+	4.92E-01	0.000	0.000	0.000	0.000	0.000
2.5.1.78|g__Peptoniphilus.s__Peptoniphilus_lacrimalis	+	4.92E-01	0.000	0.000	0.000	0.000	0.000
2.5.1.78|g__Peptoniphilus.s__Peptoniphilus_sp_HMSC062D09	+	3.30E-01	0.000	0.000	0.000	0.000	0.000
2.5.1.78|g__Peptoniphilus.s__Peptoniphilus_sp_oral_taxon_375	+	4.92E-01	0.000	0.000	0.000	0.000	0.000
2.5.1.78|g__Peptostreptococcaceae_unclassified.s__Clostridium_hiranonis	-	5.95E-01	0.000	0.000	0.000	0.000	0.000
2.5.1.78|g__Peptostreptococcus.s__Peptostreptococcus_anaerobius	+	3.30E-01	0.000	0.000	0.000	0.000	0.000
2.5.1.78|g__Phascolarctobacterium.s__Phascolarctobacterium_succinatutens	+	2.42E-01	0.000	0.000	0.000	0.001	0.001
2.5.1.78|g__Pluralibacter.s__Pluralibacter_gergoviae	-	1.48E-01	0.000	0.000	0.000	0.000	0.000
2.5.1.78|g__Porphyromonas.s__Porphyromonas_asaccharolytica	+	5.65E-02	0.000	0.000	0.000	0.000	0.000
2.5.1.78|g__Porphyromonas.s__Porphyromonas_sp_HMSC065F10	+	1.67E-01	0.000	0.000	0.000	0.000	0.000
2.5.1.78|g__Porphyromonas.s__Porphyromonas_sp_HMSC077F02	-	9.70E-01	0.000	0.000	0.000	0.000	0.000
2.5.1.78|g__Porphyromonas.s__Porphyromonas_uenonis	-	5.95E-01	0.000	0.000	0.000	0.000	0.000
2.5.1.78|g__Prevotella.s__Prevotella_bivia	+	3.06E-01	0.000	0.000	0.000	0.000	0.000
2.5.1.78|g__Prevotella.s__Prevotella_buccae	-	9.73E-01	0.000	0.000	0.000	0.000	0.000

2.5.1.78|g__Prevotella.s__Prevotella_buccalis	+	9.00E-02	0.000	0.000	0.000	0.000	0.000
2.5.1.78|g__Prevotella.s__Prevotella_colorans	+	2.32E-01	0.000	0.000	0.000	0.000	0.000
2.5.1.78|g__Prevotella.s__Prevotella_copri	+	4.79E-01	0.000	0.000	0.000	0.001	0.001
2.5.1.78|g__Prevotella.s__Prevotella_corporis	+	9.00E-02	0.000	0.000	0.000	0.000	0.000
2.5.1.78|g__Prevotella.s__Prevotella_disiens	-	7.14E-01	0.000	0.000	0.000	0.000	0.000
2.5.1.78|g__Prevotella.s__Prevotella_sp_109	-	7.84E-01	0.000	0.000	0.000	0.002	0.001
2.5.1.78|g__Prevotella.s__Prevotella_sp_885	-	2.01E-01	0.000	0.000	0.000	0.000	0.000
2.5.1.78|g__Prevotella.s__Prevotella_sp_AM42_24	-	2.20E-02	0.000	0.000	0.000	0.001	0.000
2.5.1.78|g__Prevotella.s__Prevotella_stercorea	-	3.41E-01	0.000	0.000	0.000	0.003	0.001
2.5.1.78|g__Prevotella.s__Prevotella_timonensis	+	3.13E-01	0.000	0.000	0.000	0.000	0.000
2.5.1.78|g__Propionibacterium.s__Propionibacterium_freudenreichii	-	5.92E-01	0.000	0.000	0.000	0.000	0.000
2.5.1.78|g__Proteus.s__Proteus_mirabilis	-	5.92E-01	0.000	0.000	0.000	0.000	0.000
2.5.1.78|g__Pseudocitrobacter.s__Pseudocitrobacter_faecalis	+	2.32E-01	0.000	0.000	0.000	0.000	0.000
2.5.1.78|g__Pseudoflavonifractor.s__Pseudoflavonifractor_sp_An184	+	5.88E-02	0.000	0.000	0.000	0.000	0.000
2.5.1.78|g__Pseudomonas.s__Pseudomonas_aeruginosa	+	5.17E-01	0.000	0.000	0.000	0.000	0.000
2.5.1.78|g__Pseudomonas.s__Pseudomonas_aeruginosa_group	+	4.09E-01	0.000	0.000	0.000	0.000	0.000
2.5.1.78|g__Pyramidobacter.s__Pyramidobacter_piscolens	+	4.02E-01	0.000	0.000	0.000	0.000	0.000
2.5.1.78|g__Pyramidobacter.s__Pyramidobacter_sp_C12_8	+	4.92E-01	0.000	0.000	0.000	0.000	0.000
2.5.1.78|g__Raoultella.s__Raoultella_ornithinolytica	-	5.92E-01	0.000	0.000	0.000	0.000	0.000
2.5.1.78|g__Raoultella.s__Raoultella_planticola	-	2.01E-01	0.000	0.000	0.000	0.000	0.000
2.5.1.78|g__Rikenella.s__Rikenella_microfusus	+	4.92E-01	0.000	0.000	0.000	0.000	0.000
2.5.1.78|g__Roseburia.s__Roseburia_inulinivorans	-	1.85E-01	0.000	0.000	0.000	0.002	0.001
2.5.1.78|g__Rothia.s__Rothia_dentocariosa	+	7.60E-01	0.000	0.000	0.000	0.000	0.000
2.5.1.78|g__Rothia.s__Rothia_mucilaginosa	+	4.38E-01	0.000	0.000	0.000	0.000	0.000
2.5.1.78|g__Ruminococcaceae_unclassified.s__Ruminococcaceae_bacteriun	+	2.40E-02	0.000	0.000	0.000	0.001	0.003
2.5.1.78|g__Ruminococcaceae_unclassified.s__Ruminococcaceae_bacteriun	+	1.64E-02	0.000	0.000	0.000	0.000	0.001
2.5.1.78|g__Ruminococcus.s__Ruminococcus_callidus	-	2.05E-03	0.000	0.000	0.000	0.003	0.001
2.5.1.78|g__Ruminococcus.s__Ruminococcus_sp_AF31_8BH	-	3.65E-04	0.000	0.000	0.000	0.001	0.000
2.5.1.78|g__Saccharomyces.s__Saccharomyces_cerevisiae	-	1.48E-01	0.000	0.000	0.000	0.000	0.000
2.5.1.78|g__Salmonella.s__Salmonella_enterica	+	4.92E-01	0.000	0.000	0.000	0.000	0.000
2.5.1.78|g__Sanguibacteroides.s__Sanguibacteroides_justesenii	+	9.00E-02	0.000	0.000	0.000	0.000	0.000
2.5.1.78|g__Senegalimassilia.s__Senegalimassilia_anaerobia	+	4.90E-01	0.000	0.000	0.000	0.001	0.000
2.5.1.78|g__Slackia.s__Slackia_isoflavoniconvertens	+	7.59E-02	0.000	0.000	0.000	0.000	0.000
2.5.1.78|g__Staphylococcus.s__Staphylococcus_aureus	+	3.30E-01	0.000	0.000	0.000	0.000	0.000
2.5.1.78|g__Stomatobaculum.s__Stomatobaculum_longum	+	4.92E-01	0.000	0.000	0.000	0.000	0.000
2.5.1.78|g__Streptococcus.s__Streptococcus_agalactiae	-	5.95E-01	0.000	0.000	0.000	0.000	0.000
2.5.1.78|g__Streptococcus.s__Streptococcus_equinus	+	4.13E-01	0.000	0.000	0.000	0.000	0.000
2.5.1.78|g__Streptococcus.s__Streptococcus_gallolyticus	+	9.00E-02	0.000	0.000	0.000	0.000	0.000
2.5.1.78|g__Streptococcus.s__Streptococcus_infantarius	+	2.28E-01	0.000	0.000	0.000	0.000	0.000
2.5.1.78|g__Streptococcus.s__Streptococcus_lutetiensis	+	1.97E-02	0.000	0.000	0.000	0.001	0.002
2.5.1.78|g__Streptococcus.s__Streptococcus_macedonicus	+	9.16E-02	0.000	0.000	0.000	0.000	0.002
2.5.1.78|g__Streptococcus.s__Streptococcus_mitis	-	5.95E-01	0.000	0.000	0.000	0.000	0.000
2.5.1.78|g__Streptococcus.s__Streptococcus_oralis	-	7.14E-01	0.000	0.000	0.000	0.000	0.000
2.5.1.78|g__Streptococcus.s__Streptococcus_pasteurianus	+	4.35E-02	0.000	0.000	0.000	0.000	0.002
2.5.1.78|g__Streptococcus.s__Streptococcus_pneumoniae	-	9.25E-01	0.000	0.000	0.000	0.000	0.000
2.5.1.78|g__Succinatimonas.s__Succinatimonas_hippei	+	2.32E-01	0.000	0.000	0.000	0.000	0.000
2.5.1.78|g__Sutterella.s__Sutterella_wadsworthensis	+	2.45E-01	0.000	0.000	0.000	0.003	0.003
2.5.1.78|g__Terrisporobacter.s__Terrisporobacter_othiniensis	+	1.67E-01	0.000	0.000	0.000	0.000	0.000
2.5.1.78|g__Turicimonas.s__Turicimonas_muris	+	6.50E-01	0.000	0.000	0.000	0.000	0.000
2.5.1.78|g__Varibaculum.s__Varibaculum_cambriense	+	3.30E-01	0.000	0.000	0.000	0.000	0.000
2.5.1.78|g__Veillonella.s__Veillonella_atypica	-	4.50E-01	0.000	0.000	0.000	0.000	0.001
2.5.1.78|g__Veillonella.s__Veillonella_dispar	-	3.85E-01	0.000	0.000	0.000	0.000	0.000
2.5.1.78|g__Veillonella.s__Veillonella_infantium	-	8.30E-01	0.000	0.000	0.000	0.000	0.000
2.5.1.78|g__Veillonella.s__Veillonella_parvula	+	2.01E-01	0.000	0.000	0.000	0.000	0.001
2.5.1.78|g__Veillonella.s__Veillonella_rogosae	-	7.12E-01	0.000	0.000	0.000	0.000	0.000
2.5.1.78|g__Veillonella.s__Veillonella_seminalis	-	4.47E-01	0.000	0.000	0.000	0.000	0.000
2.5.1.78|g__Vibrio.s__Vibrio_parahaemolyticus	+	5.60E-01	0.000	0.000	0.000	0.000	0.000
2.5.1.78|g__Victivallales_unclassified.s__Victivallales_bacterium_CCUG_4	+	7.68E-02	0.000	0.000	0.000	0.000	0.000
2.5.1.78|g__Victivallis.s__Victivallis_vadensis	+	3.26E-02	0.000	0.000	0.000	0.000	0.000
2.5.1.78|g__Weeksella.s__Weeksella_virosa	+	4.92E-01	0.000	0.000	0.000	0.000	0.000
2.5.1.78|g__Weissella.s__Weissella_cibaria	-	9.70E-01	0.000	0.000	0.000	0.000	0.000
2.5.1.78|g__Weissella.s__Weissella_confusa	-	2.03E-01	0.000	0.000	0.000	0.000	0.000
2.5.1.78|g__Yokenella.s__Yokenella_regensburgei	+	2.32E-01	0.000	0.000	0.000	0.000	0.000
2.5.1.78|g__Bacteroides.s__Bacteroides_ovatus	+	9.19E-01	0.385	0.413	0.028	0.015	0.013
2.5.1.78|g__Bacteroides.s__Bacteroides_thetaiotaomicron	+	4.90E-01	0.214	0.252	0.038	0.007	0.009
2.5.1.78|g__Alistipes.s__Alistipes_shahii	+	4.28E-01	0.594	0.651	0.058	0.014	0.015
2.5.1.78|g__Phascolarctobacterium.s__Phascolarctobacterium_faecium	+	4.77E-01	0.241	0.320	0.079	0.009	0.010
2.5.1.78|g__Bacteroides.s__Bacteroides_caccae	+	3.08E-01	0.000	0.101	0.101	0.005	0.006
2.5.1.78|g__Bilophila.s__Bilophila_wadsworthia	+	6.25E-02	0.000	0.157	0.157	0.003	0.004
2.5.1.78|g__Parabacteroides.s__Parabacteroides_merdae	+	3.54E-02	0.407	0.601	0.195	0.008	0.010
2.5.1.78|g__Bacteroides.s__Bacteroides_cellulosilyticus	+	3.58E-01	0.016	0.222	0.206	0.012	0.014
2.5.1.78|g__Alistipes.s__Alistipes_onderdonkii	+	7.54E-03	0.162	0.404	0.242	0.011	0.015

	2.5.1.78|g__Bacteroides.s__Bacteroides_stercoris	+	1.12E-01	0.090	0.345	0.255	0.032	0.038
	2.5.1.78|g__Parabacteroides.s__Parabacteroides_distasonis	+	4.95E-03	0.869	1.367	0.499	0.019	0.023
	2.5.1.78|g__Alistipes.s__Alistipes_finegoldii	+	4.61E-03	0.618	1.176	0.559	0.022	0.029
	2.5.1.78|unclassified	+	8.38E-01	11.147	12.160	1.013	0.144	0.147
Germany	2.5.1.78	-	1.74E-01	124.324	120.541	-3.783	1.000	1.000
	2.5.1.78|g__Faecalibacterium.s__Faecalibacterium_prausnitzii	-	2.72E-02	10.451	4.963	-5.488	0.090	0.060
	2.5.1.78|g__Bacteroides.s__Bacteroides_uniformis	+	7.85E-01	3.220	2.423	-0.797	0.043	0.057
	2.5.1.78|g__Roseburia.s__Roseburia_faecis	-	1.47E-01	0.868	0.323	-0.545	0.018	0.011
	2.5.1.78|g__Bacteroides.s__Bacteroides_dorei	-	7.76E-01	0.501	0.136	-0.365	0.019	0.015
	2.5.1.78|g__Bacteroides.s__Bacteroides_caccae	-	3.80E-01	0.684	0.358	-0.326	0.016	0.010
	2.5.1.78|g__Blautia.s__Blautia_obeum	-	3.95E-01	0.728	0.458	-0.270	0.007	0.009
	2.5.1.78|g__Dorea.s__Dorea_longicatena	-	9.88E-01	0.934	0.713	-0.222	0.010	0.013
	2.5.1.78|g__Coprococcus.s__Coprococcus_catus	-	1.20E-01	0.177	0.000	-0.177	0.002	0.002
	2.5.1.78|g__Escherichia.s__Escherichia_coli	-	1.86E-01	0.168	0.000	-0.168	0.039	0.015
	2.5.1.78|g__Bacteroides.s__Bacteroides_ovatus	-	5.67E-01	0.564	0.420	-0.144	0.010	0.018
	2.5.1.78|g__Dorea.s__Dorea_formicigenerans	-	1.44E-01	0.250	0.120	-0.130	0.003	0.002
	2.5.1.78|g__Blautia.s__Blautia_wexlerae	-	1.25E-01	0.286	0.172	-0.114	0.005	0.003
	2.5.1.78|g__Roseburia.s__Roseburia_intestinalis	-	2.17E-01	0.112	0.000	-0.112	0.020	0.006
	2.5.1.78|g__Blautia.s__Ruminococcus_torques	+	5.67E-01	0.713	0.601	-0.112	0.012	0.019
	2.5.1.78|g__Parabacteroides.s__Parabacteroides_distasonis	+	9.58E-01	1.224	1.147	-0.077	0.012	0.013
	2.5.1.78|g__Bacteroides.s__Bacteroides_vulgatus	-	9.82E-01	2.878	2.821	-0.058	0.056	0.051
	2.5.1.78|g__Slackia.s__Slackia_isoflavoniconvertens	-	2.99E-01	0.056	0.000	-0.056	0.003	0.002
	2.5.1.78|g__Coprococcus.s__Coprococcus_comes	+	8.36E-01	0.382	0.336	-0.046	0.006	0.006
	2.5.1.78|g__Paraprevotella.s__Paraprevotella_clara	-	1.07E-02	0.041	0.000	-0.041	0.004	0.002
	2.5.1.78|g__Barnesiella.s__Barnesiella_intestinihominis	+	6.49E-01	1.133	1.122	-0.010	0.011	0.014
	2.5.1.78|g__Odoribacter.s__Odoribacter_splanchnicus	-	8.73E-01	0.464	0.462	-0.002	0.005	0.005
	2.5.1.78|g__Acidaminococcus.s__Acidaminococcus_fermentans	-	9.42E-01	0.000	0.000	0.000	0.001	0.000
	2.5.1.78|g__Acidaminococcus.s__Acidaminococcus_intestini	+	6.32E-01	0.000	0.000	0.000	0.001	0.002
	2.5.1.78|g__Actinomyces.s__Actinomyces_naeslundii	-	9.81E-01	0.000	0.000	0.000	0.000	0.000
	2.5.1.78|g__Actinomyces.s__Actinomyces_oris	-	3.09E-01	0.000	0.000	0.000	0.000	0.000
	2.5.1.78|g__Actinomyces.s__Actinomyces_viscosus	-	9.81E-01	0.000	0.000	0.000	0.000	0.000
	2.5.1.78|g__Adlercreutzia.s__Adlercreutzia_equolifaciens	+	5.32E-02	0.000	0.000	0.000	0.000	0.000
	2.5.1.78|g__Alistipes.s__Alistipes_indistinctus	+	8.69E-01	0.000	0.000	0.000	0.001	0.001
	2.5.1.78|g__Alistipes.s__Alistipes_inops	+	1.27E-01	0.000	0.000	0.000	0.002	0.004
	2.5.1.78|g__Alistipes.s__Alistipes_sp_An66	+	1.83E-01	0.000	0.000	0.000	0.000	0.000
	2.5.1.78|g__Alistipes.s__Alistipes_timonensis	+	6.32E-01	0.000	0.000	0.000	0.000	0.001
	2.5.1.78|g__Allisonella.s__Allisonella_histaminiformans	-	9.42E-01	0.000	0.000	0.000	0.000	0.000
	2.5.1.78|g__Anaeromassilibacillus.s__Anaeromassilibacillus_sp_An250	-	9.42E-01	0.000	0.000	0.000	0.000	0.000
	2.5.1.78|g__Anaerotignum.s__Anaerotignum_lactatifermentans	-	9.81E-01	0.000	0.000	0.000	0.000	0.000
	2.5.1.78|g__Asaccharobacter.s__Asaccharobacter_celatus	+	5.32E-02	0.000	0.000	0.000	0.000	0.000
	2.5.1.78|g__Bacteroides.s__Bacteroides_clarus	-	7.39E-01	0.000	0.000	0.000	0.003	0.001
	2.5.1.78|g__Bacteroides.s__Bacteroides_coprocola	-	5.28E-01	0.000	0.000	0.000	0.015	0.006
	2.5.1.78|g__Bacteroides.s__Bacteroides_coprophilus	+	8.14E-01	0.000	0.000	0.000	0.005	0.000
	2.5.1.78|g__Bacteroides.s__Bacteroides_eggerthii	+	7.06E-01	0.000	0.000	0.000	0.011	0.004
	2.5.1.78|g__Bacteroides.s__Bacteroides_faecichinchillae	+	1.83E-01	0.000	0.000	0.000	0.000	0.000
	2.5.1.78|g__Bacteroides.s__Bacteroides_faecis	+	9.11E-01	0.000	0.000	0.000	0.003	0.003
	2.5.1.78|g__Bacteroides.s__Bacteroides_finegoldii	+	3.37E-01	0.000	0.000	0.000	0.002	0.004
	2.5.1.78|g__Bacteroides.s__Bacteroides_fluxus	-	3.09E-01	0.000	0.000	0.000	0.000	0.000
	2.5.1.78|g__Bacteroides.s__Bacteroides_fragilis	+	4.62E-01	0.000	0.000	0.000	0.001	0.001
	2.5.1.78|g__Bacteroides.s__Bacteroides_intestinalis	+	9.41E-01	0.000	0.000	0.000	0.001	0.001
	2.5.1.78|g__Bacteroides.s__Bacteroides_massiliensis	+	9.87E-01	0.000	0.000	0.000	0.014	0.013
	2.5.1.78|g__Bacteroides.s__Bacteroides_nordii	-	8.51E-01	0.000	0.000	0.000	0.000	0.000
	2.5.1.78|g__Bacteroides.s__Bacteroides_plebeius	+	3.37E-01	0.000	0.000	0.000	0.002	0.007
	2.5.1.78|g__Bacteroides.s__Bacteroides_salyersiae	-	7.46E-01	0.000	0.000	0.000	0.001	0.003
	2.5.1.78|g__Bacteroides.s__Bacteroides_stercoris	-	5.00E-01	0.000	0.000	0.000	0.017	0.011
	2.5.1.78|g__Blautia.s__Blautia_hansenii	+	3.59E-01	0.000	0.000	0.000	0.000	0.000
	2.5.1.78|g__Butyricicoccus.s__Butyricicoccus_pullicaecorum	+	3.59E-01	0.000	0.000	0.000	0.000	0.000
	2.5.1.78|g__Butyricimonas.s__Butyricimonas_virosa	+	9.92E-01	0.000	0.000	0.000	0.002	0.001
	2.5.1.78|g__Butyrivibrio.s__Butyrivibrio_crossotus	+	8.14E-01	0.000	0.000	0.000	0.011	0.004
	2.5.1.78|g__Campylobacter.s__Campylobacter_concisus	-	3.09E-01	0.000	0.000	0.000	0.000	0.000
	2.5.1.78|g__Candidatus_Gastranaerophilales_unclassified.s__Candidatus_Ga	-	9.65E-01	0.000	0.000	0.000	0.001	0.001
	2.5.1.78|g__Candidatus_Methanomethylophilus.s__Candidatus_Methanome	+	3.59E-01	0.000	0.000	0.000	0.000	0.001
	2.5.1.78|g__Catenibacterium.s__Catenibacterium_mitsuokai	-	1.02E-01	0.000	0.000	0.000	0.009	0.004
	2.5.1.78|g__Citrobacter.s__Citrobacter_koseri	-	3.09E-01	0.000	0.000	0.000	0.000	0.000
	2.5.1.78|g__Citrobacter.s__Citrobacter_sp_MGH106	+	3.59E-01	0.000	0.000	0.000	0.000	0.000
	2.5.1.78|g__Cloacibacillus.s__Cloacibacillus_porcorum	+	6.04E-01	0.000	0.000	0.000	0.000	0.000
	2.5.1.78|g__Clostridioides.s__Clostridioides_difficile	+	6.32E-01	0.000	0.000	0.000	0.000	0.000
	2.5.1.78|g__Clostridium.s__Clostridium_celatum	-	3.09E-01	0.000	0.000	0.000	0.000	0.000
	2.5.1.78|g__Clostridium.s__Clostridium_disporicum	-	1.40E-01	0.000	0.000	0.000	0.000	0.000
	2.5.1.78|g__Clostridium.s__Clostridium_perfringens	-	1.40E-01	0.000	0.000	0.000	0.000	0.000
	2.5.1.78|g__Coprobacillus.s__Coprobacillus_cateniformis	+	4.43E-01	0.000	0.000	0.000	0.001	0.001
	2.5.1.78|g__Coprobacter.s__Coprobacter_fastidiosus	-	4.53E-01	0.000	0.000	0.000	0.003	0.001

2.5.1.78|g__Coprobacter.s__Coprobacter_secundus	+	5.53E-01	0.000	0.000	0.000	0.000	0.000
2.5.1.78|g__Coprococcus.s__Coprococcus_eutactus	-	8.00E-01	0.000	0.000	0.000	0.008	0.007
2.5.1.78|g__Desulfovibrio.s__Desulfovibrio_fairfieldensis	+	3.38E-01	0.000	0.000	0.000	0.000	0.001
2.5.1.78|g__Desulfovibrio.s__Desulfovibrio_piger	-	7.71E-01	0.000	0.000	0.000	0.001	0.005
2.5.1.78|g__Desulfovibrionaceae_unclassified.s__Desulfovibrionaceae_bact	-	3.09E-01	0.000	0.000	0.000	0.000	0.000
2.5.1.78|g__Dialister.s__Dialister_invisus	-	7.18E-01	0.000	0.000	0.000	0.009	0.011
2.5.1.78|g__Dialister.s__Dialister_succinatiphilus	-	9.42E-01	0.000	0.000	0.000	0.002	0.001
2.5.1.78|g__Dorea.s__Dorea_sp_OM02_2LB	-	5.11E-01	0.000	0.000	0.000	0.000	0.000
2.5.1.78|g__Eggerthella.s__Eggerthella_lenta	-	9.42E-01	0.000	0.000	0.000	0.000	0.000
2.5.1.78|g__Eisenbergiella.s__Eisenbergiella_tayi	-	3.09E-01	0.000	0.000	0.000	0.000	0.000
2.5.1.78|g__Enterococcus.s__Enterococcus_faecium	+	3.59E-01	0.000	0.000	0.000	0.000	0.000
2.5.1.78|g__Enterococcus.s__Enterococcus_hirae	-	3.09E-01	0.000	0.000	0.000	0.000	0.000
2.5.1.78|g__Erysipelatoclostridium.s__Clostridium_spiroforme	-	8.75E-01	0.000	0.000	0.000	0.001	0.000
2.5.1.78|g__Erysipelotrichaceae_unclassified.s__Erysipelotrichaceae_bacteri	-	3.09E-01	0.000	0.000	0.000	0.000	0.000
2.5.1.78|g__Escherichia.s__Escherichia_fergusonii	-	8.75E-01	0.000	0.000	0.000	0.000	0.000
2.5.1.78|g__Escherichia.s__Escherichia_marmotae	-	3.09E-01	0.000	0.000	0.000	0.000	0.000
2.5.1.78|g__Eubacterium.s__Eubacterium_ramulus	-	7.82E-01	0.000	0.000	0.000	0.002	0.002
2.5.1.78|g__Eubacterium.s__Eubacterium_sp_AF17_7	+	7.66E-01	0.000	0.000	0.000	0.000	0.001
2.5.1.78|g__Faecalicatena.s__Faecalicatena_contorta	+	3.59E-01	0.000	0.000	0.000	0.000	0.000
2.5.1.78|g__Faecalitalea.s__Faecalitalea_cylindroides	-	8.75E-01	0.000	0.000	0.000	0.002	0.000
2.5.1.78|g__Firmicutes_unclassified.s__Firmicutes_bacterium_AM10_47	-	8.75E-01	0.000	0.000	0.000	0.001	0.000
2.5.1.78|g__Flavonifractor.s__Flavonifractor_plautii	+	1.12E-02	0.000	0.000	0.000	0.000	0.002
2.5.1.78|g__Flavonifractor.s__Flavonifractor_sp_An10	+	6.32E-01	0.000	0.000	0.000	0.000	0.000
2.5.1.78|g__Fusobacterium.s__Fusobacterium_mortiferum	-	3.09E-01	0.000	0.000	0.000	0.000	0.000
2.5.1.78|g__Haemophilus.s__Haemophilus_parainfluenzae	-	3.92E-02	0.000	0.000	0.000	0.002	0.000
2.5.1.78|g__Hafnia.s__Hafnia_alvei	+	6.04E-01	0.000	0.000	0.000	0.000	0.001
2.5.1.78|g__Hafnia.s__Hafnia_paralvei	+	3.59E-01	0.000	0.000	0.000	0.000	0.001
2.5.1.78|g__Holdemanella.s__Holdemanella_biformis	-	3.09E-01	0.000	0.000	0.000	0.000	0.000
2.5.1.78|g__Hungatella.s__Hungatella_hathewayi	+	7.46E-01	0.000	0.000	0.000	0.000	0.000
2.5.1.78|g__Intestinibacter.s__Intestinibacter_bartlettii	-	8.51E-01	0.000	0.000	0.000	0.000	0.000
2.5.1.78|g__Klebsiella.s__Klebsiella_aerogenes	-	1.40E-01	0.000	0.000	0.000	0.000	0.000
2.5.1.78|g__Klebsiella.s__Klebsiella_oxytoca	+	3.56E-01	0.000	0.000	0.000	0.000	0.000
2.5.1.78|g__Klebsiella.s__Klebsiella_pneumoniae	-	1.68E-01	0.000	0.000	0.000	0.006	0.000
2.5.1.78|g__Klebsiella.s__Klebsiella_variicola	-	3.32E-01	0.000	0.000	0.000	0.001	0.000
2.5.1.78|g__Lachnoclostridium.s__Clostridium_aldenense	-	1.40E-01	0.000	0.000	0.000	0.000	0.000
2.5.1.78|g__Lachnoclostridium.s__Clostridium_bolteae	+	3.56E-01	0.000	0.000	0.000	0.000	0.000
2.5.1.78|g__Lachnoclostridium.s__Clostridium_citroniae	-	3.09E-01	0.000	0.000	0.000	0.000	0.000
2.5.1.78|g__Lachnoclostridium.s__Clostridium_clostridioforme	-	4.91E-01	0.000	0.000	0.000	0.001	0.000
2.5.1.78|g__Lachnoclostridium.s__Clostridium_symbiosum	-	3.09E-01	0.000	0.000	0.000	0.000	0.000
2.5.1.78|g__Lachnoclostridium.s__Lachnoclostridium_sp_An138	+	3.59E-01	0.000	0.000	0.000	0.000	0.000
2.5.1.78|g__Lachnospira.s__Lachnospira_pectinoschiza	+	6.62E-01	0.000	0.000	0.000	0.002	0.003
2.5.1.78|g__Lachnospiraceae_unclassified.s__Lachnospiraceae_bacterium_(	-	3.09E-01	0.000	0.000	0.000	0.000	0.000
2.5.1.78|g__Lactobacillus.s__Lactobacillus_antri	-	3.09E-01	0.000	0.000	0.000	0.000	0.000
2.5.1.78|g__Lactobacillus.s__Lactobacillus_crispatus	-	3.09E-01	0.000	0.000	0.000	0.000	0.000
2.5.1.78|g__Lactobacillus.s__Lactobacillus_fermentum	-	3.09E-01	0.000	0.000	0.000	0.000	0.000
2.5.1.78|g__Lactobacillus.s__Lactobacillus_gasseri	-	3.09E-01	0.000	0.000	0.000	0.001	0.000
2.5.1.78|g__Lactobacillus.s__Lactobacillus_gastricus	-	3.09E-01	0.000	0.000	0.000	0.000	0.000
2.5.1.78|g__Lactobacillus.s__Lactobacillus_oris	-	3.09E-01	0.000	0.000	0.000	0.000	0.000
2.5.1.78|g__Lactobacillus.s__Lactobacillus_paragasseri	-	3.09E-01	0.000	0.000	0.000	0.000	0.000
2.5.1.78|g__Lactobacillus.s__Lactobacillus_rogosae	+	6.35E-01	0.000	0.000	0.000	0.001	0.003
2.5.1.78|g__Lactococcus.s__Lactococcus_lactis	-	1.20E-01	0.000	0.000	0.000	0.003	0.000
2.5.1.78|g__Megamonas.s__Megamonas_funiformis	+	3.59E-01	0.000	0.000	0.000	0.000	0.001
2.5.1.78|g__Megamonas.s__Megamonas_hypermegale	+	3.59E-01	0.000	0.000	0.000	0.000	0.000
2.5.1.78|g__Megamonas.s__Megamonas_rupellensis	+	3.59E-01	0.000	0.000	0.000	0.000	0.000
2.5.1.78|g__Megasphaera.s__Megasphaera_elsdenii	-	5.11E-01	0.000	0.000	0.000	0.001	0.000
2.5.1.78|g__Megasphaera.s__Megasphaera_hexanoica	-	3.09E-01	0.000	0.000	0.000	0.000	0.000
2.5.1.78|g__Megasphaera.s__Megasphaera_sp_DISK_18	+	3.59E-01	0.000	0.000	0.000	0.000	0.001
2.5.1.78|g__Mesosutterella.s__Mesosutterella_multiformis	+	1.29E-01	0.000	0.000	0.000	0.000	0.001
2.5.1.78|g__Mitsuokella.s__Mitsuokella_jalaludinii	-	5.11E-01	0.000	0.000	0.000	0.001	0.000
2.5.1.78|g__Mitsuokella.s__Mitsuokella_multacida	-	3.09E-01	0.000	0.000	0.000	0.000	0.000
2.5.1.78|g__Obesumbacterium.s__Obesumbacterium_proteus	+	1.83E-01	0.000	0.000	0.000	0.000	0.000
2.5.1.78|g__Oxalobacter.s__Oxalobacter_formigenes	+	2.32E-01	0.000	0.000	0.000	0.000	0.000
2.5.1.78|g__Parabacteroides.s__Parabacteroides_goldsteinii	-	5.77E-01	0.000	0.000	0.000	0.001	0.000
2.5.1.78|g__Parabacteroides.s__Parabacteroides_gordonii	+	3.38E-01	0.000	0.000	0.000	0.000	0.000
2.5.1.78|g__Parabacteroides.s__Parabacteroides_johnsonii	-	2.91E-01	0.000	0.000	0.000	0.001	0.000
2.5.1.78|g__Parasutterella.s__Parasutterella_excrementihominis	+	8.16E-01	0.000	0.000	0.000	0.002	0.002
2.5.1.78|g__Phascolarctobacterium.s__Phascolarctobacterium_faecium	+	2.67E-01	0.000	0.000	0.000	0.004	0.009
2.5.1.78|g__Phascolarctobacterium.s__Phascolarctobacterium_succinatutens	-	1.92E-02	0.000	0.000	0.000	0.007	0.002
2.5.1.78|g__Prevotella.s__Prevotella_copri	-	6.66E-02	0.000	0.000	0.000	0.009	0.000
2.5.1.78|g__Prevotella.s__Prevotella_disiens	-	1.40E-01	0.000	0.000	0.000	0.000	0.000
2.5.1.78|g__Prevotella.s__Prevotella_sp_109	+	1.83E-01	0.000	0.000	0.000	0.000	0.011
2.5.1.78|g__Prevotella.s__Prevotella_sp_885	-	5.37E-01	0.000	0.000	0.000	0.001	0.001

	2.5.1.78|g__Prevotella.s__Prevotella_sp_AM42_24	-	2.73E-01	0.000	0.000	0.000	0.011	0.006
	2.5.1.78|g__Pseudoflavonifractor.s__Pseudoflavonifractor_sp_An184	+	3.93E-01	0.000	0.000	0.000	0.000	0.000
	2.5.1.78|g__Pseudomonas.s__Pseudomonas_aeruginosa	-	4.85E-01	0.000	0.000	0.000	0.000	0.000
	2.5.1.78|g__Pseudomonas.s__Pseudomonas_aeruginosa_group	-	5.11E-01	0.000	0.000	0.000	0.000	0.000
	2.5.1.78|g__Pyramidobacter.s__Pyramidobacter_piscolens	-	3.09E-01	0.000	0.000	0.000	0.000	0.000
	2.5.1.78|g__Roseburia.s__Roseburia_inulinivorans	+	1.97E-01	0.000	0.000	0.000	0.002	0.002
	2.5.1.78|g__Ruminococcaceae_unclassified.s__Ruminococcaceae_bacteriun	-	3.04E-01	0.000	0.000	0.000	0.001	0.000
	2.5.1.78|g__Ruminococcaceae_unclassified.s__Ruminococcaceae_bacteriun	+	1.83E-01	0.000	0.000	0.000	0.000	0.000
	2.5.1.78|g__Ruminococcus.s__Ruminococcus_callidus	-	7.89E-01	0.000	0.000	0.000	0.002	0.001
	2.5.1.78|g__Ruminococcus.s__Ruminococcus_sp_AF31_8BH	+	2.85E-01	0.000	0.000	0.000	0.000	0.001
	2.5.1.78|g__Sanguibacteroides.s__Sanguibacteroides_justesenii	-	9.42E-01	0.000	0.000	0.000	0.000	0.000
	2.5.1.78|g__Senegalimassilia.s__Senegalimassilia_anaerobia	+	9.47E-01	0.000	0.000	0.000	0.001	0.001
	2.5.1.78|g__Streptococcus.s__Streptococcus_lutetiensis	+	3.59E-01	0.000	0.000	0.000	0.000	0.001
	2.5.1.78|g__Streptococcus.s__Streptococcus_pneumoniae	-	9.42E-01	0.000	0.000	0.000	0.000	0.000
	2.5.1.78|g__Sutterella.s__Sutterella_wadsworthensis	-	9.53E-01	0.000	0.000	0.000	0.006	0.006
	2.5.1.78|g__Turicimonas.s__Turicimonas_muris	-	3.09E-01	0.000	0.000	0.000	0.001	0.000
	2.5.1.78|g__Veillonella.s__Veillonella_atypica	-	6.66E-02	0.000	0.000	0.000	0.000	0.000
	2.5.1.78|g__Veillonella.s__Veillonella_dispar	-	8.75E-01	0.000	0.000	0.000	0.000	0.000
	2.5.1.78|g__Veillonella.s__Veillonella_infantium	+	6.61E-01	0.000	0.000	0.000	0.000	0.000
	2.5.1.78|g__Veillonella.s__Veillonella_parvula	+	3.59E-01	0.000	0.000	0.000	0.000	0.000
	2.5.1.78|g__Veillonella.s__Veillonella_rogosae	-	3.09E-01	0.000	0.000	0.000	0.000	0.000
	2.5.1.78|g__Victivallales_unclassified.s__Victivallales_bacterium_CCUG_4	-	4.64E-01	0.000	0.000	0.000	0.001	0.001
	2.5.1.78|g__Victivallis.s__Victivallis_vadensis	+	9.88E-01	0.000	0.000	0.000	0.000	0.000
	2.5.1.78|g__Eubacterium.s__Eubacterium_eligens	+	9.27E-01	0.868	0.874	0.006	0.018	0.013
	2.5.1.78|g__Bacteroides.s__Bacteroides_xylanisolvens	+	7.01E-01	0.091	0.127	0.036	0.003	0.007
	2.5.1.78|g__Bacteroides.s__Bacteroides_cellulosilyticus	+	7.06E-01	0.397	0.514	0.118	0.010	0.012
	2.5.1.78|g__Methanobrevibacter.s__Methanobrevibacter_smithii	+	1.18E-01	0.000	0.167	0.167	0.006	0.009
	2.5.1.78|g__Clostridium.s__Clostridium_sp_AM22_11AC	+	5.94E-01	0.000	0.177	0.177	0.002	0.003
	2.5.1.78|g__Bacteroides.s__Bacteroides_thetaiotaomicron	-	6.96E-01	0.219	0.436	0.217	0.010	0.005
	2.5.1.78|g__Lachnospiraceae_unclassified.s__Eubacterium_rectale	+	5.89E-01	0.539	0.778	0.239	0.015	0.016
	2.5.1.78|g__Agathobaculum.s__Agathobaculum_butyriciproducens	+	1.51E-01	0.000	0.278	0.278	0.002	0.003
	2.5.1.78|g__Bilophila.s__Bilophila_wadsworthia	+	2.43E-01	0.000	0.320	0.320	0.002	0.003
	2.5.1.78|g__Alistipes.s__Alistipes_finegoldii	+	2.33E-01	0.669	1.022	0.353	0.022	0.030
	2.5.1.78|g__Clostridium.s__Clostridium_sp_AF36_4	+	4.61E-03	0.000	0.356	0.356	0.002	0.007
	2.5.1.78|g__Parabacteroides.s__Parabacteroides_merdae	+	4.20E-01	0.479	0.894	0.415	0.008	0.011
	2.5.1.78|g__Alistipes.s__Alistipes_onderdonkii	+	3.33E-02	0.140	0.646	0.506	0.009	0.015
	2.5.1.78|g__Anaerostipes.s__Anaerostipes_hadrus	+	9.19E-02	0.148	0.664	0.516	0.004	0.011
	2.5.1.78|g__Alistipes.s__Alistipes_shahii	+	7.55E-03	0.405	1.148	0.743	0.007	0.017
	2.5.1.78|g__Alistipes.s__Alistipes_putredinis	+	2.05E-01	2.814	3.773	0.959	0.032	0.041
	2.5.1.78|unclassified	-	6.99E-01	20.516	23.702	3.187	0.240	0.229
	2.5.1.78|g__Akkermansia.s__Akkermansia_muciniphila	+	5.45E-04	0.000	3.229	3.229	0.009	0.068
China1	2.5.1.78	-	7.40E-01	137.879	141.182	3.303	1.000	1.000
	2.5.1.78|g__Bacteroides.s__Bacteroides_vulgatus	-	1.24E-02	9.467	2.845	-6.622	0.119	0.053
	2.5.1.78|g__Bacteroides.s__Bacteroides_ovatus	-	1.82E-02	5.199	2.125	-3.074	0.076	0.030
	2.5.1.78|g__Escherichia.s__Escherichia_coli	-	5.66E-01	0.628	0.237	-0.391	0.026	0.034
	2.5.1.78|g__Bacteroides.s__Bacteroides_fragilis	-	3.00E-01	0.314	0.098	-0.217	0.008	0.012
	2.5.1.78|g__Roseburia.s__Roseburia_faecis	-	4.91E-01	0.161	0.077	-0.084	0.004	0.004
	2.5.1.78|g__Blautia.s__Blautia_wexlerae	-	2.72E-01	0.026	0.000	-0.026	0.001	0.001
	2.5.1.78|g__Bacteroides.s__Bacteroides_plebeius	-	3.71E-01	0.019	0.000	-0.019	0.056	0.019
	2.5.1.78|g__Acidaminococcus.s__Acidaminococcus_intestini	+	3.30E-01	0.000	0.000	0.000	0.000	0.000
	2.5.1.78|g__Adlercreutzia.s__Adlercreutzia_equolifaciens	+	5.69E-01	0.000	0.000	0.000	0.000	0.000
	2.5.1.78|g__Aeromonas.s__Aeromonas_hydrophila	-	3.30E-01	0.000	0.000	0.000	0.000	0.000
	2.5.1.78|g__Agathobaculum.s__Agathobaculum_butyriciproducens	-	7.04E-01	0.000	0.000	0.000	0.001	0.000
	2.5.1.78|g__Aggregatibacter.s__Aggregatibacter_segnis	-	1.00E+00	0.000	0.000	0.000	0.000	0.000
	2.5.1.78|g__Akkermansia.s__Akkermansia_muciniphila	+	2.86E-02	0.000	0.000	0.000	0.001	0.004
	2.5.1.78|g__Alistipes.s__Alistipes_inops	-	9.90E-01	0.000	0.000	0.000	0.002	0.001
	2.5.1.78|g__Alistipes.s__Alistipes_sp_An66	+	3.30E-01	0.000	0.000	0.000	0.000	0.000
	2.5.1.78|g__Alistipes.s__Alistipes_timonensis	+	9.90E-01	0.000	0.000	0.000	0.001	0.000
	2.5.1.78|g__Anaeroglobus.s__Anaeroglobus_geminatus	+	1.00E+00	0.000	0.000	0.000	0.000	0.000
	2.5.1.78|g__Anaeromassilibacillus.s__Anaeromassilibacillus_sp_An250	+	1.60E-01	0.000	0.000	0.000	0.000	0.000
	2.5.1.78|g__Anaerostipes.s__Anaerostipes_caccae	-	3.30E-01	0.000	0.000	0.000	0.000	0.000
	2.5.1.78|g__Anaerostipes.s__Anaerostipes_hadrus	+	7.94E-01	0.000	0.000	0.000	0.001	0.001
	2.5.1.78|g__Anaerotignum.s__Anaerotignum_lactatifermentans	-	3.30E-01	0.000	0.000	0.000	0.000	0.000
	2.5.1.78|g__Asaccharobacter.s__Asaccharobacter_celatus	+	5.89E-01	0.000	0.000	0.000	0.000	0.000
	2.5.1.78|g__Bacteroides.s__Bacteroides_clarus	+	1.54E-01	0.000	0.000	0.000	0.001	0.006
	2.5.1.78|g__Bacteroides.s__Bacteroides_coprocola	-	2.24E-01	0.000	0.000	0.000	0.044	0.011
	2.5.1.78|g__Bacteroides.s__Bacteroides_coprophilus	-	3.93E-01	0.000	0.000	0.000	0.005	0.001
	2.5.1.78|g__Bacteroides.s__Bacteroides_eggerthii	+	9.47E-02	0.000	0.000	0.000	0.018	0.033
	2.5.1.78|g__Bacteroides.s__Bacteroides_faecichinchillae	+	4.28E-02	0.000	0.000	0.000	0.000	0.000
	2.5.1.78|g__Bacteroides.s__Bacteroides_faecis	-	1.58E-01	0.000	0.000	0.000	0.001	0.000
	2.5.1.78|g__Bacteroides.s__Bacteroides_finegoldii	+	4.14E-01	0.000	0.000	0.000	0.008	0.012
	2.5.1.78|g__Bacteroides.s__Bacteroides_fluxus	+	3.30E-01	0.000	0.000	0.000	0.000	0.000

2.5.1.78|g__Bacteroides.s__Bacteroides_massiliensis	+	8.48E-01	0.000	0.000	0.000	0.009	0.012
2.5.1.78|g__Bacteroides.s__Bacteroides_oleiciplenus	+	5.89E-01	0.000	0.000	0.000	0.000	0.000
2.5.1.78|g__Bacteroides.s__Bacteroides_salyersiae	-	4.45E-01	0.000	0.000	0.000	0.002	0.002
2.5.1.78|g__Bacteroides.s__Bacteroides_sp_OM08_11	+	8.20E-02	0.000	0.000	0.000	0.000	0.000
2.5.1.78|g__Bacteroides.s__Bacteroides_stercorirosoris	+	5.49E-01	0.000	0.000	0.000	0.000	0.001
2.5.1.78|g__Barnesiella.s__Barnesiella_intestinihominis	+	2.81E-01	0.000	0.000	0.000	0.006	0.005
2.5.1.78|g__Barnesiella.s__Barnesiella_sp_An22	-	3.30E-01	0.000	0.000	0.000	0.000	0.000
2.5.1.78|g__Blautia.s__Blautia_hansenii	+	1.60E-01	0.000	0.000	0.000	0.000	0.000
2.5.1.78|g__Butyricimonas.s__Butyricimonas_synergistica	-	1.00E+00	0.000	0.000	0.000	0.000	0.000
2.5.1.78|g__Butyricimonas.s__Butyricimonas_virosa	-	8.43E-01	0.000	0.000	0.000	0.003	0.003
2.5.1.78|g__Butyrivibrio.s__Butyrivibrio_crossotus	+	5.69E-01	0.000	0.000	0.000	0.000	0.000
2.5.1.78|g__Campylobacter.s__Campylobacter_concisus	-	3.30E-01	0.000	0.000	0.000	0.000	0.000
2.5.1.78|g__Campylobacter.s__Campylobacter_gracilis	+	3.30E-01	0.000	0.000	0.000	0.000	0.000
2.5.1.78|g__Catenibacterium.s__Catenibacterium_mitsuokai	+	6.22E-01	0.000	0.000	0.000	0.001	0.003
2.5.1.78|g__Citrobacter.s__Citrobacter_braakii	+	3.30E-01	0.000	0.000	0.000	0.000	0.000
2.5.1.78|g__Citrobacter.s__Citrobacter_farmeri	-	3.30E-01	0.000	0.000	0.000	0.000	0.000
2.5.1.78|g__Citrobacter.s__Citrobacter_freundii	+	3.30E-01	0.000	0.000	0.000	0.000	0.000
2.5.1.78|g__Citrobacter.s__Citrobacter_portucalensis	+	1.58E-01	0.000	0.000	0.000	0.000	0.000
2.5.1.78|g__Citrobacter.s__Citrobacter_youngae	+	2.96E-01	0.000	0.000	0.000	0.000	0.000
2.5.1.78|g__Cloacibacillus.s__Cloacibacillus_porcorum	+	8.20E-02	0.000	0.000	0.000	0.000	0.000
2.5.1.78|g__Clostridioides.s__Clostridioides_difficile	-	8.05E-01	0.000	0.000	0.000	0.001	0.000
2.5.1.78|g__Clostridium.s__Clostridium_disporicum	-	3.30E-01	0.000	0.000	0.000	0.000	0.000
2.5.1.78|g__Clostridium.s__Clostridium_sp_AF36_4	+	6.86E-02	0.000	0.000	0.000	0.001	0.004
2.5.1.78|g__Clostridium.s__Clostridium_sp_AM22_11AC	-	5.84E-01	0.000	0.000	0.000	0.001	0.000
2.5.1.78|g__Clostridium.s__Clostridium_sp_chh4_2	+	3.30E-01	0.000	0.000	0.000	0.000	0.000
2.5.1.78|g__Coprobacillus.s__Coprobacillus_cateniformis	+	9.39E-02	0.000	0.000	0.000	0.000	0.001
2.5.1.78|g__Coprobacter.s__Coprobacter_fastidiosus	+	8.49E-01	0.000	0.000	0.000	0.001	0.001
2.5.1.78|g__Coprobacter.s__Coprobacter_secundus	-	9.73E-01	0.000	0.000	0.000	0.000	0.000
2.5.1.78|g__Coprococcus.s__Coprococcus_catus	+	7.02E-01	0.000	0.000	0.000	0.000	0.000
2.5.1.78|g__Coprococcus.s__Coprococcus_comes	+	1.76E-01	0.000	0.000	0.000	0.000	0.001
2.5.1.78|g__Coprococcus.s__Coprococcus_eutactus	+	2.51E-01	0.000	0.000	0.000	0.000	0.001
2.5.1.78|g__Desulfovibrio.s__Desulfovibrio_piger	+	6.06E-01	0.000	0.000	0.000	0.000	0.000
2.5.1.78|g__Desulfovibrio.s__Desulfovibrio_sp_AM18_2	+	8.20E-02	0.000	0.000	0.000	0.000	0.001
2.5.1.78|g__Dialister.s__Dialister_invisus	+	9.79E-01	0.000	0.000	0.000	0.003	0.009
2.5.1.78|g__Dialister.s__Dialister_succinatiphilus	-	5.89E-01	0.000	0.000	0.000	0.000	0.000
2.5.1.78|g__Dorea.s__Dorea_formicigenerans	-	3.39E-01	0.000	0.000	0.000	0.001	0.000
2.5.1.78|g__Eggerthella.s__Eggerthella_lenta	+	7.06E-01	0.000	0.000	0.000	0.000	0.000
2.5.1.78|g__Eisenbergiella.s__Eisenbergiella_tayi	+	8.20E-02	0.000	0.000	0.000	0.000	0.000
2.5.1.78|g__Enterobacter.s__Enterobacter_cloacae	+	6.38E-01	0.000	0.000	0.000	0.000	0.000
2.5.1.78|g__Enterococcus.s__Enterococcus_faecium	+	3.30E-01	0.000	0.000	0.000	0.000	0.000
2.5.1.78|g__Enterococcus.s__Enterococcus_hirae	+	3.30E-01	0.000	0.000	0.000	0.000	0.000
2.5.1.78|g__Erysipelatoclostridium.s__Clostridium_innocuum	+	3.30E-01	0.000	0.000	0.000	0.000	0.000
2.5.1.78|g__Erysipelatoclostridium.s__Clostridium_spiroforme	+	3.30E-01	0.000	0.000	0.000	0.000	0.000
2.5.1.78|g__Erysipelotrichaceae_unclassified.s__Erysipelotrichaceae_bacteri	-	3.30E-01	0.000	0.000	0.000	0.000	0.000
2.5.1.78|g__Escherichia.s__Escherichia_fergusonii	+	1.00E+00	0.000	0.000	0.000	0.000	0.000
2.5.1.78|g__Eubacterium.s__Eubacterium_ramulus	+	3.93E-01	0.000	0.000	0.000	0.000	0.000
2.5.1.78|g__Eubacterium.s__Eubacterium_sp_AF17_7	+	2.43E-01	0.000	0.000	0.000	0.000	0.001
2.5.1.78|g__Eubacterium.s__Eubacterium_sp_AM18_10LB_B	+	1.00E+00	0.000	0.000	0.000	0.000	0.001
2.5.1.78|g__Faecalicatena.s__Faecalicatena_contorta	-	3.30E-01	0.000	0.000	0.000	0.000	0.000
2.5.1.78|g__Faecalitalea.s__Faecalitalea_cylindroides	+	3.30E-01	0.000	0.000	0.000	0.000	0.000
2.5.1.78|g__Firmicutes_unclassified.s__Firmicutes_bacterium_AM10_47	+	8.20E-02	0.000	0.000	0.000	0.000	0.000
2.5.1.78|g__Flavonifractor.s__Flavonifractor_plautii	+	8.20E-02	0.000	0.000	0.000	0.000	0.000
2.5.1.78|g__Fusobacterium.s__Fusobacterium_hwasookii	-	3.30E-01	0.000	0.000	0.000	0.000	0.000
2.5.1.78|g__Fusobacterium.s__Fusobacterium_mortiferum	+	6.71E-01	0.000	0.000	0.000	0.001	0.000
2.5.1.78|g__Fusobacterium.s__Fusobacterium_ulcerans	-	6.71E-01	0.000	0.000	0.000	0.001	0.001
2.5.1.78|g__Fusobacterium.s__Fusobacterium_varium	-	1.60E-01	0.000	0.000	0.000	0.001	0.000
2.5.1.78|g__Haemophilus.s__Haemophilus_influenzae	-	3.30E-01	0.000	0.000	0.000	0.000	0.000
2.5.1.78|g__Haemophilus.s__Haemophilus_parainfluenzae	-	6.35E-01	0.000	0.000	0.000	0.002	0.002
2.5.1.78|g__Haemophilus.s__Haemophilus_sputorum	+	3.30E-01	0.000	0.000	0.000	0.000	0.000
2.5.1.78|g__Hafnia.s__Hafnia_alvei	-	3.30E-01	0.000	0.000	0.000	0.000	0.000
2.5.1.78|g__Hafnia.s__Hafnia_paralvei	-	3.30E-01	0.000	0.000	0.000	0.000	0.000
2.5.1.78|g__Hungatella.s__Hungatella_hathewayi	+	8.60E-01	0.000	0.000	0.000	0.000	0.000
2.5.1.78|g__Intestinibacter.s__Intestinibacter_bartlettii	-	3.93E-01	0.000	0.000	0.000	0.000	0.000
2.5.1.78|g__Klebsiella.s__Klebsiella_aerogenes	-	5.89E-01	0.000	0.000	0.000	0.000	0.000
2.5.1.78|g__Klebsiella.s__Klebsiella_oxytoca	+	2.03E-01	0.000	0.000	0.000	0.000	0.000
2.5.1.78|g__Klebsiella.s__Klebsiella_pneumoniae	+	4.86E-01	0.000	0.000	0.000	0.001	0.004
2.5.1.78|g__Klebsiella.s__Klebsiella_variicola	+	4.28E-02	0.000	0.000	0.000	0.000	0.000
2.5.1.78|g__Kosakonia.s__Kosakonia_cowanii	-	3.30E-01	0.000	0.000	0.000	0.000	0.000
2.5.1.78|g__Lachnoclostridium.s__Clostridium_aldenense	-	5.79E-01	0.000	0.000	0.000	0.000	0.000
2.5.1.78|g__Lachnoclostridium.s__Clostridium_bolteae	+	7.57E-01	0.000	0.000	0.000	0.000	0.000
2.5.1.78|g__Lachnoclostridium.s__Clostridium_citroniae	+	1.60E-01	0.000	0.000	0.000	0.000	0.000
2.5.1.78|g__Lachnoclostridium.s__Clostridium_clostridioforme	+	5.77E-01	0.000	0.000	0.000	0.001	0.000

2.5.1.78|g__Lachnoclostridium.s__Clostridium_symbiosum	+	1.00E+00	0.000	0.000	0.000	0.000	0.000
2.5.1.78|g__Lachnospira.s__Lachnospira_pectinoschiza	-	1.48E-01	0.000	0.000	0.000	0.003	0.002
2.5.1.78|g__Lactobacillus.s__Lactobacillus_crispatus	-	1.60E-01	0.000	0.000	0.000	0.000	0.000
2.5.1.78|g__Lactobacillus.s__Lactobacillus_delbrueckii	+	3.30E-01	0.000	0.000	0.000	0.000	0.000
2.5.1.78|g__Lactobacillus.s__Lactobacillus_fermentum	+	3.30E-01	0.000	0.000	0.000	0.000	0.000
2.5.1.78|g__Lactobacillus.s__Lactobacillus_gasseri	+	3.30E-01	0.000	0.000	0.000	0.000	0.000
2.5.1.78|g__Lactobacillus.s__Lactobacillus_paragasseri	+	1.60E-01	0.000	0.000	0.000	0.000	0.000
2.5.1.78|g__Lactobacillus.s__Lactobacillus_rogosae	-	3.57E-01	0.000	0.000	0.000	0.003	0.002
2.5.1.78|g__Megamonas.s__Megamonas_funiformis	+	9.87E-01	0.000	0.000	0.000	0.003	0.002
2.5.1.78|g__Megamonas.s__Megamonas_hypermegale	+	9.63E-01	0.000	0.000	0.000	0.000	0.000
2.5.1.78|g__Megamonas.s__Megamonas_rupellensis	+	7.61E-01	0.000	0.000	0.000	0.002	0.000
2.5.1.78|g__Megasphaera.s__Megasphaera_micronuciformis	-	1.00E+00	0.000	0.000	0.000	0.000	0.000
2.5.1.78|g__Mesosutterella.s__Mesosutterella_multiformis	-	8.02E-01	0.000	0.000	0.000	0.003	0.004
2.5.1.78|g__Methanobrevibacter.s__Methanobrevibacter_smithii	+	1.58E-01	0.000	0.000	0.000	0.000	0.006
2.5.1.78|g__Obesumbacterium.s__Obesumbacterium_proteus	-	3.30E-01	0.000	0.000	0.000	0.000	0.000
2.5.1.78|g__Oxalobacter.s__Oxalobacter_formigenes	-	6.88E-01	0.000	0.000	0.000	0.000	0.000
2.5.1.78|g__Parabacteroides.s__Parabacteroides_goldsteinii	+	5.86E-02	0.000	0.000	0.000	0.001	0.004
2.5.1.78|g__Parabacteroides.s__Parabacteroides_gordonii	+	1.86E-01	0.000	0.000	0.000	0.000	0.001
2.5.1.78|g__Parabacteroides.s__Parabacteroides_johnsonii	+	1.76E-01	0.000	0.000	0.000	0.001	0.002
2.5.1.78|g__Paraprevotella.s__Paraprevotella_clara	+	9.34E-03	0.000	0.000	0.000	0.001	0.004
2.5.1.78|g__Paraprevotella.s__Paraprevotella_xylaniphila	+	9.90E-01	0.000	0.000	0.000	0.000	0.001
2.5.1.78|g__Parasutterella.s__Parasutterella_excrementihominis	-	5.28E-01	0.000	0.000	0.000	0.003	0.002
2.5.1.78|g__Peptostreptococcaceae_unclassified.s__Clostridium_hiranonis	-	3.30E-01	0.000	0.000	0.000	0.000	0.000
2.5.1.78|g__Phascolarctobacterium.s__Phascolarctobacterium_succinatutens	+	3.05E-01	0.000	0.000	0.000	0.002	0.003
2.5.1.78|g__Prevotella.s__Prevotella_bivia	+	1.00E+00	0.000	0.000	0.000	0.000	0.000
2.5.1.78|g__Prevotella.s__Prevotella_copri	+	1.05E-01	0.000	0.000	0.000	0.012	0.012
2.5.1.78|g__Prevotella.s__Prevotella_corporis	-	3.30E-01	0.000	0.000	0.000	0.000	0.000
2.5.1.78|g__Prevotella.s__Prevotella_denticola	+	3.30E-01	0.000	0.000	0.000	0.000	0.000
2.5.1.78|g__Prevotella.s__Prevotella_disiens	+	3.30E-01	0.000	0.000	0.000	0.000	0.000
2.5.1.78|g__Prevotella.s__Prevotella_multisaccharivorax	-	3.30E-01	0.000	0.000	0.000	0.000	0.000
2.5.1.78|g__Prevotella.s__Prevotella_sp_109	-	8.20E-02	0.000	0.000	0.000	0.001	0.000
2.5.1.78|g__Prevotella.s__Prevotella_sp_AM42_24	-	3.30E-01	0.000	0.000	0.000	0.002	0.000
2.5.1.78|g__Prevotella.s__Prevotella_stercorea	-	7.16E-01	0.000	0.000	0.000	0.008	0.005
2.5.1.78|g__Pseudocitrobacter.s__Pseudocitrobacter_faecalis	+	3.30E-01	0.000	0.000	0.000	0.000	0.000
2.5.1.78|g__Pyramidobacter.s__Pyramidobacter_piscolens	+	2.53E-02	0.000	0.000	0.000	0.000	0.000
2.5.1.78|g__Raoultella.s__Raoultella_ornithinolytica	-	3.30E-01	0.000	0.000	0.000	0.000	0.000
2.5.1.78|g__Roseburia.s__Roseburia_intestinalis	-	3.28E-01	0.000	0.000	0.000	0.001	0.001
2.5.1.78|g__Roseburia.s__Roseburia_inulinivorans	+	3.65E-01	0.000	0.000	0.000	0.001	0.001
2.5.1.78|g__Ruminococcaceae_unclassified.s__Ruminococcaceae_bacteriun	-	3.30E-01	0.000	0.000	0.000	0.000	0.000
2.5.1.78|g__Ruminococcaceae_unclassified.s__Ruminococcaceae_bacteriun	+	4.28E-02	0.000	0.000	0.000	0.000	0.001
2.5.1.78|g__Ruminococcus.s__Ruminococcus_callidus	+	4.66E-01	0.000	0.000	0.000	0.000	0.001
2.5.1.78|g__Ruminococcus.s__Ruminococcus_sp_AF31_8BH	+	9.83E-01	0.000	0.000	0.000	0.000	0.000
2.5.1.78|g__Sanguibacteroides.s__Sanguibacteroides_justesenii	+	5.49E-01	0.000	0.000	0.000	0.000	0.000
2.5.1.78|g__Senegalimassilia.s__Senegalimassilia_anaerobia	+	8.20E-02	0.000	0.000	0.000	0.000	0.000
2.5.1.78|g__Slackia.s__Slackia_isoflavoniconvertens	+	1.60E-01	0.000	0.000	0.000	0.000	0.000
2.5.1.78|g__Succinatimonas.s__Succinatimonas_hippei	-	3.30E-01	0.000	0.000	0.000	0.000	0.000
2.5.1.78|g__Sutterella.s__Sutterella_parvirubra	-	3.30E-01	0.000	0.000	0.000	0.000	0.000
2.5.1.78|g__Sutterella.s__Sutterella_wadsworthensis	+	2.24E-01	0.000	0.000	0.000	0.001	0.002
2.5.1.78|g__Synergistes.s__Synergistes_jonesii	+	3.30E-01	0.000	0.000	0.000	0.000	0.000
2.5.1.78|g__Turicimonas.s__Turicimonas_muris	+	5.49E-01	0.000	0.000	0.000	0.000	0.000
2.5.1.78|g__Veillonella.s__Veillonella_atypica	+	6.26E-01	0.000	0.000	0.000	0.001	0.001
2.5.1.78|g__Veillonella.s__Veillonella_dispar	+	2.09E-01	0.000	0.000	0.000	0.003	0.000
2.5.1.78|g__Veillonella.s__Veillonella_infantium	+	6.94E-01	0.000	0.000	0.000	0.001	0.001
2.5.1.78|g__Veillonella.s__Veillonella_parvula	+	6.82E-01	0.000	0.000	0.000	0.002	0.001
2.5.1.78|g__Veillonella.s__Veillonella_rogosae	+	1.05E-01	0.000	0.000	0.000	0.001	0.000
2.5.1.78|g__Veillonella.s__Veillonella_tobetsuensis	+	3.33E-01	0.000	0.000	0.000	0.000	0.000
2.5.1.78|g__Victivallales_unclassified.s__Victivallales_bacterium_CCUG_4	+	2.96E-01	0.000	0.000	0.000	0.000	0.001
2.5.1.78|g__Victivallis.s__Victivallis_vadensis	+	1.60E-01	0.000	0.000	0.000	0.000	0.000
2.5.1.78|g__Bacteroides.s__Bacteroides_intestinalis	+	2.87E-02	0.000	0.004	0.004	0.001	0.009
2.5.1.78|g__Faecalibacterium.s__Faecalibacterium_prausnitzii	+	7.80E-01	1.192	1.195	0.004	0.016	0.017
2.5.1.78|g__Blautia.s__Ruminococcus_torques	+	6.95E-01	0.000	0.010	0.010	0.002	0.003
2.5.1.78|g__Bacteroides.s__Bacteroides_nordii	-	7.21E-01	0.134	0.145	0.011	0.004	0.005
2.5.1.78|g__Blautia.s__Blautia_obeum	+	9.16E-01	0.011	0.042	0.031	0.001	0.001
2.5.1.78|g__Dorea.s__Dorea_longicatena	+	4.70E-01	0.000	0.049	0.049	0.001	0.001
2.5.1.78|g__Lachnospiraceae_unclassified.s__Eubacterium_rectale	-	6.69E-01	0.088	0.141	0.054	0.004	0.003
2.5.1.78|g__Alistipes.s__Alistipes_indistinctus	+	8.64E-03	0.000	0.060	0.060	0.002	0.003
2.5.1.78|g__Bacteroides.s__Bacteroides_xylanisolvens	+	9.57E-01	0.368	0.479	0.111	0.018	0.015
2.5.1.78|g__Bacteroides.s__Bacteroides_thetaiotaomicron	+	8.81E-01	1.890	2.034	0.144	0.026	0.034
2.5.1.78|g__Bilophila.s__Bilophila_wadsworthia	+	1.85E-01	0.021	0.179	0.157	0.001	0.002
2.5.1.78|g__Parabacteroides.s__Parabacteroides_distasonis	+	5.54E-01	1.571	1.764	0.193	0.024	0.018
2.5.1.78|g__Odoribacter.s__Odoribacter_splanchnicus	-	9.39E-01	0.018	0.218	0.200	0.005	0.004
2.5.1.78|g__Alistipes.s__Alistipes_onderdonkii	+	1.56E-01	0.026	0.247	0.220	0.004	0.013

	2.5.1.78|g__Bacteroides.s__Bacteroides_dorei	+	8.33E-01	0.365	0.653	0.288	0.042	0.029
	2.5.1.78|g__Phascolarctobacterium.s__Phascolarctobacterium_faecium	+	4.60E-01	0.631	0.988	0.357	0.015	0.016
	2.5.1.78|g__Alistipes.s__Alistipes_finegoldii	+	5.88E-02	0.127	0.549	0.421	0.007	0.024
	2.5.1.78|g__Parabacteroides.s__Parabacteroides_merdae	+	3.74E-01	0.130	0.609	0.479	0.010	0.013
	2.5.1.78|g__Eubacterium.s__Eubacterium_eligens	+	5.95E-02	0.306	0.808	0.502	0.007	0.017
	2.5.1.78|g__Bacteroides.s__Bacteroides_cellulosilyticus	+	4.64E-01	2.153	2.680	0.527	0.024	0.037
	2.5.1.78|g__Bacteroides.s__Bacteroides_caccae	+	4.94E-01	0.296	1.048	0.752	0.016	0.023
	2.5.1.78|g__Bacteroides.s__Bacteroides_stercoris	+	9.14E-01	0.255	1.375	1.120	0.065	0.040
	2.5.1.78|g__Alistipes.s__Alistipes_shahii	+	2.34E-02	0.346	1.559	1.213	0.010	0.019
	2.5.1.78|unclassified	+	2.02E-01	7.027	8.341	1.314	0.117	0.141
	2.5.1.78|g__Bacteroides.s__Bacteroides_uniformis	+	2.01E-01	9.144	11.935	2.792	0.076	0.113
	2.5.1.78|g__Alistipes.s__Alistipes_putredinis	+	7.23E-02	1.587	5.200	3.613	0.035	0.058
China2	2.5.1.78	-	4.67E-02	142.906	133.385	-9.521	1.000	1.000
	2.5.1.78|g__Bacteroides.s__Bacteroides_vulgatus	-	7.85E-02	11.260	5.356	-5.904	0.127	0.077
	2.5.1.78|g__Faecalibacterium.s__Faecalibacterium_prausnitzii	-	2.72E-01	7.673	5.042	-2.631	0.064	0.055
	2.5.1.78|g__Phascolarctobacterium.s__Phascolarctobacterium_faecium	-	5.39E-01	1.097	0.340	-0.757	0.009	0.009
	2.5.1.78|g__Roseburia.s__Roseburia_faecis	-	1.10E-01	0.832	0.105	-0.728	0.019	0.008
	2.5.1.78|g__Bacteroides.s__Bacteroides_xylanisolvens	-	2.18E-02	0.378	0.127	-0.251	0.014	0.008
	2.5.1.78|g__Clostridium.s__Clostridium_sp_AM22_11AC	-	2.76E-01	0.630	0.380	-0.251	0.007	0.006
	2.5.1.78|g__Bacteroides.s__Bacteroides_cellulosilyticus	-	6.73E-01	0.185	0.043	-0.142	0.008	0.011
	2.5.1.78|g__Agathobaculum.s__Agathobaculum_butyriciproducens	-	1.08E-01	0.062	0.000	-0.062	0.002	0.001
	2.5.1.78|g__Lachnospiraceae_unclassified.s__Eubacterium_rectale	-	8.62E-01	0.231	0.196	-0.034	0.005	0.004
	2.5.1.78|g__Blautia.s__Ruminococcus_torques	-	7.26E-01	0.189	0.173	-0.016	0.005	0.004
	2.5.1.78|g__Dorea.s__Dorea_formicigenerans	-	9.35E-01	0.053	0.046	-0.007	0.000	0.001
	2.5.1.78|g__Bacteroides.s__Bacteroides_fragilis	+	9.75E-01	0.027	0.021	-0.007	0.004	0.005
	2.5.1.78|g__Anaerostipes.s__Anaerostipes_hadrus	-	7.20E-01	0.147	0.146	-0.001	0.003	0.003
	2.5.1.78|g__Acidaminococcus.s__Acidaminococcus_intestini	-	5.69E-01	0.000	0.000	0.000	0.001	0.001
	2.5.1.78|g__Adlercreutzia.s__Adlercreutzia_equolifaciens	+	2.19E-01	0.000	0.000	0.000	0.000	0.000
	2.5.1.78|g__Akkermansia.s__Akkermansia_muciniphila	+	4.86E-01	0.000	0.000	0.000	0.001	0.004
	2.5.1.78|g__Alcaligenes.s__Alcaligenes_faecalis	+	3.30E-01	0.000	0.000	0.000	0.000	0.001
	2.5.1.78|g__Alistipes.s__Alistipes_indistinctus	+	3.99E-03	0.000	0.000	0.000	0.000	0.001
	2.5.1.78|g__Alistipes.s__Alistipes_inops	+	5.89E-01	0.000	0.000	0.000	0.000	0.000
	2.5.1.78|g__Alistipes.s__Alistipes_timonensis	+	1.00E+00	0.000	0.000	0.000	0.000	0.000
	2.5.1.78|g__Allisonella.s__Allisonella_histaminiformans	+	2.24E-02	0.000	0.000	0.000	0.000	0.000
	2.5.1.78|g__Anaeromassilibacillus.s__Anaeromassilibacillus_sp_An250	+	4.27E-02	0.000	0.000	0.000	0.000	0.000
	2.5.1.78|g__Anaerotignum.s__Anaerotignum_lactatifermentans	-	1.60E-01	0.000	0.000	0.000	0.000	0.000
	2.5.1.78|g__Asaccharobacter.s__Asaccharobacter_celatus	+	2.42E-01	0.000	0.000	0.000	0.000	0.000
	2.5.1.78|g__Atlantibacter.s__Atlantibacter_hermannii	-	3.30E-01	0.000	0.000	0.000	0.000	0.000
	2.5.1.78|g__Bacteroides.s__Bacteroides_clarus	-	5.65E-01	0.000	0.000	0.000	0.001	0.004
	2.5.1.78|g__Bacteroides.s__Bacteroides_coprocola	-	1.34E-01	0.000	0.000	0.000	0.025	0.007
	2.5.1.78|g__Bacteroides.s__Bacteroides_coprophilus	+	4.94E-01	0.000	0.000	0.000	0.004	0.004
	2.5.1.78|g__Bacteroides.s__Bacteroides_eggerthii	-	2.08E-01	0.000	0.000	0.000	0.008	0.002
	2.5.1.78|g__Bacteroides.s__Bacteroides_faecichinchillae	+	3.93E-01	0.000	0.000	0.000	0.000	0.000
	2.5.1.78|g__Bacteroides.s__Bacteroides_faecis	+	3.81E-01	0.000	0.000	0.000	0.000	0.002
	2.5.1.78|g__Bacteroides.s__Bacteroides_finegoldii	-	1.07E-01	0.000	0.000	0.000	0.010	0.001
	2.5.1.78|g__Bacteroides.s__Bacteroides_fluxus	+	3.30E-01	0.000	0.000	0.000	0.000	0.000
	2.5.1.78|g__Bacteroides.s__Bacteroides_intestinalis	+	4.50E-01	0.000	0.000	0.000	0.001	0.002
	2.5.1.78|g__Bacteroides.s__Bacteroides_massiliensis	-	4.80E-01	0.000	0.000	0.000	0.017	0.004
	2.5.1.78|g__Bacteroides.s__Bacteroides_nordii	+	2.78E-01	0.000	0.000	0.000	0.001	0.001
	2.5.1.78|g__Bacteroides.s__Bacteroides_oleiciplenus	-	1.60E-01	0.000	0.000	0.000	0.000	0.000
	2.5.1.78|g__Bacteroides.s__Bacteroides_plebeius	-	4.28E-01	0.000	0.000	0.000	0.083	0.032
	2.5.1.78|g__Bacteroides.s__Bacteroides_salyersiae	-	5.26E-01	0.000	0.000	0.000	0.001	0.001
	2.5.1.78|g__Bacteroides.s__Bacteroides_sartorii	-	3.30E-01	0.000	0.000	0.000	0.000	0.000
	2.5.1.78|g__Bacteroides.s__Bacteroides_sp_OM08_11	-	3.30E-01	0.000	0.000	0.000	0.000	0.000
	2.5.1.78|g__Bacteroides.s__Bacteroides_stercorirosoris	+	5.89E-01	0.000	0.000	0.000	0.000	0.000
	2.5.1.78|g__Barnesiella.s__Barnesiella_intestinihominis	+	7.14E-01	0.000	0.000	0.000	0.004	0.005
	2.5.1.78|g__Blautia.s__Blautia_hansenii	-	1.75E-01	0.000	0.000	0.000	0.001	0.001
	2.5.1.78|g__Butyricimonas.s__Butyricimonas_synergistica	+	7.02E-01	0.000	0.000	0.000	0.000	0.000
	2.5.1.78|g__Butyricimonas.s__Butyricimonas_virosa	+	1.11E-01	0.000	0.000	0.000	0.001	0.002
	2.5.1.78|g__Butyrivibrio.s__Butyrivibrio_crossotus	+	3.30E-01	0.000	0.000	0.000	0.000	0.007
	2.5.1.78|g__Campylobacter.s__Campylobacter_concisus	-	5.49E-01	0.000	0.000	0.000	0.000	0.000
	2.5.1.78|g__Campylobacter.s__Campylobacter_hominis	+	3.30E-01	0.000	0.000	0.000	0.000	0.000
	2.5.1.78|g__Catenibacterium.s__Catenibacterium_mitsuokai	+	5.49E-01	0.000	0.000	0.000	0.000	0.000
	2.5.1.78|g__Cetobacterium.s__Cetobacterium_somerae	-	3.30E-01	0.000	0.000	0.000	0.000	0.000
	2.5.1.78|g__Chryseobacterium.s__Chryseobacterium_sp_VAUSW3	+	3.30E-01	0.000	0.000	0.000	0.000	0.000
	2.5.1.78|g__Citrobacter.s__Citrobacter_amalonaticus	-	3.30E-01	0.000	0.000	0.000	0.000	0.000
	2.5.1.78|g__Citrobacter.s__Citrobacter_braakii	+	9.86E-01	0.000	0.000	0.000	0.000	0.000
	2.5.1.78|g__Citrobacter.s__Citrobacter_freundii	+	7.19E-01	0.000	0.000	0.000	0.000	0.000
	2.5.1.78|g__Citrobacter.s__Citrobacter_portucalensis	-	5.41E-01	0.000	0.000	0.000	0.000	0.004
	2.5.1.78|g__Citrobacter.s__Citrobacter_werkmanii	+	1.00E+00	0.000	0.000	0.000	0.000	0.001
	2.5.1.78|g__Citrobacter.s__Citrobacter_youngae	+	9.73E-01	0.000	0.000	0.000	0.000	0.003
	2.5.1.78|g__Cloacibacillus.s__Cloacibacillus_porcorum	+	5.69E-01	0.000	0.000	0.000	0.000	0.000

2.5.1.78|g__Clostridioides.s__Clostridioides_difficile	+	1.25E-01	0.000	0.000	0.000	0.001	0.001
2.5.1.78|g__Clostridium.s__Clostridium_butyricum	+	3.30E-01	0.000	0.000	0.000	0.000	0.000
2.5.1.78|g__Clostridium.s__Clostridium_celatum	-	1.00E+00	0.000	0.000	0.000	0.000	0.000
2.5.1.78|g__Clostridium.s__Clostridium_disporicum	-	5.04E-01	0.000	0.000	0.000	0.001	0.000
2.5.1.78|g__Clostridium.s__Clostridium_perfringens	-	4.27E-02	0.000	0.000	0.000	0.000	0.000
2.5.1.78|g__Clostridium.s__Clostridium_sp_AF36_4	+	5.80E-02	0.000	0.000	0.000	0.000	0.001
2.5.1.78|g__Comamonas.s__Comamonas_aquatica	+	3.30E-01	0.000	0.000	0.000	0.000	0.000
2.5.1.78|g__Comamonas.s__Comamonas_kerstersii	-	1.00E+00	0.000	0.000	0.000	0.000	0.000
2.5.1.78|g__Comamonas.s__Comamonas_terrigena	+	3.30E-01	0.000	0.000	0.000	0.000	0.000
2.5.1.78|g__Comamonas.s__Comamonas_testosteroni	+	3.30E-01	0.000	0.000	0.000	0.000	0.000
2.5.1.78|g__Comamonas.s__Comamonas_thiooxydans	+	3.30E-01	0.000	0.000	0.000	0.000	0.000
2.5.1.78|g__Coprobacillus.s__Coprobacillus_cateniformis	+	9.87E-02	0.000	0.000	0.000	0.000	0.002
2.5.1.78|g__Coprobacter.s__Coprobacter_fastidiosus	+	5.83E-01	0.000	0.000	0.000	0.001	0.001
2.5.1.78|g__Coprobacter.s__Coprobacter_secundus	+	9.65E-01	0.000	0.000	0.000	0.000	0.000
2.5.1.78|g__Coprococcus.s__Coprococcus_catus	+	1.28E-01	0.000	0.000	0.000	0.000	0.001
2.5.1.78|g__Coprococcus.s__Coprococcus_comes	+	4.68E-01	0.000	0.000	0.000	0.001	0.001
2.5.1.78|g__Coprococcus.s__Coprococcus_eutactus	+	3.39E-01	0.000	0.000	0.000	0.002	0.003
2.5.1.78|g__Cronobacter.s__Cronobacter_dublinensis	-	3.30E-01	0.000	0.000	0.000	0.000	0.000
2.5.1.78|g__Desulfovibrio.s__Desulfovibrio_fairfieldensis	+	1.00E+00	0.000	0.000	0.000	0.000	0.000
2.5.1.78|g__Desulfovibrio.s__Desulfovibrio_piger	+	2.72E-01	0.000	0.000	0.000	0.000	0.001
2.5.1.78|g__Desulfovibrio.s__Desulfovibrio_sp_AM18_2	+	8.19E-02	0.000	0.000	0.000	0.000	0.000
2.5.1.78|g__Desulfovibrionaceae_unclassified.s__Desulfovibrionaceae_bact	+	1.60E-01	0.000	0.000	0.000	0.000	0.000
2.5.1.78|g__Dialister.s__Dialister_invisus	-	9.34E-01	0.000	0.000	0.000	0.002	0.007
2.5.1.78|g__Dialister.s__Dialister_succinatiphilus	+	9.90E-01	0.000	0.000	0.000	0.002	0.003
2.5.1.78|g__Dorea.s__Dorea_sp_OM02_2LB	-	6.21E-01	0.000	0.000	0.000	0.000	0.000
2.5.1.78|g__Eggerthella.s__Eggerthella_lenta	+	3.78E-01	0.000	0.000	0.000	0.001	0.002
2.5.1.78|g__Eisenbergiella.s__Eisenbergiella_tayi	+	3.30E-01	0.000	0.000	0.000	0.000	0.000
2.5.1.78|g__Enterobacter.s__Enterobacter_bugandensis	-	6.17E-01	0.000	0.000	0.000	0.000	0.000
2.5.1.78|g__Enterobacter.s__Enterobacter_cloacae	+	8.49E-01	0.000	0.000	0.000	0.000	0.001
2.5.1.78|g__Enterobacter.s__Enterobacter_mori	-	1.60E-01	0.000	0.000	0.000	0.000	0.000
2.5.1.78|g__Enterococcus.s__Enterococcus_hirae	+	3.30E-01	0.000	0.000	0.000	0.000	0.000
2.5.1.78|g__Erysipelatoclostridium.s__Clostridium_innocuum	-	6.89E-01	0.000	0.000	0.000	0.000	0.000
2.5.1.78|g__Erysipelatoclostridium.s__Clostridium_spiroforme	+	1.80E-01	0.000	0.000	0.000	0.000	0.001
2.5.1.78|g__Erysipelotrichaceae_unclassified.s__Erysipelotrichaceae_bacteri	-	1.60E-01	0.000	0.000	0.000	0.000	0.000
2.5.1.78|g__Escherichia.s__Escherichia_fergusonii	+	1.16E-01	0.000	0.000	0.000	0.000	0.000
2.5.1.78|g__Eubacterium.s__Eubacterium_ramulus	+	6.29E-01	0.000	0.000	0.000	0.000	0.001
2.5.1.78|g__Eubacterium.s__Eubacterium_sp_AF17_7	+	7.02E-01	0.000	0.000	0.000	0.000	0.000
2.5.1.78|g__Eubacterium.s__Eubacterium_sp_AM18_10LB_B	-	4.05E-01	0.000	0.000	0.000	0.000	0.001
2.5.1.78|g__Faecalicatena.s__Faecalicatena_contorta	-	6.57E-01	0.000	0.000	0.000	0.000	0.000
2.5.1.78|g__Firmicutes_unclassified.s__Firmicutes_bacterium_AM10_47	-	2.68E-01	0.000	0.000	0.000	0.001	0.001
2.5.1.78|g__Flavonifractor.s__Flavonifractor_plautii	+	1.72E-01	0.000	0.000	0.000	0.000	0.000
2.5.1.78|g__Flavonifractor.s__Flavonifractor_sp_An10	+	3.30E-01	0.000	0.000	0.000	0.000	0.000
2.5.1.78|g__Fusobacterium.s__Fusobacterium_hwasookii	-	3.30E-01	0.000	0.000	0.000	0.000	0.000
2.5.1.78|g__Fusobacterium.s__Fusobacterium_mortiferum	-	6.89E-01	0.000	0.000	0.000	0.002	0.003
2.5.1.78|g__Fusobacterium.s__Fusobacterium_nucleatum	+	1.00E+00	0.000	0.000	0.000	0.000	0.000
2.5.1.78|g__Fusobacterium.s__Fusobacterium_periodonticum	-	4.27E-02	0.000	0.000	0.000	0.000	0.000
2.5.1.78|g__Fusobacterium.s__Fusobacterium_ulcerans	+	5.69E-01	0.000	0.000	0.000	0.000	0.000
2.5.1.78|g__Fusobacterium.s__Fusobacterium_varium	-	1.60E-01	0.000	0.000	0.000	0.001	0.000
2.5.1.78|g__Haemophilus.s__Haemophilus_influenzae	+	3.30E-01	0.000	0.000	0.000	0.000	0.000
2.5.1.78|g__Haemophilus.s__Haemophilus_parainfluenzae	-	7.34E-01	0.000	0.000	0.000	0.002	0.002
2.5.1.78|g__Haemophilus.s__Haemophilus_paraphrohaemolyticus	-	3.30E-01	0.000	0.000	0.000	0.000	0.000
2.5.1.78|g__Hungatella.s__Hungatella_hathewayi	-	7.71E-01	0.000	0.000	0.000	0.000	0.000
2.5.1.78|g__Intestinibacter.s__Intestinibacter_bartlettii	+	5.65E-01	0.000	0.000	0.000	0.000	0.000
2.5.1.78|g__Klebsiella.s__Klebsiella_aerogenes	+	4.23E-01	0.000	0.000	0.000	0.000	0.002
2.5.1.78|g__Klebsiella.s__Klebsiella_grimontii	+	1.60E-01	0.000	0.000	0.000	0.000	0.000
2.5.1.78|g__Klebsiella.s__Klebsiella_michiganensis	-	6.38E-01	0.000	0.000	0.000	0.000	0.000
2.5.1.78|g__Klebsiella.s__Klebsiella_oxytoca	-	5.32E-01	0.000	0.000	0.000	0.000	0.000
2.5.1.78|g__Kluyvera.s__Kluyvera_ascorbata	+	3.30E-01	0.000	0.000	0.000	0.000	0.000
2.5.1.78|g__Kluyvera.s__Kluyvera_cryocrescens	+	3.30E-01	0.000	0.000	0.000	0.000	0.000
2.5.1.78|g__Kluyvera.s__Kluyvera_georgiana	+	5.69E-01	0.000	0.000	0.000	0.000	0.000
2.5.1.78|g__Lachnoclostridium.s__Clostridium_aldenense	+	2.49E-02	0.000	0.000	0.000	0.000	0.001
2.5.1.78|g__Lachnoclostridium.s__Clostridium_bolteae	+	1.97E-01	0.000	0.000	0.000	0.000	0.001
2.5.1.78|g__Lachnoclostridium.s__Clostridium_citroniae	+	9.20E-03	0.000	0.000	0.000	0.000	0.000
2.5.1.78|g__Lachnoclostridium.s__Clostridium_clostridioforme	+	2.44E-01	0.000	0.000	0.000	0.001	0.000
2.5.1.78|g__Lachnoclostridium.s__Clostridium_symbiosum	+	6.21E-01	0.000	0.000	0.000	0.000	0.000
2.5.1.78|g__Lachnospira.s__Lachnospira_pectinoschiza	-	7.39E-01	0.000	0.000	0.000	0.006	0.003
2.5.1.78|g__Lachnospiraceae_unclassified.s__Lachnospiraceae_bacterium_(	+	3.30E-01	0.000	0.000	0.000	0.000	0.000
2.5.1.78|g__Lactobacillus.s__Lactobacillus_fermentum	+	9.90E-01	0.000	0.000	0.000	0.000	0.000
2.5.1.78|g__Lactobacillus.s__Lactobacillus_plantarum	-	3.30E-01	0.000	0.000	0.000	0.000	0.000
2.5.1.78|g__Lactobacillus.s__Lactobacillus_rogosae	-	9.17E-01	0.000	0.000	0.000	0.006	0.003
2.5.1.78|g__Leclercia.s__Leclercia_adecarboxylata	+	9.77E-01	0.000	0.000	0.000	0.000	0.000
2.5.1.78|g__Lelliottia.s__Lelliottia_amnigena	-	3.13E-01	0.000	0.000	0.000	0.000	0.000

2.5.1.78|g__Lelliottia.s__Lelliottia_nimipressuralis	-	5.65E-01	0.000	0.000	0.000	0.001	0.001
2.5.1.78|g__Megamonas.s__Megamonas_funiformis	-	8.72E-01	0.000	0.000	0.000	0.017	0.007
2.5.1.78|g__Megamonas.s__Megamonas_hypermegale	-	9.03E-01	0.000	0.000	0.000	0.006	0.004
2.5.1.78|g__Megamonas.s__Megamonas_rupellensis	+	9.59E-01	0.000	0.000	0.000	0.007	0.003
2.5.1.78|g__Megasphaera.s__Megasphaera_elsdenii	+	3.30E-01	0.000	0.000	0.000	0.000	0.000
2.5.1.78|g__Megasphaera.s__Megasphaera_micronuciformis	+	2.96E-01	0.000	0.000	0.000	0.000	0.000
2.5.1.78|g__Megasphaera.s__Megasphaera_stantonii	+	3.30E-01	0.000	0.000	0.000	0.000	0.000
2.5.1.78|g__Mesosutterella.s__Mesosutterella_multiformis	-	5.26E-01	0.000	0.000	0.000	0.002	0.001
2.5.1.78|g__Methanobrevibacter.s__Methanobrevibacter_smithii	+	8.19E-02	0.000	0.000	0.000	0.000	0.000
2.5.1.78|g__Mitsuokella.s__Mitsuokella_jalaludinii	+	3.30E-01	0.000	0.000	0.000	0.000	0.000
2.5.1.78|g__Mitsuokella.s__Mitsuokella_multacida	+	8.19E-02	0.000	0.000	0.000	0.000	0.001
2.5.1.78|g__Morganella.s__Morganella_morganii	+	3.30E-01	0.000	0.000	0.000	0.000	0.003
2.5.1.78|g__Ochrobactrum.s__Ochrobactrum_lupini	+	3.30E-01	0.000	0.000	0.000	0.000	0.000
2.5.1.78|g__Oxalobacter.s__Oxalobacter_formigenes	+	8.07E-03	0.000	0.000	0.000	0.000	0.000
2.5.1.78|g__Pantoea.s__Pantoea_dispersa	+	3.30E-01	0.000	0.000	0.000	0.000	0.000
2.5.1.78|g__Pantoea.s__Pantoea_sesami	-	4.68E-01	0.000	0.000	0.000	0.000	0.000
2.5.1.78|g__Parabacteroides.s__Parabacteroides_chinchillae	+	3.30E-01	0.000	0.000	0.000	0.000	0.000
2.5.1.78|g__Parabacteroides.s__Parabacteroides_goldsteinii	+	2.80E-01	0.000	0.000	0.000	0.000	0.002
2.5.1.78|g__Parabacteroides.s__Parabacteroides_gordonii	+	1.60E-01	0.000	0.000	0.000	0.000	0.001
2.5.1.78|g__Parabacteroides.s__Parabacteroides_johnsonii	+	6.92E-01	0.000	0.000	0.000	0.001	0.002
2.5.1.78|g__Paraprevotella.s__Paraprevotella_clara	+	8.94E-01	0.000	0.000	0.000	0.002	0.001
2.5.1.78|g__Paraprevotella.s__Paraprevotella_xylaniphila	-	3.30E-01	0.000	0.000	0.000	0.000	0.000
2.5.1.78|g__Pedobacter.s__Pedobacter_himalayensis	-	8.69E-01	0.000	0.000	0.000	0.000	0.000
2.5.1.78|g__Phascolarctobacterium.s__Phascolarctobacterium_succinatutens	+	9.77E-01	0.000	0.000	0.000	0.002	0.002
2.5.1.78|g__Prevotella.s__Prevotella_copri	+	3.05E-01	0.000	0.000	0.000	0.012	0.019
2.5.1.78|g__Prevotella.s__Prevotella_corporis	+	3.30E-01	0.000	0.000	0.000	0.000	0.000
2.5.1.78|g__Prevotella.s__Prevotella_sp_109	+	5.89E-01	0.000	0.000	0.000	0.003	0.003
2.5.1.78|g__Prevotella.s__Prevotella_sp_AM42_24	-	9.90E-01	0.000	0.000	0.000	0.004	0.002
2.5.1.78|g__Prevotella.s__Prevotella_stercorea	-	9.90E-01	0.000	0.000	0.000	0.009	0.005
2.5.1.78|g__Prevotella.s__Prevotella_timonensis	+	3.30E-01	0.000	0.000	0.000	0.000	0.000
2.5.1.78|g__Proteus.s__Proteus_hauseri	-	3.30E-01	0.000	0.000	0.000	0.000	0.000
2.5.1.78|g__Proteus.s__Proteus_mirabilis	-	3.30E-01	0.000	0.000	0.000	0.000	0.000
2.5.1.78|g__Proteus.s__Proteus_penneri	-	3.30E-01	0.000	0.000	0.000	0.000	0.000
2.5.1.78|g__Proteus.s__Proteus_vulgaris	-	5.69E-01	0.000	0.000	0.000	0.000	0.000
2.5.1.78|g__Providencia.s__Providencia_alcalifaciens	+	3.30E-01	0.000	0.000	0.000	0.000	0.000
2.5.1.78|g__Providencia.s__Providencia_rettgeri	+	3.30E-01	0.000	0.000	0.000	0.000	0.007
2.5.1.78|g__Pyramidobacter.s__Pyramidobacter_piscolens	+	1.58E-01	0.000	0.000	0.000	0.000	0.000
2.5.1.78|g__Pyramidobacter.s__Pyramidobacter_sp_C12_8	+	3.30E-01	0.000	0.000	0.000	0.000	0.000
2.5.1.78|g__Raoultella.s__Raoultella_ornithinolytica	+	5.89E-01	0.000	0.000	0.000	0.000	0.000
2.5.1.78|g__Raoultella.s__Raoultella_planticola	+	5.49E-01	0.000	0.000	0.000	0.000	0.000
2.5.1.78|g__Roseburia.s__Roseburia_intestinalis	+	9.74E-01	0.000	0.000	0.000	0.004	0.003
2.5.1.78|g__Roseburia.s__Roseburia_inulinivorans	-	2.21E-01	0.000	0.000	0.000	0.001	0.001
2.5.1.78|g__Rothia.s__Rothia_mucilaginosa	-	3.30E-01	0.000	0.000	0.000	0.000	0.000
2.5.1.78|g__Ruminococcaceae_unclassified.s__Ruminococcaceae_bacteriun	+	5.89E-01	0.000	0.000	0.000	0.000	0.000
2.5.1.78|g__Ruminococcaceae_unclassified.s__Ruminococcaceae_bacteriun	+	2.24E-02	0.000	0.000	0.000	0.000	0.000
2.5.1.78|g__Ruminococcus.s__Ruminococcus_callidus	+	7.82E-01	0.000	0.000	0.000	0.001	0.001
2.5.1.78|g__Ruminococcus.s__Ruminococcus_sp_AF31_8BH	-	6.06E-01	0.000	0.000	0.000	0.000	0.000
2.5.1.78|g__Sanguibacteroides.s__Sanguibacteroides_justesenii	+	3.30E-01	0.000	0.000	0.000	0.000	0.000
2.5.1.78|g__Staphylococcus.s__Staphylococcus_epidermidis	-	3.30E-01	0.000	0.000	0.000	0.000	0.000
2.5.1.78|g__Staphylococcus.s__Staphylococcus_warneri	-	3.30E-01	0.000	0.000	0.000	0.000	0.000
2.5.1.78|g__Stomatobaculum.s__Stomatobaculum_longum	-	3.30E-01	0.000	0.000	0.000	0.000	0.000
2.5.1.78|g__Streptococcus.s__Streptococcus_gallolyticus	+	3.30E-01	0.000	0.000	0.000	0.000	0.000
2.5.1.78|g__Streptococcus.s__Streptococcus_infantarius	-	3.30E-01	0.000	0.000	0.000	0.000	0.000
2.5.1.78|g__Streptococcus.s__Streptococcus_macedonicus	-	1.00E+00	0.000	0.000	0.000	0.000	0.000
2.5.1.78|g__Streptococcus.s__Streptococcus_mitis	-	8.19E-02	0.000	0.000	0.000	0.000	0.000
2.5.1.78|g__Streptococcus.s__Streptococcus_oralis	-	1.60E-01	0.000	0.000	0.000	0.000	0.000
2.5.1.78|g__Streptococcus.s__Streptococcus_pasteurianus	-	1.00E+00	0.000	0.000	0.000	0.000	0.000
2.5.1.78|g__Streptococcus.s__Streptococcus_pneumoniae	-	9.83E-01	0.000	0.000	0.000	0.000	0.000
2.5.1.78|g__Streptococcus.s__Streptococcus_pseudopneumoniae	-	3.30E-01	0.000	0.000	0.000	0.000	0.000
2.5.1.78|g__Sutterella.s__Sutterella_parvirubra	+	3.30E-01	0.000	0.000	0.000	0.000	0.000
2.5.1.78|g__Sutterella.s__Sutterella_wadsworthensis	+	2.89E-01	0.000	0.000	0.000	0.002	0.003
2.5.1.78|g__Veillonella.s__Veillonella_atypica	-	8.26E-01	0.000	0.000	0.000	0.001	0.001
2.5.1.78|g__Veillonella.s__Veillonella_dispar	+	9.95E-01	0.000	0.000	0.000	0.000	0.000
2.5.1.78|g__Veillonella.s__Veillonella_infantium	+	7.38E-01	0.000	0.000	0.000	0.000	0.001
2.5.1.78|g__Veillonella.s__Veillonella_parvula	-	9.39E-01	0.000	0.000	0.000	0.001	0.001
2.5.1.78|g__Veillonella.s__Veillonella_rogosae	-	1.61E-02	0.000	0.000	0.000	0.001	0.000
2.5.1.78|g__Veillonella.s__Veillonella_tobetsuensis	-	1.85E-01	0.000	0.000	0.000	0.000	0.000
2.5.1.78|g__Victivallales_unclassified.s__Victivallales_bacterium_CCUG_4	+	3.16E-03	0.000	0.000	0.000	0.000	0.001
2.5.1.78|g__Victivallis.s__Victivallis_vadensis	+	9.92E-01	0.000	0.000	0.000	0.000	0.001
2.5.1.78|g__Weissella.s__Weissella_confusa	-	3.30E-01	0.000	0.000	0.000	0.000	0.000
2.5.1.78|g__Yokenella.s__Yokenella_regensburgei	-	3.80E-01	0.000	0.000	0.000	0.000	0.000
2.5.1.78|g__Bacteroides.s__Bacteroides_dorei	-	9.71E-01	0.064	0.067	0.004	0.038	0.023

	2.5.1.78|g__Dorea.s__Dorea_longicatena	+	7.04E-01	0.202	0.208	0.005	0.002	0.002
	2.5.1.78|g__Parabacteroides.s__Parabacteroides_distasonis	+	7.42E-01	1.069	1.091	0.022	0.013	0.020
	2.5.1.78|g__Klebsiella.s__Klebsiella_variicola	+	2.40E-01	0.000	0.023	0.023	0.003	0.002
	2.5.1.78|g__Blautia.s__Blautia_obeum	+	8.10E-01	0.341	0.389	0.047	0.005	0.007
	2.5.1.78|g__Parasutterella.s__Parasutterella_excrementihominis	+	3.74E-01	0.000	0.054	0.054	0.002	0.002
	2.5.1.78|g__Blautia.s__Blautia_wexlerae	+	6.09E-01	0.208	0.283	0.075	0.006	0.008
	2.5.1.78|g__Bacteroides.s__Bacteroides_thetaiotaomicron	-	3.98E-01	0.560	0.668	0.108	0.013	0.009
	2.5.1.78|g__Klebsiella.s__Klebsiella_pneumoniae	+	2.04E-01	0.248	0.380	0.131	0.008	0.026
	2.5.1.78|g__Eubacterium.s__Eubacterium_eligens	+	7.70E-01	0.021	0.211	0.190	0.007	0.005
	2.5.1.78|g__Alistipes.s__Alistipes_onderdonkii	+	8.57E-02	0.000	0.202	0.202	0.002	0.004
	2.5.1.78|g__Bacteroides.s__Bacteroides_ovatus	-	9.92E-01	0.662	0.867	0.205	0.030	0.022
	2.5.1.78|g__Odoribacter.s__Odoribacter_splanchnicus	+	2.68E-01	0.149	0.388	0.239	0.002	0.003
	2.5.1.78|g__Bilophila.s__Bilophila_wadsworthia	+	8.48E-03	0.125	0.377	0.252	0.002	0.004
	2.5.1.78|g__Parabacteroides.s__Parabacteroides_merdae	+	2.24E-01	0.427	0.679	0.252	0.006	0.012
	2.5.1.78|g__Alistipes.s__Alistipes_finegoldii	+	7.08E-02	0.124	0.448	0.324	0.005	0.010
	2.5.1.78|g__Alistipes.s__Alistipes_shahii	+	5.75E-02	0.131	0.534	0.402	0.005	0.010
	2.5.1.78|g__Bacteroides.s__Bacteroides_caccae	+	4.58E-01	0.188	0.661	0.473	0.008	0.011
	2.5.1.78|g__Escherichia.s__Escherichia_coli	+	3.71E-01	0.509	1.026	0.517	0.035	0.048
	2.5.1.78|g__Bacteroides.s__Bacteroides_stercoris	+	6.99E-01	0.361	0.881	0.520	0.045	0.046
	2.5.1.78|g__Bacteroides.s__Bacteroides_uniformis	+	5.22E-01	3.826	5.216	1.390	0.056	0.074
	2.5.1.78|g__Alistipes.s__Alistipes_putredinis	+	2.73E-03	0.566	3.918	3.352	0.016	0.042
	2.5.1.78|unclassified	+	1.45E-01	11.646	15.560	3.914	0.148	0.204
Taiwan	2.5.1.78	-	1.82E-01	142.380	137.050	-5.330	1.000	1.000
	2.5.1.78|g__Bacteroides.s__Bacteroides_vulgatus	-	7.42E-01	6.772	5.704	-1.068	0.070	0.066
	2.5.1.78|unclassified	-	9.33E-01	14.644	13.802	-0.842	0.185	0.199
	2.5.1.78|g__Bacteroides.s__Bacteroides_plebeius	-	4.02E-01	0.986	0.281	-0.705	0.073	0.046
	2.5.1.78|g__Roseburia.s__Roseburia_faecis	-	4.00E-01	0.237	0.000	-0.237	0.004	0.005
	2.5.1.78|g__Lachnospiraceae_unclassified.s__Eubacterium_rectale	-	2.88E-02	0.366	0.148	-0.219	0.011	0.005
	2.5.1.78|g__Agathobaculum.s__Agathobaculum_butyriciproducens	-	7.99E-03	0.180	0.000	-0.180	0.003	0.002
	2.5.1.78|g__Escherichia.s__Escherichia_coli	-	1.93E-01	0.329	0.184	-0.145	0.030	0.010
	2.5.1.78|g__Dorea.s__Dorea_longicatena	-	8.83E-01	0.201	0.146	-0.056	0.002	0.004
	2.5.1.78|g__Blautia.s__Blautia_obeum	-	4.21E-01	0.124	0.074	-0.050	0.001	0.001
	2.5.1.78|g__Coprococcus.s__Coprococcus_comes	-	5.32E-01	0.047	0.000	-0.047	0.002	0.002
	2.5.1.78|g__Blautia.s__Ruminococcus_torques	-	9.40E-01	0.109	0.076	-0.033	0.008	0.006
	2.5.1.78|g__Clostridium.s__Clostridium_sp_AM22_11AC	-	4.02E-01	0.226	0.197	-0.029	0.003	0.003
	2.5.1.78|g__Acidaminococcus.s__Acidaminococcus_fermentans	+	1.96E-01	0.000	0.000	0.000	0.000	0.000
	2.5.1.78|g__Acidaminococcus.s__Acidaminococcus_intestini	-	8.76E-01	0.000	0.000	0.000	0.001	0.002
	2.5.1.78|g__Adlercreutzia.s__Adlercreutzia_equolifaciens	-	3.14E-01	0.000	0.000	0.000	0.000	0.000
	2.5.1.78|g__Aeromonas.s__Aeromonas_caviae	-	2.85E-01	0.000	0.000	0.000	0.000	0.000
	2.5.1.78|g__Aggregatibacter.s__Aggregatibacter_segnis	-	2.85E-01	0.000	0.000	0.000	0.000	0.000
	2.5.1.78|g__Akkermansia.s__Akkermansia_muciniphila	+	2.74E-01	0.000	0.000	0.000	0.006	0.008
	2.5.1.78|g__Alistipes.s__Alistipes_indistinctus	-	8.92E-01	0.000	0.000	0.000	0.002	0.004
	2.5.1.78|g__Alistipes.s__Alistipes_inops	+	6.76E-01	0.000	0.000	0.000	0.002	0.001
	2.5.1.78|g__Alistipes.s__Alistipes_timonensis	-	2.85E-01	0.000	0.000	0.000	0.000	0.000
	2.5.1.78|g__Allisonella.s__Allisonella_histaminiformans	-	8.72E-01	0.000	0.000	0.000	0.000	0.000
	2.5.1.78|g__Anaeroglobus.s__Anaeroglobus_geminatus	+	3.68E-01	0.000	0.000	0.000	0.000	0.000
	2.5.1.78|g__Anaeromassilibacillus.s__Anaeromassilibacillus_sp_An250	-	1.24E-01	0.000	0.000	0.000	0.000	0.000
	2.5.1.78|g__Anaerostipes.s__Anaerostipes_hadrus	+	2.12E-01	0.000	0.000	0.000	0.001	0.001
	2.5.1.78|g__Anaerotignum.s__Anaerotignum_lactatifermentans	-	8.32E-01	0.000	0.000	0.000	0.001	0.001
	2.5.1.78|g__Asaccharobacter.s__Asaccharobacter_celatus	-	6.09E-02	0.000	0.000	0.000	0.000	0.000
	2.5.1.78|g__Bacteroides.s__Bacteroides_clarus	+	9.28E-01	0.000	0.000	0.000	0.002	0.007
	2.5.1.78|g__Bacteroides.s__Bacteroides_coprocola	+	8.31E-01	0.000	0.000	0.000	0.027	0.026
	2.5.1.78|g__Bacteroides.s__Bacteroides_coprophilus	-	7.28E-01	0.000	0.000	0.000	0.006	0.005
	2.5.1.78|g__Bacteroides.s__Bacteroides_eggerthii	+	2.66E-01	0.000	0.000	0.000	0.007	0.008
	2.5.1.78|g__Bacteroides.s__Bacteroides_faecichinchillae	+	3.97E-01	0.000	0.000	0.000	0.000	0.000
	2.5.1.78|g__Bacteroides.s__Bacteroides_finegoldii	+	3.18E-01	0.000	0.000	0.000	0.005	0.006
	2.5.1.78|g__Bacteroides.s__Bacteroides_fluxus	-	9.26E-01	0.000	0.000	0.000	0.000	0.002
	2.5.1.78|g__Bacteroides.s__Bacteroides_fragilis	+	6.95E-01	0.000	0.000	0.000	0.003	0.004
	2.5.1.78|g__Bacteroides.s__Bacteroides_intestinalis	+	8.06E-03	0.000	0.000	0.000	0.002	0.009
	2.5.1.78|g__Bacteroides.s__Bacteroides_nordii	+	1.81E-01	0.000	0.000	0.000	0.000	0.001
	2.5.1.78|g__Bacteroides.s__Bacteroides_oleiciplenus	+	3.68E-01	0.000	0.000	0.000	0.000	0.000
	2.5.1.78|g__Bacteroides.s__Bacteroides_salyersiae	-	8.64E-01	0.000	0.000	0.000	0.004	0.003
	2.5.1.78|g__Bacteroides.s__Bacteroides_sartorii	+	8.11E-01	0.000	0.000	0.000	0.004	0.001
	2.5.1.78|g__Bacteroides.s__Bacteroides_sp_OM08_11	+	5.46E-01	0.000	0.000	0.000	0.000	0.000
	2.5.1.78|g__Barnesiella.s__Barnesiella_intestinihominis	+	1.26E-02	0.000	0.000	0.000	0.006	0.012
	2.5.1.78|g__Blautia.s__Blautia_hansenii	+	5.56E-01	0.000	0.000	0.000	0.000	0.000
	2.5.1.78|g__Butyricicoccus.s__Butyricicoccus_pullicaecorum	+	3.68E-01	0.000	0.000	0.000	0.000	0.000
	2.5.1.78|g__Butyricimonas.s__Butyricimonas_synergistica	+	6.70E-01	0.000	0.000	0.000	0.000	0.000
	2.5.1.78|g__Campylobacter.s__Campylobacter_concisus	-	2.85E-01	0.000	0.000	0.000	0.000	0.000
	2.5.1.78|g__Candidatus_Gastranaerophilales_unclassified.s__Candidatus_Ga	-	9.26E-01	0.000	0.000	0.000	0.000	0.000
	2.5.1.78|g__Catenibacterium.s__Catenibacterium_mitsuokai	-	5.99E-01	0.000	0.000	0.000	0.004	0.005
	2.5.1.78|g__Cetobacterium.s__Cetobacterium_somerae	+	1.96E-01	0.000	0.000	0.000	0.000	0.000

2.5.1.78|g__Citrobacter.s__Citrobacter_amalonaticus	-	4.71E-01	0.000	0.000	0.000	0.000	0.000
2.5.1.78|g__Citrobacter.s__Citrobacter_braakii	-	2.27E-01	0.000	0.000	0.000	0.000	0.000
2.5.1.78|g__Citrobacter.s__Citrobacter_farmeri	-	2.85E-01	0.000	0.000	0.000	0.000	0.000
2.5.1.78|g__Citrobacter.s__Citrobacter_freundii	-	1.25E-01	0.000	0.000	0.000	0.000	0.000
2.5.1.78|g__Citrobacter.s__Citrobacter_koseri	+	6.86E-01	0.000	0.000	0.000	0.000	0.000
2.5.1.78|g__Citrobacter.s__Citrobacter_portucalensis	+	9.59E-01	0.000	0.000	0.000	0.000	0.000
2.5.1.78|g__Citrobacter.s__Citrobacter_werkmanii	+	3.68E-01	0.000	0.000	0.000	0.000	0.000
2.5.1.78|g__Citrobacter.s__Citrobacter_youngae	-	2.80E-02	0.000	0.000	0.000	0.000	0.000
2.5.1.78|g__Cloacibacillus.s__Cloacibacillus_porcorum	+	2.16E-02	0.000	0.000	0.000	0.000	0.000
2.5.1.78|g__Clostridiales_unclassified.s__Clostridiales_bacterium_1_7_47FA	-	9.05E-01	0.000	0.000	0.000	0.000	0.000
2.5.1.78|g__Clostridioides.s__Clostridioides_difficile	+	2.34E-01	0.000	0.000	0.000	0.000	0.001
2.5.1.78|g__Clostridium.s__Butyribacterium_methylotrophicum	-	2.85E-01	0.000	0.000	0.000	0.000	0.000
2.5.1.78|g__Clostridium.s__Clostridium_disporicum	+	6.70E-01	0.000	0.000	0.000	0.000	0.000
2.5.1.78|g__Clostridium.s__Clostridium_neonatale	+	3.68E-01	0.000	0.000	0.000	0.000	0.000
2.5.1.78|g__Clostridium.s__Clostridium_perfringens	-	1.25E-01	0.000	0.000	0.000	0.001	0.000
2.5.1.78|g__Clostridium.s__Clostridium_sp_AF36_4	-	6.81E-01	0.000	0.000	0.000	0.001	0.000
2.5.1.78|g__Comamonas.s__Comamonas_kerstersii	-	2.85E-01	0.000	0.000	0.000	0.000	0.000
2.5.1.78|g__Coprobacillus.s__Coprobacillus_cateniformis	+	7.98E-01	0.000	0.000	0.000	0.000	0.000
2.5.1.78|g__Coprobacter.s__Coprobacter_fastidiosus	+	1.77E-01	0.000	0.000	0.000	0.001	0.004
2.5.1.78|g__Coprobacter.s__Coprobacter_secundus	+	2.20E-01	0.000	0.000	0.000	0.000	0.001
2.5.1.78|g__Coprococcus.s__Coprococcus_catus	-	2.83E-01	0.000	0.000	0.000	0.000	0.000
2.5.1.78|g__Coprococcus.s__Coprococcus_eutactus	+	5.09E-01	0.000	0.000	0.000	0.001	0.001
2.5.1.78|g__Desulfovibrio.s__Desulfovibrio_fairfieldensis	+	2.16E-02	0.000	0.000	0.000	0.000	0.000
2.5.1.78|g__Desulfovibrio.s__Desulfovibrio_legallii	+	3.68E-01	0.000	0.000	0.000	0.000	0.000
2.5.1.78|g__Desulfovibrio.s__Desulfovibrio_piger	-	9.74E-01	0.000	0.000	0.000	0.001	0.001
2.5.1.78|g__Desulfovibrio.s__Desulfovibrio_sp_AM18_2	+	1.96E-01	0.000	0.000	0.000	0.000	0.000
2.5.1.78|g__Desulfovibrionaceae_unclassified.s__Desulfovibrionaceae_bacte	-	1.24E-01	0.000	0.000	0.000	0.000	0.000
2.5.1.78|g__Dialister.s__Dialister_invisus	-	2.42E-01	0.000	0.000	0.000	0.000	0.000
2.5.1.78|g__Dialister.s__Dialister_succinatiphilus	-	3.63E-01	0.000	0.000	0.000	0.002	0.001
2.5.1.78|g__Dorea.s__Dorea_formicigenerans	-	8.63E-01	0.000	0.000	0.000	0.000	0.001
2.5.1.78|g__Dorea.s__Dorea_sp_OM02_2LB	+	2.35E-01	0.000	0.000	0.000	0.000	0.000
2.5.1.78|g__Eggerthella.s__Eggerthella_lenta	+	6.71E-01	0.000	0.000	0.000	0.000	0.001
2.5.1.78|g__Enterobacter.s__Enterobacter_bugandensis	-	1.12E-01	0.000	0.000	0.000	0.000	0.000
2.5.1.78|g__Enterobacter.s__Enterobacter_cloacae	-	2.58E-01	0.000	0.000	0.000	0.001	0.000
2.5.1.78|g__Enterococcus.s__Enterococcus_faecium	+	3.68E-01	0.000	0.000	0.000	0.000	0.000
2.5.1.78|g__Erysipelatoclostridium.s__Clostridium_innocuum	+	1.56E-01	0.000	0.000	0.000	0.000	0.000
2.5.1.78|g__Erysipelatoclostridium.s__Clostridium_spiroforme	+	2.35E-01	0.000	0.000	0.000	0.000	0.000
2.5.1.78|g__Erysipelotrichaceae_unclassified.s__Erysipelotrichaceae_bacteri	-	2.85E-01	0.000	0.000	0.000	0.000	0.000
2.5.1.78|g__Eubacterium.s__Eubacterium_ramulus	-	4.21E-02	0.000	0.000	0.000	0.000	0.000
2.5.1.78|g__Eubacterium.s__Eubacterium_sp_AF17_7	+	4.90E-01	0.000	0.000	0.000	0.000	0.000
2.5.1.78|g__Eubacterium.s__Eubacterium_sp_AM18_10LB_B	-	9.26E-01	0.000	0.000	0.000	0.000	0.000
2.5.1.78|g__Faecalicatena.s__Faecalicatena_contorta	+	5.04E-01	0.000	0.000	0.000	0.000	0.000
2.5.1.78|g__Faecalitalea.s__Faecalitalea_cylindroides	+	3.68E-01	0.000	0.000	0.000	0.000	0.000
2.5.1.78|g__Firmicutes_unclassified.s__Firmicutes_bacterium_AM10_47	+	1.10E-01	0.000	0.000	0.000	0.000	0.000
2.5.1.78|g__Flavonifractor.s__Flavonifractor_plautii	+	1.01E-01	0.000	0.000	0.000	0.000	0.000
2.5.1.78|g__Flavonifractor.s__Flavonifractor_sp_An10	+	3.68E-01	0.000	0.000	0.000	0.000	0.000
2.5.1.78|g__Fusobacterium.s__Fusobacterium_equinum	+	3.68E-01	0.000	0.000	0.000	0.000	0.000
2.5.1.78|g__Fusobacterium.s__Fusobacterium_gonidiaformans	+	3.68E-01	0.000	0.000	0.000	0.000	0.000
2.5.1.78|g__Fusobacterium.s__Fusobacterium_mortiferum	-	1.97E-01	0.000	0.000	0.000	0.004	0.001
2.5.1.78|g__Fusobacterium.s__Fusobacterium_ulcerans	+	2.35E-01	0.000	0.000	0.000	0.000	0.001
2.5.1.78|g__Fusobacterium.s__Fusobacterium_varium	-	4.99E-01	0.000	0.000	0.000	0.002	0.000
2.5.1.78|g__Haemophilus.s__Haemophilus_parainfluenzae	-	2.86E-01	0.000	0.000	0.000	0.004	0.001
2.5.1.78|g__Holdemanella.s__Holdemanella_biformis	+	3.68E-01	0.000	0.000	0.000	0.000	0.000
2.5.1.78|g__Hungatella.s__Hungatella_hathewayi	-	9.57E-01	0.000	0.000	0.000	0.000	0.000
2.5.1.78|g__Klebsiella.s__Klebsiella_aerogenes	+	4.62E-01	0.000	0.000	0.000	0.000	0.000
2.5.1.78|g__Klebsiella.s__Klebsiella_grimontii	+	3.68E-01	0.000	0.000	0.000	0.000	0.000
2.5.1.78|g__Klebsiella.s__Klebsiella_michiganensis	-	2.85E-01	0.000	0.000	0.000	0.000	0.000
2.5.1.78|g__Klebsiella.s__Klebsiella_oxytoca	-	6.27E-01	0.000	0.000	0.000	0.000	0.000
2.5.1.78|g__Klebsiella.s__Klebsiella_pneumoniae	-	6.00E-01	0.000	0.000	0.000	0.010	0.004
2.5.1.78|g__Klebsiella.s__Klebsiella_variicola	-	3.45E-01	0.000	0.000	0.000	0.000	0.000
2.5.1.78|g__Kluyvera.s__Kluyvera_ascorbata	-	2.85E-01	0.000	0.000	0.000	0.000	0.000
2.5.1.78|g__Kluyvera.s__Kluyvera_georgiana	-	2.85E-01	0.000	0.000	0.000	0.000	0.000
2.5.1.78|g__Kluyvera.s__Kluyvera_intermedia	+	3.68E-01	0.000	0.000	0.000	0.000	0.000
2.5.1.78|g__Lachnoclostridium.s__Clostridium_aldenense	+	7.11E-01	0.000	0.000	0.000	0.000	0.000
2.5.1.78|g__Lachnoclostridium.s__Clostridium_bolteae	-	4.90E-01	0.000	0.000	0.000	0.001	0.001
2.5.1.78|g__Lachnoclostridium.s__Clostridium_citroniae	-	5.05E-01	0.000	0.000	0.000	0.000	0.000
2.5.1.78|g__Lachnoclostridium.s__Clostridium_clostridioforme	+	3.18E-01	0.000	0.000	0.000	0.001	0.001
2.5.1.78|g__Lachnoclostridium.s__Clostridium_symbiosum	+	1.37E-01	0.000	0.000	0.000	0.000	0.001
2.5.1.78|g__Lachnospira.s__Lachnospira_pectinoschiza	-	1.77E-01	0.000	0.000	0.000	0.002	0.001
2.5.1.78|g__Lactobacillus.s__Lactobacillus_fermentum	+	3.68E-01	0.000	0.000	0.000	0.000	0.000
2.5.1.78|g__Lactobacillus.s__Lactobacillus_plantarum	+	3.68E-01	0.000	0.000	0.000	0.000	0.000
2.5.1.78|g__Lactobacillus.s__Lactobacillus_rogosae	-	2.25E-01	0.000	0.000	0.000	0.002	0.001

2.5.1.78|g__Lactococcus.s__Lactococcus_lactis	-	2.85E-01	0.000	0.000	0.000	0.000	0.000
2.5.1.78|g__Leclercia.s__Leclercia_adecarboxylata	-	1.25E-01	0.000	0.000	0.000	0.000	0.000
2.5.1.78|g__Lelliottia.s__Lelliottia_nimipressuralis	-	5.21E-01	0.000	0.000	0.000	0.000	0.000
2.5.1.78|g__Leuconostoc.s__Leuconostoc_garlicum	-	9.26E-01	0.000	0.000	0.000	0.000	0.000
2.5.1.78|g__Leuconostoc.s__Leuconostoc_lactis	-	9.05E-01	0.000	0.000	0.000	0.000	0.000
2.5.1.78|g__Megamonas.s__Megamonas_funiformis	-	6.74E-01	0.000	0.000	0.000	0.006	0.003
2.5.1.78|g__Megamonas.s__Megamonas_hypermegale	-	6.37E-01	0.000	0.000	0.000	0.002	0.001
2.5.1.78|g__Megamonas.s__Megamonas_rupellensis	-	5.93E-01	0.000	0.000	0.000	0.001	0.000
2.5.1.78|g__Megasphaera.s__Megasphaera_elsdenii	-	1.25E-01	0.000	0.000	0.000	0.000	0.000
2.5.1.78|g__Megasphaera.s__Megasphaera_hexanoica	-	9.26E-01	0.000	0.000	0.000	0.000	0.000
2.5.1.78|g__Megasphaera.s__Megasphaera_micronuciformis	+	1.96E-01	0.000	0.000	0.000	0.000	0.000
2.5.1.78|g__Megasphaera.s__Megasphaera_stantonii	-	1.28E-01	0.000	0.000	0.000	0.000	0.000
2.5.1.78|g__Mesosutterella.s__Mesosutterella_multiformis	-	2.75E-01	0.000	0.000	0.000	0.003	0.003
2.5.1.78|g__Metakosakonia.s__Kluyvera_intestini	+	3.68E-01	0.000	0.000	0.000	0.000	0.000
2.5.1.78|g__Methanobrevibacter.s__Methanobrevibacter_smithii	+	3.80E-02	0.000	0.000	0.000	0.000	0.010
2.5.1.78|g__Mitsuokella.s__Mitsuokella_jalaludinii	-	8.72E-01	0.000	0.000	0.000	0.000	0.000
2.5.1.78|g__Mitsuokella.s__Mitsuokella_multacida	-	7.79E-01	0.000	0.000	0.000	0.000	0.000
2.5.1.78|g__Odoribacter.s__Odoribacter_laneus	+	3.87E-01	0.000	0.000	0.000	0.000	0.002
2.5.1.78|g__Oxalobacter.s__Oxalobacter_formigenes	+	9.48E-01	0.000	0.000	0.000	0.000	0.000
2.5.1.78|g__Paeniclostridium.s__Paeniclostridium_sordellii	+	3.68E-01	0.000	0.000	0.000	0.000	0.000
2.5.1.78|g__Pantoea.s__Pantoea_sesami	-	7.47E-01	0.000	0.000	0.000	0.000	0.000
2.5.1.78|g__Parabacteroides.s__Parabacteroides_chinchillae	-	1.25E-01	0.000	0.000	0.000	0.000	0.000
2.5.1.78|g__Parabacteroides.s__Parabacteroides_goldsteinii	+	1.37E-01	0.000	0.000	0.000	0.001	0.003
2.5.1.78|g__Parabacteroides.s__Parabacteroides_gordonii	-	5.10E-01	0.000	0.000	0.000	0.000	0.000
2.5.1.78|g__Parabacteroides.s__Parabacteroides_johnsonii	+	5.88E-01	0.000	0.000	0.000	0.002	0.001
2.5.1.78|g__Paraprevotella.s__Paraprevotella_clara	-	6.33E-01	0.000	0.000	0.000	0.005	0.006
2.5.1.78|g__Paraprevotella.s__Paraprevotella_xylaniphila	+	8.65E-02	0.000	0.000	0.000	0.000	0.002
2.5.1.78|g__Parasutterella.s__Parasutterella_excrementihominis	+	3.98E-01	0.000	0.000	0.000	0.005	0.003
2.5.1.78|g__Pedobacter.s__Pedobacter_himalayensis	-	1.25E-01	0.000	0.000	0.000	0.000	0.000
2.5.1.78|g__Phascolarctobacterium.s__Phascolarctobacterium_succinatutens	+	3.98E-01	0.000	0.000	0.000	0.003	0.003
2.5.1.78|g__Phytobacter.s__Phytobacter_ursingii	+	3.68E-01	0.000	0.000	0.000	0.000	0.000
2.5.1.78|g__Plesiomonas.s__Plesiomonas_shigelloides	-	2.85E-01	0.000	0.000	0.000	0.001	0.000
2.5.1.78|g__Prevotella.s__Prevotella_buccae	-	2.85E-01	0.000	0.000	0.000	0.004	0.000
2.5.1.78|g__Prevotella.s__Prevotella_copri	-	7.68E-01	0.000	0.000	0.000	0.025	0.006
2.5.1.78|g__Prevotella.s__Prevotella_corporis	+	3.68E-01	0.000	0.000	0.000	0.000	0.000
2.5.1.78|g__Prevotella.s__Prevotella_sp_109	+	6.55E-01	0.000	0.000	0.000	0.012	0.007
2.5.1.78|g__Prevotella.s__Prevotella_sp_885	-	9.05E-01	0.000	0.000	0.000	0.005	0.001
2.5.1.78|g__Prevotella.s__Prevotella_sp_AM42_24	-	2.85E-01	0.000	0.000	0.000	0.003	0.000
2.5.1.78|g__Prevotella.s__Prevotella_stercorea	+	7.48E-01	0.000	0.000	0.000	0.027	0.012
2.5.1.78|g__Prevotella.s__Prevotella_timonensis	-	9.05E-01	0.000	0.000	0.000	0.000	0.000
2.5.1.78|g__Pseudoflavonifractor.s__Pseudoflavonifractor_sp_An184	+	3.68E-01	0.000	0.000	0.000	0.000	0.000
2.5.1.78|g__Pseudomonas.s__Pseudomonas_helleri	-	2.85E-01	0.000	0.000	0.000	0.000	0.000
2.5.1.78|g__Pyramidobacter.s__Pyramidobacter_piscolens	-	8.87E-01	0.000	0.000	0.000	0.000	0.000
2.5.1.78|g__Pyramidobacter.s__Pyramidobacter_sp_C12_8	+	3.68E-01	0.000	0.000	0.000	0.000	0.000
2.5.1.78|g__Raoultella.s__Raoultella_ornithinolytica	-	4.57E-01	0.000	0.000	0.000	0.001	0.000
2.5.1.78|g__Raoultella.s__Raoultella_planticola	-	4.71E-01	0.000	0.000	0.000	0.000	0.000
2.5.1.78|g__Roseburia.s__Roseburia_intestinalis	+	4.43E-01	0.000	0.000	0.000	0.005	0.002
2.5.1.78|g__Roseburia.s__Roseburia_inulinivorans	-	5.38E-01	0.000	0.000	0.000	0.001	0.001
2.5.1.78|g__Ruminococcaceae_unclassified.s__Ruminococcaceae_bacteriun	-	5.30E-01	0.000	0.000	0.000	0.000	0.000
2.5.1.78|g__Ruminococcaceae_unclassified.s__Ruminococcaceae_bacteriun	+	6.54E-01	0.000	0.000	0.000	0.000	0.000
2.5.1.78|g__Ruminococcus.s__Ruminococcus_callidus	-	5.05E-01	0.000	0.000	0.000	0.001	0.000
2.5.1.78|g__Ruminococcus.s__Ruminococcus_sp_AF31_8BH	+	1.19E-01	0.000	0.000	0.000	0.000	0.000
2.5.1.78|g__Sanguibacteroides.s__Sanguibacteroides_justesenii	+	3.50E-01	0.000	0.000	0.000	0.000	0.000
2.5.1.78|g__Senegalimassilia.s__Senegalimassilia_anaerobia	+	3.50E-01	0.000	0.000	0.000	0.000	0.000
2.5.1.78|g__Slackia.s__Slackia_isoflavoniconvertens	+	3.68E-01	0.000	0.000	0.000	0.000	0.000
2.5.1.78|g__Streptococcus.s__Streptococcus_macedonicus	+	1.96E-01	0.000	0.000	0.000	0.000	0.000
2.5.1.78|g__Streptococcus.s__Streptococcus_pasteurianus	+	6.36E-02	0.000	0.000	0.000	0.000	0.000
2.5.1.78|g__Streptococcus.s__Streptococcus_pneumoniae	+	3.68E-01	0.000	0.000	0.000	0.000	0.000
2.5.1.78|g__Succinatimonas.s__Succinatimonas_hippei	+	4.91E-01	0.000	0.000	0.000	0.000	0.000
2.5.1.78|g__Sutterella.s__Sutterella_parvirubra	-	1.25E-01	0.000	0.000	0.000	0.004	0.000
2.5.1.78|g__Turicimonas.s__Turicimonas_muris	+	3.68E-01	0.000	0.000	0.000	0.000	0.000
2.5.1.78|g__Veillonella.s__Veillonella_atypica	-	9.30E-02	0.000	0.000	0.000	0.000	0.000
2.5.1.78|g__Veillonella.s__Veillonella_dispar	-	6.69E-01	0.000	0.000	0.000	0.001	0.000
2.5.1.78|g__Veillonella.s__Veillonella_infantium	-	5.37E-01	0.000	0.000	0.000	0.000	0.000
2.5.1.78|g__Veillonella.s__Veillonella_parvula	-	2.08E-01	0.000	0.000	0.000	0.000	0.000
2.5.1.78|g__Veillonella.s__Veillonella_rogosae	-	8.04E-02	0.000	0.000	0.000	0.000	0.000
2.5.1.78|g__Veillonella.s__Veillonella_tobetsuensis	-	9.05E-01	0.000	0.000	0.000	0.000	0.000
2.5.1.78|g__Veillonellaceae_unclassified.s__Veillonellaceae_bacterium	+	3.68E-01	0.000	0.000	0.000	0.000	0.000
2.5.1.78|g__Victivallales_unclassified.s__Victivallales_bacterium_CCUG_4	+	2.98E-01	0.000	0.000	0.000	0.001	0.001
2.5.1.78|g__Victivallis.s__Victivallis_vadensis	+	4.17E-01	0.000	0.000	0.000	0.000	0.001
2.5.1.78|g__Weissella.s__Weissella_confusa	+	3.68E-01	0.000	0.000	0.000	0.000	0.000
2.5.1.78|g__Yokenella.s__Yokenella_regensburgei	-	2.27E-01	0.000	0.000	0.000	0.000	0.000

	2.5.1.78|g__Bilophila.s__Bilophila_wadsworthia	+	9.67E-01	0.172	0.191	0.020	0.004	0.002
	2.5.1.78|g__Blautia.s__Blautia_wexlerae	+	9.60E-01	0.000	0.031	0.031	0.001	0.001
	2.5.1.78|g__Eubacterium.s__Eubacterium_eligens	+	9.62E-01	0.364	0.414	0.050	0.008	0.007
	2.5.1.78|g__Alistipes.s__Alistipes_onderdonkii	+	3.69E-01	0.112	0.162	0.051	0.006	0.009
	2.5.1.78|g__Bacteroides.s__Bacteroides_dorei	+	5.23E-01	0.050	0.142	0.092	0.014	0.026
	2.5.1.78|g__Bacteroides.s__Bacteroides_massiliensis	+	9.58E-01	0.490	0.596	0.106	0.021	0.021
	2.5.1.78|g__Bacteroides.s__Bacteroides_xylanisolvens	+	8.09E-01	0.269	0.397	0.128	0.012	0.012
	2.5.1.78|g__Phascolarctobacterium.s__Phascolarctobacterium_faecium	+	8.12E-01	0.455	0.654	0.199	0.009	0.009
	2.5.1.78|g__Butyricimonas.s__Butyricimonas_virosa	+	2.25E-02	0.000	0.205	0.205	0.002	0.005
	2.5.1.78|g__Alistipes.s__Alistipes_finegoldii	+	1.44E-01	0.259	0.545	0.286	0.009	0.016
	2.5.1.78|g__Odoribacter.s__Odoribacter_splanchnicus	+	1.11E-01	0.317	0.617	0.300	0.006	0.007
	2.5.1.78|g__Faecalibacterium.s__Faecalibacterium_prausnitzii	+	6.82E-01	2.775	3.117	0.343	0.037	0.037
	2.5.1.78|g__Bacteroides.s__Bacteroides_ovatus	+	1.00E-01	0.413	0.834	0.421	0.015	0.019
	2.5.1.78|g__Sutterella.s__Sutterella_wadsworthensis	+	8.66E-01	0.338	0.981	0.643	0.019	0.018
	2.5.1.78|g__Parabacteroides.s__Parabacteroides_distasonis	+	3.73E-02	1.141	1.827	0.685	0.014	0.022
	2.5.1.78|g__Bacteroides.s__Bacteroides_cellulosilyticus	+	3.35E-02	0.000	0.688	0.688	0.015	0.013
	2.5.1.78|g__Bacteroides.s__Bacteroides_thetaiotaomicron	+	4.18E-02	0.831	1.646	0.815	0.013	0.021
	2.5.1.78|g__Parabacteroides.s__Parabacteroides_merdae	+	9.75E-03	0.378	1.337	0.958	0.007	0.014
	2.5.1.78|g__Alistipes.s__Alistipes_shahii	+	4.60E-02	0.409	1.378	0.969	0.009	0.017
	2.5.1.78|g__Bacteroides.s__Bacteroides_caccae	+	3.29E-02	0.413	1.488	1.075	0.013	0.018
	2.5.1.78|g__Bacteroides.s__Bacteroides_stercoris	+	9.27E-01	1.219	2.441	1.222	0.047	0.044
	2.5.1.78|g__Bacteroides.s__Bacteroides_uniformis	+	3.99E-01	3.490	5.016	1.526	0.058	0.069
	2.5.1.78|g__Alistipes.s__Alistipes_putredinis	+	3.24E-01	2.034	3.829	1.795	0.028	0.037

				Fractional CPM (median)			Relative CPM (average)	
Country	EC number|Bacteria	Increased or decreased in PD	*p* -value	Control	PD	PD - Control	Control	PD
Japan	2.1.1.197	-	6.19E-03	56.867	43.905	-12.962	1.000	1.000
	2.1.1.197|g__Blautia.s__Blautia_obeum	-	2.00E-04	6.269	2.700	-3.569	0.142	0.083
	2.1.1.197|g__Faecalibacterium.s__Faecalibacterium_prausnitzii	-	2.93E-02	2.399	1.137	-1.262	0.072	0.054
	2.1.1.197|g__Anaerostipes.s__Anaerostipes_hadrus	-	7.84E-02	1.253	0.711	-0.541	0.051	0.047
	2.1.1.197|g__Blautia.s__Blautia_wexlerae	-	1.65E-01	0.698	0.234	-0.464	0.038	0.032
	2.1.1.197|g__Blautia.s__Ruminococcus_torques	-	9.17E-02	0.541	0.262	-0.279	0.016	0.013
	2.1.1.197|g__Fusicatenibacter.s__Fusicatenibacter_saccharivorans	-	4.20E-02	0.821	0.558	-0.263	0.043	0.025
	2.1.1.197|g__Firmicutes_unclassified.s__Firmicutes_bacterium_AF16_15	-	1.63E-03	0.247	0.000	-0.247	0.019	0.008
	2.1.1.197|g__Bacteroides.s__Bacteroides_ovatus	+	7.67E-01	0.336	0.297	-0.040	0.019	0.026
	2.1.1.197|g__Acinetobacter.s__Acinetobacter_baumannii	+	3.85E-01	0.000	0.000	0.000	0.000	0.000
	2.1.1.197|g__Alistipes.s__Alistipes_indistinctus	+	5.18E-02	0.000	0.000	0.000	0.001	0.002
	2.1.1.197|g__Anaerotruncus.s__Anaerotruncus_colihominis	+	3.85E-01	0.000	0.000	0.000	0.000	0.000
	2.1.1.197|g__Bacteroides.s__Bacteroides_caccae	+	7.65E-02	0.000	0.000	0.000	0.012	0.029
	2.1.1.197|g__Bacteroides.s__Bacteroides_cellulosilyticus	+	1.58E-02	0.000	0.000	0.000	0.006	0.012
	2.1.1.197|g__Bacteroides.s__Bacteroides_clarus	+	4.43E-01	0.000	0.000	0.000	0.001	0.007
	2.1.1.197|g__Bacteroides.s__Bacteroides_coprocola	+	3.68E-01	0.000	0.000	0.000	0.021	0.028
	2.1.1.197|g__Bacteroides.s__Bacteroides_coprophilus	+	4.41E-01	0.000	0.000	0.000	0.002	0.002
	2.1.1.197|g__Bacteroides.s__Bacteroides_eggerthii	+	5.11E-01	0.000	0.000	0.000	0.006	0.021
	2.1.1.197|g__Bacteroides.s__Bacteroides_faecis	+	7.21E-01	0.000	0.000	0.000	0.000	0.000
	2.1.1.197|g__Bacteroides.s__Bacteroides_finegoldii	+	1.40E-01	0.000	0.000	0.000	0.004	0.005
	2.1.1.197|g__Bacteroides.s__Bacteroides_fluxus	-	2.62E-01	0.000	0.000	0.000	0.000	0.000
	2.1.1.197|g__Bacteroides.s__Bacteroides_fragilis	+	9.03E-01	0.000	0.000	0.000	0.017	0.018
	2.1.1.197|g__Bacteroides.s__Bacteroides_intestinalis	+	8.82E-04	0.000	0.000	0.000	0.002	0.007
	2.1.1.197|g__Bacteroides.s__Bacteroides_massiliensis	+	3.05E-01	0.000	0.000	0.000	0.006	0.008
	2.1.1.197|g__Bacteroides.s__Bacteroides_nordii	+	4.96E-01	0.000	0.000	0.000	0.000	0.001
	2.1.1.197|g__Bacteroides.s__Bacteroides_oleiciplenus	+	3.85E-01	0.000	0.000	0.000	0.000	0.000
	2.1.1.197|g__Bacteroides.s__Bacteroides_plebeius	+	3.61E-01	0.000	0.000	0.000	0.035	0.035
	2.1.1.197|g__Bacteroides.s__Bacteroides_salyersiae	+	4.91E-01	0.000	0.000	0.000	0.000	0.001
	2.1.1.197|g__Bacteroides.s__Bacteroides_stercoris	+	8.36E-01	0.000	0.000	0.000	0.045	0.043
	2.1.1.197|g__Bacteroides.s__Bacteroides_xylanisolvens	+	9.99E-01	0.000	0.000	0.000	0.018	0.020
	2.1.1.197|g__Barnesiella.s__Barnesiella_intestinihominis	+	1.54E-02	0.000	0.000	0.000	0.007	0.017
	2.1.1.197|g__Barnesiella.s__Barnesiella_sp_An22	-	2.62E-01	0.000	0.000	0.000	0.000	0.000
	2.1.1.197|g__Butyricimonas.s__Butyricimonas_virosa	+	1.21E-01	0.000	0.000	0.000	0.000	0.002
	2.1.1.197|g__Candidatus_Gastranaerophilales_unclassified.s__Candidatus_(	+	3.85E-01	0.000	0.000	0.000	0.000	0.000
	2.1.1.197|g__Catenibacterium.s__Catenibacterium_mitsuokai	-	2.34E-02	0.000	0.000	0.000	0.003	0.000
	2.1.1.197|g__Citrobacter.s__Citrobacter_amalonaticus	+	4.46E-01	0.000	0.000	0.000	0.001	0.004
	2.1.1.197|g__Citrobacter.s__Citrobacter_braakii	+	1.00E+00	0.000	0.000	0.000	0.000	0.000
	2.1.1.197|g__Citrobacter.s__Citrobacter_freundii	+	7.63E-02	0.000	0.000	0.000	0.000	0.001
	2.1.1.197|g__Citrobacter.s__Citrobacter_koseri	+	3.85E-01	0.000	0.000	0.000	0.000	0.000
	2.1.1.197|g__Citrobacter.s__Citrobacter_portucalensis	+	1.26E-01	0.000	0.000	0.000	0.000	0.001
	2.1.1.197|g__Citrobacter.s__Citrobacter_werkmanii	+	3.85E-01	0.000	0.000	0.000	0.000	0.004
	2.1.1.197|g__Citrobacter.s__Citrobacter_youngae	+	4.39E-01	0.000	0.000	0.000	0.000	0.001
	2.1.1.197|g__Clostridiales_unclassified.s__Clostridiales_bacterium_CHKCI(	-	2.62E-01	0.000	0.000	0.000	0.000	0.000
	2.1.1.197|g__Clostridium.s__Butyribacterium_methylotrophicum	+	7.31E-01	0.000	0.000	0.000	0.000	0.000
	2.1.1.197|g__Coprobacter.s__Coprobacter_fastidiosus	+	4.10E-02	0.000	0.000	0.000	0.002	0.003
	2.1.1.197|g__Coprobacter.s__Coprobacter_secundus	+	2.72E-01	0.000	0.000	0.000	0.000	0.002
	2.1.1.197|g__Eisenbergiella.s__Eisenbergiella_tayi	+	1.85E-01	0.000	0.000	0.000	0.004	0.002

	2.1.1.197|g__Enterobacter.s__Enterobacter_asburiae	-	8.71E-01	0.000	0.000	0.000	0.000	0.000
	2.1.1.197|g__Enterobacter.s__Enterobacter_cloacae	+	2.87E-01	0.000	0.000	0.000	0.001	0.001
	2.1.1.197|g__Enterobacter.s__Enterobacter_kobei	-	4.24E-01	0.000	0.000	0.000	0.002	0.000
	2.1.1.197|g__Enterobacter.s__Enterobacter_ludwigii	+	4.54E-01	0.000	0.000	0.000	0.000	0.001
	2.1.1.197|g__Enterobacter.s__Enterobacter_mori	+	2.14E-01	0.000	0.000	0.000	0.000	0.001
	2.1.1.197|g__Escherichia.s__Escherichia_coli	+	7.93E-02	0.000	0.000	0.000	0.027	0.013
	2.1.1.197|g__Escherichia.s__Escherichia_fergusonii	-	4.16E-01	0.000	0.000	0.000	0.000	0.000
	2.1.1.197|g__Eubacterium.s__Eubacterium_callanderi	+	2.72E-01	0.000	0.000	0.000	0.000	0.001
	2.1.1.197|g__Flavonifractor.s__Flavonifractor_plautii	-	8.58E-01	0.000	0.000	0.000	0.006	0.006
	2.1.1.197|g__Fusobacterium.s__Fusobacterium_mortiferum	-	2.12E-01	0.000	0.000	0.000	0.001	0.001
	2.1.1.197|g__Fusobacterium.s__Fusobacterium_nucleatum	-	2.62E-01	0.000	0.000	0.000	0.000	0.000
	2.1.1.197|g__Haemophilus.s__Haemophilus_parainfluenzae	-	9.12E-03	0.000	0.000	0.000	0.005	0.001
	2.1.1.197|g__Klebsiella.s__Klebsiella_aerogenes	-	8.57E-01	0.000	0.000	0.000	0.006	0.000
	2.1.1.197|g__Klebsiella.s__Klebsiella_grimontii	-	4.88E-02	0.000	0.000	0.000	0.003	0.000
	2.1.1.197|g__Klebsiella.s__Klebsiella_michiganensis	-	8.71E-01	0.000	0.000	0.000	0.000	0.000
	2.1.1.197|g__Klebsiella.s__Klebsiella_oxytoca	+	2.48E-01	0.000	0.000	0.000	0.009	0.007
	2.1.1.197|g__Klebsiella.s__Klebsiella_pneumoniae	-	8.34E-01	0.000	0.000	0.000	0.006	0.017
	2.1.1.197|g__Klebsiella.s__Klebsiella_variicola	-	7.04E-02	0.000	0.000	0.000	0.001	0.001
	2.1.1.197|g__Kluyvera.s__Kluyvera_cryocrescens	+	1.00E+00	0.000	0.000	0.000	0.000	0.000
	2.1.1.197|g__Lelliottia.s__Lelliottia_nimipressuralis	-	2.62E-01	0.000	0.000	0.000	0.000	0.000
	2.1.1.197|g__Odoribacter.s__Odoribacter_laneus	+	3.87E-01	0.000	0.000	0.000	0.000	0.003
	2.1.1.197|g__Odoribacter.s__Odoribacter_splanchnicus	+	5.19E-02	0.000	0.000	0.000	0.003	0.006
	2.1.1.197|g__Paeniclostridium.s__Paeniclostridium_sordellii	-	2.62E-01	0.000	0.000	0.000	0.000	0.000
	2.1.1.197|g__Parabacteroides.s__Parabacteroides_goldsteinii	+	2.92E-02	0.000	0.000	0.000	0.000	0.003
	2.1.1.197|g__Parabacteroides.s__Parabacteroides_gordonii	+	1.00E+00	0.000	0.000	0.000	0.000	0.000
	2.1.1.197|g__Parabacteroides.s__Parabacteroides_johnsonii	+	6.51E-01	0.000	0.000	0.000	0.002	0.002
	2.1.1.197|g__Paraprevotella.s__Paraprevotella_clara	+	5.49E-01	0.000	0.000	0.000	0.003	0.003
	2.1.1.197|g__Paraprevotella.s__Paraprevotella_xylaniphila	+	4.39E-01	0.000	0.000	0.000	0.000	0.001
	2.1.1.197|g__Raoultella.s__Raoultella_ornithinolytica	-	4.88E-02	0.000	0.000	0.000	0.008	0.000
	2.1.1.197|g__Raoultella.s__Raoultella_planticola	-	2.62E-01	0.000	0.000	0.000	0.000	0.000
	2.1.1.197|g__Rikenella.s__Rikenella_microfusus	+	2.14E-01	0.000	0.000	0.000	0.000	0.001
	2.1.1.197|g__Ruminococcus.s__Ruminococcus_sp_AF31_8BH	+	2.77E-01	0.000	0.000	0.000	0.000	0.001
	2.1.1.197|g__Salmonella.s__Salmonella_enterica	+	3.85E-01	0.000	0.000	0.000	0.000	0.000
	2.1.1.197|g__Victivallales_unclassified.s__Victivallales_bacterium_CCUG_	+	4.61E-01	0.000	0.000	0.000	0.001	0.000
	2.1.1.197|g__Victivallis.s__Victivallis_vadensis	-	8.71E-01	0.000	0.000	0.000	0.000	0.000
	2.1.1.197|g__Bacteroides.s__Bacteroides_vulgatus	-	6.08E-01	1.298	1.315	0.017	0.076	0.063
	2.1.1.197|g__Bacteroides.s__Bacteroides_dorei	+	7.68E-01	0.795	1.010	0.215	0.057	0.059
	2.1.1.197|g__Bacteroides.s__Bacteroides_thetaiotaomicron	+	9.81E-02	0.276	0.527	0.251	0.011	0.021
	2.1.1.197|unclassified	+	3.10E-01	0.753	1.072	0.319	0.037	0.041
	2.1.1.197|g__Parabacteroides.s__Parabacteroides_distasonis	+	1.09E-02	0.549	0.883	0.334	0.020	0.034
	2.1.1.197|g__Bacteroides.s__Bacteroides_uniformis	+	5.62E-01	3.611	4.091	0.480	0.090	0.126
	2.1.1.197|g__Parabacteroides.s__Parabacteroides_merdae	+	1.62E-01	0.000	0.552	0.552	0.017	0.021
USA	2.1.1.197	-	2.37E-04	62.143	55.063	-7.080	1.000	1.000
	2.1.1.197|g__Blautia.s__Blautia_obeum	-	8.54E-08	8.352	4.835	-3.517	0.188	0.139
	2.1.1.197|g__Bacteroides.s__Bacteroides_vulgatus	-	8.24E-02	4.423	3.276	-1.147	0.092	0.083
	2.1.1.197|g__Faecalibacterium.s__Faecalibacterium_prausnitzii	-	6.04E-04	1.748	0.911	-0.837	0.062	0.048
	2.1.1.197|g__Fusicatenibacter.s__Fusicatenibacter_saccharivorans	-	9.17E-04	1.093	0.710	-0.383	0.038	0.028
	2.1.1.197|g__Anaerostipes.s__Anaerostipes_hadrus	-	8.81E-04	0.389	0.077	-0.312	0.015	0.014
	2.1.1.197|g__Bacteroides.s__Bacteroides_uniformis	-	8.18E-01	5.394	5.199	-0.196	0.115	0.128
	2.1.1.197|g__Blautia.s__Ruminococcus_torques	-	2.59E-02	0.889	0.694	-0.194	0.026	0.025
	2.1.1.197|g__Flavonifractor.s__Flavonifractor_plautii	-	4.09E-01	0.537	0.407	-0.130	0.019	0.018
	2.1.1.197|g__Bacteroides.s__Bacteroides_dorei	-	9.69E-01	0.547	0.525	-0.022	0.021	0.028
	2.1.1.197|g__Aeromonas.s__Aeromonas_caviae	+	4.92E-01	0.000	0.000	0.000	0.000	0.000
	2.1.1.197|g__Aggregatibacter.s__Aggregatibacter_segnis	+	4.92E-01	0.000	0.000	0.000	0.000	0.000
	2.1.1.197|g__Alistipes.s__Alistipes_indistinctus	+	2.44E-05	0.000	0.000	0.000	0.001	0.003
	2.1.1.197|g__Anaerotruncus.s__Anaerotruncus_colihominis	+	9.56E-03	0.000	0.000	0.000	0.000	0.000
	2.1.1.197|g__Bacteroides.s__Bacteroides_clarus	+	8.01E-01	0.000	0.000	0.000	0.002	0.001
	2.1.1.197|g__Bacteroides.s__Bacteroides_coprocola	-	5.55E-02	0.000	0.000	0.000	0.008	0.005
	2.1.1.197|g__Bacteroides.s__Bacteroides_coprophilus	+	1.32E-01	0.000	0.000	0.000	0.000	0.001
	2.1.1.197|g__Bacteroides.s__Bacteroides_eggerthii	+	6.13E-01	0.000	0.000	0.000	0.016	0.016
	2.1.1.197|g__Bacteroides.s__Bacteroides_faecichinchillae	+	2.32E-01	0.000	0.000	0.000	0.000	0.000
	2.1.1.197|g__Bacteroides.s__Bacteroides_faecis	+	1.46E-01	0.000	0.000	0.000	0.003	0.003
	2.1.1.197|g__Bacteroides.s__Bacteroides_finegoldii	+	3.90E-01	0.000	0.000	0.000	0.003	0.002
	2.1.1.197|g__Bacteroides.s__Bacteroides_fluxus	+	2.32E-01	0.000	0.000	0.000	0.000	0.000
	2.1.1.197|g__Bacteroides.s__Bacteroides_fragilis	-	6.58E-01	0.000	0.000	0.000	0.014	0.013
	2.1.1.197|g__Bacteroides.s__Bacteroides_intestinalis	+	1.57E-01	0.000	0.000	0.000	0.008	0.010
	2.1.1.197|g__Bacteroides.s__Bacteroides_massiliensis	+	3.58E-01	0.000	0.000	0.000	0.015	0.013
	2.1.1.197|g__Bacteroides.s__Bacteroides_nordii	-	4.31E-01	0.000	0.000	0.000	0.001	0.001
	2.1.1.197|g__Bacteroides.s__Bacteroides_plebeius	-	4.29E-01	0.000	0.000	0.000	0.020	0.008
	2.1.1.197|g__Bacteroides.s__Bacteroides_salyersiae	+	5.73E-02	0.000	0.000	0.000	0.002	0.004
	2.1.1.197|g__Bacteroides.s__Bacteroides_sartorii	-	1.48E-01	0.000	0.000	0.000	0.000	0.000
	2.1.1.197|g__Bacteroides.s__Bacteroides_sp_OM08_11	+	4.92E-01	0.000	0.000	0.000	0.000	0.000

2.1.1.197|g__Bacteroides.s__Bacteroides_stercorirosoris	+	5.59E-01	0.000	0.000	0.000	0.000	0.000
2.1.1.197|g__Bacteroides.s__Bacteroides_xylanisolvens	+	3.65E-01	0.000	0.000	0.000	0.016	0.015
2.1.1.197|g__Barnesiella.s__Barnesiella_intestinihominis	+	1.01E-01	0.000	0.000	0.000	0.006	0.009
2.1.1.197|g__Blautia.s__Blautia_wexlerae	-	4.23E-01	0.000	0.000	0.000	0.006	0.005
2.1.1.197|g__Butyricimonas.s__Butyricimonas_synergistica	-	1.48E-01	0.000	0.000	0.000	0.000	0.000
2.1.1.197|g__Butyricimonas.s__Butyricimonas_virosa	+	2.02E-01	0.000	0.000	0.000	0.001	0.001
2.1.1.197|g__Candidatus_Gastranaerophilales_unclassified.s__Candidatus_(	+	2.32E-01	0.000	0.000	0.000	0.000	0.000
2.1.1.197|g__Catenibacterium.s__Catenibacterium_mitsuokai	+	3.53E-01	0.000	0.000	0.000	0.003	0.005
2.1.1.197|g__Citrobacter.s__Citrobacter_amalonaticus	+	5.20E-01	0.000	0.000	0.000	0.000	0.000
2.1.1.197|g__Citrobacter.s__Citrobacter_braakii	-	1.48E-01	0.000	0.000	0.000	0.001	0.000
2.1.1.197|g__Citrobacter.s__Citrobacter_farmeri	+	2.32E-01	0.000	0.000	0.000	0.000	0.000
2.1.1.197|g__Citrobacter.s__Citrobacter_freundii	+	1.94E-01	0.000	0.000	0.000	0.001	0.000
2.1.1.197|g__Citrobacter.s__Citrobacter_koseri	+	3.30E-01	0.000	0.000	0.000	0.000	0.000
2.1.1.197|g__Citrobacter.s__Citrobacter_portucalensis	-	9.51E-01	0.000	0.000	0.000	0.000	0.000
2.1.1.197|g__Citrobacter.s__Citrobacter_werkmanii	-	5.95E-01	0.000	0.000	0.000	0.000	0.000
2.1.1.197|g__Citrobacter.s__Citrobacter_youngae	+	4.15E-01	0.000	0.000	0.000	0.000	0.000
2.1.1.197|g__Clostridiales_unclassified.s__Clostridiales_bacterium_CHKCI(	+	3.30E-01	0.000	0.000	0.000	0.000	0.000
2.1.1.197|g__Clostridium.s__Butyribacterium_methylotrophicum	+	5.49E-04	0.000	0.000	0.000	0.001	0.002
2.1.1.197|g__Coprobacter.s__Coprobacter_fastidiosus	-	6.34E-04	0.000	0.000	0.000	0.000	0.000
2.1.1.197|g__Coprobacter.s__Coprobacter_secundus	-	5.95E-01	0.000	0.000	0.000	0.000	0.000
2.1.1.197|g__Corynebacterium.s__Corynebacterium_imitans	+	4.92E-01	0.000	0.000	0.000	0.000	0.000
2.1.1.197|g__Eisenbergiella.s__Eisenbergiella_tayi	+	1.13E-04	0.000	0.000	0.000	0.005	0.005
2.1.1.197|g__Enterobacter.s__Enterobacter_asburiae	-	1.48E-01	0.000	0.000	0.000	0.000	0.000
2.1.1.197|g__Enterobacter.s__Enterobacter_cloacae	+	9.51E-02	0.000	0.000	0.000	0.000	0.000
2.1.1.197|g__Enterobacter.s__Enterobacter_kobei	+	2.32E-01	0.000	0.000	0.000	0.000	0.000
2.1.1.197|g__Enterobacter.s__Enterobacter_ludwigii	+	4.92E-01	0.000	0.000	0.000	0.000	0.000
2.1.1.197|g__Enterobacter.s__Enterobacter_mori	+	4.92E-01	0.000	0.000	0.000	0.000	0.000
2.1.1.197|g__Enterobacter.s__Enterobacter_roggenkampii	+	5.57E-01	0.000	0.000	0.000	0.000	0.000
2.1.1.197|g__Escherichia.s__Escherichia_coli	+	3.38E-02	0.000	0.000	0.000	0.022	0.026
2.1.1.197|g__Escherichia.s__Escherichia_fergusonii	+	5.58E-01	0.000	0.000	0.000	0.000	0.000
2.1.1.197|g__Eubacterium.s__Eubacterium_callanderi	+	4.40E-04	0.000	0.000	0.000	0.000	0.002
2.1.1.197|g__Eubacterium.s__Eubacterium_limosum	+	1.94E-01	0.000	0.000	0.000	0.000	0.000
2.1.1.197|g__Eubacterium.s__Eubacterium_maltosivorans	+	2.41E-01	0.000	0.000	0.000	0.000	0.000
2.1.1.197|g__Firmicutes_unclassified.s__Firmicutes_bacterium_AF16_15	-	8.68E-04	0.000	0.000	0.000	0.016	0.010
2.1.1.197|g__Fusobacterium.s__Fusobacterium_mortiferum	+	7.57E-01	0.000	0.000	0.000	0.000	0.000
2.1.1.197|g__Fusobacterium.s__Fusobacterium_nucleatum	-	1.48E-01	0.000	0.000	0.000	0.000	0.000
2.1.1.197|g__Haemophilus.s__Haemophilus_influenzae	+	4.92E-01	0.000	0.000	0.000	0.000	0.000
2.1.1.197|g__Haemophilus.s__Haemophilus_parainfluenzae	+	7.46E-01	0.000	0.000	0.000	0.000	0.000
2.1.1.197|g__Hafnia.s__Hafnia_paralvei	+	4.92E-01	0.000	0.000	0.000	0.000	0.000
2.1.1.197|g__Klebsiella.s__Klebsiella_aerogenes	+	4.79E-01	0.000	0.000	0.000	0.000	0.001
2.1.1.197|g__Klebsiella.s__Klebsiella_grimontii	-	9.73E-01	0.000	0.000	0.000	0.000	0.000
2.1.1.197|g__Klebsiella.s__Klebsiella_michiganensis	-	4.45E-01	0.000	0.000	0.000	0.000	0.000
2.1.1.197|g__Klebsiella.s__Klebsiella_oxytoca	+	4.74E-02	0.000	0.000	0.000	0.006	0.008
2.1.1.197|g__Klebsiella.s__Klebsiella_pneumoniae	+	1.04E-03	0.000	0.000	0.000	0.010	0.011
2.1.1.197|g__Klebsiella.s__Klebsiella_variicola	+	5.73E-02	0.000	0.000	0.000	0.001	0.002
2.1.1.197|g__Kluyvera.s__Kluyvera_ascorbata	+	4.92E-01	0.000	0.000	0.000	0.000	0.000
2.1.1.197|g__Kluyvera.s__Kluyvera_cryocrescens	+	3.30E-01	0.000	0.000	0.000	0.000	0.000
2.1.1.197|g__Kosakonia.s__Kosakonia_cowanii	+	4.92E-01	0.000	0.000	0.000	0.000	0.000
2.1.1.197|g__Lelliottia.s__Lelliottia_nimipressuralis	+	5.60E-01	0.000	0.000	0.000	0.000	0.000
2.1.1.197|g__Morganella.s__Morganella_morganii	+	4.99E-02	0.000	0.000	0.000	0.000	0.000
2.1.1.197|g__Odoribacter.s__Odoribacter_laneus	+	6.77E-01	0.000	0.000	0.000	0.003	0.006
2.1.1.197|g__Oligella.s__Oligella_urethralis	+	4.92E-01	0.000	0.000	0.000	0.000	0.001
2.1.1.197|g__Oxalobacter.s__Oxalobacter_formigenes	+	2.42E-01	0.000	0.000	0.000	0.000	0.000
2.1.1.197|g__Pantoea.s__Pantoea_sesami	-	9.67E-01	0.000	0.000	0.000	0.000	0.000
2.1.1.197|g__Parabacteroides.s__Parabacteroides_goldsteinii	+	1.32E-02	0.000	0.000	0.000	0.002	0.003
2.1.1.197|g__Parabacteroides.s__Parabacteroides_gordonii	+	8.84E-01	0.000	0.000	0.000	0.000	0.000
2.1.1.197|g__Parabacteroides.s__Parabacteroides_johnsonii	-	1.76E-01	0.000	0.000	0.000	0.001	0.001
2.1.1.197|g__Paraprevotella.s__Paraprevotella_clara	-	3.34E-02	0.000	0.000	0.000	0.005	0.004
2.1.1.197|g__Paraprevotella.s__Paraprevotella_xylaniphila	+	4.16E-01	0.000	0.000	0.000	0.000	0.000
2.1.1.197|g__Pedobacter.s__Pedobacter_himalayensis	+	3.30E-01	0.000	0.000	0.000	0.000	0.000
2.1.1.197|g__Pluralibacter.s__Pluralibacter_gergoviae	-	1.48E-01	0.000	0.000	0.000	0.000	0.000
2.1.1.197|g__Porphyromonas.s__Porphyromonas_sp_HMSC077F02	+	5.59E-01	0.000	0.000	0.000	0.000	0.000
2.1.1.197|g__Proteus.s__Proteus_mirabilis	+	8.89E-01	0.000	0.000	0.000	0.000	0.000
2.1.1.197|g__Pseudocitrobacter.s__Pseudocitrobacter_faecalis	+	4.92E-01	0.000	0.000	0.000	0.000	0.000
2.1.1.197|g__Pseudomonas.s__Pseudomonas_aeruginosa	+	3.10E-01	0.000	0.000	0.000	0.000	0.000
2.1.1.197|g__Pseudomonas.s__Pseudomonas_aeruginosa_group	+	2.33E-01	0.000	0.000	0.000	0.000	0.000
2.1.1.197|g__Pseudomonas.s__Pseudomonas_citronellolis	+	4.92E-01	0.000	0.000	0.000	0.000	0.000
2.1.1.197|g__Raoultella.s__Raoultella_ornithinolytica	-	1.48E-01	0.000	0.000	0.000	0.000	0.000
2.1.1.197|g__Raoultella.s__Raoultella_planticola	-	4.06E-02	0.000	0.000	0.000	0.000	0.000
2.1.1.197|g__Rikenella.s__Rikenella_microfusus	+	4.92E-01	0.000	0.000	0.000	0.000	0.000
2.1.1.197|g__Ruminococcus.s__Ruminococcus_sp_AF31_8BH	-	1.58E-01	0.000	0.000	0.000	0.001	0.001
2.1.1.197|g__Salmonella.s__Salmonella_enterica	+	4.12E-01	0.000	0.000	0.000	0.000	0.000

	2.1.1.197|g__Sanguibacteroides.s__Sanguibacteroides_justesenii	+	2.32E-01	0.000	0.000	0.000	0.000	0.000
	2.1.1.197|g__Vibrio.s__Vibrio_parahaemolyticus	+	3.06E-01	0.000	0.000	0.000	0.000	0.000
	2.1.1.197|g__Victivallales_unclassified.s__Victivallales_bacterium_CCUG_	+	2.15E-02	0.000	0.000	0.000	0.000	0.000
	2.1.1.197|g__Victivallis.s__Victivallis_vadensis	+	6.68E-02	0.000	0.000	0.000	0.000	0.000
	2.1.1.197|g__Bacteroides.s__Bacteroides_ovatus	+	5.87E-01	0.388	0.433	0.046	0.021	0.024
	2.1.1.197|g__Bacteroides.s__Bacteroides_thetaiotaomicron	+	5.53E-01	0.278	0.330	0.053	0.015	0.017
	2.1.1.197|g__Bacteroides.s__Bacteroides_cellulosilyticus	+	6.40E-01	0.078	0.146	0.068	0.015	0.018
	2.1.1.197|g__Odoribacter.s__Odoribacter_splanchnicus	+	2.58E-01	0.000	0.098	0.098	0.004	0.006
	2.1.1.197|g__Parabacteroides.s__Parabacteroides_merdae	+	1.94E-01	0.562	0.692	0.129	0.017	0.020
	2.1.1.197|unclassified	+	2.57E-02	0.869	1.111	0.242	0.031	0.040
	2.1.1.197|g__Bacteroides.s__Bacteroides_caccae	+	1.32E-01	0.203	0.569	0.366	0.022	0.037
	2.1.1.197|g__Parabacteroides.s__Parabacteroides_distasonis	+	7.58E-03	1.130	1.609	0.479	0.038	0.051
	2.1.1.197|g__Bacteroides.s__Bacteroides_stercoris	+	1.20E-01	0.000	0.519	0.519	0.058	0.070
Germany	2.1.1.197	-	3.91E-01	52.673	50.140	-2.533	1.000	1.000
	2.1.1.197|g__Faecalibacterium.s__Faecalibacterium_prausnitzii	-	4.71E-01	4.208	3.509	-0.699	0.106	0.089
	2.1.1.197|g__Blautia.s__Blautia_obeum	-	2.77E-01	0.922	0.451	-0.471	0.031	0.026
	2.1.1.197|g__Firmicutes_unclassified.s__Firmicutes_bacterium_AF16_15	-	8.25E-01	1.685	1.370	-0.315	0.042	0.041
	2.1.1.197|g__Bacteroides.s__Bacteroides_caccae	-	4.75E-01	1.362	1.156	-0.206	0.049	0.045
	2.1.1.197|g__Bacteroides.s__Bacteroides_vulgatus	-	6.43E-01	2.822	2.625	-0.197	0.104	0.096
	2.1.1.197|g__Bacteroides.s__Bacteroides_thetaiotaomicron	-	3.44E-01	0.265	0.180	-0.085	0.017	0.010
	2.1.1.197|g__Bacteroides.s__Bacteroides_ovatus	-	7.98E-01	0.304	0.252	-0.052	0.016	0.023
	2.1.1.197|g__Klebsiella.s__Klebsiella_oxytoca	-	7.39E-02	0.047	0.000	-0.047	0.008	0.005
	2.1.1.197|g__Escherichia.s__Escherichia_coli	-	3.89E-01	0.014	0.000	-0.014	0.032	0.014
	2.1.1.197|g__Blautia.s__Blautia_wexlerae	+	8.64E-01	0.010	0.000	-0.010	0.001	0.004
	2.1.1.197|g__Alistipes.s__Alistipes_indistinctus	-	8.21E-01	0.000	0.000	0.000	0.002	0.005
	2.1.1.197|g__Anaerostipes.s__Anaerostipes_hadrus	+	2.91E-01	0.000	0.000	0.000	0.006	0.017
	2.1.1.197|g__Bacteroides.s__Bacteroides_clarus	-	9.92E-01	0.000	0.000	0.000	0.006	0.003
	2.1.1.197|g__Bacteroides.s__Bacteroides_coprocola	-	7.50E-01	0.000	0.000	0.000	0.037	0.012
	2.1.1.197|g__Bacteroides.s__Bacteroides_coprophilus	-	4.85E-01	0.000	0.000	0.000	0.008	0.000
	2.1.1.197|g__Bacteroides.s__Bacteroides_eggerthii	+	7.02E-01	0.000	0.000	0.000	0.016	0.011
	2.1.1.197|g__Bacteroides.s__Bacteroides_faecichinchillae	+	3.59E-01	0.000	0.000	0.000	0.000	0.000
	2.1.1.197|g__Bacteroides.s__Bacteroides_faecis	-	8.80E-01	0.000	0.000	0.000	0.006	0.005
	2.1.1.197|g__Bacteroides.s__Bacteroides_finegoldii	+	3.47E-01	0.000	0.000	0.000	0.003	0.007
	2.1.1.197|g__Bacteroides.s__Bacteroides_fluxus	-	3.09E-01	0.000	0.000	0.000	0.000	0.000
	2.1.1.197|g__Bacteroides.s__Bacteroides_fragilis	-	8.27E-01	0.000	0.000	0.000	0.006	0.012
	2.1.1.197|g__Bacteroides.s__Bacteroides_massiliensis	-	8.18E-01	0.000	0.000	0.000	0.032	0.020
	2.1.1.197|g__Bacteroides.s__Bacteroides_nordii	-	8.06E-01	0.000	0.000	0.000	0.001	0.000
	2.1.1.197|g__Bacteroides.s__Bacteroides_plebeius	+	3.37E-01	0.000	0.000	0.000	0.006	0.010
	2.1.1.197|g__Bacteroides.s__Bacteroides_salyersiae	+	5.69E-01	0.000	0.000	0.000	0.002	0.006
	2.1.1.197|g__Bacteroides.s__Bacteroides_stercorirosoris	+	3.59E-01	0.000	0.000	0.000	0.000	0.000
	2.1.1.197|g__Bacteroides.s__Bacteroides_stercoris	+	7.35E-01	0.000	0.000	0.000	0.029	0.018
	2.1.1.197|g__Butyricimonas.s__Butyricimonas_virosa	-	9.15E-01	0.000	0.000	0.000	0.003	0.003
	2.1.1.197|g__Candidatus_Gastranaerophilales_unclassified.s__Candidatus_(	+	2.20E-01	0.000	0.000	0.000	0.001	0.002
	2.1.1.197|g__Catenibacterium.s__Catenibacterium_mitsuokai	-	3.72E-02	0.000	0.000	0.000	0.009	0.005
	2.1.1.197|g__Citrobacter.s__Citrobacter_sp_MGH106	+	3.59E-01	0.000	0.000	0.000	0.000	0.001
	2.1.1.197|g__Coprobacter.s__Coprobacter_fastidiosus	+	9.63E-01	0.000	0.000	0.000	0.005	0.003
	2.1.1.197|g__Coprobacter.s__Coprobacter_secundus	+	3.59E-01	0.000	0.000	0.000	0.000	0.000
	2.1.1.197|g__Eisenbergiella.s__Eisenbergiella_tayi	-	3.04E-01	0.000	0.000	0.000	0.002	0.000
	2.1.1.197|g__Escherichia.s__Escherichia_fergusonii	-	1.51E-01	0.000	0.000	0.000	0.001	0.001
	2.1.1.197|g__Escherichia.s__Escherichia_marmotae	-	3.09E-01	0.000	0.000	0.000	0.000	0.000
	2.1.1.197|g__Flavonifractor.s__Flavonifractor_plautii	-	9.47E-01	0.000	0.000	0.000	0.001	0.004
	2.1.1.197|g__Fusobacterium.s__Fusobacterium_mortiferum	-	3.09E-01	0.000	0.000	0.000	0.000	0.000
	2.1.1.197|g__Haemophilus.s__Haemophilus_influenzae	-	3.09E-01	0.000	0.000	0.000	0.000	0.000
	2.1.1.197|g__Haemophilus.s__Haemophilus_parainfluenzae	-	1.55E-02	0.000	0.000	0.000	0.005	0.000
	2.1.1.197|g__Hafnia.s__Hafnia_alvei	+	6.04E-01	0.000	0.000	0.000	0.000	0.001
	2.1.1.197|g__Hafnia.s__Hafnia_paralvei	+	3.59E-01	0.000	0.000	0.000	0.000	0.002
	2.1.1.197|g__Klebsiella.s__Klebsiella_pneumoniae	-	3.52E-01	0.000	0.000	0.000	0.008	0.002
	2.1.1.197|g__Klebsiella.s__Klebsiella_variicola	+	6.32E-01	0.000	0.000	0.000	0.000	0.000
	2.1.1.197|g__Obesumbacterium.s__Obesumbacterium_proteus	+	3.59E-01	0.000	0.000	0.000	0.000	0.001
	2.1.1.197|g__Oxalobacter.s__Oxalobacter_formigenes	+	3.56E-01	0.000	0.000	0.000	0.000	0.000
	2.1.1.197|g__Parabacteroides.s__Parabacteroides_goldsteinii	-	5.93E-01	0.000	0.000	0.000	0.001	0.000
	2.1.1.197|g__Parabacteroides.s__Parabacteroides_gordonii	+	1.83E-01	0.000	0.000	0.000	0.000	0.000
	2.1.1.197|g__Parabacteroides.s__Parabacteroides_johnsonii	-	1.44E-01	0.000	0.000	0.000	0.003	0.000
	2.1.1.197|g__Paraprevotella.s__Paraprevotella_clara	-	4.95E-02	0.000	0.000	0.000	0.009	0.005
	2.1.1.197|g__Pseudomonas.s__Pseudomonas_aeruginosa	-	1.40E-01	0.000	0.000	0.000	0.000	0.000
	2.1.1.197|g__Pseudomonas.s__Pseudomonas_aeruginosa_group	-	1.40E-01	0.000	0.000	0.000	0.000	0.000
	2.1.1.197|g__Ruminococcus.s__Ruminococcus_sp_AF31_8BH	+	2.32E-01	0.000	0.000	0.000	0.001	0.001
	2.1.1.197|g__Sanguibacteroides.s__Sanguibacteroides_justesenii	-	5.37E-01	0.000	0.000	0.000	0.001	0.000
	2.1.1.197|g__Victivallales_unclassified.s__Victivallales_bacterium_CCUG_	-	9.54E-01	0.000	0.000	0.000	0.001	0.005
	2.1.1.197|g__Victivallis.s__Victivallis_vadensis	+	6.94E-01	0.000	0.000	0.000	0.000	0.001
	2.1.1.197|g__Parabacteroides.s__Parabacteroides_distasonis	+	7.78E-01	1.309	1.329	0.020	0.028	0.032
	2.1.1.197|g__Odoribacter.s__Odoribacter_splanchnicus	-	6.58E-01	0.482	0.508	0.027	0.010	0.010

	2.1.1.197|g__Bacteroides.s__Bacteroides_xylanisolvens	+	6.04E-01	0.148	0.222	0.075	0.009	0.022
	2.1.1.197|unclassified	+	6.00E-01	2.989	3.133	0.144	0.078	0.106
	2.1.1.197|g__Blautia.s__Ruminococcus_torques	+	4.66E-02	0.150	0.297	0.147	0.005	0.011
	2.1.1.197|g__Fusicatenibacter.s__Fusicatenibacter_saccharivorans	+	8.85E-01	2.135	2.287	0.152	0.070	0.071
	2.1.1.197|g__Bacteroides.s__Bacteroides_cellulosilyticus	+	4.99E-01	0.215	0.396	0.182	0.011	0.015
	2.1.1.197|g__Bacteroides.s__Bacteroides_intestinalis	+	3.22E-01	0.054	0.284	0.230	0.009	0.009
	2.1.1.197|g__Barnesiella.s__Barnesiella_intestinihominis	+	3.09E-01	0.652	0.956	0.303	0.020	0.031
	2.1.1.197|g__Bacteroides.s__Bacteroides_dorei	-	8.79E-01	1.518	1.873	0.355	0.043	0.044
	2.1.1.197|g__Parabacteroides.s__Parabacteroides_merdae	+	4.98E-01	0.304	0.952	0.648	0.015	0.021
	2.1.1.197|g__Bacteroides.s__Bacteroides_uniformis	+	6.54E-01	2.646	3.415	0.769	0.087	0.110
China1	2.1.1.197	-	1.43E-02	87.968	68.567	-19.401	1.000	1.000
	2.1.1.197|g__Bacteroides.s__Bacteroides_vulgatus	-	6.01E-03	7.679	2.410	-5.269	0.161	0.089
	2.1.1.197|g__Bacteroides.s__Bacteroides_fragilis	-	2.32E-02	4.211	1.878	-2.334	0.081	0.053
	2.1.1.197|g__Bacteroides.s__Bacteroides_ovatus	-	5.37E-02	2.783	1.563	-1.220	0.056	0.035
	2.1.1.197|g__Bacteroides.s__Bacteroides_dorei	-	3.84E-01	1.347	0.966	-0.382	0.071	0.050
	2.1.1.197|g__Escherichia.s__Escherichia_coli	-	8.45E-01	0.124	0.039	-0.085	0.007	0.015
	2.1.1.197|g__Bacteroides.s__Bacteroides_cellulosilyticus	-	9.88E-01	0.505	0.431	-0.073	0.017	0.022
	2.1.1.197|g__Klebsiella.s__Klebsiella_oxytoca	-	3.71E-01	0.046	0.000	-0.046	0.010	0.002
	2.1.1.197|g__Flavonifractor.s__Flavonifractor_plautii	-	9.11E-01	0.061	0.029	-0.032	0.003	0.004
	2.1.1.197|g__Blautia.s__Blautia_obeum	-	9.57E-01	0.047	0.028	-0.019	0.002	0.003
	2.1.1.197|g__Aeromonas.s__Aeromonas_diversa	-	3.30E-01	0.000	0.000	0.000	0.000	0.000
	2.1.1.197|g__Aeromonas.s__Aeromonas_veronii	-	3.30E-01	0.000	0.000	0.000	0.000	0.000
	2.1.1.197|g__Aggregatibacter.s__Aggregatibacter_segnis	-	1.00E+00	0.000	0.000	0.000	0.000	0.000
	2.1.1.197|g__Anaerostipes.s__Anaerostipes_hadrus	-	8.62E-01	0.000	0.000	0.000	0.001	0.001
	2.1.1.197|g__Anaerotruncus.s__Anaerotruncus_colihominis	-	3.30E-01	0.000	0.000	0.000	0.000	0.000
	2.1.1.197|g__Bacteroides.s__Bacteroides_clarus	+	5.20E-02	0.000	0.000	0.000	0.001	0.005
	2.1.1.197|g__Bacteroides.s__Bacteroides_coprocola	-	2.17E-01	0.000	0.000	0.000	0.052	0.023
	2.1.1.197|g__Bacteroides.s__Bacteroides_coprophilus	-	4.05E-01	0.000	0.000	0.000	0.007	0.001
	2.1.1.197|g__Bacteroides.s__Bacteroides_eggerthii	+	9.75E-02	0.000	0.000	0.000	0.022	0.039
	2.1.1.197|g__Bacteroides.s__Bacteroides_faecichinchillae	+	8.20E-02	0.000	0.000	0.000	0.000	0.000
	2.1.1.197|g__Bacteroides.s__Bacteroides_faecis	-	1.58E-01	0.000	0.000	0.000	0.001	0.000
	2.1.1.197|g__Bacteroides.s__Bacteroides_finegoldii	+	2.93E-01	0.000	0.000	0.000	0.009	0.017
	2.1.1.197|g__Bacteroides.s__Bacteroides_fluxus	+	3.30E-01	0.000	0.000	0.000	0.000	0.000
	2.1.1.197|g__Bacteroides.s__Bacteroides_massiliensis	+	7.19E-01	0.000	0.000	0.000	0.012	0.020
	2.1.1.197|g__Bacteroides.s__Bacteroides_oleiciplenus	+	5.89E-01	0.000	0.000	0.000	0.000	0.000
	2.1.1.197|g__Bacteroides.s__Bacteroides_plebeius	-	3.26E-01	0.000	0.000	0.000	0.079	0.032
	2.1.1.197|g__Bacteroides.s__Bacteroides_salyersiae	-	7.21E-01	0.000	0.000	0.000	0.003	0.002
	2.1.1.197|g__Bacteroides.s__Bacteroides_sp_OM08_11	+	8.20E-02	0.000	0.000	0.000	0.000	0.000
	2.1.1.197|g__Bacteroides.s__Bacteroides_stercorirosoris	+	5.49E-01	0.000	0.000	0.000	0.000	0.001
	2.1.1.197|g__Barnesiella.s__Barnesiella_intestinihominis	+	4.86E-01	0.000	0.000	0.000	0.009	0.009
	2.1.1.197|g__Barnesiella.s__Barnesiella_sp_An22	-	3.30E-01	0.000	0.000	0.000	0.000	0.000
	2.1.1.197|g__Blautia.s__Blautia_wexlerae	-	9.20E-01	0.000	0.000	0.000	0.001	0.001
	2.1.1.197|g__Blautia.s__Ruminococcus_torques	+	8.39E-01	0.000	0.000	0.000	0.000	0.000
	2.1.1.197|g__Butyricimonas.s__Butyricimonas_virosa	+	7.60E-01	0.000	0.000	0.000	0.004	0.005
	2.1.1.197|g__Catenibacterium.s__Catenibacterium_mitsuokai	+	6.06E-01	0.000	0.000	0.000	0.001	0.009
	2.1.1.197|g__Citrobacter.s__Citrobacter_amalonaticus	+	5.49E-01	0.000	0.000	0.000	0.000	0.000
	2.1.1.197|g__Citrobacter.s__Citrobacter_freundii	+	1.60E-01	0.000	0.000	0.000	0.000	0.000
	2.1.1.197|g__Citrobacter.s__Citrobacter_portucalensis	+	5.69E-01	0.000	0.000	0.000	0.000	0.001
	2.1.1.197|g__Citrobacter.s__Citrobacter_youngae	+	3.20E-01	0.000	0.000	0.000	0.000	0.000
	2.1.1.197|g__Coprobacter.s__Coprobacter_fastidiosus	+	8.38E-01	0.000	0.000	0.000	0.002	0.002
	2.1.1.197|g__Coprobacter.s__Coprobacter_secundus	-	5.30E-01	0.000	0.000	0.000	0.000	0.001
	2.1.1.197|g__Eisenbergiella.s__Eisenbergiella_tayi	+	2.25E-02	0.000	0.000	0.000	0.000	0.000
	2.1.1.197|g__Enterobacter.s__Enterobacter_cloacae	+	9.70E-01	0.000	0.000	0.000	0.000	0.000
	2.1.1.197|g__Enterobacter.s__Enterobacter_ludwigii	+	3.30E-01	0.000	0.000	0.000	0.000	0.000
	2.1.1.197|g__Escherichia.s__Escherichia_fergusonii	-	9.70E-01	0.000	0.000	0.000	0.000	0.000
	2.1.1.197|g__Fusobacterium.s__Fusobacterium_hwasookii	-	3.30E-01	0.000	0.000	0.000	0.000	0.000
	2.1.1.197|g__Fusobacterium.s__Fusobacterium_mortiferum	+	5.89E-01	0.000	0.000	0.000	0.001	0.000
	2.1.1.197|g__Haemophilus.s__Haemophilus_influenzae	-	1.60E-01	0.000	0.000	0.000	0.000	0.000
	2.1.1.197|g__Haemophilus.s__Haemophilus_parainfluenzae	-	4.41E-01	0.000	0.000	0.000	0.007	0.006
	2.1.1.197|g__Klebsiella.s__Klebsiella_aerogenes	+	3.30E-01	0.000	0.000	0.000	0.000	0.000
	2.1.1.197|g__Klebsiella.s__Klebsiella_pneumoniae	+	4.82E-01	0.000	0.000	0.000	0.004	0.014
	2.1.1.197|g__Klebsiella.s__Klebsiella_variicola	+	8.20E-02	0.000	0.000	0.000	0.000	0.001
	2.1.1.197|g__Kosakonia.s__Kosakonia_cowanii	-	3.30E-01	0.000	0.000	0.000	0.000	0.000
	2.1.1.197|g__Oxalobacter.s__Oxalobacter_formigenes	+	6.22E-01	0.000	0.000	0.000	0.000	0.001
	2.1.1.197|g__Parabacteroides.s__Parabacteroides_goldsteinii	+	4.73E-02	0.000	0.000	0.000	0.001	0.006
	2.1.1.197|g__Parabacteroides.s__Parabacteroides_gordonii	+	1.80E-01	0.000	0.000	0.000	0.000	0.002
	2.1.1.197|g__Parabacteroides.s__Parabacteroides_johnsonii	+	1.73E-01	0.000	0.000	0.000	0.001	0.002
	2.1.1.197|g__Paraprevotella.s__Paraprevotella_clara	+	8.99E-03	0.000	0.000	0.000	0.003	0.010
	2.1.1.197|g__Paraprevotella.s__Paraprevotella_xylaniphila	+	9.70E-01	0.000	0.000	0.000	0.000	0.002
	2.1.1.197|g__Proteus.s__Proteus_mirabilis	+	3.30E-01	0.000	0.000	0.000	0.000	0.000
	2.1.1.197|g__Pseudocitrobacter.s__Pseudocitrobacter_faecalis	+	3.30E-01	0.000	0.000	0.000	0.000	0.000
	2.1.1.197|g__Ruminococcus.s__Ruminococcus_sp_AF31_8BH	-	4.71E-01	0.000	0.000	0.000	0.000	0.000

	2.1.1.197|g__Salmonella.s__Salmonella_enterica	+	3.30E-01	0.000	0.000	0.000	0.000	0.000
	2.1.1.197|g__Sanguibacteroides.s__Sanguibacteroides_justesenii	+	5.49E-01	0.000	0.000	0.000	0.000	0.000
	2.1.1.197|g__Victivallales_unclassified.s__Victivallales_bacterium_CCUG_	+	2.96E-01	0.000	0.000	0.000	0.000	0.004
	2.1.1.197|g__Victivallis.s__Victivallis_vadensis	+	1.00E+00	0.000	0.000	0.000	0.000	0.001
	2.1.1.197|g__Bacteroides.s__Bacteroides_intestinalis	+	3.32E-01	0.089	0.097	0.007	0.002	0.006
	2.1.1.197|g__Alistipes.s__Alistipes_indistinctus	+	3.13E-03	0.000	0.042	0.042	0.003	0.006
	2.1.1.197|g__Bacteroides.s__Bacteroides_xylanisolvens	-	9.34E-01	0.704	0.749	0.045	0.042	0.039
	2.1.1.197|g__Firmicutes_unclassified.s__Firmicutes_bacterium_AF16_15	+	1.22E-01	0.000	0.050	0.050	0.002	0.004
	2.1.1.197|g__Bacteroides.s__Bacteroides_nordii	+	9.96E-01	0.058	0.128	0.070	0.004	0.005
	2.1.1.197|g__Fusicatenibacter.s__Fusicatenibacter_saccharivorans	+	7.49E-02	0.006	0.121	0.114	0.002	0.004
	2.1.1.197|g__Faecalibacterium.s__Faecalibacterium_prausnitzii	+	5.53E-01	0.518	0.641	0.123	0.014	0.021
	2.1.1.197|g__Odoribacter.s__Odoribacter_splanchnicus	-	8.83E-01	0.073	0.237	0.164	0.008	0.008
	2.1.1.197|g__Bacteroides.s__Bacteroides_thetaiotaomicron	-	9.58E-01	1.449	1.617	0.168	0.033	0.052
	2.1.1.197|g__Parabacteroides.s__Parabacteroides_distasonis	+	5.41E-01	1.040	1.278	0.237	0.031	0.030
	2.1.1.197|g__Parabacteroides.s__Parabacteroides_merdae	+	2.65E-01	0.170	0.459	0.289	0.015	0.025
	2.1.1.197|g__Bacteroides.s__Bacteroides_stercoris	-	9.60E-01	0.246	0.609	0.363	0.076	0.056
	2.1.1.197|unclassified	+	2.65E-02	0.630	1.547	0.917	0.017	0.039
	2.1.1.197|g__Bacteroides.s__Bacteroides_caccae	+	5.38E-01	0.311	1.759	1.448	0.036	0.061
	2.1.1.197|g__Bacteroides.s__Bacteroides_uniformis	+	1.99E-01	5.142	8.127	2.985	0.084	0.150
China2	2.1.1.197	-	1.89E-02	92.506	68.626	-23.880	1.000	1.000
	2.1.1.197|g__Bacteroides.s__Bacteroides_vulgatus	-	3.21E-02	8.831	4.049	-4.782	0.178	0.120
	2.1.1.197|g__Bacteroides.s__Bacteroides_dorei	-	9.51E-02	3.020	1.026	-1.995	0.079	0.053
	2.1.1.197|g__Bacteroides.s__Bacteroides_xylanisolvens	-	7.81E-02	0.511	0.206	-0.305	0.036	0.020
	2.1.1.197|g__Parabacteroides.s__Parabacteroides_distasonis	+	4.78E-01	1.094	0.904	-0.190	0.021	0.036
	2.1.1.197|g__Bacteroides.s__Bacteroides_thetaiotaomicron	-	2.84E-01	0.515	0.405	-0.111	0.019	0.014
	2.1.1.197|g__Anaerostipes.s__Anaerostipes_hadrus	-	3.51E-01	0.102	0.000	-0.102	0.005	0.005
	2.1.1.197|unclassified	+	2.87E-01	1.326	1.282	-0.043	0.037	0.038
	2.1.1.197|g__Fusicatenibacter.s__Fusicatenibacter_saccharivorans	-	8.73E-01	0.562	0.537	-0.025	0.012	0.013
	2.1.1.197|g__Klebsiella.s__Klebsiella_oxytoca	+	9.68E-01	0.140	0.125	-0.015	0.007	0.009
	2.1.1.197|g__Aggregatibacter.s__Aggregatibacter_segnis	-	1.60E-01	0.000	0.000	0.000	0.000	0.000
	2.1.1.197|g__Alistipes.s__Alistipes_indistinctus	+	3.99E-03	0.000	0.000	0.000	0.000	0.004
	2.1.1.197|g__Atlantibacter.s__Atlantibacter_hermannii	-	3.30E-01	0.000	0.000	0.000	0.000	0.000
	2.1.1.197|g__Bacteroides.s__Bacteroides_clarus	-	5.77E-01	0.000	0.000	0.000	0.001	0.005
	2.1.1.197|g__Bacteroides.s__Bacteroides_coprocola	-	1.34E-01	0.000	0.000	0.000	0.043	0.019
	2.1.1.197|g__Bacteroides.s__Bacteroides_coprophilus	+	2.85E-01	0.000	0.000	0.000	0.013	0.012
	2.1.1.197|g__Bacteroides.s__Bacteroides_eggerthii	-	3.99E-01	0.000	0.000	0.000	0.013	0.005
	2.1.1.197|g__Bacteroides.s__Bacteroides_faecichinchillae	+	5.49E-01	0.000	0.000	0.000	0.000	0.000
	2.1.1.197|g__Bacteroides.s__Bacteroides_faecis	+	3.93E-01	0.000	0.000	0.000	0.001	0.003
	2.1.1.197|g__Bacteroides.s__Bacteroides_finegoldii	-	1.30E-01	0.000	0.000	0.000	0.011	0.002
	2.1.1.197|g__Bacteroides.s__Bacteroides_massiliensis	-	4.87E-01	0.000	0.000	0.000	0.030	0.009
	2.1.1.197|g__Bacteroides.s__Bacteroides_nordii	+	2.74E-01	0.000	0.000	0.000	0.001	0.002
	2.1.1.197|g__Bacteroides.s__Bacteroides_oleiciplenus	-	1.60E-01	0.000	0.000	0.000	0.000	0.000
	2.1.1.197|g__Bacteroides.s__Bacteroides_plebeius	-	3.48E-01	0.000	0.000	0.000	0.108	0.052
	2.1.1.197|g__Bacteroides.s__Bacteroides_salyersiae	-	5.91E-01	0.000	0.000	0.000	0.002	0.002
	2.1.1.197|g__Bacteroides.s__Bacteroides_sartorii	-	3.30E-01	0.000	0.000	0.000	0.000	0.000
	2.1.1.197|g__Bacteroides.s__Bacteroides_stercorirosoris	-	1.00E+00	0.000	0.000	0.000	0.000	0.000
	2.1.1.197|g__Barnesiella.s__Barnesiella_intestinihominis	+	6.73E-01	0.000	0.000	0.000	0.006	0.010
	2.1.1.197|g__Butyricimonas.s__Butyricimonas_synergistica	+	7.02E-01	0.000	0.000	0.000	0.000	0.000
	2.1.1.197|g__Butyricimonas.s__Butyricimonas_virosa	+	1.30E-01	0.000	0.000	0.000	0.002	0.003
	2.1.1.197|g__Catenibacterium.s__Catenibacterium_mitsuokai	+	3.30E-01	0.000	0.000	0.000	0.000	0.000
	2.1.1.197|g__Citrobacter.s__Citrobacter_amalonaticus	+	3.08E-01	0.000	0.000	0.000	0.000	0.002
	2.1.1.197|g__Citrobacter.s__Citrobacter_braakii	+	3.30E-01	0.000	0.000	0.000	0.000	0.000
	2.1.1.197|g__Citrobacter.s__Citrobacter_freundii	-	7.32E-01	0.000	0.000	0.000	0.000	0.001
	2.1.1.197|g__Citrobacter.s__Citrobacter_portucalensis	+	7.59E-01	0.000	0.000	0.000	0.000	0.002
	2.1.1.197|g__Citrobacter.s__Citrobacter_werkmanii	-	1.00E+00	0.000	0.000	0.000	0.000	0.000
	2.1.1.197|g__Citrobacter.s__Citrobacter_youngae	-	9.92E-01	0.000	0.000	0.000	0.000	0.003
	2.1.1.197|g__Coprobacter.s__Coprobacter_fastidiosus	+	3.04E-01	0.000	0.000	0.000	0.001	0.001
	2.1.1.197|g__Coprobacter.s__Coprobacter_secundus	-	6.38E-01	0.000	0.000	0.000	0.000	0.000
	2.1.1.197|g__Eisenbergiella.s__Eisenbergiella_tayi	+	6.12E-03	0.000	0.000	0.000	0.000	0.002
	2.1.1.197|g__Enterobacter.s__Enterobacter_asburiae	-	3.30E-01	0.000	0.000	0.000	0.000	0.000
	2.1.1.197|g__Enterobacter.s__Enterobacter_bugandensis	-	5.89E-01	0.000	0.000	0.000	0.000	0.000
	2.1.1.197|g__Enterobacter.s__Enterobacter_cancerogenus	+	3.30E-01	0.000	0.000	0.000	0.000	0.000
	2.1.1.197|g__Enterobacter.s__Enterobacter_cloacae	-	9.08E-01	0.000	0.000	0.000	0.001	0.003
	2.1.1.197|g__Enterobacter.s__Enterobacter_kobei	+	6.55E-01	0.000	0.000	0.000	0.000	0.001
	2.1.1.197|g__Enterobacter.s__Enterobacter_ludwigii	-	1.60E-01	0.000	0.000	0.000	0.000	0.000
	2.1.1.197|g__Enterobacter.s__Enterobacter_mori	-	3.30E-01	0.000	0.000	0.000	0.000	0.000
	2.1.1.197|g__Enterobacter.s__Enterobacter_roggenkampii	+	1.00E+00	0.000	0.000	0.000	0.000	0.000
	2.1.1.197|g__Firmicutes_unclassified.s__Firmicutes_bacterium_AF16_15	+	9.22E-01	0.000	0.000	0.000	0.008	0.008
	2.1.1.197|g__Fusobacterium.s__Fusobacterium_hwasookii	-	3.30E-01	0.000	0.000	0.000	0.000	0.000
	2.1.1.197|g__Fusobacterium.s__Fusobacterium_mortiferum	-	6.89E-01	0.000	0.000	0.000	0.002	0.005
	2.1.1.197|g__Fusobacterium.s__Fusobacterium_nucleatum	-	3.30E-01	0.000	0.000	0.000	0.000	0.000
	2.1.1.197|g__Fusobacterium.s__Fusobacterium_periodonticum	-	3.30E-01	0.000	0.000	0.000	0.000	0.000

	2.1.1.197|g__Haemophilus.s__Haemophilus_haemolyticus	+	3.30E-01	0.000	0.000	0.000	0.000	0.000
	2.1.1.197|g__Haemophilus.s__Haemophilus_influenzae	+	8.15E-01	0.000	0.000	0.000	0.000	0.000
	2.1.1.197|g__Haemophilus.s__Haemophilus_parainfluenzae	-	2.92E-01	0.000	0.000	0.000	0.008	0.012
	2.1.1.197|g__Klebsiella.s__Klebsiella_aerogenes	-	3.23E-01	0.000	0.000	0.000	0.001	0.002
	2.1.1.197|g__Klebsiella.s__Klebsiella_grimontii	+	1.60E-01	0.000	0.000	0.000	0.000	0.000
	2.1.1.197|g__Klebsiella.s__Klebsiella_michiganensis	+	7.06E-01	0.000	0.000	0.000	0.000	0.000
	2.1.1.197|g__Klebsiella.s__Klebsiella_variicola	+	4.82E-01	0.000	0.000	0.000	0.003	0.004
	2.1.1.197|g__Kluyvera.s__Kluyvera_cryocrescens	+	3.30E-01	0.000	0.000	0.000	0.000	0.000
	2.1.1.197|g__Kluyvera.s__Kluyvera_georgiana	-	3.30E-01	0.000	0.000	0.000	0.000	0.000
	2.1.1.197|g__Leclercia.s__Leclercia_adecarboxylata	-	1.60E-01	0.000	0.000	0.000	0.000	0.000
	2.1.1.197|g__Lelliottia.s__Lelliottia_amnigena	-	2.96E-01	0.000	0.000	0.000	0.000	0.000
	2.1.1.197|g__Lelliottia.s__Lelliottia_nimipressuralis	-	5.76E-01	0.000	0.000	0.000	0.000	0.001
	2.1.1.197|g__Morganella.s__Morganella_morganii	+	1.00E+00	0.000	0.000	0.000	0.000	0.003
	2.1.1.197|g__Oxalobacter.s__Oxalobacter_formigenes	+	2.64E-02	0.000	0.000	0.000	0.000	0.001
	2.1.1.197|g__Pantoea.s__Pantoea_dispersa	+	3.30E-01	0.000	0.000	0.000	0.000	0.000
	2.1.1.197|g__Pantoea.s__Pantoea_sesami	-	3.33E-01	0.000	0.000	0.000	0.000	0.000
	2.1.1.197|g__Parabacteroides.s__Parabacteroides_chinchillae	+	3.30E-01	0.000	0.000	0.000	0.000	0.000
	2.1.1.197|g__Parabacteroides.s__Parabacteroides_goldsteinii	+	2.03E-01	0.000	0.000	0.000	0.000	0.003
	2.1.1.197|g__Parabacteroides.s__Parabacteroides_gordonii	+	1.60E-01	0.000	0.000	0.000	0.000	0.000
	2.1.1.197|g__Parabacteroides.s__Parabacteroides_johnsonii	+	9.65E-01	0.000	0.000	0.000	0.001	0.002
	2.1.1.197|g__Paraprevotella.s__Paraprevotella_clara	+	8.90E-01	0.000	0.000	0.000	0.005	0.004
	2.1.1.197|g__Paraprevotella.s__Paraprevotella_xylaniphila	-	3.30E-01	0.000	0.000	0.000	0.000	0.000
	2.1.1.197|g__Pedobacter.s__Pedobacter_himalayensis	-	4.59E-01	0.000	0.000	0.000	0.000	0.000
	2.1.1.197|g__Proteus.s__Proteus_mirabilis	-	1.00E+00	0.000	0.000	0.000	0.000	0.000
	2.1.1.197|g__Proteus.s__Proteus_vulgaris	+	1.00E+00	0.000	0.000	0.000	0.000	0.000
	2.1.1.197|g__Providencia.s__Providencia_rettgeri	+	3.30E-01	0.000	0.000	0.000	0.000	0.005
	2.1.1.197|g__Raoultella.s__Raoultella_ornithinolytica	-	1.00E+00	0.000	0.000	0.000	0.000	0.000
	2.1.1.197|g__Raoultella.s__Raoultella_planticola	+	1.60E-01	0.000	0.000	0.000	0.000	0.000
	2.1.1.197|g__Ruminococcus.s__Ruminococcus_sp_AF31_8BH	-	4.84E-01	0.000	0.000	0.000	0.000	0.001
	2.1.1.197|g__Salmonella.s__Salmonella_enterica	+	5.89E-01	0.000	0.000	0.000	0.000	0.000
	2.1.1.197|g__Sanguibacteroides.s__Sanguibacteroides_justesenii	+	3.30E-01	0.000	0.000	0.000	0.000	0.000
	2.1.1.197|g__Victivallales_unclassified.s__Victivallales_bacterium_CCUG_	+	3.36E-02	0.000	0.000	0.000	0.000	0.001
	2.1.1.197|g__Victivallis.s__Victivallis_vadensis	+	7.05E-01	0.000	0.000	0.000	0.001	0.001
	2.1.1.197|g__Yokenella.s__Yokenella_regensburgei	-	1.60E-01	0.000	0.000	0.000	0.000	0.000
	2.1.1.197|g__Escherichia.s__Escherichia_fergusonii	+	5.86E-01	0.000	0.007	0.007	0.001	0.001
	2.1.1.197|g__Blautia.s__Blautia_wexlerae	-	9.52E-01	0.140	0.158	0.017	0.008	0.010
	2.1.1.197|g__Bacteroides.s__Bacteroides_cellulosilyticus	+	9.45E-01	0.007	0.025	0.019	0.008	0.013
	2.1.1.197|g__Bacteroides.s__Bacteroides_intestinalis	+	1.22E-01	0.000	0.029	0.029	0.002	0.004
	2.1.1.197|g__Klebsiella.s__Klebsiella_pneumoniae	+	3.56E-01	0.146	0.176	0.030	0.019	0.048
	2.1.1.197|g__Blautia.s__Ruminococcus_torques	+	1.80E-01	0.055	0.105	0.051	0.001	0.004
	2.1.1.197|g__Flavonifractor.s__Flavonifractor_plautii	+	5.80E-01	0.081	0.174	0.094	0.003	0.005
	2.1.1.197|g__Bacteroides.s__Bacteroides_ovatus	+	8.57E-01	0.714	0.810	0.096	0.027	0.023
	2.1.1.197|g__Parabacteroides.s__Parabacteroides_merdae	+	2.26E-01	0.503	0.648	0.145	0.013	0.024
	2.1.1.197|g__Bacteroides.s__Bacteroides_fragilis	+	1.00E+00	0.215	0.405	0.190	0.029	0.034
	2.1.1.197|g__Escherichia.s__Escherichia_coli	+	3.73E-01	0.276	0.483	0.207	0.023	0.045
	2.1.1.197|g__Faecalibacterium.s__Faecalibacterium_prausnitzii	-	9.36E-01	1.311	1.534	0.223	0.037	0.037
	2.1.1.197|g__Odoribacter.s__Odoribacter_splanchnicus	+	1.79E-01	0.097	0.333	0.236	0.005	0.008
	2.1.1.197|g__Blautia.s__Blautia_obeum	+	2.63E-01	0.190	0.468	0.278	0.011	0.027
	2.1.1.197|g__Bacteroides.s__Bacteroides_stercoris	+	8.01E-01	0.265	0.831	0.566	0.055	0.064
	2.1.1.197|g__Bacteroides.s__Bacteroides_caccae	+	4.46E-01	0.288	1.009	0.721	0.026	0.036
	2.1.1.197|g__Bacteroides.s__Bacteroides_uniformis	+	4.47E-01	2.576	3.820	1.244	0.069	0.110
Taiwan	2.1.1.197	-	2.98E-01	76.424	69.078	-7.346	1.000	1.000
	2.1.1.197|g__Bacteroides.s__Bacteroides_vulgatus	-	6.10E-01	7.301	5.355	-1.946	0.120	0.109
	2.1.1.197|g__Bacteroides.s__Bacteroides_massiliensis	-	9.97E-01	0.671	0.115	-0.556	0.038	0.037
	2.1.1.197|g__Bacteroides.s__Bacteroides_plebeius	-	3.71E-01	0.633	0.203	-0.430	0.118	0.065
	2.1.1.197|g__Escherichia.s__Escherichia_coli	-	4.02E-02	0.363	0.023	-0.340	0.020	0.006
	2.1.1.197|g__Bacteroides.s__Bacteroides_stercoris	-	7.98E-01	1.935	1.693	-0.242	0.079	0.067
	2.1.1.197|g__Flavonifractor.s__Flavonifractor_plautii	-	4.93E-01	0.153	0.093	-0.060	0.007	0.007
	2.1.1.197|g__Fusicatenibacter.s__Fusicatenibacter_saccharivorans	-	7.10E-01	0.288	0.254	-0.034	0.010	0.011
	2.1.1.197|g__Aeromonas.s__Aeromonas_caviae	-	2.85E-01	0.000	0.000	0.000	0.000	0.000
	2.1.1.197|g__Aggregatibacter.s__Aggregatibacter_aphrophilus	-	2.85E-01	0.000	0.000	0.000	0.000	0.000
	2.1.1.197|g__Aggregatibacter.s__Aggregatibacter_segnis	-	2.85E-01	0.000	0.000	0.000	0.000	0.000
	2.1.1.197|g__Alistipes.s__Alistipes_indistinctus	-	9.58E-01	0.000	0.000	0.000	0.002	0.009
	2.1.1.197|g__Anaerostipes.s__Anaerostipes_hadrus	+	1.42E-02	0.000	0.000	0.000	0.001	0.002
	2.1.1.197|g__Anaerotruncus.s__Anaerotruncus_colihominis	+	5.15E-01	0.000	0.000	0.000	0.000	0.000
	2.1.1.197|g__Bacteroides.s__Bacteroides_clarus	+	9.67E-01	0.000	0.000	0.000	0.004	0.010
	2.1.1.197|g__Bacteroides.s__Bacteroides_coprocola	+	9.25E-01	0.000	0.000	0.000	0.054	0.041
	2.1.1.197|g__Bacteroides.s__Bacteroides_coprophilus	-	6.76E-01	0.000	0.000	0.000	0.020	0.009
	2.1.1.197|g__Bacteroides.s__Bacteroides_eggerthii	+	1.91E-01	0.000	0.000	0.000	0.010	0.011
	2.1.1.197|g__Bacteroides.s__Bacteroides_faecichinchillae	+	3.87E-01	0.000	0.000	0.000	0.000	0.000
	2.1.1.197|g__Bacteroides.s__Bacteroides_fluxus	-	9.26E-01	0.000	0.000	0.000	0.000	0.002
	2.1.1.197|g__Bacteroides.s__Bacteroides_fragilis	+	4.29E-01	0.000	0.000	0.000	0.019	0.028

2.1.1.197|g__Bacteroides.s__Bacteroides_nordii	+	2.62E-01	0.000	0.000	0.000	0.001	0.002
2.1.1.197|g__Bacteroides.s__Bacteroides_salyersiae	-	9.30E-01	0.000	0.000	0.000	0.006	0.004
2.1.1.197|g__Bacteroides.s__Bacteroides_sartorii	+	8.11E-01	0.000	0.000	0.000	0.005	0.002
2.1.1.197|g__Bacteroides.s__Bacteroides_sp_OM08_11	+	2.56E-01	0.000	0.000	0.000	0.001	0.001
2.1.1.197|g__Barnesiella.s__Barnesiella_intestinihominis	+	3.44E-02	0.000	0.000	0.000	0.006	0.017
2.1.1.197|g__Blautia.s__Ruminococcus_torques	-	6.49E-01	0.000	0.000	0.000	0.001	0.001
2.1.1.197|g__Butyricimonas.s__Butyricimonas_synergistica	+	6.70E-01	0.000	0.000	0.000	0.000	0.000
2.1.1.197|g__Candidatus_Gastranaerophilales_unclassified.s__Candidatus_(	+	3.68E-01	0.000	0.000	0.000	0.000	0.000
2.1.1.197|g__Catenibacterium.s__Catenibacterium_mitsuokai	-	7.89E-01	0.000	0.000	0.000	0.002	0.004
2.1.1.197|g__Citrobacter.s__Citrobacter_amalonaticus	-	4.57E-01	0.000	0.000	0.000	0.000	0.000
2.1.1.197|g__Citrobacter.s__Citrobacter_farmeri	-	2.85E-01	0.000	0.000	0.000	0.000	0.000
2.1.1.197|g__Citrobacter.s__Citrobacter_freundii	-	8.87E-01	0.000	0.000	0.000	0.000	0.000
2.1.1.197|g__Citrobacter.s__Citrobacter_koseri	+	6.54E-01	0.000	0.000	0.000	0.000	0.000
2.1.1.197|g__Citrobacter.s__Citrobacter_portucalensis	-	5.79E-01	0.000	0.000	0.000	0.000	0.000
2.1.1.197|g__Citrobacter.s__Citrobacter_werkmanii	-	9.26E-01	0.000	0.000	0.000	0.000	0.000
2.1.1.197|g__Citrobacter.s__Citrobacter_youngae	-	4.71E-01	0.000	0.000	0.000	0.000	0.000
2.1.1.197|g__Coprobacter.s__Coprobacter_fastidiosus	+	3.36E-01	0.000	0.000	0.000	0.001	0.005
2.1.1.197|g__Coprobacter.s__Coprobacter_secundus	+	1.39E-01	0.000	0.000	0.000	0.000	0.001
2.1.1.197|g__Eisenbergiella.s__Eisenbergiella_tayi	+	6.86E-01	0.000	0.000	0.000	0.001	0.000
2.1.1.197|g__Enterobacter.s__Enterobacter_asburiae	-	2.85E-01	0.000	0.000	0.000	0.000	0.000
2.1.1.197|g__Enterobacter.s__Enterobacter_cloacae	+	9.36E-01	0.000	0.000	0.000	0.000	0.000
2.1.1.197|g__Enterobacter.s__Enterobacter_kobei	-	5.86E-02	0.000	0.000	0.000	0.000	0.000
2.1.1.197|g__Enterobacter.s__Enterobacter_ludwigii	-	2.85E-01	0.000	0.000	0.000	0.000	0.000
2.1.1.197|g__Enterobacter.s__Enterobacter_roggenkampii	-	1.20E-01	0.000	0.000	0.000	0.000	0.000
2.1.1.197|g__Escherichia.s__Escherichia_fergusonii	-	8.72E-01	0.000	0.000	0.000	0.000	0.000
2.1.1.197|g__Firmicutes_unclassified.s__Firmicutes_bacterium_AF16_15	+	7.75E-01	0.000	0.000	0.000	0.006	0.007
2.1.1.197|g__Fusobacterium.s__Fusobacterium_mortiferum	-	3.03E-01	0.000	0.000	0.000	0.007	0.002
2.1.1.197|g__Haemophilus.s__Haemophilus_influenzae	+	6.86E-01	0.000	0.000	0.000	0.000	0.000
2.1.1.197|g__Haemophilus.s__Haemophilus_parahaemolyticus	+	3.68E-01	0.000	0.000	0.000	0.000	0.000
2.1.1.197|g__Haemophilus.s__Haemophilus_parainfluenzae	-	9.11E-02	0.000	0.000	0.000	0.007	0.002
2.1.1.197|g__Hafnia.s__Hafnia_paralvei	-	2.85E-01	0.000	0.000	0.000	0.000	0.000
2.1.1.197|g__Klebsiella.s__Klebsiella_aerogenes	-	2.85E-01	0.000	0.000	0.000	0.000	0.000
2.1.1.197|g__Klebsiella.s__Klebsiella_grimontii	-	2.85E-01	0.000	0.000	0.000	0.000	0.000
2.1.1.197|g__Klebsiella.s__Klebsiella_michiganensis	-	2.85E-01	0.000	0.000	0.000	0.000	0.000
2.1.1.197|g__Klebsiella.s__Klebsiella_oxytoca	-	6.80E-01	0.000	0.000	0.000	0.019	0.002
2.1.1.197|g__Klebsiella.s__Klebsiella_pneumoniae	-	5.08E-01	0.000	0.000	0.000	0.025	0.009
2.1.1.197|g__Klebsiella.s__Klebsiella_variicola	-	6.35E-01	0.000	0.000	0.000	0.001	0.000
2.1.1.197|g__Kluyvera.s__Kluyvera_ascorbata	-	2.85E-01	0.000	0.000	0.000	0.000	0.000
2.1.1.197|g__Kluyvera.s__Kluyvera_georgiana	-	2.85E-01	0.000	0.000	0.000	0.001	0.000
2.1.1.197|g__Leclercia.s__Leclercia_adecarboxylata	-	2.85E-01	0.000	0.000	0.000	0.000	0.000
2.1.1.197|g__Lelliottia.s__Lelliottia_nimipressuralis	-	1.64E-01	0.000	0.000	0.000	0.000	0.000
2.1.1.197|g__Metakosakonia.s__Kluyvera_intestini	+	3.68E-01	0.000	0.000	0.000	0.000	0.000
2.1.1.197|g__Morganella.s__Morganella_morganii	+	3.68E-01	0.000	0.000	0.000	0.000	0.000
2.1.1.197|g__Odoribacter.s__Odoribacter_laneus	+	3.97E-01	0.000	0.000	0.000	0.000	0.003
2.1.1.197|g__Oxalobacter.s__Oxalobacter_formigenes	+	5.69E-01	0.000	0.000	0.000	0.000	0.001
2.1.1.197|g__Pantoea.s__Pantoea_sesami	-	2.42E-01	0.000	0.000	0.000	0.000	0.000
2.1.1.197|g__Parabacteroides.s__Parabacteroides_chinchillae	-	2.85E-01	0.000	0.000	0.000	0.000	0.000
2.1.1.197|g__Parabacteroides.s__Parabacteroides_goldsteinii	+	4.65E-02	0.000	0.000	0.000	0.001	0.004
2.1.1.197|g__Parabacteroides.s__Parabacteroides_gordonii	-	5.21E-01	0.000	0.000	0.000	0.000	0.000
2.1.1.197|g__Parabacteroides.s__Parabacteroides_johnsonii	+	4.10E-01	0.000	0.000	0.000	0.002	0.002
2.1.1.197|g__Paraprevotella.s__Paraprevotella_clara	-	5.58E-01	0.000	0.000	0.000	0.012	0.012
2.1.1.197|g__Paraprevotella.s__Paraprevotella_xylaniphila	+	8.39E-02	0.000	0.000	0.000	0.000	0.003
2.1.1.197|g__Pedobacter.s__Pedobacter_himalayensis	-	2.85E-01	0.000	0.000	0.000	0.000	0.000
2.1.1.197|g__Plesiomonas.s__Plesiomonas_shigelloides	-	2.85E-01	0.000	0.000	0.000	0.001	0.000
2.1.1.197|g__Pseudocitrobacter.s__Pseudocitrobacter_faecalis	+	3.68E-01	0.000	0.000	0.000	0.000	0.000
2.1.1.197|g__Raoultella.s__Raoultella_ornithinolytica	-	4.57E-01	0.000	0.000	0.000	0.001	0.000
2.1.1.197|g__Raoultella.s__Raoultella_planticola	+	3.68E-01	0.000	0.000	0.000	0.000	0.000
2.1.1.197|g__Ruminococcus.s__Ruminococcus_sp_AF31_8BH	+	4.47E-01	0.000	0.000	0.000	0.000	0.000
2.1.1.197|g__Salmonella.s__Salmonella_enterica	+	6.70E-01	0.000	0.000	0.000	0.000	0.000
2.1.1.197|g__Sanguibacteroides.s__Sanguibacteroides_justesenii	+	1.10E-01	0.000	0.000	0.000	0.000	0.000
2.1.1.197|g__Vibrio.s__Vibrio_parahaemolyticus	-	2.85E-01	0.000	0.000	0.000	0.000	0.000
2.1.1.197|g__Victivallales_unclassified.s__Victivallales_bacterium_CCUG_	+	1.77E-01	0.000	0.000	0.000	0.001	0.003
2.1.1.197|g__Victivallis.s__Victivallis_vadensis	+	2.42E-01	0.000	0.000	0.000	0.000	0.001
2.1.1.197|g__Blautia.s__Blautia_obeum	+	3.93E-01	0.090	0.099	0.008	0.003	0.003
2.1.1.197|g__Blautia.s__Blautia_wexlerae	+	5.97E-01	0.030	0.042	0.012	0.002	0.003
2.1.1.197|g__Bacteroides.s__Bacteroides_finegoldii	+	2.52E-01	0.000	0.031	0.031	0.006	0.009
2.1.1.197|g__Bacteroides.s__Bacteroides_dorei	+	7.15E-01	1.141	1.222	0.081	0.034	0.044
2.1.1.197|g__Bacteroides.s__Bacteroides_xylanisolvens	+	9.53E-01	0.688	0.770	0.082	0.034	0.032
2.1.1.197|g__Butyricimonas.s__Butyricimonas_virosa	+	3.67E-02	0.000	0.082	0.082	0.003	0.007
2.1.1.197|g__Bacteroides.s__Bacteroides_intestinalis	+	1.37E-03	0.000	0.148	0.148	0.004	0.007
2.1.1.197|g__Bacteroides.s__Bacteroides_ovatus	+	1.94E-01	0.350	0.537	0.186	0.015	0.022
2.1.1.197|g__Bacteroides.s__Bacteroides_thetaiotaomicron	+	1.78E-01	0.745	0.999	0.255	0.021	0.028

	2.1.1.197|g__Bacteroides.s__Bacteroides_cellulosilyticus	+	2.02E-02	0.000	0.328	0.328	0.010	0.016
	2.1.1.197|unclassified	+	1.78E-01	1.295	1.624	0.328	0.033	0.051
	2.1.1.197|g__Odoribacter.s__Odoribacter_splanchnicus	+	5.77E-02	0.198	0.619	0.421	0.012	0.015
	2.1.1.197|g__Parabacteroides.s__Parabacteroides_merdae	+	1.55E-02	0.478	1.153	0.676	0.014	0.029
	2.1.1.197|g__Faecalibacterium.s__Faecalibacterium_prausnitzii	+	2.14E-01	0.864	1.580	0.715	0.037	0.046
	2.1.1.197|g__Parabacteroides.s__Parabacteroides_distasonis	+	2.98E-02	0.986	1.777	0.791	0.024	0.040
	2.1.1.197|g__Bacteroides.s__Bacteroides_uniformis	+	9.50E-01	3.613	4.630	1.017	0.098	0.090
	2.1.1.197|g__Bacteroides.s__Bacteroides_caccae	+	2.40E-02	0.844	2.549	1.704	0.036	0.052

				Fractional CPM (median)			Relative CPM (average)	
Country	EC number|Bacteria	Increased or decreased in PD	*p* -value	Control	PD	PD - Control	Control	PD
Japan	4.2.1.59	-	3.14E-03	145.551	131.427	-14.125	1.000	1.000
	4.2.1.59|g__Faecalibacterium.s__Faecalibacterium_prausnitzii	-	8.83E-03	8.227	3.613	-4.614	0.054	0.041
	4.2.1.59|g__Fusicatenibacter.s__Fusicatenibacter_saccharivorans	-	1.02E-03	5.753	2.465	-3.288	0.048	0.026
	4.2.1.59|g__Blautia.s__Blautia_obeum	-	1.52E-03	7.393	4.641	-2.752	0.060	0.045
	4.2.1.59|unclassified	-	4.51E-01	21.043	18.481	-2.562	0.144	0.154
	4.2.1.59|g__Gemmiger.s__Gemmiger_formicilis	-	3.06E-02	2.012	0.521	-1.492	0.021	0.015
	4.2.1.59|g__Blautia.s__Ruminococcus_torques	-	1.74E-01	5.278	4.109	-1.168	0.047	0.040
	4.2.1.59|g__Blautia.s__Blautia_wexlerae	-	2.47E-05	1.443	0.388	-1.055	0.014	0.010
	4.2.1.59|g__Anaerostipes.s__Anaerostipes_hadrus	-	7.44E-02	2.621	1.863	-0.758	0.031	0.024
	4.2.1.59|g__Clostridiales_unclassified.s__Clostridiales_bacterium_KLE161	-	1.58E-03	0.585	0.000	-0.585	0.008	0.004
	4.2.1.59|g__Lachnospiraceae_unclassified.s__Eubacterium_rectale	-	1.23E-01	0.482	0.000	-0.482	0.021	0.018
	4.2.1.59|g__Blautia.s__Ruminococcus_gnavus	-	1.45E-01	0.571	0.127	-0.444	0.012	0.008
	4.2.1.59|g__Dorea.s__Dorea_longicatena	-	4.07E-01	0.217	0.000	-0.217	0.008	0.007
	4.2.1.59|g__Bacteroides.s__Bacteroides_vulgatus	-	6.43E-01	1.307	1.123	-0.183	0.031	0.025
	4.2.1.59|g__Acidaminococcus.s__Acidaminococcus_fermentans	+	3.85E-01	0.000	0.000	0.000	0.000	0.000
	4.2.1.59|g__Acidaminococcus.s__Acidaminococcus_intestini	-	9.97E-01	0.000	0.000	0.000	0.001	0.001
	4.2.1.59|g__Adlercreutzia.s__Adlercreutzia_equolifaciens	+	1.93E-01	0.000	0.000	0.000	0.000	0.001
	4.2.1.59|g__Akkermansia.s__Akkermansia_muciniphila	+	1.73E-03	0.000	0.000	0.000	0.003	0.019
	4.2.1.59|g__Alistipes.s__Alistipes_indistinctus	+	5.42E-01	0.000	0.000	0.000	0.000	0.001
	4.2.1.59|g__Alistipes.s__Alistipes_inops	-	8.62E-01	0.000	0.000	0.000	0.001	0.001
	4.2.1.59|g__Alistipes.s__Alistipes_onderdonkii	+	7.64E-03	0.000	0.000	0.000	0.002	0.006
	4.2.1.59|g__Alistipes.s__Alistipes_sp_An31A	-	8.57E-01	0.000	0.000	0.000	0.001	0.000
	4.2.1.59|g__Alistipes.s__Alistipes_sp_An66	+	3.85E-01	0.000	0.000	0.000	0.000	0.000
	4.2.1.59|g__Alistipes.s__Alistipes_sp_CHKCI003	-	2.62E-01	0.000	0.000	0.000	0.000	0.000
	4.2.1.59|g__Alistipes.s__Alistipes_timonensis	+	3.85E-01	0.000	0.000	0.000	0.000	0.000
	4.2.1.59|g__Allisonella.s__Allisonella_histaminiformans	-	7.45E-01	0.000	0.000	0.000	0.000	0.000
	4.2.1.59|g__Anaerofustis.s__Anaerofustis_stercorihominis	+	2.14E-01	0.000	0.000	0.000	0.000	0.000
	4.2.1.59|g__Anaeroglobus.s__Anaeroglobus_geminatus	+	3.85E-01	0.000	0.000	0.000	0.000	0.000
	4.2.1.59|g__Anaerostipes.s__Anaerostipes_caccae	-	7.37E-01	0.000	0.000	0.000	0.000	0.000
	4.2.1.59|g__Anaerotruncus.s__Anaerotruncus_colihominis	+	8.87E-01	0.000	0.000	0.000	0.000	0.000
	4.2.1.59|g__Asaccharobacter.s__Asaccharobacter_celatus	+	3.38E-01	0.000	0.000	0.000	0.001	0.001
	4.2.1.59|g__Bacillus.s__Bacillus_gibsonii	+	4.35E-01	0.000	0.000	0.000	0.000	0.000
	4.2.1.59|g__Bacillus.s__Bacillus_murimartini	+	7.24E-01	0.000	0.000	0.000	0.000	0.000
	4.2.1.59|g__Bacillus.s__Bacillus_subtilis_group	+	4.39E-01	0.000	0.000	0.000	0.000	0.000
	4.2.1.59|g__Bacteroides.s__Bacteroides_cellulosilyticus	+	3.37E-03	0.000	0.000	0.000	0.003	0.005
	4.2.1.59|g__Bacteroides.s__Bacteroides_clarus	+	3.33E-01	0.000	0.000	0.000	0.000	0.002
	4.2.1.59|g__Bacteroides.s__Bacteroides_coprocola	+	2.07E-01	0.000	0.000	0.000	0.006	0.008
	4.2.1.59|g__Bacteroides.s__Bacteroides_coprophilus	+	3.02E-01	0.000	0.000	0.000	0.000	0.001
	4.2.1.59|g__Bacteroides.s__Bacteroides_eggerthii	+	2.90E-01	0.000	0.000	0.000	0.003	0.006
	4.2.1.59|g__Bacteroides.s__Bacteroides_faecichinchillae	+	3.85E-01	0.000	0.000	0.000	0.000	0.000
	4.2.1.59|g__Bacteroides.s__Bacteroides_faecis	-	2.60E-01	0.000	0.000	0.000	0.000	0.000
	4.2.1.59|g__Bacteroides.s__Bacteroides_finegoldii	+	2.01E-01	0.000	0.000	0.000	0.002	0.002
	4.2.1.59|g__Bacteroides.s__Bacteroides_fluxus	-	1.09E-01	0.000	0.000	0.000	0.000	0.000
	4.2.1.59|g__Bacteroides.s__Bacteroides_fragilis	-	8.43E-01	0.000	0.000	0.000	0.003	0.002
	4.2.1.59|g__Bacteroides.s__Bacteroides_massiliensis	+	2.68E-01	0.000	0.000	0.000	0.002	0.003
	4.2.1.59|g__Bacteroides.s__Bacteroides_nordii	+	2.86E-01	0.000	0.000	0.000	0.000	0.001
	4.2.1.59|g__Bacteroides.s__Bacteroides_oleiciplenus	+	3.85E-01	0.000	0.000	0.000	0.000	0.000
	4.2.1.59|g__Bacteroides.s__Bacteroides_plebeius	+	1.92E-01	0.000	0.000	0.000	0.013	0.010
	4.2.1.59|g__Bacteroides.s__Bacteroides_salyersiae	+	2.39E-02	0.000	0.000	0.000	0.000	0.001
	4.2.1.59|g__Bacteroides.s__Bacteroides_stercoris	-	9.43E-01	0.000	0.000	0.000	0.022	0.016
	4.2.1.59|g__Barnesiella.s__Barnesiella_intestinihominis	+	9.16E-03	0.000	0.000	0.000	0.002	0.005
	4.2.1.59|g__Barnesiella.s__Barnesiella_sp_An22	-	2.62E-01	0.000	0.000	0.000	0.000	0.000
	4.2.1.59|g__Bifidobacterium.s__Bifidobacterium_animalis	+	4.48E-01	0.000	0.000	0.000	0.011	0.007
	4.2.1.59|g__Bifidobacterium.s__Bifidobacterium_breve	+	1.29E-01	0.000	0.000	0.000	0.003	0.002
	4.2.1.59|g__Bilophila.s__Bilophila_wadsworthia	+	8.14E-02	0.000	0.000	0.000	0.002	0.002
	4.2.1.59|g__Blautia.s__Blautia_hydrogenotrophica	+	2.33E-01	0.000	0.000	0.000	0.000	0.001
	4.2.1.59|g__Blautia.s__Blautia_sp_AF19_10LB	+	4.65E-01	0.000	0.000	0.000	0.002	0.004
	4.2.1.59|g__Blautia.s__Blautia_sp_An249	+	3.85E-01	0.000	0.000	0.000	0.000	0.000
	4.2.1.59|g__Butyrivibrio.s__Butyrivibrio_crossotus	+	3.85E-01	0.000	0.000	0.000	0.000	0.000
	4.2.1.59|g__Campylobacter.s__Campylobacter_concisus	-	1.09E-01	0.000	0.000	0.000	0.000	0.000
	4.2.1.59|g__Candidatus_Gastranaerophilales_unclassified.s__Candidatus_G	+	3.85E-01	0.000	0.000	0.000	0.000	0.000
	4.2.1.59|g__Catabacter.s__Catabacter_hongkongensis	+	3.85E-01	0.000	0.000	0.000	0.000	0.000

4.2.1.59|g__Catenibacterium.s__Catenibacterium_mitsuokai	-	5.29E-03	0.000	0.000	0.000	0.003	0.000
4.2.1.59|g__Cellulosilyticum.s__Cellulosilyticum_lentocellum	+	3.85E-01	0.000	0.000	0.000	0.000	0.000
4.2.1.59|g__Cetobacterium.s__Cetobacterium_somerae	-	2.62E-01	0.000	0.000	0.000	0.000	0.000
4.2.1.59|g__Citrobacter.s__Citrobacter_amalonaticus	+	7.21E-01	0.000	0.000	0.000	0.000	0.001
4.2.1.59|g__Citrobacter.s__Citrobacter_braakii	+	2.82E-01	0.000	0.000	0.000	0.000	0.001
4.2.1.59|g__Citrobacter.s__Citrobacter_freundii	+	2.72E-01	0.000	0.000	0.000	0.000	0.001
4.2.1.59|g__Citrobacter.s__Citrobacter_koseri	-	2.62E-01	0.000	0.000	0.000	0.000	0.000
4.2.1.59|g__Citrobacter.s__Citrobacter_portucalensis	+	4.54E-01	0.000	0.000	0.000	0.000	0.000
4.2.1.59|g__Citrobacter.s__Citrobacter_werkmanii	+	3.85E-01	0.000	0.000	0.000	0.000	0.001
4.2.1.59|g__Citrobacter.s__Citrobacter_youngae	+	2.14E-01	0.000	0.000	0.000	0.000	0.000
4.2.1.59|g__Cloacibacillus.s__Cloacibacillus_porcorum	+	2.14E-01	0.000	0.000	0.000	0.000	0.000
4.2.1.59|g__Clostridiales_unclassified.s__Clostridiales_bacterium_1_7_47FA	+	3.85E-01	0.000	0.000	0.000	0.000	0.000
4.2.1.59|g__Clostridioides.s__Clostridioides_difficile	+	2.26E-01	0.000	0.000	0.000	0.001	0.001
4.2.1.59|g__Clostridium.s__Butyribacterium_methylotrophicum	+	2.82E-01	0.000	0.000	0.000	0.000	0.000
4.2.1.59|g__Clostridium.s__Clostridium_celatum	-	2.62E-01	0.000	0.000	0.000	0.000	0.000
4.2.1.59|g__Clostridium.s__Clostridium_disporicum	-	7.12E-01	0.000	0.000	0.000	0.002	0.001
4.2.1.59|g__Clostridium.s__Clostridium_paraputrificum	-	7.37E-01	0.000	0.000	0.000	0.000	0.000
4.2.1.59|g__Clostridium.s__Clostridium_perfringens	-	7.22E-01	0.000	0.000	0.000	0.000	0.000
4.2.1.59|g__Clostridium.s__Clostridium_sp_AF36_4	-	8.90E-01	0.000	0.000	0.000	0.001	0.001
4.2.1.59|g__Clostridium.s__Clostridium_sp_AM22_11AC	-	6.90E-02	0.000	0.000	0.000	0.008	0.003
4.2.1.59|g__Clostridium.s__Clostridium_sp_D5	+	3.85E-01	0.000	0.000	0.000	0.000	0.000
4.2.1.59|g__Coprobacillus.s__Coprobacillus_cateniformis	+	7.63E-02	0.000	0.000	0.000	0.000	0.000
4.2.1.59|g__Coprobacter.s__Coprobacter_fastidiosus	+	4.53E-01	0.000	0.000	0.000	0.001	0.001
4.2.1.59|g__Coprobacter.s__Coprobacter_secundus	+	1.76E-01	0.000	0.000	0.000	0.000	0.000
4.2.1.59|g__Coprococcus.s__Coprococcus_catus	-	2.78E-01	0.000	0.000	0.000	0.002	0.002
4.2.1.59|g__Coprococcus.s__Coprococcus_comes	-	2.29E-02	0.000	0.000	0.000	0.004	0.003
4.2.1.59|g__Desulfovibrio.s__Desulfovibrio_desulfuricans	+	2.88E-02	0.000	0.000	0.000	0.000	0.001
4.2.1.59|g__Desulfovibrio.s__Desulfovibrio_fairfieldensis	+	1.26E-01	0.000	0.000	0.000	0.000	0.000
4.2.1.59|g__Desulfovibrio.s__Desulfovibrio_piger	+	7.44E-01	0.000	0.000	0.000	0.000	0.000
4.2.1.59|g__Desulfovibrio.s__Desulfovibrio_sp_AM18_2	+	3.85E-01	0.000	0.000	0.000	0.000	0.000
4.2.1.59|g__Dialister.s__Dialister_invisus	-	3.27E-01	0.000	0.000	0.000	0.005	0.002
4.2.1.59|g__Dialister.s__Dialister_succinatiphilus	+	7.10E-01	0.000	0.000	0.000	0.000	0.001
4.2.1.59|g__Dorea.s__Dorea_formicigenerans	-	5.62E-01	0.000	0.000	0.000	0.005	0.004
4.2.1.59|g__Dorea.s__Dorea_sp_OM02_2LB	+	3.85E-01	0.000	0.000	0.000	0.000	0.000
4.2.1.59|g__Eisenbergiella.s__Eisenbergiella_massiliensis	+	4.24E-03	0.000	0.000	0.000	0.000	0.000
4.2.1.59|g__Eisenbergiella.s__Eisenbergiella_tayi	+	8.44E-02	0.000	0.000	0.000	0.000	0.001
4.2.1.59|g__Enterobacter.s__Enterobacter_asburiae	+	3.44E-01	0.000	0.000	0.000	0.000	0.000
4.2.1.59|g__Enterobacter.s__Enterobacter_bugandensis	-	2.07E-01	0.000	0.000	0.000	0.001	0.000
4.2.1.59|g__Enterobacter.s__Enterobacter_cloacae	+	2.14E-01	0.000	0.000	0.000	0.000	0.000
4.2.1.59|g__Enterobacter.s__Enterobacter_mori	+	1.26E-01	0.000	0.000	0.000	0.000	0.001
4.2.1.59|g__Enterococcus.s__Enterococcus_asini	-	2.62E-01	0.000	0.000	0.000	0.000	0.000
4.2.1.59|g__Enterococcus.s__Enterococcus_avium	+	1.82E-01	0.000	0.000	0.000	0.000	0.001
4.2.1.59|g__Enterococcus.s__Enterococcus_casseliflavus	+	3.85E-01	0.000	0.000	0.000	0.000	0.000
4.2.1.59|g__Enterococcus.s__Enterococcus_faecalis	-	4.32E-01	0.000	0.000	0.000	0.000	0.001
4.2.1.59|g__Enterococcus.s__Enterococcus_faecium	+	7.89E-01	0.000	0.000	0.000	0.002	0.009
4.2.1.59|g__Enterococcus.s__Enterococcus_gallinarum	+	1.26E-01	0.000	0.000	0.000	0.000	0.000
4.2.1.59|g__Enterococcus.s__Enterococcus_raffinosus	+	2.14E-01	0.000	0.000	0.000	0.000	0.000
4.2.1.59|g__Enterococcus.s__Enterococcus_saccharolyticus	+	2.14E-01	0.000	0.000	0.000	0.000	0.000
4.2.1.59|g__Enterococcus.s__Enterococcus_thailandicus	+	3.85E-01	0.000	0.000	0.000	0.000	0.000
4.2.1.59|g__Erysipelatoclostridium.s__Clostridium_innocuum	+	3.70E-01	0.000	0.000	0.000	0.000	0.000
4.2.1.59|g__Erysipelatoclostridium.s__Clostridium_spiroforme	+	8.88E-01	0.000	0.000	0.000	0.001	0.001
4.2.1.59|g__Erysipelatoclostridium.s__Erysipelatoclostridium_ramosum	+	2.42E-01	0.000	0.000	0.000	0.005	0.004
4.2.1.59|g__Erysipelotrichaceae_unclassified.s__Erysipelotrichaceae_bacteri	+	2.88E-02	0.000	0.000	0.000	0.000	0.000
4.2.1.59|g__Escherichia.s__Escherichia_coli	+	9.21E-01	0.000	0.000	0.000	0.034	0.017
4.2.1.59|g__Escherichia.s__Escherichia_fergusonii	-	1.82E-01	0.000	0.000	0.000	0.000	0.000
4.2.1.59|g__Eubacterium.s__Eubacterium_callanderi	+	1.26E-01	0.000	0.000	0.000	0.000	0.000
4.2.1.59|g__Eubacterium.s__Eubacterium_limosum	+	2.14E-01	0.000	0.000	0.000	0.000	0.000
4.2.1.59|g__Eubacterium.s__Eubacterium_maltosivorans	+	3.85E-01	0.000	0.000	0.000	0.000	0.000
4.2.1.59|g__Eubacterium.s__Eubacterium_ramulus	-	1.96E-01	0.000	0.000	0.000	0.002	0.001
4.2.1.59|g__Eubacterium.s__Eubacterium_sp_AF17_7	+	4.31E-02	0.000	0.000	0.000	0.001	0.003
4.2.1.59|g__Eubacterium.s__Eubacterium_ventriosum	-	2.70E-01	0.000	0.000	0.000	0.004	0.002
4.2.1.59|g__Faecalicatena.s__Faecalicatena_contorta	+	2.14E-01	0.000	0.000	0.000	0.000	0.000
4.2.1.59|g__Faecalicoccus.s__Faecalicoccus_pleomorphus	-	4.74E-01	0.000	0.000	0.000	0.000	0.001
4.2.1.59|g__Faecalitalea.s__Faecalitalea_cylindroides	+	1.10E-02	0.000	0.000	0.000	0.000	0.002
4.2.1.59|g__Firmicutes_unclassified.s__Firmicutes_bacterium_AF16_15	-	7.44E-02	0.000	0.000	0.000	0.002	0.003
4.2.1.59|g__Flavonifractor.s__Flavonifractor_plautii	+	2.03E-01	0.000	0.000	0.000	0.003	0.003
4.2.1.59|g__Flavonifractor.s__Flavonifractor_sp_An10	+	3.03E-02	0.000	0.000	0.000	0.000	0.002
4.2.1.59|g__Fusobacterium.s__Fusobacterium_mortiferum	-	1.03E-01	0.000	0.000	0.000	0.001	0.000
4.2.1.59|g__Fusobacterium.s__Fusobacterium_ulcerans	+	2.92E-01	0.000	0.000	0.000	0.000	0.000
4.2.1.59|g__Gardnerella.s__Gardnerella_vaginalis	+	4.48E-02	0.000	0.000	0.000	0.000	0.004
4.2.1.59|g__Gemmiger.s__Gemmiger_sp_An194	+	4.67E-02	0.000	0.000	0.000	0.000	0.001
4.2.1.59|g__Gemmiger.s__Gemmiger_sp_An50	-	8.57E-01	0.000	0.000	0.000	0.000	0.000

4.2.1.59|g__Gordonibacter.s__Gordonibacter_sp_An232A	+	3.85E-01	0.000	0.000	0.000	0.000	0.000
4.2.1.59|g__Haemophilus.s__Haemophilus_parainfluenzae	-	2.90E-02	0.000	0.000	0.000	0.002	0.001
4.2.1.59|g__Haemophilus.s__Haemophilus_sputorum	-	2.62E-01	0.000	0.000	0.000	0.000	0.000
4.2.1.59|g__Hungatella.s__Hungatella_effluvii	+	3.85E-01	0.000	0.000	0.000	0.000	0.000
4.2.1.59|g__Hungatella.s__Hungatella_hathewayi	-	8.92E-01	0.000	0.000	0.000	0.002	0.002
4.2.1.59|g__Intestinibacter.s__Intestinibacter_bartlettii	-	2.00E-02	0.000	0.000	0.000	0.003	0.002
4.2.1.59|g__Intestinimonas.s__Intestinimonas_butyriciproducens	+	8.04E-02	0.000	0.000	0.000	0.000	0.001
4.2.1.59|g__Klebsiella.s__Klebsiella_aerogenes	+	6.90E-01	0.000	0.000	0.000	0.001	0.000
4.2.1.59|g__Klebsiella.s__Klebsiella_grimontii	-	8.57E-01	0.000	0.000	0.000	0.000	0.000
4.2.1.59|g__Klebsiella.s__Klebsiella_michiganensis	-	8.12E-01	0.000	0.000	0.000	0.000	0.000
4.2.1.59|g__Klebsiella.s__Klebsiella_oxytoca	-	8.32E-01	0.000	0.000	0.000	0.004	0.002
4.2.1.59|g__Klebsiella.s__Klebsiella_pneumoniae	+	9.93E-01	0.000	0.000	0.000	0.003	0.009
4.2.1.59|g__Klebsiella.s__Klebsiella_variicola	-	7.03E-01	0.000	0.000	0.000	0.001	0.000
4.2.1.59|g__Lachnoclostridium.s__Clostridium_aldenense	+	5.23E-01	0.000	0.000	0.000	0.000	0.001
4.2.1.59|g__Lachnoclostridium.s__Clostridium_asparagiforme	+	7.04E-01	0.000	0.000	0.000	0.000	0.000
4.2.1.59|g__Lachnoclostridium.s__Clostridium_bolteae	+	5.07E-01	0.000	0.000	0.000	0.000	0.001
4.2.1.59|g__Lachnoclostridium.s__Clostridium_citroniae	+	9.52E-01	0.000	0.000	0.000	0.000	0.000
4.2.1.59|g__Lachnoclostridium.s__Clostridium_clostridioforme	+	6.90E-01	0.000	0.000	0.000	0.000	0.000
4.2.1.59|g__Lachnoclostridium.s__Clostridium_hylemonae	+	3.85E-01	0.000	0.000	0.000	0.000	0.000
4.2.1.59|g__Lachnoclostridium.s__Clostridium_lavalense	+	1.72E-01	0.000	0.000	0.000	0.000	0.000
4.2.1.59|g__Lachnoclostridium.s__Clostridium_saccharolyticum	-	7.24E-01	0.000	0.000	0.000	0.000	0.000
4.2.1.59|g__Lachnoclostridium.s__Clostridium_scindens	+	3.43E-01	0.000	0.000	0.000	0.001	0.002
4.2.1.59|g__Lachnoclostridium.s__Clostridium_symbiosum	-	4.81E-01	0.000	0.000	0.000	0.000	0.000
4.2.1.59|g__Lachnoclostridium.s__Lachnoclostridium_sp_An118	+	8.70E-01	0.000	0.000	0.000	0.000	0.001
4.2.1.59|g__Lachnoclostridium.s__Lachnoclostridium_sp_An14	-	2.62E-01	0.000	0.000	0.000	0.000	0.000
4.2.1.59|g__Lachnoclostridium.s__Lachnoclostridium_sp_An76	+	3.85E-01	0.000	0.000	0.000	0.000	0.000
4.2.1.59|g__Lachnospira.s__Lachnospira_pectinoschiza	-	5.34E-01	0.000	0.000	0.000	0.002	0.002
4.2.1.59|g__Lachnospiraceae_unclassified.s__Lachnospiraceae_bacterium_2	-	1.09E-01	0.000	0.000	0.000	0.000	0.000
4.2.1.59|g__Lachnospiraceae_unclassified.s__Lachnospiraceae_bacterium_(	+	2.72E-01	0.000	0.000	0.000	0.000	0.000
4.2.1.59|g__Lachnospiraceae_unclassified.s__Lachnospiraceae_bacterium_(	-	2.62E-01	0.000	0.000	0.000	0.000	0.000
4.2.1.59|g__Lactobacillus.s__Lactobacillus_antri	+	1.26E-01	0.000	0.000	0.000	0.000	0.000
4.2.1.59|g__Lactobacillus.s__Lactobacillus_casei_group	+	8.44E-02	0.000	0.000	0.000	0.000	0.001
4.2.1.59|g__Lactobacillus.s__Lactobacillus_delbrueckii	-	2.62E-01	0.000	0.000	0.000	0.000	0.000
4.2.1.59|g__Lactobacillus.s__Lactobacillus_fermentum	+	1.35E-01	0.000	0.000	0.000	0.001	0.003
4.2.1.59|g__Lactobacillus.s__Lactobacillus_kimbladii	+	3.85E-01	0.000	0.000	0.000	0.000	0.000
4.2.1.59|g__Lactobacillus.s__Lactobacillus_kullabergensis	+	3.85E-01	0.000	0.000	0.000	0.000	0.000
4.2.1.59|g__Lactobacillus.s__Lactobacillus_mucosae	+	1.42E-01	0.000	0.000	0.000	0.004	0.009
4.2.1.59|g__Lactobacillus.s__Lactobacillus_oris	+	3.07E-01	0.000	0.000	0.000	0.001	0.007
4.2.1.59|g__Lactobacillus.s__Lactobacillus_paracasei	+	1.26E-01	0.000	0.000	0.000	0.000	0.001
4.2.1.59|g__Lactobacillus.s__Lactobacillus_paraplantarum	-	4.32E-01	0.000	0.000	0.000	0.000	0.000
4.2.1.59|g__Lactobacillus.s__Lactobacillus_plantarum	+	4.61E-01	0.000	0.000	0.000	0.000	0.000
4.2.1.59|g__Lactobacillus.s__Lactobacillus_reuteri	+	3.85E-01	0.000	0.000	0.000	0.000	0.000
4.2.1.59|g__Lactobacillus.s__Lactobacillus_rhamnosus	+	3.85E-01	0.000	0.000	0.000	0.000	0.000
4.2.1.59|g__Lactobacillus.s__Lactobacillus_rogosae	-	1.58E-01	0.000	0.000	0.000	0.002	0.002
4.2.1.59|g__Lactobacillus.s__Lactobacillus_ruminis	-	8.71E-01	0.000	0.000	0.000	0.000	0.001
4.2.1.59|g__Lactobacillus.s__Lactobacillus_sakei	-	2.62E-01	0.000	0.000	0.000	0.000	0.000
4.2.1.59|g__Lactobacillus.s__Lactobacillus_salivarius	+	2.24E-02	0.000	0.000	0.000	0.003	0.021
4.2.1.59|g__Lactobacillus.s__Lactobacillus_sanfranciscensis	+	3.85E-01	0.000	0.000	0.000	0.000	0.000
4.2.1.59|g__Lactococcus.s__Lactococcus_garvieae	-	2.62E-01	0.000	0.000	0.000	0.000	0.000
4.2.1.59|g__Lactococcus.s__Lactococcus_lactis	+	7.36E-01	0.000	0.000	0.000	0.001	0.001
4.2.1.59|g__Lawsonibacter.s__Lawsonibacter_asaccharolyticus	-	7.16E-01	0.000	0.000	0.000	0.002	0.002
4.2.1.59|g__Leclercia.s__Leclercia_adecarboxylata	-	1.99E-01	0.000	0.000	0.000	0.000	0.000
4.2.1.59|g__Lelliottia.s__Lelliottia_nimipressuralis	-	1.99E-01	0.000	0.000	0.000	0.000	0.000
4.2.1.59|g__Leuconostoc.s__Leuconostoc_garlicum	+	2.14E-01	0.000	0.000	0.000	0.000	0.000
4.2.1.59|g__Leuconostoc.s__Leuconostoc_lactis	+	2.14E-01	0.000	0.000	0.000	0.000	0.000
4.2.1.59|g__Leuconostoc.s__Leuconostoc_mesenteroides	-	2.62E-01	0.000	0.000	0.000	0.000	0.000
4.2.1.59|g__Listeria.s__Listeria_monocytogenes	-	4.32E-01	0.000	0.000	0.000	0.000	0.003
4.2.1.59|g__Megamonas.s__Megamonas_funiformis	-	8.02E-01	0.000	0.000	0.000	0.004	0.005
4.2.1.59|g__Megamonas.s__Megamonas_hypermegale	+	9.61E-01	0.000	0.000	0.000	0.004	0.002
4.2.1.59|g__Megamonas.s__Megamonas_rupellensis	+	8.78E-01	0.000	0.000	0.000	0.009	0.003
4.2.1.59|g__Megasphaera.s__Megasphaera_elsdenii	-	8.71E-01	0.000	0.000	0.000	0.000	0.000
4.2.1.59|g__Megasphaera.s__Megasphaera_hexanoica	+	3.85E-01	0.000	0.000	0.000	0.000	0.000
4.2.1.59|g__Megasphaera.s__Megasphaera_micronuciformis	-	2.62E-01	0.000	0.000	0.000	0.000	0.000
4.2.1.59|g__Megasphaera.s__Megasphaera_sp_DISK_18	-	1.26E-01	0.000	0.000	0.000	0.004	0.000
4.2.1.59|g__Megasphaera.s__Megasphaera_sp_MJR8396C	-	7.68E-01	0.000	0.000	0.000	0.001	0.001
4.2.1.59|g__Megasphaera.s__Megasphaera_stantonii	+	7.21E-01	0.000	0.000	0.000	0.000	0.000
4.2.1.59|g__Mesosutterella.s__Mesosutterella_multiformis	-	1.01E-01	0.000	0.000	0.000	0.000	0.000
4.2.1.59|g__Mitsuokella.s__Mitsuokella_jalaludinii	+	3.85E-01	0.000	0.000	0.000	0.000	0.000
4.2.1.59|g__Mitsuokella.s__Mitsuokella_multacida	+	2.14E-01	0.000	0.000	0.000	0.000	0.000
4.2.1.59|g__Monoglobus.s__Monoglobus_pectinilyticus	-	6.85E-01	0.000	0.000	0.000	0.000	0.000
4.2.1.59|g__Odoribacter.s__Odoribacter_laneus	+	6.72E-01	0.000	0.000	0.000	0.000	0.001
4.2.1.59|g__Odoribacter.s__Odoribacter_splanchnicus	+	6.20E-02	0.000	0.000	0.000	0.001	0.002

4.2.1.59|g__Oxalobacter.s__Oxalobacter_formigenes	-	2.62E-01	0.000	0.000	0.000	0.000	0.000
4.2.1.59|g__Pantoea.s__Pantoea_sesami	-	2.56E-01	0.000	0.000	0.000	0.000	0.000
4.2.1.59|g__Parabacteroides.s__Parabacteroides_goldsteinii	+	2.93E-01	0.000	0.000	0.000	0.000	0.001
4.2.1.59|g__Parabacteroides.s__Parabacteroides_gordonii	+	1.26E-01	0.000	0.000	0.000	0.000	0.000
4.2.1.59|g__Parabacteroides.s__Parabacteroides_johnsonii	+	5.45E-01	0.000	0.000	0.000	0.001	0.001
4.2.1.59|g__Paraprevotella.s__Paraprevotella_clara	+	5.49E-01	0.000	0.000	0.000	0.001	0.001
4.2.1.59|g__Paraprevotella.s__Paraprevotella_xylaniphila	+	5.99E-01	0.000	0.000	0.000	0.000	0.000
4.2.1.59|g__Parasutterella.s__Parasutterella_excrementihominis	-	2.97E-01	0.000	0.000	0.000	0.001	0.001
4.2.1.59|g__Pediococcus.s__Pediococcus_acidilactici	+	8.96E-01	0.000	0.000	0.000	0.000	0.000
4.2.1.59|g__Peptostreptococcaceae_unclassified.s__Clostridium_hiranonis	-	2.62E-01	0.000	0.000	0.000	0.000	0.000
4.2.1.59|g__Phascolarctobacterium.s__Phascolarctobacterium_faecium	+	2.05E-01	0.000	0.000	0.000	0.003	0.006
4.2.1.59|g__Phascolarctobacterium.s__Phascolarctobacterium_succinatutens	-	6.68E-01	0.000	0.000	0.000	0.005	0.004
4.2.1.59|g__Prevotella.s__Prevotella_buccae	-	1.09E-01	0.000	0.000	0.000	0.000	0.000
4.2.1.59|g__Prevotella.s__Prevotella_sp_109	-	1.01E-01	0.000	0.000	0.000	0.002	0.001
4.2.1.59|g__Proteus.s__Proteus_vulgaris	+	3.85E-01	0.000	0.000	0.000	0.000	0.000
4.2.1.59|g__Pseudoflavonifractor.s__Pseudoflavonifractor_capillosus	+	3.85E-01	0.000	0.000	0.000	0.000	0.000
4.2.1.59|g__Pseudoflavonifractor.s__Pseudoflavonifractor_sp_An184	+	2.83E-02	0.000	0.000	0.000	0.001	0.003
4.2.1.59|g__Pyramidobacter.s__Pyramidobacter_piscolens	+	2.14E-01	0.000	0.000	0.000	0.000	0.000
4.2.1.59|g__Raoultella.s__Raoultella_ornithinolytica	-	2.44E-01	0.000	0.000	0.000	0.004	0.000
4.2.1.59|g__Raoultella.s__Raoultella_planticola	-	1.09E-01	0.000	0.000	0.000	0.001	0.000
4.2.1.59|g__Rikenella.s__Rikenella_microfusus	+	1.26E-01	0.000	0.000	0.000	0.000	0.000
4.2.1.59|g__Roseburia.s__Roseburia_faecis	-	1.46E-03	0.000	0.000	0.000	0.015	0.005
4.2.1.59|g__Roseburia.s__Roseburia_hominis	-	7.50E-01	0.000	0.000	0.000	0.004	0.003
4.2.1.59|g__Roseburia.s__Roseburia_intestinalis	-	7.01E-04	0.000	0.000	0.000	0.011	0.002
4.2.1.59|g__Roseburia.s__Roseburia_inulinivorans	+	9.65E-01	0.000	0.000	0.000	0.005	0.005
4.2.1.59|g__Rubneribacter.s__Rubneribacter_badeniensis	+	3.85E-01	0.000	0.000	0.000	0.000	0.000
4.2.1.59|g__Ruminococcaceae_unclassified.s__Clostridium_methylpentosun	+	3.85E-01	0.000	0.000	0.000	0.000	0.000
4.2.1.59|g__Ruminococcaceae_unclassified.s__Ruminococcaceae_bacteriun	+	1.60E-01	0.000	0.000	0.000	0.000	0.001
4.2.1.59|g__Ruminococcus.s__Ruminococcus_lactaris	-	1.95E-01	0.000	0.000	0.000	0.002	0.001
4.2.1.59|g__Ruminococcus.s__Ruminococcus_sp_AF31_8BH	+	4.10E-01	0.000	0.000	0.000	0.000	0.001
4.2.1.59|g__Salmonella.s__Salmonella_enterica	-	7.80E-01	0.000	0.000	0.000	0.001	0.001
4.2.1.59|g__Sellimonas.s__Sellimonas_intestinalis	+	5.43E-01	0.000	0.000	0.000	0.002	0.003
4.2.1.59|g__Staphylococcus.s__Staphylococcus_aureus	+	3.85E-01	0.000	0.000	0.000	0.000	0.000
4.2.1.59|g__Streptococcus.s__Streptococcus_anginosus_group	+	1.26E-01	0.000	0.000	0.000	0.000	0.000
4.2.1.59|g__Streptococcus.s__Streptococcus_australis	-	2.91E-01	0.000	0.000	0.000	0.000	0.000
4.2.1.59|g__Streptococcus.s__Streptococcus_constellatus	-	2.62E-01	0.000	0.000	0.000	0.000	0.000
4.2.1.59|g__Streptococcus.s__Streptococcus_cristatus	+	8.78E-01	0.000	0.000	0.000	0.000	0.000
4.2.1.59|g__Streptococcus.s__Streptococcus_downei	+	4.67E-02	0.000	0.000	0.000	0.000	0.000
4.2.1.59|g__Streptococcus.s__Streptococcus_equinus	-	4.88E-02	0.000	0.000	0.000	0.002	0.000
4.2.1.59|g__Streptococcus.s__Streptococcus_gallolyticus	-	4.80E-01	0.000	0.000	0.000	0.000	0.000
4.2.1.59|g__Streptococcus.s__Streptococcus_gordonii	+	3.89E-01	0.000	0.000	0.000	0.000	0.000
4.2.1.59|g__Streptococcus.s__Streptococcus_infantarius	-	1.09E-01	0.000	0.000	0.000	0.001	0.000
4.2.1.59|g__Streptococcus.s__Streptococcus_infantis	-	2.69E-01	0.000	0.000	0.000	0.000	0.000
4.2.1.59|g__Streptococcus.s__Streptococcus_lutetiensis	-	4.88E-02	0.000	0.000	0.000	0.002	0.000
4.2.1.59|g__Streptococcus.s__Streptococcus_macedonicus	+	5.99E-01	0.000	0.000	0.000	0.000	0.000
4.2.1.59|g__Streptococcus.s__Streptococcus_milleri	-	8.03E-01	0.000	0.000	0.000	0.000	0.000
4.2.1.59|g__Streptococcus.s__Streptococcus_mitis	-	2.70E-01	0.000	0.000	0.000	0.000	0.000
4.2.1.59|g__Streptococcus.s__Streptococcus_mutans	+	1.13E-04	0.000	0.000	0.000	0.000	0.002
4.2.1.59|g__Streptococcus.s__Streptococcus_oralis	-	1.56E-01	0.000	0.000	0.000	0.000	0.000
4.2.1.59|g__Streptococcus.s__Streptococcus_parasanguinis	-	3.96E-01	0.000	0.000	0.000	0.010	0.007
4.2.1.59|g__Streptococcus.s__Streptococcus_pasteurianus	-	7.60E-01	0.000	0.000	0.000	0.001	0.001
4.2.1.59|g__Streptococcus.s__Streptococcus_pneumoniae	-	1.27E-01	0.000	0.000	0.000	0.000	0.000
4.2.1.59|g__Streptococcus.s__Streptococcus_pseudopneumoniae	-	8.71E-01	0.000	0.000	0.000	0.000	0.000
4.2.1.59|g__Streptococcus.s__Streptococcus_sanguinis	-	4.24E-01	0.000	0.000	0.000	0.000	0.000
4.2.1.59|g__Streptococcus.s__Streptococcus_sobrinus	+	1.69E-01	0.000	0.000	0.000	0.000	0.001
4.2.1.59|g__Streptococcus.s__Streptococcus_sp_A12	-	6.03E-02	0.000	0.000	0.000	0.001	0.000
4.2.1.59|g__Streptococcus.s__Streptococcus_thermophilus	+	4.27E-01	0.000	0.000	0.000	0.004	0.006
4.2.1.59|g__Streptococcus.s__Streptococcus_vestibularis	+	2.42E-02	0.000	0.000	0.000	0.002	0.001
4.2.1.59|g__Streptococcus.s__Streptococcus_viridans	+	2.90E-02	0.000	0.000	0.000	0.000	0.002
4.2.1.59|g__Succinatimonas.s__Succinatimonas_hippei	+	7.21E-01	0.000	0.000	0.000	0.000	0.000
4.2.1.59|g__Sutterella.s__Sutterella_parvirubra	+	3.85E-01	0.000	0.000	0.000	0.000	0.000
4.2.1.59|g__Sutterella.s__Sutterella_wadsworthensis	-	7.66E-01	0.000	0.000	0.000	0.003	0.004
4.2.1.59|g__Terrisporobacter.s__Terrisporobacter_othiniensis	+	3.85E-01	0.000	0.000	0.000	0.000	0.000
4.2.1.59|g__Turicibacter.s__Turicibacter_sanguinis	-	4.39E-01	0.000	0.000	0.000	0.001	0.000
4.2.1.59|g__Tyzzerella.s__Tyzzerella_nexilis	-	7.85E-01	0.000	0.000	0.000	0.005	0.005
4.2.1.59|g__Veillonella.s__Veillonella_atypica	-	2.62E-02	0.000	0.000	0.000	0.002	0.000
4.2.1.59|g__Veillonella.s__Veillonella_denticariosi	+	2.14E-01	0.000	0.000	0.000	0.000	0.000
4.2.1.59|g__Veillonella.s__Veillonella_dispar	-	1.60E-03	0.000	0.000	0.000	0.002	0.000
4.2.1.59|g__Veillonella.s__Veillonella_infantium	-	1.05E-02	0.000	0.000	0.000	0.000	0.000
4.2.1.59|g__Veillonella.s__Veillonella_parvula	-	3.57E-01	0.000	0.000	0.000	0.001	0.001
4.2.1.59|g__Veillonella.s__Veillonella_rodentium	-	2.62E-01	0.000	0.000	0.000	0.000	0.000
4.2.1.59|g__Veillonella.s__Veillonella_rogosae	-	2.27E-03	0.000	0.000	0.000	0.000	0.000

	4.2.1.59|g__Veillonella.s__Veillonella_tobetsuensis	-	4.88E-02	0.000	0.000	0.000	0.000	0.000
	4.2.1.59|g__Weissella.s__Weissella_cibaria	+	2.87E-01	0.000	0.000	0.000	0.000	0.001
	4.2.1.59|g__Weissella.s__Weissella_confusa	+	3.85E-01	0.000	0.000	0.000	0.000	0.000
	4.2.1.59|g__Yokenella.s__Yokenella_regensburgei	-	4.24E-01	0.000	0.000	0.000	0.000	0.000
	4.2.1.59|g__Bacteroides.s__Bacteroides_ovatus	+	9.91E-01	0.372	0.372	0.000	0.008	0.010
	4.2.1.59|g__Bacteroides.s__Bacteroides_caccae	+	2.42E-01	0.000	0.024	0.024	0.003	0.005
	4.2.1.59|g__Bacteroides.s__Bacteroides_xylanisolvens	+	7.79E-01	0.070	0.113	0.043	0.006	0.007
	4.2.1.59|g__Bacteroides.s__Bacteroides_intestinalis	+	8.67E-03	0.000	0.054	0.054	0.002	0.004
	4.2.1.59|g__Streptococcus.s__Streptococcus_salivarius	+	9.81E-01	0.858	0.923	0.065	0.026	0.024
	4.2.1.59|g__Bacteroides.s__Bacteroides_uniformis	+	5.50E-01	3.053	3.176	0.123	0.027	0.033
	4.2.1.59|g__Bacteroides.s__Bacteroides_dorei	+	7.62E-01	0.888	1.033	0.145	0.024	0.021
	4.2.1.59|g__Bacteroides.s__Bacteroides_thetaiotaomicron	+	3.63E-01	0.378	0.530	0.152	0.007	0.010
	4.2.1.59|g__Eubacterium.s__Eubacterium_eligens	+	1.34E-01	0.000	0.192	0.192	0.008	0.014
	4.2.1.59|g__Alistipes.s__Alistipes_finegoldii	+	5.83E-04	0.000	0.194	0.194	0.004	0.012
	4.2.1.59|g__Parabacteroides.s__Parabacteroides_merdae	+	4.09E-01	0.340	0.575	0.235	0.006	0.006
	4.2.1.59|g__Parabacteroides.s__Parabacteroides_distasonis	+	1.18E-02	0.624	1.037	0.412	0.008	0.011
	4.2.1.59|g__Alistipes.s__Alistipes_shahii	+	4.33E-03	0.000	0.573	0.573	0.003	0.009
	4.2.1.59|g__Bifidobacterium.s__Bifidobacterium_longum	+	5.52E-02	1.312	2.455	1.143	0.026	0.042
	4.2.1.59|g__Alistipes.s__Alistipes_putredinis	+	1.62E-02	0.000	1.479	1.479	0.008	0.016
USA	4.2.1.59	-	1.72E-02	128.331	123.835	-4.496	1.000	1.000
	4.2.1.59|g__Faecalibacterium.s__Faecalibacterium_prausnitzii	-	2.09E-05	8.537	2.901	-5.637	0.079	0.058
	4.2.1.59|g__Blautia.s__Blautia_obeum	-	2.85E-07	7.269	5.071	-2.199	0.069	0.054
	4.2.1.59|g__Bacteroides.s__Bacteroides_vulgatus	-	7.32E-02	6.387	4.459	-1.929	0.064	0.056
	4.2.1.59|g__Blautia.s__Ruminococcus_torques	-	5.66E-03	4.290	3.302	-0.987	0.044	0.036
	4.2.1.59|g__Anaerostipes.s__Anaerostipes_hadrus	-	1.31E-03	1.451	0.561	-0.890	0.018	0.014
	4.2.1.59|g__Lachnospiraceae_unclassified.s__Eubacterium_rectale	-	2.37E-03	0.983	0.157	-0.827	0.023	0.017
	4.2.1.59|g__Fusicatenibacter.s__Fusicatenibacter_saccharivorans	-	1.60E-05	1.414	0.595	-0.819	0.016	0.011
	4.2.1.59|unclassified	-	3.15E-01	17.736	17.032	-0.703	0.145	0.148
	4.2.1.59|g__Bacteroides.s__Bacteroides_uniformis	-	6.83E-01	3.506	3.029	-0.476	0.037	0.038
	4.2.1.59|g__Blautia.s__Blautia_wexlerae	-	7.89E-06	0.894	0.504	-0.390	0.016	0.011
	4.2.1.59|g__Dorea.s__Dorea_longicatena	-	6.87E-02	0.366	0.000	-0.366	0.010	0.008
	4.2.1.59|g__Alistipes.s__Alistipes_putredinis	-	5.25E-01	2.374	2.135	-0.239	0.021	0.021
	4.2.1.59|g__Lawsonibacter.s__Lawsonibacter_asaccharolyticus	-	1.75E-02	0.149	0.000	-0.149	0.003	0.002
	4.2.1.59|g__Bacteroides.s__Bacteroides_dorei	-	7.55E-01	0.809	0.703	-0.106	0.018	0.019
	4.2.1.59|g__Bacteroides.s__Bacteroides_ovatus	+	7.42E-01	0.422	0.370	-0.052	0.014	0.012
	4.2.1.59|g__Roseburia.s__Roseburia_intestinalis	-	5.62E-06	0.034	0.000	-0.034	0.008	0.004
	4.2.1.59|g__Acidaminococcus.s__Acidaminococcus_fermentans	+	2.32E-01	0.000	0.000	0.000	0.000	0.000
	4.2.1.59|g__Acidaminococcus.s__Acidaminococcus_intestini	+	4.45E-03	0.000	0.000	0.000	0.002	0.004
	4.2.1.59|g__Acidovorax.s__Acidovorax_temperans	+	4.92E-01	0.000	0.000	0.000	0.000	0.000
	4.2.1.59|g__Adlercreutzia.s__Adlercreutzia_equolifaciens	-	5.34E-01	0.000	0.000	0.000	0.001	0.001
	4.2.1.59|g__Aeromonas.s__Aeromonas_caviae	+	4.92E-01	0.000	0.000	0.000	0.000	0.000
	4.2.1.59|g__Aeromonas.s__Aeromonas_diversa	-	1.48E-01	0.000	0.000	0.000	0.000	0.000
	4.2.1.59|g__Aeromonas.s__Aeromonas_veronii	-	1.48E-01	0.000	0.000	0.000	0.000	0.000
	4.2.1.59|g__Aggregatibacter.s__Aggregatibacter_segnis	+	4.92E-01	0.000	0.000	0.000	0.000	0.000
	4.2.1.59|g__Akkermansia.s__Akkermansia_muciniphila	+	2.09E-01	0.000	0.000	0.000	0.020	0.034
	4.2.1.59|g__Alistipes.s__Alistipes_indistinctus	+	1.53E-04	0.000	0.000	0.000	0.000	0.001
	4.2.1.59|g__Alistipes.s__Alistipes_inops	+	1.71E-01	0.000	0.000	0.000	0.000	0.001
	4.2.1.59|g__Alistipes.s__Alistipes_sp_An66	-	1.48E-01	0.000	0.000	0.000	0.000	0.000
	4.2.1.59|g__Alistipes.s__Alistipes_timonensis	+	6.71E-01	0.000	0.000	0.000	0.000	0.000
	4.2.1.59|g__Allisonella.s__Allisonella_histaminiformans	-	9.27E-01	0.000	0.000	0.000	0.000	0.000
	4.2.1.59|g__Anaerofilum.s__Anaerofilum_sp_An201	+	7.60E-01	0.000	0.000	0.000	0.000	0.000
	4.2.1.59|g__Anaerofustis.s__Anaerofustis_stercorihominis	-	4.06E-02	0.000	0.000	0.000	0.000	0.000
	4.2.1.59|g__Anaeroglobus.s__Anaeroglobus_geminatus	+	6.64E-01	0.000	0.000	0.000	0.000	0.000
	4.2.1.59|g__Anaerostipes.s__Anaerostipes_caccae	+	1.28E-01	0.000	0.000	0.000	0.000	0.000
	4.2.1.59|g__Anaerostipes.s__Anaerostipes_sp_494a	-	1.48E-01	0.000	0.000	0.000	0.000	0.000
	4.2.1.59|g__Anaerotruncus.s__Anaerotruncus_colihominis	+	8.85E-03	0.000	0.000	0.000	0.000	0.000
	4.2.1.59|g__Asaccharobacter.s__Asaccharobacter_celatus	-	4.16E-02	0.000	0.000	0.000	0.004	0.003
	4.2.1.59|g__Bacteroides.s__Bacteroides_clarus	+	4.18E-01	0.000	0.000	0.000	0.001	0.001
	4.2.1.59|g__Bacteroides.s__Bacteroides_coprocola	-	1.03E-01	0.000	0.000	0.000	0.003	0.002
	4.2.1.59|g__Bacteroides.s__Bacteroides_coprophilus	+	3.19E-01	0.000	0.000	0.000	0.000	0.000
	4.2.1.59|g__Bacteroides.s__Bacteroides_eggerthii	+	9.14E-01	0.000	0.000	0.000	0.010	0.009
	4.2.1.59|g__Bacteroides.s__Bacteroides_faecichinchillae	+	1.22E-01	0.000	0.000	0.000	0.000	0.000
	4.2.1.59|g__Bacteroides.s__Bacteroides_faecis	+	6.32E-02	0.000	0.000	0.000	0.002	0.002
	4.2.1.59|g__Bacteroides.s__Bacteroides_finegoldii	+	4.58E-01	0.000	0.000	0.000	0.002	0.002
	4.2.1.59|g__Bacteroides.s__Bacteroides_fluxus	+	2.32E-01	0.000	0.000	0.000	0.000	0.000
	4.2.1.59|g__Bacteroides.s__Bacteroides_fragilis	-	7.55E-01	0.000	0.000	0.000	0.004	0.004
	4.2.1.59|g__Bacteroides.s__Bacteroides_massiliensis	+	4.77E-01	0.000	0.000	0.000	0.009	0.008
	4.2.1.59|g__Bacteroides.s__Bacteroides_nordii	-	3.79E-01	0.000	0.000	0.000	0.001	0.001
	4.2.1.59|g__Bacteroides.s__Bacteroides_oleiciplenus	+	4.92E-01	0.000	0.000	0.000	0.000	0.000
	4.2.1.59|g__Bacteroides.s__Bacteroides_plebeius	-	3.08E-01	0.000	0.000	0.000	0.010	0.003
	4.2.1.59|g__Bacteroides.s__Bacteroides_salyersiae	+	2.94E-02	0.000	0.000	0.000	0.001	0.002
	4.2.1.59|g__Bacteroides.s__Bacteroides_sartorii	-	4.06E-02	0.000	0.000	0.000	0.000	0.000

4.2.1.59|g__Bacteroides.s__Bacteroides_sp_OM08_11	+	4.92E-01	0.000	0.000	0.000	0.000	0.000
4.2.1.59|g__Bacteroides.s__Bacteroides_stercorirosoris	-	9.55E-01	0.000	0.000	0.000	0.000	0.000
4.2.1.59|g__Barnesiella.s__Barnesiella_intestinihominis	+	1.19E-01	0.000	0.000	0.000	0.003	0.003
4.2.1.59|g__Bifidobacterium.s__Bifidobacterium_animalis	+	9.94E-01	0.000	0.000	0.000	0.001	0.001
4.2.1.59|g__Bifidobacterium.s__Bifidobacterium_breve	+	1.83E-02	0.000	0.000	0.000	0.000	0.001
4.2.1.59|g__Blautia.s__Blautia_hydrogenotrophica	-	3.71E-02	0.000	0.000	0.000	0.001	0.001
4.2.1.59|g__Blautia.s__Blautia_sp_AF19_10LB	+	7.13E-01	0.000	0.000	0.000	0.001	0.001
4.2.1.59|g__Blautia.s__Blautia_sp_An249	-	3.56E-01	0.000	0.000	0.000	0.000	0.000
4.2.1.59|g__Blautia.s__Ruminococcus_gnavus	-	1.19E-01	0.000	0.000	0.000	0.010	0.006
4.2.1.59|g__Butyrivibrio.s__Butyrivibrio_crossotus	-	3.41E-01	0.000	0.000	0.000	0.001	0.001
4.2.1.59|g__Campylobacter.s__Campylobacter_curvus	+	4.92E-01	0.000	0.000	0.000	0.000	0.000
4.2.1.59|g__Campylobacter.s__Campylobacter_gracilis	-	6.30E-01	0.000	0.000	0.000	0.000	0.000
4.2.1.59|g__Campylobacter.s__Campylobacter_ureolyticus	+	4.92E-01	0.000	0.000	0.000	0.000	0.000
4.2.1.59|g__Candida.s__Candida_albicans	-	9.67E-01	0.000	0.000	0.000	0.000	0.000
4.2.1.59|g__Candidatus_Gastranaerophilales_unclassified.s__Candidatus_Ga	+	6.68E-02	0.000	0.000	0.000	0.000	0.000
4.2.1.59|g__Catabacter.s__Catabacter_hongkongensis	+	2.27E-01	0.000	0.000	0.000	0.000	0.000
4.2.1.59|g__Catenibacterium.s__Catenibacterium_mitsuokai	+	4.56E-01	0.000	0.000	0.000	0.002	0.003
4.2.1.59|g__Cetobacterium.s__Cetobacterium_somerae	+	4.92E-01	0.000	0.000	0.000	0.000	0.000
4.2.1.59|g__Citrobacter.s__Citrobacter_amalonaticus	+	3.14E-01	0.000	0.000	0.000	0.000	0.000
4.2.1.59|g__Citrobacter.s__Citrobacter_braakii	-	7.12E-01	0.000	0.000	0.000	0.000	0.000
4.2.1.59|g__Citrobacter.s__Citrobacter_farmeri	+	9.00E-02	0.000	0.000	0.000	0.000	0.000
4.2.1.59|g__Citrobacter.s__Citrobacter_freundii	+	7.10E-01	0.000	0.000	0.000	0.001	0.000
4.2.1.59|g__Citrobacter.s__Citrobacter_koseri	+	3.30E-01	0.000	0.000	0.000	0.000	0.000
4.2.1.59|g__Citrobacter.s__Citrobacter_portucalensis	+	3.21E-01	0.000	0.000	0.000	0.000	0.000
4.2.1.59|g__Citrobacter.s__Citrobacter_werkmanii	+	3.30E-01	0.000	0.000	0.000	0.000	0.000
4.2.1.59|g__Citrobacter.s__Citrobacter_youngae	+	6.59E-01	0.000	0.000	0.000	0.000	0.000
4.2.1.59|g__Cloacibacillus.s__Cloacibacillus_porcorum	+	3.84E-01	0.000	0.000	0.000	0.000	0.001
4.2.1.59|g__Clostridiales_Family_XIII_Incertae_Sedis_unclassified.s__Euba	+	4.92E-01	0.000	0.000	0.000	0.000	0.000
4.2.1.59|g__Clostridiales_unclassified.s__Bacteroides_pectinophilus	-	9.70E-01	0.000	0.000	0.000	0.000	0.000
4.2.1.59|g__Clostridiales_unclassified.s__Clostridiales_bacterium_1_7_47F/	-	8.30E-01	0.000	0.000	0.000	0.000	0.000
4.2.1.59|g__Clostridiales_unclassified.s__Clostridiales_bacterium_CHKCI0(	+	4.92E-01	0.000	0.000	0.000	0.000	0.000
4.2.1.59|g__Clostridiales_unclassified.s__Clostridiales_bacterium_KLE1615	-	2.71E-06	0.000	0.000	0.000	0.004	0.002
4.2.1.59|g__Clostridioides.s__Clostridioides_difficile	+	3.19E-01	0.000	0.000	0.000	0.001	0.002
4.2.1.59|g__Clostridium.s__Butyribacterium_methylotrophicum	+	5.99E-04	0.000	0.000	0.000	0.000	0.001
4.2.1.59|g__Clostridium.s__Clostridium_butyricum	-	1.48E-01	0.000	0.000	0.000	0.000	0.000
4.2.1.59|g__Clostridium.s__Clostridium_cadaveris	-	5.95E-01	0.000	0.000	0.000	0.000	0.000
4.2.1.59|g__Clostridium.s__Clostridium_celatum	-	1.48E-01	0.000	0.000	0.000	0.000	0.000
4.2.1.59|g__Clostridium.s__Clostridium_disporicum	-	7.24E-01	0.000	0.000	0.000	0.000	0.000
4.2.1.59|g__Clostridium.s__Clostridium_neonatale	-	5.95E-01	0.000	0.000	0.000	0.000	0.000
4.2.1.59|g__Clostridium.s__Clostridium_paraputrificum	+	2.28E-01	0.000	0.000	0.000	0.000	0.000
4.2.1.59|g__Clostridium.s__Clostridium_perfringens	-	4.29E-01	0.000	0.000	0.000	0.000	0.000
4.2.1.59|g__Clostridium.s__Clostridium_sp_AF36_4	-	1.41E-01	0.000	0.000	0.000	0.002	0.002
4.2.1.59|g__Clostridium.s__Clostridium_sp_AM22_11AC	-	7.78E-03	0.000	0.000	0.000	0.003	0.002
4.2.1.59|g__Clostridium.s__Clostridium_sp_D5	+	1.22E-01	0.000	0.000	0.000	0.000	0.000
4.2.1.59|g__Clostridium.s__Clostridium_sp_MSTE9	+	4.92E-01	0.000	0.000	0.000	0.000	0.000
4.2.1.59|g__Clostridium.s__Clostridium_sp_chh4_2	+	4.92E-01	0.000	0.000	0.000	0.000	0.000
4.2.1.59|g__Clostridium.s__Clostridium_ventriculi	-	1.48E-01	0.000	0.000	0.000	0.000	0.000
4.2.1.59|g__Comamonas.s__Comamonas_kerstersii	+	3.30E-01	0.000	0.000	0.000	0.000	0.000
4.2.1.59|g__Comamonas.s__Comamonas_thiooxydans	+	4.92E-01	0.000	0.000	0.000	0.000	0.000
4.2.1.59|g__Coprobacillus.s__Coprobacillus_cateniformis	+	7.70E-02	0.000	0.000	0.000	0.000	0.000
4.2.1.59|g__Coprobacter.s__Coprobacter_fastidiosus	-	5.66E-02	0.000	0.000	0.000	0.000	0.000
4.2.1.59|g__Coprobacter.s__Coprobacter_secundus	-	5.50E-01	0.000	0.000	0.000	0.000	0.000
4.2.1.59|g__Coprococcus.s__Coprococcus_catus	+	4.06E-01	0.000	0.000	0.000	0.001	0.001
4.2.1.59|g__Coprococcus.s__Coprococcus_comes	-	8.99E-01	0.000	0.000	0.000	0.004	0.004
4.2.1.59|g__Cronobacter.s__Cronobacter_malonaticus	+	4.92E-01	0.000	0.000	0.000	0.000	0.000
4.2.1.59|g__Cronobacter.s__Cronobacter_sakazakii	+	4.92E-01	0.000	0.000	0.000	0.000	0.000
4.2.1.59|g__Delftia.s__Delftia_lacustris	+	4.92E-01	0.000	0.000	0.000	0.000	0.000
4.2.1.59|g__Delftia.s__Delftia_tsuruhatensis	+	4.92E-01	0.000	0.000	0.000	0.000	0.000
4.2.1.59|g__Desulfovibrio.s__Desulfovibrio_desulfuricans	+	1.67E-01	0.000	0.000	0.000	0.000	0.000
4.2.1.59|g__Desulfovibrio.s__Desulfovibrio_fairfieldensis	+	3.86E-01	0.000	0.000	0.000	0.001	0.002
4.2.1.59|g__Desulfovibrio.s__Desulfovibrio_legallii	+	4.92E-01	0.000	0.000	0.000	0.000	0.000
4.2.1.59|g__Desulfovibrio.s__Desulfovibrio_piger	+	2.54E-01	0.000	0.000	0.000	0.000	0.001
4.2.1.59|g__Desulfovibrio.s__Desulfovibrio_sp_AM18_2	+	3.08E-01	0.000	0.000	0.000	0.000	0.000
4.2.1.59|g__Desulfovibrionaceae_unclassified.s__Desulfovibrionaceae_bact(	+	1.29E-01	0.000	0.000	0.000	0.000	0.000
4.2.1.59|g__Dialister.s__Dialister_invisus	-	4.16E-03	0.000	0.000	0.000	0.003	0.002
4.2.1.59|g__Dialister.s__Dialister_pneumosintes	+	3.30E-01	0.000	0.000	0.000	0.000	0.000
4.2.1.59|g__Dialister.s__Dialister_succinatiphilus	+	4.92E-01	0.000	0.000	0.000	0.000	0.000
4.2.1.59|g__Dorea.s__Dorea_formicigenerans	+	3.22E-01	0.000	0.000	0.000	0.002	0.002
4.2.1.59|g__Dorea.s__Dorea_sp_OM02_2LB	+	6.66E-02	0.000	0.000	0.000	0.000	0.000
4.2.1.59|g__Dysgonomonas.s__Dysgonomonas_mossii	+	4.92E-01	0.000	0.000	0.000	0.000	0.000
4.2.1.59|g__Eisenbergiella.s__Eisenbergiella_massiliensis	+	4.01E-02	0.000	0.000	0.000	0.001	0.001
4.2.1.59|g__Eisenbergiella.s__Eisenbergiella_tayi	+	1.57E-04	0.000	0.000	0.000	0.001	0.002

4.2.1.59|g__Enterobacter.s__Enterobacter_asburiae	+	6.58E-01	0.000	0.000	0.000	0.000	0.000
4.2.1.59|g__Enterobacter.s__Enterobacter_bugandensis	+	3.07E-01	0.000	0.000	0.000	0.000	0.000
4.2.1.59|g__Enterobacter.s__Enterobacter_cloacae	+	9.17E-03	0.000	0.000	0.000	0.000	0.000
4.2.1.59|g__Enterobacter.s__Enterobacter_mori	+	4.92E-01	0.000	0.000	0.000	0.000	0.000
4.2.1.59|g__Enterococcus.s__Enterococcus_asini	+	2.81E-02	0.000	0.000	0.000	0.000	0.000
4.2.1.59|g__Enterococcus.s__Enterococcus_avium	+	1.81E-04	0.000	0.000	0.000	0.000	0.001
4.2.1.59|g__Enterococcus.s__Enterococcus_canintestini	+	1.22E-01	0.000	0.000	0.000	0.000	0.000
4.2.1.59|g__Enterococcus.s__Enterococcus_casseliflavus	+	2.32E-01	0.000	0.000	0.000	0.000	0.000
4.2.1.59|g__Enterococcus.s__Enterococcus_cecorum	+	4.92E-01	0.000	0.000	0.000	0.000	0.000
4.2.1.59|g__Enterococcus.s__Enterococcus_dispar	+	3.74E-02	0.000	0.000	0.000	0.000	0.000
4.2.1.59|g__Enterococcus.s__Enterococcus_durans	+	1.67E-01	0.000	0.000	0.000	0.000	0.000
4.2.1.59|g__Enterococcus.s__Enterococcus_faecalis	+	4.18E-02	0.000	0.000	0.000	0.000	0.000
4.2.1.59|g__Enterococcus.s__Enterococcus_faecium	+	1.95E-02	0.000	0.000	0.000	0.001	0.001
4.2.1.59|g__Enterococcus.s__Enterococcus_gallinarum	+	2.81E-02	0.000	0.000	0.000	0.000	0.000
4.2.1.59|g__Enterococcus.s__Enterococcus_hirae	+	4.92E-01	0.000	0.000	0.000	0.000	0.000
4.2.1.59|g__Enterococcus.s__Enterococcus_malodoratus	+	4.92E-01	0.000	0.000	0.000	0.000	0.000
4.2.1.59|g__Enterococcus.s__Enterococcus_mundtii	+	4.92E-01	0.000	0.000	0.000	0.000	0.000
4.2.1.59|g__Enterococcus.s__Enterococcus_pallens	+	4.92E-01	0.000	0.000	0.000	0.000	0.000
4.2.1.59|g__Enterococcus.s__Enterococcus_raffinosus	+	2.32E-01	0.000	0.000	0.000	0.000	0.000
4.2.1.59|g__Enterococcus.s__Enterococcus_saccharolyticus	+	3.74E-02	0.000	0.000	0.000	0.000	0.000
4.2.1.59|g__Enterococcus.s__Enterococcus_thailandicus	+	4.92E-01	0.000	0.000	0.000	0.000	0.000
4.2.1.59|g__Erysipelatoclostridium.s__Clostridium_innocuum	+	3.08E-01	0.000	0.000	0.000	0.000	0.001
4.2.1.59|g__Erysipelatoclostridium.s__Clostridium_spiroforme	+	3.04E-01	0.000	0.000	0.000	0.000	0.000
4.2.1.59|g__Erysipelatoclostridium.s__Erysipelatoclostridium_ramosum	-	7.12E-01	0.000	0.000	0.000	0.001	0.001
4.2.1.59|g__Erysipelotrichaceae_unclassified.s__Erysipelotrichaceae_bacteri	-	8.95E-01	0.000	0.000	0.000	0.000	0.000
4.2.1.59|g__Escherichia.s__Escherichia_coli	+	7.86E-03	0.000	0.000	0.000	0.022	0.031
4.2.1.59|g__Escherichia.s__Escherichia_fergusonii	+	8.86E-03	0.000	0.000	0.000	0.000	0.001
4.2.1.59|g__Escherichia.s__Escherichia_marmotae	+	4.92E-01	0.000	0.000	0.000	0.000	0.000
4.2.1.59|g__Eubacteriaceae_unclassified.s__Eubacteriaceae_bacterium_CHI	+	6.68E-02	0.000	0.000	0.000	0.000	0.000
4.2.1.59|g__Eubacterium.s__Eubacterium_callanderi	+	1.54E-01	0.000	0.000	0.000	0.000	0.000
4.2.1.59|g__Eubacterium.s__Eubacterium_eligens	-	1.17E-01	0.000	0.000	0.000	0.005	0.004
4.2.1.59|g__Eubacterium.s__Eubacterium_limosum	+	2.46E-01	0.000	0.000	0.000	0.000	0.000
4.2.1.59|g__Eubacterium.s__Eubacterium_maltosivorans	+	8.31E-01	0.000	0.000	0.000	0.000	0.000
4.2.1.59|g__Eubacterium.s__Eubacterium_ramulus	-	1.43E-01	0.000	0.000	0.000	0.001	0.001
4.2.1.59|g__Eubacterium.s__Eubacterium_sp_AF17_7	+	9.49E-01	0.000	0.000	0.000	0.000	0.000
4.2.1.59|g__Eubacterium.s__Eubacterium_sp_An11	+	6.59E-01	0.000	0.000	0.000	0.000	0.000
4.2.1.59|g__Eubacterium.s__Eubacterium_ventriosum	+	9.11E-01	0.000	0.000	0.000	0.000	0.000
4.2.1.59|g__Faecalicatena.s__Faecalicatena_contorta	+	4.44E-01	0.000	0.000	0.000	0.000	0.000
4.2.1.59|g__Faecalicatena.s__Faecalicatena_orotica	+	5.59E-01	0.000	0.000	0.000	0.000	0.000
4.2.1.59|g__Faecalicoccus.s__Faecalicoccus_pleomorphus	+	4.96E-02	0.000	0.000	0.000	0.000	0.001
4.2.1.59|g__Faecalitalea.s__Faecalitalea_cylindroides	+	5.45E-02	0.000	0.000	0.000	0.000	0.002
4.2.1.59|g__Firmicutes_unclassified.s__Firmicutes_bacterium_AF16_15	-	2.37E-02	0.000	0.000	0.000	0.001	0.001
4.2.1.59|g__Flavonifractor.s__Flavonifractor_sp_An10	+	2.00E-01	0.000	0.000	0.000	0.000	0.001
4.2.1.59|g__Flavonifractor.s__Flavonifractor_sp_An100	+	9.00E-02	0.000	0.000	0.000	0.000	0.000
4.2.1.59|g__Fusobacterium.s__Fusobacterium_mortiferum	-	9.55E-01	0.000	0.000	0.000	0.000	0.000
4.2.1.59|g__Fusobacterium.s__Fusobacterium_naviforme	+	4.92E-01	0.000	0.000	0.000	0.000	0.000
4.2.1.59|g__Fusobacterium.s__Fusobacterium_nucleatum	-	9.67E-01	0.000	0.000	0.000	0.000	0.000
4.2.1.59|g__Fusobacterium.s__Fusobacterium_ulcerans	-	6.55E-02	0.000	0.000	0.000	0.000	0.000
4.2.1.59|g__Fusobacterium.s__Fusobacterium_varium	+	4.92E-01	0.000	0.000	0.000	0.000	0.000
4.2.1.59|g__Gardnerella.s__Gardnerella_vaginalis	+	1.54E-01	0.000	0.000	0.000	0.000	0.000
4.2.1.59|g__Gemmiger.s__Gemmiger_formicilis	-	8.43E-01	0.000	0.000	0.000	0.009	0.011
4.2.1.59|g__Gemmiger.s__Gemmiger_sp_An120	+	1.67E-01	0.000	0.000	0.000	0.000	0.000
4.2.1.59|g__Gemmiger.s__Gemmiger_sp_An194	+	4.92E-01	0.000	0.000	0.000	0.000	0.000
4.2.1.59|g__Gemmiger.s__Gemmiger_sp_An50	+	3.30E-01	0.000	0.000	0.000	0.000	0.000
4.2.1.59|g__Globicatella.s__Globicatella_sanguinis	+	4.92E-01	0.000	0.000	0.000	0.000	0.000
4.2.1.59|g__Gordonibacter.s__Gordonibacter_sp_An232A	-	9.70E-01	0.000	0.000	0.000	0.000	0.000
4.2.1.59|g__Haemophilus.s__Haemophilus_parainfluenzae	-	3.96E-01	0.000	0.000	0.000	0.000	0.000
4.2.1.59|g__Haemophilus.s__Haemophilus_sputorum	-	1.48E-01	0.000	0.000	0.000	0.000	0.000
4.2.1.59|g__Hafnia.s__Hafnia_alvei	+	4.92E-01	0.000	0.000	0.000	0.000	0.000
4.2.1.59|g__Hafnia.s__Hafnia_paralvei	-	9.70E-01	0.000	0.000	0.000	0.000	0.000
4.2.1.59|g__Hungatella.s__Hungatella_effluvii	+	4.09E-02	0.000	0.000	0.000	0.000	0.000
4.2.1.59|g__Intestinibacter.s__Intestinibacter_bartlettii	+	9.66E-01	0.000	0.000	0.000	0.001	0.001
4.2.1.59|g__Intestinimonas.s__Intestinimonas_butyriciproducens	-	7.79E-01	0.000	0.000	0.000	0.001	0.001
4.2.1.59|g__Klebsiella.s__Klebsiella_aerogenes	+	8.37E-02	0.000	0.000	0.000	0.001	0.001
4.2.1.59|g__Klebsiella.s__Klebsiella_grimontii	+	3.30E-01	0.000	0.000	0.000	0.000	0.000
4.2.1.59|g__Klebsiella.s__Klebsiella_michiganensis	+	6.65E-01	0.000	0.000	0.000	0.000	0.000
4.2.1.59|g__Klebsiella.s__Klebsiella_oxytoca	+	7.44E-02	0.000	0.000	0.000	0.001	0.001
4.2.1.59|g__Klebsiella.s__Klebsiella_pneumoniae	+	5.68E-03	0.000	0.000	0.000	0.005	0.006
4.2.1.59|g__Klebsiella.s__Klebsiella_variicola	+	5.92E-03	0.000	0.000	0.000	0.001	0.003
4.2.1.59|g__Kluyvera.s__Kluyvera_ascorbata	-	7.14E-01	0.000	0.000	0.000	0.000	0.000
4.2.1.59|g__Kluyvera.s__Kluyvera_cryocrescens	+	3.30E-01	0.000	0.000	0.000	0.000	0.000
4.2.1.59|g__Kosakonia.s__Kosakonia_cowanii	+	4.92E-01	0.000	0.000	0.000	0.000	0.000

4.2.1.59|g__Lachnoclostridium.s__Clostridium_aldenense	+	5.58E-01	0.000	0.000	0.000	0.001	0.001
4.2.1.59|g__Lachnoclostridium.s__Clostridium_asparagiforme	+	4.26E-01	0.000	0.000	0.000	0.000	0.001
4.2.1.59|g__Lachnoclostridium.s__Clostridium_bolteae	+	7.09E-01	0.000	0.000	0.000	0.003	0.003
4.2.1.59|g__Lachnoclostridium.s__Clostridium_citroniae	+	6.66E-01	0.000	0.000	0.000	0.000	0.000
4.2.1.59|g__Lachnoclostridium.s__Clostridium_clostridioforme	-	8.32E-01	0.000	0.000	0.000	0.002	0.004
4.2.1.59|g__Lachnoclostridium.s__Clostridium_hylemonae	+	1.67E-01	0.000	0.000	0.000	0.000	0.000
4.2.1.59|g__Lachnoclostridium.s__Clostridium_lavalense	+	2.32E-01	0.000	0.000	0.000	0.001	0.001
4.2.1.59|g__Lachnoclostridium.s__Clostridium_saccharolyticum	+	8.48E-01	0.000	0.000	0.000	0.000	0.001
4.2.1.59|g__Lachnoclostridium.s__Clostridium_scindens	+	1.42E-01	0.000	0.000	0.000	0.003	0.003
4.2.1.59|g__Lachnoclostridium.s__Clostridium_symbiosum	+	7.97E-01	0.000	0.000	0.000	0.001	0.001
4.2.1.59|g__Lachnoclostridium.s__Lachnoclostridium_sp_An118	+	5.89E-01	0.000	0.000	0.000	0.000	0.000
4.2.1.59|g__Lachnoclostridium.s__Lachnoclostridium_sp_An14	-	5.47E-01	0.000	0.000	0.000	0.000	0.000
4.2.1.59|g__Lachnoclostridium.s__Lachnoclostridium_sp_An181	+	3.30E-01	0.000	0.000	0.000	0.000	0.000
4.2.1.59|g__Lachnoclostridium.s__Lachnoclostridium_sp_An76	+	3.30E-01	0.000	0.000	0.000	0.000	0.000
4.2.1.59|g__Lachnospira.s__Lachnospira_pectinoschiza	-	8.33E-01	0.000	0.000	0.000	0.001	0.001
4.2.1.59|g__Lachnospiraceae_unclassified.s__Lachnospiraceae_bacterium_2	+	5.59E-01	0.000	0.000	0.000	0.000	0.000
4.2.1.59|g__Lachnospiraceae_unclassified.s__Lachnospiraceae_bacterium_(	-	5.06E-01	0.000	0.000	0.000	0.000	0.000
4.2.1.59|g__Lachnospiraceae_unclassified.s__Lachnospiraceae_bacterium_(	-	2.01E-01	0.000	0.000	0.000	0.000	0.000
4.2.1.59|g__Lactobacillus.s__Lactobacillus_acidipiscis	+	3.30E-01	0.000	0.000	0.000	0.000	0.000
4.2.1.59|g__Lactobacillus.s__Lactobacillus_animalis	+	2.43E-01	0.000	0.000	0.000	0.001	0.001
4.2.1.59|g__Lactobacillus.s__Lactobacillus_casei_group	+	5.23E-01	0.000	0.000	0.000	0.000	0.000
4.2.1.59|g__Lactobacillus.s__Lactobacillus_crispatus	+	1.67E-01	0.000	0.000	0.000	0.000	0.000
4.2.1.59|g__Lactobacillus.s__Lactobacillus_curvatus	+	4.92E-01	0.000	0.000	0.000	0.000	0.000
4.2.1.59|g__Lactobacillus.s__Lactobacillus_delbrueckii	-	1.86E-01	0.000	0.000	0.000	0.000	0.000
4.2.1.59|g__Lactobacillus.s__Lactobacillus_fermentum	+	4.45E-03	0.000	0.000	0.000	0.001	0.002
4.2.1.59|g__Lactobacillus.s__Lactobacillus_kimbladii	+	4.92E-01	0.000	0.000	0.000	0.000	0.000
4.2.1.59|g__Lactobacillus.s__Lactobacillus_kullabergensis	+	4.92E-01	0.000	0.000	0.000	0.000	0.000
4.2.1.59|g__Lactobacillus.s__Lactobacillus_mucosae	-	7.12E-01	0.000	0.000	0.000	0.002	0.001
4.2.1.59|g__Lactobacillus.s__Lactobacillus_oris	+	2.22E-03	0.000	0.000	0.000	0.001	0.002
4.2.1.59|g__Lactobacillus.s__Lactobacillus_pantheris	+	4.92E-01	0.000	0.000	0.000	0.000	0.000
4.2.1.59|g__Lactobacillus.s__Lactobacillus_paracasei	+	1.90E-01	0.000	0.000	0.000	0.000	0.000
4.2.1.59|g__Lactobacillus.s__Lactobacillus_paraplantarum	+	8.38E-01	0.000	0.000	0.000	0.000	0.000
4.2.1.59|g__Lactobacillus.s__Lactobacillus_pentosus	+	2.32E-01	0.000	0.000	0.000	0.000	0.000
4.2.1.59|g__Lactobacillus.s__Lactobacillus_plantarum	+	8.36E-01	0.000	0.000	0.000	0.000	0.000
4.2.1.59|g__Lactobacillus.s__Lactobacillus_rapi	+	4.92E-01	0.000	0.000	0.000	0.000	0.000
4.2.1.59|g__Lactobacillus.s__Lactobacillus_reuteri	+	3.79E-02	0.000	0.000	0.000	0.000	0.002
4.2.1.59|g__Lactobacillus.s__Lactobacillus_rhamnosus	+	1.85E-02	0.000	0.000	0.000	0.000	0.000
4.2.1.59|g__Lactobacillus.s__Lactobacillus_rogosae	-	7.49E-01	0.000	0.000	0.000	0.001	0.001
4.2.1.59|g__Lactobacillus.s__Lactobacillus_ruminis	+	9.00E-02	0.000	0.000	0.000	0.000	0.000
4.2.1.59|g__Lactobacillus.s__Lactobacillus_sakei	-	1.48E-01	0.000	0.000	0.000	0.000	0.000
4.2.1.59|g__Lactobacillus.s__Lactobacillus_salivarius	+	2.35E-04	0.000	0.000	0.000	0.000	0.003
4.2.1.59|g__Lactobacillus.s__Lactobacillus_sanfranciscensis	+	4.92E-01	0.000	0.000	0.000	0.000	0.000
4.2.1.59|g__Lactobacillus.s__Lactobacillus_vaccinostercus	+	1.67E-01	0.000	0.000	0.000	0.000	0.000
4.2.1.59|g__Lactobacillus.s__Lactobacillus_vaginalis	-	4.47E-01	0.000	0.000	0.000	0.000	0.000
4.2.1.59|g__Lactococcus.s__Lactococcus_lactis	-	4.15E-01	0.000	0.000	0.000	0.001	0.000
4.2.1.59|g__Leclercia.s__Leclercia_adecarboxylata	+	1.22E-01	0.000	0.000	0.000	0.000	0.000
4.2.1.59|g__Lelliottia.s__Lelliottia_nimipressuralis	+	7.52E-02	0.000	0.000	0.000	0.000	0.000
4.2.1.59|g__Leuconostoc.s__Leuconostoc_citreum	+	2.32E-01	0.000	0.000	0.000	0.000	0.000
4.2.1.59|g__Leuconostoc.s__Leuconostoc_garlicum	+	4.92E-01	0.000	0.000	0.000	0.000	0.000
4.2.1.59|g__Leuconostoc.s__Leuconostoc_gelidum	+	7.57E-01	0.000	0.000	0.000	0.000	0.000
4.2.1.59|g__Leuconostoc.s__Leuconostoc_lactis	+	3.30E-01	0.000	0.000	0.000	0.000	0.000
4.2.1.59|g__Leuconostoc.s__Leuconostoc_mesenteroides	-	9.70E-01	0.000	0.000	0.000	0.000	0.000
4.2.1.59|g__Listeria.s__Listeria_monocytogenes	+	7.20E-02	0.000	0.000	0.000	0.000	0.000
4.2.1.59|g__Marvinbryantia.s__Marvinbryantia_formatexigens	+	2.32E-01	0.000	0.000	0.000	0.000	0.000
4.2.1.59|g__Megamonas.s__Megamonas_funiformis	+	7.84E-01	0.000	0.000	0.000	0.000	0.000
4.2.1.59|g__Megamonas.s__Megamonas_hypermegale	-	7.73E-01	0.000	0.000	0.000	0.000	0.000
4.2.1.59|g__Megamonas.s__Megamonas_rupellensis	-	4.61E-01	0.000	0.000	0.000	0.000	0.000
4.2.1.59|g__Megasphaera.s__Megasphaera_elsdenii	+	5.55E-01	0.000	0.000	0.000	0.000	0.000
4.2.1.59|g__Megasphaera.s__Megasphaera_hexanoica	+	4.92E-01	0.000	0.000	0.000	0.000	0.000
4.2.1.59|g__Megasphaera.s__Megasphaera_micronuciformis	+	3.30E-01	0.000	0.000	0.000	0.000	0.000
4.2.1.59|g__Megasphaera.s__Megasphaera_sp_DISK_18	+	8.11E-04	0.000	0.000	0.000	0.000	0.002
4.2.1.59|g__Megasphaera.s__Megasphaera_sp_MJR8396C	+	9.25E-02	0.000	0.000	0.000	0.001	0.002
4.2.1.59|g__Megasphaera.s__Megasphaera_stantonii	-	5.92E-01	0.000	0.000	0.000	0.000	0.000
4.2.1.59|g__Mesosutterella.s__Mesosutterella_multiformis	+	2.00E-02	0.000	0.000	0.000	0.000	0.000
4.2.1.59|g__Mitsuokella.s__Mitsuokella_jalaludinii	-	1.48E-01	0.000	0.000	0.000	0.000	0.000
4.2.1.59|g__Mitsuokella.s__Mitsuokella_multacida	+	4.92E-01	0.000	0.000	0.000	0.000	0.000
4.2.1.59|g__Monoglobus.s__Monoglobus_pectinilyticus	-	7.22E-01	0.000	0.000	0.000	0.001	0.001
4.2.1.59|g__Morganella.s__Morganella_morganii	+	3.30E-01	0.000	0.000	0.000	0.000	0.000
4.2.1.59|g__Muribaculum.s__Muribaculum_intestinale	-	5.92E-01	0.000	0.000	0.000	0.000	0.000
4.2.1.59|g__Murimonas.s__Murimonas_intestini	+	1.67E-01	0.000	0.000	0.000	0.000	0.000
4.2.1.59|g__Negativicoccus.s__Negativicoccus_succinicivorans	+	4.92E-01	0.000	0.000	0.000	0.000	0.000
4.2.1.59|g__Odoribacter.s__Odoribacter_laneus	+	3.34E-01	0.000	0.000	0.000	0.001	0.003

4.2.1.59|g__Oligella.s__Oligella_urethralis	+	4.92E-01	0.000	0.000	0.000	0.000	0.000
4.2.1.59|g__Oxalobacter.s__Oxalobacter_formigenes	+	2.41E-01	0.000	0.000	0.000	0.000	0.000
4.2.1.59|g__Pantoea.s__Pantoea_sesami	+	2.33E-01	0.000	0.000	0.000	0.000	0.000
4.2.1.59|g__Parabacteroides.s__Parabacteroides_chinchillae	+	4.92E-01	0.000	0.000	0.000	0.000	0.000
4.2.1.59|g__Parabacteroides.s__Parabacteroides_goldsteinii	+	1.02E-02	0.000	0.000	0.000	0.001	0.002
4.2.1.59|g__Parabacteroides.s__Parabacteroides_gordonii	-	7.89E-01	0.000	0.000	0.000	0.000	0.000
4.2.1.59|g__Parabacteroides.s__Parabacteroides_johnsonii	-	3.21E-01	0.000	0.000	0.000	0.000	0.000
4.2.1.59|g__Paraprevotella.s__Paraprevotella_clara	-	1.38E-01	0.000	0.000	0.000	0.002	0.002
4.2.1.59|g__Paraprevotella.s__Paraprevotella_xylaniphila	+	4.16E-01	0.000	0.000	0.000	0.000	0.000
4.2.1.59|g__Parasutterella.s__Parasutterella_excrementihominis	-	8.62E-01	0.000	0.000	0.000	0.003	0.003
4.2.1.59|g__Parvimonas.s__Parvimonas_micra	+	4.92E-01	0.000	0.000	0.000	0.000	0.000
4.2.1.59|g__Pediococcus.s__Pediococcus_acidilactici	+	3.05E-03	0.000	0.000	0.000	0.000	0.001
4.2.1.59|g__Pediococcus.s__Pediococcus_argentinicus	+	4.92E-01	0.000	0.000	0.000	0.000	0.000
4.2.1.59|g__Pedobacter.s__Pedobacter_himalayensis	+	3.30E-01	0.000	0.000	0.000	0.000	0.000
4.2.1.59|g__Peptoniphilus.s__Peptoniphilus_lacrimalis	+	4.92E-01	0.000	0.000	0.000	0.000	0.000
4.2.1.59|g__Peptostreptococcaceae_unclassified.s__Clostridium_hiranonis	-	5.48E-01	0.000	0.000	0.000	0.000	0.000
4.2.1.59|g__Peptostreptococcus.s__Peptostreptococcus_anaerobius	-	4.50E-01	0.000	0.000	0.000	0.000	0.000
4.2.1.59|g__Peptostreptococcus.s__Peptostreptococcus_stomatis	+	4.92E-01	0.000	0.000	0.000	0.000	0.000
4.2.1.59|g__Phascolarctobacterium.s__Phascolarctobacterium_succinatutens	+	4.35E-01	0.000	0.000	0.000	0.000	0.001
4.2.1.59|g__Pluralibacter.s__Pluralibacter_gergoviae	-	1.48E-01	0.000	0.000	0.000	0.000	0.000
4.2.1.59|g__Porphyromonas.s__Porphyromonas_asaccharolytica	+	9.82E-02	0.000	0.000	0.000	0.000	0.000
4.2.1.59|g__Porphyromonas.s__Porphyromonas_uenonis	-	9.70E-01	0.000	0.000	0.000	0.000	0.000
4.2.1.59|g__Prevotella.s__Prevotella_bivia	+	8.25E-01	0.000	0.000	0.000	0.000	0.000
4.2.1.59|g__Prevotella.s__Prevotella_buccae	-	9.73E-01	0.000	0.000	0.000	0.000	0.000
4.2.1.59|g__Prevotella.s__Prevotella_buccalis	+	9.00E-02	0.000	0.000	0.000	0.000	0.000
4.2.1.59|g__Prevotella.s__Prevotella_corporis	+	6.68E-02	0.000	0.000	0.000	0.000	0.000
4.2.1.59|g__Prevotella.s__Prevotella_disiens	-	9.57E-01	0.000	0.000	0.000	0.000	0.000
4.2.1.59|g__Prevotella.s__Prevotella_histicola	+	4.92E-01	0.000	0.000	0.000	0.000	0.000
4.2.1.59|g__Prevotella.s__Prevotella_sp_109	-	9.14E-01	0.000	0.000	0.000	0.002	0.001
4.2.1.59|g__Prevotella.s__Prevotella_timonensis	+	1.60E-02	0.000	0.000	0.000	0.000	0.000
4.2.1.59|g__Proteus.s__Proteus_mirabilis	+	5.60E-01	0.000	0.000	0.000	0.000	0.000
4.2.1.59|g__Pseudocitrobacter.s__Pseudocitrobacter_faecalis	+	4.92E-01	0.000	0.000	0.000	0.000	0.000
4.2.1.59|g__Pseudoflavonifractor.s__Pseudoflavonifractor_capillosus	+	4.35E-01	0.000	0.000	0.000	0.000	0.000
4.2.1.59|g__Pseudoflavonifractor.s__Pseudoflavonifractor_sp_An184	+	6.05E-03	0.000	0.000	0.000	0.001	0.001
4.2.1.59|g__Pseudomonas.s__Pseudomonas_aeruginosa	+	4.16E-01	0.000	0.000	0.000	0.000	0.000
4.2.1.59|g__Pseudomonas.s__Pseudomonas_aeruginosa_group	+	3.10E-01	0.000	0.000	0.000	0.000	0.000
4.2.1.59|g__Pseudomonas.s__Pseudomonas_alcaligenes	-	1.48E-01	0.000	0.000	0.000	0.000	0.000
4.2.1.59|g__Pseudomonas.s__Pseudomonas_delhiensis	+	4.92E-01	0.000	0.000	0.000	0.000	0.000
4.2.1.59|g__Pseudomonas.s__Pseudomonas_fluorescens_group	-	1.48E-01	0.000	0.000	0.000	0.000	0.000
4.2.1.59|g__Pyramidobacter.s__Pyramidobacter_piscolens	+	1.29E-01	0.000	0.000	0.000	0.000	0.001
4.2.1.59|g__Pyramidobacter.s__Pyramidobacter_sp_C12_8	+	4.92E-01	0.000	0.000	0.000	0.000	0.000
4.2.1.59|g__Raoultella.s__Raoultella_ornithinolytica	-	9.67E-01	0.000	0.000	0.000	0.000	0.000
4.2.1.59|g__Raoultella.s__Raoultella_planticola	-	2.03E-01	0.000	0.000	0.000	0.000	0.000
4.2.1.59|g__Rikenella.s__Rikenella_microfusus	+	4.92E-01	0.000	0.000	0.000	0.000	0.000
4.2.1.59|g__Romboutsia.s__Romboutsia_hominis	+	4.92E-01	0.000	0.000	0.000	0.000	0.000
4.2.1.59|g__Roseburia.s__Roseburia_faecis	-	1.64E-03	0.000	0.000	0.000	0.013	0.009
4.2.1.59|g__Roseburia.s__Roseburia_hominis	-	3.23E-01	0.000	0.000	0.000	0.004	0.003
4.2.1.59|g__Roseburia.s__Roseburia_inulinivorans	-	5.66E-03	0.000	0.000	0.000	0.003	0.002
4.2.1.59|g__Rubneribacter.s__Rubneribacter_badeniensis	-	5.95E-01	0.000	0.000	0.000	0.000	0.000
4.2.1.59|g__Ruminococcaceae_unclassified.s__Clostridium_methylpentosum	+	2.32E-01	0.000	0.000	0.000	0.000	0.000
4.2.1.59|g__Ruminococcaceae_unclassified.s__Ruminococcaceae_bacterium	+	2.22E-01	0.000	0.000	0.000	0.001	0.002
4.2.1.59|g__Ruminococcus.s__Ruminococcus_lactaris	-	1.17E-03	0.000	0.000	0.000	0.005	0.002
4.2.1.59|g__Ruminococcus.s__Ruminococcus_sp_AF31_8BH	-	1.63E-01	0.000	0.000	0.000	0.000	0.001
4.2.1.59|g__Salmonella.s__Salmonella_enterica	+	4.55E-03	0.000	0.000	0.000	0.001	0.001
4.2.1.59|g__Sanguibacteroides.s__Sanguibacteroides_justesenii	+	3.30E-01	0.000	0.000	0.000	0.000	0.000
4.2.1.59|g__Sellimonas.s__Sellimonas_intestinalis	+	5.06E-01	0.000	0.000	0.000	0.002	0.003
4.2.1.59|g__Staphylococcus.s__Staphylococcus_aureus	+	2.32E-01	0.000	0.000	0.000	0.000	0.000
4.2.1.59|g__Staphylococcus.s__Staphylococcus_haemolyticus	+	4.92E-01	0.000	0.000	0.000	0.000	0.000
4.2.1.59|g__Streptococcus.s__Streptococcus_agalactiae	+	7.60E-01	0.000	0.000	0.000	0.000	0.000
4.2.1.59|g__Streptococcus.s__Streptococcus_anginosus_group	+	3.30E-01	0.000	0.000	0.000	0.000	0.000
4.2.1.59|g__Streptococcus.s__Streptococcus_australis	-	2.65E-03	0.000	0.000	0.000	0.000	0.000
4.2.1.59|g__Streptococcus.s__Streptococcus_constellatus	+	1.67E-01	0.000	0.000	0.000	0.000	0.000
4.2.1.59|g__Streptococcus.s__Streptococcus_cristatus	-	2.55E-01	0.000	0.000	0.000	0.000	0.000
4.2.1.59|g__Streptococcus.s__Streptococcus_downei	+	4.92E-01	0.000	0.000	0.000	0.000	0.000
4.2.1.59|g__Streptococcus.s__Streptococcus_dysgalactiae_group	+	4.92E-01	0.000	0.000	0.000	0.000	0.000
4.2.1.59|g__Streptococcus.s__Streptococcus_equinus	+	7.90E-02	0.000	0.000	0.000	0.000	0.001
4.2.1.59|g__Streptococcus.s__Streptococcus_gallolyticus	+	1.81E-01	0.000	0.000	0.000	0.000	0.001
4.2.1.59|g__Streptococcus.s__Streptococcus_gordonii	+	6.51E-01	0.000	0.000	0.000	0.000	0.000
4.2.1.59|g__Streptococcus.s__Streptococcus_infantarius	+	3.12E-02	0.000	0.000	0.000	0.000	0.000
4.2.1.59|g__Streptococcus.s__Streptococcus_infantis	+	4.92E-01	0.000	0.000	0.000	0.000	0.000
4.2.1.59|g__Streptococcus.s__Streptococcus_lutetiensis	+	2.51E-02	0.000	0.000	0.000	0.000	0.001
4.2.1.59|g__Streptococcus.s__Streptococcus_macedonicus	+	3.24E-02	0.000	0.000	0.000	0.000	0.000

	4.2.1.59|g__Streptococcus.s__Streptococcus_milleri	+	6.61E-01	0.000	0.000	0.000	0.000	0.000
	4.2.1.59|g__Streptococcus.s__Streptococcus_mitis	-	9.56E-02	0.000	0.000	0.000	0.000	0.000
	4.2.1.59|g__Streptococcus.s__Streptococcus_mutans	+	9.44E-06	0.000	0.000	0.000	0.000	0.001
	4.2.1.59|g__Streptococcus.s__Streptococcus_oralis	-	3.57E-01	0.000	0.000	0.000	0.000	0.000
	4.2.1.59|g__Streptococcus.s__Streptococcus_parasanguinis	-	7.75E-01	0.000	0.000	0.000	0.004	0.003
	4.2.1.59|g__Streptococcus.s__Streptococcus_pasteurianus	+	1.41E-01	0.000	0.000	0.000	0.000	0.002
	4.2.1.59|g__Streptococcus.s__Streptococcus_pneumoniae	-	6.03E-01	0.000	0.000	0.000	0.001	0.000
	4.2.1.59|g__Streptococcus.s__Streptococcus_pseudopneumoniae	-	2.46E-02	0.000	0.000	0.000	0.000	0.000
	4.2.1.59|g__Streptococcus.s__Streptococcus_sanguinis	-	9.90E-01	0.000	0.000	0.000	0.000	0.000
	4.2.1.59|g__Streptococcus.s__Streptococcus_sobrinus	+	1.22E-01	0.000	0.000	0.000	0.000	0.000
	4.2.1.59|g__Streptococcus.s__Streptococcus_sp_A12	-	2.82E-03	0.000	0.000	0.000	0.000	0.000
	4.2.1.59|g__Streptococcus.s__Streptococcus_thermophilus	-	7.32E-01	0.000	0.000	0.000	0.005	0.006
	4.2.1.59|g__Streptococcus.s__Streptococcus_vestibularis	+	1.97E-04	0.000	0.000	0.000	0.001	0.003
	4.2.1.59|g__Streptococcus.s__Streptococcus_viridans	+	8.93E-03	0.000	0.000	0.000	0.000	0.000
	4.2.1.59|g__Succinatimonas.s__Succinatimonas_hippei	+	7.53E-01	0.000	0.000	0.000	0.000	0.000
	4.2.1.59|g__Sutterella.s__Sutterella_wadsworthensis	+	5.88E-01	0.000	0.000	0.000	0.003	0.003
	4.2.1.59|g__Terrisporobacter.s__Terrisporobacter_othiniensis	+	1.67E-01	0.000	0.000	0.000	0.000	0.000
	4.2.1.59|g__Trichococcus.s__Trichococcus_flocculiformis	+	4.92E-01	0.000	0.000	0.000	0.000	0.000
	4.2.1.59|g__Turicibacter.s__Turicibacter_sanguinis	+	1.37E-02	0.000	0.000	0.000	0.000	0.000
	4.2.1.59|g__Turicimonas.s__Turicimonas_muris	+	3.05E-01	0.000	0.000	0.000	0.000	0.000
	4.2.1.59|g__Tyzzerella.s__Tyzzerella_nexilis	+	3.97E-01	0.000	0.000	0.000	0.002	0.002
	4.2.1.59|g__Veillonella.s__Veillonella_atypica	-	2.97E-01	0.000	0.000	0.000	0.000	0.000
	4.2.1.59|g__Veillonella.s__Veillonella_dispar	-	3.60E-01	0.000	0.000	0.000	0.000	0.000
	4.2.1.59|g__Veillonella.s__Veillonella_infantium	-	7.88E-01	0.000	0.000	0.000	0.000	0.000
	4.2.1.59|g__Veillonella.s__Veillonella_parvula	+	5.71E-01	0.000	0.000	0.000	0.000	0.001
	4.2.1.59|g__Veillonella.s__Veillonella_rogosae	-	5.93E-01	0.000	0.000	0.000	0.000	0.000
	4.2.1.59|g__Veillonella.s__Veillonella_seminalis	-	4.48E-01	0.000	0.000	0.000	0.000	0.000
	4.2.1.59|g__Veillonella.s__Veillonella_tobetsuensis	-	1.48E-01	0.000	0.000	0.000	0.000	0.000
	4.2.1.59|g__Vibrio.s__Vibrio_parahaemolyticus	+	6.65E-01	0.000	0.000	0.000	0.000	0.000
	4.2.1.59|g__Weeksella.s__Weeksella_virosa	+	4.92E-01	0.000	0.000	0.000	0.000	0.000
	4.2.1.59|g__Weissella.s__Weissella_cibaria	-	9.67E-01	0.000	0.000	0.000	0.000	0.000
	4.2.1.59|g__Weissella.s__Weissella_confusa	+	8.39E-01	0.000	0.000	0.000	0.000	0.000
	4.2.1.59|g__Weissella.s__Weissella_viridescens	+	4.92E-01	0.000	0.000	0.000	0.000	0.000
	4.2.1.59|g__Yokenella.s__Yokenella_regensburgei	+	3.30E-01	0.000	0.000	0.000	0.000	0.000
	4.2.1.59|g__Flavonifractor.s__Flavonifractor_plautii	+	9.11E-01	0.482	0.504	0.022	0.007	0.007
	4.2.1.59|g__Bacteroides.s__Bacteroides_cellulosilyticus	+	1.37E-01	0.000	0.039	0.039	0.011	0.011
	4.2.1.59|g__Bacteroides.s__Bacteroides_xylanisolvens	+	1.94E-01	0.000	0.050	0.050	0.008	0.007
	4.2.1.59|g__Alistipes.s__Alistipes_shahii	+	4.26E-01	0.457	0.510	0.053	0.008	0.010
	4.2.1.59|g__Streptococcus.s__Streptococcus_salivarius	+	5.06E-01	0.219	0.273	0.053	0.010	0.011
	4.2.1.59|g__Hungatella.s__Hungatella_hathewayi	+	9.24E-02	0.000	0.061	0.061	0.004	0.004
	4.2.1.59|g__Bacteroides.s__Bacteroides_thetaiotaomicron	+	4.05E-01	0.357	0.433	0.076	0.010	0.011
	4.2.1.59|g__Parabacteroides.s__Parabacteroides_merdae	+	1.62E-01	0.515	0.628	0.113	0.006	0.007
	4.2.1.59|g__Alistipes.s__Alistipes_onderdonkii	+	2.70E-02	0.043	0.174	0.131	0.005	0.007
	4.2.1.59|g__Bacteroides.s__Bacteroides_caccae	+	1.30E-01	0.171	0.308	0.137	0.006	0.009
	4.2.1.59|g__Bacteroides.s__Bacteroides_intestinalis	+	1.29E-01	0.000	0.147	0.147	0.008	0.010
	4.2.1.59|g__Odoribacter.s__Odoribacter_splanchnicus	+	1.63E-01	0.000	0.153	0.153	0.002	0.002
	4.2.1.59|g__Phascolarctobacterium.s__Phascolarctobacterium_faecium	+	4.32E-01	0.000	0.171	0.171	0.004	0.005
	4.2.1.59|g__Bilophila.s__Bilophila_wadsworthia	+	2.26E-02	0.228	0.447	0.218	0.004	0.006
	4.2.1.59|g__Bifidobacterium.s__Bifidobacterium_longum	+	1.90E-03	0.000	0.234	0.234	0.006	0.017
	4.2.1.59|g__Alistipes.s__Alistipes_finegoldii	+	2.82E-02	0.579	0.857	0.278	0.016	0.021
	4.2.1.59|g__Bacteroides.s__Bacteroides_stercoris	+	1.91E-01	0.113	0.460	0.347	0.032	0.036
	4.2.1.59|g__Parabacteroides.s__Parabacteroides_distasonis	+	6.49E-03	0.692	1.090	0.398	0.013	0.015
Germany	4.2.1.59	-	8.14E-01	131.835	139.849	8.014	1.000	1.000
	4.2.1.59|g__Faecalibacterium.s__Faecalibacterium_prausnitzii	-	4.30E-01	9.628	5.210	-4.418	0.082	0.078
	4.2.1.59|g__Roseburia.s__Roseburia_faecis	-	4.28E-01	0.973	0.400	-0.573	0.020	0.012
	4.2.1.59|g__Gemmiger.s__Gemmiger_formicilis	-	8.14E-01	1.588	1.128	-0.460	0.017	0.015
	4.2.1.59|g__Blautia.s__Ruminococcus_torques	-	5.79E-01	3.235	2.783	-0.452	0.027	0.026
	4.2.1.59|g__Parabacteroides.s__Parabacteroides_distasonis	-	8.20E-01	1.511	1.105	-0.407	0.012	0.012
	4.2.1.59|g__Roseburia.s__Roseburia_inulinivorans	-	7.10E-01	0.843	0.476	-0.367	0.012	0.007
	4.2.1.59|g__Lachnospiraceae_unclassified.s__Eubacterium_rectale	+	7.26E-01	1.110	0.791	-0.319	0.015	0.019
	4.2.1.59|g__Bacteroides.s__Bacteroides_caccae	-	5.44E-01	0.729	0.412	-0.317	0.012	0.008
	4.2.1.59|g__Roseburia.s__Roseburia_intestinalis	-	1.90E-01	0.312	0.000	-0.312	0.014	0.004
	4.2.1.59|g__Escherichia.s__Escherichia_coli	-	2.68E-01	0.230	0.000	-0.230	0.041	0.018
	4.2.1.59|g__Bacteroides.s__Bacteroides_xylanisolvens	-	4.57E-01	0.545	0.333	-0.213	0.013	0.010
	4.2.1.59|g__Blautia.s__Blautia_obeum	-	6.65E-01	1.261	1.068	-0.193	0.011	0.017
	4.2.1.59|g__Bacteroides.s__Bacteroides_dorei	-	8.14E-01	1.678	1.495	-0.184	0.021	0.018
	4.2.1.59|g__Dorea.s__Dorea_longicatena	-	9.09E-01	0.646	0.499	-0.147	0.008	0.009
	4.2.1.59|g__Coprococcus.s__Coprococcus_catus	-	1.37E-01	0.139	0.000	-0.139	0.002	0.001
	4.2.1.59|g__Dorea.s__Dorea_formicigenerans	-	5.31E-01	0.425	0.303	-0.122	0.003	0.002
	4.2.1.59|g__Bacteroides.s__Bacteroides_thetaiotaomicron	-	4.99E-01	0.420	0.299	-0.120	0.009	0.005
	4.2.1.59|g__Coprococcus.s__Coprococcus_comes	-	5.04E-01	0.515	0.399	-0.116	0.006	0.006
	4.2.1.59|g__Streptococcus.s__Streptococcus_thermophilus	-	2.18E-01	0.081	0.000	-0.081	0.002	0.002

4.2.1.59|g__Alistipes.s__Alistipes_finegoldii	-	6.27E-01	0.601	0.533	-0.068	0.011	0.022
4.2.1.59|g__Blautia.s__Blautia_wexlerae	-	4.38E-01	0.212	0.181	-0.031	0.004	0.002
4.2.1.59|g__Streptococcus.s__Streptococcus_salivarius	-	6.49E-01	0.030	0.000	-0.030	0.001	0.004
4.2.1.59|g__Clostridiales_unclassified.s__Clostridiales_bacterium_KLE1615	+	9.75E-01	0.210	0.191	-0.019	0.002	0.003
4.2.1.59|g__Bacteroides.s__Bacteroides_stercoris	-	5.94E-01	0.016	0.000	-0.016	0.015	0.009
4.2.1.59|g__Bacteroides.s__Bacteroides_ovatus	+	9.57E-01	0.094	0.089	-0.005	0.003	0.013
4.2.1.59|g__Acidaminococcus.s__Acidaminococcus_fermentans	-	9.42E-01	0.000	0.000	0.000	0.000	0.000
4.2.1.59|g__Acidaminococcus.s__Acidaminococcus_intestini	-	5.37E-01	0.000	0.000	0.000	0.000	0.001
4.2.1.59|g__Adlercreutzia.s__Adlercreutzia_equolifaciens	+	7.41E-01	0.000	0.000	0.000	0.000	0.000
4.2.1.59|g__Alistipes.s__Alistipes_indistinctus	-	8.24E-01	0.000	0.000	0.000	0.001	0.001
4.2.1.59|g__Alistipes.s__Alistipes_inops	+	1.56E-01	0.000	0.000	0.000	0.003	0.003
4.2.1.59|g__Alistipes.s__Alistipes_sp_An66	+	1.83E-01	0.000	0.000	0.000	0.000	0.000
4.2.1.59|g__Alistipes.s__Alistipes_timonensis	+	6.32E-01	0.000	0.000	0.000	0.000	0.001
4.2.1.59|g__Allisonella.s__Allisonella_histaminiformans	-	1.40E-01	0.000	0.000	0.000	0.000	0.000
4.2.1.59|g__Anaerotruncus.s__Anaerotruncus_colihominis	+	6.61E-01	0.000	0.000	0.000	0.000	0.000
4.2.1.59|g__Asaccharobacter.s__Asaccharobacter_celatus	+	4.33E-01	0.000	0.000	0.000	0.000	0.002
4.2.1.59|g__Bacteroides.s__Bacteroides_clarus	-	7.56E-01	0.000	0.000	0.000	0.003	0.001
4.2.1.59|g__Bacteroides.s__Bacteroides_coprocola	-	5.25E-01	0.000	0.000	0.000	0.013	0.004
4.2.1.59|g__Bacteroides.s__Bacteroides_coprophilus	+	8.14E-01	0.000	0.000	0.000	0.004	0.000
4.2.1.59|g__Bacteroides.s__Bacteroides_eggerthii	-	7.14E-01	0.000	0.000	0.000	0.012	0.003
4.2.1.59|g__Bacteroides.s__Bacteroides_faecichinchillae	+	1.83E-01	0.000	0.000	0.000	0.000	0.000
4.2.1.59|g__Bacteroides.s__Bacteroides_faecis	+	7.54E-01	0.000	0.000	0.000	0.003	0.003
4.2.1.59|g__Bacteroides.s__Bacteroides_finegoldii	+	2.89E-01	0.000	0.000	0.000	0.002	0.003
4.2.1.59|g__Bacteroides.s__Bacteroides_fluxus	-	1.40E-01	0.000	0.000	0.000	0.000	0.000
4.2.1.59|g__Bacteroides.s__Bacteroides_fragilis	+	7.63E-01	0.000	0.000	0.000	0.002	0.002
4.2.1.59|g__Bacteroides.s__Bacteroides_massiliensis	+	9.93E-01	0.000	0.000	0.000	0.014	0.012
4.2.1.59|g__Bacteroides.s__Bacteroides_nordii	+	8.94E-01	0.000	0.000	0.000	0.000	0.000
4.2.1.59|g__Bacteroides.s__Bacteroides_plebeius	-	9.19E-01	0.000	0.000	0.000	0.002	0.003
4.2.1.59|g__Bacteroides.s__Bacteroides_salyersiae	+	9.74E-01	0.000	0.000	0.000	0.001	0.003
4.2.1.59|g__Bacteroides.s__Bacteroides_sp_OM08_11	+	3.59E-01	0.000	0.000	0.000	0.000	0.000
4.2.1.59|g__Bacteroides.s__Bacteroides_stercorirosoris	+	3.59E-01	0.000	0.000	0.000	0.000	0.000
4.2.1.59|g__Bifidobacterium.s__Bifidobacterium_animalis	-	9.81E-01	0.000	0.000	0.000	0.000	0.000
4.2.1.59|g__Blautia.s__Blautia_hydrogenotrophica	-	3.09E-01	0.000	0.000	0.000	0.000	0.000
4.2.1.59|g__Blautia.s__Blautia_sp_AF19_10LB	+	2.41E-01	0.000	0.000	0.000	0.001	0.001
4.2.1.59|g__Blautia.s__Ruminococcus_gnavus	-	5.77E-01	0.000	0.000	0.000	0.005	0.003
4.2.1.59|g__Butyrivibrio.s__Butyrivibrio_crossotus	+	8.14E-01	0.000	0.000	0.000	0.011	0.003
4.2.1.59|g__Candidatus_Gastranaerophilales_unclassified.s__Candidatus_Ga	+	4.61E-01	0.000	0.000	0.000	0.000	0.001
4.2.1.59|g__Catenibacterium.s__Catenibacterium_mitsuokai	-	3.12E-02	0.000	0.000	0.000	0.015	0.005
4.2.1.59|g__Citrobacter.s__Citrobacter_sp_MGH106	+	3.59E-01	0.000	0.000	0.000	0.000	0.000
4.2.1.59|g__Cloacibacillus.s__Cloacibacillus_porcorum	-	9.81E-01	0.000	0.000	0.000	0.000	0.000
4.2.1.59|g__Clostridiales_unclassified.s__Bacteroides_pectinophilus	+	5.32E-02	0.000	0.000	0.000	0.000	0.001
4.2.1.59|g__Clostridiales_unclassified.s__Clostridiales_bacterium_CHKCI0	-	3.09E-01	0.000	0.000	0.000	0.000	0.000
4.2.1.59|g__Clostridioides.s__Clostridioides_difficile	+	3.59E-01	0.000	0.000	0.000	0.000	0.000
4.2.1.59|g__Clostridium.s__Clostridium_celatum	-	3.09E-01	0.000	0.000	0.000	0.000	0.000
4.2.1.59|g__Clostridium.s__Clostridium_disporicum	-	1.40E-01	0.000	0.000	0.000	0.000	0.000
4.2.1.59|g__Clostridium.s__Clostridium_perfringens	-	3.09E-01	0.000	0.000	0.000	0.000	0.000
4.2.1.59|g__Coprobacillus.s__Coprobacillus_cateniformis	+	3.74E-01	0.000	0.000	0.000	0.000	0.000
4.2.1.59|g__Coprobacter.s__Coprobacter_fastidiosus	+	8.43E-01	0.000	0.000	0.000	0.002	0.001
4.2.1.59|g__Coprobacter.s__Coprobacter_secundus	+	3.56E-01	0.000	0.000	0.000	0.000	0.000
4.2.1.59|g__Desulfovibrio.s__Desulfovibrio_fairfieldensis	+	6.94E-01	0.000	0.000	0.000	0.000	0.001
4.2.1.59|g__Desulfovibrio.s__Desulfovibrio_piger	-	3.60E-01	0.000	0.000	0.000	0.001	0.002
4.2.1.59|g__Desulfovibrionaceae_unclassified.s__Desulfovibrionaceae_bact	-	3.09E-01	0.000	0.000	0.000	0.001	0.000
4.2.1.59|g__Dialister.s__Dialister_invisus	-	5.82E-01	0.000	0.000	0.000	0.010	0.009
4.2.1.59|g__Dialister.s__Dialister_succinatiphilus	-	9.42E-01	0.000	0.000	0.000	0.002	0.001
4.2.1.59|g__Dorea.s__Dorea_sp_OM02_2LB	-	9.81E-01	0.000	0.000	0.000	0.000	0.000
4.2.1.59|g__Eisenbergiella.s__Eisenbergiella_massiliensis	-	3.09E-01	0.000	0.000	0.000	0.000	0.000
4.2.1.59|g__Eisenbergiella.s__Eisenbergiella_tayi	-	3.04E-01	0.000	0.000	0.000	0.001	0.000
4.2.1.59|g__Enterococcus.s__Enterococcus_durans	+	3.59E-01	0.000	0.000	0.000	0.000	0.000
4.2.1.59|g__Enterococcus.s__Enterococcus_faecalis	-	9.42E-01	0.000	0.000	0.000	0.000	0.000
4.2.1.59|g__Enterococcus.s__Enterococcus_faecium	-	8.75E-01	0.000	0.000	0.000	0.000	0.000
4.2.1.59|g__Enterococcus.s__Enterococcus_hirae	-	3.09E-01	0.000	0.000	0.000	0.000	0.000
4.2.1.59|g__Erysipelatoclostridium.s__Clostridium_spiroforme	+	8.29E-01	0.000	0.000	0.000	0.000	0.000
4.2.1.59|g__Erysipelatoclostridium.s__Erysipelatoclostridium_ramosum	-	9.19E-01	0.000	0.000	0.000	0.000	0.001
4.2.1.59|g__Escherichia.s__Escherichia_fergusonii	-	4.25E-01	0.000	0.000	0.000	0.002	0.001
4.2.1.59|g__Escherichia.s__Escherichia_marmotae	-	3.09E-01	0.000	0.000	0.000	0.000	0.000
4.2.1.59|g__Eubacterium.s__Eubacterium_ramulus	-	5.91E-01	0.000	0.000	0.000	0.002	0.001
4.2.1.59|g__Eubacterium.s__Eubacterium_sp_AF17_7	+	4.32E-02	0.000	0.000	0.000	0.000	0.001
4.2.1.59|g__Eubacterium.s__Eubacterium_ventriosum	-	8.54E-01	0.000	0.000	0.000	0.002	0.002
4.2.1.59|g__Faecalicatena.s__Faecalicatena_contorta	+	3.59E-01	0.000	0.000	0.000	0.000	0.000
4.2.1.59|g__Faecalicoccus.s__Faecalicoccus_pleomorphus	-	3.09E-01	0.000	0.000	0.000	0.000	0.000
4.2.1.59|g__Faecalitalea.s__Faecalitalea_cylindroides	-	5.11E-01	0.000	0.000	0.000	0.001	0.000
4.2.1.59|g__Firmicutes_unclassified.s__Firmicutes_bacterium_AF16_15	+	9.00E-01	0.000	0.000	0.000	0.005	0.003

4.2.1.59|g__Flavonifractor.s__Flavonifractor_plautii	+	7.66E-01	0.000	0.000	0.000	0.002	0.003
4.2.1.59|g__Flavonifractor.s__Flavonifractor_sp_An10	-	9.58E-01	0.000	0.000	0.000	0.000	0.000
4.2.1.59|g__Fusobacterium.s__Fusobacterium_mortiferum	-	3.09E-01	0.000	0.000	0.000	0.000	0.000
4.2.1.59|g__Gardnerella.s__Gardnerella_vaginalis	-	9.42E-01	0.000	0.000	0.000	0.000	0.000
4.2.1.59|g__Gordonibacter.s__Gordonibacter_sp_An232A	+	3.59E-01	0.000	0.000	0.000	0.000	0.000
4.2.1.59|g__Granulicatella.s__Granulicatella_adiacens	-	3.09E-01	0.000	0.000	0.000	0.000	0.000
4.2.1.59|g__Haemophilus.s__Haemophilus_parainfluenzae	-	1.10E-02	0.000	0.000	0.000	0.003	0.000
4.2.1.59|g__Hafnia.s__Hafnia_alvei	+	1.83E-01	0.000	0.000	0.000	0.000	0.001
4.2.1.59|g__Hafnia.s__Hafnia_paralvei	+	3.59E-01	0.000	0.000	0.000	0.000	0.001
4.2.1.59|g__Hungatella.s__Hungatella_effluvii	-	9.42E-01	0.000	0.000	0.000	0.000	0.000
4.2.1.59|g__Hungatella.s__Hungatella_hathewayi	-	2.03E-01	0.000	0.000	0.000	0.001	0.000
4.2.1.59|g__Intestinibacter.s__Intestinibacter_bartlettii	-	2.43E-01	0.000	0.000	0.000	0.000	0.000
4.2.1.59|g__Intestinimonas.s__Intestinimonas_butyriciproducens	+	8.71E-01	0.000	0.000	0.000	0.000	0.000
4.2.1.59|g__Klebsiella.s__Klebsiella_aerogenes	-	1.36E-01	0.000	0.000	0.000	0.000	0.000
4.2.1.59|g__Klebsiella.s__Klebsiella_oxytoca	-	3.48E-02	0.000	0.000	0.000	0.001	0.000
4.2.1.59|g__Klebsiella.s__Klebsiella_pneumoniae	-	1.52E-01	0.000	0.000	0.000	0.005	0.000
4.2.1.59|g__Klebsiella.s__Klebsiella_variicola	-	5.50E-01	0.000	0.000	0.000	0.001	0.000
4.2.1.59|g__Lachnoclostridium.s__Clostridium_aldenense	-	6.66E-02	0.000	0.000	0.000	0.000	0.000
4.2.1.59|g__Lachnoclostridium.s__Clostridium_asparagiforme	-	8.75E-01	0.000	0.000	0.000	0.001	0.000
4.2.1.59|g__Lachnoclostridium.s__Clostridium_bolteae	-	3.77E-01	0.000	0.000	0.000	0.000	0.000
4.2.1.59|g__Lachnoclostridium.s__Clostridium_citroniae	-	1.40E-01	0.000	0.000	0.000	0.000	0.000
4.2.1.59|g__Lachnoclostridium.s__Clostridium_clostridioforme	-	2.88E-01	0.000	0.000	0.000	0.000	0.000
4.2.1.59|g__Lachnoclostridium.s__Clostridium_lavalense	+	3.59E-01	0.000	0.000	0.000	0.000	0.000
4.2.1.59|g__Lachnoclostridium.s__Clostridium_saccharolyticum	-	3.22E-02	0.000	0.000	0.000	0.001	0.000
4.2.1.59|g__Lachnoclostridium.s__Clostridium_scindens	-	6.66E-02	0.000	0.000	0.000	0.000	0.000
4.2.1.59|g__Lachnoclostridium.s__Clostridium_symbiosum	-	9.03E-01	0.000	0.000	0.000	0.000	0.000
4.2.1.59|g__Lachnoclostridium.s__Lachnoclostridium_sp_An118	-	3.09E-01	0.000	0.000	0.000	0.000	0.000
4.2.1.59|g__Lachnospira.s__Lachnospira_pectinoschiza	+	3.47E-01	0.000	0.000	0.000	0.001	0.003
4.2.1.59|g__Lachnospiraceae_unclassified.s__Lachnospiraceae_bacterium_(	-	1.40E-01	0.000	0.000	0.000	0.000	0.000
4.2.1.59|g__Lachnospiraceae_unclassified.s__Lachnospiraceae_bacterium_(	+	1.83E-01	0.000	0.000	0.000	0.000	0.000
4.2.1.59|g__Lactobacillus.s__Lactobacillus_antri	-	9.42E-01	0.000	0.000	0.000	0.000	0.000
4.2.1.59|g__Lactobacillus.s__Lactobacillus_casei_group	-	9.42E-01	0.000	0.000	0.000	0.000	0.000
4.2.1.59|g__Lactobacillus.s__Lactobacillus_crispatus	-	3.09E-01	0.000	0.000	0.000	0.000	0.000
4.2.1.59|g__Lactobacillus.s__Lactobacillus_delbrueckii	-	3.09E-01	0.000	0.000	0.000	0.000	0.000
4.2.1.59|g__Lactobacillus.s__Lactobacillus_fermentum	-	9.42E-01	0.000	0.000	0.000	0.000	0.000
4.2.1.59|g__Lactobacillus.s__Lactobacillus_gallinarum	-	3.09E-01	0.000	0.000	0.000	0.000	0.000
4.2.1.59|g__Lactobacillus.s__Lactobacillus_gastricus	-	3.09E-01	0.000	0.000	0.000	0.000	0.000
4.2.1.59|g__Lactobacillus.s__Lactobacillus_helveticus	-	3.09E-01	0.000	0.000	0.000	0.000	0.000
4.2.1.59|g__Lactobacillus.s__Lactobacillus_kefiranofaciens	+	3.59E-01	0.000	0.000	0.000	0.000	0.001
4.2.1.59|g__Lactobacillus.s__Lactobacillus_mucosae	-	9.03E-01	0.000	0.000	0.000	0.000	0.000
4.2.1.59|g__Lactobacillus.s__Lactobacillus_oris	-	3.09E-01	0.000	0.000	0.000	0.001	0.000
4.2.1.59|g__Lactobacillus.s__Lactobacillus_paracasei	-	5.11E-01	0.000	0.000	0.000	0.000	0.000
4.2.1.59|g__Lactobacillus.s__Lactobacillus_rogosae	+	4.40E-01	0.000	0.000	0.000	0.001	0.002
4.2.1.59|g__Lactobacillus.s__Lactobacillus_sakei	+	3.59E-01	0.000	0.000	0.000	0.000	0.000
4.2.1.59|g__Lactobacillus.s__Lactobacillus_salivarius	-	3.09E-01	0.000	0.000	0.000	0.006	0.000
4.2.1.59|g__Lactobacillus.s__Lactobacillus_vaginalis	-	3.09E-01	0.000	0.000	0.000	0.000	0.000
4.2.1.59|g__Lactococcus.s__Lactococcus_lactis	-	6.31E-01	0.000	0.000	0.000	0.004	0.000
4.2.1.59|g__Listeria.s__Listeria_monocytogenes	+	6.61E-01	0.000	0.000	0.000	0.000	0.000
4.2.1.59|g__Megamonas.s__Megamonas_funiformis	+	3.59E-01	0.000	0.000	0.000	0.000	0.000
4.2.1.59|g__Megamonas.s__Megamonas_hypermegale	+	3.59E-01	0.000	0.000	0.000	0.000	0.000
4.2.1.59|g__Megamonas.s__Megamonas_rupellensis	+	3.59E-01	0.000	0.000	0.000	0.000	0.001
4.2.1.59|g__Megasphaera.s__Megasphaera_elsdenii	-	5.11E-01	0.000	0.000	0.000	0.002	0.000
4.2.1.59|g__Megasphaera.s__Megasphaera_hexanoica	-	1.40E-01	0.000	0.000	0.000	0.000	0.000
4.2.1.59|g__Megasphaera.s__Megasphaera_micronuciformis	-	3.09E-01	0.000	0.000	0.000	0.000	0.000
4.2.1.59|g__Megasphaera.s__Megasphaera_sp_DISK_18	+	3.59E-01	0.000	0.000	0.000	0.000	0.001
4.2.1.59|g__Mesosutterella.s__Mesosutterella_multiformis	+	1.02E-01	0.000	0.000	0.000	0.000	0.001
4.2.1.59|g__Mitsuokella.s__Mitsuokella_jalaludinii	-	5.37E-01	0.000	0.000	0.000	0.001	0.001
4.2.1.59|g__Mitsuokella.s__Mitsuokella_multacida	-	3.09E-01	0.000	0.000	0.000	0.000	0.000
4.2.1.59|g__Monoglobus.s__Monoglobus_pectinilyticus	+	9.80E-02	0.000	0.000	0.000	0.000	0.000
4.2.1.59|g__Obesumbacterium.s__Obesumbacterium_proteus	+	1.83E-01	0.000	0.000	0.000	0.000	0.000
4.2.1.59|g__Oxalobacter.s__Oxalobacter_formigenes	+	6.32E-01	0.000	0.000	0.000	0.000	0.000
4.2.1.59|g__Parabacteroides.s__Parabacteroides_goldsteinii	-	4.23E-01	0.000	0.000	0.000	0.000	0.000
4.2.1.59|g__Parabacteroides.s__Parabacteroides_gordonii	+	3.38E-01	0.000	0.000	0.000	0.000	0.000
4.2.1.59|g__Parabacteroides.s__Parabacteroides_johnsonii	-	2.91E-01	0.000	0.000	0.000	0.001	0.000
4.2.1.59|g__Paraprevotella.s__Paraprevotella_clara	-	3.96E-02	0.000	0.000	0.000	0.004	0.002
4.2.1.59|g__Parasutterella.s__Parasutterella_excrementihominis	+	9.92E-01	0.000	0.000	0.000	0.001	0.002
4.2.1.59|g__Phascolarctobacterium.s__Phascolarctobacterium_faecium	+	3.10E-01	0.000	0.000	0.000	0.004	0.007
4.2.1.59|g__Phascolarctobacterium.s__Phascolarctobacterium_succinatutens	-	1.39E-02	0.000	0.000	0.000	0.008	0.001
4.2.1.59|g__Prevotella.s__Prevotella_corporis	+	3.59E-01	0.000	0.000	0.000	0.000	0.000
4.2.1.59|g__Prevotella.s__Prevotella_disiens	-	3.09E-01	0.000	0.000	0.000	0.000	0.000
4.2.1.59|g__Prevotella.s__Prevotella_sp_109	+	1.83E-01	0.000	0.000	0.000	0.000	0.008
4.2.1.59|g__Pseudoflavonifractor.s__Pseudoflavonifractor_capillosus	+	3.93E-01	0.000	0.000	0.000	0.000	0.000

	4.2.1.59|g__Pseudoflavonifractor.s__Pseudoflavonifractor_sp_An184	-	9.29E-01	0.000	0.000	0.000	0.001	0.000
	4.2.1.59|g__Pseudomonas.s__Pseudomonas_aeruginosa	-	3.09E-01	0.000	0.000	0.000	0.000	0.000
	4.2.1.59|g__Pseudomonas.s__Pseudomonas_aeruginosa_group	-	3.09E-01	0.000	0.000	0.000	0.000	0.000
	4.2.1.59|g__Pyramidobacter.s__Pyramidobacter_piscolens	-	3.09E-01	0.000	0.000	0.000	0.000	0.000
	4.2.1.59|g__Raoultella.s__Raoultella_ornithinolytica	+	3.59E-01	0.000	0.000	0.000	0.000	0.000
	4.2.1.59|g__Raoultella.s__Raoultella_planticola	+	3.59E-01	0.000	0.000	0.000	0.000	0.000
	4.2.1.59|g__Ruminococcaceae_unclassified.s__Ruminococcaceae_bacteriun	-	3.04E-01	0.000	0.000	0.000	0.002	0.000
	4.2.1.59|g__Ruminococcus.s__Ruminococcus_lactaris	+	3.12E-02	0.000	0.000	0.000	0.002	0.005
	4.2.1.59|g__Ruminococcus.s__Ruminococcus_sp_AF31_8BH	+	6.18E-01	0.000	0.000	0.000	0.001	0.001
	4.2.1.59|g__Salmonella.s__Salmonella_enterica	-	4.21E-01	0.000	0.000	0.000	0.001	0.000
	4.2.1.59|g__Sanguibacteroides.s__Sanguibacteroides_justesenii	-	5.37E-01	0.000	0.000	0.000	0.000	0.000
	4.2.1.59|g__Sellimonas.s__Sellimonas_intestinalis	-	1.40E-01	0.000	0.000	0.000	0.000	0.000
	4.2.1.59|g__Staphylococcus.s__Staphylococcus_saprophyticus	+	3.59E-01	0.000	0.000	0.000	0.000	0.000
	4.2.1.59|g__Streptococcus.s__Streptococcus_anginosus_group	-	3.09E-01	0.000	0.000	0.000	0.000	0.000
	4.2.1.59|g__Streptococcus.s__Streptococcus_australis	-	1.40E-01	0.000	0.000	0.000	0.000	0.000
	4.2.1.59|g__Streptococcus.s__Streptococcus_constellatus	-	3.09E-01	0.000	0.000	0.000	0.000	0.000
	4.2.1.59|g__Streptococcus.s__Streptococcus_cristatus	-	3.09E-01	0.000	0.000	0.000	0.000	0.000
	4.2.1.59|g__Streptococcus.s__Streptococcus_equinus	+	3.59E-01	0.000	0.000	0.000	0.000	0.000
	4.2.1.59|g__Streptococcus.s__Streptococcus_gordonii	-	2.58E-01	0.000	0.000	0.000	0.000	0.000
	4.2.1.59|g__Streptococcus.s__Streptococcus_infantarius	+	3.59E-01	0.000	0.000	0.000	0.000	0.000
	4.2.1.59|g__Streptococcus.s__Streptococcus_infantis	-	1.40E-01	0.000	0.000	0.000	0.000	0.000
	4.2.1.59|g__Streptococcus.s__Streptococcus_lutetiensis	+	3.59E-01	0.000	0.000	0.000	0.000	0.000
	4.2.1.59|g__Streptococcus.s__Streptococcus_milleri	-	3.09E-01	0.000	0.000	0.000	0.000	0.000
	4.2.1.59|g__Streptococcus.s__Streptococcus_mitis	-	6.66E-02	0.000	0.000	0.000	0.000	0.000
	4.2.1.59|g__Streptococcus.s__Streptococcus_mutans	-	1.40E-01	0.000	0.000	0.000	0.000	0.000
	4.2.1.59|g__Streptococcus.s__Streptococcus_oralis	-	1.95E-01	0.000	0.000	0.000	0.000	0.000
	4.2.1.59|g__Streptococcus.s__Streptococcus_parasanguinis	-	7.14E-01	0.000	0.000	0.000	0.003	0.002
	4.2.1.59|g__Streptococcus.s__Streptococcus_pasteurianus	-	3.09E-01	0.000	0.000	0.000	0.000	0.000
	4.2.1.59|g__Streptococcus.s__Streptococcus_pneumoniae	-	2.39E-01	0.000	0.000	0.000	0.000	0.000
	4.2.1.59|g__Streptococcus.s__Streptococcus_pseudopneumoniae	-	6.66E-02	0.000	0.000	0.000	0.000	0.000
	4.2.1.59|g__Streptococcus.s__Streptococcus_sanguinis	-	1.40E-01	0.000	0.000	0.000	0.000	0.000
	4.2.1.59|g__Streptococcus.s__Streptococcus_sp_A12	+	7.41E-01	0.000	0.000	0.000	0.000	0.000
	4.2.1.59|g__Streptococcus.s__Streptococcus_vestibularis	-	9.09E-01	0.000	0.000	0.000	0.000	0.000
	4.2.1.59|g__Streptococcus.s__Streptococcus_viridans	-	8.06E-01	0.000	0.000	0.000	0.001	0.000
	4.2.1.59|g__Sutterella.s__Sutterella_wadsworthensis	-	9.80E-01	0.000	0.000	0.000	0.004	0.004
	4.2.1.59|g__Turicibacter.s__Turicibacter_sanguinis	-	3.09E-01	0.000	0.000	0.000	0.000	0.000
	4.2.1.59|g__Turicimonas.s__Turicimonas_muris	-	1.40E-01	0.000	0.000	0.000	0.000	0.000
	4.2.1.59|g__Tyzzerella.s__Tyzzerella_nexilis	-	9.03E-01	0.000	0.000	0.000	0.002	0.001
	4.2.1.59|g__Veillonella.s__Veillonella_atypica	-	3.22E-02	0.000	0.000	0.000	0.000	0.000
	4.2.1.59|g__Veillonella.s__Veillonella_dispar	-	9.03E-01	0.000	0.000	0.000	0.001	0.000
	4.2.1.59|g__Veillonella.s__Veillonella_infantium	+	6.61E-01	0.000	0.000	0.000	0.000	0.000
	4.2.1.59|g__Veillonella.s__Veillonella_parvula	+	3.56E-01	0.000	0.000	0.000	0.000	0.000
	4.2.1.59|g__Veillonella.s__Veillonella_rogosae	-	9.81E-01	0.000	0.000	0.000	0.000	0.000
	4.2.1.59|g__Lawsonibacter.s__Lawsonibacter_asaccharolyticus	-	8.48E-01	0.253	0.259	0.006	0.003	0.003
	4.2.1.59|g__Roseburia.s__Roseburia_hominis	+	4.89E-01	0.288	0.326	0.039	0.006	0.006
	4.2.1.59|g__Eubacterium.s__Eubacterium_eligens	-	9.63E-01	0.898	0.957	0.059	0.018	0.013
	4.2.1.59|g__Odoribacter.s__Odoribacter_splanchnicus	+	8.31E-01	0.480	0.558	0.078	0.004	0.004
	4.2.1.59|g__Bifidobacterium.s__Bifidobacterium_longum	+	5.29E-01	0.000	0.154	0.154	0.003	0.003
	4.2.1.59|g__Clostridium.s__Clostridium_sp_AM22_11AC	+	3.53E-01	0.175	0.427	0.252	0.005	0.005
	4.2.1.59|g__Bacteroides.s__Bacteroides_cellulosilyticus	+	3.48E-01	0.257	0.511	0.254	0.010	0.010
	4.2.1.59|g__Clostridium.s__Clostridium_sp_AF36_4	+	6.45E-02	0.000	0.256	0.256	0.002	0.005
	4.2.1.59|g__Alistipes.s__Alistipes_onderdonkii	+	9.17E-03	0.083	0.371	0.289	0.006	0.010
	4.2.1.59|g__Parabacteroides.s__Parabacteroides_merdae	+	6.18E-01	0.463	0.756	0.293	0.007	0.008
	4.2.1.59|g__Anaerostipes.s__Anaerostipes_hadrus	+	1.89E-01	0.202	0.500	0.298	0.005	0.010
	4.2.1.59|g__Bilophila.s__Bilophila_wadsworthia	+	9.17E-02	0.000	0.318	0.318	0.002	0.003
	4.2.1.59|g__Bacteroides.s__Bacteroides_intestinalis	+	2.61E-01	0.177	0.572	0.396	0.004	0.007
	4.2.1.59|g__Barnesiella.s__Barnesiella_intestinihominis	+	3.80E-01	0.647	1.236	0.589	0.009	0.011
	4.2.1.59|g__Fusicatenibacter.s__Fusicatenibacter_saccharivorans	+	4.72E-02	0.745	1.353	0.609	0.008	0.013
	4.2.1.59|g__Alistipes.s__Alistipes_shahii	+	3.27E-03	0.259	0.982	0.723	0.004	0.012
	4.2.1.59|g__Bacteroides.s__Bacteroides_uniformis	+	2.39E-01	0.813	1.649	0.836	0.022	0.034
	4.2.1.59|g__Bacteroides.s__Bacteroides_vulgatus	-	8.50E-01	2.737	3.583	0.846	0.051	0.044
	4.2.1.59|g__Alistipes.s__Alistipes_putredinis	+	2.52E-01	2.052	3.119	1.067	0.024	0.030
	4.2.1.59|unclassified	+	5.90E-01	28.729	30.225	1.497	0.230	0.257
	4.2.1.59|g__Akkermansia.s__Akkermansia_muciniphila	+	1.39E-03	0.079	2.311	2.232	0.011	0.054
China1	4.2.1.59	-	4.91E-01	117.438	112.800	-4.638	1.000	1.000
	4.2.1.59|g__Bacteroides.s__Bacteroides_vulgatus	-	1.15E-02	8.160	2.256	-5.904	0.125	0.055
	4.2.1.59|g__Bacteroides.s__Bacteroides_ovatus	-	1.30E-01	2.342	1.262	-1.079	0.053	0.023
	4.2.1.59|g__Bacteroides.s__Bacteroides_xylanisolvens	-	3.60E-01	1.342	0.691	-0.651	0.028	0.019
	4.2.1.59|g__Faecalibacterium.s__Faecalibacterium_prausnitzii	-	7.91E-01	1.230	0.902	-0.328	0.020	0.019
	4.2.1.59|g__Bacteroides.s__Bacteroides_fragilis	-	3.91E-01	0.383	0.151	-0.232	0.018	0.024
	4.2.1.59|g__Escherichia.s__Escherichia_coli	-	6.30E-01	0.407	0.288	-0.119	0.036	0.040
	4.2.1.59|g__Bacteroides.s__Bacteroides_cellulosilyticus	+	9.73E-01	0.266	0.156	-0.110	0.008	0.013

4.2.1.59|g__Blautia.s__Ruminococcus_torques	+	8.96E-01	0.557	0.467	-0.090	0.009	0.010
4.2.1.59|g__Blautia.s__Ruminococcus_gnavus	-	8.42E-03	0.087	0.000	-0.087	0.004	0.001
4.2.1.59|g__Roseburia.s__Roseburia_faecis	-	5.78E-01	0.194	0.147	-0.047	0.007	0.006
4.2.1.59|g__Flavonifractor.s__Flavonifractor_plautii	-	7.89E-01	0.129	0.087	-0.042	0.003	0.004
4.2.1.59|g__Parabacteroides.s__Parabacteroides_distasonis	+	6.83E-01	1.134	1.096	-0.038	0.023	0.017
4.2.1.59|g__Bacteroides.s__Bacteroides_plebeius	-	3.02E-01	0.012	0.000	-0.012	0.052	0.020
4.2.1.59|g__Acidaminococcus.s__Acidaminococcus_intestini	+	3.30E-01	0.000	0.000	0.000	0.000	0.000
4.2.1.59|g__Adlercreutzia.s__Adlercreutzia_equolifaciens	+	3.08E-01	0.000	0.000	0.000	0.000	0.000
4.2.1.59|g__Aeromonas.s__Aeromonas_diversa	-	3.30E-01	0.000	0.000	0.000	0.000	0.000
4.2.1.59|g__Aeromonas.s__Aeromonas_hydrophila	-	3.30E-01	0.000	0.000	0.000	0.000	0.000
4.2.1.59|g__Aeromonas.s__Aeromonas_veronii	-	3.30E-01	0.000	0.000	0.000	0.000	0.000
4.2.1.59|g__Aggregatibacter.s__Aggregatibacter_segnis	-	3.30E-01	0.000	0.000	0.000	0.000	0.000
4.2.1.59|g__Akkermansia.s__Akkermansia_muciniphila	+	1.72E-02	0.000	0.000	0.000	0.001	0.004
4.2.1.59|g__Alistipes.s__Alistipes_inops	+	6.88E-01	0.000	0.000	0.000	0.002	0.001
4.2.1.59|g__Alistipes.s__Alistipes_sp_An66	+	5.49E-01	0.000	0.000	0.000	0.000	0.000
4.2.1.59|g__Alistipes.s__Alistipes_timonensis	+	6.55E-01	0.000	0.000	0.000	0.001	0.001
4.2.1.59|g__Allisonella.s__Allisonella_histaminiformans	+	5.18E-01	0.000	0.000	0.000	0.000	0.000
4.2.1.59|g__Anaeroglobus.s__Anaeroglobus_geminatus	+	3.30E-01	0.000	0.000	0.000	0.000	0.000
4.2.1.59|g__Anaerostipes.s__Anaerostipes_hadrus	+	7.04E-01	0.000	0.000	0.000	0.001	0.001
4.2.1.59|g__Anaerotruncus.s__Anaerotruncus_colihominis	-	9.73E-01	0.000	0.000	0.000	0.000	0.000
4.2.1.59|g__Bacteroides.s__Bacteroides_clarus	+	2.08E-01	0.000	0.000	0.000	0.001	0.005
4.2.1.59|g__Bacteroides.s__Bacteroides_coprocola	-	1.98E-01	0.000	0.000	0.000	0.038	0.011
4.2.1.59|g__Bacteroides.s__Bacteroides_coprophilus	-	3.93E-01	0.000	0.000	0.000	0.005	0.001
4.2.1.59|g__Bacteroides.s__Bacteroides_eggerthii	+	1.55E-01	0.000	0.000	0.000	0.015	0.029
4.2.1.59|g__Bacteroides.s__Bacteroides_faecichinchillae	+	4.28E-02	0.000	0.000	0.000	0.000	0.000
4.2.1.59|g__Bacteroides.s__Bacteroides_faecis	-	1.58E-01	0.000	0.000	0.000	0.001	0.000
4.2.1.59|g__Bacteroides.s__Bacteroides_finegoldii	+	2.73E-01	0.000	0.000	0.000	0.007	0.010
4.2.1.59|g__Bacteroides.s__Bacteroides_fluxus	+	3.30E-01	0.000	0.000	0.000	0.000	0.000
4.2.1.59|g__Bacteroides.s__Bacteroides_massiliensis	+	9.23E-01	0.000	0.000	0.000	0.008	0.011
4.2.1.59|g__Bacteroides.s__Bacteroides_oleiciplenus	+	9.90E-01	0.000	0.000	0.000	0.000	0.000
4.2.1.59|g__Bacteroides.s__Bacteroides_salyersiae	-	7.33E-01	0.000	0.000	0.000	0.002	0.001
4.2.1.59|g__Bacteroides.s__Bacteroides_sp_OM08_11	+	8.20E-02	0.000	0.000	0.000	0.000	0.000
4.2.1.59|g__Bacteroides.s__Bacteroides_stercorirosoris	+	9.70E-01	0.000	0.000	0.000	0.000	0.001
4.2.1.59|g__Barnesiella.s__Barnesiella_intestinihominis	+	3.03E-01	0.000	0.000	0.000	0.006	0.005
4.2.1.59|g__Barnesiella.s__Barnesiella_sp_An22	-	3.30E-01	0.000	0.000	0.000	0.000	0.000
4.2.1.59|g__Bifidobacterium.s__Bifidobacterium_animalis	-	3.30E-01	0.000	0.000	0.000	0.000	0.000
4.2.1.59|g__Bifidobacterium.s__Bifidobacterium_breve	+	1.00E+00	0.000	0.000	0.000	0.000	0.000
4.2.1.59|g__Bifidobacterium.s__Bifidobacterium_longum	-	6.87E-01	0.000	0.000	0.000	0.000	0.000
4.2.1.59|g__Blautia.s__Blautia_sp_AF19_10LB	+	9.66E-01	0.000	0.000	0.000	0.000	0.000
4.2.1.59|g__Blautia.s__Blautia_wexlerae	-	3.38E-01	0.000	0.000	0.000	0.000	0.000
4.2.1.59|g__Butyrivibrio.s__Butyrivibrio_crossotus	+	5.69E-01	0.000	0.000	0.000	0.000	0.001
4.2.1.59|g__Campylobacter.s__Campylobacter_concisus	-	3.30E-01	0.000	0.000	0.000	0.000	0.000
4.2.1.59|g__Campylobacter.s__Campylobacter_gracilis	+	3.30E-01	0.000	0.000	0.000	0.000	0.000
4.2.1.59|g__Catenibacterium.s__Catenibacterium_mitsuokai	+	6.06E-01	0.000	0.000	0.000	0.001	0.002
4.2.1.59|g__Citrobacter.s__Citrobacter_amalonaticus	+	1.60E-01	0.000	0.000	0.000	0.000	0.000
4.2.1.59|g__Citrobacter.s__Citrobacter_braakii	+	8.20E-02	0.000	0.000	0.000	0.000	0.000
4.2.1.59|g__Citrobacter.s__Citrobacter_farmeri	+	3.30E-01	0.000	0.000	0.000	0.000	0.000
4.2.1.59|g__Citrobacter.s__Citrobacter_freundii	+	1.58E-01	0.000	0.000	0.000	0.000	0.000
4.2.1.59|g__Citrobacter.s__Citrobacter_portucalensis	+	5.69E-01	0.000	0.000	0.000	0.000	0.000
4.2.1.59|g__Citrobacter.s__Citrobacter_youngae	+	9.90E-01	0.000	0.000	0.000	0.000	0.000
4.2.1.59|g__Cloacibacillus.s__Cloacibacillus_porcorum	+	8.20E-02	0.000	0.000	0.000	0.000	0.000
4.2.1.59|g__Clostridiales_unclassified.s__Clostridiales_bacterium_KLE1615	+	5.27E-01	0.000	0.000	0.000	0.001	0.001
4.2.1.59|g__Clostridioides.s__Clostridioides_difficile	+	7.61E-01	0.000	0.000	0.000	0.000	0.000
4.2.1.59|g__Clostridium.s__Clostridium_disporicum	-	3.30E-01	0.000	0.000	0.000	0.000	0.000
4.2.1.59|g__Clostridium.s__Clostridium_neonatale	-	1.60E-01	0.000	0.000	0.000	0.000	0.000
4.2.1.59|g__Clostridium.s__Clostridium_perfringens	-	3.30E-01	0.000	0.000	0.000	0.000	0.000
4.2.1.59|g__Clostridium.s__Clostridium_sp_7_2_43FAA	+	3.30E-01	0.000	0.000	0.000	0.000	0.000
4.2.1.59|g__Clostridium.s__Clostridium_sp_AF36_4	+	5.18E-02	0.000	0.000	0.000	0.001	0.005
4.2.1.59|g__Clostridium.s__Clostridium_sp_AM22_11AC	-	8.44E-01	0.000	0.000	0.000	0.002	0.001
4.2.1.59|g__Clostridium.s__Clostridium_sp_chh4_2	+	3.30E-01	0.000	0.000	0.000	0.000	0.000
4.2.1.59|g__Coprobacillus.s__Coprobacillus_cateniformis	+	8.40E-02	0.000	0.000	0.000	0.000	0.000
4.2.1.59|g__Coprobacter.s__Coprobacter_fastidiosus	+	7.00E-01	0.000	0.000	0.000	0.001	0.001
4.2.1.59|g__Coprobacter.s__Coprobacter_secundus	+	9.47E-01	0.000	0.000	0.000	0.000	0.000
4.2.1.59|g__Coprococcus.s__Coprococcus_catus	+	1.91E-01	0.000	0.000	0.000	0.000	0.000
4.2.1.59|g__Coprococcus.s__Coprococcus_comes	+	6.94E-01	0.000	0.000	0.000	0.001	0.002
4.2.1.59|g__Desulfovibrio.s__Desulfovibrio_piger	+	6.59E-01	0.000	0.000	0.000	0.000	0.001
4.2.1.59|g__Desulfovibrio.s__Desulfovibrio_sp_AM18_2	+	8.20E-02	0.000	0.000	0.000	0.000	0.001
4.2.1.59|g__Desulfovibrionaceae_unclassified.s__Desulfovibrionaceae_bact	+	1.60E-01	0.000	0.000	0.000	0.000	0.000
4.2.1.59|g__Dialister.s__Dialister_invisus	+	8.14E-01	0.000	0.000	0.000	0.003	0.013
4.2.1.59|g__Dialister.s__Dialister_succinatiphilus	+	1.00E+00	0.000	0.000	0.000	0.000	0.001
4.2.1.59|g__Dorea.s__Dorea_formicigenerans	-	3.47E-01	0.000	0.000	0.000	0.001	0.000
4.2.1.59|g__Dorea.s__Dorea_longicatena	+	6.97E-01	0.000	0.000	0.000	0.001	0.001

4.2.1.59|g__Dorea.s__Dorea_sp_OM02_2LB	+	3.30E-01	0.000	0.000	0.000	0.000	0.000
4.2.1.59|g__Eisenbergiella.s__Eisenbergiella_massiliensis	+	5.49E-01	0.000	0.000	0.000	0.000	0.001
4.2.1.59|g__Eisenbergiella.s__Eisenbergiella_tayi	+	1.18E-02	0.000	0.000	0.000	0.000	0.000
4.2.1.59|g__Enterobacter.s__Enterobacter_asburiae	+	3.30E-01	0.000	0.000	0.000	0.000	0.000
4.2.1.59|g__Enterobacter.s__Enterobacter_cloacae	+	1.58E-01	0.000	0.000	0.000	0.000	0.000
4.2.1.59|g__Enterococcus.s__Enterococcus_faecium	+	1.60E-01	0.000	0.000	0.000	0.000	0.000
4.2.1.59|g__Enterococcus.s__Enterococcus_hirae	+	3.30E-01	0.000	0.000	0.000	0.000	0.000
4.2.1.59|g__Erysipelatoclostridium.s__Clostridium_innocuum	+	6.55E-01	0.000	0.000	0.000	0.000	0.000
4.2.1.59|g__Erysipelatoclostridium.s__Erysipelatoclostridium_ramosum	-	3.13E-01	0.000	0.000	0.000	0.000	0.000
4.2.1.59|g__Escherichia.s__Escherichia_fergusonii	-	6.30E-01	0.000	0.000	0.000	0.000	0.000
4.2.1.59|g__Eubacterium.s__Eubacterium_ramulus	+	9.70E-01	0.000	0.000	0.000	0.000	0.000
4.2.1.59|g__Eubacterium.s__Eubacterium_sp_AF17_7	+	4.41E-01	0.000	0.000	0.000	0.000	0.001
4.2.1.59|g__Eubacterium.s__Eubacterium_ventriosum	+	5.94E-01	0.000	0.000	0.000	0.006	0.005
4.2.1.59|g__Faecalitalea.s__Faecalitalea_cylindroides	+	3.30E-01	0.000	0.000	0.000	0.000	0.000
4.2.1.59|g__Firmicutes_unclassified.s__Firmicutes_bacterium_AF16_15	+	8.76E-02	0.000	0.000	0.000	0.001	0.002
4.2.1.59|g__Fusobacterium.s__Fusobacterium_hwasookii	-	3.30E-01	0.000	0.000	0.000	0.000	0.000
4.2.1.59|g__Fusobacterium.s__Fusobacterium_mortiferum	+	6.55E-01	0.000	0.000	0.000	0.001	0.000
4.2.1.59|g__Fusobacterium.s__Fusobacterium_ulcerans	-	6.55E-01	0.000	0.000	0.000	0.001	0.001
4.2.1.59|g__Fusobacterium.s__Fusobacterium_varium	-	1.60E-01	0.000	0.000	0.000	0.000	0.000
4.2.1.59|g__Gardnerella.s__Gardnerella_vaginalis	+	8.20E-02	0.000	0.000	0.000	0.000	0.000
4.2.1.59|g__Gemmiger.s__Gemmiger_formicilis	+	5.49E-01	0.000	0.000	0.000	0.001	0.001
4.2.1.59|g__Haemophilus.s__Haemophilus_parainfluenzae	+	9.96E-01	0.000	0.000	0.000	0.007	0.007
4.2.1.59|g__Haemophilus.s__Haemophilus_paraphrohaemolyticus	+	3.30E-01	0.000	0.000	0.000	0.000	0.000
4.2.1.59|g__Haemophilus.s__Haemophilus_sputorum	+	5.69E-01	0.000	0.000	0.000	0.000	0.000
4.2.1.59|g__Hafnia.s__Hafnia_paralvei	+	1.00E+00	0.000	0.000	0.000	0.000	0.000
4.2.1.59|g__Hungatella.s__Hungatella_effluvii	+	6.38E-01	0.000	0.000	0.000	0.000	0.000
4.2.1.59|g__Hungatella.s__Hungatella_hathewayi	-	6.69E-01	0.000	0.000	0.000	0.002	0.001
4.2.1.59|g__Intestinibacter.s__Intestinibacter_bartlettii	+	7.91E-01	0.000	0.000	0.000	0.000	0.000
4.2.1.59|g__Intestinimonas.s__Intestinimonas_butyriciproducens	+	4.71E-02	0.000	0.000	0.000	0.000	0.001
4.2.1.59|g__Klebsiella.s__Klebsiella_aerogenes	-	7.78E-01	0.000	0.000	0.000	0.000	0.000
4.2.1.59|g__Klebsiella.s__Klebsiella_oxytoca	+	4.75E-03	0.000	0.000	0.000	0.000	0.001
4.2.1.59|g__Klebsiella.s__Klebsiella_pneumoniae	+	2.47E-01	0.000	0.000	0.000	0.002	0.005
4.2.1.59|g__Klebsiella.s__Klebsiella_variicola	+	3.56E-02	0.000	0.000	0.000	0.000	0.001
4.2.1.59|g__Kosakonia.s__Kosakonia_cowanii	-	3.30E-01	0.000	0.000	0.000	0.000	0.000
4.2.1.59|g__Lachnoclostridium.s__Clostridium_aldenense	-	8.85E-01	0.000	0.000	0.000	0.000	0.000
4.2.1.59|g__Lachnoclostridium.s__Clostridium_asparagiforme	+	8.20E-02	0.000	0.000	0.000	0.000	0.000
4.2.1.59|g__Lachnoclostridium.s__Clostridium_bolteae	+	8.37E-01	0.000	0.000	0.000	0.001	0.001
4.2.1.59|g__Lachnoclostridium.s__Clostridium_citroniae	+	1.00E-01	0.000	0.000	0.000	0.000	0.000
4.2.1.59|g__Lachnoclostridium.s__Clostridium_clostridioforme	-	2.73E-01	0.000	0.000	0.000	0.000	0.000
4.2.1.59|g__Lachnoclostridium.s__Clostridium_lavalense	+	1.60E-01	0.000	0.000	0.000	0.000	0.000
4.2.1.59|g__Lachnoclostridium.s__Clostridium_saccharolyticum	+	3.30E-01	0.000	0.000	0.000	0.000	0.000
4.2.1.59|g__Lachnoclostridium.s__Clostridium_scindens	+	3.30E-01	0.000	0.000	0.000	0.000	0.000
4.2.1.59|g__Lachnoclostridium.s__Clostridium_symbiosum	-	6.55E-01	0.000	0.000	0.000	0.000	0.000
4.2.1.59|g__Lachnospira.s__Lachnospira_pectinoschiza	-	2.03E-01	0.000	0.000	0.000	0.004	0.002
4.2.1.59|g__Lachnospiraceae_unclassified.s__Lachnospiraceae_bacterium_2	-	3.30E-01	0.000	0.000	0.000	0.000	0.000
4.2.1.59|g__Lactobacillus.s__Lactobacillus_delbrueckii	+	3.30E-01	0.000	0.000	0.000	0.000	0.000
4.2.1.59|g__Lactobacillus.s__Lactobacillus_fermentum	+	1.00E+00	0.000	0.000	0.000	0.000	0.000
4.2.1.59|g__Lactobacillus.s__Lactobacillus_mucosae	+	2.59E-01	0.000	0.000	0.000	0.000	0.001
4.2.1.59|g__Lactobacillus.s__Lactobacillus_rogosae	-	1.02E-01	0.000	0.000	0.000	0.004	0.002
4.2.1.59|g__Lactobacillus.s__Lactobacillus_salivarius	+	5.56E-02	0.000	0.000	0.000	0.000	0.006
4.2.1.59|g__Lawsonibacter.s__Lawsonibacter_asaccharolyticus	+	5.46E-01	0.000	0.000	0.000	0.000	0.000
4.2.1.59|g__Megamonas.s__Megamonas_funiformis	+	7.72E-01	0.000	0.000	0.000	0.003	0.001
4.2.1.59|g__Megamonas.s__Megamonas_hypermegale	-	9.73E-01	0.000	0.000	0.000	0.001	0.000
4.2.1.59|g__Megamonas.s__Megamonas_rupellensis	+	7.78E-01	0.000	0.000	0.000	0.003	0.002
4.2.1.59|g__Megasphaera.s__Megasphaera_micronuciformis	-	3.30E-01	0.000	0.000	0.000	0.000	0.000
4.2.1.59|g__Mesosutterella.s__Mesosutterella_multiformis	+	8.57E-01	0.000	0.000	0.000	0.004	0.005
4.2.1.59|g__Mitsuokella.s__Mitsuokella_multacida	-	3.30E-01	0.000	0.000	0.000	0.000	0.000
4.2.1.59|g__Monoglobus.s__Monoglobus_pectinilyticus	+	1.60E-01	0.000	0.000	0.000	0.000	0.000
4.2.1.59|g__Oxalobacter.s__Oxalobacter_formigenes	+	9.66E-01	0.000	0.000	0.000	0.000	0.001
4.2.1.59|g__Parabacteroides.s__Parabacteroides_goldsteinii	+	1.36E-01	0.000	0.000	0.000	0.001	0.003
4.2.1.59|g__Parabacteroides.s__Parabacteroides_gordonii	+	7.47E-01	0.000	0.000	0.000	0.000	0.001
4.2.1.59|g__Parabacteroides.s__Parabacteroides_johnsonii	+	6.24E-02	0.000	0.000	0.000	0.001	0.003
4.2.1.59|g__Paraprevotella.s__Paraprevotella_clara	+	5.90E-03	0.000	0.000	0.000	0.002	0.005
4.2.1.59|g__Paraprevotella.s__Paraprevotella_xylaniphila	+	9.70E-01	0.000	0.000	0.000	0.000	0.001
4.2.1.59|g__Parasutterella.s__Parasutterella_excrementihominis	-	5.20E-01	0.000	0.000	0.000	0.004	0.003
4.2.1.59|g__Peptostreptococcaceae_unclassified.s__Clostridium_hiranonis	-	3.30E-01	0.000	0.000	0.000	0.000	0.000
4.2.1.59|g__Phascolarctobacterium.s__Phascolarctobacterium_succinatutens	+	1.91E-01	0.000	0.000	0.000	0.003	0.004
4.2.1.59|g__Prevotella.s__Prevotella_bivia	+	3.30E-01	0.000	0.000	0.000	0.000	0.000
4.2.1.59|g__Prevotella.s__Prevotella_corporis	-	3.30E-01	0.000	0.000	0.000	0.000	0.000
4.2.1.59|g__Prevotella.s__Prevotella_multisaccharivorax	-	1.60E-01	0.000	0.000	0.000	0.000	0.000
4.2.1.59|g__Prevotella.s__Prevotella_sp_109	-	1.60E-01	0.000	0.000	0.000	0.001	0.000
4.2.1.59|g__Proteus.s__Proteus_mirabilis	+	3.30E-01	0.000	0.000	0.000	0.000	0.000

	4.2.1.59|g__Pseudocitrobacter.s__Pseudocitrobacter_faecalis	+	3.30E-01	0.000	0.000	0.000	0.000	0.000
	4.2.1.59|g__Pyramidobacter.s__Pyramidobacter_piscolens	+	3.27E-02	0.000	0.000	0.000	0.000	0.001
	4.2.1.59|g__Pyramidobacter.s__Pyramidobacter_sp_C12_8	+	3.30E-01	0.000	0.000	0.000	0.000	0.000
	4.2.1.59|g__Roseburia.s__Roseburia_intestinalis	-	8.77E-01	0.000	0.000	0.000	0.001	0.001
	4.2.1.59|g__Ruminococcaceae_unclassified.s__Clostridium_methylpentosun	+	3.30E-01	0.000	0.000	0.000	0.000	0.000
	4.2.1.59|g__Ruminococcaceae_unclassified.s__Ruminococcaceae_bacteriun	-	3.30E-01	0.000	0.000	0.000	0.000	0.000
	4.2.1.59|g__Ruminococcus.s__Ruminococcus_lactaris	-	9.61E-01	0.000	0.000	0.000	0.001	0.002
	4.2.1.59|g__Ruminococcus.s__Ruminococcus_sp_AF31_8BH	+	9.70E-01	0.000	0.000	0.000	0.000	0.000
	4.2.1.59|g__Salmonella.s__Salmonella_enterica	+	8.19E-01	0.000	0.000	0.000	0.001	0.002
	4.2.1.59|g__Sanguibacteroides.s__Sanguibacteroides_justesenii	+	9.70E-01	0.000	0.000	0.000	0.000	0.000
	4.2.1.59|g__Sellimonas.s__Sellimonas_intestinalis	+	6.55E-01	0.000	0.000	0.000	0.000	0.000
	4.2.1.59|g__Streptococcus.s__Streptococcus_cristatus	+	3.30E-01	0.000	0.000	0.000	0.000	0.000
	4.2.1.59|g__Streptococcus.s__Streptococcus_gallolyticus	-	3.30E-01	0.000	0.000	0.000	0.000	0.000
	4.2.1.59|g__Streptococcus.s__Streptococcus_gordonii	+	3.30E-01	0.000	0.000	0.000	0.000	0.000
	4.2.1.59|g__Streptococcus.s__Streptococcus_mitis	+	1.60E-01	0.000	0.000	0.000	0.000	0.000
	4.2.1.59|g__Streptococcus.s__Streptococcus_mutans	+	3.30E-01	0.000	0.000	0.000	0.000	0.000
	4.2.1.59|g__Streptococcus.s__Streptococcus_oralis	+	8.20E-02	0.000	0.000	0.000	0.000	0.000
	4.2.1.59|g__Streptococcus.s__Streptococcus_parasanguinis	+	2.58E-01	0.000	0.000	0.000	0.000	0.001
	4.2.1.59|g__Streptococcus.s__Streptococcus_pneumoniae	+	1.60E-01	0.000	0.000	0.000	0.000	0.000
	4.2.1.59|g__Streptococcus.s__Streptococcus_pseudopneumoniae	+	1.60E-01	0.000	0.000	0.000	0.000	0.000
	4.2.1.59|g__Streptococcus.s__Streptococcus_salivarius	+	1.41E-02	0.000	0.000	0.000	0.000	0.002
	4.2.1.59|g__Streptococcus.s__Streptococcus_thermophilus	+	1.65E-01	0.000	0.000	0.000	0.000	0.000
	4.2.1.59|g__Streptococcus.s__Streptococcus_vestibularis	+	3.08E-01	0.000	0.000	0.000	0.000	0.000
	4.2.1.59|g__Streptococcus.s__Streptococcus_viridans	+	1.60E-01	0.000	0.000	0.000	0.000	0.000
	4.2.1.59|g__Succinatimonas.s__Succinatimonas_hippei	-	3.30E-01	0.000	0.000	0.000	0.000	0.000
	4.2.1.59|g__Sutterella.s__Sutterella_parvirubra	-	3.30E-01	0.000	0.000	0.000	0.001	0.000
	4.2.1.59|g__Sutterella.s__Sutterella_wadsworthensis	+	2.36E-01	0.000	0.000	0.000	0.002	0.004
	4.2.1.59|g__Synergistes.s__Synergistes_jonesii	+	3.30E-01	0.000	0.000	0.000	0.000	0.000
	4.2.1.59|g__Turicibacter.s__Turicibacter_sanguinis	-	1.00E+00	0.000	0.000	0.000	0.000	0.000
	4.2.1.59|g__Turicimonas.s__Turicimonas_muris	+	1.60E-01	0.000	0.000	0.000	0.000	0.000
	4.2.1.59|g__Tyzzerella.s__Tyzzerella_nexilis	+	7.59E-01	0.000	0.000	0.000	0.001	0.001
	4.2.1.59|g__Veillonella.s__Veillonella_atypica	+	4.57E-01	0.000	0.000	0.000	0.001	0.001
	4.2.1.59|g__Veillonella.s__Veillonella_denticariosi	-	3.30E-01	0.000	0.000	0.000	0.000	0.000
	4.2.1.59|g__Veillonella.s__Veillonella_dispar	+	4.76E-01	0.000	0.000	0.000	0.006	0.001
	4.2.1.59|g__Veillonella.s__Veillonella_infantium	+	4.05E-01	0.000	0.000	0.000	0.000	0.000
	4.2.1.59|g__Veillonella.s__Veillonella_parvula	+	3.32E-01	0.000	0.000	0.000	0.003	0.002
	4.2.1.59|g__Veillonella.s__Veillonella_rogosae	+	1.72E-01	0.000	0.000	0.000	0.000	0.000
	4.2.1.59|g__Veillonella.s__Veillonella_tobetsuensis	-	9.90E-01	0.000	0.000	0.000	0.000	0.000
	4.2.1.59|g__Fusicatenibacter.s__Fusicatenibacter_saccharivorans	+	9.71E-01	0.000	0.030	0.030	0.002	0.002
	4.2.1.59|g__Roseburia.s__Roseburia_inulinivorans	+	8.94E-01	0.171	0.201	0.030	0.007	0.007
	4.2.1.59|g__Alistipes.s__Alistipes_onderdonkii	+	1.93E-02	0.000	0.040	0.040	0.002	0.004
	4.2.1.59|g__Roseburia.s__Roseburia_hominis	+	4.54E-01	0.000	0.044	0.044	0.003	0.004
	4.2.1.59|g__Alistipes.s__Alistipes_indistinctus	+	2.76E-02	0.000	0.064	0.064	0.002	0.003
	4.2.1.59|g__Bacteroides.s__Bacteroides_nordii	-	7.85E-01	0.072	0.136	0.064	0.003	0.005
	4.2.1.59|g__Blautia.s__Blautia_obeum	+	2.85E-01	0.021	0.097	0.075	0.002	0.003
	4.2.1.59|g__Lachnospiraceae_unclassified.s__Eubacterium_rectale	+	8.60E-01	0.041	0.123	0.081	0.007	0.004
	4.2.1.59|g__Bacteroides.s__Bacteroides_thetaiotaomicron	+	9.42E-01	1.776	1.878	0.102	0.034	0.039
	4.2.1.59|g__Bacteroides.s__Bacteroides_dorei	-	4.91E-01	0.666	0.787	0.121	0.048	0.028
	4.2.1.59|g__Bilophila.s__Bilophila_wadsworthia	+	1.09E-01	0.000	0.179	0.179	0.002	0.002
	4.2.1.59|unclassified	+	2.83E-01	7.002	7.236	0.235	0.090	0.115
	4.2.1.59|g__Odoribacter.s__Odoribacter_splanchnicus	-	9.80E-01	0.000	0.271	0.271	0.006	0.004
	4.2.1.59|g__Parabacteroides.s__Parabacteroides_merdae	+	2.16E-01	0.082	0.381	0.299	0.009	0.012
	4.2.1.59|g__Alistipes.s__Alistipes_finegoldii	+	1.11E-01	0.122	0.454	0.332	0.010	0.035
	4.2.1.59|g__Phascolarctobacterium.s__Phascolarctobacterium_faecium	+	3.21E-01	0.823	1.248	0.425	0.026	0.025
	4.2.1.59|g__Bacteroides.s__Bacteroides_intestinalis	+	2.11E-02	0.221	0.669	0.448	0.005	0.016
	4.2.1.59|g__Bacteroides.s__Bacteroides_stercoris	+	8.73E-01	0.252	0.763	0.511	0.054	0.036
	4.2.1.59|g__Eubacterium.s__Eubacterium_eligens	+	4.77E-02	0.316	0.902	0.585	0.014	0.024
	4.2.1.59|g__Bacteroides.s__Bacteroides_caccae	+	4.36E-01	0.159	0.775	0.616	0.013	0.019
	4.2.1.59|g__Alistipes.s__Alistipes_shahii	+	1.93E-02	0.206	1.151	0.945	0.010	0.019
	4.2.1.59|g__Bacteroides.s__Bacteroides_uniformis	+	2.13E-01	4.703	6.498	1.796	0.055	0.080
	4.2.1.59|g__Alistipes.s__Alistipes_putredinis	+	6.97E-02	0.951	3.577	2.626	0.035	0.057
China2	4.2.1.59	-	1.01E-01	154.060	142.159	-11.901	1.000	1.000
	4.2.1.59|g__Bacteroides.s__Bacteroides_vulgatus	-	4.15E-02	8.259	3.652	-4.607	0.094	0.060
	4.2.1.59|g__Faecalibacterium.s__Faecalibacterium_prausnitzii	-	1.31E-01	7.913	4.716	-3.196	0.074	0.056
	4.2.1.59|g__Bacteroides.s__Bacteroides_dorei	-	2.30E-01	2.270	0.815	-1.455	0.041	0.027
	4.2.1.59|g__Roseburia.s__Roseburia_faecis	-	1.27E-01	1.308	0.135	-1.173	0.028	0.013
	4.2.1.59|g__Phascolarctobacterium.s__Phascolarctobacterium_faecium	-	4.83E-01	0.887	0.224	-0.663	0.009	0.010
	4.2.1.59|g__Clostridium.s__Clostridium_sp_AM22_11AC	-	2.93E-01	1.045	0.793	-0.252	0.013	0.012
	4.2.1.59|g__Roseburia.s__Roseburia_inulinivorans	-	2.30E-01	0.439	0.216	-0.223	0.006	0.005
	4.2.1.59|g__Bacteroides.s__Bacteroides_thetaiotaomicron	-	2.30E-01	0.523	0.371	-0.152	0.012	0.008
	4.2.1.59|g__Blautia.s__Ruminococcus_gnavus	-	2.71E-01	0.143	0.000	-0.143	0.005	0.004
	4.2.1.59|g__Bacteroides.s__Bacteroides_xylanisolvens	-	1.32E-01	0.355	0.244	-0.111	0.019	0.011

4.2.1.59|g__Roseburia.s__Roseburia_intestinalis	-	7.24E-01	0.073	0.000	-0.073	0.006	0.003
4.2.1.59|g__Fusicatenibacter.s__Fusicatenibacter_saccharivorans	-	8.96E-01	0.324	0.259	-0.065	0.005	0.005
4.2.1.59|g__Streptococcus.s__Streptococcus_salivarius	-	3.56E-01	0.122	0.075	-0.047	0.002	0.003
4.2.1.59|g__Veillonella.s__Veillonella_parvula	+	9.49E-01	0.033	0.000	-0.033	0.002	0.003
4.2.1.59|g__Lachnospiraceae_unclassified.s__Eubacterium_rectale	-	8.30E-01	0.261	0.233	-0.028	0.005	0.005
4.2.1.59|g__Dorea.s__Dorea_longicatena	+	5.57E-01	0.144	0.123	-0.020	0.001	0.001
4.2.1.59|g__Dorea.s__Dorea_formicigenerans	+	5.52E-01	0.067	0.054	-0.013	0.000	0.001
4.2.1.59|g__Bacteroides.s__Bacteroides_fragilis	-	9.83E-01	0.045	0.043	-0.003	0.007	0.010
4.2.1.59|g__Gemmiger.s__Gemmiger_formicilis	-	8.33E-01	0.327	0.325	-0.002	0.011	0.007
4.2.1.59|g__Blautia.s__Blautia_wexlerae	+	5.34E-01	0.147	0.145	-0.002	0.004	0.006
4.2.1.59|g__Parabacteroides.s__Parabacteroides_distasonis	+	4.78E-01	1.109	1.107	-0.001	0.013	0.019
4.2.1.59|g__Acidaminococcus.s__Acidaminococcus_intestini	-	5.69E-01	0.000	0.000	0.000	0.000	0.000
4.2.1.59|g__Adlercreutzia.s__Adlercreutzia_equolifaciens	+	2.96E-01	0.000	0.000	0.000	0.000	0.000
4.2.1.59|g__Aggregatibacter.s__Aggregatibacter_segnis	-	1.60E-01	0.000	0.000	0.000	0.000	0.000
4.2.1.59|g__Akkermansia.s__Akkermansia_muciniphila	+	6.20E-01	0.000	0.000	0.000	0.001	0.006
4.2.1.59|g__Alcaligenes.s__Alcaligenes_faecalis	+	3.30E-01	0.000	0.000	0.000	0.000	0.001
4.2.1.59|g__Alistipes.s__Alistipes_indistinctus	+	6.02E-04	0.000	0.000	0.000	0.000	0.002
4.2.1.59|g__Alistipes.s__Alistipes_inops	+	5.89E-01	0.000	0.000	0.000	0.000	0.000
4.2.1.59|g__Alistipes.s__Alistipes_timonensis	+	1.00E+00	0.000	0.000	0.000	0.000	0.000
4.2.1.59|g__Allisonella.s__Allisonella_histaminiformans	+	4.09E-01	0.000	0.000	0.000	0.000	0.000
4.2.1.59|g__Anaerostipes.s__Anaerostipes_caccae	-	3.30E-01	0.000	0.000	0.000	0.000	0.000
4.2.1.59|g__Anaerotruncus.s__Anaerotruncus_colihominis	+	2.37E-01	0.000	0.000	0.000	0.000	0.000
4.2.1.59|g__Atlantibacter.s__Atlantibacter_hermannii	-	3.30E-01	0.000	0.000	0.000	0.000	0.000
4.2.1.59|g__Bacteroides.s__Bacteroides_clarus	-	5.75E-01	0.000	0.000	0.000	0.001	0.003
4.2.1.59|g__Bacteroides.s__Bacteroides_coprocola	-	9.04E-02	0.000	0.000	0.000	0.025	0.008
4.2.1.59|g__Bacteroides.s__Bacteroides_coprophilus	+	2.85E-01	0.000	0.000	0.000	0.005	0.004
4.2.1.59|g__Bacteroides.s__Bacteroides_eggerthii	-	2.85E-01	0.000	0.000	0.000	0.008	0.002
4.2.1.59|g__Bacteroides.s__Bacteroides_faecichinchillae	+	2.42E-01	0.000	0.000	0.000	0.000	0.000
4.2.1.59|g__Bacteroides.s__Bacteroides_faecis	+	4.05E-01	0.000	0.000	0.000	0.000	0.001
4.2.1.59|g__Bacteroides.s__Bacteroides_finegoldii	-	1.54E-01	0.000	0.000	0.000	0.009	0.001
4.2.1.59|g__Bacteroides.s__Bacteroides_fluxus	+	3.30E-01	0.000	0.000	0.000	0.000	0.000
4.2.1.59|g__Bacteroides.s__Bacteroides_massiliensis	-	5.23E-01	0.000	0.000	0.000	0.016	0.004
4.2.1.59|g__Bacteroides.s__Bacteroides_nordii	+	2.17E-01	0.000	0.000	0.000	0.001	0.001
4.2.1.59|g__Bacteroides.s__Bacteroides_oleiciplenus	-	1.60E-01	0.000	0.000	0.000	0.000	0.000
4.2.1.59|g__Bacteroides.s__Bacteroides_plebeius	-	3.00E-01	0.000	0.000	0.000	0.067	0.030
4.2.1.59|g__Bacteroides.s__Bacteroides_salyersiae	-	4.61E-01	0.000	0.000	0.000	0.001	0.001
4.2.1.59|g__Bacteroides.s__Bacteroides_sartorii	-	3.30E-01	0.000	0.000	0.000	0.000	0.000
4.2.1.59|g__Bacteroides.s__Bacteroides_sp_OM08_11	-	3.30E-01	0.000	0.000	0.000	0.000	0.000
4.2.1.59|g__Bacteroides.s__Bacteroides_stercorirosoris	+	5.89E-01	0.000	0.000	0.000	0.000	0.000
4.2.1.59|g__Barnesiella.s__Barnesiella_intestinihominis	+	6.33E-01	0.000	0.000	0.000	0.003	0.005
4.2.1.59|g__Bifidobacterium.s__Bifidobacterium_longum	+	5.39E-02	0.000	0.000	0.000	0.000	0.001
4.2.1.59|g__Blautia.s__Blautia_hydrogenotrophica	+	1.60E-01	0.000	0.000	0.000	0.000	0.000
4.2.1.59|g__Blautia.s__Blautia_sp_AF19_10LB	+	4.03E-01	0.000	0.000	0.000	0.002	0.005
4.2.1.59|g__Butyrivibrio.s__Butyrivibrio_crossotus	+	3.30E-01	0.000	0.000	0.000	0.000	0.007
4.2.1.59|g__Campylobacter.s__Campylobacter_concisus	-	3.30E-01	0.000	0.000	0.000	0.000	0.000
4.2.1.59|g__Catabacter.s__Catabacter_hongkongensis	-	3.30E-01	0.000	0.000	0.000	0.000	0.000
4.2.1.59|g__Catenibacterium.s__Catenibacterium_mitsuokai	+	3.08E-01	0.000	0.000	0.000	0.000	0.001
4.2.1.59|g__Cetobacterium.s__Cetobacterium_somerae	-	3.30E-01	0.000	0.000	0.000	0.000	0.000
4.2.1.59|g__Chryseobacterium.s__Chryseobacterium_sp_VAUSW3	+	3.30E-01	0.000	0.000	0.000	0.000	0.000
4.2.1.59|g__Citrobacter.s__Citrobacter_amalonaticus	+	9.62E-01	0.000	0.000	0.000	0.000	0.001
4.2.1.59|g__Citrobacter.s__Citrobacter_braakii	+	6.72E-01	0.000	0.000	0.000	0.000	0.001
4.2.1.59|g__Citrobacter.s__Citrobacter_freundii	-	7.54E-01	0.000	0.000	0.000	0.000	0.001
4.2.1.59|g__Citrobacter.s__Citrobacter_portucalensis	-	7.92E-01	0.000	0.000	0.000	0.000	0.003
4.2.1.59|g__Citrobacter.s__Citrobacter_werkmanii	+	1.00E+00	0.000	0.000	0.000	0.000	0.000
4.2.1.59|g__Citrobacter.s__Citrobacter_youngae	-	3.38E-01	0.000	0.000	0.000	0.000	0.001
4.2.1.59|g__Cloacibacillus.s__Cloacibacillus_porcorum	+	3.30E-01	0.000	0.000	0.000	0.000	0.000
4.2.1.59|g__Clostridioides.s__Clostridioides_difficile	+	2.72E-01	0.000	0.000	0.000	0.000	0.000
4.2.1.59|g__Clostridium.s__Clostridium_butyricum	+	3.30E-01	0.000	0.000	0.000	0.000	0.000
4.2.1.59|g__Clostridium.s__Clostridium_celatum	+	3.30E-01	0.000	0.000	0.000	0.000	0.000
4.2.1.59|g__Clostridium.s__Clostridium_disporicum	-	6.72E-01	0.000	0.000	0.000	0.001	0.000
4.2.1.59|g__Clostridium.s__Clostridium_neonatale	+	3.30E-01	0.000	0.000	0.000	0.000	0.000
4.2.1.59|g__Clostridium.s__Clostridium_perfringens	-	8.19E-02	0.000	0.000	0.000	0.000	0.000
4.2.1.59|g__Clostridium.s__Clostridium_sp_AF36_4	+	2.97E-02	0.000	0.000	0.000	0.000	0.001
4.2.1.59|g__Clostridium.s__Clostridium_sp_D5	+	3.30E-01	0.000	0.000	0.000	0.000	0.000
4.2.1.59|g__Comamonas.s__Comamonas_aquatica	+	3.30E-01	0.000	0.000	0.000	0.000	0.000
4.2.1.59|g__Comamonas.s__Comamonas_kerstersii	-	5.69E-01	0.000	0.000	0.000	0.000	0.000
4.2.1.59|g__Comamonas.s__Comamonas_terrigena	+	3.30E-01	0.000	0.000	0.000	0.000	0.000
4.2.1.59|g__Comamonas.s__Comamonas_testosteroni	+	3.30E-01	0.000	0.000	0.000	0.000	0.000
4.2.1.59|g__Comamonas.s__Comamonas_thiooxydans	+	3.30E-01	0.000	0.000	0.000	0.000	0.000
4.2.1.59|g__Coprobacillus.s__Coprobacillus_cateniformis	+	6.48E-02	0.000	0.000	0.000	0.000	0.002
4.2.1.59|g__Coprobacter.s__Coprobacter_fastidiosus	+	3.11E-01	0.000	0.000	0.000	0.001	0.001
4.2.1.59|g__Coprobacter.s__Coprobacter_secundus	+	9.83E-01	0.000	0.000	0.000	0.000	0.000

4.2.1.59|g__Coprococcus.s__Coprococcus_catus	+	9.61E-01	0.000	0.000	0.000	0.000	0.001
4.2.1.59|g__Coprococcus.s__Coprococcus_comes	+	9.91E-01	0.000	0.000	0.000	0.001	0.001
4.2.1.59|g__Cronobacter.s__Cronobacter_dublinensis	-	3.30E-01	0.000	0.000	0.000	0.000	0.000
4.2.1.59|g__Desulfovibrio.s__Desulfovibrio_fairfieldensis	-	5.89E-01	0.000	0.000	0.000	0.000	0.000
4.2.1.59|g__Desulfovibrio.s__Desulfovibrio_piger	+	3.14E-01	0.000	0.000	0.000	0.000	0.001
4.2.1.59|g__Desulfovibrio.s__Desulfovibrio_sp_AM18_2	+	1.60E-01	0.000	0.000	0.000	0.000	0.000
4.2.1.59|g__Desulfovibrionaceae_unclassified.s__Desulfovibrionaceae_bact	+	1.60E-01	0.000	0.000	0.000	0.000	0.000
4.2.1.59|g__Dialister.s__Dialister_invisus	+	8.93E-01	0.000	0.000	0.000	0.002	0.006
4.2.1.59|g__Dialister.s__Dialister_succinatiphilus	+	9.90E-01	0.000	0.000	0.000	0.003	0.006
4.2.1.59|g__Dorea.s__Dorea_sp_OM02_2LB	-	5.07E-01	0.000	0.000	0.000	0.000	0.000
4.2.1.59|g__Eisenbergiella.s__Eisenbergiella_massiliensis	+	2.83E-01	0.000	0.000	0.000	0.000	0.000
4.2.1.59|g__Eisenbergiella.s__Eisenbergiella_tayi	+	4.60E-02	0.000	0.000	0.000	0.000	0.002
4.2.1.59|g__Enterobacter.s__Enterobacter_asburiae	+	5.77E-01	0.000	0.000	0.000	0.000	0.000
4.2.1.59|g__Enterobacter.s__Enterobacter_bugandensis	-	5.90E-01	0.000	0.000	0.000	0.000	0.001
4.2.1.59|g__Enterobacter.s__Enterobacter_cloacae	+	5.24E-01	0.000	0.000	0.000	0.001	0.001
4.2.1.59|g__Enterobacter.s__Enterobacter_mori	-	1.60E-01	0.000	0.000	0.000	0.000	0.000
4.2.1.59|g__Enterococcus.s__Enterococcus_hirae	+	1.00E+00	0.000	0.000	0.000	0.000	0.000
4.2.1.59|g__Erysipelatoclostridium.s__Clostridium_innocuum	+	1.17E-01	0.000	0.000	0.000	0.000	0.000
4.2.1.59|g__Erysipelatoclostridium.s__Clostridium_spiroforme	+	5.69E-01	0.000	0.000	0.000	0.000	0.000
4.2.1.59|g__Erysipelatoclostridium.s__Erysipelatoclostridium_ramosum	-	3.40E-01	0.000	0.000	0.000	0.000	0.000
4.2.1.59|g__Erysipelotrichaceae_unclassified.s__Erysipelotrichaceae_bacteri	-	1.60E-01	0.000	0.000	0.000	0.000	0.000
4.2.1.59|g__Eubacterium.s__Eubacterium_ramulus	+	1.08E-01	0.000	0.000	0.000	0.000	0.000
4.2.1.59|g__Eubacterium.s__Eubacterium_sp_AF17_7	+	4.43E-01	0.000	0.000	0.000	0.000	0.000
4.2.1.59|g__Eubacterium.s__Eubacterium_ventriosum	+	3.05E-01	0.000	0.000	0.000	0.001	0.001
4.2.1.59|g__Faecalicatena.s__Faecalicatena_contorta	-	1.60E-01	0.000	0.000	0.000	0.000	0.000
4.2.1.59|g__Firmicutes_unclassified.s__Firmicutes_bacterium_AF16_15	-	8.26E-01	0.000	0.000	0.000	0.002	0.001
4.2.1.59|g__Flavonifractor.s__Flavonifractor_sp_An10	+	3.30E-01	0.000	0.000	0.000	0.000	0.000
4.2.1.59|g__Fusobacterium.s__Fusobacterium_hwasookii	-	3.30E-01	0.000	0.000	0.000	0.000	0.000
4.2.1.59|g__Fusobacterium.s__Fusobacterium_mortiferum	-	6.89E-01	0.000	0.000	0.000	0.001	0.003
4.2.1.59|g__Fusobacterium.s__Fusobacterium_nucleatum	-	3.30E-01	0.000	0.000	0.000	0.000	0.000
4.2.1.59|g__Fusobacterium.s__Fusobacterium_periodonticum	-	5.89E-01	0.000	0.000	0.000	0.000	0.000
4.2.1.59|g__Fusobacterium.s__Fusobacterium_ulcerans	+	9.69E-01	0.000	0.000	0.000	0.000	0.000
4.2.1.59|g__Fusobacterium.s__Fusobacterium_varium	-	1.60E-01	0.000	0.000	0.000	0.000	0.000
4.2.1.59|g__Haemophilus.s__Haemophilus_influenzae	-	1.00E+00	0.000	0.000	0.000	0.000	0.000
4.2.1.59|g__Haemophilus.s__Haemophilus_parainfluenzae	-	4.18E-01	0.000	0.000	0.000	0.006	0.006
4.2.1.59|g__Haemophilus.s__Haemophilus_paraphrohaemolyticus	-	3.30E-01	0.000	0.000	0.000	0.001	0.000
4.2.1.59|g__Hungatella.s__Hungatella_effluvii	+	2.56E-01	0.000	0.000	0.000	0.000	0.000
4.2.1.59|g__Intestinibacter.s__Intestinibacter_bartlettii	+	3.24E-01	0.000	0.000	0.000	0.000	0.000
4.2.1.59|g__Intestinimonas.s__Intestinimonas_butyriciproducens	+	1.41E-01	0.000	0.000	0.000	0.000	0.000
4.2.1.59|g__Klebsiella.s__Klebsiella_grimontii	+	1.60E-01	0.000	0.000	0.000	0.000	0.000
4.2.1.59|g__Klebsiella.s__Klebsiella_michiganensis	-	1.00E+00	0.000	0.000	0.000	0.000	0.000
4.2.1.59|g__Kluyvera.s__Kluyvera_ascorbata	+	3.30E-01	0.000	0.000	0.000	0.000	0.000
4.2.1.59|g__Kluyvera.s__Kluyvera_cryocrescens	+	3.30E-01	0.000	0.000	0.000	0.000	0.000
4.2.1.59|g__Kluyvera.s__Kluyvera_georgiana	+	5.69E-01	0.000	0.000	0.000	0.000	0.000
4.2.1.59|g__Lachnoclostridium.s__Clostridium_aldenense	+	1.44E-01	0.000	0.000	0.000	0.000	0.000
4.2.1.59|g__Lachnoclostridium.s__Clostridium_asparagiforme	+	4.32E-02	0.000	0.000	0.000	0.000	0.000
4.2.1.59|g__Lachnoclostridium.s__Clostridium_citroniae	+	2.11E-02	0.000	0.000	0.000	0.000	0.001
4.2.1.59|g__Lachnoclostridium.s__Clostridium_clostridioforme	+	6.17E-01	0.000	0.000	0.000	0.001	0.000
4.2.1.59|g__Lachnoclostridium.s__Clostridium_lavalense	-	5.69E-01	0.000	0.000	0.000	0.000	0.000
4.2.1.59|g__Lachnoclostridium.s__Clostridium_saccharolyticum	+	3.30E-01	0.000	0.000	0.000	0.000	0.000
4.2.1.59|g__Lachnoclostridium.s__Clostridium_scindens	+	1.00E+00	0.000	0.000	0.000	0.000	0.000
4.2.1.59|g__Lachnoclostridium.s__Clostridium_symbiosum	+	6.61E-01	0.000	0.000	0.000	0.000	0.000
4.2.1.59|g__Lachnospira.s__Lachnospira_pectinoschiza	-	7.07E-01	0.000	0.000	0.000	0.007	0.004
4.2.1.59|g__Lachnospiraceae_unclassified.s__Lachnospiraceae_bacterium_2	-	1.60E-01	0.000	0.000	0.000	0.000	0.000
4.2.1.59|g__Lachnospiraceae_unclassified.s__Lachnospiraceae_bacterium_(	+	3.30E-01	0.000	0.000	0.000	0.000	0.000
4.2.1.59|g__Lachnospiraceae_unclassified.s__Lachnospiraceae_bacterium_(	-	3.30E-01	0.000	0.000	0.000	0.000	0.000
4.2.1.59|g__Lachnospiraceae_unclassified.s__Lachnospiraceae_bacterium_c	-	3.30E-01	0.000	0.000	0.000	0.000	0.000
4.2.1.59|g__Lactobacillus.s__Lactobacillus_fermentum	-	7.02E-01	0.000	0.000	0.000	0.000	0.000
4.2.1.59|g__Lactobacillus.s__Lactobacillus_mucosae	+	4.27E-02	0.000	0.000	0.000	0.000	0.000
4.2.1.59|g__Lactobacillus.s__Lactobacillus_paraplantarum	-	1.00E+00	0.000	0.000	0.000	0.000	0.000
4.2.1.59|g__Lactobacillus.s__Lactobacillus_plantarum	+	1.00E+00	0.000	0.000	0.000	0.000	0.000
4.2.1.59|g__Lactobacillus.s__Lactobacillus_reuteri	-	3.30E-01	0.000	0.000	0.000	0.000	0.000
4.2.1.59|g__Lactobacillus.s__Lactobacillus_rogosae	-	7.16E-01	0.000	0.000	0.000	0.006	0.005
4.2.1.59|g__Lactobacillus.s__Lactobacillus_ruminis	-	1.00E+00	0.000	0.000	0.000	0.000	0.000
4.2.1.59|g__Lactobacillus.s__Lactobacillus_salivarius	+	4.38E-02	0.000	0.000	0.000	0.000	0.001
4.2.1.59|g__Lactobacillus.s__Lactobacillus_sanfranciscensis	-	5.49E-01	0.000	0.000	0.000	0.000	0.000
4.2.1.59|g__Lactococcus.s__Lactococcus_garvieae	+	3.30E-01	0.000	0.000	0.000	0.000	0.000
4.2.1.59|g__Lactococcus.s__Lactococcus_petauri	+	3.30E-01	0.000	0.000	0.000	0.000	0.000
4.2.1.59|g__Leclercia.s__Leclercia_adecarboxylata	-	3.60E-01	0.000	0.000	0.000	0.000	0.000
4.2.1.59|g__Lelliottia.s__Lelliottia_amnigena	-	3.08E-01	0.000	0.000	0.000	0.000	0.000
4.2.1.59|g__Lelliottia.s__Lelliottia_nimipressuralis	-	6.61E-01	0.000	0.000	0.000	0.002	0.004
4.2.1.59|g__Leuconostoc.s__Leuconostoc_mesenteroides	+	3.30E-01	0.000	0.000	0.000	0.000	0.000

4.2.1.59|g__Megamonas.s__Megamonas_funiformis	-	8.72E-01	0.000	0.000	0.000	0.017	0.007
4.2.1.59|g__Megamonas.s__Megamonas_hypermegale	-	1.00E+00	0.000	0.000	0.000	0.008	0.006
4.2.1.59|g__Megamonas.s__Megamonas_rupellensis	-	9.36E-01	0.000	0.000	0.000	0.016	0.010
4.2.1.59|g__Megasphaera.s__Megasphaera_elsdenii	+	3.30E-01	0.000	0.000	0.000	0.000	0.000
4.2.1.59|g__Megasphaera.s__Megasphaera_micronuciformis	+	3.08E-01	0.000	0.000	0.000	0.000	0.000
4.2.1.59|g__Megasphaera.s__Megasphaera_stantonii	+	3.30E-01	0.000	0.000	0.000	0.000	0.000
4.2.1.59|g__Mesosutterella.s__Mesosutterella_multiformis	-	7.12E-01	0.000	0.000	0.000	0.002	0.001
4.2.1.59|g__Mitsuokella.s__Mitsuokella_jalaludinii	+	3.30E-01	0.000	0.000	0.000	0.000	0.000
4.2.1.59|g__Mitsuokella.s__Mitsuokella_multacida	+	8.19E-02	0.000	0.000	0.000	0.000	0.001
4.2.1.59|g__Monoglobus.s__Monoglobus_pectinilyticus	+	8.19E-02	0.000	0.000	0.000	0.000	0.000
4.2.1.59|g__Morganella.s__Morganella_morganii	+	1.00E+00	0.000	0.000	0.000	0.000	0.000
4.2.1.59|g__Murimonas.s__Murimonas_intestini	+	3.30E-01	0.000	0.000	0.000	0.000	0.000
4.2.1.59|g__Ochrobactrum.s__Ochrobactrum_cytisi	+	3.30E-01	0.000	0.000	0.000	0.000	0.000
4.2.1.59|g__Ochrobactrum.s__Ochrobactrum_lupini	+	3.30E-01	0.000	0.000	0.000	0.000	0.000
4.2.1.59|g__Oribacterium.s__Oribacterium_sinus	-	3.30E-01	0.000	0.000	0.000	0.000	0.000
4.2.1.59|g__Oxalobacter.s__Oxalobacter_formigenes	+	1.50E-02	0.000	0.000	0.000	0.000	0.000
4.2.1.59|g__Pantoea.s__Pantoea_agglomerans	-	3.30E-01	0.000	0.000	0.000	0.000	0.000
4.2.1.59|g__Pantoea.s__Pantoea_dispersa	+	3.30E-01	0.000	0.000	0.000	0.000	0.000
4.2.1.59|g__Pantoea.s__Pantoea_sesami	-	8.50E-01	0.000	0.000	0.000	0.000	0.000
4.2.1.59|g__Parabacteroides.s__Parabacteroides_chinchillae	+	3.30E-01	0.000	0.000	0.000	0.000	0.000
4.2.1.59|g__Parabacteroides.s__Parabacteroides_goldsteinii	+	4.46E-01	0.000	0.000	0.000	0.000	0.001
4.2.1.59|g__Parabacteroides.s__Parabacteroides_gordonii	+	1.60E-01	0.000	0.000	0.000	0.000	0.000
4.2.1.59|g__Parabacteroides.s__Parabacteroides_johnsonii	+	6.92E-01	0.000	0.000	0.000	0.001	0.001
4.2.1.59|g__Paraprevotella.s__Paraprevotella_clara	+	9.58E-01	0.000	0.000	0.000	0.002	0.001
4.2.1.59|g__Paraprevotella.s__Paraprevotella_xylaniphila	-	3.30E-01	0.000	0.000	0.000	0.000	0.000
4.2.1.59|g__Parasutterella.s__Parasutterella_excrementihominis	+	6.75E-01	0.000	0.000	0.000	0.003	0.003
4.2.1.59|g__Pedobacter.s__Pedobacter_himalayensis	-	5.06E-01	0.000	0.000	0.000	0.000	0.000
4.2.1.59|g__Phascolarctobacterium.s__Phascolarctobacterium_succinatutens	+	9.92E-01	0.000	0.000	0.000	0.006	0.008
4.2.1.59|g__Prevotella.s__Prevotella_corporis	+	1.60E-01	0.000	0.000	0.000	0.000	0.000
4.2.1.59|g__Prevotella.s__Prevotella_sp_109	-	1.00E+00	0.000	0.000	0.000	0.002	0.003
4.2.1.59|g__Proteus.s__Proteus_mirabilis	-	3.30E-01	0.000	0.000	0.000	0.000	0.000
4.2.1.59|g__Proteus.s__Proteus_penneri	+	1.00E+00	0.000	0.000	0.000	0.000	0.000
4.2.1.59|g__Proteus.s__Proteus_vulgaris	+	1.00E+00	0.000	0.000	0.000	0.000	0.000
4.2.1.59|g__Providencia.s__Providencia_alcalifaciens	+	3.30E-01	0.000	0.000	0.000	0.000	0.000
4.2.1.59|g__Providencia.s__Providencia_rettgeri	+	3.30E-01	0.000	0.000	0.000	0.000	0.006
4.2.1.59|g__Pseudoflavonifractor.s__Pseudoflavonifractor_capillosus	+	3.30E-01	0.000	0.000	0.000	0.000	0.000
4.2.1.59|g__Pseudoflavonifractor.s__Pseudoflavonifractor_sp_An184	+	1.60E-01	0.000	0.000	0.000	0.000	0.000
4.2.1.59|g__Pyramidobacter.s__Pyramidobacter_piscolens	+	2.24E-02	0.000	0.000	0.000	0.000	0.000
4.2.1.59|g__Pyramidobacter.s__Pyramidobacter_sp_C12_8	+	3.30E-01	0.000	0.000	0.000	0.000	0.000
4.2.1.59|g__Raoultella.s__Raoultella_ornithinolytica	+	5.89E-01	0.000	0.000	0.000	0.000	0.000
4.2.1.59|g__Raoultella.s__Raoultella_planticola	+	5.49E-01	0.000	0.000	0.000	0.000	0.000
4.2.1.59|g__Roseburia.s__Roseburia_hominis	-	3.69E-01	0.000	0.000	0.000	0.002	0.002
4.2.1.59|g__Ruminococcaceae_unclassified.s__Ruminococcaceae_bacteriun	+	5.89E-01	0.000	0.000	0.000	0.000	0.000
4.2.1.59|g__Ruminococcus.s__Ruminococcus_lactaris	+	2.49E-01	0.000	0.000	0.000	0.001	0.001
4.2.1.59|g__Ruminococcus.s__Ruminococcus_sp_AF31_8BH	-	1.28E-01	0.000	0.000	0.000	0.000	0.000
4.2.1.59|g__Sellimonas.s__Sellimonas_intestinalis	+	5.89E-01	0.000	0.000	0.000	0.000	0.000
4.2.1.59|g__Staphylococcus.s__Staphylococcus_warneri	-	3.30E-01	0.000	0.000	0.000	0.000	0.000
4.2.1.59|g__Streptococcus.s__Streptococcus_australis	-	4.14E-01	0.000	0.000	0.000	0.000	0.000
4.2.1.59|g__Streptococcus.s__Streptococcus_cristatus	+	2.42E-01	0.000	0.000	0.000	0.000	0.000
4.2.1.59|g__Streptococcus.s__Streptococcus_equinus	-	5.69E-01	0.000	0.000	0.000	0.000	0.000
4.2.1.59|g__Streptococcus.s__Streptococcus_gallolyticus	-	5.89E-01	0.000	0.000	0.000	0.000	0.000
4.2.1.59|g__Streptococcus.s__Streptococcus_gordonii	+	1.36E-01	0.000	0.000	0.000	0.000	0.000
4.2.1.59|g__Streptococcus.s__Streptococcus_infantarius	-	1.00E+00	0.000	0.000	0.000	0.000	0.000
4.2.1.59|g__Streptococcus.s__Streptococcus_infantis	+	3.30E-01	0.000	0.000	0.000	0.000	0.000
4.2.1.59|g__Streptococcus.s__Streptococcus_lutetiensis	-	3.30E-01	0.000	0.000	0.000	0.000	0.000
4.2.1.59|g__Streptococcus.s__Streptococcus_macedonicus	-	5.89E-01	0.000	0.000	0.000	0.000	0.000
4.2.1.59|g__Streptococcus.s__Streptococcus_mitis	-	5.53E-01	0.000	0.000	0.000	0.000	0.000
4.2.1.59|g__Streptococcus.s__Streptococcus_oralis	+	2.49E-01	0.000	0.000	0.000	0.000	0.000
4.2.1.59|g__Streptococcus.s__Streptococcus_parasanguinis	+	6.19E-01	0.000	0.000	0.000	0.001	0.002
4.2.1.59|g__Streptococcus.s__Streptococcus_pasteurianus	+	1.00E+00	0.000	0.000	0.000	0.000	0.000
4.2.1.59|g__Streptococcus.s__Streptococcus_pneumoniae	-	3.26E-01	0.000	0.000	0.000	0.000	0.000
4.2.1.59|g__Streptococcus.s__Streptococcus_pseudopneumoniae	-	4.65E-01	0.000	0.000	0.000	0.000	0.000
4.2.1.59|g__Streptococcus.s__Streptococcus_sanguinis	-	3.30E-01	0.000	0.000	0.000	0.000	0.000
4.2.1.59|g__Streptococcus.s__Streptococcus_sobrinus	-	3.30E-01	0.000	0.000	0.000	0.000	0.000
4.2.1.59|g__Streptococcus.s__Streptococcus_sp_A12	-	1.58E-01	0.000	0.000	0.000	0.000	0.000
4.2.1.59|g__Streptococcus.s__Streptococcus_thermophilus	-	6.87E-01	0.000	0.000	0.000	0.000	0.000
4.2.1.59|g__Streptococcus.s__Streptococcus_vestibularis	+	1.47E-01	0.000	0.000	0.000	0.000	0.000
4.2.1.59|g__Streptococcus.s__Streptococcus_viridans	+	2.24E-02	0.000	0.000	0.000	0.000	0.000
4.2.1.59|g__Sutterella.s__Sutterella_parvirubra	+	3.30E-01	0.000	0.000	0.000	0.000	0.000
4.2.1.59|g__Sutterella.s__Sutterella_wadsworthensis	+	2.89E-01	0.000	0.000	0.000	0.003	0.003
4.2.1.59|g__Turicibacter.s__Turicibacter_sanguinis	-	2.96E-01	0.000	0.000	0.000	0.000	0.000
4.2.1.59|g__Tyzzerella.s__Tyzzerella_nexilis	-	6.20E-01	0.000	0.000	0.000	0.001	0.001

	4.2.1.59|g__Veillonella.s__Veillonella_atypica	-	8.16E-01	0.000	0.000	0.000	0.001	0.001
	4.2.1.59|g__Veillonella.s__Veillonella_dispar	-	4.53E-01	0.000	0.000	0.000	0.001	0.001
	4.2.1.59|g__Veillonella.s__Veillonella_infantium	-	8.63E-01	0.000	0.000	0.000	0.000	0.000
	4.2.1.59|g__Veillonella.s__Veillonella_rogosae	-	2.22E-02	0.000	0.000	0.000	0.001	0.001
	4.2.1.59|g__Veillonella.s__Veillonella_tobetsuensis	-	1.25E-01	0.000	0.000	0.000	0.000	0.001
	4.2.1.59|g__Weissella.s__Weissella_cibaria	-	3.30E-01	0.000	0.000	0.000	0.000	0.000
	4.2.1.59|g__Weissella.s__Weissella_confusa	-	1.00E+00	0.000	0.000	0.000	0.000	0.000
	4.2.1.59|g__Yokenella.s__Yokenella_regensburgei	-	7.23E-01	0.000	0.000	0.000	0.000	0.000
	4.2.1.59|g__Bacteroides.s__Bacteroides_cellulosilyticus	+	4.18E-01	0.000	0.010	0.010	0.003	0.005
	4.2.1.59|g__Klebsiella.s__Klebsiella_aerogenes	+	4.04E-01	0.024	0.039	0.015	0.002	0.005
	4.2.1.59|g__Klebsiella.s__Klebsiella_oxytoca	+	4.51E-01	0.023	0.040	0.017	0.001	0.003
	4.2.1.59|g__Hungatella.s__Hungatella_hathewayi	+	1.05E-01	0.000	0.019	0.019	0.000	0.002
	4.2.1.59|g__Clostridiales_unclassified.s__Clostridiales_bacterium_KLE1615	-	9.27E-01	0.000	0.035	0.035	0.002	0.001
	4.2.1.59|g__Salmonella.s__Salmonella_enterica	+	3.50E-01	0.031	0.069	0.038	0.001	0.001
	4.2.1.59|g__Anaerostipes.s__Anaerostipes_hadrus	-	7.98E-01	0.171	0.219	0.047	0.004	0.005
	4.2.1.59|g__Lachnoclostridium.s__Clostridium_bolteae	+	2.06E-01	0.000	0.049	0.049	0.001	0.001
	4.2.1.59|g__Escherichia.s__Escherichia_fergusonii	+	4.87E-01	0.024	0.073	0.049	0.002	0.002
	4.2.1.59|g__Flavonifractor.s__Flavonifractor_plautii	+	6.95E-01	0.148	0.200	0.052	0.003	0.003
	4.2.1.59|g__Alistipes.s__Alistipes_onderdonkii	+	9.05E-02	0.016	0.068	0.052	0.001	0.003
	4.2.1.59|g__Klebsiella.s__Klebsiella_pneumoniae	+	1.72E-01	0.084	0.152	0.068	0.010	0.032
	4.2.1.59|g__Lawsonibacter.s__Lawsonibacter_asaccharolyticus	+	3.75E-03	0.000	0.071	0.071	0.000	0.001
	4.2.1.59|g__Blautia.s__Blautia_obeum	+	5.42E-01	0.325	0.407	0.082	0.006	0.009
	4.2.1.59|g__Klebsiella.s__Klebsiella_variicola	+	5.94E-02	0.000	0.112	0.112	0.005	0.005
	4.2.1.59|g__Bacteroides.s__Bacteroides_intestinalis	+	4.22E-01	0.030	0.142	0.112	0.004	0.004
	4.2.1.59|g__Bacteroides.s__Bacteroides_ovatus	-	9.68E-01	0.214	0.339	0.124	0.017	0.013
	4.2.1.59|g__Parabacteroides.s__Parabacteroides_merdae	+	1.92E-01	0.304	0.511	0.207	0.005	0.010
	4.2.1.59|g__Odoribacter.s__Odoribacter_splanchnicus	+	2.41E-01	0.164	0.396	0.231	0.003	0.004
	4.2.1.59|g__Bilophila.s__Bilophila_wadsworthia	+	4.17E-03	0.088	0.350	0.262	0.001	0.004
	4.2.1.59|g__Eubacterium.s__Eubacterium_eligens	+	8.21E-01	0.000	0.302	0.302	0.009	0.007
	4.2.1.59|g__Bacteroides.s__Bacteroides_caccae	+	4.20E-01	0.116	0.493	0.376	0.007	0.009
	4.2.1.59|g__Blautia.s__Ruminococcus_torques	+	2.42E-01	0.740	1.130	0.390	0.011	0.013
	4.2.1.59|g__Alistipes.s__Alistipes_shahii	+	5.56E-02	0.104	0.509	0.405	0.005	0.010
	4.2.1.59|g__Bacteroides.s__Bacteroides_stercoris	+	5.18E-01	0.307	0.826	0.519	0.037	0.037
	4.2.1.59|g__Alistipes.s__Alistipes_finegoldii	+	1.11E-01	0.064	0.586	0.523	0.005	0.011
	4.2.1.59|g__Bacteroides.s__Bacteroides_uniformis	+	5.55E-01	2.044	3.211	1.167	0.033	0.045
	4.2.1.59|unclassified	+	7.19E-01	15.002	16.654	1.652	0.127	0.137
	4.2.1.59|g__Escherichia.s__Escherichia_coli	+	2.59E-01	1.240	3.045	1.805	0.071	0.103
	4.2.1.59|g__Alistipes.s__Alistipes_putredinis	+	1.63E-03	0.124	3.814	3.690	0.013	0.035
Taiwan	4.2.1.59	-	6.74E-02	131.539	116.404	-15.136	1.000	1.000
	4.2.1.59|g__Bacteroides.s__Bacteroides_vulgatus	-	5.18E-01	7.219	5.204	-2.015	0.081	0.070
	4.2.1.59|g__Bacteroides.s__Bacteroides_uniformis	+	7.95E-01	3.665	2.692	-0.973	0.040	0.046
	4.2.1.59|g__Escherichia.s__Escherichia_coli	-	1.72E-01	0.731	0.253	-0.478	0.050	0.016
	4.2.1.59|g__Bacteroides.s__Bacteroides_plebeius	-	4.73E-01	0.792	0.325	-0.466	0.052	0.040
	4.2.1.59|g__Lachnospiraceae_unclassified.s__Eubacterium_rectale	-	1.56E-02	0.546	0.091	-0.455	0.013	0.008
	4.2.1.59|g__Roseburia.s__Roseburia_inulinivorans	-	9.04E-03	0.534	0.201	-0.332	0.014	0.006
	4.2.1.59|g__Fusicatenibacter.s__Fusicatenibacter_saccharivorans	-	9.12E-02	0.441	0.154	-0.287	0.004	0.004
	4.2.1.59|g__Clostridium.s__Clostridium_sp_AM22_11AC	-	6.90E-02	0.452	0.258	-0.195	0.009	0.006
	4.2.1.59|g__Coprococcus.s__Coprococcus_comes	-	1.07E-01	0.161	0.000	-0.161	0.004	0.002
	4.2.1.59|g__Roseburia.s__Roseburia_faecis	-	5.43E-01	0.398	0.245	-0.153	0.006	0.008
	4.2.1.59|g__Flavonifractor.s__Flavonifractor_plautii	-	8.01E-01	0.307	0.217	-0.090	0.007	0.006
	4.2.1.59|g__Bacteroides.s__Bacteroides_massiliensis	-	9.95E-01	0.388	0.326	-0.061	0.020	0.021
	4.2.1.59|g__Bacteroides.s__Bacteroides_xylanisolvens	+	8.61E-01	0.595	0.534	-0.061	0.014	0.016
	4.2.1.59|g__Blautia.s__Blautia_wexlerae	-	5.12E-01	0.032	0.000	-0.032	0.001	0.001
	4.2.1.59|g__Blautia.s__Blautia_obeum	-	8.25E-01	0.156	0.138	-0.018	0.002	0.002
	4.2.1.59|g__Bilophila.s__Bilophila_wadsworthia	-	7.16E-01	0.262	0.244	-0.018	0.004	0.004
	4.2.1.59|g__Bacteroides.s__Bacteroides_dorei	+	4.04E-01	0.775	0.758	-0.018	0.020	0.028
	4.2.1.59|g__Acidaminococcus.s__Acidaminococcus_fermentans	+	1.10E-01	0.000	0.000	0.000	0.000	0.000
	4.2.1.59|g__Acidaminococcus.s__Acidaminococcus_intestini	+	8.89E-01	0.000	0.000	0.000	0.001	0.002
	4.2.1.59|g__Adlercreutzia.s__Adlercreutzia_equolifaciens	-	5.88E-02	0.000	0.000	0.000	0.000	0.000
	4.2.1.59|g__Aeromonas.s__Aeromonas_caviae	-	2.85E-01	0.000	0.000	0.000	0.000	0.000
	4.2.1.59|g__Aggregatibacter.s__Aggregatibacter_segnis	-	2.85E-01	0.000	0.000	0.000	0.000	0.000
	4.2.1.59|g__Akkermansia.s__Akkermansia_muciniphila	+	1.48E-01	0.000	0.000	0.000	0.007	0.011
	4.2.1.59|g__Alistipes.s__Alistipes_indistinctus	+	9.91E-01	0.000	0.000	0.000	0.002	0.004
	4.2.1.59|g__Alistipes.s__Alistipes_inops	+	9.13E-01	0.000	0.000	0.000	0.002	0.001
	4.2.1.59|g__Alistipes.s__Alistipes_timonensis	-	2.85E-01	0.000	0.000	0.000	0.000	0.000
	4.2.1.59|g__Allisonella.s__Allisonella_histaminiformans	+	8.85E-01	0.000	0.000	0.000	0.000	0.000
	4.2.1.59|g__Anaerostipes.s__Anaerostipes_hadrus	+	6.68E-01	0.000	0.000	0.000	0.002	0.002
	4.2.1.59|g__Anaerotruncus.s__Anaerotruncus_colihominis	-	2.44E-01	0.000	0.000	0.000	0.000	0.000
	4.2.1.59|g__Asaccharobacter.s__Asaccharobacter_celatus	-	2.85E-01	0.000	0.000	0.000	0.000	0.000
	4.2.1.59|g__Bacteroides.s__Bacteroides_clarus	-	7.52E-01	0.000	0.000	0.000	0.002	0.007
	4.2.1.59|g__Bacteroides.s__Bacteroides_coprocola	+	8.67E-01	0.000	0.000	0.000	0.026	0.024
	4.2.1.59|g__Bacteroides.s__Bacteroides_coprophilus	-	7.13E-01	0.000	0.000	0.000	0.008	0.005

4.2.1.59|g__Bacteroides.s__Bacteroides_eggerthii	+	3.87E-01	0.000	0.000	0.000	0.005	0.007
4.2.1.59|g__Bacteroides.s__Bacteroides_faecichinchillae	+	3.50E-01	0.000	0.000	0.000	0.000	0.000
4.2.1.59|g__Bacteroides.s__Bacteroides_fluxus	-	9.26E-01	0.000	0.000	0.000	0.000	0.002
4.2.1.59|g__Bacteroides.s__Bacteroides_fragilis	+	6.21E-01	0.000	0.000	0.000	0.007	0.007
4.2.1.59|g__Bacteroides.s__Bacteroides_nordii	+	3.84E-01	0.000	0.000	0.000	0.000	0.001
4.2.1.59|g__Bacteroides.s__Bacteroides_oleiciplenus	+	3.68E-01	0.000	0.000	0.000	0.000	0.000
4.2.1.59|g__Bacteroides.s__Bacteroides_salyersiae	-	8.92E-01	0.000	0.000	0.000	0.005	0.003
4.2.1.59|g__Bacteroides.s__Bacteroides_sartorii	-	8.72E-01	0.000	0.000	0.000	0.004	0.001
4.2.1.59|g__Bacteroides.s__Bacteroides_sp_OM08_11	+	5.46E-01	0.000	0.000	0.000	0.000	0.000
4.2.1.59|g__Barnesiella.s__Barnesiella_intestinihominis	+	4.25E-02	0.000	0.000	0.000	0.004	0.008
4.2.1.59|g__Bifidobacterium.s__Bifidobacterium_longum	+	7.08E-01	0.000	0.000	0.000	0.001	0.001
4.2.1.59|g__Blautia.s__Blautia_sp_AF19_10LB	-	4.98E-01	0.000	0.000	0.000	0.000	0.000
4.2.1.59|g__Blautia.s__Ruminococcus_gnavus	+	7.27E-01	0.000	0.000	0.000	0.009	0.009
4.2.1.59|g__Candidatus_Gastranaerophilales_unclassified.s__Candidatus_Ga	-	9.26E-01	0.000	0.000	0.000	0.000	0.000
4.2.1.59|g__Catabacter.s__Catabacter_hongkongensis	-	2.85E-01	0.000	0.000	0.000	0.000	0.000
4.2.1.59|g__Catenibacterium.s__Catenibacterium_mitsuokai	-	5.72E-01	0.000	0.000	0.000	0.007	0.008
4.2.1.59|g__Cetobacterium.s__Cetobacterium_somerae	+	3.68E-01	0.000	0.000	0.000	0.000	0.000
4.2.1.59|g__Citrobacter.s__Citrobacter_amalonaticus	-	5.86E-02	0.000	0.000	0.000	0.000	0.000
4.2.1.59|g__Citrobacter.s__Citrobacter_braakii	-	1.25E-01	0.000	0.000	0.000	0.000	0.000
4.2.1.59|g__Citrobacter.s__Citrobacter_farmeri	-	2.85E-01	0.000	0.000	0.000	0.000	0.000
4.2.1.59|g__Citrobacter.s__Citrobacter_freundii	+	8.42E-01	0.000	0.000	0.000	0.000	0.000
4.2.1.59|g__Citrobacter.s__Citrobacter_koseri	+	6.86E-01	0.000	0.000	0.000	0.000	0.000
4.2.1.59|g__Citrobacter.s__Citrobacter_portucalensis	+	8.86E-01	0.000	0.000	0.000	0.000	0.000
4.2.1.59|g__Citrobacter.s__Citrobacter_werkmanii	+	3.68E-01	0.000	0.000	0.000	0.000	0.000
4.2.1.59|g__Citrobacter.s__Citrobacter_youngae	+	8.32E-01	0.000	0.000	0.000	0.000	0.000
4.2.1.59|g__Cloacibacillus.s__Cloacibacillus_porcorum	+	2.16E-02	0.000	0.000	0.000	0.000	0.000
4.2.1.59|g__Clostridiales_unclassified.s__Clostridiales_bacterium_1_7_47FA	-	4.71E-01	0.000	0.000	0.000	0.000	0.000
4.2.1.59|g__Clostridiales_unclassified.s__Clostridiales_bacterium_KLE1615	-	8.27E-01	0.000	0.000	0.000	0.002	0.002
4.2.1.59|g__Clostridioides.s__Clostridioides_difficile	-	8.69E-01	0.000	0.000	0.000	0.000	0.000
4.2.1.59|g__Clostridium.s__Butyribacterium_methylotrophicum	+	3.68E-01	0.000	0.000	0.000	0.000	0.000
4.2.1.59|g__Clostridium.s__Clostridium_disporicum	+	6.54E-01	0.000	0.000	0.000	0.000	0.000
4.2.1.59|g__Clostridium.s__Clostridium_neonatale	+	3.68E-01	0.000	0.000	0.000	0.000	0.000
4.2.1.59|g__Clostridium.s__Clostridium_paraputrificum	-	2.85E-01	0.000	0.000	0.000	0.000	0.000
4.2.1.59|g__Clostridium.s__Clostridium_perfringens	-	1.25E-01	0.000	0.000	0.000	0.000	0.000
4.2.1.59|g__Clostridium.s__Clostridium_sp_AF36_4	+	9.19E-01	0.000	0.000	0.000	0.001	0.000
4.2.1.59|g__Clostridium.s__Clostridium_sp_D5	+	6.86E-01	0.000	0.000	0.000	0.000	0.000
4.2.1.59|g__Comamonas.s__Comamonas_kerstersii	-	9.26E-01	0.000	0.000	0.000	0.000	0.000
4.2.1.59|g__Coprobacillus.s__Coprobacillus_cateniformis	+	3.68E-01	0.000	0.000	0.000	0.000	0.000
4.2.1.59|g__Coprobacter.s__Coprobacter_fastidiosus	+	4.84E-01	0.000	0.000	0.000	0.001	0.003
4.2.1.59|g__Coprobacter.s__Coprobacter_secundus	+	5.93E-01	0.000	0.000	0.000	0.000	0.000
4.2.1.59|g__Coprococcus.s__Coprococcus_catus	+	8.33E-01	0.000	0.000	0.000	0.000	0.000
4.2.1.59|g__Desulfovibrio.s__Desulfovibrio_desulfuricans	+	3.68E-01	0.000	0.000	0.000	0.000	0.000
4.2.1.59|g__Desulfovibrio.s__Desulfovibrio_fairfieldensis	+	3.87E-01	0.000	0.000	0.000	0.000	0.000
4.2.1.59|g__Desulfovibrio.s__Desulfovibrio_legallii	+	3.68E-01	0.000	0.000	0.000	0.000	0.000
4.2.1.59|g__Desulfovibrio.s__Desulfovibrio_piger	+	8.94E-01	0.000	0.000	0.000	0.001	0.001
4.2.1.59|g__Desulfovibrio.s__Desulfovibrio_sp_AM18_2	+	1.96E-01	0.000	0.000	0.000	0.000	0.000
4.2.1.59|g__Desulfovibrionaceae_unclassified.s__Desulfovibrionaceae_bacte	-	2.97E-01	0.000	0.000	0.000	0.000	0.000
4.2.1.59|g__Dialister.s__Dialister_invisus	-	4.71E-01	0.000	0.000	0.000	0.001	0.000
4.2.1.59|g__Dialister.s__Dialister_succinatiphilus	-	3.76E-01	0.000	0.000	0.000	0.006	0.002
4.2.1.59|g__Dorea.s__Dorea_formicigenerans	+	4.99E-01	0.000	0.000	0.000	0.000	0.001
4.2.1.59|g__Dorea.s__Dorea_longicatena	-	9.36E-01	0.000	0.000	0.000	0.001	0.002
4.2.1.59|g__Dorea.s__Dorea_sp_OM02_2LB	+	3.68E-01	0.000	0.000	0.000	0.000	0.000
4.2.1.59|g__Eisenbergiella.s__Eisenbergiella_massiliensis	+	7.98E-01	0.000	0.000	0.000	0.000	0.000
4.2.1.59|g__Eisenbergiella.s__Eisenbergiella_tayi	+	4.19E-01	0.000	0.000	0.000	0.001	0.000
4.2.1.59|g__Enterobacter.s__Enterobacter_asburiae	-	1.67E-01	0.000	0.000	0.000	0.000	0.000
4.2.1.59|g__Enterobacter.s__Enterobacter_bugandensis	-	5.21E-01	0.000	0.000	0.000	0.000	0.000
4.2.1.59|g__Enterobacter.s__Enterobacter_cloacae	-	4.69E-01	0.000	0.000	0.000	0.000	0.000
4.2.1.59|g__Enterococcus.s__Enterococcus_faecium	+	4.08E-01	0.000	0.000	0.000	0.000	0.000
4.2.1.59|g__Erysipelatoclostridium.s__Clostridium_innocuum	-	9.02E-01	0.000	0.000	0.000	0.000	0.000
4.2.1.59|g__Erysipelatoclostridium.s__Clostridium_spiroforme	-	1.25E-01	0.000	0.000	0.000	0.000	0.000
4.2.1.59|g__Erysipelatoclostridium.s__Erysipelatoclostridium_ramosum	+	8.44E-01	0.000	0.000	0.000	0.000	0.000
4.2.1.59|g__Erysipelotrichaceae_unclassified.s__Erysipelotrichaceae_bacteri	-	9.26E-01	0.000	0.000	0.000	0.000	0.000
4.2.1.59|g__Escherichia.s__Escherichia_fergusonii	-	8.54E-01	0.000	0.000	0.000	0.000	0.000
4.2.1.59|g__Eubacterium.s__Eubacterium_callanderi	-	2.85E-01	0.000	0.000	0.000	0.000	0.000
4.2.1.59|g__Eubacterium.s__Eubacterium_limosum	+	3.68E-01	0.000	0.000	0.000	0.000	0.000
4.2.1.59|g__Eubacterium.s__Eubacterium_ramulus	-	7.02E-01	0.000	0.000	0.000	0.000	0.000
4.2.1.59|g__Eubacterium.s__Eubacterium_sp_AF17_7	+	2.38E-01	0.000	0.000	0.000	0.000	0.001
4.2.1.59|g__Eubacterium.s__Eubacterium_ventriosum	+	1.52E-02	0.000	0.000	0.000	0.000	0.002
4.2.1.59|g__Faecalicatena.s__Faecalicatena_contorta	+	3.50E-01	0.000	0.000	0.000	0.000	0.000
4.2.1.59|g__Faecalicatena.s__Faecalicatena_orotica	+	3.68E-01	0.000	0.000	0.000	0.000	0.000
4.2.1.59|g__Faecalicoccus.s__Faecalicoccus_pleomorphus	+	3.68E-01	0.000	0.000	0.000	0.000	0.000
4.2.1.59|g__Firmicutes_unclassified.s__Firmicutes_bacterium_AF16_15	+	6.11E-01	0.000	0.000	0.000	0.001	0.001

4.2.1.59|g__Flavonifractor.s__Flavonifractor_sp_An10	+	3.68E-01	0.000	0.000	0.000	0.000	0.000
4.2.1.59|g__Fusobacterium.s__Fusobacterium_mortiferum	-	4.57E-01	0.000	0.000	0.000	0.004	0.001
4.2.1.59|g__Fusobacterium.s__Fusobacterium_nucleatum	+	3.68E-01	0.000	0.000	0.000	0.000	0.000
4.2.1.59|g__Fusobacterium.s__Fusobacterium_ulcerans	+	2.28E-01	0.000	0.000	0.000	0.000	0.002
4.2.1.59|g__Fusobacterium.s__Fusobacterium_varium	-	4.57E-01	0.000	0.000	0.000	0.001	0.000
4.2.1.59|g__Haemophilus.s__Haemophilus_parainfluenzae	-	8.66E-02	0.000	0.000	0.000	0.006	0.002
4.2.1.59|g__Hafnia.s__Hafnia_paralvei	-	2.85E-01	0.000	0.000	0.000	0.000	0.000
4.2.1.59|g__Hungatella.s__Hungatella_effluvii	+	2.10E-01	0.000	0.000	0.000	0.000	0.000
4.2.1.59|g__Hungatella.s__Hungatella_hathewayi	+	5.70E-01	0.000	0.000	0.000	0.001	0.003
4.2.1.59|g__Intestinibacter.s__Intestinibacter_bartlettii	+	3.68E-01	0.000	0.000	0.000	0.000	0.000
4.2.1.59|g__Intestinimonas.s__Intestinimonas_butyriciproducens	+	7.85E-02	0.000	0.000	0.000	0.001	0.002
4.2.1.59|g__Klebsiella.s__Klebsiella_aerogenes	-	2.31E-01	0.000	0.000	0.000	0.000	0.000
4.2.1.59|g__Klebsiella.s__Klebsiella_grimontii	-	9.26E-01	0.000	0.000	0.000	0.000	0.000
4.2.1.59|g__Klebsiella.s__Klebsiella_michiganensis	-	9.05E-01	0.000	0.000	0.000	0.000	0.000
4.2.1.59|g__Klebsiella.s__Klebsiella_oxytoca	-	4.44E-01	0.000	0.000	0.000	0.002	0.001
4.2.1.59|g__Klebsiella.s__Klebsiella_pneumoniae	-	4.60E-01	0.000	0.000	0.000	0.009	0.005
4.2.1.59|g__Klebsiella.s__Klebsiella_variicola	+	9.94E-01	0.000	0.000	0.000	0.001	0.001
4.2.1.59|g__Kluyvera.s__Kluyvera_ascorbata	-	2.85E-01	0.000	0.000	0.000	0.000	0.000
4.2.1.59|g__Kluyvera.s__Kluyvera_georgiana	-	2.85E-01	0.000	0.000	0.000	0.001	0.000
4.2.1.59|g__Kluyvera.s__Kluyvera_intermedia	+	3.68E-01	0.000	0.000	0.000	0.000	0.000
4.2.1.59|g__Lachnoclostridium.s__Clostridium_aldenense	+	1.83E-01	0.000	0.000	0.000	0.000	0.001
4.2.1.59|g__Lachnoclostridium.s__Clostridium_asparagiforme	+	9.67E-01	0.000	0.000	0.000	0.000	0.000
4.2.1.59|g__Lachnoclostridium.s__Clostridium_bolteae	+	3.27E-01	0.000	0.000	0.000	0.001	0.001
4.2.1.59|g__Lachnoclostridium.s__Clostridium_citroniae	+	8.28E-01	0.000	0.000	0.000	0.000	0.000
4.2.1.59|g__Lachnoclostridium.s__Clostridium_clostridioforme	-	2.16E-02	0.000	0.000	0.000	0.000	0.000
4.2.1.59|g__Lachnoclostridium.s__Clostridium_lavalense	-	2.65E-01	0.000	0.000	0.000	0.000	0.000
4.2.1.59|g__Lachnoclostridium.s__Clostridium_saccharolyticum	-	1.84E-02	0.000	0.000	0.000	0.001	0.001
4.2.1.59|g__Lachnoclostridium.s__Clostridium_scindens	+	6.42E-01	0.000	0.000	0.000	0.000	0.001
4.2.1.59|g__Lachnoclostridium.s__Clostridium_symbiosum	+	2.34E-01	0.000	0.000	0.000	0.000	0.001
4.2.1.59|g__Lachnoclostridium.s__Lachnoclostridium_sp_An118	+	1.96E-01	0.000	0.000	0.000	0.000	0.000
4.2.1.59|g__Lachnospira.s__Lachnospira_pectinoschiza	-	1.56E-01	0.000	0.000	0.000	0.003	0.001
4.2.1.59|g__Lactobacillus.s__Lactobacillus_delbrueckii	+	3.68E-01	0.000	0.000	0.000	0.000	0.000
4.2.1.59|g__Lactobacillus.s__Lactobacillus_fermentum	-	9.05E-01	0.000	0.000	0.000	0.000	0.000
4.2.1.59|g__Lactobacillus.s__Lactobacillus_rogosae	-	1.36E-01	0.000	0.000	0.000	0.003	0.001
4.2.1.59|g__Lactobacillus.s__Lactobacillus_ruminis	+	3.68E-01	0.000	0.000	0.000	0.000	0.000
4.2.1.59|g__Lactococcus.s__Lactococcus_garvieae	-	9.05E-01	0.000	0.000	0.000	0.000	0.000
4.2.1.59|g__Lactococcus.s__Lactococcus_lactis	+	3.68E-01	0.000	0.000	0.000	0.000	0.000
4.2.1.59|g__Lactococcus.s__Lactococcus_petauri	+	6.86E-01	0.000	0.000	0.000	0.000	0.000
4.2.1.59|g__Lawsonibacter.s__Lawsonibacter_asaccharolyticus	+	6.94E-01	0.000	0.000	0.000	0.001	0.001
4.2.1.59|g__Leclercia.s__Leclercia_adecarboxylata	-	4.89E-01	0.000	0.000	0.000	0.000	0.000
4.2.1.59|g__Lelliottia.s__Lelliottia_nimipressuralis	-	3.14E-01	0.000	0.000	0.000	0.002	0.000
4.2.1.59|g__Leuconostoc.s__Leuconostoc_garlicum	+	6.86E-01	0.000	0.000	0.000	0.000	0.000
4.2.1.59|g__Listeria.s__Listeria_monocytogenes	+	1.96E-01	0.000	0.000	0.000	0.000	0.000
4.2.1.59|g__Megamonas.s__Megamonas_funiformis	-	4.29E-01	0.000	0.000	0.000	0.004	0.003
4.2.1.59|g__Megamonas.s__Megamonas_hypermegale	-	2.34E-01	0.000	0.000	0.000	0.006	0.001
4.2.1.59|g__Megamonas.s__Megamonas_rupellensis	-	3.33E-01	0.000	0.000	0.000	0.004	0.004
4.2.1.59|g__Megasphaera.s__Megasphaera_elsdenii	-	1.25E-01	0.000	0.000	0.000	0.000	0.000
4.2.1.59|g__Megasphaera.s__Megasphaera_hexanoica	+	3.68E-01	0.000	0.000	0.000	0.000	0.000
4.2.1.59|g__Megasphaera.s__Megasphaera_micronuciformis	+	1.96E-01	0.000	0.000	0.000	0.000	0.000
4.2.1.59|g__Megasphaera.s__Megasphaera_stantonii	-	5.44E-01	0.000	0.000	0.000	0.000	0.000
4.2.1.59|g__Mesosutterella.s__Mesosutterella_multiformis	-	6.23E-01	0.000	0.000	0.000	0.004	0.003
4.2.1.59|g__Metakosakonia.s__Kluyvera_intestini	+	3.68E-01	0.000	0.000	0.000	0.000	0.000
4.2.1.59|g__Mitsuokella.s__Mitsuokella_jalaludinii	-	2.50E-01	0.000	0.000	0.000	0.000	0.000
4.2.1.59|g__Mitsuokella.s__Mitsuokella_multacida	-	5.37E-01	0.000	0.000	0.000	0.001	0.001
4.2.1.59|g__Murimonas.s__Murimonas_intestini	+	3.68E-01	0.000	0.000	0.000	0.000	0.000
4.2.1.59|g__Neisseria.s__Neisseria_mucosa	-	2.85E-01	0.000	0.000	0.000	0.000	0.000
4.2.1.59|g__Neisseria.s__Neisseria_subflava	-	2.85E-01	0.000	0.000	0.000	0.000	0.000
4.2.1.59|g__Odoribacter.s__Odoribacter_laneus	+	3.87E-01	0.000	0.000	0.000	0.000	0.002
4.2.1.59|g__Oscillibacter.s__Oscillibacter_sp_PC13	+	3.68E-01	0.000	0.000	0.000	0.000	0.000
4.2.1.59|g__Oxalobacter.s__Oxalobacter_formigenes	-	1.90E-01	0.000	0.000	0.000	0.000	0.000
4.2.1.59|g__Paeniclostridium.s__Paeniclostridium_sordellii	+	3.68E-01	0.000	0.000	0.000	0.000	0.000
4.2.1.59|g__Pantoea.s__Pantoea_sesami	-	3.07E-01	0.000	0.000	0.000	0.000	0.000
4.2.1.59|g__Parabacteroides.s__Parabacteroides_chinchillae	-	1.25E-01	0.000	0.000	0.000	0.000	0.000
4.2.1.59|g__Parabacteroides.s__Parabacteroides_gordonii	-	8.60E-01	0.000	0.000	0.000	0.000	0.000
4.2.1.59|g__Parabacteroides.s__Parabacteroides_johnsonii	+	7.50E-01	0.000	0.000	0.000	0.002	0.001
4.2.1.59|g__Paraprevotella.s__Paraprevotella_clara	-	6.82E-01	0.000	0.000	0.000	0.007	0.007
4.2.1.59|g__Paraprevotella.s__Paraprevotella_xylaniphila	+	8.39E-02	0.000	0.000	0.000	0.000	0.002
4.2.1.59|g__Parasutterella.s__Parasutterella_excrementihominis	+	6.61E-01	0.000	0.000	0.000	0.005	0.002
4.2.1.59|g__Pedobacter.s__Pedobacter_himalayensis	-	2.85E-01	0.000	0.000	0.000	0.000	0.000
4.2.1.59|g__Phascolarctobacterium.s__Phascolarctobacterium_succinatutens	+	3.66E-01	0.000	0.000	0.000	0.003	0.004
4.2.1.59|g__Phytobacter.s__Phytobacter_ursingii	+	3.68E-01	0.000	0.000	0.000	0.000	0.000
4.2.1.59|g__Plesiomonas.s__Plesiomonas_shigelloides	-	2.85E-01	0.000	0.000	0.000	0.001	0.000

4.2.1.59|g__Prevotella.s__Prevotella_buccae	-	2.85E-01	0.000	0.000	0.000	0.001	0.000
4.2.1.59|g__Prevotella.s__Prevotella_corporis	+	1.96E-01	0.000	0.000	0.000	0.000	0.000
4.2.1.59|g__Prevotella.s__Prevotella_sp_109	+	5.12E-01	0.000	0.000	0.000	0.011	0.007
4.2.1.59|g__Pseudocitrobacter.s__Pseudocitrobacter_faecalis	-	9.26E-01	0.000	0.000	0.000	0.000	0.000
4.2.1.59|g__Pseudoflavonifractor.s__Pseudoflavonifractor_capillosus	+	3.68E-01	0.000	0.000	0.000	0.000	0.000
4.2.1.59|g__Pseudoflavonifractor.s__Pseudoflavonifractor_sp_An184	-	8.72E-01	0.000	0.000	0.000	0.000	0.000
4.2.1.59|g__Pseudomonas.s__Pseudomonas_helleri	-	2.85E-01	0.000	0.000	0.000	0.000	0.000
4.2.1.59|g__Pyramidobacter.s__Pyramidobacter_piscolens	-	9.26E-01	0.000	0.000	0.000	0.000	0.000
4.2.1.59|g__Pyramidobacter.s__Pyramidobacter_sp_C12_8	+	3.68E-01	0.000	0.000	0.000	0.000	0.000
4.2.1.59|g__Raoultella.s__Raoultella_ornithinolytica	-	4.57E-01	0.000	0.000	0.000	0.001	0.000
4.2.1.59|g__Raoultella.s__Raoultella_planticola	-	4.84E-01	0.000	0.000	0.000	0.000	0.000
4.2.1.59|g__Roseburia.s__Roseburia_intestinalis	-	8.16E-01	0.000	0.000	0.000	0.006	0.003
4.2.1.59|g__Ruminococcaceae_unclassified.s__Ruminococcaceae_bacteriun	-	3.48E-01	0.000	0.000	0.000	0.000	0.000
4.2.1.59|g__Ruminococcus.s__Ruminococcus_lactaris	-	1.94E-01	0.000	0.000	0.000	0.005	0.001
4.2.1.59|g__Ruminococcus.s__Ruminococcus_sp_AF31_8BH	+	2.15E-01	0.000	0.000	0.000	0.000	0.000
4.2.1.59|g__Salmonella.s__Salmonella_enterica	-	2.04E-01	0.000	0.000	0.000	0.001	0.000
4.2.1.59|g__Sanguibacteroides.s__Sanguibacteroides_justesenii	+	4.19E-01	0.000	0.000	0.000	0.000	0.000
4.2.1.59|g__Sellimonas.s__Sellimonas_intestinalis	-	9.02E-01	0.000	0.000	0.000	0.000	0.000
4.2.1.59|g__Streptococcus.s__Streptococcus_australis	+	3.68E-01	0.000	0.000	0.000	0.000	0.000
4.2.1.59|g__Streptococcus.s__Streptococcus_cristatus	-	2.85E-01	0.000	0.000	0.000	0.000	0.000
4.2.1.59|g__Streptococcus.s__Streptococcus_gallolyticus	+	1.96E-01	0.000	0.000	0.000	0.000	0.000
4.2.1.59|g__Streptococcus.s__Streptococcus_gordonii	-	9.26E-01	0.000	0.000	0.000	0.000	0.000
4.2.1.59|g__Streptococcus.s__Streptococcus_macedonicus	+	3.68E-01	0.000	0.000	0.000	0.000	0.000
4.2.1.59|g__Streptococcus.s__Streptococcus_milleri	+	3.68E-01	0.000	0.000	0.000	0.000	0.000
4.2.1.59|g__Streptococcus.s__Streptococcus_oralis	-	2.85E-01	0.000	0.000	0.000	0.000	0.000
4.2.1.59|g__Streptococcus.s__Streptococcus_parasanguinis	+	5.92E-01	0.000	0.000	0.000	0.001	0.001
4.2.1.59|g__Streptococcus.s__Streptococcus_pasteurianus	+	1.96E-01	0.000	0.000	0.000	0.000	0.000
4.2.1.59|g__Streptococcus.s__Streptococcus_pneumoniae	-	3.32E-02	0.000	0.000	0.000	0.000	0.000
4.2.1.59|g__Streptococcus.s__Streptococcus_pseudopneumoniae	-	2.85E-01	0.000	0.000	0.000	0.000	0.000
4.2.1.59|g__Streptococcus.s__Streptococcus_salivarius	+	4.95E-01	0.000	0.000	0.000	0.001	0.001
4.2.1.59|g__Streptococcus.s__Streptococcus_sanguinis	-	2.85E-01	0.000	0.000	0.000	0.000	0.000
4.2.1.59|g__Streptococcus.s__Streptococcus_thermophilus	-	4.71E-01	0.000	0.000	0.000	0.000	0.000
4.2.1.59|g__Streptococcus.s__Streptococcus_vestibularis	+	1.10E-01	0.000	0.000	0.000	0.000	0.000
4.2.1.59|g__Succinatimonas.s__Succinatimonas_hippei	+	2.68E-01	0.000	0.000	0.000	0.000	0.001
4.2.1.59|g__Sutterella.s__Sutterella_parvirubra	-	4.57E-01	0.000	0.000	0.000	0.004	0.000
4.2.1.59|g__Turicibacter.s__Turicibacter_sanguinis	-	9.05E-01	0.000	0.000	0.000	0.000	0.000
4.2.1.59|g__Turicimonas.s__Turicimonas_muris	+	3.68E-01	0.000	0.000	0.000	0.000	0.000
4.2.1.59|g__Tyzzerella.s__Tyzzerella_nexilis	+	7.35E-01	0.000	0.000	0.000	0.001	0.001
4.2.1.59|g__Veillonella.s__Veillonella_atypica	-	2.24E-01	0.000	0.000	0.000	0.000	0.000
4.2.1.59|g__Veillonella.s__Veillonella_dispar	-	7.02E-01	0.000	0.000	0.000	0.001	0.000
4.2.1.59|g__Veillonella.s__Veillonella_infantium	+	8.90E-01	0.000	0.000	0.000	0.000	0.000
4.2.1.59|g__Veillonella.s__Veillonella_parvula	-	1.22E-01	0.000	0.000	0.000	0.001	0.000
4.2.1.59|g__Veillonella.s__Veillonella_rogosae	-	2.87E-02	0.000	0.000	0.000	0.000	0.000
4.2.1.59|g__Veillonella.s__Veillonella_tobetsuensis	-	8.87E-01	0.000	0.000	0.000	0.000	0.000
4.2.1.59|g__Veillonellaceae_unclassified.s__Veillonellaceae_bacterium	+	3.68E-01	0.000	0.000	0.000	0.000	0.000
4.2.1.59|g__Vibrio.s__Vibrio_parahaemolyticus	-	2.85E-01	0.000	0.000	0.000	0.000	0.000
4.2.1.59|g__Weissella.s__Weissella_confusa	-	9.26E-01	0.000	0.000	0.000	0.000	0.000
4.2.1.59|g__Yokenella.s__Yokenella_regensburgei	-	4.99E-01	0.000	0.000	0.000	0.000	0.000
4.2.1.59|g__Alistipes.s__Alistipes_onderdonkii	+	8.99E-01	0.067	0.081	0.014	0.008	0.009
4.2.1.59|g__Parabacteroides.s__Parabacteroides_goldsteinii	+	2.74E-02	0.000	0.024	0.024	0.001	0.002
4.2.1.59|g__Bacteroides.s__Bacteroides_finegoldii	+	1.89E-01	0.000	0.029	0.029	0.004	0.006
4.2.1.59|g__Faecalibacterium.s__Faecalibacterium_prausnitzii	+	7.61E-01	2.764	2.839	0.076	0.045	0.042
4.2.1.59|g__Eubacterium.s__Eubacterium_eligens	+	7.23E-01	0.295	0.372	0.077	0.009	0.009
4.2.1.59|g__Phascolarctobacterium.s__Phascolarctobacterium_faecium	+	4.61E-01	0.412	0.503	0.091	0.010	0.013
4.2.1.59|g__Alistipes.s__Alistipes_finegoldii	+	2.20E-01	0.110	0.220	0.110	0.006	0.013
4.2.1.59|g__Roseburia.s__Roseburia_hominis	+	7.14E-01	0.036	0.152	0.116	0.002	0.006
4.2.1.59|g__Bacteroides.s__Bacteroides_ovatus	+	3.05E-01	0.310	0.484	0.174	0.011	0.014
4.2.1.59|g__Gemmiger.s__Gemmiger_formicilis	+	1.89E-01	0.000	0.284	0.284	0.005	0.006
4.2.1.59|g__Odoribacter.s__Odoribacter_splanchnicus	+	5.30E-02	0.290	0.603	0.313	0.006	0.008
4.2.1.59|g__Bacteroides.s__Bacteroides_stercoris	-	8.38E-01	1.435	1.760	0.325	0.047	0.038
4.2.1.59|g__Bacteroides.s__Bacteroides_cellulosilyticus	+	7.86E-03	0.000	0.349	0.349	0.009	0.011
4.2.1.59|g__Bacteroides.s__Bacteroides_thetaiotaomicron	+	6.37E-02	0.755	1.221	0.466	0.014	0.022
4.2.1.59|g__Bacteroides.s__Bacteroides_intestinalis	+	4.46E-03	0.010	0.479	0.469	0.005	0.010
4.2.1.59|g__Parabacteroides.s__Parabacteroides_distasonis	+	2.21E-02	0.954	1.464	0.510	0.013	0.021
4.2.1.59|g__Sutterella.s__Sutterella_wadsworthensis	+	9.08E-01	0.325	0.847	0.522	0.020	0.019
4.2.1.59|g__Parabacteroides.s__Parabacteroides_merdae	+	1.89E-02	0.494	1.049	0.555	0.009	0.015
4.2.1.59|g__Blautia.s__Ruminococcus_torques	+	2.91E-01	0.582	1.211	0.629	0.019	0.020
4.2.1.59|g__Alistipes.s__Alistipes_shahii	+	3.71E-02	0.256	0.904	0.648	0.007	0.014
4.2.1.59|g__Bacteroides.s__Bacteroides_caccae	+	3.10E-02	0.383	1.301	0.918	0.011	0.015
4.2.1.59|g__Alistipes.s__Alistipes_putredinis	+	1.80E-01	1.376	2.607	1.230	0.024	0.033
4.2.1.59|unclassified	+	9.43E-01	13.331	17.224	3.893	0.149	0.167
				Fractional CPM (median)		Relative CPM (average)	

Country	EC number|Bacteria	Increased or decreased in PD	*p* -value	Control	PD	PD - Control	Control	PD
Japan	2.6.1.62	-	4.06E-02	45.965	41.156	-4.809	1.000	1.000
	2.6.1.62|g__Blautia.s__Blautia_obeum	-	5.03E-05	3.427	1.264	-2.164	0.117	0.063
	2.6.1.62|g__Blautia.s__Blautia_wexlerae	-	4.26E-04	2.766	1.320	-1.447	0.080	0.053
	2.6.1.62|unclassified	-	4.23E-01	1.909	1.543	-0.366	0.062	0.065
	2.6.1.62|g__Acidaminococcus.s__Acidaminococcus_intestini	-	8.39E-01	0.000	0.000	0.000	0.018	0.006
	2.6.1.62|g__Akkermansia.s__Akkermansia_muciniphila	+	4.53E-03	0.000	0.000	0.000	0.012	0.060
	2.6.1.62|g__Alistipes.s__Alistipes_indistinctus	+	2.27E-02	0.000	0.000	0.000	0.001	0.003
	2.6.1.62|g__Anaeromassilibacillus.s__Anaeromassilibacillus_sp_An172	+	2.14E-01	0.000	0.000	0.000	0.000	0.001
	2.6.1.62|g__Bacteroides.s__Bacteroides_caccae	+	7.00E-02	0.000	0.000	0.000	0.008	0.014
	2.6.1.62|g__Bacteroides.s__Bacteroides_cellulosilyticus	-	9.16E-01	0.000	0.000	0.000	0.002	0.002
	2.6.1.62|g__Bacteroides.s__Bacteroides_clarus	+	5.62E-02	0.000	0.000	0.000	0.000	0.007
	2.6.1.62|g__Bacteroides.s__Bacteroides_coprocola	+	2.38E-01	0.000	0.000	0.000	0.025	0.031
	2.6.1.62|g__Bacteroides.s__Bacteroides_coprophilus	+	4.35E-01	0.000	0.000	0.000	0.002	0.002
	2.6.1.62|g__Bacteroides.s__Bacteroides_eggerthii	+	1.80E-01	0.000	0.000	0.000	0.007	0.019
	2.6.1.62|g__Bacteroides.s__Bacteroides_faecis	-	2.60E-01	0.000	0.000	0.000	0.000	0.001
	2.6.1.62|g__Bacteroides.s__Bacteroides_finegoldii	+	1.94E-01	0.000	0.000	0.000	0.005	0.006
	2.6.1.62|g__Bacteroides.s__Bacteroides_fluxus	-	2.62E-01	0.000	0.000	0.000	0.001	0.000
	2.6.1.62|g__Bacteroides.s__Bacteroides_fragilis	+	7.84E-01	0.000	0.000	0.000	0.009	0.014
	2.6.1.62|g__Bacteroides.s__Bacteroides_massiliensis	+	2.20E-01	0.000	0.000	0.000	0.006	0.008
	2.6.1.62|g__Bacteroides.s__Bacteroides_nordii	+	5.86E-01	0.000	0.000	0.000	0.000	0.001
	2.6.1.62|g__Bacteroides.s__Bacteroides_plebeius	+	3.95E-01	0.000	0.000	0.000	0.042	0.035
	2.6.1.62|g__Bacteroides.s__Bacteroides_salyersiae	+	1.75E-01	0.000	0.000	0.000	0.000	0.001
	2.6.1.62|g__Barnesiella.s__Barnesiella_intestinihominis	+	9.17E-03	0.000	0.000	0.000	0.006	0.017
	2.6.1.62|g__Barnesiella.s__Barnesiella_sp_An22	-	2.62E-01	0.000	0.000	0.000	0.000	0.000
	2.6.1.62|g__Blautia.s__Blautia_sp_AF19_10LB	+	7.58E-01	0.000	0.000	0.000	0.007	0.013
	2.6.1.62|g__Blautia.s__Ruminococcus_torques	-	2.43E-01	0.000	0.000	0.000	0.023	0.024
	2.6.1.62|g__Butyricimonas.s__Butyricimonas_virosa	+	4.22E-01	0.000	0.000	0.000	0.001	0.001
	2.6.1.62|g__Catenibacterium.s__Catenibacterium_mitsuokai	-	8.54E-03	0.000	0.000	0.000	0.006	0.000
	2.6.1.62|g__Citrobacter.s__Citrobacter_freundii	+	2.82E-01	0.000	0.000	0.000	0.000	0.003
	2.6.1.62|g__Citrobacter.s__Citrobacter_koseri	+	3.85E-01	0.000	0.000	0.000	0.000	0.000
	2.6.1.62|g__Citrobacter.s__Citrobacter_portucalensis	+	3.85E-01	0.000	0.000	0.000	0.000	0.000
	2.6.1.62|g__Citrobacter.s__Citrobacter_werkmanii	+	3.85E-01	0.000	0.000	0.000	0.000	0.004
	2.6.1.62|g__Citrobacter.s__Citrobacter_youngae	+	2.14E-01	0.000	0.000	0.000	0.000	0.001
	2.6.1.62|g__Cloacibacillus.s__Cloacibacillus_porcorum	+	1.26E-01	0.000	0.000	0.000	0.000	0.002
	2.6.1.62|g__Coprobacter.s__Coprobacter_fastidiosus	+	2.72E-01	0.000	0.000	0.000	0.002	0.002
	2.6.1.62|g__Coprobacter.s__Coprobacter_secundus	+	1.82E-01	0.000	0.000	0.000	0.000	0.001
	2.6.1.62|g__Desulfovibrio.s__Desulfovibrio_sp_AM18_2	+	3.85E-01	0.000	0.000	0.000	0.000	0.000
	2.6.1.62|g__Enterobacter.s__Enterobacter_asburiae	-	8.71E-01	0.000	0.000	0.000	0.000	0.000
	2.6.1.62|g__Enterobacter.s__Enterobacter_bugandensis	-	4.24E-01	0.000	0.000	0.000	0.000	0.000
	2.6.1.62|g__Enterobacter.s__Enterobacter_cloacae	+	3.34E-01	0.000	0.000	0.000	0.002	0.001
	2.6.1.62|g__Enterobacter.s__Enterobacter_mori	+	1.26E-01	0.000	0.000	0.000	0.000	0.001
	2.6.1.62|g__Escherichia.s__Escherichia_coli	+	2.48E-01	0.000	0.000	0.000	0.033	0.015
	2.6.1.62|g__Escherichia.s__Escherichia_fergusonii	-	2.48E-01	0.000	0.000	0.000	0.000	0.000
	2.6.1.62|g__Fusobacterium.s__Fusobacterium_mortiferum	-	1.03E-01	0.000	0.000	0.000	0.001	0.001
	2.6.1.62|g__Fusobacterium.s__Fusobacterium_varium	-	2.62E-01	0.000	0.000	0.000	0.000	0.000
	2.6.1.62|g__Haemophilus.s__Haemophilus_parainfluenzae	-	4.48E-02	0.000	0.000	0.000	0.002	0.000
	2.6.1.62|g__Hungatella.s__Hungatella_effluvii	+	3.85E-01	0.000	0.000	0.000	0.000	0.000
	2.6.1.62|g__Hungatella.s__Hungatella_hathewayi	+	4.46E-01	0.000	0.000	0.000	0.000	0.000
	2.6.1.62|g__Klebsiella.s__Klebsiella_aerogenes	+	8.23E-01	0.000	0.000	0.000	0.003	0.001
	2.6.1.62|g__Klebsiella.s__Klebsiella_grimontii	-	2.62E-01	0.000	0.000	0.000	0.001	0.000
	2.6.1.62|g__Klebsiella.s__Klebsiella_michiganensis	-	8.12E-01	0.000	0.000	0.000	0.000	0.001
	2.6.1.62|g__Klebsiella.s__Klebsiella_oxytoca	+	7.34E-01	0.000	0.000	0.000	0.009	0.004
	2.6.1.62|g__Klebsiella.s__Klebsiella_pneumoniae	+	8.78E-01	0.000	0.000	0.000	0.006	0.014
	2.6.1.62|g__Klebsiella.s__Klebsiella_variicola	-	1.31E-01	0.000	0.000	0.000	0.001	0.000
	2.6.1.62|g__Lachnoclostridium.s__Clostridium_saccharolyticum	+	9.81E-01	0.000	0.000	0.000	0.001	0.001
	2.6.1.62|g__Lachnoclostridium.s__Clostridium_symbiosum	+	7.93E-01	0.000	0.000	0.000	0.000	0.000
	2.6.1.62|g__Leclercia.s__Leclercia_adecarboxylata	+	3.85E-01	0.000	0.000	0.000	0.000	0.000
	2.6.1.62|g__Lelliottia.s__Lelliottia_amnigena	-	1.09E-01	0.000	0.000	0.000	0.000	0.000
	2.6.1.62|g__Lelliottia.s__Lelliottia_nimipressuralis	-	4.54E-01	0.000	0.000	0.000	0.000	0.000
	2.6.1.62|g__Megamonas.s__Megamonas_funiformis	-	9.10E-01	0.000	0.000	0.000	0.020	0.016
	2.6.1.62|g__Megamonas.s__Megamonas_hypermegale	-	7.75E-01	0.000	0.000	0.000	0.006	0.003
	2.6.1.62|g__Megamonas.s__Megamonas_rupellensis	-	8.05E-01	0.000	0.000	0.000	0.004	0.001
	2.6.1.62|g__Metakosakonia.s__Kluyvera_intestini	+	3.85E-01	0.000	0.000	0.000	0.000	0.000
	2.6.1.62|g__Odoribacter.s__Odoribacter_laneus	+	4.67E-02	0.000	0.000	0.000	0.000	0.002
	2.6.1.62|g__Odoribacter.s__Odoribacter_splanchnicus	+	7.05E-02	0.000	0.000	0.000	0.003	0.006
	2.6.1.62|g__Paeniclostridium.s__Paeniclostridium_sordellii	-	2.62E-01	0.000	0.000	0.000	0.000	0.000
	2.6.1.62|g__Pantoea.s__Pantoea_sesami	-	2.56E-01	0.000	0.000	0.000	0.001	0.000
	2.6.1.62|g__Parabacteroides.s__Parabacteroides_goldsteinii	+	1.62E-02	0.000	0.000	0.000	0.000	0.002
	2.6.1.62|g__Parabacteroides.s__Parabacteroides_gordonii	+	1.00E+00	0.000	0.000	0.000	0.000	0.000
	2.6.1.62|g__Parabacteroides.s__Parabacteroides_johnsonii	+	4.34E-01	0.000	0.000	0.000	0.002	0.002
	2.6.1.62|g__Paraprevotella.s__Paraprevotella_clara	+	5.60E-01	0.000	0.000	0.000	0.003	0.003

	2.6.1.62|g__Paraprevotella.s__Paraprevotella_xylaniphila	+	2.77E-01	0.000	0.000	0.000	0.000	0.001
	2.6.1.62|g__Phascolarctobacterium.s__Phascolarctobacterium_faecium	+	2.22E-01	0.000	0.000	0.000	0.008	0.019
	2.6.1.62|g__Phascolarctobacterium.s__Phascolarctobacterium_succinatutens	-	5.93E-01	0.000	0.000	0.000	0.009	0.006
	2.6.1.62|g__Raoultella.s__Raoultella_ornithinolytica	-	4.88E-02	0.000	0.000	0.000	0.005	0.000
	2.6.1.62|g__Raoultella.s__Raoultella_planticola	-	2.62E-01	0.000	0.000	0.000	0.000	0.000
	2.6.1.62|g__Rikenella.s__Rikenella_microfusus	+	2.14E-01	0.000	0.000	0.000	0.000	0.002
	2.6.1.62|g__Roseburia.s__Roseburia_intestinalis	-	2.31E-03	0.000	0.000	0.000	0.026	0.004
	2.6.1.62|g__Sanguibacteroides.s__Sanguibacteroides_justesenii	+	3.85E-01	0.000	0.000	0.000	0.000	0.000
	2.6.1.62|g__Staphylococcus.s__Staphylococcus_aureus	+	2.14E-01	0.000	0.000	0.000	0.000	0.000
	2.6.1.62|g__Streptococcus.s__Streptococcus_pneumoniae	-	2.62E-01	0.000	0.000	0.000	0.000	0.000
	2.6.1.62|g__Succinatimonas.s__Succinatimonas_hippei	+	7.21E-01	0.000	0.000	0.000	0.000	0.001
	2.6.1.62|g__Terrisporobacter.s__Terrisporobacter_othiniensis	+	3.85E-01	0.000	0.000	0.000	0.000	0.001
	2.6.1.62|g__Veillonella.s__Veillonella_atypica	-	2.98E-03	0.000	0.000	0.000	0.004	0.000
	2.6.1.62|g__Veillonella.s__Veillonella_denticariosi	+	2.14E-01	0.000	0.000	0.000	0.000	0.000
	2.6.1.62|g__Veillonella.s__Veillonella_dispar	-	2.06E-03	0.000	0.000	0.000	0.005	0.000
	2.6.1.62|g__Veillonella.s__Veillonella_infantium	-	3.43E-03	0.000	0.000	0.000	0.005	0.000
	2.6.1.62|g__Veillonella.s__Veillonella_parvula	-	3.63E-02	0.000	0.000	0.000	0.003	0.001
	2.6.1.62|g__Veillonella.s__Veillonella_rodentium	+	7.10E-01	0.000	0.000	0.000	0.000	0.000
	2.6.1.62|g__Veillonella.s__Veillonella_rogosae	-	4.95E-04	0.000	0.000	0.000	0.001	0.000
	2.6.1.62|g__Veillonella.s__Veillonella_tobetsuensis	-	4.88E-02	0.000	0.000	0.000	0.000	0.000
	2.6.1.62|g__Yokenella.s__Yokenella_regensburgei	-	7.93E-01	0.000	0.000	0.000	0.000	0.000
	2.6.1.62|g__Bacteroides.s__Bacteroides_stercoris	+	9.29E-01	0.000	0.017	0.017	0.052	0.044
	2.6.1.62|g__Bacteroides.s__Bacteroides_ovatus	+	4.81E-01	0.293	0.318	0.026	0.021	0.025
	2.6.1.62|g__Bacteroides.s__Bacteroides_xylanisolvens	+	9.65E-01	0.033	0.061	0.027	0.018	0.020
	2.6.1.62|g__Bacteroides.s__Bacteroides_vulgatus	-	7.33E-01	0.999	1.117	0.118	0.080	0.061
	2.6.1.62|g__Bacteroides.s__Bacteroides_thetaiotaomicron	+	1.68E-01	0.200	0.374	0.174	0.013	0.021
	2.6.1.62|g__Bacteroides.s__Bacteroides_dorei	+	7.04E-01	0.942	1.131	0.189	0.061	0.062
	2.6.1.62|g__Parabacteroides.s__Parabacteroides_merdae	+	2.34E-01	0.339	0.579	0.240	0.018	0.023
	2.6.1.62|g__Parabacteroides.s__Parabacteroides_distasonis	+	5.63E-03	0.481	1.064	0.583	0.022	0.035
	2.6.1.62|g__Bacteroides.s__Bacteroides_uniformis	+	3.93E-01	3.477	4.063	0.586	0.105	0.134
USA	2.6.1.62	-	4.41E-02	68.112	61.741	-6.371	1.000	1.000
	2.6.1.62|g__Blautia.s__Blautia_obeum	-	1.50E-09	4.687	2.548	-2.140	0.093	0.065
	2.6.1.62|g__Blautia.s__Blautia_wexlerae	-	3.40E-09	4.816	2.699	-2.116	0.099	0.067
	2.6.1.62|g__Bacteroides.s__Bacteroides_vulgatus	-	6.73E-02	5.633	3.788	-1.845	0.100	0.089
	2.6.1.62|g__Bacteroides.s__Bacteroides_uniformis	-	7.00E-01	7.608	6.638	-0.970	0.138	0.142
	2.6.1.62|g__Roseburia.s__Roseburia_intestinalis	-	8.17E-07	0.299	0.000	-0.299	0.023	0.011
	2.6.1.62|g__Bacteroides.s__Bacteroides_dorei	-	7.82E-01	0.906	0.854	-0.052	0.034	0.037
	2.6.1.62|g__Odoribacter.s__Odoribacter_splanchnicus	+	6.93E-01	0.250	0.235	-0.014	0.006	0.007
	2.6.1.62|g__Bacteroides.s__Bacteroides_ovatus	+	5.70E-01	0.664	0.650	-0.014	0.027	0.026
	2.6.1.62|g__Acidaminococcus.s__Acidaminococcus_intestini	+	3.94E-03	0.000	0.000	0.000	0.009	0.019
	2.6.1.62|g__Aeromonas.s__Aeromonas_allosaccharophila	-	1.48E-01	0.000	0.000	0.000	0.000	0.000
	2.6.1.62|g__Aeromonas.s__Aeromonas_caviae	+	4.92E-01	0.000	0.000	0.000	0.000	0.000
	2.6.1.62|g__Aeromonas.s__Aeromonas_diversa	-	1.48E-01	0.000	0.000	0.000	0.000	0.000
	2.6.1.62|g__Aeromonas.s__Aeromonas_veronii	-	1.48E-01	0.000	0.000	0.000	0.000	0.000
	2.6.1.62|g__Aggregatibacter.s__Aggregatibacter_segnis	+	4.92E-01	0.000	0.000	0.000	0.000	0.000
	2.6.1.62|g__Akkermansia.s__Akkermansia_muciniphila	+	2.33E-01	0.000	0.000	0.000	0.038	0.065
	2.6.1.62|g__Alistipes.s__Alistipes_indistinctus	+	1.68E-02	0.000	0.000	0.000	0.001	0.003
	2.6.1.62|g__Anaerobiospirillum.s__Anaerobiospirillum_thomasii	+	4.92E-01	0.000	0.000	0.000	0.000	0.000
	2.6.1.62|g__Anaerococcus.s__Anaerococcus_nagyae	+	4.92E-01	0.000	0.000	0.000	0.000	0.000
	2.6.1.62|g__Anaeromassilibacillus.s__Anaeromassilibacillus_sp_An172	-	2.03E-01	0.000	0.000	0.000	0.000	0.000
	2.6.1.62|g__Bacillus.s__Bacillus_coagulans	+	4.92E-01	0.000	0.000	0.000	0.000	0.000
	2.6.1.62|g__Bacteroides.s__Bacteroides_cellulosilyticus	+	9.56E-01	0.000	0.000	0.000	0.004	0.005
	2.6.1.62|g__Bacteroides.s__Bacteroides_clarus	+	6.83E-01	0.000	0.000	0.000	0.002	0.001
	2.6.1.62|g__Bacteroides.s__Bacteroides_coprocola	-	8.56E-02	0.000	0.000	0.000	0.010	0.005
	2.6.1.62|g__Bacteroides.s__Bacteroides_coprophilus	+	4.06E-01	0.000	0.000	0.000	0.000	0.001
	2.6.1.62|g__Bacteroides.s__Bacteroides_eggerthii	+	9.03E-01	0.000	0.000	0.000	0.018	0.018
	2.6.1.62|g__Bacteroides.s__Bacteroides_faecichinchillae	+	3.30E-01	0.000	0.000	0.000	0.000	0.000
	2.6.1.62|g__Bacteroides.s__Bacteroides_faecis	+	5.75E-02	0.000	0.000	0.000	0.004	0.004
	2.6.1.62|g__Bacteroides.s__Bacteroides_finegoldii	+	6.18E-01	0.000	0.000	0.000	0.004	0.004
	2.6.1.62|g__Bacteroides.s__Bacteroides_fluxus	+	2.32E-01	0.000	0.000	0.000	0.000	0.000
	2.6.1.62|g__Bacteroides.s__Bacteroides_fragilis	-	4.25E-01	0.000	0.000	0.000	0.013	0.013
	2.6.1.62|g__Bacteroides.s__Bacteroides_massiliensis	+	4.44E-01	0.000	0.000	0.000	0.021	0.017
	2.6.1.62|g__Bacteroides.s__Bacteroides_nordii	-	1.41E-01	0.000	0.000	0.000	0.001	0.001
	2.6.1.62|g__Bacteroides.s__Bacteroides_plebeius	-	2.73E-01	0.000	0.000	0.000	0.020	0.008
	2.6.1.62|g__Bacteroides.s__Bacteroides_salyersiae	+	2.98E-02	0.000	0.000	0.000	0.003	0.004
	2.6.1.62|g__Bacteroides.s__Bacteroides_sartorii	-	4.06E-02	0.000	0.000	0.000	0.000	0.000
	2.6.1.62|g__Barnesiella.s__Barnesiella_intestinihominis	+	1.57E-01	0.000	0.000	0.000	0.009	0.012
	2.6.1.62|g__Blautia.s__Blautia_sp_AF19_10LB	+	4.09E-01	0.000	0.000	0.000	0.002	0.003
	2.6.1.62|g__Blautia.s__Ruminococcus_torques	-	2.74E-02	0.000	0.000	0.000	0.012	0.009
	2.6.1.62|g__Butyricimonas.s__Butyricimonas_synergistica	-	1.48E-01	0.000	0.000	0.000	0.000	0.000
	2.6.1.62|g__Butyricimonas.s__Butyricimonas_virosa	+	1.67E-01	0.000	0.000	0.000	0.002	0.002
	2.6.1.62|g__Campylobacter.s__Campylobacter_coli	+	4.92E-01	0.000	0.000	0.000	0.000	0.000

2.6.1.62|g__Campylobacter.s__Campylobacter_jejuni	+	4.92E-01	0.000	0.000	0.000	0.000	0.000
2.6.1.62|g__Candidatus_Gastranaerophilales_unclassified.s__Candidatus_Ga	+	6.68E-02	0.000	0.000	0.000	0.000	0.000
2.6.1.62|g__Catenibacterium.s__Catenibacterium_mitsuokai	+	5.54E-01	0.000	0.000	0.000	0.004	0.006
2.6.1.62|g__Citrobacter.s__Citrobacter_amalonaticus	+	3.08E-01	0.000	0.000	0.000	0.000	0.000
2.6.1.62|g__Citrobacter.s__Citrobacter_braakii	-	9.67E-01	0.000	0.000	0.000	0.000	0.000
2.6.1.62|g__Citrobacter.s__Citrobacter_farmeri	+	3.30E-01	0.000	0.000	0.000	0.000	0.000
2.6.1.62|g__Citrobacter.s__Citrobacter_freundii	+	4.27E-01	0.000	0.000	0.000	0.000	0.000
2.6.1.62|g__Citrobacter.s__Citrobacter_portucalensis	-	9.67E-01	0.000	0.000	0.000	0.000	0.000
2.6.1.62|g__Citrobacter.s__Citrobacter_werkmanii	+	3.30E-01	0.000	0.000	0.000	0.000	0.000
2.6.1.62|g__Citrobacter.s__Citrobacter_youngae	+	4.16E-01	0.000	0.000	0.000	0.000	0.000
2.6.1.62|g__Cloacibacillus.s__Cloacibacillus_porcorum	+	1.50E-01	0.000	0.000	0.000	0.001	0.001
2.6.1.62|g__Clostridium.s__Clostridium_butyricum	-	5.92E-01	0.000	0.000	0.000	0.000	0.000
2.6.1.62|g__Coprobacter.s__Coprobacter_fastidiosus	-	3.20E-01	0.000	0.000	0.000	0.001	0.001
2.6.1.62|g__Coprobacter.s__Coprobacter_secundus	-	2.81E-01	0.000	0.000	0.000	0.000	0.000
2.6.1.62|g__Corynebacterium.s__Corynebacterium_amycolatum	+	3.30E-01	0.000	0.000	0.000	0.000	0.000
2.6.1.62|g__Corynebacterium.s__Corynebacterium_imitans	+	4.92E-01	0.000	0.000	0.000	0.000	0.000
2.6.1.62|g__Corynebacterium.s__Corynebacterium_jeikeium	+	3.30E-01	0.000	0.000	0.000	0.000	0.000
2.6.1.62|g__Corynebacterium.s__Corynebacterium_kroppenstedtii	+	4.92E-01	0.000	0.000	0.000	0.000	0.000
2.6.1.62|g__Corynebacterium.s__Corynebacterium_minutissimum	+	4.92E-01	0.000	0.000	0.000	0.000	0.000
2.6.1.62|g__Corynebacterium.s__Corynebacterium_pseudogenitalium	+	4.92E-01	0.000	0.000	0.000	0.000	0.000
2.6.1.62|g__Corynebacterium.s__Corynebacterium_sp_HMSC058E07	+	4.92E-01	0.000	0.000	0.000	0.000	0.000
2.6.1.62|g__Corynebacterium.s__Corynebacterium_tuberculostearicum	+	4.92E-01	0.000	0.000	0.000	0.000	0.000
2.6.1.62|g__Cronobacter.s__Cronobacter_malonaticus	+	4.92E-01	0.000	0.000	0.000	0.000	0.000
2.6.1.62|g__Cronobacter.s__Cronobacter_sakazakii	+	4.92E-01	0.000	0.000	0.000	0.000	0.000
2.6.1.62|g__Cutibacterium.s__Cutibacterium_avidum	+	3.30E-01	0.000	0.000	0.000	0.000	0.000
2.6.1.62|g__Delftia.s__Delftia_acidovorans	+	4.92E-01	0.000	0.000	0.000	0.000	0.000
2.6.1.62|g__Delftia.s__Delftia_lacustris	+	4.92E-01	0.000	0.000	0.000	0.000	0.000
2.6.1.62|g__Delftia.s__Delftia_tsuruhatensis	+	4.92E-01	0.000	0.000	0.000	0.000	0.000
2.6.1.62|g__Desulfovibrio.s__Desulfovibrio_sp_AM18_2	+	2.28E-01	0.000	0.000	0.000	0.000	0.000
2.6.1.62|g__Enterobacter.s__Enterobacter_asburiae	+	2.32E-01	0.000	0.000	0.000	0.000	0.000
2.6.1.62|g__Enterobacter.s__Enterobacter_bugandensis	+	5.57E-01	0.000	0.000	0.000	0.000	0.000
2.6.1.62|g__Enterobacter.s__Enterobacter_cloacae	+	6.49E-03	0.000	0.000	0.000	0.000	0.001
2.6.1.62|g__Enterobacter.s__Enterobacter_mori	+	4.92E-01	0.000	0.000	0.000	0.000	0.000
2.6.1.62|g__Escherichia.s__Escherichia_coli	+	6.39E-03	0.000	0.000	0.000	0.021	0.023
2.6.1.62|g__Escherichia.s__Escherichia_fergusonii	+	6.33E-01	0.000	0.000	0.000	0.000	0.000
2.6.1.62|g__Escherichia.s__Escherichia_marmotae	+	4.92E-01	0.000	0.000	0.000	0.000	0.000
2.6.1.62|g__Eubacteriaceae_unclassified.s__Eubacteriaceae_bacterium_CHK	-	9.70E-01	0.000	0.000	0.000	0.000	0.000
2.6.1.62|g__Fusobacterium.s__Fusobacterium_mortiferum	+	5.59E-01	0.000	0.000	0.000	0.000	0.000
2.6.1.62|g__Fusobacterium.s__Fusobacterium_nucleatum	-	1.48E-01	0.000	0.000	0.000	0.000	0.000
2.6.1.62|g__Fusobacterium.s__Fusobacterium_varium	+	4.92E-01	0.000	0.000	0.000	0.000	0.000
2.6.1.62|g__Haemophilus.s__Haemophilus_influenzae	+	4.92E-01	0.000	0.000	0.000	0.000	0.000
2.6.1.62|g__Haemophilus.s__Haemophilus_parainfluenzae	-	1.87E-01	0.000	0.000	0.000	0.000	0.000
2.6.1.62|g__Hafnia.s__Hafnia_paralvei	+	4.92E-01	0.000	0.000	0.000	0.000	0.000
2.6.1.62|g__Hungatella.s__Hungatella_effluvii	+	1.04E-02	0.000	0.000	0.000	0.000	0.000
2.6.1.62|g__Hungatella.s__Hungatella_hathewayi	+	1.09E-02	0.000	0.000	0.000	0.001	0.001
2.6.1.62|g__Klebsiella.s__Klebsiella_aerogenes	+	7.07E-03	0.000	0.000	0.000	0.000	0.001
2.6.1.62|g__Klebsiella.s__Klebsiella_grimontii	+	3.30E-01	0.000	0.000	0.000	0.000	0.000
2.6.1.62|g__Klebsiella.s__Klebsiella_michiganensis	-	9.57E-01	0.000	0.000	0.000	0.000	0.000
2.6.1.62|g__Klebsiella.s__Klebsiella_oxytoca	+	2.05E-02	0.000	0.000	0.000	0.004	0.005
2.6.1.62|g__Klebsiella.s__Klebsiella_pneumoniae	+	2.02E-03	0.000	0.000	0.000	0.010	0.012
2.6.1.62|g__Klebsiella.s__Klebsiella_variicola	+	2.72E-03	0.000	0.000	0.000	0.001	0.003
2.6.1.62|g__Kluyvera.s__Kluyvera_ascorbata	+	4.92E-01	0.000	0.000	0.000	0.000	0.000
2.6.1.62|g__Kluyvera.s__Kluyvera_cryocrescens	+	3.30E-01	0.000	0.000	0.000	0.000	0.000
2.6.1.62|g__Kosakonia.s__Kosakonia_cowanii	+	4.92E-01	0.000	0.000	0.000	0.000	0.000
2.6.1.62|g__Lachnoclostridium.s__Clostridium_saccharolyticum	+	8.69E-01	0.000	0.000	0.000	0.001	0.001
2.6.1.62|g__Lachnoclostridium.s__Clostridium_symbiosum	+	2.12E-01	0.000	0.000	0.000	0.002	0.003
2.6.1.62|g__Leclercia.s__Leclercia_adecarboxylata	+	2.32E-01	0.000	0.000	0.000	0.000	0.000
2.6.1.62|g__Lelliottia.s__Lelliottia_amnigena	+	1.67E-01	0.000	0.000	0.000	0.000	0.000
2.6.1.62|g__Lelliottia.s__Lelliottia_nimipressuralis	+	2.32E-01	0.000	0.000	0.000	0.000	0.000
2.6.1.62|g__Megamonas.s__Megamonas_funiformis	+	6.64E-01	0.000	0.000	0.000	0.001	0.001
2.6.1.62|g__Megamonas.s__Megamonas_hypermegale	+	6.62E-01	0.000	0.000	0.000	0.000	0.000
2.6.1.62|g__Megamonas.s__Megamonas_rupellensis	+	6.56E-01	0.000	0.000	0.000	0.000	0.000
2.6.1.62|g__Morganella.s__Morganella_morganii	+	2.32E-01	0.000	0.000	0.000	0.000	0.000
2.6.1.62|g__Odoribacter.s__Odoribacter_laneus	+	2.69E-01	0.000	0.000	0.000	0.003	0.005
2.6.1.62|g__Oligella.s__Oligella_urethralis	+	4.92E-01	0.000	0.000	0.000	0.000	0.001
2.6.1.62|g__Oxalobacter.s__Oxalobacter_formigenes	+	4.72E-01	0.000	0.000	0.000	0.000	0.000
2.6.1.62|g__Pantoea.s__Pantoea_sesami	+	2.31E-01	0.000	0.000	0.000	0.000	0.000
2.6.1.62|g__Parabacteroides.s__Parabacteroides_goldsteinii	+	2.50E-02	0.000	0.000	0.000	0.002	0.003
2.6.1.62|g__Parabacteroides.s__Parabacteroides_gordonii	-	6.39E-01	0.000	0.000	0.000	0.000	0.000
2.6.1.62|g__Parabacteroides.s__Parabacteroides_johnsonii	-	5.98E-01	0.000	0.000	0.000	0.001	0.001
2.6.1.62|g__Paraprevotella.s__Paraprevotella_clara	-	1.30E-01	0.000	0.000	0.000	0.007	0.006
2.6.1.62|g__Paraprevotella.s__Paraprevotella_xylaniphila	+	3.10E-01	0.000	0.000	0.000	0.001	0.000

	2.6.1.62|g__Pedobacter.s__Pedobacter_himalayensis	-	9.70E-01	0.000	0.000	0.000	0.000	0.000
	2.6.1.62|g__Peptoniphilus.s__Peptoniphilus_harei	+	2.32E-01	0.000	0.000	0.000	0.000	0.000
	2.6.1.62|g__Phascolarctobacterium.s__Phascolarctobacterium_succinatutens	+	3.52E-01	0.000	0.000	0.000	0.002	0.001
	2.6.1.62|g__Pluralibacter.s__Pluralibacter_gergoviae	-	1.48E-01	0.000	0.000	0.000	0.000	0.000
	2.6.1.62|g__Porphyromonas.s__Porphyromonas_asaccharolytica	+	1.88E-01	0.000	0.000	0.000	0.000	0.001
	2.6.1.62|g__Porphyromonas.s__Porphyromonas_uenonis	-	5.95E-01	0.000	0.000	0.000	0.000	0.000
	2.6.1.62|g__Proteus.s__Proteus_mirabilis	+	6.64E-01	0.000	0.000	0.000	0.000	0.000
	2.6.1.62|g__Pseudocitrobacter.s__Pseudocitrobacter_faecalis	+	4.92E-01	0.000	0.000	0.000	0.000	0.000
	2.6.1.62|g__Pseudomonas.s__Pseudomonas_aeruginosa	+	9.02E-01	0.000	0.000	0.000	0.001	0.000
	2.6.1.62|g__Pseudomonas.s__Pseudomonas_aeruginosa_group	+	7.61E-01	0.000	0.000	0.000	0.001	0.000
	2.6.1.62|g__Pseudomonas.s__Pseudomonas_citronellolis	+	4.92E-01	0.000	0.000	0.000	0.000	0.000
	2.6.1.62|g__Pseudomonas.s__Pseudomonas_delhiensis	+	4.92E-01	0.000	0.000	0.000	0.000	0.000
	2.6.1.62|g__Raoultella.s__Raoultella_ornithinolytica	-	9.67E-01	0.000	0.000	0.000	0.000	0.000
	2.6.1.62|g__Raoultella.s__Raoultella_planticola	+	4.92E-01	0.000	0.000	0.000	0.000	0.000
	2.6.1.62|g__Rikenella.s__Rikenella_microfusus	+	4.92E-01	0.000	0.000	0.000	0.000	0.000
	2.6.1.62|g__Salmonella.s__Salmonella_enterica	+	4.92E-01	0.000	0.000	0.000	0.000	0.000
	2.6.1.62|g__Sanguibacteroides.s__Sanguibacteroides_justesenii	+	4.99E-02	0.000	0.000	0.000	0.000	0.000
	2.6.1.62|g__Staphylococcus.s__Staphylococcus_aureus	+	1.67E-01	0.000	0.000	0.000	0.000	0.000
	2.6.1.62|g__Streptococcus.s__Streptococcus_pneumoniae	-	9.57E-01	0.000	0.000	0.000	0.000	0.000
	2.6.1.62|g__Succinatimonas.s__Succinatimonas_hippei	-	9.73E-01	0.000	0.000	0.000	0.000	0.000
	2.6.1.62|g__Terrisporobacter.s__Terrisporobacter_othiniensis	+	1.67E-01	0.000	0.000	0.000	0.000	0.000
	2.6.1.62|g__Veillonella.s__Veillonella_atypica	-	1.98E-01	0.000	0.000	0.000	0.001	0.001
	2.6.1.62|g__Veillonella.s__Veillonella_dispar	-	9.30E-02	0.000	0.000	0.000	0.000	0.000
	2.6.1.62|g__Veillonella.s__Veillonella_infantium	-	2.95E-01	0.000	0.000	0.000	0.000	0.000
	2.6.1.62|g__Veillonella.s__Veillonella_parvula	+	8.63E-01	0.000	0.000	0.000	0.001	0.001
	2.6.1.62|g__Veillonella.s__Veillonella_rogosae	-	7.47E-02	0.000	0.000	0.000	0.000	0.000
	2.6.1.62|g__Veillonella.s__Veillonella_seminalis	-	4.47E-01	0.000	0.000	0.000	0.000	0.000
	2.6.1.62|g__Veillonella.s__Veillonella_tobetsuensis	-	4.06E-02	0.000	0.000	0.000	0.000	0.000
	2.6.1.62|g__Vibrio.s__Vibrio_parahaemolyticus	+	5.59E-01	0.000	0.000	0.000	0.000	0.000
	2.6.1.62|g__Yokenella.s__Yokenella_regensburgei	-	9.70E-01	0.000	0.000	0.000	0.000	0.000
	2.6.1.62|g__Bacteroides.s__Bacteroides_thetaiotaomicron	+	3.11E-01	0.423	0.441	0.017	0.018	0.019
	2.6.1.62|g__Bacteroides.s__Bacteroides_xylanisolvens	+	3.84E-01	0.000	0.020	0.020	0.013	0.010
	2.6.1.62|unclassified	+	4.19E-01	2.704	2.732	0.028	0.064	0.076
	2.6.1.62|g__Parabacteroides.s__Parabacteroides_merdae	+	6.18E-02	0.653	0.828	0.175	0.016	0.020
	2.6.1.62|g__Bacteroides.s__Bacteroides_caccae	+	1.38E-01	0.176	0.405	0.228	0.013	0.020
	2.6.1.62|g__Phascolarctobacterium.s__Phascolarctobacterium_faecium	+	3.23E-01	0.000	0.325	0.325	0.010	0.011
	2.6.1.62|g__Parabacteroides.s__Parabacteroides_distasonis	+	6.90E-03	1.096	1.442	0.346	0.033	0.040
	2.6.1.62|g__Bacteroides.s__Bacteroides_stercoris	+	8.11E-02	0.344	0.864	0.520	0.072	0.085
Germany	2.6.1.62	-	4.71E-01	44.798	42.481	-2.316	1.000	1.000
	2.6.1.62|g__Bacteroides.s__Bacteroides_dorei	-	6.93E-01	1.797	1.395	-0.402	0.048	0.050
	2.6.1.62|g__Roseburia.s__Roseburia_intestinalis	-	2.31E-01	0.348	0.000	-0.348	0.046	0.017
	2.6.1.62|g__Blautia.s__Blautia_obeum	-	3.53E-01	0.466	0.290	-0.176	0.015	0.022
	2.6.1.62|g__Bacteroides.s__Bacteroides_thetaiotaomicron	-	4.88E-01	0.400	0.263	-0.138	0.021	0.011
	2.6.1.62|g__Blautia.s__Blautia_wexlerae	-	2.75E-01	0.435	0.366	-0.069	0.014	0.018
	2.6.1.62|g__Parabacteroides.s__Parabacteroides_distasonis	+	7.32E-01	1.243	1.175	-0.069	0.030	0.032
	2.6.1.62|g__Blautia.s__Ruminococcus_torques	-	2.93E-01	0.046	0.000	-0.046	0.014	0.006
	2.6.1.62|g__Bacteroides.s__Bacteroides_xylanisolvens	-	7.92E-01	0.210	0.197	-0.013	0.060	0.035
	2.6.1.62|g__Acidaminococcus.s__Acidaminococcus_intestini	-	9.31E-01	0.000	0.000	0.000	0.003	0.004
	2.6.1.62|g__Alistipes.s__Alistipes_indistinctus	-	7.52E-01	0.000	0.000	0.000	0.002	0.003
	2.6.1.62|g__Bacteroides.s__Bacteroides_cellulosilyticus	+	6.90E-01	0.000	0.000	0.000	0.002	0.002
	2.6.1.62|g__Bacteroides.s__Bacteroides_clarus	-	9.27E-01	0.000	0.000	0.000	0.007	0.005
	2.6.1.62|g__Bacteroides.s__Bacteroides_coprocola	-	7.50E-01	0.000	0.000	0.000	0.045	0.015
	2.6.1.62|g__Bacteroides.s__Bacteroides_coprophilus	-	8.75E-01	0.000	0.000	0.000	0.008	0.000
	2.6.1.62|g__Bacteroides.s__Bacteroides_eggerthii	+	7.06E-01	0.000	0.000	0.000	0.017	0.009
	2.6.1.62|g__Bacteroides.s__Bacteroides_faecichinchillae	+	3.59E-01	0.000	0.000	0.000	0.000	0.000
	2.6.1.62|g__Bacteroides.s__Bacteroides_faecis	+	7.71E-01	0.000	0.000	0.000	0.006	0.005
	2.6.1.62|g__Bacteroides.s__Bacteroides_finegoldii	+	4.18E-01	0.000	0.000	0.000	0.004	0.006
	2.6.1.62|g__Bacteroides.s__Bacteroides_fluxus	-	1.40E-01	0.000	0.000	0.000	0.000	0.000
	2.6.1.62|g__Bacteroides.s__Bacteroides_fragilis	-	8.73E-01	0.000	0.000	0.000	0.010	0.012
	2.6.1.62|g__Bacteroides.s__Bacteroides_massiliensis	-	7.83E-01	0.000	0.000	0.000	0.034	0.025
	2.6.1.62|g__Bacteroides.s__Bacteroides_nordii	+	5.53E-01	0.000	0.000	0.000	0.000	0.001
	2.6.1.62|g__Bacteroides.s__Bacteroides_plebeius	+	3.37E-01	0.000	0.000	0.000	0.007	0.012
	2.6.1.62|g__Bacteroides.s__Bacteroides_salyersiae	+	5.69E-01	0.000	0.000	0.000	0.003	0.006
	2.6.1.62|g__Bacteroides.s__Bacteroides_stercoris	+	8.85E-01	0.000	0.000	0.000	0.031	0.019
	2.6.1.62|g__Blautia.s__Blautia_sp_AF19_10LB	+	2.23E-01	0.000	0.000	0.000	0.001	0.004
	2.6.1.62|g__Butyricimonas.s__Butyricimonas_virosa	-	6.86E-02	0.000	0.000	0.000	0.005	0.002
	2.6.1.62|g__Candidatus_Gastranaerophilales_unclassified.s__Candidatus_Ga	+	2.20E-01	0.000	0.000	0.000	0.002	0.003
	2.6.1.62|g__Catenibacterium.s__Catenibacterium_mitsuokai	-	9.36E-02	0.000	0.000	0.000	0.024	0.012
	2.6.1.62|g__Cloacibacillus.s__Cloacibacillus_porcorum	+	6.04E-01	0.000	0.000	0.000	0.000	0.001
	2.6.1.62|g__Coprobacter.s__Coprobacter_fastidiosus	-	7.33E-01	0.000	0.000	0.000	0.004	0.003
	2.6.1.62|g__Coprobacter.s__Coprobacter_secundus	+	6.32E-01	0.000	0.000	0.000	0.000	0.000
	2.6.1.62|g__Enterobacter.s__Enterobacter_cloacae	-	9.42E-01	0.000	0.000	0.000	0.000	0.000

	2.6.1.62|g__Escherichia.s__Escherichia_coli	-	3.08E-01	0.000	0.000	0.000	0.051	0.027
	2.6.1.62|g__Escherichia.s__Escherichia_fergusonii	-	1.84E-01	0.000	0.000	0.000	0.001	0.000
	2.6.1.62|g__Escherichia.s__Escherichia_marmotae	-	3.09E-01	0.000	0.000	0.000	0.000	0.000
	2.6.1.62|g__Eubacteriaceae_unclassified.s__Eubacteriaceae_bacterium_CHI	-	9.42E-01	0.000	0.000	0.000	0.000	0.000
	2.6.1.62|g__Fusobacterium.s__Fusobacterium_mortiferum	-	3.09E-01	0.000	0.000	0.000	0.000	0.000
	2.6.1.62|g__Haemophilus.s__Haemophilus_influenzae	-	1.40E-01	0.000	0.000	0.000	0.000	0.000
	2.6.1.62|g__Haemophilus.s__Haemophilus_parainfluenzae	-	6.66E-02	0.000	0.000	0.000	0.001	0.000
	2.6.1.62|g__Hafnia.s__Hafnia_alvei	+	1.83E-01	0.000	0.000	0.000	0.000	0.002
	2.6.1.62|g__Hafnia.s__Hafnia_paralvei	+	3.59E-01	0.000	0.000	0.000	0.000	0.003
	2.6.1.62|g__Hungatella.s__Hungatella_effluvii	+	3.59E-01	0.000	0.000	0.000	0.000	0.000
	2.6.1.62|g__Hungatella.s__Hungatella_hathewayi	+	3.59E-01	0.000	0.000	0.000	0.000	0.000
	2.6.1.62|g__Klebsiella.s__Klebsiella_aerogenes	-	6.66E-02	0.000	0.000	0.000	0.000	0.000
	2.6.1.62|g__Klebsiella.s__Klebsiella_oxytoca	-	7.75E-02	0.000	0.000	0.000	0.012	0.002
	2.6.1.62|g__Klebsiella.s__Klebsiella_pneumoniae	-	7.51E-02	0.000	0.000	0.000	0.012	0.002
	2.6.1.62|g__Klebsiella.s__Klebsiella_variicola	+	6.32E-01	0.000	0.000	0.000	0.000	0.000
	2.6.1.62|g__Lachnoclostridium.s__Clostridium_saccharolyticum	-	3.22E-02	0.000	0.000	0.000	0.001	0.000
	2.6.1.62|g__Lachnoclostridium.s__Clostridium_symbiosum	-	9.31E-01	0.000	0.000	0.000	0.000	0.000
	2.6.1.62|g__Megamonas.s__Megamonas_funiformis	+	3.59E-01	0.000	0.000	0.000	0.000	0.001
	2.6.1.62|g__Megamonas.s__Megamonas_hypermegale	+	3.59E-01	0.000	0.000	0.000	0.000	0.001
	2.6.1.62|g__Megamonas.s__Megamonas_rupellensis	+	3.59E-01	0.000	0.000	0.000	0.000	0.000
	2.6.1.62|g__Obesumbacterium.s__Obesumbacterium_proteus	+	3.59E-01	0.000	0.000	0.000	0.000	0.001
	2.6.1.62|g__Oxalobacter.s__Oxalobacter_formigenes	+	4.78E-01	0.000	0.000	0.000	0.001	0.001
	2.6.1.62|g__Parabacteroides.s__Parabacteroides_goldsteinii	-	2.24E-01	0.000	0.000	0.000	0.001	0.001
	2.6.1.62|g__Parabacteroides.s__Parabacteroides_gordonii	+	1.83E-01	0.000	0.000	0.000	0.000	0.000
	2.6.1.62|g__Parabacteroides.s__Parabacteroides_johnsonii	-	3.18E-01	0.000	0.000	0.000	0.003	0.000
	2.6.1.62|g__Paraprevotella.s__Paraprevotella_clara	-	5.76E-02	0.000	0.000	0.000	0.010	0.006
	2.6.1.62|g__Phascolarctobacterium.s__Phascolarctobacterium_faecium	+	2.52E-01	0.000	0.000	0.000	0.010	0.021
	2.6.1.62|g__Phascolarctobacterium.s__Phascolarctobacterium_succinatutens	-	5.96E-02	0.000	0.000	0.000	0.012	0.003
	2.6.1.62|g__Pseudomonas.s__Pseudomonas_aeruginosa	-	9.42E-01	0.000	0.000	0.000	0.000	0.000
	2.6.1.62|g__Pseudomonas.s__Pseudomonas_aeruginosa_group	-	9.42E-01	0.000	0.000	0.000	0.000	0.000
	2.6.1.62|g__Raoultella.s__Raoultella_ornithinolytica	+	3.59E-01	0.000	0.000	0.000	0.000	0.000
	2.6.1.62|g__Raoultella.s__Raoultella_planticola	+	3.59E-01	0.000	0.000	0.000	0.000	0.000
	2.6.1.62|g__Streptococcus.s__Streptococcus_pneumoniae	-	6.66E-02	0.000	0.000	0.000	0.000	0.000
	2.6.1.62|g__Veillonella.s__Veillonella_atypica	-	1.40E-01	0.000	0.000	0.000	0.000	0.000
	2.6.1.62|g__Veillonella.s__Veillonella_dispar	-	8.75E-01	0.000	0.000	0.000	0.001	0.000
	2.6.1.62|g__Veillonella.s__Veillonella_infantium	-	5.11E-01	0.000	0.000	0.000	0.001	0.000
	2.6.1.62|g__Veillonella.s__Veillonella_parvula	+	6.04E-01	0.000	0.000	0.000	0.000	0.000
	2.6.1.62|g__Veillonella.s__Veillonella_rogosae	-	9.81E-01	0.000	0.000	0.000	0.000	0.000
	2.6.1.62|g__Bacteroides.s__Bacteroides_ovatus	-	5.68E-01	0.300	0.310	0.009	0.014	0.021
	2.6.1.62|g__Odoribacter.s__Odoribacter_splanchnicus	-	7.67E-01	0.422	0.499	0.077	0.014	0.012
	2.6.1.62|g__Bacteroides.s__Bacteroides_caccae	-	5.60E-01	0.686	0.819	0.133	0.029	0.022
	2.6.1.62|g__Bacteroides.s__Bacteroides_vulgatus	-	9.94E-01	2.640	2.798	0.158	0.105	0.107
	2.6.1.62|unclassified	+	7.56E-01	3.386	3.588	0.202	0.093	0.120
	2.6.1.62|g__Parabacteroides.s__Parabacteroides_merdae	+	6.61E-01	0.477	0.697	0.220	0.019	0.021
	2.6.1.62|g__Barnesiella.s__Barnesiella_intestinihominis	+	5.28E-01	0.622	0.952	0.331	0.028	0.032
	2.6.1.62|g__Bacteroides.s__Bacteroides_uniformis	+	5.29E-01	2.645	3.104	0.459	0.094	0.108
	2.6.1.62|g__Akkermansia.s__Akkermansia_muciniphila	+	1.32E-03	0.000	2.337	2.337	0.022	0.141
China1	2.6.1.62	-	5.34E-03	73.494	55.505	-17.989	1.000	1.000
	2.6.1.62|g__Bacteroides.s__Bacteroides_vulgatus	-	1.22E-02	7.568	2.159	-5.410	0.174	0.097
	2.6.1.62|g__Bacteroides.s__Bacteroides_fragilis	-	1.40E-01	2.022	0.973	-1.049	0.050	0.039
	2.6.1.62|g__Bacteroides.s__Bacteroides_ovatus	-	2.72E-02	1.949	1.040	-0.909	0.052	0.029
	2.6.1.62|g__Bacteroides.s__Bacteroides_dorei	-	2.88E-01	1.245	0.932	-0.313	0.079	0.054
	2.6.1.62|g__Bacteroides.s__Bacteroides_cellulosilyticus	-	4.83E-01	0.112	0.059	-0.053	0.009	0.015
	2.6.1.62|g__Blautia.s__Blautia_obeum	-	3.34E-01	0.048	0.000	-0.048	0.002	0.002
	2.6.1.62|g__Bacteroides.s__Bacteroides_thetaiotaomicron	-	8.97E-01	1.254	1.208	-0.046	0.034	0.054
	2.6.1.62|g__Klebsiella.s__Klebsiella_oxytoca	-	2.28E-01	0.034	0.000	-0.034	0.004	0.005
	2.6.1.62|g__Escherichia.s__Escherichia_coli	+	9.72E-01	0.155	0.122	-0.033	0.024	0.036
	2.6.1.62|g__Blautia.s__Blautia_wexlerae	-	2.10E-01	0.018	0.000	-0.018	0.001	0.001
	2.6.1.62|unclassified	-	9.12E-01	1.008	0.991	-0.017	0.025	0.029
	2.6.1.62|g__Acidaminococcus.s__Acidaminococcus_intestini	+	3.30E-01	0.000	0.000	0.000	0.000	0.000
	2.6.1.62|g__Aeromonas.s__Aeromonas_allosaccharophila	-	3.30E-01	0.000	0.000	0.000	0.000	0.000
	2.6.1.62|g__Aeromonas.s__Aeromonas_diversa	-	3.30E-01	0.000	0.000	0.000	0.000	0.000
	2.6.1.62|g__Aeromonas.s__Aeromonas_hydrophila	-	3.30E-01	0.000	0.000	0.000	0.000	0.000
	2.6.1.62|g__Aeromonas.s__Aeromonas_sobria	-	3.30E-01	0.000	0.000	0.000	0.000	0.000
	2.6.1.62|g__Aeromonas.s__Aeromonas_veronii	-	3.30E-01	0.000	0.000	0.000	0.000	0.000
	2.6.1.62|g__Aggregatibacter.s__Aggregatibacter_segnis	-	3.30E-01	0.000	0.000	0.000	0.000	0.000
	2.6.1.62|g__Akkermansia.s__Akkermansia_muciniphila	+	2.77E-02	0.000	0.000	0.000	0.001	0.011
	2.6.1.62|g__Bacteroides.s__Bacteroides_clarus	+	1.43E-01	0.000	0.000	0.000	0.001	0.006
	2.6.1.62|g__Bacteroides.s__Bacteroides_coprocola	-	2.76E-01	0.000	0.000	0.000	0.052	0.024
	2.6.1.62|g__Bacteroides.s__Bacteroides_coprophilus	-	6.22E-01	0.000	0.000	0.000	0.008	0.001
	2.6.1.62|g__Bacteroides.s__Bacteroides_eggerthii	+	1.47E-01	0.000	0.000	0.000	0.022	0.038
	2.6.1.62|g__Bacteroides.s__Bacteroides_faecichinchillae	+	8.20E-02	0.000	0.000	0.000	0.000	0.000

	2.6.1.62|g__Bacteroides.s__Bacteroides_faecis	-	1.58E-01	0.000	0.000	0.000	0.002	0.000
	2.6.1.62|g__Bacteroides.s__Bacteroides_finegoldii	+	3.21E-01	0.000	0.000	0.000	0.008	0.017
	2.6.1.62|g__Bacteroides.s__Bacteroides_fluxus	+	3.30E-01	0.000	0.000	0.000	0.000	0.001
	2.6.1.62|g__Bacteroides.s__Bacteroides_massiliensis	+	7.52E-01	0.000	0.000	0.000	0.012	0.020
	2.6.1.62|g__Bacteroides.s__Bacteroides_plebeius	-	3.40E-01	0.000	0.000	0.000	0.077	0.033
	2.6.1.62|g__Bacteroides.s__Bacteroides_salyersiae	-	7.85E-01	0.000	0.000	0.000	0.003	0.003
	2.6.1.62|g__Barnesiella.s__Barnesiella_intestinihominis	+	2.86E-01	0.000	0.000	0.000	0.010	0.010
	2.6.1.62|g__Barnesiella.s__Barnesiella_sp_An22	-	3.30E-01	0.000	0.000	0.000	0.000	0.000
	2.6.1.62|g__Blautia.s__Blautia_sp_AF19_10LB	-	9.93E-01	0.000	0.000	0.000	0.000	0.001
	2.6.1.62|g__Blautia.s__Ruminococcus_torques	-	5.18E-01	0.000	0.000	0.000	0.001	0.001
	2.6.1.62|g__Butyricimonas.s__Butyricimonas_virosa	+	9.68E-01	0.000	0.000	0.000	0.004	0.005
	2.6.1.62|g__Catenibacterium.s__Catenibacterium_mitsuokai	+	6.06E-01	0.000	0.000	0.000	0.001	0.008
	2.6.1.62|g__Citrobacter.s__Citrobacter_freundii	+	4.28E-02	0.000	0.000	0.000	0.000	0.000
	2.6.1.62|g__Citrobacter.s__Citrobacter_portucalensis	+	1.60E-01	0.000	0.000	0.000	0.000	0.000
	2.6.1.62|g__Citrobacter.s__Citrobacter_youngae	+	5.49E-01	0.000	0.000	0.000	0.000	0.000
	2.6.1.62|g__Cloacibacillus.s__Cloacibacillus_porcorum	+	8.20E-02	0.000	0.000	0.000	0.000	0.000
	2.6.1.62|g__Coprobacter.s__Coprobacter_fastidiosus	+	8.60E-01	0.000	0.000	0.000	0.002	0.002
	2.6.1.62|g__Coprobacter.s__Coprobacter_secundus	+	9.48E-01	0.000	0.000	0.000	0.000	0.001
	2.6.1.62|g__Desulfovibrio.s__Desulfovibrio_sp_AM18_2	+	8.20E-02	0.000	0.000	0.000	0.000	0.002
	2.6.1.62|g__Enterobacter.s__Enterobacter_cloacae	+	3.08E-01	0.000	0.000	0.000	0.000	0.000
	2.6.1.62|g__Escherichia.s__Escherichia_fergusonii	+	8.71E-01	0.000	0.000	0.000	0.000	0.000
	2.6.1.62|g__Fusobacterium.s__Fusobacterium_mortiferum	-	6.71E-01	0.000	0.000	0.000	0.001	0.000
	2.6.1.62|g__Fusobacterium.s__Fusobacterium_varium	-	5.49E-01	0.000	0.000	0.000	0.001	0.000
	2.6.1.62|g__Haemophilus.s__Haemophilus_influenzae	-	5.69E-01	0.000	0.000	0.000	0.000	0.000
	2.6.1.62|g__Haemophilus.s__Haemophilus_parainfluenzae	-	7.14E-01	0.000	0.000	0.000	0.001	0.001
	2.6.1.62|g__Hafnia.s__Hafnia_paralvei	-	3.30E-01	0.000	0.000	0.000	0.000	0.000
	2.6.1.62|g__Hungatella.s__Hungatella_effluvii	-	7.16E-01	0.000	0.000	0.000	0.000	0.000
	2.6.1.62|g__Hungatella.s__Hungatella_hathewayi	-	4.86E-01	0.000	0.000	0.000	0.000	0.000
	2.6.1.62|g__Klebsiella.s__Klebsiella_aerogenes	+	3.30E-01	0.000	0.000	0.000	0.000	0.000
	2.6.1.62|g__Klebsiella.s__Klebsiella_pneumoniae	+	6.82E-01	0.000	0.000	0.000	0.002	0.008
	2.6.1.62|g__Klebsiella.s__Klebsiella_variicola	+	4.28E-02	0.000	0.000	0.000	0.000	0.001
	2.6.1.62|g__Lachnoclostridium.s__Clostridium_saccharolyticum	+	3.30E-01	0.000	0.000	0.000	0.000	0.000
	2.6.1.62|g__Lachnoclostridium.s__Clostridium_symbiosum	-	9.57E-01	0.000	0.000	0.000	0.000	0.000
	2.6.1.62|g__Megamonas.s__Megamonas_funiformis	+	4.40E-01	0.000	0.000	0.000	0.006	0.004
	2.6.1.62|g__Megamonas.s__Megamonas_hypermegale	+	4.17E-01	0.000	0.000	0.000	0.000	0.000
	2.6.1.62|g__Megamonas.s__Megamonas_rupellensis	+	4.17E-01	0.000	0.000	0.000	0.000	0.001
	2.6.1.62|g__Oxalobacter.s__Oxalobacter_formigenes	+	3.93E-01	0.000	0.000	0.000	0.000	0.001
	2.6.1.62|g__Parabacteroides.s__Parabacteroides_goldsteinii	+	6.11E-02	0.000	0.000	0.000	0.001	0.007
	2.6.1.62|g__Parabacteroides.s__Parabacteroides_gordonii	+	2.59E-01	0.000	0.000	0.000	0.001	0.002
	2.6.1.62|g__Parabacteroides.s__Parabacteroides_johnsonii	+	6.61E-02	0.000	0.000	0.000	0.001	0.004
	2.6.1.62|g__Paraprevotella.s__Paraprevotella_clara	+	9.34E-03	0.000	0.000	0.000	0.003	0.010
	2.6.1.62|g__Paraprevotella.s__Paraprevotella_xylaniphila	+	9.90E-01	0.000	0.000	0.000	0.000	0.002
	2.6.1.62|g__Phascolarctobacterium.s__Phascolarctobacterium_succinatutens	+	1.52E-01	0.000	0.000	0.000	0.002	0.005
	2.6.1.62|g__Proteus.s__Proteus_mirabilis	+	3.30E-01	0.000	0.000	0.000	0.000	0.000
	2.6.1.62|g__Pseudocitrobacter.s__Pseudocitrobacter_faecalis	+	3.30E-01	0.000	0.000	0.000	0.000	0.000
	2.6.1.62|g__Roseburia.s__Roseburia_intestinalis	-	7.39E-01	0.000	0.000	0.000	0.001	0.001
	2.6.1.62|g__Sanguibacteroides.s__Sanguibacteroides_justesenii	+	8.20E-02	0.000	0.000	0.000	0.000	0.000
	2.6.1.62|g__Streptococcus.s__Streptococcus_pneumoniae	+	1.87E-01	0.000	0.000	0.000	0.000	0.000
	2.6.1.62|g__Succinatimonas.s__Succinatimonas_hippei	-	3.30E-01	0.000	0.000	0.000	0.000	0.000
	2.6.1.62|g__Veillonella.s__Veillonella_atypica	+	3.16E-01	0.000	0.000	0.000	0.001	0.002
	2.6.1.62|g__Veillonella.s__Veillonella_dispar	+	9.19E-01	0.000	0.000	0.000	0.003	0.001
	2.6.1.62|g__Veillonella.s__Veillonella_infantium	+	8.73E-01	0.000	0.000	0.000	0.003	0.001
	2.6.1.62|g__Veillonella.s__Veillonella_parvula	+	5.89E-01	0.000	0.000	0.000	0.003	0.003
	2.6.1.62|g__Veillonella.s__Veillonella_rodentium	-	3.30E-01	0.000	0.000	0.000	0.000	0.000
	2.6.1.62|g__Veillonella.s__Veillonella_rogosae	+	1.05E-01	0.000	0.000	0.000	0.000	0.000
	2.6.1.62|g__Veillonella.s__Veillonella_tobetsuensis	+	4.05E-01	0.000	0.000	0.000	0.000	0.000
	2.6.1.62|g__Parabacteroides.s__Parabacteroides_distasonis	+	6.97E-01	0.922	0.973	0.051	0.032	0.033
	2.6.1.62|g__Alistipes.s__Alistipes_indistinctus	+	8.54E-03	0.000	0.051	0.051	0.004	0.008
	2.6.1.62|g__Bacteroides.s__Bacteroides_nordii	+	7.59E-01	0.054	0.124	0.070	0.004	0.006
	2.6.1.62|g__Bacteroides.s__Bacteroides_stercoris	+	7.60E-01	0.705	0.803	0.099	0.078	0.061
	2.6.1.62|g__Bacteroides.s__Bacteroides_xylanisolvens	+	8.96E-01	0.325	0.509	0.184	0.024	0.025
	2.6.1.62|g__Phascolarctobacterium.s__Phascolarctobacterium_faecium	+	4.56E-01	0.730	0.929	0.199	0.030	0.034
	2.6.1.62|g__Odoribacter.s__Odoribacter_splanchnicus	+	9.07E-01	0.015	0.242	0.227	0.009	0.010
	2.6.1.62|g__Parabacteroides.s__Parabacteroides_merdae	+	2.86E-01	0.073	0.406	0.332	0.015	0.027
	2.6.1.62|g__Bacteroides.s__Bacteroides_caccae	+	4.31E-01	0.193	0.678	0.485	0.018	0.031
	2.6.1.62|g__Bacteroides.s__Bacteroides_uniformis	+	1.92E-01	4.581	7.749	3.168	0.094	0.165
China2	2.6.1.62	-	8.93E-02	79.956	66.626	-13.330	1.000	1.000
	2.6.1.62|g__Bacteroides.s__Bacteroides_vulgatus	-	7.95E-02	7.133	3.671	-3.462	0.168	0.111
	2.6.1.62|g__Bacteroides.s__Bacteroides_dorei	-	1.36E-01	1.870	0.721	-1.149	0.060	0.043
	2.6.1.62|g__Phascolarctobacterium.s__Phascolarctobacterium_faecium	-	4.54E-01	0.859	0.292	-0.567	0.015	0.016
	2.6.1.62|g__Bacteroides.s__Bacteroides_ovatus	+	8.34E-01	0.964	0.601	-0.363	0.023	0.023
	2.6.1.62|g__Bacteroides.s__Bacteroides_thetaiotaomicron	-	2.42E-01	0.448	0.300	-0.148	0.017	0.012

2.6.1.62|g__Bacteroides.s__Bacteroides_xylanisolvens	-	6.83E-02	0.271	0.166	-0.105	0.020	0.010
2.6.1.62|g__Veillonella.s__Veillonella_parvula	-	6.22E-01	0.040	0.000	-0.040	0.003	0.004
2.6.1.62|g__Blautia.s__Blautia_obeum	+	9.36E-01	0.282	0.270	-0.012	0.015	0.021
2.6.1.62|g__Acidaminococcus.s__Acidaminococcus_intestini	-	5.69E-01	0.000	0.000	0.000	0.001	0.001
2.6.1.62|g__Aggregatibacter.s__Aggregatibacter_segnis	-	1.60E-01	0.000	0.000	0.000	0.000	0.000
2.6.1.62|g__Akkermansia.s__Akkermansia_muciniphila	+	3.92E-01	0.000	0.000	0.000	0.003	0.012
2.6.1.62|g__Alistipes.s__Alistipes_indistinctus	+	5.71E-03	0.000	0.000	0.000	0.000	0.004
2.6.1.62|g__Atlantibacter.s__Atlantibacter_hermannii	-	3.30E-01	0.000	0.000	0.000	0.000	0.000
2.6.1.62|g__Bacteroides.s__Bacteroides_cellulosilyticus	-	9.17E-01	0.000	0.000	0.000	0.005	0.007
2.6.1.62|g__Bacteroides.s__Bacteroides_clarus	-	5.86E-01	0.000	0.000	0.000	0.001	0.006
2.6.1.62|g__Bacteroides.s__Bacteroides_coprocola	-	1.34E-01	0.000	0.000	0.000	0.042	0.016
2.6.1.62|g__Bacteroides.s__Bacteroides_coprophilus	+	2.85E-01	0.000	0.000	0.000	0.015	0.011
2.6.1.62|g__Bacteroides.s__Bacteroides_eggerthii	-	3.10E-01	0.000	0.000	0.000	0.013	0.005
2.6.1.62|g__Bacteroides.s__Bacteroides_faecichinchillae	+	5.49E-01	0.000	0.000	0.000	0.000	0.000
2.6.1.62|g__Bacteroides.s__Bacteroides_faecis	+	3.93E-01	0.000	0.000	0.000	0.001	0.003
2.6.1.62|g__Bacteroides.s__Bacteroides_finegoldii	-	1.32E-01	0.000	0.000	0.000	0.014	0.002
2.6.1.62|g__Bacteroides.s__Bacteroides_massiliensis	-	4.27E-01	0.000	0.000	0.000	0.028	0.008
2.6.1.62|g__Bacteroides.s__Bacteroides_nordii	+	2.01E-01	0.000	0.000	0.000	0.001	0.002
2.6.1.62|g__Bacteroides.s__Bacteroides_plebeius	-	2.79E-01	0.000	0.000	0.000	0.099	0.047
2.6.1.62|g__Bacteroides.s__Bacteroides_salyersiae	-	6.25E-01	0.000	0.000	0.000	0.002	0.002
2.6.1.62|g__Bacteroides.s__Bacteroides_sartorii	-	3.30E-01	0.000	0.000	0.000	0.000	0.000
2.6.1.62|g__Barnesiella.s__Barnesiella_intestinihominis	+	5.94E-01	0.000	0.000	0.000	0.005	0.009
2.6.1.62|g__Blautia.s__Blautia_sp_AF19_10LB	+	4.59E-01	0.000	0.000	0.000	0.003	0.008
2.6.1.62|g__Blautia.s__Ruminococcus_torques	+	4.77E-01	0.000	0.000	0.000	0.002	0.003
2.6.1.62|g__Butyricimonas.s__Butyricimonas_synergistica	+	9.65E-01	0.000	0.000	0.000	0.000	0.000
2.6.1.62|g__Butyricimonas.s__Butyricimonas_virosa	+	1.51E-01	0.000	0.000	0.000	0.002	0.004
2.6.1.62|g__Catenibacterium.s__Catenibacterium_mitsuokai	+	5.49E-01	0.000	0.000	0.000	0.000	0.001
2.6.1.62|g__Citrobacter.s__Citrobacter_braakii	+	3.30E-01	0.000	0.000	0.000	0.000	0.000
2.6.1.62|g__Citrobacter.s__Citrobacter_freundii	+	4.17E-01	0.000	0.000	0.000	0.000	0.003
2.6.1.62|g__Citrobacter.s__Citrobacter_portucalensis	+	4.30E-01	0.000	0.000	0.000	0.000	0.001
2.6.1.62|g__Citrobacter.s__Citrobacter_werkmanii	+	1.00E+00	0.000	0.000	0.000	0.000	0.000
2.6.1.62|g__Citrobacter.s__Citrobacter_youngae	+	7.17E-01	0.000	0.000	0.000	0.000	0.002
2.6.1.62|g__Cloacibacillus.s__Cloacibacillus_porcorum	+	1.60E-01	0.000	0.000	0.000	0.000	0.000
2.6.1.62|g__Clostridium.s__Clostridium_butyricum	+	3.30E-01	0.000	0.000	0.000	0.000	0.000
2.6.1.62|g__Coprobacter.s__Coprobacter_fastidiosus	+	7.60E-01	0.000	0.000	0.000	0.001	0.001
2.6.1.62|g__Coprobacter.s__Coprobacter_secundus	-	7.17E-01	0.000	0.000	0.000	0.000	0.000
2.6.1.62|g__Desulfovibrio.s__Desulfovibrio_sp_AM18_2	+	8.19E-02	0.000	0.000	0.000	0.000	0.000
2.6.1.62|g__Enterobacter.s__Enterobacter_asburiae	-	1.60E-01	0.000	0.000	0.000	0.000	0.000
2.6.1.62|g__Enterobacter.s__Enterobacter_bugandensis	-	3.64E-01	0.000	0.000	0.000	0.000	0.000
2.6.1.62|g__Enterobacter.s__Enterobacter_cloacae	+	8.47E-01	0.000	0.000	0.000	0.001	0.003
2.6.1.62|g__Enterobacter.s__Enterobacter_mori	-	1.60E-01	0.000	0.000	0.000	0.000	0.000
2.6.1.62|g__Fusobacterium.s__Fusobacterium_mortiferum	-	6.89E-01	0.000	0.000	0.000	0.001	0.004
2.6.1.62|g__Fusobacterium.s__Fusobacterium_varium	-	8.19E-02	0.000	0.000	0.000	0.001	0.000
2.6.1.62|g__Haemophilus.s__Haemophilus_haemolyticus	+	3.30E-01	0.000	0.000	0.000	0.000	0.000
2.6.1.62|g__Haemophilus.s__Haemophilus_influenzae	+	1.00E-01	0.000	0.000	0.000	0.000	0.000
2.6.1.62|g__Haemophilus.s__Haemophilus_parainfluenzae	+	7.38E-01	0.000	0.000	0.000	0.001	0.003
2.6.1.62|g__Hungatella.s__Hungatella_effluvii	+	1.62E-03	0.000	0.000	0.000	0.000	0.000
2.6.1.62|g__Hungatella.s__Hungatella_hathewayi	+	8.35E-04	0.000	0.000	0.000	0.000	0.001
2.6.1.62|g__Klebsiella.s__Klebsiella_aerogenes	-	8.52E-01	0.000	0.000	0.000	0.003	0.002
2.6.1.62|g__Klebsiella.s__Klebsiella_grimontii	+	3.30E-01	0.000	0.000	0.000	0.000	0.000
2.6.1.62|g__Klebsiella.s__Klebsiella_michiganensis	+	1.00E+00	0.000	0.000	0.000	0.000	0.000
2.6.1.62|g__Kluyvera.s__Kluyvera_ascorbata	+	3.30E-01	0.000	0.000	0.000	0.000	0.000
2.6.1.62|g__Kluyvera.s__Kluyvera_cryocrescens	+	3.30E-01	0.000	0.000	0.000	0.000	0.000
2.6.1.62|g__Kluyvera.s__Kluyvera_georgiana	-	3.30E-01	0.000	0.000	0.000	0.000	0.000
2.6.1.62|g__Lachnoclostridium.s__Clostridium_saccharolyticum	+	3.30E-01	0.000	0.000	0.000	0.000	0.000
2.6.1.62|g__Lachnoclostridium.s__Clostridium_symbiosum	+	6.92E-01	0.000	0.000	0.000	0.001	0.001
2.6.1.62|g__Leclercia.s__Leclercia_adecarboxylata	-	9.83E-01	0.000	0.000	0.000	0.000	0.000
2.6.1.62|g__Lelliottia.s__Lelliottia_amnigena	-	3.89E-01	0.000	0.000	0.000	0.000	0.000
2.6.1.62|g__Lelliottia.s__Lelliottia_nimipressuralis	-	8.16E-01	0.000	0.000	0.000	0.001	0.002
2.6.1.62|g__Megamonas.s__Megamonas_funiformis	+	9.86E-01	0.000	0.000	0.000	0.037	0.028
2.6.1.62|g__Megamonas.s__Megamonas_hypermegale	+	9.14E-01	0.000	0.000	0.000	0.008	0.006
2.6.1.62|g__Megamonas.s__Megamonas_rupellensis	+	9.32E-01	0.000	0.000	0.000	0.006	0.004
2.6.1.62|g__Oxalobacter.s__Oxalobacter_formigenes	+	1.50E-02	0.000	0.000	0.000	0.000	0.001
2.6.1.62|g__Pantoea.s__Pantoea_dispersa	+	3.30E-01	0.000	0.000	0.000	0.000	0.000
2.6.1.62|g__Pantoea.s__Pantoea_sesami	-	7.82E-01	0.000	0.000	0.000	0.000	0.001
2.6.1.62|g__Parabacteroides.s__Parabacteroides_chinchillae	+	3.30E-01	0.000	0.000	0.000	0.000	0.000
2.6.1.62|g__Parabacteroides.s__Parabacteroides_goldsteinii	+	3.97E-02	0.000	0.000	0.000	0.000	0.003
2.6.1.62|g__Parabacteroides.s__Parabacteroides_gordonii	+	1.60E-01	0.000	0.000	0.000	0.000	0.001
2.6.1.62|g__Parabacteroides.s__Parabacteroides_johnsonii	+	6.92E-01	0.000	0.000	0.000	0.001	0.003
2.6.1.62|g__Paraprevotella.s__Paraprevotella_clara	-	8.92E-01	0.000	0.000	0.000	0.006	0.003
2.6.1.62|g__Paraprevotella.s__Paraprevotella_xylaniphila	-	3.30E-01	0.000	0.000	0.000	0.000	0.000
2.6.1.62|g__Pedobacter.s__Pedobacter_himalayensis	-	1.00E+00	0.000	0.000	0.000	0.000	0.000

	2.6.1.62|g__Phascolarctobacterium.s__Phascolarctobacterium_succinatutens	-	9.92E-01	0.000	0.000	0.000	0.015	0.011
	2.6.1.62|g__Proteus.s__Proteus_hauseri	-	3.30E-01	0.000	0.000	0.000	0.000	0.000
	2.6.1.62|g__Proteus.s__Proteus_mirabilis	-	5.69E-01	0.000	0.000	0.000	0.000	0.000
	2.6.1.62|g__Proteus.s__Proteus_penneri	-	3.30E-01	0.000	0.000	0.000	0.000	0.000
	2.6.1.62|g__Proteus.s__Proteus_vulgaris	-	5.89E-01	0.000	0.000	0.000	0.000	0.000
	2.6.1.62|g__Providencia.s__Providencia_rettgeri	+	3.30E-01	0.000	0.000	0.000	0.000	0.007
	2.6.1.62|g__Raoultella.s__Raoultella_ornithinolytica	+	5.89E-01	0.000	0.000	0.000	0.000	0.000
	2.6.1.62|g__Raoultella.s__Raoultella_planticola	+	3.08E-01	0.000	0.000	0.000	0.000	0.000
	2.6.1.62|g__Roseburia.s__Roseburia_intestinalis	-	9.74E-01	0.000	0.000	0.000	0.013	0.008
	2.6.1.62|g__Salmonella.s__Salmonella_enterica	+	5.69E-01	0.000	0.000	0.000	0.000	0.000
	2.6.1.62|g__Streptococcus.s__Streptococcus_pneumoniae	-	8.43E-01	0.000	0.000	0.000	0.000	0.000
	2.6.1.62|g__Veillonella.s__Veillonella_atypica	-	5.49E-01	0.000	0.000	0.000	0.003	0.001
	2.6.1.62|g__Veillonella.s__Veillonella_dispar	-	4.87E-01	0.000	0.000	0.000	0.001	0.001
	2.6.1.62|g__Veillonella.s__Veillonella_infantium	-	6.38E-01	0.000	0.000	0.000	0.001	0.001
	2.6.1.62|g__Veillonella.s__Veillonella_rogosae	-	8.13E-03	0.000	0.000	0.000	0.003	0.002
	2.6.1.62|g__Veillonella.s__Veillonella_tobetsuensis	-	2.36E-01	0.000	0.000	0.000	0.001	0.003
	2.6.1.62|g__Yokenella.s__Yokenella_regensburgei	-	5.30E-01	0.000	0.000	0.000	0.000	0.000
	2.6.1.62|g__Escherichia.s__Escherichia_fergusonii	+	6.00E-01	0.004	0.007	0.003	0.001	0.001
	2.6.1.62|g__Klebsiella.s__Klebsiella_variicola	+	3.35E-01	0.000	0.005	0.005	0.004	0.004
	2.6.1.62|g__Klebsiella.s__Klebsiella_oxytoca	+	6.00E-01	0.033	0.070	0.037	0.004	0.004
	2.6.1.62|g__Bacteroides.s__Bacteroides_fragilis	-	9.25E-01	0.096	0.164	0.068	0.022	0.022
	2.6.1.62|g__Klebsiella.s__Klebsiella_pneumoniae	+	3.39E-01	0.300	0.369	0.069	0.024	0.058
	2.6.1.62|g__Parabacteroides.s__Parabacteroides_distasonis	+	5.22E-01	0.825	0.902	0.077	0.019	0.032
	2.6.1.62|g__Blautia.s__Blautia_wexlerae	+	7.53E-01	0.137	0.256	0.119	0.012	0.018
	2.6.1.62|g__Odoribacter.s__Odoribacter_splanchnicus	+	1.43E-01	0.114	0.274	0.161	0.005	0.008
	2.6.1.62|g__Parabacteroides.s__Parabacteroides_merdae	+	2.94E-01	0.445	0.629	0.184	0.013	0.022
	2.6.1.62|g__Bacteroides.s__Bacteroides_caccae	+	3.37E-01	0.157	0.440	0.283	0.011	0.016
	2.6.1.62|g__Bacteroides.s__Bacteroides_stercoris	+	7.46E-01	0.379	0.682	0.303	0.052	0.065
	2.6.1.62|g__Escherichia.s__Escherichia_coli	+	2.58E-01	0.610	1.519	0.909	0.057	0.093
	2.6.1.62|unclassified	+	1.74E-01	2.223	3.139	0.916	0.043	0.056
	2.6.1.62|g__Bacteroides.s__Bacteroides_uniformis	+	4.12E-01	2.219	3.453	1.234	0.068	0.101
Taiwan	2.6.1.62	-	1.26E-01	74.578	69.167	-5.411	1.000	1.000
	2.6.1.62|g__Bacteroides.s__Bacteroides_vulgatus	-	3.76E-01	6.420	4.120	-2.300	0.116	0.103
	2.6.1.62|g__Bacteroides.s__Bacteroides_plebeius	-	4.32E-01	0.778	0.162	-0.616	0.106	0.067
	2.6.1.62|g__Bacteroides.s__Bacteroides_massiliensis	-	9.35E-01	0.667	0.277	-0.390	0.040	0.037
	2.6.1.62|g__Escherichia.s__Escherichia_coli	-	1.69E-01	0.422	0.130	-0.293	0.044	0.015
	2.6.1.62|g__Blautia.s__Blautia_wexlerae	-	8.27E-01	0.082	0.067	-0.015	0.002	0.003
	2.6.1.62|g__Klebsiella.s__Klebsiella_oxytoca	-	1.15E-01	0.008	0.000	-0.008	0.010	0.001
	2.6.1.62|g__Acidaminococcus.s__Acidaminococcus_intestini	-	9.08E-01	0.000	0.000	0.000	0.003	0.007
	2.6.1.62|g__Aeromonas.s__Aeromonas_caviae	-	2.85E-01	0.000	0.000	0.000	0.000	0.000
	2.6.1.62|g__Aeromonas.s__Aeromonas_hydrophila	-	2.85E-01	0.000	0.000	0.000	0.000	0.000
	2.6.1.62|g__Aggregatibacter.s__Aggregatibacter_aphrophilus	-	2.85E-01	0.000	0.000	0.000	0.000	0.000
	2.6.1.62|g__Aggregatibacter.s__Aggregatibacter_segnis	-	2.85E-01	0.000	0.000	0.000	0.000	0.000
	2.6.1.62|g__Akkermansia.s__Akkermansia_muciniphila	+	1.13E-01	0.000	0.000	0.000	0.009	0.017
	2.6.1.62|g__Alistipes.s__Alistipes_indistinctus	-	6.29E-01	0.000	0.000	0.000	0.003	0.010
	2.6.1.62|g__Bacteroides.s__Bacteroides_cellulosilyticus	-	7.36E-01	0.000	0.000	0.000	0.005	0.006
	2.6.1.62|g__Bacteroides.s__Bacteroides_clarus	-	9.05E-01	0.000	0.000	0.000	0.004	0.010
	2.6.1.62|g__Bacteroides.s__Bacteroides_coprocola	+	9.30E-01	0.000	0.000	0.000	0.058	0.042
	2.6.1.62|g__Bacteroides.s__Bacteroides_coprophilus	-	6.83E-01	0.000	0.000	0.000	0.020	0.010
	2.6.1.62|g__Bacteroides.s__Bacteroides_eggerthii	+	3.26E-01	0.000	0.000	0.000	0.010	0.012
	2.6.1.62|g__Bacteroides.s__Bacteroides_faecichinchillae	+	3.68E-01	0.000	0.000	0.000	0.000	0.000
	2.6.1.62|g__Bacteroides.s__Bacteroides_fluxus	-	9.26E-01	0.000	0.000	0.000	0.000	0.002
	2.6.1.62|g__Bacteroides.s__Bacteroides_fragilis	+	4.78E-01	0.000	0.000	0.000	0.012	0.016
	2.6.1.62|g__Bacteroides.s__Bacteroides_nordii	+	5.66E-01	0.000	0.000	0.000	0.001	0.002
	2.6.1.62|g__Bacteroides.s__Bacteroides_salyersiae	-	7.90E-01	0.000	0.000	0.000	0.008	0.005
	2.6.1.62|g__Bacteroides.s__Bacteroides_sartorii	+	7.98E-01	0.000	0.000	0.000	0.004	0.002
	2.6.1.62|g__Barnesiella.s__Barnesiella_intestinihominis	+	2.76E-02	0.000	0.000	0.000	0.007	0.019
	2.6.1.62|g__Blautia.s__Blautia_sp_AF19_10LB	-	9.74E-01	0.000	0.000	0.000	0.000	0.000
	2.6.1.62|g__Blautia.s__Ruminococcus_torques	+	1.11E-01	0.000	0.000	0.000	0.001	0.003
	2.6.1.62|g__Butyricimonas.s__Butyricimonas_synergistica	+	6.70E-01	0.000	0.000	0.000	0.000	0.000
	2.6.1.62|g__Candidatus_Gastranaerophilales_unclassified.s__Candidatus_Ga	+	3.68E-01	0.000	0.000	0.000	0.000	0.000
	2.6.1.62|g__Catenibacterium.s__Catenibacterium_mitsuokai	-	5.86E-01	0.000	0.000	0.000	0.005	0.008
	2.6.1.62|g__Citrobacter.s__Citrobacter_farmeri	-	2.85E-01	0.000	0.000	0.000	0.000	0.000
	2.6.1.62|g__Citrobacter.s__Citrobacter_freundii	-	2.76E-01	0.000	0.000	0.000	0.000	0.000
	2.6.1.62|g__Citrobacter.s__Citrobacter_koseri	+	6.86E-01	0.000	0.000	0.000	0.000	0.000
	2.6.1.62|g__Citrobacter.s__Citrobacter_portucalensis	-	8.72E-01	0.000	0.000	0.000	0.000	0.000
	2.6.1.62|g__Citrobacter.s__Citrobacter_youngae	+	4.19E-01	0.000	0.000	0.000	0.000	0.000
	2.6.1.62|g__Cloacibacillus.s__Cloacibacillus_porcorum	+	3.70E-02	0.000	0.000	0.000	0.000	0.000
	2.6.1.62|g__Coprobacter.s__Coprobacter_fastidiosus	+	5.09E-01	0.000	0.000	0.000	0.002	0.005
	2.6.1.62|g__Coprobacter.s__Coprobacter_secundus	-	8.00E-01	0.000	0.000	0.000	0.000	0.001
	2.6.1.62|g__Desulfovibrio.s__Desulfovibrio_sp_AM18_2	+	1.96E-01	0.000	0.000	0.000	0.000	0.000
	2.6.1.62|g__Enterobacter.s__Enterobacter_asburiae	-	1.25E-01	0.000	0.000	0.000	0.000	0.000

2.6.1.62|g__Enterobacter.s__Enterobacter_bugandensis	-	2.27E-01	0.000	0.000	0.000	0.000	0.000
2.6.1.62|g__Enterobacter.s__Enterobacter_cloacae	-	4.23E-01	0.000	0.000	0.000	0.000	0.000
2.6.1.62|g__Escherichia.s__Escherichia_fergusonii	-	6.28E-01	0.000	0.000	0.000	0.000	0.000
2.6.1.62|g__Fusobacterium.s__Fusobacterium_mortiferum	-	3.02E-01	0.000	0.000	0.000	0.006	0.002
2.6.1.62|g__Fusobacterium.s__Fusobacterium_varium	-	5.10E-01	0.000	0.000	0.000	0.002	0.000
2.6.1.62|g__Haemophilus.s__Haemophilus_influenzae	+	1.96E-01	0.000	0.000	0.000	0.000	0.000
2.6.1.62|g__Haemophilus.s__Haemophilus_parainfluenzae	-	7.43E-01	0.000	0.000	0.000	0.001	0.001
2.6.1.62|g__Hungatella.s__Hungatella_effluvii	+	6.26E-01	0.000	0.000	0.000	0.000	0.000
2.6.1.62|g__Hungatella.s__Hungatella_hathewayi	+	3.62E-01	0.000	0.000	0.000	0.000	0.001
2.6.1.62|g__Klebsiella.s__Klebsiella_aerogenes	-	5.34E-01	0.000	0.000	0.000	0.000	0.000
2.6.1.62|g__Klebsiella.s__Klebsiella_grimontii	-	9.26E-01	0.000	0.000	0.000	0.000	0.000
2.6.1.62|g__Klebsiella.s__Klebsiella_michiganensis	-	4.57E-01	0.000	0.000	0.000	0.000	0.000
2.6.1.62|g__Klebsiella.s__Klebsiella_pneumoniae	-	1.82E-01	0.000	0.000	0.000	0.017	0.008
2.6.1.62|g__Klebsiella.s__Klebsiella_variicola	+	9.39E-01	0.000	0.000	0.000	0.001	0.001
2.6.1.62|g__Kluyvera.s__Kluyvera_georgiana	-	2.85E-01	0.000	0.000	0.000	0.002	0.000
2.6.1.62|g__Lachnoclostridium.s__Clostridium_saccharolyticum	-	1.81E-01	0.000	0.000	0.000	0.001	0.001
2.6.1.62|g__Lachnoclostridium.s__Clostridium_symbiosum	+	9.03E-02	0.000	0.000	0.000	0.000	0.001
2.6.1.62|g__Leclercia.s__Leclercia_adecarboxylata	-	1.25E-01	0.000	0.000	0.000	0.000	0.000
2.6.1.62|g__Lelliottia.s__Lelliottia_amnigena	-	1.25E-01	0.000	0.000	0.000	0.000	0.000
2.6.1.62|g__Lelliottia.s__Lelliottia_nimipressuralis	-	3.01E-01	0.000	0.000	0.000	0.001	0.000
2.6.1.62|g__Megamonas.s__Megamonas_funiformis	+	9.64E-01	0.000	0.000	0.000	0.013	0.010
2.6.1.62|g__Megamonas.s__Megamonas_hypermegale	-	5.24E-01	0.000	0.000	0.000	0.002	0.002
2.6.1.62|g__Megamonas.s__Megamonas_rupellensis	-	4.92E-01	0.000	0.000	0.000	0.001	0.001
2.6.1.62|g__Metakosakonia.s__Kluyvera_intestini	+	3.68E-01	0.000	0.000	0.000	0.000	0.000
2.6.1.62|g__Neisseria.s__Neisseria_flavescens	-	2.85E-01	0.000	0.000	0.000	0.000	0.000
2.6.1.62|g__Neisseria.s__Neisseria_lactamica	-	2.85E-01	0.000	0.000	0.000	0.000	0.000
2.6.1.62|g__Neisseria.s__Neisseria_meningitidis	-	2.85E-01	0.000	0.000	0.000	0.000	0.000
2.6.1.62|g__Neisseria.s__Neisseria_mucosa	-	2.85E-01	0.000	0.000	0.000	0.000	0.000
2.6.1.62|g__Neisseria.s__Neisseria_perflava	-	2.85E-01	0.000	0.000	0.000	0.000	0.000
2.6.1.62|g__Odoribacter.s__Odoribacter_laneus	+	3.97E-01	0.000	0.000	0.000	0.000	0.004
2.6.1.62|g__Oxalobacter.s__Oxalobacter_formigenes	+	9.20E-01	0.000	0.000	0.000	0.000	0.001
2.6.1.62|g__Pantoea.s__Pantoea_sesami	-	5.12E-01	0.000	0.000	0.000	0.000	0.000
2.6.1.62|g__Parabacteroides.s__Parabacteroides_chinchillae	-	2.85E-01	0.000	0.000	0.000	0.000	0.000
2.6.1.62|g__Parabacteroides.s__Parabacteroides_goldsteinii	+	8.52E-02	0.000	0.000	0.000	0.001	0.005
2.6.1.62|g__Parabacteroides.s__Parabacteroides_gordonii	-	9.26E-01	0.000	0.000	0.000	0.000	0.000
2.6.1.62|g__Parabacteroides.s__Parabacteroides_johnsonii	+	9.35E-01	0.000	0.000	0.000	0.002	0.001
2.6.1.62|g__Paraprevotella.s__Paraprevotella_clara	-	5.87E-01	0.000	0.000	0.000	0.013	0.014
2.6.1.62|g__Paraprevotella.s__Paraprevotella_xylaniphila	+	8.65E-02	0.000	0.000	0.000	0.001	0.003
2.6.1.62|g__Pedobacter.s__Pedobacter_himalayensis	-	5.86E-02	0.000	0.000	0.000	0.000	0.000
2.6.1.62|g__Phascolarctobacterium.s__Phascolarctobacterium_succinatutens	+	2.88E-01	0.000	0.000	0.000	0.002	0.008
2.6.1.62|g__Phytobacter.s__Phytobacter_ursingii	+	3.68E-01	0.000	0.000	0.000	0.000	0.000
2.6.1.62|g__Plesiomonas.s__Plesiomonas_shigelloides	-	2.85E-01	0.000	0.000	0.000	0.001	0.000
2.6.1.62|g__Raoultella.s__Raoultella_ornithinolytica	-	2.34E-01	0.000	0.000	0.000	0.001	0.000
2.6.1.62|g__Raoultella.s__Raoultella_planticola	-	9.26E-01	0.000	0.000	0.000	0.000	0.000
2.6.1.62|g__Roseburia.s__Roseburia_intestinalis	-	8.51E-01	0.000	0.000	0.000	0.011	0.006
2.6.1.62|g__Salmonella.s__Salmonella_enterica	-	9.26E-01	0.000	0.000	0.000	0.000	0.000
2.6.1.62|g__Sanguibacteroides.s__Sanguibacteroides_justesenii	-	8.72E-01	0.000	0.000	0.000	0.000	0.000
2.6.1.62|g__Streptococcus.s__Streptococcus_pneumoniae	-	8.57E-01	0.000	0.000	0.000	0.000	0.000
2.6.1.62|g__Succinatimonas.s__Succinatimonas_hippei	+	4.69E-01	0.000	0.000	0.000	0.000	0.001
2.6.1.62|g__Veillonella.s__Veillonella_atypica	-	2.24E-01	0.000	0.000	0.000	0.001	0.000
2.6.1.62|g__Veillonella.s__Veillonella_dispar	-	5.30E-01	0.000	0.000	0.000	0.002	0.000
2.6.1.62|g__Veillonella.s__Veillonella_infantium	-	5.30E-01	0.000	0.000	0.000	0.001	0.000
2.6.1.62|g__Veillonella.s__Veillonella_parvula	-	2.93E-01	0.000	0.000	0.000	0.002	0.001
2.6.1.62|g__Veillonella.s__Veillonella_rogosae	-	1.03E-01	0.000	0.000	0.000	0.001	0.000
2.6.1.62|g__Veillonella.s__Veillonella_tobetsuensis	-	8.57E-01	0.000	0.000	0.000	0.000	0.000
2.6.1.62|g__Yokenella.s__Yokenella_regensburgei	-	2.83E-01	0.000	0.000	0.000	0.000	0.000
2.6.1.62|g__Bacteroides.s__Bacteroides_xylanisolvens	+	9.25E-01	0.372	0.384	0.012	0.021	0.020
2.6.1.62|unclassified	-	9.98E-01	2.891	2.907	0.017	0.063	0.075
2.6.1.62|g__Bacteroides.s__Bacteroides_finegoldii	+	3.05E-01	0.000	0.029	0.029	0.007	0.011
2.6.1.62|g__Blautia.s__Blautia_obeum	+	9.31E-01	0.049	0.077	0.029	0.002	0.003
2.6.1.62|g__Bacteroides.s__Bacteroides_ovatus	+	2.19E-01	0.401	0.501	0.099	0.018	0.023
2.6.1.62|g__Butyricimonas.s__Butyricimonas_virosa	+	3.32E-02	0.000	0.141	0.141	0.004	0.009
2.6.1.62|g__Bacteroides.s__Bacteroides_dorei	+	7.57E-01	0.935	1.096	0.162	0.037	0.050
2.6.1.62|g__Bacteroides.s__Bacteroides_stercoris	-	7.32E-01	1.795	1.978	0.183	0.083	0.066
2.6.1.62|g__Parabacteroides.s__Parabacteroides_distasonis	+	1.09E-01	1.074	1.262	0.188	0.021	0.035
2.6.1.62|g__Phascolarctobacterium.s__Phascolarctobacterium_faecium	+	6.13E-01	0.331	0.548	0.218	0.013	0.019
2.6.1.62|g__Odoribacter.s__Odoribacter_splanchnicus	+	4.43E-02	0.274	0.533	0.259	0.012	0.017
2.6.1.62|g__Bacteroides.s__Bacteroides_thetaiotaomicron	+	1.36E-01	0.831	1.122	0.291	0.024	0.033
2.6.1.62|g__Parabacteroides.s__Parabacteroides_merdae	+	1.91E-02	0.474	1.069	0.595	0.015	0.033
2.6.1.62|g__Bacteroides.s__Bacteroides_uniformis	-	9.68E-01	3.649	4.296	0.647	0.103	0.101
2.6.1.62|g__Bacteroides.s__Bacteroides_caccae	+	2.29E-02	0.325	1.402	1.076	0.019	0.028

Fractional CPM (median) Relative CPM (average)

Country	EC number|Bacteria	Increased or decreased in PD	*p* -value	Control	PD	PD - Control	Control	PD
Japan	2.8.1.6	-	6.54E-05	91.830	77.906	-13.924	1.000	1.000
	2.8.1.6|unclassified	-	4.34E-02	13.186	10.745	-2.442	0.157	0.157
	2.8.1.6|g__Blautia.s__Blautia_obeum	-	3.29E-04	5.875	3.604	-2.270	0.079	0.058
	2.8.1.6|g__Blautia.s__Ruminococcus_torques	-	6.52E-02	2.933	0.691	-2.242	0.050	0.042
	2.8.1.6|g__Blautia.s__Blautia_wexlerae	-	4.69E-05	3.888	1.795	-2.093	0.052	0.033
	2.8.1.6|g__Lachnospiraceae_unclassified.s__Eubacterium_rectale	-	7.46E-02	0.855	0.000	-0.855	0.032	0.024
	2.8.1.6|g__Clostridiales_unclassified.s__Clostridiales_bacterium_KLE1615	-	4.23E-04	0.850	0.000	-0.850	0.013	0.006
	2.8.1.6|g__Anaerostipes.s__Anaerostipes_hadrus	-	2.62E-01	3.152	2.813	-0.339	0.066	0.062
	2.8.1.6|g__Dorea.s__Dorea_formicigenerans	-	3.85E-01	0.313	0.000	-0.313	0.005	0.005
	2.8.1.6|g__Agathobaculum.s__Agathobaculum_butyriciproducens	-	1.23E-02	0.270	0.000	-0.270	0.004	0.003
	2.8.1.6|g__Blautia.s__Ruminococcus_gnavus	-	1.90E-01	0.402	0.208	-0.194	0.020	0.013
	2.8.1.6|g__Bacteroides.s__Bacteroides_vulgatus	-	5.53E-01	1.347	1.177	-0.170	0.047	0.041
	2.8.1.6|g__Acidaminococcus.s__Acidaminococcus_fermentans	+	3.85E-01	0.000	0.000	0.000	0.000	0.000
	2.8.1.6|g__Acidaminococcus.s__Acidaminococcus_intestini	-	9.93E-01	0.000	0.000	0.000	0.014	0.004
	2.8.1.6|g__Akkermansia.s__Akkermansia_muciniphila	+	3.70E-03	0.000	0.000	0.000	0.006	0.037
	2.8.1.6|g__Alistipes.s__Alistipes_indistinctus	+	7.17E-02	0.000	0.000	0.000	0.000	0.002
	2.8.1.6|g__Allisonella.s__Allisonella_histaminiformans	-	1.09E-01	0.000	0.000	0.000	0.000	0.000
	2.8.1.6|g__Anaerofustis.s__Anaerofustis_stercorihominis	+	3.85E-01	0.000	0.000	0.000	0.000	0.000
	2.8.1.6|g__Anaeromassilibacillus.s__Anaeromassilibacillus_sp_An172	+	2.14E-01	0.000	0.000	0.000	0.000	0.000
	2.8.1.6|g__Anaerostipes.s__Anaerostipes_caccae	-	6.30E-01	0.000	0.000	0.000	0.001	0.001
	2.8.1.6|g__Anaerotignum.s__Anaerotignum_lactatifermentans	+	1.89E-03	0.000	0.000	0.000	0.000	0.003
	2.8.1.6|g__Anaerotruncus.s__Anaerotruncus_colihominis	+	4.67E-02	0.000	0.000	0.000	0.000	0.000
	2.8.1.6|g__Bacillus.s__Bacillus_gibsonii	+	6.34E-01	0.000	0.000	0.000	0.000	0.001
	2.8.1.6|g__Bacillus.s__Bacillus_murimartini	+	7.40E-01	0.000	0.000	0.000	0.000	0.000
	2.8.1.6|g__Bacteroides.s__Bacteroides_caccae	+	7.00E-02	0.000	0.000	0.000	0.004	0.008
	2.8.1.6|g__Bacteroides.s__Bacteroides_cellulosilyticus	+	7.25E-02	0.000	0.000	0.000	0.006	0.010
	2.8.1.6|g__Bacteroides.s__Bacteroides_clarus	+	5.62E-02	0.000	0.000	0.000	0.000	0.004
	2.8.1.6|g__Bacteroides.s__Bacteroides_coprocola	+	2.53E-01	0.000	0.000	0.000	0.013	0.017
	2.8.1.6|g__Bacteroides.s__Bacteroides_coprophilus	+	4.41E-01	0.000	0.000	0.000	0.001	0.001
	2.8.1.6|g__Bacteroides.s__Bacteroides_eggerthii	+	1.80E-01	0.000	0.000	0.000	0.003	0.011
	2.8.1.6|g__Bacteroides.s__Bacteroides_faecis	-	2.60E-01	0.000	0.000	0.000	0.000	0.000
	2.8.1.6|g__Bacteroides.s__Bacteroides_finegoldii	+	1.94E-01	0.000	0.000	0.000	0.002	0.003
	2.8.1.6|g__Bacteroides.s__Bacteroides_fluxus	-	2.62E-01	0.000	0.000	0.000	0.000	0.000
	2.8.1.6|g__Bacteroides.s__Bacteroides_fragilis	+	7.84E-01	0.000	0.000	0.000	0.005	0.009
	2.8.1.6|g__Bacteroides.s__Bacteroides_intestinalis	+	7.08E-01	0.000	0.000	0.000	0.002	0.002
	2.8.1.6|g__Bacteroides.s__Bacteroides_massiliensis	+	2.34E-01	0.000	0.000	0.000	0.003	0.005
	2.8.1.6|g__Bacteroides.s__Bacteroides_nordii	+	5.86E-01	0.000	0.000	0.000	0.000	0.001
	2.8.1.6|g__Bacteroides.s__Bacteroides_plebeius	+	4.33E-01	0.000	0.000	0.000	0.021	0.019
	2.8.1.6|g__Bacteroides.s__Bacteroides_salyersiae	+	1.75E-01	0.000	0.000	0.000	0.000	0.001
	2.8.1.6|g__Barnesiella.s__Barnesiella_intestinihominis	+	1.94E-02	0.000	0.000	0.000	0.003	0.009
	2.8.1.6|g__Barnesiella.s__Barnesiella_sp_An22	-	2.62E-01	0.000	0.000	0.000	0.000	0.000
	2.8.1.6|g__Bilophila.s__Bilophila_wadsworthia	+	3.75E-02	0.000	0.000	0.000	0.002	0.003
	2.8.1.6|g__Blautia.s__Blautia_coccoides	-	4.24E-01	0.000	0.000	0.000	0.000	0.000
	2.8.1.6|g__Blautia.s__Blautia_producta	+	4.91E-01	0.000	0.000	0.000	0.000	0.001
	2.8.1.6|g__Blautia.s__Blautia_sp_AF19_10LB	+	6.09E-01	0.000	0.000	0.000	0.000	0.002
	2.8.1.6|g__Blautia.s__Blautia_sp_An249	+	2.14E-01	0.000	0.000	0.000	0.000	0.000
	2.8.1.6|g__Butyricicoccus.s__Butyricicoccus_pullicaecorum	-	8.57E-01	0.000	0.000	0.000	0.000	0.000
	2.8.1.6|g__Butyricimonas.s__Butyricimonas_virosa	+	1.65E-01	0.000	0.000	0.000	0.000	0.001
	2.8.1.6|g__Butyrivibrio.s__Butyrivibrio_crossotus	+	3.85E-01	0.000	0.000	0.000	0.000	0.000
	2.8.1.6|g__Campylobacter.s__Campylobacter_concisus	-	2.62E-01	0.000	0.000	0.000	0.000	0.000
	2.8.1.6|g__Candidatus_Gastranaerophilales_unclassified.s__Candidatus_Ga	-	8.57E-01	0.000	0.000	0.000	0.000	0.000
	2.8.1.6|g__Catabacter.s__Catabacter_hongkongensis	+	2.14E-01	0.000	0.000	0.000	0.000	0.000
	2.8.1.6|g__Catenibacterium.s__Catenibacterium_mitsuokai	-	8.54E-03	0.000	0.000	0.000	0.002	0.000
	2.8.1.6|g__Cellulosilyticum.s__Cellulosilyticum_lentocellum	+	3.85E-01	0.000	0.000	0.000	0.000	0.000
	2.8.1.6|g__Cetobacterium.s__Cetobacterium_somerae	-	2.62E-01	0.000	0.000	0.000	0.000	0.000
	2.8.1.6|g__Christensenella.s__Christensenella_minuta	+	3.85E-01	0.000	0.000	0.000	0.000	0.000
	2.8.1.6|g__Citrobacter.s__Citrobacter_braakii	+	3.85E-01	0.000	0.000	0.000	0.000	0.000
	2.8.1.6|g__Citrobacter.s__Citrobacter_freundii	+	1.78E-01	0.000	0.000	0.000	0.000	0.002
	2.8.1.6|g__Citrobacter.s__Citrobacter_koseri	-	8.57E-01	0.000	0.000	0.000	0.000	0.000
	2.8.1.6|g__Citrobacter.s__Citrobacter_portucalensis	+	2.72E-01	0.000	0.000	0.000	0.000	0.000
	2.8.1.6|g__Citrobacter.s__Citrobacter_werkmanii	+	3.85E-01	0.000	0.000	0.000	0.000	0.002
	2.8.1.6|g__Citrobacter.s__Citrobacter_youngae	+	1.75E-01	0.000	0.000	0.000	0.000	0.000
	2.8.1.6|g__Cloacibacillus.s__Cloacibacillus_porcorum	+	1.26E-01	0.000	0.000	0.000	0.000	0.001
	2.8.1.6|g__Clostridioides.s__Clostridioides_difficile	+	3.85E-01	0.000	0.000	0.000	0.000	0.000
	2.8.1.6|g__Clostridium.s__Butyribacterium_methylotrophicum	+	2.59E-01	0.000	0.000	0.000	0.000	0.001
	2.8.1.6|g__Clostridium.s__Clostridium_disporicum	-	7.03E-01	0.000	0.000	0.000	0.002	0.001
	2.8.1.6|g__Clostridium.s__Clostridium_perfringens	-	9.89E-02	0.000	0.000	0.000	0.001	0.000
	2.8.1.6|g__Clostridium.s__Clostridium_sp_AF36_4	-	8.25E-01	0.000	0.000	0.000	0.001	0.001
	2.8.1.6|g__Clostridium.s__Clostridium_sp_D5	+	3.85E-01	0.000	0.000	0.000	0.000	0.000
	2.8.1.6|g__Coprobacter.s__Coprobacter_fastidiosus	+	2.72E-01	0.000	0.000	0.000	0.001	0.002
	2.8.1.6|g__Coprobacter.s__Coprobacter_secundus	+	7.10E-01	0.000	0.000	0.000	0.000	0.001

2.8.1.6|g__Coprococcus.s__Coprococcus_comes	-	1.69E-02	0.000	0.000	0.000	0.009	0.005
2.8.1.6|g__Coprococcus.s__Coprococcus_eutactus	-	2.89E-01	0.000	0.000	0.000	0.003	0.002
2.8.1.6|g__Corynebacterium.s__Corynebacterium_argentoratense	+	3.85E-01	0.000	0.000	0.000	0.000	0.000
2.8.1.6|g__Corynebacterium.s__Corynebacterium_striatum	+	3.85E-01	0.000	0.000	0.000	0.000	0.000
2.8.1.6|g__Desulfovibrio.s__Desulfovibrio_desulfuricans	+	1.78E-02	0.000	0.000	0.000	0.000	0.001
2.8.1.6|g__Desulfovibrio.s__Desulfovibrio_fairfieldensis	+	1.26E-01	0.000	0.000	0.000	0.000	0.000
2.8.1.6|g__Desulfovibrio.s__Desulfovibrio_piger	+	2.92E-01	0.000	0.000	0.000	0.000	0.000
2.8.1.6|g__Desulfovibrio.s__Desulfovibrio_sp_AM18_2	+	3.85E-01	0.000	0.000	0.000	0.000	0.000
2.8.1.6|g__Desulfovibrionaceae_unclassified.s__Desulfovibrionaceae_bacte	+	3.85E-01	0.000	0.000	0.000	0.000	0.000
2.8.1.6|g__Dialister.s__Dialister_invisus	-	3.06E-01	0.000	0.000	0.000	0.007	0.003
2.8.1.6|g__Dialister.s__Dialister_succinatiphilus	+	7.10E-01	0.000	0.000	0.000	0.001	0.002
2.8.1.6|g__Dorea.s__Dorea_longicatena	-	5.25E-01	0.000	0.000	0.000	0.007	0.005
2.8.1.6|g__Eisenbergiella.s__Eisenbergiella_tayi	+	4.46E-01	0.000	0.000	0.000	0.000	0.001
2.8.1.6|g__Enterobacter.s__Enterobacter_bugandensis	-	4.16E-01	0.000	0.000	0.000	0.000	0.000
2.8.1.6|g__Enterobacter.s__Enterobacter_cloacae	+	3.85E-01	0.000	0.000	0.000	0.000	0.000
2.8.1.6|g__Enterobacter.s__Enterobacter_mori	+	1.26E-01	0.000	0.000	0.000	0.000	0.001
2.8.1.6|g__Erysipelatoclostridium.s__Clostridium_spiroforme	+	4.22E-01	0.000	0.000	0.000	0.001	0.001
2.8.1.6|g__Erysipelatoclostridium.s__Erysipelatoclostridium_ramosum	+	3.93E-01	0.000	0.000	0.000	0.007	0.007
2.8.1.6|g__Escherichia.s__Escherichia_coli	+	5.01E-01	0.000	0.000	0.000	0.021	0.012
2.8.1.6|g__Escherichia.s__Escherichia_fergusonii	-	8.71E-01	0.000	0.000	0.000	0.000	0.000
2.8.1.6|g__Eubacterium.s__Eubacterium_callanderi	+	2.59E-01	0.000	0.000	0.000	0.000	0.001
2.8.1.6|g__Eubacterium.s__Eubacterium_eligens	+	2.20E-01	0.000	0.000	0.000	0.010	0.020
2.8.1.6|g__Eubacterium.s__Eubacterium_limosum	-	8.22E-01	0.000	0.000	0.000	0.000	0.000
2.8.1.6|g__Eubacterium.s__Eubacterium_maltosivorans	+	3.85E-01	0.000	0.000	0.000	0.000	0.000
2.8.1.6|g__Eubacterium.s__Eubacterium_ramulus	+	4.31E-01	0.000	0.000	0.000	0.003	0.004
2.8.1.6|g__Eubacterium.s__Eubacterium_sp_AF17_7	+	1.59E-01	0.000	0.000	0.000	0.001	0.002
2.8.1.6|g__Faecalicatena.s__Faecalicatena_contorta	-	8.57E-01	0.000	0.000	0.000	0.000	0.000
2.8.1.6|g__Firmicutes_unclassified.s__Firmicutes_bacterium_AM10_47	+	5.08E-01	0.000	0.000	0.000	0.003	0.002
2.8.1.6|g__Fusobacterium.s__Fusobacterium_mortiferum	-	2.12E-01	0.000	0.000	0.000	0.000	0.000
2.8.1.6|g__Fusobacterium.s__Fusobacterium_nucleatum	-	2.62E-01	0.000	0.000	0.000	0.000	0.000
2.8.1.6|g__Fusobacterium.s__Fusobacterium_ulcerans	+	7.31E-01	0.000	0.000	0.000	0.000	0.000
2.8.1.6|g__Gemmiger.s__Gemmiger_sp_An50	+	3.85E-01	0.000	0.000	0.000	0.000	0.001
2.8.1.6|g__Gordonibacter.s__Gordonibacter_pamelaeae	+	3.98E-02	0.000	0.000	0.000	0.000	0.001
2.8.1.6|g__Haemophilus.s__Haemophilus_parainfluenzae	-	9.95E-03	0.000	0.000	0.000	0.002	0.001
2.8.1.6|g__Hungatella.s__Hungatella_effluvii	+	7.63E-02	0.000	0.000	0.000	0.000	0.000
2.8.1.6|g__Hungatella.s__Hungatella_hathewayi	+	7.41E-02	0.000	0.000	0.000	0.000	0.001
2.8.1.6|g__Klebsiella.s__Klebsiella_aerogenes	+	1.85E-01	0.000	0.000	0.000	0.002	0.000
2.8.1.6|g__Klebsiella.s__Klebsiella_grimontii	-	8.57E-01	0.000	0.000	0.000	0.000	0.000
2.8.1.6|g__Klebsiella.s__Klebsiella_michiganensis	-	8.03E-01	0.000	0.000	0.000	0.000	0.000
2.8.1.6|g__Klebsiella.s__Klebsiella_oxytoca	-	9.54E-01	0.000	0.000	0.000	0.002	0.000
2.8.1.6|g__Klebsiella.s__Klebsiella_pneumoniae	+	8.18E-01	0.000	0.000	0.000	0.002	0.009
2.8.1.6|g__Klebsiella.s__Klebsiella_variicola	-	8.65E-02	0.000	0.000	0.000	0.001	0.000
2.8.1.6|g__Kluyvera.s__Kluyvera_cryocrescens	+	1.00E+00	0.000	0.000	0.000	0.000	0.000
2.8.1.6|g__Lachnoclostridium.s__Clostridium_citroniae	+	1.26E-01	0.000	0.000	0.000	0.000	0.000
2.8.1.6|g__Lachnoclostridium.s__Clostridium_saccharolyticum	-	9.41E-01	0.000	0.000	0.000	0.001	0.001
2.8.1.6|g__Lachnoclostridium.s__Clostridium_scindens	+	7.45E-01	0.000	0.000	0.000	0.001	0.003
2.8.1.6|g__Lachnoclostridium.s__Clostridium_symbiosum	+	3.04E-01	0.000	0.000	0.000	0.000	0.000
2.8.1.6|g__Lachnoclostridium.s__Lachnoclostridium_sp_An14	+	3.85E-01	0.000	0.000	0.000	0.000	0.000
2.8.1.6|g__Lachnospira.s__Lachnospira_pectinoschiza	-	4.91E-01	0.000	0.000	0.000	0.005	0.004
2.8.1.6|g__Lactobacillus.s__Lactobacillus_rogosae	-	2.99E-01	0.000	0.000	0.000	0.003	0.002
2.8.1.6|g__Lawsonibacter.s__Lawsonibacter_asaccharolyticus	+	3.80E-01	0.000	0.000	0.000	0.002	0.002
2.8.1.6|g__Leclercia.s__Leclercia_adecarboxylata	-	8.57E-01	0.000	0.000	0.000	0.000	0.000
2.8.1.6|g__Lelliottia.s__Lelliottia_nimipressuralis	-	7.93E-01	0.000	0.000	0.000	0.000	0.000
2.8.1.6|g__Megamonas.s__Megamonas_funiformis	-	7.30E-01	0.000	0.000	0.000	0.011	0.007
2.8.1.6|g__Megamonas.s__Megamonas_hypermegale	+	9.37E-01	0.000	0.000	0.000	0.003	0.003
2.8.1.6|g__Megamonas.s__Megamonas_rupellensis	-	8.10E-01	0.000	0.000	0.000	0.006	0.006
2.8.1.6|g__Megasphaera.s__Megasphaera_elsdenii	-	8.71E-01	0.000	0.000	0.000	0.000	0.000
2.8.1.6|g__Megasphaera.s__Megasphaera_hexanoica	+	3.85E-01	0.000	0.000	0.000	0.000	0.000
2.8.1.6|g__Megasphaera.s__Megasphaera_sp_DISK_18	-	4.59E-02	0.000	0.000	0.000	0.004	0.001
2.8.1.6|g__Megasphaera.s__Megasphaera_sp_MJR8396C	+	8.78E-01	0.000	0.000	0.000	0.002	0.001
2.8.1.6|g__Megasphaera.s__Megasphaera_stantonii	+	7.21E-01	0.000	0.000	0.000	0.000	0.000
2.8.1.6|g__Metakosakonia.s__Kluyvera_intestini	+	3.85E-01	0.000	0.000	0.000	0.000	0.000
2.8.1.6|g__Mitsuokella.s__Mitsuokella_jalaludinii	+	3.85E-01	0.000	0.000	0.000	0.000	0.000
2.8.1.6|g__Mitsuokella.s__Mitsuokella_multacida	+	3.85E-01	0.000	0.000	0.000	0.000	0.000
2.8.1.6|g__Odoribacter.s__Odoribacter_laneus	+	5.02E-01	0.000	0.000	0.000	0.000	0.001
2.8.1.6|g__Odoribacter.s__Odoribacter_splanchnicus	+	1.60E-02	0.000	0.000	0.000	0.002	0.003
2.8.1.6|g__Paeniclostridium.s__Paeniclostridium_sordellii	-	2.62E-01	0.000	0.000	0.000	0.000	0.000
2.8.1.6|g__Pantoea.s__Pantoea_sesami	-	8.03E-01	0.000	0.000	0.000	0.000	0.000
2.8.1.6|g__Parabacteroides.s__Parabacteroides_goldsteinii	+	2.62E-03	0.000	0.000	0.000	0.000	0.001
2.8.1.6|g__Parabacteroides.s__Parabacteroides_gordonii	-	2.62E-01	0.000	0.000	0.000	0.000	0.000
2.8.1.6|g__Parabacteroides.s__Parabacteroides_johnsonii	+	7.58E-01	0.000	0.000	0.000	0.001	0.001
2.8.1.6|g__Paraeggerthella.s__Paraeggerthella_hongkongensis	+	2.14E-01	0.000	0.000	0.000	0.000	0.000

	2.8.1.6|g__Paraprevotella.s__Paraprevotella_clara	+	1.50E-01	0.000	0.000	0.000	0.001	0.002
	2.8.1.6|g__Paraprevotella.s__Paraprevotella_xylaniphila	+	2.87E-01	0.000	0.000	0.000	0.000	0.000
	2.8.1.6|g__Phascolarctobacterium.s__Phascolarctobacterium_faecium	+	4.08E-01	0.000	0.000	0.000	0.004	0.009
	2.8.1.6|g__Phascolarctobacterium.s__Phascolarctobacterium_succinatutens	-	5.39E-01	0.000	0.000	0.000	0.011	0.009
	2.8.1.6|g__Pseudoflavonifractor.s__Pseudoflavonifractor_sp_An184	+	5.77E-03	0.000	0.000	0.000	0.001	0.005
	2.8.1.6|g__Pseudoramibacter.s__Pseudoramibacter_alactolyticus	-	2.62E-01	0.000	0.000	0.000	0.000	0.000
	2.8.1.6|g__Raoultella.s__Raoultella_ornithinolytica	-	1.29E-01	0.000	0.000	0.000	0.004	0.000
	2.8.1.6|g__Raoultella.s__Raoultella_planticola	-	7.22E-01	0.000	0.000	0.000	0.000	0.000
	2.8.1.6|g__Rikenella.s__Rikenella_microfusus	+	1.26E-01	0.000	0.000	0.000	0.000	0.001
	2.8.1.6|g__Roseburia.s__Roseburia_faecis	-	1.97E-04	0.000	0.000	0.000	0.020	0.006
	2.8.1.6|g__Roseburia.s__Roseburia_intestinalis	-	1.26E-02	0.000	0.000	0.000	0.021	0.004
	2.8.1.6|g__Roseburia.s__Roseburia_inulinivorans	+	7.50E-01	0.000	0.000	0.000	0.005	0.006
	2.8.1.6|g__Ruminococcaceae_unclassified.s__Eubacterium_siraeum	+	7.07E-02	0.000	0.000	0.000	0.003	0.007
	2.8.1.6|g__Ruminococcaceae_unclassified.s__Ruminococcaceae_bacterium_	+	7.53E-02	0.000	0.000	0.000	0.000	0.001
	2.8.1.6|g__Ruminococcaceae_unclassified.s__Ruminococcaceae_bacterium_	-	8.03E-01	0.000	0.000	0.000	0.000	0.000
	2.8.1.6|g__Ruminococcus.s__Ruminococcus_lactaris	-	1.69E-01	0.000	0.000	0.000	0.003	0.002
	2.8.1.6|g__Saccharomyces.s__Saccharomyces_cerevisiae	-	2.62E-01	0.000	0.000	0.000	0.000	0.000
	2.8.1.6|g__Salmonella.s__Salmonella_enterica	+	3.85E-01	0.000	0.000	0.000	0.000	0.000
	2.8.1.6|g__Sanguibacteroides.s__Sanguibacteroides_justesenii	+	3.85E-01	0.000	0.000	0.000	0.000	0.000
	2.8.1.6|g__Staphylococcus.s__Staphylococcus_aureus	+	3.85E-01	0.000	0.000	0.000	0.000	0.000
	2.8.1.6|g__Streptococcus.s__Streptococcus_equinus	-	7.37E-01	0.000	0.000	0.000	0.000	0.000
	2.8.1.6|g__Streptococcus.s__Streptococcus_gallolyticus	-	7.37E-01	0.000	0.000	0.000	0.000	0.001
	2.8.1.6|g__Streptococcus.s__Streptococcus_pasteurianus	-	7.37E-01	0.000	0.000	0.000	0.000	0.000
	2.8.1.6|g__Streptococcus.s__Streptococcus_pneumoniae	+	2.11E-01	0.000	0.000	0.000	0.000	0.000
	2.8.1.6|g__Succinatimonas.s__Succinatimonas_hippei	+	3.85E-01	0.000	0.000	0.000	0.000	0.000
	2.8.1.6|g__Sutterella.s__Sutterella_parvirubra	-	8.71E-01	0.000	0.000	0.000	0.000	0.000
	2.8.1.6|g__Sutterella.s__Sutterella_wadsworthensis	-	4.36E-01	0.000	0.000	0.000	0.005	0.004
	2.8.1.6|g__Terrisporobacter.s__Terrisporobacter_othiniensis	+	3.85E-01	0.000	0.000	0.000	0.000	0.000
	2.8.1.6|g__Veillonella.s__Veillonella_atypica	-	2.51E-02	0.000	0.000	0.000	0.003	0.000
	2.8.1.6|g__Veillonella.s__Veillonella_denticariosi	+	7.21E-01	0.000	0.000	0.000	0.000	0.000
	2.8.1.6|g__Veillonella.s__Veillonella_dispar	-	2.09E-03	0.000	0.000	0.000	0.003	0.000
	2.8.1.6|g__Veillonella.s__Veillonella_infantium	-	1.55E-02	0.000	0.000	0.000	0.002	0.000
	2.8.1.6|g__Veillonella.s__Veillonella_parvula	-	2.32E-01	0.000	0.000	0.000	0.002	0.001
	2.8.1.6|g__Veillonella.s__Veillonella_rogosae	-	4.95E-04	0.000	0.000	0.000	0.002	0.000
	2.8.1.6|g__Veillonella.s__Veillonella_tobetsuensis	-	1.09E-01	0.000	0.000	0.000	0.000	0.000
	2.8.1.6|g__Victivallales_unclassified.s__Victivallales_bacterium_CCUG_44	-	8.57E-01	0.000	0.000	0.000	0.000	0.000
	2.8.1.6|g__Victivallis.s__Victivallis_vadensis	-	8.71E-01	0.000	0.000	0.000	0.000	0.000
	2.8.1.6|g__Yokenella.s__Yokenella_regensburgei	-	8.12E-01	0.000	0.000	0.000	0.000	0.000
	2.8.1.6|g__Bacteroides.s__Bacteroides_stercoris	+	9.29E-01	0.000	0.017	0.017	0.029	0.026
	2.8.1.6|g__Bacteroides.s__Bacteroides_ovatus	+	4.81E-01	0.293	0.318	0.026	0.011	0.015
	2.8.1.6|g__Bacteroides.s__Bacteroides_xylanisolvens	+	9.81E-01	0.033	0.061	0.027	0.008	0.008
	2.8.1.6|g__Bacteroides.s__Bacteroides_thetaiotaomicron	+	1.68E-01	0.200	0.374	0.174	0.006	0.012
	2.8.1.6|g__Bacteroides.s__Bacteroides_dorei	+	8.17E-01	0.463	0.697	0.234	0.032	0.033
	2.8.1.6|g__Parabacteroides.s__Parabacteroides_merdae	+	1.51E-01	0.313	0.596	0.283	0.010	0.012
	2.8.1.6|g__Parabacteroides.s__Parabacteroides_distasonis	+	2.24E-02	0.431	0.987	0.556	0.012	0.019
	2.8.1.6|g__Bacteroides.s__Bacteroides_uniformis	+	3.93E-01	3.477	4.063	0.586	0.053	0.077
USA	2.8.1.6	-	1.23E-04	105.836	96.955	-8.882	1.000	1.000
	2.8.1.6|g__Blautia.s__Blautia_wexlerae	-	6.09E-09	4.673	2.261	-2.412	0.061	0.043
	2.8.1.6|g__Bacteroides.s__Bacteroides_vulgatus	-	1.23E-01	7.893	5.581	-2.313	0.091	0.086
	2.8.1.6|g__Blautia.s__Ruminococcus_torques	-	4.78E-03	2.637	1.320	-1.317	0.046	0.036
	2.8.1.6|g__Blautia.s__Blautia_obeum	-	2.61E-05	6.213	4.939	-1.274	0.077	0.061
	2.8.1.6|g__Bacteroides.s__Bacteroides_uniformis	-	7.00E-01	7.608	6.638	-0.970	0.091	0.098
	2.8.1.6|g__Anaerostipes.s__Anaerostipes_hadrus	-	2.48E-04	1.307	0.626	-0.681	0.024	0.020
	2.8.1.6|g__Roseburia.s__Roseburia_intestinalis	-	5.37E-07	0.599	0.000	-0.599	0.021	0.010
	2.8.1.6|g__Lachnospiraceae_unclassified.s__Eubacterium_rectale	-	5.02E-03	0.820	0.294	-0.526	0.024	0.018
	2.8.1.6|g__Roseburia.s__Roseburia_faecis	-	1.21E-03	0.224	0.000	-0.224	0.019	0.013
	2.8.1.6|g__Dorea.s__Dorea_longicatena	-	2.26E-02	0.198	0.000	-0.198	0.008	0.006
	2.8.1.6|g__Lawsonibacter.s__Lawsonibacter_asaccharolyticus	-	3.18E-02	0.291	0.181	-0.110	0.004	0.003
	2.8.1.6|g__Bacteroides.s__Bacteroides_ovatus	+	5.70E-01	0.664	0.650	-0.014	0.018	0.017
	2.8.1.6|g__Acidaminococcus.s__Acidaminococcus_fermentans	+	2.32E-01	0.000	0.000	0.000	0.000	0.000
	2.8.1.6|g__Acidaminococcus.s__Acidaminococcus_intestini	+	1.28E-03	0.000	0.000	0.000	0.004	0.009
	2.8.1.6|g__Acidipropionibacterium.s__Acidipropionibacterium_acidipropior	+	4.92E-01	0.000	0.000	0.000	0.000	0.000
	2.8.1.6|g__Aeromonas.s__Aeromonas_allosaccharophila	-	1.48E-01	0.000	0.000	0.000	0.000	0.000
	2.8.1.6|g__Aeromonas.s__Aeromonas_caviae	+	4.92E-01	0.000	0.000	0.000	0.000	0.000
	2.8.1.6|g__Aeromonas.s__Aeromonas_diversa	-	1.48E-01	0.000	0.000	0.000	0.000	0.000
	2.8.1.6|g__Aeromonas.s__Aeromonas_veronii	-	1.48E-01	0.000	0.000	0.000	0.000	0.000
	2.8.1.6|g__Agathobaculum.s__Agathobaculum_butyriciproducens	-	5.64E-03	0.000	0.000	0.000	0.004	0.003
	2.8.1.6|g__Akkermansia.s__Akkermansia_muciniphila	+	2.80E-01	0.000	0.000	0.000	0.024	0.044
	2.8.1.6|g__Alistipes.s__Alistipes_indistinctus	+	7.87E-04	0.000	0.000	0.000	0.000	0.001
	2.8.1.6|g__Allisonella.s__Allisonella_histaminiformans	+	4.74E-01	0.000	0.000	0.000	0.000	0.000
	2.8.1.6|g__Anaerobiospirillum.s__Anaerobiospirillum_thomasii	+	4.92E-01	0.000	0.000	0.000	0.000	0.000
	2.8.1.6|g__Anaerococcus.s__Anaerococcus_hydrogenalis	+	4.92E-01	0.000	0.000	0.000	0.000	0.000

2.8.1.6|g__Anaerococcus.s__Anaerococcus_nagyae	+	4.92E-01	0.000	0.000	0.000	0.000	0.000
2.8.1.6|g__Anaerococcus.s__Anaerococcus_octavius	+	4.92E-01	0.000	0.000	0.000	0.000	0.000
2.8.1.6|g__Anaerococcus.s__Anaerococcus_vaginalis	+	2.32E-01	0.000	0.000	0.000	0.000	0.000
2.8.1.6|g__Anaerofilum.s__Anaerofilum_sp_An201	-	5.92E-01	0.000	0.000	0.000	0.000	0.000
2.8.1.6|g__Anaerofustis.s__Anaerofustis_stercorihominis	+	7.57E-01	0.000	0.000	0.000	0.000	0.000
2.8.1.6|g__Anaeroglobus.s__Anaeroglobus_geminatus	-	7.49E-01	0.000	0.000	0.000	0.000	0.000
2.8.1.6|g__Anaeromassilibacillus.s__Anaeromassilibacillus_sp_An172	-	5.92E-01	0.000	0.000	0.000	0.000	0.000
2.8.1.6|g__Anaerostipes.s__Anaerostipes_caccae	-	8.93E-01	0.000	0.000	0.000	0.001	0.001
2.8.1.6|g__Anaerostipes.s__Anaerostipes_sp_494a	-	1.48E-01	0.000	0.000	0.000	0.000	0.000
2.8.1.6|g__Anaerotignum.s__Anaerotignum_lactatifermentans	-	2.69E-01	0.000	0.000	0.000	0.001	0.001
2.8.1.6|g__Anaerotruncus.s__Anaerotruncus_colihominis	+	8.08E-04	0.000	0.000	0.000	0.000	0.000
2.8.1.6|g__Bacteroides.s__Bacteroides_clarus	+	6.83E-01	0.000	0.000	0.000	0.001	0.001
2.8.1.6|g__Bacteroides.s__Bacteroides_coprocola	-	1.06E-01	0.000	0.000	0.000	0.006	0.003
2.8.1.6|g__Bacteroides.s__Bacteroides_coprophilus	+	4.06E-01	0.000	0.000	0.000	0.000	0.001
2.8.1.6|g__Bacteroides.s__Bacteroides_eggerthii	+	9.03E-01	0.000	0.000	0.000	0.013	0.012
2.8.1.6|g__Bacteroides.s__Bacteroides_faecichinchillae	+	3.30E-01	0.000	0.000	0.000	0.000	0.000
2.8.1.6|g__Bacteroides.s__Bacteroides_faecis	+	5.75E-02	0.000	0.000	0.000	0.003	0.003
2.8.1.6|g__Bacteroides.s__Bacteroides_finegoldii	+	6.18E-01	0.000	0.000	0.000	0.003	0.003
2.8.1.6|g__Bacteroides.s__Bacteroides_fluxus	+	2.32E-01	0.000	0.000	0.000	0.000	0.000
2.8.1.6|g__Bacteroides.s__Bacteroides_fragilis	-	4.25E-01	0.000	0.000	0.000	0.008	0.008
2.8.1.6|g__Bacteroides.s__Bacteroides_intestinalis	+	2.23E-01	0.000	0.000	0.000	0.004	0.008
2.8.1.6|g__Bacteroides.s__Bacteroides_massiliensis	+	3.88E-01	0.000	0.000	0.000	0.013	0.011
2.8.1.6|g__Bacteroides.s__Bacteroides_nordii	-	1.41E-01	0.000	0.000	0.000	0.001	0.001
2.8.1.6|g__Bacteroides.s__Bacteroides_plebeius	-	2.51E-01	0.000	0.000	0.000	0.014	0.006
2.8.1.6|g__Bacteroides.s__Bacteroides_salyersiae	+	2.98E-02	0.000	0.000	0.000	0.002	0.002
2.8.1.6|g__Bacteroides.s__Bacteroides_sartorii	-	4.06E-02	0.000	0.000	0.000	0.000	0.000
2.8.1.6|g__Bacteroides.s__Bacteroides_sp_OM08_11	+	4.92E-01	0.000	0.000	0.000	0.000	0.000
2.8.1.6|g__Bacteroides.s__Bacteroides_stercorirosoris	+	5.59E-01	0.000	0.000	0.000	0.000	0.000
2.8.1.6|g__Barnesiella.s__Barnesiella_intestinihominis	+	1.20E-01	0.000	0.000	0.000	0.004	0.005
2.8.1.6|g__Blautia.s__Blautia_coccoides	+	6.89E-03	0.000	0.000	0.000	0.001	0.003
2.8.1.6|g__Blautia.s__Blautia_producta	+	1.22E-01	0.000	0.000	0.000	0.002	0.001
2.8.1.6|g__Blautia.s__Blautia_sp_AF19_10LB	+	5.66E-01	0.000	0.000	0.000	0.001	0.000
2.8.1.6|g__Blautia.s__Blautia_sp_An249	-	1.36E-01	0.000	0.000	0.000	0.000	0.000
2.8.1.6|g__Blautia.s__Ruminococcus_gnavus	-	2.25E-01	0.000	0.000	0.000	0.011	0.008
2.8.1.6|g__Brevibacterium.s__Brevibacterium_ravenspurgense	+	4.92E-01	0.000	0.000	0.000	0.000	0.000
2.8.1.6|g__Butyricicoccus.s__Butyricicoccus_pullicaecorum	-	6.65E-02	0.000	0.000	0.000	0.000	0.000
2.8.1.6|g__Butyricimonas.s__Butyricimonas_synergistica	-	1.48E-01	0.000	0.000	0.000	0.000	0.000
2.8.1.6|g__Butyricimonas.s__Butyricimonas_virosa	+	3.77E-02	0.000	0.000	0.000	0.000	0.001
2.8.1.6|g__Butyrivibrio.s__Butyrivibrio_crossotus	-	4.80E-01	0.000	0.000	0.000	0.001	0.001
2.8.1.6|g__Campylobacter.s__Campylobacter_coli	+	4.92E-01	0.000	0.000	0.000	0.000	0.000
2.8.1.6|g__Campylobacter.s__Campylobacter_curvus	+	3.30E-01	0.000	0.000	0.000	0.000	0.000
2.8.1.6|g__Campylobacter.s__Campylobacter_gracilis	-	7.76E-01	0.000	0.000	0.000	0.000	0.000
2.8.1.6|g__Campylobacter.s__Campylobacter_jejuni	+	4.92E-01	0.000	0.000	0.000	0.000	0.000
2.8.1.6|g__Campylobacter.s__Campylobacter_ureolyticus	+	4.92E-01	0.000	0.000	0.000	0.000	0.000
2.8.1.6|g__Candidatus_Gastranaerophilales_unclassified.s__Candidatus_Gas	+	6.68E-02	0.000	0.000	0.000	0.000	0.000
2.8.1.6|g__Catabacter.s__Catabacter_hongkongensis	+	4.09E-01	0.000	0.000	0.000	0.000	0.000
2.8.1.6|g__Catenibacterium.s__Catenibacterium_mitsuokai	+	5.54E-01	0.000	0.000	0.000	0.002	0.004
2.8.1.6|g__Cellulosilyticum.s__Cellulosilyticum_lentocellum	+	4.92E-01	0.000	0.000	0.000	0.000	0.000
2.8.1.6|g__Cetobacterium.s__Cetobacterium_somerae	+	3.30E-01	0.000	0.000	0.000	0.000	0.000
2.8.1.6|g__Christensenella.s__Christensenella_minuta	+	7.36E-02	0.000	0.000	0.000	0.000	0.000
2.8.1.6|g__Citrobacter.s__Citrobacter_amalonaticus	+	2.32E-01	0.000	0.000	0.000	0.000	0.000
2.8.1.6|g__Citrobacter.s__Citrobacter_braakii	-	9.67E-01	0.000	0.000	0.000	0.000	0.000
2.8.1.6|g__Citrobacter.s__Citrobacter_farmeri	+	1.67E-01	0.000	0.000	0.000	0.000	0.000
2.8.1.6|g__Citrobacter.s__Citrobacter_freundii	+	9.43E-02	0.000	0.000	0.000	0.001	0.000
2.8.1.6|g__Citrobacter.s__Citrobacter_koseri	+	2.32E-01	0.000	0.000	0.000	0.000	0.000
2.8.1.6|g__Citrobacter.s__Citrobacter_portucalensis	+	4.13E-01	0.000	0.000	0.000	0.000	0.000
2.8.1.6|g__Citrobacter.s__Citrobacter_werkmanii	-	5.95E-01	0.000	0.000	0.000	0.000	0.000
2.8.1.6|g__Citrobacter.s__Citrobacter_youngae	+	5.87E-01	0.000	0.000	0.000	0.000	0.000
2.8.1.6|g__Cloacibacillus.s__Cloacibacillus_porcorum	+	9.43E-02	0.000	0.000	0.000	0.000	0.001
2.8.1.6|g__Clostridiales_Family_XIII_Incertae_Sedis_unclassified.s__Eubac	+	4.92E-01	0.000	0.000	0.000	0.000	0.000
2.8.1.6|g__Clostridiales_unclassified.s__Bacteroides_pectinophilus	-	9.70E-01	0.000	0.000	0.000	0.000	0.000
2.8.1.6|g__Clostridiales_unclassified.s__Clostridiales_bacterium_KLE1615	-	2.68E-05	0.000	0.000	0.000	0.006	0.004
2.8.1.6|g__Clostridioides.s__Clostridioides_difficile	+	1.67E-01	0.000	0.000	0.000	0.000	0.000
2.8.1.6|g__Clostridium.s__Butyribacterium_methylotrophicum	+	1.75E-05	0.000	0.000	0.000	0.001	0.002
2.8.1.6|g__Clostridium.s__Clostridium_butyricum	-	1.48E-01	0.000	0.000	0.000	0.000	0.000
2.8.1.6|g__Clostridium.s__Clostridium_cadaveris	-	5.95E-01	0.000	0.000	0.000	0.000	0.000
2.8.1.6|g__Clostridium.s__Clostridium_disporicum	+	9.39E-01	0.000	0.000	0.000	0.000	0.000
2.8.1.6|g__Clostridium.s__Clostridium_perfringens	-	7.67E-01	0.000	0.000	0.000	0.000	0.000
2.8.1.6|g__Clostridium.s__Clostridium_sp_AF36_4	-	2.93E-01	0.000	0.000	0.000	0.003	0.003
2.8.1.6|g__Clostridium.s__Clostridium_sp_D5	+	3.30E-01	0.000	0.000	0.000	0.000	0.000
2.8.1.6|g__Clostridium.s__Clostridium_sp_MSTE9	+	3.30E-01	0.000	0.000	0.000	0.000	0.000
2.8.1.6|g__Clostridium.s__Clostridium_sp_chh4_2	+	3.30E-01	0.000	0.000	0.000	0.000	0.000

2.8.1.6|g__Clostridium.s__Clostridium_ventriculi	+	7.60E-01	0.000	0.000	0.000	0.000	0.000
2.8.1.6|g__Comamonas.s__Comamonas_kerstersii	+	3.30E-01	0.000	0.000	0.000	0.000	0.000
2.8.1.6|g__Comamonas.s__Comamonas_testosteroni	+	4.92E-01	0.000	0.000	0.000	0.000	0.000
2.8.1.6|g__Comamonas.s__Comamonas_thiooxydans	+	4.92E-01	0.000	0.000	0.000	0.000	0.000
2.8.1.6|g__Coprobacter.s__Coprobacter_fastidiosus	-	7.58E-03	0.000	0.000	0.000	0.001	0.000
2.8.1.6|g__Coprobacter.s__Coprobacter_secundus	+	4.92E-01	0.000	0.000	0.000	0.000	0.000
2.8.1.6|g__Coprococcus.s__Coprococcus_comes	-	7.86E-01	0.000	0.000	0.000	0.006	0.006
2.8.1.6|g__Coprococcus.s__Coprococcus_eutactus	-	6.26E-01	0.000	0.000	0.000	0.004	0.003
2.8.1.6|g__Corynebacterium.s__Corynebacterium_amycolatum	+	3.30E-01	0.000	0.000	0.000	0.000	0.000
2.8.1.6|g__Corynebacterium.s__Corynebacterium_argentoratense	+	3.06E-01	0.000	0.000	0.000	0.000	0.000
2.8.1.6|g__Corynebacterium.s__Corynebacterium_aurimucosum	+	4.92E-01	0.000	0.000	0.000	0.000	0.000
2.8.1.6|g__Corynebacterium.s__Corynebacterium_coyleae	+	4.92E-01	0.000	0.000	0.000	0.000	0.000
2.8.1.6|g__Corynebacterium.s__Corynebacterium_frankenforstense	-	1.48E-01	0.000	0.000	0.000	0.000	0.000
2.8.1.6|g__Corynebacterium.s__Corynebacterium_freneyi	+	4.92E-01	0.000	0.000	0.000	0.000	0.000
2.8.1.6|g__Corynebacterium.s__Corynebacterium_imitans	+	4.92E-01	0.000	0.000	0.000	0.000	0.000
2.8.1.6|g__Corynebacterium.s__Corynebacterium_jeikeium	+	3.30E-01	0.000	0.000	0.000	0.000	0.000
2.8.1.6|g__Corynebacterium.s__Corynebacterium_kroppenstedtii	+	4.92E-01	0.000	0.000	0.000	0.000	0.000
2.8.1.6|g__Corynebacterium.s__Corynebacterium_pseudogenitalium	+	4.92E-01	0.000	0.000	0.000	0.000	0.000
2.8.1.6|g__Corynebacterium.s__Corynebacterium_riegelii	+	4.92E-01	0.000	0.000	0.000	0.000	0.000
2.8.1.6|g__Corynebacterium.s__Corynebacterium_tuberculostearicum	+	4.92E-01	0.000	0.000	0.000	0.000	0.000
2.8.1.6|g__Corynebacterium.s__Corynebacterium_tuscaniense	+	4.92E-01	0.000	0.000	0.000	0.000	0.000
2.8.1.6|g__Desulfovibrio.s__Desulfovibrio_desulfuricans	+	1.67E-01	0.000	0.000	0.000	0.000	0.000
2.8.1.6|g__Desulfovibrio.s__Desulfovibrio_fairfieldensis	+	2.53E-01	0.000	0.000	0.000	0.001	0.002
2.8.1.6|g__Desulfovibrio.s__Desulfovibrio_legallii	+	4.92E-01	0.000	0.000	0.000	0.000	0.000
2.8.1.6|g__Desulfovibrio.s__Desulfovibrio_piger	+	2.91E-01	0.000	0.000	0.000	0.000	0.001
2.8.1.6|g__Desulfovibrio.s__Desulfovibrio_sp_AM18_2	+	3.04E-01	0.000	0.000	0.000	0.000	0.000
2.8.1.6|g__Desulfovibrionaceae_unclassified.s__Desulfovibrionaceae_bacte[illegible]	+	1.30E-01	0.000	0.000	0.000	0.000	0.000
2.8.1.6|g__Dialister.s__Dialister_invisus	-	4.57E-03	0.000	0.000	0.000	0.005	0.003
2.8.1.6|g__Dialister.s__Dialister_pneumosintes	+	4.92E-01	0.000	0.000	0.000	0.000	0.000
2.8.1.6|g__Dialister.s__Dialister_succinatiphilus	+	4.92E-01	0.000	0.000	0.000	0.000	0.000
2.8.1.6|g__Dorea.s__Dorea_formicigenerans	-	7.61E-01	0.000	0.000	0.000	0.002	0.002
2.8.1.6|g__Dorea.s__Dorea_sp_OM02_2LB	+	2.97E-01	0.000	0.000	0.000	0.000	0.000
2.8.1.6|g__Eisenbergiella.s__Eisenbergiella_tayi	+	1.34E-04	0.000	0.000	0.000	0.001	0.003
2.8.1.6|g__Enterobacter.s__Enterobacter_bugandensis	+	4.13E-01	0.000	0.000	0.000	0.000	0.000
2.8.1.6|g__Enterobacter.s__Enterobacter_cloacae	+	2.81E-02	0.000	0.000	0.000	0.000	0.000
2.8.1.6|g__Enterobacter.s__Enterobacter_kobei	+	3.30E-01	0.000	0.000	0.000	0.000	0.000
2.8.1.6|g__Enterobacter.s__Enterobacter_mori	+	4.92E-01	0.000	0.000	0.000	0.000	0.000
2.8.1.6|g__Erysipelatoclostridium.s__Clostridium_innocuum	-	7.86E-01	0.000	0.000	0.000	0.000	0.000
2.8.1.6|g__Erysipelatoclostridium.s__Clostridium_spiroforme	+	1.22E-01	0.000	0.000	0.000	0.000	0.000
2.8.1.6|g__Erysipelatoclostridium.s__Erysipelatoclostridium_ramosum	+	3.48E-01	0.000	0.000	0.000	0.000	0.001
2.8.1.6|g__Escherichia.s__Escherichia_coli	+	2.35E-04	0.000	0.000	0.000	0.012	0.018
2.8.1.6|g__Escherichia.s__Escherichia_fergusonii	+	1.01E-01	0.000	0.000	0.000	0.000	0.000
2.8.1.6|g__Escherichia.s__Escherichia_marmotae	+	4.92E-01	0.000	0.000	0.000	0.000	0.000
2.8.1.6|g__Eubacteriaceae_unclassified.s__Eubacteriaceae_bacterium_CHK[illegible]	+	5.18E-01	0.000	0.000	0.000	0.000	0.000
2.8.1.6|g__Eubacterium.s__Eubacterium_callanderi	+	2.67E-05	0.000	0.000	0.000	0.001	0.002
2.8.1.6|g__Eubacterium.s__Eubacterium_eligens	-	1.04E-01	0.000	0.000	0.000	0.006	0.005
2.8.1.6|g__Eubacterium.s__Eubacterium_limosum	+	7.27E-03	0.000	0.000	0.000	0.000	0.000
2.8.1.6|g__Eubacterium.s__Eubacterium_maltosivorans	+	1.50E-01	0.000	0.000	0.000	0.000	0.000
2.8.1.6|g__Eubacterium.s__Eubacterium_ramulus	-	2.19E-02	0.000	0.000	0.000	0.003	0.002
2.8.1.6|g__Eubacterium.s__Eubacterium_sp_AF17_7	-	5.41E-01	0.000	0.000	0.000	0.000	0.000
2.8.1.6|g__Faecalicatena.s__Faecalicatena_contorta	+	2.11E-02	0.000	0.000	0.000	0.000	0.000
2.8.1.6|g__Firmicutes_unclassified.s__Firmicutes_bacterium_AM10_47	-	5.02E-01	0.000	0.000	0.000	0.001	0.000
2.8.1.6|g__Fusobacterium.s__Fusobacterium_mortiferum	-	4.48E-01	0.000	0.000	0.000	0.000	0.000
2.8.1.6|g__Fusobacterium.s__Fusobacterium_nucleatum	-	5.92E-01	0.000	0.000	0.000	0.000	0.000
2.8.1.6|g__Fusobacterium.s__Fusobacterium_ulcerans	-	1.60E-01	0.000	0.000	0.000	0.000	0.000
2.8.1.6|g__Fusobacterium.s__Fusobacterium_varium	+	4.92E-01	0.000	0.000	0.000	0.000	0.000
2.8.1.6|g__Gordonibacter.s__Gordonibacter_pamelaeae	+	8.49E-01	0.000	0.000	0.000	0.001	0.001
2.8.1.6|g__Haemophilus.s__Haemophilus_parainfluenzae	-	2.58E-01	0.000	0.000	0.000	0.000	0.000
2.8.1.6|g__Hafnia.s__Hafnia_paralvei	-	5.95E-01	0.000	0.000	0.000	0.000	0.000
2.8.1.6|g__Hungatella.s__Hungatella_effluvii	+	3.16E-02	0.000	0.000	0.000	0.000	0.000
2.8.1.6|g__Hungatella.s__Hungatella_hathewayi	+	3.61E-02	0.000	0.000	0.000	0.001	0.001
2.8.1.6|g__Klebsiella.s__Klebsiella_aerogenes	+	1.94E-01	0.000	0.000	0.000	0.000	0.001
2.8.1.6|g__Klebsiella.s__Klebsiella_grimontii	-	9.73E-01	0.000	0.000	0.000	0.000	0.000
2.8.1.6|g__Klebsiella.s__Klebsiella_michiganensis	-	9.55E-01	0.000	0.000	0.000	0.000	0.000
2.8.1.6|g__Klebsiella.s__Klebsiella_oxytoca	+	1.78E-01	0.000	0.000	0.000	0.000	0.000
2.8.1.6|g__Klebsiella.s__Klebsiella_pneumoniae	+	3.65E-03	0.000	0.000	0.000	0.005	0.005
2.8.1.6|g__Klebsiella.s__Klebsiella_variicola	+	8.20E-03	0.000	0.000	0.000	0.000	0.001
2.8.1.6|g__Kluyvera.s__Kluyvera_ascorbata	+	7.55E-01	0.000	0.000	0.000	0.000	0.000
2.8.1.6|g__Kluyvera.s__Kluyvera_cryocrescens	+	3.30E-01	0.000	0.000	0.000	0.000	0.000
2.8.1.6|g__Kosakonia.s__Kosakonia_cowanii	+	4.92E-01	0.000	0.000	0.000	0.000	0.000
2.8.1.6|g__Lachnoclostridium.s__Clostridium_citroniae	+	2.81E-02	0.000	0.000	0.000	0.000	0.000
2.8.1.6|g__Lachnoclostridium.s__Clostridium_saccharolyticum	+	7.90E-01	0.000	0.000	0.000	0.001	0.001

2.8.1.6|g__Lachnoclostridium.s__Clostridium_scindens	+	5.03E-02	0.000	0.000	0.000	0.003	0.004
2.8.1.6|g__Lachnoclostridium.s__Clostridium_symbiosum	+	2.13E-01	0.000	0.000	0.000	0.002	0.003
2.8.1.6|g__Lachnoclostridium.s__Lachnoclostridium_sp_An14	-	2.03E-01	0.000	0.000	0.000	0.000	0.000
2.8.1.6|g__Lachnospira.s__Lachnospira_pectinoschiza	-	6.40E-01	0.000	0.000	0.000	0.002	0.002
2.8.1.6|g__Lactobacillus.s__Lactobacillus_rogosae	-	8.32E-01	0.000	0.000	0.000	0.001	0.001
2.8.1.6|g__Leclercia.s__Leclercia_adecarboxylata	+	4.09E-01	0.000	0.000	0.000	0.000	0.000
2.8.1.6|g__Lelliottia.s__Lelliottia_amnigena	+	4.92E-01	0.000	0.000	0.000	0.000	0.000
2.8.1.6|g__Lelliottia.s__Lelliottia_nimipressuralis	+	3.09E-01	0.000	0.000	0.000	0.000	0.000
2.8.1.6|g__Megamonas.s__Megamonas_funiformis	+	8.67E-01	0.000	0.000	0.000	0.001	0.000
2.8.1.6|g__Megamonas.s__Megamonas_hypermegale	+	8.67E-01	0.000	0.000	0.000	0.000	0.000
2.8.1.6|g__Megamonas.s__Megamonas_rupellensis	+	4.20E-01	0.000	0.000	0.000	0.000	0.000
2.8.1.6|g__Megasphaera.s__Megasphaera_cerevisiae	+	4.92E-01	0.000	0.000	0.000	0.000	0.000
2.8.1.6|g__Megasphaera.s__Megasphaera_elsdenii	-	9.60E-01	0.000	0.000	0.000	0.000	0.000
2.8.1.6|g__Megasphaera.s__Megasphaera_hexanoica	+	4.92E-01	0.000	0.000	0.000	0.000	0.000
2.8.1.6|g__Megasphaera.s__Megasphaera_micronuciformis	+	2.32E-01	0.000	0.000	0.000	0.000	0.000
2.8.1.6|g__Megasphaera.s__Megasphaera_sp_DISK_18	+	6.88E-04	0.000	0.000	0.000	0.001	0.003
2.8.1.6|g__Megasphaera.s__Megasphaera_sp_MJR8396C	+	5.59E-02	0.000	0.000	0.000	0.002	0.003
2.8.1.6|g__Megasphaera.s__Megasphaera_stantonii	-	5.92E-01	0.000	0.000	0.000	0.000	0.000
2.8.1.6|g__Mitsuokella.s__Mitsuokella_jalaludinii	-	5.95E-01	0.000	0.000	0.000	0.000	0.000
2.8.1.6|g__Mitsuokella.s__Mitsuokella_multacida	+	4.92E-01	0.000	0.000	0.000	0.000	0.000
2.8.1.6|g__Morganella.s__Morganella_morganii	+	1.22E-01	0.000	0.000	0.000	0.000	0.000
2.8.1.6|g__Muribaculum.s__Muribaculum_intestinale	-	5.92E-01	0.000	0.000	0.000	0.000	0.000
2.8.1.6|g__Odoribacter.s__Odoribacter_laneus	+	2.66E-01	0.000	0.000	0.000	0.002	0.004
2.8.1.6|g__Oligella.s__Oligella_urethralis	+	4.92E-01	0.000	0.000	0.000	0.000	0.001
2.8.1.6|g__Oxalobacter.s__Oxalobacter_formigenes	+	2.79E-01	0.000	0.000	0.000	0.000	0.000
2.8.1.6|g__Pantoea.s__Pantoea_sesami	+	3.09E-01	0.000	0.000	0.000	0.000	0.000
2.8.1.6|g__Parabacteroides.s__Parabacteroides_goldsteinii	+	1.30E-01	0.000	0.000	0.000	0.001	0.002
2.8.1.6|g__Parabacteroides.s__Parabacteroides_gordonii	-	2.33E-01	0.000	0.000	0.000	0.000	0.000
2.8.1.6|g__Parabacteroides.s__Parabacteroides_johnsonii	-	4.54E-01	0.000	0.000	0.000	0.001	0.001
2.8.1.6|g__Paraeggerthella.s__Paraeggerthella_hongkongensis	+	3.30E-01	0.000	0.000	0.000	0.000	0.000
2.8.1.6|g__Paraprevotella.s__Paraprevotella_clara	-	4.68E-02	0.000	0.000	0.000	0.003	0.003
2.8.1.6|g__Paraprevotella.s__Paraprevotella_xylaniphila	+	4.16E-01	0.000	0.000	0.000	0.000	0.000
2.8.1.6|g__Pedobacter.s__Pedobacter_himalayensis	+	3.30E-01	0.000	0.000	0.000	0.000	0.000
2.8.1.6|g__Peptoniphilus.s__Peptoniphilus_coxii	-	1.48E-01	0.000	0.000	0.000	0.000	0.000
2.8.1.6|g__Peptoniphilus.s__Peptoniphilus_harei	+	4.92E-01	0.000	0.000	0.000	0.000	0.000
2.8.1.6|g__Peptoniphilus.s__Peptoniphilus_sp_HMSC062D09	+	3.30E-01	0.000	0.000	0.000	0.000	0.000
2.8.1.6|g__Peptostreptococcus.s__Peptostreptococcus_anaerobius	+	4.92E-01	0.000	0.000	0.000	0.000	0.000
2.8.1.6|g__Peptostreptococcus.s__Peptostreptococcus_stomatis	+	4.92E-01	0.000	0.000	0.000	0.000	0.000
2.8.1.6|g__Phascolarctobacterium.s__Phascolarctobacterium_succinatutens	+	1.40E-01	0.000	0.000	0.000	0.002	0.002
2.8.1.6|g__Pluralibacter.s__Pluralibacter_gergoviae	-	1.48E-01	0.000	0.000	0.000	0.000	0.000
2.8.1.6|g__Porphyromonas.s__Porphyromonas_asaccharolytica	+	2.45E-01	0.000	0.000	0.000	0.000	0.000
2.8.1.6|g__Porphyromonas.s__Porphyromonas_sp_HMSC065F10	+	1.67E-01	0.000	0.000	0.000	0.000	0.000
2.8.1.6|g__Porphyromonas.s__Porphyromonas_sp_HMSC077F02	+	5.59E-01	0.000	0.000	0.000	0.000	0.000
2.8.1.6|g__Porphyromonas.s__Porphyromonas_uenonis	-	5.95E-01	0.000	0.000	0.000	0.000	0.000
2.8.1.6|g__Propionibacterium.s__Propionibacterium_acidifaciens	+	3.30E-01	0.000	0.000	0.000	0.000	0.000
2.8.1.6|g__Propionibacterium.s__Propionibacterium_freudenreichii	-	4.50E-01	0.000	0.000	0.000	0.000	0.000
2.8.1.6|g__Proteus.s__Proteus_mirabilis	-	9.59E-01	0.000	0.000	0.000	0.000	0.000
2.8.1.6|g__Pseudocitrobacter.s__Pseudocitrobacter_faecalis	+	3.30E-01	0.000	0.000	0.000	0.000	0.000
2.8.1.6|g__Pseudoflavonifractor.s__Pseudoflavonifractor_sp_An184	+	1.30E-02	0.000	0.000	0.000	0.000	0.001
2.8.1.6|g__Pseudomonas.s__Pseudomonas_aeruginosa_group	+	4.16E-01	0.000	0.000	0.000	0.000	0.000
2.8.1.6|g__Pseudomonas.s__Pseudomonas_alcaligenes	+	4.16E-01	0.000	0.000	0.000	0.000	0.000
2.8.1.6|g__Pseudomonas.s__Pseudomonas_delhiensis	+	4.92E-01	0.000	0.000	0.000	0.000	0.000
2.8.1.6|g__Pseudomonas.s__Pseudomonas_flavescens	-	1.48E-01	0.000	0.000	0.000	0.000	0.000
2.8.1.6|g__Pseudomonas.s__Pseudomonas_fluorescens_group	-	1.48E-01	0.000	0.000	0.000	0.000	0.000
2.8.1.6|g__Pseudoramibacter.s__Pseudoramibacter_alactolyticus	+	4.92E-01	0.000	0.000	0.000	0.000	0.000
2.8.1.6|g__Raoultella.s__Raoultella_ornithinolytica	-	9.67E-01	0.000	0.000	0.000	0.000	0.000
2.8.1.6|g__Raoultella.s__Raoultella_planticola	-	2.03E-01	0.000	0.000	0.000	0.000	0.000
2.8.1.6|g__Rikenella.s__Rikenella_microfusus	+	4.92E-01	0.000	0.000	0.000	0.000	0.000
2.8.1.6|g__Romboutsia.s__Romboutsia_hominis	+	4.92E-01	0.000	0.000	0.000	0.000	0.000
2.8.1.6|g__Roseburia.s__Roseburia_inulinivorans	-	5.86E-03	0.000	0.000	0.000	0.003	0.002
2.8.1.6|g__Ruminococcaceae_unclassified.s__Eubacterium_siraeum	-	8.14E-01	0.000	0.000	0.000	0.019	0.015
2.8.1.6|g__Ruminococcaceae_unclassified.s__Ruminococcaceae_bacterium_	+	1.27E-01	0.000	0.000	0.000	0.001	0.002
2.8.1.6|g__Ruminococcaceae_unclassified.s__Ruminococcaceae_bacterium_	+	2.41E-02	0.000	0.000	0.000	0.000	0.000
2.8.1.6|g__Ruminococcus.s__Ruminococcus_champanellensis	+	2.32E-01	0.000	0.000	0.000	0.000	0.000
2.8.1.6|g__Ruminococcus.s__Ruminococcus_lactaris	-	3.20E-04	0.000	0.000	0.000	0.005	0.003
2.8.1.6|g__Salmonella.s__Salmonella_enterica	+	4.92E-01	0.000	0.000	0.000	0.000	0.000
2.8.1.6|g__Sanguibacteroides.s__Sanguibacteroides_justesenii	+	4.92E-01	0.000	0.000	0.000	0.000	0.000
2.8.1.6|g__Staphylococcus.s__Staphylococcus_aureus	+	1.22E-01	0.000	0.000	0.000	0.000	0.000
2.8.1.6|g__Staphylococcus.s__Staphylococcus_epidermidis	+	4.92E-01	0.000	0.000	0.000	0.000	0.000
2.8.1.6|g__Staphylococcus.s__Staphylococcus_haemolyticus	+	4.92E-01	0.000	0.000	0.000	0.000	0.000
2.8.1.6|g__Streptococcus.s__Streptococcus_agalactiae	+	4.92E-01	0.000	0.000	0.000	0.000	0.000
2.8.1.6|g__Streptococcus.s__Streptococcus_equinus	+	1.44E-01	0.000	0.000	0.000	0.000	0.001

	2.8.1.6|g__Streptococcus.s__Streptococcus_gallolyticus	+	2.23E-01	0.000	0.000	0.000	0.000	0.001
	2.8.1.6|g__Streptococcus.s__Streptococcus_pasteurianus	+	1.44E-01	0.000	0.000	0.000	0.000	0.001
	2.8.1.6|g__Streptococcus.s__Streptococcus_pneumoniae	-	2.01E-01	0.000	0.000	0.000	0.000	0.000
	2.8.1.6|g__Succinatimonas.s__Succinatimonas_hippei	+	3.30E-01	0.000	0.000	0.000	0.000	0.000
	2.8.1.6|g__Sutterella.s__Sutterella_wadsworthensis	+	2.41E-01	0.000	0.000	0.000	0.002	0.002
	2.8.1.6|g__Terrisporobacter.s__Terrisporobacter_othiniensis	+	1.67E-01	0.000	0.000	0.000	0.000	0.000
	2.8.1.6|g__Trichococcus.s__Trichococcus_flocculiformis	+	4.92E-01	0.000	0.000	0.000	0.000	0.000
	2.8.1.6|g__Trichococcus.s__Trichococcus_pasteurii	+	4.92E-01	0.000	0.000	0.000	0.000	0.000
	2.8.1.6|g__Veillonella.s__Veillonella_atypica	-	4.01E-02	0.000	0.000	0.000	0.000	0.001
	2.8.1.6|g__Veillonella.s__Veillonella_dispar	-	3.37E-01	0.000	0.000	0.000	0.000	0.000
	2.8.1.6|g__Veillonella.s__Veillonella_infantium	-	5.02E-01	0.000	0.000	0.000	0.000	0.000
	2.8.1.6|g__Veillonella.s__Veillonella_parvula	+	8.52E-01	0.000	0.000	0.000	0.000	0.001
	2.8.1.6|g__Veillonella.s__Veillonella_rogosae	-	1.33E-01	0.000	0.000	0.000	0.000	0.000
	2.8.1.6|g__Veillonella.s__Veillonella_seminalis	-	4.47E-01	0.000	0.000	0.000	0.000	0.000
	2.8.1.6|g__Vibrio.s__Vibrio_parahaemolyticus	+	2.33E-01	0.000	0.000	0.000	0.000	0.000
	2.8.1.6|g__Victivallales_unclassified.s__Victivallales_bacterium_CCUG_44	+	3.48E-03	0.000	0.000	0.000	0.000	0.000
	2.8.1.6|g__Victivallis.s__Victivallis_vadensis	+	2.39E-01	0.000	0.000	0.000	0.000	0.000
	2.8.1.6|g__Yokenella.s__Yokenella_regensburgei	+	7.55E-01	0.000	0.000	0.000	0.000	0.000
	2.8.1.6|g__Bacteroides.s__Bacteroides_thetaiotaomicron	+	3.11E-01	0.423	0.441	0.017	0.012	0.013
	2.8.1.6|g__Bacteroides.s__Bacteroides_xylanisolvens	+	3.73E-01	0.000	0.020	0.020	0.008	0.006
	2.8.1.6|g__Bacteroides.s__Bacteroides_dorei	+	3.34E-01	0.079	0.105	0.027	0.019	0.023
	2.8.1.6|g__Phascolarctobacterium.s__Phascolarctobacterium_faecium	+	4.00E-01	0.213	0.270	0.056	0.006	0.007
	2.8.1.6|unclassified	-	5.21E-01	9.647	9.742	0.096	0.113	0.121
	2.8.1.6|g__Parabacteroides.s__Parabacteroides_merdae	+	1.38E-01	0.745	0.907	0.162	0.011	0.014
	2.8.1.6|g__Odoribacter.s__Odoribacter_splanchnicus	+	2.39E-01	0.000	0.213	0.213	0.003	0.004
	2.8.1.6|g__Bilophila.s__Bilophila_wadsworthia	+	4.07E-03	0.000	0.222	0.222	0.002	0.004
	2.8.1.6|g__Bacteroides.s__Bacteroides_caccae	+	1.38E-01	0.176	0.405	0.228	0.009	0.013
	2.8.1.6|g__Bacteroides.s__Bacteroides_cellulosilyticus	+	2.78E-01	0.084	0.324	0.239	0.020	0.022
	2.8.1.6|g__Parabacteroides.s__Parabacteroides_distasonis	+	2.32E-03	1.083	1.497	0.414	0.021	0.026
	2.8.1.6|g__Bacteroides.s__Bacteroides_stercoris	+	8.11E-02	0.344	0.864	0.520	0.050	0.060
Germany	2.8.1.6	-	8.97E-01	86.350	89.515	3.164	1.000	1.000
	2.8.1.6|g__Blautia.s__Ruminococcus_torques	-	2.39E-01	3.007	1.376	-1.631	0.048	0.032
	2.8.1.6|g__Parabacteroides.s__Parabacteroides_distasonis	-	9.94E-01	1.499	1.030	-0.469	0.018	0.017
	2.8.1.6|g__Roseburia.s__Roseburia_intestinalis	-	5.72E-02	0.481	0.038	-0.442	0.040	0.011
	2.8.1.6|g__Roseburia.s__Roseburia_faecis	-	2.20E-01	0.930	0.498	-0.432	0.030	0.018
	2.8.1.6|g__Bacteroides.s__Bacteroides_dorei	-	5.59E-01	1.265	0.903	-0.362	0.027	0.020
	2.8.1.6|g__Blautia.s__Blautia_obeum	-	7.61E-01	1.381	1.045	-0.336	0.016	0.021
	2.8.1.6|g__Escherichia.s__Escherichia_coli	-	1.27E-01	0.301	0.000	-0.301	0.033	0.015
	2.8.1.6|g__Blautia.s__Blautia_wexlerae	-	2.96E-01	0.562	0.389	-0.173	0.009	0.008
	2.8.1.6|g__Bacteroides.s__Bacteroides_thetaiotaomicron	-	4.88E-01	0.400	0.263	-0.138	0.012	0.006
	2.8.1.6|g__Coprococcus.s__Coprococcus_comes	-	7.61E-01	0.526	0.400	-0.127	0.010	0.010
	2.8.1.6|g__Lachnospiraceae_unclassified.s__Eubacterium_rectale	+	7.20E-01	0.853	0.758	-0.095	0.019	0.022
	2.8.1.6|g__Roseburia.s__Roseburia_inulinivorans	-	8.40E-01	0.395	0.309	-0.086	0.012	0.008
	2.8.1.6|g__Dorea.s__Dorea_formicigenerans	-	4.32E-01	0.242	0.183	-0.059	0.004	0.003
	2.8.1.6|g__Eubacterium.s__Eubacterium_ramulus	-	5.71E-01	0.052	0.000	-0.052	0.003	0.003
	2.8.1.6|g__Bacteroides.s__Bacteroides_xylanisolvens	-	8.65E-01	0.210	0.197	-0.013	0.014	0.013
	2.8.1.6|g__Acidaminococcus.s__Acidaminococcus_fermentans	-	9.42E-01	0.000	0.000	0.000	0.001	0.000
	2.8.1.6|g__Acidaminococcus.s__Acidaminococcus_intestini	-	9.31E-01	0.000	0.000	0.000	0.001	0.002
	2.8.1.6|g__Alistipes.s__Alistipes_indistinctus	+	6.62E-01	0.000	0.000	0.000	0.002	0.002
	2.8.1.6|g__Allisonella.s__Allisonella_histaminiformans	-	1.40E-01	0.000	0.000	0.000	0.000	0.000
	2.8.1.6|g__Anaerotignum.s__Anaerotignum_lactatifermentans	-	9.81E-01	0.000	0.000	0.000	0.000	0.000
	2.8.1.6|g__Anaerotruncus.s__Anaerotruncus_colihominis	+	6.61E-01	0.000	0.000	0.000	0.000	0.000
	2.8.1.6|g__Bacteroides.s__Bacteroides_clarus	-	9.27E-01	0.000	0.000	0.000	0.004	0.002
	2.8.1.6|g__Bacteroides.s__Bacteroides_coprocola	-	7.50E-01	0.000	0.000	0.000	0.023	0.009
	2.8.1.6|g__Bacteroides.s__Bacteroides_coprophilus	-	8.75E-01	0.000	0.000	0.000	0.005	0.000
	2.8.1.6|g__Bacteroides.s__Bacteroides_eggerthii	+	7.06E-01	0.000	0.000	0.000	0.014	0.004
	2.8.1.6|g__Bacteroides.s__Bacteroides_faecichinchillae	+	3.59E-01	0.000	0.000	0.000	0.000	0.000
	2.8.1.6|g__Bacteroides.s__Bacteroides_faecis	+	7.71E-01	0.000	0.000	0.000	0.003	0.003
	2.8.1.6|g__Bacteroides.s__Bacteroides_finegoldii	+	4.18E-01	0.000	0.000	0.000	0.002	0.003
	2.8.1.6|g__Bacteroides.s__Bacteroides_fluxus	-	1.40E-01	0.000	0.000	0.000	0.000	0.000
	2.8.1.6|g__Bacteroides.s__Bacteroides_fragilis	-	8.73E-01	0.000	0.000	0.000	0.006	0.007
	2.8.1.6|g__Bacteroides.s__Bacteroides_intestinalis	+	3.87E-01	0.000	0.000	0.000	0.001	0.002
	2.8.1.6|g__Bacteroides.s__Bacteroides_massiliensis	-	8.74E-01	0.000	0.000	0.000	0.017	0.013
	2.8.1.6|g__Bacteroides.s__Bacteroides_nordii	+	5.53E-01	0.000	0.000	0.000	0.000	0.000
	2.8.1.6|g__Bacteroides.s__Bacteroides_plebeius	+	5.66E-01	0.000	0.000	0.000	0.003	0.009
	2.8.1.6|g__Bacteroides.s__Bacteroides_salyersiae	+	5.69E-01	0.000	0.000	0.000	0.001	0.004
	2.8.1.6|g__Bacteroides.s__Bacteroides_sp_OM08_11	+	3.59E-01	0.000	0.000	0.000	0.000	0.000
	2.8.1.6|g__Bacteroides.s__Bacteroides_stercorirosoris	+	3.59E-01	0.000	0.000	0.000	0.000	0.000
	2.8.1.6|g__Bacteroides.s__Bacteroides_stercoris	+	8.85E-01	0.000	0.000	0.000	0.018	0.012
	2.8.1.6|g__Blautia.s__Blautia_sp_AF19_10LB	+	2.91E-01	0.000	0.000	0.000	0.001	0.001
	2.8.1.6|g__Blautia.s__Ruminococcus_gnavus	-	3.71E-01	0.000	0.000	0.000	0.006	0.004
	2.8.1.6|g__Butyricicoccus.s__Butyricicoccus_pullicaecorum	+	3.59E-01	0.000	0.000	0.000	0.000	0.000

2.8.1.6|g__Butyricimonas.s__Butyricimonas_virosa	-	2.53E-01	0.000	0.000	0.000	0.003	0.001
2.8.1.6|g__Butyrivibrio.s__Butyrivibrio_crossotus	-	5.37E-01	0.000	0.000	0.000	0.002	0.003
2.8.1.6|g__Candidatus_Gastranaerophilales_unclassified.s__Candidatus_Gas	+	6.32E-01	0.000	0.000	0.000	0.001	0.001
2.8.1.6|g__Catenibacterium.s__Catenibacterium_mitsuokai	-	9.36E-02	0.000	0.000	0.000	0.013	0.005
2.8.1.6|g__Citrobacter.s__Citrobacter_koseri	-	3.09E-01	0.000	0.000	0.000	0.000	0.000
2.8.1.6|g__Cloacibacillus.s__Cloacibacillus_porcorum	+	1.83E-01	0.000	0.000	0.000	0.000	0.001
2.8.1.6|g__Clostridiales_unclassified.s__Bacteroides_pectinophilus	+	5.32E-02	0.000	0.000	0.000	0.000	0.002
2.8.1.6|g__Clostridium.s__Clostridium_disporicum	-	1.40E-01	0.000	0.000	0.000	0.000	0.000
2.8.1.6|g__Clostridium.s__Clostridium_perfringens	-	3.09E-01	0.000	0.000	0.000	0.000	0.000
2.8.1.6|g__Coprobacter.s__Coprobacter_fastidiosus	+	7.61E-01	0.000	0.000	0.000	0.003	0.002
2.8.1.6|g__Coprobacter.s__Coprobacter_secundus	+	3.93E-01	0.000	0.000	0.000	0.000	0.000
2.8.1.6|g__Coprococcus.s__Coprococcus_eutactus	-	7.96E-01	0.000	0.000	0.000	0.020	0.012
2.8.1.6|g__Desulfovibrio.s__Desulfovibrio_fairfieldensis	+	6.71E-01	0.000	0.000	0.000	0.000	0.001
2.8.1.6|g__Desulfovibrio.s__Desulfovibrio_piger	-	5.38E-01	0.000	0.000	0.000	0.001	0.008
2.8.1.6|g__Desulfovibrionaceae_unclassified.s__Desulfovibrionaceae_bacter	-	3.09E-01	0.000	0.000	0.000	0.001	0.000
2.8.1.6|g__Dialister.s__Dialister_invisus	-	8.09E-01	0.000	0.000	0.000	0.012	0.014
2.8.1.6|g__Dialister.s__Dialister_succinatiphilus	-	9.42E-01	0.000	0.000	0.000	0.009	0.003
2.8.1.6|g__Dorea.s__Dorea_sp_OM02_2LB	-	9.81E-01	0.000	0.000	0.000	0.000	0.000
2.8.1.6|g__Eisenbergiella.s__Eisenbergiella_tayi	-	5.09E-01	0.000	0.000	0.000	0.001	0.000
2.8.1.6|g__Enterococcus.s__Enterococcus_hirae	-	3.09E-01	0.000	0.000	0.000	0.000	0.000
2.8.1.6|g__Erysipelatoclostridium.s__Clostridium_spiroforme	-	8.06E-01	0.000	0.000	0.000	0.000	0.000
2.8.1.6|g__Erysipelatoclostridium.s__Erysipelatoclostridium_ramosum	-	3.47E-01	0.000	0.000	0.000	0.001	0.000
2.8.1.6|g__Escherichia.s__Escherichia_fergusonii	-	5.80E-02	0.000	0.000	0.000	0.001	0.000
2.8.1.6|g__Escherichia.s__Escherichia_marmotae	-	3.09E-01	0.000	0.000	0.000	0.000	0.000
2.8.1.6|g__Eubacteriaceae_unclassified.s__Eubacteriaceae_bacterium_CHK	-	5.11E-01	0.000	0.000	0.000	0.000	0.000
2.8.1.6|g__Eubacterium.s__Eubacterium_sp_AF17_7	+	1.15E-01	0.000	0.000	0.000	0.000	0.001
2.8.1.6|g__Firmicutes_unclassified.s__Firmicutes_bacterium_AM10_47	-	5.30E-01	0.000	0.000	0.000	0.001	0.000
2.8.1.6|g__Fusobacterium.s__Fusobacterium_mortiferum	-	3.09E-01	0.000	0.000	0.000	0.000	0.000
2.8.1.6|g__Gordonibacter.s__Gordonibacter_pamelaeae	+	6.04E-01	0.000	0.000	0.000	0.000	0.001
2.8.1.6|g__Haemophilus.s__Haemophilus_parainfluenzae	-	2.78E-01	0.000	0.000	0.000	0.003	0.000
2.8.1.6|g__Hafnia.s__Hafnia_alvei	+	1.83E-01	0.000	0.000	0.000	0.000	0.001
2.8.1.6|g__Hafnia.s__Hafnia_paralvei	+	3.59E-01	0.000	0.000	0.000	0.000	0.002
2.8.1.6|g__Hungatella.s__Hungatella_effluvii	+	6.32E-01	0.000	0.000	0.000	0.000	0.000
2.8.1.6|g__Hungatella.s__Hungatella_hathewayi	+	6.32E-01	0.000	0.000	0.000	0.000	0.000
2.8.1.6|g__Klebsiella.s__Klebsiella_aerogenes	-	9.42E-01	0.000	0.000	0.000	0.000	0.000
2.8.1.6|g__Klebsiella.s__Klebsiella_pneumoniae	-	1.20E-01	0.000	0.000	0.000	0.004	0.000
2.8.1.6|g__Klebsiella.s__Klebsiella_variicola	+	6.32E-01	0.000	0.000	0.000	0.000	0.000
2.8.1.6|g__Lachnoclostridium.s__Clostridium_saccharolyticum	-	2.96E-02	0.000	0.000	0.000	0.001	0.000
2.8.1.6|g__Lachnoclostridium.s__Clostridium_scindens	-	3.22E-02	0.000	0.000	0.000	0.000	0.000
2.8.1.6|g__Lachnoclostridium.s__Clostridium_symbiosum	-	2.58E-01	0.000	0.000	0.000	0.001	0.000
2.8.1.6|g__Lachnospira.s__Lachnospira_pectinoschiza	+	8.26E-01	0.000	0.000	0.000	0.002	0.004
2.8.1.6|g__Lactobacillus.s__Lactobacillus_rogosae	+	9.63E-01	0.000	0.000	0.000	0.002	0.003
2.8.1.6|g__Megamonas.s__Megamonas_funiformis	+	3.59E-01	0.000	0.000	0.000	0.000	0.001
2.8.1.6|g__Megamonas.s__Megamonas_hypermegale	+	3.59E-01	0.000	0.000	0.000	0.000	0.001
2.8.1.6|g__Megamonas.s__Megamonas_rupellensis	+	3.59E-01	0.000	0.000	0.000	0.000	0.000
2.8.1.6|g__Megasphaera.s__Megasphaera_elsdenii	-	4.85E-01	0.000	0.000	0.000	0.001	0.000
2.8.1.6|g__Megasphaera.s__Megasphaera_hexanoica	-	3.09E-01	0.000	0.000	0.000	0.000	0.000
2.8.1.6|g__Megasphaera.s__Megasphaera_sp_DISK_18	+	3.59E-01	0.000	0.000	0.000	0.000	0.001
2.8.1.6|g__Mitsuokella.s__Mitsuokella_jalaludinii	-	5.11E-01	0.000	0.000	0.000	0.003	0.001
2.8.1.6|g__Mitsuokella.s__Mitsuokella_multacida	-	3.09E-01	0.000	0.000	0.000	0.000	0.000
2.8.1.6|g__Obesumbacterium.s__Obesumbacterium_proteus	+	1.83E-01	0.000	0.000	0.000	0.000	0.001
2.8.1.6|g__Oxalobacter.s__Oxalobacter_formigenes	+	2.32E-01	0.000	0.000	0.000	0.000	0.000
2.8.1.6|g__Parabacteroides.s__Parabacteroides_goldsteinii	-	8.28E-01	0.000	0.000	0.000	0.001	0.000
2.8.1.6|g__Parabacteroides.s__Parabacteroides_gordonii	+	1.83E-01	0.000	0.000	0.000	0.000	0.000
2.8.1.6|g__Parabacteroides.s__Parabacteroides_johnsonii	-	2.91E-01	0.000	0.000	0.000	0.002	0.000
2.8.1.6|g__Paraprevotella.s__Paraprevotella_clara	-	2.12E-02	0.000	0.000	0.000	0.006	0.002
2.8.1.6|g__Phascolarctobacterium.s__Phascolarctobacterium_faecium	+	3.10E-01	0.000	0.000	0.000	0.006	0.011
2.8.1.6|g__Phascolarctobacterium.s__Phascolarctobacterium_succinatutens	-	4.20E-02	0.000	0.000	0.000	0.024	0.003
2.8.1.6|g__Pseudoflavonifractor.s__Pseudoflavonifractor_sp_An184	+	8.29E-01	0.000	0.000	0.000	0.001	0.001
2.8.1.6|g__Pseudomonas.s__Pseudomonas_aeruginosa_group	-	3.09E-01	0.000	0.000	0.000	0.000	0.000
2.8.1.6|g__Pseudomonas.s__Pseudomonas_alcaligenes	-	3.09E-01	0.000	0.000	0.000	0.000	0.000
2.8.1.6|g__Ruminococcaceae_unclassified.s__Ruminococcaceae_bacterium_	-	1.36E-01	0.000	0.000	0.000	0.001	0.000
2.8.1.6|g__Ruminococcaceae_unclassified.s__Ruminococcaceae_bacterium_	-	9.42E-01	0.000	0.000	0.000	0.000	0.000
2.8.1.6|g__Ruminococcus.s__Ruminococcus_champanellensis	+	3.59E-01	0.000	0.000	0.000	0.000	0.001
2.8.1.6|g__Ruminococcus.s__Ruminococcus_lactaris	+	1.89E-01	0.000	0.000	0.000	0.004	0.008
2.8.1.6|g__Sanguibacteroides.s__Sanguibacteroides_justesenii	-	9.81E-01	0.000	0.000	0.000	0.000	0.000
2.8.1.6|g__Staphylococcus.s__Staphylococcus_saprophyticus	+	3.59E-01	0.000	0.000	0.000	0.000	0.000
2.8.1.6|g__Streptococcus.s__Streptococcus_equinus	-	3.09E-01	0.000	0.000	0.000	0.000	0.000
2.8.1.6|g__Streptococcus.s__Streptococcus_gallolyticus	-	3.09E-01	0.000	0.000	0.000	0.000	0.000
2.8.1.6|g__Streptococcus.s__Streptococcus_pasteurianus	-	3.09E-01	0.000	0.000	0.000	0.000	0.000
2.8.1.6|g__Streptococcus.s__Streptococcus_pneumoniae	-	9.42E-01	0.000	0.000	0.000	0.000	0.000
2.8.1.6|g__Sutterella.s__Sutterella_wadsworthensis	-	6.70E-01	0.000	0.000	0.000	0.008	0.005

	2.8.1.6|g__Veillonella.s__Veillonella_atypica	-	3.09E-01	0.000	0.000	0.000	0.000	0.000
	2.8.1.6|g__Veillonella.s__Veillonella_dispar	-	5.50E-01	0.000	0.000	0.000	0.000	0.000
	2.8.1.6|g__Veillonella.s__Veillonella_infantium	-	2.73E-01	0.000	0.000	0.000	0.000	0.000
	2.8.1.6|g__Veillonella.s__Veillonella_parvula	+	1.83E-01	0.000	0.000	0.000	0.000	0.000
	2.8.1.6|g__Veillonella.s__Veillonella_rogosae	-	9.42E-01	0.000	0.000	0.000	0.000	0.000
	2.8.1.6|g__Victivallales_unclassified.s__Victivallales_bacterium_CCUG_44	+	9.13E-01	0.000	0.000	0.000	0.001	0.001
	2.8.1.6|g__Victivallis.s__Victivallis_vadensis	-	9.65E-01	0.000	0.000	0.000	0.000	0.000
	2.8.1.6|g__Bacteroides.s__Bacteroides_ovatus	-	5.68E-01	0.300	0.310	0.009	0.008	0.012
	2.8.1.6|g__Odoribacter.s__Odoribacter_splanchnicus	+	7.31E-01	0.456	0.491	0.035	0.006	0.006
	2.8.1.6|g__Dorea.s__Dorea_longicatena	+	6.57E-01	0.387	0.423	0.037	0.008	0.008
	2.8.1.6|g__Clostridiales_unclassified.s__Clostridiales_bacterium_KLE1615	+	5.33E-01	0.136	0.201	0.065	0.003	0.004
	2.8.1.6|g__Ruminococcaceae_unclassified.s__Eubacterium_siraeum	+	1.06E-01	0.478	0.559	0.080	0.013	0.032
	2.8.1.6|g__Lawsonibacter.s__Lawsonibacter_asaccharolyticus	+	4.38E-01	0.218	0.325	0.107	0.005	0.007
	2.8.1.6|g__Bacteroides.s__Bacteroides_caccae	-	5.60E-01	0.686	0.819	0.133	0.017	0.012
	2.8.1.6|g__Agathobaculum.s__Agathobaculum_butyriciproducens	+	4.62E-01	0.000	0.163	0.163	0.002	0.003
	2.8.1.6|g__Eubacterium.s__Eubacterium_eligens	+	7.19E-01	0.606	0.819	0.213	0.021	0.016
	2.8.1.6|g__Barnesiella.s__Barnesiella_intestinihominis	+	5.00E-01	0.581	0.806	0.226	0.013	0.019
	2.8.1.6|g__Bilophila.s__Bilophila_wadsworthia	+	5.80E-02	0.000	0.226	0.226	0.002	0.004
	2.8.1.6|g__Bacteroides.s__Bacteroides_cellulosilyticus	+	5.65E-01	0.395	0.673	0.278	0.017	0.019
	2.8.1.6|g__Bacteroides.s__Bacteroides_vulgatus	-	7.56E-01	2.498	2.798	0.300	0.067	0.060
	2.8.1.6|g__Parabacteroides.s__Parabacteroides_merdae	+	7.06E-01	0.427	0.830	0.403	0.011	0.012
	2.8.1.6|g__Clostridium.s__Clostridium_sp_AF36_4	+	9.39E-03	0.000	0.436	0.436	0.003	0.008
	2.8.1.6|g__Bacteroides.s__Bacteroides_uniformis	+	5.29E-01	2.645	3.104	0.459	0.053	0.062
	2.8.1.6|g__Anaerostipes.s__Anaerostipes_hadrus	+	1.77E-01	0.356	0.896	0.540	0.012	0.021
	2.8.1.6|g__Akkermansia.s__Akkermansia_muciniphila	+	1.27E-03	0.000	2.291	2.291	0.016	0.088
	2.8.1.6|unclassified	+	1.09E-01	11.626	14.450	2.823	0.178	0.218
China1	2.8.1.6	-	1.09E-01	89.346	80.226	-9.120	1.000	1.000
	2.8.1.6|g__Bacteroides.s__Bacteroides_vulgatus	-	7.58E-03	10.836	2.602	-8.234	0.170	0.082
	2.8.1.6|g__Bacteroides.s__Bacteroides_fragilis	-	1.40E-01	2.022	0.973	-1.049	0.042	0.030
	2.8.1.6|g__Bacteroides.s__Bacteroides_ovatus	-	2.72E-02	1.949	1.040	-0.909	0.042	0.020
	2.8.1.6|g__Escherichia.s__Escherichia_coli	-	5.98E-01	0.171	0.082	-0.089	0.022	0.024
	2.8.1.6|g__Blautia.s__Ruminococcus_gnavus	-	4.33E-02	0.058	0.000	-0.058	0.004	0.001
	2.8.1.6|g__Bacteroides.s__Bacteroides_thetaiotaomicron	-	8.97E-01	1.254	1.208	-0.046	0.027	0.037
	2.8.1.6|g__Roseburia.s__Roseburia_faecis	-	5.92E-01	0.177	0.140	-0.037	0.008	0.008
	2.8.1.6|g__Blautia.s__Blautia_obeum	-	8.59E-01	0.075	0.056	-0.019	0.002	0.002
	2.8.1.6|g__Acidaminococcus.s__Acidaminococcus_intestini	+	3.30E-01	0.000	0.000	0.000	0.000	0.000
	2.8.1.6|g__Aeromonas.s__Aeromonas_diversa	-	3.30E-01	0.000	0.000	0.000	0.000	0.000
	2.8.1.6|g__Aeromonas.s__Aeromonas_hydrophila	-	3.30E-01	0.000	0.000	0.000	0.000	0.000
	2.8.1.6|g__Agathobaculum.s__Agathobaculum_butyriciproducens	-	2.70E-02	0.000	0.000	0.000	0.001	0.000
	2.8.1.6|g__Aggregatibacter.s__Aggregatibacter_segnis	-	1.00E+00	0.000	0.000	0.000	0.000	0.000
	2.8.1.6|g__Akkermansia.s__Akkermansia_muciniphila	+	7.43E-03	0.000	0.000	0.000	0.001	0.007
	2.8.1.6|g__Allisonella.s__Allisonella_histaminiformans	+	2.85E-01	0.000	0.000	0.000	0.000	0.000
	2.8.1.6|g__Anaeroglobus.s__Anaeroglobus_geminatus	+	5.89E-01	0.000	0.000	0.000	0.000	0.000
	2.8.1.6|g__Anaerostipes.s__Anaerostipes_caccae	-	3.30E-01	0.000	0.000	0.000	0.000	0.000
	2.8.1.6|g__Anaerostipes.s__Anaerostipes_hadrus	+	5.57E-01	0.000	0.000	0.000	0.002	0.002
	2.8.1.6|g__Anaerotruncus.s__Anaerotruncus_colihominis	+	9.78E-01	0.000	0.000	0.000	0.000	0.000
	2.8.1.6|g__Bacteroides.s__Bacteroides_clarus	+	1.43E-01	0.000	0.000	0.000	0.001	0.005
	2.8.1.6|g__Bacteroides.s__Bacteroides_coprocola	-	2.12E-01	0.000	0.000	0.000	0.047	0.018
	2.8.1.6|g__Bacteroides.s__Bacteroides_coprophilus	-	6.38E-01	0.000	0.000	0.000	0.007	0.001
	2.8.1.6|g__Bacteroides.s__Bacteroides_eggerthii	+	1.47E-01	0.000	0.000	0.000	0.018	0.031
	2.8.1.6|g__Bacteroides.s__Bacteroides_faecichinchillae	+	8.20E-02	0.000	0.000	0.000	0.000	0.000
	2.8.1.6|g__Bacteroides.s__Bacteroides_faecis	-	1.58E-01	0.000	0.000	0.000	0.001	0.000
	2.8.1.6|g__Bacteroides.s__Bacteroides_finegoldii	+	3.21E-01	0.000	0.000	0.000	0.007	0.013
	2.8.1.6|g__Bacteroides.s__Bacteroides_fluxus	+	3.30E-01	0.000	0.000	0.000	0.000	0.000
	2.8.1.6|g__Bacteroides.s__Bacteroides_massiliensis	+	6.51E-01	0.000	0.000	0.000	0.010	0.014
	2.8.1.6|g__Bacteroides.s__Bacteroides_oleiciplenus	+	6.55E-01	0.000	0.000	0.000	0.000	0.000
	2.8.1.6|g__Bacteroides.s__Bacteroides_plebeius	-	4.45E-01	0.000	0.000	0.000	0.067	0.023
	2.8.1.6|g__Bacteroides.s__Bacteroides_salyersiae	-	7.85E-01	0.000	0.000	0.000	0.002	0.002
	2.8.1.6|g__Bacteroides.s__Bacteroides_sp_OM08_11	+	8.20E-02	0.000	0.000	0.000	0.000	0.000
	2.8.1.6|g__Bacteroides.s__Bacteroides_stercorirosoris	+	9.70E-01	0.000	0.000	0.000	0.000	0.000
	2.8.1.6|g__Barnesiella.s__Barnesiella_intestinihominis	+	2.14E-01	0.000	0.000	0.000	0.010	0.009
	2.8.1.6|g__Barnesiella.s__Barnesiella_sp_An22	-	3.30E-01	0.000	0.000	0.000	0.000	0.000
	2.8.1.6|g__Blautia.s__Blautia_coccoides	+	1.00E+00	0.000	0.000	0.000	0.000	0.000
	2.8.1.6|g__Blautia.s__Blautia_sp_AF19_10LB	-	7.16E-01	0.000	0.000	0.000	0.000	0.000
	2.8.1.6|g__Butyricimonas.s__Butyricimonas_synergistica	-	3.30E-01	0.000	0.000	0.000	0.000	0.000
	2.8.1.6|g__Butyricimonas.s__Butyricimonas_virosa	-	6.91E-01	0.000	0.000	0.000	0.003	0.003
	2.8.1.6|g__Butyrivibrio.s__Butyrivibrio_crossotus	-	3.30E-01	0.000	0.000	0.000	0.000	0.000
	2.8.1.6|g__Campylobacter.s__Campylobacter_concisus	-	3.30E-01	0.000	0.000	0.000	0.000	0.000
	2.8.1.6|g__Campylobacter.s__Campylobacter_gracilis	+	3.30E-01	0.000	0.000	0.000	0.000	0.000
	2.8.1.6|g__Catabacter.s__Catabacter_hongkongensis	-	3.30E-01	0.000	0.000	0.000	0.000	0.000
	2.8.1.6|g__Catenibacterium.s__Catenibacterium_mitsuokai	+	6.06E-01	0.000	0.000	0.000	0.001	0.006
	2.8.1.6|g__Citrobacter.s__Citrobacter_amalonaticus	-	3.30E-01	0.000	0.000	0.000	0.000	0.000

2.8.1.6|g__Citrobacter.s__Citrobacter_farmeri	+	1.00E+00	0.000	0.000	0.000	0.000	0.000
2.8.1.6|g__Citrobacter.s__Citrobacter_freundii	+	4.28E-02	0.000	0.000	0.000	0.000	0.000
2.8.1.6|g__Citrobacter.s__Citrobacter_portucalensis	+	9.70E-01	0.000	0.000	0.000	0.000	0.000
2.8.1.6|g__Citrobacter.s__Citrobacter_youngae	+	6.22E-01	0.000	0.000	0.000	0.000	0.000
2.8.1.6|g__Cloacibacillus.s__Cloacibacillus_porcorum	+	8.20E-02	0.000	0.000	0.000	0.000	0.000
2.8.1.6|g__Clostridiales_unclassified.s__Clostridiales_bacterium_KLE1615	+	8.64E-01	0.000	0.000	0.000	0.001	0.001
2.8.1.6|g__Clostridioides.s__Clostridioides_difficile	-	3.30E-01	0.000	0.000	0.000	0.000	0.000
2.8.1.6|g__Clostridium.s__Clostridium_neonatale	-	8.20E-02	0.000	0.000	0.000	0.000	0.000
2.8.1.6|g__Clostridium.s__Clostridium_perfringens	+	3.30E-01	0.000	0.000	0.000	0.000	0.000
2.8.1.6|g__Clostridium.s__Clostridium_sp_AF36_4	+	4.18E-02	0.000	0.000	0.000	0.001	0.004
2.8.1.6|g__Clostridium.s__Clostridium_sp_chh4_2	+	3.30E-01	0.000	0.000	0.000	0.000	0.000
2.8.1.6|g__Coprobacter.s__Coprobacter_fastidiosus	+	5.77E-01	0.000	0.000	0.000	0.002	0.001
2.8.1.6|g__Coprobacter.s__Coprobacter_secundus	+	6.93E-01	0.000	0.000	0.000	0.000	0.001
2.8.1.6|g__Coprococcus.s__Coprococcus_comes	+	2.63E-01	0.000	0.000	0.000	0.001	0.002
2.8.1.6|g__Coprococcus.s__Coprococcus_eutactus	+	3.62E-01	0.000	0.000	0.000	0.001	0.003
2.8.1.6|g__Desulfovibrio.s__Desulfovibrio_piger	+	1.65E-01	0.000	0.000	0.000	0.000	0.000
2.8.1.6|g__Desulfovibrio.s__Desulfovibrio_sp_AM18_2	+	8.20E-02	0.000	0.000	0.000	0.000	0.002
2.8.1.6|g__Desulfovibrionaceae_unclassified.s__Desulfovibrionaceae_bacte	+	1.60E-01	0.000	0.000	0.000	0.000	0.000
2.8.1.6|g__Dialister.s__Dialister_invisus	+	6.69E-01	0.000	0.000	0.000	0.005	0.013
2.8.1.6|g__Dialister.s__Dialister_succinatiphilus	-	5.89E-01	0.000	0.000	0.000	0.001	0.003
2.8.1.6|g__Dorea.s__Dorea_formicigenerans	-	9.26E-01	0.000	0.000	0.000	0.001	0.000
2.8.1.6|g__Dorea.s__Dorea_longicatena	+	4.67E-01	0.000	0.000	0.000	0.001	0.001
2.8.1.6|g__Dorea.s__Dorea_sp_OM02_2LB	+	3.30E-01	0.000	0.000	0.000	0.000	0.000
2.8.1.6|g__Eisenbergiella.s__Eisenbergiella_tayi	+	6.17E-03	0.000	0.000	0.000	0.000	0.000
2.8.1.6|g__Enterobacter.s__Enterobacter_cloacae	+	3.30E-01	0.000	0.000	0.000	0.000	0.000
2.8.1.6|g__Enterobacter.s__Enterobacter_kobei	+	3.30E-01	0.000	0.000	0.000	0.000	0.000
2.8.1.6|g__Enterococcus.s__Enterococcus_hirae	+	3.30E-01	0.000	0.000	0.000	0.000	0.000
2.8.1.6|g__Erysipelatoclostridium.s__Clostridium_innocuum	+	1.60E-01	0.000	0.000	0.000	0.000	0.000
2.8.1.6|g__Erysipelatoclostridium.s__Erysipelatoclostridium_ramosum	-	1.22E-01	0.000	0.000	0.000	0.000	0.000
2.8.1.6|g__Escherichia.s__Escherichia_fergusonii	+	9.50E-01	0.000	0.000	0.000	0.000	0.000
2.8.1.6|g__Eubacterium.s__Eubacterium_ramulus	-	6.71E-01	0.000	0.000	0.000	0.000	0.000
2.8.1.6|g__Eubacterium.s__Eubacterium_sp_AF17_7	+	1.67E-01	0.000	0.000	0.000	0.000	0.001
2.8.1.6|g__Faecalicatena.s__Faecalicatena_contorta	+	3.30E-01	0.000	0.000	0.000	0.000	0.000
2.8.1.6|g__Firmicutes_unclassified.s__Firmicutes_bacterium_AM10_47	+	6.38E-01	0.000	0.000	0.000	0.000	0.000
2.8.1.6|g__Fusobacterium.s__Fusobacterium_hwasookii	-	3.30E-01	0.000	0.000	0.000	0.000	0.000
2.8.1.6|g__Fusobacterium.s__Fusobacterium_mortiferum	+	3.33E-01	0.000	0.000	0.000	0.001	0.000
2.8.1.6|g__Fusobacterium.s__Fusobacterium_ulcerans	-	6.88E-01	0.000	0.000	0.000	0.001	0.001
2.8.1.6|g__Fusobacterium.s__Fusobacterium_varium	-	5.49E-01	0.000	0.000	0.000	0.001	0.000
2.8.1.6|g__Haemophilus.s__Haemophilus_parainfluenzae	+	9.82E-01	0.000	0.000	0.000	0.004	0.004
2.8.1.6|g__Haemophilus.s__Haemophilus_sputorum	+	1.00E+00	0.000	0.000	0.000	0.000	0.000
2.8.1.6|g__Hafnia.s__Hafnia_paralvei	-	3.30E-01	0.000	0.000	0.000	0.000	0.000
2.8.1.6|g__Hungatella.s__Hungatella_effluvii	-	4.63E-01	0.000	0.000	0.000	0.000	0.000
2.8.1.6|g__Hungatella.s__Hungatella_hathewayi	-	3.14E-01	0.000	0.000	0.000	0.000	0.000
2.8.1.6|g__Klebsiella.s__Klebsiella_aerogenes	+	1.00E+00	0.000	0.000	0.000	0.000	0.000
2.8.1.6|g__Klebsiella.s__Klebsiella_oxytoca	+	2.25E-02	0.000	0.000	0.000	0.000	0.001
2.8.1.6|g__Klebsiella.s__Klebsiella_pneumoniae	+	1.60E-01	0.000	0.000	0.000	0.001	0.003
2.8.1.6|g__Klebsiella.s__Klebsiella_variicola	+	4.28E-02	0.000	0.000	0.000	0.000	0.000
2.8.1.6|g__Kosakonia.s__Kosakonia_cowanii	-	3.30E-01	0.000	0.000	0.000	0.000	0.000
2.8.1.6|g__Lachnoclostridium.s__Clostridium_citroniae	+	1.80E-01	0.000	0.000	0.000	0.000	0.000
2.8.1.6|g__Lachnoclostridium.s__Clostridium_saccharolyticum	+	1.00E+00	0.000	0.000	0.000	0.000	0.000
2.8.1.6|g__Lachnoclostridium.s__Clostridium_scindens	-	3.30E-01	0.000	0.000	0.000	0.000	0.000
2.8.1.6|g__Lachnoclostridium.s__Clostridium_symbiosum	+	8.63E-01	0.000	0.000	0.000	0.001	0.001
2.8.1.6|g__Lachnospira.s__Lachnospira_pectinoschiza	-	4.77E-01	0.000	0.000	0.000	0.006	0.003
2.8.1.6|g__Lactobacillus.s__Lactobacillus_rogosae	-	4.81E-01	0.000	0.000	0.000	0.003	0.002
2.8.1.6|g__Lawsonibacter.s__Lawsonibacter_asaccharolyticus	+	5.66E-01	0.000	0.000	0.000	0.000	0.000
2.8.1.6|g__Megamonas.s__Megamonas_funiformis	+	8.05E-01	0.000	0.000	0.000	0.007	0.003
2.8.1.6|g__Megamonas.s__Megamonas_hypermegale	+	9.94E-01	0.000	0.000	0.000	0.000	0.000
2.8.1.6|g__Megamonas.s__Megamonas_rupellensis	+	6.58E-01	0.000	0.000	0.000	0.001	0.000
2.8.1.6|g__Megasphaera.s__Megasphaera_micronuciformis	-	3.30E-01	0.000	0.000	0.000	0.000	0.000
2.8.1.6|g__Oxalobacter.s__Oxalobacter_formigenes	+	6.55E-01	0.000	0.000	0.000	0.000	0.001
2.8.1.6|g__Parabacteroides.s__Parabacteroides_goldsteinii	+	6.45E-02	0.000	0.000	0.000	0.001	0.006
2.8.1.6|g__Parabacteroides.s__Parabacteroides_gordonii	+	2.59E-01	0.000	0.000	0.000	0.001	0.001
2.8.1.6|g__Parabacteroides.s__Parabacteroides_johnsonii	+	1.44E-01	0.000	0.000	0.000	0.001	0.003
2.8.1.6|g__Paraprevotella.s__Paraprevotella_clara	+	1.80E-02	0.000	0.000	0.000	0.002	0.007
2.8.1.6|g__Paraprevotella.s__Paraprevotella_xylaniphila	+	9.70E-01	0.000	0.000	0.000	0.001	0.001
2.8.1.6|g__Phascolarctobacterium.s__Phascolarctobacterium_succinatutens	+	1.97E-01	0.000	0.000	0.000	0.005	0.009
2.8.1.6|g__Pseudocitrobacter.s__Pseudocitrobacter_faecalis	+	3.30E-01	0.000	0.000	0.000	0.000	0.000
2.8.1.6|g__Raoultella.s__Raoultella_ornithinolytica	-	3.30E-01	0.000	0.000	0.000	0.000	0.000
2.8.1.6|g__Roseburia.s__Roseburia_intestinalis	-	1.94E-01	0.000	0.000	0.000	0.002	0.001
2.8.1.6|g__Ruminococcaceae_unclassified.s__Eubacterium_siraeum	+	5.56E-01	0.000	0.000	0.000	0.003	0.001
2.8.1.6|g__Ruminococcaceae_unclassified.s__Ruminococcaceae_bacterium_	-	3.30E-01	0.000	0.000	0.000	0.000	0.000
2.8.1.6|g__Ruminococcaceae_unclassified.s__Ruminococcaceae_bacterium_	+	1.58E-01	0.000	0.000	0.000	0.000	0.001

	2.8.1.6|g__Ruminococcus.s__Ruminococcus_champanellensis	+	1.60E-01	0.000	0.000	0.000	0.000	0.000
	2.8.1.6|g__Ruminococcus.s__Ruminococcus_lactaris	-	7.51E-01	0.000	0.000	0.000	0.001	0.002
	2.8.1.6|g__Salmonella.s__Salmonella_enterica	+	3.30E-01	0.000	0.000	0.000	0.000	0.000
	2.8.1.6|g__Sanguibacteroides.s__Sanguibacteroides_justesenii	+	6.22E-01	0.000	0.000	0.000	0.000	0.000
	2.8.1.6|g__Succinatimonas.s__Succinatimonas_hippei	-	3.30E-01	0.000	0.000	0.000	0.000	0.000
	2.8.1.6|g__Sutterella.s__Sutterella_parvirubra	-	3.30E-01	0.000	0.000	0.000	0.000	0.000
	2.8.1.6|g__Sutterella.s__Sutterella_wadsworthensis	+	2.30E-01	0.000	0.000	0.000	0.002	0.004
	2.8.1.6|g__Veillonella.s__Veillonella_atypica	+	1.32E-01	0.000	0.000	0.000	0.001	0.002
	2.8.1.6|g__Veillonella.s__Veillonella_denticariosi	-	3.30E-01	0.000	0.000	0.000	0.000	0.000
	2.8.1.6|g__Veillonella.s__Veillonella_dispar	+	4.99E-01	0.000	0.000	0.000	0.002	0.001
	2.8.1.6|g__Veillonella.s__Veillonella_infantium	+	5.66E-01	0.000	0.000	0.000	0.004	0.000
	2.8.1.6|g__Veillonella.s__Veillonella_parvula	+	3.88E-01	0.000	0.000	0.000	0.003	0.002
	2.8.1.6|g__Veillonella.s__Veillonella_rogosae	+	1.05E-01	0.000	0.000	0.000	0.000	0.000
	2.8.1.6|g__Veillonella.s__Veillonella_tobetsuensis	+	2.96E-01	0.000	0.000	0.000	0.000	0.000
	2.8.1.6|g__Victivallales_unclassified.s__Victivallales_bacterium_CCUG_44	+	1.65E-01	0.000	0.000	0.000	0.000	0.002
	2.8.1.6|g__Victivallis.s__Victivallis_vadensis	+	5.69E-01	0.000	0.000	0.000	0.000	0.000
	2.8.1.6|g__Parabacteroides.s__Parabacteroides_distasonis	+	5.54E-01	1.193	1.194	0.000	0.030	0.025
	2.8.1.6|g__Blautia.s__Blautia_wexlerae	+	8.60E-01	0.019	0.052	0.033	0.001	0.001
	2.8.1.6|g__Alistipes.s__Alistipes_indistinctus	+	2.14E-02	0.000	0.038	0.038	0.003	0.005
	2.8.1.6|g__Bilophila.s__Bilophila_wadsworthia	+	2.23E-01	0.058	0.101	0.043	0.002	0.002
	2.8.1.6|g__Blautia.s__Ruminococcus_torques	+	7.88E-01	0.014	0.069	0.055	0.005	0.006
	2.8.1.6|g__Bacteroides.s__Bacteroides_nordii	+	7.59E-01	0.054	0.124	0.070	0.004	0.006
	2.8.1.6|g__Bacteroides.s__Bacteroides_stercoris	+	7.60E-01	0.705	0.803	0.099	0.062	0.043
	2.8.1.6|g__Roseburia.s__Roseburia_inulinivorans	+	1.51E-01	0.048	0.229	0.181	0.006	0.007
	2.8.1.6|g__Bacteroides.s__Bacteroides_xylanisolvens	+	9.19E-01	0.325	0.509	0.184	0.020	0.017
	2.8.1.6|g__Lachnospiraceae_unclassified.s__Eubacterium_rectale	-	8.61E-01	0.055	0.241	0.186	0.007	0.006
	2.8.1.6|g__Odoribacter.s__Odoribacter_splanchnicus	+	9.06E-01	0.000	0.227	0.227	0.007	0.006
	2.8.1.6|g__Bacteroides.s__Bacteroides_dorei	+	8.58E-01	0.279	0.510	0.231	0.058	0.037
	2.8.1.6|g__Eubacterium.s__Eubacterium_eligens	+	8.08E-02	0.273	0.568	0.295	0.014	0.024
	2.8.1.6|g__Parabacteroides.s__Parabacteroides_merdae	+	2.17E-01	0.071	0.499	0.428	0.013	0.020
	2.8.1.6|g__Bacteroides.s__Bacteroides_cellulosilyticus	+	5.76E-01	0.832	1.280	0.448	0.019	0.031
	2.8.1.6|g__Phascolarctobacterium.s__Phascolarctobacterium_faecium	+	4.70E-01	0.612	1.074	0.463	0.025	0.027
	2.8.1.6|g__Bacteroides.s__Bacteroides_caccae	+	4.31E-01	0.193	0.678	0.485	0.014	0.022
	2.8.1.6|g__Bacteroides.s__Bacteroides_intestinalis	+	2.90E-02	0.031	0.609	0.578	0.007	0.020
	2.8.1.6|unclassified	+	2.46E-02	3.780	5.412	1.632	0.059	0.128
	2.8.1.6|g__Bacteroides.s__Bacteroides_uniformis	+	1.92E-01	4.581	7.749	3.168	0.078	0.126
China2	2.8.1.6	-	8.03E-02	108.276	99.106	-9.170	1.000	1.000
	2.8.1.6|g__Bacteroides.s__Bacteroides_vulgatus	-	7.13E-02	7.792	5.023	-2.769	0.138	0.096
	2.8.1.6|g__Roseburia.s__Roseburia_faecis	-	1.90E-01	1.082	0.182	-0.900	0.031	0.016
	2.8.1.6|g__Phascolarctobacterium.s__Phascolarctobacterium_faecium	-	5.18E-01	0.922	0.264	-0.658	0.012	0.012
	2.8.1.6|g__Bacteroides.s__Bacteroides_ovatus	+	8.34E-01	0.964	0.601	-0.363	0.017	0.016
	2.8.1.6|g__Bacteroides.s__Bacteroides_dorei	-	2.40E-01	0.525	0.336	-0.189	0.042	0.026
	2.8.1.6|g__Roseburia.s__Roseburia_inulinivorans	-	2.99E-01	0.388	0.228	-0.160	0.007	0.005
	2.8.1.6|g__Blautia.s__Ruminococcus_gnavus	-	4.52E-01	0.195	0.036	-0.159	0.007	0.005
	2.8.1.6|g__Bacteroides.s__Bacteroides_thetaiotaomicron	-	2.42E-01	0.448	0.300	-0.148	0.013	0.008
	2.8.1.6|g__Bacteroides.s__Bacteroides_xylanisolvens	-	6.83E-02	0.271	0.166	-0.105	0.014	0.006
	2.8.1.6|g__Roseburia.s__Roseburia_intestinalis	-	8.07E-01	0.098	0.000	-0.098	0.013	0.008
	2.8.1.6|g__Veillonella.s__Veillonella_parvula	-	3.31E-01	0.087	0.000	-0.087	0.003	0.004
	2.8.1.6|g__Blautia.s__Blautia_obeum	+	7.30E-01	0.804	0.733	-0.071	0.016	0.027
	2.8.1.6|g__Lachnospiraceae_unclassified.s__Eubacterium_rectale	-	7.90E-01	0.261	0.201	-0.060	0.009	0.007
	2.8.1.6|g__Bacteroides.s__Bacteroides_cellulosilyticus	-	8.00E-01	0.045	0.000	-0.045	0.008	0.012
	2.8.1.6|g__Agathobaculum.s__Agathobaculum_butyriciproducens	-	1.11E-01	0.026	0.000	-0.026	0.001	0.000
	2.8.1.6|g__Anaerostipes.s__Anaerostipes_hadrus	-	8.71E-01	0.419	0.398	-0.021	0.010	0.010
	2.8.1.6|g__Dorea.s__Dorea_formicigenerans	-	4.64E-01	0.059	0.051	-0.008	0.001	0.001
	2.8.1.6|g__Parabacteroides.s__Parabacteroides_distasonis	+	5.49E-01	1.085	1.081	-0.004	0.017	0.026
	2.8.1.6|g__Acidaminococcus.s__Acidaminococcus_intestini	-	5.69E-01	0.000	0.000	0.000	0.001	0.001
	2.8.1.6|g__Aggregatibacter.s__Aggregatibacter_segnis	-	3.30E-01	0.000	0.000	0.000	0.000	0.000
	2.8.1.6|g__Akkermansia.s__Akkermansia_muciniphila	+	5.01E-01	0.000	0.000	0.000	0.002	0.009
	2.8.1.6|g__Alcaligenes.s__Alcaligenes_faecalis	+	3.30E-01	0.000	0.000	0.000	0.000	0.001
	2.8.1.6|g__Alistipes.s__Alistipes_indistinctus	+	1.82E-03	0.000	0.000	0.000	0.000	0.002
	2.8.1.6|g__Allisonella.s__Allisonella_histaminiformans	+	7.59E-01	0.000	0.000	0.000	0.000	0.000
	2.8.1.6|g__Anaerostipes.s__Anaerostipes_caccae	-	3.30E-01	0.000	0.000	0.000	0.000	0.000
	2.8.1.6|g__Anaerotignum.s__Anaerotignum_lactatifermentans	-	1.60E-01	0.000	0.000	0.000	0.000	0.000
	2.8.1.6|g__Anaerotruncus.s__Anaerotruncus_colihominis	+	9.58E-02	0.000	0.000	0.000	0.000	0.000
	2.8.1.6|g__Atlantibacter.s__Atlantibacter_hermannii	-	3.30E-01	0.000	0.000	0.000	0.000	0.000
	2.8.1.6|g__Bacteroides.s__Bacteroides_clarus	-	5.86E-01	0.000	0.000	0.000	0.001	0.004
	2.8.1.6|g__Bacteroides.s__Bacteroides_coprocola	-	1.34E-01	0.000	0.000	0.000	0.036	0.011
	2.8.1.6|g__Bacteroides.s__Bacteroides_coprophilus	+	2.85E-01	0.000	0.000	0.000	0.007	0.007
	2.8.1.6|g__Bacteroides.s__Bacteroides_eggerthii	-	3.10E-01	0.000	0.000	0.000	0.009	0.003
	2.8.1.6|g__Bacteroides.s__Bacteroides_faecichinchillae	+	5.49E-01	0.000	0.000	0.000	0.000	0.000
	2.8.1.6|g__Bacteroides.s__Bacteroides_faecis	+	3.93E-01	0.000	0.000	0.000	0.000	0.002
	2.8.1.6|g__Bacteroides.s__Bacteroides_finegoldii	-	1.32E-01	0.000	0.000	0.000	0.011	0.002

2.8.1.6|g__Bacteroides.s__Bacteroides_massiliensis	-	4.72E-01	0.000	0.000	0.000	0.021	0.005
2.8.1.6|g__Bacteroides.s__Bacteroides_nordii	+	2.01E-01	0.000	0.000	0.000	0.001	0.001
2.8.1.6|g__Bacteroides.s__Bacteroides_oleiciplenus	-	3.30E-01	0.000	0.000	0.000	0.000	0.000
2.8.1.6|g__Bacteroides.s__Bacteroides_plebeius	-	4.41E-01	0.000	0.000	0.000	0.075	0.033
2.8.1.6|g__Bacteroides.s__Bacteroides_salyersiae	-	6.25E-01	0.000	0.000	0.000	0.002	0.001
2.8.1.6|g__Bacteroides.s__Bacteroides_sartorii	-	3.30E-01	0.000	0.000	0.000	0.000	0.000
2.8.1.6|g__Bacteroides.s__Bacteroides_stercorirosoris	+	5.89E-01	0.000	0.000	0.000	0.000	0.000
2.8.1.6|g__Barnesiella.s__Barnesiella_intestinihominis	+	7.58E-01	0.000	0.000	0.000	0.004	0.006
2.8.1.6|g__Blautia.s__Blautia_producta	-	5.49E-01	0.000	0.000	0.000	0.000	0.000
2.8.1.6|g__Blautia.s__Blautia_sp_AF19_10LB	+	3.90E-01	0.000	0.000	0.000	0.001	0.003
2.8.1.6|g__Butyricimonas.s__Butyricimonas_synergistica	+	9.83E-01	0.000	0.000	0.000	0.000	0.000
2.8.1.6|g__Butyricimonas.s__Butyricimonas_virosa	+	2.11E-01	0.000	0.000	0.000	0.001	0.003
2.8.1.6|g__Butyrivibrio.s__Butyrivibrio_crossotus	+	3.30E-01	0.000	0.000	0.000	0.000	0.002
2.8.1.6|g__Campylobacter.s__Campylobacter_concisus	-	4.27E-02	0.000	0.000	0.000	0.000	0.000
2.8.1.6|g__Campylobacter.s__Campylobacter_hominis	+	3.30E-01	0.000	0.000	0.000	0.000	0.000
2.8.1.6|g__Catabacter.s__Catabacter_hongkongensis	-	3.30E-01	0.000	0.000	0.000	0.000	0.000
2.8.1.6|g__Catenibacterium.s__Catenibacterium_mitsuokai	+	5.49E-01	0.000	0.000	0.000	0.000	0.000
2.8.1.6|g__Cetobacterium.s__Cetobacterium_somerae	-	3.30E-01	0.000	0.000	0.000	0.000	0.000
2.8.1.6|g__Citrobacter.s__Citrobacter_amalonaticus	-	3.30E-01	0.000	0.000	0.000	0.000	0.000
2.8.1.6|g__Citrobacter.s__Citrobacter_braakii	+	6.21E-01	0.000	0.000	0.000	0.000	0.000
2.8.1.6|g__Citrobacter.s__Citrobacter_freundii	-	5.76E-01	0.000	0.000	0.000	0.000	0.002
2.8.1.6|g__Citrobacter.s__Citrobacter_portucalensis	+	1.00E+00	0.000	0.000	0.000	0.000	0.002
2.8.1.6|g__Citrobacter.s__Citrobacter_werkmanii	+	1.00E+00	0.000	0.000	0.000	0.000	0.000
2.8.1.6|g__Citrobacter.s__Citrobacter_youngae	-	8.04E-01	0.000	0.000	0.000	0.000	0.003
2.8.1.6|g__Cloacibacillus.s__Cloacibacillus_porcorum	+	3.30E-01	0.000	0.000	0.000	0.000	0.000
2.8.1.6|g__Clostridium.s__Clostridium_butyricum	+	3.30E-01	0.000	0.000	0.000	0.000	0.000
2.8.1.6|g__Clostridium.s__Clostridium_disporicum	-	5.30E-02	0.000	0.000	0.000	0.001	0.000
2.8.1.6|g__Clostridium.s__Clostridium_perfringens	-	1.65E-01	0.000	0.000	0.000	0.001	0.000
2.8.1.6|g__Clostridium.s__Clostridium_sp_AF36_4	+	1.32E-01	0.000	0.000	0.000	0.000	0.001
2.8.1.6|g__Comamonas.s__Comamonas_kerstersii	-	1.00E+00	0.000	0.000	0.000	0.000	0.000
2.8.1.6|g__Comamonas.s__Comamonas_testosteroni	+	3.30E-01	0.000	0.000	0.000	0.000	0.000
2.8.1.6|g__Comamonas.s__Comamonas_thiooxydans	+	3.30E-01	0.000	0.000	0.000	0.000	0.000
2.8.1.6|g__Coprobacter.s__Coprobacter_fastidiosus	+	5.86E-01	0.000	0.000	0.000	0.001	0.001
2.8.1.6|g__Coprobacter.s__Coprobacter_secundus	-	4.17E-01	0.000	0.000	0.000	0.000	0.000
2.8.1.6|g__Coprococcus.s__Coprococcus_eutactus	+	1.17E-01	0.000	0.000	0.000	0.005	0.006
2.8.1.6|g__Corynebacterium.s__Corynebacterium_argentoratense	+	3.30E-01	0.000	0.000	0.000	0.000	0.000
2.8.1.6|g__Cronobacter.s__Cronobacter_dublinensis	-	3.30E-01	0.000	0.000	0.000	0.000	0.000
2.8.1.6|g__Desulfovibrio.s__Desulfovibrio_fairfieldensis	+	1.00E+00	0.000	0.000	0.000	0.000	0.000
2.8.1.6|g__Desulfovibrio.s__Desulfovibrio_piger	+	2.03E-01	0.000	0.000	0.000	0.000	0.001
2.8.1.6|g__Desulfovibrio.s__Desulfovibrio_sp_AM18_2	+	8.19E-02	0.000	0.000	0.000	0.000	0.000
2.8.1.6|g__Desulfovibrionaceae_unclassified.s__Desulfovibrionaceae_bacte	+	1.60E-01	0.000	0.000	0.000	0.000	0.000
2.8.1.6|g__Dialister.s__Dialister_invisus	-	7.66E-01	0.000	0.000	0.000	0.003	0.009
2.8.1.6|g__Dialister.s__Dialister_succinatiphilus	+	9.90E-01	0.000	0.000	0.000	0.009	0.017
2.8.1.6|g__Dorea.s__Dorea_longicatena	-	7.20E-01	0.000	0.000	0.000	0.001	0.001
2.8.1.6|g__Dorea.s__Dorea_sp_OM02_2LB	+	5.89E-01	0.000	0.000	0.000	0.000	0.000
2.8.1.6|g__Eisenbergiella.s__Eisenbergiella_tayi	+	8.36E-02	0.000	0.000	0.000	0.000	0.001
2.8.1.6|g__Enterobacter.s__Enterobacter_bugandensis	-	4.59E-01	0.000	0.000	0.000	0.000	0.000
2.8.1.6|g__Enterobacter.s__Enterobacter_cloacae	+	9.05E-01	0.000	0.000	0.000	0.000	0.000
2.8.1.6|g__Enterobacter.s__Enterobacter_kobei	+	1.58E-01	0.000	0.000	0.000	0.000	0.000
2.8.1.6|g__Enterobacter.s__Enterobacter_mori	-	1.60E-01	0.000	0.000	0.000	0.000	0.000
2.8.1.6|g__Enterococcus.s__Enterococcus_hirae	+	3.30E-01	0.000	0.000	0.000	0.000	0.000
2.8.1.6|g__Erysipelatoclostridium.s__Clostridium_spiroforme	+	8.19E-02	0.000	0.000	0.000	0.000	0.000
2.8.1.6|g__Erysipelatoclostridium.s__Erysipelatoclostridium_ramosum	-	2.55E-01	0.000	0.000	0.000	0.000	0.000
2.8.1.6|g__Escherichia.s__Escherichia_fergusonii	+	9.11E-01	0.000	0.000	0.000	0.001	0.000
2.8.1.6|g__Eubacterium.s__Eubacterium_ramulus	+	1.72E-01	0.000	0.000	0.000	0.000	0.001
2.8.1.6|g__Eubacterium.s__Eubacterium_sp_AF17_7	+	3.20E-01	0.000	0.000	0.000	0.000	0.000
2.8.1.6|g__Faecalicatena.s__Faecalicatena_contorta	+	1.65E-01	0.000	0.000	0.000	0.000	0.000
2.8.1.6|g__Firmicutes_unclassified.s__Firmicutes_bacterium_AM10_47	-	2.59E-01	0.000	0.000	0.000	0.002	0.001
2.8.1.6|g__Fusobacterium.s__Fusobacterium_hwasookii	-	3.30E-01	0.000	0.000	0.000	0.000	0.000
2.8.1.6|g__Fusobacterium.s__Fusobacterium_mortiferum	-	6.89E-01	0.000	0.000	0.000	0.001	0.004
2.8.1.6|g__Fusobacterium.s__Fusobacterium_nucleatum	-	5.69E-01	0.000	0.000	0.000	0.000	0.000
2.8.1.6|g__Fusobacterium.s__Fusobacterium_periodonticum	-	1.65E-01	0.000	0.000	0.000	0.000	0.000
2.8.1.6|g__Fusobacterium.s__Fusobacterium_ulcerans	+	1.00E+00	0.000	0.000	0.000	0.000	0.000
2.8.1.6|g__Fusobacterium.s__Fusobacterium_varium	-	4.27E-02	0.000	0.000	0.000	0.001	0.000
2.8.1.6|g__Gordonibacter.s__Gordonibacter_pamelaeae	+	1.72E-01	0.000	0.000	0.000	0.000	0.000
2.8.1.6|g__Haemophilus.s__Haemophilus_parainfluenzae	-	4.43E-01	0.000	0.000	0.000	0.004	0.004
2.8.1.6|g__Haemophilus.s__Haemophilus_paraphrohaemolyticus	-	1.60E-01	0.000	0.000	0.000	0.001	0.000
2.8.1.6|g__Hungatella.s__Hungatella_effluvii	+	1.35E-02	0.000	0.000	0.000	0.000	0.001
2.8.1.6|g__Hungatella.s__Hungatella_hathewayi	+	4.16E-02	0.000	0.000	0.000	0.000	0.002
2.8.1.6|g__Klebsiella.s__Klebsiella_aerogenes	-	9.91E-01	0.000	0.000	0.000	0.001	0.002
2.8.1.6|g__Klebsiella.s__Klebsiella_grimontii	+	1.60E-01	0.000	0.000	0.000	0.000	0.000
2.8.1.6|g__Klebsiella.s__Klebsiella_michiganensis	+	9.83E-01	0.000	0.000	0.000	0.000	0.000

2.8.1.6|g__Klebsiella.s__Klebsiella_oxytoca	+	4.52E-01	0.000	0.000	0.000	0.000	0.000
2.8.1.6|g__Klebsiella.s__Klebsiella_variicola	+	3.62E-01	0.000	0.000	0.000	0.002	0.002
2.8.1.6|g__Kluyvera.s__Kluyvera_cryocrescens	+	3.30E-01	0.000	0.000	0.000	0.000	0.000
2.8.1.6|g__Kluyvera.s__Kluyvera_georgiana	-	1.00E+00	0.000	0.000	0.000	0.000	0.000
2.8.1.6|g__Lachnoclostridium.s__Clostridium_citroniae	+	5.47E-03	0.000	0.000	0.000	0.000	0.001
2.8.1.6|g__Lachnoclostridium.s__Clostridium_saccharolyticum	+	3.30E-01	0.000	0.000	0.000	0.000	0.000
2.8.1.6|g__Lachnoclostridium.s__Clostridium_scindens	-	5.69E-01	0.000	0.000	0.000	0.000	0.000
2.8.1.6|g__Lachnoclostridium.s__Clostridium_symbiosum	+	4.89E-01	0.000	0.000	0.000	0.001	0.001
2.8.1.6|g__Lachnospira.s__Lachnospira_pectinoschiza	-	8.07E-01	0.000	0.000	0.000	0.011	0.008
2.8.1.6|g__Lactobacillus.s__Lactobacillus_rogosae	-	8.24E-01	0.000	0.000	0.000	0.007	0.006
2.8.1.6|g__Leclercia.s__Leclercia_adecarboxylata	-	6.63E-01	0.000	0.000	0.000	0.000	0.000
2.8.1.6|g__Lelliottia.s__Lelliottia_amnigena	-	3.30E-01	0.000	0.000	0.000	0.000	0.000
2.8.1.6|g__Lelliottia.s__Lelliottia_nimipressuralis	-	6.71E-01	0.000	0.000	0.000	0.001	0.002
2.8.1.6|g__Megamonas.s__Megamonas_funiformis	+	8.56E-01	0.000	0.000	0.000	0.030	0.017
2.8.1.6|g__Megamonas.s__Megamonas_hypermegale	-	9.50E-01	0.000	0.000	0.000	0.008	0.002
2.8.1.6|g__Megamonas.s__Megamonas_rupellensis	-	9.32E-01	0.000	0.000	0.000	0.010	0.004
2.8.1.6|g__Megasphaera.s__Megasphaera_elsdenii	+	3.30E-01	0.000	0.000	0.000	0.000	0.000
2.8.1.6|g__Megasphaera.s__Megasphaera_micronuciformis	-	7.06E-01	0.000	0.000	0.000	0.000	0.000
2.8.1.6|g__Megasphaera.s__Megasphaera_stantonii	+	3.30E-01	0.000	0.000	0.000	0.000	0.000
2.8.1.6|g__Mitsuokella.s__Mitsuokella_multacida	+	8.19E-02	0.000	0.000	0.000	0.000	0.002
2.8.1.6|g__Morganella.s__Morganella_morganii	+	1.60E-01	0.000	0.000	0.000	0.000	0.001
2.8.1.6|g__Ochrobactrum.s__Ochrobactrum_cytisi	+	3.30E-01	0.000	0.000	0.000	0.000	0.000
2.8.1.6|g__Ochrobactrum.s__Ochrobactrum_lupini	+	3.30E-01	0.000	0.000	0.000	0.000	0.000
2.8.1.6|g__Oxalobacter.s__Oxalobacter_formigenes	+	2.51E-02	0.000	0.000	0.000	0.000	0.000
2.8.1.6|g__Pantoea.s__Pantoea_dispersa	+	3.30E-01	0.000	0.000	0.000	0.000	0.000
2.8.1.6|g__Pantoea.s__Pantoea_sesami	-	9.40E-01	0.000	0.000	0.000	0.000	0.000
2.8.1.6|g__Parabacteroides.s__Parabacteroides_chinchillae	+	3.30E-01	0.000	0.000	0.000	0.000	0.000
2.8.1.6|g__Parabacteroides.s__Parabacteroides_goldsteinii	+	1.18E-01	0.000	0.000	0.000	0.000	0.002
2.8.1.6|g__Parabacteroides.s__Parabacteroides_gordonii	+	3.30E-01	0.000	0.000	0.000	0.000	0.001
2.8.1.6|g__Parabacteroides.s__Parabacteroides_johnsonii	+	6.79E-01	0.000	0.000	0.000	0.001	0.002
2.8.1.6|g__Paraprevotella.s__Paraprevotella_clara	+	9.58E-01	0.000	0.000	0.000	0.003	0.002
2.8.1.6|g__Paraprevotella.s__Paraprevotella_xylaniphila	-	3.30E-01	0.000	0.000	0.000	0.000	0.000
2.8.1.6|g__Pedobacter.s__Pedobacter_himalayensis	-	2.17E-01	0.000	0.000	0.000	0.000	0.000
2.8.1.6|g__Peptostreptococcus.s__Peptostreptococcus_anaerobius	+	3.30E-01	0.000	0.000	0.000	0.000	0.000
2.8.1.6|g__Phascolarctobacterium.s__Phascolarctobacterium_succinatutens	+	9.92E-01	0.000	0.000	0.000	0.014	0.018
2.8.1.6|g__Proteus.s__Proteus_hauseri	+	3.30E-01	0.000	0.000	0.000	0.000	0.000
2.8.1.6|g__Proteus.s__Proteus_mirabilis	-	3.30E-01	0.000	0.000	0.000	0.000	0.000
2.8.1.6|g__Proteus.s__Proteus_vulgaris	-	5.89E-01	0.000	0.000	0.000	0.000	0.000
2.8.1.6|g__Providencia.s__Providencia_alcalifaciens	+	3.30E-01	0.000	0.000	0.000	0.000	0.000
2.8.1.6|g__Providencia.s__Providencia_rettgeri	+	3.30E-01	0.000	0.000	0.000	0.000	0.006
2.8.1.6|g__Pseudoflavonifractor.s__Pseudoflavonifractor_sp_An184	+	1.60E-01	0.000	0.000	0.000	0.000	0.000
2.8.1.6|g__Raoultella.s__Raoultella_ornithinolytica	+	3.33E-01	0.000	0.000	0.000	0.000	0.000
2.8.1.6|g__Raoultella.s__Raoultella_planticola	+	3.08E-01	0.000	0.000	0.000	0.000	0.000
2.8.1.6|g__Ruminococcaceae_unclassified.s__Eubacterium_siraeum	+	4.26E-01	0.000	0.000	0.000	0.001	0.004
2.8.1.6|g__Ruminococcaceae_unclassified.s__Ruminococcaceae_bacterium_	+	5.89E-01	0.000	0.000	0.000	0.001	0.000
2.8.1.6|g__Ruminococcaceae_unclassified.s__Ruminococcaceae_bacterium_	+	2.24E-02	0.000	0.000	0.000	0.000	0.000
2.8.1.6|g__Ruminococcus.s__Ruminococcus_champanellensis	+	1.00E+00	0.000	0.000	0.000	0.000	0.000
2.8.1.6|g__Ruminococcus.s__Ruminococcus_lactaris	+	5.26E-01	0.000	0.000	0.000	0.001	0.001
2.8.1.6|g__Salmonella.s__Salmonella_enterica	+	3.89E-01	0.000	0.000	0.000	0.000	0.000
2.8.1.6|g__Sanguibacteroides.s__Sanguibacteroides_justesenii	+	3.30E-01	0.000	0.000	0.000	0.000	0.000
2.8.1.6|g__Staphylococcus.s__Staphylococcus_pasteuri	-	3.30E-01	0.000	0.000	0.000	0.000	0.000
2.8.1.6|g__Staphylococcus.s__Staphylococcus_warneri	-	3.30E-01	0.000	0.000	0.000	0.000	0.000
2.8.1.6|g__Streptococcus.s__Streptococcus_equinus	-	3.30E-01	0.000	0.000	0.000	0.000	0.000
2.8.1.6|g__Streptococcus.s__Streptococcus_gallolyticus	-	3.30E-01	0.000	0.000	0.000	0.000	0.000
2.8.1.6|g__Streptococcus.s__Streptococcus_pasteurianus	-	3.30E-01	0.000	0.000	0.000	0.000	0.000
2.8.1.6|g__Streptococcus.s__Streptococcus_pneumoniae	-	3.30E-01	0.000	0.000	0.000	0.000	0.000
2.8.1.6|g__Sutterella.s__Sutterella_parvirubra	+	3.30E-01	0.000	0.000	0.000	0.000	0.001
2.8.1.6|g__Sutterella.s__Sutterella_wadsworthensis	+	2.96E-01	0.000	0.000	0.000	0.004	0.004
2.8.1.6|g__Veillonella.s__Veillonella_atypica	-	7.69E-01	0.000	0.000	0.000	0.003	0.001
2.8.1.6|g__Veillonella.s__Veillonella_dispar	-	7.25E-01	0.000	0.000	0.000	0.001	0.001
2.8.1.6|g__Veillonella.s__Veillonella_infantium	-	6.04E-01	0.000	0.000	0.000	0.001	0.001
2.8.1.6|g__Veillonella.s__Veillonella_rogosae	-	8.13E-03	0.000	0.000	0.000	0.002	0.001
2.8.1.6|g__Veillonella.s__Veillonella_tobetsuensis	-	3.05E-01	0.000	0.000	0.000	0.000	0.002
2.8.1.6|g__Victivallales_unclassified.s__Victivallales_bacterium_CCUG_44	+	2.39E-02	0.000	0.000	0.000	0.000	0.001
2.8.1.6|g__Victivallis.s__Victivallis_vadensis	+	4.71E-01	0.000	0.000	0.000	0.001	0.001
2.8.1.6|g__Yokenella.s__Yokenella_regensburgei	-	8.17E-01	0.000	0.000	0.000	0.000	0.000
2.8.1.6|g__Bacteroides.s__Bacteroides_intestinalis	+	1.11E-01	0.000	0.015	0.015	0.002	0.003
2.8.1.6|g__Clostridiales_unclassified.s__Clostridiales_bacterium_KLE1615	-	6.90E-01	0.040	0.055	0.015	0.002	0.002
2.8.1.6|g__Coprococcus.s__Coprococcus_comes	+	8.12E-01	0.053	0.081	0.028	0.001	0.002
2.8.1.6|g__Lawsonibacter.s__Lawsonibacter_asaccharolyticus	+	5.46E-03	0.000	0.038	0.038	0.000	0.001
2.8.1.6|g__Bacteroides.s__Bacteroides_fragilis	-	9.25E-01	0.096	0.164	0.068	0.016	0.016
2.8.1.6|g__Klebsiella.s__Klebsiella_pneumoniae	+	3.16E-01	0.027	0.100	0.073	0.009	0.029

	2.8.1.6|g__Blautia.s__Blautia_wexlerae	+	6.75E-01	0.230	0.304	0.074	0.011	0.012
	2.8.1.6|g__Bilophila.s__Bilophila_wadsworthia	+	2.58E-02	0.095	0.232	0.137	0.002	0.004
	2.8.1.6|g__Parabacteroides.s__Parabacteroides_merdae	+	1.86E-01	0.395	0.545	0.150	0.007	0.015
	2.8.1.6|g__Blautia.s__Ruminococcus_torques	+	6.35E-01	0.289	0.481	0.193	0.009	0.009
	2.8.1.6|g__Odoribacter.s__Odoribacter_splanchnicus	+	1.13E-01	0.092	0.331	0.239	0.003	0.005
	2.8.1.6|g__Eubacterium.s__Eubacterium_eligens	+	7.07E-01	0.000	0.257	0.257	0.011	0.008
	2.8.1.6|g__Bacteroides.s__Bacteroides_caccae	+	3.37E-01	0.157	0.440	0.283	0.008	0.011
	2.8.1.6|g__Bacteroides.s__Bacteroides_stercoris	+	7.46E-01	0.379	0.682	0.303	0.041	0.043
	2.8.1.6|g__Escherichia.s__Escherichia_coli	+	1.49E-01	0.312	1.001	0.689	0.035	0.061
	2.8.1.6|g__Bacteroides.s__Bacteroides_uniformis	+	4.12E-01	2.219	3.453	1.234	0.050	0.070
	2.8.1.6|unclassified	+	6.89E-02	10.523	13.165	2.642	0.120	0.164
Taiwan	2.8.1.6	-	5.86E-02	96.275	90.408	-5.867	1.000	1.000
	2.8.1.6|g__Bacteroides.s__Bacteroides_vulgatus	-	5.59E-01	7.501	5.562	-1.939	0.106	0.099
	2.8.1.6|g__Bacteroides.s__Bacteroides_plebeius	-	4.02E-01	0.781	0.298	-0.483	0.086	0.048
	2.8.1.6|unclassified	+	7.62E-01	8.198	7.773	-0.425	0.112	0.137
	2.8.1.6|g__Lachnospiraceae_unclassified.s__Eubacterium_rectale	-	4.27E-02	0.380	0.107	-0.273	0.017	0.010
	2.8.1.6|g__Roseburia.s__Roseburia_inulinivorans	-	2.29E-02	0.322	0.100	-0.222	0.010	0.006
	2.8.1.6|g__Roseburia.s__Roseburia_faecis	-	3.80E-01	0.232	0.044	-0.188	0.007	0.009
	2.8.1.6|g__Escherichia.s__Escherichia_coli	-	1.38E-01	0.229	0.078	-0.151	0.032	0.009
	2.8.1.6|g__Bacteroides.s__Bacteroides_massiliensis	-	8.46E-01	0.437	0.313	-0.124	0.026	0.025
	2.8.1.6|g__Agathobaculum.s__Agathobaculum_butyriciproducens	-	2.74E-01	0.123	0.032	-0.091	0.003	0.003
	2.8.1.6|g__Blautia.s__Ruminococcus_gnavus	-	7.35E-01	0.065	0.000	-0.065	0.010	0.013
	2.8.1.6|g__Bilophila.s__Bilophila_wadsworthia	-	4.93E-01	0.195	0.147	-0.048	0.004	0.003
	2.8.1.6|g__Coprococcus.s__Coprococcus_comes	-	4.00E-01	0.108	0.067	-0.041	0.006	0.004
	2.8.1.6|g__Blautia.s__Blautia_wexlerae	-	5.39E-01	0.105	0.093	-0.012	0.002	0.002
	2.8.1.6|g__Acidaminococcus.s__Acidaminococcus_fermentans	+	1.10E-01	0.000	0.000	0.000	0.000	0.000
	2.8.1.6|g__Acidaminococcus.s__Acidaminococcus_intestini	-	7.27E-01	0.000	0.000	0.000	0.001	0.004
	2.8.1.6|g__Aeromonas.s__Aeromonas_caviae	-	2.85E-01	0.000	0.000	0.000	0.000	0.000
	2.8.1.6|g__Aeromonas.s__Aeromonas_hydrophila	-	2.85E-01	0.000	0.000	0.000	0.000	0.000
	2.8.1.6|g__Aggregatibacter.s__Aggregatibacter_aphrophilus	-	2.85E-01	0.000	0.000	0.000	0.000	0.000
	2.8.1.6|g__Aggregatibacter.s__Aggregatibacter_segnis	-	2.85E-01	0.000	0.000	0.000	0.000	0.000
	2.8.1.6|g__Akkermansia.s__Akkermansia_muciniphila	+	7.58E-02	0.000	0.000	0.000	0.009	0.015
	2.8.1.6|g__Alistipes.s__Alistipes_indistinctus	-	8.60E-01	0.000	0.000	0.000	0.003	0.007
	2.8.1.6|g__Allisonella.s__Allisonella_histaminiformans	-	5.37E-01	0.000	0.000	0.000	0.000	0.000
	2.8.1.6|g__Anaerotignum.s__Anaerotignum_lactatifermentans	-	8.53E-01	0.000	0.000	0.000	0.001	0.001
	2.8.1.6|g__Anaerotruncus.s__Anaerotruncus_colihominis	-	9.98E-02	0.000	0.000	0.000	0.001	0.000
	2.8.1.6|g__Bacteroides.s__Bacteroides_clarus	-	9.05E-01	0.000	0.000	0.000	0.003	0.008
	2.8.1.6|g__Bacteroides.s__Bacteroides_coprocola	-	9.83E-01	0.000	0.000	0.000	0.047	0.036
	2.8.1.6|g__Bacteroides.s__Bacteroides_coprophilus	-	6.91E-01	0.000	0.000	0.000	0.013	0.008
	2.8.1.6|g__Bacteroides.s__Bacteroides_eggerthii	+	3.26E-01	0.000	0.000	0.000	0.007	0.009
	2.8.1.6|g__Bacteroides.s__Bacteroides_faecichinchillae	+	3.68E-01	0.000	0.000	0.000	0.000	0.000
	2.8.1.6|g__Bacteroides.s__Bacteroides_fluxus	-	9.26E-01	0.000	0.000	0.000	0.000	0.002
	2.8.1.6|g__Bacteroides.s__Bacteroides_fragilis	+	4.78E-01	0.000	0.000	0.000	0.010	0.011
	2.8.1.6|g__Bacteroides.s__Bacteroides_nordii	+	5.66E-01	0.000	0.000	0.000	0.001	0.001
	2.8.1.6|g__Bacteroides.s__Bacteroides_salyersiae	-	7.90E-01	0.000	0.000	0.000	0.006	0.003
	2.8.1.6|g__Bacteroides.s__Bacteroides_sartorii	+	8.11E-01	0.000	0.000	0.000	0.004	0.002
	2.8.1.6|g__Bacteroides.s__Bacteroides_sp_OM08_11	+	6.86E-01	0.000	0.000	0.000	0.000	0.000
	2.8.1.6|g__Barnesiella.s__Barnesiella_intestinihominis	+	4.48E-02	0.000	0.000	0.000	0.006	0.012
	2.8.1.6|g__Blautia.s__Blautia_sp_AF19_10LB	-	5.90E-01	0.000	0.000	0.000	0.000	0.000
	2.8.1.6|g__Butyricimonas.s__Butyricimonas_synergistica	-	8.87E-01	0.000	0.000	0.000	0.000	0.000
	2.8.1.6|g__Campylobacter.s__Campylobacter_concisus	-	2.85E-01	0.000	0.000	0.000	0.000	0.000
	2.8.1.6|g__Candidatus_Gastranaerophilales_unclassified.s__Candidatus_Ga:	+	3.68E-01	0.000	0.000	0.000	0.000	0.000
	2.8.1.6|g__Catabacter.s__Catabacter_hongkongensis	-	9.05E-01	0.000	0.000	0.000	0.000	0.000
	2.8.1.6|g__Catenibacterium.s__Catenibacterium_mitsuokai	-	5.86E-01	0.000	0.000	0.000	0.004	0.006
	2.8.1.6|g__Cetobacterium.s__Cetobacterium_somerae	+	1.96E-01	0.000	0.000	0.000	0.000	0.000
	2.8.1.6|g__Citrobacter.s__Citrobacter_amalonaticus	-	1.25E-01	0.000	0.000	0.000	0.000	0.000
	2.8.1.6|g__Citrobacter.s__Citrobacter_braakii	-	1.25E-01	0.000	0.000	0.000	0.000	0.000
	2.8.1.6|g__Citrobacter.s__Citrobacter_farmeri	-	2.85E-01	0.000	0.000	0.000	0.000	0.000
	2.8.1.6|g__Citrobacter.s__Citrobacter_freundii	-	5.12E-01	0.000	0.000	0.000	0.000	0.000
	2.8.1.6|g__Citrobacter.s__Citrobacter_koseri	+	6.86E-01	0.000	0.000	0.000	0.000	0.000
	2.8.1.6|g__Citrobacter.s__Citrobacter_portucalensis	-	3.34E-01	0.000	0.000	0.000	0.000	0.000
	2.8.1.6|g__Citrobacter.s__Citrobacter_werkmanii	-	9.26E-01	0.000	0.000	0.000	0.000	0.000
	2.8.1.6|g__Citrobacter.s__Citrobacter_youngae	-	9.76E-01	0.000	0.000	0.000	0.000	0.000
	2.8.1.6|g__Cloacibacillus.s__Cloacibacillus_porcorum	+	2.16E-02	0.000	0.000	0.000	0.000	0.000
	2.8.1.6|g__Clostridiales_unclassified.s__Clostridiales_bacterium_KLE1615	+	4.87E-01	0.000	0.000	0.000	0.002	0.003
	2.8.1.6|g__Clostridioides.s__Clostridioides_difficile	-	9.26E-01	0.000	0.000	0.000	0.000	0.000
	2.8.1.6|g__Clostridium.s__Butyribacterium_methylotrophicum	+	3.68E-01	0.000	0.000	0.000	0.000	0.000
	2.8.1.6|g__Clostridium.s__Clostridium_disporicum	+	6.54E-01	0.000	0.000	0.000	0.000	0.000
	2.8.1.6|g__Clostridium.s__Clostridium_neonatale	+	3.68E-01	0.000	0.000	0.000	0.000	0.000
	2.8.1.6|g__Clostridium.s__Clostridium_perfringens	-	1.25E-01	0.000	0.000	0.000	0.001	0.000
	2.8.1.6|g__Clostridium.s__Clostridium_sp_AF36_4	+	7.40E-01	0.000	0.000	0.000	0.001	0.001
	2.8.1.6|g__Clostridium.s__Clostridium_sp_D5	-	4.71E-01	0.000	0.000	0.000	0.000	0.000

2.8.1.6|g__Coprobacter.s__Coprobacter_fastidiosus	+	3.36E-01	0.000	0.000	0.000	0.001	0.004
2.8.1.6|g__Coprobacter.s__Coprobacter_secundus	+	9.28E-01	0.000	0.000	0.000	0.000	0.000
2.8.1.6|g__Coprococcus.s__Coprococcus_eutactus	+	9.50E-01	0.000	0.000	0.000	0.003	0.002
2.8.1.6|g__Desulfovibrio.s__Desulfovibrio_desulfuricans	+	3.68E-01	0.000	0.000	0.000	0.000	0.000
2.8.1.6|g__Desulfovibrio.s__Desulfovibrio_fairfieldensis	+	3.14E-01	0.000	0.000	0.000	0.000	0.000
2.8.1.6|g__Desulfovibrio.s__Desulfovibrio_legallii	+	3.68E-01	0.000	0.000	0.000	0.000	0.000
2.8.1.6|g__Desulfovibrio.s__Desulfovibrio_piger	+	9.81E-01	0.000	0.000	0.000	0.001	0.001
2.8.1.6|g__Desulfovibrio.s__Desulfovibrio_sp_AM18_2	+	3.68E-01	0.000	0.000	0.000	0.000	0.000
2.8.1.6|g__Desulfovibrionaceae_unclassified.s__Desulfovibrionaceae_bacte	-	9.57E-02	0.000	0.000	0.000	0.000	0.000
2.8.1.6|g__Dialister.s__Dialister_invisus	-	4.71E-01	0.000	0.000	0.000	0.001	0.000
2.8.1.6|g__Dialister.s__Dialister_pneumosintes	+	3.68E-01	0.000	0.000	0.000	0.000	0.000
2.8.1.6|g__Dialister.s__Dialister_succinatiphilus	-	3.56E-01	0.000	0.000	0.000	0.020	0.006
2.8.1.6|g__Dorea.s__Dorea_formicigenerans	-	7.59E-01	0.000	0.000	0.000	0.001	0.001
2.8.1.6|g__Dorea.s__Dorea_longicatena	-	9.84E-01	0.000	0.000	0.000	0.001	0.002
2.8.1.6|g__Dorea.s__Dorea_sp_OM02_2LB	+	4.08E-01	0.000	0.000	0.000	0.000	0.000
2.8.1.6|g__Eisenbergiella.s__Eisenbergiella_tayi	+	6.86E-01	0.000	0.000	0.000	0.000	0.000
2.8.1.6|g__Enterobacter.s__Enterobacter_bugandensis	-	8.57E-01	0.000	0.000	0.000	0.000	0.000
2.8.1.6|g__Enterobacter.s__Enterobacter_cloacae	-	1.64E-01	0.000	0.000	0.000	0.000	0.000
2.8.1.6|g__Enterobacter.s__Enterobacter_kobei	-	2.85E-01	0.000	0.000	0.000	0.000	0.000
2.8.1.6|g__Erysipelatoclostridium.s__Clostridium_spiroforme	+	3.68E-01	0.000	0.000	0.000	0.000	0.000
2.8.1.6|g__Erysipelatoclostridium.s__Erysipelatoclostridium_ramosum	+	5.60E-01	0.000	0.000	0.000	0.000	0.000
2.8.1.6|g__Escherichia.s__Escherichia_fergusonii	-	9.86E-01	0.000	0.000	0.000	0.000	0.000
2.8.1.6|g__Eubacterium.s__Eubacterium_limosum	+	3.68E-01	0.000	0.000	0.000	0.000	0.000
2.8.1.6|g__Eubacterium.s__Eubacterium_maltosivorans	+	3.68E-01	0.000	0.000	0.000	0.000	0.000
2.8.1.6|g__Eubacterium.s__Eubacterium_ramulus	-	1.90E-01	0.000	0.000	0.000	0.000	0.000
2.8.1.6|g__Eubacterium.s__Eubacterium_sp_AF17_7	+	2.20E-01	0.000	0.000	0.000	0.000	0.000
2.8.1.6|g__Faecalicatena.s__Faecalicatena_contorta	+	9.01E-01	0.000	0.000	0.000	0.000	0.000
2.8.1.6|g__Firmicutes_unclassified.s__Firmicutes_bacterium_AM10_47	+	2.28E-01	0.000	0.000	0.000	0.000	0.000
2.8.1.6|g__Fusobacterium.s__Fusobacterium_mortiferum	-	4.50E-01	0.000	0.000	0.000	0.005	0.001
2.8.1.6|g__Fusobacterium.s__Fusobacterium_ulcerans	+	3.87E-01	0.000	0.000	0.000	0.000	0.001
2.8.1.6|g__Fusobacterium.s__Fusobacterium_varium	-	4.89E-01	0.000	0.000	0.000	0.002	0.000
2.8.1.6|g__Gordonibacter.s__Gordonibacter_pamelaeae	+	6.54E-01	0.000	0.000	0.000	0.000	0.000
2.8.1.6|g__Haemophilus.s__Haemophilus_parahaemolyticus	+	3.68E-01	0.000	0.000	0.000	0.000	0.000
2.8.1.6|g__Haemophilus.s__Haemophilus_parainfluenzae	-	3.05E-02	0.000	0.000	0.000	0.004	0.001
2.8.1.6|g__Hungatella.s__Hungatella_effluvii	+	6.11E-02	0.000	0.000	0.000	0.000	0.001
2.8.1.6|g__Hungatella.s__Hungatella_hathewayi	+	1.70E-02	0.000	0.000	0.000	0.000	0.001
2.8.1.6|g__Klebsiella.s__Klebsiella_aerogenes	-	6.06E-01	0.000	0.000	0.000	0.000	0.000
2.8.1.6|g__Klebsiella.s__Klebsiella_grimontii	-	2.85E-01	0.000	0.000	0.000	0.000	0.000
2.8.1.6|g__Klebsiella.s__Klebsiella_michiganensis	-	9.05E-01	0.000	0.000	0.000	0.000	0.000
2.8.1.6|g__Klebsiella.s__Klebsiella_oxytoca	-	1.77E-01	0.000	0.000	0.000	0.000	0.000
2.8.1.6|g__Klebsiella.s__Klebsiella_pneumoniae	-	9.63E-01	0.000	0.000	0.000	0.009	0.003
2.8.1.6|g__Klebsiella.s__Klebsiella_variicola	+	6.14E-01	0.000	0.000	0.000	0.000	0.000
2.8.1.6|g__Kluyvera.s__Kluyvera_ascorbata	-	2.85E-01	0.000	0.000	0.000	0.000	0.000
2.8.1.6|g__Kluyvera.s__Kluyvera_georgiana	-	2.85E-01	0.000	0.000	0.000	0.001	0.000
2.8.1.6|g__Lachnoclostridium.s__Clostridium_citroniae	-	4.06E-01	0.000	0.000	0.000	0.000	0.000
2.8.1.6|g__Lachnoclostridium.s__Clostridium_saccharolyticum	-	2.54E-02	0.000	0.000	0.000	0.001	0.001
2.8.1.6|g__Lachnoclostridium.s__Clostridium_scindens	+	4.69E-01	0.000	0.000	0.000	0.000	0.001
2.8.1.6|g__Lachnoclostridium.s__Clostridium_symbiosum	+	8.50E-02	0.000	0.000	0.000	0.000	0.002
2.8.1.6|g__Lachnospira.s__Lachnospira_pectinoschiza	-	2.40E-01	0.000	0.000	0.000	0.004	0.002
2.8.1.6|g__Lactobacillus.s__Lactobacillus_rogosae	-	2.70E-01	0.000	0.000	0.000	0.002	0.001
2.8.1.6|g__Lawsonibacter.s__Lawsonibacter_asaccharolyticus	-	8.56E-01	0.000	0.000	0.000	0.001	0.001
2.8.1.6|g__Leclercia.s__Leclercia_adecarboxylata	-	2.97E-01	0.000	0.000	0.000	0.000	0.000
2.8.1.6|g__Lelliottia.s__Lelliottia_nimipressuralis	-	3.01E-01	0.000	0.000	0.000	0.001	0.000
2.8.1.6|g__Megamonas.s__Megamonas_funiformis	-	4.90E-01	0.000	0.000	0.000	0.007	0.006
2.8.1.6|g__Megamonas.s__Megamonas_hypermegale	-	6.37E-01	0.000	0.000	0.000	0.002	0.001
2.8.1.6|g__Megamonas.s__Megamonas_rupellensis	-	6.43E-01	0.000	0.000	0.000	0.004	0.003
2.8.1.6|g__Megasphaera.s__Megasphaera_elsdenii	-	1.25E-01	0.000	0.000	0.000	0.000	0.000
2.8.1.6|g__Megasphaera.s__Megasphaera_hexanoica	+	3.68E-01	0.000	0.000	0.000	0.000	0.000
2.8.1.6|g__Megasphaera.s__Megasphaera_micronuciformis	+	1.96E-01	0.000	0.000	0.000	0.000	0.000
2.8.1.6|g__Megasphaera.s__Megasphaera_stantonii	-	3.27E-01	0.000	0.000	0.000	0.000	0.000
2.8.1.6|g__Metakosakonia.s__Kluyvera_intestini	+	3.68E-01	0.000	0.000	0.000	0.000	0.000
2.8.1.6|g__Mitsuokella.s__Mitsuokella_jalaludinii	-	2.50E-01	0.000	0.000	0.000	0.000	0.000
2.8.1.6|g__Mitsuokella.s__Mitsuokella_multacida	-	5.20E-01	0.000	0.000	0.000	0.001	0.001
2.8.1.6|g__Odoribacter.s__Odoribacter_laneus	+	3.87E-01	0.000	0.000	0.000	0.000	0.003
2.8.1.6|g__Oxalobacter.s__Oxalobacter_formigenes	-	7.97E-01	0.000	0.000	0.000	0.000	0.000
2.8.1.6|g__Paeniclostridium.s__Paeniclostridium_sordellii	+	3.68E-01	0.000	0.000	0.000	0.000	0.000
2.8.1.6|g__Pantoea.s__Pantoea_sesami	-	4.93E-01	0.000	0.000	0.000	0.000	0.000
2.8.1.6|g__Parabacteroides.s__Parabacteroides_chinchillae	-	1.25E-01	0.000	0.000	0.000	0.000	0.000
2.8.1.6|g__Parabacteroides.s__Parabacteroides_goldsteinii	+	7.77E-02	0.000	0.000	0.000	0.001	0.003
2.8.1.6|g__Parabacteroides.s__Parabacteroides_gordonii	-	5.21E-01	0.000	0.000	0.000	0.000	0.000
2.8.1.6|g__Parabacteroides.s__Parabacteroides_johnsonii	+	7.42E-01	0.000	0.000	0.000	0.002	0.001
2.8.1.6|g__Paraprevotella.s__Paraprevotella_clara	-	6.66E-01	0.000	0.000	0.000	0.008	0.008

2.8.1.6|g__Paraprevotella.s__Paraprevotella_xylaniphila	+	8.39E-02	0.000	0.000	0.000	0.000	0.003
2.8.1.6|g__Pedobacter.s__Pedobacter_himalayensis	-	2.85E-01	0.000	0.000	0.000	0.000	0.000
2.8.1.6|g__Phascolarctobacterium.s__Phascolarctobacterium_succinatutens	+	3.71E-01	0.000	0.000	0.000	0.005	0.010
2.8.1.6|g__Plesiomonas.s__Plesiomonas_shigelloides	-	2.85E-01	0.000	0.000	0.000	0.000	0.000
2.8.1.6|g__Pseudocitrobacter.s__Pseudocitrobacter_faecalis	+	3.68E-01	0.000	0.000	0.000	0.000	0.000
2.8.1.6|g__Pseudoflavonifractor.s__Pseudoflavonifractor_sp_An184	-	4.71E-01	0.000	0.000	0.000	0.000	0.000
2.8.1.6|g__Raoultella.s__Raoultella_ornithinolytica	-	5.10E-01	0.000	0.000	0.000	0.000	0.000
2.8.1.6|g__Raoultella.s__Raoultella_planticola	-	4.84E-01	0.000	0.000	0.000	0.000	0.000
2.8.1.6|g__Roseburia.s__Roseburia_intestinalis	-	9.07E-01	0.000	0.000	0.000	0.010	0.006
2.8.1.6|g__Ruminococcaceae_unclassified.s__Eubacterium_siraeum	+	1.83E-01	0.000	0.000	0.000	0.004	0.009
2.8.1.6|g__Ruminococcaceae_unclassified.s__Ruminococcaceae_bacterium_	-	2.90E-01	0.000	0.000	0.000	0.000	0.000
2.8.1.6|g__Ruminococcus.s__Ruminococcus_lactaris	-	3.22E-01	0.000	0.000	0.000	0.006	0.002
2.8.1.6|g__Salmonella.s__Salmonella_enterica	-	9.26E-01	0.000	0.000	0.000	0.000	0.000
2.8.1.6|g__Sanguibacteroides.s__Sanguibacteroides_justesenii	-	8.72E-01	0.000	0.000	0.000	0.000	0.000
2.8.1.6|g__Streptococcus.s__Streptococcus_equinus	+	1.96E-01	0.000	0.000	0.000	0.000	0.000
2.8.1.6|g__Streptococcus.s__Streptococcus_gallolyticus	+	1.96E-01	0.000	0.000	0.000	0.000	0.000
2.8.1.6|g__Streptococcus.s__Streptococcus_pasteurianus	+	1.96E-01	0.000	0.000	0.000	0.000	0.000
2.8.1.6|g__Succinatimonas.s__Succinatimonas_hippei	+	6.34E-01	0.000	0.000	0.000	0.000	0.001
2.8.1.6|g__Sutterella.s__Sutterella_parvirubra	-	4.57E-01	0.000	0.000	0.000	0.003	0.000
2.8.1.6|g__Veillonella.s__Veillonella_atypica	-	1.09E-01	0.000	0.000	0.000	0.000	0.000
2.8.1.6|g__Veillonella.s__Veillonella_dispar	-	7.11E-01	0.000	0.000	0.000	0.001	0.000
2.8.1.6|g__Veillonella.s__Veillonella_infantium	-	6.85E-01	0.000	0.000	0.000	0.001	0.000
2.8.1.6|g__Veillonella.s__Veillonella_parvula	-	1.91E-01	0.000	0.000	0.000	0.001	0.000
2.8.1.6|g__Veillonella.s__Veillonella_rogosae	-	5.61E-02	0.000	0.000	0.000	0.001	0.000
2.8.1.6|g__Veillonella.s__Veillonella_tobetsuensis	-	8.57E-01	0.000	0.000	0.000	0.000	0.000
2.8.1.6|g__Veillonellaceae_unclassified.s__Veillonellaceae_bacterium	-	9.26E-01	0.000	0.000	0.000	0.000	0.000
2.8.1.6|g__Vibrio.s__Vibrio_parahaemolyticus	-	2.85E-01	0.000	0.000	0.000	0.000	0.000
2.8.1.6|g__Victivallales_unclassified.s__Victivallales_bacterium_CCUG_44	+	2.48E-01	0.000	0.000	0.000	0.001	0.002
2.8.1.6|g__Victivallis.s__Victivallis_vadensis	+	1.01E-01	0.000	0.000	0.000	0.000	0.001
2.8.1.6|g__Yokenella.s__Yokenella_regensburgei	-	5.10E-01	0.000	0.000	0.000	0.000	0.000
2.8.1.6|g__Bacteroides.s__Bacteroides_xylanisolvens	+	9.25E-01	0.372	0.384	0.012	0.015	0.015
2.8.1.6|g__Anaerostipes.s__Anaerostipes_hadrus	+	1.66E-01	0.000	0.019	0.019	0.002	0.003
2.8.1.6|g__Bacteroides.s__Bacteroides_finegoldii	+	3.05E-01	0.000	0.029	0.029	0.005	0.007
2.8.1.6|g__Bacteroides.s__Bacteroides_intestinalis	+	4.22E-02	0.000	0.051	0.051	0.004	0.011
2.8.1.6|g__Blautia.s__Blautia_obeum	+	5.28E-01	0.159	0.222	0.064	0.003	0.005
2.8.1.6|g__Butyricimonas.s__Butyricimonas_virosa	+	7.23E-02	0.000	0.073	0.073	0.003	0.006
2.8.1.6|g__Bacteroides.s__Bacteroides_ovatus	+	2.19E-01	0.401	0.501	0.099	0.012	0.017
2.8.1.6|g__Phascolarctobacterium.s__Phascolarctobacterium_faecium	+	4.88E-01	0.399	0.581	0.182	0.010	0.015
2.8.1.6|g__Bacteroides.s__Bacteroides_stercoris	-	7.32E-01	1.795	1.978	0.183	0.065	0.050
2.8.1.6|g__Eubacterium.s__Eubacterium_eligens	+	6.55E-01	0.192	0.397	0.206	0.009	0.010
2.8.1.6|g__Bacteroides.s__Bacteroides_dorei	+	2.00E-01	0.066	0.288	0.222	0.021	0.033
2.8.1.6|g__Blautia.s__Ruminococcus_torques	+	4.09E-01	0.244	0.487	0.243	0.010	0.013
2.8.1.6|g__Sutterella.s__Sutterella_wadsworthensis	+	8.30E-01	0.189	0.478	0.289	0.018	0.022
2.8.1.6|g__Bacteroides.s__Bacteroides_thetaiotaomicron	+	1.36E-01	0.831	1.122	0.291	0.017	0.024
2.8.1.6|g__Odoribacter.s__Odoribacter_splanchnicus	+	4.03E-02	0.200	0.589	0.389	0.009	0.011
2.8.1.6|g__Parabacteroides.s__Parabacteroides_distasonis	+	2.62E-02	0.779	1.237	0.458	0.015	0.025
2.8.1.6|g__Bacteroides.s__Bacteroides_cellulosilyticus	+	1.43E-02	0.000	0.616	0.616	0.016	0.018
2.8.1.6|g__Bacteroides.s__Bacteroides_uniformis	-	9.68E-01	3.649	4.296	0.647	0.079	0.076
2.8.1.6|g__Parabacteroides.s__Parabacteroides_merdae	+	2.84E-02	0.404	1.085	0.681	0.010	0.020
2.8.1.6|g__Bacteroides.s__Bacteroides_caccae	+	2.29E-02	0.325	1.402	1.076	0.014	0.020

P-value was calculated for fractional CPM of each bacterium contributing to 9 EC numbers between Control and PD by the Wilcoxon rank sum test.

Supplementary Table 13. Statistical measures of the numbers of read counts in six datasets

Country (sample size)	Average	SD	Median	Max	Min
Japan (167)	8.8	1.4	8.8	12.2	1.4
USA (725)	24.5	15.5	20.4	192.5	8.9
Taiwan (107)	29.6	7.7	28.7	54.4	4.9
China1 (80)	43.5	19.6	41.8	141.1	3.3
China2 (78)	63.5	21.4	59.1	120.3	27.3
Germany (59)	24.9	8.8	22.3	39.9	7.1

(x 1,000,000 read counts)
