# Supplementary material for: Meta-analysis of shotgun sequencing of gut microbiota in Parkinson’s disease
Source: NPJ Parkinsons Dis. 2024 May 21;10:106. doi: 10.1038/s41531-024-00724-z (PMC11109112; doi:10.1038/s41531-024-00724-z)
Supplement: Supplementary file 2 — reporting-summary [file 41531_2024_724_MOESM2_ESM.pdf]

nature portfolio

Corresponding author(s): Kinji Ohni, Masaaki Hirayama, Hiroshi Nishiwaki

Last updated by author(s): May 2, 2024

Reporting Summary

Nature Portfolio wishes to improve the reproducibility of the work that we publish. This form provides structure for consistency and transparency in reporting. For further information on Nature Portfolio policies, see our Editorial Policies and the Editorial Policy Checklist.

Statistics

For all statistical analyses, confirm that the following items are present in the figure legend, table legend, main text, or Methods section.

n/a	Confirmed	
☐	☒	The exact sample size (n) for each experimental group/condition, given as a discrete number and unit of measurement
☐	☒	A statement on whether measurements were taken from distinct samples or whether the same sample was measured repeatedly
☐	☒	The statistical test(s) used AND whether they are one- or two-sided<br>*Only common tests should be described solely by name; describe more complex techniques in the Methods section.*
☐	☒	A description of all covariates tested
☐	☒	A description of any assumptions or corrections, such as tests of normality and adjustment for multiple comparisons
☐	☒	A full description of the statistical parameters including central tendency (e.g. means) or other basic estimates (e.g. regression coefficient) AND variation (e.g. standard deviation) or associated estimates of uncertainty (e.g. confidence intervals)
☐	☒	For null hypothesis testing, the test statistic (e.g. F, t, r) with confidence intervals, effect sizes, degrees of freedom and P value noted<br>*Give P values as exact values whenever suitable.*
☒	☐	For Bayesian analysis, information on the choice of priors and Markov chain Monte Carlo settings
☒	☐	For hierarchical and complex designs, identification of the appropriate level for tests and full reporting of outcomes
☐	☒	Estimates of effect sizes (e.g. Cohen's d, Pearson's r), indicating how they were calculated

Our web collection on statistics for biologists contains articles on many of the points above.

Software and code

Policy information about availability of computer code

Data collection	No software was used to collect data.
Data analysis	we conducted quality control with fastp (v0.23.4) and removed human genome (GRCh38) with bowtie2 (Version 2.5.0) using default parameters.

For manuscripts utilizing custom algorithms or software that are central to the research but not yet described in published literature, software must be made available to editors and reviewers. We strongly encourage code deposition in a community repository (e.g. GitHub). See the Nature Portfolio guidelines for submitting code & software for further information.

Data

Policy information about availability of data

All manuscripts must include a data availability statement. This statement should provide the following information, where applicable:

- Accession codes, unique identifiers, or web links for publicly available datasets
- A description of any restrictions on data availability
- For clinical datasets or third party data, please ensure that the statement adheres to our policy

Sequence data of our dataset are available at the DNA Data Bank of Japan (DDBJ) under the accession number of DRA016410.

Research involving human participants, their data, or biological material

Policy information about studies with human participants or human data. See also policy information about sex, gender (identity/presentation), and sexual orientation and race, ethnicity and racism.

Reporting on sex and gender	We carefully collected data on gender methods. There were no examples indicating gender incongruity.
Reporting on race, ethnicity, or other socially relevant groupings	We recruited PD patients and controls in Iwate, Nagoya, Okayama, Fukuoka in Japan.
Population characteristics	The control group's average age is approximately 68 years, while the age of PD patients is approximately 69 years. It has been about 8 years since they were diagnosed with PD, and 80% are taking Levodopa.
Recruitment	We recruited 94 patients with idiopathic PD and their 73 healthy spouses who lived together from September 2015 to February 2018.
Ethics oversight	All studies were approved by the Ethical Review Committees (ERC) of the Nagoya University Graduate School of Medicine (approval #2016-0151), Iwate Medical College Hospital (H28-123), Okayama Kyokuto Hospital (approval #kyoIR-2016002), and Fukuoka Medical College (approval #2016M027).

Note that full information on the approval of the study protocol must also be provided in the manuscript.

Field-specific reporting

Please select the one below that is the best fit for your research. If you are not sure, read the appropriate sections before making your selection.

☒ Life sciences ☐ Behavioural & social sciences ☐ Ecological, evolutionary & environmental sciences

For a reference copy of the document with all sections, see nature.com/documents/nr-reporting-summary-flat.pdf

Life sciences study design

All studies must disclose on these points even when the disclosure is negative.

Sample size	We performed an explanatory study and the sample size was not determined in advance.
Data exclusions	PD patients with other chronic diseases including diabetes mellitus, heart failure, liver cirrhosis, malignancy, hematological diseases, and autoimmune diseases were excluded. Similarly, our cohort did not include PD patients who claimed to have taken antibiotics in the past one month.
Replication	Our study is an observatory study of a cohort of PD patients, and fecal samples and clinical data could not be replicated.
Randomization	No
Blinding	No

Reporting for specific materials, systems and methods

We require information from authors about some types of materials, experimental systems and methods used in many studies. Here, indicate whether each material, system or method listed is relevant to your study. If you are not sure if a list item applies to your research, read the appropriate section before selecting a response.

Materials & experimental systems

n/a	Involved in the study	
☒	☐	Antibodies
☒	☐	Eukaryotic cell lines
☒	☐	Palaeontology and archaeology
☒	☐	Animals and other organisms
☒	☐	Clinical data
☒	☐	Dual use research of concern
☒	☐	Plants

Methods

n/a	Involved in the study	
☒	☐	ChIP-seq
☒	☐	Flow cytometry
☒	☐	MRI-based neuroimaging

Plants

Seed stocks	*Report on the source of all seed stocks or other plant material used. If applicable, state the seed stock centre and catalogue number. If plant specimens were collected from the field, describe the collection location, date and sampling procedures.*
Novel plant genotypes	*Describe the methods by which all novel plant genotypes were produced. This includes those generated by transgenic approaches, gene editing, chemical/radiation-based mutagenesis and hybridization. For transgenic lines, describe the transformation method, the number of independent lines analyzed and the generation upon which experiments were performed. For gene-edited lines, describe the editor used, the endogenous sequence targeted for editing, the targeting guide RNA sequence (if applicable) and how the editor was applied.*
Authentication	*Describe any authentication procedures for each seed stock used or novel genotype generated. Describe any experiments used to assess the effect of a mutation and, where applicable, how potential secondary effects (e.g. second site T-DNA insertions, mosiacism, off-target gene editing) were examined.*
